# A Novel Cuproptosis-Associated Gene Signature to Predict Prognosis in Patients with Pancreatic Cancer

**DOI:** 10.1155/2023/3419401

**Published:** 2023-01-18

**Authors:** Yan Du, Wenkai Jiang, Shuang Hou, Zhou Chen, Wence Zhou

**Affiliations:** ^1^The Second School of Clinical Medicine, Lanzhou University, Lanzhou 730030, China; ^2^Department of General Surgery, The First Hospital of Lanzhou University, Lanzhou 730030, China; ^3^Department of General Surgery, Lanzhou University Second Hospital, Lanzhou 730030, China

## Abstract

**Background:**

Pancreatic cancer (PAAD) is a malignant tumor with a poor prognosis and lacks sensitive biomarkers for diagnosis and targeted therapy. Cuproptosis, a recently proposed form of cell death based on cellular copper ion concentration, plays a key role in cancer biology. This study is aimed at constructing a risk model for predicting the prognosis of PAAD patients based on cuproptosis-related genes.

**Methods:**

Pancreatic-related data from UCSC-TCGA and UCSC-GTEx databases were extracted for analysis, and TCGA-PAAD samples were randomly divided into the training and validation groups. Pearson correlation analysis was used to obtain cuproptosis-related genes coexpressed with 19 copper death genes. Univariate Cox and Lasso regression analyses were used to obtain cuproptosis-related prognostic genes. Multivariate Cox regression analysis was used to construct the final prognostic risk model. The risk score curve, Kaplan-Meier survival curves, and ROC curve were used to evaluate the predictive ability of the Cox risk model. Finally, the functional annotation of the risk model was obtained through enrichment analysis.

**Results:**

The Cox risk model has an eight prognostic cuproptosis-related gene signature. Kaplan-Meier survival curves demonstrated that the high-risk group had a shorter survival time. The ROC curve of the risk score was well created to predict one-, three-, and five-year survival rates, and AUC of the risk score was higher than other clinical characteristics. Cox regression analysis revealed that the risk score has an independent prognostic value for PAAD. GSEA reveals specific tumor pathways associated with the risk model (Myc targets v1, mTORC1 signaling, and E2F targets).

**Conclusions:**

We constructed a prognostic model containing eight cuproptosis-related genes (AKR1B10, KLHL29, PROM2, PIP5K1C, KIF18B, AMIGO2, MRPL3, and PI4KB) that can accurately predict the prognosis of PAAD patients. The results will provide new perspectives for individualized outcome prediction and new therapy development for PAAD patients.

## 1. Introduction

Pancreatic adenocarcinoma (PAAD) exhibits a poor prognosis in digestive system tumors because of its insidious onset, rapid development, and tendency to metastasize [1]. Due to the lack of effective diagnostic methods and nontypical symptoms, patients are usually diagnosed in a locally advanced or metastatic stage. Over 80% of patients miss the chance of surgical treatment when diagnosed [2, 3]. At present, early diagnosis, prognosis prediction, and potential immunotherapy targets for PAAD patients are key issues in clinical practice.

Copper is a basic element that is critical to the health of organisms [4]. Normally, the concentration of intracellular copper ions is kept at a low level, and when copper ions gradually accumulate beyond the threshold, reactive oxygen species will be generated, and cell death will be induced. Copper is closely related to cancer occurrence. In 1975, Schwartz proposed that various trace elements, including copper, could be used to diagnose or predict cancer [5]. Copper is involved in the growth of cancer cells and can promote neovascularization and metastasis, leading to cancer occurrence and development [6]. In 2022, researchers proposed a different mechanism for regulating cell death from known mechanisms, including apoptosis, ferroptosis, and necrosis [7]. This independent copper-related cell death mechanism is called cuproptosis. Cuproptosis occurs through the direct binding of copper to the ribosylated portions of the tricarboxylic acid cycle (TAC), leading to aggregation of ribosylated proteins and instability of Fe-S cluster proteins resulting in proteotoxic stress and cell death. The association between PAAD and copper was strong. The serum copper level is significantly higher in PAAD patients than in normal patients, and higher levels of copper are associated with a greater risk of being diagnosed with PAAD [8]. However, the relationship between copper metabolism-related genes and the prognosis of patients with PAAD remains unclear. This study used bioinformatics methods to identify eight cuproptosis-related genes, and a prognostic model was established in PAAD patients. Based on the risk score, we aimed to better determine the prognosis of PAAD patients and better understand the intrinsic relationship between PAAD and cuproptosis.

## 2. Methods

### 2.1. Data Download

The Cancer Genome Atlas (TCGA) database contains RNA-seq data from 178 pancreatic and four normal pancreatic tissue samples [9]. The GTEx database contains RNA-seq data from 167 normal pancreas tissue samples [9]. The University of California Santa Cruz (UCSC) database collates and standardizes data from TCGA and GTEx databases [10]. All transcripts per kilobase million (TPM) data were obtained in the UCSC database on 1 April 2022. The Wilcox test, suitable for large sample size and TPM data, was used to analyze differences in gene expression between the pancreatic cancer tumor group and the normal group. Differentially expressed genes (DEGs) were defined as the absolute value of *Log* *Fold* *Change* (LogFC) > 1 and *p* value < 0.05. Meanwhile, clinical information for all samples was downloaded, including age, sex, TNM stage, AJCC stage, overall survival (OS) time, progression-free survival (PFS) time, and disease-specific survival (DSS) time. Excluding samples with incomplete data, 178 patients were included.

### 2.2. Identification of Cuproptosis-Related Genes

Tsvetkov et al., Deng et al., and Emami et al. proposed key genes involved in the tumor copper death mechanism, including ATP7A, ATP7B, CDKN2A, DBT, DLAT, DLD, DLST, FDX1, GCSH, GLS, LIAS, LIPT1, LIPT2, MTF1, NFE2L2, NLRP3, PDHA1, PDHB, and SLC31A1 [7, 11, 12]. The cuproptosis-related genes coexpressed with cuproptosis genes were obtained based on TCGA-PAAD samples. Pearson's correlation analysis was performed between 19 genes and DEGs in samples to identify cuproptosis-related genes according to the correlation coefficient > 0.5 and *p* value < 0.05.

### 2.3. Construction of the Cox Risk Model

With R package “caret,” 178 cases were randomly assigned to the training (*n* = 88) and validation (*n* = 90) groups. The chi-squared test compared the clinical characteristics of patients in the training group and the validation group, aiming to evaluate the heterogeneity of the two sets of data. The data of the training group were used to construct the Cox risk model of cuproptosis-related genes. The cuproptosis-related genes were subjected to univariate Cox regression analysis based on OS, and the genes with prognostic significance were screened out (*p* < 0.01). To avoid overfitting the model, Least Absolute Shrinkage and Selection Operator (Lasso) regression analysis was performed on potential prognostic genes. When the cross-validation error of Lasso regression is the smallest, the list of prognostic genes corresponding to the best penalty parameter is obtained. Lasso prognostic genes were subjected to multivariate Cox regression analysis to determine the final risk model. The risk score of each patient was calculated by the following formula: risk score = *β*mRNA1 × ExpressionmRNA1 + *β*mRNA2 × ExpressionmRNA2 + ⋯+*β*mRNAn × ExpressionmRNAn.

### 2.4. Prediction Capacity of the Cox Risk Model

Using the median risk score value of the training group, patients in the training and validation groups were divided into the high- and low-risk groups. A risk score curve was drawn to visually display each patient's risk score and disease outcome. Kaplan-Meier survival curves were utilized to assess differences in OS, PFS, and DSS between patients in the high- and low-risk groups. The receiver operating characteristic (ROC) curve and the area under ROC curve (AUC) were employed to assess the predictive power of the risk score and each clinical characteristic. Finally, principal component analysis (PCA) was utilized to reduce the dimensions of four sets of genes (DEGs, cuproptosis genes, cuproptosis-related genes, and risk model genes) in the high- and low-risk groups, aiming to visualize the distinguishing ability of risk scores.

### 2.5. Functional Enrichment Analysis of Risk Scores

Differential analysis was performed on the high-risk group (*n* = 89) and the low-risk group (*n* = 89) of the entire cohort, and genes with absolute values of LogFC > 1 and *p* < 0.05 were defined as risk differential genes. Gene ontology (GO) can analyze biological processes (BP), cellular components (CC), and molecular functions (MF) of gene sets. The Kyoto encyclopedia of genes and genomes (KEGG) analysis is a systematic analysis of cellular pathways involved in genomes. GO and KEGG enrichment analyses were performed using “ClusterProfiler” and “ggplot2” packages in R (Version 3.6.3). Significant enrichment was considered eligible if *p* < 0.05. Gene set enrichment analysis (GSEA) can evaluate the regulation of pathways in the high- and low-risk groups. For GSEA in the entire cohort, GSEA software (GSEA 4.1.0) and h.all.v7.5.symbols were employed. A GMT dataset in MsigDB was used as the control group. The number of permutations was set to 1000. The screening criteria for significant enrichment pathways were false discovery rates (FDR) < 0.25 and absolute values of normalized enrichment score (NES) > 1. The nominal *p* value (NOM *p* value) was <0.05.

### 2.6. External Dataset Validation of the Cox Risk Model

GEO microarray expression data with survival information were screened for external validation of the Cox risk model [13]. The GSE62452 dataset (69 PAAD samples) and the GSE28735 dataset (45 PAAD samples) were included for analysis. The probe file GPL6244 was downloaded for the conversion of gene IDs [14, 15]. The SVA package was used to remove batch effects after merging the GSE62452 and GSE28735 datasets [16]. In total, the survival time and survival status of 107 PAAD cases were used to assess the prognostic value of the Cox risk model. The Survminer package was used to identify the optimal cutoff value, and the samples were divided into the high- and low-risk groups based on the cutoff value.

### 2.7. Differential Expression Analysis of Risk Genes

We further confirmed the differential expression of 8 risk genes at the cellular level by qRT-PCR. PAAD cell lines (ASPC-1, SW1990, BXPC3, and PANC-1) and human pancreatic cell lines were obtained from the Shanghai Institute of Nutrition and Health (Shanghai, China). RNA was isolated from samples using TRIzol, after which the PrimeScript RT Reagent Kit was employed to prepare cDNA. A StepOne Real-Time PCR Instrument (Applied Biosystems, NY, USA) was used for all qRT-PCR analyses. Relative gene expression was analyzed using the 2-*ΔΔ*Ct method, with GAPDH being employed for normalization purposes. The primers used in this study are shown in Table 1, and the analyses were repeated three times.

### 2.8. Statistical Analysis

Statistical analysis was performed using R (Version 3.6.3). Cox regression was used to evaluate each gene for the prognosis of PAAD patients by calculating the hazard ratio (HR) and its 95% confidence interval (CI). Log-rank tests were used to evaluate OS, DSS, and PFS of the three groups (entire, training, and validation). The Pearson correlation test was used to analyze correlations. The ggplot2 R package was used to visualize all data. Statistical significance was set at *p* < 0.05.

## 3. Results

### 3.1. Data Acquisition and Grouping

**Figure 1:**
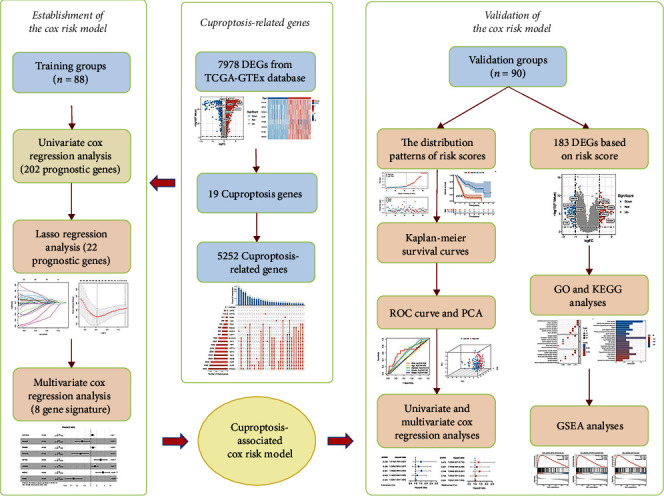
Flow chart of the study. DEG: differentially expressed gene; Lasso: Least Absolute Shrinkage and Selection Operator; ROC: receiver operating characteristic; PCA: principal component analysis; GO: gene ontology; KEGG: Kyoto encyclopedia of genes and genomes; GSEA: gene set enrichment analysis.

**Figure 2:**
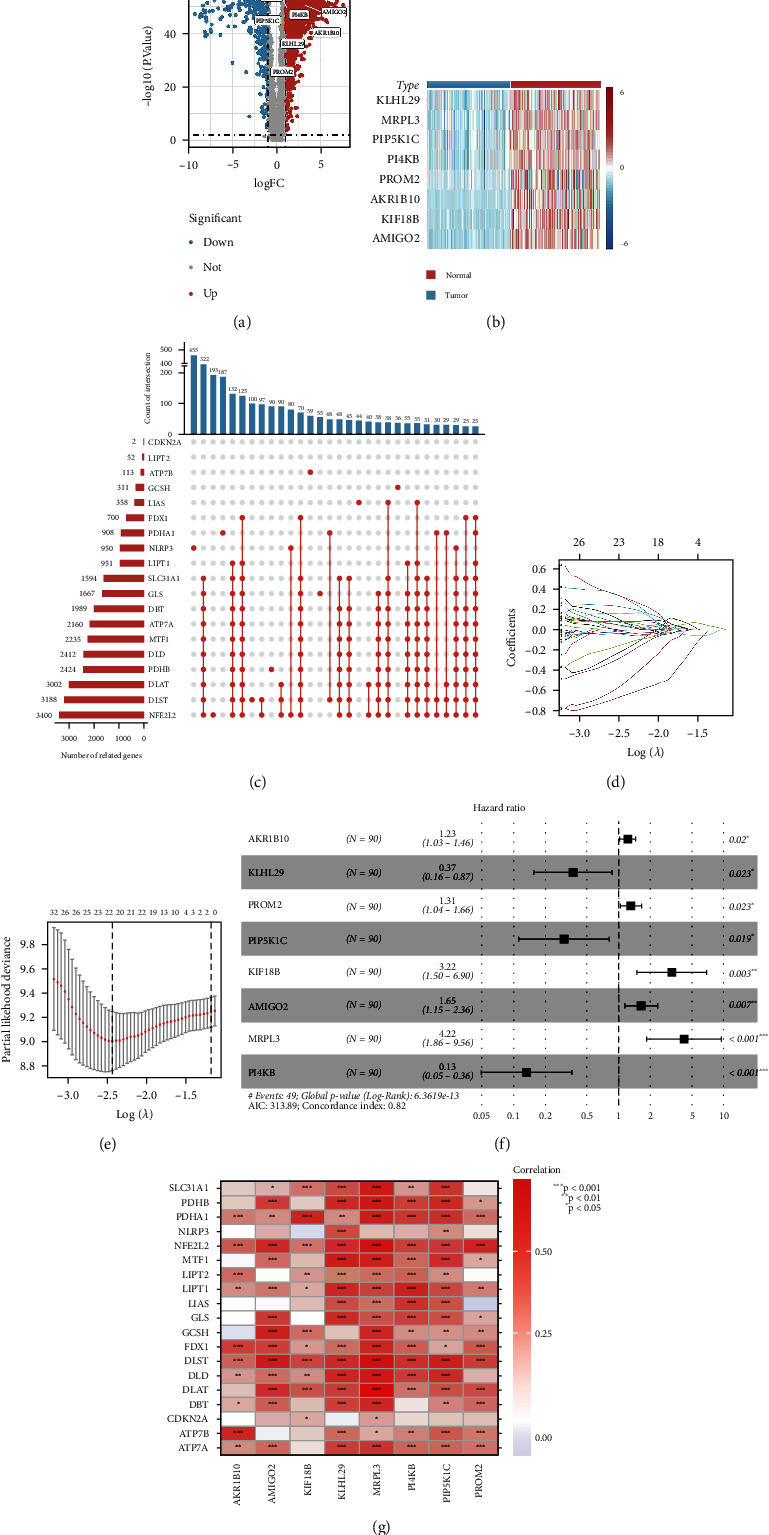
Construction process of Cox risk model: (a) differential analysis results of 178 tumor tissues and 171 normal tissues in TCGA-GTEx database; (b) the expression levels of Cox risk model genes in tumor tissues and normal tissues; (c) the upset plot shows the number and cross-linking of coexpressed genes of the 19 cuproptosis genes; (d) distribution of Lasso coefficients of the 202 potential prognostic cuproptosis-related genes in the training group; (e) the cross-validation curve of Lasso regression shows the best penalty parameter value in the training group; (f) the 8 prognostic cuproptosis-related gene signature constructs the Cox risk model in the training group; (g) the correlation between 8-gene signature and 19 cuproptosis genes. FC: fold change. ^∗^*p* < 0.05;  ^∗∗^*p* < 0.01;  ^∗∗∗^*p* < 0.001.

The present study's flow diagram is displayed in Figure 1. Based on 349 samples from TCGA-GTEx database (178 tumor tissues and 171 normal tissues), we obtained 7978 DEGs (303 downregulated genes and 7675 upregulated genes, Supplementary Table 1). The results of all difference analyses are displayed in the volcano plot (Figure 2(a)), and the expression levels of risk score genes are displayed in the heatmap (Figure 2(b)). According to R package “caret,” 178 samples were randomly assigned to the training (*n* = 88) and validation (*n* = 90) groups. The clinical characteristics of 178 PAAD patients are detailed in Table 2, without statistical differences in the clinical characteristics between the training and validation groups.

### 3.2. Establishment of the Cox Risk Model

A correlation analysis between 19 cuproptosis genes and 7978 DEGs was conducted. A total of 5252 genes with a correlation coefficient > 0.5 and *p* < 0.05 were identified as cuproptosis-related genes (Figure 2(c), Supplementary Table 2). All cuproptosis-related genes were selected for univariate Cox regression analysis, and 202 genes with prognostic values were obtained (Supplementary Table 3). The following Lasso regression analysis revealed that the best penalty parameter corresponds to 22 prognostic genes (Figures 2(d) and 2(e), Table 3). Finally, a multivariate Cox regression analysis revealed an eight prognostic cuproptosis-related gene signature for PAAD patients (Figure 2(f)). The correlation between the eight gene signatures and 19 cuproptosis genes is presented in Figure 2(g). Based on the eight-gene signature, we calculated the following risk scores: 0.205∗ExpressionAKR1B10 − 0.994∗ExpressionKLHL29 + 0.272∗ExpressionPROM2 − 1.192∗ExpressionPIP5K1C + 1.169∗ExpressionKIF18B + 0.498∗ExpressionAMIGO2 + 1.439∗ExpressionMRPL3 − 2.012∗ExpressionPI4KB.

### 3.3. Validation of the Cox Risk Model

**Figure 3:**
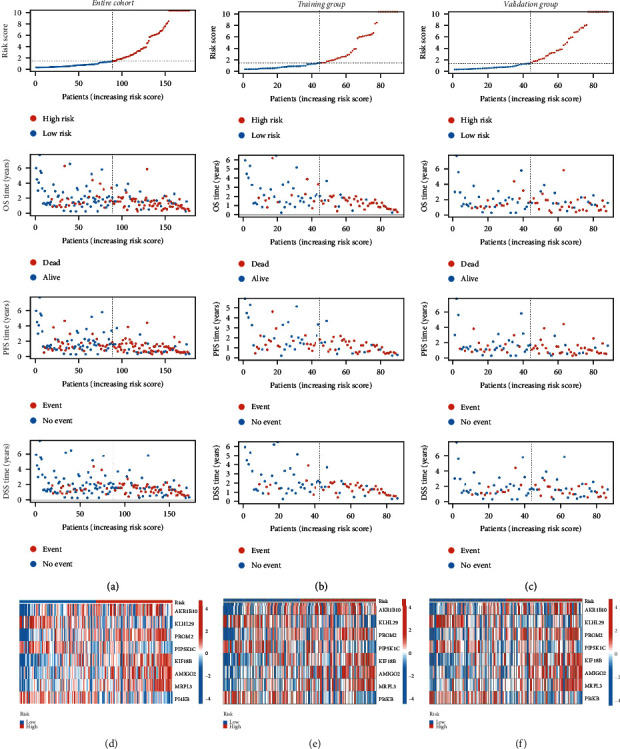
Risk scores, risk curves, and 8-gene signature heatmaps of PAAD patients in all cohorts. The distribution trend of risk score, OS time, PFS time, and DSS time in the training group (a), validation group (b), and entire cohort (c). Expression levels of 8-gene signature in the high-risk and low-risk groups in the training group (d), validation group (e), and entire cohort (f). OS: overall survival; PFS: progression-free survival; DSS: disease-specific survival.

**Figure 4:**
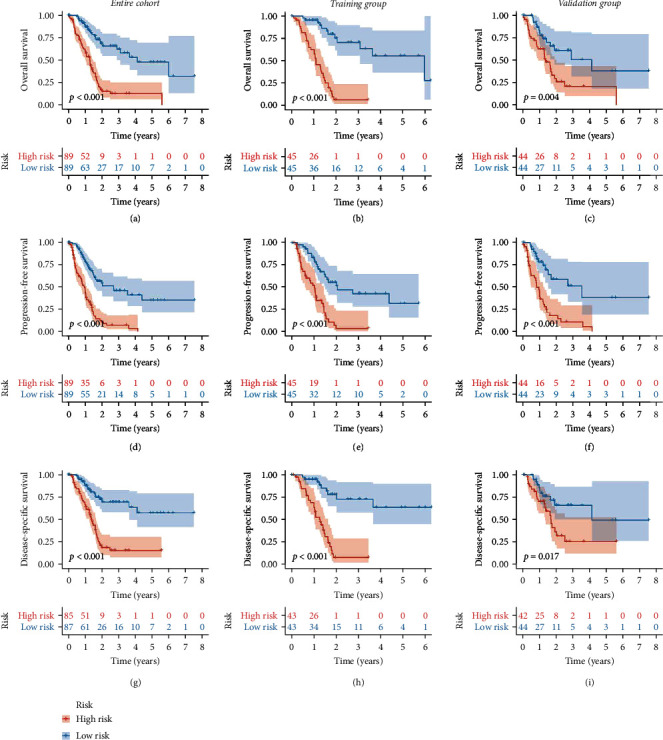
The differences in survival time of PAAD patients between high- and low-risk groups. The OS time KM curve in the training group (a), validation group (b), and entire cohort (c). The PFS time KM curve in the training group (d), validation group (e), and entire cohort (f). The DSS time KM curve in the training group (g), validation group (h), and entire cohort (i). The differences between the high- and low-risk groups were measured by log rank. PAAD: pancreatic cancer; KM: Kaplan-Meier; OS: overall survival; PFS: progression-free survival; DSS: disease-specific survival.

**Figure 5:**
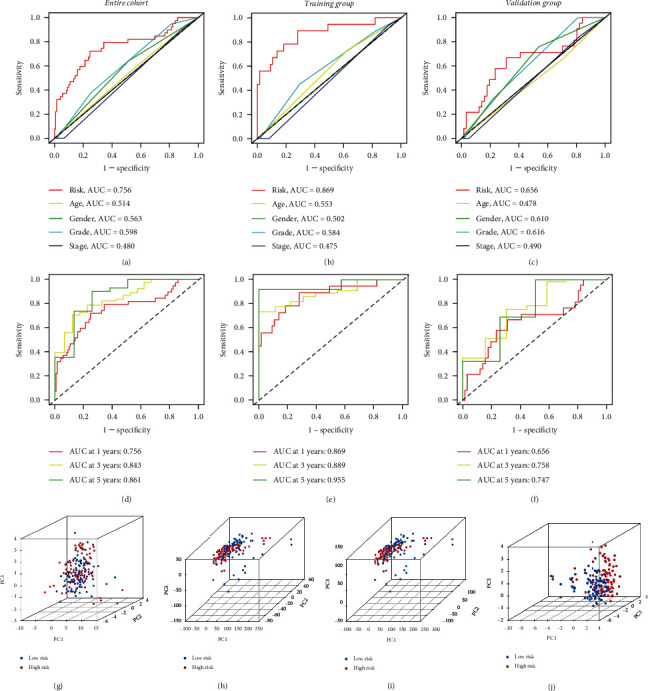
Validating the prognostic predictive power of the Cox risk models. ROC curve of the Cox risk model and clinical characteristics in the training group (a), validation group (b), and entire cohort (c). ROC curves of risk models predict 1-, 3-, and 5-year OS rates in the training group (d), validation group (e), and entire cohort (f). PCA of DEGs (g), cuproptosis genes (h), cuproptosis-related genes (i), and 8-gene signature (j) in PAAD patients. ROC: receiver operating characteristic; AUC: area under ROC curve; OS: overall survival; DEG: differentially expressed gene; PCA: principal component analysis; PAAD: pancreatic cancer.

All patients were classified into either the high-score group or the low-score group according to the training group median risk score (riskScore = 1.121), and risk scores and risk groups for all patients are listed in Supplementary Table 4. The distribution patterns of risk scores and outcome status across the training and validation groups and the entire cohort indicated that outcome events tended to occur more frequently as patient risk scores increased (Figures 3(a)–3(c)). The eight-gene signature expression profiles exhibited that AKR1B10, PROM2, KIF18B, AMIGO2, and MRP had higher expression levels in the high-risk group, and KLHL29, PI4KB, and PIP5K1C had higher expression levels in the low-risk group (Figures 3(d)–3(f)). Among three datasets, Kaplan-Meier survival curves demonstrated that the high-risk group had a shorter OS time (Figures 4(a)–4(c)), a shorter PFS time (Figures 4(d)–4(f)), and a shorter DSS time (Figures 4(g)–4(i)). The ROC curve was used to evaluate the predictive power of the Cox prognostic model. The risk score AUC of the entire cohort (0.756) was higher than other clinical characteristics (Figure 5(a)), the training group (AUC = 0.869, Figure 5(b)), and the validation group (AUC = 0.656, Figure 5(c)) to obtain consistent results. In addition, a time-dependent survival ROC curve of the risk score was well created to predict one-, three-, and five-year OS rates (Figures 5(d)–5(f)).

### 3.4. PCA and Independent Prognostic Factors

**Figure 6:**
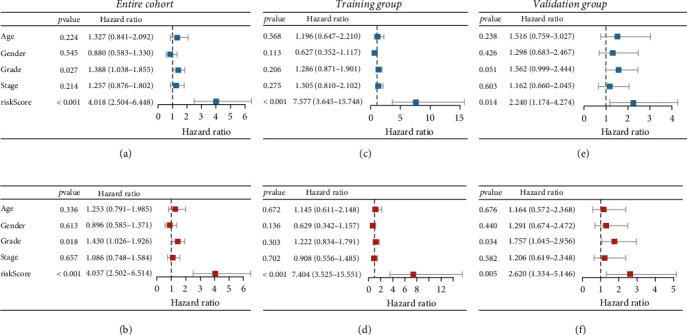
Risk score from Cox risk model is an independent predictor of prognosis in PAAD patients. Univariate Cox analysis of risk score and clinical characteristics in the training group (a), validation group (b), and entire cohort (c). Multivariate Cox analysis of risk score and clinical characteristics in the training group (d), validation group (e), and entire cohort (f). PAAD: pancreatic cancer.

PCA disclosed that compared with DEGs (Figure 5(g)), cuproptosis genes (Figure 5(h)), and cuproptosis-related genes (Figure 5(i)), the eight-gene signatures could more clearly divide all patients into two risk groups (Figure 5(j)). Univariate and multivariate Cox regression analyses were performed to evaluate the prognostic model in combination with various clinicopathological parameters. Univariate Cox regression analysis displayed that histologic grade and risk score were influencing factors for patient prognosis (Figure 6(a)). Multivariate Cox regression analysis revealed that risk score was an independent risk factor for patient prognosis (HR = 4.037, 95% CI: 2.502-6.514, *p* < 0.001) (Figure 6(b)). The same results were observed in the training group (HR = 7.404, 95% CI: 3.525-15.551, *p* < 0.001) (Figures 6(c) and 6(d)) and the validation group (HR = 2.620, 95% CI: 1.334-5.146, *p* < 0.01) (Figures 6(e) and 6(f)).

### 3.5. GO and KEGG Analyses

**Figure 7:**
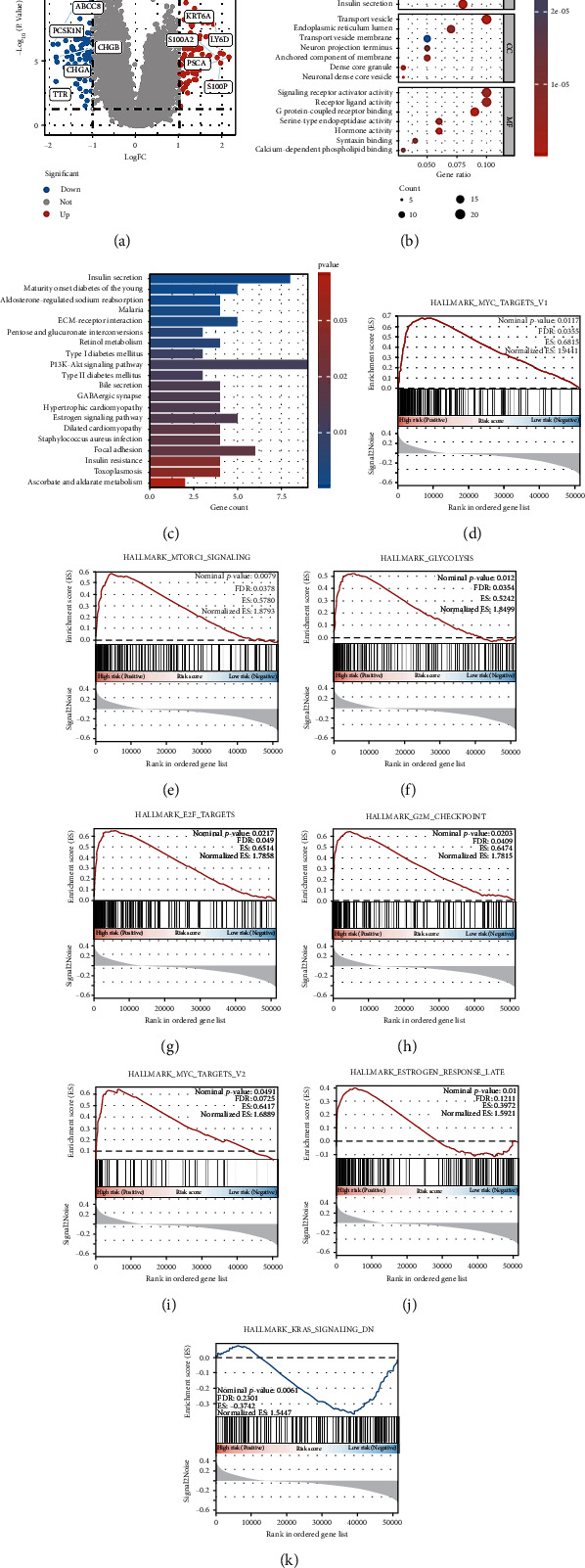
Risk score-related enrichment analysis in PAAD patients. (a) The volcano plot presents the difference analysis results between the high- and low-risk groups. GO analysis (b) and KEGG analysis (c) results of risk score-related differentially expressed genes. (d) GSEA according to the high-risk and low-risk groups of PAAD patients. PAAD: pancreatic cancer; FC: fold change; GO: gene ontology; KEGG: Kyoto encyclopedia of genes and genomes; GSEA: gene set enrichment analysis; FDR: false discovery rates; ES: enrichment score.

DEGs between the high- and low-risk groups were used for functional enrichment analysis. We obtained 94 downregulated and 89 upregulated genes (Figure 7(a), Supplementary Table 5). GO and KEGG enrichment analyses disclosed that DEGs are involved in biological effects and signaling pathways. The top 21 significant terms of GO analysis are shown in Figure 7(b), and the top 20 significant pathways of KEGG analysis are shown in Figure 7(c). For instance, molecular function includes the establishment of protein localization to extracellular regions, protein secretion, hormone transport, and hormone secretion. The cellular component contains a transport vesicle, endoplasmic reticulum lumen, anchored membrane component, and neuron projection terminus. In addition, the biological process includes signaling receptor activator activity, receptor-ligand activity, G protein-coupled receptor binding, and serine-type endopeptidase activity. KEGG analysis indicated eight cuproptosis-related genes in cancer-associated pathways, such as insulin secretion, ECM-receptor interaction, and PI3K-Akt signaling pathway.

### 3.6. GSEA

GSEA enrichment analysis was performed based on the high- and low-risk groups of the entire cohort samples, and eight signaling pathways that met the screening criteria were obtained (Figures 7(d)–7(k)). Among them, seven signaling pathways were activated in the high-risk group, including Myc targets v1, mTORC1 signaling, glycolysis, E2F targets, G2M checkpoint, Myc targets v2, and estrogen response late, while one signaling pathway was activated in the low-risk group: Kras signaling. We predicted that the difference between the high- and low-risk groups is related to pancreatic cancer pathogenesis.

### 3.7. External Validation of the Cox Risk Model and Risk Genes

**Figure 8:**
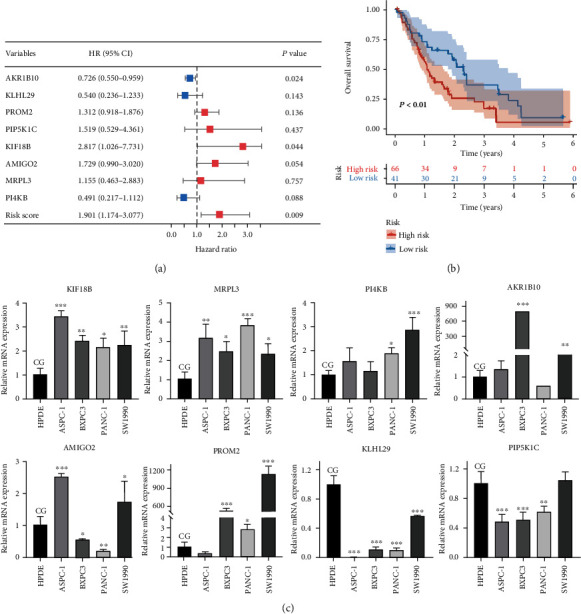
Validation of the Cox risk model and risk genes: (a) univariate Cox analysis based on the GEO dataset was used to assess the impact of the risk score and risk genes on the prognosis of PAAD patients; (b) the differences in survival time of PAAD patients between the high- and low-risk groups in the GEO dataset; (c) in vitro validation of risk gene expression by qRT-PCR in normal pancreatic cells and PAAD cells. PAAD: pancreatic cancer; CG: control group.

Survival information and risk gene expression data of 107 PAAD samples were obtained by combining the GSE62452 and GSE28735 datasets. The risk scores of all patients were calculated based on the Cox risk model, and patients were divided into the high-risk and low-risk groups according to the best cutoff values. The risk scores for all samples used for external validation are shown in Supplementary Table 6. The results of the univariate Cox regression analysis showed that AKR1B10, KIF18B, and risk score were influential factors for survival in PAAD patients in the GEO dataset (Figure 8(a)). Further results of survival analysis were consistent with previous results, with patients in the low-risk group having better survival times than the high-risk group (Figure 8(b)). Finally, we assessed the expression of 8 risk genes at the cellular level, and the results showed that AKR1B10 and PROM2 were highly expressed in PAAD cell lines, but KLHL29 was highly expressed in human pancreatic cell lines. These results suggest that risk genes have the potential as biomarkers for PAAD (Figure 8(c)).

## 4. Discussion

Copper-dependent controlled cell death in human cells is a novel cell death mechanism different from the known cell death mechanism. It occurs through the direct binding of copper ions with lipacylated components of TAC in mitochondrial respiration. Cuproptosis leads to the aggregation of ribosylated proteins and subsequent downregulation of Fe-S cluster proteins, resulting in toxic protein stress and cell death [6]. The five-year survival rate of PAAD is only 5%. Due to difficult diagnosis and poor prognosis, research in PAAD in recent years has focused on finding new biomarkers to improve prognosis [17]. Cell death-related genes are potential biomarkers for the diagnosis and prognosis of PAAD. ADP ribosylation factor 6 (a ferroptosis-related gene) functions downstream of the Kras/ERK signaling pathway and can promote proliferation and the Warburg effect in PAAD cells [18, 19]. The expression of circular RNA ATG7 (an autophagy-related circular RNA) was positively correlated with tumor diameter and lymph node infiltration. Overexpression of ATG7 promoted the proliferation, migration, and autophagy of PAAD cells, indicating that ATG7 may be a potential therapeutic target for PAAD [20]. In addition, with the development of multiomics data, a large number of genetic features and risk models provide new insights for tumor diagnosis and prognosis prediction. Among them, a number of cell death-based disease models have shown considerable clinical value, such as autophagy, ferroptosis, and pyroptosis [21, 22]. Considering the complexity of tumor biology, predictive models constructed from multiple genetic signatures are more accurate and reliable than single pathological features or single biomarkers.

The current study constructed an eight-cuproptosis-related gene signature to predict OS, DSS, and PFS in PAAD patients. First, PAAD patients were randomly assigned to the training and validation groups. Based on the training group, prognostic cuproptosis-related genes were confirmed using Lasso regression and Cox regression models. According to the risk score, patients were categorized into the high- and low-risk groups. The prognoses between the high- and low-risk groups were statistically significant, and AUC suggests the prediction ability of our risk score signature. More importantly, by comparing ROC curves of patient survival, we found that the risk score based on cuproptosis-related genes had more accurate predictive power than clinicopathological indicators as follow-up years increased. The model was validated in the validation and entire groups. In addition, the model was externally validated using the GEO database, and the results showed similar predictive power. We refer to other cell death-based risk models for comparison with the cuproptosis risk model. The AUC values of the risk model in our study led to the predictive performance of overall survival at 1, 3, and 5 years, reaching 0.75, 0.84, and 0.86, respectively. In other cell death-related studies, the AUC values for overall survival at 1, 3, and 5 years were 0.537, 0.731, and 0.852 for autophagy [21]; 0.665, 0.738, and 0.871 for ferroptosis [23]; and 0.643, 0.705, and 0.807 for pyroptosis [22], respectively. Our risk model exhibits more robust and accurate predictive performance than models of other forms of cell death.

Our risk score included eight cuproptosis-related genes: AKR1B10, KLHL29, PROM2, PIP5K1C, KIF18B, AMIGO2, MRPL, and PI4KB. As a carcinogenic protein, AKR1B10 promotes tumor occurrence and development by enhancing fat production and is involved in pancreatic carcinogenesis via modulating the Kras-E-cadherin pathway [24, 25]. PROM2, a transmembrane glycoprotein, is upregulated in PAAD cells, and higher PROM2 expression is associated with a poor prognosis in PAAD patients [26]. PIP5K1C is a lipid kinase that regulates adhesion dynamics and cell attachment through the site-specific formation of phosphatidylinositol-4,5-diphosphate. A study disclosed that PIP5K1C is a negative regulator of cell migration and invasion and that the phosphorylation status of PIP5K1C may serve as an indicator of adenocarcinoma invasion [27]. KIF18B is highly expressed in human PAAD tissues and is associated with poor prognosis and clinical characteristics of PAAD patients, such as tumor size and TNM stage. In PAAD cells, KIF18B could bind to the cell division cycle-related promoter region 8, thereby activating its transcription [28]. The interaction of extracellular matrix receptors causes cancer metastasis, and AMIGO2 promotes the adhesion of tumor cells to endothelial cells, accelerating this process [29].

We performed an enrichment analysis based on the risk scores of pancreatic cancer patients. GSEA revealed that various signaling pathways involved in cancer occurrence and development are enhanced in the high-risk group. The Myc oncoprotein family is involved in regulating metabolic reprogramming and providing sufficient energy for cancer cell proliferation [30]. Aerobic glycolysis is a metabolic modality exhibited in various tumors, and there is evidence that mTORC1 signaling regulates aerobic glycolysis by upregulating hypoxia-inducible factor- (HIF-) 1*α* [31]. mTORC1 signaling is also involved in promoting lipid metabolism and nucleotide synthesis in tumor cells [32]. The E2F signaling pathway is involved in the transcriptional machinery driving cell cycle progression, and E2F activity is abnormally increased in various tumors, leading to uncontrolled proliferation [33]. Similarly, the enrichment analysis of DEGs between the high- and low-risk groups involves tumor-related mechanisms. Studies have demonstrated that PI3K/AKT pathway inactivation in PAAD cells can inhibit mutant p53, thus inducing S-phase arrest and apoptosis of PAAD cells [34]. In addition, there is a close relationship between extracellular matrix (ECM) accumulation and cancer progression, and pancreatic stellate cells play a role in this process. Interactions between extracellular matrix receptors alter the tumor microenvironment in PAAD, accelerating its progression, including angiogenesis, invasion, and metastasis [35].

This study has limitations. First, some incomplete information was deleted, which led to a certain degree of information deviation. Next, the concept of cuproptosis was only recently proposed, and sufficient literature cannot be obtained to clarify the possible mechanism of an eight-gene signature involved in cuproptosis. Multicenter, large-scale clinical trials of prediction models require further validation.

## 5. Conclusion

In conclusion, we constructed a prognostic model containing eight cuproptosis-related genes and analyzed their MF in PAAD. Our study highlights that the risk score we constructed can be used as a marker of outcome in PAAD patients. Our findings provide new insights into the role of cuproptosis-related genes in PAAD pathogenesis and as potential biomarkers for PAAD diagnosis and treatment.

## Figures and Tables

**Table 1:** Primers for risk genes.

Gene	Direction	Sequence
AKR1B10	Forward primer	GGCAACCATACTCAGCTTCA
	Reverse primer	TGGGACATGAGTGGAGGTAG
KLHL29	Forward primer	CATCTCCAAGGACGACTTCATC
	Reverse primer	GTCAACCACATTGAGCAGGTA
PROM2	Forward primer	GGGCCACAGACTGCAAGTT
	Reverse primer	AGCTCATTCAGTAGGGCCTTTA
PIP5K1C	Forward primer	AGACCGTCATGCACAAGGAG
	Reverse primer	CAGTACAGCCCATAGAACTTGG
KIF18B	Forward primer	TGCTCAAAGACTCCCTCGG
	Reverse primer	GTTGTACGTGTCCTCGTAGGT
AMIGO2	Forward primer	AGCATTTCCACGGGCAGTTT
	Reverse primer	CCGTCTTCAGCTTATTGGACGA
MRPL3	Forward primer	CAAGGATGGTCAAAAGCATGTG
	Reverse primer	GCAATCCAAGTTCCCGGTAAAA
PI4KB	Forward primer	CCTGCTCAACCATAAGCTCCC
	Reverse primer	AGTTTTCTACGGACCTCGTACT

**Table 2:** Characteristics of pancreatic cancer patients.

Characteristics	No. (%)			*p* value
	Entire cohort (*n* = 178)	Training group (*n* = 88)	Validation group (*n* = 90)	
Age (years)				0.4868
≤60	58 (32.58%)	26 (29.55%)	32 (35.56%)	
>60	120 (67.42%)	62 (70.45%)	58 (64.44%)	
Gender				0.2222
Female	80 (44.94%)	35 (39.77%)	45 (50%)	
Male	98 (55.06%)	53 (60.23%)	45 (50%)	
Histologic grade				0.3903
G1	31 (17.42%)	14 (15.91%)	17 (18.89%)	
G2	95 (53.37%)	51 (57.95%)	44 (48.89%)	
G3	48 (26.97%)	20 (22.73%)	28 (31.11%)	
G4	2 (1.12%)	1 (1.14%)	1 (1.11%)	
Unknown	2 (1.12%)	2 (2.27%)	0 (0%)	
Pathologic stage				0.2498
Stage I	21 (11.8%)	15 (17.05%)	6 (6.67%)	
Stage II	146 (82.02%)	68 (77.27%)	78 (86.67%)	
Stage III	3 (1.69%)	1 (1.14%)	2 (2.22%)	
Stage IV	5 (2.81%)	2 (2.27%)	3 (3.33%)	
Unknown	3 (1.69%)	2 (2.27%)	1 (1.11%)	
T stage				0.6067
T1	7 (3.93%)	5 (5.68%)	2 (2.22%)	
T2	24 (13.48%)	14 (15.91%)	10 (11.11%)	
T3	142 (79.78%)	67 (76.14%)	75 (83.33%)	
T4	3 (1.69%)	1 (1.14%)	2 (2.22%)	
Unknown	2 (1.12%)	1 (1.14%)	1 (1.11%)	
M stage				0.3219
M0	79 (44.38%)	44 (50%)	35 (38.89%)	
M1	5 (2.81%)	2 (2.27%)	3 (3.33%)	
Unknown	94 (52.81%)	42 (47.73%)	52 (57.78%)	
N stage				0.8822
N0	50 (28.09%)	25 (28.41%)	25 (27.78%)	
N1	123 (69.1%)	60 (68.18%)	63 (70%)	
Unknown	5 (2.81%)	3 (3.41%)	2 (2.22%)	

**Table 3:** Cuproptosis-related prognostic genes obtained from Lasso and univariate Cox regression model.

Gene symbol	Lasso coefficient	HR	HR.95L	HR.95H	*p* value
AKR1B10	0.044	1.201	1.051	1.373	0.007
AMIGO2	0.040	1.445	1.128	1.851	0.004
ARRB2	-0.045	0.594	0.418	0.844	0.004
DSG2	0.085	1.638	1.180	2.274	0.003
ENPP2	-0.018	0.754	0.623	0.913	0.004
FGF2	0.222	1.508	1.135	2.003	0.005
FIG4	-0.199	0.525	0.330	0.837	0.007
FRMD5	0.080	2.099	1.353	3.257	0.001
GRAMD4	-0.034	0.627	0.446	0.880	0.007
ITGA3	0.033	1.405	1.099	1.796	0.007
KIF18B	0.081	1.846	1.188	2.868	0.006
KIF2C	0.068	1.720	1.199	2.467	0.003
KLHL29	-0.646	0.429	0.270	0.682	<0.001
KNL1	0.159	2.403	1.394	4.141	0.002
MRPL3	0.316	2.505	1.251	5.016	0.010
NUP37	0.320	2.344	1.294	4.246	0.005
PBX3	-0.007	0.567	0.393	0.818	0.002
PI4KB	-0.528	0.505	0.323	0.790	0.003
PIP5K1C	-0.255	0.574	0.381	0.867	0.008
PROM2	0.089	1.402	1.147	1.715	0.001
RAD51C	-0.140	0.453	0.285	0.722	0.001
SEMA4D	-0.002	0.575	0.405	0.815	0.002

## Data Availability

The raw data presented in this study can be found in online repositories: UCSC, https://xena.ucsc.edu. Data processing results are available in the article and supplementary file.

## Supplementary Materials

Supplementary Materials Supplementary Table 1: 7978 DEGs between 178 tumor tissues and 171 normal tissues. Supplementary Table 2: 5252 cuproptosis-related genes based on 19 cuproptosis genes. Supplementary Table 3: 202 prognostic genes obtained by univariate Cox regression analysis. Supplementary Table 4: the risk scores and risk groups for all patients. Supplementary Table 5: 183 DEGs between high- and low-risk groups. Supplementary Table 6: risk scores for samples sourced from the GSE62452 and GSE28735 datasets.
