# Supplementary material for: A Novel Cuproptosis-Associated Gene Signature to Predict Prognosis in Patients with Pancreatic Cancer
Source: Biomed Res Int. 2023 Jan 18;2023:3419401. doi: 10.1155/2023/3419401 (PMC9876676; doi:10.1155/2023/3419401)
Supplement: Supplementary Materials — Supplementary Table 1: 7978 DEGs between 178 tumor tissues and 171 normal tissues. Supplementary Table 2: 5252 cuproptosis-related genes based on 19 cuproptosis genes. Supplementary Table 3: 202 prognostic genes obtained by univariate Cox regression analysis. Supplementary Table 4: the risk scores and risk groups for all patients. Supplementary Table 5: 183 DEGs between high- and low-risk groups. Supplementary Table 6: risk scores for samples sourced from the GSE62452 and GSE28735 datasets. [file 3419401.f1.zip › 3419401.f1.pdf]

gene	logFC	pValue
CLPS	-9.25209	1.48E-48
CPA1	-8.69179	1.94E-47
CELA3B	-8.52947	1.41E-48
CELA3A	-8.40151	1.01E-47
GP2	-8.3084	5.62E-52
PLA2G1B	-8.25912	5.26E-50
CELA2A	-8.1286	1.38E-41
CTRB1	-8.10072	4.06E-49
PNLIP	-8.05046	5.31E-40
CEL	-8.03896	1.2E-49
CTRB2	-7.99342	3.2E-47
SYCN	-7.93535	1.46E-46
PRSS1	-7.93288	1.42E-45
AMY2A	-7.83256	1.4E-40
PNLIPRP1	-7.82586	3.33E-53
CPA2	-7.61758	1.53E-49
REG1B	-7.4843	1.46E-42
CPB1	-7.39863	1.3E-41
CTRC	-7.35926	2.12E-44
PRSS2	-7.23353	7.17E-50
RBPJL	-7.03075	1.82E-54
CUZD1	-6.6079	8.93E-51
CTRL	-6.36575	1.54E-46
CELA2B	-6.35516	1.86E-40
REG1A	-6.18736	2.22E-47
AQP12B	-5.96771	8.6E-54
PDIA2	-5.82527	2.42E-50
GNMT	-5.5072	1.57E-51
AMY2B	-5.45595	1.01E-43
SERPINI2	-5.44602	3.87E-50
KLK1	-5.36301	5.02E-50
AQP8	-5.24655	3.3E-48
BHLHA15	-5.17202	7.95E-52
ALB	-5.09669	3.76E-51
REG3G	-4.98384	8.21E-30
AQP12A	-4.92734	8.39E-53
FGL1	-4.71736	1.08E-43
PRSS3	-4.62833	3.89E-48
TMEM52	-4.3178	2.36E-52
SLC39A5	-4.28017	3.45E-51
PLIN5	-4.1979	8.43E-54
GSTA2	-4.15804	3.97E-50
BRSK2	-4.02523	1.07E-52
ARHGDIG	-3.92536	1.37E-52
CBS	-3.77349	1.74E-52
ERP27	-3.75465	2.45E-45
PM20D1	-3.59706	1.58E-45
GOLGA8B	-3.52939	2.66E-54
PTF1A	-3.47342	7.22E-52
SPX	-3.43858	8.42E-54
SPINK1	-3.42659	2.17E-47
CLPSL1	-3.41421	2.04E-26
PGGHG	-3.32421	1.35E-46
HPN	-3.314	4.19E-49
GATM	-3.17244	4.36E-49
GPHA2	-3.16013	4.6E-42
ACADL	-3.14275	3.81E-54
EGF	-3.13826	6.98E-52

REG3A	-3.07756	1.16E-09
BNIP3	-3.05917	1.7E-49
GUCA1C	-3.05344	1.79E-51
P2RX1	-3.05334	5.07E-53
KIRREL2	-3.04353	3.86E-50
SLC30A2	-3.01358	2.61E-43
TRIM50	-2.97551	6.42E-53
TMED6	-2.94533	3.39E-41
RBM20	-2.92231	1.28E-53
ANPEP	-2.86351	2.36E-42
NPHS1	-2.85748	2.31E-54
GLS2	-2.83508	2.68E-49
IGFN1	-2.66009	2.08E-52
SLC43A1	-2.63924	9.06E-52
AOX1	-2.62254	7.3E-47
DPEP1	-2.58713	4.83E-48
AC011498.	-2.57743	1.1E-53
MAT1A	-2.57697	1.55E-51
SLC38A3	-2.573	5.75E-47
PAIP2B	-2.46207	1.24E-50
ERO1B	-2.43631	4.97E-40
IL22RA1	-2.43465	2.01E-47
TPST2	-2.42165	8.54E-50
ALKAL2	-2.3845	5.81E-42
CHAD	-2.36871	1.55E-48
KLF15	-2.35593	8.36E-52
SLC22A31	-2.30973	1.93E-35
CPA4	-2.30143	1.56E-53
EPB41L4B	-2.27626	2.38E-47
BEGAIN	-2.26658	9.12E-52
CSDC2	-2.21697	9.66E-38
CBSL	-2.20201	1.28E-25
KSR1	-2.19782	1.54E-50
RIC3	-2.19044	4.42E-46
CAMK2N2	-2.18158	6.28E-42
RNF186	-2.13899	3.59E-41
NRG4	-2.12299	1.19E-46
TRPV6	-2.11852	1.49E-33
MKNK1	-2.11555	7.79E-55
AZGP1	-2.10794	2.29E-39
PGA3	-2.06222	1.21E-29
GPT2	-2.04959	2.3E-46
ADHFE1	-2.04221	3.75E-52
VIPR2	-2.0374	9.19E-51
F11	-2.03543	2.28E-43
C2CD4B	-2.00175	6.95E-38
ATP4A	-1.99262	1.72E-53
SLC16A12	-1.96856	2.38E-38
AKR7A3	-1.96126	1.7E-25
GDF10	-1.94322	8.78E-52
PSAT1	-1.93735	2.13E-40
CA4	-1.90971	3.6E-31
CERS4	-1.90628	1.51E-52
PDCD4	-1.89653	5.18E-49
KIF1A	-1.87186	6.97E-37
MT1G	-1.8676	4.05E-18
COCH	-1.86424	1.59E-34
SPSB4	-1.86038	4.66E-51
HOMER2	-1.84237	4.48E-42

PAK3	-1.83035	4.98E-42
SLC38A5	-1.8278	2.79E-33
ITIH4	-1.82278	9.47E-27
MT1H	-1.79581	5.98E-18
GAMT	-1.78945	7.4E-43
SARDH	-1.77802	5.31E-50
PGA4	-1.77601	2.01E-28
CBFA2T3	-1.76544	1.72E-46
RNF212	-1.73285	8.51E-45
NR5A2	-1.71286	3.06E-38
SEL1L	-1.70327	9.1E-45
BICDL2	-1.68569	3.98E-42
MCOLN3	-1.68348	5.63E-45
CYP3A4	-1.64939	1.82E-11
AC006254.	-1.64916	7.55E-53
SERPINA3	-1.6482	2.66E-13
C5	-1.64799	9.46E-48
ANKRD53	-1.64632	2.74E-54
SYBU	-1.64216	3.39E-31
ALDOB	-1.64078	9.01E-13
SLC17A4	-1.63951	6.52E-35
SLC25A45	-1.62926	1.92E-49
FITM1	-1.62388	4.7E-50
POMC	-1.61845	1.09E-39
GOLGA8A	-1.6073	1.9E-37
ONECUT1	-1.60147	2.7E-35
ALDH1L2	-1.58802	2.12E-45
FBXW12	-1.58174	1.65E-52
GRB14	-1.58005	2.51E-41
FLRT2	-1.57853	1.72E-33
TEX11	-1.5733	1.22E-41
SLC1A2	-1.57065	3.03E-50
IZUMO1	-1.56443	1.34E-52
WNK2	-1.55369	3.1E-32
GJC3	-1.54733	9.56E-47
CRACR2B	-1.5426	5.81E-35
EPO	-1.54242	1.92E-51
CYP3A43	-1.52931	2.21E-48
GOLGA8N	-1.52484	2.6E-51
ALDH1L1	-1.52035	4.78E-27
AMHR2	-1.51972	1.4E-44
LMO3	-1.51847	1.38E-37
KCNK3	-1.51582	4.5E-31
CCDC110	-1.51236	2.69E-48
AL049839.:	-1.50832	8.99E-11
ZNF98	-1.50719	5.14E-46
PLIN4	-1.50044	6.84E-25
SLC25A27	-1.49328	9.7E-39
KCNJ16	-1.48866	9.61E-21
MTUS2	-1.48661	1.16E-40
SHC2	-1.48166	7.38E-33
BACE1	-1.48019	1.58E-42
XBP1	-1.47505	2.36E-42
PRODH2	-1.47129	6.27E-51
TTYH1	-1.46973	7.11E-32
AASS	-1.46924	8.38E-39
FGFR3	-1.46723	9.43E-32
ABCA5	-1.46393	3.71E-43
LPAR3	-1.45552	1.45E-49

IMPA2	-1.44996	2.31E-42
FUT1	-1.44906	1.73E-43
CCKBR	-1.44454	8.22E-39
SFRP5	-1.42975	2.11E-09
CHRD	-1.42852	8.37E-38
GRPR	-1.41674	2.21E-44
BTG2	-1.39957	2.59E-26
INS	-1.39765	0.030829
RAB26	-1.38931	4.19E-28
SLC2A14	-1.38844	1.35E-50
RGN	-1.37888	3.59E-36
PNPLA7	-1.37769	3.05E-44
C2orf92	-1.37472	9.8E-46
GCAT	-1.36953	3.9E-42
CTNND2	-1.36737	1.53E-33
PLEKHH1	-1.36099	3.54E-38
NRTN	-1.34872	4.62E-44
IFITM5	-1.34354	6.51E-46
SLC7A2	-1.34002	6.71E-27
TUBG2	-1.3399	2.1E-31
TECPR1	-1.33871	2.61E-38
SLC4A4	-1.32732	3.16E-14
RNPC3	-1.32227	3.49E-46
UMOD	-1.31347	7.77E-50
PAN2	-1.30041	7.76E-42
DMD	-1.29616	5.32E-42
IFRD1	-1.28899	2.72E-46
COQ8A	-1.2867	1.67E-46
MYLK2	-1.27525	2.96E-34
SEMA6D	-1.26589	9.57E-40
MAPK8IP1	-1.26272	7.34E-38
PABPC1L	-1.25635	7.29E-32
EPHX2	-1.2561	2.64E-37
GPR150	-1.25501	1.15E-44
GRB10	-1.25017	1.65E-41
FBXO2	-1.24406	2.45E-25
LENG8	-1.24286	8.37E-37
LHB	-1.24057	7.96E-27
GATA4	-1.24019	1.77E-26
ACTA1	-1.23953	3.61E-34
GSTM2	-1.23399	4.41E-36
MATN4	-1.22874	3.78E-48
TP53INP1	-1.22778	3.71E-36
DTNA	-1.22581	8.29E-35
SPACA3	-1.21827	1.1E-38
MYCL	-1.21651	5.28E-26
ZDHHC11E	-1.20923	1E-34
OGT	-1.20757	3.87E-45
DPP10	-1.20504	1.02E-44
TRHDE	-1.19604	1.37E-41
INPP5J	-1.19563	2.33E-42
CLPSL2	-1.1926	1.71E-41
NRCAM	-1.19045	7.19E-27
ECHDC3	-1.18243	1.05E-34
NPY1R	-1.18207	2.7E-26
LHFPL5	-1.18145	3.42E-49
PDK4	-1.18035	3.22E-12
LSMEM1	-1.1775	7.5E-43
ARHGEF4	-1.17447	1.66E-33

CHRM3	-1.17332	5.6E-37
RGS11	-1.17138	2.72E-31
TCEA3	-1.16738	6.34E-36
FBXL8	-1.16158	1.25E-34
TTLL7	-1.15918	1.59E-36
GSTA1	-1.15791	1.85E-11
BTNL9	-1.15774	5.85E-27
RPL3L	-1.15315	1.47E-31
NAA16	-1.1512	6.12E-50
CCDC198	-1.15104	6.3E-18
LARGE2	-1.14975	9.09E-35
FAM153B	-1.14947	1.74E-24
HPCAL4	-1.14645	1.13E-37
WDFY2	-1.14373	2.1E-48
DEFB1	-1.13074	8.89E-11
ECE2	-1.12742	1.86E-38
IZUMO4	-1.12026	3.38E-34
CYP2E1	-1.11841	1.91E-35
AMBP	-1.11607	1.31E-05
RYR2	-1.11142	3.06E-43
MYH7	-1.11131	1.97E-53
TMEM266	-1.10827	8.94E-52
MUC6	-1.10144	1.11E-06
PHYHD1	-1.10031	7.93E-27
CLCNKA	-1.09981	3.56E-45
TMEM131l	-1.09928	8.56E-34
TPRN	-1.09444	2.37E-32
COMTD1	-1.09265	1.06E-35
SYTL1	-1.08827	2.06E-31
PSMD6	-1.08286	4.2E-44
MPV17L	-1.08201	1.33E-38
FAM3B	-1.08178	5.03E-17
BCAT1	-1.08029	3.69E-27
FAM153A	-1.07874	3.46E-25
SLC39A14	-1.07459	5.73E-36
TMEM97	-1.07297	6.83E-27
RGL3	-1.06601	2.73E-23
SLC16A10	-1.06347	1.06E-33
SLC6A16	-1.0567	4.9E-43
BANF2	-1.05618	1.3E-41
MYEF2	-1.05299	2.22E-28
TSPOAP1	-1.05244	3.9E-29
COL28A1	-1.05221	6.05E-27
HSF4	-1.05061	3.72E-20
PILRB	-1.04807	2.33E-23
KCNJ5	-1.04588	9.74E-26
MAT2A	-1.04263	2.62E-27
CCDC196	-1.03528	3.82E-47
AADAC	-1.03476	7.42E-12
TM7SF2	-1.02929	2.58E-19
ABAT	-1.02875	4.66E-24
CATSPERB	-1.02674	8.39E-19
DNASE1	-1.02631	9.67E-24
PWWP3B	-1.02107	8.31E-24
GMNN	-1.01876	1.63E-39
SIX5	-1.01794	6.17E-39
VXN	-1.01321	2.01E-38
MT1X	-1.01252	1.22E-15
SNTG2	-1.01185	5.25E-35

PLEKHH3	-1.01121	2.98E-27
CXCL2	-1.01098	6.16E-06
CDRT4	-1.0104	7.5E-22
FSCN2	-1.00639	3.21E-36
PEX5L	-1.00307	2.31E-50
FO681492.	-1.00174	1.39E-45
SCAMP5	-1.00027	2.83E-26
CKM	-1.00014	3.7E-26
CSAD	-0.99935	1.36E-28
CPOX	1.000335	7.45E-43
EPN2	1.000389	3.79E-42
RINL	1.000846	4.33E-37
SMN1	1.001055	2.73E-42
ZNF350	1.001243	2.59E-42
IQSEC1	1.001305	2.52E-40
MBNL3	1.001699	2.89E-41
LRRC41	1.001797	1.63E-43
TPP2	1.002023	3.33E-43
CLEC12A	1.002181	2.04E-30
CALY	1.002292	1.2E-08
NOC4L	1.00245	1.91E-36
CTSG	1.002556	4.35E-20
DGKQ	1.002711	5.03E-37
VEZF1	1.00274	1.69E-37
ARL17B	1.003	2.74E-42
PCSK7	1.003054	6E-39
TSPAN13	1.003095	5.21E-37
GSTM4	1.003238	6.18E-28
BLOC1S6	1.003529	3.07E-40
SNRNP200	1.003599	3.71E-43
FP565260.(	1.004	2.27E-41
SART3	1.005033	3.58E-43
NR1D2	1.005085	1.1E-35
HSPA2	1.005221	9.08E-28
NUDT3	1.005469	1.51E-43
CXorf21	1.005532	4.64E-45
PCDHGB2	1.005705	1.56E-34
RASA2	1.005725	1.08E-37
RPL23A	1.005759	1.76E-45
TMEM150(	1.005836	3.2E-31
AP4S1	1.005878	2.08E-44
ANKRD13(	1.005934	3.43E-45
UGDH	1.006111	4.6E-25
GC	1.006228	1.4E-07
PDCD2L	1.006251	1.8E-47
ICE2	1.006305	8.57E-45
MDM2	1.00637	2.04E-30
LAT	1.006387	6.46E-26
NUP50	1.006423	4.4E-43
JPH2	1.006469	1.49E-44
CHUK	1.006503	1.72E-46
CNOT10	1.006661	7.55E-45
TMEM86B	1.006762	7.03E-30
DUSP4	1.00684	8.19E-23
DKK4	1.006936	5.26E-53
ATPAF2	1.006949	3.38E-44
SMIM13	1.007095	1.75E-50
FYTTD1	1.007097	1.11E-31
EXO5	1.007194	1.3E-42

HIPK1	1.007419	6.37E-40
PPP1CC	1.007459	1.97E-42
NARS2	1.007747	1.57E-47
C19orf38	1.007758	5.18E-44
ADD3	1.007948	1.84E-29
MINPP1	1.007973	4.5E-45
BPTF	1.008113	1.34E-38
DCAF1	1.008123	2.61E-46
MED14	1.00822	3.94E-43
ZNF586	1.008223	4.36E-43
PTPN22	1.008272	7.15E-35
TMED9	1.008329	4.15E-43
LAS1L	1.008364	4.39E-49
TRAK2	1.008386	8.03E-40
MSANTD3	1.008451	6.35E-50
HBP1	1.008553	7.7E-40
GUF1	1.00859	9.17E-49
FXR1	1.008707	1.02E-43
TMEM107	1.008757	2.5E-40
MRPS31	1.008799	4.94E-43
ARNT2	1.00905	3.71E-33
ZNF488	1.009069	4.76E-56
LRRC8E	1.009086	3.17E-36
DCHS1	1.009151	4.54E-32
QTRT2	1.009218	4.98E-44
SLCO2A1	1.009352	4.19E-21
TMEM209	1.009368	2.26E-44
CLN8	1.009425	1.83E-43
PRRC1	1.009478	2.35E-44
SELENON	1.009624	2.17E-37
DCUN1D3	1.009713	1.35E-43
ALG1	1.009734	4.18E-50
HUS1	1.009919	1.31E-45
SUPT5H	1.010045	1.29E-40
PCGF1	1.010172	7.22E-42
ZHX2	1.010278	6.02E-40
ZNF329	1.010359	1.33E-40
DCUN1D5	1.010533	1.83E-41
DDX21	1.010583	3.64E-28
RFC3	1.010701	1.16E-45
ILK	1.010757	1.36E-39
MSX1	1.010823	4.14E-39
SHISAL2A	1.010852	1.16E-47
CASTOR1	1.010855	2.61E-36
IRF3	1.010861	2.68E-33
ZSWIM3	1.010872	8.45E-52
TAF2	1.010917	2.26E-40
TRAM1	1.011018	1.04E-35
CD40	1.011733	9.96E-29
LARGE1	1.011865	3.32E-42
CASP4	1.011915	1.21E-28
BORCS5	1.011959	4.59E-45
NAA10	1.01232	8.69E-46
GSTCD	1.012376	6.13E-50
GJB6	1.012659	6.95E-42
PLCD1	1.012784	1.16E-38
HNRNPA1l	1.013093	2.52E-34
KAT6A	1.013355	1.78E-40
PPP1R15B	1.013459	7.03E-43

ZC3H15	1.013627	2.7E-45
RPRD1B	1.013867	1.01E-43
GPATCH11	1.013904	2.84E-48
FOXN3	1.014081	5.88E-34
CHMP4C	1.014085	9.61E-29
ZNF552	1.014111	1.18E-43
PRR14L	1.014223	3.06E-46
ITGB3BP	1.014366	1.18E-36
WDR47	1.014514	5.88E-43
COPZ1	1.014728	3.39E-47
PMPCA	1.014806	1.65E-46
PPFIA1	1.014843	1.35E-43
CEP72	1.015392	2.1E-44
GOLT1B	1.015587	1.36E-41
ARHGAP12	1.015904	2.56E-40
ASB2	1.015965	1.27E-43
ZDHHC6	1.015973	7.22E-44
KLHL29	1.015991	7.66E-41
COX5A	1.016209	6.13E-44
SURF2	1.016271	1.93E-42
AP4M1	1.016636	1.32E-42
HFE	1.016808	1.37E-38
RTL5	1.01686	4.57E-32
N4BP3	1.016863	1.11E-52
AP3S1	1.017384	4.71E-42
HIVEP2	1.017412	3.56E-38
INTS9	1.01742	1.7E-44
ENPEP	1.01761	1.38E-35
STAB1	1.017781	9.86E-21
IPPK	1.017909	4.22E-46
ZNF184	1.018276	1.07E-48
TRRAP	1.018313	2.81E-43
IMMP2L	1.018338	3.78E-44
ZNF707	1.018406	9.07E-44
EEF2KMT	1.018513	6.25E-49
ALS2	1.018753	8.35E-46
AP5Z1	1.018862	7.1E-33
ZNF286A	1.01892	1.68E-40
KLK11	1.019001	6.86E-15
USF1	1.019069	4.04E-38
SLC30A5	1.019085	2.58E-45
PIK3CG	1.019214	8.14E-46
CACUL1	1.019706	1.72E-46
LIN9	1.019762	1.36E-52
TMTC1	1.019776	1.87E-32
ZMYM5	1.019814	4.76E-43
KATNBL1	1.019919	8.42E-41
ZNF274	1.020171	7.51E-37
RPL36A	1.020276	2E-39
MBNL2	1.020327	6.7E-33
ZNF385D	1.020661	3.86E-31
CAVIN2	1.020778	8.72E-23
FER	1.021169	1.86E-48
TLDC2	1.021175	9.73E-25
UNC50	1.021323	1.67E-47
SPIDR	1.02143	3.64E-38
GINS4	1.021465	4.29E-50
NSRP1	1.021654	8.88E-44
RFWD3	1.021853	1.58E-39

AP5S1	1.022022	1.32E-46
SH3BP5L	1.022126	7.16E-42
NT5DC1	1.022577	3.21E-37
DYRK1A	1.022627	1.29E-42
MFSD14A	1.022674	2.77E-42
ZBTB41	1.022692	5.53E-48
NOC2L	1.023085	8.02E-43
ZFP90	1.023222	3.42E-43
NDUFS2	1.023271	7.77E-44
TLN2	1.023277	7.67E-41
BCKDHA	1.023281	2.32E-41
HADH	1.023319	5.48E-42
TRMT61A	1.023336	2.43E-40
PPP1R16B	1.023432	1.31E-34
BRD3OS	1.02344	4.3E-41
DPM1	1.023545	1.44E-45
GMCL1	1.023684	7.45E-43
FBXO38	1.023889	4.49E-44
CDK16	1.023956	2.83E-43
PQBP1	1.024088	1.29E-45
RNF157	1.024304	8.43E-36
NKIRAS1	1.024321	4.62E-44
FASTKD2	1.024368	1.27E-47
CD46	1.024528	1.12E-35
ATP11A	1.02485	2.29E-30
PRRG2	1.024946	8.44E-39
MIS18BP1	1.025353	1.39E-38
LANCL2	1.025531	3.13E-49
ARHGAP6	1.025536	1.02E-37
VPS11	1.025661	2.57E-44
CFAP45	1.025712	9.66E-51
SIGMAR1	1.025913	3.78E-45
SAP130	1.026063	8.69E-48
PACRGL	1.026211	9.17E-45
CDON	1.026227	2.42E-36
EFNB3	1.026467	6.52E-42
WWC1	1.026487	3.6E-32
MRPL16	1.026524	9.11E-49
NEK1	1.026562	8.34E-46
PNMA8A	1.026735	2.68E-24
ANKRD36(	1.026737	6.76E-25
MAP4K2	1.026784	7.19E-41
CIB2	1.026933	2.49E-36
TRMT2B	1.027097	2.45E-47
LYRM1	1.027114	6.58E-43
MECR	1.027118	7.83E-44
MSL2	1.027134	1.62E-44
C1QTNF7	1.027373	5.26E-42
HIRIP3	1.027431	2.65E-47
CCDC57	1.027849	3.07E-33
CWF19L2	1.027881	1.28E-46
ZFAND2A	1.027889	2.19E-28
SGO2	1.028094	1.57E-54
MYOM3	1.028311	8.59E-32
FGF13	1.028653	1.14E-39
TOPORS	1.028678	7.67E-43
MBD3	1.028695	3.24E-32
ZNF564	1.028943	3.79E-48
DIS3L2	1.028955	1.29E-45

SSR1	1.029081	1.79E-39
ZBTB4	1.029176	3.23E-40
FBXW2	1.029231	1.7E-39
EDC3	1.029249	2.02E-45
PIM3	1.029296	1.75E-24
DUS4L	1.029366	2.1E-48
CHML	1.029823	4.31E-45
ANO7	1.030116	1.39E-39
NOL7	1.030145	5.47E-48
FBXO46	1.030172	1.37E-41
CHST2	1.030596	2.96E-43
STAM	1.03085	1.02E-44
MRPL24	1.030934	6.87E-49
LILRA6	1.031115	3.49E-27
RPL22	1.031237	3.52E-46
STARD7	1.031524	7.75E-48
CD1D	1.031816	7.93E-38
NPHP1	1.0321	8.43E-45
RPL31	1.032307	1.89E-45
KDM6B	1.032442	2.79E-35
HSPA14	1.032623	3.28E-47
WDR20	1.03265	1.65E-48
OTULIN	1.032771	8.89E-45
GPR157	1.032836	1.49E-38
PRR29	1.032898	5.79E-48
ZBTB6	1.03296	2.69E-48
TAF10	1.032973	5.53E-41
RIOX1	1.033462	7.5E-50
FXYD1	1.033561	6.29E-22
NFYA	1.033562	1.13E-39
LAMA5	1.033642	1.59E-21
GTF2E1	1.033738	2.3E-48
FBXL14	1.033769	1.95E-42
ZC3H11A	1.03384	1.92E-41
ELK4	1.033907	2.76E-39
CSGALNA(	1.034058	3.07E-30
RCOR2	1.034075	3.92E-51
BCORL1	1.034076	2.06E-43
HECW2	1.034514	2.82E-47
KIAA1549L	1.034808	4.63E-46
PLA2G2D	1.0349	6.56E-55
SLX1B	1.034921	4.3E-18
PPIL3	1.035128	4.43E-37
RFC4	1.035215	2.58E-42
KRT81	1.035298	1.36E-40
SPATA17	1.035326	7.05E-51
SNTB2	1.035579	7.59E-44
KATNB1	1.035792	2.42E-46
CLIP1	1.036132	4.08E-40
ANKRD11	1.036291	2.18E-42
ZNF638	1.036309	2.65E-39
NRN1	1.036346	3.08E-29
CYP2W1	1.036547	4.74E-26
FBXO5	1.036661	2.19E-47
SLC35C2	1.036765	3.67E-45
MAGEH1	1.036823	1.02E-30
UPRT	1.036886	1.15E-49
APBB1	1.036938	2.63E-28
FAM122B	1.036965	9.03E-45

LRRN4CL	1.037312	8.8E-36
MANF	1.037618	3.24E-37
RSPH3	1.037949	1.9E-49
ESF1	1.038032	8.31E-45
TOMM20	1.038035	1.44E-47
VDAC2	1.038184	2.94E-41
KIF21B	1.038308	3.66E-47
CENPE	1.038374	5.28E-54
RASGRP3	1.03846	1.28E-31
FXR2	1.039572	2.32E-43
NF1	1.039576	6.58E-43
NEDD4	1.039795	1.43E-43
URM1	1.039823	3.24E-44
ACVR2A	1.04003	4.08E-45
FES	1.040057	3.65E-29
SLC30A6	1.040112	1.12E-48
RIPK4	1.040449	6.35E-32
DDX49	1.04045	3.84E-45
RANBP9	1.040472	9.92E-44
DEPDC5	1.040726	4.08E-45
HGS	1.041043	2.69E-36
SMC6	1.041187	5.97E-41
PBX3	1.041429	1.58E-33
GALNT7	1.041437	6.03E-31
GNG4	1.041729	4.8E-17
TSPYL1	1.041916	1.6E-43
PTPRN	1.041938	6.22E-08
MMP13	1.042082	9.18E-58
MLH1	1.042242	9.73E-46
FAM83G	1.042363	1.87E-35
ARMCX6	1.042541	7.38E-45
YWHAQ	1.042784	1.63E-40
MAP3K7	1.042959	1.22E-42
MIGA1	1.043016	2.21E-44
MPHOSPH	1.043172	4.5E-48
SLC22A4	1.043481	9.84E-51
TIMM22	1.043591	3.8E-50
AMOTL2	1.043625	1.59E-29
SPOUT1	1.043674	6.34E-43
RPL19	1.044065	1.43E-47
RASA4	1.044153	1.03E-27
METTL2B	1.044217	7.94E-50
OXTR	1.044276	4.33E-52
NUDT19	1.04428	4E-48
RALGDS	1.044483	4.23E-35
IVD	1.04476	4.36E-43
LIN52	1.044833	5.01E-50
GPC3	1.044915	7.26E-21
GIMAP8	1.044989	1.66E-32
NRDE2	1.045276	1.37E-49
DIDO1	1.045384	6.96E-42
SLC49A3	1.045411	2.3E-29
SLC27A1	1.04553	6.27E-38
SLC19A1	1.045606	1.23E-33
HOXD9	1.045638	4.36E-52
PGM5	1.045664	4.69E-25
HOXB8	1.045921	1.49E-49
CD180	1.046022	2.13E-49
ZNF277	1.046054	2.83E-43

ZC3H18	1.046067	4.14E-44
TMEM251	1.046259	4.17E-47
TMED4	1.046261	1.74E-48
GRID1	1.046262	1.32E-44
SMO	1.046848	1.24E-29
AIM2	1.046928	2.69E-40
JMJD8	1.04701	2.15E-46
TTC23	1.047081	9.77E-42
ZNF48	1.047133	2.03E-50
TOP1MT	1.047149	3.03E-40
RPUSD1	1.047517	1.22E-42
CCT8	1.047722	4.87E-44
GPBP1L1	1.047806	1.8E-42
NPLOC4	1.048017	2.8E-41
FTSJ3	1.048336	1.57E-42
UQCRFS1	1.0484	1.13E-49
TCOF1	1.048589	4.92E-46
CNST	1.048666	2.79E-47
NUP107	1.04873	1.9E-43
HACD1	1.048973	2.96E-25
ZNF45	1.04907	3.13E-46
NANOS3	1.049175	1.38E-47
NCKAP5L	1.049242	7.09E-36
WDR76	1.049496	3.83E-49
CCDC78	1.049499	7.18E-37
FRG1	1.049573	5.17E-47
SOSTDC1	1.049647	3.4E-36
PITHD1	1.049749	1.93E-46
ATP10D	1.049972	1.19E-38
PIAS3	1.050061	1.41E-35
WDR61	1.050271	7.75E-48
PIGW	1.050391	6.96E-47
PTEN	1.0505	4.08E-40
CYC1	1.050518	2.18E-47
KCND2	1.050585	2.83E-50
HPS1	1.0507	2.48E-42
PHACTR4	1.050726	2.47E-47
LGALS9C	1.050853	1.2E-43
HS3ST3B1	1.050993	4.78E-48
MRPS5	1.051033	6.56E-44
LRPPRC	1.051121	1.63E-46
SPRED3	1.051304	1.65E-43
SLC44A3	1.05137	1.9E-33
ARL14EP	1.051559	2.57E-46
PEX3	1.051617	1.43E-46
ACTR8	1.051844	6.02E-47
RPP21	1.051978	3.85E-43
UFC1	1.052088	7.17E-48
TMEM182	1.052681	1.37E-52
VCPIP1	1.052714	4.28E-48
WDR81	1.052911	3.04E-37
C8orf33	1.05319	2.26E-46
PPP2R5D	1.053531	5.77E-46
LINS1	1.053548	3.81E-44
SOAT1	1.053579	4.53E-36
GJC1	1.053682	3.13E-46
SLC45A1	1.053696	4.24E-45
KIAA0586	1.053721	5.93E-47
ATL2	1.053756	3.59E-38

SYTL4	1.053839	1.76E-34
UBXN2B	1.053912	1.35E-44
USP34	1.053919	3E-42
SIGIRR	1.054092	9.4E-33
SAYSD1	1.054264	1.31E-48
TTC26	1.054319	5.4E-51
GEMIN5	1.054355	7.13E-49
CLNS1A	1.054393	6.37E-46
CNFN	1.054565	6.95E-33
PDE12	1.054678	8.75E-45
GSTZ1	1.055357	1.08E-40
PARD3B	1.055791	1.33E-42
NUP133	1.055801	5.02E-48
KIF3B	1.055944	1.17E-45
LRRC27	1.056332	6.07E-43
MAPK14	1.056349	2.12E-43
MRPS27	1.056461	1.05E-46
ZNF250	1.056605	1.93E-44
RNF126	1.056673	5.19E-39
METTL5	1.056767	1.78E-48
EPS15L1	1.056852	1.92E-44
WDR3	1.056943	3.99E-35
RGS9	1.056962	1.63E-34
NMNAT2	1.057006	2.89E-36
CD33	1.057033	3.55E-30
HMGXB3	1.05709	2.34E-42
CNOT11	1.057226	8.83E-45
TOX4	1.057321	3.16E-43
PLCG1	1.05735	7.37E-35
SENP1	1.057596	1.47E-48
PCNX1	1.057623	2.1E-42
EIF3M	1.057904	1.01E-41
RAI2	1.057967	3.05E-29
EXOSC1	1.058129	1.23E-47
AK6	1.058359	9.88E-46
OXLD1	1.058792	1.12E-32
CTLA4	1.058831	9.7E-48
ZNF200	1.058863	3.37E-48
CDK12	1.058905	1.57E-42
CRYAB	1.058956	1.86E-20
DHX30	1.059092	7.49E-45
CANX	1.059401	1.16E-38
ATP6V1FN	1.059692	6.55E-49
ALCAM	1.059943	1.17E-32
WDR89	1.060291	5.13E-50
CASP6	1.060418	3.35E-40
IFT140	1.060537	1.93E-43
CDKN1C	1.060574	1.59E-27
TCF7	1.06067	3.65E-32
R3HDM1	1.061193	6.95E-48
VRK2	1.061237	2.58E-42
MYO5A	1.061292	1.12E-35
RMND1	1.06135	2.34E-48
TRAPPC6B	1.061521	2.48E-46
PCP4	1.06155	4.84E-15
PRAM1	1.06157	2.97E-31
YOD1	1.06157	2.11E-48
DCLRE1C	1.061701	1.07E-45
CCDC22	1.061779	1.91E-47

FRY	1.061992	4.19E-32
FYCO1	1.062071	1.87E-43
MPRIP	1.062082	2.11E-39
TMEM38B	1.06227	5.76E-41
UXT	1.062385	1.88E-50
RBM4	1.062437	2.32E-46
TMED5	1.062463	1.31E-37
YIPF5	1.06256	1.18E-39
MZT2A	1.06259	2.48E-31
WIPI2	1.06279	3.48E-48
DCAF15	1.063017	1.01E-39
GADD45B	1.063588	2.26E-13
BTBD9	1.063625	1.71E-50
STARD3	1.063686	9.05E-41
CEP131	1.063883	2.47E-33
ZNF423	1.064141	4.59E-39
CA14	1.064212	4.45E-51
DESI1	1.0644	3.06E-47
FEM1C	1.064416	6.13E-44
LRCH3	1.064462	1.38E-40
C1orf50	1.064517	8.12E-48
PLRG1	1.064555	2.06E-46
ZDHHC4	1.06472	2.38E-49
HNRNPD	1.064792	1.46E-44
GPATCH2	1.064961	2.2E-48
CIPC	1.065097	4.11E-45
STAT3	1.065169	1.2E-29
RGS14	1.065333	1.54E-33
AGPAT2	1.065374	5.4E-26
MVK	1.065376	6.02E-43
MCUR1	1.06553	1.54E-48
TVP23B	1.065715	5.89E-42
IRF2BP1	1.06576	1.11E-42
KAT7	1.065875	5.07E-46
SRSF7	1.065939	7.5E-38
CALHM3	1.065995	2.04E-20
ZNF639	1.06628	5.36E-44
ACBD6	1.066358	2.36E-47
ZNF174	1.0664	1.18E-49
ABCB10	1.066454	2.07E-45
SLC25A46	1.066459	3.23E-43
RAB33A	1.066564	5.59E-51
UTP11	1.066579	9.65E-46
TOMM7	1.066634	5.07E-46
LRRCC1	1.066645	2.52E-45
UBFD1	1.066945	2.12E-45
PDHX	1.066978	4.39E-49
TRNAU1AF	1.067004	5E-41
CBY1	1.067056	2.82E-47
LENG1	1.06722	2.71E-46
BPI	1.06754	6.37E-47
ITK	1.067711	4.97E-37
KIF3A	1.067754	8.35E-44
FCGR1B	1.067852	2.88E-35
MTFR2	1.067858	3.45E-54
RNFT2	1.067959	2.08E-51
TADA1	1.06815	3.57E-47
UTP25	1.068259	1.52E-49
ARV1	1.068631	1.72E-49

DPP7	1.068751	3.57E-35
NAA30	1.069179	6.09E-48
SEMA3E	1.069315	4.09E-40
PIP5K1C	1.069374	9.15E-44
ARSJ	1.069446	2.03E-41
NPM1	1.069596	4.74E-35
AC008764.	1.070164	4.01E-15
LRRC75A	1.070347	2.5E-46
EDF1	1.070374	6.54E-51
ZBTB22	1.07041	3.92E-47
POLG	1.070748	1.63E-40
BMX	1.070769	2.6E-40
SMAD5	1.070931	3.96E-43
ALKBH2	1.070966	5.21E-44
SPIN4	1.071019	3.84E-45
RBCK1	1.071055	9.34E-38
BTBD16	1.071055	4.7E-48
EDEM2	1.071155	2.96E-48
WBP4	1.071166	2.02E-47
KLC1	1.071256	1.03E-38
ERV3-1	1.0713	1.45E-30
TTC39B	1.071302	1.49E-44
ARHGAP11	1.071456	1.79E-41
COX15	1.07167	2.72E-48
DHX35	1.071809	1.96E-47
TAF9B	1.071873	1.55E-43
TIAM2	1.071963	1.17E-43
NDUFAF8	1.071968	1.79E-43
FPGS	1.072079	1.08E-40
FAM200A	1.072141	3.1E-49
LRRFIP2	1.072401	1.69E-42
CDX1	1.072431	4.91E-52
BEND6	1.072554	1.18E-49
MYRF	1.07259	1.9E-24
SMARCC1	1.072642	1.61E-41
CAMSAP1	1.072651	1.04E-43
CIAO2A	1.072778	1.47E-45
TMEM216	1.07305	8.31E-48
CARMIL2	1.073062	6.56E-36
REST	1.073218	2.78E-40
SELENOM	1.073229	3.3E-27
TLR5	1.073255	1.33E-35
CCDC24	1.073369	3.64E-34
PER2	1.073586	1.49E-40
ODF2L	1.073737	1.76E-35
NACA	1.073828	1.37E-47
GPN3	1.074135	7.82E-47
GSPT1	1.074325	1.33E-40
MRPL40	1.074858	4.75E-50
SUPV3L1	1.075277	1.07E-45
CXCL17	1.07544	1.05E-09
LMBRD1	1.075674	1.63E-44
PWP1	1.076057	2.12E-47
SMIM27	1.076178	2.57E-46
DDX23	1.07622	2.51E-43
SRRD	1.076362	4E-46
GOSR1	1.076588	2.38E-47
MRPL53	1.076749	2.72E-38
CLEC2D	1.076978	2.11E-30

PPP1R1B	1.07702	2.17E-15
NBPF14	1.077732	1.44E-29
LSM5	1.077817	3.11E-47
ZNF468	1.077834	5.93E-42
TTC22	1.077926	7.98E-37
TEX10	1.077942	2.1E-47
TMUB2	1.078029	2.89E-45
TPM1	1.078061	1.51E-17
REEP5	1.078378	6.59E-48
ZNF398	1.078581	2.46E-52
CD19	1.07864	2.34E-44
AJUBA	1.07869	3.19E-32
PHLDB1	1.07877	3.49E-33
RAB3GAP2	1.078774	1.34E-45
C2CD3	1.079119	1.71E-43
ACE2	1.079214	1.09E-16
DZIP3	1.079259	1.03E-40
DNAH2	1.079664	9.26E-51
ZFR	1.07968	2.44E-44
EPC2	1.079822	9.48E-43
NARF	1.080159	2.53E-35
RASD2	1.08028	1.23E-42
CDK5R1	1.080322	9E-44
IRX5	1.080457	1.39E-49
GNAO1	1.080654	1.04E-24
CHORDC1	1.080724	5.65E-37
MED26	1.080741	3.69E-49
ADAMTS9	1.080957	1.67E-26
SDHB	1.080957	2.88E-49
WDR43	1.081016	4.95E-42
EIF4G1	1.08113	2.34E-45
ANKRD35	1.081303	5.19E-48
ECEL1	1.081361	3.8E-40
CDV3	1.081411	7.87E-40
COQ6	1.081514	6.95E-45
CNOT6L	1.081776	2E-42
DHDDS	1.081798	3.24E-44
PPP3CB	1.081923	1.1E-46
ZBTB45	1.0821	1.53E-44
GHDC	1.082151	5.72E-45
TTL	1.082287	1.41E-42
ALKAL1	1.082383	2.73E-36
SOS2	1.082491	4.29E-44
NDUFS7	1.082656	6.13E-35
IMMT	1.082663	2.29E-49
TOX	1.082792	5.6E-35
RNF170	1.082792	1.12E-47
TESK1	1.082878	5.6E-39
NUDCD1	1.082915	1.08E-43
CAMKK2	1.082991	5.44E-42
TMCO3	1.08314	2.98E-41
NIPA1	1.083513	2.61E-45
PPP1R3F	1.083545	1.77E-42
GAS2L1	1.083857	2.82E-36
MATK	1.084215	1.68E-40
EPB41L4A	1.084256	2.28E-36
TNFSF8	1.084292	1.54E-41
TSPAN12	1.084324	1.71E-33
MRPL54	1.084394	1.3E-44

ZNRF2	1.084518	8.55E-49
SP2	1.084652	8.57E-47
HSPA12B	1.084706	1.31E-37
TMTC2	1.084712	4.57E-39
VIPAS39	1.084973	4.63E-46
SLC19A3	1.085047	1.89E-22
ATXN7L1	1.085146	1.97E-47
MOGAT2	1.085253	2.5E-47
CCDC58	1.085267	1.1E-46
SMC3	1.085279	3.48E-45
CEP126	1.085454	3.83E-45
ABHD14B	1.086044	4.66E-46
TRIR	1.0863	7.66E-45
FGD4	1.086473	8.09E-39
KLF12	1.086685	3.51E-47
ZNF562	1.087216	1.8E-46
CCL2	1.08738	7.64E-11
SF3A1	1.087524	1.73E-37
PAQR3	1.087798	1.31E-43
ANKS1A	1.087907	2.89E-44
CTBS	1.088025	1.65E-44
SETX	1.08825	8.07E-45
SMIM3	1.08853	4.14E-29
CRMP1	1.088554	3.57E-26
SPDYE3	1.088674	1.08E-52
ARHGAP22	1.088695	2.54E-37
SUV39H1	1.088755	6.81E-52
PSMD12	1.088871	1.58E-44
BCL11B	1.089123	7.4E-52
PRRC2A	1.089156	3.84E-37
KATNAL1	1.089407	5.16E-45
RNASEH1	1.089594	7.57E-46
ARHGAP17	1.089627	2.16E-41
PRKAG2	1.089675	3.66E-43
AHCYL1	1.089807	2.38E-42
UBN1	1.089853	2.25E-44
MANEAL	1.090421	1.22E-39
HGH1	1.090573	3.33E-47
ZBTB9	1.090704	2.1E-51
PDCD5	1.090787	3.52E-47
NLRP3	1.090878	3.2E-43
CREB3L1	1.0909	1.83E-20
NXPH3	1.090961	1.53E-46
TMEM40	1.091152	6.21E-37
WDR62	1.091158	3.36E-54
HINT3	1.091214	1.39E-48
MRPS17	1.091216	2.25E-51
ODF2	1.091427	3.24E-44
LPL	1.091615	8.46E-26
FBXW9	1.091813	3.85E-46
DOHH	1.092083	1.66E-42
DHX36	1.092113	6.7E-45
PLSCR4	1.092697	2.22E-31
GORASP2	1.092827	2.07E-45
FUT4	1.092917	5.13E-37
TBC1D14	1.092956	1.44E-43
FBXL12	1.092999	1.14E-43
SMIM26	1.093067	4.04E-48
MGRN1	1.093119	1.26E-43

EEF1G	1.093457	2.36E-49
DCP1B	1.09359	6.02E-45
CTR9	1.093599	1.03E-46
TMEM183A	1.093862	1.47E-47
ARMC8	1.093887	1.51E-44
LY6K	1.093917	3.85E-27
HOMER1	1.094011	1.01E-50
MAPK11	1.09417	1.79E-33
SRSF1	1.094253	1.83E-40
KRTCAP3	1.094742	7.54E-28
LDOC1	1.094826	2.54E-26
PLEKHM2	1.095351	4.63E-40
TAF9	1.09537	9.23E-46
SPCS2	1.09565	6.66E-49
HIVEP1	1.095849	7.05E-44
HAS1	1.095991	9.73E-35
GNL1	1.096126	2.71E-43
ZNF777	1.096413	4.35E-49
NABP2	1.096416	3.46E-50
CIB1	1.096462	1.02E-33
RAB3GAP1	1.096462	9.26E-48
WAC	1.096462	1.04E-41
UNC45A	1.09647	4.57E-45
LRRC31	1.096531	1.76E-49
SSNA1	1.096612	2.84E-45
ZNF623	1.096712	3.23E-45
LYST	1.096783	2.03E-46
TLE4	1.096865	3.19E-38
ZMYND19	1.096884	1.31E-46
SRRM3	1.097005	5.71E-33
RPS19	1.097026	3.51E-39
PUM2	1.097081	1.15E-45
MED8	1.097325	1.87E-47
ZNF143	1.097365	1.81E-44
POPDC2	1.097427	1.52E-39
IL17RE	1.097485	1.79E-25
GAD1	1.097693	6.35E-42
DLC1	1.097711	9.82E-30
GPR108	1.098017	2.2E-43
SCUBE3	1.09807	8.11E-54
BTBD19	1.0983	1.14E-33
DYRK3	1.098366	3.28E-46
AK7	1.098474	5.34E-44
CBX8	1.098485	4.98E-46
PTGES2	1.098495	2.12E-45
ZNF827	1.098516	2.68E-43
CUL4A	1.098637	7.3E-47
USP8	1.09869	3.6E-47
GLI3	1.098778	7.44E-41
SS18	1.098806	2.96E-43
RUBCNL	1.098946	2.58E-39
RNASE2	1.098978	2.95E-29
ZBTB12	1.099397	6.33E-49
PAGR1	1.099405	3.23E-45
FAM241B	1.099415	7.6E-44
PCNX4	1.09942	3.27E-42
TEX30	1.099453	5.05E-48
RMDN2	1.099858	2.49E-44
ANP32B	1.100108	2.56E-45

GPS1	1.100133	2.07E-47
MRPL50	1.100544	4.03E-49
H6PD	1.100583	2.68E-33
PGAM5	1.100595	2.44E-48
PEG10	1.100819	4.07E-18
NPR2	1.100954	3.12E-35
POLE2	1.101164	2.21E-48
PPM1B	1.101197	4.56E-44
RPS29	1.10143	2.9E-44
WDR18	1.101523	4.8E-38
GPANK1	1.101626	1.29E-50
C12orf57	1.101748	1.27E-44
URGCP	1.101771	6.59E-48
MRPL48	1.102142	3.18E-49
CD1A	1.102221	8.83E-52
SLC35B1	1.102308	1.03E-47
LDAH	1.102368	9.69E-48
JHY	1.102606	3.9E-47
JMJD4	1.10261	1.21E-48
SPEG	1.102782	1.17E-23
PLPPR2	1.103023	6.8E-41
DNAJC22	1.103412	1.95E-32
IPO13	1.103457	3.62E-45
ALG11	1.103474	3.77E-49
RASGRP1	1.103589	3.4E-36
GNL3L	1.103919	1.53E-42
DPM3	1.103967	1.03E-40
GTF2H5	1.104025	2.69E-53
PRDM8	1.104082	8.83E-43
ZNF416	1.104147	1.65E-52
ZRSR2	1.104219	2.26E-42
SDE2	1.104237	7.88E-44
TBX3	1.104276	4.43E-35
DIAPH3	1.104282	1.15E-53
ARHGAP35	1.10449	1.36E-43
BAG2	1.104786	6.23E-44
RNMT	1.104963	1.2E-47
RPIA	1.105067	1.99E-49
STK16	1.105213	5.79E-50
MOSPD1	1.105352	2.76E-48
NRROS	1.105391	1.63E-42
EVI5	1.105394	2.69E-45
IQCD	1.105453	4.06E-54
ARFGEF1	1.105456	3E-45
TIGD2	1.105556	1.94E-51
SNX14	1.105616	1.18E-44
BMT2	1.105616	1.29E-49
TMED10	1.105741	5.64E-46
SLC25A40	1.105858	3.27E-42
TMEM223	1.105914	9.77E-49
CNOT7	1.105958	8.63E-43
NEMP2	1.106007	1.92E-49
HEY1	1.10619	2.16E-45
KTI12	1.106258	1.76E-49
LIMK2	1.106349	2.05E-34
SHMT1	1.106419	5.93E-40
NUP88	1.106431	1.06E-42
CHPF	1.106454	7.29E-32
CEP250	1.106777	1.57E-42

ZNF836	1.106848	6.22E-44
IKZF3	1.106902	1.27E-46
RIOK2	1.106984	1.08E-47
MLKL	1.107118	5.73E-37
NACAD	1.107416	2.61E-43
PENK	1.107647	1.77E-33
PRR5	1.107817	1.7E-40
CCDC34	1.10791	1.47E-47
CRLS1	1.10809	3.21E-47
NKAP	1.108685	7.5E-53
C2orf15	1.108705	2.11E-48
HOXB4	1.108714	2.48E-44
WASF2	1.108765	3.39E-37
JMJD6	1.108892	8.99E-40
PPP1R35	1.109103	4.31E-33
PPP1R12B	1.109188	3.27E-33
NOCT	1.109206	6.58E-45
MOAP1	1.109243	1.32E-45
RAI1	1.10945	1.61E-38
SAMD10	1.109507	4.06E-39
HEPACAM	1.109789	1.07E-13
CHCHD4	1.110026	3.81E-53
HIVEP3	1.110218	5.87E-47
USP12	1.110481	1.25E-42
RER1	1.11053	3.62E-45
SMG5	1.110663	2.35E-41
PRPH	1.11076	6.44E-15
ACAN	1.110879	6.43E-45
CGB7	1.110926	1.45E-37
HSPB2-C1	1.110973	8.21E-33
MPV17L2	1.111031	2.18E-47
DISP1	1.111112	2.44E-50
CGRRF1	1.111201	3.57E-44
COG2	1.111228	4.04E-48
MPZL3	1.111255	9.84E-39
E2F8	1.111291	3.14E-53
CSRNP1	1.111478	2.26E-23
AL132780.:	1.111531	1.73E-31
NLGN2	1.111596	5.19E-32
ISY1-RAB4	1.111626	4.96E-41
ZNF821	1.111664	1.07E-43
NGDN	1.112025	9.55E-48
PRSS36	1.112051	7.5E-46
ATMIN	1.112328	1.18E-48
ZNF816	1.112419	2.64E-43
TMED8	1.112468	1.84E-47
EMC2	1.112595	1.33E-45
DDX18	1.112669	1.24E-42
VHL	1.112706	9.22E-42
LRRN2	1.112899	1.13E-34
COG7	1.112923	6.02E-42
VKORC1	1.113017	6.16E-49
MED20	1.113041	7.2E-49
PRDX2	1.113227	1.98E-45
LARS2	1.113258	4.9E-49
PKD2	1.113742	1.47E-30
TTC4	1.113812	3.9E-49
TEK	1.113958	6.4E-36
NDUFAF5	1.113996	9.58E-46

DNAJC5B	1.114054	1.68E-48
SLC9A4	1.114106	1.66E-40
C12orf43	1.11434	4.6E-50
GLYR1	1.11439	1.99E-45
RMDN1	1.114502	1.2E-44
POMGNT1	1.114514	2.92E-42
MRPL43	1.1146	4.33E-49
GFM1	1.114636	1.49E-48
RIPOR2	1.114718	1.47E-36
SLC38A1	1.114823	1.34E-34
TINF2	1.114833	8.05E-46
PDS5A	1.114853	2.9E-47
JARID2	1.114898	3.01E-47
SNX1	1.11509	1.78E-45
MRPS14	1.115092	2.48E-51
TP53I13	1.115141	2.54E-35
MED13	1.115364	5.1E-46
ZMAT3	1.11586	5.71E-45
AGO2	1.115946	3.57E-43
MCAT	1.116045	1.21E-48
PI4KA	1.116076	4.46E-43
ZNF358	1.116276	9.84E-43
COPS2	1.116285	5.37E-41
RNF123	1.116611	1.57E-46
FIG4	1.116618	7.11E-48
CHD9	1.11671	1.38E-45
ZMYM4	1.116724	1.5E-45
ST13	1.116814	3.37E-42
LRP12	1.116878	2.19E-39
POLR1C	1.116959	2.8E-46
ZNHIT3	1.117094	1.47E-48
EXTL3	1.117147	3.97E-43
NDEL1	1.117203	1.51E-43
MICA	1.117296	9.4E-33
NDUFAF4	1.117346	4.78E-45
SH3BP2	1.117667	3.99E-30
NMT1	1.117865	3.46E-44
MSH5-SAF	1.11792	1.2E-34
ASCC3	1.118387	3.91E-46
ZYG11B	1.118422	6.85E-49
PRR16	1.118495	1.26E-37
SCAND1	1.118522	1.53E-29
TCFL5	1.118688	5.55E-50
MTIF3	1.118854	2.58E-50
UBE3A	1.11888	2.52E-45
SUCNR1	1.118899	4.79E-51
AKTIP	1.118904	5.23E-45
SERPINB6	1.119055	3.18E-42
RASSF6	1.119091	1.2E-29
MIA2	1.119152	8.51E-45
POLR3C	1.119229	2.57E-47
HPS5	1.119322	1.62E-42
NUDT16	1.119374	3.7E-44
ZNF688	1.119496	4.64E-48
HSDL1	1.119628	6.58E-48
TAF1A	1.119853	2.35E-46
GANAB	1.119901	8.2E-45
OSTC	1.119934	4.08E-45
VTI1A	1.119951	2.45E-49

GINS3	1.119964	2.8E-50
SNAP25	1.119967	8.82E-16
SLC35B4	1.120017	3.56E-50
MRPL58	1.120418	2.85E-49
ISCU	1.120666	1.02E-43
ZFAT	1.121112	6.15E-49
LARP4B	1.121538	1.08E-46
NR1D1	1.121659	3.91E-22
UPF1	1.121801	1.4E-45
MYB	1.121886	3.28E-53
PIP4K2B	1.122041	7.55E-45
RNF223	1.122351	2.21E-47
TRIM13	1.122396	1.22E-46
AQR	1.122523	1.04E-47
PLA2G5	1.12263	9.61E-38
VRK3	1.122638	5.8E-47
PGAP3	1.122816	1.25E-43
CYP2U1	1.12286	5.12E-45
DISP2	1.123133	1.69E-42
MTHFS	1.123226	3.66E-43
OS9	1.123246	6.37E-50
RAD18	1.123332	9.31E-52
CCDC96	1.12342	1.33E-55
RPL38	1.123674	4.67E-45
CUL4B	1.123729	8.44E-48
TPT1	1.123806	3E-50
TRMT6	1.123819	1.89E-49
TMC4	1.12383	1.14E-29
BTN3A1	1.123871	1.94E-33
PHTF1	1.123897	1.2E-45
EXOC6	1.123936	3.43E-39
AKR1C1	1.123987	9.88E-15
AGPAT4	1.124058	2.86E-42
KPNA6	1.124073	1.93E-46
ABCC4	1.124079	6E-43
TRIL	1.124124	1.21E-48
SMYD2	1.124314	1.1E-47
TNPO1	1.124617	2.35E-41
LDB1	1.124845	7.96E-41
CTF1	1.124856	1.01E-40
LRRC6	1.124888	2.15E-36
INO80	1.124998	2.33E-44
GATD1	1.125122	2.72E-41
HSPA13	1.125206	9.63E-39
RASL11A	1.125244	1.98E-32
RBM43	1.125249	8.62E-47
RRP1	1.125545	3.74E-47
RAB15	1.125576	2.36E-32
PCDHB9	1.125589	5.82E-49
SAMD4A	1.126036	1.73E-35
BCL9L	1.126123	9.86E-28
DIP2C	1.12625	4.23E-43
PRKCB	1.126371	1.18E-40
OTUD5	1.126421	3.56E-45
COL5A3	1.126525	2.09E-28
COQ5	1.126647	1.08E-49
SAMM50	1.12679	5.8E-47
SLC8A1	1.126877	1.68E-43
TUT7	1.126944	2.43E-45

CAMK4	1.12701	5.82E-51
TEFM	1.127105	4.82E-49
TOR1AIP2	1.127157	2.67E-48
NKRF	1.127364	5.13E-50
FAAP24	1.127485	4.72E-50
RNF10	1.127621	3.23E-45
BORCS7	1.127758	7.38E-45
DDX19A	1.127864	2.72E-48
PLAA	1.12797	2.73E-44
HGF	1.127998	2.52E-37
SLC18B1	1.12801	5.05E-44
NOL12	1.128103	9.44E-46
TGIF2-RAE	1.128244	9.17E-32
MYCT1	1.128263	6.8E-40
RTN4R	1.128336	3.85E-31
ATG9A	1.128426	1.38E-46
LIN54	1.128454	6.42E-52
FBXO42	1.128672	2.48E-46
MICU2	1.128732	2.3E-47
FAM171B	1.128808	3.26E-48
TMEM138	1.128816	9.45E-45
BCL2L12	1.12893	8.21E-39
BTG1	1.128977	1.28E-33
AK5	1.129162	3.28E-49
MAGED4B	1.12923	1.72E-23
YAE1	1.129286	4.68E-47
SPRTN	1.129446	9.11E-52
IFT27	1.12945	6.3E-49
CABYR	1.129465	1.22E-46
RNPEPL1	1.129506	2.8E-36
RPL8	1.12962	1.83E-46
PRKCZ	1.129651	4.53E-43
NPAT	1.129666	2.01E-46
LTA4H	1.129712	7.89E-44
OSCP1	1.129716	7.77E-45
TMEM14A	1.129889	2.07E-42
TCTA	1.129996	2.81E-43
ASXL2	1.130015	2.95E-44
AGMAT	1.130028	1.2E-51
TMEM42	1.130099	8.62E-44
MMRN1	1.130291	1.29E-22
KLC4	1.130424	4.32E-40
LRRC61	1.130439	1.92E-41
BOC	1.130456	1.09E-23
RNASE4	1.130738	8.71E-33
RDH13	1.130926	1.63E-35
KLHL12	1.130953	1.32E-50
GMPR2	1.131465	6.71E-47
NETO2	1.131504	1.71E-33
CCDC61	1.131583	1.55E-44
GRSF1	1.131647	2.16E-46
ESPL1	1.13167	1.48E-50
DKK2	1.131741	5.21E-52
LAG3	1.131958	1.18E-48
PI4KB	1.132025	9.44E-46
TBC1D25	1.132226	3.88E-47
NCL	1.132245	1.21E-42
DDX10	1.132457	1.54E-49
TMEM170A	1.132473	1.26E-47

TESPA1	1.132505	5.21E-45
R3HCC1L	1.132619	7.24E-47
HRCT1	1.132742	2.39E-34
KCNMB4	1.132765	6.36E-49
METTL2A	1.132843	2.38E-51
CPTP	1.132864	4.09E-38
ME1	1.133527	1.98E-38
HMGXB4	1.133628	2.16E-46
BTF3L4	1.133737	4.35E-46
TTC33	1.13374	1.9E-44
CRYL1	1.133752	9.25E-40
FGG	1.133776	1.32E-08
GBE1	1.133891	4.38E-40
SURF4	1.134081	3.62E-44
PDZD8	1.134257	2.2E-32
C19orf25	1.134592	9.71E-41
MYO18A	1.134648	3.94E-46
USP1	1.134742	8.7E-47
HS3ST3A1	1.13484	4.98E-49
MOB3B	1.134874	1.1E-40
ASCC2	1.134888	4.01E-47
DLD	1.13495	1.01E-46
PARP10	1.135014	1.66E-34
ASGR1	1.135056	1.43E-47
COPS5	1.135322	7.04E-49
MRPL33	1.135328	9.4E-47
ADAMTSL1	1.135555	3.95E-45
UGT1A1	1.135614	2.84E-49
SSRP1	1.135619	3.28E-47
WWOX	1.13565	1.21E-48
CCN6	1.136049	5.36E-20
TTF1	1.136051	5.05E-47
SMIM10L1	1.136074	4.85E-51
SLCO2B1	1.136116	2.47E-21
CTIF	1.136148	4.87E-40
NDUFAF2	1.136149	1.33E-50
ABCB4	1.136161	5.31E-53
DDX50	1.136486	3.71E-48
PSMD9	1.13652	3.85E-49
RDH14	1.136754	3.65E-48
HAUS2	1.137053	1.05E-47
ABL2	1.13711	1.42E-45
NUDT16L1	1.137231	1.43E-48
CACNB1	1.137235	2.14E-35
SLC25A19	1.13724	1.31E-45
ATF6B	1.137421	2.17E-45
PNPO	1.137558	4.9E-48
BMF	1.137601	5.46E-33
DNM3	1.137774	2.66E-50
C4orf33	1.137808	2.49E-44
PIAS1	1.137875	5.85E-46
GDF11	1.137911	3.81E-46
WRNIP1	1.137964	9.17E-50
DOLK	1.138196	4.56E-50
SMIM10	1.138288	1.14E-42
MBOAT1	1.138316	9.66E-32
ING2	1.138361	1.43E-47
VWA7	1.138396	7.78E-36
CCDC74B	1.138432	1.55E-42

LTC4S	1.138518	2.81E-31
FKBPL	1.138542	1.36E-52
FOXO1	1.13858	3.39E-37
SLC24A1	1.138883	4.94E-49
CNOT4	1.1389	2.77E-50
CRTC2	1.138942	3.55E-42
HAT1	1.138981	6.04E-46
ZFAND5	1.139058	2.56E-33
ZNF322	1.139205	2.23E-48
SAMD1	1.139213	1.1E-39
MAFG	1.139315	6.11E-43
API5	1.13937	3.82E-49
SPRY2	1.139464	2.44E-39
LSM14B	1.139599	2.49E-47
TMEM98	1.139649	3.21E-34
PPIL4	1.139676	7.13E-46
RRN3	1.139831	1.87E-46
ZNF652	1.139844	2.39E-46
AC106886.	1.140096	4.46E-48
PCYOX1L	1.140208	9.03E-45
SIGLEC14	1.140212	2.32E-29
KLHL18	1.140371	3.46E-47
ORAI3	1.140408	4.05E-44
GABARAP	1.140486	5.71E-47
SH2B2	1.140543	3.73E-46
DBF4	1.140617	1.14E-47
WIPF2	1.14072	8.24E-48
B3GALT6	1.140788	1.98E-45
FAM110B	1.140795	1.15E-42
BBS12	1.140983	7.3E-52
MARK4	1.141092	6.61E-46
TRIM8	1.141172	8.62E-40
LCORL	1.141312	8.26E-49
ATP11C	1.141511	2.32E-41
BNC2	1.141571	2.54E-43
FP565260.:	1.14161	8.18E-35
MED22	1.14224	2.3E-45
FBH1	1.142356	2.71E-43
GPC4	1.142359	6.72E-36
SAP30BP	1.14236	4.73E-46
SPNS3	1.142418	1.94E-48
SLC41A3	1.142451	2.84E-47
RBBP4	1.142485	2.24E-42
PPM1A	1.142734	6.1E-48
DCSTAMP	1.142759	1.26E-47
RPS24	1.142853	2.12E-47
MT-CO1	1.142931	1.96E-23
UBL7	1.142949	6.16E-49
DMAC1	1.143107	1.62E-44
RNF111	1.143299	5.75E-47
ERI1	1.143321	2.39E-47
SCAMP4	1.14338	2.51E-44
TMEM263	1.143844	1.43E-42
SCG5	1.143886	5.19E-09
FOXN2	1.143914	1.89E-44
ACSS2	1.144108	2.27E-41
SLC26A2	1.144253	3.79E-47
BMI1	1.144641	4.27E-39
ALOX15B	1.144643	1.42E-29

ARMH3	1.14469	2.27E-49
TYW1	1.145065	8.85E-51
RANGAP1	1.145259	7.92E-40
SMG8	1.145274	7.07E-46
PAK1IP1	1.145355	7.57E-48
RHBDD1	1.145481	4.07E-48
SAR1B	1.145689	8.51E-47
FGD2	1.145755	1.01E-27
MBTPS1	1.145756	1.57E-48
SMIM31	1.145865	1.65E-14
ZNF790	1.145881	6.63E-49
AC073896.	1.145895	9.35E-20
ZER1	1.146011	5.55E-48
GAB2	1.146023	2.4E-38
SGO1	1.146415	2.48E-54
CD99L2	1.146545	1.49E-34
CCL11	1.146578	8.31E-26
HDAC5	1.146839	1.5E-41
RAB3A	1.146958	1.18E-47
GPR160	1.147285	1.55E-29
GTF3C4	1.147546	4.3E-51
RHOU	1.147939	1.4E-33
DEAF1	1.148031	8.77E-47
MT2A	1.148051	1.41E-12
TOE1	1.148051	7.6E-49
RBBP5	1.148122	5.24E-53
TONSL	1.148125	9.56E-45
RCBTB2	1.14821	1.05E-40
APRT	1.14835	4.06E-43
RPRD2	1.148383	1.35E-43
BOLA3	1.14865	7.23E-50
CCDC68	1.148812	6.67E-36
SPOP	1.14882	2.67E-48
TRIM32	1.148939	3.1E-48
ARCN1	1.148943	9.92E-50
SLC9A7	1.14932	1.87E-43
PTPN4	1.149373	1.37E-46
ALDH3B2	1.149438	2.52E-42
KDM1B	1.149488	2.2E-43
TSC22D2	1.149532	4.36E-43
KDM3B	1.149622	3.05E-46
CMKLR1	1.149773	3.18E-31
SPATA18	1.149867	3.92E-42
NLN	1.149948	6.29E-43
IL21R	1.149974	4.06E-49
AACS	1.150019	1E-44
SGCA	1.150252	3.42E-32
LRRC57	1.150369	2.08E-46
MED17	1.150494	1.46E-42
ADCY2	1.150657	3.86E-44
RNF20	1.150689	5.56E-49
ZNF263	1.150693	7.76E-47
C1orf112	1.150941	8.73E-53
ISLR2	1.151534	2.03E-47
ISCA1	1.15161	4.9E-47
SLC25A26	1.151648	5.98E-50
STX3	1.151772	4.16E-46
SMAD1	1.151855	2.09E-40
TMEM156	1.152227	3.08E-47

INTS14	1.152244	1.35E-49
NCOA2	1.152278	2.42E-41
VPS33B	1.152297	9.54E-47
PPCS	1.152337	1.7E-48
ITPR1	1.152484	1.12E-36
STIM1	1.152536	1.63E-44
DCTN3	1.152571	9.93E-48
CPSF6	1.152709	1.67E-47
NMUR2	1.15293	2.85E-50
ALDH7A1	1.152951	1.75E-43
CALCOCO:	1.153009	1.38E-40
ISL1	1.153216	1.07E-29
CD300C	1.153324	6.03E-47
TOMM5	1.153359	4.91E-49
NELFCD	1.153361	6.65E-45
IL20RA	1.153742	1.39E-36
NR4A3	1.153882	1.2E-25
SIK1B	1.153976	1.81E-12
GRAMD4	1.154174	6.39E-39
DNM1L	1.154281	1.78E-44
B4GALNT1	1.154455	2.19E-46
CSDE1	1.154484	1.86E-43
HARS2	1.154651	2.23E-45
NTSR1	1.154707	1.01E-18
POLR2I	1.155019	7.53E-47
SIRPB1	1.155075	4.13E-37
INSIG2	1.155279	2.02E-41
EXOC6B	1.155595	3.3E-47
MFSD13A	1.155611	2.3E-43
ZNHIT6	1.155649	1.15E-45
PRPF8	1.155836	4.5E-43
HIPK3	1.155848	1.34E-40
CBLN3	1.155913	7.54E-43
ANKRD42	1.156366	1.89E-50
ZC3H3	1.156457	9.43E-39
BORCS8-M	1.156475	2.15E-45
NDUFB11	1.156511	6.84E-50
LPGAT1	1.156678	4.66E-43
PRKAG1	1.156719	2.42E-49
EEFSEC	1.156903	8.26E-50
RAVER1	1.157361	4.89E-38
EBI3	1.15746	1.59E-37
SIAE	1.157473	7.96E-43
DNAJC2	1.157683	8.69E-49
SPATA33	1.15816	9.84E-48
TTC5	1.158333	1.84E-48
ZBTB33	1.158769	1.86E-49
JMJD1C	1.158839	1.12E-40
GPR171	1.158948	2.19E-31
MTERF3	1.158962	2.41E-47
C16orf70	1.159024	3.51E-48
TMEM240	1.159033	1.14E-43
ZMAT5	1.159094	2.05E-50
PCDHB10	1.159708	3.17E-51
LAGE3	1.15995	2.34E-47
SELENBP1	1.160052	2.05E-29
ADGRF5	1.160228	9.44E-37
PJA2	1.160306	4.46E-41
ZNF512	1.160432	8.69E-43

CAPN6	1.16044	2.58E-22
BBS2	1.160446	1.36E-44
NUMB	1.160464	1.06E-45
TK2	1.160494	1.97E-44
ARHGEF1	1.160497	1.5E-35
SDHAF4	1.160606	4.57E-49
DLST	1.160607	2.77E-47
SENP3	1.160654	3.8E-46
GPR4	1.160681	4.85E-41
MAPK7	1.160711	9.35E-44
METAP1	1.160713	6.21E-45
NAA20	1.160866	3.34E-46
CCDC97	1.161197	3.63E-46
GIMAP6	1.161212	1.04E-27
GRHPR	1.16137	4.75E-49
SEH1L	1.161505	4.22E-50
REEP2	1.1616	1.45E-32
C8orf58	1.161775	1.29E-42
NMRAL1	1.161854	1.24E-49
TP53I3	1.16189	2.16E-35
POLDIP3	1.162029	6.27E-46
SNRPA1	1.162057	6.56E-44
ABCF1	1.162069	6.85E-48
FAM53B	1.162438	1.48E-42
CGB3	1.162513	2.97E-29
SERGEF	1.162555	8.91E-48
PARVA	1.162587	1.59E-42
CBX5	1.162603	3.97E-48
KYAT1	1.16306	3.64E-45
SMC1A	1.163087	1.09E-38
HYLS1	1.163194	7.6E-54
CTDNEP1	1.163391	1.54E-47
SCFD2	1.163406	8.43E-51
SCRN1	1.163476	4.9E-37
CLK3	1.163518	7.76E-42
TMEM237	1.164092	3.31E-46
FTH1	1.164137	6.77E-36
IQCK	1.164258	2.22E-43
SLC25A39	1.164759	2.89E-46
GBGT1	1.164865	1.43E-37
AIFM1	1.16511	6.2E-52
NIN	1.165473	9.63E-41
HMGCS1	1.165671	2.07E-32
ZBTB8OS	1.165699	6.07E-50
STX11	1.165813	2.2E-41
PMS2	1.165967	8.37E-50
CMAS	1.166036	2.08E-48
OSR2	1.16605	5.86E-30
BRI3	1.166068	5.82E-44
RCE1	1.166106	2.5E-45
ERVK3-1	1.166118	5.92E-49
CLEC5A	1.166676	2.86E-31
MED25	1.166934	8.77E-45
ACVRL1	1.166989	1.35E-35
RTF1	1.166999	5.18E-48
FAM81B	1.167315	1.32E-34
C6orf15	1.167497	3.12E-48
KCTD13	1.167565	1.11E-42
PNRC1	1.167601	1.17E-32

KPNA1	1.167795	1.74E-41
TBCD	1.167856	1.48E-42
MDGA1	1.167877	3.57E-40
STIL	1.167893	1.35E-51
TULP3	1.167955	1.82E-44
C9orf116	1.168006	3.73E-42
RASSF7	1.168113	2.09E-25
AK8	1.168285	2.08E-50
RNF181	1.168293	3.02E-46
TDP1	1.168308	9.92E-49
PITPNA	1.168589	1.75E-46
CD72	1.169064	2.35E-30
DRG1	1.169239	4.26E-49
EIF1AX	1.1693	6.86E-42
CCDC184	1.169578	5.23E-49
COLCA2	1.169653	7.16E-31
AP2A1	1.169774	1.69E-42
TMEM30B	1.169896	5.46E-38
FADS1	1.16993	1.17E-33
VDAC3	1.170187	7.06E-50
TFCP2L1	1.170275	4.22E-33
NEK11	1.170355	4.78E-47
MKRN1	1.170355	3.71E-48
HERC3	1.170386	6.83E-37
ADAP2	1.170504	1.39E-38
SCYL1	1.170509	1.81E-47
FRS2	1.170595	8.55E-44
IGFL1	1.170635	7E-40
HPF1	1.170781	6.01E-49
TSHZ1	1.17107	2.22E-43
KRR1	1.171263	2.17E-47
ABTB1	1.171468	8.43E-37
LY9	1.171513	1.09E-45
ZDHHC16	1.171744	5.69E-49
PXMP4	1.172097	3.31E-53
SIGLEC7	1.172211	6.32E-41
THRAP3	1.172665	2.58E-45
GRHL1	1.172705	2.99E-36
TMEM267	1.172827	9.47E-48
ZNF101	1.172854	6.83E-50
TBC1D5	1.17295	1.16E-42
PRKD3	1.173136	8.89E-38
ZNF296	1.173227	3.53E-51
EXOC4	1.173276	1.97E-50
KATNA1	1.173326	2.81E-49
RPTOR	1.173411	1.24E-49
CBL	1.173552	1.23E-45
INKA1	1.173579	6.45E-48
DNAAF5	1.173947	9.26E-48
POLR2C	1.174182	2.75E-50
ADH6	1.174264	2.12E-43
ORC4	1.174317	1.86E-40
LACC1	1.174463	2.94E-49
P2RY8	1.174498	2.32E-42
RPL27	1.174532	9.85E-49
SELENOH	1.174593	3.57E-44
STARD4	1.174709	1.95E-40
RNF138	1.174733	1.23E-44
B3GNT2	1.174839	7.43E-42

GAPT	1.174907	1.93E-38
MAPK12	1.174917	1.66E-34
CDKN2AIP	1.17529	3.07E-46
PFDN5	1.17536	7.91E-49
TMEM165	1.175646	8.42E-41
BEX4	1.175681	1.1E-30
RNF4	1.17576	5.86E-46
LBR	1.175827	5.21E-42
CDCA2	1.175869	1.29E-54
COA7	1.176137	3.36E-51
DNM2	1.176148	1.35E-40
CA11	1.176162	5.68E-39
MRPS34	1.176208	2.4E-48
BFAR	1.176332	8.04E-42
BMS1	1.176926	3.75E-53
ABHD17B	1.177173	1.01E-47
DYNC2LI1	1.177234	2.7E-46
NT5C	1.17725	6.32E-40
TSR3	1.177307	3.55E-47
CSTF2T	1.177372	2.13E-49
FGF11	1.177393	1.61E-43
APAF1	1.177745	1.63E-46
FZD6	1.177777	6.1E-41
SMIM7	1.177874	5.48E-50
AMDHD2	1.178026	1.06E-40
RNF135	1.178153	1.97E-43
GRPEL2	1.178206	5.34E-51
BCL2L13	1.17844	1.49E-48
KIF20B	1.178625	1.71E-53
THAP11	1.178842	1.87E-50
UPK1B	1.179128	9.67E-05
GIMAP5	1.179457	4.28E-26
NABP1	1.179521	6.88E-29
ARMH4	1.1797	9.35E-49
DEXI	1.179898	4.28E-50
PLEKHG5	1.180059	1.64E-40
GLT8D1	1.180092	3.66E-47
LRSAM1	1.180227	2.79E-42
PI16	1.180355	1.42E-13
UROS	1.180535	4.98E-48
SDCCAG8	1.18068	5.56E-49
CDIPT	1.180683	1.63E-45
TXNIP	1.180798	1.22E-25
ADIPOR2	1.181164	1.94E-42
KNL1	1.18119	1.21E-53
WASHC2A	1.181223	4.07E-47
KCTD3	1.181247	1.93E-43
ROM1	1.181264	1.17E-45
SRFBP1	1.181415	5.01E-50
ZNF131	1.181481	1.01E-47
FKBP15	1.181512	1.23E-46
KCNS3	1.181519	2.06E-31
NOTCH2	1.18153	8.64E-32
TNXB	1.181532	1.02E-22
ATG3	1.181902	4.9E-50
PIK3R5	1.181938	2.45E-29
U2AF2	1.181986	1.5E-44
RNLS	1.182001	1.74E-47
DCAKD	1.182005	1.17E-47

ANKMY2	1.182158	2.55E-49
TIMM8A	1.182199	8.27E-54
ZAP70	1.182293	1.29E-31
BORA	1.182642	2.81E-51
AGPS	1.182647	2.09E-46
TUBGCP2	1.182841	1.35E-44
TNFAIP8L3	1.182922	1.58E-41
RAB9A	1.183019	1.69E-46
SLC38A7	1.183093	1.09E-47
WRAP53	1.183099	1.86E-49
RAB11FIP5	1.183617	2.83E-42
AKT1	1.183685	9.84E-47
WDR26	1.183789	2.09E-44
CTDSPL2	1.18422	1.61E-45
FKBP2	1.184431	4.15E-50
C5orf51	1.184456	1.57E-48
HPS3	1.184499	5.19E-45
TCEAL3	1.184644	1.69E-42
TNFSF12-T	1.184723	5.36E-34
DROSHA	1.184854	3.76E-44
KLC2	1.185059	5.48E-43
HNRNPA2I	1.185068	1.38E-45
PF4	1.185114	7.86E-50
TPRA1	1.185195	3.95E-47
USP9X	1.185644	2.4E-46
RTN4IP1	1.185677	1.61E-52
RAB3B	1.185708	2.34E-46
MTM1	1.185804	4.1E-47
INMT	1.185925	1.01E-26
RCAN3	1.186324	4.04E-47
ROCK1	1.186329	8.14E-40
XRN2	1.18637	1.04E-42
RAB27A	1.186376	1.67E-36
ZFPM2	1.186543	4.04E-44
PI15	1.186723	1.25E-45
GLG1	1.186733	8.49E-49
PCYT1A	1.187349	3.16E-45
VPS9D1	1.187594	6.06E-40
RRP8	1.187626	5.42E-48
POLA2	1.187846	5.18E-50
MIB1	1.187912	4.84E-45
VPS33A	1.187922	1.51E-49
ARHGEF5	1.188079	1.73E-38
C14orf132	1.188355	2.3E-38
MED31	1.188366	2.69E-48
EIF1B	1.188724	3.18E-45
SIMC1	1.188759	9.4E-48
ANKLE2	1.188893	6.16E-43
UTP6	1.188894	3.9E-45
NSG1	1.18897	3.06E-36
LY6H	1.189037	2.99E-39
GLTP	1.189047	2.47E-48
SOCS4	1.189107	3.76E-48
CCL28	1.189275	4.29E-23
PFN2	1.189578	1.93E-30
SERBP1	1.189652	1.28E-44
PDSS1	1.18979	9.05E-51
CDKL1	1.189856	2.14E-41
JRK	1.189889	3.46E-40

FAM180A	1.18992	7.61E-43
AFG3L2	1.190029	1.54E-47
HOMEZ	1.190203	8.06E-50
HPCAL1	1.19025	1.22E-39
EEF1AKMT	1.19031	7.79E-51
NTF4	1.19032	9.24E-45
MID1	1.190441	5.69E-38
FGF2	1.190892	1.28E-44
STRN	1.190984	2.55E-46
CYYR1	1.191049	4.71E-36
CPNE3	1.191076	2.2E-43
CENPV	1.191081	5.89E-35
TERF1	1.191105	1.75E-49
AGFG2	1.191403	3.84E-33
PIF1	1.191464	1.06E-47
NAPG	1.191544	1.85E-48
TUFM	1.191606	5.66E-51
MPP5	1.191625	1.44E-49
UQCRB	1.191893	3.92E-51
ENOX1	1.191922	4.45E-47
ERCC5	1.191996	5.68E-45
PRKAB1	1.192025	1.47E-43
PHKG2	1.192105	1.91E-44
DENND1C	1.192119	9.7E-43
SAC3D1	1.192143	3.18E-45
FLVCR1	1.19237	2.19E-51
SLFN12	1.192734	2.12E-41
SAP30L	1.192748	1.14E-48
GNAI3	1.192929	2.66E-51
RNF168	1.193069	1.17E-49
IMPA1	1.193407	1.37E-43
CELSR3	1.193461	3.62E-37
MVB12A	1.193484	8.69E-43
MCRIP1	1.193492	9.53E-38
ABCA2	1.193556	3.33E-37
HSPA9	1.193557	6.29E-48
CHSY3	1.193621	1.04E-49
AKT1S1	1.193641	2.41E-47
FAM167A	1.193778	4.37E-39
CENPS	1.193847	8.69E-49
JAK2	1.193962	7.89E-43
VIPR1	1.19433	2.76E-28
RPGRIP1L	1.194939	7.05E-54
NAGPA	1.195401	3.59E-48
RNF220	1.195557	4.91E-48
IQSEC2	1.195683	1.95E-49
RBM22	1.196277	1.78E-48
SLC2A9	1.196368	8.6E-46
TRIM28	1.196442	1.56E-45
KCNMA1	1.196708	1.26E-28
ZC3HC1	1.196789	1.2E-50
NAP1L5	1.19693	2.04E-46
LYRM2	1.197436	3.19E-46
IKBKG	1.197647	8.05E-46
SLC34A2	1.19776	5.17E-16
NUDT8	1.197784	7.57E-34
ALKBH1	1.197896	1.19E-49
NXPE3	1.197901	3.64E-42
SLC4A1AP	1.198178	3.65E-50

NCSTN	1.198363	9.24E-50
ZNF766	1.198435	2.18E-49
OPA3	1.19848	4.89E-52
MTG2	1.198538	2.02E-46
ASB7	1.198679	6.38E-52
LAMB2	1.198812	1.43E-33
CTDSP1	1.198887	4.27E-45
AIMP1	1.198899	3.79E-49
CD3G	1.199288	8.19E-46
AKR7A2	1.199314	4.79E-48
ARRDC1	1.19943	2.2E-34
HDAC8	1.199458	2.4E-52
SDSL	1.1995	2.57E-47
ADORA2A	1.199932	9.27E-42
MAN2B2	1.199982	1.68E-47
EXOC1	1.200022	2.69E-47
CYREN	1.200104	1.31E-45
ZNF207	1.200235	2.48E-46
GNPAT	1.200305	1.22E-47
PYCR2	1.200704	1.3E-49
SLC12A6	1.20074	2.1E-42
NEDD1	1.200771	3.51E-46
KDM1A	1.200803	2.99E-47
RPL30	1.200924	1.87E-49
SYNGR3	1.201	5.85E-48
ZMYM2	1.20142	7.55E-44
SNAPC5	1.201638	1.48E-49
FRYL	1.201958	1.69E-46
CHMP3	1.202104	4.29E-49
FAM83B	1.202152	2.31E-40
TRAF3IP2	1.202551	9.55E-48
LRRC8D	1.202653	1.35E-47
RBMX2	1.202763	1.33E-50
DLG1	1.202769	3.06E-45
RELA	1.2029	4.73E-43
SIKE1	1.203012	9.99E-47
CDC23	1.203027	8.29E-49
SELENOP	1.203255	4.09E-25
LRRC49	1.203477	4.25E-47
TEX9	1.203665	3.01E-45
CCAR2	1.203982	1.81E-47
PURB	1.204116	7.22E-50
SOX21	1.204251	2.64E-46
SAMD5	1.204354	7.27E-49
SEMA5A	1.204462	2.47E-44
TAF12	1.205016	4.1E-48
NFKBIA	1.205497	1.93E-33
RO60	1.205804	1.01E-43
NDUFB10	1.205836	7.84E-52
WDHD1	1.205842	1.61E-52
ATXN3	1.206246	1.18E-43
PALMD	1.206409	2.79E-33
NHP2	1.206538	1.13E-49
ZNF526	1.206812	6.56E-54
DUT	1.206861	3.09E-45
ATP5F1C	1.206921	5.1E-49
FCGR3B	1.206991	2.03E-32
PFKFB2	1.207012	8.83E-38
MOB1B	1.207147	4.63E-45

AGGF1	1.207261	7.16E-50
CNPPD1	1.207305	1.36E-46
HOXD8	1.207372	8.65E-48
EML2	1.207491	3.81E-44
RPS11	1.207506	6.27E-50
PNOC	1.207558	8.21E-51
MBTPS2	1.20756	5.92E-50
TPGS2	1.208202	6.8E-45
AFF4	1.208411	1.02E-40
LPAR1	1.208587	3.66E-36
TMLHE	1.20865	3.5E-51
RNGTT	1.208662	4.97E-51
LEPR	1.208729	3.06E-35
HS6ST1	1.208788	1.26E-33
ENAH	1.208796	7.12E-31
COPB1	1.208944	3.19E-49
TP53I11	1.208984	1.21E-35
PIK3R3	1.208987	1.23E-40
SUZ12	1.20915	3.55E-49
CIR1	1.209184	9.39E-50
GYPC	1.209322	7.57E-30
HPDL	1.209474	1.96E-51
COQ8B	1.209502	2.57E-43
BRD3	1.209577	1.84E-45
FABP1	1.209633	7.89E-06
DLGAP4	1.20973	6.51E-44
TBX2	1.210163	4.16E-40
YEATS2	1.210215	9.22E-46
GFPT1	1.210287	2.82E-41
THOP1	1.210526	7.49E-44
PSMC6	1.210563	4.64E-49
TMEM117	1.210648	8.69E-51
RDH5	1.210866	2.79E-35
ADGRL2	1.210941	4.04E-35
HNRNPA1	1.21119	5.21E-43
MIS18A	1.211814	2.07E-51
PTPMT1	1.211842	3.95E-51
C16orf95	1.211984	3.55E-51
KIF7	1.212007	9.42E-47
RHBDF2	1.212612	3.32E-33
SLC35D2	1.212676	2.63E-47
GAL3ST1	1.212827	4.94E-31
TBX15	1.212924	1.04E-51
GPATCH4	1.213107	3.52E-47
PCDH12	1.213501	5.79E-46
INPP1	1.213585	1.46E-40
MYCBP	1.213733	1.91E-48
KIF16B	1.213815	2.49E-47
ALDH4A1	1.213912	2E-46
TRIM56	1.213936	9.03E-45
SNX16	1.214139	7.55E-46
NDUFAF1	1.214673	1.74E-48
TRPS1	1.214708	8.94E-43
DNAJC10	1.214864	4.88E-42
HADHA	1.214939	2.03E-49
SPAST	1.214972	1.16E-51
DAG1	1.215185	2.04E-42
SP6	1.215357	8.4E-54
PRPF4	1.215448	1.19E-46

ABCB8	1.215791	5.77E-46
MASTL	1.216015	5.17E-50
IPO9	1.216121	7.15E-49
RNF144B	1.216296	1.05E-44
XPR1	1.216325	6.63E-43
RAB37	1.216383	1.48E-38
TSEN34	1.216415	9.85E-48
GABRD	1.216524	1.22E-53
VAPB	1.216535	1.56E-50
SLC25A17	1.21666	2.97E-50
CTTN	1.216732	9.12E-40
FRMD8	1.216767	6.95E-41
UBE2Q1	1.216812	1.41E-48
PPP4R3B	1.216949	1.27E-48
BCL3	1.21715	1.04E-22
PRKX	1.217304	4.5E-38
SIN3A	1.217479	1.09E-47
AGTRAP	1.217563	7.39E-31
LIPE	1.217577	1.11E-31
MGMT	1.2176	1.34E-43
NSD3	1.217801	2.3E-46
TMEM71	1.217948	6.64E-45
SPART	1.218225	1.23E-33
AURKAIP1	1.21859	1.62E-47
TEDC2	1.218635	2.25E-51
TM2D3	1.218777	1.9E-48
CIT	1.219242	1.06E-48
MEGF9	1.219497	1.06E-46
NFATC3	1.219508	2.37E-46
ABHD15	1.219774	8.57E-48
URI1	1.219779	1.75E-50
C3orf14	1.219841	1.95E-39
IRF5	1.219934	1.53E-38
DNAJB14	1.21997	4.92E-45
DLG5	1.22013	9.57E-41
PRG4	1.220208	3.43E-37
OVCA2	1.220276	8.91E-47
GNPTAB	1.220332	9.03E-49
ATF7IP	1.22039	3.28E-47
MARK2	1.220452	2.11E-46
ZNF134	1.22052	1E-44
MGME1	1.220551	8.24E-48
PLEKHA1	1.220769	3.85E-40
CHCHD7	1.220806	5.14E-50
GDI1	1.220874	1.21E-41
ZNF260	1.220878	1.23E-48
PC	1.220892	1.65E-40
DNAJB13	1.220917	4.94E-22
BORCS8	1.22096	1.74E-48
PLEKHA7	1.221034	1.2E-34
DOCK6	1.221088	5.54E-38
BTN2A2	1.221181	1.92E-40
CFAP298	1.221552	4.4E-49
PPIL1	1.221695	1.59E-45
CDK17	1.22186	4.95E-42
ZNF609	1.222025	8.35E-46
TIMM29	1.222298	1.22E-51
MTHFD1	1.222443	1.13E-49
PET117	1.222652	1.35E-51

DNAJC21	1.222873	1.27E-50
ITIH2	1.222892	4.98E-44
TMEM79	1.222924	1.29E-45
EFHD1	1.223585	7E-34
EPHA1	1.224108	1.17E-40
GMEB1	1.22416	6.01E-50
TNFRSF25	1.224319	5.56E-31
KLHDC8B	1.22445	1.23E-40
BCCIP	1.224458	4.68E-53
CZIB	1.224834	2.35E-44
CNOT1	1.224913	2.53E-47
FAM104A	1.224967	2.83E-50
ATPSCKMT	1.225067	6.8E-51
RAB40B	1.225374	1.14E-45
ST3GAL5	1.225574	2.29E-39
CDK5RAP2	1.225592	7.81E-48
UTP14C	1.225666	2.79E-50
SLC31A1	1.226021	2.18E-48
FIP1L1	1.226369	4.4E-48
PANK3	1.22645	3.7E-45
UGGT1	1.226668	3.36E-49
ASH2L	1.226873	3.5E-51
CHRDL2	1.227059	3.2E-20
ENTPD8	1.227165	5.88E-24
BUD13	1.227182	1.53E-51
KIF9	1.22721	9.11E-47
NBN	1.227305	1.22E-44
CASP7	1.227619	8.01E-41
RRP15	1.227901	1.31E-45
ARIH1	1.227901	5.39E-45
ANK1	1.227937	2.16E-44
YIF1A	1.228043	1.12E-48
GZMM	1.228114	1.45E-48
DAP3	1.228132	7.76E-50
OLFM2	1.228359	1E-35
KDM5A	1.228361	1.15E-45
MED19	1.228477	6.16E-49
ZNF362	1.228511	5.72E-45
HEATR5A	1.22859	9.5E-51
NCOA3	1.228849	3.57E-46
SCAF11	1.228956	6.49E-48
SNRNP25	1.22913	2.15E-52
PCYT2	1.229135	4.88E-46
CNOT2	1.229167	1.97E-47
MEF2A	1.22917	1.7E-43
CLOCK	1.229194	7.33E-50
RPL7L1	1.229222	6.37E-50
SCGB3A1	1.229424	1.49E-06
DUSP10	1.229849	1.54E-34
PTPRM	1.229872	5.98E-41
RAD1	1.229887	1.53E-51
CREB3L2	1.230035	8.7E-45
CWC15	1.230289	4.94E-50
OTUD1	1.230339	6.79E-48
ERCC2	1.230417	1.68E-49
SYF2	1.230496	3.43E-48
EIF4A1	1.230497	3.76E-37
GPATCH3	1.230567	1.19E-51
MAST4	1.230636	1.45E-45

SIRPG	1.230702	3.07E-48
DNAJB12	1.230715	1.76E-50
STPG1	1.231001	1.29E-49
RHEB	1.231449	6.76E-47
LRPAP1	1.231456	8.97E-49
TFAM	1.231472	1.27E-45
CAND1	1.231533	1.38E-47
SUMF2	1.231726	9.47E-49
RRP36	1.231888	6.56E-52
HHAT	1.232084	8.82E-51
CCNDBP1	1.232181	2.46E-45
MRPS15	1.232517	2.58E-51
NDUFB7	1.232705	1.15E-44
MMAA	1.232705	2.06E-49
LILRA2	1.232722	1.57E-36
TINAG	1.232723	1.5E-51
REC8	1.23281	3.19E-26
SMIM32	1.232819	3.11E-36
DOK3	1.233212	6.19E-32
LRWD1	1.233476	2.75E-47
CTBP2	1.233494	1.73E-43
CCR5	1.233628	2.06E-42
CFAP97	1.233636	1.47E-43
RETSAT	1.233734	1.13E-39
ZC3H13	1.233768	8.18E-48
CLPTM1	1.233857	2.57E-48
CCDC59	1.233927	2.66E-49
CRTAC1	1.234016	2.29E-42
MOK	1.234078	2.37E-41
UBE2H	1.23417	2.78E-45
FOXC2	1.234299	7.99E-54
DPY19L4	1.23431	3.36E-47
GON7	1.234491	4.25E-50
METTL14	1.234497	1.08E-43
MOV10	1.234519	7.77E-39
ITCH	1.23479	2.69E-47
OSTM1	1.235049	6.97E-43
TBC1D22A	1.235208	5.45E-47
SLC9A9	1.235332	2.75E-43
GOT1	1.235384	5.65E-44
FH	1.235634	9.51E-52
SOGA1	1.235695	6.29E-49
COL4A5	1.235838	1.5E-39
ZBTB2	1.236135	6.88E-50
SLC16A13	1.236546	1.34E-48
TXNDC15	1.236856	1.36E-49
EXOC2	1.236925	2.27E-50
TACC2	1.237002	2.59E-43
CDC25C	1.237318	4.13E-55
BBC3	1.237401	1.48E-31
RTL6	1.237463	3.88E-46
WDR46	1.237583	3.69E-47
TMEM128	1.238338	1.43E-50
TRMT1L	1.23851	6.33E-51
FP565260.:	1.238576	1.05E-40
ACKR4	1.23864	2.79E-35
MORF4L2	1.238727	7.64E-46
PCGF5	1.238754	2.57E-49
ACAA2	1.238846	2.56E-45

NAGK	1.238855	2.33E-46
SMCO4	1.23893	9.15E-43
ESS2	1.239041	6.12E-50
DIO3	1.239146	4.69E-35
CNOT9	1.239153	1.68E-47
S100A8	1.239525	1.6E-16
PIK3C3	1.239671	2.4E-48
PIK3CD	1.23974	3.1E-37
GTPBP10	1.23995	1.35E-49
KCTD9	1.240069	4.73E-43
PKP2	1.24028	1.07E-35
DLG4	1.240385	1.58E-33
ZC4H2	1.240476	7.28E-48
CHMP6	1.240489	2.22E-47
STON1	1.240491	5.88E-40
ERP29	1.240666	6.03E-53
UBA6	1.241109	3.65E-41
FARSB	1.241315	3.32E-52
TNFAIP3	1.241511	3.71E-27
TRAF4	1.241532	2.88E-37
MFAP3	1.241626	4.38E-36
SLC25A1	1.241694	8.89E-46
SPINK5	1.241772	3.36E-42
BCAS3	1.241986	1.19E-50
KIF2A	1.241998	5.39E-43
COPS4	1.242005	5.22E-50
P2RX5	1.242055	6.59E-30
NAPEPLD	1.2421	2.76E-48
CEACAM2:	1.242225	2.99E-47
ING4	1.242236	7.45E-43
CDKN1B	1.24249	1.59E-45
ATP5MD	1.242577	8.04E-49
PRSS8	1.2426	2.61E-31
PTDSS1	1.242753	6.17E-45
PRPF38A	1.242995	2.92E-49
PPM1H	1.243182	1.3E-44
ILVBL	1.243183	3.09E-49
PLEKHA5	1.243237	2.44E-43
DDX28	1.243249	4.59E-52
PCIF1	1.243276	1.14E-48
YY1AP1	1.243531	8.18E-48
POLR2H	1.243753	1.39E-43
ABHD13	1.243803	1.05E-51
MRPL3	1.243925	3.17E-50
DHDH	1.24394	9.54E-54
TNRC18	1.244006	1.3E-41
RAB1A	1.244141	2.9E-49
UBXN1	1.244227	6.87E-47
PPP1R12A	1.244429	3.09E-40
CLN5	1.244512	4.42E-50
ANAPC15	1.244523	3.57E-50
RUNDC1	1.24458	1.78E-52
CDC42SE1	1.244698	1.02E-40
PCMT1	1.244758	7.14E-51
BCO1	1.244771	5.49E-54
IVNS1ABP	1.244816	3.36E-39
RPS20	1.244826	3.63E-46
LGR5	1.244927	3.35E-53
NXT2	1.245077	5.12E-48

CLPTM1L	1.245144	2.46E-51
TRIO	1.245161	2.42E-38
MRPL34	1.245175	1.09E-47
PPFIBP2	1.245243	1.34E-38
CCDC86	1.245308	1.9E-47
MESD	1.245376	1.9E-51
COQ7	1.245719	1.27E-52
PRR12	1.245745	9.73E-45
ATXN7L3B	1.245767	1.66E-49
JADE2	1.245831	3.37E-42
NIT2	1.245844	2.51E-48
PRELID2	1.245901	1.72E-40
CCDC153	1.246067	9.32E-51
PARG	1.246246	4.63E-50
GDI2	1.24653	3.09E-47
DPF2	1.246562	3.8E-48
STRAP	1.246584	8.95E-50
FAIM2	1.246667	1.1E-38
RITA1	1.246987	3.49E-52
CCNY	1.246988	6.71E-49
VPS29	1.247065	2.89E-48
OSBPL5	1.247098	6.05E-39
FNBP1L	1.24725	1.38E-41
UBE3C	1.247278	2.48E-46
CCNH	1.247708	1.65E-48
CSF2	1.247805	1.5E-57
MAP3K7CL	1.247904	4.23E-45
MAP3K2	1.247926	1.6E-47
UQCRC2	1.247934	2.23E-50
TRERF1	1.248082	3.15E-50
RRAS2	1.248144	2.94E-42
FBXL2	1.248152	9.98E-41
HDDC2	1.248446	1.19E-49
PCNP	1.248494	6.27E-46
NUP58	1.248562	2.53E-47
SDF4	1.24888	3.49E-48
SRPK2	1.249015	8.7E-47
COPG1	1.249059	3.22E-50
FASTKD5	1.249184	6.32E-51
DAAM1	1.249211	5.25E-41
FAM161B	1.249363	1.19E-50
CHM	1.249423	2.79E-50
PEX2	1.249441	1.61E-46
MPND	1.249475	7.51E-53
RBM47	1.249738	2.91E-43
ELP6	1.249795	8.01E-50
TERF2	1.249876	1.05E-50
HNRNPM	1.249898	1.99E-47
NR4A1	1.249904	2.57E-14
TLR2	1.250053	1.22E-33
MCTS1	1.250139	2.47E-53
ZKSCAN5	1.250293	4.66E-53
TSPAN6	1.250458	2.85E-43
PAFAH1B1	1.250517	3.54E-48
GIT1	1.250673	1.06E-47
MEIS2	1.250735	1.93E-36
CDC5L	1.251086	9.81E-52
GPBP1	1.251614	1.84E-47
XYLT2	1.251845	2.1E-49

FBL	1.251883	3.28E-47
ACACA	1.251986	6.71E-49
MRPL12	1.252337	2.09E-45
PGP	1.252394	1.89E-49
ILDR1	1.252576	8E-44
GEMIN2	1.252581	1.15E-48
STT3B	1.252773	1.84E-48
CPT1A	1.252943	9.92E-42
SSU72	1.253128	3.31E-51
TRIM2	1.253234	5.68E-42
L3MBTL2	1.253286	1.67E-49
HS3ST2	1.253314	2.18E-45
DCTN6	1.253513	4.2E-48
UBAP2L	1.253816	1.06E-47
UBLCP1	1.253826	2.03E-49
EHMT2	1.253877	1.47E-45
DIS3	1.254013	3.54E-48
DHRS3	1.254104	1.41E-33
PPA1	1.25417	2.33E-42
TMEM126E	1.254212	2.39E-47
SIGLEC1	1.254215	2.18E-32
CCNJL	1.254322	4.57E-51
OPA1	1.254324	4.43E-45
KIAA2013	1.254792	3.71E-50
ABHD14A	1.255085	3.2E-48
GMPS	1.255357	8.3E-46
LMAN2L	1.255935	4.36E-48
PI4K2B	1.256234	3.47E-49
GINM1	1.256254	2.93E-47
MLH3	1.25698	1.65E-46
CD38	1.256998	1.59E-43
MINDY2	1.25705	7.57E-47
GATD3B	1.257067	2.9E-40
ALDH9A1	1.257124	3.05E-50
COL18A1	1.257167	3.28E-31
TDRD7	1.257241	2.25E-49
ZNF317	1.257328	2.7E-49
DGCR2	1.257604	3.95E-48
ATP5MC2	1.257647	7.03E-51
CXADR	1.257831	8.09E-37
TRIM38	1.257887	9.72E-45
TAF15	1.257899	2.99E-49
AC008763.	1.258075	1.39E-21
CCNI2	1.258239	7.04E-45
DNAL4	1.25827	8.42E-49
CUEDC2	1.258554	1.96E-48
TTC8	1.258692	2.4E-46
CUL2	1.258845	5.52E-50
ERGIC2	1.259388	7.13E-47
SVIP	1.259399	1.04E-47
RRM2B	1.259426	6.17E-44
FAU	1.259445	6.44E-51
ABCB9	1.259531	8.88E-49
HCFC1	1.25958	6.81E-47
FNDC3B	1.259795	2.5E-37
NAPA	1.259998	4.43E-48
RABL3	1.260013	1.43E-50
TLR3	1.260049	1.34E-36
THOC7	1.260199	2.86E-50

SREK1IP1	1.260384	1.82E-48
CRCP	1.260608	4.22E-49
SGPL1	1.260637	4.43E-45
PA2G4	1.260816	1.22E-48
ATP6V0B	1.261111	1.14E-47
FANK1	1.261211	5.55E-43
OVOL2	1.261257	9.31E-40
GOLPH3L	1.261457	4.17E-45
AQP3	1.261545	7.81E-23
TRAPPC9	1.261823	9.84E-49
TBL1X	1.261831	3.23E-45
C8orf88	1.262251	1.18E-44
MORC2	1.262346	1.26E-47
FAM111B	1.262573	1.58E-51
PCBP4	1.263133	6.29E-42
SULT1A3	1.263182	3.39E-30
METTL18	1.263445	2.91E-51
SF3B5	1.263598	7.67E-51
SNX3	1.263699	3.41E-46
LILRA5	1.263882	2.4E-40
FIS1	1.263918	9.98E-52
LONP1	1.264202	1.31E-46
ADAM22	1.264376	1.27E-48
BRCA1	1.264557	2.62E-52
KIAA1191	1.264698	7.23E-48
ELP5	1.264857	1.09E-46
TMEM60	1.26492	1.22E-50
CCT2	1.265	2.25E-49
ALKBH6	1.265068	1.37E-44
QDPR	1.265132	5.55E-46
CMC1	1.265312	1.69E-48
CLP1	1.265436	1.6E-50
SLC15A4	1.265828	3.39E-44
PCBP1	1.265992	3.71E-43
CD70	1.266202	1.37E-53
SH2D3A	1.266221	5.82E-32
DCP2	1.266248	1.36E-48
TRUB1	1.266322	3.86E-50
ABHD16A	1.266758	1.83E-47
TARDBP	1.267057	7.91E-49
CNPY2	1.267181	3.03E-51
SPRR3	1.267198	1.41E-05
CCNF	1.267304	6.12E-54
CROT	1.267542	1.35E-50
GNAZ	1.267581	6.99E-43
SHLD1	1.267606	3.94E-51
ANAPC13	1.267703	2.64E-51
GCC2	1.267711	4.66E-43
ZMYND15	1.267781	2.67E-46
TMEM11	1.267886	2.48E-51
ZFP91	1.267997	1.28E-46
CCDC106	1.268007	8.86E-39
IER2	1.268312	3.41E-25
CYBC1	1.268553	1.02E-40
FGA	1.268584	5.32E-09
DOCK1	1.268787	4.94E-44
NUB1	1.268847	1E-49
CLCN4	1.268916	5.35E-52
NIF3L1	1.269107	3.13E-51

EGFL7	1.269209	4.12E-28
SCO1	1.269512	6.66E-52
SEC14L1	1.269706	1.74E-44
PIP5K1A	1.269845	3.02E-45
FCHO2	1.269916	6.06E-42
ZNF830	1.270007	2.8E-48
AMMECR1	1.270019	1.54E-52
TMEM168	1.270089	1.1E-37
SLC9A2	1.270143	1.13E-52
PPP5C	1.270375	3.86E-47
GATB	1.271147	5.62E-52
KIF22	1.27128	4.68E-47
ARHGAP21	1.271576	4.13E-42
UBXN2A	1.271729	1.09E-51
MAGT1	1.272397	9.31E-50
MED30	1.272478	2.08E-50
MKLN1	1.272598	5.3E-50
PPIG	1.27267	2.88E-47
CYB5D2	1.272676	7.17E-50
UBR4	1.273108	1.83E-46
CDADC1	1.273343	1.73E-53
PKNOX1	1.273746	1.4E-48
NKAIN4	1.274521	5.54E-51
CDK14	1.274607	2.78E-36
TRA2B	1.275122	7.19E-47
PSMB10	1.275439	6.3E-43
ITLN1	1.275458	1.28E-09
TGS1	1.275525	5.6E-50
UCN3	1.275594	8.04E-14
SYNC	1.275836	1.59E-45
UTP18	1.275879	2.13E-52
ERBB3	1.276045	1.34E-31
TMX2	1.276159	6.56E-52
COPE	1.276214	1.45E-50
SF3B2	1.276227	1.51E-50
IER3IP1	1.27668	2.95E-51
CYTH1	1.276793	7.4E-39
APEX1	1.276852	3.49E-47
NUDCD2	1.276972	3.22E-50
PSIP1	1.27717	4.7E-45
MDP1	1.277412	3.5E-49
KLF6	1.277475	1.44E-25
DOCK5	1.277535	2.88E-37
OXNAD1	1.277672	1.45E-51
FOXD1	1.277693	6.64E-50
SPRED2	1.277786	4.81E-46
SAMD13	1.277976	1.9E-46
MCC	1.278066	3.66E-45
C6orf132	1.278351	8.96E-34
ZNF148	1.278536	1.86E-50
CD207	1.278753	2.12E-49
SMPD1	1.278776	1.27E-43
EGFLAM	1.278778	4.04E-43
SMG7	1.278792	2.75E-50
INTS13	1.27896	7.37E-51
F2RL3	1.279024	1.18E-30
MINDY4	1.279252	5.47E-51
METTL21A	1.279325	1.12E-49
TMBIM4	1.279482	9.26E-49

SAMD8	1.279535	1.48E-47
CCZ1B	1.279649	3.41E-44
AKAP11	1.280027	4.45E-46
ZFYVE1	1.280208	5.16E-47
GPR34	1.280259	8.1E-31
HOXA4	1.280369	1.78E-45
CHI3L1	1.280475	3.06E-15
TARS2	1.280746	1.98E-50
ATXN1	1.280761	1.78E-46
PSMF1	1.280828	9.48E-48
ACOT1	1.281134	8.63E-48
SF3A3	1.28119	3.67E-49
USP4	1.28137	8.38E-47
ACO2	1.281445	2.25E-52
EPHX3	1.281515	3.19E-50
RANBP3	1.281857	4.29E-49
SNAP23	1.28188	1.28E-42
RAD23B	1.2819	5.86E-46
PRMT5	1.28203	4.47E-49
PTN	1.282174	2.66E-34
ARL13B	1.282229	4.38E-49
GPALPP1	1.282325	2.88E-50
RASAL2	1.282425	4.96E-41
PIGBOS1	1.282428	2.3E-50
RMDN3	1.283045	2.88E-50
TBL3	1.283276	2.7E-46
KLHL20	1.283356	5.49E-47
CRK	1.283534	8.74E-50
CLEC4E	1.283603	4.7E-30
RAD51C	1.283628	4.43E-49
FBXO45	1.283799	3.42E-54
MSH2	1.28386	3.56E-48
TMTC3	1.283961	1.31E-51
LYPD5	1.28421	1.77E-51
CASP10	1.284412	4.94E-43
GRWD1	1.284447	8.4E-50
SNRPC	1.284457	2.06E-50
COMMD2	1.284651	1.74E-46
THG1L	1.284953	1.44E-49
AL391650.	1.28516	3.22E-24
USF2	1.28525	2.77E-47
C4BPB	1.285333	4.88E-15
TMEM86A	1.285482	2.19E-49
SNAP47	1.285646	6.2E-52
BLOC1S5	1.285807	2.12E-51
RFFL	1.286009	9.26E-48
LSM3	1.286381	5.49E-54
EXOSC9	1.286388	1.48E-50
IPO7	1.28653	1.21E-43
FAM78A	1.286891	4.06E-45
INIP	1.28693	1.79E-50
IPO11	1.287203	3.98E-51
MAP1LC3E	1.287297	3.33E-51
RPS21	1.28737	6.46E-49
FUS	1.287451	3.62E-45
TENM4	1.287591	1.64E-48
RDH10	1.287649	3.17E-33
FARS2	1.287674	3.01E-53
FAM98A	1.288234	2.17E-49

RECK	1.288245	1.32E-42
SLC16A2	1.288336	5.97E-42
TM4SF20	1.288477	5.61E-34
DSG3	1.288552	1.5E-34
MRPL20	1.288684	3.26E-51
AMPD3	1.28871	2.84E-45
SETD7	1.28893	2.97E-45
FAM210A	1.288957	2.53E-49
GPR143	1.289178	1.29E-43
NELFE	1.28997	1.21E-51
ZNF585A	1.290063	4E-48
MTX2	1.290084	4.45E-51
INTS12	1.290208	3.73E-51
IMP3	1.290335	6.64E-51
CCDC102A	1.290365	2.25E-44
CTPS1	1.290768	3.07E-42
RMND5A	1.290781	1.2E-50
DIPK2B	1.290901	2.32E-41
EIF3C	1.290989	6.93E-43
HMG20B	1.291007	3.3E-43
GID8	1.291171	3.94E-52
BBS7	1.291514	2.34E-50
GAA	1.291567	7.41E-41
FDX2	1.291593	3.2E-48
RBM12	1.291826	2.41E-47
CKAP2L	1.291865	6.04E-56
CCDC137	1.292001	3.25E-42
TIGIT	1.292018	5.33E-49
CXCR6	1.292498	6.23E-42
FGFR1OP2	1.292678	1.03E-45
UBE2D3	1.29288	5.91E-48
SMURF2	1.293035	1.07E-45
MACROD2	1.293229	9.68E-46
FBXL3	1.293472	1.68E-46
DAGLB	1.293581	2.89E-48
RNF41	1.293606	3.31E-46
MALSU1	1.293735	1.03E-50
REXO2	1.293804	5.82E-44
TESC	1.294374	5.83E-30
CREB1	1.294489	2.42E-46
FAM131A	1.294589	4.33E-45
HEXIM1	1.294657	4.68E-47
TREX1	1.294708	5.29E-44
TAB2	1.295073	3.26E-47
TOR1AIP1	1.295244	5.05E-47
ZNF487	1.295269	1.73E-51
DCLRE1B	1.295482	5.35E-52
TCEA1	1.295551	1.97E-48
B3GNTL1	1.295563	4.79E-49
HAS3	1.295574	1.16E-38
GUCY1B1	1.295621	5.64E-39
SRC	1.295639	3.4E-36
ALKBH4	1.295652	1.55E-52
PCNX2	1.295674	5.18E-45
CADM1	1.295927	6.98E-35
FAM204A	1.296056	3.23E-51
DGKH	1.296207	1.24E-52
ZNF227	1.296224	2.32E-48
SEMA3A	1.296241	8.01E-53

NAALADL2	1.296466	1.56E-47
RPA1	1.296919	2.51E-48
NKD2	1.297041	6.74E-28
SLC41A2	1.297318	6.71E-39
GNPTG	1.297414	4.83E-52
ATF2	1.297645	1.62E-47
PEAK1	1.297688	3.82E-43
VPS41	1.297774	3.74E-46
OSBPL1A	1.298036	9.31E-40
MIPEP	1.29808	2.42E-51
DENND3	1.298264	7.51E-37
FBXL17	1.298337	3.14E-50
C12orf65	1.298376	1.55E-48
ABL1	1.298538	1.52E-43
CYB561D2	1.298863	2.72E-48
ZMIZ1	1.298898	1.15E-41
TMED1	1.29907	1.86E-51
CLIP2	1.299299	9.69E-47
RAB28	1.299373	4.09E-49
TAF1B	1.299406	8.73E-50
FAM167B	1.299438	1.04E-38
NIPBL	1.299685	1.31E-47
ETFA	1.299767	1.34E-49
ZNF281	1.299821	4.6E-47
SPATS2	1.299836	9.45E-50
FAM200B	1.299871	2.64E-49
BMP2K	1.300477	3.6E-46
SREBF1	1.300595	5.61E-41
SLC12A8	1.30064	4.93E-41
FMO1	1.300805	1.78E-29
PRKCSH	1.300867	1.48E-50
NUFIP2	1.301002	2.71E-47
TFCP2	1.301168	6.94E-50
TMX1	1.301362	1.48E-44
DOLPP1	1.301431	1.93E-51
OTUD7B	1.301537	2.88E-54
HNRNPUL2	1.301672	2.64E-49
ARF5	1.301845	1.7E-49
SMARCA5	1.302018	9.77E-49
OIP5	1.302027	2.67E-53
MEST	1.30205	1.74E-42
PPP1R21	1.30251	3.11E-45
SPOPL	1.302535	9.46E-47
BCL9	1.302707	4.82E-47
PES1	1.302858	2.5E-50
SLC25A13	1.302975	1.1E-49
ADAM23	1.303321	8.74E-47
RNF121	1.303707	1.02E-50
THEM6	1.304071	4.17E-44
TNFSF12	1.304347	3.37E-42
TRPV4	1.304399	1.77E-35
ZDHHC9	1.304674	8.63E-43
RCC1L	1.304948	1.28E-50
MRPS7	1.304985	1.54E-50
PDE4DIP	1.304985	5.56E-43
AGPAT5	1.305344	1.57E-51
SNX15	1.305397	2.98E-48
BACH1	1.305551	6.92E-43
CLEC16A	1.305573	5.75E-47

DDX56	1.305629	5.8E-47
NEO1	1.305633	4.28E-42
DAB2IP	1.305878	2.39E-41
CHST3	1.305925	9.92E-38
SLC50A1	1.306232	3.11E-42
CXorf56	1.306234	1.9E-50
CIAPIN1	1.306269	5.92E-49
RAD51B	1.306276	3.95E-50
ARHGEF6	1.306321	5.48E-40
PDGFRL	1.30642	7.8E-30
ATP2B1	1.306479	1.15E-42
DUSP3	1.306494	4.79E-49
CDK19	1.306514	4.42E-49
BOK	1.30652	1.52E-39
OSBPL11	1.306681	2.03E-49
GNA11	1.306763	4.37E-48
TLK1	1.306865	1.9E-50
DOCK8	1.307224	1.08E-37
ANKRD39	1.30748	2.98E-51
SDHAF3	1.307508	1.06E-49
GPRIN1	1.307739	4.69E-52
PIGB	1.308115	2.25E-49
YIPF4	1.309044	1.02E-50
RAB36	1.309243	1.38E-47
ACTL6A	1.309308	4.28E-50
DNAJC18	1.309381	1.84E-47
PCCB	1.309522	7.92E-55
LDB2	1.309585	3.63E-36
SERP2	1.309623	1.32E-44
ARMC1	1.309671	1.36E-50
PCSK2	1.309877	3.03E-09
ANAPC10	1.309883	1.62E-51
NOD1	1.310256	2.01E-43
GALK2	1.3103	3.14E-50
SEC22A	1.31063	1.24E-49
VANGL1	1.310803	3.78E-46
BAG6	1.310818	5.21E-47
FAAP20	1.310905	2.83E-35
DGCR6L	1.311011	3.49E-50
DSTN	1.311013	5.98E-47
SLC27A4	1.311184	5.09E-47
PAK4	1.311195	1.84E-42
SNX27	1.311231	1.59E-49
UQCC3	1.311312	1.8E-49
NXF3	1.311494	7.71E-20
RETREG2	1.311582	2.64E-50
FTSJ1	1.311667	1.68E-49
NELFB	1.311729	8.74E-50
NRBP1	1.312386	5.1E-48
WDR44	1.312633	1.53E-48
TUSC3	1.312663	7.66E-26
NEU1	1.312678	5.31E-46
OGFR	1.312686	2.02E-41
BRAP	1.312713	8.9E-53
PRRC2C	1.312828	1.4E-44
SNW1	1.312865	5.31E-51
PLA2G2A	1.313195	3.49E-05
TCTN1	1.313242	1.49E-46
KIT	1.313328	9.15E-41

SAMD12	1.313618	6.14E-47
EID2	1.313659	4.31E-51
IDH3G	1.313661	1.18E-50
EIF2S2	1.313687	3.35E-52
TNFRSF10[	1.313862	2.45E-41
YES1	1.313966	1.98E-45
L3MBTL3	1.313995	7.32E-48
TCTN2	1.314139	2.09E-50
AP1AR	1.314407	1.12E-47
FERMT2	1.314568	1.65E-35
WWC3	1.314644	3.52E-47
YTHDF3	1.314664	7.75E-48
MMP24OS	1.314665	1.01E-46
WDR12	1.315007	1.31E-47
KCTD2	1.315127	3.62E-48
CHCHD2	1.315169	1.07E-48
KIF15	1.315391	5.5E-54
CCDC32	1.316028	1.33E-48
RAB43	1.316136	1.01E-37
GXYLT2	1.316213	2.89E-40
ZSWIM1	1.316265	9.57E-53
SNX5	1.3164	1.21E-47
ZNF28	1.316407	3.3E-43
NUP98	1.316606	2.47E-48
TMED7	1.316678	1.13E-44
NCKIPSD	1.317014	8.05E-48
DGKA	1.317104	4.31E-34
IFT57	1.317246	5.5E-47
KPNA7	1.317346	3.54E-50
TCTN3	1.317352	1.51E-52
USP38	1.317477	1.02E-50
VPS45	1.317704	7.4E-50
ZNF462	1.317879	2.41E-50
PLEKHF1	1.317925	2.56E-38
PPA2	1.318013	2.66E-53
C1QTNF12	1.318222	1.27E-33
STRN3	1.318521	5.32E-43
SLC45A4	1.318645	1.08E-43
TBRG4	1.318688	1.91E-48
OAZ1	1.31883	4.98E-49
MEOX1	1.319001	3.56E-34
TUFT1	1.31915	4.05E-37
CDK6	1.319171	5.38E-46
GNB5	1.319407	3.07E-43
ZGPAT	1.319433	7.19E-47
ADORA3	1.319721	6.4E-33
NCDN	1.319885	3.74E-46
TMEM167E	1.319939	5.78E-49
TACO1	1.320423	8.02E-53
GINS1	1.320601	7.9E-55
SLC1A3	1.320643	2.54E-28
NDFIP2	1.32066	4.8E-44
THADA	1.320786	2.29E-49
HSD3B7	1.320879	1.03E-42
INTS5	1.3213	1.39E-52
NAP1L3	1.321308	4.32E-46
DNAJC13	1.321433	1.64E-48
HMGA2	1.32145	2.6E-37
TOP2B	1.321463	5.44E-50

CENPO	1.321505	9.9E-54
IL7	1.321779	3.96E-48
ARL1	1.321899	3.38E-50
EVI5L	1.322268	1.58E-51
MIER2	1.322407	3.74E-46
DNPEP	1.32268	9.55E-49
PDE1B	1.322763	1.12E-40
PPP1R37	1.322812	2.06E-44
TLCD2	1.322915	3.51E-51
CCR6	1.323608	1.75E-56
CYB561A3	1.323616	9.12E-48
RBM27	1.323676	6.07E-51
NUDC	1.323695	4.24E-51
MDH2	1.323717	3.5E-49
ACAP2	1.323943	9.1E-47
MATR3	1.324501	6.47E-50
VWA5A	1.324791	2.63E-40
EPPK1	1.32482	1.8E-42
STOML2	1.324971	1.58E-51
FHL1	1.325162	4.26E-27
SH2D3C	1.325181	8.03E-40
PPP2R2B	1.325331	6.06E-47
MT-ND6	1.325472	5.14E-23
SLC30A9	1.32589	4.53E-50
LARP6	1.325965	2.09E-42
TOMM6	1.326077	1.57E-53
NUP155	1.326105	7.75E-54
EIF2S1	1.326199	5.13E-43
UBE2W	1.326391	5.3E-48
RPL27A	1.326424	1.68E-49
PTPRU	1.326723	4.28E-34
ZWILCH	1.32705	2.23E-48
MTFMT	1.327053	5.68E-53
NUP214	1.327244	3.09E-49
EIF3H	1.327383	1.7E-50
FAM120AC	1.327502	1.02E-49
KIN	1.327524	1.22E-51
PORCN	1.327897	2.45E-49
KDM4A	1.327938	6.73E-50
APEH	1.327956	8.36E-52
EIF3CL	1.328064	1.28E-45
ALKBH5	1.328385	2.81E-50
VWA2	1.32844	4.02E-37
ZBED1	1.32865	3.15E-48
SLC12A9	1.328773	1.05E-47
YTHDF2	1.328902	2.58E-50
SLC4A7	1.329011	2.22E-41
MRPL21	1.329178	2.23E-51
RTN3	1.329181	2.89E-51
STX10	1.329527	3.21E-47
ARHGEF12	1.329545	7.84E-43
OVOL1	1.329568	1.02E-29
DECR1	1.329572	2.15E-49
ADA	1.329652	4.35E-39
NFU1	1.329728	1.06E-50
S1PR1	1.329843	6.15E-32
ZP3	1.330004	2.64E-30
PARK7	1.330139	3.66E-53
DDX58	1.330501	1.01E-44

S1PR2	1.330622	4.56E-49
TBPL1	1.330666	1.91E-47
SMARCA2	1.330962	4.2E-47
SIGLEC10	1.330965	1.95E-29
LAMB1	1.331131	1.55E-32
METTL4	1.33115	2.74E-50
LURAP1L	1.331281	9.49E-44
SYNJ2	1.33144	7.57E-50
LDLRAD2	1.331475	1.61E-46
NUDT21	1.33159	1.86E-45
CENPL	1.331651	1.44E-54
COX4I2	1.331723	6.55E-46
FNIP2	1.331822	3.47E-42
PRPF40A	1.331871	5.72E-46
IRAK3	1.332002	3.91E-39
PSMB7	1.332008	2.87E-52
CCT6A	1.332108	9.61E-50
VMA21	1.332361	2.51E-53
SAMD14	1.332609	2.2E-49
IMPACT	1.332705	1.86E-50
TMEM222	1.333002	3.42E-49
PRPF18	1.33322	3.94E-52
DENND6A	1.333336	3.63E-49
DAB2	1.333339	1.24E-35
FRRS1	1.333452	5.01E-38
VPS4B	1.333526	3.8E-50
NAA15	1.33358	4.82E-51
KREMEN1	1.333735	5.02E-46
GTF2A1	1.334131	1.6E-49
UBQLN1	1.334325	6.27E-50
EREG	1.334362	3.35E-43
BICDL1	1.334506	2.99E-36
IRS2	1.334655	1.99E-44
AMACR	1.334703	1.46E-46
BRD7	1.334747	2.92E-52
PAEP	1.335147	2.36E-56
DPP9	1.335156	2.93E-47
HSPA5	1.335195	2.57E-44
MAPK1	1.335445	2.09E-40
LNPEP	1.335448	2.76E-50
KLHDC3	1.335521	1.51E-50
CCDC124	1.33558	8.97E-42
RPL39	1.335663	2.18E-49
KCNH2	1.335878	4.75E-27
PIK3AP1	1.335891	7.44E-31
FAM135A	1.335911	3.84E-42
MRPL19	1.335929	2.63E-48
GABPB1	1.33601	4.22E-49
CISD2	1.336092	4.75E-51
SYNCRIP	1.336435	3.21E-46
RUFY1	1.336452	1.07E-48
TOB2	1.336563	2.17E-45
WBP11	1.336638	1.35E-49
CEP89	1.336673	2.87E-49
CMTM1	1.336901	3.84E-50
TMEM205	1.336931	6E-52
PAX8	1.336993	3.88E-27
C3orf80	1.337032	6.04E-51
CERS2	1.337042	7.08E-46

C2orf74	1.337338	2.2E-40
CEBPZOS	1.33755	5.7E-51
CNDP2	1.337779	1.38E-46
ATF5	1.337888	1.1E-42
ARMC10	1.337952	2.25E-49
IGSF8	1.338262	3.48E-48
CD1E	1.338289	1.84E-45
ROCK2	1.338444	5.26E-49
NOL4L	1.338489	4.43E-49
CLIP4	1.338543	4.73E-40
EMCN	1.339121	6E-36
BAG1	1.339154	5.57E-44
RABEPK	1.339607	4.71E-51
ID2	1.340101	1.47E-38
TOX2	1.340112	4.49E-48
CCDC90B	1.340215	5.18E-50
MPG	1.340242	2.55E-48
SH2B3	1.340356	4.26E-44
MRPS11	1.340499	1.05E-51
RIPK3	1.340791	4.93E-37
OLFML2A	1.341024	5.99E-44
DDX19B	1.341264	1.39E-49
ITGAE	1.341532	2.29E-47
AKAP13	1.34157	1.98E-42
LTBR	1.341652	7.72E-44
ATP8B2	1.341658	1.97E-36
ZNF644	1.342536	7.49E-49
GLE1	1.342829	4.86E-51
AEBP2	1.343219	8.24E-48
TMEM14B	1.34337	4.31E-52
FARP2	1.343427	2.63E-46
ELL3	1.343433	5.65E-37
P3H4	1.343586	2.11E-46
JAM2	1.343667	8.95E-37
PIH1D1	1.343701	5.66E-50
BTF3	1.344305	6.97E-51
PLBD2	1.344528	2.32E-47
NUP205	1.344556	1.68E-49
GSTM5	1.344704	1.36E-36
PTK2B	1.344712	4.26E-40
TMEM135	1.344859	4E-49
METTL6	1.345274	1.69E-52
CRYM	1.345329	5.63E-55
TRUB2	1.345359	1.02E-52
TBC1D9	1.345368	4.41E-40
INTS7	1.345375	1.03E-53
TENM3	1.345457	1.42E-43
C1orf53	1.345774	5.05E-50
ATG5	1.345874	5.95E-52
UBAC1	1.345971	2.42E-49
VSIG10L	1.345976	3.56E-47
NAIF1	1.346019	3.94E-53
CIP2A	1.34618	4.73E-52
MMRN2	1.346205	6.11E-43
HNRNPLL	1.346316	1.85E-48
HNRNPU	1.346428	2.36E-49
LTF	1.346573	9.91E-14
SLC39A7	1.346696	2.76E-52
FAM91A1	1.346704	2.57E-47

FAM234A	1.346712	4.43E-49
ECHDC1	1.346714	1.78E-49
RHBDF1	1.346919	8.68E-35
PARP12	1.347023	1.57E-42
MVD	1.3471	2.27E-40
DFFA	1.347202	6.61E-52
CEBPZ	1.347279	3.52E-52
CRKL	1.347405	1.58E-49
CDK2	1.34744	7.06E-42
GAS2L3	1.347451	1.1E-51
F3	1.347671	4.21E-15
ABT1	1.347681	6.64E-53
COPRS	1.347691	1.77E-50
CHD1L	1.348246	6.9E-48
TRAF3IP1	1.348329	5.94E-52
ERI3	1.348361	1.59E-51
BBS9	1.348408	6.73E-48
SSBP4	1.348489	5.65E-40
TRIM37	1.348504	8.15E-52
SLC39A6	1.348579	5.06E-44
TSPAN11	1.348673	6.95E-44
ERCC1	1.348715	1.29E-51
RHOD	1.348786	3.79E-31
COMMD8	1.348819	2.31E-49
TTK	1.348925	1.71E-53
SNX2	1.349045	2.29E-47
NDUFAB1	1.34909	3.14E-52
ISY1	1.349396	1.9E-50
ATP5PF	1.349723	3.94E-53
SPDL1	1.349892	4.78E-50
ZFP1	1.350723	2.43E-51
SUFU	1.350756	3.51E-50
NUMBL	1.351069	1.44E-38
PRPS1	1.351247	2.59E-48
UROD	1.351308	2.6E-49
UCHL3	1.3514	2.16E-50
CREG1	1.351457	5.8E-47
ADNP	1.351721	8.84E-48
AGA	1.351745	2.18E-49
HAUS7	1.351886	5.58E-47
IGF2BP3	1.351973	3.92E-27
AP1B1	1.352127	4.76E-48
PPP2R2A	1.352134	3.35E-45
PLVAP	1.352173	1.92E-37
WFS1	1.352367	1.1E-41
PSMD3	1.352375	2.96E-51
PDCD6IP	1.352482	8.93E-40
NRSN2	1.352538	6.9E-38
METTL8	1.352547	1.32E-48
GIMAP7	1.352646	4.56E-29
STAC3	1.352708	2.73E-48
TRMT61B	1.352712	5.44E-52
ONECUT2	1.352787	5.97E-33
ING1	1.352901	1.53E-50
STX12	1.353544	8.06E-47
C9orf78	1.35377	2.16E-50
CLPP	1.353825	1.07E-52
ST20	1.354046	1.08E-43
CAMK2G	1.354162	6.12E-47

MAPKAP1	1.354217	1.67E-51
KRCC1	1.354241	8.49E-49
FBXL5	1.354246	1.53E-48
ZNF629	1.354364	5.9E-52
OXR1	1.354414	6.3E-43
FZR1	1.35458	1.6E-48
ERBIN	1.354772	3.57E-44
RAC3	1.355008	6.56E-42
ATP5PB	1.355021	1.11E-50
SBDS	1.355353	1.19E-49
NT5C2	1.355361	4.63E-46
PIGX	1.355767	5.58E-54
NPSR1	1.356049	3.09E-40
FNTB	1.356385	2.11E-49
MAIP1	1.356542	6E-52
SNX10	1.356581	2.85E-30
MRPL55	1.356696	2.13E-47
C16orf72	1.356802	6.75E-51
C1QBP	1.357196	1.1E-50
TFIP11	1.357451	7.59E-52
SNX11	1.357527	2.36E-51
CHAF1A	1.3576	3.77E-50
CACNA2D1	1.35768	1.77E-46
SEC13	1.357682	1.19E-51
MRPL36	1.358366	9.59E-53
RSU1	1.358558	1.29E-40
PARPBP	1.358775	3.06E-54
GSDMD	1.358793	1.93E-38
MBIP	1.358859	2.05E-46
PPP2CB	1.358946	2.12E-47
HDAC2	1.358953	9.02E-50
SLC9A3R1	1.359022	1.37E-41
GYS1	1.359065	1.5E-48
CDK4	1.359155	2.15E-49
SNX21	1.359258	5.22E-48
IL6ST	1.359432	2.51E-39
CPNE1	1.35974	1.23E-42
FOLH1	1.359814	8.85E-54
TMEM68	1.359858	4.13E-52
UEVLD	1.360007	6.32E-51
NIFK	1.360128	1.97E-49
MOCOS	1.360163	1.33E-45
CPA3	1.360376	1.62E-24
NDC1	1.360394	3.37E-52
IL15	1.360473	1.72E-44
RTCB	1.360635	2.97E-50
ARHGAP31	1.361036	4.38E-48
ZNF880	1.361358	9.24E-40
MITD1	1.361534	2.74E-45
ATP5MF-P	1.361617	2.82E-52
TRIP6	1.361656	8.66E-37
CSRP2	1.361914	1.88E-36
INCENP	1.361922	3.41E-47
ZCCHC3	1.361922	3.15E-51
SYNPO2	1.36196	9.94E-33
SEC11A	1.362252	1.27E-50
MORN2	1.362269	5.62E-51
DYNC2H1	1.362646	2.34E-41
FJX1	1.362652	4.84E-36

DENND2A	1.362722	2.54E-48
PPTC7	1.363021	1.6E-49
ATP6V0E2	1.363275	1.26E-36
CLIC6	1.363352	1.87E-35
FAXDC2	1.363457	2.06E-37
POLE3	1.363755	1.33E-49
RPLP2	1.363956	3.98E-51
CCNQ	1.364531	8.22E-52
IRAK1	1.364787	1.44E-47
PITPNB	1.364812	3.53E-49
ABHD6	1.36516	5.13E-51
DDX27	1.365266	2.15E-50
EFS	1.365514	3.64E-43
EIF1AD	1.365531	7E-52
AHCTF1	1.365603	1.29E-50
ARHGEF18	1.365606	1.78E-44
AQP9	1.365942	1.76E-39
POFUT1	1.36595	2.06E-50
IFT43	1.36597	3.79E-49
PTP4A1	1.366034	4.33E-26
NOXO1	1.366405	4.66E-37
PPP4R2	1.366425	7.51E-48
AGAP1	1.366454	1.01E-51
TMIGD3	1.36668	2.17E-43
BICD2	1.366742	3.51E-45
LARP7	1.36681	2.34E-49
TTI2	1.366892	5.82E-53
PRXL2C	1.36692	9.11E-48
CA2	1.367027	1.98E-21
WDR70	1.367097	1.87E-51
TIAM1	1.367212	1.86E-45
AXIN2	1.367546	1.6E-42
PTRHD1	1.367697	2.99E-52
WAPL	1.367739	1.71E-51
TMEM102	1.367798	1.09E-47
SH3RF3	1.367904	3.45E-50
ARHGEF25	1.36847	3.74E-39
GPN2	1.368488	7E-52
ARAP1	1.368812	3.6E-41
GPX7	1.369267	2.11E-38
TCEAL4	1.369299	2.06E-45
GOLGA7	1.369365	2.23E-50
CD8B	1.369416	1.96E-42
ATL3	1.369451	8.3E-46
MAP3K8	1.369622	8.27E-36
HOXC10	1.36975	2.82E-29
L1CAM	1.369944	8.14E-31
PADI2	1.369964	4.91E-41
NDFIP1	1.370316	4.56E-51
MRPL1	1.370358	3.37E-53
SLC2A5	1.370385	1.19E-44
CDC6	1.370484	3.05E-45
RGL1	1.37107	3.86E-42
IGFBP1	1.371784	7.2E-48
STK17A	1.37182	9.05E-41
GNGT2	1.371855	1.58E-44
ATP1A1	1.371979	2.5E-44
MMGT1	1.37204	1.59E-51
EDAR	1.372104	1.02E-49

C15orf40	1.372176	2.16E-52
NFAM1	1.37231	5.15E-46
CLEC7A	1.372334	1.91E-30
RTL8B	1.372937	7.5E-43
WNT5A	1.372979	4.23E-33
PTBP1	1.373267	1.05E-50
SLC12A7	1.3733	1.7E-40
SEMA4D	1.373388	6.03E-45
TUBB2A	1.373583	6.93E-40
LEPROT	1.373628	3.74E-47
PSD	1.373756	1.73E-37
SPTY2D1	1.37394	5.84E-51
LLGL1	1.374059	3.31E-47
CLN3	1.374126	7.12E-50
SMU1	1.374375	6.7E-53
SFT2D2	1.374599	7.58E-52
MRPL11	1.374619	2.03E-52
GPN1	1.374663	5.27E-51
IL1B	1.374683	1.44E-40
GRTP1	1.374806	1.1E-36
AC105052.	1.374837	2.08E-28
CLEC3B	1.375006	6.53E-31
ADIPOR1	1.375419	1.51E-53
RASSF8	1.375883	1.06E-40
ANKRD13D	1.375924	9.37E-39
B3GAT3	1.375925	3.16E-46
BRMS1	1.376036	2.09E-51
PSRC1	1.3761	1.99E-50
SMARCA1	1.376152	1E-37
MRAP2	1.376242	5.69E-38
RBBP7	1.376424	1.23E-50
RAMP3	1.376468	1.09E-34
EXO1	1.376539	2.9E-56
NFIL3	1.376686	6.85E-32
PRIM2	1.376891	6.42E-53
STAMBPL1	1.376959	4.86E-50
TXN2	1.37701	2.39E-53
ERC1	1.377035	6.14E-48
KCTD21	1.377081	2.14E-52
AC093323.	1.377085	4.2E-47
AL139300.	1.377322	4.8E-32
ZNF768	1.377729	2.61E-48
LMAN2	1.377858	7.23E-52
ST8SIA4	1.37786	4.9E-49
SRXN1	1.377862	1.28E-45
AFAP1L1	1.378093	1.74E-46
CYFIP1	1.378154	1.99E-48
SMNDC1	1.378172	5.38E-48
GLB1L	1.37839	2.14E-50
HAUS1	1.378402	1.12E-50
ERAL1	1.378531	1.85E-52
ABCB6	1.378594	1.6E-46
DOCK7	1.378722	4.7E-50
IST1	1.378843	6.46E-49
GUCY1A1	1.378859	2.74E-38
RPL37	1.378969	1.4E-51
PTPRB	1.379029	1.12E-44
CNOT6	1.379395	1.16E-50
SPAG7	1.379742	4.05E-50

IL23A	1.379834	3.52E-47
TMBIM1	1.379913	2.98E-38
KLHL6	1.380158	2.28E-43
FOXP3	1.380191	2.02E-53
NANS	1.380566	2.64E-49
SC5D	1.380654	2.25E-47
CACNA2D4	1.380714	1.34E-42
PTPRK	1.380793	8.08E-43
UPK3B	1.381101	2.59E-21
IL12RB1	1.381188	7.97E-51
ARL5A	1.381205	4.01E-47
PREP	1.381308	3.43E-48
SNX24	1.381401	1.96E-48
DNAJC14	1.381955	6.05E-48
MAP3K6	1.381958	2.32E-37
UBE2V1	1.382073	6.3E-52
ENTPD5	1.382112	2.89E-48
SKA3	1.38212	6.02E-56
INPP5A	1.382122	9.59E-51
WWP1	1.382154	1.47E-44
DIAPH2	1.382306	5.8E-52
MUL1	1.38263	2.23E-50
LZTS2	1.382701	5.94E-42
MCL1	1.382702	3.11E-37
RNF149	1.382813	9.66E-46
CELF2	1.382876	8.08E-37
LSAMP	1.382892	2.6E-40
TFDP2	1.383327	1.98E-50
RBBP8	1.383408	1.3E-46
PANK2	1.383411	1.08E-49
CDC37	1.383527	2.82E-47
OSER1	1.383733	8.49E-49
C5orf24	1.383924	3.23E-45
TEAD1	1.383935	1.58E-42
CD81	1.384069	1.13E-41
RNF13	1.384352	1.06E-48
IQANK1	1.384377	7.86E-32
SMIM5	1.384763	4.54E-37
RPUSD2	1.385168	3.59E-53
CSNK2A1	1.385226	1.18E-50
SMC2	1.385261	9.1E-53
CSF1	1.385284	3.22E-31
CYLD	1.385382	1.67E-47
FHDC1	1.385511	5.38E-53
SIAH1	1.385914	1.1E-48
CREB3	1.386064	3.31E-49
TJP2	1.386065	5.3E-40
STK3	1.386162	5.54E-47
TM6SF1	1.386477	6.45E-42
ZCCHC9	1.38674	1.36E-50
TBK1	1.386976	3.3E-50
ZPR1	1.387122	1.8E-46
TSPAN17	1.387197	4.33E-48
ATP5PO	1.387548	2.87E-52
DBP	1.387607	4.03E-39
SSH3	1.387681	3.19E-38
HERC6	1.387914	2.98E-41
RBFOX2	1.388042	4.27E-45
DCPS	1.388051	2.64E-50

CPNE8	1.388212	1.48E-44
SRD5A1	1.388528	1.4E-48
POP7	1.388839	1.62E-51
BDH2	1.388911	7.38E-40
QKI	1.389303	2.53E-36
SBF2	1.389321	1.13E-46
ERG	1.38938	7.36E-42
PEX14	1.389533	7.67E-51
TNPO3	1.38992	9.14E-51
PIK3CA	1.390118	1.24E-45
CCSER2	1.39051	6.17E-50
MOB2	1.390573	1.15E-49
RNF14	1.390788	2.44E-50
PIK3R1	1.390803	4.5E-40
CASP2	1.390958	4.63E-50
HMGCL	1.391026	2.65E-52
RAET1L	1.391148	5.21E-47
PLEKHA3	1.391181	5.3E-45
GLOD4	1.391196	2.97E-52
SERF1B	1.391205	1.19E-48
FGF7	1.391214	2.95E-20
AMZ2	1.39136	8.86E-51
PIK3R4	1.391399	5.14E-51
CEP41	1.391611	2.81E-50
UCK2	1.391623	4.05E-50
SSH1	1.391668	2.11E-47
CTNNBIP1	1.391947	1.26E-50
BYSL	1.392064	6.56E-52
CDKN1A	1.392336	1.81E-29
EGFL6	1.392466	1.41E-54
SRGAP2C	1.392474	1.07E-46
EMC1	1.392495	1.27E-48
ATE1	1.392503	1.38E-51
B3GALNT1	1.392556	1.64E-47
HK3	1.39261	3.78E-32
JSRP1	1.392884	2.18E-42
CDC42BPB	1.392934	7.17E-44
CCDC102B	1.392993	2.29E-46
ZFYVE19	1.393002	9.35E-44
TAF8	1.393106	3.19E-52
RHOBTB2	1.393168	1.95E-49
TMEM115	1.39355	1.14E-51
ISCA2	1.39405	1.23E-52
ZBED2	1.394147	2.71E-51
MCOLN1	1.394344	2.93E-47
CD151	1.394469	3.45E-42
THUMPD3	1.394741	8.02E-53
CCNK	1.394762	1.24E-50
CSNK2B	1.394909	1.68E-50
CRLF3	1.395107	3.41E-49
PNO1	1.395166	5.36E-52
CNNM4	1.395387	8.16E-46
VPREB3	1.395466	7.67E-37
HAND2	1.395508	7.94E-30
ENOX2	1.395549	3.52E-51
SIDT1	1.39559	4.78E-44
CACFD1	1.395792	9.78E-48
SLC35A4	1.395802	2.84E-50
ATP6V0A1	1.39591	2.06E-45

FAM43A	1.396305	2.82E-43
CWC22	1.396715	1.71E-51
SRF	1.397192	6.85E-48
RNF34	1.397334	8.93E-51
TSPAN31	1.397678	4.98E-48
ANKRD27	1.397787	1.74E-51
ZNF469	1.397823	2.15E-52
SLC35A3	1.397979	4.63E-45
ADK	1.39804	2.75E-51
ZNF720	1.398107	2.73E-51
C4orf48	1.398324	8.19E-23
NHS	1.398373	1.23E-51
SH3GLB1	1.398413	1.23E-47
ATG16L1	1.398599	4.08E-50
MICB	1.398801	6.56E-47
CTCF	1.399016	1.46E-51
FAM3C	1.399284	2.68E-46
TAOK1	1.399317	2.64E-50
MYO6	1.399456	3.35E-42
PHF5A	1.399479	2.78E-52
FANCD2	1.399665	1.7E-47
NCK2	1.399806	4.46E-45
GATAD2A	1.399869	6.35E-49
PIGC	1.399997	9.28E-52
EBNA1BP2	1.400137	1.01E-52
VAPA	1.400164	1.42E-47
HLA-G	1.40031	1.39E-34
UGP2	1.400599	7.7E-50
SLC25A43	1.400728	8.46E-50
IL34	1.400815	1E-42
NOLC1	1.400877	8.14E-50
SV2A	1.401087	8.38E-48
RNF2	1.401288	3.26E-49
NASP	1.401364	5.17E-47
CSNK1G3	1.40145	1.48E-49
TRIM34	1.40169	1.14E-44
ELF1	1.402039	3.26E-44
LGALSL	1.402055	1.23E-52
TCAIM	1.402473	1.41E-52
AMOT	1.403006	7.11E-49
SPPL3	1.403055	1.15E-50
TCF7L2	1.403267	4.42E-41
GLUD1	1.403315	1.44E-50
DSN1	1.40373	2.34E-52
ZC2HC1A	1.403791	2.63E-46
SYNJ2BP	1.40416	2.34E-51
PMF1	1.404258	3.23E-51
DDX3X	1.404609	9.89E-45
ZNF511	1.404727	4.56E-51
MRPS23	1.404746	3.59E-51
MPDU1	1.404821	6.72E-52
ELP3	1.405088	9.51E-52
DDX6	1.405108	1.5E-50
CTNNAL1	1.405127	4.99E-46
SNF8	1.405201	1.61E-51
PRKG1	1.405293	1.32E-47
ATXN7L3	1.405375	2.34E-49
MECP2	1.405394	5.93E-50
CLDND1	1.405577	2.23E-47

KBTBD4	1.405849	2.45E-47
MOSPD3	1.406153	5.02E-49
KRTCAP2	1.406238	9.36E-55
RALGPS2	1.406501	6.62E-50
FBXL19	1.406544	1.03E-45
SNX4	1.406967	1.14E-50
TM6SF2	1.407044	4.13E-49
DEDD	1.407065	2.11E-51
SLC3A2	1.407155	3.62E-45
CSF3R	1.407186	5.61E-26
PFKL	1.407304	4.68E-47
MAP6	1.407384	2.34E-38
CLCN3	1.407558	1.04E-47
RPP25	1.407618	8.9E-48
SLC11A1	1.407873	3.66E-20
PEX13	1.408171	4.13E-52
TM2D1	1.408235	6.33E-51
ACSL1	1.40825	2.32E-37
ATXN10	1.408307	1.04E-50
ERMP1	1.408577	8.78E-51
PARL	1.408781	4.68E-53
ITGB3	1.408908	5.85E-49
NR2C2AP	1.409022	1.2E-51
HYAL1	1.409054	1.2E-27
ETF1	1.409399	3.77E-50
SSX2IP	1.409426	2.96E-49
PLPP1	1.409544	7.11E-44
HIBADH	1.409576	2.16E-51
ERF	1.409662	3.18E-45
FBXO22	1.409741	1.04E-49
NR3C1	1.409775	7.99E-46
SIT1	1.409887	6.96E-47
TRAPPC12	1.409969	1.19E-48
PKP4	1.409983	1.76E-49
PAPLN	1.409989	1.2E-32
CUTA	1.410018	1.3E-51
SCIMP	1.410258	2.55E-44
KPNB1	1.410348	1.03E-48
RBBP9	1.410909	2.07E-52
FAF2	1.411096	1.96E-51
OAZ2	1.411148	1.01E-47
DUOX1	1.411158	7.3E-42
MANBA	1.411221	1.71E-46
ITGA9	1.411282	3.07E-49
RERG	1.411379	6.26E-38
LRBA	1.411445	3.12E-50
RCAN1	1.411528	3.39E-39
YIPF6	1.41166	2.52E-52
TDP2	1.411853	7.03E-51
PUM1	1.412031	3.56E-49
MITF	1.412323	4.93E-40
MRPL22	1.412334	5.12E-53
SUCLA2	1.41234	7.88E-50
ZNF408	1.412406	8.98E-52
RILPL1	1.412447	1.73E-47
BTG3	1.412532	1.29E-43
RPP25L	1.41262	3.63E-52
MBD5	1.41271	5.3E-50
COX16	1.412721	4.33E-54

CAMLG	1.413063	8.14E-50
PDHB	1.413212	4.27E-52
EMC8	1.413546	2.97E-52
STN1	1.413571	4.61E-47
FAM220A	1.41363	2.08E-52
COQ2	1.413733	1.51E-52
TNFRSF19	1.413736	5.1E-39
CRTC3	1.413835	2.74E-48
DNAJC9	1.41425	1.48E-51
GTPBP4	1.414255	4.91E-49
HOXA5	1.414297	1.03E-46
SMUG1	1.41431	5.91E-52
ATP5MPL	1.414363	5.87E-54
TRIP10	1.414509	2.54E-34
AATF	1.414775	1.61E-51
MEX3C	1.414786	3.11E-43
YME1L1	1.41479	4.21E-51
SNRNP35	1.415029	6.07E-50
LRR1	1.415459	9.2E-53
MSI2	1.41559	5.91E-48
DDR1	1.415672	5.98E-41
BRI3BP	1.415703	6.39E-52
DIP2B	1.415741	4.24E-51
RNF8	1.415889	1.24E-52
HNF4A	1.415903	1.42E-40
DNMT1	1.416132	5.14E-49
VPS37A	1.416185	3.58E-49
SNCAIP	1.416281	3.02E-50
LYNX1	1.416353	1.86E-24
CCNI	1.416903	4.33E-48
PDXK	1.417244	1.1E-49
GCC1	1.417272	3.75E-53
NR2F1	1.417633	3.52E-35
EIF3I	1.417636	1.62E-51
IL6	1.417638	2.45E-26
APOBEC3E	1.417644	3.48E-39
DCUN1D1	1.417678	7.48E-49
PPP1R7	1.418174	4.27E-52
TNK2	1.41828	6.9E-35
RNF122	1.418303	5.52E-42
POLR3H	1.418495	4.67E-52
MED6	1.418558	5.4E-52
SNX18	1.418558	3.99E-49
DNAJB11	1.418744	2.51E-53
FRMD5	1.418749	1.51E-54
TMEM179E	1.418752	6.3E-52
NUDT15	1.419144	1.57E-52
TTC9C	1.419537	5.61E-50
GNPDA2	1.419682	1.34E-50
CLEC4A	1.419956	3.81E-42
SLFN11	1.420235	2.38E-35
CHI3L2	1.420397	2.09E-43
SLC25A32	1.420509	2.02E-49
AK2	1.420697	3.72E-52
SMAD6	1.420765	7.92E-42
MMP3	1.42085	1.21E-46
IDI1	1.420972	2.83E-44
KTN1	1.421074	1.62E-50
OXSR1	1.421078	3.03E-48

HEG1	1.421084	2.59E-34
LILRB2	1.421126	2.07E-37
GTF2H3	1.421318	1.01E-49
CHCHD6	1.421568	3.69E-53
MRPL57	1.421853	8.56E-53
TM9SF2	1.421864	1.22E-52
POLR2D	1.421958	7.45E-53
SH3TC1	1.422019	4.71E-42
ITIH3	1.4222	5.26E-43
DYRK1B	1.422273	8.82E-46
PLXNA1	1.422351	1.58E-40
POLD1	1.422517	1.37E-43
CUEDC1	1.422573	3.26E-45
SETD3	1.422623	1.59E-52
TNFRSF10(	1.422669	9.15E-49
TOR1B	1.422905	3.78E-52
FASN	1.422979	9.91E-36
DQX1	1.423085	1.63E-42
SMAD2	1.423089	4.59E-46
BABAM1	1.423129	2.52E-51
NSMCE1	1.423221	4.01E-52
CILP	1.423448	5.6E-18
CCDC43	1.423487	9.37E-54
SLC25A23	1.423496	4.54E-48
ZNF428	1.423553	2.15E-49
CD22	1.423799	3.31E-37
IFNAR1	1.423868	4.42E-51
MED7	1.424244	1.95E-50
NPM3	1.424672	1.68E-50
UBA3	1.424894	2.96E-48
PLK4	1.424986	2.73E-54
NAT1	1.425038	5.77E-49
ANXA2R	1.42531	4.19E-49
PPM1M	1.425466	7.66E-44
BAG4	1.42578	1.12E-52
PITPNM1	1.425802	1.43E-39
AFTPH	1.425997	3.51E-53
IGF2R	1.426629	6.81E-47
CACNA1H	1.426725	1.51E-38
GAS6	1.426774	1.83E-39
RC3H2	1.426845	6.08E-51
XKR9	1.427018	2.52E-46
SH2D4A	1.427067	1.69E-36
SORCS2	1.427427	9.7E-44
TNNI2	1.427564	1.89E-17
KIAA0232	1.427988	2.42E-51
TLK2	1.428138	5.4E-51
SOX7	1.428304	2.13E-43
INHBB	1.428432	3.56E-36
LRRC8C	1.428455	1.51E-48
MRPS30	1.428773	1.71E-54
VSNL1	1.429063	7.55E-49
CFP	1.429432	1.17E-39
APBB1IP	1.429449	2.51E-33
GTF2B	1.429768	6.35E-52
HMGN1	1.429952	1.72E-48
PCTP	1.4301	7.25E-51
LCLAT1	1.430116	5.21E-54
PDIA4	1.430354	8.95E-44

LRRC66	1.430401	3.78E-50
RNASEH2C	1.430636	1.48E-50
IQCE	1.430749	2.34E-50
PSME4	1.430827	1.56E-48
DCTD	1.430963	2.03E-51
FN3KRP	1.431126	3.75E-52
STYXL1	1.431381	8.63E-52
EIF5A	1.43152	1.27E-51
PNPLA8	1.431565	4.23E-48
FGR	1.431769	3.23E-30
SHISA4	1.431881	1.44E-43
TMEM39B	1.432037	1.29E-50
C19orf47	1.432289	1.37E-52
AKNA	1.432307	3.56E-37
MFN2	1.432577	7.86E-51
GTDC1	1.433228	3.13E-45
FAM104B	1.433272	2.09E-52
HSPE1-MC	1.433293	2.91E-34
DDIT3	1.43338	5.49E-41
PDS5B	1.433402	6.95E-48
MED10	1.433437	4.27E-52
NMB	1.433783	5.72E-46
FAM189B	1.433898	2.95E-47
SPIN1	1.434111	1.06E-46
NFATC2	1.434305	3.24E-51
NDRG3	1.43431	2.32E-51
MCEMP1	1.434377	1.68E-35
TFG	1.434681	7.52E-50
GTF3A	1.434764	3.07E-55
SPAG1	1.434884	2.57E-48
ITPKB	1.435016	1.26E-47
NDUFA5	1.435097	9.83E-51
VIRMA	1.435199	1.39E-52
USP11	1.435348	3.26E-46
MRPL30	1.435371	3.26E-54
NR1H3	1.435394	8.38E-40
ACOT8	1.435454	3.75E-52
FAM120A	1.43572	8.72E-51
WDR83OS	1.435814	6.33E-53
GPD1	1.436077	2.02E-27
LIMCH1	1.436394	5.24E-44
SLC39A9	1.436694	9.27E-55
NOL11	1.436809	1.63E-52
RNF24	1.437123	1.39E-42
CEBPG	1.43714	2.25E-49
ZNF410	1.437177	6.84E-50
PRKCA	1.437518	2.03E-48
CLTA	1.437733	1.99E-49
FAM81A	1.437783	4.53E-55
TRMT12	1.437832	7.71E-55
BZW2	1.437861	1.45E-46
SLU7	1.438041	1.15E-52
MAP4	1.4381	3.04E-47
SCARB2	1.438259	1.2E-49
FAM72A	1.438463	1.88E-53
GPA33	1.438552	1.92E-53
CABP4	1.438767	5.33E-44
AC004832.	1.438795	1.3E-44
MRPS16	1.438803	4.21E-53

ZCRB1	1.438858	3.43E-52
GALC	1.438922	1.59E-47
BLOC1S3	1.439435	2.47E-53
MRPL35	1.439445	5.88E-53
KLHL2	1.43992	1.18E-47
RCC1	1.440131	3.13E-38
MRPL51	1.44014	8.63E-53
RLIM	1.440219	2.13E-51
ASB13	1.440386	3.05E-46
VPS28	1.440976	5.48E-51
FARSA	1.440986	1.28E-52
CLINT1	1.440992	7.03E-46
BRF2	1.441013	2.35E-53
RTKN2	1.44113	1.26E-54
PAIP2	1.441179	2.54E-49
PRKCH	1.442464	1.2E-42
PLCG2	1.442596	2.6E-41
DST	1.442629	1.45E-45
SMC4	1.442744	1.09E-46
IDH3A	1.442866	2.84E-47
CD5	1.442894	1.87E-49
RAMAC	1.443004	3.69E-53
FBXO8	1.443124	3.08E-51
ASNSD1	1.443192	3.63E-53
TMEM238L	1.443305	3.78E-38
USP41	1.443496	2.51E-24
PRPF19	1.443551	1.46E-53
GPR39	1.443598	3.8E-44
TMEM230	1.444122	6.23E-51
NDUFV2	1.444339	4.45E-52
INAFM2	1.444667	6.18E-51
MNS1	1.444684	2.2E-51
AC068580.	1.444708	7.19E-46
SPIRE1	1.444868	6.7E-44
SP1	1.444921	1.44E-47
FAM98B	1.444939	9.19E-52
HMGCR	1.445012	1.58E-45
MFSD11	1.445132	1.86E-51
PSMG2	1.445134	3.69E-52
UBE2D2	1.445151	1.86E-50
KIAA1143	1.445249	1.87E-51
OLA1	1.445387	1.8E-50
SYMPK	1.446167	5.03E-46
KDSR	1.446588	5.01E-47
ABCF2	1.446723	2.11E-51
ACP6	1.446986	2.23E-45
PTPN11	1.447287	6.38E-48
CHMP4A	1.447638	6.82E-49
KNSTRN	1.447799	8.85E-51
IL18BP	1.447811	1.94E-35
CHST15	1.447859	8.68E-39
TMEM185B	1.447864	6.75E-51
GSPT2	1.447998	5.84E-43
DYNC1LI1	1.448087	8.15E-52
HPS6	1.44816	2.03E-53
TIPRL	1.448169	3.08E-53
GFOD2	1.448183	4.78E-51
TMEM177	1.448287	1.96E-51
MED18	1.448304	5.06E-52

ABRAXAS2	1.448512	1.87E-53
COL13A1	1.448755	1.02E-45
KDM2A	1.448865	1.06E-47
C16orf87	1.449431	1.71E-51
FUCA2	1.449601	9.52E-53
POLRMT	1.449879	6.61E-47
NFATC4	1.44997	3.91E-35
MRPL46	1.450175	2.79E-53
PRUNE1	1.450333	4.21E-53
PHF13	1.450661	9.68E-50
XIAP	1.450706	3.13E-53
CAMK1	1.450715	4.91E-44
RIOK3	1.450775	7.53E-47
TSEN15	1.45086	7.82E-53
MEAF6	1.450964	5.69E-49
MTFP1	1.45101	2.42E-43
NGEF	1.451066	2.51E-37
RASGRF2	1.451284	1.06E-47
SLIT2	1.451475	6.3E-38
MAP3K13	1.451606	1.63E-51
UHRF1BP1	1.451862	4.36E-48
TAF6	1.452243	1.96E-48
RAD54L	1.452254	4.76E-53
FAM168A	1.452424	1.39E-47
HSPB2	1.452678	1.95E-47
STAU2	1.453016	5.39E-50
FNDC4	1.453132	1.47E-25
PLB1	1.453218	1.65E-49
DNAL1	1.453524	3.05E-54
COPS9	1.453879	5.83E-53
RNF139	1.454181	3.31E-51
NCOR2	1.454525	2.12E-44
KIF18B	1.454793	3.01E-53
VTI1B	1.455096	2.84E-53
SEMA4A	1.455149	9.76E-43
HMGN2	1.455478	2.98E-48
ECD	1.455551	8.56E-52
IRF2BP2	1.455694	4.02E-50
CXCL1	1.455719	2.7E-18
NID2	1.455721	4.98E-36
JKAMP	1.455842	7.67E-51
MIEN1	1.455992	3.42E-51
RELL1	1.456006	6.64E-47
PRKDC	1.456228	1.84E-46
C20orf27	1.456369	1.33E-49
SHARPIN	1.456699	1.03E-49
FTO	1.456784	1.28E-49
RPP38	1.457066	4.83E-53
ICAM3	1.457337	5.06E-48
AXIN1	1.457368	2.14E-51
AP5M1	1.457394	8.73E-50
SNAP29	1.457485	6.28E-53
ZNF226	1.457592	8.74E-50
DHRS1	1.457845	2.3E-45
ACER3	1.458185	1.53E-52
RNF187	1.458326	1.18E-52
MAF1	1.458351	2.13E-52
TRAPPC4	1.458566	3.4E-50
BLM	1.45861	6.52E-55

DCAF12	1.458644	1.62E-50
ZDHHC14	1.45869	2.02E-48
ADAMDEC	1.458702	4.79E-49
PTPRG	1.458957	2.97E-46
EXOSC3	1.45902	3.51E-50
PARN	1.459156	3.14E-52
SNX17	1.459589	1.61E-51
TBCC	1.459914	5.18E-50
FBLN7	1.460086	1.12E-46
CFAP36	1.460347	5.31E-51
MED4	1.460496	5.76E-52
PDK3	1.460556	1.17E-50
RASAL3	1.460598	1.61E-39
SOCS5	1.460947	4.79E-48
CR2	1.461207	1.51E-26
TMEM203	1.461357	4.53E-53
EIF3K	1.461362	1.77E-53
TRIP4	1.461381	3.19E-52
ARHGEF9	1.461462	5.14E-52
NT5C3A	1.46154	3.23E-47
ZNF574	1.461906	4.87E-53
MAP3K20	1.461949	1.94E-43
CLN6	1.461977	5.06E-52
IL11	1.462113	2.49E-50
KLHL7	1.462317	1.95E-50
ITGB8	1.462486	2.31E-38
TNNC1	1.462884	2.21E-32
STX8	1.463414	6.56E-52
IFIH1	1.463968	8.31E-44
LOXL4	1.465026	3.62E-32
XYLT1	1.465045	7.73E-46
PPP4R1	1.465114	6.95E-48
LSM14A	1.465788	7.06E-50
LIMS2	1.465995	2.09E-37
KLF3	1.466121	7.49E-45
VPS35L	1.466223	1.71E-48
M6PR	1.46625	3.83E-50
JAK1	1.466531	2.3E-46
STAG2	1.466572	1.27E-50
XPO6	1.46684	4.87E-49
ADRM1	1.466921	2.62E-50
NDUFB9	1.46721	2.7E-54
PSME3	1.467333	1.18E-50
MFAP1	1.467353	6.48E-51
TCEA2	1.467578	2.92E-44
PPIB	1.4676	6.23E-53
CHTF8	1.467685	6.7E-53
RNPEP	1.467739	1.87E-50
SFRP1	1.468034	1.6E-23
TXNDC12	1.46814	8.9E-55
STAT5A	1.468354	1.21E-45
RTN1	1.468402	1.54E-27
WDR53	1.468783	5.23E-52
PRXL2A	1.469355	3.91E-52
CHMP7	1.469454	2.91E-51
APOO	1.469705	8.71E-54
DARS2	1.469912	7.81E-53
HOXC9	1.470052	3.09E-50
ANXA4	1.470408	1.47E-33

HILPDA	1.47069	2.56E-36
CORO7	1.470792	2.37E-45
COX5B	1.470813	4.56E-52
WASHC5	1.47086	2.44E-52
RNH1	1.471622	1.04E-48
SYDE1	1.471868	7.67E-43
TBC1D10B	1.472098	1.27E-50
TNFSF4	1.472105	1.75E-48
KRT20	1.472387	1.5E-30
NCKAP1	1.472426	3.55E-46
PGGT1B	1.472456	5.63E-48
DHRS7B	1.472581	1.69E-54
CMPK2	1.472838	4.91E-42
BTN2A1	1.472905	3.68E-47
MICAL1	1.472915	1.32E-38
TSHZ3	1.472942	1.25E-46
ERGIC1	1.472967	3.49E-48
JRKL	1.472996	8.43E-48
COPG2	1.473063	2.94E-49
PSMA3	1.473463	2.71E-51
MEPCE	1.473873	1.74E-51
HELLS	1.473998	4.71E-54
ASL	1.474285	6.27E-46
RCAN2	1.474362	1.06E-41
RNF141	1.47437	3.05E-51
AKIRIN1	1.474446	2.99E-49
CLDN5	1.474771	2.13E-29
FER1L6	1.47482	3.57E-45
CDC27	1.474885	5.8E-51
TRAPPC3	1.47494	1.14E-52
SERPINB7	1.475297	4.88E-47
ADD1	1.475368	1.96E-48
PHYHIP	1.475378	3.3E-47
ACOT9	1.475764	8.82E-44
C1orf43	1.476301	1.34E-53
ACTR6	1.476394	2.2E-50
STUB1	1.476583	6.59E-54
MKKS	1.476644	2.75E-53
SCOC	1.476871	2.47E-47
HNRNPUL1	1.477058	8.86E-51
ERLIN1	1.477175	1.39E-48
MME	1.477234	2.96E-41
DUS2	1.477312	3.73E-51
LSM1	1.47755	4.04E-53
HTR2B	1.477565	6.51E-49
IKZF1	1.477804	1.4E-43
RPAP3	1.477873	4.63E-52
ETNK1	1.47822	1.21E-47
RABAC1	1.47845	8.04E-49
C11orf86	1.478956	3.26E-55
TMEM139	1.479149	1.01E-32
STX4	1.479268	7.34E-48
ID3	1.479414	6.36E-34
ATP6V1C1	1.479438	5.02E-50
PIBF1	1.479896	6.99E-52
XRCC1	1.480463	4.79E-51
FAM210B	1.480738	3.85E-51
INO80C	1.480843	1.83E-50
UQCC2	1.48086	1.43E-53

SUGT1	1.481109	3.22E-52
PVR	1.48242	1.17E-44
RBP4	1.482511	1.7E-21
EPS15	1.482831	4.3E-48
VIL1	1.483274	1.91E-20
SPSB1	1.483625	8.27E-33
RPL17-C18	1.483637	3.07E-33
RPF1	1.484025	2.71E-51
GPSM2	1.484136	1.83E-48
PRKACA	1.484277	1.84E-49
NIPSNAP3A	1.48428	1.66E-49
NRG3	1.484457	4.37E-50
CSPG4	1.484535	3.48E-47
NPNT	1.484635	3.79E-33
PNRC2	1.484887	1.45E-47
HEBP2	1.485219	1.25E-49
FLT1	1.485634	7.16E-42
SLC25A44	1.485831	3.52E-52
DRAM1	1.486123	2.7E-45
EIF2AK4	1.486813	1.29E-50
TMEM41A	1.486854	1.2E-52
TOR3A	1.48701	2.31E-52
RTRAF	1.487407	1.63E-53
KRT80	1.487442	8.67E-28
TMEM167A	1.487483	7.17E-52
LHFPL2	1.487652	1.82E-42
ZC3H12A	1.487894	2.45E-31
LRRC1	1.487995	3.47E-49
CLIC5	1.488274	1.07E-47
RBMS2	1.48866	8.31E-45
HPRT1	1.488955	2.27E-52
SMURF1	1.489118	5.33E-44
SYNJ2BP-C	1.489191	1.13E-31
SRSF3	1.489249	4.27E-47
LRRC17	1.489481	4.72E-40
SNX29	1.489481	1.7E-50
ANKRD22	1.489602	5.23E-24
TMEM87A	1.489635	1.06E-49
ATP11B	1.489707	4.7E-46
NME2	1.489784	6.86E-51
GLCE	1.489884	3.41E-51
SS18L2	1.490061	6.61E-52
COPA	1.490064	5.8E-51
HTRA2	1.490211	2.68E-49
ARL8B	1.490595	3.67E-51
LRRC25	1.490793	3.1E-42
ZMYND8	1.4912	1.06E-48
AVL9	1.491516	1.98E-52
NCOA6	1.491544	3.51E-53
OCIAD1	1.491572	1.05E-52
WTIP	1.491723	7.3E-41
YTHDF1	1.491891	3.26E-53
RPP30	1.492098	1.42E-51
SLC12A5	1.492172	1.35E-40
MRPL9	1.492426	3.72E-53
LOXL3	1.492478	7.03E-45
CTSV	1.492769	4.36E-37
USP39	1.492951	1.9E-53
GLRX3	1.493164	4.38E-51

SYS1	1.493376	1.43E-52
RAB18	1.493483	2.76E-49
HMGCS2	1.493535	1.57E-11
TMEM219	1.493604	2.01E-52
THYN1	1.493839	4.35E-50
GCLM	1.493984	1.54E-46
PRF1	1.493985	5.63E-50
SMG6	1.494008	4.68E-49
LSM12	1.494383	5.49E-54
NEURL1B	1.494854	1.74E-41
HSPB11	1.494977	7E-52
GLI2	1.495108	2E-47
AC010132.	1.495135	2.08E-53
DENR	1.495158	5.95E-52
NDUFS1	1.49524	2.27E-50
POT1	1.495348	3.69E-52
PDZRN3	1.495367	9.49E-44
B3GNT6	1.495452	5.24E-50
PDRG1	1.495627	1.13E-53
PRDX3	1.495756	5.19E-52
FBXO7	1.495786	2.03E-50
NPDC1	1.495879	2.76E-31
RBM7	1.496024	3.16E-47
HNRNPK	1.496136	3.63E-50
RBM18	1.496243	3.9E-53
TMA7	1.496375	3.67E-52
GTF2F2	1.496427	9.36E-53
SUPT16H	1.496461	5.86E-52
C4A	1.496517	6.35E-17
NCALD	1.496728	2.87E-44
G6PC3	1.496845	1.45E-51
TSPAN4	1.497004	3.88E-43
CHPT1	1.497108	2.72E-45
AD000671.	1.49721	1.94E-51
RASSF5	1.497453	9.92E-41
GGH	1.497474	8.18E-48
TFE3	1.49778	2.15E-47
ISOC2	1.498124	5.48E-51
CXorf38	1.498564	1.31E-50
ADI1	1.49908	1.43E-50
TRIB2	1.499121	1.81E-29
SIPA1L1	1.499523	1.31E-49
FBXL7	1.499526	6.1E-45
UBA2	1.499611	1.64E-50
SLC2A4RG	1.499628	7.02E-47
MB21D2	1.499726	3.45E-52
CUL3	1.499846	9.38E-54
GADD45GI	1.499925	2.73E-51
ZNF846	1.499967	5.88E-50
FDPS	1.500112	2.15E-50
KLHL23	1.500335	9.97E-50
FAM177A1	1.500533	3.06E-49
POGK	1.500851	3.63E-52
NIPSNAP1	1.500997	2.29E-51
SF3B3	1.501034	2.56E-50
GLRX5	1.501235	4.31E-53
ATP6V1B2	1.501528	3.65E-48
TPR	1.50157	7.92E-51
AGMO	1.50191	5.87E-53

BDKRB2	1.502023	7.37E-44
ATRAID	1.502165	4.81E-55
TMEM199	1.502404	1.57E-51
LPXN	1.502644	3.96E-44
KIF13A	1.502711	3.5E-51
UBE2Q2	1.502779	1.03E-48
BDH1	1.503063	2.26E-46
SLC29A3	1.503133	8.44E-51
YIPF1	1.503506	4.6E-52
PINK1	1.503775	8.29E-52
MS4A1	1.503902	2.34E-22
RMC1	1.503902	1.86E-50
TMEM200I	1.50417	3.08E-45
PRUNE2	1.50419	1.01E-36
RFXANK	1.504242	1.48E-49
RIC8A	1.504467	3.38E-50
USP32	1.504609	4.98E-52
RAB5B	1.504749	1.8E-50
DYNLL2	1.504806	5.24E-53
GINS2	1.505132	2.19E-55
CPQ	1.505242	2.7E-46
GIMAP1-G	1.505298	7.4E-45
GAP43	1.505602	4.13E-47
UBB	1.505811	5.12E-53
ADAMTSL5	1.506044	1.59E-45
PDGFRA	1.506295	3.62E-30
RSRC1	1.506566	2.99E-52
NECTIN3	1.506899	6.94E-45
NDUFA11	1.50695	1.78E-51
GCH1	1.50701	1.52E-45
PTK2	1.507161	7.37E-51
CENPX	1.507167	1.09E-50
DUSP18	1.507277	1.02E-52
FGD6	1.50762	2.45E-44
APOD	1.508172	1.88E-22
TLR4	1.508272	9.05E-46
TSC22D4	1.508313	4.92E-46
DCAF7	1.508602	1.3E-50
TNFAIP8	1.508609	4.21E-36
CCDC47	1.508669	1.29E-49
SNN	1.508937	2.91E-46
SELENOT	1.508965	2.82E-51
SDHA	1.509131	1.13E-53
MAGEF1	1.509686	1.95E-52
TRADD	1.510055	1.07E-45
RAB12	1.510561	2.53E-48
CREM	1.510596	8.62E-46
ANO10	1.510703	8.56E-52
GJA4	1.510754	1.92E-42
TTLL1	1.510767	9.41E-53
NDUFB4	1.510936	5.75E-51
CUL1	1.51125	6.67E-52
SCAMP3	1.511355	7.51E-54
ATAD2	1.511374	1.48E-52
KIAA1217	1.511716	1.5E-41
TP53BP2	1.511756	6.01E-49
LMBR1	1.511794	2.36E-52
RBM38	1.511881	9.09E-42
PALB2	1.511899	1.83E-54

GALNT18	1.511918	4.14E-45
ATP5F1B	1.511972	8.36E-54
BCAS2	1.512408	4.42E-51
PAQR7	1.513366	3.14E-50
COX20	1.513458	1.67E-49
OSM	1.513468	1.75E-44
AP1S2	1.513524	5.28E-44
PKP1	1.513564	7.36E-43
GUCD1	1.513671	4.79E-51
TPK1	1.513678	1.22E-50
LZIC	1.514117	1.22E-51
EXT2	1.51432	2.13E-51
C1orf216	1.51473	1.41E-49
NRXN2	1.51485	8.42E-33
KCNE4	1.515019	4.56E-27
RHOJ	1.515067	1.95E-43
ACOT2	1.515201	2.01E-50
S1PR3	1.515319	6.55E-41
STK32C	1.515341	1.35E-49
SLC9A3	1.515671	2.54E-30
EEF1A2	1.515771	8.83E-20
HSD17B11	1.515918	1.66E-41
P2RY2	1.516111	2.2E-45
ARSI	1.516193	5.12E-50
KIAA1671	1.51622	1.8E-48
TSSC4	1.516412	2.02E-49
MACF1	1.516551	7.69E-48
CD2BP2	1.517336	1.19E-52
SLC38A6	1.51765	6.01E-49
NR1H2	1.517928	3.6E-50
DCAF13	1.517961	2.15E-52
TNKS1BP1	1.517974	4.47E-42
ANK2	1.518243	1.15E-43
ABCC1	1.519166	7.87E-48
UBE2E3	1.519677	1.22E-49
IARS2	1.51997	3.35E-52
MRTFA	1.520229	1.86E-47
CHCHD10	1.520507	2.27E-49
ALOX5	1.520576	6.08E-32
ACYP1	1.520593	2.41E-45
UQCRQ	1.520939	3.75E-55
MRPS18C	1.521086	3.95E-54
PLOD2	1.52116	2.61E-33
HECA	1.52139	3.9E-49
PSMA1	1.521694	7.31E-51
RAB38	1.521853	3.52E-48
EMC7	1.521933	1.02E-52
RFC2	1.522008	1.75E-50
MED1	1.522377	6.98E-53
JAG1	1.5225	2.57E-43
FCHSD2	1.522526	4.07E-47
WDR72	1.522682	1.64E-24
FBXO28	1.522687	1.2E-53
ESCO1	1.523293	6E-52
SORBS3	1.523756	1.14E-45
ITM2A	1.523898	3.47E-30
VAMP7	1.524202	7.64E-50
SERTAD2	1.52431	9.91E-48
ZCCHC17	1.524798	2.54E-52

AC005943.	1.524837	3.6E-21
HTR1D	1.525362	4.49E-57
FLVCR2	1.525584	1.39E-47
SHB	1.525598	5.98E-45
LSM6	1.525677	8.49E-52
PDCL	1.525786	3.84E-52
RASA1	1.525946	2.18E-48
AKAP1	1.526155	5.31E-51
SCAF1	1.526322	2.66E-51
TBL1XR1	1.526497	2.83E-49
SNAPC1	1.526775	1.1E-52
NOS3	1.526958	1.43E-39
PIN4	1.52722	5.36E-54
SLC35A5	1.527267	1.12E-50
TRIOBP	1.527549	1.01E-46
BAZ1B	1.527921	1.66E-51
AKT3	1.52857	2.16E-41
MAP4K1	1.528958	1.49E-43
CLUAP1	1.528967	1.63E-52
DAXX	1.529149	2.01E-50
LYPLA1	1.529241	7.28E-48
SEMA4B	1.529254	8.26E-37
SLC9A6	1.52934	4.94E-53
GPR107	1.529697	3.31E-51
GOLPH3	1.529816	8.51E-51
BAZ1A	1.529911	2.6E-45
ZCCHC10	1.530004	1.17E-52
AAMP	1.530012	2E-53
CDH1	1.530309	2.77E-42
YY1	1.531247	1.22E-50
SAR1A	1.531306	8.7E-53
MRPL44	1.531396	2.47E-53
WNT4	1.531834	8.89E-34
TRIM11	1.531873	4.9E-51
ICOSLG	1.5321	8.81E-51
EAPP	1.532224	1.88E-52
TAF5L	1.532716	1.13E-53
ZBTB38	1.533316	2.69E-44
CLDN7	1.533576	2.86E-37
TTLL5	1.533752	8.28E-53
ACKR1	1.533804	2.99E-18
CHGB	1.533846	6.52E-11
TRAF7	1.533872	4.5E-49
ARL3	1.53393	3.94E-53
ACLY	1.534097	1.04E-49
TAGAP	1.534119	1.24E-46
EPB41L2	1.534435	1.58E-42
ADAMTSL4	1.534562	1.25E-33
TST	1.534719	1.44E-37
GEMIN6	1.534982	3.46E-54
HSD17B10	1.535082	4.01E-53
NCOA4	1.535249	2.54E-50
SOX9	1.535632	6.62E-39
PSPC1	1.535643	2.69E-51
MANSC1	1.535699	1.51E-46
UMPS	1.536025	3.03E-54
TM9SF4	1.53665	4.71E-52
B9D2	1.536864	1.27E-51
RASL11B	1.536929	8.85E-44

SLK	1.537058	4.75E-47
PINLYP	1.537144	1.35E-44
LPIN2	1.537436	7.6E-44
TMEM9B	1.537456	2.1E-53
SP100	1.537574	1.67E-40
KHDRBS3	1.537743	2.39E-45
ITGA4	1.53777	1.59E-43
GUSB	1.53808	7.35E-52
SLC35E4	1.538728	1.38E-55
NDUFC1	1.539423	2.51E-53
CCN3	1.539848	1.15E-42
PAQR4	1.539905	4.8E-44
EAF1	1.540444	4E-53
RAB42	1.54046	9.1E-49
FAM47E-S	1.540496	1.11E-49
METTL26	1.540845	1.85E-49
CSE1L	1.540935	6.75E-54
RCN2	1.541075	2.29E-49
ARF1	1.541087	5.25E-53
PPHLN1	1.541386	2.13E-51
TMEM9	1.541482	6.59E-54
CHST1	1.541494	2.55E-46
HACD2	1.541508	2.09E-51
CLEC10A	1.54173	3.9E-36
HAS2	1.541768	1.58E-51
LYSMD2	1.541847	1.71E-49
GPAT3	1.542101	1.45E-51
SLAMF6	1.542689	6.5E-46
CDC42SE2	1.542795	1.3E-50
CFLAR	1.542893	7.56E-43
PIGK	1.543065	2.25E-52
MFSD10	1.54308	1.56E-37
SUCLG2	1.543332	5.61E-50
ZFAND3	1.543366	1.4E-49
SP140	1.543424	2.96E-45
F8A1	1.543426	1.76E-42
TCF20	1.543739	4.24E-52
ARL6IP6	1.543886	3.54E-48
CAPN1	1.543917	4.1E-46
SH3GL1	1.544074	6.52E-50
ZDHHC13	1.544081	2.69E-52
DTNBP1	1.544367	3.36E-51
PDP1	1.544483	2.02E-45
HOXC6	1.544759	5.68E-50
THOC6	1.544831	1.51E-49
GALM	1.544851	8.97E-45
TCF19	1.544987	1.17E-52
PSMB1	1.545273	1.22E-52
SNAPC2	1.545273	3.63E-50
MIDN	1.545294	3.95E-38
REEP4	1.545316	4.94E-48
IWS1	1.545908	9.59E-53
PPP1R9B	1.546159	6.76E-49
UGT8	1.546367	1.52E-44
FST	1.546685	2.07E-40
CPSF2	1.54684	1.06E-48
KLHDC7B	1.546954	6.43E-49
SMIM12	1.547023	2.98E-51
RSPH1	1.547346	9.61E-48

C11orf53	1.547668	1.25E-44
FOXP1	1.547876	2.6E-49
RNF6	1.547879	5.53E-52
OSBPL8	1.547935	2.06E-46
NFKB2	1.548149	3.16E-39
RHEX	1.548248	9.54E-32
COX7A2L	1.548259	1.08E-52
CYB5R4	1.548345	1.18E-48
BAIAP2L1	1.54851	2.23E-36
VTA1	1.548615	2.75E-51
DEPDC1B	1.548694	8.26E-55
RAD23A	1.548711	1.02E-52
PCYOX1	1.549058	8.72E-51
MRPS12	1.549433	2.8E-51
PLN	1.549463	7.15E-35
AC005041.	1.549737	4.09E-53
B3GNT7	1.549789	1.02E-33
CKLF-CMT	1.549923	4.82E-53
ACTR10	1.550092	3.91E-49
LRP11	1.550607	6.75E-53
VEGFB	1.550817	1.24E-47
IFIT5	1.550835	1.1E-49
ADAT1	1.550969	2.74E-52
GNA12	1.550984	1.54E-47
SIM2	1.551078	2.32E-55
USP14	1.551526	3.2E-51
ELAVL1	1.551673	7.84E-52
ABHD3	1.551757	5.18E-48
DHX58	1.551838	1.17E-44
CRIPT	1.552519	1.12E-53
NAV1	1.552563	1.57E-45
JOSD1	1.552592	3.62E-48
APBB2	1.554432	1.65E-46
KNOP1	1.554458	1.83E-50
PIK3R2	1.555031	1.06E-50
CCR1	1.555603	2.5E-43
CIART	1.555844	1.78E-44
TMEM127	1.556007	8.79E-51
PATL1	1.556159	8.64E-48
TMEM208	1.556391	1E-53
TMCC1	1.557513	9.29E-51
VPS25	1.557535	8.02E-53
SHOC2	1.558017	2.44E-52
RSF1	1.558316	7.4E-50
SYTL3	1.558489	8.32E-49
EGR1	1.558494	2.71E-17
C4B	1.558558	8.79E-18
ULBP2	1.55868	4.84E-53
EFL1	1.558763	8.91E-53
LATS2	1.559204	7.42E-44
CGAS	1.559813	2.62E-50
MAP3K11	1.56029	1.04E-50
BARX2	1.560396	1.58E-36
PARP8	1.560461	2.65E-47
ARRDC3	1.560468	3.45E-33
SLC2A6	1.560944	5.97E-43
DUSP1	1.560961	4.86E-25
ZNF438	1.560964	3.59E-52
ADAR	1.561558	5.1E-49

LMOD1	1.561692	1.81E-33
CD163	1.561871	3.9E-21
POP4	1.561976	2.22E-53
C3orf38	1.562064	4.98E-53
RAB22A	1.562273	8.49E-54
NRGN	1.56251	6.45E-47
CHEK2	1.56259	1.99E-48
MFSD5	1.562615	1.43E-53
SPAG16	1.562976	3.79E-51
TRANK1	1.563004	5.61E-47
LRP8	1.563212	6.21E-52
TMEM273	1.563562	1.23E-38
EIF2B2	1.563599	1.1E-51
SEMA4C	1.563732	1.69E-43
HSPBP1	1.564007	1.12E-49
TMEM154	1.564172	3.56E-51
CHD8	1.564374	1.84E-52
MNAT1	1.564619	2.11E-52
ZFP64	1.564801	2.63E-54
TP53	1.564806	1.78E-45
MED15	1.564809	4.97E-47
C9orf16	1.56498	1.41E-41
DTX3L	1.56513	4.2E-47
NFE2L3	1.565237	3.91E-43
ZNF146	1.56544	5.98E-50
FEM1B	1.565915	3.1E-51
PTMA	1.565931	2.76E-45
ERGIC3	1.566166	8.61E-55
CDC73	1.566259	2.78E-52
DYNC1I2	1.566299	4.42E-50
NUAK1	1.566447	1.21E-46
IL15RA	1.567021	2.11E-37
MIER1	1.567061	2.38E-50
PODXL2	1.56715	2.5E-40
VEZT	1.567324	1.15E-50
AAR2	1.567343	2.96E-53
BRCC3	1.567487	4.54E-54
SLC25A11	1.567496	7.23E-52
COX7C	1.567906	2.33E-54
CKB	1.56823	1.91E-39
ISG20L2	1.568393	4.42E-53
MCMBP	1.568594	7.34E-52
AC005833.	1.5693	8.3E-43
ATP10B	1.569527	7.75E-24
GEMIN7	1.569528	4.38E-52
TCIM	1.569901	4.92E-30
UQCRC1	1.570476	1.73E-54
CHMP2B	1.570512	2.85E-48
TIMM50	1.570575	3.88E-53
MREG	1.570771	3.8E-53
ARRB2	1.570956	1.98E-41
TMEM248	1.57173	1.72E-53
MTDH	1.571859	4.2E-45
MYO9B	1.571934	8.11E-46
ARAP3	1.572389	1.83E-45
TFRC	1.572424	4.08E-45
USP6NL	1.572743	4.63E-52
NDUFS3	1.573158	6.75E-54
SNUPN	1.573163	6.98E-53

SLC35A1	1.573182	1.29E-51
JAK3	1.573222	1.47E-43
KCTD20	1.573226	3.84E-49
SUSD6	1.573292	7.01E-48
LNPK	1.57369	9.29E-54
PBX4	1.573724	1.44E-46
ATP6V1A	1.573899	1.52E-51
NDN	1.574162	7.21E-42
LSM2	1.574191	2.38E-52
PSMC5	1.57421	2.74E-52
ALG8	1.574365	4.51E-54
APOLD1	1.574561	1.25E-38
TMEM185A	1.574676	1.45E-52
RAB27B	1.575004	2.14E-45
UNG	1.575012	3.54E-53
APOBEC3D	1.575338	5.87E-53
LYL1	1.576095	2.53E-45
SP3	1.576309	3.23E-49
CYTH4	1.576605	8.33E-42
MN1	1.576676	1.39E-49
CLDN12	1.57669	3.66E-47
CREBL2	1.576751	5.18E-50
NEK7	1.576839	1.06E-47
ATF1	1.577096	9.91E-50
ARHGDIA	1.577312	1.02E-50
PPP1R8	1.577439	9.9E-52
GSTK1	1.577593	1.27E-51
ADGRF4	1.5776	2.1E-52
COA4	1.577661	3.64E-54
AAGAB	1.577802	3.11E-53
TPMT	1.577886	1.01E-53
EMC4	1.577945	1.16E-54
ACOT13	1.578301	1.07E-52
RRAGA	1.578445	1.57E-50
GDE1	1.578581	1.79E-50
RNF7	1.57886	1.71E-52
CORIN	1.579171	9.84E-49
ASB1	1.579723	3.73E-51
SULT1E1	1.580026	4.82E-45
TMX4	1.58006	1.43E-50
CCNE1	1.580097	5.56E-54
CCDC6	1.580641	5.44E-51
ANKRD40C	1.580668	5.68E-45
DLAT	1.58081	1.41E-51
JAZF1	1.581016	2.05E-46
HNRNPH2	1.581127	1.08E-51
ORC6	1.581272	5.86E-54
NDUFB5	1.581278	2.33E-53
ASAP1	1.58137	1.39E-45
SLC7A4	1.581871	3.36E-55
TNIP2	1.581874	4.4E-49
TNFRSF14	1.581962	9.86E-40
HPGDS	1.58207	2.12E-47
LYPD6B	1.582274	2.76E-31
TOMM40L	1.582359	1.43E-51
SOCS6	1.582442	4.11E-50
UTP3	1.582474	3.63E-52
C1D	1.582983	6.3E-52
GNL2	1.583086	1.2E-52

DIAPH1	1.583281	1.51E-47
FABP5	1.583385	1.77E-39
BABAM2	1.583629	7.11E-52
ZNF217	1.583835	3.37E-43
ZNF394	1.583838	6.17E-53
STOML1	1.584014	5.22E-51
KRAS	1.584363	1.03E-48
CCDC28B	1.58458	1.01E-50
SNRNP40	1.584628	3.75E-53
EIF4G3	1.584922	4.5E-49
ITPRIPL2	1.585034	7.95E-45
C8orf76	1.585186	1.63E-53
PPP2R3C	1.585279	1.27E-49
CCT7	1.585986	2.68E-53
NCAPG2	1.586257	4.93E-52
PEX26	1.586403	1.93E-51
FNBP1	1.586413	2.44E-46
DNAJC7	1.586471	4.66E-54
ANG	1.586554	2.92E-40
TBC1D10C	1.586662	2.03E-40
SHC1	1.586696	4.14E-47
ACOT11	1.586721	2.26E-51
PACS1	1.586828	1.2E-45
MICOS13	1.586869	2.72E-52
MGAT5	1.586974	6.8E-51
ABTB2	1.587088	4.62E-45
ECHS1	1.587384	2.93E-54
CPNE5	1.587429	5.52E-49
MRGBP	1.587668	5.05E-54
CXCR3	1.587734	1.54E-50
PLEKHM1	1.588073	1.54E-51
LRIF1	1.588205	5.65E-51
AP3B1	1.588537	6.61E-52
ABLIM2	1.588555	4.49E-46
RRP9	1.588558	1.75E-53
SCT	1.588595	5.74E-47
GOLGA5	1.588687	1.71E-54
CHEK1	1.588729	3.03E-55
PHACTR2	1.589222	5.67E-48
AMBRA1	1.589359	2.11E-51
HS1BP3	1.589453	1.12E-50
MRPL49	1.589601	1.54E-52
ATP5PD	1.590018	1.25E-53
PTPRF	1.590293	1.08E-43
EPB41L3	1.59031	2.58E-38
VBP1	1.590416	7.39E-53
BTK	1.590498	5.16E-38
YIF1B	1.590858	1.02E-50
APOBEC3F	1.591091	1.13E-52
ADCY3	1.591271	6.65E-45
ONECUT3	1.591334	2.14E-50
TANC2	1.591381	1.61E-47
TBC1D7	1.591619	2.28E-53
SLC17A5	1.591722	7.52E-52
LUZP1	1.591848	2.21E-50
ARMT1	1.592735	2.06E-51
GPR137	1.59313	7.87E-48
PILRA	1.593206	1.42E-35
C15orf39	1.593267	1.13E-50

ABHD4	1.593364	5.93E-50
MRPS28	1.593552	4.66E-54
F10	1.593614	1.73E-35
EPHB4	1.593723	1.49E-45
ADGRB2	1.593757	1.95E-52
NCBP2	1.594091	7.21E-53
PLEKHF2	1.594555	3.7E-51
NAXE	1.595149	1.77E-54
CCK	1.595261	1.99E-30
SMAD3	1.595338	9.5E-42
AKIP1	1.595443	1.51E-50
CMC2	1.595614	3.81E-53
C11orf68	1.59571	1.36E-51
PHTF2	1.595999	4.22E-48
LAMTOR1	1.596062	1.05E-51
MZT1	1.596264	3.69E-54
SART1	1.596358	4.07E-52
DDX60L	1.596435	9.47E-49
BMP7	1.596496	2.69E-46
SHCBP1	1.596604	1.72E-52
PEX19	1.596722	2.63E-52
PIN1	1.596761	2.16E-50
AKIRIN2	1.596781	3.17E-50
DDB1	1.596814	4.28E-53
NDUFS8	1.596848	3.72E-53
HIKESHI	1.596922	2.55E-53
SGCE	1.597291	2.71E-44
MTLN	1.597358	2.81E-44
C16orf54	1.597366	1.52E-51
MLF2	1.597694	1.21E-51
DEK	1.59791	1.35E-49
NAP1L4	1.597923	3.13E-51
PHF14	1.597948	1E-53
ZNF365	1.598055	3.42E-52
MRPS21	1.59836	5.99E-51
CCR7	1.598763	1.55E-43
PIP4K2C	1.598952	3.14E-52
CENPB	1.599131	2.54E-52
RECQL4	1.599182	4.49E-46
DRAM2	1.599185	7.34E-50
NENF	1.599998	4.38E-53
SMARCAL1	1.600408	7.76E-54
DCBLD1	1.600441	1.2E-47
COPS7A	1.60074	1.87E-51
P2RY6	1.600839	8.04E-43
CAPS	1.601725	3.33E-36
ATIC	1.601743	1.22E-51
MFHAS1	1.60188	1.11E-49
PPP2CA	1.601945	1.66E-51
VPS26A	1.60198	1.53E-51
BIRC7	1.602165	1.68E-51
SMARCB1	1.602463	6.92E-51
CD8A	1.602615	6.29E-39
FGF1	1.602622	6.39E-51
PRMT1	1.602927	2.24E-49
PTRH2	1.603048	7.21E-54
TERF2IP	1.603069	2.29E-49
DPEP2	1.603426	1.4E-44
CERS5	1.603493	2.62E-49

SPRY1	1.603509	5.41E-39
NDUFA6	1.603756	3.92E-54
FCF1	1.603778	3.98E-47
RAB21	1.603921	2.32E-49
ZNF576	1.603935	4.15E-54
SPNS1	1.604311	7.92E-51
HABP4	1.604473	5.06E-50
GNG11	1.60509	5.09E-44
TRIM26	1.605097	6.15E-52
CATSPER1	1.605414	1.29E-52
IL2RB	1.605568	2.56E-50
CDC26	1.605744	1.16E-52
SHKBP1	1.605757	4.87E-48
EXOSC7	1.605958	1.8E-53
LGALS7B	1.606046	4.03E-17
KANK2	1.606383	6.91E-42
EXOC5	1.606466	3.01E-49
NDUFB1	1.606804	3.94E-52
CD34	1.607215	7.48E-42
TRAM2	1.607235	1.62E-46
NSMCE3	1.607449	6.38E-51
CPSF3	1.607475	5.96E-54
LAPTM4A	1.607503	2.44E-50
EFR3A	1.607762	2.66E-49
GALNS	1.608216	2.37E-46
SMAP2	1.608996	9.1E-45
GMDS	1.609142	2.05E-39
MRPS10	1.609192	1.37E-53
PGM2L1	1.609215	1.3E-47
GRAMD2B	1.609413	4.64E-50
GZMH	1.609914	1.58E-49
RNF39	1.610003	3.15E-39
ENY2	1.610387	2.92E-52
SPARCL1	1.611214	2.72E-33
HSPA6	1.611412	3.73E-36
HNRNPA0	1.611412	2.36E-51
STON2	1.611491	3.37E-53
TCTEX1D2	1.611801	2.05E-50
SUSD3	1.611847	3.34E-47
DTX4	1.611975	8.01E-45
DUSP23	1.612022	8.3E-34
NUP62	1.612585	1.06E-51
MFSD4A	1.612843	1.58E-43
NDUFA4	1.612957	3.19E-52
CAVIN3	1.612965	6.9E-38
SLC35B2	1.613163	8.09E-54
LSM10	1.613267	4.41E-52
ETV6	1.613287	1.66E-50
ANO6	1.613465	5.34E-46
RAE1	1.613572	3.4E-53
THAP4	1.613745	2.33E-54
NDUFA2	1.613982	3.64E-54
JOSD2	1.614078	1.57E-44
ITGA6	1.614658	1.44E-39
MYH10	1.61497	4.69E-43
PRPF31	1.615197	2.94E-52
MRGPRF	1.615539	7.21E-42
DERA	1.615663	7.41E-52
ILF2	1.61595	5.12E-53

DPYD	1.616062	7.92E-42
RAB29	1.616295	3.55E-49
FAM50A	1.616336	5.35E-49
KIFAP3	1.616416	6.72E-52
PDZD3	1.616488	5.13E-28
RNASEL	1.616522	2.95E-51
DUSP11	1.616746	8.49E-52
C11orf58	1.617203	5.66E-51
CD96	1.617464	2.23E-47
RNF5	1.617853	4.07E-53
PYGO2	1.617935	3.38E-52
UBE2D1	1.618667	3.75E-49
TRIM59	1.61868	1.09E-53
KBTBD2	1.620588	8.93E-51
DHX9	1.620681	5.29E-53
RARB	1.620799	1.06E-49
GTSE1	1.620819	4.42E-53
PLA2G4A	1.620946	5.22E-46
TMEM62	1.621129	2.14E-51
HS2ST1	1.621436	2.2E-52
DNAJA2	1.621678	1.87E-52
TRIP12	1.621752	7E-50
SNX20	1.622102	1.35E-48
HNRNPL	1.622318	2.32E-53
UVRAG	1.62234	8.78E-51
MRPL52	1.622476	5.12E-53
CCDC71	1.623096	1.18E-52
ARL2	1.623118	9.91E-51
ST6GALNA	1.623284	7.27E-52
RCOR1	1.623359	1.39E-51
PPP2R2C	1.623457	9.31E-45
IGSF3	1.62358	5.62E-46
MAZ	1.623743	3.39E-51
KDM5B	1.624336	5.93E-50
VAC14	1.624499	1.38E-51
FLRT3	1.62459	5.22E-42
SAMD11	1.624728	1.28E-39
PRICKLE1	1.625048	1.04E-42
GOT2	1.625247	6.92E-54
MRPL18	1.625249	6.43E-53
PAIP1	1.625279	1.3E-52
ATAD1	1.625289	5.06E-54
HDGFL2	1.625397	1.85E-50
BCAR1	1.625873	6.71E-46
SNRPA	1.625944	4.86E-51
WDFY1	1.626038	2.72E-49
RAB13	1.626359	2.04E-51
MACC1	1.626385	1.14E-40
SAP30	1.626667	2.49E-49
OMD	1.626701	1.63E-37
ADAMTS7	1.626758	7.87E-50
STAP2	1.626869	2.43E-42
MRFAP1L1	1.62706	1.05E-51
ALG1L	1.627795	1.51E-36
NME1-NM	1.627858	5.58E-47
C9orf64	1.627861	1.36E-52
NDUFAF3	1.627979	4.67E-51
PDE6D	1.628245	3.67E-51
LYAR	1.62825	1.28E-53

PERM1	1.628293	6.59E-44
STAU1	1.628407	1.79E-52
DNAJC25-	1.628484	4.27E-51
RECQL	1.628747	7.6E-45
MRPL27	1.628789	5.19E-54
SQSTM1	1.629066	1.05E-49
INPP5F	1.629151	9.66E-51
SYNGR2	1.629339	1.98E-50
ADAMTS4	1.629379	6.47E-32
ZFYVE21	1.629821	3.66E-50
GAR1	1.630208	1.31E-54
SMARCA4	1.630528	3.17E-50
LYPD1	1.631848	3.2E-29
NDUFA4L2	1.631873	1.88E-41
C9orf152	1.63189	1.65E-30
IL20RB	1.632402	1.11E-47
BBX	1.632459	4.16E-49
NAP1L1	1.632574	1.43E-49
ACTG1	1.632589	1.12E-43
RASSF3	1.632669	8.19E-45
NPTX1	1.632791	1.73E-48
GSK3A	1.632993	7.57E-53
WDFY4	1.633299	1.61E-46
IL33	1.633611	7.92E-37
KLRB1	1.633697	2.21E-50
RAB5A	1.633761	1.25E-51
POLD4	1.634691	2.47E-42
POLR2J	1.634892	2.53E-53
COMMD9	1.635142	6.46E-52
RRM1	1.635601	2.77E-54
ANKRD29	1.635886	2.76E-47
NUTF2	1.636019	2.32E-51
TSR2	1.636245	1.37E-53
CBLC	1.636423	4.29E-35
USP10	1.636832	1.13E-52
MEA1	1.636878	6.75E-54
CNN3	1.637241	5.64E-39
MYO1G	1.637492	2.72E-45
ITPA	1.637581	7.35E-50
TLE3	1.637814	2.72E-45
LPP	1.637952	3.46E-45
VPS37B	1.637969	6.35E-49
MAN1C1	1.638355	2.13E-42
OGN	1.638505	4.71E-26
ITIH5	1.638515	2.24E-34
GRPEL1	1.63857	1.21E-54
SFR1	1.639025	1.17E-53
MAP1LC3A	1.639504	3E-45
CBLB	1.639524	7.79E-49
TNFRSF4	1.639921	2.62E-47
RPLP1	1.639927	2.79E-50
ASPM	1.639976	6.79E-55
CLSTN3	1.639993	1.41E-43
FKBP1B	1.640093	2.44E-44
FOXJ1	1.640247	1.95E-41
APOL4	1.640257	5.42E-42
C1orf198	1.640258	8E-48
SLC43A3	1.640748	2.42E-39
CALM1	1.640833	2.95E-50

NFATC1	1.641036	1.16E-44
MPC2	1.641134	1.4E-52
NTAN1	1.641146	1.16E-47
EEPD1	1.641173	1.66E-42
TSFM	1.64132	1.35E-54
FZD1	1.641485	5.55E-49
ACAT2	1.641604	6.61E-46
TNFRSF18	1.641643	1.41E-50
PTS	1.641861	1.37E-51
OTUB1	1.641974	8.08E-53
NYNRIN	1.64205	1.48E-47
HOXB9	1.642128	4.17E-53
NDUFA9	1.642396	1.3E-53
MRPL4	1.642762	5.86E-52
EFNA2	1.642832	3.26E-56
FKBP3	1.642926	1.28E-51
MSANTD3	1.642948	9.58E-52
KRT5	1.643005	1.03E-09
ERAP1	1.64301	1.62E-48
SLC10A3	1.643065	2.5E-50
TRIP13	1.643108	4.77E-52
LAMTOR2	1.643176	5.16E-53
TRMT10C	1.643567	4.47E-54
RAPH1	1.643681	2.82E-48
CLEC14A	1.643914	5.75E-47
DGAT1	1.643932	1.18E-48
CHURC1	1.643997	1.21E-52
SGCD	1.644354	6.98E-52
CES2	1.644393	6.17E-50
MCTP1	1.6445	6.78E-45
SDHD	1.644601	1.01E-51
CARD10	1.644653	1.69E-46
PSMC3	1.644672	1.7E-53
MYO1F	1.645017	4.38E-36
SPTLC1	1.645493	3.14E-50
CRYBG1	1.645603	8.45E-42
SFXN1	1.645798	3.57E-52
PLCB4	1.64593	7.08E-50
MEF2C	1.646023	2.03E-43
ANAPC11	1.646399	2.1E-53
BEX3	1.646504	3.22E-50
RNF26	1.646634	3.54E-53
GJC2	1.64691	7.65E-53
VKORC1L1	1.646991	4.97E-54
PCSK5	1.647065	9.32E-43
FCN1	1.647501	1.39E-28
NFKB1	1.647668	8.9E-49
UBTD2	1.647747	4.59E-51
BAD	1.647854	1.2E-50
MED28	1.648048	8.63E-52
TMEM38A	1.648123	2.48E-52
PSMC1	1.648505	3.25E-52
AZIN2	1.648525	1.11E-48
EIF2AK1	1.648662	1.97E-54
PDE4D	1.648832	6.11E-51
PPP2R5E	1.648859	5.95E-52
THSD4	1.648953	2.96E-41
CCDC92	1.649883	9.25E-49
ANKRD50	1.649897	4.25E-48

CHD4	1.649908	2.08E-52
NUDT5	1.649969	5.12E-53
ARHGAP4	1.650037	1.07E-34
IFI27L1	1.650261	4.42E-51
CPM	1.650275	1.46E-37
ANOS1	1.650419	1.11E-53
S1PR4	1.650443	7.85E-49
RFX2	1.650907	1.46E-49
NIPA2	1.650918	2.52E-52
PPM1G	1.651158	3.6E-53
TP53RK	1.651222	8.73E-56
SLFN13	1.651263	2.11E-42
CST3	1.651424	2.78E-52
ARHGAP9	1.651872	2.09E-34
TEP1	1.652302	5.47E-48
ATG7	1.652364	5.29E-53
SERTAD4	1.652543	2.61E-54
DDX41	1.652685	4.56E-51
ZDHHC7	1.652763	7.3E-46
POLR2F	1.652855	8.7E-53
CUX1	1.653053	1.77E-53
MEDAG	1.653177	1.75E-26
NR2F2	1.653563	2.26E-46
PLD3	1.653808	3.47E-49
MEX3D	1.654294	1.9E-50
TSNAX	1.654451	1.51E-53
VRK1	1.654558	6.71E-52
TLCD1	1.654726	5.31E-51
TOMM22	1.655018	8.54E-55
HINT1	1.655279	2.39E-53
RIN1	1.65528	3.98E-47
KLF7	1.655312	8.94E-46
RETN	1.655666	1.24E-33
MED21	1.655749	1.18E-52
SLC6A20	1.655933	7.67E-31
RAB14	1.655956	2.22E-52
BLOC1S2	1.656057	9.51E-52
EYA2	1.656062	2.68E-40
SSBP3	1.656091	4.45E-51
SCNN1A	1.656479	9.39E-29
SKI	1.656556	5.22E-48
SPSB2	1.656665	3.36E-49
EPDR1	1.656721	6.34E-43
ENOPH1	1.656997	1.6E-53
HOXC4	1.657193	4.82E-53
CCDC167	1.65748	1.29E-51
EIF2AK2	1.657681	6.08E-51
SLC20A1	1.65787	5.65E-36
SYK	1.657943	1.18E-43
VCP	1.658117	1.37E-50
RILPL2	1.658621	9.03E-53
CLMP	1.658624	5.28E-36
CWC27	1.658726	1.5E-54
CC2D2A	1.658946	1.65E-46
BUB3	1.660175	1.3E-52
TMEM87B	1.660305	2.68E-50
PPP3R1	1.660341	6.3E-52
HAUS8	1.660597	6.09E-52
DTL	1.66069	1.04E-55

SNRPE	1.660766	2.82E-53
AP003108.	1.661013	7.17E-52
LAMTOR3	1.661223	1.54E-51
PIR	1.661475	1.32E-50
DDX39A	1.661672	1.91E-48
STARD3NL	1.661745	1.49E-50
MBOAT7	1.66189	1.02E-47
IL3RA	1.662083	4.03E-49
ITGAL	1.662391	6.2E-39
FUT8	1.662637	4.98E-48
DTD1	1.66264	6.27E-54
GSR	1.663139	5.88E-53
DIPK1A	1.663201	3.75E-51
C11orf24	1.663559	1.02E-52
LACTB	1.664358	1.13E-49
ATP10A	1.664393	4.4E-51
AAMDC	1.664554	8.35E-53
PYM1	1.664989	4.5E-55
SLAIN2	1.665006	4.47E-48
ADAM17	1.665119	4.82E-47
NFKBIB	1.665389	5.72E-52
RFK	1.666359	5.61E-51
CA13	1.6665	1.7E-52
HSPE1	1.666628	6.69E-48
PKN1	1.666657	4.42E-50
AP1M1	1.666894	9.53E-50
UBTD1	1.667722	8.62E-49
RGS12	1.667744	2.56E-45
MDH1	1.668064	1.25E-52
LYPD2	1.66883	7.05E-32
SRA1	1.668915	5.31E-54
TCF3	1.668986	2.13E-49
PRPF6	1.669006	1.62E-54
STK39	1.669027	6.16E-49
PSTPIP1	1.669076	2.45E-41
SP140L	1.669254	8.82E-43
MRPS36	1.669271	2.04E-54
TMEM141	1.669675	1.13E-48
SCGB2A1	1.66997	8.37E-26
BIN2	1.670027	1.36E-43
DSC2	1.670127	4.4E-47
STEAP2	1.670153	2.39E-47
IAH1	1.670483	2.03E-51
DDAH1	1.670493	9.14E-51
TIMM17A	1.670786	1.12E-53
SGIP1	1.670981	3.32E-47
CDH5	1.671471	2.3E-45
ZCCHC24	1.671534	2.22E-40
CLDN1	1.671712	1.37E-32
WSB2	1.672752	3.63E-52
CDS2	1.672903	9.43E-52
SPRR1A	1.673047	1.11E-12
CSF2RB	1.673341	1.45E-43
FILIP1L	1.673458	4.09E-38
CHPF2	1.673468	8.7E-52
FRMD4B	1.673524	1.78E-46
BCAP29	1.674408	8.22E-53
PFDN6	1.674925	1.35E-53
COX14	1.67516	9.21E-53

RIN2	1.675284	3.76E-49
EEF1AKNM	1.675334	8.02E-54
DENND1A	1.675695	1.04E-52
DPH3	1.675842	3.32E-53
CIDEC	1.675983	4.86E-25
TOR1A	1.676364	1.82E-53
WNT5B	1.676372	1.27E-47
SEC23IP	1.6766	2.73E-51
PSMD7	1.676965	8.77E-53
BLZF1	1.677111	2.47E-53
RASA3	1.677139	7.24E-46
MED29	1.677203	2.01E-54
NRM	1.677635	1.6E-49
SCAMP2	1.677738	5.74E-53
POLB	1.677779	7.09E-53
CREG2	1.67809	1.45E-55
CERKL	1.678603	3.88E-50
IL1R1	1.67873	1.54E-35
TAOK3	1.679072	2.4E-50
NAGS	1.679367	7.63E-52
BMP2	1.67985	3.13E-47
SESN3	1.679959	4.06E-46
SCAMP1	1.679977	1.64E-51
SLC35A2	1.6802	1.02E-53
ARHGAP42	1.680337	3.56E-50
ATP5ME	1.680401	4.73E-55
NRP1	1.680501	8.14E-45
GRAP	1.680864	3.53E-51
CLIC2	1.680991	3.53E-44
SPINDOC	1.681098	2.64E-51
ITPKA	1.681302	7.11E-40
EPYC	1.681327	2.89E-49
YARS2	1.681546	3.82E-54
TMBIM6	1.681631	4.22E-54
NTMT1	1.681753	1.5E-53
TCAF1	1.681848	2.23E-52
UFD1	1.681944	9.16E-54
FLAD1	1.682241	1.55E-53
RAD51AP1	1.682486	6.48E-53
SULT1C2	1.682742	1.12E-15
NOP16	1.683222	2.39E-53
TTC39A	1.683242	4.27E-45
PNPLA6	1.683433	5.22E-48
NSMCE2	1.683682	4.18E-54
CYTH3	1.68372	3.06E-45
MAN1B1	1.684558	1.14E-51
HSD17B6	1.684636	8.28E-51
ARPP19	1.685125	2.62E-51
PAICS	1.685226	9.21E-53
RASL12	1.685571	2.51E-46
JPT2	1.685742	2.81E-50
SNX7	1.685769	3.54E-50
CD2AP	1.685955	1.32E-46
BLOC1S4	1.686067	6.47E-54
FIBP	1.686347	2.61E-52
MIF	1.686476	4.74E-37
B3GNT8	1.686546	2.72E-51
RRAGC	1.686828	4.28E-51
PSMA2	1.687382	5.14E-54

TIMMDC1	1.687409	1.95E-53
GZMB	1.687636	3.83E-48
DCAF6	1.687893	1.33E-52
NID1	1.687901	1.49E-36
DOP1B	1.687902	1.93E-51
NIP7	1.688039	3.97E-52
MARCKS	1.688635	1.34E-49
CHRAC1	1.688731	4.14E-53
ACAP1	1.688768	1.28E-43
AL162231.	1.689105	1.9E-47
CORO1B	1.689517	1.06E-50
PSMD13	1.6904	1.25E-53
MRPS18A	1.690513	5.75E-56
SDHAF2	1.690667	5.1E-54
NME1	1.690725	6.25E-52
CH25H	1.690794	1.58E-44
CARMIL1	1.690798	2.91E-51
SOCS1	1.691039	2.78E-43
MOB3A	1.691105	4.98E-50
RNASEK	1.69124	1.24E-50
MTMR14	1.691764	3.82E-51
PCOLCE2	1.692107	2.11E-42
UBASH3B	1.692244	3.7E-48
PDE4B	1.69252	2.95E-48
CDK7	1.692554	1.96E-54
NUP37	1.692893	8.85E-54
TCEAL8	1.693004	5.54E-52
USB1	1.693638	2.46E-51
BCL6B	1.693974	6.17E-46
MAPK6	1.693986	4.13E-52
AP3M2	1.693993	6.59E-51
EDN1	1.694523	9.65E-40
EFHD2	1.694617	6.8E-45
SUPT4H1	1.695444	1.86E-53
GPD2	1.696085	8.21E-53
MAPK1IP1I	1.696153	2.13E-51
PSMD8	1.696167	3.95E-54
BCL2L15	1.696227	1.64E-35
PBK	1.696271	4.29E-56
BECN1	1.696409	2.77E-53
ADCK2	1.696617	2.6E-53
IL27RA	1.696645	6.6E-49
RNF25	1.696653	1.93E-53
PSMD4	1.696761	3.03E-53
FAM20B	1.696847	3.63E-54
NAMPT	1.697388	1.43E-31
FKBP8	1.697433	7.71E-52
RANBP1	1.69816	2.15E-53
PAMR1	1.698308	2.5E-42
SERINC1	1.698428	2.79E-47
MAGOHB	1.698685	8.86E-54
GALNT3	1.698738	8.44E-39
CCM2	1.699507	2.71E-51
CASP8	1.699558	2.94E-44
PIMREG	1.700093	3.22E-56
SVIL	1.700134	1.29E-44
VASH1	1.700321	1.18E-42
OSMR	1.700418	2.44E-38
ARHGAP5	1.70054	2.32E-48

TDO2	1.700624	3.26E-48
PPP1R1C	1.700849	2.81E-47
COX4I1	1.701016	6.6E-55
PLA1A	1.701104	5.04E-47
MAPKAPK2	1.701177	5.75E-50
PAF1	1.701613	3.69E-53
YWHAE	1.701763	1.95E-53
CD6	1.701779	2.08E-50
PPP2R5C	1.701806	3.01E-54
FOXF1	1.701903	8.92E-50
AKR1B1	1.701929	2.25E-46
CXCL6	1.701949	6.65E-22
ANKS4B	1.702574	7.11E-51
IFT22	1.702586	4.66E-54
TIGAR	1.702803	5.15E-53
ZBP1	1.702965	1.84E-49
PLEKHN1	1.703336	2.79E-39
CETN3	1.703377	5.57E-51
PTPRJ	1.703601	1.08E-51
RABGAP1L	1.703616	5.33E-53
SNU13	1.704704	7.7E-53
BUB1B	1.704935	3.45E-54
SIPA1L3	1.70497	6.66E-49
RPS6KA3	1.705107	4.06E-49
SLC46A3	1.705201	2.22E-48
DAPK3	1.705726	3.09E-50
CDC42BPA	1.705775	4.82E-52
SPTBN1	1.706035	2.93E-47
VAMP8	1.706068	4.82E-47
HDAC7	1.706123	3.07E-45
COG4	1.706485	1.66E-51
PLXNB2	1.706529	5.1E-48
MAD2L1BP	1.706751	2.75E-55
CKAP5	1.706767	2.13E-51
TRAPPC5	1.707049	3.69E-47
MMP15	1.70709	3.94E-46
SYNPO	1.707572	1.66E-42
FLOT1	1.707795	1.26E-49
EFCAB14	1.707831	1.18E-50
RYBP	1.708073	1.34E-51
RNF40	1.708102	9.7E-54
TRPM2	1.708272	8.53E-46
HES1	1.708304	4.16E-42
BSCL2	1.708454	2.2E-51
PEX11B	1.708632	6.17E-55
AIFM2	1.708771	2.5E-52
CBX1	1.709127	1.13E-50
CYP2C9	1.70921	4.21E-38
LRP10	1.709254	1.66E-45
RFLNB	1.709351	6.04E-48
NINJ1	1.70953	2.75E-50
MND1	1.70961	1.57E-56
SNAI2	1.709776	3.03E-33
MEAK7	1.709873	4.28E-50
CAPRIN1	1.710128	8.22E-53
ARHGEF16	1.710297	1.07E-42
CNOT8	1.71033	2.86E-51
ADH1C	1.710689	1.75E-22
ACTR1A	1.710777	6.83E-52

TSN	1.711079	6.92E-54
LLPH	1.711127	3.58E-54
RNF11	1.711798	5.43E-49
COLEC12	1.712549	4.71E-36
DIPK2A	1.712918	6.88E-50
FXYD6	1.713108	1.05E-39
PPP1R11	1.713175	4.42E-53
MICOS10-	1.713179	1.32E-49
PLAC9	1.713457	6.31E-44
CD99	1.713544	2.29E-48
LEO1	1.713854	7.63E-54
PDAP1	1.715694	3.64E-52
EPHX4	1.715877	1.54E-57
ZC3HAV1	1.715967	1.46E-52
CFDP1	1.716019	2.97E-52
UBAP1	1.716036	2.75E-50
MRAS	1.716159	3.49E-42
BIN1	1.716272	1.13E-48
SLIRP	1.716423	7.6E-55
CD200	1.71663	3.4E-43
MOB4	1.716689	4.6E-52
COMMD7	1.716948	3.85E-53
GK	1.717088	2.06E-51
ETV3	1.717111	8.2E-53
PICALM	1.717189	8.9E-49
GTF3C6	1.717244	3.3E-52
AGFG1	1.717436	2.29E-48
ACHE	1.717525	1.75E-33
SET	1.717721	6.35E-52
CHRNA5	1.717955	1.1E-48
TRAF3	1.718096	1.12E-50
TMEM45A	1.718134	3.01E-39
SEMA6B	1.7182	8.04E-46
SAMD4B	1.718201	2.88E-50
NME4	1.718422	6.16E-49
PRSS21	1.718447	3.16E-20
SWI5	1.71888	4.42E-53
RPA2	1.71895	2.86E-51
SLC27A3	1.719061	1.03E-47
MXRA7	1.719901	2.98E-43
GALNT6	1.720034	1.02E-41
GLB1	1.720054	7.1E-53
ADH5	1.720081	2.4E-51
RAP2A	1.720189	8.29E-51
AGRN	1.720342	1.68E-37
ABLIM1	1.720705	1.06E-47
C6orf47	1.720735	1.99E-54
COPS6	1.720772	9.75E-55
UBE2E1	1.721321	1.1E-49
MAML2	1.72138	1.49E-49
OSGIN2	1.721784	4.23E-53
RUSC1	1.721825	5.35E-50
METTL9	1.722731	2.5E-52
SERF2	1.722919	5.41E-52
DNAJB5	1.723732	7.71E-45
GMFB	1.724263	3.61E-51
ESAM	1.724429	7.94E-47
CFI	1.724701	7.77E-37
BATF3	1.724719	1.5E-49

ARMC6	1.724745	9.5E-53
KCTD1	1.724918	2.9E-50
NPC1	1.725336	9.53E-50
SLC13A5	1.725569	6.28E-37
PREX1	1.725755	3.33E-44
TMEM14C	1.725768	1.11E-52
TMEM250	1.726046	5.74E-53
AIDA	1.726716	5.63E-52
GNB2	1.726786	6.15E-52
PDE9A	1.726899	8.75E-46
ITGB7	1.727136	3.24E-48
CD69	1.727665	7.23E-38
KPNA3	1.728081	1.69E-52
TCF12	1.72847	3.29E-49
HDAC9	1.728646	1.09E-46
PLIN2	1.72867	8.62E-35
AL669918.	1.728689	6.24E-43
NCAPG	1.728939	6.67E-55
TENT5A	1.729204	8.64E-51
SLC39A3	1.729568	4.98E-54
SLC37A2	1.729572	2.43E-41
PCDH18	1.729616	2.94E-47
ATP5MC1	1.729759	6.33E-55
CPED1	1.729967	1.21E-45
IRS1	1.730405	3.01E-50
SMIM4	1.73046	4.94E-52
MAPRE2	1.73065	2.23E-50
PUDP	1.730673	1.14E-51
EGR3	1.730785	2.44E-42
TANK	1.731015	6.64E-51
UACA	1.731421	2.04E-47
PKIB	1.731465	3.57E-46
SYT17	1.731544	4.32E-48
SH3D19	1.73193	3.39E-46
DUOXA1	1.732028	1.2E-48
TPRG1L	1.732102	1.46E-54
ATP13A3	1.732224	6.09E-48
TPD52	1.732741	5.62E-47
DYSF	1.732953	5.67E-48
TMEM106I	1.733732	8.91E-53
DNAJA1	1.733778	5.14E-48
HNRNPR	1.733838	4.98E-52
SNAPIN	1.733993	7.46E-52
GMIP	1.734702	3.36E-49
RIPK2	1.734796	2.26E-46
CDC25B	1.735245	4.4E-47
PALM	1.735556	2.23E-42
PTGES3	1.736112	6.78E-52
PCGF2	1.736166	1.27E-47
HSPA1B	1.736469	1.53E-17
LRRC23	1.737158	9.97E-52
PRKD2	1.737158	2.76E-49
ELMO1	1.737229	2.14E-43
DOK1	1.737322	9.92E-49
TLN1	1.737364	1.12E-46
CENPH	1.737463	4.59E-55
UBE2V2	1.737537	1.17E-52
ANGPT2	1.737552	1.51E-51
AHCYL2	1.737586	2.94E-44

ZFP36L2	1.737601	9.84E-43
CYP2J2	1.73766	3E-49
NDUFB8	1.737679	6.17E-54
BRK1	1.738255	9.14E-53
SSBP1	1.738412	1.8E-53
TGFBR2	1.738445	1.69E-44
OGFOD1	1.738634	7.23E-52
TTLL12	1.738656	9.09E-50
RAB6A	1.738811	2.16E-51
EHD3	1.739015	2.4E-50
EIF4G2	1.739227	1.23E-50
AMFR	1.73974	1.39E-52
COP1	1.739745	9.05E-55
STK4	1.739792	3.66E-52
SDF2	1.739806	3.01E-54
RAB35	1.740362	1.67E-51
TRAFD1	1.740397	5.31E-50
GHITM	1.74072	6.81E-54
KIF4A	1.741103	3.09E-57
SMAD7	1.741262	3.86E-46
SAP18	1.741335	7.73E-55
DIABLO	1.741836	3.35E-53
CFAP300	1.742705	1.44E-53
NRBF2	1.742769	5.93E-53
HLA-DOB	1.742804	3.79E-46
CYTH2	1.742816	2.48E-51
CDH6	1.742822	6.81E-50
CALM3	1.743239	1.41E-52
CACNG4	1.743542	2.37E-43
NRAS	1.744651	3.22E-50
PARP9	1.744673	1.15E-45
GRK2	1.745174	2.7E-50
PRSS22	1.745185	1.6E-32
KCNAB2	1.745535	5.52E-42
ZNF480	1.745608	2.42E-52
DR1	1.745715	1.09E-50
NUP93	1.746012	4.17E-52
CACYBP	1.746147	4.01E-52
CDYL	1.746279	3.54E-53
MRPL42	1.747342	6.59E-53
ZNF668	1.747535	1.29E-53
UBE2E2	1.747601	3.07E-43
BIN3	1.747823	1.41E-51
COMMD5	1.748052	1.61E-53
FMN1	1.748141	2.52E-51
SORD	1.748915	2.58E-50
COMMD1	1.749096	1.05E-55
IGF2BP2	1.749224	3.96E-40
C11orf96	1.749361	6.76E-26
AIMP2	1.749732	1.81E-53
DNAJC17	1.750008	5.93E-53
AC026464.	1.75035	2.55E-55
ARID5A	1.750789	1.39E-42
DEDD2	1.751341	5.65E-53
C16orf91	1.751348	4.69E-55
GLI1	1.751481	3.08E-45
DDX24	1.751764	5.07E-52
CLSTN1	1.752016	2.83E-52
GRINA	1.752536	1.12E-49

RNPS1	1.752693	6.59E-53
ADGRL4	1.752813	2.52E-46
RB1	1.753	4.4E-48
TMEM19	1.753111	1.98E-52
SLIT3	1.753195	1.33E-38
ABCB1	1.753358	5.67E-42
TMC8	1.753551	5.39E-43
PDCD10	1.753944	5.75E-51
SNRNP27	1.753968	1.46E-51
RDH11	1.753991	1.98E-52
NUDT4	1.754194	6.68E-50
APMAP	1.754292	2.94E-52
UBXN10	1.754641	3.68E-54
NDNF	1.754732	4.77E-50
CMPK1	1.754859	8.81E-50
MAP1B	1.755035	5.05E-44
EHF	1.755137	1.19E-37
CD164	1.755592	8.05E-51
ESYT2	1.755674	6.38E-51
TNIP1	1.755962	1.31E-50
CSGALNA(	1.756274	9.11E-47
EMC3	1.756572	8.47E-55
HIC1	1.756935	1.27E-43
RPL28	1.75699	1.39E-49
SIRPA	1.757191	1.18E-41
HRAS	1.757192	1.31E-50
KCP	1.757408	7.99E-47
HIGD2A	1.757479	1.6E-52
SBNO2	1.757665	9.12E-47
MAX	1.758314	1.01E-51
CYFIP2	1.758375	1.36E-46
KPNA4	1.758443	1.13E-53
EFTUD2	1.758539	3.08E-53
BTNL8	1.758541	1.41E-28
PLCB2	1.758694	7.46E-35
DCTN1	1.758786	2.44E-52
TRAF3IP3	1.758924	8.51E-42
CSTF2	1.758927	2.89E-55
TNFAIP8L2	1.759419	5.09E-46
DNAJA4	1.75948	1.68E-43
GLRX2	1.759699	1.04E-52
TMEM140	1.760115	6.61E-49
HIP1	1.760332	2.15E-48
RPS6KA1	1.760411	1.35E-49
TEX264	1.760414	1.68E-55
ESD	1.760846	1.03E-52
TIMM10	1.761281	1.89E-54
ITGAM	1.761361	3.39E-40
PIP4P2	1.76142	1.08E-49
PSMD11	1.76169	1.03E-53
TMEM109	1.761848	1.89E-51
SORT1	1.761938	1.46E-50
LZTS1	1.762426	1.39E-46
ANXA6	1.762631	1.63E-45
TMEM59	1.762911	6.6E-55
ZNRF1	1.762955	5.15E-52
NCAPD2	1.762979	1.43E-51
FZD2	1.763075	5.45E-50
ST3GAL2	1.763274	8.59E-50

PTPN7	1.763603	3.15E-46
PPME1	1.763967	4.14E-53
FOXC1	1.764638	6.75E-47
UBE2M	1.764639	2.45E-53
GRK6	1.764946	1.21E-52
CCZ1	1.765045	2.84E-54
CAPZA1	1.765872	2.55E-48
GLO1	1.765993	1.08E-53
FAM32A	1.766155	2.58E-53
TRAPPC1	1.76631	5.72E-52
TMEM35B	1.767026	1.35E-50
MAP2K1	1.767413	6.67E-52
TBCA	1.767644	2.83E-52
U2AF1L5	1.767712	1.07E-53
SRGAP2B	1.767833	5.32E-47
EPHA3	1.768002	2.66E-44
SLC25A12	1.76819	7.79E-51
TTC1	1.768199	7.27E-54
NDUFS6	1.768282	6.49E-55
IYD	1.7683	8.04E-47
SH3PXD2B	1.768554	1.8E-44
PLOD3	1.768558	8.18E-51
SLC26A9	1.768781	8.44E-23
MANBAL	1.768895	7.96E-54
CKAP4	1.76892	1.74E-52
CARD6	1.768947	4.38E-46
LRRC20	1.769208	2.11E-53
RNF183	1.769255	5.6E-49
PSMB4	1.769959	7.64E-54
PRKAR1A	1.769961	7.09E-49
KIF5B	1.770231	3.08E-53
THBD	1.770318	2.77E-43
CRP	1.770781	5.22E-08
DOK2	1.770851	3.11E-45
TADA3	1.770981	1.07E-52
VSTM4	1.771139	5.07E-49
ACBD3	1.771399	5.25E-53
PEF1	1.771874	3.85E-52
CHP1	1.771966	2.42E-51
STMN3	1.771992	1.07E-41
PRCP	1.772003	5.35E-49
ENG	1.772022	1.92E-45
RUVBL2	1.772224	6.07E-54
BTN3A3	1.772273	1.03E-44
NUCKS1	1.772626	2.87E-52
WNK1	1.772765	3.19E-46
RPN2	1.773483	1.2E-54
NUDT4B	1.773697	6.19E-41
MFSD1	1.773719	1.57E-50
UGCG	1.773761	6.26E-47
TNNT1	1.773762	1.02E-20
ITPKC	1.773852	3.55E-47
WASL	1.774267	4.2E-52
PROM2	1.774331	3.09E-30
FHOD3	1.774389	4.92E-44
GNG5	1.775104	7.74E-51
TMEM47	1.775221	8.48E-46
SLBP	1.775436	5.29E-53
DNTTIP2	1.775562	1.28E-54

FAM102B	1.775881	6.29E-49
ARFRP1	1.77589	3.03E-51
EHD4	1.776032	1.29E-49
WDR82	1.776101	1.7E-51
SLC30A1	1.776198	8.29E-52
EIF4A3	1.776395	3.32E-52
CAMSAP2	1.777703	2.93E-51
FLI1	1.778322	5.07E-45
POLR2E	1.778388	1.43E-53
SUMF1	1.778391	2.94E-53
IFT52	1.778659	5.51E-53
GJA5	1.778777	1.76E-49
PHF20	1.779081	9.91E-53
SNX9	1.779088	1.23E-50
SPHK1	1.77935	1.56E-37
PLD4	1.77948	4.76E-49
CNRIP1	1.779794	4.92E-45
PLEKHB1	1.779968	1.41E-46
NUDCD3	1.780247	4.7E-54
UBA1	1.780459	1.85E-52
BTN3A2	1.780468	1.29E-42
HNRNPC	1.780472	2.61E-52
ADORA2B	1.780832	2.52E-54
C11orf49	1.780865	1.09E-53
CD247	1.780918	1.6E-50
MAGOH	1.781065	1.07E-53
CTSW	1.781071	1.09E-47
YPEL5	1.781349	7.17E-52
ZSWIM4	1.781351	9.91E-50
DTX1	1.78143	3.38E-44
DHX8	1.781479	2.33E-54
FOXF2	1.781509	5.62E-51
PLK3	1.781723	2.89E-42
MRM2	1.781784	5.18E-55
ISM1	1.781788	4.43E-35
ATP6V0C	1.782224	7.8E-51
ACBD5	1.782368	4.17E-53
PLEKHG2	1.782403	6.31E-44
IL10RB	1.782507	1.91E-52
RAB3IL1	1.782574	8.61E-44
GUK1	1.782838	1.04E-50
HSP90AA1	1.782868	5.07E-46
GGCT	1.783143	2.79E-54
NSL1	1.783342	9.59E-53
CST4	1.783899	4.62E-60
ERBB2	1.783927	1.68E-42
IFITM10	1.783983	5.28E-49
ARF4	1.783986	1.23E-50
STAMBP	1.784397	3.82E-54
GPKOW	1.784672	5.63E-55
LRRN1	1.784706	1.08E-39
MYH9	1.784729	1.91E-44
SYAP1	1.784763	7.72E-55
BTBD1	1.78486	5.27E-52
HSD17B12	1.785031	4.19E-54
ABI1	1.785323	4.94E-52
HAGHL	1.7855	3.03E-44
IL4R	1.785523	4.44E-36
ADO	1.785579	8.52E-55

AC008982.	1.785636	1.65E-23
EMG1	1.785639	1.13E-54
SH3BGRL2	1.785801	1.64E-50
TPP1	1.786064	1.06E-48
DAD1	1.786271	6.71E-55
CYP51A1	1.787179	4.01E-51
BAG5	1.787342	4.36E-54
BANF1	1.787739	3.94E-53
PTGDS	1.788055	5.61E-25
ERG28	1.789074	1.8E-54
FSTL3	1.78913	3E-30
ADAMTS14	1.789352	1.1E-51
GPC1	1.789601	7.22E-42
RIT1	1.789729	2.2E-51
TRAPPC2L	1.789913	8.61E-55
IKBKE	1.790151	6.56E-50
ECH1	1.790536	1.94E-54
B3GALT5	1.791095	3.97E-42
PLGRKT	1.791157	3.43E-53
ATG101	1.791199	5.27E-54
ARL2BP	1.791431	5.81E-52
COPZ2	1.791658	1.07E-45
RAB2A	1.791841	1.36E-52
PSMB6	1.791881	3.85E-54
POLR3K	1.792658	2.46E-56
SLC35C1	1.792778	3.55E-52
LPCAT1	1.792951	6.36E-47
STIP1	1.793181	1.83E-50
RFX5	1.793183	1.28E-49
MRFAP1	1.793233	1.9E-51
MTA2	1.793487	1.32E-52
SPG21	1.79359	5.81E-52
SSB	1.794492	2.4E-52
GRIN2D	1.794646	2.56E-54
TBC1D10A	1.794772	1.2E-50
TNFSF13B	1.79511	4.88E-47
SLC35F6	1.795124	1.63E-53
DUSP2	1.795493	3.32E-41
CBX3	1.795651	5.66E-51
HPSE	1.795724	3.8E-55
PPARD	1.795787	2.82E-48
LRP5	1.795872	9.46E-50
NBR1	1.796489	5.82E-54
AOAH	1.796546	1.62E-44
FOS	1.796577	3.71E-21
AIP	1.796598	1.32E-51
SIPA1L2	1.796615	2.3E-46
MEGF6	1.796969	7.19E-38
CIITA	1.796982	1.36E-40
DOCK10	1.79715	3.11E-45
FLT3LG	1.797242	4.39E-49
PRRG1	1.797524	2.26E-52
FAHD1	1.797592	9.51E-55
E2F3	1.797666	1.18E-52
DCTN5	1.797698	3.46E-53
ELK1	1.798248	3.97E-52
BTBD6	1.798419	1.74E-43
PARVG	1.798513	5.51E-35
LAMP2	1.79908	4.45E-52

UBE2Z	1.79918	1.28E-52
PPP1CA	1.799369	2.01E-53
TMEM130	1.799562	1.53E-49
COX6A1	1.799613	6.6E-55
IL2RA	1.799959	1.23E-51
JDP2	1.800043	1.35E-48
SRGAP2	1.801018	1.39E-46
IK	1.801082	7.45E-54
ARMC7	1.801677	4.89E-55
RANGRF	1.801856	1.37E-46
NSFL1C	1.801922	1.17E-53
CCT3	1.802656	5.32E-54
PAK1	1.802747	2.56E-49
PSMD2	1.802788	3.43E-53
CCL26	1.803444	1.4E-50
NAB1	1.804121	1.1E-51
WEE1	1.804372	2.36E-48
LYPLA2	1.804429	8.5E-52
ARHGEF35	1.804798	9.71E-41
SYPL1	1.804857	2.23E-51
FAM83H	1.80486	1.84E-44
CTXN1	1.805372	2.16E-45
GABARAPL	1.80538	3.3E-52
R3HDM4	1.805483	1.02E-49
LACTB2	1.806145	6.25E-49
MTHFD1L	1.806869	1.72E-49
PIK3C2B	1.807316	6.29E-52
CCNG2	1.80775	9.56E-44
FANCI	1.808174	1.14E-53
HTATSF1	1.80831	1.82E-53
TSPAN14	1.808671	6.1E-48
SPIB	1.809198	9.92E-36
UBE2I	1.809211	1.67E-52
RPS10-NU	1.809388	4.57E-48
TMEM63B	1.809598	1.54E-52
GALNT12	1.81013	5.13E-47
ENSA	1.810201	1.22E-54
CCL22	1.81035	2.71E-52
SERPING1	1.810613	3.42E-37
SFXN3	1.810688	1.63E-46
CABLES1	1.810962	2.81E-53
ZNF521	1.811306	2.98E-47
PSMA6	1.811337	3.64E-54
ATG9B	1.81138	1.6E-51
YBX1	1.811908	3.79E-51
TMEM53	1.812335	7.27E-54
POLR2G	1.812883	8.35E-53
SKP1	1.813014	1.52E-51
PUF60	1.813028	2.49E-54
RRP7A	1.813431	2.06E-54
AGPAT1	1.813705	8.57E-54
SGMS2	1.813925	3.36E-47
RABIF	1.81408	1.5E-55
SQOR	1.814154	4.61E-49
VWF	1.814262	5.41E-47
HEXA	1.814326	1.01E-51
MTMR2	1.814401	4.14E-52
ATP2A2	1.814609	1.45E-50
PSMA7	1.815154	5.12E-53

BCL7B	1.815166	3.73E-54
OGDH	1.815404	3.52E-53
PODN	1.815552	1.47E-35
CILP2	1.815561	8.25E-53
TIMM8B	1.815692	4.25E-54
PTPRA	1.81577	3.49E-52
PRCC	1.815775	3.87E-55
TRPM4	1.816784	2.08E-40
SMPD3	1.817061	2.7E-44
C1GALT1C	1.81764	6.76E-55
SMARCE1	1.817838	9.7E-54
NAA38	1.817848	1.49E-53
C17orf49	1.818078	2.44E-52
VASN	1.818254	2.3E-43
WASHC3	1.819027	1.12E-53
SP110	1.819159	3.41E-44
DPY30	1.820614	1.73E-54
UHMK1	1.820679	6.64E-54
HMMR	1.821021	1.65E-54
FUT11	1.82145	4.78E-52
SLC52A2	1.821606	1.96E-52
COX6C	1.822041	5.5E-55
NDUFA3	1.822402	3.14E-54
PRR11	1.822522	4.55E-56
COQ10B	1.82285	3.37E-53
CDH2	1.822898	2.86E-48
PDCD6	1.822932	9.51E-55
STK26	1.823326	1.64E-51
ENC1	1.823343	3.68E-39
MICOS10	1.823413	1.85E-54
BLVRB	1.823522	2.27E-50
LTBP4	1.82353	3.55E-47
CAPN12	1.823803	2.14E-30
SH2D2A	1.823987	2.3E-47
SLC35F2	1.824234	1.09E-46
RTF2	1.82426	4.86E-54
TNFRSF11/	1.824368	5.36E-48
ZBTB7A	1.82441	6.86E-54
FAH	1.824765	1.55E-53
PTPN1	1.824785	8.93E-51
FABP6	1.824808	1.1E-43
ZNF532	1.825189	1.58E-44
SYT12	1.82523	5.03E-48
EFNA1	1.825582	3.71E-39
COX8A	1.825672	3.21E-54
CHST4	1.826183	2.61E-31
PIGS	1.826408	5.19E-52
SPC25	1.826649	1.79E-54
SINHCAF	1.826682	6.96E-48
SEL1L3	1.826782	5.91E-44
CISD1	1.827697	2.09E-54
S100A9	1.828841	8.57E-26
C11orf80	1.828902	9.47E-47
MYO5B	1.829225	1.6E-48
IGSF9	1.829896	9.07E-38
PSMB5	1.830144	1.22E-54
EIF4E2	1.830183	3.64E-52
USP5	1.830261	3.88E-54
HOXB3	1.830373	5.17E-44

DOCK2	1.830807	7.49E-43
DNAJC8	1.831032	1.02E-50
XRCC6	1.832761	1.92E-54
TCF4	1.833234	1.88E-45
MPHOSPH	1.833256	7.05E-52
TPST1	1.833329	8.44E-48
UBE2K	1.833645	9.7E-54
PHAX	1.833845	7.57E-53
KCTD11	1.834311	2.4E-52
SPN	1.834527	9.36E-49
CCDC115	1.834613	1.36E-52
XRCC5	1.834693	5.56E-53
CRISPLD1	1.835031	1.56E-52
ST3GAL1	1.835369	1.17E-44
S100A3	1.835486	9.89E-51
APH1B	1.835798	6.48E-51
DTYMK	1.835875	1.32E-53
IRF2	1.836062	3.03E-51
FLOT2	1.836175	3.89E-48
TXNDC17	1.836435	4.02E-54
HNRNPA3	1.836462	2.75E-53
CAMTA1	1.836922	7.59E-52
GNAQ	1.836947	1.06E-52
IRF9	1.836958	4.08E-44
CMBL	1.836984	2.29E-50
APEX2	1.837053	7.28E-55
SERPINF1	1.837315	2.48E-36
TOMM34	1.837399	6.58E-54
PARP14	1.837499	2.73E-47
CLCF1	1.837996	3.12E-40
APOL3	1.838171	4.45E-46
WWTR1	1.838701	3.68E-42
PDE1A	1.838731	6.1E-50
DOCK9	1.839512	2.31E-48
DESI2	1.839849	2.28E-53
BANK1	1.839975	4.44E-51
IFNGR1	1.84003	3.71E-47
EHBP1	1.840135	5.79E-50
EIF5A2	1.841043	1.69E-52
FAM222B	1.841101	8.02E-54
TOP1	1.841512	4.49E-52
DACT3	1.841563	7.08E-50
SMIM30	1.841671	8.91E-53
FHL3	1.841803	1.2E-43
MICALL2	1.842266	2.43E-37
COX17	1.842319	2.64E-55
KCNE3	1.843093	1.12E-36
PLSCR3	1.843378	1.06E-48
CDC123	1.843453	2.2E-54
NCS1	1.843577	2.7E-50
TRIM21	1.843659	2.23E-50
SNCA	1.843699	9.9E-47
TCEAL7	1.843866	1.17E-49
KIF11	1.844092	5.36E-56
NCK1	1.844386	7.64E-50
PRMT2	1.84484	3.43E-50
MAP1A	1.844906	6.4E-52
TRIQK	1.845197	5.42E-53
GLIS2	1.845206	3.18E-41

SERPINB3	1.845375	3.62E-32
NDUFS4	1.845412	1.23E-53
POLE4	1.84558	6.36E-49
CENPA	1.846141	8.62E-54
ITFG1	1.846829	2.75E-53
HMGB3	1.846831	4.26E-46
SUSD1	1.847407	1.7E-51
KDELR1	1.84742	2.12E-53
G3BP1	1.84759	7.17E-52
PECR	1.84817	1.17E-53
SLMAP	1.848292	1.7E-51
GOLIM4	1.850105	4.5E-49
PARVB	1.850753	1.92E-45
ABHD2	1.85077	5.39E-46
ITSN1	1.850827	5.97E-49
POU2AF1	1.851728	5.57E-47
PGM2	1.852077	1.18E-49
DERL1	1.852082	2.26E-53
TGOLN2	1.852104	5.85E-51
TIMM17B	1.852728	3.27E-54
RAMACL	1.853032	2.57E-42
PBDC1	1.853201	1.19E-53
CHCHD3	1.853234	5.58E-54
TNIK	1.853845	1.23E-49
MFSD14B	1.854558	2.15E-52
ZEB2	1.854871	1.07E-42
FCGRT	1.855895	1.08E-52
NAALADL1	1.856377	1.58E-47
HR	1.856531	2.88E-54
CPT1C	1.856789	1.87E-46
PPM1N	1.856901	8.43E-54
TIE1	1.857418	1.4E-45
SCG2	1.857536	9.52E-24
UCHL1	1.857801	9.52E-37
TNFSF9	1.858009	1.1E-53
MAST2	1.858063	4.9E-50
TM9SF3	1.858696	2.37E-54
PLA2G15	1.858725	7.92E-51
ATP6V1H	1.858886	2.6E-53
CCL15	1.859966	1.05E-49
KDELR2	1.86054	3.76E-54
TMCC3	1.860886	2.32E-52
CPNE2	1.861153	6.59E-48
SZRD1	1.861279	8.5E-52
SCARB1	1.861656	8.86E-51
LAMTOR4	1.861966	7.83E-53
LAMP1	1.862267	4.98E-54
TRAPPC6A	1.862477	5.65E-53
CAB39	1.862526	6.83E-52
PIGT	1.862985	5.68E-55
FOXA1	1.863135	2.11E-49
GNLY	1.863699	5.12E-49
ABI3BP	1.864012	1.51E-36
ATP9A	1.864245	2.11E-50
TECR	1.865082	1.39E-54
GPR176	1.865102	3.1E-49
VMP1	1.865405	3.27E-39
SWAP70	1.866444	1.63E-47
PLK2	1.866481	1.13E-44

TEAD3	1.866556	4.45E-46
PRRG4	1.866623	3.14E-51
SPTAN1	1.867336	7.83E-50
CHMP1A	1.868014	6.59E-53
UBE2A	1.868133	1.3E-52
IRF7	1.868227	5.24E-37
PCSK6	1.868239	6.07E-45
KRT18	1.868975	2.13E-40
GATA3	1.869169	1.3E-52
TPD52L2	1.869425	7.83E-53
BUD23	1.870226	2.36E-56
CFAP20	1.870345	6.98E-53
GNAS	1.871139	2.73E-51
LEPROTL1	1.871825	5.98E-53
IDS	1.87204	2.83E-49
KIAA1755	1.87228	2.91E-47
C19orf53	1.872491	7.96E-54
RGS19	1.872869	5.55E-48
GNE	1.872999	2.29E-53
DENND2D	1.873	2.97E-49
EIPR1	1.873711	7.72E-55
DTX2	1.873788	1.29E-49
VPS72	1.874159	3.46E-54
CENPN	1.87526	1.33E-54
RBM34	1.876095	1.37E-54
EFCAB11	1.876632	2.36E-55
POLR2K	1.877135	1.64E-53
ATP5IF1	1.87739	1.64E-54
KHDRBS1	1.877694	5.12E-53
AC011511.	1.878157	8.93E-51
SLFN5	1.878313	4.49E-49
ADAMTSL2	1.87842	2.01E-42
DEGS2	1.878628	9.64E-40
KIF23	1.878911	7.82E-53
WFDC1	1.879403	3.7E-52
PFDN4	1.879803	2.2E-54
MCM4	1.880062	2.54E-50
BROX	1.880183	3.82E-54
ZDHHC5	1.880184	8.33E-55
NAA50	1.880276	1.16E-51
KIF13B	1.880699	1.52E-50
RAB20	1.880806	1.55E-51
PIP4K2A	1.880849	1.2E-48
TAF13	1.880895	7.95E-53
SEPHS2	1.881125	2.97E-55
NDUFS5	1.881247	1.35E-52
HOXA10	1.881264	2.03E-52
CAMK2D	1.881938	1.01E-50
DUSP14	1.882129	1.23E-52
BCAP31	1.882558	3.47E-56
TAX1BP1	1.882897	5.14E-54
CDCA4	1.88338	2.52E-55
NFE2L2	1.883436	5.66E-50
AHSA1	1.88366	9.78E-54
HMOX2	1.884364	4.43E-55
CHCHD1	1.884561	2.43E-55
FAM229B	1.884648	1.17E-50
NTPCR	1.884882	5.31E-55
BAMBI	1.884972	1.69E-50

SMOC2	1.885115	5.17E-38
ZBTB7C	1.88574	2.51E-51
EXOSC4	1.885866	1.41E-53
GDPD3	1.885915	7.89E-45
ARMC9	1.885977	4.98E-52
KIF3C	1.886005	4.89E-50
STMP1	1.886215	9.08E-54
RAB4B	1.886254	7.58E-50
HNRNPF	1.886389	1.24E-51
CENPK	1.886487	1.79E-52
PCDHGC3	1.886807	1.61E-46
MBNL1	1.887049	1.56E-49
SPOCD1	1.887149	1.09E-52
TNFAIP8L1	1.887418	2.55E-51
VGLL4	1.887485	1.38E-48
ANXA7	1.887548	3.94E-53
MCM5	1.887573	1.51E-51
TSC22D3	1.887676	8.06E-36
DHRS7	1.887748	4.53E-53
SEMA3F	1.888744	6.35E-49
CNP	1.889262	2.1E-53
DNAJB6	1.889692	4.52E-52
SERINC3	1.889719	3.29E-54
SMIM6	1.89078	4.78E-48
TUBB6	1.890783	1.37E-48
ABI2	1.891043	9.14E-51
TSG101	1.891363	1.55E-53
FOSL2	1.891507	5.37E-39
TWF1	1.891549	9.67E-53
OST4	1.891791	2.06E-54
NACC1	1.891829	6.26E-54
HOXA3	1.891986	8.22E-52
CCDC71L	1.892428	2.09E-50
RHOB	1.892877	2.42E-39
HDAC1	1.893386	3.63E-53
RAB10	1.89344	8.23E-52
CD1C	1.893814	2.27E-47
MSX2	1.894534	3.33E-49
ADGRG1	1.894644	4.87E-48
TSPAN9	1.895197	4.17E-48
FBP1	1.895495	6.56E-44
IL10RA	1.895827	4.54E-42
VAT1	1.896374	4.29E-49
PFKM	1.89764	2.22E-50
POLR3GL	1.897748	9.21E-53
CXCR5	1.897869	6.21E-49
PRKCD	1.897968	1.36E-50
FUNDC1	1.898443	9E-54
CACNA1C	1.898983	6.58E-51
ITGAX	1.898995	1.06E-35
NFIX	1.899873	1.69E-47
MRPS6	1.900128	1.01E-52
RUVBL1	1.900136	6.54E-55
OLFML3	1.900479	3.39E-39
SELP	1.901182	1.42E-41
TAF7	1.901322	3.38E-52
CASP3	1.90137	4.64E-53
PELI1	1.902117	8.27E-50
TXNDC9	1.902291	5.06E-54

DCK	1.902366	1.14E-50
PABPC1	1.902393	8.5E-52
TRIB1	1.902527	8.98E-47
CD300LF	1.902579	3.15E-49
DSE	1.902766	2.12E-44
CYTIP	1.902781	7.88E-44
LMO2	1.902845	7.94E-47
SERINC5	1.903383	4.11E-51
LSM8	1.903808	1.14E-52
G0S2	1.903822	1.04E-29
VSIG4	1.9044	6.31E-30
TOX3	1.904633	1.24E-43
RNASEH2A	1.904763	4.28E-55
ODF3B	1.904818	7.09E-38
TNFSF13	1.904898	6.31E-45
DGAT2	1.904925	2.75E-42
ELOVL1	1.905108	3.03E-51
GZMK	1.906392	2.24E-48
FAS	1.906442	1.53E-49
RP2	1.907096	1.88E-52
RAP1B	1.90711	3.12E-50
STS	1.907178	9.88E-52
NGRN	1.907275	5.93E-53
MED27	1.907552	3.13E-54
EFEMP1	1.90785	2.65E-33
LGR6	1.908288	6.86E-49
DAZAP2	1.908561	1.78E-51
PANX1	1.908592	6.09E-52
UBE2L3	1.909102	1.2E-54
NDUFA8	1.909459	1.69E-55
TNFSF11	1.909671	3.94E-51
ARHGAP25	1.909779	6.37E-48
FGF19	1.909852	2.29E-50
PON2	1.90993	1.85E-49
MRPL15	1.91021	1.09E-54
AP1M2	1.91045	1.83E-50
TMEM184B	1.910622	1.34E-51
POC1B	1.910764	5.8E-52
GBP5	1.910978	3.51E-47
COMMD4	1.911337	2.14E-54
PPP1R13L	1.911951	1.09E-41
NDUFA12	1.912072	1.22E-54
GSDMB	1.912082	5.89E-37
PTPN9	1.912554	6.66E-52
HJURP	1.912624	2.47E-54
APH1A	1.913402	2.01E-54
NTHL1	1.913597	2.76E-52
GSK3B	1.913838	8.25E-55
RUNX3	1.913892	1.06E-50
RTL8A	1.914135	5.03E-52
PTPN12	1.914156	3.09E-49
UQCRH	1.914702	1.63E-56
WNT7A	1.915291	2.56E-45
CCDC12	1.915701	2.39E-54
ADCY7	1.91576	2.35E-43
EPHB2	1.916184	3.28E-46
MYO1D	1.916654	9.24E-50
FCMR	1.916962	1.98E-45
NPTN	1.917922	2.63E-52

PSMD10	1.917988	3.29E-53
PELO	1.918211	9.98E-53
ABI3	1.918556	1.13E-47
TRIM22	1.918623	1.88E-38
UBQLN2	1.919367	1.61E-53
RAB1B	1.920637	2.45E-54
ZFAND6	1.920669	4.45E-52
MAGED1	1.921122	3.7E-54
CRNKL1	1.921571	3.65E-55
SUSD2	1.922099	2.96E-41
NLRC5	1.922237	8.64E-45
ADM	1.922381	1.88E-33
KLHL5	1.922531	1.2E-47
RELT	1.922544	4.16E-50
ZMPSTE24	1.922729	2.06E-53
SPC24	1.922876	1.93E-55
NXT1	1.923427	4.43E-54
ITPK1	1.923615	4.62E-54
RGCC	1.924442	9.47E-47
PXN	1.924576	5.94E-51
UXS1	1.924748	1.53E-52
BRIX1	1.924929	1.42E-55
MILR1	1.924952	4.45E-46
POR	1.925601	1.05E-50
FBLIM1	1.92566	1.26E-47
PRKAR1B	1.925692	9.45E-54
TP53INP2	1.926028	2.3E-50
CDC45	1.926107	6.8E-55
RAD51	1.92654	2.61E-55
DPP4	1.926564	3E-48
ZNHIT1	1.926623	2.72E-54
PPP1R3B	1.926666	4.46E-49
EHD2	1.926669	5.25E-44
NDUFA7	1.92715	4.62E-56
CTSO	1.927389	7.34E-50
PNKD	1.92782	6.77E-55
PKDCC	1.928318	4.23E-40
SDHC	1.928547	1.76E-54
ZNF267	1.928672	8.75E-53
MSMB	1.928959	1.71E-26
FCER1A	1.929071	2.32E-49
RAP1A	1.929676	1.08E-49
ATP6AP1	1.930991	1.01E-53
MTX1	1.931178	2.51E-55
EVA1B	1.932037	6.91E-42
HSPA4	1.932086	1.12E-55
KAZALD1	1.932656	2.47E-50
RRAD	1.93313	2.37E-42
GGPS1	1.933453	1.3E-54
FAM110A	1.933499	4.38E-51
CNTN1	1.933798	6.88E-45
PSMC2	1.934012	1.65E-53
SCNM1	1.934417	5.46E-53
GALNT1	1.934427	6.48E-53
P3H3	1.934792	1.13E-48
SOWAHC	1.934848	1.08E-54
NDUFB6	1.934926	4.73E-55
TAPBPL	1.935685	1.2E-52
CPE	1.935765	2.58E-35

COMMD6	1.936429	1.45E-53
FCGBP	1.936446	6.91E-34
NEK6	1.937377	9.48E-48
CDH13	1.937468	6.61E-54
ADIRF	1.937498	7.99E-39
TRABD2A	1.938707	2.53E-53
EMC10	1.938992	1.41E-51
PMVK	1.938997	2.52E-56
OXCT1	1.939092	1.34E-50
NDUFA1	1.939733	4.29E-56
NKIRAS2	1.939918	1.87E-53
FBXO34	1.9401	1.47E-51
KLHL25	1.940113	1.71E-54
RALY	1.940139	6.23E-53
TOM1	1.940265	5.86E-52
BMP1	1.940505	1.14E-45
POC1A	1.940603	8.47E-57
SERPINB8	1.940808	5.18E-49
PRKCI	1.941587	9.9E-53
PAK2	1.941927	8.08E-53
ORAI2	1.942366	6.33E-51
IRF6	1.942501	5.31E-50
PKIA	1.942605	8.64E-52
PHF23	1.942639	2.96E-53
XXYLT1	1.943578	3.69E-54
LHPP	1.944342	1.01E-53
RELB	1.944696	6.68E-43
EGLN2	1.944833	9.98E-52
TBC1D1	1.945669	1.48E-50
SLC44A2	1.945736	3.14E-49
CDT1	1.945871	7.76E-56
CASK	1.945907	2.4E-52
C5AR1	1.946984	7.18E-43
SAMSN1	1.947261	8.86E-44
BEAN1	1.947624	1.66E-51
TFDP1	1.948187	4.05E-54
MAP1LC3E	1.948289	2.06E-51
EIF4E	1.948367	3.63E-52
PET100	1.948458	1.73E-54
NUF2	1.948646	8.15E-56
ORAI1	1.94936	7.21E-53
STK38L	1.949685	3.18E-49
IL16	1.950043	6.62E-43
POU2F3	1.950156	5.46E-44
TIPARP	1.950931	1.38E-49
CDK5	1.951642	5.45E-54
ATP2C1	1.95171	3.01E-51
ROR2	1.951727	5.94E-48
CD79B	1.952694	9.77E-45
G3BP2	1.952829	1.19E-52
FDFT1	1.952865	2.46E-51
CCN1	1.953247	2.23E-28
G6PD	1.95333	7.29E-52
FAM114A1	1.953751	5.58E-52
TPRG1	1.954242	3.9E-47
SPIRE2	1.954497	8.63E-45
PKIG	1.955294	5.98E-50
TGFB2	1.955588	7.75E-42
MGAT3	1.955792	3.13E-46

COLGALT1	1.955822	2.66E-49
LAMTOR5	1.955955	1.5E-53
TMEM204	1.95676	1.88E-45
BLACAT1	1.956959	1.08E-49
C16orf74	1.957616	2.17E-44
HCCS	1.957682	4E-55
CALCRL	1.957882	5.62E-47
SARNP	1.958064	5.33E-53
FKBP1A	1.958245	7.06E-52
FAM118B	1.958452	1.95E-54
NUCB1	1.95859	3.24E-53
OLFML1	1.958672	1.47E-45
SERPINB2	1.959436	6.84E-36
DLL4	1.959984	1.65E-48
RPL26L1	1.960865	1.64E-54
PEPD	1.960967	5.8E-51
SNAI1	1.961261	3.23E-46
CCDC51	1.961435	4.9E-56
TEDC1	1.961567	2.01E-50
SH3RF1	1.962972	2.27E-52
TIMM23	1.963063	1.6E-55
MMP10	1.963313	2.98E-49
GSDME	1.96342	3.62E-46
TFEB	1.963457	5.56E-49
C14orf119	1.963878	9.46E-54
SRP9	1.964502	1.1E-54
UBAC2	1.96501	4.98E-54
RPE	1.965082	2.52E-52
MMP19	1.965316	2.82E-38
SKA2	1.965468	4.7E-54
FIBIN	1.965728	3.71E-38
ATP5MC3	1.966175	6.07E-55
HSH2D	1.967367	1.87E-47
HEXB	1.968143	2.26E-53
TALDO1	1.968679	2.84E-54
KCTD10	1.969224	1.16E-50
RBM42	1.969635	7.77E-54
PPIH	1.969829	2.57E-54
E2F1	1.97045	4E-56
GLA	1.971481	1.7E-52
PSMA5	1.971914	1.51E-54
MRPL28	1.971935	4.38E-53
SF3B6	1.972328	1.53E-54
CCT5	1.972399	3.95E-54
HMGN4	1.973112	1.96E-51
SEZ6L2	1.973817	1.34E-52
TPBG	1.974431	1.49E-45
DPP3	1.974531	4.38E-53
NPL	1.97457	3.58E-49
PI4K2A	1.974916	2.42E-52
ABR	1.97525	3.45E-49
VPS35	1.975601	2.73E-53
MNDA	1.97569	1.92E-41
HMGB1	1.975762	8.29E-53
UQCR10	1.975868	1.42E-55
GPR132	1.976065	3.42E-50
CCL13	1.976194	2.01E-43
ICMT	1.976242	4.43E-54
SCO2	1.977874	1.64E-52

TMEM43	1.978062	1.39E-50
SMIM29	1.978098	7.86E-51
ARL8A	1.978161	2.03E-53
PRTFDC1	1.978736	1.73E-52
SPOCK1	1.978818	1.02E-37
C1orf21	1.978871	2.05E-48
TRIM54	1.978986	9.72E-41
GNB4	1.979255	7.73E-48
TCEAL9	1.979409	3.17E-50
TMC6	1.979515	2.65E-42
PTGS1	1.98026	2.71E-48
SYT8	1.98044	2.82E-20
PLEKHG1	1.980452	5.55E-51
IDH1	1.980482	2.52E-51
SF3B4	1.981623	3.38E-54
CTSL	1.98184	1.33E-47
GAS7	1.981858	4.06E-46
FEN1	1.981897	9.35E-56
ARID5B	1.982019	1.05E-49
STX7	1.98202	5.56E-49
MSC	1.982409	1.13E-42
OPTN	1.982539	6.08E-53
ATP6V0D1	1.982841	4.75E-52
GALNT14	1.982899	1.14E-41
SAMD9L	1.983662	1.17E-44
PAFAH1B2	1.983699	2.91E-54
KITLG	1.983797	4.99E-45
YAP1	1.983841	7.55E-44
MFNG	1.984078	1.24E-48
STK17B	1.984883	1.9E-46
GIMAP4	1.984993	1.89E-45
RPS6KA4	1.985043	1.63E-51
CKAP2	1.985261	1.47E-54
SNX12	1.985377	2.39E-54
DCTPP1	1.98571	7.54E-55
DNASE2	1.986394	4.43E-54
ASAH1	1.986395	4.11E-52
PTPN6	1.986501	2.64E-51
NME7	1.986728	2.41E-54
SLC39A11	1.986768	1.99E-55
SPTSSA	1.986788	4.94E-52
MORF4L1	1.986798	1.25E-51
MRPL13	1.986934	8.53E-56
UQCR11	1.987958	9.05E-56
NPR3	1.988726	1.53E-49
CCNA2	1.988994	9.79E-54
PRAG1	1.989009	3.75E-49
NSDHL	1.989047	2.33E-55
GTF2IRD1	1.990847	1.68E-51
SAPCD2	1.990861	2.01E-56
PDGFC	1.990942	3.7E-41
NCF4	1.991055	1.99E-43
XDH	1.991832	4.39E-50
TGFA	1.992661	7.16E-44
C11orf98	1.993047	3.42E-55
FMNL3	1.993319	1.33E-48
PIGU	1.994223	4.07E-56
KEAP1	1.994706	8.53E-56
TRMT112	1.994954	1.51E-54

PDCL3	1.995523	8.71E-54
RCSD1	1.995906	3.93E-48
IGF2	1.996759	4.84E-32
MAF	1.997061	1.45E-48
NAAA	1.997455	6.69E-51
COMT	1.997712	2.89E-53
ECM2	1.9997	7.97E-48
F11R	1.999839	1.4E-48
DLGAP5	2.000177	1.08E-55
CPNE7	2.000341	3.62E-47
PLEK	2.000478	2.26E-44
CRIM1	2.00194	1.27E-48
CNN2	2.001943	3.97E-43
HNMT	2.001954	2E-44
PLXNC1	2.002054	4.63E-49
THOC3	2.00259	1.44E-53
RAB8A	2.00316	5.93E-53
SMOX	2.003386	2.62E-50
CHMP2A	2.003764	4.89E-55
CARHSP1	2.003816	1.1E-52
SH3D21	2.00457	4.42E-51
ARPC5L	2.004695	4.46E-55
EXT1	2.004754	1.9E-50
NDUFB2	2.005511	2.68E-54
AVPI1	2.005746	4.24E-53
OSCAR	2.005788	3.73E-47
AP2S1	2.006034	6.92E-54
ARHGAP45	2.00607	1.82E-49
CPPED1	2.006194	2.55E-50
DDR2	2.006238	2.79E-45
FANCA	2.006345	1.53E-50
PRC1	2.006411	5.58E-52
SPRED1	2.006619	2.23E-51
PDGFB	2.006823	2E-47
SLC6A8	2.007116	1.4E-38
RTL8C	2.007726	4.87E-50
PHB	2.007747	8.68E-56
WAS	2.007903	8E-44
B4GALT1	2.008095	8.88E-44
NLRP1	2.008416	3.93E-42
PLA2R1	2.008738	2.46E-51
PROM1	2.009089	4.05E-36
CGB8	2.009539	2.99E-51
SASH3	2.009761	6.15E-43
IER5	2.01006	8.84E-52
CYGB	2.010827	4.04E-46
BUB1	2.010906	1.72E-55
RHNO1	2.010956	7.57E-54
RGS3	2.011033	1.1E-50
PIM1	2.01139	1.83E-40
NCKAP1L	2.011807	3.47E-43
CEBPB	2.011829	1.64E-45
IGSF6	2.012617	6.38E-50
LYN	2.013046	1.94E-46
PLXDC1	2.013838	9.52E-45
IKBIP	2.014877	6.09E-52
ICAM2	2.015776	1.66E-51
FADD	2.016241	1.14E-54
PRELP	2.016395	3.35E-36

BPGM	2.016503	6.75E-53
SRP14	2.016568	1.8E-52
ENPP2	2.017009	4.33E-39
SPINT1	2.017237	1.94E-47
PDLIM5	2.017307	2.4E-51
TMEM37	2.01769	1.88E-44
EVA1C	2.01785	4.68E-50
SLC17A9	2.017871	6.06E-38
P4HA1	2.018739	8.51E-48
C1orf162	2.018805	3.07E-40
SPA17	2.018808	3.27E-55
MS4A7	2.018825	1.62E-38
PSMB2	2.018853	1.2E-54
NCF1	2.020337	4.9E-44
SAA2	2.020379	2.83E-25
MFSD6	2.020653	1.19E-53
DPY19L1	2.020671	9.05E-53
TSPAN18	2.020695	1.16E-49
PNMA2	2.021022	1.23E-49
PPP1R2	2.02173	8.35E-53
ZEB1	2.022001	9.01E-46
CDS1	2.022228	1.65E-53
MUC17	2.022308	6.12E-46
MPEG1	2.022598	8.06E-45
PKP3	2.023218	8.24E-46
RHOH	2.02378	2.66E-50
PTP4A2	2.024124	7.7E-53
OAT	2.024747	3.77E-50
VPS18	2.02488	1.31E-53
MVP	2.025528	4.79E-47
FLNC	2.025982	3.04E-49
HMCN1	2.026205	1.39E-46
MAGED2	2.026318	3.76E-52
PTGER2	2.026409	1.99E-50
APCDD1	2.026436	1.94E-43
BTBD10	2.026471	1.84E-54
GNA13	2.026549	2.34E-51
SVBP	2.026697	2.87E-52
LSM7	2.026781	7.08E-51
COL9A2	2.027918	3.51E-46
CBFB	2.028905	3.02E-52
CCRL2	2.02944	4.35E-49
TRAF2	2.029604	3.75E-53
DHCR7	2.029803	1.06E-52
PCNA	2.030782	4.02E-54
ALDH1A3	2.031392	1.64E-38
RASSF2	2.031938	2.38E-46
CHST14	2.033051	2.42E-51
GFPT2	2.033337	3.89E-37
PTPRR	2.033354	4.24E-55
EID1	2.033779	6.1E-52
PARP4	2.034338	1.08E-50
ASPHD1	2.034463	4.82E-47
PLEKHG6	2.034567	1.16E-49
PLSCR1	2.034581	7.96E-45
ARPC1A	2.03467	3.22E-55
AIG1	2.035239	3.02E-55
PJA1	2.035313	1.72E-53
GRB2	2.035962	1.62E-51

SLCO4A1	2.036342	4.86E-38
RAB5IF	2.036738	2.6E-55
DEGS1	2.036803	1.23E-52
FPR1	2.037011	5.72E-40
LMNB2	2.037493	3.24E-54
RPN1	2.037512	1.95E-55
HELZ2	2.037623	1.95E-50
ELOC	2.038218	4.85E-55
SNX8	2.038439	3.3E-52
C3AR1	2.038601	2.83E-42
JUNB	2.038626	3.3E-34
ZMAT2	2.039218	5.02E-54
CENPU	2.039402	1.02E-53
DYNC1H1	2.040002	6.43E-53
RAB5C	2.04004	3.43E-52
FAM20C	2.040351	6.87E-49
HKDC1	2.040804	5.33E-40
SULT1B1	2.042584	6.61E-49
PHC2	2.042624	3.21E-51
DNASE1L1	2.043264	1.01E-53
SLC43A2	2.04347	1.21E-49
GBA	2.043515	1.19E-55
MRPL17	2.043531	1.59E-56
SYNDIG1	2.044933	2.56E-54
RAB8B	2.0454	3.77E-50
CIAO2B	2.045857	2.18E-54
LETM2	2.046039	5.45E-53
DUSP7	2.046096	2.36E-52
PDIA3	2.046876	7.28E-56
ARL4D	2.047254	6.93E-50
CEMIP2	2.0473	5.59E-46
NR1I2	2.047413	3.32E-39
CTDSPL	2.047466	7.45E-53
ALDH1B1	2.047509	2.76E-52
ARHGAP15	2.047537	7.41E-49
SH3PXD2A	2.048576	1.17E-49
ARHGAP1	2.049352	1.79E-50
FKBP9	2.049592	1.02E-52
CYP4F3	2.049765	2.58E-50
SLA	2.05002	1.01E-39
APOBR	2.050256	6.29E-50
P3H1	2.050617	3.86E-50
NDUFC2	2.050821	6.15E-56
INPP5D	2.051698	5.34E-45
HSPA1A	2.051875	2.42E-22
LIX1L	2.05299	2.27E-49
IFIT2	2.053237	9.88E-51
LRCH1	2.053444	2.21E-52
HSPB1	2.053841	2.11E-35
BCL7C	2.053886	6.01E-54
FCHO1	2.054139	5.05E-53
DYRK2	2.054838	1.55E-52
FLYWCH2	2.055546	2.19E-53
BAG3	2.056379	4.32E-50
SAT1	2.056621	1.18E-38
CBR1	2.056967	1.9E-53
VMO1	2.0572	6.73E-53
IRAK2	2.058781	2.11E-50
MCM3	2.058813	8.39E-55

FLNB	2.059082	7.82E-47
ECSCR	2.059091	3.97E-49
OR2I1P	2.059467	2.18E-31
HLX	2.059554	9.69E-48
GPBAR1	2.059592	2.94E-49
ARFIP1	2.060212	7.85E-55
CD83	2.06141	5.3E-50
AKR1C2	2.063106	8.06E-33
CALHM2	2.063406	2.79E-47
SORL1	2.063814	1.92E-49
EPHA4	2.063888	1.03E-53
SPECC1	2.064044	2.68E-51
CXCL3	2.064456	5.38E-37
PLXND1	2.064599	1.43E-48
HIGD1A	2.065274	1.36E-51
MAGED4	2.06536	5.16E-44
CHMP1B	2.065446	4.17E-53
CSRP1	2.065447	7.08E-51
CLIP3	2.066221	1.8E-48
SKAP1	2.066524	2.12E-45
SNRPB2	2.066744	2.45E-54
CPD	2.066779	6.06E-54
MAP2K3	2.067588	7.76E-54
EBP	2.067674	6.12E-54
SNRPF	2.067857	1.4E-55
WDR54	2.068135	1.13E-53
RGS16	2.068436	3.2E-42
MAPRE1	2.069504	4.72E-53
CAV2	2.069851	2.59E-43
F12	2.070212	2.36E-48
FAM102A	2.070583	3.57E-50
SAE1	2.070587	9.83E-55
NEDD8	2.071635	4.65E-55
TYMS	2.072036	3.46E-52
SCARF2	2.072064	1.55E-44
SPAG5	2.072136	2.27E-54
HSPH1	2.072259	3.71E-48
TMOD3	2.07312	7.64E-52
LAT2	2.073121	2.46E-45
ATP6V1G1	2.073654	1.92E-53
TICAM1	2.073808	8.15E-53
LRRC42	2.075656	5.87E-54
USP18	2.075786	1.5E-51
SUMO3	2.076048	8.42E-53
TRAPPC2B	2.076346	1.25E-54
RSAD2	2.076484	4.88E-47
VEGFC	2.076808	3.02E-51
PMAIP1	2.077304	1.89E-50
SNRPD2	2.078188	4.21E-55
COPS8	2.078627	7.35E-52
TMEM33	2.078811	5.77E-55
UNC13D	2.078936	1.83E-39
NCAPH	2.079307	1.39E-55
GAL3ST4	2.079481	1.84E-50
TMEM150B	2.079746	1.92E-49
BACE2	2.081038	2.13E-41
LIME1	2.081124	1.48E-32
MCM2	2.081197	1.17E-54
ANP32A	2.081784	2.98E-53

ADA2	2.081873	3.67E-41
RAB7A	2.08223	4.14E-53
FGL2	2.083783	7.97E-46
LASP1	2.083868	8.92E-53
HSD11B2	2.084224	5.62E-38
P4HA3	2.084734	1.08E-48
SH3BGRL	2.085116	3.99E-50
TMC7	2.085536	6.15E-52
FIBCD1	2.085541	5.12E-56
PFDN1	2.086794	5.46E-53
PPIA	2.086913	3.24E-54
CHIC2	2.087001	4.6E-53
RAB23	2.087008	5.69E-51
DIO2	2.087214	4.52E-51
RND1	2.087653	1.67E-44
ATP6V0E1	2.087791	1.31E-53
TNFRSF12A	2.088255	2.74E-41
ZDHHC3	2.088525	3.11E-53
RESF1	2.088585	1.42E-50
LDLRAD4	2.08878	6.42E-51
SNX6	2.089075	1.59E-51
SERPINB9	2.089506	5.59E-50
NES	2.090243	3.43E-48
PTGR1	2.09072	2.13E-50
MXD1	2.091049	1.31E-50
STK24	2.091108	7.64E-54
SMIM15	2.091327	3.46E-54
CSNK1A1	2.092284	3.46E-53
MRPS35	2.092521	3.47E-56
VAMP5	2.092733	6.61E-52
ARL15	2.092794	5.76E-52
PROC	2.092795	1.19E-53
CLIC4	2.09312	2.6E-46
CCDC3	2.093444	1.56E-48
KIF20A	2.093461	2.05E-55
MAFB	2.093604	1.87E-41
CBR3	2.093967	1.58E-52
LSM4	2.094921	3.53E-55
STK38	2.095244	4.21E-51
SYT11	2.095255	5.64E-50
TRPV2	2.095851	9.85E-48
DBNL	2.096039	7.33E-53
GNG2	2.096295	6.02E-47
RAD21	2.09645	9.21E-53
HSD17B2	2.09651	5.5E-32
MAN2B1	2.096626	2.37E-53
ATP6V1E1	2.096897	2.55E-53
PLEKHA2	2.097111	2.39E-48
ELF3	2.09782	3.03E-36
SCP2	2.097887	1.95E-53
SCPEP1	2.099223	2.1E-47
PTPA	2.099527	3.19E-53
PPT1	2.099664	9.98E-52
COL6A1	2.099706	2.08E-40
GPC6	2.09976	1.06E-53
IER3	2.100199	9.65E-37
PRDX5	2.100269	2.09E-55
BLOC1S1	2.100954	2.68E-55
COL15A1	2.103933	3.62E-41

TES	2.1043	4.4E-49
LCP2	2.105157	2.15E-43
PRELID3B	2.106354	6.07E-55
HOMER3	2.106776	1.65E-45
PLOD1	2.106853	4.46E-53
NDRG1	2.106919	2.31E-48
MGST3	2.107388	1.51E-54
GNPDA1	2.107413	3.94E-53
HDGFL3	2.107581	3.21E-46
HVCN1	2.108234	6.98E-50
DRAP1	2.10839	1.07E-54
COX6B2	2.108513	1.44E-47
DPCD	2.108736	8.97E-56
SELENOF	2.108834	3.46E-53
ARHGAP3(	2.10897	3.79E-47
TMEM123	2.109047	2.7E-49
RTN2	2.109958	2.87E-52
CNIH1	2.110467	8.86E-54
CCDC88B	2.110588	1.23E-47
PSMA4	2.11065	7.9E-54
P3H2	2.111133	5.89E-46
MTCH2	2.112576	9.23E-54
SESTD1	2.112826	2.1E-50
CD163L1	2.113076	1.74E-53
PLEKHB2	2.113088	6.48E-54
STX1A	2.113109	6.85E-44
BUD31	2.114408	7.92E-55
GNG12	2.115045	1.2E-49
MDFIC	2.115126	9.79E-46
FBLN1	2.115853	4.36E-36
FYB1	2.116161	1.2E-47
CD7	2.116837	1.53E-50
SNRPG	2.116871	2.73E-55
B3GNT9	2.117442	8.34E-53
PLEKHA4	2.117724	3.89E-47
COA6	2.118089	1.89E-56
CLDN23	2.118303	5.48E-44
TTC9	2.118719	5.9E-50
HACD3	2.119079	8.82E-55
ADAM15	2.119087	5.18E-51
MELK	2.119381	6.24E-54
ADGRE5	2.119442	2.33E-46
RBMS1	2.11995	6.22E-46
PYGL	2.120755	5.25E-47
ROMO1	2.121227	4.81E-55
LPAR6	2.121683	1.16E-46
PDLIM1	2.121864	4.61E-48
HAVCR2	2.121882	2.54E-47
SLC44A1	2.122823	6.12E-53
CAPZA2	2.123075	1.32E-51
IL7R	2.123284	2.32E-38
PGF	2.123698	4.68E-48
APCS	2.1238	6.38E-13
S100B	2.124137	1.51E-41
BAK1	2.12427	6.37E-54
GNAI1	2.124389	2.01E-50
RPA3	2.12669	7.78E-56
RAPGEFL1	2.127194	3.22E-42
TMEM171	2.127487	4.03E-50

LIMK1	2.128348	4.86E-52
LIPG	2.129057	1.28E-48
RNF128	2.129587	1.41E-44
BMPR2	2.129662	3.43E-52
CTNNA1	2.129745	8.16E-52
RGS4	2.130225	3.68E-43
NET1	2.130507	1.5E-47
FEZ1	2.13061	1.73E-48
CHMP5	2.130834	4.9E-54
ERH	2.131267	5.32E-55
FZD7	2.132255	6.87E-50
GPR87	2.132721	2.61E-47
CCL4	2.133132	1.74E-45
PSMG3	2.133286	1.29E-54
NAT14	2.133647	1.93E-50
PAFAH1B3	2.133728	3.79E-54
F13A1	2.13439	1.23E-36
HCLS1	2.134776	2.24E-39
ARF3	2.135021	1.08E-52
COX6B1	2.135026	5.75E-56
ERAP2	2.135103	3.39E-40
MAT2B	2.135884	8.56E-52
PSME2	2.137449	1.6E-53
ATP5MG	2.137566	7.72E-56
CGREF1	2.138056	3.32E-52
IQGAP1	2.138869	1.23E-51
SECTM1	2.13894	7.1E-44
FAM177B	2.139543	5.28E-50
TMEM44	2.140352	1.09E-51
PPP6R1	2.14053	4.87E-53
TM2D2	2.140536	7.21E-54
IL4I1	2.140945	4.41E-52
WBP1L	2.14171	7.7E-53
AP2M1	2.142102	3.46E-53
COX7A2	2.14229	1.68E-56
NECAP2	2.142556	3.89E-51
MS4A4A	2.142698	1.4E-39
DNAJC15	2.145025	3.16E-53
PLA2G10	2.145867	8.08E-34
VGLL1	2.145896	5.23E-43
SRI	2.146164	6.33E-53
KIRREL1	2.14694	1.21E-46
PRDM1	2.147333	7.69E-51
FMNL1	2.147851	4.7E-43
GRK5	2.147939	7.64E-52
RBX1	2.148678	9.78E-54
IL1RAP	2.149883	3.87E-48
ORMDL2	2.150955	5.87E-55
ETHE1	2.151405	5.36E-52
VCAM1	2.151577	7.79E-36
CYB5R2	2.151785	1.57E-50
BLCAP	2.151956	1.42E-54
FEZ2	2.152325	5.6E-53
SLC40A1	2.152779	2.15E-45
TSKU	2.153415	1.99E-46
SHISA2	2.153626	2.81E-55
IFNAR2	2.154247	6.23E-53
NAB2	2.15449	1.28E-50
SEC23A	2.155243	9.67E-51

AOC3	2.15671	8.13E-44
NDUFB3	2.156737	3.94E-56
SH3BP5	2.157013	1.14E-47
ITGAV	2.157187	3.8E-47
VNN2	2.15735	1.07E-43
CTTNBP2N	2.157636	1.14E-51
PPP1CB	2.158417	1.07E-51
ETV7	2.159152	1.48E-52
DBNDD2	2.15925	1.38E-53
CD63	2.159476	1.71E-55
PLCB3	2.160309	4.08E-50
XAF1	2.160491	6.97E-37
KCTD5	2.160586	1.25E-53
RAMP2	2.160657	5.06E-49
NCEH1	2.161846	1.59E-49
SLCO3A1	2.162367	4.75E-48
IER5L	2.163048	3.11E-45
MATN2	2.163062	2.63E-47
SPTSSB	2.163621	3.39E-49
RASGEF1B	2.163662	1.21E-52
ADGRG6	2.163666	2.59E-46
TCN2	2.164961	1.32E-52
ARPC4	2.165722	7.51E-53
SUB1	2.166153	4.66E-54
HOXB5	2.166517	1.69E-52
C1QTNF3	2.166686	4.94E-44
DHCR24	2.166856	1.98E-42
ASAP2	2.167212	5.86E-48
CERCAM	2.167319	7.94E-47
TXNRD1	2.167339	5.36E-52
APOBEC1	2.167391	4.62E-54
ZYX	2.167447	1.87E-47
FAR2	2.167668	1.94E-55
CD27	2.168291	2.74E-49
BLVRA	2.168864	2.08E-52
CLTC	2.168944	1.23E-53
COL22A1	2.169084	1.7E-50
GSKIP	2.169597	6.17E-55
MLF1	2.169939	8.62E-53
UBE2N	2.170125	1.58E-52
B3GNT5	2.170272	1.15E-50
CRTAP	2.170447	1.04E-49
PECAM1	2.171365	7.69E-48
FNDC10	2.172051	2.91E-54
CHST11	2.172453	2.95E-45
CD109	2.172537	2.79E-52
C4orf3	2.172988	2.47E-53
LPCAT2	2.173635	7.39E-50
DCBLD2	2.173826	1.96E-46
VCL	2.173975	5.98E-50
COX7A1	2.174226	2.67E-48
CDC42EP3	2.175665	6.08E-51
UHRF1	2.176397	5.41E-55
CNIH4	2.177019	9.28E-55
SLC22A18	2.177253	9.78E-44
CSK	2.17767	1.01E-51
BCR	2.178559	7.41E-55
KIAA0930	2.178681	8.09E-52
PPP2R1A	2.178704	3.46E-54

LAMC1	2.178972	1.62E-48
STK10	2.179782	1.3E-51
TGIF1	2.179938	3.07E-46
PTPRCAP	2.180469	6.8E-46
POF1B	2.180844	2.43E-42
PARP3	2.181199	4.28E-51
B4GALT4	2.181607	4.57E-55
BSG	2.182266	1.29E-55
MRPL47	2.182679	2.75E-55
RHOA	2.18336	4.72E-53
CCL4L2	2.183389	3.57E-47
PTPRE	2.18351	7.1E-52
MRC1	2.183518	1.84E-40
SLC52A3	2.183806	9.05E-50
SLC22A18/	2.185239	9.47E-57
RAI14	2.185356	1.55E-49
PRR13	2.18559	3.7E-54
SLC25A24	2.18566	1.36E-50
UBL5	2.185805	1.76E-54
CX3CL1	2.186509	1.31E-45
APOL6	2.186593	5.11E-53
RAB7B	2.186855	8.73E-52
PDZD11	2.187354	3.2E-55
SOX4	2.18836	1.52E-47
SH3BP1	2.189531	2.01E-51
PSMC4	2.190531	1.16E-54
MMP23B	2.190889	1.65E-46
PFKFB3	2.190925	2.91E-45
PSAP	2.191148	2.42E-52
MEIS3	2.191191	1.46E-46
ALYREF	2.191215	3.17E-55
CD93	2.19173	6.48E-48
AP2B1	2.193512	2.84E-54
TNFRSF10/	2.194262	3.79E-48
CEP170	2.195028	5.47E-49
SMG9	2.195137	1.43E-52
GJB4	2.195345	2.44E-48
HYAL2	2.195867	4.67E-51
F2RL1	2.196084	1.26E-48
MYD88	2.196827	1.81E-51
TGFBR1	2.198004	1.48E-49
APP	2.19861	3.07E-52
ADTRP	2.19888	1.35E-51
CDKN2D	2.1993	2.72E-53
CDCA8	2.199333	1.08E-55
KIF2C	2.199976	3.57E-56
ARPC3	2.200428	7.9E-54
MGAT4B	2.201455	6.25E-52
PPP4C	2.201472	6.49E-55
CD86	2.202651	3.38E-50
ROBO1	2.203348	3.51E-47
ATP2B4	2.203972	8.69E-46
ACY1	2.204311	3.54E-50
CSF1R	2.204877	2.51E-41
PSMD14	2.205522	5.01E-55
MGST2	2.2061	2.4E-56
MCM6	2.207012	9.11E-56
PLEKHA6	2.207132	8.81E-50
CHN1	2.207277	9.83E-50

XRCC4	2.207435	1.21E-55
GADD45A	2.207942	7.14E-51
GLMP	2.208096	1.95E-53
ZNFX1	2.208805	8.29E-54
EXOC3L4	2.208948	1.98E-43
FOSB	2.209187	2.57E-27
SCARA3	2.20923	5.02E-46
YWHAG	2.210151	1.65E-53
FAM20A	2.210206	4.43E-45
DHFR	2.210244	9.02E-56
LHFPL6	2.21062	2.21E-46
TNFRSF1A	2.210625	1.15E-51
LIMD2	2.210738	1.45E-46
DEPTOR	2.210942	7.15E-54
CLDN11	2.21146	1.49E-46
GTF2A2	2.211997	2.07E-54
ATP5MF	2.212066	9.05E-56
GPR68	2.212496	2.18E-52
ITPR3	2.212864	2.37E-44
NDC80	2.214756	6.33E-56
KLF16	2.214901	3.56E-55
PGRMC1	2.215022	2.98E-54
LIMS1	2.215814	1.48E-48
DGUOK	2.216091	3.45E-55
PPP3CA	2.216123	6.82E-55
ETFB	2.216634	2.68E-55
LSR	2.2171	3.34E-51
REEP3	2.217884	6.29E-52
RTN4	2.218238	1.14E-51
LAPTM4B	2.219561	1.9E-53
SHH	2.221241	5.58E-52
BCAS4	2.221986	2.3E-54
CYP4F12	2.222002	5.16E-42
ACSL4	2.222466	4.15E-50
C5orf15	2.222866	3.97E-52
SMIM22	2.223221	1.11E-49
FYN	2.22345	5.1E-49
PXDC1	2.223799	2.24E-48
PTBP3	2.224559	1.89E-53
PSEN1	2.225191	4.74E-54
TIMP3	2.225341	3.86E-41
GSTO1	2.226341	1.73E-54
SDCBP	2.227036	1.16E-50
FOLR1	2.227478	2.5E-37
SHF	2.227566	1.11E-53
SCCPDH	2.228008	1.49E-55
C1QTNF6	2.22889	2.79E-49
CCND3	2.228901	6.56E-52
GCNT1	2.228913	4.96E-52
CANT1	2.229041	2.47E-55
CETN2	2.230125	1.26E-54
FADS2	2.230267	4.27E-45
AURKA	2.230721	2.54E-55
THBS4	2.231871	3.39E-34
ARHGAP26	2.232097	6.35E-49
ANKH	2.232408	4.72E-53
F2RL2	2.232424	1.31E-48
CALU	2.232562	1.96E-51
UBALD2	2.232772	3.27E-53

RALBP1	2.23344	5.31E-55
A4GALT	2.233936	7.28E-48
STX6	2.23399	3.1E-54
JAM3	2.23416	2.7E-48
LRRFIP1	2.234463	6.72E-52
NAPRT	2.234479	1.85E-43
GCHFR	2.235175	1.2E-53
PSME1	2.235986	7.9E-54
FAT1	2.236631	2.15E-49
MOB1A	2.237144	1.81E-51
PSENEN	2.237808	2.77E-55
POMP	2.238359	6.17E-55
MICU1	2.239069	7.29E-55
GJB5	2.239233	5.93E-44
VAMP3	2.240296	1.14E-52
GNB1	2.240929	2.03E-52
NFE2L1	2.241257	3.54E-53
HDGF	2.24126	2.75E-54
TAPBP	2.241287	6.65E-51
RHBDD2	2.241402	5.32E-54
ANP32E	2.242862	4.07E-53
RUNX2	2.242869	5.39E-52
GALNT10	2.244813	2.61E-52
ETV4	2.245693	7.46E-46
LRRC15	2.246098	9.75E-54
PCED1B	2.246287	1.5E-48
ST3GAL4	2.247369	1.8E-44
KIF26B	2.247556	1.75E-53
BOLA2	2.247861	6.02E-53
CAST	2.248283	5.63E-52
LRFN4	2.248536	2.11E-47
IFITM3	2.248557	5.14E-42
AKR1C3	2.248644	9.92E-43
PPIC	2.249386	3.85E-49
LYPD3	2.250032	9.51E-52
SMAGP	2.250679	3.62E-48
CERS6	2.250875	5.87E-55
DOK4	2.252028	8.84E-53
GNS	2.252487	4.35E-53
DBI	2.25294	9.46E-54
ERO1A	2.253488	2.86E-51
FAM83D	2.25357	5.45E-55
CDCA3	2.254552	1.13E-54
NCOA7	2.256472	2.2E-49
HSD17B14	2.256732	4.68E-48
OSTF1	2.257268	5.79E-53
CALR	2.257307	3.41E-54
PDLIM3	2.257454	4.29E-40
LAIR1	2.257746	6.99E-41
PTPRC	2.257866	1.3E-40
IQGAP3	2.257943	2.71E-54
MICAL2	2.258065	3.83E-50
LAYN	2.258113	4.04E-50
RBM8A	2.258678	2.23E-55
GRP	2.259266	2.05E-42
VWA1	2.259376	3.36E-45
CCDC74A	2.261948	5.05E-50
SLC9A3R2	2.26324	1.26E-49
ANXA11	2.263753	2.27E-51

DYNLT3	2.26385	2.51E-53
DNAJB1	2.26388	9.92E-47
CTNNB1	2.264735	1.98E-53
METRNL	2.264765	3.86E-47
RAN	2.265303	4.43E-54
ACSL3	2.265813	9.51E-55
AP1S1	2.266326	7.98E-56
SPRY4	2.267584	3.66E-53
GRAMD1B	2.268014	1.01E-51
PGAM1	2.268731	7.09E-54
OLFM4	2.268868	5.5E-13
CNPY4	2.269042	1.1E-51
CCND1	2.269309	2.22E-53
TWIST1	2.269469	8.22E-47
PRKACB	2.270413	4.2E-52
TRNP1	2.270891	1.88E-46
SNRPD3	2.270996	1.52E-55
DOK5	2.272159	3.15E-50
MS4A8	2.272621	3.3E-40
ELOVL6	2.272828	3.72E-52
HSBP1	2.273363	5.82E-55
NEDD9	2.274547	1.33E-50
OPN3	2.275713	4.34E-51
LIMA1	2.277489	1.85E-50
PSMD1	2.27824	1.26E-55
CCL3	2.278943	1.51E-48
MMD	2.279212	1.21E-50
MAB21L4	2.280228	7.65E-52
VDAC1	2.28123	6.94E-55
LAP3	2.281743	1.89E-51
CLEC2B	2.281948	6.05E-49
SGCB	2.282184	3.11E-53
MLLT3	2.282591	2.39E-53
CYB5R3	2.282658	1.8E-51
PDZK1IP1	2.282868	1.27E-41
BOLA2-SM	2.28426	5.9E-56
ANXA5	2.285261	2.18E-50
RCC2	2.285444	8.09E-52
TSHZ2	2.285591	9.54E-49
BNIP3L	2.285921	1.68E-52
PLEKHO2	2.286005	1.02E-49
RHOG	2.28768	2.37E-52
SSPN	2.287764	4.63E-49
DDA1	2.288159	1.25E-53
CYCS	2.28822	1.05E-54
ITPRIP	2.288268	3.25E-51
EPS8	2.289176	4.17E-48
ODAM	2.289631	3.13E-41
SPTLC2	2.290856	4.93E-55
TACSTD2	2.29324	2.56E-36
MSRB3	2.293843	7.87E-47
DOCK11	2.294183	2.02E-51
CMTM7	2.295121	4.9E-47
NOP10	2.29537	1.24E-54
CYBB	2.295674	3.31E-44
HHLA2	2.295725	2.98E-40
CDCA7	2.296145	1.41E-44
TROAP	2.296741	4.85E-53
YWHAH	2.299093	3.46E-53

CTSA	2.29991	9.06E-53
CHSY1	2.300013	2.82E-51
BICD1	2.300379	6.21E-53
RIN3	2.300777	1.05E-50
TSPAN2	2.301624	2.01E-48
ALDOA	2.302533	6.44E-53
PCOLCE	2.303221	5.57E-44
PLA2G7	2.30361	1.15E-52
ARL6IP5	2.30451	6.76E-53
CDC42	2.304522	4.04E-52
TMCO1	2.304555	4.55E-56
EPB41L1	2.30528	7.96E-52
SLAMF7	2.305453	2.35E-51
ST14	2.30547	8.63E-49
AKAP12	2.305715	2.3E-43
MUC12	2.305786	3.93E-55
LIPA	2.305818	3.82E-51
DACT1	2.306338	5.41E-48
HNRNPAB	2.306749	5.14E-55
EFNA4	2.307126	9.53E-47
SLC24A3	2.307787	2.38E-54
MFAP4	2.309202	2.58E-39
FPR3	2.309209	5.57E-47
SIPA1	2.311116	7.92E-51
GSTP1	2.311176	7.76E-47
BCL10	2.3113	1.34E-53
ARRDC2	2.311961	7.52E-50
GPI	2.31202	2.66E-54
KRT8	2.312347	1.69E-46
PTHLH	2.312395	2.63E-52
ITGB1BP1	2.312553	3.27E-53
TAP2	2.314619	6.13E-51
SUMO2	2.316044	9.67E-55
IFITM2	2.316182	8.89E-43
STYK1	2.316732	5.84E-55
MFGE8	2.316811	2.35E-46
PRNP	2.317094	4.56E-51
MEI1	2.317427	7.03E-52
UGT1A6	2.317891	1.18E-50
OSBPL3	2.318349	2.43E-47
ADAM10	2.318402	4.02E-50
PIM2	2.320155	2.4E-52
PPY	2.321835	1.01E-09
PPP1R14C	2.322467	2.93E-52
QSOX1	2.322519	4.76E-53
HSD11B1	2.324203	5.61E-47
TAX1BP3	2.327034	6.44E-51
TPI1	2.329251	1.18E-54
CXCL13	2.329678	5.14E-44
CTNNBL1	2.330129	3.84E-55
RHPN2	2.330265	1.02E-45
THBS1	2.33053	8.54E-33
TSPAN5	2.331823	5.29E-54
HMCES	2.331837	2.51E-55
SLC1A5	2.333385	1E-48
ALAS1	2.334292	4.21E-55
KDELR3	2.33591	1.13E-50
ANXA3	2.336672	4.35E-42
NT5DC2	2.337171	3.61E-51

PBXIP1	2.337803	2.29E-54
ATP6V1D	2.338058	6.81E-54
VAV1	2.338739	7.17E-49
CD47	2.339111	4.38E-52
FGD5	2.339293	1.38E-51
KCTD17	2.340032	1.62E-51
TPSAB1	2.341919	1.86E-41
ZFP36	2.342297	1.06E-40
RAB25	2.343176	9.6E-51
RACGAP1	2.343209	2.61E-55
NKG7	2.34414	9.73E-51
RENBP	2.344492	4.94E-49
TRIM69	2.344595	1.67E-51
FAM214B	2.344672	2E-53
TMEM50A	2.345223	3.7E-54
IFT172	2.346095	1.12E-52
AK1	2.346201	1.49E-54
APOBEC3G	2.346365	9.57E-52
PAM	2.34708	1.65E-50
MYO1A	2.34799	3.26E-48
PGD	2.348722	3.4E-53
CFB	2.348743	3.09E-34
SLC16A5	2.349616	1.03E-44
PHF19	2.350583	7.21E-53
SLC39A1	2.350762	2.32E-53
DYNLL1	2.350913	1.18E-54
GSS	2.351653	1.11E-55
CAPN2	2.352295	5.87E-54
NEK2	2.352452	1.47E-56
CDCA5	2.352862	2.47E-56
MAP1S	2.35371	5.59E-55
IL13RA1	2.353853	4.15E-54
SDC3	2.354422	1.58E-51
SLC12A2	2.355102	1.62E-49
WFDC3	2.357741	2.38E-47
ACVR1	2.3586	2.18E-52
CARD19	2.359595	6.87E-53
PROS1	2.35974	1.11E-51
TBCB	2.363304	8.71E-54
ITGA1	2.364014	3.16E-50
C3	2.366938	9.79E-29
NDUFA13	2.367244	1.45E-56
C1orf116	2.367502	7.77E-52
TUBB4B	2.367699	7.27E-56
MSMO1	2.36776	2.15E-51
SHISA5	2.371552	7.27E-54
HRH1	2.371784	6.98E-56
AGT	2.371951	1.3E-39
SLC22A3	2.372255	4.57E-50
HOXB2	2.373487	1.6E-49
TUBB	2.373865	6.23E-53
ASPHD2	2.374345	3.23E-56
PTGFRN	2.37652	2.79E-49
HENMT1	2.377495	1.09E-53
SRD5A3	2.378463	5.97E-54
TMEM159	2.378674	1.1E-50
RAP2B	2.378812	7.1E-52
MRPL14	2.379457	1.52E-56
IGFBP6	2.381036	8.25E-44

MYL6B	2.381612	1.72E-53
KIFC1	2.381908	4.45E-55
PPP1R15A	2.38199	4.94E-50
CALML4	2.382023	4.98E-51
PPL	2.382745	1.76E-48
SLC37A1	2.386291	4.89E-54
GM2A	2.38684	1.18E-52
ARHGAP27	2.387037	2.2E-48
COX7B	2.387322	1.57E-56
GBP1	2.387356	2.07E-45
CSF2RA	2.387943	1.02E-48
ELOVL5	2.388077	4.22E-50
LMO4	2.388204	1.87E-49
MX2	2.390535	4.04E-47
EMILIN2	2.390877	3.57E-52
BIRC5	2.391377	5.62E-52
CD2	2.391802	3.6E-49
ATP2C2	2.392351	3.23E-49
BHLHE41	2.392796	7.11E-50
RAB32	2.393785	5.83E-49
VIM	2.394951	7.19E-47
TFAP2A	2.395126	2.35E-53
FUT2	2.395225	9.96E-45
ARHGAP23	2.395896	4.79E-49
DSG2	2.395965	4.38E-51
FRZB	2.397772	1.36E-49
MYH11	2.398873	3.56E-38
NOTCH3	2.398921	4.68E-47
ADAM12	2.399967	6.48E-51
RGS5	2.400658	8.36E-49
WNT10A	2.400892	6.72E-55
MPZL2	2.401598	5.32E-42
TNFSF10	2.40245	3.96E-50
PTMS	2.402638	3.56E-51
FRMD6	2.402648	9.18E-47
SOD1	2.403931	4E-55
PRAF2	2.404096	2.31E-52
ADGRA2	2.405426	8.57E-47
TFPI	2.406691	5.49E-44
RMI2	2.407617	1.33E-54
SNRPB	2.408122	4.11E-55
PLBD1	2.408653	3.5E-43
BST1	2.408865	1.17E-49
CMIP	2.408873	1.58E-52
SDS	2.409882	1.25E-49
SOCS3	2.412117	1.37E-39
ADAP1	2.414243	2.39E-45
RARA	2.415368	1.23E-51
LPAR5	2.415407	4.78E-55
UPP1	2.415918	2.48E-51
RARG	2.416459	4.24E-51
NAGA	2.416892	1.99E-54
MR1	2.417574	8.42E-54
HCK	2.41854	7.86E-48
TPSB2	2.418725	1.15E-37
SUMO1	2.421027	1.89E-55
STOM	2.422182	1.3E-50
GNG10	2.423164	1.18E-53
SAA1	2.423507	1.05E-27

MAD1L1	2.423641	1.84E-53
SKIL	2.425078	1.45E-50
WNT7B	2.426305	1.08E-51
GPR153	2.426724	7.23E-54
ARL14	2.428708	3.46E-37
PPDPF	2.429014	2.31E-51
ENDOD1	2.429398	7.57E-53
STMN2	2.429549	2.68E-44
NMI	2.430882	1.05E-52
FGFBP1	2.431125	2.59E-44
MMEL1	2.433246	2.02E-44
UBC	2.435371	5.18E-55
CHMP4B	2.435522	3.22E-55
ZNF385A	2.436106	5.05E-51
ENTPD2	2.436583	4.44E-51
SPRR1B	2.437263	6.96E-23
SELENOW	2.438107	6.07E-55
APOL2	2.438256	2.54E-52
BPIFB1	2.439959	1.07E-46
ALDH2	2.440175	1.07E-51
MYL6	2.440607	1.98E-53
RNF145	2.441581	2.38E-52
ATP6V1F	2.441752	7.9E-54
STC1	2.442146	1.05E-47
PHLDA3	2.44295	5.93E-50
APOC2	2.443119	1.64E-49
SLC4A11	2.445108	5.61E-55
SELL	2.445177	2.03E-50
CALHM6	2.44526	4.69E-51
C1S	2.445261	1.44E-41
ELOB	2.445376	3.47E-56
ITM2C	2.445672	9.16E-54
CYB5B	2.448217	1.04E-54
C1GALT1	2.449271	4.53E-55
ETV1	2.451387	4.58E-53
PTGS2	2.451504	5.24E-53
B3GALT4	2.45287	3.8E-55
TMEM200A	2.453955	1.22E-49
BPNT1	2.454374	7.64E-56
ITGA5	2.454674	1.15E-46
PLPP2	2.458339	7.84E-45
IFI44	2.460756	2.47E-42
ZNF185	2.462689	1.6E-51
SLAMF8	2.462848	7.5E-50
ESPN	2.464906	7.57E-50
EFNA3	2.465084	4.16E-55
IRF1	2.465702	4.64E-49
EFEMP2	2.466073	4.2E-47
RPL39L	2.466121	2.76E-49
NFKBIE	2.467187	3.02E-52
ADAMTS12	2.468353	2.07E-50
EPCAM	2.468427	5.97E-54
CMTM6	2.468558	2.54E-52
IDO1	2.468919	2.51E-53
CCL24	2.470567	7.31E-55
CEP55	2.470577	5.64E-56
PRRX2	2.470794	1.47E-51
EVI2A	2.471069	9.44E-49
AP1S3	2.473083	8.01E-52

MAD2L1	2.473972	1.16E-56
AFAP1	2.474304	4.7E-52
JAML	2.475737	1.9E-45
BZW1	2.476881	6.59E-53
PML	2.477497	1.09E-52
TMEM158	2.478209	8.67E-53
ESM1	2.47876	5.05E-54
TINAGL1	2.479725	1.28E-45
GASK1B	2.480351	2.58E-50
SLC7A5	2.481115	2.13E-44
A2M	2.482667	2.2E-49
ST6GAL1	2.485698	1.26E-47
ASPH	2.485991	2.2E-51
CYP1B1	2.486168	7.24E-41
CORO1C	2.486995	2.71E-51
C1QTNF1	2.48704	1.03E-49
RNF19B	2.487401	5.03E-53
IRF8	2.487538	3.5E-49
CPXM2	2.48782	2.79E-47
FUCA1	2.487981	1.3E-54
TJP3	2.488683	2.3E-43
FOXS1	2.488759	1.69E-52
PROCR	2.490333	1.29E-51
GIMAP2	2.490857	1.98E-50
CST7	2.491089	1.95E-50
EMP2	2.491357	3.82E-52
KCTD12	2.491602	2.46E-50
WNT2	2.492451	4.13E-50
HTATIP2	2.49321	1.03E-54
SLC9A1	2.495167	4.12E-54
FUT6	2.495197	7.15E-45
BCL2L1	2.495517	3.09E-55
MUC20	2.495583	2.71E-40
STAT1	2.496506	5.1E-50
CAPN9	2.496852	3.11E-51
CLDN4	2.498485	3.82E-42
ZDHHC20	2.498516	9.98E-55
RALB	2.498579	1.77E-54
FOXM1	2.499527	1.35E-54
INF2	2.500388	3.35E-50
LOX	2.50116	5.22E-45
EVI2B	2.501793	5.28E-47
ARNTL2	2.501968	3.5E-54
CD300A	2.502548	1.77E-50
DBN1	2.50304	4.94E-50
EHBP1L1	2.503669	5.48E-50
PHLDA1	2.504455	4.63E-52
CDCP1	2.505801	5.23E-52
TRAK1	2.507195	3.06E-54
YWHAB	2.510395	3.64E-54
SULT1A4	2.512752	2.34E-50
CHIT1	2.513154	7.09E-44
DYNLT1	2.515605	5.23E-54
GZMA	2.516371	5.08E-52
FOLR2	2.517492	1.16E-47
TWSG1	2.518544	2.7E-53
DYNLRB1	2.518833	6.69E-56
CAPNS1	2.51919	7.29E-55
IFIT3	2.519227	7.46E-50

IFIT1	2.519279	1.19E-45
TNFRSF1B	2.519284	1.83E-50
DAPP1	2.520001	1.06E-50
WIPF1	2.520483	2.32E-48
HIF1A	2.521108	1.02E-49
RALA	2.521285	1.96E-54
MCU	2.521294	2.18E-54
TXN	2.521531	9.36E-56
PDGFRB	2.521963	2.7E-46
ACTN4	2.523055	8.22E-53
PSORS1C1	2.525114	8.25E-49
SPINT2	2.526039	4.65E-55
NPC1L1	2.526483	5.88E-48
SMYD3	2.527513	5.28E-56
GALNT4	2.52855	2.47E-47
IHH	2.529665	3.24E-54
MELTF	2.529918	1.66E-45
CXCL10	2.53009	1.78E-48
PHACTR3	2.532024	6.9E-49
ZNF703	2.533068	4.93E-54
WDR1	2.534539	2.08E-52
PFDN2	2.535337	2.2E-55
ITGB6	2.535576	4.65E-41
ETS2	2.537131	1.05E-49
IFI44L	2.537367	2.35E-43
TFPT	2.537629	1.25E-54
ITM2B	2.538466	7.41E-55
FTL	2.539147	3.24E-53
CDC42EP2	2.539208	1.96E-53
ALDH3A1	2.539217	1.31E-46
SPP1	2.54166	2.02E-28
MYL12A	2.541983	4.09E-50
FKBP7	2.542255	8.41E-53
CASP1	2.542522	3.15E-48
PIGY	2.542537	4.55E-38
PIEZO1	2.543209	3.41E-47
CRISPLD2	2.543363	1.05E-44
LILRB4	2.543676	1.1E-47
ACTR3	2.544079	5.44E-51
VSIR	2.544693	9.07E-51
SDC4	2.545983	3.38E-48
GPR183	2.548581	8.49E-47
NPC2	2.549055	7.27E-54
HCFC1R1	2.550251	1.19E-53
ACOT7	2.550563	1.67E-55
LY86	2.551647	4.63E-49
MAOB	2.551815	5.59E-48
SLC1A1	2.553749	8.25E-50
UNC93B1	2.554858	4.07E-53
MTPN	2.555233	1.74E-53
SLC2A3	2.555843	1.08E-44
LRRC8A	2.556276	1.18E-53
CD276	2.556742	5.04E-53
OSBPL10	2.557135	8.72E-55
PSMB3	2.557384	3.81E-55
EIF6	2.557428	4.18E-56
RASAL1	2.558875	1.05E-47
PTAFR	2.559676	6.73E-51
ABRACL	2.559836	5.52E-50

RTP4	2.561523	6.42E-53
PLEC	2.561981	1.9E-51
KLF2	2.563227	6.43E-48
CDK2AP1	2.563829	7.45E-54
HSPG2	2.565056	5.27E-49
C12orf75	2.565457	1.8E-48
CXCL9	2.565649	1.23E-50
ASF1B	2.565828	2.56E-56
ACE	2.566195	3.63E-54
ARHGEF2	2.566352	3.88E-52
C5orf46	2.566586	1.62E-44
PAQR8	2.567263	1.93E-55
GLIPR1	2.567581	3.08E-47
APOBEC3C	2.568273	4.56E-50
ETS1	2.568493	2.18E-50
NBDY	2.571347	4.66E-56
SCIN	2.572365	3.69E-53
BAX	2.572481	8.9E-55
ATOX1	2.573859	1.07E-54
AURKB	2.575588	1.26E-53
PTGIS	2.575707	5.56E-44
UNC5B	2.576412	8.83E-53
SULT2B1	2.57669	3.97E-51
BCL2A1	2.577054	4.86E-49
MPZL1	2.57712	1.72E-53
LXN	2.579333	3.6E-52
SSC5D	2.579628	1.41E-51
LMCD1	2.580488	2.62E-50
CD59	2.580655	3.21E-54
BHLHE40	2.580874	9.64E-44
PDLIM2	2.581326	2.29E-51
SHROOM3	2.584258	1.56E-53
SCEL	2.584869	6.19E-47
ATP1B3	2.586561	1.03E-52
CYP27A1	2.587023	6.35E-52
ALDOC	2.589888	3.51E-53
SAMD9	2.591611	1.44E-53
ARL4A	2.591666	1.46E-54
LMNB1	2.591753	7.08E-54
LAD1	2.593628	4.33E-45
TPM3	2.594937	1.97E-54
PFN1	2.59723	6.59E-53
CACNB3	2.597674	3.84E-53
RAB11A	2.597744	8.61E-55
ATP1B1	2.598443	1.68E-53
SPON1	2.599479	4.84E-45
ADAM19	2.601172	4.39E-51
CCN2	2.60155	6.85E-41
B4GALNT3	2.602821	1.51E-52
FMOD	2.603458	1.94E-50
CES1	2.605765	2.12E-40
APLP2	2.60683	6.37E-54
RNASET2	2.608555	3.87E-55
HMGB2	2.609641	2.22E-52
ARPC2	2.610512	2.19E-53
NDE1	2.610669	1.59E-53
CTSH	2.611541	2.3E-46
S100A13	2.61237	2.13E-52
MCUB	2.612384	1.41E-51

SPI1	2.612705	1.21E-46
ARF6	2.61376	3.21E-54
ACTN1	2.615517	2.16E-51
ACTR2	2.617218	1.26E-52
LMO7	2.617472	1.36E-52
MAP7D1	2.617508	1.08E-52
SMS	2.618749	3.12E-55
CCL15-CC	2.619301	4.04E-47
ANXA10	2.61943	5.31E-25
FABP3	2.619545	2.21E-49
EGR2	2.620846	1.74E-51
EVPL	2.621181	1.28E-45
HLA-E	2.621679	2.97E-52
FOXQ1	2.623256	1.54E-47
TUBA4A	2.624288	1.15E-54
NUSAP1	2.626416	5.32E-53
GPX4	2.627328	4.29E-54
PKMYT1	2.627597	3.96E-55
EFNA5	2.627975	1.46E-54
RNASE6	2.628809	1.03E-47
PLS3	2.629118	1.9E-51
CTSD	2.629397	5.82E-54
MSR1	2.63103	1.49E-47
CDKN2A	2.63125	6.06E-47
SPINK4	2.631617	2.59E-40
AC011462.	2.631752	1.08E-49
TPPP3	2.632167	7.67E-49
GPR137B	2.633543	4.95E-53
EHD1	2.634095	2.68E-54
GLRX	2.634783	4.38E-52
CD37	2.635155	3.38E-45
BID	2.636233	4.38E-52
ZWINT	2.638795	2.74E-56
SLC39A4	2.639796	6.02E-49
FCGR2B	2.642094	1.35E-43
TNFRSF11E	2.642524	4.15E-48
CAV1	2.64334	2.36E-49
CDHR5	2.643653	1.66E-44
AHNAK	2.64495	5.14E-51
CCN4	2.646685	2.53E-50
ATP6AP2	2.647469	3.38E-54
PLK1	2.647681	4.2E-55
ADAMTS2	2.648498	2.13E-48
THEMIS2	2.650131	1.21E-48
STEAP1	2.650964	3.58E-49
ATP5F1E	2.651099	3.78E-56
NUDT1	2.651211	5.59E-55
RAC1	2.652197	4.14E-55
GBP4	2.653145	1.04E-52
CD4	2.655365	8.82E-49
EPS8L1	2.655426	1.25E-40
C1R	2.657564	1.22E-44
BCAR3	2.657701	9.82E-55
NOX4	2.658137	6.55E-52
NECTIN2	2.658435	1.95E-55
TSC22D1	2.658665	6.33E-51
CD3E	2.659481	1.81E-48
GGT5	2.659755	3.14E-49
TRIM16	2.660788	2.35E-54

PIK3IP1	2.661096	1.36E-51
CALB2	2.66196	1.7E-50
PTTG1IP	2.662818	1.43E-54
GLT8D2	2.663318	4.78E-51
TOR4A	2.663501	1.07E-52
MMP12	2.66363	1.68E-57
SERTAD1	2.665256	1.68E-55
SLC16A4	2.665308	1.28E-53
MYLK	2.666397	4.23E-45
ID1	2.668218	7.82E-47
HLA-DOA	2.668293	7.77E-49
AK4	2.668797	2.58E-53
MAPK3	2.668905	9.51E-56
CCL17	2.669076	4.11E-51
NECTIN4	2.669362	8.18E-44
LDLR	2.670683	1.44E-45
FA2H	2.671488	5.34E-48
DPYSL2	2.672141	7.51E-54
KLF4	2.672494	3.76E-51
SEM1	2.672821	1.83E-56
CD58	2.673144	4.78E-52
CDKN2B	2.673766	5.09E-49
PTPRH	2.674426	2.38E-49
PEA15	2.675153	6E-52
ANLN	2.677753	5.6E-55
MYL12B	2.678418	1.42E-54
SNCG	2.678649	4.19E-46
CSTA	2.679354	3.84E-44
LCK	2.679547	1.28E-50
ECT2	2.680977	3.49E-55
LITAF	2.683789	1.43E-50
CORO1A	2.684432	9.93E-48
TRIM14	2.686038	4.43E-54
IFI35	2.688115	3.21E-54
INPP4B	2.6882	9.96E-53
NCF2	2.688269	5.07E-45
MYO7B	2.688289	5.42E-44
MUC1	2.688595	1.35E-39
PMP22	2.689048	5.18E-49
VOPP1	2.689696	1.09E-54
DUSP5	2.690129	3.22E-50
CD3D	2.690174	1.96E-50
BOLA2B	2.694025	1.37E-56
MFAP2	2.695906	5.56E-43
SLC9B2	2.696044	2.07E-51
ELF4	2.696601	5.81E-54
TSPAN3	2.697561	9.05E-55
CALM2	2.701122	5.77E-54
TMEM132A	2.704036	4.75E-51
TNFAIP2	2.704359	6.07E-41
DDX60	2.70639	3.52E-52
MAP4K4	2.706654	8.86E-54
CELSR1	2.706805	5.69E-52
METTL7B	2.708106	6.53E-51
SRPX2	2.709051	8.5E-47
GLIPR2	2.709399	4.43E-49
FLNA	2.710384	1.54E-45
PGK1	2.716224	2.96E-54
KRT15	2.717551	4.34E-46

HOXB6	2.718987	4.33E-53
TMPRSS3	2.719595	5.01E-47
MAFF	2.719813	1.4E-51
HLA-DQA1	2.720342	4.16E-48
CENPM	2.722417	4.73E-53
CP	2.723804	1.57E-32
SNRPD1	2.728721	1.5E-56
SPOCK2	2.728757	5.91E-53
PTK7	2.729942	1.13E-50
MFSD2A	2.730273	1.13E-55
TGFB1I1	2.732425	3.21E-49
B4GALT5	2.733206	3.54E-53
OAS3	2.735577	1.82E-52
LRP1	2.73563	9.99E-51
TAP1	2.735852	5.02E-51
OLR1	2.736214	3.1E-42
SQLE	2.736844	1.74E-53
CLEC11A	2.737213	5.47E-50
SH3BGRL3	2.738712	1.68E-53
FKBP10	2.739465	9.53E-50
FCGR1A	2.73963	1.4E-48
ABLIM3	2.741203	1.07E-51
BIRC3	2.74139	1.56E-41
ABCG1	2.741463	1.6E-53
ELK3	2.741481	3.38E-52
TBC1D2	2.741862	2.4E-54
IFITM1	2.743944	1.27E-46
TNFAIP6	2.745306	5.56E-52
ARPC5	2.74716	8.43E-54
IL18	2.749946	1.09E-46
ACTG2	2.750586	7.87E-42
ENTPD1	2.751749	6.21E-53
GIPC1	2.751778	3.87E-55
KRT6B	2.751964	4.14E-40
TWF2	2.753185	1.72E-53
LPCAT4	2.753748	1.05E-52
KRT6A	2.754472	1.89E-25
TYMP	2.754941	1.32E-44
APLNR	2.756586	1.34E-49
MET	2.757794	2.08E-47
PLIN3	2.759277	2.61E-52
GNAI2	2.759485	2.15E-49
LTBP2	2.760947	1.77E-48
CD9	2.762079	7.06E-50
TACC3	2.762277	7.04E-55
APOC4-AF	2.763229	1.13E-49
PNP	2.763444	7.15E-54
LDHA	2.768007	2.23E-47
CCNB2	2.769189	8.07E-55
CYBRD1	2.769908	9.92E-47
HMOX1	2.773095	1.22E-49
PXDN	2.774222	1.21E-50
NINJ2	2.775026	5.3E-52
CD14	2.775592	1.96E-49
DNTTIP1	2.775621	1.42E-55
JUP	2.776118	1.12E-51
PRR5L	2.782113	3.54E-52
CD79A	2.782565	8.26E-48
CEACAM7	2.783346	1.09E-32

GALE	2.784562	1.19E-51
GDA	2.786773	7.8E-50
CKS1B	2.789286	5.24E-56
CGB5	2.790357	5.59E-52
DDAH2	2.790685	4.31E-51
PCDH7	2.79326	7.61E-54
CDR2L	2.79424	6.37E-54
GPX8	2.796136	1.07E-49
CKMT1A	2.796646	5.53E-52
MSRB2	2.797794	2.93E-54
RARRES1	2.798528	1.61E-40
ANXA13	2.800763	3.9E-54
VDR	2.801892	1.94E-53
HCST	2.802582	2.3E-50
PERP	2.803406	5.18E-55
SLC5A1	2.805569	1.06E-49
LY75	2.807035	6.46E-51
TSPO	2.808173	3.09E-47
TIMP2	2.808193	3.07E-50
IL1R2	2.808266	1.18E-47
LGMN	2.808524	1.5E-53
SPATS2L	2.810328	4.31E-53
RFTN1	2.81434	9.01E-50
CTSZ	2.816671	5.07E-52
LY96	2.817183	1.68E-49
PYGB	2.817313	5.38E-56
HBEGF	2.819808	5.69E-53
PHGR1	2.819916	3.17E-31
SKAP2	2.820144	4.95E-53
SLC16A1	2.820188	1.78E-55
GBP3	2.820643	3.6E-47
CRIP2	2.821402	1.12E-50
CKMT1B	2.82232	1.07E-51
CD248	2.822506	3.71E-47
ICAM1	2.822607	3.6E-46
CAPZB	2.822754	4.42E-53
MUC16	2.822926	1.54E-43
FCGR2A	2.82355	1.33E-46
CENPW	2.823583	8.1E-55
PPP1R18	2.824444	5.88E-49
EZR	2.824603	1.27E-54
TPM2	2.830239	1.26E-49
CDKN3	2.830492	3.52E-56
AXL	2.830745	1.36E-50
PCDH1	2.83139	7.64E-54
AL136295.	2.832254	2.1E-49
ARL6IP1	2.832274	2.13E-54
COL6A2	2.833175	6.46E-46
HS3ST1	2.833437	1.29E-55
TK1	2.835108	1.82E-51
CEMIP	2.835767	2.75E-54
SERTAD3	2.836136	6.36E-56
CCL21	2.836319	1.02E-31
IGFBP4	2.836465	1.68E-50
FOXL1	2.839659	5.45E-56
PDPN	2.840276	1.91E-46
UBE2L6	2.843205	5.81E-52
EDNRA	2.847147	3.01E-51
KPNA2	2.848061	7.33E-56

RHBDL2	2.84978	3.62E-54
PRDX1	2.849787	2.22E-54
RND3	2.849917	6.12E-53
PRRX1	2.850651	1.12E-45
CRYBG2	2.850742	1.99E-54
MKI67	2.851308	2.37E-55
VNN1	2.852177	3.67E-46
PLS1	2.854238	1.73E-50
CALD1	2.855456	2.3E-47
GMFG	2.855474	2.5E-50
MS4A6A	2.857633	1.92E-44
PRXL2B	2.857718	9.35E-56
CPVL	2.858361	3.88E-49
CD48	2.858669	1.8E-49
LRRC32	2.859788	2.44E-51
HK1	2.860768	1.34E-53
MGP	2.863915	2.46E-46
ENO2	2.868359	2.01E-51
CDH17	2.869297	1.44E-50
CD53	2.870338	3.76E-45
ALDH3B1	2.875395	1.44E-48
RHOF	2.87579	4.48E-52
IFNGR2	2.876669	5.45E-54
ADAM8	2.878241	2.08E-48
ASS1	2.878851	8.93E-54
MUC4	2.881118	2.99E-38
PPP1R14D	2.883303	5.33E-55
SLC7A7	2.884269	2.13E-50
CXCL8	2.887089	2.32E-36
PODNL1	2.890647	3.91E-50
CLTB	2.891062	1.4E-55
CYP2C18	2.891217	7.13E-50
TMEM54	2.891221	8.54E-55
ITGA11	2.892944	4.32E-51
CCNB1	2.900869	1.14E-56
GJA1	2.900902	1.12E-50
SPON2	2.901704	2.15E-47
RCN1	2.902788	1.02E-54
TAGLN2	2.903597	3.7E-54
COL8A2	2.904238	5.48E-50
TMEM119	2.904841	1.46E-46
CRISP3	2.90486	3.98E-25
CLRN3	2.906199	3.26E-54
ACP5	2.906262	2.63E-48
HLA-DQB2	2.907162	3.53E-52
CAP1	2.907335	1.02E-52
SEC14L2	2.909792	3.84E-55
HLA-C	2.913586	1.25E-53
BAIAP2	2.915732	2.53E-55
MYADM	2.925882	2.06E-52
ENO1	2.927013	4.98E-54
PLXDC2	2.927064	2.79E-50
DCN	2.931619	1.88E-44
INAVA	2.937195	4.53E-45
IGFBP7	2.937556	2.05E-49
HAPLN3	2.938471	7.87E-50
YWHAZ	2.94174	1.37E-54
RHOC	2.945825	1.23E-53
TMSB4X	2.94873	6.86E-51

GPX1	2.949667	8.86E-54
TMEM265	2.95206	1.04E-52
PTK6	2.955331	7E-45
SGPP2	2.957745	1.48E-51
CD82	2.965011	1.56E-54
MX1	2.965178	1.94E-49
LOXL2	2.96839	6.94E-52
PDE4C	2.968559	3.06E-42
C1orf54	2.969125	1.4E-51
EMILIN1	2.970278	2.08E-46
RRAS	2.971142	8.29E-53
SYTL2	2.973015	2.13E-50
SLC45A3	2.973707	1.21E-52
MARCO	2.978151	4.97E-42
EPHA2	2.97874	5.33E-47
CAVIN1	2.979402	3.01E-51
FBXO32	2.985944	8.65E-51
MAL2	2.987246	1.29E-53
FERMT3	2.987299	8.58E-51
CYSTM1	2.990384	3.97E-56
CENPF	2.99401	2.54E-53
RRM2	2.998355	2.85E-54
CNN1	2.998788	1.68E-47
ANTXR2	3.002157	2.55E-48
TUBA1A	3.002649	6.47E-50
ANXA8L1	3.004356	6.69E-45
AMIGO2	3.004754	2.06E-52
FCER1G	3.007432	1.55E-46
TM4SF1	3.016751	2.03E-44
GSN	3.017843	4.01E-53
GOLM1	3.018116	7.05E-55
TRIM15	3.019233	1.94E-51
MOXD1	3.019778	4.48E-50
TUBA1C	3.022935	8.72E-51
ITGB1	3.028255	5.72E-52
TBXAS1	3.029391	9.51E-53
CDK1	3.031728	2.17E-56
PSMB8	3.032113	2.82E-53
PADI1	3.0322	3.45E-48
TUBA1B	3.032903	1.22E-52
PCLAF	3.035085	3.74E-55
CYBA	3.036344	4.07E-47
MSN	3.03933	7.57E-48
TPX2	3.040298	6.89E-57
LSP1	3.041275	2.27E-47
FUT3	3.043168	8.9E-45
GALNT5	3.045451	8.03E-54
MYH14	3.045652	6.28E-53
QPCT	3.046874	1.28E-51
KRT23	3.05332	4.6E-42
FAM83A	3.053928	2.35E-50
RCN3	3.056655	2.9E-50
PNMA1	3.056892	2.35E-54
DUSP6	3.05731	1.42E-55
FBLN2	3.058042	4.36E-48
LY6D	3.060503	3.73E-31
AMN	3.061326	5.18E-47
LIPH	3.064912	2.05E-49
GPSM3	3.067519	1.7E-51

CTSC	3.074094	9.21E-53
PLEKHO1	3.074497	3.05E-51
TREM1	3.074905	2.58E-50
CPXM1	3.076534	6.04E-48
HTRA1	3.081267	1.12E-51
LMNA	3.081323	1.03E-55
SERINC2	3.08411	6.05E-48
CFH	3.084182	1.5E-49
PRSS23	3.088332	3.81E-53
HLA-DMA	3.088356	5.83E-49
TTYH3	3.098123	7.51E-53
SDCBP2	3.098383	2.87E-46
MZB1	3.099652	3.38E-48
MARVELD1	3.107637	2.18E-53
ANGPTL2	3.109733	4.55E-52
FERMT1	3.110111	6.23E-52
COL14A1	3.115521	1.81E-49
TREM2	3.115904	5.88E-50
MTMR11	3.119874	9.52E-51
CCDC80	3.120714	2.83E-43
LIF	3.12273	6.91E-49
SYT13	3.132342	6.86E-53
CLDN2	3.13566	1.04E-33
UBE2S	3.136918	7.66E-57
IFI30	3.137487	6.04E-51
ALOX5AP	3.138471	3.95E-47
ADAM28	3.139621	3.63E-47
KYNU	3.147119	2.98E-53
SLC6A6	3.147528	6.55E-52
AHR	3.148535	4.01E-54
LCP1	3.149494	1.18E-49
F5	3.151607	9.74E-50
SUGCT	3.156132	8.48E-51
RGS1	3.15772	4.77E-43
PMEPA1	3.160899	5.55E-48
ADGRF1	3.161408	4.65E-50
MLPH	3.161977	1.62E-51
COL6A3	3.168338	2.64E-41
EFNB2	3.16836	1.95E-55
CARD16	3.169409	2.06E-52
C1QTNF5	3.170845	1.84E-52
MYO1E	3.182132	3.57E-53
FNDC1	3.182903	3.07E-49
BAIAP2L2	3.184363	3.01E-52
TSPAN15	3.190769	4.07E-52
UBE2T	3.190992	2.8E-57
FDCSP	3.192504	6.61E-45
COL16A1	3.192682	6.01E-49
TAGLN	3.196565	2.17E-47
SRGN	3.200752	3.97E-49
CSTB	3.203563	8.19E-55
NMU	3.207529	1.23E-54
TM4SF4	3.209208	5.89E-42
EMB	3.215374	5.7E-54
HOXB7	3.216553	1.17E-55
PRR15	3.217419	4.16E-52
FOSL1	3.219713	1.36E-51
IFI16	3.222088	3.25E-48
MBOAT2	3.222365	2.54E-54

MXRA5	3.223608	4.56E-45
VSIG1	3.224439	1.38E-50
ABHD17C	3.224912	8.06E-57
LAMA4	3.22704	1.15E-53
ADAM9	3.240713	6.35E-52
CAPN5	3.243222	1.59E-52
MST1R	3.24458	3.67E-45
RAB34	3.244657	4.86E-52
HEPH	3.25304	1.04E-53
DKK1	3.261196	3.89E-48
SCD	3.261387	2.97E-49
AIF1	3.264559	6.57E-50
TOP2A	3.265593	1.91E-56
PLPP4	3.273633	7.38E-53
HK2	3.275218	1.96E-50
CCL5	3.278262	8.09E-52
LTB	3.279378	6.28E-48
SLC6A14	3.283348	7.8E-51
C1QC	3.284394	3.31E-47
FCGR3A	3.284484	1.35E-47
C1QB	3.285026	1.01E-46
PRAP1	3.285208	1.14E-51
HLA-F	3.285732	9.06E-52
GRN	3.287045	1.18E-54
CLIC1	3.287922	5.38E-56
OAS2	3.290287	3.1E-52
PYCARD	3.29196	4.62E-54
FSTL1	3.295	5.84E-50
COL7A1	3.297937	1.53E-50
TNC	3.298717	8.56E-51
COL4A1	3.301478	9.08E-51
COL4A2	3.30162	5.75E-51
BASP1	3.306057	1.12E-50
RUNX1	3.312529	1.09E-50
PPARG	3.3168	6.58E-53
EFNB1	3.319734	2.07E-54
OASL	3.319803	4.3E-55
GNA15	3.32089	2.47E-54
EDIL3	3.326257	3.1E-54
EGLN3	3.32635	2.64E-51
LBH	3.327834	5.31E-50
KCNK1	3.328581	8.15E-54
BMP4	3.331886	1.23E-52
KLK8	3.338808	5.63E-47
PLCD3	3.340031	4.24E-53
CXCL16	3.340451	6.07E-54
CTSB	3.340651	2.07E-54
LST1	3.341291	4.86E-51
VASP	3.346331	6.28E-55
ARL4C	3.35435	2.25E-50
IFI27L2	3.355023	2.75E-54
SH3KBP1	3.35511	3.09E-55
SERPINE2	3.355581	3.69E-52
PITX1	3.358096	4.37E-52
CORO2A	3.359191	1.77E-57
EPSTI1	3.360551	8.96E-53
GPR35	3.362575	5.48E-52
MFAP5	3.363308	2.27E-45
FAP	3.365101	3.98E-47

CFL1	3.370315	4.21E-55
NT5E	3.379599	5.66E-55
MUC5AC	3.38503	2.24E-43
SPDEF	3.385218	1.11E-50
GAPDH	3.392496	5.51E-57
MYL9	3.39283	8.72E-51
CA9	3.392909	4.95E-36
ARPC1B	3.400602	8.86E-54
STMN1	3.40151	2.79E-54
MATN3	3.404794	1.33E-52
MROH6	3.409166	8.74E-55
CDC20	3.4129	5.25E-56
GEM	3.414857	6.97E-51
LEMD1	3.416868	1.17E-52
ISG20	3.418244	5.51E-53
STRA6	3.41914	4.64E-53
CST6	3.420998	5.11E-48
UCP2	3.422256	4.64E-53
C2	3.423876	4.59E-53
PDLIM7	3.425144	2.42E-51
LEF1	3.436092	5.12E-52
ITGA2	3.439079	3.57E-55
PLEK2	3.441141	7.94E-47
CDC42EP5	3.450162	1.12E-52
CPZ	3.450997	2.39E-54
MYOF	3.453815	1.4E-50
ITGA3	3.455086	1.24E-54
HLA-DMB	3.466486	1.94E-50
DKK3	3.476309	2.37E-53
NREP	3.476477	5.88E-53
ARHGDIB	3.481159	3.82E-51
ITGB2	3.484463	4.29E-49
SEMA3C	3.487359	5.43E-51
NRP2	3.487991	6.1E-55
LTBP1	3.489462	8.35E-52
VSTM2L	3.492699	2.14E-51
BIK	3.493149	4.13E-55
MXRA8	3.494226	2.9E-49
EMP3	3.499078	8.84E-53
MDFI	3.503715	1.99E-52
CKLF	3.504504	6.25E-56
EMP1	3.510636	2.42E-51
CARD11	3.521144	9.91E-54
SERPINE1	3.521758	1.38E-47
CDHR2	3.522682	2.24E-53
ITGB5	3.525915	3.12E-55
AL121845.:	3.527502	5.49E-55
MMP2	3.530095	1.56E-46
HMGA1	3.530412	7.52E-56
TPM4	3.532147	2.09E-52
ANXA8	3.533346	2.64E-46
ACTB	3.533643	6.07E-54
TYROBP	3.547829	2.73E-50
SELPLG	3.547844	1.85E-52
TNFRSF21	3.549114	3.27E-54
NTM	3.550058	5.17E-52
BATF	3.550818	1.21E-54
RAB31	3.553388	1.74E-51
TNFRSF6B	3.554509	8.12E-43

TGM2	3.562405	4.61E-47
TMEM92	3.56341	8.26E-49
TMEM176A	3.563859	7.83E-54
RGS10	3.566507	1.01E-53
GPNMB	3.566719	4.07E-48
F2R	3.567429	1.48E-52
PALLD	3.56848	1.54E-51
HLA-B	3.572005	2.89E-53
PDLIM4	3.574185	7.65E-52
PFKP	3.580497	2.7E-54
LAMP5	3.583711	1.82E-51
RFLNA	3.594204	5.4E-56
FBN1	3.600014	1.45E-50
TMEM176B	3.602097	2.1E-53
MMP14	3.618874	1.19E-48
B2M	3.629654	2.75E-53
CEACAM1	3.632854	2.31E-55
LGALS3	3.633217	3.38E-50
CDA	3.634236	4.36E-55
TRIM31	3.637357	1.65E-50
KLF5	3.640802	3.64E-49
PTGES	3.645808	2.51E-52
AREG	3.657899	5.71E-52
SERPINH1	3.660235	2.98E-53
C1QA	3.666802	1.06E-50
GBP2	3.668153	1.21E-52
MYEOV	3.668189	3.73E-44
CCL19	3.66895	3.65E-39
SEMA7A	3.675819	1.42E-51
LGALS9	3.676675	2.42E-49
ITGBL1	3.677686	6.2E-49
SLC15A3	3.678333	1.63E-52
OCIAD2	3.685273	6.64E-57
CMTM3	3.689599	1.9E-52
USH1C	3.696203	9.21E-53
KRT7	3.697759	7.16E-45
ANXA2	3.700644	1.92E-52
CCL20	3.701619	2.57E-47
COTL1	3.702072	7.83E-53
LOXL1	3.713559	8.22E-53
MUC5B	3.718818	8.89E-37
IGFL2	3.727607	1.19E-53
SDR16C5	3.741996	1.01E-49
LAPTM5	3.746118	9E-51
HPGD	3.766102	4.98E-54
BCAS1	3.776104	5.57E-51
DHRS9	3.780415	1.23E-52
PSMB9	3.785608	2.84E-53
IGFBP5	3.788203	1.73E-48
FSCN1	3.789444	8.68E-55
AKR1B10	3.791835	1.16E-45
ASPN	3.793591	1.12E-50
INHBA	3.797826	2.47E-54
PTTG1	3.80195	9.21E-57
PKM	3.802192	2.48E-56
MGLL	3.813229	4.1E-55
CST2	3.821072	1.64E-51
GREM1	3.825123	9.57E-46
NNMT	3.825225	9.61E-50

TMEM45B	3.828563	7.96E-55
MUC3A	3.834548	1.42E-48
CKS2	3.850578	1.06E-56
KLK7	3.861664	8.46E-47
RAC2	3.867441	3.51E-53
ST6GALNA	3.873891	4.01E-50
DMBT1	3.874042	2.84E-41
HLA-DRB5	3.87471	1.11E-43
ACSL5	3.881112	4.2E-52
UBD	3.890088	2.51E-41
HLA-DPB1	3.903697	1.29E-51
TMSB10	3.906318	7.79E-55
BST2	3.909903	5.6E-53
PIGR	3.910694	3.14E-39
HLA-DQB1	3.919355	2.12E-45
SMIM24	3.920852	8.72E-51
LGALS3BP	3.926862	5.06E-57
PHLDA2	3.938135	6.52E-50
TM4SF5	3.941848	2.81E-52
CDH11	3.948118	1.28E-49
CD55	3.949104	2.98E-54
CXCR4	3.954432	2.2E-51
CLIC3	3.959574	4.43E-53
HLA-DPA1	3.960531	2.75E-51
LGALS1	3.96107	5.49E-52
SERPINA1	3.96646	1.89E-47
MARCKSL1	3.96806	1.31E-55
ACTA2	3.981499	8.51E-51
APOE	3.988474	8.34E-50
UGT1A10	3.989425	1.08E-55
SERPINB5	3.994827	5.77E-50
IGLL5	4.000492	1.95E-45
COL8A1	4.015491	1E-51
LY6E	4.024322	4.49E-53
OAS1	4.032335	6.99E-55
TUBB3	4.03547	3.01E-53
ECM1	4.04911	7.7E-54
AEBP1	4.054033	7.58E-50
DPYSL3	4.0559	2.66E-53
ANO1	4.063277	2.51E-55
FAM3D	4.065178	1.54E-51
CAMK2N1	4.067617	2.2E-56
ANXA1	4.069107	5.58E-52
VILL	4.07414	2.66E-53
TNS4	4.075526	1.06E-49
CXCL14	4.077753	7.84E-45
COL12A1	4.082652	6.75E-51
FAM83E	4.099168	6.68E-54
LGALS4	4.099483	2.3E-43
HLA-A	4.106666	1.4E-55
OLFML2B	4.109562	8.55E-54
MMP9	4.111686	2.4E-54
SFTA2	4.111806	1.12E-43
CYP2S1	4.115806	1.37E-54
HLA-DRB1	4.129361	2.98E-51
TGFBI	4.132574	5.38E-53
S100A4	4.136986	4.9E-52
DUOXA2	4.137569	1.14E-47
SDC1	4.151061	2E-53

AQP5	4.152766	2.16E-44
CD74	4.166868	2.83E-52
DUOX2	4.169211	4.2E-46
ANTXR1	4.177104	1.81E-52
IL1RN	4.184299	1.15E-50
S100A11	4.188534	1.14E-51
SULF2	4.196135	6.38E-55
HOPX	4.199535	3.57E-52
CAPG	4.203322	1.03E-48
UBE2C	4.203353	1.22E-56
GJB3	4.206514	6.33E-50
AGR3	4.211233	1.25E-48
WFDC2	4.213481	5.06E-51
ISG15	4.218295	1.98E-53
CTSK	4.226929	1.55E-51
TSPAN8	4.239168	9.56E-48
ERN2	4.241524	1.68E-46
ZG16B	4.244747	4.89E-51
B3GNT3	4.251451	7.98E-55
CXCL5	4.253291	1.56E-46
CTSS	4.258274	7.41E-52
CD52	4.297812	7.96E-52
AHNAK2	4.301342	3.84E-51
BGN	4.312572	9.59E-52
HLA-DQA1	4.320565	7.71E-52
JCHAIN	4.324147	7.9E-43
GJB2	4.329275	1.02E-46
SLC44A4	4.330147	1.86E-50
S100A16	4.330278	8.29E-54
MMP28	4.341281	8.83E-57
S100A10	4.343818	3.91E-56
COL11A1	4.349859	5.43E-47
CCL18	4.354892	6.58E-55
S100A14	4.356093	1.72E-46
MUCL3	4.357101	2.01E-42
ITGB4	4.35956	1.69E-50
GABRP	4.380437	1.54E-48
CAPN8	4.384966	6.69E-48
TMC5	4.396066	1.06E-49
CD68	4.411805	8.49E-53
KRT16	4.418376	1.56E-46
KLK6	4.418531	7.82E-52
C15orf48	4.42982	2.08E-51
MMP7	4.437144	3.33E-43
FHL2	4.448838	8.04E-56
COL5A2	4.457366	5.73E-53
JPT1	4.463216	7.4E-56
PLAU	4.469085	2.75E-53
THY1	4.485634	1.2E-52
IFI6	4.50108	5.1E-54
CRABP2	4.514963	1.78E-53
TIMP1	4.515055	7.39E-54
MALL	4.526852	6.35E-56
CDH3	4.537167	3.51E-55
HTRA3	4.542931	7.11E-52
S100A2	4.54753	1.41E-51
NBL1	4.558508	2.23E-55
LAMA3	4.563913	1.58E-55
HLA-DRA	4.597315	5.58E-52

ISLR	4.602975	7.84E-52
MUC13	4.604879	3.46E-52
FXYD5	4.613883	3.25E-55
COL5A1	4.618773	5.32E-52
FXYD3	4.639002	1.19E-53
VCAN	4.659901	1.52E-51
AOC1	4.682907	6.91E-56
EPS8L3	4.710243	1.13E-51
COMP	4.71706	1.32E-46
SLC16A3	4.719659	3.68E-55
SFRP4	4.724302	5.52E-50
IL2RG	4.729336	3.1E-53
PLAUR	4.747157	2.29E-54
THBS2	4.760562	3.06E-52
MDK	4.767227	2.81E-56
LUM	4.783876	1.09E-50
PI3	4.785305	7.48E-49
PLAT	4.833329	1.67E-55
APOL1	4.842983	7.15E-56
CLDN18	4.856171	1.29E-40
APOC1	4.884309	7.11E-52
SLC2A1	4.925783	1.26E-55
MIA	4.939463	9.34E-54
SLPI	4.948361	1.22E-51
PLAC8	5.002141	2.44E-55
SFRP2	5.016809	8.57E-47
GCNT3	5.025071	6.01E-53
VSIG2	5.060383	2.47E-50
GPX2	5.131797	3.66E-52
MISP	5.133336	2.97E-57
SPARC	5.1357	1.43E-53
KLK10	5.164017	1.55E-49
TCN1	5.183044	1.08E-46
MMP1	5.190302	8.69E-53
TRIM29	5.1966	8.62E-47
TFF3	5.198172	1.19E-48
KCNN4	5.207049	2.85E-53
IGFBP3	5.234595	9.67E-55
COL17A1	5.297069	6.18E-54
CTHRC1	5.33775	1.46E-53
SULF1	5.364462	5.41E-53
FN1	5.374386	4.83E-52
NQO1	5.419994	8.13E-57
COL1A2	5.473531	8.77E-53
LYZ	5.489223	3.18E-51
PSCA	5.518666	5.8E-46
LAMC2	5.555488	2.89E-52
GPRC5A	5.569079	4.98E-49
LCN2	5.58968	3.36E-46
COL10A1	5.659595	2.79E-51
IFI27	5.69835	1.63E-56
TFF2	5.746152	2.92E-45
COL3A1	5.750717	3.07E-52
TSPAN1	5.772587	6.86E-56
LAMB3	5.785353	9.65E-52
SFN	5.794626	2.3E-49
AGR2	5.860365	3.13E-53
POSTN	5.869665	1.28E-52
CRIP1	5.945293	2.4E-56

KRT19	5.991461	7.1E-53
COL1A1	5.996666	2.06E-52
CST1	6.067517	4.51E-57
S100A6	6.17025	3.93E-57
TMPRSS4	6.292358	1.53E-52
CEACAM5	6.369322	1.41E-50
MSLN	6.533003	8.65E-52
KRT17	6.652364	3.27E-50
CTSE	6.664849	2.91E-48
C19orf33	6.665058	5.83E-53
CEACAM6	6.793315	5.48E-51
MMP11	6.847906	2.9E-54
TFF1	7.301595	2.66E-50
S100P	7.531404	3.24E-52
