# Supplementary material for: A Novel Cuproptosis-Associated Gene Signature to Predict Prognosis in Patients with Pancreatic Cancer
Source: Biomed Res Int. 2023 Jan 18;2023:3419401. doi: 10.1155/2023/3419401 (PMC9876676; doi:10.1155/2023/3419401)
Supplement: Supplementary Materials — Supplementary Table 1: 7978 DEGs between 178 tumor tissues and 171 normal tissues. Supplementary Table 2: 5252 cuproptosis-related genes based on 19 cuproptosis genes. Supplementary Table 3: 202 prognostic genes obtained by univariate Cox regression analysis. Supplementary Table 4: the risk scores and risk groups for all patients. Supplementary Table 5: 183 DEGs between high- and low-risk groups. Supplementary Table 6: risk scores for samples sourced from the GSE62452 and GSE28735 datasets. [file 3419401.f1.zip › 3419401.f2.pdf]

cuproptosis genes	cuproptosis-related genes	correlation	pvalue
ATP7A	GPBP1L1	0.50006571	1.19E-12
ATP7A	CMIP	0.50055934	1.12E-12
ATP7A	JHY	0.50071728	1.1E-12
ATP7A	FAM102A	0.50080676	1.09E-12
ATP7A	SLC38A7	0.50096203	1.07E-12
ATP7A	SPIDR	0.5011072	1.05E-12
ATP7A	TSG101	0.50116744	1.04E-12
ATP7A	CAPZA1	0.50118668	1.04E-12
ATP7A	VMA21	0.50130833	1.02E-12
ATP7A	LRRFIP2	0.50131859	1.02E-12
ATP7A	B3GNT5	0.50132109	1.02E-12
ATP7A	DERA	0.5013737	1.02E-12
ATP7A	TIAM2	0.50149188	1E-12
ATP7A	ARHGAP12	0.50154448	9.95E-13
ATP7A	ATXN7L1	0.50154899	9.94E-13
ATP7A	PRTFDC1	0.5015757	9.91E-13
ATP7A	LARGE1	0.5016239	9.85E-13
ATP7A	PLA2G4A	0.50174069	9.72E-13
ATP7A	GNA12	0.50185094	9.59E-13
ATP7A	CALD1	0.50187832	9.56E-13
ATP7A	USP34	0.5019168	9.51E-13
ATP7A	OSTF1	0.50201407	9.4E-13
ATP7A	IRS1	0.50211002	9.3E-13
ATP7A	PAIP2	0.50230431	9.08E-13
ATP7A	NT5C3A	0.50233365	9.05E-13
ATP7A	ST3GAL2	0.50249853	8.87E-13
ATP7A	FBN1	0.50259108	8.77E-13
ATP7A	PGK1	0.50259674	8.77E-13
ATP7A	ABI3BP	0.50264522	8.72E-13
ATP7A	PDGFRA	0.50270738	8.65E-13
ATP7A	PECAM1	0.50275136	8.61E-13
ATP7A	NSMCE3	0.50282482	8.53E-13
ATP7A	FOSL2	0.50303688	8.32E-13
ATP7A	ZNF552	0.50305216	8.3E-13
ATP7A	CAMKK2	0.50314777	8.21E-13
ATP7A	CORO1C	0.50321476	8.14E-13
ATP7A	TAF12	0.50324861	8.11E-13
ATP7A	MANSC1	0.50325538	8.1E-13
ATP7A	SMURF1	0.50331897	8.04E-13
ATP7A	HECW2	0.50331912	8.04E-13
ATP7A	CTSS	0.50346537	7.9E-13
ATP7A	GK	0.50353039	7.84E-13
ATP7A	NUP155	0.50355215	7.81E-13
ATP7A	ATP2B4	0.50356888	7.8E-13
ATP7A	TMEM165	0.50388082	7.51E-13
ATP7A	JARID2	0.50392663	7.47E-13
ATP7A	UTP11	0.50394642	7.45E-13
ATP7A	GNB4	0.50421885	7.21E-13
ATP7A	IVNS1ABP	0.50429724	7.14E-13
ATP7A	HPRT1	0.50430262	7.14E-13
ATP7A	MRPL50	0.5043704	7.08E-13
ATP7A	ROBO1	0.50450531	6.96E-13
ATP7A	CLNS1A	0.50450848	6.96E-13
ATP7A	PDGFRB	0.5045274	6.95E-13
ATP7A	DDX50	0.50464239	6.85E-13
ATP7A	CDK2	0.50471918	6.79E-13
ATP7A	TUT7	0.50474448	6.77E-13

ATP7A	CNNM4	0.50476046	6.75E-13
ATP7A	CRYBG1	0.5047856	6.73E-13
ATP7A	CDK14	0.50484737	6.68E-13
ATP7A	PRICKLE1	0.50499053	6.57E-13
ATP7A	RRAGC	0.50507577	6.5E-13
ATP7A	VDR	0.50525195	6.36E-13
ATP7A	SAR1A	0.5052867	6.34E-13
ATP7A	ZCRB1	0.5053762	6.27E-13
ATP7A	ODF2	0.50540003	6.25E-13
ATP7A	MYH9	0.50543491	6.22E-13
ATP7A	LTBP2	0.50545238	6.21E-13
ATP7A	FUT11	0.5054588	6.2E-13
ATP7A	SPRY1	0.50552971	6.15E-13
ATP7A	RARB	0.50559378	6.1E-13
ATP7A	CHIC2	0.505772	5.97E-13
ATP7A	AK6	0.50611868	5.73E-13
ATP7A	CASP2	0.5061203	5.73E-13
ATP7A	TIPARP	0.50632176	5.59E-13
ATP7A	CDS1	0.50666316	5.36E-13
ATP7A	TTL	0.50675243	5.3E-13
ATP7A	UBE2K	0.50675764	5.3E-13
ATP7A	COPG2	0.50679427	5.27E-13
ATP7A	ARHGAP11A	0.50708304	5.09E-13
ATP7A	TMEM237	0.50708543	5.09E-13
ATP7A	CHMP5	0.50727971	4.97E-13
ATP7A	TMTC1	0.50729977	4.96E-13
ATP7A	UGT8	0.50733213	4.94E-13
ATP7A	OLA1	0.50741781	4.89E-13
ATP7A	IGFBP5	0.5077379	4.7E-13
ATP7A	ALKBH1	0.50775872	4.69E-13
ATP7A	PARVA	0.50779298	4.67E-13
ATP7A	SLC9B2	0.5078375	4.64E-13
ATP7A	TMEM43	0.5080773	4.51E-13
ATP7A	HMGB1	0.50826203	4.41E-13
ATP7A	C12orf43	0.50844775	4.31E-13
ATP7A	DDX19B	0.5085548	4.25E-13
ATP7A	ADAM22	0.50874221	4.16E-13
ATP7A	DRAM2	0.50879721	4.13E-13
ATP7A	PIAS3	0.50885131	4.1E-13
ATP7A	PNMA1	0.50906326	4E-13
ATP7A	TFPI	0.50924153	3.91E-13
ATP7A	KLHL5	0.50931273	3.87E-13
ATP7A	ZNF277	0.50944162	3.81E-13
ATP7A	DACT1	0.50962276	3.73E-13
ATP7A	SUPT16H	0.5096794	3.7E-13
ATP7A	PPP1R12B	0.50972759	3.68E-13
ATP7A	PRRG4	0.50981339	3.64E-13
ATP7A	SELENOP	0.51003902	3.54E-13
ATP7A	ZNF28	0.51008877	3.52E-13
ATP7A	PKP4	0.51016227	3.49E-13
ATP7A	TMEM128	0.51024226	3.46E-13
ATP7A	BECN1	0.51030662	3.43E-13
ATP7A	YTHDF1	0.51095809	3.16E-13
ATP7A	HAT1	0.51102395	3.14E-13
ATP7A	CALCRL	0.5111783	3.08E-13
ATP7A	MEST	0.51126897	3.04E-13
ATP7A	AP1S2	0.51135395	3.01E-13
ATP7A	ADIPOR1	0.51144454	2.98E-13

ATP7A	CDH6	0.51144823	2.98E-13
ATP7A	SLBP	0.51146367	2.97E-13
ATP7A	CNOT11	0.51156832	2.93E-13
ATP7A	PRKACB	0.51169347	2.89E-13
ATP7A	RETREG2	0.51172619	2.88E-13
ATP7A	SERPINB8	0.5117973	2.85E-13
ATP7A	FEZ2	0.51202952	2.77E-13
ATP7A	PRSS23	0.51208735	2.75E-13
ATP7A	BCAS2	0.51213758	2.73E-13
ATP7A	SUMO2	0.51218191	2.72E-13
ATP7A	NOTCH3	0.51242282	2.64E-13
ATP7A	COG2	0.51263239	2.57E-13
ATP7A	PDGFC	0.51282156	2.51E-13
ATP7A	SNX24	0.51286971	2.5E-13
ATP7A	NDUFA5	0.51289043	2.49E-13
ATP7A	UNC50	0.51313539	2.42E-13
ATP7A	TM2D3	0.5134142	2.33E-13
ATP7A	EXTL3	0.51345039	2.32E-13
ATP7A	SLC15A4	0.51345456	2.32E-13
ATP7A	PA2G4	0.51353234	2.3E-13
ATP7A	AP4S1	0.51360391	2.28E-13
ATP7A	ICMT	0.51361902	2.27E-13
ATP7A	UBAP1	0.51366867	2.26E-13
ATP7A	MAFG	0.5138649	2.21E-13
ATP7A	CDV3	0.51410196	2.14E-13
ATP7A	FAM47E-STBD1	0.51420766	2.11E-13
ATP7A	NSD3	0.51421898	2.11E-13
ATP7A	ABR	0.51430059	2.09E-13
ATP7A	INSIG2	0.51441062	2.06E-13
ATP7A	CALM3	0.51454954	2.02E-13
ATP7A	DDR2	0.51456832	2.02E-13
ATP7A	PTGES3	0.51464651	2E-13
ATP7A	FBXO7	0.51477903	1.97E-13
ATP7A	GRPEL2	0.51491477	1.93E-13
ATP7A	SMIM30	0.51501572	1.91E-13
ATP7A	CCDC97	0.51508	1.89E-13
ATP7A	ADGRA2	0.51511182	1.89E-13
ATP7A	LHFPL2	0.51511852	1.89E-13
ATP7A	PNRC1	0.51515057	1.88E-13
ATP7A	SZRD1	0.5153537	1.83E-13
ATP7A	RNGTT	0.51566959	1.76E-13
ATP7A	HNRNPD	0.5158	1.73E-13
ATP7A	OAZ2	0.51602079	1.68E-13
ATP7A	ZNF512	0.51603829	1.68E-13
ATP7A	ACSL3	0.5160587	1.68E-13
ATP7A	SEMA5A	0.51617287	1.65E-13
ATP7A	AP3S1	0.51640054	1.61E-13
ATP7A	PEA15	0.51641155	1.6E-13
ATP7A	ZNF526	0.51644962	1.6E-13
ATP7A	DIDO1	0.51657726	1.57E-13
ATP7A	WWTR1	0.51661477	1.56E-13
ATP7A	EPB41L1	0.51675187	1.54E-13
ATP7A	PAIP1	0.51675553	1.53E-13
ATP7A	BHLHE41	0.51684255	1.52E-13
ATP7A	ADGRG6	0.51690145	1.51E-13
ATP7A	UGGT1	0.51696713	1.49E-13
ATP7A	DSE	0.51720724	1.45E-13
ATP7A	TJP2	0.5172376	1.44E-13

ATP7A	RBMS2	0.51724827	1.44E-13
ATP7A	PRMT2	0.51733832	1.43E-13
ATP7A	TMEM47	0.51744386	1.41E-13
ATP7A	TMEM9B	0.51748325	1.4E-13
ATP7A	MRFAP1L1	0.51756371	1.39E-13
ATP7A	APMAP	0.51758707	1.38E-13
ATP7A	KANK2	0.51770608	1.36E-13
ATP7A	RAB23	0.51775138	1.35E-13
ATP7A	FAM200A	0.51780682	1.34E-13
ATP7A	SOWAHC	0.51810444	1.29E-13
ATP7A	ASPH	0.51821542	1.28E-13
ATP7A	HGF	0.51833258	1.26E-13
ATP7A	ABHD3	0.51838344	1.25E-13
ATP7A	RAD51B	0.51838469	1.25E-13
ATP7A	SMU1	0.51841548	1.24E-13
ATP7A	NUDT4	0.51845032	1.24E-13
ATP7A	HNRNPA0	0.51848572	1.23E-13
ATP7A	RASSF6	0.51862096	1.21E-13
ATP7A	CYCS	0.51866237	1.21E-13
ATP7A	TRIM34	0.51883873	1.18E-13
ATP7A	PECR	0.51891846	1.17E-13
ATP7A	RAB7A	0.51911276	1.14E-13
ATP7A	TRIO	0.5191979	1.13E-13
ATP7A	YWHAZ	0.51922377	1.12E-13
ATP7A	FGL2	0.51928159	1.12E-13
ATP7A	UBE2V2	0.51930217	1.11E-13
ATP7A	SBDS	0.51931328	1.11E-13
ATP7A	ZEB2	0.51958989	1.07E-13
ATP7A	SHC1	0.5196152	1.07E-13
ATP7A	WDR1	0.5196662	1.06E-13
ATP7A	LACTB2	0.519676	1.06E-13
ATP7A	UBE2H	0.51973625	1.05E-13
ATP7A	LY75	0.51978221	1.05E-13
ATP7A	COPZ1	0.51983677	1.04E-13
ATP7A	KNOP1	0.51987625	1.03E-13
ATP7A	GSKIP	0.51991411	1.03E-13
ATP7A	RTN3	0.52002339	1.01E-13
ATP7A	SF3B3	0.52004647	1.01E-13
ATP7A	TDP2	0.52016682	9.96E-14
ATP7A	TBC1D14	0.52020086	9.92E-14
ATP7A	EPDR1	0.52044676	9.62E-14
ATP7A	LEPR	0.52060924	9.42E-14
ATP7A	DGKH	0.5206785	9.34E-14
ATP7A	ZNF174	0.52069794	9.31E-14
ATP7A	SMARCA2	0.52082621	9.16E-14
ATP7A	SCRN1	0.52085178	9.13E-14
ATP7A	EMC3	0.52102496	8.93E-14
ATP7A	MAST4	0.5210765	8.87E-14
ATP7A	CD93	0.52115417	8.79E-14
ATP7A	NYNRIN	0.52134454	8.57E-14
ATP7A	BICD1	0.52192829	7.96E-14
ATP7A	SIMC1	0.52200085	7.88E-14
ATP7A	PDE4DIP	0.52200344	7.88E-14
ATP7A	YWHAE	0.52204196	7.84E-14
ATP7A	RGL1	0.52234188	7.55E-14
ATP7A	CDH11	0.52236807	7.52E-14
ATP7A	NUDCD2	0.52239892	7.49E-14
ATP7A	POLR3C	0.52254428	7.35E-14

ATP7A	PRDM1	0.52260213	7.3E-14
ATP7A	SEC22A	0.52283526	7.08E-14
ATP7A	ZCCHC10	0.52308222	6.86E-14
ATP7A	VGLL4	0.52311377	6.83E-14
ATP7A	GOLM1	0.52325536	6.71E-14
ATP7A	TCF7L2	0.52327863	6.69E-14
ATP7A	ZFPM2	0.52330191	6.67E-14
ATP7A	LRRC8C	0.52335754	6.62E-14
ATP7A	TRAF3	0.5235248	6.48E-14
ATP7A	INPP5A	0.52364228	6.38E-14
ATP7A	CDK6	0.5237375	6.31E-14
ATP7A	HOMEZ	0.52385906	6.21E-14
ATP7A	MEAF6	0.52387382	6.2E-14
ATP7A	TMEM251	0.52387872	6.19E-14
ATP7A	PPT1	0.52389752	6.18E-14
ATP7A	FAM177A1	0.52390104	6.18E-14
ATP7A	MGAT5	0.52391184	6.17E-14
ATP7A	TRIM69	0.52391905	6.16E-14
ATP7A	ENTPD1	0.52408775	6.03E-14
ATP7A	PLAA	0.52429258	5.87E-14
ATP7A	CCAR2	0.52435429	5.82E-14
ATP7A	SPART	0.52446781	5.74E-14
ATP7A	PAICS	0.52454035	5.69E-14
ATP7A	LGALSL	0.52472732	5.55E-14
ATP7A	PIGW	0.52496362	5.38E-14
ATP7A	SORD	0.52519408	5.23E-14
ATP7A	DLC1	0.52521733	5.21E-14
ATP7A	CDKN1B	0.52528248	5.17E-14
ATP7A	LEPROTL1	0.5253208	5.14E-14
ATP7A	AMBRA1	0.5254101	5.08E-14
ATP7A	TOP2B	0.52551859	5.01E-14
ATP7A	TFE3	0.52557763	4.97E-14
ATP7A	MSANTD3-TMEFF1	0.52559231	4.96E-14
ATP7A	CLIP2	0.52575988	4.86E-14
ATP7A	RHOA	0.52601949	4.69E-14
ATP7A	FARP2	0.52620668	4.58E-14
ATP7A	DDX23	0.52637034	4.49E-14
ATP7A	BTN3A3	0.52642774	4.45E-14
ATP7A	GLE1	0.52645331	4.44E-14
ATP7A	DYNC2LI1	0.5266108	4.35E-14
ATP7A	DYNC1LI1	0.52661585	4.34E-14
ATP7A	TRAFD1	0.52677958	4.25E-14
ATP7A	ANKH	0.52678823	4.25E-14
ATP7A	NCSTN	0.52682349	4.23E-14
ATP7A	UBAP2L	0.52686965	4.2E-14
ATP7A	EIF1AD	0.52688582	4.19E-14
ATP7A	ARHGAP31	0.52709569	4.08E-14
ATP7A	SUSD1	0.52720287	4.02E-14
ATP7A	PRKCI	0.52729163	3.98E-14
ATP7A	RSU1	0.52732612	3.96E-14
ATP7A	CENPL	0.5273762	3.94E-14
ATP7A	MRPL42	0.52748029	3.88E-14
ATP7A	SLIT2	0.52757935	3.83E-14
ATP7A	SEH1L	0.52762257	3.81E-14
ATP7A	SLC16A2	0.52767326	3.79E-14
ATP7A	HDAC2	0.52773108	3.76E-14
ATP7A	PRNP	0.52800726	3.62E-14
ATP7A	IDH1	0.52806267	3.6E-14

ATP7A	MBOAT1	0.52836948	3.46E-14
ATP7A	FAM122B	0.52848133	3.41E-14
ATP7A	DCBLD1	0.52849469	3.4E-14
ATP7A	HSPA9	0.52865239	3.33E-14
ATP7A	RECK	0.52874761	3.29E-14
ATP7A	SUCLG2	0.52887845	3.23E-14
ATP7A	CBLB	0.52895703	3.2E-14
ATP7A	ABCF1	0.52991395	2.82E-14
ATP7A	ETS1	0.5300476	2.77E-14
ATP7A	RUNDC1	0.53014562	2.74E-14
ATP7A	VAPB	0.53022826	2.71E-14
ATP7A	SNX29	0.53029036	2.69E-14
ATP7A	FSTL1	0.53058257	2.58E-14
ATP7A	SMAD7	0.53065146	2.56E-14
ATP7A	SNX5	0.53080753	2.51E-14
ATP7A	ASH2L	0.53084029	2.5E-14
ATP7A	MAP3K20	0.53109658	2.42E-14
ATP7A	HIBADH	0.53116704	2.39E-14
ATP7A	SPRY2	0.53123701	2.37E-14
ATP7A	ABLIM1	0.53149407	2.29E-14
ATP7A	LIN9	0.53171489	2.23E-14
ATP7A	SEMA3C	0.53203158	2.13E-14
ATP7A	PCBP1	0.53214477	2.1E-14
ATP7A	SLC12A2	0.53234327	2.05E-14
ATP7A	MR1	0.53238462	2.04E-14
ATP7A	HPS3	0.53257672	1.99E-14
ATP7A	ERGIC2	0.53260453	1.98E-14
ATP7A	MYH10	0.53271577	1.95E-14
ATP7A	PDCD10	0.53290216	1.9E-14
ATP7A	PTPN1	0.53293251	1.89E-14
ATP7A	CACNA2D1	0.53313869	1.84E-14
ATP7A	UBXN2A	0.53316079	1.84E-14
ATP7A	AFAP1	0.53322054	1.82E-14
ATP7A	H6PD	0.53325782	1.81E-14
ATP7A	SH3KBP1	0.53334621	1.79E-14
ATP7A	PROS1	0.53355647	1.74E-14
ATP7A	MED6	0.53356275	1.74E-14
ATP7A	OGT	0.53358191	1.74E-14
ATP7A	DSC2	0.5336427	1.72E-14
ATP7A	NCOA7	0.53366843	1.72E-14
ATP7A	AMFR	0.53369289	1.71E-14
ATP7A	ADAT1	0.53371052	1.71E-14
ATP7A	TBC1D25	0.53379087	1.69E-14
ATP7A	GNAI3	0.53390657	1.66E-14
ATP7A	ZNF267	0.53402311	1.64E-14
ATP7A	GUCY1B1	0.534073	1.63E-14
ATP7A	HNRNPA1	0.53417487	1.61E-14
ATP7A	PTPRE	0.53430114	1.58E-14
ATP7A	METTL2B	0.53433129	1.57E-14
ATP7A	CREG1	0.53446773	1.54E-14
ATP7A	TMEM126B	0.53459125	1.52E-14
ATP7A	ANXA5	0.53464934	1.51E-14
ATP7A	PIP4K2A	0.53475036	1.49E-14
ATP7A	TOR1B	0.53491151	1.46E-14
ATP7A	AKT3	0.53494371	1.45E-14
ATP7A	ECHDC1	0.53507866	1.42E-14
ATP7A	TPM3	0.53510019	1.42E-14
ATP7A	ANP32A	0.53530621	1.38E-14

ATP7A	RNASEH1	0.53549872	1.35E-14
ATP7A	GTF2E1	0.53553204	1.34E-14
ATP7A	METTL21A	0.53563958	1.32E-14
ATP7A	TFRC	0.53566191	1.32E-14
ATP7A	ATF6B	0.53568016	1.31E-14
ATP7A	JAM3	0.53575796	1.3E-14
ATP7A	TMEM30B	0.53576148	1.3E-14
ATP7A	SH2B3	0.53581285	1.29E-14
ATP7A	KIAA0586	0.53601661	1.25E-14
ATP7A	HMGCR	0.53614592	1.23E-14
ATP7A	CYTH3	0.53615266	1.23E-14
ATP7A	NDNF	0.53635641	1.2E-14
ATP7A	SERBP1	0.53640823	1.19E-14
ATP7A	YWHAH	0.53653043	1.17E-14
ATP7A	METTL4	0.53655875	1.17E-14
ATP7A	RCN2	0.5366395	1.15E-14
ATP7A	DEGS1	0.53710341	1.08E-14
ATP7A	CTNNA1	0.53735939	1.05E-14
ATP7A	JAZF1	0.5373772	1.05E-14
ATP7A	PAK1	0.53739249	1.04E-14
ATP7A	AP1S3	0.53750339	1.03E-14
ATP7A	GMPR2	0.53752495	1.02E-14
ATP7A	ZNF274	0.5375933	1.02E-14
ATP7A	PDE4D	0.53790057	9.74E-15
ATP7A	BFAR	0.53797148	9.65E-15
ATP7A	SKI	0.53800747	9.6E-15
ATP7A	RABGAP1L	0.53801273	9.59E-15
ATP7A	VRK2	0.5381513	9.41E-15
ATP7A	PRKAG1	0.53815977	9.4E-15
ATP7A	CPM	0.53827277	9.26E-15
ATP7A	ARPC5	0.53860507	8.85E-15
ATP7A	HNRNPC	0.53867414	8.77E-15
ATP7A	SIPA1L1	0.53871691	8.72E-15
ATP7A	TMEM154	0.53872009	8.72E-15
ATP7A	PIK3C3	0.53915025	8.22E-15
ATP7A	IPO11	0.53987722	7.45E-15
ATP7A	CCDC6	0.53994987	7.38E-15
ATP7A	CYBRD1	0.54000956	7.32E-15
ATP7A	HMCN1	0.5402467	7.09E-15
ATP7A	AP3M2	0.54036599	6.97E-15
ATP7A	GINM1	0.54038587	6.95E-15
ATP7A	PHF20	0.54055752	6.79E-15
ATP7A	SNX7	0.54060804	6.75E-15
ATP7A	NCL	0.54072499	6.64E-15
ATP7A	GGPS1	0.54086749	6.51E-15
ATP7A	DCLRE1B	0.54107725	6.33E-15
ATP7A	GPN3	0.54107966	6.33E-15
ATP7A	ERI1	0.54116004	6.26E-15
ATP7A	CARD6	0.54123285	6.19E-15
ATP7A	JMJD1C	0.54125339	6.18E-15
ATP7A	LTBP1	0.5413629	6.09E-15
ATP7A	RHOU	0.54149639	5.98E-15
ATP7A	ZNF250	0.54154569	5.94E-15
ATP7A	KIAA1217	0.54156919	5.92E-15
ATP7A	ECD	0.54180555	5.73E-15
ATP7A	RAB12	0.54189449	5.66E-15
ATP7A	DISP1	0.54193297	5.63E-15
ATP7A	GNB5	0.54195815	5.61E-15

ATP7A	IFNAR2	0.54220772	5.42E-15
ATP7A	SEC14L1	0.542271	5.37E-15
ATP7A	IFT52	0.54228507	5.36E-15
ATP7A	PCSK5	0.54239163	5.29E-15
ATP7A	SSPN	0.54242419	5.26E-15
ATP7A	C1QTNF7	0.54254302	5.18E-15
ATP7A	MKRN1	0.54292042	4.92E-15
ATP7A	NUP107	0.54294396	4.9E-15
ATP7A	PFKFB3	0.54296665	4.89E-15
ATP7A	AHCYL2	0.54330425	4.66E-15
ATP7A	NOLC1	0.54342035	4.59E-15
ATP7A	PITPNB	0.54346732	4.56E-15
ATP7A	MFHAS1	0.54352566	4.52E-15
ATP7A	GNA11	0.54355554	4.51E-15
ATP7A	ECM2	0.54362133	4.47E-15
ATP7A	ABCC4	0.54371238	4.41E-15
ATP7A	GNAI1	0.54377553	4.37E-15
ATP7A	GDI2	0.5439891	4.24E-15
ATP7A	PLEKHA2	0.54400984	4.23E-15
ATP7A	MEAK7	0.54419042	4.13E-15
ATP7A	JADE2	0.54444663	3.99E-15
ATP7A	TTC23	0.54456801	3.92E-15
ATP7A	SLC39A14	0.54472539	3.83E-15
ATP7A	IVD	0.54472583	3.83E-15
ATP7A	EMB	0.54473196	3.83E-15
ATP7A	CLIC4	0.54477095	3.81E-15
ATP7A	ZCCHC9	0.5448408	3.77E-15
ATP7A	NAAA	0.54488545	3.75E-15
ATP7A	RDH10	0.54496055	3.71E-15
ATP7A	C14orf119	0.54549403	3.45E-15
ATP7A	STX6	0.54557364	3.41E-15
ATP7A	EIF5A2	0.54562265	3.39E-15
ATP7A	RAB9A	0.54566355	3.37E-15
ATP7A	PLEKHA5	0.54587807	3.27E-15
ATP7A	SVIP	0.54600156	3.21E-15
ATP7A	GLB1	0.54612369	3.16E-15
ATP7A	VPS33A	0.54615661	3.15E-15
ATP7A	FASTKD5	0.54629475	3.09E-15
ATP7A	ISY1-RAB43	0.54651069	2.99E-15
ATP7A	SLFN5	0.54674436	2.9E-15
ATP7A	NAGA	0.54675033	2.9E-15
ATP7A	MED28	0.54702932	2.79E-15
ATP7A	MRPS27	0.54746688	2.62E-15
ATP7A	EFL1	0.54748554	2.61E-15
ATP7A	SMC4	0.54755213	2.59E-15
ATP7A	MAP3K13	0.54764242	2.56E-15
ATP7A	TMEM230	0.54769049	2.54E-15
ATP7A	RALB	0.54769612	2.54E-15
ATP7A	ANKRD27	0.54781577	2.5E-15
ATP7A	APEX1	0.54796662	2.45E-15
ATP7A	PLEKHA6	0.54800878	2.43E-15
ATP7A	GPBP1	0.54801201	2.43E-15
ATP7A	FBXO42	0.54801752	2.43E-15
ATP7A	PIP4K2C	0.54808506	2.41E-15
ATP7A	WASF2	0.54836155	2.31E-15
ATP7A	NDRG3	0.54849076	2.27E-15
ATP7A	UXS1	0.54855264	2.25E-15
ATP7A	ERGIC1	0.54865026	2.22E-15

ATP7A	ZNF131	0.54866789	2.22E-15
ATP7A	KIRREL1	0.54866879	2.22E-15
ATP7A	B4GALT5	0.54868173	2.21E-15
ATP7A	CYP2U1	0.54876199	2.19E-15
ATP7A	RUFY1	0.54889506	2.15E-15
ATP7A	PANX1	0.54895509	2.13E-15
ATP7A	TLR4	0.54903684	2.11E-15
ATP7A	TTC8	0.54912685	2.08E-15
ATP7A	LIMCH1	0.54923313	2.05E-15
ATP7A	UTP3	0.54993461	1.86E-15
ATP7A	NHS	0.55015552	1.8E-15
ATP7A	ATP6V1D	0.55015804	1.8E-15
ATP7A	CASP8	0.55041157	1.74E-15
ATP7A	DHX35	0.55053951	1.7E-15
ATP7A	MFAP1	0.55057368	1.7E-15
ATP7A	PPP4R1	0.55077768	1.65E-15
ATP7A	ASAP2	0.55087095	1.63E-15
ATP7A	TMEM200A	0.55088809	1.62E-15
ATP7A	MAX	0.5510824	1.58E-15
ATP7A	XRCC5	0.55131699	1.53E-15
ATP7A	LACTB	0.55137811	1.51E-15
ATP7A	CIR1	0.55144905	1.5E-15
ATP7A	CALU	0.55149227	1.49E-15
ATP7A	C4orf33	0.55150834	1.49E-15
ATP7A	RAB27A	0.55162056	1.46E-15
ATP7A	KLF6	0.5517827	1.43E-15
ATP7A	ELF4	0.55183776	1.42E-15
ATP7A	SPTBN1	0.55205891	1.38E-15
ATP7A	SCO1	0.55251255	1.29E-15
ATP7A	TLR5	0.55274419	1.25E-15
ATP7A	GNB1	0.55298007	1.21E-15
ATP7A	MFSD11	0.55314279	1.18E-15
ATP7A	PLXNC1	0.55316986	1.18E-15
ATP7A	NIP7	0.5531807	1.17E-15
ATP7A	HDGFL3	0.55352153	1.12E-15
ATP7A	AFTPH	0.55366726	1.1E-15
ATP7A	EXOC1	0.55369551	1.09E-15
ATP7A	RMDN2	0.55373867	1.08E-15
ATP7A	SLC40A1	0.55394544	1.05E-15
ATP7A	MEIS2	0.55404114	1.04E-15
ATP7A	WBP4	0.55408228	1.03E-15
ATP7A	GABPB1	0.55415389	1.02E-15
ATP7A	MESD	0.55420685	1.01E-15
ATP7A	TCEAL8	0.55421122	1.01E-15
ATP7A	DCLRE1C	0.55422177	1.01E-15
ATP7A	SLC25A43	0.55422701	1.01E-15
ATP7A	PRKDC	0.55424272	1.01E-15
ATP7A	DNAJC13	0.55463644	9.54E-16
ATP7A	TMX4	0.55503796	9.01E-16
ATP7A	SPRY4	0.5555015	8.43E-16
ATP7A	MOB1B	0.55560172	8.31E-16
ATP7A	EDIL3	0.55571418	8.18E-16
ATP7A	MRPL35	0.5557222	8.17E-16
ATP7A	IQGAP1	0.55578678	8.09E-16
ATP7A	LIMA1	0.55596679	7.89E-16
ATP7A	SLFN11	0.5560301	7.81E-16
ATP7A	EMC1	0.55614852	7.68E-16
ATP7A	KSR1	0.5562813	7.54E-16

ATP7A	ABL2	0.55643352	7.37E-16
ATP7A	SYAP1	0.55649385	7.31E-16
ATP7A	VCL	0.5568775	6.92E-16
ATP7A	SPTSSA	0.55690143	6.89E-16
ATP7A	MACF1	0.55702045	6.78E-16
ATP7A	DNAJB6	0.55706785	6.73E-16
ATP7A	PSMD12	0.55710373	6.7E-16
ATP7A	SLC8A1	0.55731054	6.5E-16
ATP7A	INTS14	0.55759171	6.24E-16
ATP7A	C9orf78	0.55765591	6.19E-16
ATP7A	MTFMT	0.55774689	6.1E-16
ATP7A	PSMC6	0.55786184	6E-16
ATP7A	PHTF2	0.5578968	5.97E-16
ATP7A	CRISPLD1	0.55793914	5.94E-16
ATP7A	PNO1	0.55804379	5.85E-16
ATP7A	DUSP18	0.55808672	5.81E-16
ATP7A	RCBTB2	0.55810488	5.8E-16
ATP7A	TEX9	0.55838909	5.56E-16
ATP7A	DPF2	0.55849643	5.48E-16
ATP7A	PER2	0.55863716	5.37E-16
ATP7A	MSI2	0.55865838	5.35E-16
ATP7A	DCP2	0.55874095	5.29E-16
ATP7A	ARID5B	0.55876499	5.27E-16
ATP7A	YTHDF2	0.55921329	4.94E-16
ATP7A	LRIF1	0.55938714	4.82E-16
ATP7A	PARN	0.55939659	4.81E-16
ATP7A	OSTM1	0.55940307	4.8E-16
ATP7A	ERC1	0.55947211	4.76E-16
ATP7A	PRKG1	0.55965391	4.63E-16
ATP7A	IRAK3	0.55978294	4.55E-16
ATP7A	DTX3L	0.55983644	4.51E-16
ATP7A	BRAP	0.55988144	4.48E-16
ATP7A	ELOVL5	0.55995705	4.43E-16
ATP7A	HECA	0.56011429	4.33E-16
ATP7A	RAI14	0.56012426	4.33E-16
ATP7A	RASA2	0.56017124	4.3E-16
ATP7A	EXOC6	0.56035112	4.19E-16
ATP7A	FNTB	0.56040539	4.15E-16
ATP7A	CEP250	0.56050796	4.09E-16
ATP7A	RBMS1	0.56052992	4.08E-16
ATP7A	ATXN3	0.56053663	4.07E-16
ATP7A	CLDND1	0.56061412	4.03E-16
ATP7A	ARCN1	0.56099345	3.81E-16
ATP7A	TOX3	0.56104549	3.78E-16
ATP7A	LRCH3	0.56117191	3.71E-16
ATP7A	PPP1R3B	0.56121084	3.69E-16
ATP7A	ARMC8	0.56148335	3.55E-16
ATP7A	AMOT	0.5616309	3.47E-16
ATP7A	NAPEPLD	0.56170492	3.44E-16
ATP7A	RNF139	0.56170858	3.43E-16
ATP7A	RCAN3	0.56184381	3.37E-16
ATP7A	XIAP	0.56202198	3.28E-16
ATP7A	RGS5	0.56214141	3.22E-16
ATP7A	MAP1LC3B	0.56226027	3.17E-16
ATP7A	NUP98	0.56245846	3.08E-16
ATP7A	ATP11C	0.56250915	3.05E-16
ATP7A	ARL6IP5	0.56265746	2.99E-16
ATP7A	BTN3A1	0.56287686	2.89E-16

ATP7A	APLP2	0.56302863	2.83E-16
ATP7A	COPS4	0.56303705	2.83E-16
ATP7A	ZNF350	0.56308425	2.81E-16
ATP7A	ZNF816	0.56331962	2.71E-16
ATP7A	VIPAS39	0.56350342	2.64E-16
ATP7A	REEP5	0.56363355	2.59E-16
ATP7A	STARD4	0.5638074	2.52E-16
ATP7A	SLC25A44	0.56381811	2.52E-16
ATP7A	GLO1	0.56421806	2.38E-16
ATP7A	HCCS	0.56435309	2.33E-16
ATP7A	BMP2K	0.56446797	2.29E-16
ATP7A	COQ10B	0.56453723	2.27E-16
ATP7A	AHNAK	0.56458146	2.25E-16
ATP7A	MFN2	0.56467528	2.22E-16
ATP7A	MGRN1	0.5647384	2.2E-16
ATP7A	GALNT10	0.56494541	2.13E-16
ATP7A	SYS1	0.56499332	2.12E-16
ATP7A	STIM1	0.5650485	2.1E-16
ATP7A	ASNSD1	0.56509048	2.09E-16
ATP7A	SESN3	0.56548577	1.97E-16
ATP7A	SNX12	0.56567011	1.92E-16
ATP7A	BMP2	0.56573348	1.9E-16
ATP7A	ZC4H2	0.5657929	1.88E-16
ATP7A	WEE1	0.5658096	1.88E-16
ATP7A	ELAVL1	0.56583619	1.87E-16
ATP7A	CCDC43	0.56586167	1.86E-16
ATP7A	CDS2	0.56607185	1.81E-16
ATP7A	DIPK1A	0.56610582	1.8E-16
ATP7A	TMEM14A	0.56617494	1.78E-16
ATP7A	GSPT1	0.56626209	1.76E-16
ATP7A	ARSJ	0.56629729	1.75E-16
ATP7A	NIPSNAP3A	0.56630457	1.75E-16
ATP7A	MEF2C	0.56645394	1.71E-16
ATP7A	GRWD1	0.56650685	1.69E-16
ATP7A	MAN2B2	0.56656315	1.68E-16
ATP7A	CMPK1	0.5665978	1.67E-16
ATP7A	COL4A1	0.56660149	1.67E-16
ATP7A	KDM6B	0.56674715	1.63E-16
ATP7A	U2AF2	0.56698063	1.58E-16
ATP7A	RPA1	0.56705863	1.56E-16
ATP7A	PRKAG2	0.56710886	1.55E-16
ATP7A	SSB	0.56716498	1.54E-16
ATP7A	ATP5MF-PTCD1	0.56718857	1.53E-16
ATP7A	GASK1B	0.56732301	1.5E-16
ATP7A	FAS	0.56738725	1.49E-16
ATP7A	FAM120AOS	0.56756413	1.45E-16
ATP7A	EDC3	0.56794068	1.37E-16
ATP7A	ZNF532	0.56803078	1.35E-16
ATP7A	TMEM185B	0.56804825	1.35E-16
ATP7A	LYN	0.56808933	1.34E-16
ATP7A	SPIN4	0.5680955	1.34E-16
ATP7A	KIT	0.56864087	1.23E-16
ATP7A	STX3	0.56866527	1.23E-16
ATP7A	RAD18	0.56899617	1.17E-16
ATP7A	CDC42SE1	0.56900463	1.17E-16
ATP7A	LATS2	0.56916498	1.14E-16
ATP7A	PIGX	0.56924966	1.12E-16
ATP7A	DENR	0.56931446	1.11E-16

ATP7A	AKTIP	0.56945472	1.09E-16
ATP7A	UBE2D1	0.56955167	1.07E-16
ATP7A	ITGA4	0.56973616	1.05E-16
ATP7A	KCTD12	0.56977723	1.04E-16
ATP7A	SNAPC1	0.5698138	1.03E-16
ATP7A	TRAM2	0.57006398	9.95E-17
ATP7A	NCBP2	0.57015602	9.82E-17
ATP7A	LIPA	0.5702911	9.62E-17
ATP7A	JAG1	0.57034401	9.54E-17
ATP7A	SH3PXD2A	0.57034783	9.54E-17
ATP7A	YPEL5	0.57046358	9.37E-17
ATP7A	ABCC1	0.5706145	9.16E-17
ATP7A	KLF12	0.57070144	9.04E-17
ATP7A	HARS2	0.57072532	9.01E-17
ATP7A	PIP4K2B	0.57077063	8.95E-17
ATP7A	SORT1	0.57091387	8.76E-17
ATP7A	EMC2	0.57091997	8.75E-17
ATP7A	EAPP	0.57097044	8.68E-17
ATP7A	WAPL	0.57119281	8.4E-17
ATP7A	OXSR1	0.57124989	8.33E-17
ATP7A	PPTC7	0.57147386	8.05E-17
ATP7A	TM2D1	0.57164065	7.85E-17
ATP7A	ZNF200	0.57171261	7.76E-17
ATP7A	RNF24	0.57179913	7.66E-17
ATP7A	SMIM13	0.57214782	7.27E-17
ATP7A	AGA	0.57224625	7.16E-17
ATP7A	B3GALNT1	0.57242357	6.97E-17
ATP7A	TMED8	0.5724482	6.95E-17
ATP7A	RASGEF1B	0.57251208	6.88E-17
ATP7A	PIK3CG	0.57253133	6.86E-17
ATP7A	MINPP1	0.57255632	6.83E-17
ATP7A	SLC31A1	0.5726295	6.76E-17
ATP7A	ARF6	0.57289289	6.49E-17
ATP7A	PLEKHG1	0.57313971	6.25E-17
ATP7A	CASP3	0.5732642	6.14E-17
ATP7A	KLHL23	0.57350834	5.91E-17
ATP7A	NDEL1	0.57399176	5.49E-17
ATP7A	MICU1	0.57403963	5.45E-17
ATP7A	ZFAND6	0.57438672	5.17E-17
ATP7A	UBE2Q2	0.57459187	5.01E-17
ATP7A	NID1	0.57474744	4.9E-17
ATP7A	GHITM	0.57500753	4.7E-17
ATP7A	IFNGR1	0.57516002	4.6E-17
ATP7A	ZHX2	0.57531123	4.49E-17
ATP7A	ARFGEF1	0.57538046	4.44E-17
ATP7A	LRRC8D	0.57545266	4.39E-17
ATP7A	PRKCA	0.57567395	4.25E-17
ATP7A	CPOX	0.57592877	4.09E-17
ATP7A	NCK1	0.57597348	4.06E-17
ATP7A	SH3BGRL2	0.57601748	4.03E-17
ATP7A	ITGA2	0.57602679	4.02E-17
ATP7A	VBP1	0.57603423	4.02E-17
ATP7A	E2F3	0.57608187	3.99E-17
ATP7A	COPB1	0.57612718	3.96E-17
ATP7A	UGCG	0.57629272	3.86E-17
ATP7A	TDP1	0.57635651	3.83E-17
ATP7A	KDM2A	0.5764244	3.79E-17
ATP7A	FOXP1	0.57652088	3.73E-17

ATP7A	CBX1	0.57655696	3.71E-17
ATP7A	SHROOM3	0.57657512	3.7E-17
ATP7A	SNX1	0.57665849	3.65E-17
ATP7A	KCTD21	0.57676712	3.59E-17
ATP7A	PRR14L	0.57682142	3.56E-17
ATP7A	TTC39B	0.57684757	3.55E-17
ATP7A	ATPSCKMT	0.5771233	3.4E-17
ATP7A	CXorf38	0.57712784	3.4E-17
ATP7A	SH3GLB1	0.57728873	3.31E-17
ATP7A	SLC4A1AP	0.57733644	3.29E-17
ATP7A	MRPL49	0.57744715	3.23E-17
ATP7A	PLS1	0.57763335	3.14E-17
ATP7A	ITM2B	0.57767363	3.12E-17
ATP7A	AKIRIN1	0.57779339	3.07E-17
ATP7A	PPP3CB	0.57783412	3.05E-17
ATP7A	LBR	0.57800058	2.97E-17
ATP7A	TAF7	0.57801094	2.97E-17
ATP7A	ARL8B	0.57819831	2.88E-17
ATP7A	CUL1	0.57851551	2.74E-17
ATP7A	LIMS1	0.57854234	2.73E-17
ATP7A	ETV1	0.57861019	2.7E-17
ATP7A	TCF20	0.57888798	2.59E-17
ATP7A	GATAD2A	0.57907031	2.52E-17
ATP7A	COPS8	0.5790945	2.51E-17
ATP7A	PSMC2	0.57911063	2.5E-17
ATP7A	MAP4K4	0.57947922	2.36E-17
ATP7A	DNAL1	0.57949587	2.36E-17
ATP7A	TDRD7	0.57966071	2.3E-17
ATP7A	C2CD3	0.57975658	2.26E-17
ATP7A	TFDP1	0.57985178	2.23E-17
ATP7A	HSP90AA1	0.579929	2.2E-17
ATP7A	SPATA18	0.58005667	2.16E-17
ATP7A	RPGRIP1L	0.5803058	2.08E-17
ATP7A	PLS3	0.58049332	2.02E-17
ATP7A	SOGA1	0.58070749	1.95E-17
ATP7A	CEP170	0.58072957	1.94E-17
ATP7A	MFSD6	0.58119223	1.81E-17
ATP7A	BBS7	0.58125196	1.79E-17
ATP7A	FER	0.58140705	1.75E-17
ATP7A	STS	0.58143917	1.74E-17
ATP7A	SF3A3	0.58161283	1.69E-17
ATP7A	SDHC	0.58169745	1.67E-17
ATP7A	DOP1B	0.58184833	1.63E-17
ATP7A	SERTAD2	0.58187287	1.63E-17
ATP7A	ZDHHC9	0.58194575	1.61E-17
ATP7A	ARL2BP	0.58207305	1.58E-17
ATP7A	BBS9	0.58207619	1.58E-17
ATP7A	PRPF4	0.58218465	1.55E-17
ATP7A	IMPACT	0.58237627	1.5E-17
ATP7A	COX15	0.58241198	1.5E-17
ATP7A	KIF13B	0.58261598	1.45E-17
ATP7A	XYLT1	0.58267931	1.43E-17
ATP7A	POT1	0.58277731	1.41E-17
ATP7A	LRP12	0.5828292	1.4E-17
ATP7A	PGM2L1	0.58285299	1.4E-17
ATP7A	STEAP2	0.58316113	1.33E-17
ATP7A	VPS26A	0.58330812	1.3E-17
ATP7A	USP6NL	0.58335823	1.29E-17

ATP7A	CPED1	0.58351722	1.26E-17
ATP7A	FAM220A	0.58361146	1.24E-17
ATP7A	ANKS1A	0.5836171	1.24E-17
ATP7A	GNPDA1	0.58367708	1.23E-17
ATP7A	IWS1	0.58376171	1.21E-17
ATP7A	PPP2R2A	0.5837963	1.2E-17
ATP7A	RTN4	0.58402593	1.16E-17
ATP7A	EHD4	0.58416871	1.13E-17
ATP7A	PALLD	0.58424952	1.12E-17
ATP7A	CCDC90B	0.58426032	1.12E-17
ATP7A	ARL6IP1	0.58430605	1.11E-17
ATP7A	ANTXR2	0.58430944	1.11E-17
ATP7A	MBIP	0.5843601	1.1E-17
ATP7A	HIVEP1	0.5844386	1.09E-17
ATP7A	CTBP2	0.58444111	1.09E-17
ATP7A	MITF	0.58464507	1.05E-17
ATP7A	PRPF18	0.58471845	1.04E-17
ATP7A	BRI3BP	0.58479743	1.03E-17
ATP7A	FKBP9	0.58480966	1.02E-17
ATP7A	SLC39A6	0.58517531	9.67E-18
ATP7A	ADO	0.58523493	9.58E-18
ATP7A	PTPN4	0.585377	9.37E-18
ATP7A	SIPA1L2	0.58540543	9.33E-18
ATP7A	SAMD12	0.58545543	9.25E-18
ATP7A	AKT1	0.58583633	8.71E-18
ATP7A	KPNB1	0.58602645	8.45E-18
ATP7A	SINHCAF	0.58605976	8.41E-18
ATP7A	CMTM6	0.58606508	8.4E-18
ATP7A	RALBP1	0.5860713	8.39E-18
ATP7A	CLDN12	0.58608182	8.38E-18
ATP7A	VWA5A	0.58632005	8.07E-18
ATP7A	EHF	0.5863423	8.04E-18
ATP7A	LEO1	0.58637654	8E-18
ATP7A	TBC1D9	0.58676691	7.51E-18
ATP7A	MCC	0.58685696	7.41E-18
ATP7A	UBE2Q1	0.58695273	7.3E-18
ATP7A	GPR137B	0.58705556	7.18E-18
ATP7A	AC010132.3	0.58724574	6.96E-18
ATP7A	MOB4	0.58725466	6.95E-18
ATP7A	TRA2B	0.58730004	6.9E-18
ATP7A	SGPP2	0.58750293	6.68E-18
ATP7A	NOCT	0.58759723	6.58E-18
ATP7A	BMS1	0.58762956	6.55E-18
ATP7A	PTPRK	0.58773587	6.44E-18
ATP7A	ADH5	0.58781304	6.36E-18
ATP7A	FXR1	0.58785539	6.32E-18
ATP7A	WDR43	0.587868	6.3E-18
ATP7A	TCF4	0.58799257	6.18E-18
ATP7A	SETX	0.58810543	6.07E-18
ATP7A	PCDH18	0.58812453	6.05E-18
ATP7A	ABL1	0.58823936	5.94E-18
ATP7A	CHSY1	0.58830368	5.88E-18
ATP7A	OGDH	0.58835979	5.83E-18
ATP7A	OAT	0.58849923	5.7E-18
ATP7A	VTI1A	0.58858387	5.62E-18
ATP7A	DCTD	0.58884569	5.39E-18
ATP7A	TULP3	0.58886788	5.37E-18
ATP7A	RASSF8	0.58900966	5.25E-18

ATP7A	GRSF1	0.58911221	5.17E-18
ATP7A	HMGXB3	0.58912091	5.16E-18
ATP7A	LAPTM4A	0.58917898	5.11E-18
ATP7A	PGRMC1	0.58950951	4.85E-18
ATP7A	SYPL1	0.58953554	4.83E-18
ATP7A	PCGF5	0.58955862	4.81E-18
ATP7A	SPTLC2	0.58958701	4.79E-18
ATP7A	TOR1AIP2	0.58965033	4.74E-18
ATP7A	TPD52	0.58972719	4.68E-18
ATP7A	MOB3B	0.5898444	4.59E-18
ATP7A	RFX5	0.58987312	4.57E-18
ATP7A	KATNBL1	0.58989279	4.56E-18
ATP7A	GAS2L3	0.58993494	4.53E-18
ATP7A	AP003108.2	0.59013787	4.38E-18
ATP7A	COQ7	0.59014687	4.37E-18
ATP7A	MICU2	0.59018129	4.35E-18
ATP7A	FAM118B	0.59027404	4.29E-18
ATP7A	BCAP29	0.59030632	4.26E-18
ATP7A	YAP1	0.59044172	4.17E-18
ATP7A	COA7	0.59076678	3.96E-18
ATP7A	SLMAP	0.59089131	3.88E-18
ATP7A	DSG2	0.5909204	3.86E-18
ATP7A	DEDD	0.59095347	3.84E-18
ATP7A	KCTD10	0.5909993	3.81E-18
ATP7A	RPAP3	0.59111021	3.74E-18
ATP7A	GUCY1A1	0.59115342	3.72E-18
ATP7A	GLUD1	0.59134678	3.6E-18
ATP7A	EPB41L2	0.59139096	3.58E-18
ATP7A	RAP1B	0.59144125	3.55E-18
ATP7A	GSR	0.5916353	3.44E-18
ATP7A	PPHLN1	0.59164967	3.43E-18
ATP7A	COMMD8	0.5917183	3.39E-18
ATP7A	PLRG1	0.59194246	3.27E-18
ATP7A	HNRNPA2B1	0.59226737	3.11E-18
ATP7A	UBE2D2	0.59238797	3.05E-18
ATP7A	CTNNB1	0.5924403	3.02E-18
ATP7A	RMND5A	0.5925004	2.99E-18
ATP7A	MBTPS1	0.59277304	2.86E-18
ATP7A	TMBIM6	0.59281723	2.84E-18
ATP7A	PRRG1	0.59283227	2.83E-18
ATP7A	CNOT9	0.59284302	2.83E-18
ATP7A	EXOC4	0.592845	2.83E-18
ATP7A	VAMP3	0.59289503	2.8E-18
ATP7A	PPP2R5D	0.59316673	2.68E-18
ATP7A	ACTR6	0.59323829	2.65E-18
ATP7A	MPHOSPH10	0.59337371	2.59E-18
ATP7A	PIGS	0.59351188	2.54E-18
ATP7A	UBLCP1	0.5936819	2.47E-18
ATP7A	RAB29	0.59376357	2.43E-18
ATP7A	FBXO45	0.59401879	2.34E-18
ATP7A	ZNF644	0.59445025	2.18E-18
ATP7A	SPDL1	0.59446342	2.17E-18
ATP7A	HSD17B11	0.59465326	2.11E-18
ATP7A	DCTN6	0.59476249	2.07E-18
ATP7A	ADCY7	0.5947626	2.07E-18
ATP7A	YWHAQ	0.59490113	2.02E-18
ATP7A	APAF1	0.59500323	1.99E-18
ATP7A	PLEKHF2	0.59506156	1.97E-18

ATP7A	C4orf3	0.59514869	1.94E-18
ATP7A	ZNFX1	0.59535381	1.88E-18
ATP7A	RAB5C	0.5954043	1.86E-18
ATP7A	POLR2D	0.595544	1.82E-18
ATP7A	NUDCD1	0.59578707	1.75E-18
ATP7A	SSX2IP	0.59584252	1.73E-18
ATP7A	GALNT1	0.59596376	1.7E-18
ATP7A	DCTN5	0.5960904	1.66E-18
ATP7A	NAA16	0.59626047	1.62E-18
ATP7A	PAFAH1B2	0.59645932	1.57E-18
ATP7A	ATL3	0.59676479	1.49E-18
ATP7A	ITFG1	0.59714765	1.4E-18
ATP7A	KRCC1	0.59763428	1.29E-18
ATP7A	KATNAL1	0.59792682	1.23E-18
ATP7A	ACER3	0.59801404	1.21E-18
ATP7A	SUFU	0.59814355	1.19E-18
ATP7A	RFWD3	0.59815153	1.18E-18
ATP7A	TAF9B	0.59820024	1.17E-18
ATP7A	MANBA	0.59826143	1.16E-18
ATP7A	PIAS1	0.59892144	1.04E-18
ATP7A	NECTIN3	0.59907739	1.02E-18
ATP7A	LARP4B	0.59909172	1.01E-18
ATP7A	MYO1D	0.59914091	1E-18
ATP7A	TSPYL1	0.5991827	9.98E-19
ATP7A	YEATS2	0.59922032	9.92E-19
ATP7A	HACD3	0.59934681	9.71E-19
ATP7A	DAG1	0.60005363	8.63E-19
ATP7A	PRELID3B	0.60016532	8.47E-19
ATP7A	TARDBP	0.60020056	8.42E-19
ATP7A	TP53BP2	0.60035506	8.21E-19
ATP7A	URI1	0.60045766	8.07E-19
ATP7A	PACRGL	0.60077604	7.65E-19
ATP7A	TNRC18	0.60100619	7.36E-19
ATP7A	ZDHHC5	0.60106104	7.3E-19
ATP7A	NUP62	0.6010815	7.27E-19
ATP7A	OTUD1	0.60139155	6.9E-19
ATP7A	NAMPT	0.60149272	6.79E-19
ATP7A	SIPA1L3	0.60150826	6.77E-19
ATP7A	PFKM	0.60178192	6.47E-19
ATP7A	NUB1	0.60187694	6.36E-19
ATP7A	RAB35	0.60199221	6.24E-19
ATP7A	SKIL	0.60204343	6.19E-19
ATP7A	EXOC2	0.60224094	5.99E-19
ATP7A	FRY	0.60241603	5.81E-19
ATP7A	TPP2	0.60259371	5.64E-19
ATP7A	CHMP1B	0.60266839	5.57E-19
ATP7A	MSH2	0.60316033	5.13E-19
ATP7A	MORF4L1	0.60324497	5.06E-19
ATP7A	WASHC2A	0.60333225	4.98E-19
ATP7A	AGAP1	0.6033358	4.98E-19
ATP7A	KHDRBS1	0.60346414	4.87E-19
ATP7A	GCC1	0.60347204	4.87E-19
ATP7A	RNF145	0.60388917	4.54E-19
ATP7A	TCEA1	0.60389969	4.53E-19
ATP7A	GDE1	0.60391249	4.52E-19
ATP7A	PLBD2	0.60409334	4.38E-19
ATP7A	RAB28	0.60410199	4.38E-19
ATP7A	TMEM87A	0.60419256	4.31E-19

ATP7A	TEX10	0.60420888	4.3E-19
ATP7A	ZKSCAN5	0.60426407	4.26E-19
ATP7A	PHACTR4	0.60442833	4.14E-19
ATP7A	SNW1	0.60448988	4.1E-19
ATP7A	SELENOF	0.60449917	4.09E-19
ATP7A	SRSF3	0.60452255	4.08E-19
ATP7A	UBE2A	0.60463738	4E-19
ATP7A	TRPS1	0.6047433	3.93E-19
ATP7A	BAG4	0.60477329	3.91E-19
ATP7A	SLK	0.60514672	3.67E-19
ATP7A	SYNJ2	0.60531938	3.56E-19
ATP7A	HIP1	0.60535703	3.54E-19
ATP7A	DPYD	0.60581102	3.28E-19
ATP7A	SIAE	0.60603049	3.16E-19
ATP7A	UBE2N	0.6060389	3.15E-19
ATP7A	NRAS	0.6062253	3.05E-19
ATP7A	PAFAH1B1	0.60624505	3.04E-19
ATP7A	TM9SF4	0.60633189	3E-19
ATP7A	PSMD10	0.60636236	2.98E-19
ATP7A	DERL1	0.60648052	2.92E-19
ATP7A	LAMC1	0.60652658	2.9E-19
ATP7A	FAM114A1	0.60658513	2.87E-19
ATP7A	LYST	0.60669244	2.82E-19
ATP7A	OTULIN	0.6068527	2.74E-19
ATP7A	ENTPD5	0.60690646	2.72E-19
ATP7A	GOLGA7	0.60697504	2.69E-19
ATP7A	ITGA1	0.60712816	2.62E-19
ATP7A	NRBF2	0.60724334	2.57E-19
ATP7A	FYCO1	0.60732217	2.53E-19
ATP7A	DCUN1D3	0.60753057	2.44E-19
ATP7A	CYFIP1	0.60753251	2.44E-19
ATP7A	ADGRF5	0.60758008	2.42E-19
ATP7A	RRP15	0.60765673	2.39E-19
ATP7A	CALM1	0.60769464	2.37E-19
ATP7A	NLN	0.60808514	2.22E-19
ATP7A	INO80	0.60808737	2.22E-19
ATP7A	RAB1A	0.60815037	2.2E-19
ATP7A	ZBTB4	0.60827286	2.15E-19
ATP7A	TSPAN31	0.60835107	2.12E-19
ATP7A	RAP1A	0.6086648	2.01E-19
ATP7A	GPALPP1	0.60879623	1.97E-19
ATP7A	LINS1	0.60879983	1.96E-19
ATP7A	ARFIP1	0.60882457	1.96E-19
ATP7A	OCIAD1	0.60889484	1.93E-19
ATP7A	ITGB1	0.6089457	1.92E-19
ATP7A	HNRNPF	0.60896648	1.91E-19
ATP7A	FRMD4B	0.60901896	1.89E-19
ATP7A	PATL1	0.60918297	1.84E-19
ATP7A	STARD3NL	0.60934663	1.79E-19
ATP7A	ARL6IP6	0.60941437	1.77E-19
ATP7A	ENOPH1	0.60941997	1.77E-19
ATP7A	BTBD10	0.60972212	1.68E-19
ATP7A	GALK2	0.60977173	1.66E-19
ATP7A	SPIRE1	0.60995414	1.61E-19
ATP7A	RFK	0.61042108	1.49E-19
ATP7A	CREBL2	0.61057791	1.45E-19
ATP7A	PTK2	0.61074385	1.41E-19
ATP7A	GMEB1	0.61084749	1.38E-19

ATP7A	PCNX1	0.61085268	1.38E-19
ATP7A	ATL2	0.61086813	1.38E-19
ATP7A	PRKACA	0.61088561	1.37E-19
ATP7A	BPNT1	0.61095087	1.36E-19
ATP7A	FAM210A	0.61114665	1.31E-19
ATP7A	FUT8	0.61143587	1.25E-19
ATP7A	UGP2	0.61166797	1.2E-19
ATP7A	ZMAT2	0.61175806	1.18E-19
ATP7A	DYNLT3	0.61182173	1.17E-19
ATP7A	PDCD6IP	0.61187391	1.16E-19
ATP7A	AC008764.4	0.61193604	1.14E-19
ATP7A	RDH14	0.61214931	1.1E-19
ATP7A	CPNE8	0.61226171	1.08E-19
ATP7A	CUL4A	0.6126113	1.02E-19
ATP7A	CBX5	0.6129153	9.64E-20
ATP7A	TLR3	0.61294483	9.59E-20
ATP7A	ZNF410	0.61297779	9.53E-20
ATP7A	AMMECR1	0.61327576	9.05E-20
ATP7A	MFSD14B	0.61333257	8.96E-20
ATP7A	NR2F2	0.61337064	8.9E-20
ATP7A	SET	0.6138521	8.18E-20
ATP7A	ZFAND5	0.61390451	8.11E-20
ATP7A	FRYL	0.61396701	8.02E-20
ATP7A	AFG3L2	0.61402389	7.94E-20
ATP7A	HERC3	0.61415187	7.76E-20
ATP7A	TMLHE	0.61430199	7.56E-20
ATP7A	PTDSS1	0.61446574	7.35E-20
ATP7A	EPS8	0.61476853	6.97E-20
ATP7A	DHDDS	0.61489445	6.82E-20
ATP7A	CHRAC1	0.61507765	6.6E-20
ATP7A	KLF3	0.61523024	6.43E-20
ATP7A	BBS12	0.61526567	6.39E-20
ATP7A	FAM204A	0.61533513	6.31E-20
ATP7A	NAV1	0.61535119	6.29E-20
ATP7A	VPS37A	0.61551957	6.11E-20
ATP7A	OSBPL1A	0.6156706	5.95E-20
ATP7A	KITLG	0.61575097	5.86E-20
ATP7A	NUCKS1	0.6158981	5.71E-20
ATP7A	LPIN2	0.61593505	5.68E-20
ATP7A	CDC42	0.61595163	5.66E-20
ATP7A	NUP50	0.61602496	5.59E-20
ATP7A	BTN2A1	0.61612973	5.48E-20
ATP7A	MOSPD1	0.61637695	5.25E-20
ATP7A	DHX36	0.61640496	5.22E-20
ATP7A	BLZF1	0.61676042	4.91E-20
ATP7A	LZIC	0.61682696	4.85E-20
ATP7A	PNPO	0.61685055	4.83E-20
ATP7A	FASTKD2	0.61685736	4.82E-20
ATP7A	KRR1	0.61697404	4.72E-20
ATP7A	FAM83B	0.61702319	4.68E-20
ATP7A	DIP2C	0.61711887	4.61E-20
ATP7A	CXorf56	0.61719085	4.55E-20
ATP7A	CHMP2B	0.61728131	4.47E-20
ATP7A	POC1B	0.61737064	4.4E-20
ATP7A	DNM1L	0.61737768	4.4E-20
ATP7A	FZD6	0.61743513	4.35E-20
ATP7A	KCTD3	0.61747135	4.33E-20
ATP7A	RNF121	0.61779812	4.08E-20

ATP7A	RIN2	0.61795952	3.97E-20
ATP7A	KDM5A	0.61808166	3.88E-20
ATP7A	TNIK	0.61816229	3.83E-20
ATP7A	NFE2L1	0.61816277	3.83E-20
ATP7A	TMEM263	0.61843155	3.65E-20
ATP7A	SNX9	0.6184651	3.63E-20
ATP7A	F2R	0.6185909	3.55E-20
ATP7A	SIAH1	0.6186471	3.51E-20
ATP7A	SKP1	0.61884455	3.39E-20
ATP7A	SRP9	0.61886577	3.38E-20
ATP7A	TMEM19	0.61888531	3.37E-20
ATP7A	ANAPC10	0.61889933	3.36E-20
ATP7A	SRGAP2C	0.61912674	3.22E-20
ATP7A	KTN1	0.61928567	3.13E-20
ATP7A	AASS	0.61932776	3.11E-20
ATP7A	SLC24A1	0.61951249	3.01E-20
ATP7A	POLR2C	0.61958928	2.97E-20
ATP7A	BLOC1S5	0.61966076	2.93E-20
ATP7A	TEFM	0.61972347	2.9E-20
ATP7A	EID1	0.61972549	2.9E-20
ATP7A	MAP2K1	0.61972905	2.9E-20
ATP7A	ZFP64	0.61989029	2.81E-20
ATP7A	HEG1	0.62000834	2.75E-20
ATP7A	HSD17B12	0.62002008	2.75E-20
ATP7A	LIN52	0.62009628	2.71E-20
ATP7A	SDCBP	0.62029301	2.62E-20
ATP7A	UBA3	0.62052191	2.51E-20
ATP7A	UTP25	0.62063694	2.46E-20
ATP7A	OSGIN2	0.62080343	2.39E-20
ATP7A	TNPO3	0.62087129	2.36E-20
ATP7A	NOTCH2	0.62094912	2.33E-20
ATP7A	RAB22A	0.62096052	2.32E-20
ATP7A	KIF3A	0.62105307	2.28E-20
ATP7A	HHAT	0.6211708	2.24E-20
ATP7A	SCP2	0.62119353	2.23E-20
ATP7A	ATP6AP2	0.62147418	2.12E-20
ATP7A	DLST	0.62157892	2.08E-20
ATP7A	ITSN1	0.62168126	2.04E-20
ATP7A	STK26	0.62175143	2.01E-20
ATP7A	SPATS2	0.62183383	1.98E-20
ATP7A	MIS18BP1	0.62284598	1.65E-20
ATP7A	MOB1A	0.62291081	1.63E-20
ATP7A	PELI1	0.62297914	1.61E-20
ATP7A	TYW1	0.62306196	1.59E-20
ATP7A	AP2B1	0.62315835	1.56E-20
ATP7A	ALDH9A1	0.62327156	1.53E-20
ATP7A	PTPRJ	0.6236286	1.44E-20
ATP7A	SOAT1	0.62399087	1.34E-20
ATP7A	IFT57	0.62416431	1.3E-20
ATP7A	RNASEL	0.62425923	1.28E-20
ATP7A	ZBTB2	0.62426031	1.28E-20
ATP7A	TOB2	0.62459538	1.2E-20
ATP7A	TMBIM4	0.62466698	1.19E-20
ATP7A	SMC2	0.62506065	1.11E-20
ATP7A	RANBP9	0.62511409	1.1E-20
ATP7A	ZMYM5	0.62520854	1.08E-20
ATP7A	MSL2	0.62527625	1.06E-20
ATP7A	TMED10	0.62536442	1.05E-20

ATP7A	REEP3	0.62543548	1.03E-20
ATP7A	RAD1	0.62557676	1.01E-20
ATP7A	LPP	0.6255842	1.01E-20
ATP7A	EPHA3	0.62585845	9.57E-21
ATP7A	USP10	0.62611541	9.13E-21
ATP7A	OSBPL10	0.6262449	8.92E-21
ATP7A	BROX	0.62628974	8.84E-21
ATP7A	ZNF263	0.62629983	8.83E-21
ATP7A	TMEM209	0.62642978	8.62E-21
ATP7A	EXT1	0.62679481	8.06E-21
ATP7A	C1GALT1C1	0.62691157	7.89E-21
ATP7A	NAA50	0.62697484	7.8E-21
ATP7A	LARS2	0.62704534	7.7E-21
ATP7A	SGPL1	0.62726695	7.39E-21
ATP7A	GCNT1	0.62729711	7.35E-21
ATP7A	SLC30A5	0.62779515	6.71E-21
ATP7A	MAPRE1	0.6279525	6.52E-21
ATP7A	FLT1	0.62797042	6.5E-21
ATP7A	KDM5B	0.62805364	6.4E-21
ATP7A	C16orf70	0.62812978	6.31E-21
ATP7A	HOMER1	0.62822423	6.2E-21
ATP7A	YWHAB	0.62831818	6.09E-21
ATP7A	NRP1	0.6283259	6.08E-21
ATP7A	C9orf64	0.62833219	6.08E-21
ATP7A	EPB41L4A	0.62836128	6.05E-21
ATP7A	LPGAT1	0.62863935	5.74E-21
ATP7A	LDB1	0.62880049	5.57E-21
ATP7A	ZEB1	0.62889936	5.47E-21
ATP7A	ITPRIPL2	0.62893334	5.44E-21
ATP7A	TMEM33	0.62899129	5.38E-21
ATP7A	AIDA	0.62910106	5.27E-21
ATP7A	TMCO1	0.62924696	5.13E-21
ATP7A	WBP11	0.62927438	5.11E-21
ATP7A	ZFYVE1	0.6293162	5.07E-21
ATP7A	ARL15	0.62934486	5.04E-21
ATP7A	RBM47	0.62941798	4.97E-21
ATP7A	MPZL1	0.62952295	4.88E-21
ATP7A	TERF2	0.6298175	4.62E-21
ATP7A	VAPA	0.62987055	4.57E-21
ATP7A	PDP1	0.62987514	4.57E-21
ATP7A	SS18	0.62991819	4.53E-21
ATP7A	RCOR1	0.62998233	4.48E-21
ATP7A	ELK4	0.63054157	4.04E-21
ATP7A	GBE1	0.63064814	3.96E-21
ATP7A	RBM22	0.63069485	3.92E-21
ATP7A	C3orf38	0.63069854	3.92E-21
ATP7A	CAST	0.6307612	3.88E-21
ATP7A	ZFP90	0.63093	3.76E-21
ATP7A	CSDE1	0.63101546	3.7E-21
ATP7A	TGFBR2	0.63106618	3.66E-21
ATP7A	IRF2BP2	0.63122893	3.55E-21
ATP7A	APOL6	0.63127473	3.52E-21
ATP7A	SPOP	0.63130428	3.5E-21
ATP7A	MAPRE2	0.63147277	3.4E-21
ATP7A	KLHL7	0.63150832	3.37E-21
ATP7A	INCENP	0.63154106	3.35E-21
ATP7A	FKBP15	0.631862	3.16E-21
ATP7A	VHL	0.63189621	3.14E-21

ATP7A	SLC41A2	0.6319459	3.11E-21
ATP7A	DUSP11	0.63196235	3.1E-21
ATP7A	SPCS2	0.63208828	3.03E-21
ATP7A	TSN	0.63234559	2.89E-21
ATP7A	EIF2S1	0.63259144	2.76E-21
ATP7A	ZNF398	0.63269417	2.7E-21
ATP7A	KDM4A	0.63289595	2.6E-21
ATP7A	ABHD17B	0.63295237	2.58E-21
ATP7A	RPRD1B	0.63321334	2.45E-21
ATP7A	SMG7	0.63335479	2.39E-21
ATP7A	RASA1	0.6336391	2.26E-21
ATP7A	NR1D2	0.63369468	2.24E-21
ATP7A	GALNT7	0.63382968	2.18E-21
ATP7A	SLC26A2	0.634297	2E-21
ATP7A	PGM2	0.63440969	1.96E-21
ATP7A	TMEM68	0.63480619	1.82E-21
ATP7A	LYRM2	0.63483296	1.81E-21
ATP7A	TFCP2	0.63488122	1.79E-21
ATP7A	LRRFIP1	0.63499337	1.75E-21
ATP7A	AGFG1	0.63508739	1.72E-21
ATP7A	RDH11	0.63524654	1.67E-21
ATP7A	LCLAT1	0.63533962	1.64E-21
ATP7A	HNRNPUL1	0.63556286	1.58E-21
ATP7A	SP2	0.63556399	1.58E-21
ATP7A	KBTBD2	0.63566454	1.55E-21
ATP7A	ZNF720	0.635762	1.52E-21
ATP7A	CASK	0.63585142	1.49E-21
ATP7A	GCLM	0.63597197	1.46E-21
ATP7A	PARP4	0.63601517	1.45E-21
ATP7A	MRPL19	0.63625288	1.38E-21
ATP7A	CDC42BPA	0.6363224	1.36E-21
ATP7A	PCNX4	0.63643102	1.34E-21
ATP7A	ZNF143	0.63650239	1.32E-21
ATP7A	CDKL1	0.63671207	1.27E-21
ATP7A	TRIM2	0.63681281	1.24E-21
ATP7A	CDYL	0.63713978	1.17E-21
ATP7A	ITGAV	0.63733382	1.13E-21
ATP7A	TMEM248	0.63734152	1.12E-21
ATP7A	GPR107	0.63734303	1.12E-21
ATP7A	RIT1	0.63735312	1.12E-21
ATP7A	TTC26	0.63760892	1.07E-21
ATP7A	FAM98A	0.63769215	1.05E-21
ATP7A	IST1	0.63771332	1.05E-21
ATP7A	KIAA1143	0.63800095	9.91E-22
ATP7A	FRRS1	0.63811932	9.69E-22
ATP7A	UBN1	0.6381421	9.65E-22
ATP7A	TCTN3	0.63814919	9.64E-22
ATP7A	PRUNE1	0.63834615	9.28E-22
ATP7A	CERS2	0.63845201	9.09E-22
ATP7A	SWAP70	0.63856274	8.9E-22
ATP7A	RAB2A	0.63865336	8.75E-22
ATP7A	NFIX	0.63868768	8.69E-22
ATP7A	SEL1L	0.63892835	8.3E-22
ATP7A	HEATR5A	0.63920201	7.88E-22
ATP7A	ADAM9	0.63933849	7.67E-22
ATP7A	TLE4	0.639378	7.62E-22
ATP7A	TGFBR1	0.63943713	7.53E-22
ATP7A	LRRC57	0.63957821	7.33E-22

ATP7A	PTPN12	0.63984739	6.96E-22
ATP7A	CREB3L2	0.64029534	6.38E-22
ATP7A	IL7	0.640377	6.29E-22
ATP7A	DPY19L1	0.64041035	6.24E-22
ATP7A	ZNHIT6	0.6405337	6.1E-22
ATP7A	HMGN4	0.64055536	6.07E-22
ATP7A	BCL2L13	0.64059571	6.03E-22
ATP7A	LDAH	0.640708	5.9E-22
ATP7A	ABHD2	0.64090356	5.68E-22
ATP7A	FCHSD2	0.64092912	5.65E-22
ATP7A	RSPH3	0.64094059	5.64E-22
ATP7A	GAB2	0.64098859	5.59E-22
ATP7A	TRANK1	0.64112317	5.44E-22
ATP7A	KIFAP3	0.64112962	5.44E-22
ATP7A	CD164	0.64115024	5.41E-22
ATP7A	TRIM56	0.64127446	5.29E-22
ATP7A	TAX1BP1	0.64135736	5.2E-22
ATP7A	ZMAT3	0.64144217	5.12E-22
ATP7A	SLC35A3	0.64146043	5.1E-22
ATP7A	ANKMY2	0.64171539	4.85E-22
ATP7A	TRIM32	0.64210024	4.51E-22
ATP7A	ASAH1	0.64212652	4.48E-22
ATP7A	RALGPS2	0.64239056	4.26E-22
ATP7A	REST	0.64240921	4.24E-22
ATP7A	CHML	0.64266356	4.04E-22
ATP7A	CRCP	0.64286915	3.88E-22
ATP7A	PITPNA	0.64287205	3.88E-22
ATP7A	PDHX	0.64313078	3.69E-22
ATP7A	CISD2	0.64313293	3.69E-22
ATP7A	IGF2R	0.64319256	3.64E-22
ATP7A	LYPLA1	0.64339177	3.51E-22
ATP7A	RNF141	0.6435867	3.37E-22
ATP7A	PSME3	0.64373699	3.28E-22
ATP7A	SKAP2	0.64401469	3.1E-22
ATP7A	ERMP1	0.6440474	3.08E-22
ATP7A	NFKB1	0.64430375	2.93E-22
ATP7A	PTEN	0.64436717	2.9E-22
ATP7A	ACLY	0.64437846	2.89E-22
ATP7A	NAPG	0.64452059	2.81E-22
ATP7A	PTPRB	0.64471942	2.71E-22
ATP7A	RNF170	0.64508882	2.52E-22
ATP7A	RAB5B	0.64510287	2.51E-22
ATP7A	SC5D	0.64530839	2.41E-22
ATP7A	SMURF2	0.64552004	2.31E-22
ATP7A	SESTD1	0.64559565	2.28E-22
ATP7A	ACVR2A	0.6456308	2.26E-22
ATP7A	GPATCH11	0.64584253	2.17E-22
ATP7A	CHMP3	0.64596324	2.12E-22
ATP7A	TMEM127	0.64603649	2.09E-22
ATP7A	HUS1	0.64620991	2.02E-22
ATP7A	USP12	0.64638409	1.95E-22
ATP7A	ZNF462	0.64641973	1.94E-22
ATP7A	PPP1R8	0.64643881	1.93E-22
ATP7A	ARHGAP35	0.64648571	1.91E-22
ATP7A	ARL1	0.64658377	1.88E-22
ATP7A	DYNC1I2	0.64659135	1.87E-22
ATP7A	PTP4A1	0.64659429	1.87E-22
ATP7A	TSC22D2	0.64665811	1.85E-22

ATP7A	TSHZ1	0.64680432	1.8E-22
ATP7A	MAP3K7	0.64692611	1.75E-22
ATP7A	AGPAT5	0.64695182	1.74E-22
ATP7A	ATG5	0.64697145	1.74E-22
ATP7A	RNF168	0.64708406	1.7E-22
ATP7A	FAM98B	0.64725427	1.64E-22
ATP7A	HSPA14	0.64730308	1.63E-22
ATP7A	PSEN1	0.64756408	1.55E-22
ATP7A	TIPRL	0.64766913	1.51E-22
ATP7A	CWF19L2	0.64768665	1.51E-22
ATP7A	PEX13	0.64793644	1.44E-22
ATP7A	SCFD2	0.64857958	1.26E-22
ATP7A	LRCH1	0.64868739	1.24E-22
ATP7A	DAZAP2	0.64879566	1.21E-22
ATP7A	NPM1	0.6488662	1.19E-22
ATP7A	CNOT2	0.64891207	1.18E-22
ATP7A	AIMP1	0.6489365	1.18E-22
ATP7A	CRLF3	0.64912001	1.14E-22
ATP7A	CCNK	0.6492352	1.11E-22
ATP7A	MFSD1	0.64931114	1.09E-22
ATP7A	ANKRD42	0.64945999	1.06E-22
ATP7A	TGS1	0.64950842	1.05E-22
ATP7A	GNS	0.64979043	9.93E-23
ATP7A	DESI2	0.65009992	9.34E-23
ATP7A	QKI	0.6501353	9.27E-23
ATP7A	SLU7	0.65014004	9.26E-23
ATP7A	MARK2	0.65019472	9.16E-23
ATP7A	TAF5L	0.65024899	9.06E-23
ATP7A	NXT2	0.65033959	8.9E-23
ATP7A	SLC6A6	0.65036432	8.86E-23
ATP7A	SNAP23	0.65038818	8.82E-23
ATP7A	TMTC2	0.65052286	8.58E-23
ATP7A	SART3	0.65065979	8.35E-23
ATP7A	GOLT1B	0.65067074	8.33E-23
ATP7A	IQCK	0.65088959	7.97E-23
ATP7A	KLF7	0.650951	7.88E-23
ATP7A	RNF20	0.65110877	7.63E-23
ATP7A	DDX19A	0.6511453	7.58E-23
ATP7A	UBFD1	0.65134372	7.28E-23
ATP7A	SFT2D2	0.65135262	7.27E-23
ATP7A	ARRDC3	0.65139906	7.2E-23
ATP7A	L3MBTL3	0.65156948	6.96E-23
ATP7A	TRERF1	0.65166309	6.83E-23
ATP7A	NT5C2	0.6517801	6.67E-23
ATP7A	KPNA4	0.65194732	6.45E-23
ATP7A	EAF1	0.65225692	6.06E-23
ATP7A	TMCC1	0.65231997	5.98E-23
ATP7A	KCTD20	0.6523553	5.94E-23
ATP7A	CD2AP	0.65257766	5.68E-23
ATP7A	USP14	0.65263119	5.62E-23
ATP7A	NKRF	0.65273926	5.5E-23
ATP7A	HINT3	0.65276167	5.47E-23
ATP7A	ZNF766	0.65281541	5.42E-23
ATP7A	CHORDC1	0.65286718	5.36E-23
ATP7A	CROT	0.65292663	5.3E-23
ATP7A	LRBA	0.65308317	5.13E-23
ATP7A	GRAMD2B	0.65317124	5.04E-23
ATP7A	PI4K2B	0.65327721	4.93E-23

ATP7A	RNF138	0.65333054	4.88E-23
ATP7A	R3HDM1	0.65333283	4.88E-23
ATP7A	SNX2	0.65341198	4.8E-23
ATP7A	PARP8	0.65351379	4.7E-23
ATP7A	RC3H2	0.65357607	4.64E-23
ATP7A	SLC35A4	0.65359127	4.63E-23
ATP7A	WDR3	0.653703	4.53E-23
ATP7A	SLC25A32	0.65395761	4.3E-23
ATP7A	SMG8	0.65418453	4.11E-23
ATP7A	PRKD3	0.65465592	3.73E-23
ATP7A	GOLPH3L	0.65475417	3.66E-23
ATP7A	TRMT2B	0.65479371	3.63E-23
ATP7A	VEZT	0.65502012	3.46E-23
ATP7A	DCAF12	0.65522112	3.33E-23
ATP7A	SYNCRIP	0.65522336	3.32E-23
ATP7A	PNRC2	0.65544542	3.18E-23
ATP7A	BACH1	0.65560954	3.07E-23
ATP7A	DOCK9	0.65587734	2.91E-23
ATP7A	PRPF40A	0.65606469	2.8E-23
ATP7A	GPATCH2	0.65616517	2.74E-23
ATP7A	ATXN7L3B	0.65649735	2.56E-23
ATP7A	CRTC3	0.65660571	2.51E-23
ATP7A	TMEM123	0.65671617	2.45E-23
ATP7A	PAQR3	0.65687381	2.37E-23
ATP7A	CYLD	0.65689005	2.37E-23
ATP7A	CDC42EP3	0.65690988	2.36E-23
ATP7A	FNDC3B	0.65693085	2.35E-23
ATP7A	DENND6A	0.65694922	2.34E-23
ATP7A	NEO1	0.65697763	2.32E-23
ATP7A	KIAA1671	0.65711756	2.26E-23
ATP7A	DOCK1	0.65719745	2.22E-23
ATP7A	TAF1A	0.65757034	2.06E-23
ATP7A	MID1	0.65778824	1.97E-23
ATP7A	SSR1	0.65782575	1.95E-23
ATP7A	RTL6	0.65862388	1.66E-23
ATP7A	PPP2CB	0.65878875	1.6E-23
ATP7A	TANK	0.65886856	1.57E-23
ATP7A	ZNF207	0.65895044	1.55E-23
ATP7A	ZNF134	0.65903222	1.52E-23
ATP7A	APP	0.65919964	1.47E-23
ATP7A	BNIP3L	0.65933123	1.43E-23
ATP7A	ANO6	0.65933278	1.43E-23
ATP7A	SLC25A13	0.65951289	1.38E-23
ATP7A	PIP4P2	0.65951847	1.38E-23
ATP7A	SLC4A7	0.66024328	1.18E-23
ATP7A	RAB8A	0.66059878	1.1E-23
ATP7A	ATP9A	0.66075083	1.07E-23
ATP7A	MEX3C	0.6609796	1.02E-23
ATP7A	INTS12	0.66113202	9.85E-24
ATP7A	ATP2B1	0.66122017	9.67E-24
ATP7A	DLD	0.66176644	8.63E-24
ATP7A	ZDHHC20	0.66191381	8.37E-24
ATP7A	CLN5	0.66191924	8.36E-24
ATP7A	SMARCE1	0.66192351	8.35E-24
ATP7A	CPSF6	0.66215503	7.96E-24
ATP7A	C1GALT1	0.66232102	7.69E-24
ATP7A	SH3RF1	0.66260354	7.24E-24
ATP7A	SLC44A1	0.66266931	7.15E-24

ATP7A	DCAF7	0.66275093	7.02E-24
ATP7A	ASAP1	0.66371286	5.74E-24
ATP7A	UHRF1BP1L	0.66371397	5.74E-24
ATP7A	PPP1CC	0.66387399	5.55E-24
ATP7A	WNK1	0.6639481	5.46E-24
ATP7A	JRKL	0.66409699	5.29E-24
ATP7A	SH3BGRL	0.66428092	5.09E-24
ATP7A	CAPRIN1	0.66433969	5.03E-24
ATP7A	TRAM1	0.6648578	4.51E-24
ATP7A	ETF1	0.66491851	4.45E-24
ATP7A	ATP2C1	0.66502444	4.35E-24
ATP7A	CCNY	0.66528098	4.12E-24
ATP7A	NAB1	0.66551377	3.93E-24
ATP7A	PTBP3	0.66579826	3.7E-24
ATP7A	NR3C1	0.66595577	3.57E-24
ATP7A	MORF4L2	0.66624436	3.36E-24
ATP7A	KPNA6	0.66653988	3.16E-24
ATP7A	EIF1AX	0.66659228	3.12E-24
ATP7A	EIF2AK2	0.66665676	3.08E-24
ATP7A	ZNF217	0.66680652	2.98E-24
ATP7A	SAP130	0.66684296	2.96E-24
ATP7A	BRD3	0.66691464	2.92E-24
ATP7A	NAALADL2	0.66730996	2.68E-24
ATP7A	IRF2	0.66743772	2.61E-24
ATP7A	ANXA7	0.66744493	2.6E-24
ATP7A	CDK17	0.66748621	2.58E-24
ATP7A	TMEM167A	0.66761145	2.51E-24
ATP7A	TMEM182	0.66771511	2.46E-24
ATP7A	PPP3R1	0.66787468	2.38E-24
ATP7A	IL6ST	0.66801378	2.31E-24
ATP7A	APBB2	0.6681679	2.23E-24
ATP7A	ZMPSTE24	0.66830623	2.17E-24
ATP7A	STK3	0.66845781	2.1E-24
ATP7A	SNRNP27	0.66845864	2.1E-24
ATP7A	CSTF2T	0.66850411	2.08E-24
ATP7A	PTPRG	0.668558	2.05E-24
ATP7A	TBL1XR1	0.66867044	2E-24
ATP7A	CDC42BPB	0.66874279	1.97E-24
ATP7A	METTL9	0.66903419	1.85E-24
ATP7A	BPTF	0.66911224	1.82E-24
ATP7A	CD46	0.66912978	1.82E-24
ATP7A	YWHAG	0.66956527	1.65E-24
ATP7A	NSRP1	0.66957856	1.65E-24
ATP7A	JAK2	0.66979458	1.57E-24
ATP7A	NDFIP1	0.66987271	1.55E-24
ATP7A	TM9SF2	0.67006349	1.49E-24
ATP7A	COPS2	0.67020208	1.44E-24
ATP7A	ZC3H15	0.6703556	1.39E-24
ATP7A	C16orf87	0.67048629	1.36E-24
ATP7A	MED20	0.67082071	1.26E-24
ATP7A	LRP10	0.67101448	1.21E-24
ATP7A	COMMD2	0.67115455	1.17E-24
ATP7A	FBXW2	0.67119123	1.16E-24
ATP7A	SMARCC1	0.67131834	1.13E-24
ATP7A	CNOT4	0.67145109	1.1E-24
ATP7A	ANP32E	0.67153916	1.08E-24
ATP7A	ICE2	0.67176457	1.03E-24
ATP7A	DIAPH2	0.67182084	1.02E-24

ATP7A	ATXN1	0.67201193	9.75E-25
ATP7A	IARS2	0.67223158	9.29E-25
ATP7A	BCORL1	0.67228202	9.19E-25
ATP7A	KCTD9	0.67228836	9.18E-25
ATP7A	RBM43	0.67244184	8.88E-25
ATP7A	NFE2L2	0.67258182	8.61E-25
ATP7A	IDS	0.67292176	8E-25
ATP7A	CRK	0.67298817	7.88E-25
ATP7A	PRDX3	0.6735981	6.9E-25
ATP7A	NUDT3	0.67389037	6.47E-25
ATP7A	PCYT1A	0.67398441	6.34E-25
ATP7A	MBNL1	0.67400425	6.31E-25
ATP7A	KIF13A	0.67401354	6.3E-25
ATP7A	PIGK	0.67406682	6.23E-25
ATP7A	RAP2A	0.67459133	5.55E-25
ATP7A	HNRNPLL	0.67479598	5.31E-25
ATP7A	SLC17A5	0.67500646	5.07E-25
ATP7A	FAM120A	0.67502377	5.05E-25
ATP7A	FAM102B	0.6750896	4.98E-25
ATP7A	RPE	0.67522165	4.83E-25
ATP7A	RFFL	0.67533698	4.71E-25
ATP7A	PPP1CB	0.67535127	4.7E-25
ATP7A	CNOT7	0.67535168	4.7E-25
ATP7A	ZNF281	0.67536141	4.69E-25
ATP7A	TMOD3	0.67537886	4.67E-25
ATP7A	HAUS2	0.67538809	4.66E-25
ATP7A	TANC2	0.67549568	4.55E-25
ATP7A	GNPDA2	0.67550656	4.54E-25
ATP7A	POFUT1	0.67553235	4.51E-25
ATP7A	PARD3B	0.67554007	4.51E-25
ATP7A	HNRNPR	0.6758768	4.18E-25
ATP7A	PPIL4	0.67600178	4.07E-25
ATP7A	USP1	0.67631499	3.8E-25
ATP7A	PPP1R15B	0.67636178	3.76E-25
ATP7A	MAML2	0.67656025	3.6E-25
ATP7A	NEK1	0.6766071	3.56E-25
ATP7A	MYO6	0.6768869	3.35E-25
ATP7A	LACC1	0.67713745	3.17E-25
ATP7A	PIK3R1	0.67725709	3.08E-25
ATP7A	RAB5A	0.67761838	2.85E-25
ATP7A	PEX26	0.67779865	2.73E-25
ATP7A	EHBP1	0.6778281	2.72E-25
ATP7A	DYRK2	0.67815769	2.53E-25
ATP7A	HNRNPU	0.67846618	2.36E-25
ATP7A	STK4	0.67847576	2.35E-25
ATP7A	DEPTOR	0.67857937	2.3E-25
ATP7A	ACTR3	0.6786582	2.26E-25
ATP7A	ETNK1	0.67865896	2.26E-25
ATP7A	SGMS2	0.67870245	2.24E-25
ATP7A	HBP1	0.67890528	2.14E-25
ATP7A	TBK1	0.67892406	2.13E-25
ATP7A	MINDY2	0.67899793	2.09E-25
ATP7A	RNF4	0.67908193	2.06E-25
ATP7A	KBTBD4	0.67909589	2.05E-25
ATP7A	HIVEP2	0.67919785	2E-25
ATP7A	ZNF639	0.67961449	1.82E-25
ATP7A	ST13	0.6797742	1.76E-25
ATP7A	RBM18	0.67995917	1.69E-25

ATP7A	NDFIP2	0.67996353	1.69E-25
ATP7A	TPP1	0.6799945	1.68E-25
ATP7A	CAMSAP1	0.68010808	1.63E-25
ATP7A	ZC2HC1A	0.68024767	1.58E-25
ATP7A	NIPA2	0.68028506	1.57E-25
ATP7A	KDSR	0.68030067	1.56E-25
ATP7A	PTPRA	0.68039396	1.53E-25
ATP7A	ATP6V1A	0.68053246	1.49E-25
ATP7A	CLIP1	0.68065583	1.44E-25
ATP7A	VTA1	0.6807247	1.42E-25
ATP7A	ARL14EP	0.68078666	1.4E-25
ATP7A	DDX18	0.68086045	1.38E-25
ATP7A	CPT1A	0.68108128	1.31E-25
ATP7A	SRSF1	0.6810949	1.31E-25
ATP7A	SBF2	0.68120993	1.28E-25
ATP7A	BAG5	0.68125988	1.26E-25
ATP7A	GLCE	0.68127415	1.26E-25
ATP7A	SLC39A9	0.68156884	1.18E-25
ATP7A	RBBP4	0.68164323	1.16E-25
ATP7A	FOXN3	0.6816919	1.14E-25
ATP7A	CRKL	0.68196343	1.08E-25
ATP7A	NIPA1	0.68215375	1.03E-25
ATP7A	CDKN2AIP	0.68251342	9.5E-26
ATP7A	CEP41	0.68266348	9.19E-26
ATP7A	TRRAP	0.6833501	7.86E-26
ATP7A	ATP11A	0.6833802	7.81E-26
ATP7A	CYB5R4	0.68341698	7.74E-26
ATP7A	UBQLN1	0.68350491	7.59E-26
ATP7A	RAB10	0.68360708	7.42E-26
ATP7A	METAP1	0.68385343	7.01E-26
ATP7A	CHM	0.68409211	6.64E-26
ATP7A	STX12	0.68416148	6.54E-26
ATP7A	TTC5	0.68418773	6.5E-26
ATP7A	NT5DC1	0.68439985	6.19E-26
ATP7A	GANAB	0.68446482	6.1E-26
ATP7A	SELENOT	0.6845023	6.05E-26
ATP7A	WDR26	0.68454538	5.99E-26
ATP7A	BCL9L	0.68466099	5.83E-26
ATP7A	CAB39	0.68474343	5.72E-26
ATP7A	MAPK6	0.68475453	5.71E-26
ATP7A	AGPS	0.68477568	5.68E-26
ATP7A	TMTC3	0.68494161	5.47E-26
ATP7A	CACUL1	0.68502492	5.37E-26
ATP7A	IDH3A	0.68529292	5.05E-26
ATP7A	CDC27	0.68539824	4.93E-26
ATP7A	SH3D19	0.68545393	4.87E-26
ATP7A	PPP3CA	0.68558334	4.72E-26
ATP7A	PURB	0.68571796	4.58E-26
ATP7A	SETD3	0.68590322	4.39E-26
ATP7A	UVRAG	0.68605033	4.24E-26
ATP7A	FGFR1OP2	0.68616161	4.14E-26
ATP7A	LRRCC1	0.68629821	4.01E-26
ATP7A	ZNF227	0.68686932	3.52E-26
ATP7A	SPRED2	0.68704591	3.38E-26
ATP7A	FBXO28	0.6872592	3.21E-26
ATP7A	CSGALNACT2	0.68749635	3.04E-26
ATP7A	BZW1	0.68757284	2.99E-26
ATP7A	ARHGAP42	0.6876211	2.96E-26

ATP7A	TWSG1	0.68771138	2.9E-26
ATP7A	FBXO34	0.68803142	2.69E-26
ATP7A	ZMIZ1	0.68815657	2.61E-26
ATP7A	UBA2	0.68823595	2.56E-26
ATP7A	KLHL2	0.68831051	2.52E-26
ATP7A	YES1	0.68840296	2.47E-26
ATP7A	ZNF468	0.68881888	2.24E-26
ATP7A	SDHD	0.68901062	2.14E-26
ATP7A	HACD2	0.68905414	2.12E-26
ATP7A	SP1	0.68906778	2.11E-26
ATP7A	GFM1	0.68923414	2.03E-26
ATP7A	HSPA13	0.68974097	1.81E-26
ATP7A	DNAJA2	0.68984148	1.77E-26
ATP7A	SUCLA2	0.68984433	1.76E-26
ATP7A	RSRC1	0.69023581	1.61E-26
ATP7A	STT3B	0.69025344	1.6E-26
ATP7A	POGK	0.69029449	1.59E-26
ATP7A	UBE3C	0.69035348	1.57E-26
ATP7A	SPRTN	0.69063953	1.47E-26
ATP7A	SPPL3	0.69089062	1.38E-26
ATP7A	RAD21	0.69096376	1.36E-26
ATP7A	ISG20L2	0.69131255	1.25E-26
ATP7A	SCOC	0.6916329	1.16E-26
ATP7A	TM9SF3	0.6919961	1.07E-26
ATP7A	STAU1	0.69243559	9.62E-27
ATP7A	SAMD8	0.69297367	8.47E-27
ATP7A	ATAD1	0.69305939	8.3E-27
ATP7A	RAB14	0.69306268	8.29E-27
ATP7A	NEDD4	0.69313651	8.15E-27
ATP7A	RPL7L1	0.69358685	7.33E-27
ATP7A	SFXN1	0.69387062	6.85E-27
ATP7A	ARF3	0.69389906	6.81E-27
ATP7A	GEMIN5	0.69391044	6.79E-27
ATP7A	MTMR2	0.69442774	6E-27
ATP7A	SERINC1	0.69454	5.85E-27
ATP7A	STK38L	0.6947348	5.58E-27
ATP7A	CDC42SE2	0.69479335	5.5E-27
ATP7A	WDR20	0.69479525	5.5E-27
ATP7A	CALCOCO2	0.69508491	5.14E-27
ATP7A	PLSCR4	0.69508783	5.13E-27
ATP7A	QTRT2	0.69517399	5.03E-27
ATP7A	IL13RA1	0.69535227	4.82E-27
ATP7A	SUSD6	0.69540232	4.76E-27
ATP7A	NDUFS1	0.69549307	4.66E-27
ATP7A	NEK7	0.69554458	4.6E-27
ATP7A	RYBP	0.69555534	4.59E-27
ATP7A	LMBRD1	0.69568783	4.45E-27
ATP7A	SMARCA5	0.69582502	4.3E-27
ATP7A	DNAJC21	0.69592733	4.2E-27
ATP7A	ASB7	0.69603541	4.09E-27
ATP7A	ARHGAP21	0.69617956	3.95E-27
ATP7A	GLG1	0.69624217	3.9E-27
ATP7A	TOPORS	0.69627692	3.86E-27
ATP7A	EPS15	0.69633927	3.81E-27
ATP7A	CNOT8	0.6963428	3.8E-27
ATP7A	B3GNT2	0.69634355	3.8E-27
ATP7A	PIP5K1A	0.6964691	3.69E-27
ATP7A	CCNI	0.69670025	3.49E-27

ATP7A	DIP2B	0.69679585	3.41E-27
ATP7A	CIPC	0.69680019	3.41E-27
ATP7A	ZNRF2	0.69683401	3.38E-27
ATP7A	TRIP12	0.69694004	3.3E-27
ATP7A	KIAA0232	0.69702041	3.23E-27
ATP7A	RABL3	0.69709269	3.18E-27
ATP7A	FGD4	0.69713671	3.14E-27
ATP7A	SGCB	0.69746751	2.9E-27
ATP7A	ZNF184	0.69763923	2.79E-27
ATP7A	FBXO8	0.69774738	2.71E-27
ATP7A	ARMT1	0.69780163	2.68E-27
ATP7A	ESYT2	0.69785395	2.65E-27
ATP7A	LEPROT	0.69824741	2.41E-27
ATP7A	ADAM17	0.69836774	2.34E-27
ATP7A	TRAK2	0.69854115	2.24E-27
ATP7A	MEGF9	0.69862568	2.2E-27
ATP7A	DIPK2A	0.69889992	2.06E-27
ATP7A	SEC23A	0.69898133	2.02E-27
ATP7A	CNST	0.69901551	2E-27
ATP7A	DCAF1	0.69921916	1.9E-27
ATP7A	TCF12	0.69935723	1.84E-27
ATP7A	TXNRD1	0.6995282	1.77E-27
ATP7A	DOCK7	0.69955895	1.75E-27
ATP7A	TRAPPC6B	0.69959567	1.74E-27
ATP7A	UPRT	0.69990047	1.61E-27
ATP7A	RECQL	0.70006979	1.55E-27
ATP7A	ABRAXAS2	0.70010059	1.54E-27
ATP7A	RAD23B	0.70010917	1.53E-27
ATP7A	PICALM	0.7001971	1.5E-27
ATP7A	DDX21	0.70023169	1.49E-27
ATP7A	LUZP1	0.70031967	1.46E-27
ATP7A	PPP1R2	0.70042165	1.42E-27
ATP7A	TOP1	0.70051929	1.39E-27
ATP7A	RBBP9	0.70059134	1.36E-27
ATP7A	ABI2	0.70068396	1.33E-27
ATP7A	NFYA	0.70082536	1.29E-27
ATP7A	PLEKHB2	0.70104385	1.22E-27
ATP7A	ETV3	0.70129534	1.15E-27
ATP7A	SRGAP2B	0.70143126	1.11E-27
ATP7A	FAM168A	0.7016961	1.04E-27
ATP7A	DEK	0.7020605	9.52E-28
ATP7A	HS2ST1	0.70216884	9.27E-28
ATP7A	CKAP5	0.70221607	9.17E-28
ATP7A	ARMC1	0.7022751	9.04E-28
ATP7A	HIF1A	0.70246093	8.63E-28
ATP7A	JKAMP	0.70248684	8.58E-28
ATP7A	SMC6	0.7027169	8.11E-28
ATP7A	EXT2	0.70282824	7.89E-28
ATP7A	CWC22	0.70316482	7.26E-28
ATP7A	SLC25A24	0.70317916	7.24E-28
ATP7A	RAPH1	0.70360631	6.51E-28
ATP7A	LCORL	0.70367155	6.41E-28
ATP7A	TWF1	0.70373859	6.3E-28
ATP7A	RBFOX2	0.70409731	5.77E-28
ATP7A	FAF2	0.70410491	5.76E-28
ATP7A	LMBR1	0.70416053	5.68E-28
ATP7A	CDC23	0.70420335	5.62E-28
ATP7A	C5orf15	0.704247	5.56E-28

ATP7A	STAM	0.70430356	5.48E-28
ATP7A	SSH1	0.70437512	5.39E-28
ATP7A	BMT2	0.70438329	5.38E-28
ATP7A	CBL	0.7045153	5.2E-28
ATP7A	SORL1	0.7045693	5.13E-28
ATP7A	MED21	0.70463411	5.05E-28
ATP7A	OSBPL11	0.70466535	5.01E-28
ATP7A	AHCYL1	0.70471264	4.95E-28
ATP7A	JAK1	0.70475229	4.91E-28
ATP7A	TADA1	0.70483083	4.81E-28
ATP7A	VPS35	0.70543902	4.14E-28
ATP7A	MTPN	0.70568013	3.9E-28
ATP7A	ACVR1	0.70612675	3.49E-28
ATP7A	PRKAR1A	0.70637664	3.28E-28
ATP7A	VKORC1L1	0.70650071	3.18E-28
ATP7A	GLYR1	0.70660247	3.1E-28
ATP7A	LAMP2	0.70672004	3.01E-28
ATP7A	MCMBP	0.70693	2.85E-28
ATP7A	VAMP7	0.70698721	2.81E-28
ATP7A	CTR9	0.70711458	2.72E-28
ATP7A	THRAP3	0.70765271	2.38E-28
ATP7A	MAT2B	0.70787253	2.25E-28
ATP7A	PTPN9	0.70793929	2.22E-28
ATP7A	AHR	0.70796247	2.2E-28
ATP7A	FBXL5	0.70797495	2.2E-28
ATP7A	CSNK2A1	0.70801803	2.17E-28
ATP7A	TRAF3IP1	0.70848757	1.93E-28
ATP7A	PEX2	0.70864898	1.85E-28
ATP7A	ZC3H11A	0.7086932	1.83E-28
ATP7A	ATP2A2	0.70876056	1.8E-28
ATP7A	TAB2	0.70877259	1.8E-28
ATP7A	NUDCD3	0.7088775	1.75E-28
ATP7A	PNPLA8	0.70892956	1.73E-28
ATP7A	CTSO	0.70894469	1.72E-28
ATP7A	ZNF623	0.70918651	1.62E-28
ATP7A	SAR1B	0.71027438	1.23E-28
ATP7A	CSNK1A1	0.71036131	1.2E-28
ATP7A	NPTN	0.71042504	1.18E-28
ATP7A	WIPF2	0.71062628	1.12E-28
ATP7A	AHCTF1	0.71100062	1.02E-28
ATP7A	GLOD4	0.71107751	1E-28
ATP7A	CCSER2	0.71113286	9.88E-29
ATP7A	PDLIM5	0.71160112	8.77E-29
ATP7A	MED14	0.71175584	8.43E-29
ATP7A	KAT7	0.71187215	8.18E-29
ATP7A	ENAH	0.71187721	8.17E-29
ATP7A	PRRC2C	0.71207665	7.76E-29
ATP7A	LAMTOR3	0.71234126	7.26E-29
ATP7A	MFSD14A	0.71247101	7.02E-29
ATP7A	MAPK14	0.71273517	6.56E-29
ATP7A	BRCC3	0.71292117	6.25E-29
ATP7A	TSNAX	0.71316939	5.87E-29
ATP7A	MBNL3	0.71362413	5.22E-29
ATP7A	GORASP2	0.71367414	5.15E-29
ATP7A	ABCB10	0.71379374	5E-29
ATP7A	WDR47	0.71456272	4.1E-29
ATP7A	NBN	0.71470297	3.95E-29
ATP7A	CDK19	0.71474874	3.91E-29

ATP7A	SERINC5	0.71510517	3.56E-29
ATP7A	CTTNBP2NL	0.71552924	3.19E-29
ATP7A	HPS5	0.71564284	3.1E-29
ATP7A	GNG12	0.71614735	2.72E-29
ATP7A	FTO	0.71617068	2.7E-29
ATP7A	RAB8B	0.7163812	2.56E-29
ATP7A	STAT3	0.71688681	2.24E-29
ATP7A	SNX27	0.71689466	2.24E-29
ATP7A	ATP6V1C1	0.7169539	2.2E-29
ATP7A	ZNF260	0.71713054	2.1E-29
ATP7A	TAF1B	0.71724922	2.04E-29
ATP7A	NSL1	0.71737828	1.97E-29
ATP7A	CHUK	0.71744515	1.94E-29
ATP7A	KPNA3	0.71766987	1.83E-29
ATP7A	PAK2	0.7179265	1.71E-29
ATP7A	KIF3B	0.71821004	1.59E-29
ATP7A	ERAP1	0.71833791	1.53E-29
ATP7A	PKD2	0.71840329	1.51E-29
ATP7A	COPA	0.7184305	1.5E-29
ATP7A	DNAJC10	0.71854759	1.45E-29
ATP7A	ACBD5	0.71858634	1.44E-29
ATP7A	ARPP19	0.71868613	1.4E-29
ATP7A	ADD3	0.71897628	1.3E-29
ATP7A	EIF4G2	0.71903538	1.28E-29
ATP7A	CDC73	0.7190812	1.26E-29
ATP7A	LNPEP	0.71919209	1.22E-29
ATP7A	DAAM1	0.71951765	1.12E-29
ATP7A	DCK	0.71973244	1.06E-29
ATP7A	TBC1D5	0.71979074	1.05E-29
ATP7A	CBFB	0.71999136	9.92E-30
ATP7A	CNIH1	0.72002586	9.83E-30
ATP7A	GFPT1	0.72003697	9.8E-30
ATP7A	DLAT	0.7201395	9.54E-30
ATP7A	VPS4B	0.72075956	8.09E-30
ATP7A	INIP	0.7216202	6.44E-30
ATP7A	PLEKHA1	0.72194738	5.9E-30
ATP7A	KRAS	0.72195739	5.88E-30
ATP7A	KDM1B	0.7222132	5.49E-30
ATP7A	SPTLC1	0.72222771	5.47E-30
ATP7A	MBD5	0.72273334	4.78E-30
ATP7A	WDR82	0.72280286	4.69E-30
ATP7A	RAB3GAP1	0.72327609	4.13E-30
ATP7A	ATMIN	0.72329837	4.11E-30
ATP7A	SOCS5	0.72361863	3.77E-30
ATP7A	GNA13	0.72379101	3.6E-30
ATP7A	DR1	0.72390275	3.49E-30
ATP7A	STX7	0.72417211	3.25E-30
ATP7A	CTBS	0.72424605	3.19E-30
ATP7A	FEM1B	0.72425442	3.18E-30
ATP7A	PPFIA1	0.72431639	3.13E-30
ATP7A	TRMT1L	0.72512141	2.51E-30
ATP7A	SLC30A6	0.72521527	2.45E-30
ATP7A	SPRED1	0.72524456	2.43E-30
ATP7A	CAMSAP2	0.72529776	2.4E-30
ATP7A	RBM7	0.7253511	2.36E-30
ATP7A	SERINC3	0.72539669	2.33E-30
ATP7A	ATF7IP	0.72554457	2.24E-30
ATP7A	UBTD2	0.72684804	1.57E-30

ATP7A	SNX6	0.72688986	1.56E-30
ATP7A	BCL9	0.72700677	1.51E-30
ATP7A	PMS2	0.72716308	1.44E-30
ATP7A	UGDH	0.72736088	1.37E-30
ATP7A	GOLPH3	0.72738616	1.36E-30
ATP7A	SLC12A6	0.7274171	1.35E-30
ATP7A	PEAK1	0.72774687	1.23E-30
ATP7A	TMEM106B	0.72777631	1.22E-30
ATP7A	UBA6	0.7277908	1.22E-30
ATP7A	XPR1	0.72816329	1.1E-30
ATP7A	SLC30A1	0.72825626	1.07E-30
ATP7A	HFE	0.72835076	1.04E-30
ATP7A	XRN2	0.72843695	1.02E-30
ATP7A	ZMYM4	0.7285985	9.75E-31
ATP7A	BTBD9	0.72890526	8.96E-31
ATP7A	NFATC3	0.72907634	8.55E-31
ATP7A	FAM20B	0.7290992	8.49E-31
ATP7A	WDFY1	0.7290995	8.49E-31
ATP7A	FNBP1L	0.72937564	7.87E-31
ATP7A	PHACTR2	0.72940065	7.82E-31
ATP7A	GNPTAB	0.72945265	7.71E-31
ATP7A	EPC2	0.72952537	7.55E-31
ATP7A	LARP7	0.72961487	7.37E-31
ATP7A	CNOT6L	0.72986235	6.88E-31
ATP7A	ORC4	0.73000692	6.61E-31
ATP7A	ARIH1	0.73039362	5.94E-31
ATP7A	CDC5L	0.73084586	5.24E-31
ATP7A	BLOC1S6	0.73103422	4.97E-31
ATP7A	C1D	0.7311751	4.78E-31
ATP7A	SMNDC1	0.73120294	4.75E-31
ATP7A	BTBD1	0.73131684	4.6E-31
ATP7A	DLG1	0.73181289	4.01E-31
ATP7A	UHMK1	0.73195652	3.85E-31
ATP7A	TMEM135	0.73210617	3.69E-31
ATP7A	WDR89	0.73214506	3.65E-31
ATP7A	PPP1R12A	0.73253129	3.28E-31
ATP7A	KIF5B	0.73365575	2.39E-31
ATP7A	TMEM87B	0.73406157	2.13E-31
ATP7A	ZFP1	0.73421359	2.04E-31
ATP7A	SNX4	0.7342669	2.01E-31
ATP7A	ADAR	0.73436648	1.96E-31
ATP7A	HNRNPK	0.73440846	1.94E-31
ATP7A	PARG	0.7346186	1.82E-31
ATP7A	NUDT4B	0.73471893	1.77E-31
ATP7A	ZNF146	0.73497987	1.65E-31
ATP7A	RAB21	0.73526404	1.52E-31
ATP7A	MAPK1IP1L	0.73587276	1.28E-31
ATP7A	SF3A1	0.73614386	1.18E-31
ATP7A	CUL2	0.73635528	1.12E-31
ATP7A	TMEM267	0.73637096	1.11E-31
ATP7A	DYRK1A	0.73648484	1.07E-31
ATP7A	TEAD1	0.73656123	1.05E-31
ATP7A	GTF3C4	0.7366626	1.02E-31
ATP7A	CTCF	0.73699835	9.28E-32
ATP7A	FRS2	0.73715447	8.88E-32
ATP7A	ENOX2	0.73744212	8.18E-32
ATP7A	SNTB2	0.73804761	6.88E-32
ATP7A	MBNL2	0.73806101	6.85E-32

ATP7A	RNF2	0.73809327	6.79E-32
ATP7A	RB1	0.73812901	6.72E-32
ATP7A	KIAA1191	0.73819915	6.59E-32
ATP7A	KPNA1	0.73832541	6.35E-32
ATP7A	RAB6A	0.73854884	5.96E-32
ATP7A	TMEM167B	0.73861175	5.85E-32
ATP7A	USP8	0.73865161	5.78E-32
ATP7A	CCDC47	0.73871743	5.67E-32
ATP7A	ANKRD13C	0.73912557	5.05E-32
ATP7A	OGFOD1	0.73921026	4.92E-32
ATP7A	ZNF45	0.73929709	4.8E-32
ATP7A	CCNG2	0.73938874	4.68E-32
ATP7A	ZBTB6	0.7395414	4.48E-32
ATP7A	SOCS6	0.73966792	4.32E-32
ATP7A	CLINT1	0.73996527	3.96E-32
ATP7A	MEF2A	0.74052242	3.37E-32
ATP7A	WASL	0.74062945	3.27E-32
ATP7A	TMED5	0.74118772	2.78E-32
ATP7A	SMC3	0.7414424	2.58E-32
ATP7A	NAA30	0.74177182	2.34E-32
ATP7A	GTF2H3	0.74195432	2.22E-32
ATP7A	FOXN2	0.7422424	2.04E-32
ATP7A	FYTTD1	0.7425668	1.86E-32
ATP7A	SCAMP1	0.74270989	1.78E-32
ATP7A	IFNAR1	0.74319364	1.55E-32
ATP7A	UBQLN2	0.74320066	1.54E-32
ATP7A	SMAD2	0.74322504	1.53E-32
ATP7A	EIF4G3	0.74333635	1.48E-32
ATP7A	UBXN2B	0.7434807	1.42E-32
ATP7A	CUL4B	0.74359764	1.37E-32
ATP7A	SENP1	0.74373637	1.32E-32
ATP7A	NUP58	0.74377686	1.3E-32
ATP7A	ATF1	0.74385718	1.27E-32
ATP7A	TVP23B	0.74416674	1.16E-32
ATP7A	DNAJC14	0.74449406	1.05E-32
ATP7A	MED17	0.74474284	9.8E-33
ATP7A	MED1	0.74476602	9.73E-33
ATP7A	MMGT1	0.74489021	9.38E-33
ATP7A	NPAT	0.74501846	9.03E-33
ATP7A	PDS5B	0.74518624	8.59E-33
ATP7A	C11orf58	0.74590876	6.93E-33
ATP7A	TAOK3	0.74591675	6.92E-33
ATP7A	GOSR1	0.74610978	6.53E-33
ATP7A	ABHD13	0.74651151	5.79E-33
ATP7A	RTF1	0.74711537	4.84E-33
ATP7A	ZYG11B	0.74726904	4.62E-33
ATP7A	C16orf72	0.74762905	4.15E-33
ATP7A	FAM91A1	0.74779783	3.94E-33
ATP7A	ZFAND3	0.74791466	3.81E-33
ATP7A	DIS3	0.74796529	3.75E-33
ATP7A	SCAF11	0.74828584	3.41E-33
ATP7A	ATP11B	0.74829583	3.4E-33
ATP7A	SUZ12	0.74907429	2.69E-33
ATP7A	ZNF322	0.74925166	2.55E-33
ATP7A	NCKAP1	0.74927861	2.53E-33
ATP7A	PTPN11	0.7493767	2.45E-33
ATP7A	ABI1	0.74939259	2.44E-33
ATP7A	GMCL1	0.74947775	2.38E-33

ATP7A	TFAM	0.74974356	2.19E-33
ATP7A	ZNF609	0.74976	2.18E-33
ATP7A	RHBDD1	0.74985601	2.12E-33
ATP7A	ACTR2	0.74990781	2.09E-33
ATP7A	ERLIN1	0.75008493	1.98E-33
ATP7A	AEBP2	0.7502049	1.91E-33
ATP7A	FEM1C	0.75047524	1.76E-33
ATP7A	SHOC2	0.75071017	1.64E-33
ATP7A	PDE12	0.75080023	1.59E-33
ATP7A	UBE2D3	0.75082368	1.58E-33
ATP7A	OTUD7B	0.75125931	1.39E-33
ATP7A	PCYOX1	0.75155456	1.27E-33
ATP7A	RRN3	0.75164887	1.23E-33
ATP7A	TCAF1	0.75175997	1.19E-33
ATP7A	STRN	0.75181725	1.17E-33
ATP7A	RASAL2	0.75211471	1.07E-33
ATP7A	VEZF1	0.75222502	1.03E-33
ATP7A	ALS2	0.75250455	9.47E-34
ATP7A	NCOA4	0.75257777	9.25E-34
ATP7A	SIN3A	0.75267857	8.97E-34
ATP7A	FBXL17	0.75276249	8.74E-34
ATP7A	SNX16	0.75299225	8.15E-34
ATP7A	SMIM15	0.75385456	6.25E-34
ATP7A	TMEM170A	0.75392828	6.11E-34
ATP7A	RNF11	0.75461904	4.93E-34
ATP7A	CLOCK	0.75470958	4.8E-34
ATP7A	PDCL	0.75498964	4.4E-34
ATP7A	PPP4R2	0.75504517	4.32E-34
ATP7A	ACSL4	0.75518759	4.14E-34
ATP7A	BMI1	0.75562432	3.61E-34
ATP7A	RNF41	0.75569282	3.53E-34
ATP7A	TOX4	0.75598393	3.23E-34
ATP7A	RRM2B	0.7560739	3.14E-34
ATP7A	PPP2CA	0.7562767	2.95E-34
ATP7A	YY1	0.75639075	2.84E-34
ATP7A	GPD2	0.75690705	2.42E-34
ATP7A	GNAQ	0.75707014	2.3E-34
ATP7A	SYNJ2BP	0.75709566	2.28E-34
ATP7A	TERF1	0.75720025	2.21E-34
ATP7A	AGGF1	0.75844509	1.49E-34
ATP7A	IPO7	0.75845311	1.49E-34
ATP7A	OSBPL8	0.75904746	1.23E-34
ATP7A	GOLIM4	0.75911217	1.21E-34
ATP7A	ATP10D	0.75941981	1.1E-34
ATP7A	CPNE3	0.75974065	9.9E-35
ATP7A	EXOC6B	0.75988031	9.47E-35
ATP7A	ERBIN	0.7599619	9.22E-35
ATP7A	KIF16B	0.76007533	8.9E-35
ATP7A	MIER1	0.76028795	8.32E-35
ATP7A	ADAM10	0.76031866	8.23E-35
ATP7A	RNF13	0.76049291	7.79E-35
ATP7A	API5	0.76063193	7.45E-35
ATP7A	ADNP	0.76069739	7.3E-35
ATP7A	RNF6	0.76100391	6.62E-35
ATP7A	SCARB2	0.76142504	5.78E-35
ATP7A	UBE2W	0.76149696	5.65E-35
ATP7A	C5orf51	0.76165525	5.37E-35
ATP7A	YME1L1	0.7618199	5.1E-35

ATP7A	PCNP	0.76231187	4.35E-35
ATP7A	PIK3CA	0.76253173	4.05E-35
ATP7A	BAZ1B	0.76311288	3.36E-35
ATP7A	NUMB	0.76345427	3.01E-35
ATP7A	GSTCD	0.76373269	2.75E-35
ATP7A	TAF2	0.76382183	2.67E-35
ATP7A	TGOLN2	0.76384503	2.65E-35
ATP7A	SLC35A5	0.76390954	2.6E-35
ATP7A	RNF14	0.76407756	2.46E-35
ATP7A	SLC30A9	0.76410001	2.44E-35
ATP7A	VCPIP1	0.76424451	2.33E-35
ATP7A	PPIG	0.76428983	2.3E-35
ATP7A	ZBTB38	0.76461131	2.07E-35
ATP7A	PDZD8	0.76499761	1.82E-35
ATP7A	GALC	0.76507871	1.78E-35
ATP7A	WWP1	0.76508724	1.77E-35
ATP7A	DCUN1D1	0.76513633	1.74E-35
ATP7A	LSM14A	0.7653318	1.64E-35
ATP7A	OPA1	0.76577423	1.42E-35
ATP7A	RNF111	0.76636142	1.17E-35
ATP7A	DPY19L4	0.76681823	1.01E-35
ATP7A	AP1AR	0.76698277	9.52E-36
ATP7A	SNX18	0.76700038	9.47E-36
ATP7A	TRIQK	0.76736311	8.4E-36
ATP7A	SEC23IP	0.76741197	8.27E-36
ATP7A	ACBD3	0.76762191	7.71E-36
ATP7A	FBXL3	0.76802973	6.74E-36
ATP7A	SMAD1	0.76819571	6.38E-36
ATP7A	CPSF2	0.76947553	4.17E-36
ATP7A	ESF1	0.76966256	3.92E-36
ATP7A	SLC9A6	0.77020156	3.27E-36
ATP7A	PLEKHA3	0.77030802	3.16E-36
ATP7A	HNRNPH2	0.77044068	3.02E-36
ATP7A	CRIM1	0.77059675	2.87E-36
ATP7A	DDX3X	0.77061388	2.85E-36
ATP7A	RBBP5	0.77071611	2.75E-36
ATP7A	SRPK2	0.77194402	1.82E-36
ATP7A	CERS6	0.77199194	1.79E-36
ATP7A	RELL1	0.77231825	1.61E-36
ATP7A	CDK12	0.77238447	1.57E-36
ATP7A	ARL13B	0.77244576	1.54E-36
ATP7A	SPAST	0.77260066	1.46E-36
ATP7A	PPM1B	0.77267335	1.42E-36
ATP7A	DDX6	0.77267406	1.42E-36
ATP7A	CPD	0.7730507	1.25E-36
ATP7A	KDM3B	0.77370114	1E-36
ATP7A	ELF1	0.77378191	9.77E-37
ATP7A	PUM2	0.77396781	9.17E-37
ATP7A	EIF4E	0.77400859	9.04E-37
ATP7A	NBR1	0.77471862	7.1E-37
ATP7A	USP9X	0.77608358	4.44E-37
ATP7A	G3BP1	0.77617107	4.31E-37
ATP7A	NEDD1	0.77624404	4.2E-37
ATP7A	PEX19	0.77637227	4.02E-37
ATP7A	CAPZA2	0.77667601	3.62E-37
ATP7A	NCOA3	0.7774789	2.74E-37
ATP7A	SPIN1	0.77769669	2.54E-37
ATP7A	ROCK2	0.77776701	2.48E-37

ATP7A	SPTY2D1	0.77808781	2.22E-37
ATP7A	SOCS4	0.77860101	1.85E-37
ATP7A	EVI5	0.77864243	1.83E-37
ATP7A	NUFIP2	0.7788346	1.71E-37
ATP7A	HIPK3	0.77909322	1.56E-37
ATP7A	VPS41	0.77913243	1.54E-37
ATP7A	OXR1	0.77941793	1.39E-37
ATP7A	YIPF5	0.77948	1.36E-37
ATP7A	FCF1	0.77995818	1.15E-37
ATP7A	FBXO38	0.78012612	1.09E-37
ATP7A	MPP5	0.7802046	1.06E-37
ATP7A	LNPK	0.7809846	8.02E-38
ATP7A	NAA15	0.78113658	7.6E-38
ATP7A	MBTPS2	0.78121177	7.4E-38
ATP7A	RSF1	0.78160185	6.45E-38
ATP7A	ATP13A3	0.78163793	6.36E-38
ATP7A	MTDH	0.78171233	6.2E-38
ATP7A	ANKRD50	0.78203408	5.53E-38
ATP7A	ATE1	0.78204878	5.5E-38
ATP7A	MED13	0.78323545	3.6E-38
ATP7A	HMGXB4	0.78330927	3.51E-38
ATP7A	TMX1	0.7835873	3.18E-38
ATP7A	SP3	0.78366141	3.09E-38
ATP7A	CAND1	0.78386147	2.88E-38
ATP7A	IMPA1	0.78404934	2.69E-38
ATP7A	LIN54	0.78435073	2.41E-38
ATP7A	BMPR2	0.78502048	1.9E-38
ATP7A	SOS2	0.78517002	1.8E-38
ATP7A	SLAIN2	0.78538558	1.66E-38
ATP7A	SETD7	0.78583585	1.41E-38
ATP7A	TRUB1	0.78656269	1.08E-38
ATP7A	CANX	0.78678482	9.99E-39
ATP7A	PHAX	0.78684846	9.76E-39
ATP7A	RAB18	0.78758628	7.45E-39
ATP7A	YIPF6	0.78767053	7.23E-39
ATP7A	SLC25A40	0.7880846	6.21E-39
ATP7A	RPRD2	0.78828861	5.76E-39
ATP7A	GMFB	0.78903241	4.38E-39
ATP7A	SMC1A	0.78966471	3.47E-39
ATP7A	C5orf24	0.78970397	3.42E-39
ATP7A	FCHO2	0.78979521	3.3E-39
ATP7A	MAPK1	0.7908484	2.23E-39
ATP7A	PPM1A	0.79097861	2.13E-39
ATP7A	PRRC1	0.79185329	1.53E-39
ATP7A	UEVLD	0.79221908	1.34E-39
ATP7A	CREB1	0.79236885	1.26E-39
ATP7A	GCC2	0.79326956	9E-40
ATP7A	METTL14	0.79355839	8.07E-40
ATP7A	ITCH	0.79392017	7.03E-40
ATP7A	ZFP91	0.79393236	7E-40
ATP7A	FNIP2	0.79410648	6.55E-40
ATP7A	CNOT6	0.79493702	4.78E-40
ATP7A	WAC	0.79534169	4.09E-40
ATP7A	KAT6A	0.79635949	2.77E-40
ATP7A	KIF2A	0.79671576	2.42E-40
ATP7A	CHD9	0.79713817	2.05E-40
ATP7A	YTHDF3	0.7973684	1.88E-40
ATP7A	STAU2	0.79775269	1.62E-40

ATP7A	EFCAB14	0.79993372	6.92E-41
ATP7A	RLIM	0.8024795	2.53E-41
ATP7A	RAB3GAP2	0.80260058	2.41E-41
ATP7A	CAMK2D	0.80340788	1.75E-41
ATP7A	ATF2	0.80353503	1.66E-41
ATP7A	CLCN3	0.80373199	1.53E-41
ATP7A	MATR3	0.80376985	1.51E-41
ATP7A	STRN3	0.80431381	1.22E-41
ATP7A	ZBTB33	0.80499262	9.24E-42
ATP7A	PDS5A	0.8050749	8.94E-42
ATP7A	TMED7	0.80509526	8.87E-42
ATP7A	ARL5A	0.80531337	8.12E-42
ATP7A	SDE2	0.80562155	7.17E-42
ATP7A	HIPK1	0.80681796	4.41E-42
ATP7A	PPP2R5E	0.80711603	3.9E-42
ATP7A	PPP4R3B	0.80722204	3.74E-42
ATP7A	NF1	0.80771649	3.05E-42
ATP7A	TNPO1	0.80848994	2.22E-42
ATP7A	MTM1	0.8085994	2.12E-42
ATP7A	ZC3H13	0.81162757	6.02E-43
ATP7A	AP5M1	0.81235774	4.43E-43
ATP7A	PUM1	0.81265094	3.91E-43
ATP7A	ASXL2	0.8132784	3E-43
ATP7A	ZNF562	0.81342851	2.81E-43
ATP7A	EFR3A	0.81500769	1.44E-43
ATP7A	TAOK1	0.81533912	1.25E-43
ATP7A	ALG11	0.81554449	1.14E-43
ATP7A	UTP14C	0.81556119	1.13E-43
ATP7A	TOR1AIP1	0.81590423	9.77E-44
ATP7A	ZFR	0.81626892	8.35E-44
ATP7A	SMAD5	0.81640657	7.86E-44
ATP7A	PJA2	0.81643387	7.77E-44
ATP7A	TLK1	0.81646323	7.67E-44
ATP7A	MAGT1	0.81676655	6.73E-44
ATP7A	MAP3K2	0.81812747	3.72E-44
ATP7A	SRFBP1	0.81900932	2.53E-44
ATP7A	SLC25A46	0.82013571	1.54E-44
ATP7A	EXOC5	0.8203995	1.37E-44
ATP7A	SPOPL	0.82120584	9.59E-45
ATP7A	ZNF148	0.82232936	5.8E-45
ATP7A	G3BP2	0.822371	5.7E-45
ATP7A	MIGA1	0.82306379	4.17E-45
ATP7A	RBM12	0.82437719	2.3E-45
ATP7A	ROCK1	0.82518726	1.59E-45
ATP7A	RP2	0.82537159	1.46E-45
ATP7A	GNL3L	0.82661073	8.3E-46
ATP7A	AKAP11	0.82666736	8.08E-46
ATP7A	ACAP2	0.82765806	5.11E-46
ATP7A	CTDSPL2	0.82813121	4.11E-46
ATP7A	ZNF827	0.82832346	3.75E-46
ATP7A	PANK3	0.82980881	1.87E-46
ATP7A	KLHL20	0.83184937	7.13E-47
ATP7A	RO60	0.83207473	6.41E-47
ATP7A	MIB1	0.8355486	1.2E-47
ATP7A	MFAP3	0.83733972	4.97E-48
ATP7A	WDR44	0.83771201	4.13E-48
ATP7A	USP38	0.84149364	6.19E-49
ATP7A	PGGT1B	0.84183014	5.21E-49

ATP7A	CSNK1G3	0.84319417	2.59E-49
ATP7A	UBE3A	0.84348428	2.23E-49
ATP7A	TTC33	0.8442973	1.47E-49
ATP7A	ARHGEF12	0.84537963	8.35E-50
ATP7A	BBX	0.8475119	2.72E-50
ATP7A	STAG2	0.850531	5.38E-51
ATP7A	RBM27	0.8507794	4.7E-51
ATP7A	CFAP97	0.85083012	4.57E-51
ATP7A	GTF2A1	0.85195109	2.48E-51
ATP7A	NCOA2	0.85217036	2.2E-51
ATP7A	ARHGAP5	0.85282579	1.53E-51
ATP7A	RPS6KA3	0.85530976	3.84E-52
ATP7A	ZBTB41	0.86438305	1.96E-54
ATP7A	AFF4	0.87096885	3.32E-56
ATP7A	DNAJB14	0.88020613	7.34E-59
ATP7B	INPP5A	0.50124546	1.03E-12
ATP7B	ONECUT2	0.50156759	9.92E-13
ATP7B	TM6SF2	0.50156918	9.92E-13
ATP7B	DOK4	0.50169757	9.77E-13
ATP7B	SLC25A23	0.50237439	9.01E-13
ATP7B	TEP1	0.5027804	8.58E-13
ATP7B	BCAS1	0.50281262	8.54E-13
ATP7B	GOLM1	0.50348123	7.88E-13
ATP7B	TRAK1	0.50485473	6.68E-13
ATP7B	MICU1	0.50508985	6.49E-13
ATP7B	ATP10B	0.5051936	6.41E-13
ATP7B	ANXA10	0.50606166	5.77E-13
ATP7B	AKAP1	0.50710745	5.08E-13
ATP7B	RNF128	0.50711349	5.07E-13
ATP7B	HHLA2	0.507123	5.07E-13
ATP7B	STX3	0.5083656	4.35E-13
ATP7B	RETSAT	0.50836628	4.35E-13
ATP7B	RBM47	0.50842258	4.32E-13
ATP7B	TPD52	0.50865626	4.2E-13
ATP7B	ITPK1	0.50881157	4.12E-13
ATP7B	RFFL	0.50913778	3.96E-13
ATP7B	ABHD3	0.50920969	3.92E-13
ATP7B	EFNA2	0.50976157	3.67E-13
ATP7B	CALML4	0.51057978	3.32E-13
ATP7B	SLC6A20	0.5115533	2.94E-13
ATP7B	CCDC68	0.51196504	2.79E-13
ATP7B	TMEM238L	0.51269808	2.55E-13
ATP7B	ETNK1	0.51382965	2.21E-13
ATP7B	EPS8L3	0.51444148	2.05E-13
ATP7B	CDHR2	0.51451365	2.03E-13
ATP7B	PLEKHH1	0.51461723	2.01E-13
ATP7B	AKR1B10	0.5146847	1.99E-13
ATP7B	DISP1	0.51484985	1.95E-13
ATP7B	CA13	0.51616631	1.65E-13
ATP7B	UTP14C	0.51658662	1.57E-13
ATP7B	FAM177B	0.5166036	1.56E-13
ATP7B	SLC40A1	0.51670894	1.54E-13
ATP7B	USH1C	0.51700984	1.49E-13
ATP7B	MYO1A	0.51722472	1.45E-13
ATP7B	CDKN2AIP	0.51820576	1.28E-13
ATP7B	CEBPG	0.5182793	1.27E-13
ATP7B	SMIM15	0.51907198	1.15E-13
ATP7B	DNM2	0.52026983	9.83E-14

ATP7B	ANG	0.52229079	7.6E-14
ATP7B	HMGCS2	0.52262292	7.28E-14
ATP7B	MYRF	0.52293026	7E-14
ATP7B	ACOT11	0.52331042	6.66E-14
ATP7B	SYNJ2BP	0.52515779	5.25E-14
ATP7B	TM9SF3	0.52529915	5.15E-14
ATP7B	SELENBP1	0.52619412	4.59E-14
ATP7B	DDAH1	0.52637929	4.48E-14
ATP7B	KLC4	0.52653623	4.39E-14
ATP7B	SMIM31	0.52774442	3.75E-14
ATP7B	XRCC4	0.5283345	3.47E-14
ATP7B	BMP2	0.52889326	3.23E-14
ATP7B	SLC9A3	0.52900597	3.18E-14
ATP7B	CAPN5	0.53014632	2.74E-14
ATP7B	SLC35C1	0.53043425	2.64E-14
ATP7B	TNIK	0.53063335	2.57E-14
ATP7B	SMIM24	0.53259624	1.98E-14
ATP7B	SH3RF1	0.53413823	1.61E-14
ATP7B	SHROOM3	0.53471726	1.49E-14
ATP7B	TSPAN3	0.53511368	1.42E-14
ATP7B	ATP1B1	0.53519574	1.4E-14
ATP7B	CLINT1	0.53722421	1.07E-14
ATP7B	IHH	0.53898075	8.41E-15
ATP7B	ANKRD40CL	0.53928349	8.08E-15
ATP7B	PPFIBP2	0.53989366	7.43E-15
ATP7B	RASSF6	0.54021437	7.12E-15
ATP7B	SLC41A2	0.54075494	6.61E-15
ATP7B	CA2	0.54101978	6.38E-15
ATP7B	INPP1	0.54234095	5.32E-15
ATP7B	FARP2	0.54757424	2.58E-15
ATP7B	FER1L6	0.54784834	2.49E-15
ATP7B	GNA11	0.55136558	1.52E-15
ATP7B	PLEKHA6	0.55312991	1.18E-15
ATP7B	TNFRSF11A	0.55573537	8.15E-16
ATP7B	RAPGEFL1	0.55589675	7.96E-16
ATP7B	FAM135A	0.55617582	7.65E-16
ATP7B	DOP1B	0.55617755	7.65E-16
ATP7B	PDZD3	0.55688675	6.91E-16
ATP7B	SMPD3	0.55778891	6.07E-16
ATP7B	CDHR5	0.55819193	5.72E-16
ATP7B	TRIM2	0.56001847	4.39E-16
ATP7B	SLC37A1	0.56023823	4.26E-16
ATP7B	NPC1L1	0.56266516	2.99E-16
ATP7B	ALDH2	0.56277407	2.94E-16
ATP7B	B3GALT5	0.56382616	2.52E-16
ATP7B	TMEM45B	0.56419222	2.39E-16
ATP7B	MUC3A	0.56502741	2.11E-16
ATP7B	MCU	0.56540762	1.99E-16
ATP7B	CMBL	0.56660394	1.67E-16
ATP7B	BTNL8	0.57185961	7.59E-17
ATP7B	TOX3	0.57339168	6.02E-17
ATP7B	IYD	0.57446929	5.11E-17
ATP7B	CKMT1B	0.57474735	4.9E-17
ATP7B	CYP2C18	0.57735157	3.28E-17
ATP7B	SLC9A4	0.5824934	1.48E-17
ATP7B	PAQR8	0.58628571	8.11E-18
ATP7B	MUC17	0.58652285	7.81E-18
ATP7B	CES2	0.58912578	5.15E-18

ATP7B	CRYL1	0.59151013	3.51E-18
ATP7B	ANKS4B	0.59830316	1.15E-18
ATP7B	CYSTM1	0.5989652	1.03E-18
ATP7B	SH3BGRL2	0.59978171	9.03E-19
ATP7B	ENTPD5	0.60168002	6.58E-19
ATP7B	CKMT1A	0.60180139	6.45E-19
ATP7B	HNF4A	0.60188627	6.35E-19
ATP7B	SLC9A2	0.61189138	1.15E-19
ATP7B	KCNE3	0.61225253	1.08E-19
ATP7B	AHCYL2	0.62067502	2.44E-20
ATP7B	NR1I2	0.63737097	1.12E-21
ATP7B	LRRC66	0.64773257	1.5E-22
CDKN2A	CDKN2B	0.60560613	3.39E-19
CDKN2A	CDKN2A	1	0
DBT	COMTD1	-0.5791936	2.47E-17
DBT	C4orf48	-0.5562886	7.53E-16
DBT	SCAND1	-0.5472122	2.72E-15
DBT	TRAPPC5	-0.508677	4.19E-13
DBT	MIF	-0.5016354	9.84E-13
DBT	FIG4	0.50017228	1.17E-12
DBT	TMEM35B	0.50042477	1.14E-12
DBT	NDNF	0.50054496	1.12E-12
DBT	MYH9	0.50086403	1.08E-12
DBT	ASAP2	0.50102221	1.06E-12
DBT	NIPA1	0.50111732	1.05E-12
DBT	NUP155	0.50133255	1.02E-12
DBT	GLI3	0.50141743	1.01E-12
DBT	PIK3R3	0.50165047	9.82E-13
DBT	RASGEF1B	0.5016736	9.8E-13
DBT	CXorf21	0.50170631	9.76E-13
DBT	OCIAD1	0.50170654	9.76E-13
DBT	B2M	0.5018169	9.63E-13
DBT	ALDH9A1	0.50182554	9.62E-13
DBT	MEAK7	0.50191733	9.51E-13
DBT	SNX24	0.50220664	9.19E-13
DBT	SINHCAF	0.50239854	8.98E-13
DBT	UBE2E1	0.5025085	8.86E-13
DBT	PAIP2	0.50271304	8.65E-13
DBT	CRIPT	0.50273856	8.62E-13
DBT	EMC2	0.50279573	8.56E-13
DBT	CDKN1B	0.50292548	8.43E-13
DBT	TIMP2	0.50303986	8.31E-13
DBT	GLT8D1	0.50307923	8.27E-13
DBT	BTG1	0.50320222	8.15E-13
DBT	MS4A7	0.50324218	8.11E-13
DBT	LY75	0.50326503	8.09E-13
DBT	SNN	0.50348171	7.88E-13
DBT	PLOD2	0.50377363	7.61E-13
DBT	CDH6	0.50395529	7.44E-13
DBT	PTGR1	0.5043583	7.09E-13
DBT	ERGIC1	0.50446102	7E-13
DBT	SCRN1	0.50457423	6.91E-13
DBT	NUP62	0.50485386	6.68E-13
DBT	TMX4	0.504866	6.67E-13
DBT	CIP2A	0.5048778	6.66E-13
DBT	TMEM68	0.50495631	6.59E-13
DBT	VSTM4	0.50543786	6.22E-13
DBT	NOTCH3	0.50547562	6.19E-13

DBT	NREP	0.50548869	6.18E-13
DBT	WBP1L	0.50559415	6.1E-13
DBT	FOSL2	0.50561406	6.09E-13
DBT	SOWAHC	0.50565712	6.06E-13
DBT	LXN	0.50572157	6.01E-13
DBT	STARD3NL	0.50583784	5.93E-13
DBT	C4orf3	0.50595877	5.84E-13
DBT	COL5A2	0.50601164	5.8E-13
DBT	SNX27	0.50606133	5.77E-13
DBT	SLC1A3	0.50613553	5.71E-13
DBT	ECD	0.50614379	5.71E-13
DBT	UBASH3B	0.50619608	5.67E-13
DBT	PELI1	0.5063239	5.59E-13
DBT	KREMEN1	0.50660009	5.4E-13
DBT	SYF2	0.50663079	5.38E-13
DBT	TRAFD1	0.5068235	5.26E-13
DBT	STIL	0.50707608	5.1E-13
DBT	SLC2A9	0.50724264	4.99E-13
DBT	GPNMB	0.50737085	4.92E-13
DBT	ARL15	0.50740441	4.9E-13
DBT	PDCD4	0.50743316	4.88E-13
DBT	PIAS1	0.50748592	4.85E-13
DBT	KLF6	0.50748613	4.85E-13
DBT	RALBP1	0.50753937	4.82E-13
DBT	LRIF1	0.50763826	4.76E-13
DBT	SMARCA2	0.50769228	4.73E-13
DBT	RBM22	0.50777912	4.68E-13
DBT	DSC2	0.50782356	4.65E-13
DBT	SYPL1	0.50783491	4.64E-13
DBT	EDIL3	0.50784448	4.64E-13
DBT	GINS4	0.50785821	4.63E-13
DBT	EDC3	0.5082473	4.42E-13
DBT	PRELP	0.508248	4.42E-13
DBT	CHST3	0.50845296	4.31E-13
DBT	TFCP2	0.50864021	4.21E-13
DBT	CMKLR1	0.50867425	4.19E-13
DBT	DEPTOR	0.50870987	4.17E-13
DBT	KDM5B	0.50871837	4.17E-13
DBT	SLC30A5	0.508762	4.15E-13
DBT	GJA1	0.50885354	4.1E-13
DBT	PNPO	0.50886408	4.09E-13
DBT	OSTF1	0.5090669	3.99E-13
DBT	GIMAP4	0.50917228	3.94E-13
DBT	LEO1	0.50919111	3.93E-13
DBT	MRPL3	0.50927414	3.89E-13
DBT	SLC16A1	0.50941764	3.83E-13
DBT	SNX5	0.50944181	3.81E-13
DBT	ZNF263	0.50951694	3.78E-13
DBT	SPIN4	0.50966159	3.71E-13
DBT	HSD11B1	0.5097946	3.65E-13
DBT	C3AR1	0.50979554	3.65E-13
DBT	SH3KBP1	0.50988301	3.61E-13
DBT	CLIP4	0.50989179	3.61E-13
DBT	TIPRL	0.50990347	3.6E-13
DBT	ALDH1A3	0.51000988	3.56E-13
DBT	SDHB	0.5100236	3.55E-13
DBT	VTI1A	0.51006167	3.53E-13
DBT	IFIT2	0.51007381	3.53E-13

DBT	AP3S1	0.51012522	3.51E-13
DBT	MXRA7	0.51024484	3.45E-13
DBT	CYBB	0.51045578	3.37E-13
DBT	CDC42BPB	0.51066191	3.28E-13
DBT	CALHM2	0.51085681	3.2E-13
DBT	PPCS	0.51092242	3.18E-13
DBT	AMMECR1	0.51092257	3.18E-13
DBT	PXDN	0.51107039	3.12E-13
DBT	EVI2B	0.5112056	3.07E-13
DBT	PIGS	0.51128449	3.04E-13
DBT	HDAC9	0.51156821	2.93E-13
DBT	ZDHHC5	0.51166491	2.9E-13
DBT	MPEG1	0.51169204	2.89E-13
DBT	TTC4	0.51175739	2.87E-13
DBT	ITGB3	0.51186934	2.83E-13
DBT	ASPN	0.51193205	2.8E-13
DBT	DNM1L	0.51206645	2.76E-13
DBT	KPNB1	0.51211037	2.74E-13
DBT	GLO1	0.51233762	2.67E-13
DBT	CDK2	0.51240757	2.64E-13
DBT	ZPR1	0.5124611	2.63E-13
DBT	PTPN22	0.51248569	2.62E-13
DBT	C1S	0.51255674	2.6E-13
DBT	SPRY1	0.51259529	2.58E-13
DBT	SLCO2B1	0.51261625	2.58E-13
DBT	KATNBL1	0.51266824	2.56E-13
DBT	MLH1	0.51282838	2.51E-13
DBT	OLA1	0.51285361	2.5E-13
DBT	SELENOP	0.51287078	2.5E-13
DBT	COL4A5	0.51288011	2.49E-13
DBT	POT1	0.5128921	2.49E-13
DBT	NRP2	0.51309372	2.43E-13
DBT	TSPAN14	0.51310058	2.43E-13
DBT	RASSF3	0.51313854	2.41E-13
DBT	GALNT10	0.51319399	2.4E-13
DBT	UBE2D2	0.51319424	2.4E-13
DBT	DAG1	0.51330817	2.36E-13
DBT	AXL	0.51332128	2.36E-13
DBT	CISD2	0.51342458	2.33E-13
DBT	SNAP29	0.51356292	2.29E-13
DBT	SVIP	0.51371055	2.25E-13
DBT	ZNF329	0.51375766	2.23E-13
DBT	RASSF2	0.51399124	2.17E-13
DBT	VGLL4	0.51417804	2.12E-13
DBT	DBF4	0.51418015	2.12E-13
DBT	BTN3A2	0.5143029	2.09E-13
DBT	CRISPLD2	0.51436349	2.07E-13
DBT	PRKX	0.51438312	2.07E-13
DBT	MGAT5	0.51445259	2.05E-13
DBT	BLZF1	0.51449341	2.04E-13
DBT	SLC39A14	0.51464519	2E-13
DBT	TNFSF13B	0.51473165	1.98E-13
DBT	TMEM154	0.51478607	1.97E-13
DBT	CCN4	0.51488321	1.94E-13
DBT	TIGAR	0.51489338	1.94E-13
DBT	CALM3	0.51493463	1.93E-13
DBT	AMOT	0.51532325	1.84E-13
DBT	MAP1LC3B	0.51533406	1.84E-13

DBT	SFXN1	0.51540604	1.82E-13
DBT	TPGS2	0.51559296	1.78E-13
DBT	CRYBG1	0.51574652	1.74E-13
DBT	CSF1R	0.51579085	1.73E-13
DBT	INHBA	0.51595759	1.7E-13
DBT	AFAP1	0.51615396	1.66E-13
DBT	THY1	0.51616486	1.65E-13
DBT	CFH	0.51619024	1.65E-13
DBT	RAB5C	0.51654939	1.58E-13
DBT	RNF144B	0.51659229	1.57E-13
DBT	TRIM22	0.51665588	1.55E-13
DBT	TRIM34	0.51676681	1.53E-13
DBT	POLE3	0.51697324	1.49E-13
DBT	RFK	0.51700602	1.49E-13
DBT	USP6NL	0.51721513	1.45E-13
DBT	SYNPO2	0.51725773	1.44E-13
DBT	EVI2A	0.51759513	1.38E-13
DBT	FKBP9	0.51764514	1.37E-13
DBT	DNAJB6	0.51776701	1.35E-13
DBT	UGGT1	0.51783963	1.34E-13
DBT	PPTC7	0.51785175	1.34E-13
DBT	COL4A2	0.5178895	1.33E-13
DBT	CPXM2	0.51793489	1.32E-13
DBT	GUCY1B1	0.51805068	1.3E-13
DBT	UBE2A	0.51845379	1.24E-13
DBT	VBP1	0.51846276	1.24E-13
DBT	RTN3	0.51848127	1.23E-13
DBT	TOP2B	0.5185716	1.22E-13
DBT	COL6A3	0.51874272	1.19E-13
DBT	MLLT3	0.51879995	1.19E-13
DBT	CCDC43	0.51889929	1.17E-13
DBT	SNX10	0.51896238	1.16E-13
DBT	TFPI	0.51905575	1.15E-13
DBT	MED4	0.5191909	1.13E-13
DBT	PEA15	0.51919311	1.13E-13
DBT	TMEM47	0.5193979	1.1E-13
DBT	PRDM1	0.51956469	1.08E-13
DBT	CASP8	0.51960965	1.07E-13
DBT	BRAP	0.51960983	1.07E-13
DBT	GRPEL2	0.51977637	1.05E-13
DBT	PIP4K2B	0.51992295	1.03E-13
DBT	NFYA	0.51997294	1.02E-13
DBT	IRAK3	0.52034243	9.74E-14
DBT	ADAP2	0.52048138	9.57E-14
DBT	IFT52	0.52054943	9.49E-14
DBT	GALK2	0.52075734	9.24E-14
DBT	LHFPL6	0.52079403	9.2E-14
DBT	MBIP	0.520872	9.11E-14
DBT	MAP1B	0.5209754	8.99E-14
DBT	FGF2	0.5209955	8.97E-14
DBT	IQGAP1	0.52111149	8.83E-14
DBT	DYNLT3	0.52123479	8.7E-14
DBT	INO80	0.52126996	8.66E-14
DBT	PPP1R15B	0.52143198	8.48E-14
DBT	ITGA2	0.52149274	8.41E-14
DBT	MFHAS1	0.52149817	8.41E-14
DBT	BCORL1	0.52157555	8.32E-14
DBT	PRKCH	0.52177317	8.12E-14

DBT	LBR	0.52205397	7.83E-14
DBT	STK17B	0.52223374	7.65E-14
DBT	CPA3	0.52225396	7.63E-14
DBT	DCLRE1C	0.52229331	7.59E-14
DBT	TTC23	0.52237309	7.52E-14
DBT	TLR5	0.52243941	7.45E-14
DBT	WWC3	0.522532	7.36E-14
DBT	EXOC4	0.52262253	7.28E-14
DBT	TADA1	0.52279977	7.12E-14
DBT	VPS35L	0.52281361	7.1E-14
DBT	SERPINB8	0.52293319	6.99E-14
DBT	SHROOM3	0.52293419	6.99E-14
DBT	ANKRD42	0.52302258	6.91E-14
DBT	NOLC1	0.52313871	6.81E-14
DBT	CXorf38	0.52317909	6.78E-14
DBT	LMNB1	0.52322747	6.73E-14
DBT	HECW2	0.52341393	6.57E-14
DBT	CAPZB	0.52351806	6.49E-14
DBT	ARCN1	0.52355905	6.45E-14
DBT	PLEKHF2	0.52396213	6.13E-14
DBT	MED6	0.52415303	5.98E-14
DBT	UTP25	0.52416534	5.97E-14
DBT	CAST	0.52457569	5.66E-14
DBT	CUL4A	0.52463617	5.62E-14
DBT	EXTL3	0.52476751	5.52E-14
DBT	GABPB1	0.52486317	5.45E-14
DBT	SGO2	0.52486934	5.45E-14
DBT	ATPSCKMT	0.52501872	5.34E-14
DBT	SPSB1	0.52505464	5.32E-14
DBT	ZNF462	0.52508488	5.3E-14
DBT	AP4S1	0.52510666	5.28E-14
DBT	SLC25A13	0.52511602	5.28E-14
DBT	LDB2	0.52530487	5.15E-14
DBT	CPOX	0.52532826	5.14E-14
DBT	PAMR1	0.5253338	5.13E-14
DBT	SERTAD2	0.52541782	5.08E-14
DBT	ANKMY2	0.52555261	4.99E-14
DBT	OAT	0.52562251	4.94E-14
DBT	MED18	0.52573165	4.87E-14
DBT	AP1S3	0.52581076	4.82E-14
DBT	CASP3	0.52601739	4.7E-14
DBT	GIMAP8	0.52606566	4.67E-14
DBT	PRPF18	0.52670629	4.29E-14
DBT	HNRNPF	0.52671037	4.29E-14
DBT	URI1	0.52674717	4.27E-14
DBT	CPNE8	0.52693686	4.17E-14
DBT	RAB28	0.52746559	3.89E-14
DBT	RASA2	0.52765446	3.79E-14
DBT	NCEH1	0.52781937	3.71E-14
DBT	CNOT2	0.52804713	3.61E-14
DBT	SETX	0.52864931	3.33E-14
DBT	RAP1B	0.52871928	3.3E-14
DBT	ARF4	0.52900681	3.18E-14
DBT	GLB1	0.52904652	3.16E-14
DBT	TRERF1	0.52914021	3.12E-14
DBT	ATP9A	0.52920412	3.1E-14
DBT	TEX10	0.52933527	3.05E-14
DBT	HMGB1	0.5293546	3.04E-14

DBT	MAP3K13	0.52938253	3.03E-14
DBT	ADAT1	0.5294356	3.01E-14
DBT	CHRAC1	0.5295029	2.98E-14
DBT	SPART	0.52950307	2.98E-14
DBT	FKBP7	0.5296155	2.94E-14
DBT	LRBA	0.52965579	2.92E-14
DBT	SLFN12	0.5296743	2.91E-14
DBT	GNS	0.52973087	2.89E-14
DBT	ARMH4	0.5298366	2.85E-14
DBT	DCTN6	0.52993337	2.82E-14
DBT	TCF20	0.52999024	2.79E-14
DBT	HDAC2	0.53013903	2.74E-14
DBT	SLC17A5	0.53014796	2.74E-14
DBT	MFSD11	0.53016894	2.73E-14
DBT	BHLHE41	0.53020484	2.72E-14
DBT	FEM1C	0.5302708	2.69E-14
DBT	MAF	0.53042523	2.64E-14
DBT	PEX3	0.53043164	2.64E-14
DBT	PRKDC	0.53065258	2.56E-14
DBT	CUL1	0.53070396	2.54E-14
DBT	KIAA1217	0.53075243	2.53E-14
DBT	PSIP1	0.53089254	2.48E-14
DBT	SUCLG2	0.5309049	2.48E-14
DBT	SART3	0.53092228	2.47E-14
DBT	TDRD7	0.53092852	2.47E-14
DBT	DDX58	0.53098658	2.45E-14
DBT	HCCS	0.53111558	2.41E-14
DBT	DNAJC8	0.53124339	2.37E-14
DBT	ELP3	0.53140569	2.32E-14
DBT	DLC1	0.53142376	2.31E-14
DBT	EIF1AD	0.53147788	2.3E-14
DBT	DCTN5	0.53167231	2.24E-14
DBT	ARRDC3	0.53183458	2.19E-14
DBT	SNX29	0.53186269	2.18E-14
DBT	CSTF2	0.53190445	2.17E-14
DBT	ZCCHC17	0.53194807	2.16E-14
DBT	EAPP	0.53213197	2.11E-14
DBT	ZNF28	0.53215567	2.1E-14
DBT	TMLHE	0.53218187	2.09E-14
DBT	PGM2L1	0.53234348	2.05E-14
DBT	SIAH1	0.53239888	2.03E-14
DBT	NCL	0.53241502	2.03E-14
DBT	POGK	0.53249715	2.01E-14
DBT	OSMR	0.53253861	2E-14
DBT	ENC1	0.53254528	1.99E-14
DBT	FADS1	0.53272794	1.95E-14
DBT	PRR16	0.53274396	1.94E-14
DBT	ERI1	0.53278938	1.93E-14
DBT	TMEM182	0.53280383	1.93E-14
DBT	GALNT7	0.53281382	1.92E-14
DBT	POLR2C	0.53288594	1.91E-14
DBT	ZNF350	0.53295194	1.89E-14
DBT	VPS33A	0.53298236	1.88E-14
DBT	MICAL2	0.53301497	1.87E-14
DBT	IFIT5	0.53307978	1.86E-14
DBT	CPPED1	0.53319551	1.83E-14
DBT	DERA	0.53348907	1.76E-14
DBT	FAM210A	0.53367124	1.72E-14

DBT	FPR3	0.53373631	1.7E-14
DBT	FER	0.53381899	1.68E-14
DBT	CNOT9	0.53401204	1.64E-14
DBT	GNB5	0.53402225	1.64E-14
DBT	KSR1	0.53407167	1.63E-14
DBT	BBS12	0.53423632	1.59E-14
DBT	SNW1	0.53442916	1.55E-14
DBT	TRIM56	0.53485164	1.47E-14
DBT	DPY19L1	0.53487624	1.46E-14
DBT	FAM118B	0.53496618	1.44E-14
DBT	RNASEL	0.53518042	1.4E-14
DBT	AC008764.4	0.53521544	1.4E-14
DBT	COL8A1	0.53524128	1.39E-14
DBT	TMTC1	0.53528534	1.38E-14
DBT	DKK2	0.53535585	1.37E-14
DBT	STARD7	0.53539622	1.36E-14
DBT	MYO1E	0.5355018	1.34E-14
DBT	SVIL	0.53594571	1.27E-14
DBT	CLDND1	0.53613845	1.23E-14
DBT	ETV1	0.53617846	1.23E-14
DBT	FEZ2	0.53622883	1.22E-14
DBT	KIF3C	0.53625133	1.22E-14
DBT	SFT2D2	0.53652552	1.17E-14
DBT	PLSCR1	0.53676803	1.13E-14
DBT	PCDH7	0.53688394	1.12E-14
DBT	SCOC	0.53695953	1.11E-14
DBT	GOLPH3L	0.53705763	1.09E-14
DBT	NKRF	0.53710968	1.08E-14
DBT	GCC1	0.53723566	1.07E-14
DBT	RAB7A	0.53731057	1.05E-14
DBT	PCGF5	0.53733518	1.05E-14
DBT	COPS4	0.53740383	1.04E-14
DBT	ERMP1	0.53743886	1.04E-14
DBT	CTDSPL	0.5374477	1.04E-14
DBT	ST8SIA4	0.53745945	1.03E-14
DBT	STS	0.53749276	1.03E-14
DBT	RNASEH1	0.53767647	1E-14
DBT	WDR20	0.53778957	9.88E-15
DBT	MEAF6	0.53787002	9.78E-15
DBT	CIR1	0.53803712	9.56E-15
DBT	CARD6	0.53806784	9.52E-15
DBT	THSD4	0.53811371	9.46E-15
DBT	ANKRD27	0.53811443	9.46E-15
DBT	SELENON	0.53813775	9.43E-15
DBT	SMU1	0.53822216	9.32E-15
DBT	NCBP2	0.53825505	9.28E-15
DBT	ADO	0.53830968	9.21E-15
DBT	OAZ2	0.53830999	9.21E-15
DBT	EHD3	0.53834813	9.17E-15
DBT	NEMP2	0.53840275	9.1E-15
DBT	ETV3	0.53848881	8.99E-15
DBT	ZC3H11A	0.53856032	8.91E-15
DBT	PARN	0.53857228	8.89E-15
DBT	BACE1	0.53874772	8.68E-15
DBT	NT5C2	0.53878021	8.65E-15
DBT	MAP4	0.53890143	8.51E-15
DBT	GLI2	0.53894704	8.45E-15
DBT	DCTD	0.53897076	8.43E-15

DBT	ZNF410	0.53897692	8.42E-15
DBT	FAM220A	0.53908722	8.29E-15
DBT	TSC22D2	0.53921627	8.15E-15
DBT	ANTXR1	0.53949741	7.85E-15
DBT	ACTR6	0.53978969	7.54E-15
DBT	YPEL5	0.53981269	7.52E-15
DBT	PHF20	0.53986481	7.46E-15
DBT	ADORA3	0.53986544	7.46E-15
DBT	MUL1	0.53995471	7.37E-15
DBT	ADGRA2	0.54000451	7.32E-15
DBT	ADAM22	0.54019263	7.14E-15
DBT	INSIG2	0.5404192	6.92E-15
DBT	RCAN3	0.54046885	6.87E-15
DBT	ANAPC10	0.54056467	6.79E-15
DBT	SYNC	0.54065963	6.7E-15
DBT	IFIH1	0.54072557	6.64E-15
DBT	GRAMD2B	0.54092842	6.46E-15
DBT	BBS9	0.54115841	6.26E-15
DBT	MEIS2	0.54138487	6.07E-15
DBT	ZNF267	0.5414196	6.04E-15
DBT	LACTB	0.54170559	5.81E-15
DBT	SEMA5A	0.54175232	5.77E-15
DBT	PRPF38A	0.5417549	5.77E-15
DBT	ATXN7L3B	0.54184978	5.69E-15
DBT	BBS7	0.54214025	5.47E-15
DBT	ATG7	0.54227054	5.38E-15
DBT	SCFD2	0.54242104	5.27E-15
DBT	VKORC1L1	0.54251028	5.2E-15
DBT	LCLAT1	0.54258205	5.15E-15
DBT	AGAP1	0.54269762	5.07E-15
DBT	ATXN3	0.54285111	4.96E-15
DBT	RIT1	0.54285523	4.96E-15
DBT	INTS12	0.54286151	4.96E-15
DBT	HNRNPA2B1	0.54296605	4.89E-15
DBT	SLC39A9	0.54299334	4.87E-15
DBT	ZFAND5	0.54307166	4.82E-15
DBT	PPP2R2A	0.54311527	4.79E-15
DBT	ZNF816	0.54326863	4.69E-15
DBT	ZHX2	0.54350255	4.54E-15
DBT	UPRT	0.54368028	4.43E-15
DBT	ITPR1	0.54370878	4.41E-15
DBT	CLIC2	0.54371554	4.41E-15
DBT	LDLRAD4	0.54374244	4.39E-15
DBT	FLI1	0.54379058	4.36E-15
DBT	RAB35	0.54386106	4.32E-15
DBT	LRCH3	0.54399102	4.24E-15
DBT	IGFBP5	0.54430959	4.06E-15
DBT	FAM83B	0.54431209	4.06E-15
DBT	DKK3	0.54437023	4.03E-15
DBT	ISG20L2	0.54440846	4.01E-15
DBT	MPZL1	0.54449754	3.96E-15
DBT	COL15A1	0.54453193	3.94E-15
DBT	TMEM263	0.54461138	3.9E-15
DBT	LACTB2	0.54480971	3.79E-15
DBT	TSHZ2	0.54489545	3.75E-15
DBT	RAPH1	0.54502712	3.68E-15
DBT	NXPE3	0.54508322	3.65E-15
DBT	SAR1B	0.54517971	3.6E-15

DBT	NAGA	0.54521625	3.58E-15
DBT	SKP1	0.54537258	3.51E-15
DBT	CYP1B1	0.54551762	3.44E-15
DBT	OMD	0.54581823	3.3E-15
DBT	FLT1	0.54584722	3.28E-15
DBT	SRP9	0.54598419	3.22E-15
DBT	CELF2	0.54599284	3.22E-15
DBT	MAP4K4	0.54603458	3.2E-15
DBT	SPOCK1	0.54610928	3.17E-15
DBT	TMEM140	0.5461492	3.15E-15
DBT	CHSY3	0.54619137	3.13E-15
DBT	NUAK1	0.54638466	3.05E-15
DBT	FBXO34	0.54639262	3.04E-15
DBT	SGCD	0.54639295	3.04E-15
DBT	CLMP	0.54648651	3E-15
DBT	MRPL49	0.54651484	2.99E-15
DBT	RFFL	0.54653475	2.98E-15
DBT	CAV2	0.54662293	2.95E-15
DBT	SLC24A1	0.54669094	2.92E-15
DBT	TMEM87A	0.54672385	2.91E-15
DBT	AIMP1	0.54676492	2.89E-15
DBT	LCP2	0.5467866	2.88E-15
DBT	LRRC41	0.54692532	2.83E-15
DBT	THBS1	0.54695586	2.82E-15
DBT	OXSR1	0.54701814	2.79E-15
DBT	PIP5K1A	0.54706522	2.77E-15
DBT	CORO1C	0.5470845	2.77E-15
DBT	SC5D	0.54720092	2.72E-15
DBT	EIF5A2	0.54734709	2.67E-15
DBT	CYFIP1	0.5475833	2.58E-15
DBT	RHOA	0.54763043	2.56E-15
DBT	MYH10	0.54786242	2.48E-15
DBT	RAB9A	0.5480336	2.42E-15
DBT	OXCT1	0.54804996	2.42E-15
DBT	LDB1	0.5481005	2.4E-15
DBT	TPP2	0.54823321	2.36E-15
DBT	PANX1	0.54824869	2.35E-15
DBT	TMED10	0.54833289	2.32E-15
DBT	KIF20B	0.5484254	2.29E-15
DBT	ZKSCAN5	0.54846176	2.28E-15
DBT	PSMD12	0.54856188	2.25E-15
DBT	BTBD10	0.54857692	2.25E-15
DBT	PDGFRB	0.54873344	2.2E-15
DBT	LRP10	0.54882375	2.17E-15
DBT	DLST	0.54885541	2.16E-15
DBT	RTN4	0.54895516	2.13E-15
DBT	PRCP	0.54897358	2.12E-15
DBT	GHITM	0.54905792	2.1E-15
DBT	AFAP1L1	0.54909826	2.09E-15
DBT	PIGW	0.54910107	2.09E-15
DBT	RPA2	0.54913442	2.08E-15
DBT	SP2	0.54941795	2E-15
DBT	TEK	0.54952784	1.97E-15
DBT	TMEM200A	0.54964316	1.93E-15
DBT	TMEM126B	0.54993781	1.86E-15
DBT	MAP2K1	0.55007522	1.82E-15
DBT	DSG2	0.55014672	1.8E-15
DBT	PTPN4	0.55015733	1.8E-15

DBT	FCGR2A	0.5502158	1.78E-15
DBT	ZWILCH	0.55042666	1.73E-15
DBT	SMAD7	0.55055106	1.7E-15
DBT	YWHAG	0.5508813	1.62E-15
DBT	ISCA1	0.55092077	1.62E-15
DBT	RARB	0.55093724	1.61E-15
DBT	LTBP2	0.55097967	1.6E-15
DBT	LYN	0.55099911	1.6E-15
DBT	ZNRF2	0.55112954	1.57E-15
DBT	BICD2	0.55128857	1.53E-15
DBT	EMC3	0.55155599	1.48E-15
DBT	UGCG	0.55155983	1.48E-15
DBT	FGF7	0.55161951	1.46E-15
DBT	DCP2	0.55164306	1.46E-15
DBT	PAFAH1B1	0.55174526	1.44E-15
DBT	SPARCL1	0.55197316	1.39E-15
DBT	RIN2	0.55222826	1.34E-15
DBT	JARID2	0.55235358	1.32E-15
DBT	DCBLD1	0.55238016	1.31E-15
DBT	NAPG	0.55240406	1.31E-15
DBT	PTPRC	0.55285999	1.23E-15
DBT	LINS1	0.5530526	1.2E-15
DBT	RASA1	0.55349602	1.12E-15
DBT	ATP6AP2	0.55350642	1.12E-15
DBT	TAF5L	0.55351556	1.12E-15
DBT	PNO1	0.55359353	1.11E-15
DBT	SAR1A	0.55364295	1.1E-15
DBT	PEX13	0.55366435	1.1E-15
DBT	CDK6	0.55419818	1.02E-15
DBT	OTULIN	0.55420044	1.02E-15
DBT	AP2B1	0.55422687	1.01E-15
DBT	MARK2	0.55441069	9.85E-16
DBT	PGD	0.55466355	9.5E-16
DBT	UBE2E2	0.55470434	9.45E-16
DBT	SH3BP5	0.55471678	9.43E-16
DBT	TSPYL1	0.55472072	9.42E-16
DBT	RAB1A	0.55473275	9.41E-16
DBT	GREM1	0.55498904	9.07E-16
DBT	PSMC2	0.55500687	9.05E-16
DBT	RCN2	0.5550793	8.95E-16
DBT	LATS2	0.55538342	8.57E-16
DBT	BPTF	0.55559663	8.31E-16
DBT	FASTKD5	0.55566383	8.24E-16
DBT	PTPN12	0.55574847	8.14E-16
DBT	NUDCD3	0.5558418	8.03E-16
DBT	PTP4A2	0.55599392	7.85E-16
DBT	KIAA1671	0.55630127	7.52E-16
DBT	ZNF143	0.55631974	7.5E-16
DBT	ZBTB4	0.55656599	7.24E-16
DBT	RMND5A	0.55658185	7.22E-16
DBT	PFKM	0.55667802	7.12E-16
DBT	MAPRE1	0.55678084	7.02E-16
DBT	CASK	0.55703463	6.76E-16
DBT	BROX	0.55707278	6.73E-16
DBT	CMTM6	0.55717014	6.63E-16
DBT	SLC31A1	0.55719006	6.61E-16
DBT	LRRFIP1	0.55729506	6.52E-16
DBT	PSMD10	0.55741971	6.4E-16

DBT	RNF139	0.55753489	6.29E-16
DBT	RALB	0.55780188	6.06E-16
DBT	ZNF131	0.55786449	6E-16
DBT	FAM114A1	0.55794587	5.93E-16
DBT	DAZAP2	0.55808842	5.81E-16
DBT	PROS1	0.55836011	5.59E-16
DBT	ACER3	0.55849769	5.48E-16
DBT	PITPNB	0.55879684	5.25E-16
DBT	HUS1	0.5588308	5.22E-16
DBT	MED21	0.55886951	5.19E-16
DBT	PRRC2C	0.55892427	5.15E-16
DBT	IDS	0.55901001	5.09E-16
DBT	ZNF423	0.5590886	5.03E-16
DBT	FOXO1	0.55924391	4.92E-16
DBT	TSHZ3	0.55932642	4.86E-16
DBT	PTGS1	0.55940592	4.8E-16
DBT	CRTAP	0.55962211	4.65E-16
DBT	CKAP2	0.55984681	4.5E-16
DBT	ZCCHC24	0.55998925	4.41E-16
DBT	BCL9	0.56013245	4.32E-16
DBT	DCN	0.56027784	4.23E-16
DBT	PI4K2B	0.56036236	4.18E-16
DBT	SLC4A1AP	0.56036302	4.18E-16
DBT	RFC3	0.56058506	4.05E-16
DBT	CENPL	0.56059943	4.04E-16
DBT	HERC3	0.56064558	4.01E-16
DBT	PRRG1	0.56097265	3.82E-16
DBT	OSBPL10	0.56104153	3.79E-16
DBT	ACLY	0.56114435	3.73E-16
DBT	MOXD1	0.56120576	3.7E-16
DBT	MAP1A	0.56138534	3.6E-16
DBT	IMPACT	0.5614235	3.58E-16
DBT	CHML	0.56147902	3.55E-16
DBT	HDGFL3	0.56148892	3.55E-16
DBT	OTUD1	0.56159948	3.49E-16
DBT	SSB	0.56170363	3.44E-16
DBT	ZNF398	0.56177591	3.4E-16
DBT	KRAS	0.5618473	3.37E-16
DBT	RAB31	0.56193969	3.32E-16
DBT	MSR1	0.56197004	3.31E-16
DBT	SESN3	0.56240097	3.1E-16
DBT	NIPSNAP3A	0.56250131	3.06E-16
DBT	PIGX	0.56273045	2.96E-16
DBT	PARP8	0.56282152	2.92E-16
DBT	TNPO3	0.56282318	2.92E-16
DBT	XRCC5	0.56286655	2.9E-16
DBT	PRKACB	0.5632488	2.74E-16
DBT	FILIP1L	0.56330794	2.72E-16
DBT	RERG	0.56357661	2.61E-16
DBT	DIP2B	0.56374333	2.55E-16
DBT	PDCD10	0.56381337	2.52E-16
DBT	IST1	0.56381758	2.52E-16
DBT	CAMKK2	0.56402722	2.44E-16
DBT	DIAPH2	0.56402919	2.44E-16
DBT	GIMAP6	0.56409812	2.42E-16
DBT	PCNX4	0.56416918	2.39E-16
DBT	TBC1D9	0.5642039	2.38E-16
DBT	EDNRA	0.56421966	2.38E-16

DBT	AKAP12	0.56422505	2.37E-16
DBT	LAP3	0.56432285	2.34E-16
DBT	AP003108.2	0.56452821	2.27E-16
DBT	AGFG1	0.56467079	2.22E-16
DBT	ASH2L	0.56467879	2.22E-16
DBT	PLRG1	0.56473316	2.2E-16
DBT	UXS1	0.56494725	2.13E-16
DBT	KCND2	0.56505315	2.1E-16
DBT	SPON1	0.56527381	2.03E-16
DBT	PEX26	0.56549476	1.97E-16
DBT	MAST4	0.56574178	1.9E-16
DBT	CCNI	0.56574575	1.9E-16
DBT	ZNF200	0.56584964	1.87E-16
DBT	TMEM185B	0.56595723	1.84E-16
DBT	NUCKS1	0.56615053	1.79E-16
DBT	GPBP1L1	0.56625854	1.76E-16
DBT	TM6SF1	0.56634899	1.73E-16
DBT	HSPA14	0.56637412	1.73E-16
DBT	SKI	0.56647968	1.7E-16
DBT	DCHS1	0.56657841	1.68E-16
DBT	HIBADH	0.56662282	1.66E-16
DBT	ERG	0.56667344	1.65E-16
DBT	JKAMP	0.56683711	1.61E-16
DBT	KIF13A	0.5670717	1.56E-16
DBT	FAM204A	0.56708751	1.55E-16
DBT	TMEM167A	0.56712512	1.54E-16
DBT	TDP1	0.56734903	1.49E-16
DBT	GLYR1	0.56759663	1.44E-16
DBT	CCDC6	0.56763861	1.43E-16
DBT	PRNP	0.56787541	1.38E-16
DBT	JAM3	0.56820555	1.32E-16
DBT	NFE2L1	0.56829563	1.3E-16
DBT	GTDC1	0.56836706	1.28E-16
DBT	CBX1	0.56855311	1.25E-16
DBT	IARS2	0.56863331	1.23E-16
DBT	KDM2A	0.56875933	1.21E-16
DBT	GLE1	0.56881479	1.2E-16
DBT	NAMPT	0.56883829	1.2E-16
DBT	GPN3	0.56908995	1.15E-16
DBT	SLU7	0.56922574	1.13E-16
DBT	CCNK	0.56927703	1.12E-16
DBT	ASNSD1	0.56937183	1.1E-16
DBT	PDE1A	0.56942201	1.1E-16
DBT	NPM1	0.5698702	1.02E-16
DBT	SRSF3	0.56991223	1.02E-16
DBT	CBX5	0.56993491	1.01E-16
DBT	ZFP64	0.56993962	1.01E-16
DBT	GBP1	0.56995222	1.01E-16
DBT	CDON	0.56997671	1.01E-16
DBT	VPS37A	0.57004789	9.98E-17
DBT	TMEM209	0.57007717	9.93E-17
DBT	TRIM69	0.57051478	9.3E-17
DBT	DEGS1	0.57100923	8.63E-17
DBT	DPF2	0.57103556	8.6E-17
DBT	MOB3B	0.57125483	8.32E-17
DBT	ZNFX1	0.57129183	8.27E-17
DBT	MRAS	0.57130777	8.25E-17
DBT	WASHC2A	0.57133746	8.22E-17

DBT	RMDN2	0.57137109	8.17E-17
DBT	ABL2	0.57151763	8E-17
DBT	FYB1	0.57159726	7.9E-17
DBT	LHFPL2	0.57170029	7.78E-17
DBT	CLINT1	0.57176129	7.71E-17
DBT	MRPL35	0.57180757	7.65E-17
DBT	B4GALT5	0.57187334	7.58E-17
DBT	TM9SF3	0.57192557	7.52E-17
DBT	LOX	0.57212763	7.29E-17
DBT	SLC35A4	0.57230297	7.1E-17
DBT	VIPAS39	0.57245618	6.94E-17
DBT	GDE1	0.57252311	6.87E-17
DBT	C9orf78	0.57256631	6.82E-17
DBT	SOGA1	0.57268059	6.7E-17
DBT	FASTKD2	0.57281606	6.57E-17
DBT	DAB2	0.5730524	6.34E-17
DBT	SNX3	0.57311066	6.28E-17
DBT	EPS8	0.57311202	6.28E-17
DBT	PSEN1	0.5734019	6.01E-17
DBT	HNRNPU	0.57363066	5.8E-17
DBT	PPP3CB	0.57385062	5.61E-17
DBT	ARSJ	0.57391419	5.56E-17
DBT	COPB1	0.57404767	5.45E-17
DBT	PARP14	0.57405432	5.44E-17
DBT	GLOD4	0.57408898	5.41E-17
DBT	ERGIC2	0.57419876	5.32E-17
DBT	KLF3	0.57453353	5.06E-17
DBT	CLP1	0.57469873	4.93E-17
DBT	NRBF2	0.57482655	4.84E-17
DBT	TRRAP	0.57485912	4.81E-17
DBT	AASS	0.57490579	4.78E-17
DBT	HMGN4	0.57501828	4.7E-17
DBT	SBF2	0.57529651	4.5E-17
DBT	FXR1	0.57535751	4.46E-17
DBT	SGPL1	0.57542491	4.41E-17
DBT	SMIM15	0.57572827	4.21E-17
DBT	TAX1BP1	0.57580012	4.17E-17
DBT	MORF4L2	0.57585842	4.13E-17
DBT	VEZT	0.5758678	4.12E-17
DBT	TRA2B	0.57594719	4.07E-17
DBT	WIPF1	0.57602111	4.03E-17
DBT	IL13RA1	0.57612722	3.96E-17
DBT	MSN	0.57627376	3.87E-17
DBT	NAV1	0.57631223	3.85E-17
DBT	IPO11	0.57641814	3.79E-17
DBT	VCL	0.57651518	3.73E-17
DBT	SLC16A2	0.57662626	3.67E-17
DBT	RNF170	0.57686217	3.54E-17
DBT	TMEM33	0.57696987	3.48E-17
DBT	CAP1	0.57700399	3.46E-17
DBT	PLBD2	0.57708564	3.42E-17
DBT	SS18	0.57722523	3.35E-17
DBT	RRP15	0.57730099	3.31E-17
DBT	LIN52	0.57735807	3.28E-17
DBT	C1QTNF7	0.57740496	3.26E-17
DBT	TMEM237	0.57744676	3.24E-17
DBT	SPATS2	0.57761162	3.15E-17
DBT	WBP4	0.57767559	3.12E-17

DBT	MYO6	0.57800194	2.97E-17
DBT	USP12	0.57824887	2.86E-17
DBT	ADD3	0.57854842	2.73E-17
DBT	MACF1	0.57886329	2.6E-17
DBT	VAPA	0.57894396	2.57E-17
DBT	RNF121	0.57910155	2.5E-17
DBT	ITM2B	0.57924105	2.45E-17
DBT	YEATS2	0.57936079	2.41E-17
DBT	CCR1	0.5794113	2.39E-17
DBT	LPIN2	0.57973918	2.27E-17
DBT	TULP3	0.57989012	2.22E-17
DBT	RNGTT	0.57993161	2.2E-17
DBT	AC010132.3	0.5801417	2.13E-17
DBT	LAMTOR3	0.5805191	2.01E-17
DBT	CALM1	0.58066982	1.96E-17
DBT	OSBPL1A	0.58067237	1.96E-17
DBT	CHM	0.58100123	1.86E-17
DBT	AKTIP	0.58105891	1.85E-17
DBT	ARL2BP	0.58111786	1.83E-17
DBT	ALS2	0.5811576	1.82E-17
DBT	EHD4	0.58148835	1.73E-17
DBT	KDM3B	0.5816611	1.68E-17
DBT	RAI14	0.58168486	1.68E-17
DBT	NIPA2	0.58181048	1.64E-17
DBT	FBXO5	0.58198373	1.6E-17
DBT	RPAP3	0.58204756	1.58E-17
DBT	PDHB	0.58212265	1.56E-17
DBT	DERL1	0.58231644	1.52E-17
DBT	TWF1	0.58257218	1.46E-17
DBT	ECHDC1	0.58263866	1.44E-17
DBT	SKAP2	0.58272375	1.42E-17
DBT	ABCB10	0.58290953	1.38E-17
DBT	GLIPR1	0.58304975	1.35E-17
DBT	PIP4P2	0.58314239	1.33E-17
DBT	ATAD1	0.58317463	1.33E-17
DBT	NUB1	0.5834666	1.27E-17
DBT	LRRC57	0.58353877	1.25E-17
DBT	PPP2R5D	0.5835455	1.25E-17
DBT	DYNC1LI1	0.5835461	1.25E-17
DBT	HEATR5A	0.58368229	1.22E-17
DBT	OLFML1	0.583706	1.22E-17
DBT	UBE2D1	0.58387403	1.19E-17
DBT	RPRD1B	0.58392379	1.18E-17
DBT	TAF1A	0.58422745	1.12E-17
DBT	ABI3BP	0.58447754	1.08E-17
DBT	MBTPS1	0.5845145	1.07E-17
DBT	IQCK	0.58466827	1.05E-17
DBT	UBE2N	0.58474898	1.03E-17
DBT	YWHAB	0.58496211	1E-17
DBT	SLC6A6	0.58519558	9.64E-18
DBT	PRKACA	0.58521177	9.62E-18
DBT	UBLCP1	0.58530312	9.48E-18
DBT	TVP23B	0.58554405	9.13E-18
DBT	FBN1	0.58562124	9.01E-18
DBT	UBE2V2	0.58573193	8.86E-18
DBT	ARHGAP11A	0.58575238	8.83E-18
DBT	APP	0.58579156	8.77E-18
DBT	SNX1	0.58584674	8.7E-18

DBT	HECA	0.58585107	8.69E-18
DBT	STAU1	0.58616992	8.26E-18
DBT	VCAN	0.58618202	8.25E-18
DBT	PLEKHG1	0.58629892	8.09E-18
DBT	BNC2	0.58637619	8E-18
DBT	GLCE	0.58642048	7.94E-18
DBT	MSANTD3-TMEFF1	0.58642759	7.93E-18
DBT	STIM1	0.58644396	7.91E-18
DBT	PLEKHA2	0.58648461	7.86E-18
DBT	ZFYVE1	0.58649833	7.84E-18
DBT	ZFPM2	0.58663133	7.68E-18
DBT	ZNF521	0.58683273	7.44E-18
DBT	CHSY1	0.58683881	7.43E-18
DBT	PITPNA	0.58698375	7.26E-18
DBT	PAFAH1B2	0.5870122	7.23E-18
DBT	FOXP1	0.58709645	7.13E-18
DBT	ARHGEF6	0.58719815	7.02E-18
DBT	ISY1-RAB43	0.58729325	6.91E-18
DBT	CHORDC1	0.58734076	6.86E-18
DBT	LIPA	0.58737735	6.82E-18
DBT	FRMD6	0.58744078	6.75E-18
DBT	ZNF207	0.587485	6.7E-18
DBT	ATG3	0.58802697	6.15E-18
DBT	DFFA	0.58807801	6.1E-18
DBT	TMEM43	0.58836511	5.82E-18
DBT	C16orf70	0.58838048	5.81E-18
DBT	ZDHHC20	0.58866992	5.55E-18
DBT	RNF24	0.58870321	5.52E-18
DBT	SMC4	0.58875489	5.47E-18
DBT	RAB5B	0.58879518	5.44E-18
DBT	SH2B3	0.58888064	5.36E-18
DBT	TANC2	0.58913371	5.15E-18
DBT	DNAJC13	0.58950546	4.85E-18
DBT	SPCS2	0.58962216	4.76E-18
DBT	EXT1	0.58989362	4.56E-18
DBT	RAB8A	0.58993139	4.53E-18
DBT	MAPRE2	0.5900298	4.46E-18
DBT	CHMP2B	0.5901606	4.36E-18
DBT	PARP4	0.59046794	4.15E-18
DBT	CLN5	0.59068598	4.01E-18
DBT	EFEMP1	0.59078495	3.95E-18
DBT	SLC39A6	0.59086237	3.9E-18
DBT	GSPT1	0.59091904	3.86E-18
DBT	ARHGAP35	0.59096564	3.83E-18
DBT	PRPF4	0.59109247	3.76E-18
DBT	CACNA2D1	0.59112088	3.74E-18
DBT	CD2AP	0.59129303	3.64E-18
DBT	SNX9	0.59131172	3.62E-18
DBT	RPA1	0.5913196	3.62E-18
DBT	EHBP1	0.59133184	3.61E-18
DBT	ABL1	0.59138829	3.58E-18
DBT	ABHD2	0.59139331	3.58E-18
DBT	KCTD12	0.59151487	3.51E-18
DBT	ATL2	0.5916036	3.46E-18
DBT	CMPK1	0.59165562	3.43E-18
DBT	TEFM	0.59169821	3.41E-18
DBT	KBTBD2	0.59171576	3.4E-18
DBT	B3GALNT1	0.59171634	3.4E-18

DBT	DCUN1D3	0.5917886	3.36E-18
DBT	ITGA9	0.59183168	3.33E-18
DBT	MSRB3	0.59223045	3.12E-18
DBT	PURB	0.59242667	3.03E-18
DBT	ERC1	0.59251802	2.98E-18
DBT	KLHL5	0.59257322	2.95E-18
DBT	CYTH3	0.59296851	2.77E-18
DBT	MANBA	0.59360227	2.5E-18
DBT	CPD	0.59369814	2.46E-18
DBT	FRMD4B	0.59396931	2.35E-18
DBT	UBXN2A	0.59397006	2.35E-18
DBT	PATL1	0.59400265	2.34E-18
DBT	TMEM123	0.5940846	2.31E-18
DBT	PSME3	0.59410469	2.3E-18
DBT	MOB4	0.59421308	2.26E-18
DBT	MCC	0.59427463	2.24E-18
DBT	ITFG1	0.59436719	2.21E-18
DBT	DACT1	0.59441415	2.19E-18
DBT	JRKL	0.59455135	2.14E-18
DBT	H6PD	0.59457356	2.13E-18
DBT	TANK	0.59485599	2.04E-18
DBT	ATP11A	0.59493941	2.01E-18
DBT	ALDH1L2	0.59500576	1.99E-18
DBT	WWTR1	0.59509835	1.96E-18
DBT	TRAPPC6B	0.59542468	1.86E-18
DBT	FERMT2	0.59547825	1.84E-18
DBT	SAMD8	0.59564385	1.79E-18
DBT	CTNNB1	0.59604831	1.67E-18
DBT	DYRK2	0.59618177	1.64E-18
DBT	CACUL1	0.59648105	1.56E-18
DBT	CRKL	0.59649385	1.56E-18
DBT	STK4	0.5965592	1.54E-18
DBT	CALU	0.59708538	1.41E-18
DBT	POLR2D	0.59745324	1.33E-18
DBT	CDV3	0.59752377	1.31E-18
DBT	FRRS1	0.5978366	1.25E-18
DBT	SRSF1	0.59790755	1.23E-18
DBT	HNRNPUL1	0.59814421	1.19E-18
DBT	RSPH3	0.59835809	1.14E-18
DBT	CDH11	0.59852002	1.11E-18
DBT	ADAM9	0.59856516	1.11E-18
DBT	ARL1	0.59860854	1.1E-18
DBT	EAF1	0.59866038	1.09E-18
DBT	PDGFC	0.59869964	1.08E-18
DBT	CHMP3	0.59872283	1.08E-18
DBT	PLS3	0.59876293	1.07E-18
DBT	BAG5	0.59900969	1.03E-18
DBT	TTC5	0.59918543	9.97E-19
DBT	GEMIN5	0.59928527	9.81E-19
DBT	CDC42SE2	0.59943007	9.58E-19
DBT	CREBL2	0.59966339	9.21E-19
DBT	TLE4	0.60008048	8.59E-19
DBT	COMMD8	0.60010839	8.55E-19
DBT	IL1R1	0.60045139	8.08E-19
DBT	SLC25A32	0.60061799	7.86E-19
DBT	PDGFRA	0.60081731	7.6E-19
DBT	CPM	0.60096391	7.42E-19
DBT	UHMK1	0.60104992	7.31E-19

DBT	ARL6IP6	0.60110439	7.24E-19
DBT	SETD3	0.60112532	7.22E-19
DBT	TFDP1	0.60115447	7.18E-19
DBT	EIF2S1	0.60133385	6.97E-19
DBT	EXO5	0.60140183	6.89E-19
DBT	PIK3C3	0.60145833	6.83E-19
DBT	AHCTF1	0.60146638	6.82E-19
DBT	UBE2Q2	0.60195534	6.28E-19
DBT	MAPK6	0.6020491	6.18E-19
DBT	AP1AR	0.60207993	6.15E-19
DBT	CEP170	0.60217336	6.06E-19
DBT	FBXO42	0.60233198	5.9E-19
DBT	UBN1	0.60243841	5.79E-19
DBT	ELOVL5	0.60247899	5.75E-19
DBT	SPRED2	0.60251258	5.72E-19
DBT	NIP7	0.60264339	5.6E-19
DBT	AKIRIN1	0.60272371	5.52E-19
DBT	TBK1	0.60276553	5.48E-19
DBT	DTX3L	0.60301884	5.25E-19
DBT	RPGRIP1L	0.60304809	5.23E-19
DBT	TERF2	0.60314306	5.14E-19
DBT	JAK2	0.60318906	5.1E-19
DBT	CROT	0.60343928	4.89E-19
DBT	ELK3	0.6036153	4.75E-19
DBT	KRCC1	0.60369917	4.68E-19
DBT	CCDC90B	0.60384113	4.57E-19
DBT	IFNGR1	0.60411747	4.36E-19
DBT	SMARCA5	0.60416208	4.33E-19
DBT	KIF16B	0.60441827	4.15E-19
DBT	GAS7	0.60451272	4.08E-19
DBT	LRCH1	0.6046362	4E-19
DBT	ETF1	0.60477842	3.9E-19
DBT	ECM2	0.6049378	3.8E-19
DBT	SH3PXD2A	0.60497282	3.78E-19
DBT	MPHOSPH10	0.60509903	3.7E-19
DBT	STK38L	0.60511909	3.68E-19
DBT	CYP2U1	0.60533208	3.55E-19
DBT	KHDRBS1	0.60551674	3.44E-19
DBT	DUSP11	0.60553479	3.43E-19
DBT	PLEKHA1	0.60561409	3.39E-19
DBT	PPP2CB	0.60577918	3.29E-19
DBT	RNF4	0.60587365	3.24E-19
DBT	CNST	0.60606042	3.14E-19
DBT	TMEM267	0.60626374	3.03E-19
DBT	MYO5A	0.60644622	2.94E-19
DBT	DHX36	0.60644848	2.94E-19
DBT	ETS1	0.60671002	2.81E-19
DBT	FGD4	0.60672313	2.8E-19
DBT	GPR34	0.60676747	2.78E-19
DBT	KIAA1191	0.60700732	2.67E-19
DBT	CNN3	0.60732884	2.53E-19
DBT	NUP58	0.60734458	2.52E-19
DBT	ARMC8	0.6074244	2.49E-19
DBT	GDI2	0.60766708	2.39E-19
DBT	COL4A1	0.60770503	2.37E-19
DBT	TMTC2	0.60775766	2.35E-19
DBT	CREB3L2	0.60777399	2.34E-19
DBT	ARF3	0.60790245	2.29E-19

DBT	SUFU	0.60812818	2.2E-19
DBT	DIPK1A	0.60817769	2.19E-19
DBT	CNOT8	0.60837909	2.11E-19
DBT	ARL8B	0.60869427	2E-19
DBT	BCL2L13	0.6087888	1.97E-19
DBT	MFAP1	0.60914984	1.85E-19
DBT	VPS26A	0.60950445	1.74E-19
DBT	ZNF217	0.60957272	1.72E-19
DBT	ZMIZ1	0.6096213	1.71E-19
DBT	LSAMP	0.60998313	1.6E-19
DBT	ASAH1	0.61001161	1.59E-19
DBT	EPB41L4A	0.61029115	1.52E-19
DBT	CD93	0.61031778	1.51E-19
DBT	PRICKLE1	0.61044214	1.48E-19
DBT	ERLIN1	0.61062137	1.44E-19
DBT	CDK14	0.61067149	1.42E-19
DBT	EHF	0.61069482	1.42E-19
DBT	NFKB1	0.61073536	1.41E-19
DBT	GOLPH3	0.61104628	1.33E-19
DBT	GMEB1	0.61111365	1.32E-19
DBT	RBMS2	0.61120592	1.3E-19
DBT	TOB2	0.61127109	1.28E-19
DBT	FAM20B	0.6113884	1.26E-19
DBT	TCTN3	0.61140745	1.25E-19
DBT	ESYT2	0.61158409	1.21E-19
DBT	LAMC1	0.61178407	1.17E-19
DBT	SSX2IP	0.61193026	1.14E-19
DBT	RECK	0.61193664	1.14E-19
DBT	WIPF2	0.61195712	1.14E-19
DBT	ANP32E	0.61196208	1.14E-19
DBT	SMG8	0.61200129	1.13E-19
DBT	TOP1	0.61204021	1.12E-19
DBT	FKBP15	0.61204354	1.12E-19
DBT	ENTPD1	0.61230826	1.07E-19
DBT	PCSK5	0.61232575	1.07E-19
DBT	TM9SF2	0.61240779	1.05E-19
DBT	PLXNC1	0.6126628	1.01E-19
DBT	CRISPLD1	0.61268775	1E-19
DBT	INCENP	0.61274165	9.93E-20
DBT	REEP3	0.61277116	9.88E-20
DBT	SORT1	0.61280777	9.82E-20
DBT	ITSN1	0.61284403	9.76E-20
DBT	KLHL7	0.61287011	9.71E-20
DBT	VHL	0.61288595	9.69E-20
DBT	SPPL3	0.61299885	9.5E-20
DBT	FAF2	0.61300088	9.49E-20
DBT	SDC3	0.61303871	9.43E-20
DBT	NAB1	0.61362646	8.51E-20
DBT	RGL1	0.61364359	8.49E-20
DBT	BTN3A1	0.61381173	8.24E-20
DBT	CNIH1	0.61382503	8.22E-20
DBT	ANXA7	0.61399819	7.98E-20
DBT	SLIT2	0.6140935	7.84E-20
DBT	MYLK	0.61450936	7.29E-20
DBT	EID1	0.61454373	7.25E-20
DBT	STON1	0.61464933	7.12E-20
DBT	XYLT1	0.61472389	7.02E-20
DBT	PPP3R1	0.61482197	6.9E-20

DBT	TAOK3	0.61510253	6.57E-20
DBT	TSNAX	0.61511159	6.56E-20
DBT	IGF2R	0.6151791	6.48E-20
DBT	EIF1AX	0.61528462	6.36E-20
DBT	SKIL	0.61538767	6.25E-20
DBT	RFX5	0.61547782	6.15E-20
DBT	NIN	0.61558244	6.04E-20
DBT	EXOC2	0.61559153	6.03E-20
DBT	WDR43	0.61573277	5.88E-20
DBT	C1D	0.61574894	5.87E-20
DBT	RBMS1	0.61575756	5.86E-20
DBT	TM2D1	0.61602133	5.59E-20
DBT	CHUK	0.61606514	5.55E-20
DBT	GFPT1	0.61627757	5.34E-20
DBT	WBP11	0.61629124	5.33E-20
DBT	PARVA	0.61639425	5.23E-20
DBT	HGF	0.6164877	5.15E-20
DBT	DCK	0.61663097	5.02E-20
DBT	WDR26	0.6168145	4.86E-20
DBT	ELK4	0.61701674	4.69E-20
DBT	PLXDC2	0.61703443	4.67E-20
DBT	BNIP3L	0.61715252	4.58E-20
DBT	TAF12	0.61765566	4.19E-20
DBT	PTBP3	0.61768731	4.16E-20
DBT	ZNF134	0.61816248	3.83E-20
DBT	CDC73	0.61827534	3.75E-20
DBT	USP10	0.61830639	3.73E-20
DBT	HMCN1	0.61832756	3.72E-20
DBT	CAPZA1	0.61848344	3.61E-20
DBT	CIPC	0.61860902	3.53E-20
DBT	F13A1	0.61873027	3.46E-20
DBT	COPS8	0.61886751	3.38E-20
DBT	TMEM170A	0.61890047	3.36E-20
DBT	C16orf87	0.61895787	3.32E-20
DBT	TTL	0.61914575	3.21E-20
DBT	CCDC47	0.61949512	3.02E-20
DBT	IRF2	0.61954429	2.99E-20
DBT	RSU1	0.619658	2.93E-20
DBT	YES1	0.62035275	2.59E-20
DBT	DSE	0.620558	2.5E-20
DBT	SMIM13	0.62056339	2.49E-20
DBT	CALCOCO2	0.62057104	2.49E-20
DBT	TGFBR1	0.62067859	2.44E-20
DBT	SLFN11	0.62072991	2.42E-20
DBT	IFT57	0.62088918	2.35E-20
DBT	TAF13	0.62095571	2.32E-20
DBT	STAT1	0.62100497	2.3E-20
DBT	KIF3B	0.62112456	2.25E-20
DBT	SLMAP	0.62119405	2.23E-20
DBT	SORL1	0.62120741	2.22E-20
DBT	PCNX1	0.6212873	2.19E-20
DBT	IDH3A	0.62133991	2.17E-20
DBT	SNX7	0.62135426	2.16E-20
DBT	PEX19	0.62154844	2.09E-20
DBT	NCK1	0.62162026	2.06E-20
DBT	EMC1	0.62169602	2.03E-20
DBT	CAB39	0.62173358	2.02E-20
DBT	LMBR1	0.62176498	2.01E-20

DBT	CALCRL	0.62200421	1.92E-20
DBT	PDP1	0.62202763	1.92E-20
DBT	PHTF1	0.62203037	1.92E-20
DBT	RAD1	0.62232146	1.82E-20
DBT	PTP4A1	0.62273444	1.69E-20
DBT	PRKG1	0.62278699	1.67E-20
DBT	PGM2	0.62291746	1.63E-20
DBT	CDYL	0.62295674	1.62E-20
DBT	FAM120A	0.62301558	1.6E-20
DBT	MFSD14B	0.62318	1.56E-20
DBT	PRSS23	0.62342397	1.49E-20
DBT	YTHDF2	0.62370539	1.42E-20
DBT	AKT3	0.62372761	1.41E-20
DBT	TRAM2	0.62378706	1.39E-20
DBT	ARID5B	0.62381504	1.39E-20
DBT	LIMS1	0.62382905	1.38E-20
DBT	NT5DC1	0.6240215	1.34E-20
DBT	TMEM106B	0.62403529	1.33E-20
DBT	FGFR1OP2	0.62408088	1.32E-20
DBT	ATP2A2	0.62417475	1.3E-20
DBT	C9orf64	0.62419955	1.29E-20
DBT	TMEM127	0.624543	1.22E-20
DBT	ATP5PB	0.6247894	1.16E-20
DBT	ATP2C1	0.62486732	1.15E-20
DBT	GORASP2	0.62531594	1.06E-20
DBT	SCP2	0.62532108	1.06E-20
DBT	KIAA0232	0.62538896	1.04E-20
DBT	TTC26	0.62542322	1.04E-20
DBT	PECAM1	0.62558128	1.01E-20
DBT	KDM1B	0.62572567	9.8E-21
DBT	TENM3	0.62584986	9.58E-21
DBT	SPOP	0.6259714	9.37E-21
DBT	PAQR3	0.62651212	8.49E-21
DBT	HAT1	0.62660269	8.35E-21
DBT	ZNF720	0.62669379	8.21E-21
DBT	KCTD10	0.62710139	7.62E-21
DBT	ANKS1A	0.6271987	7.49E-21
DBT	C5orf15	0.62752821	7.05E-21
DBT	TCEA1	0.62800412	6.46E-21
DBT	GINM1	0.62801297	6.45E-21
DBT	KRR1	0.62804512	6.41E-21
DBT	RAD18	0.62810255	6.34E-21
DBT	CRLF3	0.62813839	6.3E-21
DBT	WASL	0.62825678	6.16E-21
DBT	CNOT4	0.6283368	6.07E-21
DBT	GALC	0.62836089	6.05E-21
DBT	DCAF7	0.62846468	5.93E-21
DBT	STK3	0.62870509	5.67E-21
DBT	ADAR	0.62876875	5.61E-21
DBT	FGL2	0.62890342	5.47E-21
DBT	RNF138	0.62893087	5.44E-21
DBT	PIP4K2A	0.62899248	5.38E-21
DBT	GALNT1	0.62902136	5.35E-21
DBT	FBXL17	0.62902551	5.35E-21
DBT	SEC22A	0.62912995	5.25E-21
DBT	KAT7	0.62936388	5.02E-21
DBT	SLFN5	0.62942255	4.97E-21
DBT	SLC44A1	0.62960037	4.81E-21

DBT	FBXO28	0.6297067	4.71E-21
DBT	CEP41	0.62984326	4.6E-21
DBT	HIVEP1	0.62998112	4.48E-21
DBT	NSRP1	0.62998574	4.48E-21
DBT	KIRREL1	0.63000119	4.46E-21
DBT	CSTF2T	0.63010615	4.38E-21
DBT	CSNK1A1	0.63016233	4.33E-21
DBT	SYNJ2BP	0.63028955	4.23E-21
DBT	ARHGAP21	0.63030027	4.22E-21
DBT	PDHX	0.6304352	4.12E-21
DBT	CAMSAP1	0.63044859	4.11E-21
DBT	ARL6IP5	0.63045063	4.11E-21
DBT	MED14	0.6304587	4.1E-21
DBT	TPP1	0.63052701	4.05E-21
DBT	DHDDS	0.63057746	4.01E-21
DBT	RDH14	0.6305793	4.01E-21
DBT	KIT	0.63065328	3.96E-21
DBT	KITLG	0.63066747	3.95E-21
DBT	NUP50	0.63075243	3.88E-21
DBT	KCTD21	0.63110741	3.64E-21
DBT	SMARCE1	0.63119863	3.57E-21
DBT	RCBTB2	0.63122077	3.56E-21
DBT	SSPN	0.63141363	3.43E-21
DBT	PTPRB	0.63146428	3.4E-21
DBT	AP1S2	0.63161148	3.31E-21
DBT	NUDCD1	0.63174768	3.23E-21
DBT	MDFIC	0.6319883	3.08E-21
DBT	GFM1	0.63218456	2.97E-21
DBT	MIS18BP1	0.632239	2.94E-21
DBT	AHNAK	0.63224266	2.94E-21
DBT	SDCBP	0.63253077	2.79E-21
DBT	ROBO1	0.63258973	2.76E-21
DBT	RAB2A	0.63263379	2.73E-21
DBT	RPE	0.6327697	2.67E-21
DBT	MBNL3	0.6329771	2.56E-21
DBT	KCTD9	0.63302398	2.54E-21
DBT	MAPK1IP1L	0.63314813	2.48E-21
DBT	TMCC1	0.63365737	2.26E-21
DBT	SNRNP27	0.63399876	2.12E-21
DBT	ZBTB2	0.63422067	2.03E-21
DBT	DNAJC14	0.63436346	1.98E-21
DBT	KBTBD4	0.63454536	1.91E-21
DBT	SPDL1	0.63476341	1.83E-21
DBT	NLN	0.63480389	1.82E-21
DBT	VRK2	0.63481597	1.81E-21
DBT	LCORL	0.63490819	1.78E-21
DBT	METAP1	0.63499517	1.75E-21
DBT	ZC2HC1A	0.63510736	1.72E-21
DBT	BRD3	0.63516459	1.7E-21
DBT	DCAF12	0.63519403	1.69E-21
DBT	ABRAXAS2	0.63523867	1.67E-21
DBT	EPHA3	0.63529552	1.66E-21
DBT	ATP11C	0.63566076	1.55E-21
DBT	OTUD7B	0.63574232	1.52E-21
DBT	CALD1	0.63585447	1.49E-21
DBT	CDC23	0.63590617	1.48E-21
DBT	DOCK1	0.63594949	1.46E-21
DBT	BLOC1S5	0.63612709	1.42E-21

DBT	AHR	0.63621708	1.39E-21
DBT	ACBD5	0.63623143	1.39E-21
DBT	ZNF532	0.63625276	1.38E-21
DBT	CAPRIN1	0.63626971	1.38E-21
DBT	SGCB	0.63635893	1.35E-21
DBT	MAT2B	0.63645562	1.33E-21
DBT	LRRC8C	0.63653087	1.31E-21
DBT	CRTC3	0.63664262	1.28E-21
DBT	CDK17	0.63728064	1.14E-21
DBT	GPR107	0.63731695	1.13E-21
DBT	WNK1	0.63748327	1.09E-21
DBT	CNOT7	0.6376892	1.05E-21
DBT	OSTM1	0.63797487	9.96E-22
DBT	KDM5A	0.63835256	9.27E-22
DBT	ACVR1	0.63842718	9.14E-22
DBT	METTL9	0.63846666	9.07E-22
DBT	DIP2C	0.63849279	9.02E-22
DBT	BTBD9	0.63867388	8.72E-22
DBT	NECTIN3	0.63869638	8.68E-22
DBT	RSRC1	0.638702	8.67E-22
DBT	AIDA	0.63873731	8.61E-22
DBT	TGS1	0.63892429	8.31E-22
DBT	ADGRL2	0.63934873	7.66E-22
DBT	PRDX3	0.63948657	7.46E-22
DBT	ADGRF5	0.63955475	7.36E-22
DBT	ERAP1	0.63980946	7.01E-22
DBT	SLC4A7	0.64000765	6.75E-22
DBT	PTPRA	0.64007075	6.67E-22
DBT	GBE1	0.64034726	6.32E-22
DBT	LYPLA1	0.64046723	6.18E-22
DBT	TLR4	0.64067627	5.93E-22
DBT	ABCC4	0.64072047	5.88E-22
DBT	KLF12	0.64096887	5.61E-22
DBT	PDCD6IP	0.64103949	5.53E-22
DBT	CDKL1	0.64105644	5.51E-22
DBT	GUCY1A1	0.64107392	5.49E-22
DBT	DNAJC21	0.64112856	5.44E-22
DBT	ATP11B	0.64130538	5.25E-22
DBT	RAB14	0.64133832	5.22E-22
DBT	LRP12	0.64144108	5.12E-22
DBT	SESTD1	0.64189358	4.69E-22
DBT	UBQLN1	0.64206525	4.54E-22
DBT	DESI2	0.64213756	4.47E-22
DBT	ZFP90	0.64218433	4.43E-22
DBT	ITGB1	0.64227921	4.35E-22
DBT	NR1D2	0.6422948	4.34E-22
DBT	CCNG2	0.64238761	4.26E-22
DBT	RNF168	0.64240872	4.24E-22
DBT	CSNK2A1	0.64256452	4.12E-22
DBT	UHRF1BP1L	0.64275096	3.97E-22
DBT	ZNF227	0.64297463	3.8E-22
DBT	VANGL1	0.64299685	3.79E-22
DBT	PLEKHB2	0.64303421	3.76E-22
DBT	RNF141	0.6433726	3.52E-22
DBT	ATP2B1	0.64337621	3.52E-22
DBT	TMEM135	0.64342694	3.48E-22
DBT	AEBP2	0.6434494	3.47E-22
DBT	UBA2	0.64376386	3.26E-22

DBT	DYNC1I2	0.64395985	3.14E-22
DBT	HSPA13	0.64396285	3.14E-22
DBT	GANAB	0.64422592	2.98E-22
DBT	ACBD3	0.64428579	2.94E-22
DBT	GNB4	0.64431354	2.93E-22
DBT	DRAM2	0.64431728	2.93E-22
DBT	DLD	0.64460715	2.77E-22
DBT	SCAF11	0.64471288	2.71E-22
DBT	GNB1	0.64504139	2.54E-22
DBT	CPSF6	0.64551094	2.32E-22
DBT	COPA	0.64552026	2.31E-22
DBT	HIF1A	0.64553213	2.31E-22
DBT	TLR3	0.64555232	2.3E-22
DBT	MAP3K7	0.64591046	2.14E-22
DBT	MOSPD1	0.6460184	2.1E-22
DBT	LTBP1	0.64604903	2.08E-22
DBT	TARDBP	0.64625291	2E-22
DBT	PRKAR1A	0.64656278	1.88E-22
DBT	ZEB2	0.64656607	1.88E-22
DBT	USP14	0.64663156	1.86E-22
DBT	FZD6	0.64722214	1.65E-22
DBT	RRAGC	0.6473058	1.63E-22
DBT	SLC35A3	0.64741826	1.59E-22
DBT	GNPDA2	0.64750921	1.56E-22
DBT	ZNF766	0.64752153	1.56E-22
DBT	SUSD6	0.64756601	1.55E-22
DBT	ZC3H15	0.64777346	1.48E-22
DBT	NFE2L2	0.64789428	1.45E-22
DBT	ICMT	0.64850275	1.28E-22
DBT	SLC8A1	0.64850404	1.28E-22
DBT	SZRD1	0.64851295	1.28E-22
DBT	SHOC2	0.64859059	1.26E-22
DBT	FYCO1	0.64870559	1.23E-22
DBT	BAG4	0.64901113	1.16E-22
DBT	F2R	0.64912745	1.13E-22
DBT	RAB3GAP2	0.64924702	1.11E-22
DBT	YAP1	0.64955429	1.04E-22
DBT	UVRAG	0.64961886	1.03E-22
DBT	FAM91A1	0.64964298	1.02E-22
DBT	UGDH	0.64990097	9.72E-23
DBT	PPP3CA	0.6499426	9.64E-23
DBT	LAPTM4A	0.65008903	9.36E-23
DBT	HOMER1	0.65010897	9.32E-23
DBT	MID1	0.65017499	9.2E-23
DBT	NID1	0.65030085	8.97E-23
DBT	BCL9L	0.65071024	8.27E-23
DBT	NDC1	0.65073253	8.23E-23
DBT	YWHAQ	0.65080741	8.11E-23
DBT	RAD21	0.65080782	8.11E-23
DBT	RPL7L1	0.65086098	8.02E-23
DBT	PMS2	0.65094815	7.88E-23
DBT	RAB23	0.65127687	7.38E-23
DBT	MAP3K20	0.65133571	7.29E-23
DBT	TSHZ1	0.65135545	7.26E-23
DBT	GAB2	0.65147068	7.1E-23
DBT	LMBRD1	0.65193625	6.46E-23
DBT	PARG	0.65228955	6.02E-23
DBT	SNX2	0.65253377	5.73E-23

DBT	KLF7	0.6526086	5.65E-23
DBT	PPT1	0.65265651	5.59E-23
DBT	UTP11	0.65286657	5.36E-23
DBT	ATL3	0.65301682	5.2E-23
DBT	PNPLA8	0.65307841	5.14E-23
DBT	ATG5	0.65316397	5.05E-23
DBT	LARS2	0.65352357	4.69E-23
DBT	LEPR	0.65368312	4.54E-23
DBT	EPB41L2	0.65418405	4.11E-23
DBT	VAMP3	0.6543689	3.95E-23
DBT	UBFD1	0.6544738	3.87E-23
DBT	BTN3A3	0.65453736	3.82E-23
DBT	CAMK2D	0.65460397	3.77E-23
DBT	SERINC3	0.65466589	3.72E-23
DBT	PLAA	0.65502797	3.46E-23
DBT	CPT1A	0.65527304	3.29E-23
DBT	ITPRIPL2	0.65531982	3.26E-23
DBT	DENND6A	0.65552842	3.12E-23
DBT	L3MBTL3	0.65556017	3.1E-23
DBT	ST13	0.65559947	3.08E-23
DBT	LARP7	0.65571757	3.01E-23
DBT	ITGA1	0.65580639	2.95E-23
DBT	FSTL1	0.65595191	2.87E-23
DBT	SPRTN	0.65602279	2.82E-23
DBT	SP1	0.65609834	2.78E-23
DBT	MEF2C	0.65612313	2.77E-23
DBT	ARHGAP31	0.65637578	2.63E-23
DBT	GOLT1B	0.65639682	2.62E-23
DBT	UGP2	0.65644691	2.59E-23
DBT	KANK2	0.6565542	2.53E-23
DBT	TSN	0.65704861	2.29E-23
DBT	BMP2K	0.65706329	2.28E-23
DBT	FNIP2	0.65715293	2.24E-23
DBT	NAALADL2	0.65719592	2.22E-23
DBT	MORF4L1	0.65724409	2.2E-23
DBT	ICE2	0.65759633	2.05E-23
DBT	NDFIP2	0.65763828	2.03E-23
DBT	NEK7	0.65765024	2.02E-23
DBT	RRN3	0.65815807	1.82E-23
DBT	SNAP23	0.65819076	1.81E-23
DBT	NUMB	0.65826216	1.78E-23
DBT	PTEN	0.65863527	1.65E-23
DBT	SCAMP1	0.65904907	1.52E-23
DBT	YIPF6	0.65930071	1.44E-23
DBT	MED20	0.65945085	1.4E-23
DBT	B3GNT2	0.65954644	1.37E-23
DBT	MEGF9	0.65957976	1.36E-23
DBT	MOB1A	0.65984976	1.29E-23
DBT	RTL6	0.65996673	1.25E-23
DBT	MRPL19	0.66015239	1.21E-23
DBT	SPRED1	0.66015701	1.21E-23
DBT	MFN2	0.66036727	1.15E-23
DBT	SERINC5	0.66049725	1.12E-23
DBT	TBL1XR1	0.66053897	1.11E-23
DBT	UBE2D3	0.66076976	1.06E-23
DBT	PTDSS1	0.66081829	1.05E-23
DBT	ZNF281	0.66114217	9.83E-24
DBT	ZNF827	0.66126147	9.59E-24

DBT	UBQLN2	0.66140787	9.3E-24
DBT	KIAA1143	0.66145537	9.21E-24
DBT	RNF20	0.66146153	9.2E-24
DBT	SLC30A1	0.66153981	9.05E-24
DBT	HFE	0.66169422	8.76E-24
DBT	RAB21	0.66182613	8.52E-24
DBT	DNAJA2	0.66188164	8.42E-24
DBT	RAB5A	0.66207922	8.08E-24
DBT	CDC42EP3	0.66218249	7.91E-24
DBT	ZNF623	0.66235553	7.63E-24
DBT	ITGA4	0.66249469	7.41E-24
DBT	SLC26A2	0.66261087	7.23E-24
DBT	BCAS2	0.66282383	6.92E-24
DBT	ACTR3	0.6631397	6.47E-24
DBT	FBXO45	0.66322515	6.36E-24
DBT	SMURF2	0.6634192	6.11E-24
DBT	NBR1	0.6634312	6.09E-24
DBT	DDX21	0.66371307	5.74E-24
DBT	NXT2	0.66392323	5.49E-24
DBT	BTF3L4	0.66413706	5.25E-24
DBT	SEMA3C	0.66428421	5.09E-24
DBT	RYBP	0.66445893	4.91E-24
DBT	PHACTR4	0.66467063	4.69E-24
DBT	SH3D19	0.66530164	4.11E-24
DBT	CDK12	0.66544695	3.98E-24
DBT	NEDD4	0.66556433	3.88E-24
DBT	PTPN9	0.665586	3.87E-24
DBT	LNPEP	0.66563153	3.83E-24
DBT	UBA6	0.66568914	3.78E-24
DBT	STAT3	0.66581269	3.68E-24
DBT	GTF2E1	0.66612749	3.45E-24
DBT	GOLGA7	0.66618317	3.41E-24
DBT	HIP1	0.66620361	3.39E-24
DBT	DDR2	0.6662656	3.35E-24
DBT	SH3GLB1	0.66627348	3.34E-24
DBT	PTPRK	0.66664269	3.09E-24
DBT	ZNF644	0.66671148	3.04E-24
DBT	MSL2	0.66676107	3.01E-24
DBT	SWAP70	0.66682903	2.97E-24
DBT	SLC12A6	0.66699499	2.87E-24
DBT	ERBIN	0.66734467	2.66E-24
DBT	ZNF184	0.66768942	2.47E-24
DBT	BACH1	0.66769647	2.47E-24
DBT	MTM1	0.66770772	2.46E-24
DBT	SET	0.66775467	2.44E-24
DBT	GNA13	0.66798055	2.32E-24
DBT	NOTCH2	0.66818105	2.23E-24
DBT	EXT2	0.66825307	2.19E-24
DBT	RNF2	0.66852828	2.07E-24
DBT	NSL1	0.66871132	1.99E-24
DBT	BZW1	0.66873213	1.98E-24
DBT	HNRNPR	0.66874332	1.97E-24
DBT	UBE3C	0.66883329	1.94E-24
DBT	PPP1R2	0.66912039	1.82E-24
DBT	MTPN	0.66917383	1.8E-24
DBT	SMNDC1	0.66925616	1.77E-24
DBT	HINT3	0.6693835	1.72E-24
DBT	SMC6	0.66942124	1.71E-24

DBT	ACVR2A	0.66949181	1.68E-24
DBT	ZNF468	0.66951233	1.67E-24
DBT	ANO6	0.6699586	1.52E-24
DBT	PEX2	0.67011782	1.47E-24
DBT	PRPF40A	0.67022782	1.43E-24
DBT	BCAP29	0.67031319	1.41E-24
DBT	NEO1	0.67040481	1.38E-24
DBT	EIF2AK2	0.67046884	1.36E-24
DBT	SOAT1	0.67051251	1.35E-24
DBT	NPTN	0.67056566	1.33E-24
DBT	RNF41	0.67078339	1.27E-24
DBT	XPR1	0.6714181	1.11E-24
DBT	CANX	0.67145747	1.1E-24
DBT	SDE2	0.67171683	1.04E-24
DBT	LYRM2	0.67211663	9.53E-25
DBT	TXNRD1	0.67216518	9.43E-25
DBT	CYBRD1	0.67221043	9.34E-25
DBT	SGMS2	0.67229035	9.17E-25
DBT	TRIM32	0.67286826	8.09E-25
DBT	DNAJC10	0.67287455	8.08E-25
DBT	RNF111	0.67294321	7.96E-25
DBT	MPP5	0.67298217	7.89E-25
DBT	ASB7	0.67311017	7.67E-25
DBT	OSGIN2	0.673129	7.64E-25
DBT	PPFIA1	0.67326896	7.41E-25
DBT	RNF14	0.6734279	7.16E-25
DBT	MAGT1	0.67345756	7.11E-25
DBT	PANK3	0.67374988	6.67E-25
DBT	UBA3	0.67375927	6.66E-25
DBT	TRAF3IP1	0.67396751	6.36E-25
DBT	TMEM19	0.67448743	5.68E-25
DBT	MITF	0.67451987	5.64E-25
DBT	TRIP12	0.67457326	5.57E-25
DBT	HACD2	0.67458568	5.56E-25
DBT	SEL1L	0.67460163	5.54E-25
DBT	ABI2	0.67468979	5.43E-25
DBT	WASF2	0.67493525	5.15E-25
DBT	GASK1B	0.67499804	5.08E-25
DBT	NR2F2	0.67523895	4.82E-25
DBT	XRN2	0.67526232	4.79E-25
DBT	STAM	0.67530982	4.74E-25
DBT	POFUT1	0.67534822	4.7E-25
DBT	PPIL4	0.67563721	4.41E-25
DBT	RNF6	0.67608922	3.99E-25
DBT	SMARCC1	0.67636059	3.76E-25
DBT	ABHD13	0.67636318	3.76E-25
DBT	EXOC6B	0.67641125	3.72E-25
DBT	RANBP9	0.67646236	3.68E-25
DBT	SUZ12	0.67656327	3.6E-25
DBT	DCAF1	0.67681056	3.4E-25
DBT	MCMBP	0.67696563	3.29E-25
DBT	FCHSD2	0.6770241	3.25E-25
DBT	GPD2	0.67740371	2.99E-25
DBT	CBFB	0.677514	2.91E-25
DBT	ZMAT3	0.67753817	2.9E-25
DBT	GNAQ	0.67753982	2.9E-25
DBT	KLHL2	0.67754382	2.89E-25
DBT	FBXO8	0.67758168	2.87E-25

DBT	SERBP1	0.6776381	2.83E-25
DBT	FBXO38	0.67772054	2.78E-25
DBT	ATE1	0.67790964	2.67E-25
DBT	SERINC1	0.67837206	2.41E-25
DBT	SAP130	0.6784328	2.38E-25
DBT	LPP	0.67860563	2.29E-25
DBT	CLCN3	0.67872371	2.23E-25
DBT	TRMT1L	0.67892066	2.13E-25
DBT	PCYT1A	0.67893427	2.12E-25
DBT	SLC30A9	0.67916976	2.02E-25
DBT	LDAH	0.67927657	1.97E-25
DBT	HNRNPLL	0.67936081	1.93E-25
DBT	NCOA3	0.67966306	1.8E-25
DBT	BRCC3	0.6796823	1.8E-25
DBT	CDK19	0.67974326	1.77E-25
DBT	UBTD2	0.67978023	1.76E-25
DBT	NDUFS1	0.68013353	1.62E-25
DBT	CAMSAP2	0.68021472	1.6E-25
DBT	VPS35	0.68068332	1.44E-25
DBT	TRAM1	0.6808503	1.38E-25
DBT	NFIX	0.68103418	1.33E-25
DBT	LACC1	0.68170189	1.14E-25
DBT	TMEM87B	0.68178669	1.12E-25
DBT	PALLD	0.68182761	1.11E-25
DBT	GSTCD	0.68222531	1.01E-25
DBT	ADH5	0.68236498	9.83E-26
DBT	RBM18	0.68251951	9.49E-26
DBT	CLIP1	0.68276703	8.97E-26
DBT	ACSL4	0.6828529	8.8E-26
DBT	MSH2	0.6829185	8.67E-26
DBT	BMT2	0.68319434	8.14E-26
DBT	DDX19A	0.68351802	7.57E-26
DBT	ADNP	0.68351923	7.57E-26
DBT	KIF5B	0.68363286	7.37E-26
DBT	NCKAP1	0.68413313	6.58E-26
DBT	HAUS2	0.68436729	6.24E-26
DBT	SRGAP2C	0.68466513	5.83E-26
DBT	GOSR1	0.68470277	5.78E-26
DBT	RPRD2	0.68471437	5.76E-26
DBT	LNPK	0.68526574	5.08E-26
DBT	TWSG1	0.68529552	5.05E-26
DBT	CTCF	0.68544288	4.88E-26
DBT	SLC25A40	0.6854622	4.86E-26
DBT	FNDC3B	0.68547429	4.84E-26
DBT	DOCK9	0.6858371	4.46E-26
DBT	TRANK1	0.68594989	4.34E-26
DBT	TGFBR2	0.68617177	4.13E-26
DBT	IMPA1	0.68630016	4.01E-26
DBT	SOCS6	0.68649996	3.83E-26
DBT	MEX3C	0.68672211	3.64E-26
DBT	SDHD	0.68709892	3.33E-26
DBT	SUCLA2	0.68712085	3.32E-26
DBT	RAD23B	0.68719164	3.26E-26
DBT	SNX4	0.68761002	2.96E-26
DBT	ASAP1	0.68792197	2.76E-26
DBT	DCLRE1B	0.68801321	2.7E-26
DBT	GLG1	0.68821633	2.58E-26
DBT	CYB5R4	0.68837791	2.48E-26

DBT	SEC23A	0.68843965	2.45E-26
DBT	BAZ1B	0.68862142	2.35E-26
DBT	TGOLN2	0.68864356	2.33E-26
DBT	GTF2H3	0.68925523	2.02E-26
DBT	CDC42	0.68937337	1.97E-26
DBT	GMCL1	0.68938877	1.96E-26
DBT	HPS5	0.68957738	1.88E-26
DBT	SCARB2	0.68959279	1.87E-26
DBT	NRP1	0.68966904	1.84E-26
DBT	TRPS1	0.68981216	1.78E-26
DBT	PRKD3	0.68986349	1.76E-26
DBT	RHBDD1	0.69023956	1.61E-26
DBT	PPP1CC	0.69034021	1.57E-26
DBT	SOCS4	0.69047049	1.52E-26
DBT	FAM98B	0.69049945	1.51E-26
DBT	ABI1	0.69075378	1.43E-26
DBT	PIK3CG	0.69121943	1.28E-26
DBT	PCDH18	0.69130611	1.25E-26
DBT	CWC22	0.69163747	1.16E-26
DBT	TOPORS	0.6916755	1.15E-26
DBT	TMED7	0.69171333	1.14E-26
DBT	MAPK14	0.6918076	1.11E-26
DBT	TCF4	0.69222257	1.01E-26
DBT	ARMT1	0.69247847	9.52E-27
DBT	NAA30	0.69294006	8.54E-27
DBT	DDX18	0.69315889	8.11E-27
DBT	SPTLC1	0.69354983	7.39E-27
DBT	KATNAL1	0.69371141	7.12E-27
DBT	RFWD3	0.69393015	6.76E-27
DBT	CERS6	0.69432445	6.15E-27
DBT	SLC9A6	0.6947341	5.58E-27
DBT	R3HDM1	0.69497226	5.27E-27
DBT	FRS2	0.69498391	5.26E-27
DBT	STRN3	0.69512985	5.08E-27
DBT	MBTPS2	0.69518674	5.01E-27
DBT	RAB10	0.69534359	4.83E-27
DBT	SLC25A46	0.69545598	4.7E-27
DBT	HNRNPH2	0.69575766	4.37E-27
DBT	NEK1	0.69580387	4.33E-27
DBT	NR3C1	0.6959328	4.19E-27
DBT	MEF2A	0.69612981	4E-27
DBT	PPM1A	0.6961485	3.98E-27
DBT	KCTD20	0.6965547	3.62E-27
DBT	VPS4B	0.69661068	3.57E-27
DBT	ATP6V1C1	0.69663243	3.55E-27
DBT	SIN3A	0.69665205	3.53E-27
DBT	CLOCK	0.69666348	3.52E-27
DBT	PPM1B	0.69708719	3.18E-27
DBT	HNRNPK	0.69716416	3.12E-27
DBT	SSR1	0.69722809	3.08E-27
DBT	KDSR	0.69747321	2.9E-27
DBT	RELL1	0.69771226	2.74E-27
DBT	WDR89	0.69799855	2.56E-27
DBT	RLIM	0.6983812	2.33E-27
DBT	SRPK2	0.69840831	2.31E-27
DBT	C11orf58	0.69855026	2.24E-27
DBT	TCAF1	0.69872156	2.15E-27
DBT	AGPS	0.69873402	2.14E-27

DBT	TMOD3	0.69878322	2.11E-27
DBT	TAF1B	0.69913913	1.94E-27
DBT	SELENOT	0.69943498	1.81E-27
DBT	QTRT2	0.69962416	1.72E-27
DBT	LAMP2	0.69970975	1.69E-27
DBT	ZNF562	0.7003316	1.45E-27
DBT	PDE12	0.70111843	1.2E-27
DBT	NAA50	0.70116776	1.18E-27
DBT	LZIC	0.70118981	1.18E-27
DBT	LSM14A	0.70139331	1.12E-27
DBT	ZNF639	0.70147281	1.1E-27
DBT	ITGAV	0.70188613	9.94E-28
DBT	PPP2CA	0.70196298	9.75E-28
DBT	TAF2	0.70202687	9.6E-28
DBT	SMAD5	0.70264837	8.24E-28
DBT	RBBP9	0.70272247	8.1E-28
DBT	MMGT1	0.70277756	7.99E-28
DBT	LRRC8D	0.70284382	7.86E-28
DBT	DLG1	0.70288952	7.77E-28
DBT	SELENOF	0.70292988	7.69E-28
DBT	PDS5A	0.70305804	7.45E-28
DBT	RC3H2	0.70309776	7.38E-28
DBT	G3BP2	0.70359352	6.53E-28
DBT	CKAP5	0.70362673	6.48E-28
DBT	RRM2B	0.70378168	6.24E-28
DBT	FBXL5	0.70385861	6.12E-28
DBT	GNPTAB	0.70424502	5.56E-28
DBT	GMFB	0.70437834	5.38E-28
DBT	PDZD8	0.70443266	5.31E-28
DBT	MTMR2	0.70478519	4.87E-28
DBT	CDC27	0.70478792	4.86E-28
DBT	APOL6	0.70479554	4.85E-28
DBT	TOX4	0.70503808	4.57E-28
DBT	CPED1	0.70524002	4.35E-28
DBT	SH3BGRL	0.70526298	4.32E-28
DBT	CLIC4	0.70526838	4.32E-28
DBT	DYRK1A	0.70531194	4.27E-28
DBT	EIF4E	0.70555062	4.02E-28
DBT	KPNA4	0.70567864	3.9E-28
DBT	HIVEP2	0.70590428	3.68E-28
DBT	FNBP1L	0.70599569	3.6E-28
DBT	ZNF322	0.70605376	3.55E-28
DBT	PARD3B	0.70607031	3.54E-28
DBT	RASSF8	0.70625019	3.38E-28
DBT	CTSO	0.70632399	3.32E-28
DBT	APBB2	0.70645573	3.21E-28
DBT	ATXN1	0.70657329	3.12E-28
DBT	CNOT6L	0.7071491	2.7E-28
DBT	ZMPSTE24	0.70737897	2.55E-28
DBT	ARPP19	0.70738737	2.54E-28
DBT	ARHGAP42	0.70762513	2.4E-28
DBT	FBXW2	0.70780803	2.29E-28
DBT	MAML2	0.70789592	2.24E-28
DBT	SLC25A24	0.70843125	1.96E-28
DBT	PDLIM5	0.70845887	1.94E-28
DBT	SMC2	0.70846176	1.94E-28
DBT	CRK	0.70859364	1.88E-28
DBT	NUFIP2	0.70874295	1.81E-28

DBT	SEC23IP	0.70879792	1.78E-28
DBT	MED1	0.70905682	1.67E-28
DBT	RAB8B	0.70919005	1.62E-28
DBT	CYLD	0.70922129	1.6E-28
DBT	LIN54	0.70932671	1.56E-28
DBT	C16orf72	0.70951461	1.49E-28
DBT	TRIQK	0.70956471	1.47E-28
DBT	VPS41	0.70973382	1.41E-28
DBT	ADAM17	0.70988996	1.35E-28
DBT	CSGALNACT2	0.71004162	1.3E-28
DBT	DAAM1	0.71011089	1.28E-28
DBT	LRRCC1	0.71041993	1.18E-28
DBT	KLHL20	0.71064882	1.12E-28
DBT	HBP1	0.71067741	1.11E-28
DBT	CTR9	0.71093269	1.04E-28
DBT	VEZF1	0.71107342	1E-28
DBT	C3orf38	0.71131574	9.43E-29
DBT	NUDT4B	0.7119948	7.93E-29
DBT	CRIM1	0.71295094	6.21E-29
DBT	CUL4B	0.71304366	6.06E-29
DBT	RAP2A	0.71352812	5.35E-29
DBT	BLOC1S6	0.71386274	4.91E-29
DBT	EIF4G2	0.71395624	4.79E-29
DBT	ELF1	0.71395955	4.79E-29
DBT	CCNY	0.71399731	4.74E-29
DBT	ZNF146	0.71435503	4.32E-29
DBT	RBBP5	0.71438589	4.29E-29
DBT	MFSD1	0.71457085	4.09E-29
DBT	REST	0.71460546	4.05E-29
DBT	WWP1	0.71472426	3.93E-29
DBT	PIK3R1	0.71500069	3.66E-29
DBT	IFNAR1	0.7151046	3.56E-29
DBT	CWF19L2	0.71566649	3.08E-29
DBT	TCF12	0.71665216	2.38E-29
DBT	MBNL1	0.71743248	1.94E-29
DBT	PEAK1	0.71768436	1.82E-29
DBT	PTPN11	0.71780951	1.76E-29
DBT	PICALM	0.71810313	1.63E-29
DBT	ATP13A3	0.71810548	1.63E-29
DBT	MINDY2	0.71827425	1.56E-29
DBT	DIS3	0.71836555	1.52E-29
DBT	TERF1	0.7184477	1.49E-29
DBT	NAA15	0.71865216	1.41E-29
DBT	KDM4A	0.71900427	1.29E-29
DBT	ZNF260	0.71943292	1.15E-29
DBT	DEK	0.71984177	1.03E-29
DBT	GCC2	0.71989283	1.02E-29
DBT	GPATCH11	0.72030305	9.13E-30
DBT	ARHGAP5	0.72035451	9.01E-30
DBT	UTP14C	0.72046192	8.76E-30
DBT	VTA1	0.72063601	8.36E-30
DBT	DIPK2A	0.72104452	7.5E-30
DBT	WDFY1	0.72142664	6.78E-30
DBT	STX12	0.72144111	6.75E-30
DBT	ZNF609	0.7217234	6.26E-30
DBT	RBFOX2	0.72189112	5.99E-30
DBT	ZFR	0.72192311	5.94E-30
DBT	BMI1	0.72216358	5.57E-30

DBT	PNRC2	0.7223572	5.29E-30
DBT	STT3B	0.72246676	5.13E-30
DBT	ATF1	0.72308014	4.36E-30
DBT	IL6ST	0.7233197	4.09E-30
DBT	BTBD1	0.72332461	4.08E-30
DBT	GNAI3	0.72373549	3.65E-30
DBT	YY1	0.72379933	3.59E-30
DBT	SF3A3	0.72386981	3.52E-30
DBT	ZEB1	0.72396057	3.44E-30
DBT	ROCK2	0.72421914	3.21E-30
DBT	WDR3	0.72444782	3.02E-30
DBT	NF1	0.72476703	2.77E-30
DBT	MIB1	0.72486897	2.69E-30
DBT	SENP1	0.72502931	2.58E-30
DBT	FAM98A	0.72537142	2.35E-30
DBT	RAB3GAP1	0.72559608	2.21E-30
DBT	CHD9	0.72587196	2.05E-30
DBT	SNX18	0.72587261	2.05E-30
DBT	MED13	0.72624399	1.85E-30
DBT	KPNA6	0.72630937	1.82E-30
DBT	SMC3	0.72650943	1.73E-30
DBT	COMMD2	0.72667019	1.65E-30
DBT	CNOT6	0.72706483	1.48E-30
DBT	RBM7	0.72717967	1.44E-30
DBT	USP8	0.72721549	1.42E-30
DBT	YME1L1	0.72739966	1.35E-30
DBT	NUDT3	0.72741277	1.35E-30
DBT	PHACTR2	0.7274312	1.34E-30
DBT	PRRC1	0.72773824	1.23E-30
DBT	ENOX2	0.72784479	1.2E-30
DBT	COPS2	0.72804232	1.14E-30
DBT	AGGF1	0.72885225	9.09E-31
DBT	ARIH1	0.72904684	8.62E-31
DBT	G3BP1	0.72942136	7.77E-31
DBT	ARL14EP	0.72956409	7.47E-31
DBT	MATR3	0.72977846	7.04E-31
DBT	IPO7	0.73073641	5.4E-31
DBT	USP38	0.7314832	4.39E-31
DBT	RAP1A	0.73197528	3.83E-31
DBT	RASAL2	0.73237521	3.42E-31
DBT	SOS2	0.73255705	3.25E-31
DBT	TRUB1	0.73261567	3.2E-31
DBT	FAM102B	0.73266695	3.16E-31
DBT	ANKRD50	0.7327111	3.12E-31
DBT	TMX1	0.73276257	3.07E-31
DBT	GCLM	0.73335169	2.61E-31
DBT	ZNF45	0.73345397	2.53E-31
DBT	SYNCRIP	0.73348548	2.51E-31
DBT	ATF7IP	0.73349071	2.51E-31
DBT	METTL14	0.73369194	2.37E-31
DBT	AP5M1	0.73392998	2.21E-31
DBT	PAK2	0.73411961	2.1E-31
DBT	PPP1R8	0.73433714	1.97E-31
DBT	TMTC3	0.7348456	1.71E-31
DBT	ZBTB6	0.7349316	1.67E-31
DBT	PPP1CB	0.73524349	1.53E-31
DBT	SRFBP1	0.7354265	1.45E-31
DBT	MBNL2	0.73549036	1.43E-31

DBT	RTF1	0.73581691	1.3E-31
DBT	CBL	0.73588982	1.27E-31
DBT	ESF1	0.73609513	1.2E-31
DBT	INIP	0.73634168	1.12E-31
DBT	PPP1R12A	0.73654064	1.06E-31
DBT	ACTR2	0.73675305	9.96E-32
DBT	DDX3X	0.73688628	9.59E-32
DBT	FOXN2	0.73690251	9.54E-32
DBT	PPIG	0.73698791	9.31E-32
DBT	YIPF5	0.73723033	8.69E-32
DBT	HEG1	0.73726469	8.6E-32
DBT	RP2	0.73776832	7.45E-32
DBT	KPNA3	0.73790967	7.16E-32
DBT	RBM43	0.73816381	6.65E-32
DBT	AKAP11	0.73848501	6.07E-32
DBT	CREB1	0.73967938	4.3E-32
DBT	RABL3	0.73979089	4.16E-32
DBT	FTO	0.73988627	4.05E-32
DBT	SP3	0.73993307	4E-32
DBT	GTF3C4	0.74018136	3.72E-32
DBT	ATMIN	0.74042292	3.47E-32
DBT	PGGT1B	0.74118469	2.78E-32
DBT	NRAS	0.74127849	2.71E-32
DBT	CDC5L	0.7413174	2.68E-32
DBT	CAND1	0.74158014	2.48E-32
DBT	SMAD1	0.74184793	2.29E-32
DBT	PCNP	0.74196101	2.22E-32
DBT	RO60	0.74201079	2.19E-32
DBT	OXR1	0.74212823	2.11E-32
DBT	CAPZA2	0.74275128	1.76E-32
DBT	ARMC1	0.74332874	1.49E-32
DBT	UBE2W	0.74416794	1.16E-32
DBT	STX7	0.74432748	1.11E-32
DBT	RAB6A	0.7443949	1.09E-32
DBT	PUM2	0.74450313	1.05E-32
DBT	CPNE3	0.74488057	9.4E-33
DBT	SF3A1	0.74501217	9.05E-33
DBT	DLAT	0.74531207	8.28E-33
DBT	FCHO2	0.74531946	8.26E-33
DBT	SOCS5	0.74544963	7.95E-33
DBT	SNX6	0.74550431	7.82E-33
DBT	PPP4R3B	0.7460386	6.67E-33
DBT	PDCL	0.74617979	6.4E-33
DBT	SPAST	0.74618331	6.39E-33
DBT	ORC4	0.74618885	6.38E-33
DBT	SRGAP2B	0.7462593	6.25E-33
DBT	ATF2	0.74678094	5.35E-33
DBT	RBBP4	0.74731081	4.56E-33
DBT	NPAT	0.74731635	4.56E-33
DBT	TTC33	0.74778156	3.96E-33
DBT	PJA2	0.74813226	3.57E-33
DBT	HS2ST1	0.74899217	2.75E-33
DBT	HMGXB4	0.7491661	2.61E-33
DBT	TAOK1	0.74923936	2.56E-33
DBT	UBXN2B	0.74937634	2.45E-33
DBT	SPOPL	0.74987403	2.11E-33
DBT	PIGK	0.75015276	1.94E-33
DBT	WDR82	0.75059755	1.69E-33

DBT	PDS5B	0.75117329	1.42E-33
DBT	EIF4G3	0.75120494	1.41E-33
DBT	DPY19L4	0.75136368	1.34E-33
DBT	SNX16	0.751582	1.26E-33
DBT	AFF4	0.75202062	1.1E-33
DBT	C5orf24	0.75204158	1.09E-33
DBT	PHAX	0.75210205	1.07E-33
DBT	CPSF2	0.75240478	9.76E-34
DBT	ITCH	0.75250115	9.48E-34
DBT	RPS6KA3	0.75253689	9.37E-34
DBT	ROCK1	0.75274728	8.79E-34
DBT	SLC30A6	0.7528521	8.51E-34
DBT	RSF1	0.75312315	7.83E-34
DBT	TAB2	0.75328974	7.44E-34
DBT	ZNHIT6	0.75371137	6.53E-34
DBT	RAB18	0.75395691	6.05E-34
DBT	FEM1B	0.75451317	5.1E-34
DBT	C5orf51	0.75483292	4.62E-34
DBT	DOCK7	0.75489591	4.53E-34
DBT	STRN	0.75490657	4.51E-34
DBT	MTDH	0.75500075	4.38E-34
DBT	CCSER2	0.75508837	4.26E-34
DBT	VCPIP1	0.75516425	4.17E-34
DBT	DNAJB14	0.75543025	3.83E-34
DBT	ZFAND3	0.75563428	3.6E-34
DBT	EPS15	0.75584602	3.37E-34
DBT	SSH1	0.75646512	2.78E-34
DBT	PLEKHA3	0.75674202	2.55E-34
DBT	ZBTB41	0.75683039	2.48E-34
DBT	RECQL	0.75770174	1.88E-34
DBT	KAT6A	0.75877323	1.34E-34
DBT	PTPRG	0.75900439	1.25E-34
DBT	MED17	0.75901032	1.25E-34
DBT	SPIN1	0.75985992	9.53E-35
DBT	QKI	0.7599411	9.29E-35
DBT	FOXN3	0.76139436	5.84E-35
DBT	ENAH	0.76162588	5.42E-35
DBT	CTBS	0.76263794	3.92E-35
DBT	CSNK1G3	0.76275313	3.78E-35
DBT	PLSCR4	0.76291807	3.58E-35
DBT	ATP6V1A	0.76293191	3.56E-35
DBT	ATP10D	0.76313006	3.34E-35
DBT	YTHDF3	0.7632315	3.24E-35
DBT	PPP2R5E	0.76345045	3.01E-35
DBT	ADAM10	0.76359392	2.88E-35
DBT	RBM27	0.76367103	2.81E-35
DBT	TEAD1	0.76448462	2.16E-35
DBT	DDX6	0.76553533	1.53E-35
DBT	COA7	0.76615948	1.25E-35
DBT	NEDD1	0.76627926	1.2E-35
DBT	TLK1	0.76639009	1.16E-35
DBT	RB1	0.76702523	9.39E-36
DBT	ARL13B	0.76745319	8.15E-36
DBT	OGFOD1	0.76762793	7.7E-36
DBT	STAU2	0.76939774	4.28E-36
DBT	SNTB2	0.77020979	3.26E-36
DBT	FAM168A	0.77041145	3.05E-36
DBT	TOR1AIP1	0.77141859	2.18E-36

DBT	THRAP3	0.77144103	2.16E-36
DBT	EFR3A	0.77148864	2.12E-36
DBT	DPYD	0.77178562	1.92E-36
DBT	WAC	0.7721021	1.73E-36
DBT	API5	0.77233079	1.6E-36
DBT	RBM12	0.77296023	1.29E-36
DBT	ZBTB33	0.77304053	1.26E-36
DBT	VAMP7	0.77339282	1.12E-36
DBT	NCOA2	0.7743653	8.01E-37
DBT	UBE3A	0.77488185	6.71E-37
DBT	SMAD2	0.77575479	4.97E-37
DBT	CTTNBP2NL	0.77658007	3.74E-37
DBT	OSBPL8	0.77663909	3.67E-37
DBT	PPP4R2	0.77666387	3.63E-37
DBT	KIF2A	0.77729212	2.92E-37
DBT	WDR44	0.77742997	2.79E-37
DBT	NCOA4	0.77782075	2.43E-37
DBT	PKD2	0.77792699	2.34E-37
DBT	FBXL3	0.77805741	2.24E-37
DBT	ALG11	0.77863434	1.83E-37
DBT	SETD7	0.77939001	1.41E-37
DBT	PUM1	0.780527	9.43E-38
DBT	UEVLD	0.78066969	8.97E-38
DBT	EPC2	0.78080925	8.53E-38
DBT	ZFP1	0.78125562	7.29E-38
DBT	STAG2	0.78133669	7.08E-38
DBT	SMC1A	0.78185815	5.89E-38
DBT	CUL2	0.7819353	5.73E-38
DBT	SPTY2D1	0.78211007	5.38E-38
DBT	MBD5	0.78301263	3.9E-38
DBT	LUZP1	0.7833286	3.48E-38
DBT	HIPK3	0.78459019	2.21E-38
DBT	BMPR2	0.78539377	1.66E-38
DBT	RNF13	0.78550061	1.59E-38
DBT	AHCYL1	0.78553725	1.57E-38
DBT	NBN	0.78566186	1.5E-38
DBT	USP9X	0.78572076	1.47E-38
DBT	SLAIN2	0.78646379	1.12E-38
DBT	GOLIM4	0.78703692	9.11E-39
DBT	FCF1	0.78704741	9.08E-39
DBT	PCYOX1	0.78721173	8.55E-39
DBT	ACAP2	0.78751153	7.66E-39
DBT	GNL3L	0.78789128	6.66E-39
DBT	OPA1	0.78808152	6.22E-39
DBT	EVI5	0.78825397	5.83E-39
DBT	EFCAB14	0.78861728	5.1E-39
DBT	OSBPL11	0.78963016	3.51E-39
DBT	JAK1	0.79007845	2.97E-39
DBT	TRAK2	0.79137459	1.83E-39
DBT	GTF2A1	0.79144624	1.79E-39
DBT	ZFP91	0.7948934	4.86E-40
DBT	TFAM	0.79584577	3.38E-40
DBT	HIPK1	0.79585091	3.37E-40
DBT	GNG12	0.79609594	3.07E-40
DBT	EXOC5	0.79748572	1.8E-40
DBT	NFATC3	0.7990844	9.65E-41
DBT	LEPROT	0.80027446	6.05E-41
DBT	ZMYM4	0.80035245	5.87E-41

DBT	MIGA1	0.80104495	4.47E-41
DBT	DR1	0.80396353	1.4E-41
DBT	KPNA1	0.80399179	1.38E-41
DBT	WDR47	0.8041788	1.28E-41
DBT	CFAP97	0.80553737	7.42E-42
DBT	ZC3H13	0.80562374	7.16E-42
DBT	MAP3K2	0.80568686	6.98E-42
DBT	ARL5A	0.80815325	2.55E-42
DBT	MAPK1	0.80911488	1.71E-42
DBT	MFSD14A	0.80933705	1.56E-42
DBT	TNPO1	0.81048782	9.7E-43
DBT	BBX	0.811531	6.27E-43
DBT	TBC1D5	0.81207532	4.99E-43
DBT	ARHGEF12	0.81325912	3.02E-43
DBT	MIER1	0.81535816	1.24E-43
DBT	CSDE1	0.81575722	1.04E-43
DBT	ANKRD13C	0.81731676	5.3E-44
DBT	ZBTB38	0.81840857	3.29E-44
DBT	USP1	0.81847962	3.19E-44
DBT	TMED5	0.81928999	2.24E-44
DBT	PIK3CA	0.81972496	1.85E-44
DBT	RNF11	0.82767009	5.09E-46
DBT	DCUN1D1	0.83113234	1E-46
DBT	ZYG11B	0.83505491	1.52E-47
DBT	MFAP3	0.8352499	1.39E-47
DBT	SLC35A5	0.83592619	9.96E-48
DBT	ASXL2	0.84957161	9.03E-51
DBT	TMEM167B	0.84985345	7.76E-51
DBT	ZNF148	0.85452696	5.96E-52
DBT	CTDSPL2	0.85599315	2.62E-52
DBT	FYTTD1	0.86318417	4.02E-54
DLAT	RABEPK	0.50005994	1.19E-12
DLAT	PKM	0.50010854	1.18E-12
DLAT	ITGB3	0.50044324	1.13E-12
DLAT	BMP1	0.50044985	1.13E-12
DLAT	PRCP	0.50052525	1.12E-12
DLAT	SIPA1L2	0.50066688	1.11E-12
DLAT	PSMA2	0.50078154	1.09E-12
DLAT	ADAM19	0.50092489	1.07E-12
DLAT	SPIRE1	0.50095211	1.07E-12
DLAT	ADAMTS4	0.50095281	1.07E-12
DLAT	ETNK1	0.5009561	1.07E-12
DLAT	NET1	0.50101278	1.06E-12
DLAT	CLMP	0.50103948	1.06E-12
DLAT	CMAS	0.50108503	1.05E-12
DLAT	SRPX2	0.50113566	1.04E-12
DLAT	SRF	0.50114085	1.04E-12
DLAT	BTF3	0.50115969	1.04E-12
DLAT	TRIM26	0.50124155	1.03E-12
DLAT	CREBL2	0.5012496	1.03E-12
DLAT	TNFRSF1A	0.50133268	1.02E-12
DLAT	EIPR1	0.50157982	9.91E-13
DLAT	BRK1	0.50162638	9.85E-13
DLAT	TPD52	0.50174365	9.71E-13
DLAT	NOL7	0.50178321	9.67E-13
DLAT	SLC37A2	0.50193404	9.49E-13
DLAT	AMOTL2	0.50208489	9.32E-13
DLAT	SLC41A2	0.50216593	9.23E-13

DLAT	CTDNEP1	0.50217754	9.22E-13
DLAT	AGPAT1	0.50218274	9.22E-13
DLAT	ZDHHC13	0.50238678	8.99E-13
DLAT	THAP11	0.50239939	8.98E-13
DLAT	GNE	0.50241675	8.96E-13
DLAT	RCAN1	0.50252037	8.85E-13
DLAT	FAM234A	0.50256335	8.8E-13
DLAT	C1orf216	0.50264051	8.72E-13
DLAT	IFNAR2	0.50270791	8.65E-13
DLAT	TTI2	0.50272022	8.64E-13
DLAT	CDON	0.50281015	8.55E-13
DLAT	TAF6	0.50283665	8.52E-13
DLAT	ZNF438	0.5030881	8.26E-13
DLAT	GRK2	0.50318048	8.17E-13
DLAT	ITPR3	0.50336734	7.99E-13
DLAT	GGCT	0.50346585	7.9E-13
DLAT	TARS2	0.50346974	7.89E-13
DLAT	DLGAP4	0.50358972	7.78E-13
DLAT	COL15A1	0.50362375	7.75E-13
DLAT	YIPF1	0.50369677	7.68E-13
DLAT	LRRC8A	0.50373305	7.65E-13
DLAT	PDPN	0.50376258	7.62E-13
DLAT	TSFM	0.50380695	7.58E-13
DLAT	RBM47	0.50382452	7.56E-13
DLAT	MAF1	0.50383316	7.55E-13
DLAT	NGRN	0.50389323	7.5E-13
DLAT	PAQR7	0.50416285	7.26E-13
DLAT	TMEM199	0.50436551	7.08E-13
DLAT	SF3B2	0.50440528	7.05E-13
DLAT	LIX1L	0.50464403	6.85E-13
DLAT	DYNC2LI1	0.50467923	6.82E-13
DLAT	NIT2	0.50489737	6.64E-13
DLAT	CAV1	0.50497761	6.58E-13
DLAT	ARMC9	0.50500258	6.56E-13
DLAT	FBLIM1	0.50506644	6.51E-13
DLAT	VSTM4	0.50525092	6.36E-13
DLAT	MMP14	0.50528158	6.34E-13
DLAT	CRISPLD1	0.50546723	6.2E-13
DLAT	TRIB2	0.50565419	6.06E-13
DLAT	PPM1G	0.50594148	5.85E-13
DLAT	ZNF28	0.50614035	5.71E-13
DLAT	NID2	0.50636481	5.56E-13
DLAT	TK2	0.50636699	5.56E-13
DLAT	PYGO2	0.50637394	5.55E-13
DLAT	F13A1	0.50656209	5.43E-13
DLAT	VOPP1	0.50665302	5.37E-13
DLAT	IL1R1	0.50666714	5.36E-13
DLAT	FCGR2A	0.50676028	5.3E-13
DLAT	ARHGAP26	0.50680834	5.27E-13
DLAT	PECR	0.50683577	5.25E-13
DLAT	NDUFA5	0.50688784	5.21E-13
DLAT	NDUFAF1	0.50703172	5.12E-13
DLAT	PSMA7	0.50711137	5.07E-13
DLAT	TMBIM1	0.50712097	5.07E-13
DLAT	RIOK2	0.50714627	5.05E-13
DLAT	GTF2F2	0.50715502	5.05E-13
DLAT	PVR	0.50716781	5.04E-13
DLAT	IFIT2	0.50722075	5.01E-13

DLAT	HMOX2	0.50727013	4.98E-13
DLAT	TGM2	0.50741919	4.89E-13
DLAT	COQ5	0.50742489	4.88E-13
DLAT	SGO1	0.5074479	4.87E-13
DLAT	DGKH	0.50749102	4.84E-13
DLAT	MN1	0.50761306	4.77E-13
DLAT	GLTP	0.50770271	4.72E-13
DLAT	LXN	0.50792827	4.59E-13
DLAT	EFEMP1	0.50807599	4.51E-13
DLAT	B3GNT9	0.50837582	4.35E-13
DLAT	ALKBH1	0.50849308	4.29E-13
DLAT	TSHZ1	0.50871931	4.17E-13
DLAT	PHF23	0.50876149	4.15E-13
DLAT	ZNF286A	0.50884474	4.1E-13
DLAT	SCCPDH	0.5088793	4.09E-13
DLAT	TTLL5	0.50890764	4.07E-13
DLAT	TAF9B	0.5089275	4.06E-13
DLAT	ITGB3BP	0.50894674	4.05E-13
DLAT	INTS13	0.50908978	3.98E-13
DLAT	FIG4	0.50919319	3.93E-13
DLAT	CTSB	0.50923354	3.91E-13
DLAT	LEF1	0.50932018	3.87E-13
DLAT	NABP1	0.50945743	3.81E-13
DLAT	FAM83D	0.50947928	3.8E-13
DLAT	SHROOM3	0.50959912	3.74E-13
DLAT	HHAT	0.50969185	3.7E-13
DLAT	TRAK1	0.50981167	3.64E-13
DLAT	TTK	0.50986323	3.62E-13
DLAT	KCTD11	0.51007796	3.53E-13
DLAT	NTM	0.51010144	3.52E-13
DLAT	ILK	0.51015804	3.49E-13
DLAT	JAM3	0.51016272	3.49E-13
DLAT	TSPAN6	0.51020261	3.47E-13
DLAT	CRISPLD2	0.51031942	3.42E-13
DLAT	C8orf76	0.51047768	3.36E-13
DLAT	ACACA	0.51057217	3.32E-13
DLAT	SPRY1	0.51063729	3.29E-13
DLAT	UBE2L3	0.51064514	3.29E-13
DLAT	BLOC1S2	0.51095949	3.16E-13
DLAT	GLRX	0.51098269	3.15E-13
DLAT	CHST3	0.51113983	3.09E-13
DLAT	CMC2	0.51114359	3.09E-13
DLAT	GTF2A2	0.51114534	3.09E-13
DLAT	CDCA4	0.51114752	3.09E-13
DLAT	TMEM168	0.51119194	3.07E-13
DLAT	EMP1	0.51123494	3.06E-13
DLAT	ADGRA2	0.51128003	3.04E-13
DLAT	SUB1	0.51136021	3.01E-13
DLAT	BUD13	0.51137318	3.01E-13
DLAT	ENTPD5	0.51137553	3E-13
DLAT	DAD1	0.51139261	3E-13
DLAT	EHD1	0.51153197	2.95E-13
DLAT	SNUPN	0.51156595	2.93E-13
DLAT	BAG3	0.51172281	2.88E-13
DLAT	MRPL18	0.51175459	2.87E-13
DLAT	THY1	0.51178684	2.86E-13
DLAT	F11R	0.51183352	2.84E-13
DLAT	EHD3	0.51191537	2.81E-13

DLAT	NSMCE3	0.51202816	2.77E-13
DLAT	SLC35B2	0.51205948	2.76E-13
DLAT	ECM2	0.51213194	2.74E-13
DLAT	BORA	0.51215	2.73E-13
DLAT	ARMH4	0.51216187	2.73E-13
DLAT	NMT1	0.51218872	2.72E-13
DLAT	TMEM140	0.5122037	2.71E-13
DLAT	NPC1	0.51234363	2.66E-13
DLAT	RIC8A	0.51238719	2.65E-13
DLAT	CCT6A	0.51253681	2.6E-13
DLAT	SMAD7	0.51265241	2.56E-13
DLAT	DDB1	0.51270978	2.55E-13
DLAT	NEK2	0.51272185	2.54E-13
DLAT	PNRC1	0.51275708	2.53E-13
DLAT	IVD	0.5128706	2.5E-13
DLAT	FZR1	0.51288876	2.49E-13
DLAT	NEDD9	0.51303124	2.45E-13
DLAT	GPR157	0.51308948	2.43E-13
DLAT	FN3KRP	0.51319286	2.4E-13
DLAT	ZMYND8	0.51323774	2.38E-13
DLAT	FTSJ3	0.51334098	2.35E-13
DLAT	S1PR3	0.51342964	2.33E-13
DLAT	RBMX2	0.51367445	2.26E-13
DLAT	TSKU	0.51370619	2.25E-13
DLAT	DKK3	0.51388235	2.2E-13
DLAT	CCNDBP1	0.51391537	2.19E-13
DLAT	ZBED1	0.51407941	2.15E-13
DLAT	POC1B	0.51409249	2.14E-13
DLAT	TNFAIP6	0.51411563	2.14E-13
DLAT	OXCT1	0.51416466	2.12E-13
DLAT	C12orf43	0.51442497	2.06E-13
DLAT	POSTN	0.51450461	2.04E-13
DLAT	MRPL16	0.51465931	2E-13
DLAT	TGFB2	0.51469265	1.99E-13
DLAT	CDK4	0.51480888	1.96E-13
DLAT	PANK2	0.51481776	1.96E-13
DLAT	PKP2	0.51483648	1.95E-13
DLAT	CYREN	0.51488995	1.94E-13
DLAT	TCF7L2	0.514929	1.93E-13
DLAT	XRCC1	0.51493758	1.93E-13
DLAT	MRPL50	0.51541935	1.82E-13
DLAT	THOC7	0.51542784	1.81E-13
DLAT	CD93	0.51546538	1.81E-13
DLAT	JADE2	0.51550915	1.8E-13
DLAT	HTRA2	0.51567581	1.76E-13
DLAT	TSPAN5	0.51587472	1.71E-13
DLAT	ATXN10	0.51592014	1.7E-13
DLAT	CTSC	0.51592717	1.7E-13
DLAT	TIGAR	0.51594894	1.7E-13
DLAT	VPS72	0.51595704	1.7E-13
DLAT	ING1	0.51602457	1.68E-13
DLAT	CENPF	0.51615391	1.66E-13
DLAT	DAXX	0.51615432	1.66E-13
DLAT	PIM1	0.51615759	1.65E-13
DLAT	FAAP24	0.51622536	1.64E-13
DLAT	SIPA1L3	0.5162864	1.63E-13
DLAT	STMP1	0.51630712	1.62E-13
DLAT	EIF3I	0.51640448	1.6E-13

DLAT	FANCI	0.51641041	1.6E-13
DLAT	RCC1L	0.51653325	1.58E-13
DLAT	CDKN2AIP	0.51658382	1.57E-13
DLAT	ANAPC13	0.51659539	1.57E-13
DLAT	WNT5A	0.51675441	1.54E-13
DLAT	SLC38A6	0.51675991	1.53E-13
DLAT	NKAP	0.51678361	1.53E-13
DLAT	NCAPG	0.51696869	1.49E-13
DLAT	MCUB	0.51712646	1.46E-13
DLAT	CASP7	0.517146	1.46E-13
DLAT	TBC1D9	0.51718647	1.45E-13
DLAT	RHNO1	0.51730731	1.43E-13
DLAT	PPP1R11	0.51734751	1.42E-13
DLAT	TMEM14A	0.51736094	1.42E-13
DLAT	SULF1	0.51760643	1.38E-13
DLAT	TTC39B	0.51764678	1.37E-13
DLAT	BNC2	0.51766309	1.37E-13
DLAT	ZNF185	0.51768104	1.37E-13
DLAT	PKNOX1	0.51786552	1.33E-13
DLAT	PDGFRA	0.51795785	1.32E-13
DLAT	MAP7D1	0.5180731	1.3E-13
DLAT	HNRNPM	0.51846518	1.24E-13
DLAT	INPP5F	0.51847754	1.23E-13
DLAT	ANKH	0.51889742	1.17E-13
DLAT	PNMA1	0.51909012	1.14E-13
DLAT	TMEM185A	0.51912428	1.14E-13
DLAT	SPON1	0.51945925	1.09E-13
DLAT	RDH10	0.51960407	1.07E-13
DLAT	CIAO2A	0.51979728	1.04E-13
DLAT	LOXL3	0.52007539	1.01E-13
DLAT	CHST14	0.52013388	1E-13
DLAT	FAM222B	0.52015006	9.99E-14
DLAT	TFDP2	0.52029163	9.81E-14
DLAT	EXOSC9	0.52036659	9.71E-14
DLAT	CD68	0.5204112	9.66E-14
DLAT	SLC2A9	0.52041732	9.65E-14
DLAT	TAF8	0.52042064	9.65E-14
DLAT	PIAS1	0.52047682	9.58E-14
DLAT	ASPM	0.52056655	9.47E-14
DLAT	RAC1	0.52063423	9.39E-14
DLAT	SLC35A2	0.52065856	9.36E-14
DLAT	SLC35B4	0.52090222	9.07E-14
DLAT	HNRNPL	0.52096306	9E-14
DLAT	DGCR2	0.52108704	8.86E-14
DLAT	PHF19	0.52108792	8.86E-14
DLAT	FAT1	0.52113589	8.81E-14
DLAT	SLC35A1	0.52114027	8.8E-14
DLAT	SP110	0.52122332	8.71E-14
DLAT	ANP32B	0.52125736	8.67E-14
DLAT	TP53RK	0.52127183	8.65E-14
DLAT	DOCK5	0.52136029	8.56E-14
DLAT	MREG	0.52145974	8.45E-14
DLAT	MKLN1	0.52179851	8.09E-14
DLAT	CPSF3	0.5218053	8.08E-14
DLAT	EPSTI1	0.52191261	7.97E-14
DLAT	EIF4A1	0.52206845	7.82E-14
DLAT	SVIP	0.52210469	7.78E-14
DLAT	NCOA7	0.52217911	7.71E-14

DLAT	EFNA5	0.52223435	7.65E-14
DLAT	MARVELD1	0.52252368	7.37E-14
DLAT	UHRF1	0.52254433	7.35E-14
DLAT	CDK1	0.52261386	7.29E-14
DLAT	CDK14	0.52280959	7.11E-14
DLAT	RFC2	0.52286404	7.06E-14
DLAT	RAD51	0.52290671	7.02E-14
DLAT	GLIPR1	0.52309575	6.85E-14
DLAT	MAPRE2	0.52321826	6.74E-14
DLAT	EIF4A3	0.52337346	6.61E-14
DLAT	LRRC8C	0.52339584	6.59E-14
DLAT	DEPTOR	0.52357898	6.44E-14
DLAT	ZNF816	0.52358133	6.43E-14
DLAT	BZW2	0.52362441	6.4E-14
DLAT	CEP55	0.52367616	6.36E-14
DLAT	BTG3	0.52370378	6.33E-14
DLAT	MOB3B	0.52382647	6.23E-14
DLAT	CENPO	0.52402605	6.08E-14
DLAT	CCDC34	0.52404354	6.06E-14
DLAT	CEP89	0.52404399	6.06E-14
DLAT	GM2A	0.52422488	5.92E-14
DLAT	TNFAIP8	0.52433379	5.84E-14
DLAT	TRMT10C	0.52438289	5.8E-14
DLAT	CCDC51	0.52451126	5.71E-14
DLAT	RECK	0.52451274	5.71E-14
DLAT	THBS2	0.52466339	5.6E-14
DLAT	HSDL1	0.52481242	5.49E-14
DLAT	IPPK	0.52491617	5.42E-14
DLAT	MYO5A	0.52492311	5.41E-14
DLAT	RPN1	0.52505086	5.32E-14
DLAT	SLC20A1	0.52517421	5.24E-14
DLAT	ACTR8	0.52524985	5.19E-14
DLAT	ABLIM3	0.52528854	5.16E-14
DLAT	RCAN3	0.52544537	5.06E-14
DLAT	ATXN7L3	0.52561493	4.95E-14
DLAT	PCSK7	0.52567237	4.91E-14
DLAT	DCAF6	0.52593425	4.75E-14
DLAT	FLVCR2	0.52610801	4.64E-14
DLAT	MAF	0.52624224	4.56E-14
DLAT	BUB1B	0.52625831	4.55E-14
DLAT	DDX58	0.52629586	4.53E-14
DLAT	DIDO1	0.52635137	4.5E-14
DLAT	GSR	0.52639616	4.47E-14
DLAT	CNPPD1	0.52640889	4.46E-14
DLAT	PRICKLE1	0.52655753	4.38E-14
DLAT	NAP1L1	0.52663208	4.34E-14
DLAT	FERMT2	0.5267118	4.29E-14
DLAT	TCF19	0.52673065	4.28E-14
DLAT	TIMM23	0.52675509	4.27E-14
DLAT	TMEM159	0.526954	4.16E-14
DLAT	NOD1	0.52716481	4.04E-14
DLAT	FLT1	0.52720246	4.03E-14
DLAT	PRPS1	0.5274016	3.92E-14
DLAT	GALNT3	0.52742194	3.91E-14
DLAT	CDKL1	0.52749933	3.87E-14
DLAT	EMC4	0.52755462	3.84E-14
DLAT	PCTP	0.5276226	3.81E-14
DLAT	MRAS	0.52778151	3.73E-14

DLAT	PLA2G15	0.52801315	3.62E-14
DLAT	PEX3	0.52804233	3.61E-14
DLAT	PIP4K2B	0.52807858	3.59E-14
DLAT	NCAPD2	0.52815228	3.56E-14
DLAT	HPSE	0.52820699	3.53E-14
DLAT	PARP9	0.52847674	3.41E-14
DLAT	SDF4	0.5285972	3.35E-14
DLAT	COA4	0.5286466	3.33E-14
DLAT	WIPF1	0.5286974	3.31E-14
DLAT	KIF13B	0.52872023	3.3E-14
DLAT	ALDH1A3	0.52881003	3.26E-14
DLAT	TM2D3	0.52901127	3.18E-14
DLAT	CHP1	0.52909323	3.14E-14
DLAT	ZFYVE21	0.52913918	3.12E-14
DLAT	IL7	0.52918825	3.1E-14
DLAT	RUNX1	0.52923747	3.08E-14
DLAT	THBS1	0.5292769	3.07E-14
DLAT	ADGRG6	0.52936523	3.03E-14
DLAT	EDIL3	0.52938062	3.03E-14
DLAT	SNAPC5	0.52948119	2.99E-14
DLAT	MSRB3	0.52950731	2.98E-14
DLAT	SSX2IP	0.52962611	2.93E-14
DLAT	TPR	0.52977786	2.87E-14
DLAT	VCP	0.53006099	2.77E-14
DLAT	PDAP1	0.53006548	2.77E-14
DLAT	SLCO3A1	0.53031463	2.68E-14
DLAT	ETV1	0.53037439	2.66E-14
DLAT	STMN1	0.53049169	2.62E-14
DLAT	SP100	0.53052443	2.6E-14
DLAT	AP2M1	0.53054378	2.6E-14
DLAT	AJUBA	0.53057635	2.59E-14
DLAT	NTAN1	0.53061775	2.57E-14
DLAT	PRR16	0.53065848	2.56E-14
DLAT	ATP6V1G1	0.53090039	2.48E-14
DLAT	CD2BP2	0.53091591	2.47E-14
DLAT	NARS2	0.53093574	2.47E-14
DLAT	C4orf33	0.53099332	2.45E-14
DLAT	DAPP1	0.53134255	2.34E-14
DLAT	PLEKHA7	0.53136076	2.33E-14
DLAT	AMBRA1	0.53146149	2.3E-14
DLAT	PRKX	0.53154984	2.27E-14
DLAT	SERPINB8	0.53179976	2.2E-14
DLAT	COL5A1	0.53189855	2.17E-14
DLAT	RARB	0.53195511	2.16E-14
DLAT	SNN	0.53200375	2.14E-14
DLAT	MGAT4B	0.53216591	2.1E-14
DLAT	WIPI2	0.5322094	2.08E-14
DLAT	CTTN	0.53228219	2.06E-14
DLAT	LAPTM4B	0.53253683	2E-14
DLAT	SRD5A1	0.53263037	1.97E-14
DLAT	DNAAF5	0.53278193	1.93E-14
DLAT	BCAR3	0.53283532	1.92E-14
DLAT	ST3GAL2	0.53292556	1.9E-14
DLAT	SLC9A7	0.53324682	1.82E-14
DLAT	TSHZ3	0.5334786	1.76E-14
DLAT	ZNF274	0.53366125	1.72E-14
DLAT	LMAN2L	0.53370739	1.71E-14
DLAT	COL3A1	0.53371508	1.71E-14

DLAT	TOMM22	0.5339167	1.66E-14
DLAT	TIGD2	0.53394065	1.66E-14
DLAT	LRP8	0.53396739	1.65E-14
DLAT	GPR176	0.53404431	1.63E-14
DLAT	TSPYL1	0.53421267	1.6E-14
DLAT	NUAK1	0.53465172	1.51E-14
DLAT	GEM	0.53473165	1.49E-14
DLAT	DIAPH1	0.53478621	1.48E-14
DLAT	PPME1	0.53487287	1.46E-14
DLAT	MXRA5	0.53490607	1.46E-14
DLAT	MRPS28	0.5349444	1.45E-14
DLAT	HMGN1	0.53501352	1.44E-14
DLAT	TMEM9B	0.53511236	1.42E-14
DLAT	GINS1	0.53524785	1.39E-14
DLAT	BST1	0.53525307	1.39E-14
DLAT	GPKOW	0.53527862	1.39E-14
DLAT	KIF4A	0.53529237	1.38E-14
DLAT	NFIL3	0.5353759	1.37E-14
DLAT	LRRC1	0.53550802	1.34E-14
DLAT	AC008764.4	0.53572988	1.3E-14
DLAT	DSN1	0.53586854	1.28E-14
DLAT	ATP6V0E1	0.53590985	1.27E-14
DLAT	GADD45A	0.53595007	1.27E-14
DLAT	ATP5MC3	0.53600704	1.26E-14
DLAT	AKT3	0.53612984	1.24E-14
DLAT	ERC1	0.53626506	1.21E-14
DLAT	NFE2L3	0.53645493	1.18E-14
DLAT	GAS7	0.53649814	1.18E-14
DLAT	RUVBL1	0.53655361	1.17E-14
DLAT	IGF2BP3	0.53694178	1.11E-14
DLAT	SF3B6	0.53712646	1.08E-14
DLAT	PIGC	0.53715389	1.08E-14
DLAT	PLXNA1	0.53724858	1.06E-14
DLAT	MYH10	0.53729732	1.06E-14
DLAT	PLXDC2	0.53732339	1.05E-14
DLAT	ADAMTS2	0.53753037	1.02E-14
DLAT	DAB2	0.53772825	9.97E-15
DLAT	TMEM184B	0.53787583	9.77E-15
DLAT	RFK	0.53795374	9.67E-15
DLAT	ZFPM2	0.53796207	9.66E-15
DLAT	TPGS2	0.538046	9.55E-15
DLAT	MED30	0.53809173	9.49E-15
DLAT	NOL4L	0.53832665	9.19E-15
DLAT	ATXN7L1	0.53832989	9.19E-15
DLAT	JHY	0.53839999	9.1E-15
DLAT	AIMP2	0.53842773	9.07E-15
DLAT	IGFBP5	0.53843899	9.05E-15
DLAT	METTL18	0.5384782	9.01E-15
DLAT	PDK3	0.53856711	8.9E-15
DLAT	SLC25A44	0.53858919	8.87E-15
DLAT	CDC6	0.53872445	8.71E-15
DLAT	TGFA	0.53878222	8.64E-15
DLAT	LIMK2	0.53885696	8.56E-15
DLAT	CASP6	0.53894468	8.46E-15
DLAT	LYAR	0.53897677	8.42E-15
DLAT	SURF4	0.53899165	8.4E-15
DLAT	COL8A1	0.53904305	8.34E-15
DLAT	ANXA5	0.5391246	8.25E-15

DLAT	KNL1	0.53916746	8.2E-15
DLAT	MCM2	0.53922078	8.15E-15
DLAT	EIF1B	0.53931979	8.04E-15
DLAT	MIS18A	0.53938556	7.97E-15
DLAT	ZEB2	0.53946312	7.88E-15
DLAT	CENPK	0.53956188	7.78E-15
DLAT	FOXO1	0.53997521	7.35E-15
DLAT	ATXN7L3B	0.54009813	7.23E-15
DLAT	ISCA1	0.54012206	7.21E-15
DLAT	HDGF	0.54012711	7.2E-15
DLAT	PMAIP1	0.54020687	7.12E-15
DLAT	STARD4	0.54036098	6.98E-15
DLAT	KREMEN1	0.54036893	6.97E-15
DLAT	POLDIP3	0.54050092	6.84E-15
DLAT	SPDYE3	0.54062454	6.73E-15
DLAT	SLFN12	0.5406613	6.7E-15
DLAT	DUSP7	0.54075117	6.62E-15
DLAT	TCEAL8	0.54085699	6.52E-15
DLAT	STEAP1	0.54093159	6.45E-15
DLAT	CPED1	0.54109431	6.31E-15
DLAT	TSPAN17	0.54110932	6.3E-15
DLAT	PTTG1IP	0.54121585	6.21E-15
DLAT	ITGB5	0.54122478	6.2E-15
DLAT	LBH	0.54127027	6.16E-15
DLAT	AMFR	0.54150117	5.97E-15
DLAT	CFL1	0.54175809	5.77E-15
DLAT	MPZL3	0.54194163	5.62E-15
DLAT	PDE4D	0.54200463	5.57E-15
DLAT	CENPN	0.54200693	5.57E-15
DLAT	MEST	0.54205296	5.54E-15
DLAT	CTDSPL	0.54223208	5.4E-15
DLAT	SRP14	0.54225255	5.39E-15
DLAT	OAZ2	0.54228086	5.37E-15
DLAT	YY1AP1	0.54242057	5.27E-15
DLAT	MB21D2	0.54243246	5.26E-15
DLAT	ARHGAP12	0.54257591	5.15E-15
DLAT	BCCIP	0.54261122	5.13E-15
DLAT	MRPS23	0.54277962	5.01E-15
DLAT	MICOS10-NBL1	0.54282523	4.98E-15
DLAT	CASP4	0.54290088	4.93E-15
DLAT	PRKCA	0.54296872	4.88E-15
DLAT	ITGA4	0.54334722	4.64E-15
DLAT	PRKG1	0.54349994	4.54E-15
DLAT	BCL2L1	0.54352272	4.53E-15
DLAT	MAGOH	0.54358929	4.49E-15
DLAT	LTBP2	0.54364762	4.45E-15
DLAT	SH3RF1	0.54393078	4.28E-15
DLAT	MDH1	0.54400216	4.24E-15
DLAT	BHLHE41	0.54401568	4.23E-15
DLAT	SLC35F6	0.54401571	4.23E-15
DLAT	ZCCHC10	0.54408593	4.19E-15
DLAT	ARHGAP31	0.54410631	4.18E-15
DLAT	KIAA0586	0.5441413	4.16E-15
DLAT	AP3B1	0.54424195	4.1E-15
DLAT	OSBPL3	0.54437637	4.02E-15
DLAT	HELZ2	0.54442421	4E-15
DLAT	TNFAIP3	0.54443036	3.99E-15
DLAT	MYCBP	0.54444675	3.99E-15

DLAT	AAGAB	0.54453748	3.94E-15
DLAT	ATP6V1B2	0.54455086	3.93E-15
DLAT	FARSB	0.54473528	3.83E-15
DLAT	TLR4	0.54474816	3.82E-15
DLAT	AK4	0.54476857	3.81E-15
DLAT	APH1A	0.54477609	3.81E-15
DLAT	INTS7	0.5448633	3.76E-15
DLAT	KIF20A	0.54489611	3.75E-15
DLAT	SUMF1	0.54494511	3.72E-15
DLAT	ETS2	0.54497072	3.71E-15
DLAT	TRMT12	0.54499673	3.69E-15
DLAT	TSEN15	0.54516419	3.61E-15
DLAT	USP34	0.54526457	3.56E-15
DLAT	METTL4	0.54533747	3.52E-15
DLAT	ATG9A	0.54540836	3.49E-15
DLAT	ATP9A	0.54543984	3.47E-15
DLAT	KIF26B	0.54552406	3.43E-15
DLAT	PTPRJ	0.54574753	3.33E-15
DLAT	ZNF526	0.54606205	3.19E-15
DLAT	CD164	0.5461018	3.17E-15
DLAT	GINS3	0.54611357	3.16E-15
DLAT	ANKRD11	0.5461717	3.14E-15
DLAT	TMEM200A	0.5463093	3.08E-15
DLAT	URM1	0.54633476	3.07E-15
DLAT	SAMD12	0.54659619	2.96E-15
DLAT	ADAMTS12	0.5466483	2.94E-15
DLAT	CDCP1	0.54674876	2.9E-15
DLAT	SMIM30	0.54679649	2.88E-15
DLAT	TNIK	0.54686069	2.85E-15
DLAT	PRMT5	0.54686919	2.85E-15
DLAT	CHSY3	0.54690324	2.84E-15
DLAT	ALDH9A1	0.54690956	2.83E-15
DLAT	SCRN1	0.54692688	2.83E-15
DLAT	COL6A3	0.54710006	2.76E-15
DLAT	ZDHHC7	0.54743041	2.64E-15
DLAT	PCMT1	0.54743421	2.63E-15
DLAT	ZMAT3	0.54755019	2.59E-15
DLAT	ZDHHC6	0.54755664	2.59E-15
DLAT	GPX8	0.54765257	2.55E-15
DLAT	AK6	0.54765812	2.55E-15
DLAT	GBP1	0.54767768	2.55E-15
DLAT	RETREG2	0.54786214	2.48E-15
DLAT	SPRY4	0.54789231	2.47E-15
DLAT	ARHGAP23	0.54790644	2.47E-15
DLAT	ITGB8	0.54798417	2.44E-15
DLAT	NUDT4	0.54802511	2.43E-15
DLAT	PDIA4	0.54811852	2.39E-15
DLAT	GOLGA5	0.54839264	2.3E-15
DLAT	PCDH1	0.54857044	2.25E-15
DLAT	BCL10	0.54860568	2.24E-15
DLAT	CLTC	0.54872797	2.2E-15
DLAT	CLDN12	0.54891764	2.14E-15
DLAT	KIF3C	0.54899919	2.12E-15
DLAT	CCNA2	0.54904094	2.1E-15
DLAT	ZNF277	0.54908766	2.09E-15
DLAT	NREP	0.54911976	2.08E-15
DLAT	TRIB1	0.54914128	2.07E-15
DLAT	DPP9	0.54917703	2.06E-15

DLAT	PTPRF	0.54919497	2.06E-15
DLAT	HTATSF1	0.54937225	2.01E-15
DLAT	TNFRSF21	0.54945635	1.99E-15
DLAT	EPB41L1	0.54964548	1.93E-15
DLAT	LPCAT2	0.54970107	1.92E-15
DLAT	UBE2E2	0.54972498	1.91E-15
DLAT	KIF23	0.54973137	1.91E-15
DLAT	MBOAT1	0.54996979	1.85E-15
DLAT	DDX23	0.55020876	1.79E-15
DLAT	ENC1	0.55062977	1.68E-15
DLAT	SPARC	0.55063696	1.68E-15
DLAT	PDGFRB	0.55076379	1.65E-15
DLAT	WDR76	0.55082542	1.64E-15
DLAT	RNF220	0.55087054	1.63E-15
DLAT	STK17A	0.55088626	1.62E-15
DLAT	THOC3	0.55105203	1.59E-15
DLAT	MED18	0.55130119	1.53E-15
DLAT	XIAP	0.5514399	1.5E-15
DLAT	HMCN1	0.55155573	1.48E-15
DLAT	HPRT1	0.55162485	1.46E-15
DLAT	AHSA1	0.55164638	1.46E-15
DLAT	RAB27A	0.55170786	1.45E-15
DLAT	ITM2B	0.55172061	1.44E-15
DLAT	WSB2	0.55189166	1.41E-15
DLAT	YTHDF1	0.55189462	1.41E-15
DLAT	BBS2	0.55196934	1.39E-15
DLAT	STON1	0.55218413	1.35E-15
DLAT	ACTR1A	0.55219207	1.35E-15
DLAT	AMPD3	0.55232731	1.32E-15
DLAT	ZNF174	0.55234849	1.32E-15
DLAT	ST3GAL1	0.55249126	1.29E-15
DLAT	NOTCH3	0.5525361	1.29E-15
DLAT	TRIM34	0.55255554	1.28E-15
DLAT	ILF2	0.55258672	1.28E-15
DLAT	LEPROTL1	0.55263329	1.27E-15
DLAT	GNAI2	0.55282733	1.23E-15
DLAT	SPSB1	0.55283902	1.23E-15
DLAT	PRXL2C	0.55295228	1.21E-15
DLAT	ELOC	0.55298811	1.21E-15
DLAT	RAMAC	0.55308456	1.19E-15
DLAT	RPL26L1	0.55311167	1.19E-15
DLAT	SLC12A2	0.55320617	1.17E-15
DLAT	TLE4	0.55323197	1.16E-15
DLAT	ADGRF5	0.55366459	1.1E-15
DLAT	LIPA	0.55368743	1.09E-15
DLAT	WBP1L	0.55371537	1.09E-15
DLAT	CNN3	0.55377631	1.08E-15
DLAT	GID8	0.55378233	1.08E-15
DLAT	FADD	0.55389876	1.06E-15
DLAT	COPZ1	0.55396088	1.05E-15
DLAT	ABCC4	0.55396799	1.05E-15
DLAT	TTC23	0.55417897	1.02E-15
DLAT	NECTIN3	0.55447962	9.75E-16
DLAT	INPP5A	0.55454272	9.67E-16
DLAT	MRFAP1	0.55500946	9.04E-16
DLAT	TM2D2	0.55512974	8.89E-16
DLAT	MAPKAP1	0.55514347	8.87E-16
DLAT	ITPRIP	0.55517054	8.84E-16

DLAT	OPN3	0.5552271	8.77E-16
DLAT	KLF12	0.55540026	8.55E-16
DLAT	CAMK2G	0.55544528	8.5E-16
DLAT	TOMM20	0.55546838	8.47E-16
DLAT	ZNF350	0.55594139	7.91E-16
DLAT	PCDH18	0.55594761	7.91E-16
DLAT	MGRN1	0.55606892	7.77E-16
DLAT	ALDH7A1	0.55608114	7.76E-16
DLAT	RAP2B	0.55611403	7.72E-16
DLAT	TLK2	0.5561303	7.7E-16
DLAT	PTGR1	0.55615402	7.68E-16
DLAT	CTPS1	0.55629059	7.53E-16
DLAT	ENSA	0.55638898	7.42E-16
DLAT	NIN	0.55639317	7.42E-16
DLAT	HMGCR	0.55643986	7.37E-16
DLAT	HMGB2	0.5564496	7.36E-16
DLAT	RABIF	0.55647883	7.33E-16
DLAT	HSD17B11	0.55658158	7.22E-16
DLAT	SDHAF3	0.55658494	7.22E-16
DLAT	TIMM8A	0.55665515	7.14E-16
DLAT	CGAS	0.55680152	6.99E-16
DLAT	PTBP1	0.55680754	6.99E-16
DLAT	IPO9	0.55695336	6.84E-16
DLAT	RAD51AP1	0.55701854	6.78E-16
DLAT	EDNRA	0.5573423	6.47E-16
DLAT	EXTL3	0.55737111	6.44E-16
DLAT	FZD7	0.55763701	6.2E-16
DLAT	IKBIP	0.55775033	6.1E-16
DLAT	CHCHD7	0.55805671	5.84E-16
DLAT	PAIP2	0.5581553	5.76E-16
DLAT	NIFK	0.55823461	5.69E-16
DLAT	EXO5	0.55826339	5.67E-16
DLAT	ERO1A	0.5582719	5.66E-16
DLAT	TAF7	0.55828765	5.65E-16
DLAT	ERH	0.55836415	5.58E-16
DLAT	LSM14B	0.55850052	5.48E-16
DLAT	TRAF7	0.55871143	5.31E-16
DLAT	TCAIM	0.55880634	5.24E-16
DLAT	ENO1	0.55889941	5.17E-16
DLAT	PSMA5	0.55906982	5.04E-16
DLAT	NDFIP1	0.55947116	4.76E-16
DLAT	MBOAT2	0.55969456	4.61E-16
DLAT	METTL2A	0.5597054	4.6E-16
DLAT	RASSF8	0.55974639	4.57E-16
DLAT	ROBO1	0.55976669	4.56E-16
DLAT	GLI2	0.55977258	4.55E-16
DLAT	GYS1	0.56003352	4.38E-16
DLAT	DNAJC9	0.56009036	4.35E-16
DLAT	MYLK	0.56017583	4.29E-16
DLAT	LAMA4	0.56030774	4.21E-16
DLAT	HSPA5	0.56042384	4.14E-16
DLAT	MBIP	0.5604625	4.12E-16
DLAT	CCDC59	0.56050006	4.1E-16
DLAT	TRNAU1AP	0.56064856	4.01E-16
DLAT	CIT	0.56099382	3.81E-16
DLAT	RELA	0.56108642	3.76E-16
DLAT	EPHB2	0.56109299	3.76E-16
DLAT	SLC39A6	0.56125381	3.67E-16

DLAT	RASSF3	0.5613122	3.64E-16
DLAT	IRAK3	0.56132675	3.63E-16
DLAT	GMPR2	0.56133019	3.63E-16
DLAT	MR1	0.56140051	3.59E-16
DLAT	C1orf198	0.56150146	3.54E-16
DLAT	TMEM35B	0.56154413	3.52E-16
DLAT	NFIX	0.56158469	3.5E-16
DLAT	BECN1	0.56178168	3.4E-16
DLAT	SPTBN1	0.56185941	3.36E-16
DLAT	TUBA1C	0.56197277	3.3E-16
DLAT	TIAM2	0.5620033	3.29E-16
DLAT	MICU2	0.5621073	3.24E-16
DLAT	IRAK2	0.56222369	3.19E-16
DLAT	OAS3	0.56224537	3.18E-16
DLAT	P4HA1	0.56225658	3.17E-16
DLAT	GPBP1	0.56239088	3.11E-16
DLAT	HACD3	0.5623984	3.1E-16
DLAT	RDH11	0.56246284	3.08E-16
DLAT	DSC2	0.5626127	3.01E-16
DLAT	AP1S2	0.56283817	2.91E-16
DLAT	KIAA1671	0.56309202	2.8E-16
DLAT	NSD3	0.56317148	2.77E-16
DLAT	DLGAP5	0.5633278	2.71E-16
DLAT	CDKN1B	0.56333719	2.71E-16
DLAT	SMN1	0.5633756	2.69E-16
DLAT	COQ2	0.56343301	2.67E-16
DLAT	SMURF1	0.56376709	2.54E-16
DLAT	PSMA6	0.56382411	2.52E-16
DLAT	ENTPD1	0.56387996	2.5E-16
DLAT	FUCA1	0.56408891	2.42E-16
DLAT	DUS4L	0.5641195	2.41E-16
DLAT	EPB41L4A	0.56414588	2.4E-16
DLAT	CRYBG1	0.56419819	2.38E-16
DLAT	KLHL18	0.56461107	2.24E-16
DLAT	GNB4	0.56472142	2.21E-16
DLAT	RAB29	0.56485391	2.16E-16
DLAT	HRH1	0.56485679	2.16E-16
DLAT	KITLG	0.56493962	2.14E-16
DLAT	CYTH3	0.56515879	2.07E-16
DLAT	GLRX5	0.56540658	1.99E-16
DLAT	RRM2	0.56541839	1.99E-16
DLAT	NAP1L4	0.56551633	1.96E-16
DLAT	RABGAP1L	0.56554474	1.95E-16
DLAT	SLC15A4	0.56567869	1.92E-16
DLAT	ZDHHC3	0.56570269	1.91E-16
DLAT	WBP4	0.56595756	1.84E-16
DLAT	HNRNPAB	0.56611207	1.8E-16
DLAT	SELENON	0.56612728	1.79E-16
DLAT	MAP1LC3B2	0.56626121	1.76E-16
DLAT	HTATIP2	0.56639371	1.72E-16
DLAT	TFPI	0.56642704	1.71E-16
DLAT	PPIL3	0.56646051	1.71E-16
DLAT	TCF4	0.56652383	1.69E-16
DLAT	PSMD8	0.56655866	1.68E-16
DLAT	LDHA	0.56662035	1.67E-16
DLAT	CYBRD1	0.5667361	1.64E-16
DLAT	AP3M2	0.56677126	1.63E-16
DLAT	SLC35F2	0.5668024	1.62E-16

DLAT	PRKCI	0.56680708	1.62E-16
DLAT	PDGFC	0.56735222	1.49E-16
DLAT	HNRNPA0	0.5673829	1.49E-16
DLAT	HDGFL3	0.56746039	1.47E-16
DLAT	GNB5	0.56748273	1.46E-16
DLAT	BRI3BP	0.56752713	1.46E-16
DLAT	AURKA	0.56771568	1.41E-16
DLAT	AXL	0.567742	1.41E-16
DLAT	AKIRIN2	0.56776962	1.4E-16
DLAT	ARSJ	0.56787612	1.38E-16
DLAT	CCN4	0.56789987	1.38E-16
DLAT	NECAP2	0.56834893	1.29E-16
DLAT	CENPE	0.56835086	1.29E-16
DLAT	ATP2B4	0.56841738	1.27E-16
DLAT	CBLB	0.56841827	1.27E-16
DLAT	GALNS	0.56848835	1.26E-16
DLAT	ETV6	0.56875693	1.21E-16
DLAT	AVL9	0.56887915	1.19E-16
DLAT	YBX1	0.56892883	1.18E-16
DLAT	COL4A2	0.56921777	1.13E-16
DLAT	TCFL5	0.56928403	1.12E-16
DLAT	ATP2B1	0.56933092	1.11E-16
DLAT	CD82	0.56943171	1.09E-16
DLAT	SIPA1L1	0.56945156	1.09E-16
DLAT	ANLN	0.56954285	1.08E-16
DLAT	TIPARP	0.56974481	1.04E-16
DLAT	LTA4H	0.56979994	1.04E-16
DLAT	VDR	0.56986942	1.02E-16
DLAT	MTHFD1L	0.56988395	1.02E-16
DLAT	DRAM1	0.56998901	1.01E-16
DLAT	SUGT1	0.5700456	9.98E-17
DLAT	LYST	0.57014952	9.83E-17
DLAT	FNTB	0.57019762	9.76E-17
DLAT	TNFRSF10A	0.57022161	9.72E-17
DLAT	ANXA1	0.57026309	9.66E-17
DLAT	TMBIM4	0.57030364	9.6E-17
DLAT	JAK2	0.57032245	9.57E-17
DLAT	PLSCR1	0.57053972	9.27E-17
DLAT	HMGCS1	0.57067421	9.08E-17
DLAT	IQCK	0.57072319	9.01E-17
DLAT	ZBTB4	0.57087645	8.81E-17
DLAT	ELOVL1	0.57095315	8.71E-17
DLAT	GREM1	0.57131225	8.25E-17
DLAT	PLOD2	0.57132455	8.23E-17
DLAT	SPOP	0.57137269	8.17E-17
DLAT	SKA2	0.57154912	7.96E-17
DLAT	MARCKS	0.57166577	7.82E-17
DLAT	TCOF1	0.57175708	7.71E-17
DLAT	SOX4	0.57178019	7.68E-17
DLAT	SGPP2	0.57196857	7.47E-17
DLAT	ANTXR1	0.57201739	7.41E-17
DLAT	NCSTN	0.57211385	7.31E-17
DLAT	EPB41L2	0.57229016	7.11E-17
DLAT	LRP11	0.57229716	7.11E-17
DLAT	COL5A2	0.57237485	7.02E-17
DLAT	INPP4B	0.57239157	7.01E-17
DLAT	PXN	0.572397	7E-17
DLAT	ARFIP1	0.57245098	6.94E-17

DLAT	PKP4	0.57278421	6.6E-17
DLAT	IFIT5	0.57288716	6.5E-17
DLAT	FAS	0.57295137	6.43E-17
DLAT	EHF	0.57295721	6.43E-17
DLAT	MRPS35	0.57298792	6.4E-17
DLAT	MSN	0.57299115	6.4E-17
DLAT	OXNAD1	0.57302608	6.36E-17
DLAT	ARF1	0.5732691	6.13E-17
DLAT	RNASEL	0.57331094	6.09E-17
DLAT	PTMA	0.57354159	5.88E-17
DLAT	POLR3C	0.573551	5.87E-17
DLAT	AFG3L2	0.57357461	5.85E-17
DLAT	PGAM5	0.57363229	5.8E-17
DLAT	RNF149	0.57420472	5.32E-17
DLAT	CLUAP1	0.5743639	5.19E-17
DLAT	AFAP1L1	0.57468977	4.94E-17
DLAT	DIPK1A	0.57472539	4.91E-17
DLAT	AKIP1	0.57473766	4.9E-17
DLAT	MYO1D	0.57478325	4.87E-17
DLAT	SH2B3	0.57493836	4.75E-17
DLAT	MITF	0.57494931	4.75E-17
DLAT	SCOC	0.57505483	4.67E-17
DLAT	MAD2L1	0.57506729	4.66E-17
DLAT	COPG2	0.57531311	4.49E-17
DLAT	FAM122B	0.57578316	4.18E-17
DLAT	SORT1	0.57611606	3.97E-17
DLAT	SHCBP1	0.57611618	3.97E-17
DLAT	PACS1	0.57620549	3.92E-17
DLAT	GPSM2	0.5763592	3.82E-17
DLAT	FAM120AOS	0.57641396	3.79E-17
DLAT	IL6ST	0.57661345	3.68E-17
DLAT	DUSP6	0.57692403	3.51E-17
DLAT	DPY30	0.57707196	3.43E-17
DLAT	LPIN2	0.57708159	3.42E-17
DLAT	IMPACT	0.57714925	3.39E-17
DLAT	NMI	0.57721928	3.35E-17
DLAT	STEAP2	0.57733121	3.29E-17
DLAT	SLC38A1	0.57752133	3.2E-17
DLAT	NEK6	0.578027	2.96E-17
DLAT	TIMP2	0.57814575	2.9E-17
DLAT	MAN2B2	0.57818429	2.89E-17
DLAT	LOX	0.57818905	2.88E-17
DLAT	TMEM263	0.57823891	2.86E-17
DLAT	HNRNPA3	0.57839608	2.79E-17
DLAT	CMIP	0.57847904	2.76E-17
DLAT	RRAS2	0.5785523	2.73E-17
DLAT	NUDT15	0.57861068	2.7E-17
DLAT	NAV1	0.57861136	2.7E-17
DLAT	CHEK1	0.57888352	2.59E-17
DLAT	DACT1	0.57894125	2.57E-17
DLAT	ESD	0.57895245	2.56E-17
DLAT	HK1	0.57912747	2.49E-17
DLAT	FKBP7	0.57918365	2.47E-17
DLAT	XRCC6	0.57933687	2.42E-17
DLAT	CDC123	0.57959468	2.32E-17
DLAT	PHC2	0.57975119	2.26E-17
DLAT	KATNA1	0.57995568	2.19E-17
DLAT	HDAC9	0.58010561	2.14E-17

DLAT	BABAM2	0.58017864	2.12E-17
DLAT	FASTKD5	0.58041609	2.04E-17
DLAT	YWHAE	0.58045701	2.03E-17
DLAT	MRPS30	0.58053425	2E-17
DLAT	KANK2	0.58059422	1.99E-17
DLAT	BTN3A3	0.58067114	1.96E-17
DLAT	PSMA3	0.58077091	1.93E-17
DLAT	MINPP1	0.58091684	1.89E-17
DLAT	TBC1D14	0.58103571	1.85E-17
DLAT	CLN5	0.58115173	1.82E-17
DLAT	PXMP4	0.58116862	1.82E-17
DLAT	UBE2Q1	0.58144487	1.74E-17
DLAT	DIAPH3	0.58159466	1.7E-17
DLAT	STK24	0.58166942	1.68E-17
DLAT	VPS29	0.58173048	1.66E-17
DLAT	GLT8D1	0.58194217	1.61E-17
DLAT	PRDM1	0.582064	1.58E-17
DLAT	BAZ1A	0.58209929	1.57E-17
DLAT	PHF5A	0.58213581	1.56E-17
DLAT	MTX2	0.58222739	1.54E-17
DLAT	NIPSNAP3A	0.58235925	1.51E-17
DLAT	B4GALT1	0.58260572	1.45E-17
DLAT	FUT11	0.58266136	1.44E-17
DLAT	ADCY7	0.58270012	1.43E-17
DLAT	ACO2	0.58271995	1.42E-17
DLAT	RPF1	0.58279198	1.41E-17
DLAT	CHST11	0.58301781	1.36E-17
DLAT	EIF3M	0.58305007	1.35E-17
DLAT	PIK3C3	0.58321983	1.32E-17
DLAT	INHBA	0.58332632	1.3E-17
DLAT	RASA2	0.58337325	1.29E-17
DLAT	LIPG	0.58348681	1.26E-17
DLAT	GAB2	0.58366223	1.23E-17
DLAT	HSBP1	0.583793	1.2E-17
DLAT	ZCRB1	0.58384198	1.19E-17
DLAT	TRIM59	0.58398828	1.17E-17
DLAT	INTS5	0.58411118	1.14E-17
DLAT	HSPA4	0.58432182	1.11E-17
DLAT	USP39	0.58470707	1.04E-17
DLAT	OSTC	0.58474827	1.03E-17
DLAT	APEX2	0.58482529	1.02E-17
DLAT	UBE2L6	0.58486022	1.02E-17
DLAT	BPTF	0.58489551	1.01E-17
DLAT	PLS1	0.58493884	1E-17
DLAT	IK	0.58500229	9.94E-18
DLAT	RNF8	0.58502253	9.91E-18
DLAT	UBAC2	0.58503104	9.9E-18
DLAT	GSKIP	0.58510635	9.78E-18
DLAT	CHCHD4	0.58511637	9.76E-18
DLAT	C1orf43	0.58528429	9.51E-18
DLAT	PCNA	0.58530599	9.48E-18
DLAT	TRMT6	0.58534154	9.42E-18
DLAT	FRMD4B	0.58535146	9.41E-18
DLAT	TJP2	0.5854805	9.22E-18
DLAT	SORD	0.58548932	9.2E-18
DLAT	GPR137B	0.58549227	9.2E-18
DLAT	COX15	0.58553635	9.14E-18
DLAT	CEP250	0.58553837	9.13E-18

DLAT	GOLPH3L	0.58558529	9.07E-18
DLAT	MDFIC	0.58589745	8.63E-18
DLAT	CDK2AP1	0.58610487	8.35E-18
DLAT	ABHD17B	0.58620537	8.22E-18
DLAT	SLFN11	0.58625357	8.15E-18
DLAT	NDUFB5	0.58628141	8.12E-18
DLAT	ARMCX6	0.58635403	8.02E-18
DLAT	XYLT1	0.58637827	7.99E-18
DLAT	ARID5B	0.58671956	7.57E-18
DLAT	SIAE	0.58678118	7.5E-18
DLAT	MICU1	0.58682012	7.45E-18
DLAT	LSM12	0.58693447	7.32E-18
DLAT	MRPS27	0.58693799	7.31E-18
DLAT	ABHD2	0.58720421	7.01E-18
DLAT	CALM3	0.58728399	6.92E-18
DLAT	SPOCK1	0.58752441	6.66E-18
DLAT	MFHAS1	0.58773058	6.44E-18
DLAT	KLHL12	0.5879022	6.27E-18
DLAT	RIN2	0.58790656	6.27E-18
DLAT	PPP3CB	0.58805106	6.12E-18
DLAT	LPGAT1	0.58808549	6.09E-18
DLAT	PRTFDC1	0.58818582	5.99E-18
DLAT	SRRD	0.5881969	5.98E-18
DLAT	KDELR2	0.58823967	5.94E-18
DLAT	SLC4A7	0.58836536	5.82E-18
DLAT	DCLRE1C	0.58840095	5.79E-18
DLAT	PXDN	0.58848795	5.71E-18
DLAT	NIPSNAP1	0.58875391	5.47E-18
DLAT	KDM1A	0.58884246	5.39E-18
DLAT	STPG1	0.58889992	5.34E-18
DLAT	RPA2	0.58892388	5.32E-18
DLAT	CETN3	0.58940935	4.93E-18
DLAT	WWTR1	0.58942751	4.91E-18
DLAT	RAB1B	0.58970892	4.69E-18
DLAT	PDE6D	0.58984572	4.59E-18
DLAT	VCAN	0.58986097	4.58E-18
DLAT	MICAL2	0.58988235	4.56E-18
DLAT	DNASE1L1	0.58993914	4.52E-18
DLAT	STK17B	0.58999425	4.48E-18
DLAT	JPT2	0.59013409	4.38E-18
DLAT	GALNT7	0.59016164	4.36E-18
DLAT	PSMA4	0.59027651	4.28E-18
DLAT	EMC7	0.59057065	4.09E-18
DLAT	SUSD1	0.59070723	4E-18
DLAT	CDH11	0.59087895	3.89E-18
DLAT	GTF2B	0.59090644	3.87E-18
DLAT	PPIL1	0.59132084	3.62E-18
DLAT	RCC2	0.59153285	3.5E-18
DLAT	PPP2R5C	0.59153684	3.5E-18
DLAT	LSM6	0.59156555	3.48E-18
DLAT	CPD	0.59170417	3.4E-18
DLAT	KIF11	0.59182202	3.34E-18
DLAT	NDRG3	0.59184594	3.32E-18
DLAT	SPATS2L	0.59218006	3.15E-18
DLAT	FRRS1	0.59219677	3.14E-18
DLAT	ANTXR2	0.59227909	3.1E-18
DLAT	CTNNBIP1	0.59238334	3.05E-18
DLAT	SLC39A1	0.59241078	3.03E-18

DLAT	VPS33A	0.59246603	3.01E-18
DLAT	NT5E	0.59255644	2.96E-18
DLAT	BICD1	0.59290433	2.8E-18
DLAT	TMEM154	0.59290543	2.8E-18
DLAT	ELP3	0.59293889	2.78E-18
DLAT	SEMA3A	0.59301002	2.75E-18
DLAT	PLBD2	0.59326561	2.64E-18
DLAT	LRP12	0.59332433	2.62E-18
DLAT	GNA12	0.5936151	2.49E-18
DLAT	SMIM12	0.59382704	2.41E-18
DLAT	TRAM2	0.59406489	2.32E-18
DLAT	BTN3A1	0.59406995	2.32E-18
DLAT	MED8	0.59408288	2.31E-18
DLAT	H6PD	0.59438168	2.2E-18
DLAT	ZMYM5	0.59441988	2.19E-18
DLAT	LCLAT1	0.5945974	2.12E-18
DLAT	PIP4K2A	0.59460568	2.12E-18
DLAT	FAM210B	0.59464152	2.11E-18
DLAT	MEGF9	0.5947302	2.08E-18
DLAT	PAK1	0.59479802	2.06E-18
DLAT	TOP2B	0.59489635	2.02E-18
DLAT	FBN1	0.59492248	2.01E-18
DLAT	SNAP29	0.5949381	2.01E-18
DLAT	RTCB	0.59496037	2E-18
DLAT	HADHA	0.59503672	1.98E-18
DLAT	ARHGAP1	0.59528434	1.9E-18
DLAT	EIF2AK1	0.59530102	1.89E-18
DLAT	FAM83B	0.59558492	1.81E-18
DLAT	BCORL1	0.59563971	1.79E-18
DLAT	DTL	0.59591439	1.71E-18
DLAT	ITGA6	0.5960482	1.67E-18
DLAT	FGD6	0.59625677	1.62E-18
DLAT	BBS9	0.59625941	1.62E-18
DLAT	SNRNP200	0.59656758	1.54E-18
DLAT	SLFN5	0.59681667	1.48E-18
DLAT	SUCLG2	0.59686787	1.46E-18
DLAT	AKTIP	0.59686794	1.46E-18
DLAT	PELO	0.59687978	1.46E-18
DLAT	PTPRK	0.59693822	1.45E-18
DLAT	NAALADL2	0.59695085	1.44E-18
DLAT	CDS2	0.59704719	1.42E-18
DLAT	MET	0.59705422	1.42E-18
DLAT	WDHD1	0.59706383	1.42E-18
DLAT	DERA	0.59707822	1.41E-18
DLAT	ATPSCKMT	0.59718336	1.39E-18
DLAT	CNOT11	0.59719847	1.39E-18
DLAT	SLC24A1	0.59720639	1.38E-18
DLAT	TIMM17A	0.59739571	1.34E-18
DLAT	MMD	0.59749985	1.32E-18
DLAT	SRXN1	0.59778883	1.26E-18
DLAT	CCDC115	0.59784043	1.25E-18
DLAT	DDR2	0.59790488	1.23E-18
DLAT	ACLY	0.59806842	1.2E-18
DLAT	PRKAG1	0.59807117	1.2E-18
DLAT	LATS2	0.5981549	1.18E-18
DLAT	PRKACA	0.59820896	1.17E-18
DLAT	GLCE	0.59821601	1.17E-18
DLAT	CNOT1	0.59835853	1.14E-18

DLAT	PRR11	0.59840651	1.14E-18
DLAT	LITAF	0.59849963	1.12E-18
DLAT	CHIC2	0.59857181	1.1E-18
DLAT	MCC	0.59863668	1.09E-18
DLAT	PAFAH1B1	0.59867076	1.09E-18
DLAT	CBX5	0.59876505	1.07E-18
DLAT	PCSK5	0.59896315	1.03E-18
DLAT	SPIDR	0.59907316	1.02E-18
DLAT	LY75	0.59908382	1.01E-18
DLAT	COPS4	0.59940684	9.61E-19
DLAT	JMJD1C	0.59949497	9.47E-19
DLAT	PLSCR4	0.59951737	9.44E-19
DLAT	TEFM	0.59957282	9.35E-19
DLAT	MRFAP1L1	0.59966485	9.21E-19
DLAT	USB1	0.59970031	9.16E-19
DLAT	PNPO	0.59981994	8.98E-19
DLAT	HSPE1-MOB4	0.59983862	8.95E-19
DLAT	ISY1	0.59985337	8.93E-19
DLAT	RNF138	0.59988339	8.88E-19
DLAT	KIAA1217	0.59993785	8.8E-19
DLAT	LRCH3	0.60009221	8.58E-19
DLAT	ACVR2A	0.60015137	8.49E-19
DLAT	TTC9C	0.60042533	8.11E-19
DLAT	PRR14L	0.60043823	8.1E-19
DLAT	TRAFD1	0.60054562	7.95E-19
DLAT	APMAP	0.60084494	7.56E-19
DLAT	DNAJC25-GNG10	0.60086088	7.54E-19
DLAT	FYCO1	0.60088087	7.52E-19
DLAT	CFLAR	0.60098808	7.39E-19
DLAT	NUP133	0.60104219	7.32E-19
DLAT	PARPBP	0.60127954	7.03E-19
DLAT	RMDN3	0.60148419	6.8E-19
DLAT	GPALPP1	0.60150496	6.77E-19
DLAT	SSPN	0.6016382	6.62E-19
DLAT	FNIP2	0.60167533	6.58E-19
DLAT	CLIP4	0.60203641	6.2E-19
DLAT	HMGXB3	0.60207299	6.16E-19
DLAT	SDC3	0.60213441	6.1E-19
DLAT	TCEAL9	0.60213697	6.09E-19
DLAT	NRP1	0.6021848	6.04E-19
DLAT	RIPK2	0.60218646	6.04E-19
DLAT	CDC42BPA	0.60232519	5.9E-19
DLAT	XXYLT1	0.60244249	5.79E-19
DLAT	TMEM128	0.60254571	5.69E-19
DLAT	PIP4K2C	0.60277044	5.48E-19
DLAT	RAB31	0.60278895	5.46E-19
DLAT	PARVA	0.60279511	5.45E-19
DLAT	YWHAH	0.60281202	5.44E-19
DLAT	NRP2	0.60283106	5.42E-19
DLAT	USP4	0.60286461	5.39E-19
DLAT	GALNT10	0.60292133	5.34E-19
DLAT	ANO10	0.60295882	5.31E-19
DLAT	FGD4	0.60305059	5.23E-19
DLAT	FADS1	0.603096	5.19E-19
DLAT	UNC5B	0.60312147	5.16E-19
DLAT	CNNM4	0.60317014	5.12E-19
DLAT	LDB1	0.60332228	4.99E-19
DLAT	DSTN	0.60338547	4.94E-19

DLAT	BICD2	0.603412	4.92E-19
DLAT	CREG1	0.60376401	4.63E-19
DLAT	PPCS	0.60376801	4.63E-19
DLAT	DAG1	0.60382856	4.58E-19
DLAT	PFDN1	0.60387856	4.54E-19
DLAT	ATG16L1	0.60389905	4.53E-19
DLAT	DNAJA1	0.60395826	4.48E-19
DLAT	BTBD9	0.60403073	4.43E-19
DLAT	AAR2	0.60417188	4.32E-19
DLAT	CLP1	0.60433031	4.21E-19
DLAT	IDS	0.60443646	4.14E-19
DLAT	IRS1	0.60457317	4.04E-19
DLAT	ARHGAP35	0.60465937	3.98E-19
DLAT	JARID2	0.60470468	3.95E-19
DLAT	VMA21	0.60484286	3.86E-19
DLAT	MBNL3	0.60491638	3.81E-19
DLAT	TMEM165	0.60508463	3.71E-19
DLAT	B2M	0.60514921	3.66E-19
DLAT	ANO1	0.60524552	3.61E-19
DLAT	HEG1	0.60531359	3.56E-19
DLAT	TUBB	0.60556007	3.42E-19
DLAT	HNRNPA1	0.60597252	3.19E-19
DLAT	VDAC1	0.60604216	3.15E-19
DLAT	ASCC3	0.60607924	3.13E-19
DLAT	UTP3	0.6061619	3.09E-19
DLAT	GRB2	0.6062877	3.02E-19
DLAT	PSMD11	0.60630284	3.01E-19
DLAT	FBXL17	0.60638657	2.97E-19
DLAT	GLB1	0.60644345	2.94E-19
DLAT	GNG10	0.60645185	2.94E-19
DLAT	DCP2	0.60656634	2.88E-19
DLAT	SLC39A9	0.60659253	2.87E-19
DLAT	RNF145	0.60668898	2.82E-19
DLAT	FAM111B	0.60688667	2.73E-19
DLAT	PARD3B	0.60695282	2.7E-19
DLAT	UBE2V1	0.60721633	2.58E-19
DLAT	UBAP1	0.60732584	2.53E-19
DLAT	DIP2C	0.60741357	2.49E-19
DLAT	GNS	0.60752735	2.44E-19
DLAT	LRBA	0.60754884	2.43E-19
DLAT	MFSD6	0.60769428	2.38E-19
DLAT	AKAP12	0.60793675	2.28E-19
DLAT	ABL2	0.60797529	2.26E-19
DLAT	G6PD	0.6081978	2.18E-19
DLAT	PRUNE1	0.6083111	2.14E-19
DLAT	ELK4	0.60835392	2.12E-19
DLAT	MNAT1	0.60835902	2.12E-19
DLAT	EFNB2	0.60847504	2.08E-19
DLAT	DSG2	0.60860448	2.03E-19
DLAT	TOR1B	0.60876252	1.98E-19
DLAT	CAV2	0.60888228	1.94E-19
DLAT	TFCP2	0.60903507	1.89E-19
DLAT	LCORL	0.60903855	1.89E-19
DLAT	CCT8	0.60916821	1.84E-19
DLAT	FMN1	0.60918654	1.84E-19
DLAT	HIP1	0.60929664	1.8E-19
DLAT	BPGM	0.60958437	1.72E-19
DLAT	TNRC18	0.60961069	1.71E-19

DLAT	ATP6V1E1	0.60964347	1.7E-19
DLAT	OSER1	0.60964483	1.7E-19
DLAT	ZHX2	0.60966158	1.69E-19
DLAT	CCDC32	0.60987506	1.63E-19
DLAT	TRMT2B	0.60999885	1.6E-19
DLAT	CTSO	0.61026356	1.53E-19
DLAT	COX7A2L	0.61030555	1.52E-19
DLAT	AGO2	0.6103112	1.51E-19
DLAT	MSANTD3	0.61033712	1.51E-19
DLAT	PPP4R1	0.61051034	1.46E-19
DLAT	PHTF1	0.61054685	1.45E-19
DLAT	KNSTRN	0.6105985	1.44E-19
DLAT	SOGA1	0.6106936	1.42E-19
DLAT	METTL6	0.61083738	1.38E-19
DLAT	NCEH1	0.61109017	1.32E-19
DLAT	ZNF462	0.61135576	1.26E-19
DLAT	TRIM56	0.6117141	1.19E-19
DLAT	LMBRD1	0.61171826	1.19E-19
DLAT	ZNF250	0.61174586	1.18E-19
DLAT	TMEM50A	0.61183895	1.16E-19
DLAT	TTC4	0.61185496	1.16E-19
DLAT	KCTD3	0.61189163	1.15E-19
DLAT	BMP2K	0.61189941	1.15E-19
DLAT	U2AF2	0.61233804	1.07E-19
DLAT	SPAG1	0.6123514	1.06E-19
DLAT	TOR1AIP2	0.6123864	1.06E-19
DLAT	MCL1	0.61240015	1.05E-19
DLAT	TBC1D25	0.6124671	1.04E-19
DLAT	RALGPS2	0.61248951	1.04E-19
DLAT	ATF6B	0.61250807	1.03E-19
DLAT	SRSF7	0.61261453	1.02E-19
DLAT	CPNE8	0.61299782	9.5E-20
DLAT	NOCT	0.6130615	9.39E-20
DLAT	STX3	0.61315678	9.24E-20
DLAT	LLPH	0.61328194	9.04E-20
DLAT	MAX	0.61350349	8.7E-20
DLAT	CALCOCO2	0.613575	8.59E-20
DLAT	C1orf112	0.6136863	8.42E-20
DLAT	USP12	0.61388698	8.13E-20
DLAT	KDM5A	0.61388929	8.13E-20
DLAT	TPD52L2	0.61399837	7.98E-20
DLAT	DSE	0.61447555	7.34E-20
DLAT	UPRT	0.61448959	7.32E-20
DLAT	ATP11A	0.61468288	7.07E-20
DLAT	SYS1	0.61504028	6.64E-20
DLAT	NEO1	0.61511941	6.55E-20
DLAT	NR1D2	0.61514994	6.52E-20
DLAT	CDC42BPB	0.6152268	6.43E-20
DLAT	NUSAP1	0.61524012	6.41E-20
DLAT	CROT	0.6153086	6.34E-20
DLAT	SMU1	0.61536658	6.27E-20
DLAT	CLCN3	0.61537744	6.26E-20
DLAT	TRANK1	0.61547486	6.16E-20
DLAT	DHX35	0.61557202	6.05E-20
DLAT	TDRD7	0.6155871	6.03E-20
DLAT	ZCCHC17	0.61562611	5.99E-20
DLAT	GSK3B	0.61624804	5.37E-20
DLAT	PLEKHA2	0.61627496	5.35E-20

DLAT	IPO11	0.61632011	5.3E-20
DLAT	PI4K2A	0.61634202	5.28E-20
DLAT	SYF2	0.61635921	5.27E-20
DLAT	CALD1	0.61646684	5.17E-20
DLAT	SLC25A12	0.61652123	5.12E-20
DLAT	WWC3	0.61654373	5.1E-20
DLAT	CRTAP	0.61655172	5.09E-20
DLAT	CHURC1	0.61657484	5.07E-20
DLAT	ANKS1A	0.61659855	5.05E-20
DLAT	ZNF200	0.61663914	5.01E-20
DLAT	SAMD9	0.61663996	5.01E-20
DLAT	PACRGL	0.61670278	4.96E-20
DLAT	MKRN1	0.61671524	4.95E-20
DLAT	CPPED1	0.61680184	4.87E-20
DLAT	BTN2A1	0.61699178	4.71E-20
DLAT	PHTF2	0.61704168	4.67E-20
DLAT	ABCF1	0.61707253	4.64E-20
DLAT	EMB	0.61734079	4.43E-20
DLAT	ANKRD42	0.61734912	4.42E-20
DLAT	PRNP	0.61739062	4.39E-20
DLAT	OSBPL1A	0.61757935	4.24E-20
DLAT	DPH3	0.61762396	4.21E-20
DLAT	PHF13	0.61799765	3.94E-20
DLAT	PFKM	0.61807021	3.89E-20
DLAT	TMEM41A	0.61818341	3.81E-20
DLAT	FRMD6	0.61865238	3.51E-20
DLAT	UGGT1	0.61889868	3.36E-20
DLAT	FUT8	0.61897492	3.31E-20
DLAT	SVIL	0.61898096	3.31E-20
DLAT	TMBIM6	0.61901405	3.29E-20
DLAT	CNOT6L	0.61901903	3.29E-20
DLAT	ZNF227	0.61907012	3.26E-20
DLAT	CDK7	0.61908785	3.25E-20
DLAT	TULP3	0.61909635	3.24E-20
DLAT	FER	0.61919576	3.18E-20
DLAT	EID1	0.61958774	2.97E-20
DLAT	SPIN4	0.61998111	2.77E-20
DLAT	TRIM14	0.62002186	2.75E-20
DLAT	PARP14	0.62040736	2.56E-20
DLAT	KRCC1	0.62053737	2.51E-20
DLAT	UBASH3B	0.62063848	2.46E-20
DLAT	TOR1A	0.62096018	2.32E-20
DLAT	SCO1	0.62151243	2.1E-20
DLAT	KIRREL1	0.62190148	1.96E-20
DLAT	WNK1	0.62193375	1.95E-20
DLAT	FOSL2	0.62202893	1.92E-20
DLAT	NEK1	0.62204307	1.91E-20
DLAT	PDCL3	0.62207093	1.9E-20
DLAT	BPNT1	0.62209078	1.9E-20
DLAT	CLINT1	0.62217127	1.87E-20
DLAT	CD109	0.62235546	1.81E-20
DLAT	UNC50	0.62245515	1.77E-20
DLAT	ZNF45	0.62245756	1.77E-20
DLAT	MLH1	0.62249025	1.76E-20
DLAT	SRGAP2	0.62260339	1.73E-20
DLAT	CNST	0.62305292	1.59E-20
DLAT	CNOT4	0.62320623	1.55E-20
DLAT	RAN	0.62321219	1.55E-20

DLAT	ZNF532	0.62326553	1.53E-20
DLAT	ZEB1	0.62375612	1.4E-20
DLAT	PCNX1	0.62391867	1.36E-20
DLAT	NAPG	0.62400081	1.34E-20
DLAT	HSPA9	0.62404631	1.33E-20
DLAT	MEX3C	0.62431401	1.27E-20
DLAT	ZFP90	0.62471101	1.18E-20
DLAT	KCTD5	0.62474513	1.17E-20
DLAT	NFE2L1	0.6247711	1.17E-20
DLAT	PSMF1	0.62486846	1.15E-20
DLAT	TGFBR1	0.62491986	1.14E-20
DLAT	SNX29	0.62495076	1.13E-20
DLAT	TAF9	0.62499637	1.12E-20
DLAT	UHRF1BP1L	0.62514782	1.09E-20
DLAT	PIGS	0.62533836	1.05E-20
DLAT	HIGD1A	0.62547088	1.03E-20
DLAT	TRUB2	0.62555157	1.01E-20
DLAT	SMARCAL1	0.62569888	9.85E-21
DLAT	PLEKHG1	0.6257923	9.68E-21
DLAT	SYNJ2	0.62586106	9.56E-21
DLAT	EIF3H	0.62588683	9.52E-21
DLAT	MSL2	0.62589736	9.5E-21
DLAT	EIF5A2	0.62594211	9.42E-21
DLAT	COQ7	0.62595449	9.4E-21
DLAT	STARD7	0.62612212	9.12E-21
DLAT	PIGW	0.62616251	9.05E-21
DLAT	DLG5	0.62619295	9E-21
DLAT	STS	0.62626249	8.89E-21
DLAT	VDAC3	0.6262738	8.87E-21
DLAT	MGAT5	0.62639027	8.68E-21
DLAT	VANGL1	0.62640009	8.67E-21
DLAT	UBE2Q2	0.62647212	8.55E-21
DLAT	THUMPD3	0.62648175	8.54E-21
DLAT	NUP37	0.62648935	8.53E-21
DLAT	FAM177A1	0.6266017	8.35E-21
DLAT	BRAP	0.62677857	8.09E-21
DLAT	TMEM19	0.62680327	8.05E-21
DLAT	GASK1B	0.62685936	7.97E-21
DLAT	FKBP1A	0.62712022	7.59E-21
DLAT	PGRMC1	0.62713165	7.58E-21
DLAT	MAFG	0.62739153	7.23E-21
DLAT	GEMIN2	0.62756137	7E-21
DLAT	AGPAT5	0.62782127	6.68E-21
DLAT	RNPS1	0.62783376	6.66E-21
DLAT	ASNSD1	0.62783773	6.66E-21
DLAT	NSMCE2	0.62785178	6.64E-21
DLAT	KIFAP3	0.62786945	6.62E-21
DLAT	YIPF4	0.62793864	6.53E-21
DLAT	HSD17B12	0.62822865	6.19E-21
DLAT	PPP1R3B	0.62828821	6.13E-21
DLAT	SNX7	0.62839912	6E-21
DLAT	LARP4B	0.62844097	5.96E-21
DLAT	ARL6IP1	0.6284985	5.89E-21
DLAT	TMEM230	0.6285907	5.79E-21
DLAT	PIAS3	0.62877474	5.6E-21
DLAT	MID1	0.62882941	5.55E-21
DLAT	REEP5	0.62890928	5.46E-21
DLAT	FOXP1	0.62892516	5.45E-21

DLAT	BUB1	0.62892888	5.44E-21
DLAT	FIP1L1	0.62899062	5.38E-21
DLAT	NR2F2	0.62907479	5.3E-21
DLAT	SMG8	0.62908347	5.29E-21
DLAT	C1GALT1C1	0.62914357	5.23E-21
DLAT	REXO2	0.62926286	5.12E-21
DLAT	CMPK1	0.62952026	4.88E-21
DLAT	GRWD1	0.62974888	4.68E-21
DLAT	SLC38A7	0.62975899	4.67E-21
DLAT	NR3C1	0.62979569	4.64E-21
DLAT	ZNF398	0.62984918	4.59E-21
DLAT	OSTM1	0.63014428	4.35E-21
DLAT	HPS3	0.63041369	4.14E-21
DLAT	MYD88	0.63044953	4.11E-21
DLAT	ARRDC3	0.63059778	4E-21
DLAT	MESD	0.63065121	3.96E-21
DLAT	SS18	0.63067515	3.94E-21
DLAT	GOT2	0.63088069	3.79E-21
DLAT	KIF13A	0.63088501	3.79E-21
DLAT	NDEL1	0.63090522	3.77E-21
DLAT	AK2	0.63091541	3.77E-21
DLAT	ZNF263	0.63097717	3.72E-21
DLAT	JAG1	0.63106222	3.67E-21
DLAT	AC010132.3	0.63109726	3.64E-21
DLAT	DDX19B	0.63120905	3.57E-21
DLAT	MED4	0.631252	3.54E-21
DLAT	TPM4	0.63132777	3.49E-21
DLAT	PRSS23	0.63139556	3.45E-21
DLAT	LRRC41	0.63193157	3.12E-21
DLAT	LIN52	0.63200869	3.07E-21
DLAT	ATP5MF-PTCD1	0.63204752	3.05E-21
DLAT	FBXO7	0.63212953	3E-21
DLAT	ODF2	0.63213538	3E-21
DLAT	HINT3	0.6322884	2.92E-21
DLAT	ZC3HAV1	0.6324749	2.82E-21
DLAT	SEC11A	0.63253746	2.78E-21
DLAT	SLC25A17	0.63263293	2.73E-21
DLAT	MSANTD3-TMEFF1	0.63267096	2.71E-21
DLAT	LIMK1	0.6327045	2.7E-21
DLAT	ELOVL5	0.63273532	2.68E-21
DLAT	HMMR	0.63277311	2.66E-21
DLAT	TTC5	0.6328494	2.63E-21
DLAT	PGK1	0.63293008	2.59E-21
DLAT	PIK3R1	0.63304302	2.53E-21
DLAT	PITPNA	0.63311143	2.5E-21
DLAT	WASHC2A	0.63316932	2.47E-21
DLAT	HNRNPUL2	0.63318718	2.46E-21
DLAT	RAB12	0.63319577	2.46E-21
DLAT	MAP4	0.63322163	2.45E-21
DLAT	ZMIZ1	0.63330058	2.41E-21
DLAT	HNRNPD	0.63334156	2.39E-21
DLAT	DNAJC8	0.63357056	2.29E-21
DLAT	EFCAB11	0.63360107	2.28E-21
DLAT	PGD	0.63369709	2.24E-21
DLAT	UBA1	0.63376254	2.21E-21
DLAT	ECT2	0.63378228	2.2E-21
DLAT	SLC26A2	0.63385004	2.18E-21
DLAT	RFFL	0.63419727	2.04E-21

DLAT	AQR	0.63440126	1.96E-21
DLAT	DCUN1D3	0.63441375	1.96E-21
DLAT	UGCG	0.6346459	1.87E-21
DLAT	UBE2Z	0.63465509	1.87E-21
DLAT	SLC30A5	0.63465616	1.87E-21
DLAT	MGME1	0.63473718	1.84E-21
DLAT	GTPBP4	0.6349294	1.78E-21
DLAT	PPHLN1	0.6350569	1.73E-21
DLAT	SEH1L	0.63512286	1.71E-21
DLAT	GATAD2A	0.63518422	1.69E-21
DLAT	SLC6A6	0.63520094	1.69E-21
DLAT	ABCB10	0.63523245	1.68E-21
DLAT	DHFR	0.6354698	1.6E-21
DLAT	JOSD1	0.63550191	1.59E-21
DLAT	ITSN1	0.63554746	1.58E-21
DLAT	ASAP2	0.63560632	1.56E-21
DLAT	PPT1	0.63565551	1.55E-21
DLAT	CAPN2	0.63571105	1.53E-21
DLAT	TMED10	0.63578103	1.51E-21
DLAT	MUL1	0.63583472	1.5E-21
DLAT	SORL1	0.63607987	1.43E-21
DLAT	RAB11A	0.63608016	1.43E-21
DLAT	MAIP1	0.63608343	1.43E-21
DLAT	DHX9	0.63641309	1.34E-21
DLAT	IFNGR1	0.63642802	1.34E-21
DLAT	F2R	0.63652363	1.31E-21
DLAT	LASP1	0.63653252	1.31E-21
DLAT	HERC3	0.63657157	1.3E-21
DLAT	GLOD4	0.63679672	1.25E-21
DLAT	MYO6	0.63681564	1.24E-21
DLAT	UBAP2L	0.63691624	1.22E-21
DLAT	LRIF1	0.63692706	1.22E-21
DLAT	CRIPT	0.63698636	1.2E-21
DLAT	SUFU	0.63701534	1.2E-21
DLAT	NRBP1	0.63715108	1.17E-21
DLAT	NOTCH2	0.6373431	1.12E-21
DLAT	SPATS2	0.6374276	1.11E-21
DLAT	ZNF134	0.63753342	1.08E-21
DLAT	RTL6	0.63766498	1.06E-21
DLAT	SNRPB2	0.63861108	8.82E-22
DLAT	RNGTT	0.63867024	8.72E-22
DLAT	ACER3	0.63889539	8.35E-22
DLAT	TRAF3	0.63896105	8.25E-22
DLAT	MRPL15	0.63912182	8E-22
DLAT	VGLL4	0.63921441	7.86E-22
DLAT	BCL9	0.63927212	7.77E-22
DLAT	WDR20	0.63929665	7.74E-22
DLAT	CKAP2L	0.63930743	7.72E-22
DLAT	MTCH2	0.63943797	7.53E-22
DLAT	TFRC	0.63957685	7.33E-22
DLAT	IFIH1	0.63964385	7.24E-22
DLAT	LTBP1	0.6400438	6.7E-22
DLAT	DNAL1	0.64008133	6.65E-22
DLAT	MACF1	0.64019494	6.51E-22
DLAT	LRRFIP2	0.64029396	6.39E-22
DLAT	IRF2BP2	0.6403438	6.33E-22
DLAT	RUFY1	0.6403458	6.32E-22
DLAT	RND3	0.64052639	6.11E-22

DLAT	FRYL	0.64068975	5.92E-22
DLAT	ZNF468	0.64079398	5.8E-22
DLAT	FRS2	0.64091614	5.66E-22
DLAT	PCDH7	0.64093919	5.64E-22
DLAT	ERMP1	0.64096657	5.61E-22
DLAT	SP2	0.64099275	5.58E-22
DLAT	GTPBP10	0.64100528	5.57E-22
DLAT	DPYD	0.64135032	5.21E-22
DLAT	PDHB	0.64137535	5.18E-22
DLAT	EDC3	0.64140186	5.16E-22
DLAT	FNBP1L	0.6414068	5.15E-22
DLAT	CASP3	0.64147984	5.08E-22
DLAT	TM9SF3	0.64165674	4.91E-22
DLAT	CNOT2	0.64168477	4.88E-22
DLAT	SETX	0.6417058	4.86E-22
DLAT	ADD3	0.64174057	4.83E-22
DLAT	STIL	0.64178263	4.79E-22
DLAT	NKIRAS2	0.64191944	4.67E-22
DLAT	TMEM183A	0.64199678	4.6E-22
DLAT	ETFA	0.64211162	4.5E-22
DLAT	ADO	0.64239206	4.26E-22
DLAT	FKBP9	0.64241463	4.24E-22
DLAT	TM9SF4	0.64245478	4.21E-22
DLAT	MYO1E	0.64245799	4.2E-22
DLAT	ZNF131	0.6426315	4.06E-22
DLAT	LAP3	0.64266796	4.04E-22
DLAT	GINS4	0.64279687	3.94E-22
DLAT	GCC1	0.64283342	3.91E-22
DLAT	SEMA3C	0.64285699	3.89E-22
DLAT	LRRC8D	0.64292838	3.84E-22
DLAT	TTC1	0.64296498	3.81E-22
DLAT	NUDCD2	0.64310495	3.71E-22
DLAT	PEA15	0.64322556	3.62E-22
DLAT	SNX27	0.64334289	3.54E-22
DLAT	KATNAL1	0.64361654	3.36E-22
DLAT	TADA1	0.64362558	3.35E-22
DLAT	CFAP20	0.64366831	3.32E-22
DLAT	ARPC2	0.64368102	3.31E-22
DLAT	INTS12	0.64376834	3.26E-22
DLAT	COL4A1	0.64386424	3.2E-22
DLAT	FSTL1	0.64395752	3.14E-22
DLAT	RAD51B	0.64400574	3.11E-22
DLAT	BRIX1	0.64437108	2.9E-22
DLAT	LINS1	0.64463416	2.75E-22
DLAT	MYH9	0.64471228	2.71E-22
DLAT	SGCB	0.64476641	2.68E-22
DLAT	GALNT1	0.64486706	2.63E-22
DLAT	RER1	0.6449605	2.58E-22
DLAT	ENY2	0.64503469	2.54E-22
DLAT	FAM3C	0.64521601	2.45E-22
DLAT	RPA1	0.64527224	2.43E-22
DLAT	RAB23	0.64530811	2.41E-22
DLAT	EIF2B2	0.64548642	2.33E-22
DLAT	IDH1	0.64564451	2.26E-22
DLAT	CYLD	0.64567276	2.24E-22
DLAT	ITGA1	0.64602545	2.09E-22
DLAT	HIVEP1	0.64634871	1.96E-22
DLAT	UBE2E3	0.64643403	1.93E-22

DLAT	GCLM	0.64651066	1.9E-22
DLAT	CCDC6	0.64654816	1.89E-22
DLAT	WBP11	0.64665119	1.85E-22
DLAT	RTF2	0.64666963	1.84E-22
DLAT	CDC42SE2	0.64692884	1.75E-22
DLAT	DIPK2A	0.64702606	1.72E-22
DLAT	DOCK1	0.64709096	1.7E-22
DLAT	GNPDA1	0.6471193	1.69E-22
DLAT	UBE2E1	0.64714836	1.68E-22
DLAT	GRSF1	0.64717885	1.67E-22
DLAT	MRPL44	0.64723961	1.65E-22
DLAT	INSIG2	0.64726004	1.64E-22
DLAT	SART3	0.64726872	1.64E-22
DLAT	DDX27	0.6474002	1.6E-22
DLAT	HARS2	0.64746953	1.57E-22
DLAT	ATG7	0.6475862	1.54E-22
DLAT	ETS1	0.64772495	1.5E-22
DLAT	GRPEL1	0.64776072	1.49E-22
DLAT	RNF24	0.64778561	1.48E-22
DLAT	CISD2	0.64809087	1.39E-22
DLAT	CSE1L	0.64827469	1.34E-22
DLAT	ELK1	0.64839588	1.31E-22
DLAT	B3GALNT1	0.64868778	1.24E-22
DLAT	RNF170	0.64871225	1.23E-22
DLAT	INTS14	0.64873483	1.23E-22
DLAT	ABL1	0.6487441	1.22E-22
DLAT	PKD2	0.64878586	1.21E-22
DLAT	TRIO	0.64879587	1.21E-22
DLAT	PARP8	0.64879987	1.21E-22
DLAT	PHACTR2	0.64896479	1.17E-22
DLAT	SHC1	0.64924552	1.11E-22
DLAT	NUP62	0.64924761	1.11E-22
DLAT	STAT1	0.64927354	1.1E-22
DLAT	PRKD3	0.649659	1.02E-22
DLAT	SH3PXD2A	0.64968912	1.01E-22
DLAT	ARPC4	0.64970892	1.01E-22
DLAT	ZFYVE1	0.64987853	9.76E-23
DLAT	NIPA1	0.65004352	9.44E-23
DLAT	STAMBP	0.65009856	9.34E-23
DLAT	ZKSCAN5	0.6501774	9.2E-23
DLAT	CHM	0.65032787	8.92E-23
DLAT	POLR2K	0.65044769	8.71E-23
DLAT	DIP2B	0.6505928	8.46E-23
DLAT	PCGF5	0.65064157	8.38E-23
DLAT	ARNTL2	0.65065472	8.36E-23
DLAT	ATP6AP2	0.65081284	8.1E-23
DLAT	SLC16A1	0.65083383	8.06E-23
DLAT	CAMKK2	0.65091758	7.93E-23
DLAT	MCM6	0.65095587	7.87E-23
DLAT	BFAR	0.65108171	7.67E-23
DLAT	EIF2S2	0.65137637	7.23E-23
DLAT	RAE1	0.65138823	7.22E-23
DLAT	AKIRIN1	0.65153711	7E-23
DLAT	SKIL	0.65163895	6.86E-23
DLAT	CTNNA1	0.65184419	6.58E-23
DLAT	SLC25A13	0.65187093	6.55E-23
DLAT	OSMR	0.65202973	6.34E-23
DLAT	RCOR1	0.6521663	6.17E-23

DLAT	VDAC2	0.65220496	6.12E-23
DLAT	SIAH1	0.65222191	6.1E-23
DLAT	PAK1IP1	0.6522536	6.06E-23
DLAT	CD46	0.65239248	5.9E-23
DLAT	OAT	0.6524019	5.89E-23
DLAT	TRERF1	0.65254237	5.72E-23
DLAT	ACTL6A	0.65256753	5.69E-23
DLAT	CIPC	0.6526502	5.6E-23
DLAT	MTFMT	0.65269832	5.54E-23
DLAT	URI1	0.65286554	5.36E-23
DLAT	SYAP1	0.65302992	5.19E-23
DLAT	SCFD2	0.65311829	5.09E-23
DLAT	SINHCAF	0.6531862	5.02E-23
DLAT	C8orf33	0.65318867	5.02E-23
DLAT	IL1RAP	0.65318906	5.02E-23
DLAT	ATP11C	0.65344079	4.77E-23
DLAT	AHCYL1	0.65346843	4.75E-23
DLAT	VEZF1	0.65365137	4.57E-23
DLAT	ARFGEF1	0.65373838	4.49E-23
DLAT	FBXO5	0.65379736	4.44E-23
DLAT	HECA	0.65392313	4.33E-23
DLAT	RNF19B	0.65410083	4.18E-23
DLAT	KLF6	0.65417714	4.11E-23
DLAT	ABHD13	0.65417827	4.11E-23
DLAT	TMEM248	0.65434008	3.98E-23
DLAT	RMDN1	0.65448683	3.86E-23
DLAT	ACTN1	0.65453293	3.83E-23
DLAT	NSFL1C	0.65469849	3.7E-23
DLAT	LYRM2	0.65483506	3.6E-23
DLAT	ZCCHC9	0.65496065	3.51E-23
DLAT	CRTC3	0.65496544	3.5E-23
DLAT	ATP6V1D	0.65514733	3.38E-23
DLAT	ACTN4	0.65515826	3.37E-23
DLAT	SNX24	0.65520194	3.34E-23
DLAT	STK38L	0.65524423	3.31E-23
DLAT	RTN3	0.65529602	3.28E-23
DLAT	DARS2	0.65543913	3.18E-23
DLAT	EFL1	0.65552461	3.13E-23
DLAT	ERGIC1	0.6557191	3E-23
DLAT	LRRFIP1	0.65581008	2.95E-23
DLAT	PTGES3	0.65581282	2.95E-23
DLAT	TUT7	0.65592691	2.88E-23
DLAT	OTULIN	0.65624867	2.7E-23
DLAT	WEE1	0.65627636	2.68E-23
DLAT	LBR	0.6563581	2.64E-23
DLAT	FBXO42	0.65657856	2.52E-23
DLAT	ASAH1	0.65667046	2.47E-23
DLAT	KIF3B	0.65672361	2.45E-23
DLAT	ECHDC1	0.65677346	2.42E-23
DLAT	SAE1	0.65685449	2.38E-23
DLAT	GINM1	0.65693428	2.34E-23
DLAT	TGFBR2	0.65706996	2.28E-23
DLAT	ASH2L	0.65712781	2.25E-23
DLAT	TSG101	0.65722479	2.21E-23
DLAT	TFG	0.65736215	2.15E-23
DLAT	SDHB	0.65749285	2.09E-23
DLAT	TYW1	0.65755818	2.06E-23
DLAT	OTUD1	0.6576432	2.03E-23

DLAT	CHMP1B	0.65775508	1.98E-23
DLAT	SKI	0.65786895	1.93E-23
DLAT	KDM5B	0.65788875	1.93E-23
DLAT	SNX3	0.65794665	1.9E-23
DLAT	GFPT1	0.65798943	1.89E-23
DLAT	TRRAP	0.65805993	1.86E-23
DLAT	TMCO1	0.65809413	1.85E-23
DLAT	TFE3	0.65860645	1.66E-23
DLAT	PARP4	0.65865085	1.65E-23
DLAT	HOMER1	0.65870227	1.63E-23
DLAT	SOCS6	0.65870372	1.63E-23
DLAT	CIR1	0.65877818	1.6E-23
DLAT	B3GNT5	0.65895449	1.55E-23
DLAT	GMPS	0.65899087	1.54E-23
DLAT	PELI1	0.65901672	1.53E-23
DLAT	OSTF1	0.65904777	1.52E-23
DLAT	ATXN3	0.65944572	1.4E-23
DLAT	TRIM69	0.65945725	1.39E-23
DLAT	MED21	0.65962717	1.35E-23
DLAT	BUB3	0.66006127	1.23E-23
DLAT	ITGA2	0.6601344	1.21E-23
DLAT	TTL	0.66017691	1.2E-23
DLAT	NUDT3	0.66020859	1.19E-23
DLAT	CREB3L2	0.66026174	1.18E-23
DLAT	SMS	0.66027656	1.18E-23
DLAT	KBTBD4	0.66061474	1.1E-23
DLAT	MTA2	0.66071342	1.07E-23
DLAT	KIF16B	0.6607807	1.06E-23
DLAT	KIAA0232	0.66080822	1.05E-23
DLAT	TPP2	0.6608156	1.05E-23
DLAT	PRIM2	0.66084879	1.04E-23
DLAT	KNOP1	0.66094675	1.02E-23
DLAT	RALA	0.66101124	1.01E-23
DLAT	MAP3K20	0.66110162	9.91E-24
DLAT	DCTD	0.6611062	9.9E-24
DLAT	STK26	0.66123164	9.65E-24
DLAT	LACTB	0.66133245	9.45E-24
DLAT	PLRG1	0.6618521	8.48E-24
DLAT	CXorf56	0.66191752	8.36E-24
DLAT	KCTD21	0.66205295	8.13E-24
DLAT	FBXO8	0.66213797	7.99E-24
DLAT	CDK17	0.66222818	7.84E-24
DLAT	MEAF6	0.66226918	7.77E-24
DLAT	ZNRF2	0.6624364	7.5E-24
DLAT	GTDC1	0.66277617	6.99E-24
DLAT	DYNLT3	0.66278136	6.98E-24
DLAT	KTN1	0.66304185	6.61E-24
DLAT	RHEB	0.66352009	5.98E-24
DLAT	PHACTR4	0.66364105	5.83E-24
DLAT	TMEM209	0.66368164	5.78E-24
DLAT	METTL21A	0.66368294	5.78E-24
DLAT	LRCH1	0.66373544	5.71E-24
DLAT	ANP32A	0.66382867	5.6E-24
DLAT	SH3BGRL	0.66383203	5.6E-24
DLAT	SPG21	0.66384494	5.58E-24
DLAT	SWAP70	0.66395485	5.46E-24
DLAT	UQCRC2	0.66398404	5.42E-24
DLAT	MTERF3	0.66400843	5.39E-24

DLAT	HIBADH	0.66403592	5.36E-24
DLAT	NFYA	0.66412232	5.27E-24
DLAT	ARL6IP5	0.66412912	5.26E-24
DLAT	CLDND1	0.66420634	5.17E-24
DLAT	RMND5A	0.66424861	5.13E-24
DLAT	SMIM13	0.66433585	5.03E-24
DLAT	CASP2	0.66439077	4.98E-24
DLAT	CRCP	0.66473194	4.63E-24
DLAT	SPTSSA	0.66477268	4.59E-24
DLAT	KLHL5	0.66531463	4.09E-24
DLAT	EMC3	0.66541793	4.01E-24
DLAT	SUMO1	0.66542371	4E-24
DLAT	CIP2A	0.66549331	3.94E-24
DLAT	NID1	0.66554763	3.9E-24
DLAT	ENDOD1	0.66554963	3.9E-24
DLAT	OGDH	0.66559527	3.86E-24
DLAT	NUP107	0.66561337	3.84E-24
DLAT	C9orf78	0.66562935	3.83E-24
DLAT	ZFAND5	0.66563346	3.83E-24
DLAT	GMCL1	0.66574099	3.74E-24
DLAT	CDC42SE1	0.66579816	3.7E-24
DLAT	E2F3	0.66592848	3.6E-24
DLAT	CASK	0.66593974	3.59E-24
DLAT	ADIPOR1	0.66605334	3.5E-24
DLAT	CTBP2	0.66616723	3.42E-24
DLAT	SUMO3	0.66626457	3.35E-24
DLAT	CALM2	0.66633477	3.3E-24
DLAT	TOPORS	0.66634027	3.29E-24
DLAT	MZT1	0.66642188	3.24E-24
DLAT	PRKAG2	0.66647893	3.2E-24
DLAT	ACTR6	0.66661839	3.11E-24
DLAT	DNM1L	0.66666431	3.08E-24
DLAT	NUDT21	0.66668011	3.07E-24
DLAT	CASP8	0.66668775	3.06E-24
DLAT	RAB35	0.66672724	3.03E-24
DLAT	STX6	0.6667606	3.01E-24
DLAT	LRRC42	0.66690081	2.92E-24
DLAT	RBMS1	0.66691498	2.92E-24
DLAT	MAP3K13	0.6669444	2.9E-24
DLAT	PRPF38A	0.66738228	2.64E-24
DLAT	RAPH1	0.66754478	2.55E-24
DLAT	PGAM1	0.6675953	2.52E-24
DLAT	GPATCH11	0.66761858	2.51E-24
DLAT	CSTF2T	0.66762631	2.51E-24
DLAT	SCP2	0.66773741	2.45E-24
DLAT	CAPZB	0.66775789	2.44E-24
DLAT	ATP10D	0.66793617	2.34E-24
DLAT	VHL	0.66797475	2.33E-24
DLAT	ATP5PB	0.66806916	2.28E-24
DLAT	SERTAD2	0.66828727	2.18E-24
DLAT	DYRK1A	0.66833934	2.15E-24
DLAT	AMMECR1	0.66837634	2.13E-24
DLAT	HNRNPUL1	0.6685264	2.07E-24
DLAT	NAGA	0.66867792	2E-24
DLAT	FOXN3	0.66883825	1.93E-24
DLAT	CRLF3	0.66907533	1.84E-24
DLAT	WIPF2	0.66930041	1.75E-24
DLAT	PLEKHF2	0.66932345	1.74E-24

DLAT	ANKMY2	0.6695539	1.66E-24
DLAT	RBM22	0.66957024	1.65E-24
DLAT	EPS8	0.66983833	1.56E-24
DLAT	C14orf119	0.66994126	1.53E-24
DLAT	UNG	0.67004647	1.49E-24
DLAT	CHMP5	0.67007803	1.48E-24
DLAT	SBF2	0.67008064	1.48E-24
DLAT	NUP50	0.67010759	1.47E-24
DLAT	PTPRG	0.67016335	1.45E-24
DLAT	ZFAND6	0.67017045	1.45E-24
DLAT	SEC22A	0.67030675	1.41E-24
DLAT	HSP90AA1	0.67040118	1.38E-24
DLAT	CBX1	0.67042417	1.37E-24
DLAT	PGM2L1	0.6704954	1.35E-24
DLAT	RCC1	0.67072338	1.29E-24
DLAT	MYOF	0.67092339	1.23E-24
DLAT	SNX18	0.67141972	1.11E-24
DLAT	ZC2HC1A	0.67150157	1.09E-24
DLAT	CHSY1	0.67150296	1.09E-24
DLAT	CD2AP	0.67167481	1.05E-24
DLAT	UBE2I	0.67171739	1.04E-24
DLAT	NBR1	0.67178758	1.02E-24
DLAT	LHFPL2	0.67185136	1.01E-24
DLAT	RBBP9	0.67188696	1E-24
DLAT	IFT57	0.67199432	9.78E-25
DLAT	GRPEL2	0.67202757	9.71E-25
DLAT	STARD3NL	0.67205381	9.66E-25
DLAT	RPRD2	0.67205561	9.65E-25
DLAT	DAZAP2	0.67247563	8.81E-25
DLAT	EAPP	0.67258257	8.61E-25
DLAT	TSC22D2	0.67258552	8.6E-25
DLAT	PCBP1	0.67265566	8.47E-25
DLAT	FBXO22	0.67277472	8.26E-25
DLAT	SCAMP1	0.67279554	8.22E-25
DLAT	CEP170	0.67285824	8.11E-25
DLAT	PTP4A1	0.67289198	8.05E-25
DLAT	DEDD	0.67293607	7.97E-25
DLAT	TOB2	0.67299333	7.87E-25
DLAT	SLC35A4	0.67307584	7.73E-25
DLAT	TCAF1	0.67324131	7.46E-25
DLAT	MBTPS1	0.67342106	7.17E-25
DLAT	IFT52	0.67344679	7.13E-25
DLAT	GPATCH2	0.67354334	6.98E-25
DLAT	BROX	0.67360709	6.89E-25
DLAT	SERINC1	0.6739443	6.4E-25
DLAT	PSMC6	0.6739971	6.32E-25
DLAT	ACSL3	0.67427818	5.95E-25
DLAT	MFSD11	0.67442285	5.76E-25
DLAT	RBMS2	0.67453692	5.62E-25
DLAT	SMG7	0.6749586	5.12E-25
DLAT	PAQR3	0.67539526	4.65E-25
DLAT	ELAVL1	0.67548709	4.56E-25
DLAT	YPEL5	0.67549904	4.55E-25
DLAT	NUCKS1	0.67555975	4.49E-25
DLAT	VIPAS39	0.67571418	4.34E-25
DLAT	ABCC1	0.67597939	4.09E-25
DLAT	WAPL	0.67609281	3.99E-25
DLAT	DCAF12	0.67610314	3.98E-25

DLAT	MASTL	0.67616108	3.93E-25
DLAT	DEGS1	0.67627216	3.83E-25
DLAT	TES	0.67629606	3.81E-25
DLAT	C1GALT1	0.67630899	3.8E-25
DLAT	DCBLD1	0.67639301	3.73E-25
DLAT	GPBP1L1	0.67652088	3.63E-25
DLAT	NAMPT	0.67667234	3.51E-25
DLAT	AP1S3	0.67671017	3.48E-25
DLAT	DENR	0.67677017	3.44E-25
DLAT	HSPA13	0.67678657	3.42E-25
DLAT	RFC3	0.67679023	3.42E-25
DLAT	RAB28	0.6768373	3.38E-25
DLAT	C2CD3	0.67685315	3.37E-25
DLAT	SNX9	0.67697363	3.28E-25
DLAT	DDX50	0.6770392	3.24E-25
DLAT	TCF20	0.67724191	3.09E-25
DLAT	PTPN4	0.67729725	3.06E-25
DLAT	SUMO2	0.67733385	3.03E-25
DLAT	TM9SF2	0.67734684	3.02E-25
DLAT	AP3S1	0.67734753	3.02E-25
DLAT	UBE2H	0.67746567	2.94E-25
DLAT	TMLHE	0.6775163	2.91E-25
DLAT	RAI14	0.67772806	2.78E-25
DLAT	KCTD20	0.67773139	2.78E-25
DLAT	LACTB2	0.67776675	2.75E-25
DLAT	LACC1	0.67783484	2.71E-25
DLAT	KLF3	0.67791195	2.67E-25
DLAT	ABCF2	0.67791389	2.67E-25
DLAT	EXOC1	0.67810233	2.56E-25
DLAT	RSU1	0.67815654	2.53E-25
DLAT	RALBP1	0.67824293	2.48E-25
DLAT	TTC26	0.67833	2.43E-25
DLAT	DRAM2	0.67845385	2.36E-25
DLAT	KDM1B	0.67849511	2.34E-25
DLAT	COPS5	0.67859163	2.29E-25
DLAT	SLU7	0.67874209	2.22E-25
DLAT	CACYBP	0.67883199	2.17E-25
DLAT	CDK2	0.67899389	2.1E-25
DLAT	CDK19	0.67901531	2.09E-25
DLAT	SH3KBP1	0.67905764	2.07E-25
DLAT	ENOPH1	0.67912237	2.04E-25
DLAT	ISY1-RAB43	0.67912908	2.03E-25
DLAT	ASPH	0.67927828	1.97E-25
DLAT	WDR12	0.67950119	1.87E-25
DLAT	GNPDA2	0.67971575	1.78E-25
DLAT	ADAT1	0.67993349	1.7E-25
DLAT	SPTLC2	0.68001793	1.67E-25
DLAT	SH3D19	0.68006341	1.65E-25
DLAT	PEX26	0.68025138	1.58E-25
DLAT	POT1	0.6802638	1.58E-25
DLAT	PPP1R15B	0.68049227	1.5E-25
DLAT	ZC3H11A	0.68052761	1.49E-25
DLAT	SLC35A3	0.68052994	1.49E-25
DLAT	LYN	0.6806602	1.44E-25
DLAT	PCNX4	0.68074174	1.42E-25
DLAT	EAF1	0.68075082	1.41E-25
DLAT	MRPL42	0.68084806	1.38E-25
DLAT	LIN9	0.68088488	1.37E-25

DLAT	GGPS1	0.68094898	1.35E-25
DLAT	TEX10	0.68114523	1.29E-25
DLAT	ARF4	0.68125128	1.26E-25
DLAT	ELK3	0.68133221	1.24E-25
DLAT	DPY19L1	0.68134176	1.24E-25
DLAT	QKI	0.6815654	1.18E-25
DLAT	SNX1	0.68160085	1.17E-25
DLAT	CHMP3	0.68168716	1.15E-25
DLAT	SC5D	0.68209396	1.04E-25
DLAT	DTX3L	0.68220544	1.02E-25
DLAT	SLC31A1	0.68228448	1E-25
DLAT	SBDS	0.68241825	9.71E-26
DLAT	ERAP1	0.68245983	9.62E-26
DLAT	ZNF267	0.68255802	9.41E-26
DLAT	NEMP2	0.68256843	9.39E-26
DLAT	AHR	0.68265395	9.21E-26
DLAT	CDK6	0.68267194	9.17E-26
DLAT	VAPB	0.6826867	9.14E-26
DLAT	C4orf3	0.68289339	8.72E-26
DLAT	CERS2	0.68293228	8.64E-26
DLAT	KATNBL1	0.68313159	8.26E-26
DLAT	BRD3	0.68315904	8.21E-26
DLAT	DYRK2	0.68343243	7.72E-26
DLAT	NUP98	0.68355209	7.51E-26
DLAT	PALLD	0.68358971	7.45E-26
DLAT	LRP10	0.68376841	7.15E-26
DLAT	GLE1	0.68378412	7.12E-26
DLAT	HFE	0.68383822	7.04E-26
DLAT	STIM1	0.68384568	7.02E-26
DLAT	CUL1	0.68389895	6.94E-26
DLAT	GPN1	0.68395019	6.86E-26
DLAT	SAR1B	0.68402465	6.74E-26
DLAT	NSRP1	0.68453004	6.01E-26
DLAT	TAF13	0.68468081	5.81E-26
DLAT	RFX5	0.68472757	5.75E-26
DLAT	AKT1	0.68495714	5.45E-26
DLAT	ARL14EP	0.68497093	5.43E-26
DLAT	TMEM43	0.68534112	4.99E-26
DLAT	KCTD10	0.6854036	4.92E-26
DLAT	GBE1	0.68541186	4.91E-26
DLAT	BLOC1S5	0.68554363	4.77E-26
DLAT	MAP1LC3B	0.68571016	4.59E-26
DLAT	METTL2B	0.68578469	4.51E-26
DLAT	CPT1A	0.68582591	4.47E-26
DLAT	HEATR5A	0.68582677	4.47E-26
DLAT	PSMC1	0.6859061	4.39E-26
DLAT	EXOC4	0.68593759	4.36E-26
DLAT	INO80	0.68617159	4.13E-26
DLAT	SRGAP2C	0.68635244	3.96E-26
DLAT	SYNJ2BP	0.68638496	3.93E-26
DLAT	TMEM182	0.68641149	3.91E-26
DLAT	SLC17A5	0.68653576	3.8E-26
DLAT	ARMC10	0.68698437	3.42E-26
DLAT	ELF4	0.68715163	3.29E-26
DLAT	EXOC2	0.68732762	3.16E-26
DLAT	PEAK1	0.68740633	3.11E-26
DLAT	ZDHHC9	0.68744875	3.08E-26
DLAT	GLUD1	0.6875043	3.04E-26

DLAT	METTL8	0.68762075	2.96E-26
DLAT	TRAK2	0.68805523	2.67E-26
DLAT	NFKB1	0.68806062	2.67E-26
DLAT	SAMD8	0.68810547	2.64E-26
DLAT	AGAP1	0.68812837	2.63E-26
DLAT	FGFR1OP2	0.68814132	2.62E-26
DLAT	SOS2	0.68853762	2.39E-26
DLAT	PRPF18	0.68856408	2.38E-26
DLAT	SSR1	0.68868342	2.31E-26
DLAT	ADK	0.68885127	2.22E-26
DLAT	CCDC43	0.68894688	2.17E-26
DLAT	CCDC47	0.68913709	2.08E-26
DLAT	DBF4	0.68922532	2.04E-26
DLAT	FBXL5	0.68937276	1.97E-26
DLAT	GLYR1	0.68941373	1.95E-26
DLAT	FBXO34	0.68950066	1.91E-26
DLAT	RRM1	0.68961168	1.86E-26
DLAT	OCIAD1	0.68961948	1.86E-26
DLAT	CPOX	0.6897675	1.8E-26
DLAT	APEX1	0.6898179	1.78E-26
DLAT	WASHC5	0.68994128	1.73E-26
DLAT	AEBP2	0.68998054	1.71E-26
DLAT	UTP14C	0.68999324	1.7E-26
DLAT	CALM1	0.69009332	1.67E-26
DLAT	MIS18BP1	0.69015241	1.64E-26
DLAT	GABPB1	0.6902216	1.62E-26
DLAT	CKAP2	0.69039447	1.55E-26
DLAT	IMMT	0.69047586	1.52E-26
DLAT	MBNL1	0.69052129	1.51E-26
DLAT	RAP1B	0.69072098	1.44E-26
DLAT	COMMD8	0.69088313	1.38E-26
DLAT	FAM118B	0.69095536	1.36E-26
DLAT	PSMD2	0.69095991	1.36E-26
DLAT	KAT7	0.69134357	1.24E-26
DLAT	CIAPIN1	0.6913983	1.23E-26
DLAT	PTP4A2	0.69154261	1.19E-26
DLAT	L3MBTL3	0.691558	1.18E-26
DLAT	ZNF766	0.69171976	1.14E-26
DLAT	LAPTM4A	0.69178096	1.12E-26
DLAT	DCAF7	0.69185444	1.1E-26
DLAT	TPM3	0.69195782	1.08E-26
DLAT	PA2G4	0.69210735	1.04E-26
DLAT	ZNF827	0.69215777	1.03E-26
DLAT	KDM4A	0.6922994	9.93E-27
DLAT	RAB9A	0.69234092	9.83E-27
DLAT	BTBD10	0.69249049	9.49E-27
DLAT	ATF7IP	0.69276342	8.9E-27
DLAT	SERINC5	0.69278592	8.85E-27
DLAT	SUPT16H	0.69285094	8.72E-27
DLAT	CYCS	0.69295459	8.51E-27
DLAT	LAMP2	0.69304574	8.33E-27
DLAT	ARHGAP21	0.69320082	8.03E-27
DLAT	RAP2A	0.69326062	7.92E-27
DLAT	SNX5	0.69349821	7.48E-27
DLAT	ARPC5	0.69350952	7.46E-27
DLAT	IRF2	0.69353749	7.41E-27
DLAT	GALC	0.69365536	7.21E-27
DLAT	PDP1	0.69380359	6.96E-27

DLAT	RAB5B	0.6939157	6.78E-27
DLAT	MPZL1	0.69465027	5.69E-27
DLAT	USP6NL	0.69469697	5.63E-27
DLAT	VCL	0.69509191	5.13E-27
DLAT	PTPRE	0.69527596	4.91E-27
DLAT	RAB5C	0.69528737	4.89E-27
DLAT	MPHOSPH6	0.69532018	4.86E-27
DLAT	SGMS2	0.69555032	4.6E-27
DLAT	STIP1	0.69555246	4.59E-27
DLAT	PLAA	0.69557786	4.57E-27
DLAT	TUBA1B	0.69560337	4.54E-27
DLAT	TAF5L	0.69564425	4.49E-27
DLAT	MRPS10	0.69579938	4.33E-27
DLAT	ERGIC2	0.69587828	4.25E-27
DLAT	TANC2	0.69618539	3.95E-27
DLAT	POLE3	0.69620334	3.93E-27
DLAT	CEP41	0.69621126	3.92E-27
DLAT	ZWILCH	0.69625016	3.89E-27
DLAT	SLK	0.69630151	3.84E-27
DLAT	SNX12	0.69640085	3.75E-27
DLAT	TRAM1	0.69641624	3.74E-27
DLAT	SPRTN	0.69645227	3.7E-27
DLAT	GALK2	0.69651982	3.65E-27
DLAT	POGK	0.69658565	3.59E-27
DLAT	AP2B1	0.69660801	3.57E-27
DLAT	WDR89	0.69666282	3.52E-27
DLAT	CUL4A	0.69681716	3.39E-27
DLAT	ARL2BP	0.69720631	3.09E-27
DLAT	VTI1A	0.69729808	3.02E-27
DLAT	LEO1	0.69752068	2.87E-27
DLAT	LPP	0.69797664	2.57E-27
DLAT	DPF2	0.69803564	2.53E-27
DLAT	DCAF13	0.69804293	2.53E-27
DLAT	TSPAN14	0.6980783	2.51E-27
DLAT	STAT3	0.6981737	2.45E-27
DLAT	UBLCP1	0.69820169	2.43E-27
DLAT	LIMS1	0.69843745	2.3E-27
DLAT	PPTC7	0.69865013	2.18E-27
DLAT	BBS7	0.69868455	2.17E-27
DLAT	PTPRA	0.69876535	2.12E-27
DLAT	OXSR1	0.6988229	2.09E-27
DLAT	PRKAR1A	0.69885209	2.08E-27
DLAT	HIVEP2	0.69891743	2.05E-27
DLAT	CBX3	0.69902178	2E-27
DLAT	GOLIM4	0.69924753	1.89E-27
DLAT	PIGX	0.69929486	1.87E-27
DLAT	MFSD1	0.69936838	1.84E-27
DLAT	APOL6	0.6994637	1.79E-27
DLAT	TBK1	0.69956156	1.75E-27
DLAT	GNPTAB	0.69958141	1.74E-27
DLAT	ARL1	0.6997454	1.67E-27
DLAT	MAP3K7	0.69976623	1.67E-27
DLAT	CLIC4	0.70011655	1.53E-27
DLAT	IGF2R	0.70047923	1.4E-27
DLAT	IST1	0.70051149	1.39E-27
DLAT	SUSD6	0.70062711	1.35E-27
DLAT	RIT1	0.70064819	1.34E-27
DLAT	GSTCD	0.70066653	1.34E-27

DLAT	BMT2	0.70074162	1.31E-27
DLAT	ZNF644	0.70108586	1.21E-27
DLAT	MEAK7	0.70147937	1.1E-27
DLAT	PDE12	0.70161458	1.06E-27
DLAT	KLHL2	0.701661	1.05E-27
DLAT	RNF26	0.70178781	1.02E-27
DLAT	ZNFX1	0.70183629	1.01E-27
DLAT	AP003108.2	0.70185368	1E-27
DLAT	FEM1C	0.70189358	9.92E-28
DLAT	PTPN1	0.70194689	9.79E-28
DLAT	LNPEP	0.7020357	9.58E-28
DLAT	UBE2K	0.70216648	9.28E-28
DLAT	CYB5B	0.70221363	9.17E-28
DLAT	MBNL2	0.70230205	8.98E-28
DLAT	MPHOSPH10	0.70234581	8.88E-28
DLAT	SMURF2	0.70243686	8.68E-28
DLAT	PHF20	0.70259806	8.35E-28
DLAT	TM2D1	0.70263976	8.26E-28
DLAT	DNAJC14	0.70278617	7.97E-28
DLAT	TMTC2	0.70280821	7.93E-28
DLAT	DPM1	0.70286342	7.82E-28
DLAT	FAM114A1	0.70298622	7.59E-28
DLAT	SUCLA2	0.70328965	7.04E-28
DLAT	RAB8B	0.70332779	6.98E-28
DLAT	ZDHHC20	0.70339808	6.86E-28
DLAT	NCAPG2	0.70346484	6.74E-28
DLAT	HIF1A	0.70353061	6.64E-28
DLAT	METTL14	0.70361262	6.5E-28
DLAT	APBB2	0.70362553	6.48E-28
DLAT	ATG3	0.70362647	6.48E-28
DLAT	KDM3B	0.7036345	6.47E-28
DLAT	RNF20	0.70377984	6.24E-28
DLAT	ANKRD27	0.70382438	6.17E-28
DLAT	CTNNB1	0.70382444	6.17E-28
DLAT	MIB1	0.70400038	5.91E-28
DLAT	MAP4K4	0.7040722	5.8E-28
DLAT	VBP1	0.70428329	5.51E-28
DLAT	SMARCE1	0.7042896	5.5E-28
DLAT	STK4	0.70431249	5.47E-28
DLAT	CAPZA1	0.7043704	5.39E-28
DLAT	ZNF184	0.70450055	5.22E-28
DLAT	DYNC1LI1	0.70488823	4.74E-28
DLAT	FCHSD2	0.70489528	4.73E-28
DLAT	CRK	0.70491306	4.71E-28
DLAT	CNOT7	0.70494361	4.68E-28
DLAT	SLC44A1	0.70524925	4.34E-28
DLAT	SMIM15	0.70530625	4.28E-28
DLAT	CHD9	0.70541243	4.16E-28
DLAT	KPNB1	0.70546805	4.11E-28
DLAT	TMEM167A	0.70554912	4.03E-28
DLAT	MED13	0.70559759	3.98E-28
DLAT	ANO6	0.70565605	3.92E-28
DLAT	TMEM68	0.70572911	3.85E-28
DLAT	BCAS2	0.70580199	3.78E-28
DLAT	DCTN6	0.705822	3.76E-28
DLAT	BMI1	0.70593646	3.66E-28
DLAT	SF3B3	0.70600602	3.59E-28
DLAT	PPP2CB	0.7061303	3.48E-28

DLAT	WASF2	0.70621602	3.41E-28
DLAT	RBM43	0.70629796	3.34E-28
DLAT	TP53BP2	0.70638726	3.27E-28
DLAT	NT5C2	0.70654818	3.14E-28
DLAT	LARP7	0.70672626	3E-28
DLAT	SRSF1	0.70677845	2.96E-28
DLAT	CMTM6	0.70687933	2.89E-28
DLAT	TXNDC9	0.70703965	2.78E-28
DLAT	SMAD5	0.70709334	2.74E-28
DLAT	TVP23B	0.70717251	2.68E-28
DLAT	CHML	0.70729425	2.6E-28
DLAT	SOWAHC	0.70735328	2.57E-28
DLAT	CRIM1	0.70739766	2.54E-28
DLAT	CDK12	0.70756902	2.43E-28
DLAT	MFAP1	0.70758449	2.42E-28
DLAT	ATXN1	0.70765879	2.38E-28
DLAT	PWP1	0.70775552	2.32E-28
DLAT	RDH14	0.70781076	2.29E-28
DLAT	RACGAP1	0.70799295	2.19E-28
DLAT	RPL7L1	0.70810021	2.13E-28
DLAT	DHX36	0.70816202	2.09E-28
DLAT	NUDT4B	0.70818201	2.08E-28
DLAT	FAM20B	0.70820519	2.07E-28
DLAT	FTO	0.70820576	2.07E-28
DLAT	CORO1C	0.7082775	2.03E-28
DLAT	SFXN1	0.70833679	2E-28
DLAT	JKAMP	0.70853713	1.91E-28
DLAT	OSBPL10	0.7085457	1.9E-28
DLAT	TMEM126B	0.70856369	1.89E-28
DLAT	SNAPC1	0.7086145	1.87E-28
DLAT	IL13RA1	0.70862233	1.87E-28
DLAT	EHD4	0.70862963	1.86E-28
DLAT	FEZ2	0.70863462	1.86E-28
DLAT	TERF2	0.70864036	1.86E-28
DLAT	CYFIP1	0.70876843	1.8E-28
DLAT	LAMC1	0.70879247	1.79E-28
DLAT	NKRF	0.7090428	1.68E-28
DLAT	PEX13	0.70910904	1.65E-28
DLAT	ZNF410	0.70920151	1.61E-28
DLAT	CSGALNACT2	0.70920393	1.61E-28
DLAT	CDV3	0.70929874	1.57E-28
DLAT	ERI1	0.70932895	1.56E-28
DLAT	PSMD7	0.70946984	1.51E-28
DLAT	ITFG1	0.70952801	1.48E-28
DLAT	CRKL	0.70964281	1.44E-28
DLAT	RASA1	0.70964812	1.44E-28
DLAT	NUB1	0.70972834	1.41E-28
DLAT	ICE2	0.70984641	1.37E-28
DLAT	RNF6	0.70989397	1.35E-28
DLAT	ARF3	0.70993659	1.34E-28
DLAT	DNAJC13	0.71047898	1.17E-28
DLAT	NSL1	0.71054846	1.15E-28
DLAT	PURB	0.7106469	1.12E-28
DLAT	SMAD1	0.71067711	1.11E-28
DLAT	PLEKHA1	0.7110922	9.98E-29
DLAT	ARF6	0.71110353	9.95E-29
DLAT	YTHDF2	0.71126181	9.56E-29
DLAT	OTUD7B	0.71136453	9.31E-29

DLAT	TAOK3	0.71141898	9.18E-29
DLAT	RCN2	0.71142201	9.18E-29
DLAT	MED28	0.71150911	8.97E-29
DLAT	CCNI	0.71155958	8.86E-29
DLAT	CTR9	0.71161955	8.73E-29
DLAT	SOCS4	0.71164986	8.66E-29
DLAT	ZMAT2	0.71172374	8.5E-29
DLAT	VEZT	0.71172907	8.49E-29
DLAT	UTP11	0.71185294	8.22E-29
DLAT	GTF2E1	0.7118829	8.16E-29
DLAT	ENOX2	0.71202206	7.87E-29
DLAT	IQGAP1	0.71237999	7.18E-29
DLAT	FKBP15	0.71258029	6.83E-29
DLAT	CLNS1A	0.71280144	6.45E-29
DLAT	NEDD4	0.71286728	6.34E-29
DLAT	CAMSAP2	0.71289691	6.29E-29
DLAT	AGGF1	0.71294424	6.22E-29
DLAT	SLBP	0.71295467	6.2E-29
DLAT	CXorf38	0.71296772	6.18E-29
DLAT	HMGB1	0.71299982	6.13E-29
DLAT	PNRC2	0.71300462	6.12E-29
DLAT	TTC33	0.71300902	6.12E-29
DLAT	CCT5	0.7131554	5.89E-29
DLAT	SAR1A	0.71327937	5.7E-29
DLAT	NDE1	0.71366614	5.16E-29
DLAT	BLZF1	0.71367662	5.15E-29
DLAT	POLR2C	0.7136962	5.13E-29
DLAT	RRAGC	0.7137035	5.12E-29
DLAT	ELF1	0.71371237	5.1E-29
DLAT	SCAF11	0.71372856	5.08E-29
DLAT	SLC9A6	0.71375242	5.05E-29
DLAT	ZNF322	0.71400943	4.73E-29
DLAT	CAP1	0.71413552	4.58E-29
DLAT	PPP2R5D	0.71422439	4.47E-29
DLAT	PTK2	0.71428589	4.4E-29
DLAT	SPPL3	0.71437276	4.3E-29
DLAT	FBXL3	0.71445071	4.22E-29
DLAT	NIF3L1	0.71448265	4.18E-29
DLAT	ZNF562	0.71453577	4.13E-29
DLAT	RNF41	0.71461499	4.04E-29
DLAT	ETV3	0.71469625	3.96E-29
DLAT	FAM102B	0.71478426	3.87E-29
DLAT	PIP4P2	0.71484437	3.81E-29
DLAT	HCCS	0.71486919	3.79E-29
DLAT	ARMC8	0.71487368	3.78E-29
DLAT	TRPS1	0.71508794	3.58E-29
DLAT	SDHC	0.71512199	3.55E-29
DLAT	SPRED2	0.7151966	3.48E-29
DLAT	TPP1	0.71530763	3.38E-29
DLAT	ZBTB6	0.715572	3.16E-29
DLAT	AKAP11	0.71568473	3.07E-29
DLAT	MANBA	0.7158077	2.97E-29
DLAT	UBE2D1	0.7159336	2.87E-29
DLAT	CAMK2D	0.71634144	2.58E-29
DLAT	MRPL3	0.71634987	2.58E-29
DLAT	FZD6	0.71654919	2.45E-29
DLAT	DIAPH2	0.71659383	2.42E-29
DLAT	APLP2	0.71665537	2.38E-29

DLAT	EXT1	0.71682206	2.28E-29
DLAT	ACBD5	0.71726601	2.03E-29
DLAT	SNAP23	0.71727989	2.02E-29
DLAT	DCK	0.71734758	1.99E-29
DLAT	PIP5K1A	0.71740647	1.96E-29
DLAT	MRPL35	0.7174478	1.94E-29
DLAT	C16orf70	0.71753739	1.89E-29
DLAT	SNX4	0.71755362	1.88E-29
DLAT	YEATS2	0.7175625	1.88E-29
DLAT	RNF139	0.71761936	1.85E-29
DLAT	SKP1	0.71766837	1.83E-29
DLAT	CHRAC1	0.71805852	1.65E-29
DLAT	ST13	0.71821678	1.58E-29
DLAT	PDZD8	0.71840673	1.51E-29
DLAT	CDC42EP3	0.71847754	1.48E-29
DLAT	CHUK	0.71894618	1.31E-29
DLAT	VPS41	0.71901348	1.28E-29
DLAT	MCM4	0.719087	1.26E-29
DLAT	GOSR1	0.71923705	1.21E-29
DLAT	MRPL30	0.71947953	1.14E-29
DLAT	ZBTB2	0.71971934	1.07E-29
DLAT	ZBTB33	0.7198732	1.02E-29
DLAT	BTF3L4	0.71987731	1.02E-29
DLAT	BNIP3L	0.71991982	1.01E-29
DLAT	SFT2D2	0.71994966	1E-29
DLAT	SLC25A43	0.72002226	9.84E-30
DLAT	RPGRIP1L	0.7204823	8.71E-30
DLAT	LAMTOR3	0.72107718	7.44E-30
DLAT	MED6	0.72112799	7.34E-30
DLAT	RRM2B	0.72138987	6.84E-30
DLAT	TWSG1	0.72167816	6.34E-30
DLAT	HDAC2	0.72216556	5.57E-30
DLAT	TFDP1	0.7221985	5.52E-30
DLAT	PAIP1	0.72231303	5.35E-30
DLAT	NUDCD3	0.72249557	5.1E-30
DLAT	PCYOX1	0.7226249	4.92E-30
DLAT	GDE1	0.72267716	4.85E-30
DLAT	TRA2B	0.72298511	4.47E-30
DLAT	COPB1	0.72300033	4.45E-30
DLAT	SP1	0.72305886	4.38E-30
DLAT	AHNAK	0.72314158	4.29E-30
DLAT	LMNB1	0.72322157	4.2E-30
DLAT	ACSL4	0.72349795	3.9E-30
DLAT	TBC1D5	0.723588	3.8E-30
DLAT	SOCS5	0.72365651	3.73E-30
DLAT	YAP1	0.72399234	3.41E-30
DLAT	FAM210A	0.72410021	3.31E-30
DLAT	GOLPH3	0.72410218	3.31E-30
DLAT	SNX2	0.72413205	3.28E-30
DLAT	HIPK3	0.72421352	3.21E-30
DLAT	NFATC3	0.72422993	3.2E-30
DLAT	AFAP1	0.72431485	3.13E-30
DLAT	UBTD2	0.72453104	2.95E-30
DLAT	SZRD1	0.72498629	2.61E-30
DLAT	CNOT9	0.72499161	2.6E-30
DLAT	ZNF281	0.72504129	2.57E-30
DLAT	PRRG1	0.7250771	2.55E-30
DLAT	BCL2L13	0.72512484	2.51E-30

DLAT	APP	0.72513286	2.51E-30
DLAT	RNF168	0.72521373	2.45E-30
DLAT	GMEB1	0.72541712	2.32E-30
DLAT	PPM1B	0.72568482	2.16E-30
DLAT	UBQLN2	0.7257453	2.12E-30
DLAT	PSEN1	0.72607151	1.94E-30
DLAT	YWHAZ	0.72617447	1.89E-30
DLAT	BMS1	0.72620928	1.87E-30
DLAT	RTN4	0.72622624	1.86E-30
DLAT	GOLT1B	0.72646447	1.75E-30
DLAT	GEMIN5	0.72651757	1.72E-30
DLAT	NDFIP2	0.72667897	1.65E-30
DLAT	TMEM185B	0.72672312	1.63E-30
DLAT	ZDHHC5	0.72698379	1.52E-30
DLAT	SLC4A1AP	0.72707085	1.48E-30
DLAT	FAM204A	0.72713161	1.46E-30
DLAT	SLMAP	0.72719814	1.43E-30
DLAT	RELL1	0.72721002	1.43E-30
DLAT	KLHL7	0.72736125	1.37E-30
DLAT	HIPK1	0.72739884	1.35E-30
DLAT	BAG5	0.72752485	1.31E-30
DLAT	ECD	0.72756064	1.3E-30
DLAT	LIN54	0.72758948	1.29E-30
DLAT	SPCS2	0.7276167	1.28E-30
DLAT	PTEN	0.72774854	1.23E-30
DLAT	DENND6A	0.72782514	1.21E-30
DLAT	PPP1CC	0.72782933	1.2E-30
DLAT	WDR3	0.72785834	1.19E-30
DLAT	PIGK	0.72788817	1.18E-30
DLAT	NUFIP2	0.72805072	1.13E-30
DLAT	TWF1	0.72812637	1.11E-30
DLAT	NIP7	0.72815034	1.1E-30
DLAT	STX12	0.7287606	9.32E-31
DLAT	PI4K2B	0.72880018	9.22E-31
DLAT	RAB8A	0.72888672	9.01E-31
DLAT	ZFP1	0.72906057	8.58E-31
DLAT	YES1	0.72913532	8.41E-31
DLAT	RB1	0.72921901	8.22E-31
DLAT	EPC2	0.72927543	8.09E-31
DLAT	PSMD10	0.72939013	7.84E-31
DLAT	TMEM127	0.72941699	7.78E-31
DLAT	NXT2	0.72941809	7.78E-31
DLAT	PITPNB	0.72952092	7.56E-31
DLAT	GNAQ	0.72953413	7.53E-31
DLAT	SDCBP	0.72973069	7.14E-31
DLAT	KCTD9	0.72973078	7.14E-31
DLAT	RAB22A	0.72982453	6.95E-31
DLAT	KIF20B	0.72983345	6.94E-31
DLAT	TMTC3	0.73011781	6.41E-31
DLAT	C9orf64	0.73045202	5.85E-31
DLAT	WDR1	0.73058861	5.63E-31
DLAT	VPS4B	0.73074787	5.39E-31
DLAT	DNAJC10	0.73080283	5.3E-31
DLAT	IFNAR1	0.73085668	5.23E-31
DLAT	GDI2	0.73112057	4.86E-31
DLAT	PPP1R12A	0.73121016	4.74E-31
DLAT	COQ10B	0.7312662	4.66E-31
DLAT	REST	0.73134488	4.56E-31

DLAT	INIP	0.7313877	4.51E-31
DLAT	TMCC1	0.73149031	4.38E-31
DLAT	ZNF260	0.7316332	4.21E-31
DLAT	EXOC6B	0.73166066	4.18E-31
DLAT	RNASEH1	0.73180562	4.01E-31
DLAT	SMARCA5	0.73208824	3.71E-31
DLAT	PSMD14	0.73209753	3.7E-31
DLAT	ZMYM4	0.73215478	3.64E-31
DLAT	FCHO2	0.73223352	3.56E-31
DLAT	VRK2	0.73232995	3.47E-31
DLAT	CCNY	0.73255037	3.26E-31
DLAT	BAG4	0.73268334	3.14E-31
DLAT	SGPL1	0.73295115	2.91E-31
DLAT	HPS5	0.73300393	2.87E-31
DLAT	PPP2R2A	0.733249	2.68E-31
DLAT	EMC1	0.73325036	2.68E-31
DLAT	CYB5R4	0.73332148	2.63E-31
DLAT	ITPRIPL2	0.7333781	2.59E-31
DLAT	GRAMD2B	0.73344887	2.54E-31
DLAT	CHORDC1	0.73349447	2.5E-31
DLAT	VPS37A	0.73354152	2.47E-31
DLAT	EPS15	0.73362677	2.41E-31
DLAT	CENPL	0.73370923	2.36E-31
DLAT	ZFP64	0.73404009	2.15E-31
DLAT	ANAPC10	0.73408748	2.12E-31
DLAT	SRP9	0.73412097	2.1E-31
DLAT	TRIQK	0.73420758	2.05E-31
DLAT	RAD18	0.73422574	2.04E-31
DLAT	UBN1	0.73424515	2.03E-31
DLAT	SSRP1	0.73427909	2.01E-31
DLAT	RAB7A	0.73434779	1.97E-31
DLAT	RNF121	0.73442404	1.93E-31
DLAT	FAM98A	0.73463882	1.81E-31
DLAT	NCK1	0.73471843	1.77E-31
DLAT	SLC25A46	0.73475909	1.75E-31
DLAT	ATE1	0.73479465	1.74E-31
DLAT	MOSPD1	0.73489117	1.69E-31
DLAT	FAM220A	0.73498516	1.64E-31
DLAT	DCTN5	0.735062	1.61E-31
DLAT	RYBP	0.73520451	1.55E-31
DLAT	SCARB2	0.73522723	1.54E-31
DLAT	SGO2	0.73528583	1.51E-31
DLAT	DCLRE1B	0.73546254	1.44E-31
DLAT	NCBP2	0.73553821	1.41E-31
DLAT	ZMPSTE24	0.73556731	1.39E-31
DLAT	NUP155	0.73561113	1.38E-31
DLAT	CHCHD3	0.73563016	1.37E-31
DLAT	GPN3	0.73567617	1.35E-31
DLAT	KDM2A	0.73568962	1.35E-31
DLAT	SPRED1	0.73578207	1.31E-31
DLAT	PCYT1A	0.73590345	1.27E-31
DLAT	PANK3	0.73606427	1.21E-31
DLAT	MORF4L1	0.73614719	1.18E-31
DLAT	ATAD2	0.73625175	1.15E-31
DLAT	GOLGA7	0.73625775	1.15E-31
DLAT	LARS2	0.73638861	1.1E-31
DLAT	PEX19	0.73641042	1.1E-31
DLAT	IWS1	0.73646421	1.08E-31

DLAT	HNRNPC	0.73671975	1.01E-31
DLAT	KRR1	0.73705612	9.13E-32
DLAT	EVI5	0.73706684	9.1E-32
DLAT	RSPH3	0.73733047	8.44E-32
DLAT	MARK2	0.73739739	8.28E-32
DLAT	RHOA	0.73758717	7.85E-32
DLAT	WDR47	0.7377571	7.47E-32
DLAT	DLST	0.73776586	7.46E-32
DLAT	SMARCC1	0.73781371	7.35E-32
DLAT	TSNAX	0.73820321	6.58E-32
DLAT	SKAP2	0.73852956	5.99E-32
DLAT	C3orf38	0.73858934	5.89E-32
DLAT	RPAP3	0.73859469	5.88E-32
DLAT	WDR43	0.73868194	5.73E-32
DLAT	SNW1	0.73873404	5.65E-32
DLAT	MRPL49	0.73890826	5.37E-32
DLAT	GNA13	0.73896295	5.29E-32
DLAT	FNDC3B	0.7391363	5.03E-32
DLAT	ALS2	0.73934464	4.74E-32
DLAT	CAST	0.73942601	4.63E-32
DLAT	CLOCK	0.73944453	4.6E-32
DLAT	ADAM9	0.73946779	4.57E-32
DLAT	EHBP1	0.73948075	4.55E-32
DLAT	XRCC5	0.73955579	4.46E-32
DLAT	PRRC2C	0.73956363	4.45E-32
DLAT	DFFA	0.73968341	4.3E-32
DLAT	MEF2A	0.73974861	4.22E-32
DLAT	KRAS	0.73984086	4.1E-32
DLAT	DERL1	0.73985346	4.09E-32
DLAT	ALG11	0.73998557	3.94E-32
DLAT	TCTN3	0.74015314	3.75E-32
DLAT	ISG20L2	0.74048846	3.4E-32
DLAT	TDP1	0.74056733	3.33E-32
DLAT	TMX2	0.7405985	3.3E-32
DLAT	FAM120A	0.74064762	3.25E-32
DLAT	WASL	0.74097972	2.95E-32
DLAT	CALU	0.74116213	2.8E-32
DLAT	VAPA	0.74120386	2.77E-32
DLAT	HSPA14	0.74120526	2.76E-32
DLAT	MINDY2	0.74165009	2.43E-32
DLAT	TMEM135	0.74174844	2.36E-32
DLAT	GLO1	0.74177964	2.34E-32
DLAT	TMEM237	0.7419072	2.25E-32
DLAT	ACVR1	0.74193691	2.23E-32
DLAT	VKORC1L1	0.74211137	2.12E-32
DLAT	TCF12	0.74212738	2.11E-32
DLAT	KBTBD2	0.74215075	2.1E-32
DLAT	NF1	0.74230554	2.01E-32
DLAT	ROCK1	0.74230556	2.01E-32
DLAT	PDCL	0.74245986	1.92E-32
DLAT	TEAD1	0.74257405	1.85E-32
DLAT	SNX16	0.7426519	1.81E-32
DLAT	CTBS	0.74271711	1.78E-32
DLAT	FXR1	0.74282699	1.72E-32
DLAT	AP5M1	0.7430054	1.63E-32
DLAT	KAT6A	0.74303029	1.62E-32
DLAT	PARN	0.74309481	1.59E-32
DLAT	ARHGAP11A	0.7432006	1.54E-32

DLAT	PLS3	0.74320121	1.54E-32
DLAT	RRP15	0.74325173	1.52E-32
DLAT	UTP25	0.743259	1.52E-32
DLAT	SLC12A6	0.74329843	1.5E-32
DLAT	MED1	0.7434791	1.42E-32
DLAT	DHDDS	0.74369247	1.34E-32
DLAT	PSMD12	0.74380212	1.29E-32
DLAT	SYPL1	0.74397148	1.23E-32
DLAT	ARHGAP42	0.74417215	1.16E-32
DLAT	COPS8	0.74433193	1.11E-32
DLAT	RNF111	0.74456968	1.03E-32
DLAT	NAA30	0.74522139	8.5E-33
DLAT	CHMP2B	0.74532617	8.24E-33
DLAT	ITGAV	0.74542972	7.99E-33
DLAT	EIF1AD	0.74548418	7.87E-33
DLAT	RC3H2	0.7455654	7.68E-33
DLAT	NPTN	0.74557587	7.65E-33
DLAT	SIN3A	0.74569286	7.39E-33
DLAT	UBA6	0.74575046	7.27E-33
DLAT	PEX2	0.74582334	7.11E-33
DLAT	ADH5	0.74583711	7.08E-33
DLAT	IMPA1	0.74631581	6.14E-33
DLAT	CCNK	0.74641528	5.96E-33
DLAT	HNRNPF	0.74646091	5.88E-33
DLAT	C5orf15	0.74660887	5.63E-33
DLAT	SETD7	0.74662274	5.61E-33
DLAT	RAB1A	0.7467881	5.34E-33
DLAT	TRIM32	0.74698996	5.02E-33
DLAT	ERLIN1	0.74711887	4.83E-33
DLAT	TRAPPC6B	0.74721356	4.7E-33
DLAT	NUMB	0.7472371	4.67E-33
DLAT	NUP58	0.74723931	4.66E-33
DLAT	RBFOX2	0.74729408	4.59E-33
DLAT	ROCK2	0.74787659	3.85E-33
DLAT	RALB	0.74801117	3.7E-33
DLAT	GTF3C4	0.74817645	3.52E-33
DLAT	MFAP3	0.74820056	3.5E-33
DLAT	NFE2L2	0.74820689	3.49E-33
DLAT	OSBPL8	0.74835373	3.34E-33
DLAT	SRPK2	0.74861093	3.09E-33
DLAT	HMGN4	0.74867926	3.03E-33
DLAT	ZNF609	0.74897858	2.77E-33
DLAT	SF3A3	0.74910596	2.66E-33
DLAT	BACH1	0.74912437	2.65E-33
DLAT	KDSR	0.74915951	2.62E-33
DLAT	UGDH	0.74926055	2.54E-33
DLAT	AP1AR	0.74955515	2.32E-33
DLAT	REEP3	0.74968064	2.24E-33
DLAT	MED20	0.74972922	2.2E-33
DLAT	UVRAG	0.74973749	2.2E-33
DLAT	TMEM267	0.74980869	2.15E-33
DLAT	LMBR1	0.74981377	2.15E-33
DLAT	BLOC1S6	0.74996624	2.05E-33
DLAT	RABL3	0.75003802	2.01E-33
DLAT	MORF4L2	0.75035075	1.83E-33
DLAT	IDH3A	0.75041635	1.79E-33
DLAT	MAML2	0.75046322	1.76E-33
DLAT	PRDX3	0.75046943	1.76E-33

DLAT	PPIL4	0.7506566	1.66E-33
DLAT	ASB7	0.75081236	1.59E-33
DLAT	FAM168A	0.75093756	1.53E-33
DLAT	STX7	0.750962	1.52E-33
DLAT	SLC25A32	0.75100861	1.5E-33
DLAT	DLD	0.75127364	1.38E-33
DLAT	TAOK1	0.7514113	1.32E-33
DLAT	RBBP5	0.75143656	1.31E-33
DLAT	AGFG1	0.75164536	1.23E-33
DLAT	B4GALT5	0.75166936	1.22E-33
DLAT	COA7	0.75193325	1.13E-33
DLAT	PDLIM5	0.75218255	1.04E-33
DLAT	POFUT1	0.75218875	1.04E-33
DLAT	PSME3	0.75223979	1.03E-33
DLAT	TGOLN2	0.75235294	9.92E-34
DLAT	PAICS	0.75250949	9.45E-34
DLAT	GNG12	0.75251941	9.42E-34
DLAT	JAK1	0.75285744	8.49E-34
DLAT	RANBP9	0.75288399	8.42E-34
DLAT	SSH1	0.75292133	8.33E-34
DLAT	UBE2N	0.75300834	8.11E-34
DLAT	ATP11B	0.75309161	7.9E-34
DLAT	ICMT	0.7532569	7.51E-34
DLAT	SETD3	0.75364588	6.66E-34
DLAT	UBE2D2	0.75385682	6.24E-34
DLAT	MIGA1	0.75393344	6.1E-34
DLAT	MTM1	0.75403574	5.91E-34
DLAT	HUS1	0.75419245	5.63E-34
DLAT	RNF141	0.75422517	5.57E-34
DLAT	ARL13B	0.75442814	5.23E-34
DLAT	PJA2	0.75444566	5.2E-34
DLAT	ZNF207	0.75447906	5.15E-34
DLAT	DCAF1	0.75469918	4.81E-34
DLAT	KIAA1191	0.75477134	4.71E-34
DLAT	BBX	0.75494261	4.46E-34
DLAT	CSTF2	0.75496232	4.43E-34
DLAT	PDCD6IP	0.75513203	4.21E-34
DLAT	STRN3	0.75529779	4E-34
DLAT	PANX1	0.75532296	3.96E-34
DLAT	UBXN2A	0.75553085	3.72E-34
DLAT	USP38	0.7555466	3.7E-34
DLAT	GPR107	0.75575077	3.47E-34
DLAT	TAX1BP1	0.75581878	3.4E-34
DLAT	UXS1	0.75585731	3.36E-34
DLAT	TIPRL	0.7560123	3.2E-34
DLAT	MFN2	0.75604614	3.17E-34
DLAT	ATP2C1	0.75671526	2.57E-34
DLAT	TRMT1L	0.75697746	2.37E-34
DLAT	ITGB1	0.7570391	2.32E-34
DLAT	TAF12	0.75730177	2.14E-34
DLAT	FAF2	0.7573027	2.14E-34
DLAT	TMEM33	0.75739957	2.07E-34
DLAT	FBXO45	0.75760904	1.94E-34
DLAT	CACUL1	0.7578969	1.77E-34
DLAT	CDC5L	0.75790863	1.77E-34
DLAT	WWP1	0.75796538	1.73E-34
DLAT	FEM1B	0.75806563	1.68E-34
DLAT	FBXO28	0.75810337	1.66E-34

DLAT	MAP2K1	0.75832833	1.55E-34
DLAT	ANKRD50	0.75850014	1.47E-34
DLAT	MPP5	0.75850041	1.47E-34
DLAT	RNF14	0.75869249	1.38E-34
DLAT	TMEM170A	0.75881124	1.33E-34
DLAT	ARL8B	0.75882616	1.32E-34
DLAT	SHOC2	0.75891642	1.29E-34
DLAT	ZNF143	0.75936022	1.12E-34
DLAT	ATF1	0.75944146	1.09E-34
DLAT	OLA1	0.75945325	1.08E-34
DLAT	RAP1A	0.75949067	1.07E-34
DLAT	SRSF3	0.75968704	1.01E-34
DLAT	PDS5B	0.75990392	9.4E-35
DLAT	CANX	0.76018185	8.6E-35
DLAT	UHMK1	0.76031884	8.23E-35
DLAT	B3GNT2	0.76055106	7.65E-35
DLAT	GCC2	0.76063673	7.44E-35
DLAT	RSRC1	0.76073713	7.21E-35
DLAT	AIMP1	0.76082726	7E-35
DLAT	LUZP1	0.76084489	6.96E-35
DLAT	SMC4	0.76127716	6.06E-35
DLAT	ATL3	0.76129587	6.03E-35
DLAT	NCOA4	0.76130031	6.02E-35
DLAT	SMAD2	0.76130321	6.01E-35
DLAT	RNF13	0.76191655	4.94E-35
DLAT	TRAF3IP1	0.76193102	4.92E-35
DLAT	PRKDC	0.76201929	4.78E-35
DLAT	FASTKD2	0.76210512	4.65E-35
DLAT	PTPN9	0.7621236	4.62E-35
DLAT	ANP32E	0.76220762	4.5E-35
DLAT	ERBIN	0.76235216	4.3E-35
DLAT	TBL1XR1	0.76238319	4.25E-35
DLAT	BCAP29	0.76242548	4.2E-35
DLAT	NEK7	0.76245433	4.16E-35
DLAT	KLHL20	0.76246038	4.15E-35
DLAT	TGS1	0.76255861	4.02E-35
DLAT	MAT2B	0.7626506	3.9E-35
DLAT	KLF7	0.76268652	3.86E-35
DLAT	GSPT1	0.76275954	3.77E-35
DLAT	NRBF2	0.76320106	3.27E-35
DLAT	RBM18	0.76325131	3.21E-35
DLAT	NCOA2	0.76337307	3.09E-35
DLAT	DUSP11	0.76343115	3.03E-35
DLAT	JRKL	0.76360845	2.86E-35
DLAT	EMC2	0.76375495	2.73E-35
DLAT	CBFB	0.76383341	2.66E-35
DLAT	MAPK6	0.76392635	2.58E-35
DLAT	PDCD10	0.7639915	2.53E-35
DLAT	HNRNPA2B1	0.76406204	2.47E-35
DLAT	ENAH	0.76407962	2.46E-35
DLAT	SLC25A40	0.76409362	2.45E-35
DLAT	PATL1	0.76413923	2.41E-35
DLAT	CDYL	0.7644762	2.16E-35
DLAT	DAAM1	0.76448549	2.15E-35
DLAT	ZNF639	0.76452966	2.12E-35
DLAT	PNPLA8	0.76454106	2.12E-35
DLAT	DNAJB6	0.76476391	1.97E-35
DLAT	SMC6	0.76477565	1.96E-35

DLAT	NPM1	0.76481005	1.94E-35
DLAT	KIAA1143	0.76490395	1.88E-35
DLAT	TNPO3	0.764996	1.83E-35
DLAT	EXT2	0.76508059	1.78E-35
DLAT	ZFR	0.76545302	1.57E-35
DLAT	CAMSAP1	0.76548536	1.56E-35
DLAT	SENP1	0.76566815	1.47E-35
DLAT	GHITM	0.76575988	1.42E-35
DLAT	AHCTF1	0.76581912	1.39E-35
DLAT	ARMT1	0.76592633	1.35E-35
DLAT	ZC3H13	0.76600253	1.31E-35
DLAT	PPM1A	0.76605114	1.29E-35
DLAT	TANK	0.7660623	1.29E-35
DLAT	C16orf87	0.76606932	1.29E-35
DLAT	SRGAP2B	0.76641821	1.15E-35
DLAT	TAF1A	0.76648297	1.12E-35
DLAT	SF3A1	0.76678235	1.02E-35
DLAT	RECQL	0.76709943	9.16E-36
DLAT	COPA	0.76710505	9.15E-36
DLAT	DDX3X	0.76713457	9.06E-36
DLAT	ANKRD13C	0.76714908	9.01E-36
DLAT	GLG1	0.76724618	8.73E-36
DLAT	HACD2	0.76740211	8.29E-36
DLAT	PNO1	0.76747858	8.09E-36
DLAT	RNF2	0.76750905	8E-36
DLAT	RNF4	0.76752694	7.96E-36
DLAT	LZIC	0.76753236	7.94E-36
DLAT	NLN	0.76759411	7.78E-36
DLAT	DOCK9	0.76759941	7.77E-36
DLAT	GNAI3	0.76766606	7.6E-36
DLAT	ZNF217	0.7678097	7.25E-36
DLAT	QTRT2	0.76781531	7.23E-36
DLAT	SESTD1	0.76791044	7.01E-36
DLAT	ABI2	0.7679201	6.99E-36
DLAT	RPRD1B	0.7679793	6.85E-36
DLAT	CDC27	0.76813816	6.5E-36
DLAT	C5orf24	0.76895544	4.96E-36
DLAT	LDAH	0.76898451	4.91E-36
DLAT	MAGT1	0.76916116	4.63E-36
DLAT	BCL9L	0.76919807	4.57E-36
DLAT	DNAJB14	0.7693417	4.36E-36
DLAT	LRRC57	0.76949554	4.14E-36
DLAT	PTPN11	0.76956523	4.05E-36
DLAT	FAM98B	0.7695921	4.01E-36
DLAT	SAP130	0.76981142	3.73E-36
DLAT	R3HDM1	0.76983779	3.7E-36
DLAT	MFSD14B	0.7699163	3.6E-36
DLAT	SERBP1	0.76996837	3.54E-36
DLAT	RAB3GAP2	0.76998975	3.51E-36
DLAT	RFWD3	0.76999523	3.51E-36
DLAT	MAPRE1	0.77026352	3.21E-36
DLAT	SUZ12	0.77029288	3.17E-36
DLAT	HBP1	0.77029518	3.17E-36
DLAT	NDC1	0.7703584	3.1E-36
DLAT	ASAP1	0.77037507	3.09E-36
DLAT	PSMC2	0.77051967	2.94E-36
DLAT	CSDE1	0.77061021	2.85E-36
DLAT	ZBTB38	0.77074312	2.73E-36

DLAT	SEC23A	0.77078694	2.69E-36
DLAT	METAP1	0.77091847	2.57E-36
DLAT	UBE2A	0.77092303	2.57E-36
DLAT	PPP1R2	0.77101329	2.49E-36
DLAT	MMGT1	0.77116221	2.37E-36
DLAT	CCDC90B	0.77126293	2.29E-36
DLAT	TMEM87B	0.77130065	2.26E-36
DLAT	ZNF720	0.77151661	2.1E-36
DLAT	SLC30A1	0.77162137	2.03E-36
DLAT	CPSF6	0.77164294	2.02E-36
DLAT	ADAR	0.77167858	1.99E-36
DLAT	FYTTD1	0.77168994	1.99E-36
DLAT	MOB4	0.77172744	1.96E-36
DLAT	KPNA6	0.77177964	1.93E-36
DLAT	SNTB2	0.77179379	1.92E-36
DLAT	BRCC3	0.77210733	1.72E-36
DLAT	CDC42	0.77250667	1.51E-36
DLAT	PTBP3	0.77268393	1.42E-36
DLAT	RAB21	0.7728712	1.33E-36
DLAT	NT5DC1	0.77298524	1.28E-36
DLAT	CTTNBP2NL	0.77309229	1.24E-36
DLAT	ZYG11B	0.77314017	1.22E-36
DLAT	ATG5	0.7731949	1.19E-36
DLAT	VPS26A	0.773298	1.15E-36
DLAT	TMEM87A	0.77333802	1.14E-36
DLAT	UGP2	0.77371372	1E-36
DLAT	C1D	0.77375229	9.87E-37
DLAT	SLC30A9	0.77389018	9.42E-37
DLAT	WDR26	0.77392903	9.29E-37
DLAT	OSBPL11	0.77396549	9.18E-37
DLAT	MAPK1IP1L	0.77407041	8.86E-37
DLAT	PRELID3B	0.77427357	8.26E-37
DLAT	NOLC1	0.77431355	8.15E-37
DLAT	WDR82	0.7749073	6.65E-37
DLAT	ARHGAP5	0.77518101	6.06E-37
DLAT	UBXN2B	0.7752864	5.84E-37
DLAT	GNB1	0.77536264	5.69E-37
DLAT	CLIP1	0.77544554	5.53E-37
DLAT	TOR1AIP1	0.77549323	5.44E-37
DLAT	USP8	0.77557772	5.28E-37
DLAT	SPAST	0.7757966	4.9E-37
DLAT	CTCF	0.77584638	4.82E-37
DLAT	GFM1	0.77652257	3.82E-37
DLAT	CCSER2	0.77666429	3.63E-37
DLAT	ARHGEF12	0.77667552	3.62E-37
DLAT	ZNF623	0.77688932	3.36E-37
DLAT	CERS6	0.77704773	3.18E-37
DLAT	CDC73	0.77738117	2.83E-37
DLAT	USP10	0.77743188	2.79E-37
DLAT	TSN	0.777689	2.55E-37
DLAT	STAM	0.77777003	2.48E-37
DLAT	UBA2	0.77781958	2.43E-37
DLAT	ATL2	0.77794885	2.33E-37
DLAT	VAMP3	0.77798014	2.3E-37
DLAT	PRPF4	0.7780529	2.24E-37
DLAT	CCNG2	0.77839391	1.99E-37
DLAT	G3BP2	0.77842688	1.97E-37
DLAT	ATP2A2	0.77856289	1.88E-37

DLAT	GNL3L	0.77857997	1.87E-37
DLAT	KIF5B	0.77914372	1.53E-37
DLAT	PMS2	0.77938174	1.41E-37
DLAT	LRRCC1	0.77972566	1.25E-37
DLAT	PTPN12	0.77981483	1.21E-37
DLAT	PPP4R3B	0.77989858	1.18E-37
DLAT	MED14	0.77997825	1.14E-37
DLAT	SLC25A24	0.78014088	1.08E-37
DLAT	UBA3	0.78033348	1.01E-37
DLAT	ANXA7	0.78040001	9.86E-38
DLAT	PPP3CA	0.78063621	9.07E-38
DLAT	ATP13A3	0.78081144	8.53E-38
DLAT	RP2	0.78090567	8.25E-38
DLAT	ABRAXAS2	0.78104373	7.86E-38
DLAT	PPFIA1	0.7811083	7.68E-38
DLAT	YWHAG	0.78123819	7.33E-38
DLAT	PGGT1B	0.78166822	6.3E-38
DLAT	SH3GLB1	0.78170047	6.22E-38
DLAT	STAU1	0.78177677	6.06E-38
DLAT	SDE2	0.78199719	5.6E-38
DLAT	AFF4	0.78200553	5.58E-38
DLAT	TXNRD1	0.78213144	5.34E-38
DLAT	SPIN1	0.78229109	5.05E-38
DLAT	GPD2	0.78253582	4.62E-38
DLAT	RAB5A	0.78308508	3.8E-38
DLAT	ARPP19	0.78327616	3.55E-38
DLAT	DESI2	0.78339167	3.41E-38
DLAT	RAB14	0.78354227	3.23E-38
DLAT	STAG2	0.78377279	2.97E-38
DLAT	SELENOF	0.78387634	2.86E-38
DLAT	CDC23	0.7842867	2.47E-38
DLAT	AIDA	0.78481497	2.04E-38
DLAT	ABI1	0.78487483	2E-38
DLAT	STT3B	0.78497983	1.92E-38
DLAT	XPR1	0.78505595	1.87E-38
DLAT	RAD1	0.78531362	1.7E-38
DLAT	RLIM	0.78538405	1.66E-38
DLAT	EFCAB14	0.78549564	1.6E-38
DLAT	YWHAQ	0.78549619	1.6E-38
DLAT	ZPR1	0.78554803	1.57E-38
DLAT	TMEM167B	0.78554986	1.57E-38
DLAT	ADNP	0.78565065	1.51E-38
DLAT	STAU2	0.78578388	1.44E-38
DLAT	SLC35A5	0.78605766	1.3E-38
DLAT	DLG1	0.78630295	1.19E-38
DLAT	HNRNPLL	0.78664223	1.05E-38
DLAT	UBE2V2	0.78671332	1.03E-38
DLAT	NCL	0.78673259	1.02E-38
DLAT	RPS6KA3	0.78677781	1E-38
DLAT	EIF4G2	0.78692434	9.49E-39
DLAT	FBXO38	0.78694024	9.44E-39
DLAT	VAMP7	0.78709597	8.92E-39
DLAT	MAPK14	0.78715693	8.72E-39
DLAT	NCOA3	0.78733651	8.17E-39
DLAT	NAB1	0.78736463	8.08E-39
DLAT	GMFB	0.7875034	7.68E-39
DLAT	TMX1	0.78800603	6.39E-39
DLAT	HAT1	0.78800608	6.39E-39

DLAT	PDHX	0.78846966	5.39E-39
DLAT	EIF2AK2	0.78880101	4.77E-39
DLAT	RASAL2	0.78881947	4.74E-39
DLAT	CSNK2A1	0.78889844	4.6E-39
DLAT	CWC22	0.78890555	4.59E-39
DLAT	ZNF148	0.78894109	4.53E-39
DLAT	C16orf72	0.7891621	4.17E-39
DLAT	OSGIN2	0.78950605	3.68E-39
DLAT	LSM14A	0.7898598	3.22E-39
DLAT	SNRNP27	0.79014585	2.9E-39
DLAT	ZBTB41	0.79017461	2.87E-39
DLAT	TMEM106B	0.79020875	2.83E-39
DLAT	NIPA2	0.79036717	2.67E-39
DLAT	DYNC1I2	0.79048979	2.55E-39
DLAT	KHDRBS1	0.7906297	2.42E-39
DLAT	EIF4G3	0.79091731	2.18E-39
DLAT	YIPF6	0.79134524	1.85E-39
DLAT	HNRNPU	0.79152428	1.73E-39
DLAT	YIPF5	0.79172285	1.61E-39
DLAT	IARS2	0.79214573	1.37E-39
DLAT	DNAJC21	0.79216115	1.37E-39
DLAT	MAPK1	0.79218026	1.36E-39
DLAT	LEPROT	0.79233385	1.28E-39
DLAT	CNOT8	0.79239047	1.25E-39
DLAT	SEC23IP	0.79255001	1.18E-39
DLAT	ZFAND3	0.79259208	1.16E-39
DLAT	C11orf58	0.79260701	1.16E-39
DLAT	BAZ1B	0.79274327	1.1E-39
DLAT	SPDL1	0.79278156	1.08E-39
DLAT	TMEM123	0.79351716	8.19E-40
DLAT	RAB3GAP1	0.79367681	7.71E-40
DLAT	RAB2A	0.7937033	7.64E-40
DLAT	DDX6	0.79404558	6.71E-40
DLAT	PUM2	0.7941465	6.45E-40
DLAT	ADAM17	0.79440561	5.85E-40
DLAT	TRUB1	0.79442793	5.8E-40
DLAT	PIK3CA	0.79446185	5.73E-40
DLAT	PRRC1	0.79449466	5.65E-40
DLAT	ARL6IP6	0.7945056	5.63E-40
DLAT	STRN	0.79541016	3.99E-40
DLAT	TAB2	0.79547478	3.89E-40
DLAT	MOB1A	0.79570298	3.57E-40
DLAT	CWF19L2	0.79644338	2.68E-40
DLAT	PPP1CB	0.79663444	2.49E-40
DLAT	ARCN1	0.79664064	2.49E-40
DLAT	TMED7	0.79667153	2.46E-40
DLAT	KPNA3	0.79671037	2.42E-40
DLAT	EXOC5	0.79671385	2.42E-40
DLAT	SYNCRIP	0.79677197	2.36E-40
DLAT	USP9X	0.7968276	2.31E-40
DLAT	HNRNPH2	0.79684042	2.3E-40
DLAT	INCENP	0.79715565	2.04E-40
DLAT	BMPR2	0.79728156	1.94E-40
DLAT	TAF1B	0.79809171	1.42E-40
DLAT	MATR3	0.79841017	1.25E-40
DLAT	MBD5	0.7986909	1.12E-40
DLAT	OXR1	0.79870736	1.12E-40
DLAT	SMC2	0.79889232	1.04E-40

DLAT	RBBP4	0.79909324	9.61E-41
DLAT	FAM91A1	0.7997081	7.56E-41
DLAT	MRPL19	0.7997526	7.43E-41
DLAT	EIF1AX	0.79983867	7.18E-41
DLAT	ACBD3	0.80014555	6.37E-41
DLAT	THRAP3	0.80063902	5.24E-41
DLAT	MIER1	0.80088194	4.77E-41
DLAT	DDX19A	0.80110103	4.37E-41
DLAT	NRAS	0.80119141	4.22E-41
DLAT	CREB1	0.80152915	3.69E-41
DLAT	FBXW2	0.80154768	3.66E-41
DLAT	PPP3R1	0.80163165	3.54E-41
DLAT	ESYT2	0.80169858	3.45E-41
DLAT	PARG	0.80225111	2.77E-41
DLAT	PPP1R8	0.80262762	2.39E-41
DLAT	POLR2D	0.8027847	2.24E-41
DLAT	ATF2	0.80287866	2.16E-41
DLAT	GORASP2	0.80313026	1.95E-41
DLAT	VCPIP1	0.80328464	1.84E-41
DLAT	FOXN2	0.80344572	1.72E-41
DLAT	CUL4B	0.80373473	1.53E-41
DLAT	PGM2	0.80387499	1.45E-41
DLAT	SMC1A	0.80400566	1.38E-41
DLAT	ARIH1	0.80404417	1.35E-41
DLAT	UBFD1	0.80409198	1.33E-41
DLAT	CAPRIN1	0.80411831	1.31E-41
DLAT	BTBD1	0.8046971	1.04E-41
DLAT	WAC	0.8054501	7.68E-42
DLAT	SMC3	0.80550622	7.51E-42
DLAT	ATP6V1A	0.8056214	7.17E-42
DLAT	UBE2D3	0.80572138	6.88E-42
DLAT	MAP3K2	0.80589221	6.42E-42
DLAT	RO60	0.80600346	6.14E-42
DLAT	SSB	0.80632128	5.39E-42
DLAT	VTA1	0.80655801	4.9E-42
DLAT	TERF1	0.8065882	4.84E-42
DLAT	RBM12	0.80672403	4.58E-42
DLAT	UEVLD	0.80684045	4.37E-42
DLAT	NEDD1	0.80723684	3.71E-42
DLAT	RSF1	0.80730736	3.61E-42
DLAT	HS2ST1	0.80747591	3.37E-42
DLAT	UBE3A	0.80786871	2.87E-42
DLAT	GTF2H3	0.80794111	2.78E-42
DLAT	STK3	0.80794673	2.78E-42
DLAT	USP14	0.80798851	2.73E-42
DLAT	PPP4R2	0.80802866	2.68E-42
DLAT	DOCK7	0.80820423	2.5E-42
DLAT	PAFAH1B2	0.80833747	2.36E-42
DLAT	UBE3C	0.80837131	2.33E-42
DLAT	YWHAB	0.80869896	2.04E-42
DLAT	ETF1	0.80882097	1.94E-42
DLAT	BZW1	0.80907768	1.74E-42
DLAT	CAND1	0.80935364	1.55E-42
DLAT	DIS3	0.80942462	1.51E-42
DLAT	PTDSS1	0.80967388	1.36E-42
DLAT	TMED5	0.80977359	1.31E-42
DLAT	EIF2S1	0.81000429	1.19E-42
DLAT	CFAP97	0.81002812	1.17E-42

DLAT	MFSD14A	0.81006074	1.16E-42
DLAT	TNPO1	0.81009424	1.14E-42
DLAT	SERINC3	0.81030436	1.05E-42
DLAT	ZNHIT6	0.81033266	1.03E-42
DLAT	GTF2A1	0.81061229	9.21E-43
DLAT	RNF11	0.81092551	8.08E-43
DLAT	WDFY1	0.81122411	7.13E-43
DLAT	ATAD1	0.8113058	6.89E-43
DLAT	OPA1	0.81161901	6.04E-43
DLAT	TCEA1	0.81220959	4.71E-43
DLAT	TARDBP	0.81260689	3.99E-43
DLAT	TAF2	0.81303953	3.32E-43
DLAT	ATMIN	0.8131259	3.2E-43
DLAT	ACAP2	0.81440242	1.86E-43
DLAT	DPY19L4	0.81469912	1.64E-43
DLAT	ESF1	0.8147082	1.63E-43
DLAT	MBTPS2	0.81513634	1.36E-43
DLAT	XRN2	0.81523628	1.3E-43
DLAT	KIF2A	0.81553176	1.15E-43
DLAT	CSNK1G3	0.81584066	1E-43
DLAT	DDX21	0.81620218	8.59E-44
DLAT	ACTR3	0.81669324	6.95E-44
DLAT	CBL	0.81679608	6.64E-44
DLAT	ARL5A	0.81694095	6.24E-44
DLAT	COMMD2	0.81696184	6.18E-44
DLAT	CSNK1A1	0.8175402	4.81E-44
DLAT	PLEKHA3	0.81778132	4.33E-44
DLAT	SPOPL	0.81788836	4.13E-44
DLAT	RPE	0.8179506	4.02E-44
DLAT	PPIG	0.81883281	2.73E-44
DLAT	PCNP	0.81894134	2.61E-44
DLAT	HMGXB4	0.8196318	1.92E-44
DLAT	MSH2	0.81972115	1.85E-44
DLAT	RAD21	0.81972533	1.85E-44
DLAT	PAK2	0.82012723	1.55E-44
DLAT	SLC30A6	0.82015721	1.53E-44
DLAT	YTHDF3	0.8202903	1.44E-44
DLAT	SPTY2D1	0.82050673	1.31E-44
DLAT	NAA50	0.82070055	1.2E-44
DLAT	UBQLN1	0.82092301	1.09E-44
DLAT	DNAJA2	0.8212628	9.35E-45
DLAT	EFR3A	0.82131262	9.14E-45
DLAT	PUM1	0.82136532	8.93E-45
DLAT	NCKAP1	0.82160702	8.02E-45
DLAT	PPP2CA	0.82167486	7.78E-45
DLAT	TMOD3	0.82241118	5.59E-45
DLAT	TLK1	0.82261441	5.11E-45
DLAT	GANAB	0.82285584	4.58E-45
DLAT	RHBDD1	0.82296347	4.37E-45
DLAT	VPS35	0.82302407	4.25E-45
DLAT	ASXL2	0.82468639	2E-45
DLAT	KPNA4	0.82532378	1.5E-45
DLAT	LYPLA1	0.82535323	1.48E-45
DLAT	RTF1	0.82603523	1.08E-45
DLAT	RAD23B	0.82624948	9.8E-46
DLAT	ADAM10	0.82633469	9.42E-46
DLAT	CNIH1	0.82667038	8.07E-46
DLAT	SP3	0.82732462	5.97E-46

DLAT	YY1	0.82761107	5.23E-46
DLAT	PRPF40A	0.82788286	4.61E-46
DLAT	PLEKHB2	0.82844139	3.55E-46
DLAT	DEK	0.82851845	3.43E-46
DLAT	DR1	0.8288829	2.89E-46
DLAT	LNPK	0.82922474	2.46E-46
DLAT	TOP1	0.82938891	2.28E-46
DLAT	API5	0.82973738	1.94E-46
DLAT	IPO7	0.83008729	1.64E-46
DLAT	RBM27	0.8301282	1.61E-46
DLAT	USP1	0.83014821	1.6E-46
DLAT	CTDSPL2	0.83128079	9.35E-47
DLAT	SELENOT	0.83128417	9.33E-47
DLAT	RAB10	0.83197168	6.73E-47
DLAT	TOX4	0.83205993	6.45E-47
DLAT	SPTLC1	0.83208231	6.38E-47
DLAT	ACTR2	0.8322992	5.75E-47
DLAT	PICALM	0.83231308	5.72E-47
DLAT	CAPZA2	0.83233691	5.65E-47
DLAT	DDX18	0.8331545	3.82E-47
DLAT	CPNE3	0.83319343	3.75E-47
DLAT	TRIP12	0.83319701	3.74E-47
DLAT	ORC4	0.83339648	3.4E-47
DLAT	KPNA1	0.83377493	2.83E-47
DLAT	C5orf51	0.83408117	2.44E-47
DLAT	EIF4E	0.8343647	2.13E-47
DLAT	CNOT6	0.83478588	1.74E-47
DLAT	AGPS	0.83507316	1.51E-47
DLAT	ZC3H15	0.83517033	1.44E-47
DLAT	ZNF146	0.83529916	1.35E-47
DLAT	DCUN1D1	0.8359198	9.99E-48
DLAT	RRN3	0.83677818	6.55E-48
DLAT	TFAM	0.83766533	4.23E-48
DLAT	NUDCD1	0.83791077	3.74E-48
DLAT	CAB39	0.83807626	3.45E-48
DLAT	SRFBP1	0.83821571	3.22E-48
DLAT	SLAIN2	0.83988724	1.39E-48
DLAT	METTL9	0.84106032	7.71E-49
DLAT	COPS2	0.84131951	6.76E-49
DLAT	NPAT	0.84148231	6.22E-49
DLAT	WDR44	0.84148752	6.21E-49
DLAT	HNRNPR	0.84222911	4.25E-49
DLAT	MTPN	0.84278559	3.2E-49
DLAT	NBN	0.843137	2.67E-49
DLAT	MTMR2	0.84351471	2.2E-49
DLAT	CPSF2	0.84372911	1.97E-49
DLAT	UBE2W	0.8439409	1.76E-49
DLAT	SMNDC1	0.84395015	1.76E-49
DLAT	RAB18	0.8443399	1.43E-49
DLAT	PHAX	0.84799047	2.11E-50
DLAT	CKAP5	0.84834342	1.74E-50
DLAT	MTDH	0.84840491	1.69E-50
DLAT	PDS5A	0.84916493	1.12E-50
DLAT	SNX6	0.84926794	1.06E-50
DLAT	MED17	0.84935832	1.01E-50
DLAT	NAA15	0.84972323	8.32E-51
DLAT	PPP2R5E	0.85078958	4.67E-51
DLAT	HAUS2	0.85138947	3.37E-51

DLAT	FCF1	0.85215514	2.22E-51
DLAT	ATP6V1C1	0.85230303	2.04E-51
DLAT	MCMBP	0.85254967	1.78E-51
DLAT	HNRNPK	0.85290766	1.46E-51
DLAT	SET	0.85338521	1.12E-51
DLAT	ITCH	0.85454045	5.92E-52
DLAT	CUL2	0.85540572	3.64E-52
DLAT	ARMC1	0.86012033	2.45E-53
DLAT	NDUFS1	0.86412878	2.28E-54
DLAT	YME1L1	0.8671685	3.58E-55
DLAT	G3BP1	0.86741292	3.08E-55
DLAT	OGFOD1	0.87016122	5.54E-56
DLAT	RBM7	0.87302688	8.86E-57
DLAT	SDHD	0.87505733	2.35E-57
DLAT	ZFP91	0.87619503	1.11E-57
DLAT	RAB6A	0.90477736	3.78E-67
DLAT	DLAT	1	0
DLD	RHNO1	0.50014516	1.18E-12
DLD	ARF1	0.50020094	1.17E-12
DLD	ARPC2	0.50020358	1.17E-12
DLD	SERTAD2	0.50044447	1.13E-12
DLD	GON7	0.50045517	1.13E-12
DLD	NAP1L1	0.50046351	1.13E-12
DLD	AKT3	0.5005458	1.12E-12
DLD	PANK2	0.50055996	1.12E-12
DLD	PPP4R1	0.5005812	1.12E-12
DLD	EXOSC3	0.50069461	1.1E-12
DLD	IFIT5	0.50077927	1.09E-12
DLD	ATG7	0.50080249	1.09E-12
DLD	BRIX1	0.50089696	1.08E-12
DLD	MAP3K13	0.50093236	1.07E-12
DLD	DIABLO	0.50094483	1.07E-12
DLD	TRIOBP	0.50096953	1.07E-12
DLD	PPIL1	0.50101477	1.06E-12
DLD	KITLG	0.50107433	1.05E-12
DLD	PRRG1	0.50116178	1.04E-12
DLD	PIP5K1C	0.50154992	9.94E-13
DLD	SIPA1L1	0.50156323	9.93E-13
DLD	PSMA4	0.50167621	9.79E-13
DLD	ENTPD1	0.50168645	9.78E-13
DLD	IDI1	0.50171042	9.75E-13
DLD	FAM83B	0.50186441	9.57E-13
DLD	PRXL2C	0.50205403	9.36E-13
DLD	PPP2R5C	0.50213701	9.27E-13
DLD	CNNM4	0.50221485	9.18E-13
DLD	ZMYM2	0.50233893	9.04E-13
DLD	DNASE2	0.50237304	9.01E-13
DLD	HMGCS1	0.50243989	8.93E-13
DLD	FBXL14	0.50253749	8.83E-13
DLD	TLN1	0.50254076	8.83E-13
DLD	NIN	0.50254212	8.83E-13
DLD	STOM	0.50257533	8.79E-13
DLD	ADGRA2	0.50288094	8.47E-13
DLD	CHSY1	0.50290318	8.45E-13
DLD	DENND1A	0.50300516	8.35E-13
DLD	POLR3GL	0.50300542	8.35E-13
DLD	DZIP3	0.50311801	8.23E-13
DLD	KCTD2	0.50320271	8.15E-13

DLD	CASP6	0.5032796	8.08E-13
DLD	PPP5C	0.50335979	8E-13
DLD	NUDT21	0.50346134	7.9E-13
DLD	MAPKAP1	0.50351557	7.85E-13
DLD	SNUPN	0.50355263	7.81E-13
DLD	CHURC1	0.50387335	7.52E-13
DLD	ZDHHC6	0.50400512	7.4E-13
DLD	GRK6	0.50420078	7.23E-13
DLD	FAM210B	0.50443594	7.02E-13
DLD	OTUD1	0.50464691	6.85E-13
DLD	ATAD2	0.50468454	6.82E-13
DLD	ALG1	0.50475713	6.76E-13
DLD	ELK3	0.50477281	6.74E-13
DLD	MREG	0.50490279	6.64E-13
DLD	YWHAZ	0.50505049	6.52E-13
DLD	RAD51B	0.50505237	6.52E-13
DLD	DCAF13	0.50513096	6.46E-13
DLD	AMACR	0.50520475	6.4E-13
DLD	NIPSNAP1	0.5052508	6.36E-13
DLD	MOB3B	0.50528849	6.33E-13
DLD	MZT1	0.50532536	6.31E-13
DLD	ZC2HC1A	0.50533448	6.3E-13
DLD	SRSF7	0.50587944	5.9E-13
DLD	TIGD2	0.50598431	5.82E-13
DLD	NOTCH2	0.50613385	5.72E-13
DLD	LIN9	0.506313	5.59E-13
DLD	TTC23	0.50645123	5.5E-13
DLD	DSC2	0.5064949	5.47E-13
DLD	CTSS	0.50659529	5.4E-13
DLD	CD93	0.50672061	5.32E-13
DLD	PLGRKT	0.50672364	5.32E-13
DLD	PARP14	0.50683723	5.25E-13
DLD	TXNDC15	0.50693025	5.19E-13
DLD	SYNPO2	0.50694153	5.18E-13
DLD	ZBTB7A	0.50694531	5.18E-13
DLD	EMB	0.50731407	4.95E-13
DLD	GPR137B	0.50739095	4.9E-13
DLD	METTL6	0.50741998	4.89E-13
DLD	DCLRE1C	0.50764747	4.75E-13
DLD	APLP2	0.50779971	4.66E-13
DLD	VDAC2	0.50781777	4.65E-13
DLD	YY1AP1	0.50781938	4.65E-13
DLD	GID8	0.50785886	4.63E-13
DLD	FP565260.3	0.50815711	4.47E-13
DLD	CHMP7	0.50830662	4.38E-13
DLD	RASSF3	0.50831723	4.38E-13
DLD	TMEM140	0.50833856	4.37E-13
DLD	PANX1	0.50837589	4.35E-13
DLD	CIAO2A	0.50845538	4.3E-13
DLD	ARL4A	0.50850109	4.28E-13
DLD	ENTPD5	0.5085179	4.27E-13
DLD	FAM222B	0.50861175	4.22E-13
DLD	AP3S1	0.50873846	4.16E-13
DLD	IQSEC1	0.50888696	4.08E-13
DLD	SLC39A14	0.508936	4.06E-13
DLD	AKAP1	0.50894048	4.06E-13
DLD	FUCA1	0.50897857	4.04E-13
DLD	EFNB2	0.50898929	4.03E-13

DLD	NDUFS4	0.50909118	3.98E-13
DLD	NAT1	0.50935642	3.85E-13
DLD	LAMP1	0.50936347	3.85E-13
DLD	PKP4	0.50938466	3.84E-13
DLD	DLGAP4	0.5095938	3.74E-13
DLD	PAK1	0.50964215	3.72E-13
DLD	MTX2	0.5097574	3.67E-13
DLD	ANAPC13	0.50979472	3.65E-13
DLD	PI4K2A	0.50989449	3.61E-13
DLD	MARCKS	0.50991647	3.6E-13
DLD	NCOA7	0.51007095	3.53E-13
DLD	CBX3	0.51011046	3.51E-13
DLD	CCDC115	0.51027331	3.44E-13
DLD	FAS	0.51034713	3.41E-13
DLD	CYP51A1	0.51035571	3.41E-13
DLD	IL10RB	0.51037656	3.4E-13
DLD	PRIM2	0.5105631	3.32E-13
DLD	DAB2IP	0.51062212	3.3E-13
DLD	SLC35B4	0.51075307	3.24E-13
DLD	RAB12	0.51076271	3.24E-13
DLD	HEG1	0.51089908	3.19E-13
DLD	RETREG2	0.51115297	3.09E-13
DLD	NXPE3	0.51132136	3.02E-13
DLD	SPRY1	0.51150076	2.96E-13
DLD	TOE1	0.51154889	2.94E-13
DLD	MIS18A	0.51179952	2.85E-13
DLD	LIMS1	0.51190416	2.81E-13
DLD	MAST4	0.51210867	2.74E-13
DLD	CCT5	0.51236773	2.66E-13
DLD	TRAF7	0.51238331	2.65E-13
DLD	CCDC59	0.5123958	2.65E-13
DLD	CALD1	0.51244461	2.63E-13
DLD	RAN	0.51248273	2.62E-13
DLD	NIPBL	0.51255272	2.6E-13
DLD	CLIP2	0.5126387	2.57E-13
DLD	DTX3L	0.51274864	2.53E-13
DLD	LIPG	0.51279767	2.52E-13
DLD	KIF13B	0.51295257	2.47E-13
DLD	RPGRIP1L	0.51298534	2.46E-13
DLD	GNE	0.51307422	2.43E-13
DLD	WWC3	0.51320624	2.39E-13
DLD	NAAA	0.51350203	2.31E-13
DLD	PPIL3	0.51364978	2.27E-13
DLD	RAMAC	0.51374561	2.24E-13
DLD	CCDC97	0.51374996	2.24E-13
DLD	PBX3	0.51375238	2.24E-13
DLD	TMEM35B	0.5139275	2.19E-13
DLD	PTPN4	0.51393577	2.19E-13
DLD	GLRX	0.51398096	2.17E-13
DLD	MEAK7	0.51411562	2.14E-13
DLD	SPAG1	0.51411683	2.14E-13
DLD	PFDN1	0.5144777	2.04E-13
DLD	CETN3	0.5144863	2.04E-13
DLD	CYB5R3	0.51453195	2.03E-13
DLD	SUSD1	0.51453329	2.03E-13
DLD	COG2	0.51461697	2.01E-13
DLD	ZHX2	0.51474415	1.98E-13
DLD	RGS5	0.5147893	1.96E-13

DLD	SLC6A6	0.51507666	1.9E-13
DLD	FUCA2	0.51573765	1.74E-13
DLD	KDM6B	0.51586063	1.72E-13
DLD	SOWAHC	0.51657631	1.57E-13
DLD	STK26	0.51661352	1.56E-13
DLD	TSC22D2	0.51713634	1.46E-13
DLD	SLFN11	0.5171369	1.46E-13
DLD	KTI12	0.51727024	1.44E-13
DLD	ARHGAP31	0.51732659	1.43E-13
DLD	PNRC1	0.51733137	1.43E-13
DLD	ZNF28	0.51733455	1.43E-13
DLD	NR3C1	0.517365	1.42E-13
DLD	ZNF277	0.51738149	1.42E-13
DLD	DSG2	0.51740985	1.41E-13
DLD	HCFC1	0.51756369	1.39E-13
DLD	ATG16L1	0.51759234	1.38E-13
DLD	STAT1	0.51787399	1.33E-13
DLD	GRPEL1	0.51787624	1.33E-13
DLD	CLDND1	0.51793046	1.32E-13
DLD	BUB3	0.5179377	1.32E-13
DLD	FAM200A	0.51796689	1.32E-13
DLD	TUT7	0.51802737	1.31E-13
DLD	CIP2A	0.51822862	1.27E-13
DLD	KCTD5	0.51849315	1.23E-13
DLD	SDC3	0.51859846	1.22E-13
DLD	RAB1B	0.51864596	1.21E-13
DLD	ZMYND8	0.51878003	1.19E-13
DLD	RHOBTB2	0.51887141	1.17E-13
DLD	GPN1	0.51912242	1.14E-13
DLD	UBE2I	0.51918065	1.13E-13
DLD	PECAM1	0.51925167	1.12E-13
DLD	RGL1	0.5192964	1.11E-13
DLD	SNX11	0.51936567	1.1E-13
DLD	UBE2V1	0.51939578	1.1E-13
DLD	MED18	0.51955829	1.08E-13
DLD	PRNP	0.51970431	1.06E-13
DLD	SLC35A1	0.51977519	1.05E-13
DLD	MAP4K4	0.51993752	1.03E-13
DLD	ACAA2	0.51994435	1.03E-13
DLD	SNX17	0.52002674	1.01E-13
DLD	JOSD1	0.52002713	1.01E-13
DLD	LGALSL	0.52009218	1.01E-13
DLD	RCBTB2	0.52029887	9.8E-14
DLD	SNN	0.52036696	9.71E-14
DLD	SEC11A	0.52043414	9.63E-14
DLD	KIF20B	0.52043474	9.63E-14
DLD	ZNF777	0.52053859	9.5E-14
DLD	FZR1	0.52058802	9.44E-14
DLD	DYNLT3	0.52059575	9.43E-14
DLD	LIMCH1	0.52063415	9.39E-14
DLD	NOL7	0.52063578	9.39E-14
DLD	SCRN1	0.52067259	9.34E-14
DLD	PTPRE	0.52080212	9.19E-14
DLD	UBE2K	0.52086104	9.12E-14
DLD	CPPED1	0.5208884	9.09E-14
DLD	LSM12	0.5210193	8.94E-14
DLD	TRMT6	0.52107296	8.88E-14
DLD	ARFIP1	0.52117298	8.76E-14

DLD	AGPAT1	0.52119811	8.74E-14
DLD	XRCC6	0.52120961	8.72E-14
DLD	DLG5	0.52137271	8.54E-14
DLD	HIP1	0.52148705	8.42E-14
DLD	DAXX	0.52151017	8.39E-14
DLD	NUCKS1	0.5215581	8.34E-14
DLD	PARVA	0.52160167	8.3E-14
DLD	STX6	0.52169156	8.2E-14
DLD	MTCH2	0.5218929	7.99E-14
DLD	IFIH1	0.52192267	7.96E-14
DLD	LIMK2	0.52206166	7.82E-14
DLD	FAM210A	0.52214081	7.74E-14
DLD	VPS18	0.52244161	7.45E-14
DLD	RNASEL	0.522689	7.22E-14
DLD	FH	0.52273563	7.17E-14
DLD	RHOU	0.52295209	6.98E-14
DLD	TIAM2	0.52296645	6.96E-14
DLD	ADGRF5	0.52296701	6.96E-14
DLD	PDK3	0.52304439	6.9E-14
DLD	APEX2	0.52318571	6.77E-14
DLD	CDC42SE1	0.52325916	6.71E-14
DLD	NCAPD2	0.52326391	6.7E-14
DLD	TRIM13	0.52327499	6.69E-14
DLD	B2M	0.52336183	6.62E-14
DLD	MOSPD1	0.52346401	6.53E-14
DLD	MEIS2	0.52347286	6.53E-14
DLD	C12orf65	0.52352825	6.48E-14
DLD	MAP1LC3B2	0.52368314	6.35E-14
DLD	LRRC42	0.5238083	6.25E-14
DLD	KANK2	0.52384747	6.22E-14
DLD	ZPR1	0.52425694	5.9E-14
DLD	HMGN1	0.52445913	5.75E-14
DLD	TBC1D25	0.52453974	5.69E-14
DLD	BTG1	0.52455797	5.67E-14
DLD	SNRPB2	0.52466768	5.59E-14
DLD	CRYBG1	0.52467468	5.59E-14
DLD	F2R	0.52489983	5.43E-14
DLD	LLPH	0.52524477	5.19E-14
DLD	PTBP1	0.52528481	5.16E-14
DLD	OSBPL10	0.5255533	4.99E-14
DLD	TLR4	0.52558878	4.96E-14
DLD	NEURL1B	0.52563116	4.94E-14
DLD	AKIP1	0.52563773	4.93E-14
DLD	ROBO1	0.52569266	4.9E-14
DLD	RPA2	0.52569636	4.9E-14
DLD	DHFR	0.5257475	4.86E-14
DLD	KLHL29	0.52595304	4.74E-14
DLD	PIP4K2A	0.52609699	4.65E-14
DLD	TFG	0.52615314	4.61E-14
DLD	MYCBP	0.52619723	4.59E-14
DLD	MANBA	0.52620766	4.58E-14
DLD	GTF2B	0.5262837	4.54E-14
DLD	SH3RF1	0.52633564	4.51E-14
DLD	NEDD9	0.526518	4.4E-14
DLD	XRCC1	0.52666618	4.32E-14
DLD	SLC35F6	0.52678874	4.25E-14
DLD	TMCO3	0.52683513	4.22E-14
DLD	COPS5	0.52708108	4.09E-14

DLD	POLR2K	0.52711312	4.07E-14
DLD	ARRDC3	0.52723035	4.01E-14
DLD	HS1BP3	0.52723079	4.01E-14
DLD	PELO	0.52728833	3.98E-14
DLD	PAIP1	0.52729654	3.98E-14
DLD	C8orf33	0.52747204	3.89E-14
DLD	HOMER1	0.52754237	3.85E-14
DLD	ZC3HC1	0.5275627	3.84E-14
DLD	DDX27	0.52774356	3.75E-14
DLD	SLC25A11	0.52790082	3.67E-14
DLD	UTP25	0.52796577	3.64E-14
DLD	TOR3A	0.52808281	3.59E-14
DLD	TMEM59	0.5281126	3.57E-14
DLD	TCOF1	0.52835178	3.46E-14
DLD	TMEM165	0.52840071	3.44E-14
DLD	ZDHHC13	0.52841393	3.44E-14
DLD	KDELR2	0.52843507	3.43E-14
DLD	MTERF3	0.52844399	3.42E-14
DLD	TMED4	0.52846911	3.41E-14
DLD	SCAMP2	0.52848416	3.4E-14
DLD	KNOP1	0.52879063	3.27E-14
DLD	FARS2	0.52884959	3.25E-14
DLD	CISD2	0.52889013	3.23E-14
DLD	SVIP	0.52912359	3.13E-14
DLD	FRY	0.52916949	3.11E-14
DLD	RALA	0.52917229	3.11E-14
DLD	AHR	0.52919428	3.1E-14
DLD	PCSK7	0.52930613	3.06E-14
DLD	KIF3A	0.52933284	3.05E-14
DLD	MYH10	0.52944374	3E-14
DLD	CLIC4	0.52947368	2.99E-14
DLD	DDAH1	0.52956464	2.96E-14
DLD	USP39	0.5296692	2.91E-14
DLD	HHAT	0.52973767	2.89E-14
DLD	POLDIP3	0.52977807	2.87E-14
DLD	PLEKHF2	0.52981531	2.86E-14
DLD	ITPR1	0.52992168	2.82E-14
DLD	RALB	0.52995055	2.81E-14
DLD	WEE1	0.52997596	2.8E-14
DLD	PEF1	0.53001349	2.79E-14
DLD	RMDN2	0.53011361	2.75E-14
DLD	ARF4	0.53014401	2.74E-14
DLD	THAP11	0.53039191	2.65E-14
DLD	GLT8D1	0.53050173	2.61E-14
DLD	IQCE	0.53054704	2.6E-14
DLD	RASGEF1B	0.53064251	2.56E-14
DLD	KIAA0586	0.53065213	2.56E-14
DLD	PSMD2	0.53070194	2.54E-14
DLD	FNBP1	0.53074989	2.53E-14
DLD	CASP8	0.5307717	2.52E-14
DLD	TCFL5	0.53083783	2.5E-14
DLD	NGRN	0.53085267	2.49E-14
DLD	CTNNA1	0.53086156	2.49E-14
DLD	PCTP	0.53097367	2.45E-14
DLD	SAE1	0.53101551	2.44E-14
DLD	STAMBP	0.53111255	2.41E-14
DLD	WDR12	0.53113848	2.4E-14
DLD	BCORL1	0.53141989	2.31E-14

DLD	KLF7	0.53143813	2.31E-14
DLD	CNPPD1	0.53145097	2.3E-14
DLD	TMEM123	0.53145495	2.3E-14
DLD	GTPBP10	0.5315076	2.29E-14
DLD	PXN	0.53151671	2.28E-14
DLD	RAB9A	0.53156278	2.27E-14
DLD	SPIDR	0.53173007	2.22E-14
DLD	EIF2S2	0.53181627	2.2E-14
DLD	RMDN3	0.53186655	2.18E-14
DLD	ARPC4	0.53190491	2.17E-14
DLD	GASK1B	0.5319081	2.17E-14
DLD	PLS1	0.53208094	2.12E-14
DLD	DNAJC2	0.53210795	2.11E-14
DLD	WASHC3	0.53216056	2.1E-14
DLD	DNASE1L1	0.53216819	2.1E-14
DLD	APMAP	0.53225051	2.07E-14
DLD	CPED1	0.53229756	2.06E-14
DLD	ATXN10	0.53233557	2.05E-14
DLD	RSU1	0.53240264	2.03E-14
DLD	AK2	0.53261721	1.97E-14
DLD	SIMC1	0.53273274	1.94E-14
DLD	ORAI2	0.53276679	1.94E-14
DLD	ITPK1	0.53282606	1.92E-14
DLD	LTA4H	0.53283492	1.92E-14
DLD	ZNF281	0.53296048	1.89E-14
DLD	PDCL3	0.53304236	1.87E-14
DLD	SAP30	0.53306723	1.86E-14
DLD	ABHD6	0.53307368	1.86E-14
DLD	PIK3R2	0.53311073	1.85E-14
DLD	PIP4K2B	0.53317907	1.83E-14
DLD	EXOSC9	0.53325414	1.81E-14
DLD	CD2AP	0.53346958	1.76E-14
DLD	SPIRE1	0.53356157	1.74E-14
DLD	ITGA9	0.53364947	1.72E-14
DLD	AQR	0.53374057	1.7E-14
DLD	GALNT1	0.53404338	1.63E-14
DLD	GSR	0.53408846	1.62E-14
DLD	HDGFL3	0.5342066	1.6E-14
DLD	TTC39B	0.53438229	1.56E-14
DLD	PRPF38A	0.53438652	1.56E-14
DLD	DERA	0.53455282	1.53E-14
DLD	TMTC1	0.53462341	1.51E-14
DLD	METTL4	0.53480259	1.48E-14
DLD	ACACA	0.53500174	1.44E-14
DLD	AFTPH	0.53502883	1.43E-14
DLD	SDHB	0.53511961	1.42E-14
DLD	CCDC6	0.53523956	1.39E-14
DLD	STEAP2	0.53529791	1.38E-14
DLD	TAF8	0.53532763	1.38E-14
DLD	DPYSL2	0.53554913	1.34E-14
DLD	MYH9	0.53557015	1.33E-14
DLD	ITGAV	0.5356438	1.32E-14
DLD	SIAE	0.53567726	1.31E-14
DLD	NUP107	0.53601976	1.25E-14
DLD	GALNT7	0.53603098	1.25E-14
DLD	ETS2	0.53617564	1.23E-14
DLD	NAMPT	0.53624869	1.22E-14
DLD	TES	0.53636139	1.2E-14

DLD	ARL6IP5	0.53652429	1.17E-14
DLD	PSIP1	0.53672618	1.14E-14
DLD	ISCU	0.53681669	1.13E-14
DLD	QKI	0.53687554	1.12E-14
DLD	AP3B1	0.5368795	1.12E-14
DLD	NOL4L	0.53699558	1.1E-14
DLD	RAB23	0.53702053	1.1E-14
DLD	KDM5B	0.5371569	1.08E-14
DLD	FRMD4B	0.53752952	1.02E-14
DLD	METTL2A	0.53799354	9.62E-15
DLD	DCUN1D3	0.5380505	9.54E-15
DLD	FXR1	0.53807927	9.51E-15
DLD	APEX1	0.53817177	9.39E-15
DLD	RIOX1	0.53833267	9.19E-15
DLD	ARHGAP11A	0.53841314	9.09E-15
DLD	CASK	0.53850728	8.97E-15
DLD	FAHD1	0.53856113	8.91E-15
DLD	DIPK2A	0.53864105	8.81E-15
DLD	CALM3	0.53899732	8.4E-15
DLD	CEBPG	0.53905571	8.33E-15
DLD	DEPTOR	0.53939219	7.96E-15
DLD	SNAPC5	0.53966452	7.67E-15
DLD	DPYD	0.53980339	7.53E-15
DLD	ACVR2A	0.53985238	7.48E-15
DLD	PRKACB	0.53989632	7.43E-15
DLD	TMX4	0.53991499	7.41E-15
DLD	PCDH18	0.54002329	7.3E-15
DLD	NCSTN	0.54004337	7.28E-15
DLD	GPATCH2	0.54014646	7.18E-15
DLD	MED8	0.54038544	6.95E-15
DLD	ACTN1	0.54048471	6.86E-15
DLD	BFAR	0.54056434	6.79E-15
DLD	HSPA9	0.5405777	6.77E-15
DLD	SGO2	0.54076117	6.61E-15
DLD	WASHC5	0.54085615	6.52E-15
DLD	PIK3R3	0.54099922	6.4E-15
DLD	ZNF250	0.54107607	6.33E-15
DLD	MYLK	0.54119139	6.23E-15
DLD	EXO5	0.54121298	6.21E-15
DLD	URM1	0.54133494	6.11E-15
DLD	ETS1	0.54137736	6.07E-15
DLD	RCOR1	0.54152803	5.95E-15
DLD	LMNB1	0.5416319	5.87E-15
DLD	TPR	0.54169585	5.81E-15
DLD	BBS12	0.54170123	5.81E-15
DLD	EPB41L3	0.54176813	5.76E-15
DLD	CDKL1	0.54182405	5.71E-15
DLD	PLCG1	0.54194251	5.62E-15
DLD	TAF12	0.54198028	5.59E-15
DLD	TAF7	0.54211707	5.49E-15
DLD	RANBP3	0.54259729	5.14E-15
DLD	JADE2	0.54283819	4.97E-15
DLD	TSPAN14	0.54285305	4.96E-15
DLD	BAG4	0.54291994	4.92E-15
DLD	CLNS1A	0.54302695	4.85E-15
DLD	VOPP1	0.54306826	4.82E-15
DLD	KIFAP3	0.54309318	4.8E-15
DLD	MRPS35	0.54349441	4.54E-15

DLD	EPS8	0.54350005	4.54E-15
DLD	ATXN7L3	0.54370355	4.42E-15
DLD	JRKL	0.54372258	4.4E-15
DLD	NR1D2	0.54376915	4.38E-15
DLD	LANCL2	0.54383349	4.34E-15
DLD	LHFPL2	0.54392327	4.28E-15
DLD	GTPBP4	0.54403175	4.22E-15
DLD	FOXP1	0.54407469	4.2E-15
DLD	ATP6V1G1	0.54419347	4.13E-15
DLD	ATP11C	0.54434834	4.04E-15
DLD	ZNF267	0.54437244	4.03E-15
DLD	DYNLL2	0.54441576	4E-15
DLD	NXT2	0.54447197	3.97E-15
DLD	NDUFAF1	0.54449921	3.96E-15
DLD	RRAGA	0.54459388	3.91E-15
DLD	MDH1	0.54486691	3.76E-15
DLD	TTC1	0.545016	3.68E-15
DLD	ZNF564	0.54517835	3.6E-15
DLD	SLK	0.54524291	3.57E-15
DLD	GUCY1A1	0.54530137	3.54E-15
DLD	FTSJ3	0.5454337	3.48E-15
DLD	ATP6V1E1	0.54557835	3.41E-15
DLD	JAZF1	0.54564239	3.38E-15
DLD	CES2	0.5457054	3.35E-15
DLD	PGAM1	0.54589132	3.26E-15
DLD	ZC3HAV1	0.54595824	3.23E-15
DLD	SLC37A1	0.54605612	3.19E-15
DLD	OSTF1	0.54606211	3.19E-15
DLD	POLR3C	0.5462798	3.09E-15
DLD	RALBP1	0.54638056	3.05E-15
DLD	L3MBTL3	0.54657061	2.97E-15
DLD	PSMD14	0.54660361	2.96E-15
DLD	RALGPS2	0.54682512	2.87E-15
DLD	JARID2	0.54683142	2.86E-15
DLD	ANKRD42	0.54690243	2.84E-15
DLD	COQ2	0.5469233	2.83E-15
DLD	CXorf38	0.54698302	2.8E-15
DLD	JMJD1C	0.54704209	2.78E-15
DLD	SMG6	0.54723036	2.71E-15
DLD	RAVER1	0.54723422	2.71E-15
DLD	C4orf33	0.54742198	2.64E-15
DLD	CAMSAP2	0.54771791	2.53E-15
DLD	OPA3	0.54774319	2.52E-15
DLD	ZNF816	0.54792633	2.46E-15
DLD	HNRNPAB	0.54795319	2.45E-15
DLD	TSPAN12	0.54797628	2.44E-15
DLD	DNAJA1	0.54800757	2.43E-15
DLD	VDAC1	0.54805274	2.42E-15
DLD	SKI	0.54812132	2.39E-15
DLD	SLC39A6	0.54823694	2.35E-15
DLD	MOAP1	0.54839039	2.3E-15
DLD	HNRNPUL2	0.54844131	2.29E-15
DLD	ZMAT3	0.5484773	2.28E-15
DLD	TMEM9B	0.54849453	2.27E-15
DLD	NRBP1	0.54855163	2.25E-15
DLD	SGPP2	0.54857793	2.25E-15
DLD	FIP1L1	0.54886309	2.16E-15
DLD	OXSR1	0.54894864	2.13E-15

DLD	RACGAP1	0.54923466	2.05E-15
DLD	COPZ1	0.5494359	1.99E-15
DLD	UBAP2L	0.54962783	1.94E-15
DLD	ATL3	0.54968886	1.92E-15
DLD	BROX	0.54970155	1.92E-15
DLD	RMDN1	0.5497254	1.91E-15
DLD	PLS3	0.54982876	1.88E-15
DLD	SINHCAF	0.54995496	1.85E-15
DLD	AMMECR1	0.55008741	1.82E-15
DLD	TTI2	0.55018217	1.79E-15
DLD	SUFU	0.55048578	1.72E-15
DLD	ZDHHC5	0.55051877	1.71E-15
DLD	STK3	0.55051974	1.71E-15
DLD	SH3PXD2A	0.55053467	1.71E-15
DLD	SEC14L1	0.55060538	1.69E-15
DLD	AHCYL2	0.55066257	1.68E-15
DLD	CHMP1B	0.55071279	1.66E-15
DLD	SMC4	0.55074782	1.66E-15
DLD	THUMPD3	0.55078841	1.65E-15
DLD	MRM2	0.55084605	1.63E-15
DLD	EHF	0.55090821	1.62E-15
DLD	ZDHHC20	0.55109803	1.58E-15
DLD	SLC38A1	0.55120139	1.55E-15
DLD	TMED8	0.55128329	1.54E-15
DLD	ARHGAP42	0.55136966	1.52E-15
DLD	MINDY2	0.55146079	1.5E-15
DLD	REST	0.55149261	1.49E-15
DLD	SUMO3	0.55150485	1.49E-15
DLD	ZNF217	0.55162977	1.46E-15
DLD	ELP3	0.55196921	1.39E-15
DLD	KSR1	0.55206129	1.38E-15
DLD	IK	0.55206191	1.38E-15
DLD	PGD	0.55217466	1.35E-15
DLD	C6orf47	0.55220128	1.35E-15
DLD	CAPZB	0.55242201	1.31E-15
DLD	TBC1D9	0.55245131	1.3E-15
DLD	PTK2	0.55251533	1.29E-15
DLD	ABLIM1	0.55251693	1.29E-15
DLD	CPOX	0.55280336	1.24E-15
DLD	NUP133	0.5528271	1.23E-15
DLD	MAP4	0.55285285	1.23E-15
DLD	EMC1	0.55306086	1.19E-15
DLD	TMEM182	0.55317965	1.17E-15
DLD	ELK4	0.55320407	1.17E-15
DLD	COQ5	0.55335359	1.15E-15
DLD	TRIM2	0.55363096	1.1E-15
DLD	HSP90AA1	0.55364095	1.1E-15
DLD	TMEM183A	0.55364643	1.1E-15
DLD	PPT1	0.55366165	1.1E-15
DLD	ZNF131	0.55369345	1.09E-15
DLD	GNAI1	0.55401156	1.04E-15
DLD	CHRAC1	0.55402437	1.04E-15
DLD	ZCCHC17	0.55411302	1.03E-15
DLD	UBAP1	0.55418477	1.02E-15
DLD	PER2	0.55419967	1.02E-15
DLD	AIMP2	0.55462327	9.56E-16
DLD	SNX29	0.5546363	9.54E-16
DLD	PTP4A2	0.5550366	9.01E-16

DLD	DCP2	0.55511178	8.91E-16
DLD	PPCS	0.55512497	8.9E-16
DLD	LIMA1	0.55516207	8.85E-16
DLD	RUFY1	0.55518578	8.82E-16
DLD	GLUD1	0.55521808	8.78E-16
DLD	RPF1	0.55530091	8.67E-16
DLD	FAM122B	0.55533074	8.64E-16
DLD	NID1	0.5554015	8.55E-16
DLD	C4orf3	0.55556072	8.36E-16
DLD	NKIRAS2	0.55562493	8.28E-16
DLD	GSKIP	0.55566913	8.23E-16
DLD	REEP5	0.55569243	8.2E-16
DLD	MLLT3	0.55606762	7.77E-16
DLD	UBA1	0.55637445	7.44E-16
DLD	MICU1	0.55655359	7.25E-16
DLD	SOAT1	0.55657322	7.23E-16
DLD	DAG1	0.55665308	7.15E-16
DLD	RAB29	0.55668945	7.11E-16
DLD	BTF3L4	0.55679169	7E-16
DLD	SRXN1	0.5568779	6.92E-16
DLD	INPP5A	0.55688869	6.91E-16
DLD	PSMC1	0.55698691	6.81E-16
DLD	EHD4	0.55715375	6.65E-16
DLD	VCL	0.55738937	6.43E-16
DLD	ELK1	0.55740298	6.41E-16
DLD	HEATR5A	0.55741935	6.4E-16
DLD	PRPS1	0.55756054	6.27E-16
DLD	TMEM237	0.55757217	6.26E-16
DLD	AMFR	0.5576628	6.18E-16
DLD	BCL7B	0.55770525	6.14E-16
DLD	TM2D3	0.55771228	6.14E-16
DLD	SLC25A12	0.55773338	6.12E-16
DLD	ACSL3	0.55785831	6.01E-16
DLD	ARMC10	0.55813367	5.77E-16
DLD	GLTP	0.55855149	5.43E-16
DLD	NLN	0.55855726	5.43E-16
DLD	CEP250	0.55871346	5.31E-16
DLD	MAN2B2	0.55875187	5.28E-16
DLD	ERGIC2	0.55897891	5.11E-16
DLD	RAPH1	0.55903508	5.07E-16
DLD	PEX13	0.55924059	4.92E-16
DLD	TRAM1	0.5594423	4.78E-16
DLD	KLF3	0.55947118	4.76E-16
DLD	FKBP9	0.55961273	4.66E-16
DLD	NUP37	0.55965559	4.63E-16
DLD	ZMYM5	0.55967612	4.62E-16
DLD	ZNF134	0.55984421	4.51E-16
DLD	ST3GAL2	0.559902	4.47E-16
DLD	AGA	0.55995392	4.44E-16
DLD	TMEM251	0.56032996	4.2E-16
DLD	CNOT11	0.56041775	4.15E-16
DLD	CACNA2D1	0.56050299	4.09E-16
DLD	CYP2U1	0.56071197	3.97E-16
DLD	PIK3C3	0.56071341	3.97E-16
DLD	KATNAL1	0.5609678	3.83E-16
DLD	IPPK	0.56097731	3.82E-16
DLD	ANKRD50	0.56106603	3.77E-16
DLD	PARP8	0.56106808	3.77E-16

DLD	PRMT2	0.56110211	3.75E-16
DLD	PHF23	0.56110604	3.75E-16
DLD	NSFL1C	0.56177947	3.4E-16
DLD	AHNAK	0.56180874	3.38E-16
DLD	AFAP1	0.56188174	3.35E-16
DLD	VCP	0.56192954	3.33E-16
DLD	PTGES3	0.56195041	3.32E-16
DLD	SPTLC2	0.56201143	3.29E-16
DLD	TPM3	0.56203503	3.27E-16
DLD	ITGA1	0.5620633	3.26E-16
DLD	NOCT	0.56223351	3.18E-16
DLD	SVIL	0.56225478	3.17E-16
DLD	GEMIN2	0.56230034	3.15E-16
DLD	TRUB2	0.56234183	3.13E-16
DLD	ABHD2	0.56244036	3.09E-16
DLD	NPLOC4	0.56259488	3.02E-16
DLD	TPD52	0.56261093	3.01E-16
DLD	PTP4A1	0.56263655	3E-16
DLD	EXTL3	0.56274862	2.95E-16
DLD	SRF	0.56276109	2.94E-16
DLD	PDE4DIP	0.56301069	2.84E-16
DLD	LASP1	0.56327662	2.73E-16
DLD	CMAS	0.56345979	2.66E-16
DLD	MRPL50	0.56357905	2.61E-16
DLD	SLC25A43	0.56361205	2.6E-16
DLD	EIF1B	0.56364429	2.59E-16
DLD	LAMC1	0.56370906	2.56E-16
DLD	CSTF2	0.56390752	2.49E-16
DLD	REEP3	0.5640006	2.45E-16
DLD	CTPS1	0.56403742	2.44E-16
DLD	PDE6D	0.56405239	2.43E-16
DLD	RBM47	0.56418732	2.39E-16
DLD	MBNL3	0.56430829	2.34E-16
DLD	URGCP	0.56431492	2.34E-16
DLD	PPP3CA	0.56442851	2.3E-16
DLD	DUSP18	0.56444295	2.3E-16
DLD	L3MBTL2	0.56466369	2.23E-16
DLD	TMEM185B	0.56469293	2.22E-16
DLD	DPM1	0.56488432	2.15E-16
DLD	E2F3	0.5649912	2.12E-16
DLD	CAP1	0.56520251	2.05E-16
DLD	ACTR1A	0.56528735	2.03E-16
DLD	TWSG1	0.56546436	1.98E-16
DLD	FAM104A	0.56548251	1.97E-16
DLD	SH3KBP1	0.56548835	1.97E-16
DLD	FAM177A1	0.5656063	1.94E-16
DLD	TAF6	0.56562767	1.93E-16
DLD	RNF139	0.56569933	1.91E-16
DLD	ZBTB9	0.56589062	1.86E-16
DLD	AC008764.4	0.56610682	1.8E-16
DLD	CAPZA1	0.5662746	1.75E-16
DLD	XYLT1	0.56632539	1.74E-16
DLD	BTN3A1	0.56634904	1.73E-16
DLD	TCEAL8	0.56642626	1.71E-16
DLD	WIPI2	0.56649128	1.7E-16
DLD	POLE3	0.56650663	1.69E-16
DLD	NAPG	0.56670563	1.64E-16
DLD	HECA	0.56673996	1.64E-16

DLD	TAF9	0.56680374	1.62E-16
DLD	IDS	0.56684444	1.61E-16
DLD	SEH1L	0.56690775	1.6E-16
DLD	TSPAN31	0.56692783	1.59E-16
DLD	SNX12	0.567083	1.55E-16
DLD	ZNF629	0.56726742	1.51E-16
DLD	NIPA1	0.56733706	1.5E-16
DLD	DARS2	0.56734983	1.49E-16
DLD	SELENOP	0.56740035	1.48E-16
DLD	DEGS1	0.56742184	1.48E-16
DLD	INTS5	0.56744142	1.47E-16
DLD	RASA1	0.56769627	1.42E-16
DLD	ZWILCH	0.56782884	1.39E-16
DLD	PSMC6	0.56795766	1.36E-16
DLD	SUPT16H	0.56805566	1.34E-16
DLD	GNB5	0.56814243	1.33E-16
DLD	SHROOM3	0.56815759	1.32E-16
DLD	AMBRA1	0.56824123	1.31E-16
DLD	AAGAB	0.56829394	1.3E-16
DLD	METTL21A	0.56843663	1.27E-16
DLD	ASH2L	0.56845924	1.27E-16
DLD	CALM2	0.56847774	1.26E-16
DLD	GK	0.56854432	1.25E-16
DLD	SLC38A7	0.56873338	1.22E-16
DLD	ADAM9	0.56873918	1.21E-16
DLD	SDHAF3	0.56880908	1.2E-16
DLD	SLC44A1	0.56885068	1.19E-16
DLD	KLHL23	0.56899901	1.17E-16
DLD	TFRC	0.56914102	1.14E-16
DLD	UXS1	0.56917448	1.14E-16
DLD	PREP	0.56920009	1.13E-16
DLD	CTNNB1	0.56930581	1.12E-16
DLD	BTN3A3	0.56935995	1.11E-16
DLD	TMEM126B	0.5693775	1.1E-16
DLD	ANP32E	0.56937754	1.1E-16
DLD	RCN2	0.56943326	1.09E-16
DLD	DNAJC8	0.5694921	1.08E-16
DLD	SRGAP2B	0.56950895	1.08E-16
DLD	CDC42BPA	0.56954416	1.08E-16
DLD	LACTB2	0.56974636	1.04E-16
DLD	ARL6IP1	0.5697767	1.04E-16
DLD	TMEM38B	0.56980415	1.03E-16
DLD	RIN2	0.5699756	1.01E-16
DLD	TSFM	0.57000739	1E-16
DLD	RMND5A	0.57008468	9.92E-17
DLD	AP1B1	0.57010503	9.89E-17
DLD	ATG9A	0.57020759	9.74E-17
DLD	SCOC	0.57024622	9.68E-17
DLD	ASAP1	0.57027754	9.64E-17
DLD	NIP7	0.57029758	9.61E-17
DLD	RAB27A	0.5703978	9.47E-17
DLD	CNOT2	0.57053989	9.27E-17
DLD	AKIRIN2	0.57070649	9.04E-17
DLD	TTC4	0.57093144	8.73E-17
DLD	SLBP	0.57107782	8.54E-17
DLD	CSGALNACT2	0.57111067	8.5E-17
DLD	BORCS7	0.57111809	8.49E-17
DLD	ALKBH1	0.57128731	8.28E-17

DLD	SPCS2	0.57129983	8.26E-17
DLD	ANKRD11	0.57141671	8.12E-17
DLD	NT5C3A	0.57169386	7.79E-17
DLD	FAM114A1	0.57171617	7.76E-17
DLD	EPB41L4A	0.57174359	7.73E-17
DLD	TMEM128	0.57186983	7.58E-17
DLD	PAK1IP1	0.57209718	7.32E-17
DLD	MRFAP1L1	0.57220181	7.21E-17
DLD	POC1B	0.5722202	7.19E-17
DLD	CALU	0.57235552	7.04E-17
DLD	SLC25A17	0.57237877	7.02E-17
DLD	ERMP1	0.57238184	7.02E-17
DLD	ESD	0.57243703	6.96E-17
DLD	ZEB1	0.572547	6.84E-17
DLD	EXOC6	0.57269587	6.69E-17
DLD	ME1	0.57282341	6.56E-17
DLD	TMLHE	0.57284884	6.54E-17
DLD	CIR1	0.57292046	6.47E-17
DLD	CLP1	0.57302213	6.37E-17
DLD	OXCT1	0.5732141	6.18E-17
DLD	CFAP20	0.57322449	6.17E-17
DLD	AP1S2	0.57323589	6.16E-17
DLD	ANO6	0.57338418	6.03E-17
DLD	BNIP3L	0.57341511	6E-17
DLD	CORO1C	0.57345549	5.96E-17
DLD	ENSA	0.57357826	5.85E-17
DLD	LYST	0.57370682	5.74E-17
DLD	CCT8	0.57375956	5.69E-17
DLD	SH2B3	0.57381391	5.64E-17
DLD	MRFAP1	0.57387797	5.59E-17
DLD	ACOT2	0.5739291	5.55E-17
DLD	MB21D2	0.57401551	5.47E-17
DLD	STX3	0.57406529	5.43E-17
DLD	NDFIP1	0.57408457	5.42E-17
DLD	OXNAD1	0.57409826	5.4E-17
DLD	RNF168	0.57437282	5.18E-17
DLD	PHTF1	0.57445758	5.12E-17
DLD	UBE2Q2	0.57453343	5.06E-17
DLD	SIPA1L3	0.574541	5.05E-17
DLD	PCNX1	0.57454892	5.05E-17
DLD	BBS2	0.57456155	5.04E-17
DLD	HNRNPD	0.57462489	4.99E-17
DLD	RUNDC1	0.5749764	4.73E-17
DLD	IFT52	0.57506018	4.67E-17
DLD	SYS1	0.57516253	4.59E-17
DLD	VPS26A	0.57520906	4.56E-17
DLD	MUL1	0.57554964	4.33E-17
DLD	PAICS	0.575683	4.24E-17
DLD	UNC50	0.57570318	4.23E-17
DLD	SPG21	0.57580247	4.17E-17
DLD	CLDN12	0.57595384	4.07E-17
DLD	SSRP1	0.57596709	4.06E-17
DLD	KLHL12	0.57608028	3.99E-17
DLD	TAF9B	0.5761909	3.92E-17
DLD	RNGTT	0.57621251	3.91E-17
DLD	DENND1C	0.57634472	3.83E-17
DLD	RBM43	0.57675578	3.6E-17
DLD	MASTL	0.57679362	3.58E-17

DLD	PTPRK	0.57690319	3.52E-17
DLD	ZDHHC9	0.57705388	3.44E-17
DLD	ARPC5	0.57708917	3.42E-17
DLD	SH3BGRL2	0.57714467	3.39E-17
DLD	PELI1	0.57728543	3.32E-17
DLD	TMEM87A	0.57744633	3.24E-17
DLD	ANKRD27	0.57761623	3.15E-17
DLD	SNX7	0.57764597	3.14E-17
DLD	SMARCAL1	0.57777983	3.07E-17
DLD	OLA1	0.57781	3.06E-17
DLD	HNRNPA1	0.57781867	3.05E-17
DLD	CDKN1B	0.57790549	3.01E-17
DLD	SMIM13	0.57806381	2.94E-17
DLD	TMED10	0.57820085	2.88E-17
DLD	LSM6	0.57833045	2.82E-17
DLD	SLC8A1	0.5786611	2.68E-17
DLD	KLHL5	0.57889675	2.59E-17
DLD	KLHL18	0.57900466	2.54E-17
DLD	TGFBR2	0.57911598	2.5E-17
DLD	RNF19B	0.57928177	2.44E-17
DLD	DEDD	0.57931994	2.42E-17
DLD	RAP1B	0.57945851	2.37E-17
DLD	BABAM2	0.57958147	2.33E-17
DLD	PTPRB	0.57962591	2.31E-17
DLD	DCLRE1B	0.57966632	2.29E-17
DLD	ASNSD1	0.57978632	2.25E-17
DLD	ITGB1	0.57983765	2.23E-17
DLD	LBR	0.57990783	2.21E-17
DLD	GPBP1	0.58001622	2.17E-17
DLD	BPGM	0.58005508	2.16E-17
DLD	ZNF766	0.58009197	2.15E-17
DLD	NCK1	0.58040773	2.04E-17
DLD	ATF6B	0.58041726	2.04E-17
DLD	SORT1	0.58050425	2.01E-17
DLD	TMCC3	0.58052029	2.01E-17
DLD	PRKD3	0.58054656	2E-17
DLD	DPH3	0.58056999	1.99E-17
DLD	ITPRIPL2	0.58071102	1.95E-17
DLD	CHORDC1	0.58075776	1.94E-17
DLD	PHF20	0.58081845	1.92E-17
DLD	XIAP	0.58083018	1.91E-17
DLD	DNAAF5	0.58086257	1.9E-17
DLD	GGPS1	0.58092526	1.89E-17
DLD	MESD	0.58099564	1.87E-17
DLD	IMMP2L	0.58099662	1.87E-17
DLD	PRKCA	0.58101445	1.86E-17
DLD	FGD4	0.58104872	1.85E-17
DLD	HSPE1-MOB4	0.58110753	1.83E-17
DLD	C12orf43	0.5811349	1.83E-17
DLD	TMBIM4	0.58117082	1.82E-17
DLD	TXNDC9	0.58120293	1.81E-17
DLD	STMP1	0.58126959	1.79E-17
DLD	CENPL	0.5812796	1.78E-17
DLD	NDE1	0.58138224	1.76E-17
DLD	TTC8	0.58151998	1.72E-17
DLD	NSMCE3	0.58170454	1.67E-17
DLD	HNRNPM	0.58173317	1.66E-17
DLD	ZFP90	0.5818331	1.64E-17

DLD	TULP3	0.5820057	1.59E-17
DLD	PXMP4	0.58217604	1.55E-17
DLD	MRPL30	0.5822133	1.54E-17
DLD	BBS7	0.58226029	1.53E-17
DLD	NAGA	0.58236773	1.51E-17
DLD	YTHDF1	0.58243555	1.49E-17
DLD	BACH1	0.58271006	1.43E-17
DLD	GBE1	0.58273816	1.42E-17
DLD	YEATS2	0.58289876	1.39E-17
DLD	SPDYE3	0.5829893	1.37E-17
DLD	BLZF1	0.58303574	1.36E-17
DLD	RRM1	0.58311692	1.34E-17
DLD	ARL6IP6	0.58322249	1.32E-17
DLD	BPNT1	0.58324861	1.31E-17
DLD	GINM1	0.58367616	1.23E-17
DLD	ZBTB4	0.5837621	1.21E-17
DLD	TMEM14A	0.58379025	1.2E-17
DLD	VPS29	0.58409685	1.15E-17
DLD	NUP155	0.58410021	1.15E-17
DLD	NFIX	0.58419319	1.13E-17
DLD	CLTC	0.58420252	1.13E-17
DLD	ADO	0.58421639	1.13E-17
DLD	RNF121	0.5842527	1.12E-17
DLD	MRPL44	0.58443528	1.09E-17
DLD	U2AF2	0.58475995	1.03E-17
DLD	COX7A2L	0.58477024	1.03E-17
DLD	PA2G4	0.58488325	1.01E-17
DLD	BBS9	0.58493842	1E-17
DLD	FBXO42	0.58525653	9.55E-18
DLD	DIDO1	0.58525686	9.55E-18
DLD	EIF2B2	0.58526865	9.53E-18
DLD	UBE2Z	0.58532765	9.44E-18
DLD	ABCC4	0.58540414	9.33E-18
DLD	TAF1A	0.58542021	9.31E-18
DLD	RNF26	0.58559266	9.06E-18
DLD	RIT1	0.5856006	9.04E-18
DLD	GOLPH3L	0.58570815	8.89E-18
DLD	DFFA	0.5858147	8.74E-18
DLD	ARF6	0.58589228	8.63E-18
DLD	HNRNPC	0.58620588	8.22E-18
DLD	KCTD21	0.58634858	8.03E-18
DLD	MEX3C	0.58642046	7.94E-18
DLD	ELF4	0.58655937	7.77E-18
DLD	KATNBL1	0.58679783	7.48E-18
DLD	APBB2	0.5868273	7.44E-18
DLD	NFYA	0.58687713	7.38E-18
DLD	LARGE1	0.58696978	7.28E-18
DLD	DIAPH2	0.58707153	7.16E-18
DLD	RNF170	0.58712338	7.1E-18
DLD	CMTM6	0.58732294	6.88E-18
DLD	ATP6V1B2	0.58744311	6.75E-18
DLD	UBE2Q1	0.58747085	6.72E-18
DLD	TPMT	0.58752233	6.66E-18
DLD	ANO10	0.58754358	6.64E-18
DLD	IVD	0.58763755	6.54E-18
DLD	DPY19L1	0.58783932	6.33E-18
DLD	NUDCD1	0.5880976	6.08E-18
DLD	ADD3	0.58816174	6.02E-18

DLD	SMIM12	0.58817716	6E-18
DLD	MTA2	0.58818828	5.99E-18
DLD	MSI2	0.58832372	5.86E-18
DLD	TSG101	0.58843932	5.75E-18
DLD	RHOA	0.58878619	5.44E-18
DLD	PLBD2	0.58885813	5.38E-18
DLD	RNPS1	0.588924	5.32E-18
DLD	ABCC1	0.58922034	5.08E-18
DLD	PTPRG	0.58927697	5.03E-18
DLD	EXOC1	0.58942676	4.91E-18
DLD	ISCA1	0.58945727	4.89E-18
DLD	TRAM2	0.58946331	4.88E-18
DLD	MRPS10	0.58947613	4.87E-18
DLD	LINS1	0.58954704	4.82E-18
DLD	SF3B3	0.58961146	4.77E-18
DLD	CCDC90B	0.58966786	4.72E-18
DLD	UNG	0.58967975	4.72E-18
DLD	PIP4K2C	0.58971955	4.69E-18
DLD	PARP4	0.58979964	4.63E-18
DLD	ATPSCKMT	0.58981374	4.62E-18
DLD	SURF4	0.58992812	4.53E-18
DLD	RRP15	0.58994825	4.52E-18
DLD	YWHAH	0.59020327	4.33E-18
DLD	OTUD7B	0.59022617	4.32E-18
DLD	CYB5B	0.59025404	4.3E-18
DLD	GABPB1	0.59030223	4.27E-18
DLD	KCTD3	0.59035032	4.23E-18
DLD	MAX	0.59068216	4.01E-18
DLD	ANP32A	0.59070316	4E-18
DLD	OSGIN2	0.59080333	3.94E-18
DLD	HIVEP2	0.5908213	3.92E-18
DLD	PATL1	0.59091474	3.87E-18
DLD	MAIP1	0.5909368	3.85E-18
DLD	RAB11A	0.59099881	3.81E-18
DLD	ETNK1	0.59116011	3.71E-18
DLD	FBXO22	0.59135178	3.6E-18
DLD	IQGAP1	0.5913558	3.6E-18
DLD	TGFBR1	0.59136673	3.59E-18
DLD	GNAI3	0.59153185	3.5E-18
DLD	TRIM69	0.59155708	3.48E-18
DLD	DHX9	0.59174853	3.38E-18
DLD	IFNGR1	0.59176423	3.37E-18
DLD	SWAP70	0.59231015	3.08E-18
DLD	SPRED1	0.59232709	3.08E-18
DLD	CBX5	0.59234098	3.07E-18
DLD	ZCRB1	0.59241029	3.03E-18
DLD	RCAN3	0.59250093	2.99E-18
DLD	DST	0.5925159	2.98E-18
DLD	TRIM37	0.59254928	2.97E-18
DLD	XRCC5	0.59264234	2.92E-18
DLD	UGDH	0.59276794	2.86E-18
DLD	GATAD2A	0.59279458	2.85E-18
DLD	ATP6V1D	0.59283042	2.83E-18
DLD	AIDA	0.5929018	2.8E-18
DLD	CD46	0.59320974	2.66E-18
DLD	PSMD7	0.59342554	2.57E-18
DLD	GRWD1	0.59353839	2.53E-18
DLD	ATP6V0D1	0.59363823	2.48E-18

DLD	ZCCHC10	0.59368314	2.47E-18
DLD	FNTB	0.59377807	2.43E-18
DLD	CDC73	0.5942581	2.25E-18
DLD	PTEN	0.59430543	2.23E-18
DLD	OSTM1	0.59445211	2.18E-18
DLD	TMX2	0.59449196	2.16E-18
DLD	FCHSD2	0.59458264	2.13E-18
DLD	DYNC2LI1	0.59459368	2.13E-18
DLD	TP53INP2	0.59475049	2.07E-18
DLD	ARHGAP1	0.59518552	1.93E-18
DLD	MAP1LC3B	0.59535035	1.88E-18
DLD	MRPS23	0.59540042	1.86E-18
DLD	ETFA	0.59551263	1.83E-18
DLD	ODF2	0.59555002	1.82E-18
DLD	SLC4A7	0.59555987	1.81E-18
DLD	PIAS1	0.59573603	1.76E-18
DLD	RNF220	0.59577118	1.75E-18
DLD	TP53BP2	0.59593002	1.71E-18
DLD	TLE4	0.59594547	1.7E-18
DLD	NEDD4	0.59600309	1.69E-18
DLD	MRPL3	0.59623891	1.62E-18
DLD	PLSCR4	0.59626353	1.62E-18
DLD	SIPA1L2	0.59631176	1.6E-18
DLD	FEZ2	0.59662312	1.52E-18
DLD	ACER3	0.59665624	1.52E-18
DLD	TMEM230	0.59666241	1.51E-18
DLD	CDS2	0.59679287	1.48E-18
DLD	PTPRJ	0.59682355	1.47E-18
DLD	ZNF260	0.59685454	1.47E-18
DLD	CKAP2	0.59688452	1.46E-18
DLD	SAR1A	0.5969158	1.45E-18
DLD	YWHAE	0.59731191	1.36E-18
DLD	PAFAH1B1	0.59743624	1.33E-18
DLD	NCDN	0.59765108	1.29E-18
DLD	CHMP5	0.59776909	1.26E-18
DLD	PGRMC1	0.59782194	1.25E-18
DLD	AK6	0.59792922	1.23E-18
DLD	MGAT5	0.59795376	1.22E-18
DLD	EIF1AD	0.59814633	1.19E-18
DLD	NEK7	0.59826927	1.16E-18
DLD	ATP11B	0.59830706	1.15E-18
DLD	SLC30A1	0.59909233	1.01E-18
DLD	IL6ST	0.59941232	9.61E-19
DLD	DDX19B	0.59945124	9.54E-19
DLD	KATNA1	0.5994584	9.53E-19
DLD	KCTD12	0.59964424	9.24E-19
DLD	DNAJC13	0.59964765	9.24E-19
DLD	ERGIC1	0.59974317	9.09E-19
DLD	ETV3	0.59979933	9.01E-19
DLD	ATP11A	0.5998998	8.86E-19
DLD	HIGD1A	0.60001721	8.69E-19
DLD	LACTB	0.60020145	8.42E-19
DLD	RAD18	0.60022926	8.38E-19
DLD	SLC26A2	0.60036862	8.19E-19
DLD	SSB	0.60040832	8.14E-19
DLD	CERS2	0.60053519	7.97E-19
DLD	UBXN2A	0.60061835	7.86E-19
DLD	TRIM26	0.60070799	7.74E-19

DLD	CHMP2B	0.60094303	7.44E-19
DLD	SORL1	0.60122265	7.1E-19
DLD	GRAMD2B	0.60122857	7.09E-19
DLD	BTBD10	0.60141218	6.88E-19
DLD	NIF3L1	0.60141954	6.87E-19
DLD	C14orf119	0.60147095	6.81E-19
DLD	NDC1	0.60147336	6.81E-19
DLD	RRAGC	0.60154136	6.73E-19
DLD	TMEM167A	0.60154302	6.73E-19
DLD	PLEKHG1	0.60186934	6.37E-19
DLD	FUT8	0.60196926	6.27E-19
DLD	TMTC3	0.60202038	6.21E-19
DLD	MED6	0.6021208	6.11E-19
DLD	GNA11	0.60233374	5.89E-19
DLD	ITSN1	0.60244142	5.79E-19
DLD	VDAC3	0.60274003	5.51E-19
DLD	SNX3	0.602791	5.46E-19
DLD	PKD2	0.60281577	5.44E-19
DLD	TOR1A	0.60282058	5.43E-19
DLD	NUDT15	0.60292416	5.34E-19
DLD	RASAL2	0.60295354	5.31E-19
DLD	USP34	0.60308696	5.19E-19
DLD	DPF2	0.60339231	4.93E-19
DLD	SP1	0.60347499	4.86E-19
DLD	PSMF1	0.60367582	4.7E-19
DLD	PDE4D	0.60387438	4.55E-19
DLD	ERI1	0.60398495	4.46E-19
DLD	PSEN1	0.60405733	4.41E-19
DLD	DOCK1	0.6041886	4.31E-19
DLD	LCLAT1	0.60420464	4.3E-19
DLD	PPTC7	0.60437017	4.18E-19
DLD	IPO11	0.60453596	4.07E-19
DLD	ARFGEF1	0.60457706	4.04E-19
DLD	C16orf87	0.60471392	3.95E-19
DLD	PAFAH1B2	0.60483811	3.86E-19
DLD	PCMT1	0.60501392	3.75E-19
DLD	TRANK1	0.60504229	3.73E-19
DLD	SNX5	0.60524253	3.61E-19
DLD	ZNF462	0.60538288	3.52E-19
DLD	TMEM199	0.60559962	3.39E-19
DLD	PPP1R12B	0.60563771	3.37E-19
DLD	HSD17B12	0.60571863	3.33E-19
DLD	BMP2K	0.60576209	3.3E-19
DLD	TFE3	0.60610882	3.11E-19
DLD	HMGB1	0.60625654	3.04E-19
DLD	RCC1L	0.60629554	3.02E-19
DLD	SMURF2	0.60632584	3E-19
DLD	TSHZ1	0.60637725	2.97E-19
DLD	SMG7	0.60654349	2.89E-19
DLD	SLU7	0.60674813	2.79E-19
DLD	CAMKK2	0.60675328	2.79E-19
DLD	MED21	0.60693033	2.71E-19
DLD	PIGW	0.60700585	2.67E-19
DLD	HMGXB3	0.60701743	2.67E-19
DLD	EXT1	0.60704749	2.65E-19
DLD	OTULIN	0.60705714	2.65E-19
DLD	NCAPG2	0.60709249	2.63E-19
DLD	EIF2AK1	0.60759358	2.42E-19

DLD	LIPA	0.60771508	2.37E-19
DLD	UTP3	0.60777191	2.34E-19
DLD	AGPAT5	0.60780133	2.33E-19
DLD	ATXN7L3B	0.6078964	2.29E-19
DLD	PGAM5	0.60802575	2.24E-19
DLD	TMEM263	0.6081507	2.2E-19
DLD	PALLD	0.60816355	2.19E-19
DLD	CCAR2	0.60831989	2.13E-19
DLD	MACF1	0.6084477	2.09E-19
DLD	PDCD10	0.60854149	2.05E-19
DLD	CDV3	0.60883919	1.95E-19
DLD	PLRG1	0.60908154	1.87E-19
DLD	TRA2B	0.60909103	1.87E-19
DLD	MBTPS1	0.60912746	1.86E-19
DLD	MAP3K20	0.60917803	1.84E-19
DLD	SIAH1	0.60936749	1.78E-19
DLD	SS18	0.60955743	1.72E-19
DLD	PCNX4	0.60957487	1.72E-19
DLD	MBNL1	0.60979563	1.66E-19
DLD	CPD	0.60983675	1.64E-19
DLD	BCL9L	0.60985724	1.64E-19
DLD	IDH1	0.60999392	1.6E-19
DLD	ZKSCAN5	0.60999963	1.6E-19
DLD	ABR	0.61005215	1.58E-19
DLD	ATXN1	0.61012585	1.56E-19
DLD	PDHB	0.61016662	1.55E-19
DLD	WDR43	0.61018224	1.55E-19
DLD	CNOT1	0.6102766	1.52E-19
DLD	ATF7IP	0.61040597	1.49E-19
DLD	WAPL	0.61112556	1.32E-19
DLD	CCDC43	0.61113278	1.31E-19
DLD	SPDL1	0.61143653	1.25E-19
DLD	IQCK	0.6115022	1.23E-19
DLD	YPEL5	0.61156933	1.22E-19
DLD	DIP2C	0.61171156	1.19E-19
DLD	LPP	0.61180386	1.17E-19
DLD	CAST	0.61197953	1.13E-19
DLD	RTN4	0.61202565	1.13E-19
DLD	LRCH3	0.61214178	1.1E-19
DLD	MFHAS1	0.61215144	1.1E-19
DLD	PACRGL	0.61218608	1.09E-19
DLD	RFK	0.61235885	1.06E-19
DLD	PTPN12	0.61239744	1.05E-19
DLD	RAB7A	0.61240978	1.05E-19
DLD	SLC18B1	0.61252187	1.03E-19
DLD	EAPP	0.61264516	1.01E-19
DLD	GPBP1L1	0.61265488	1.01E-19
DLD	ZNF623	0.61274078	9.93E-20
DLD	TM9SF3	0.61276284	9.9E-20
DLD	DESI2	0.61280491	9.82E-20
DLD	B4GALT5	0.6128288	9.78E-20
DLD	DERL1	0.61304059	9.43E-20
DLD	PCGF5	0.61326913	9.06E-20
DLD	TIGAR	0.61376295	8.31E-20
DLD	ZCCHC9	0.61391851	8.09E-20
DLD	TTC5	0.61408873	7.85E-20
DLD	MPZL1	0.61412306	7.8E-20
DLD	BCL9	0.61427963	7.59E-20

DLD	STARD4	0.6146762	7.08E-20
DLD	ENAH	0.61468327	7.07E-20
DLD	MBNL2	0.61481673	6.91E-20
DLD	GDI2	0.61483158	6.89E-20
DLD	SPRED2	0.61509943	6.58E-20
DLD	KIAA1671	0.61515349	6.51E-20
DLD	TMEM68	0.61519316	6.47E-20
DLD	PLAA	0.61523765	6.42E-20
DLD	DCAF6	0.61525997	6.39E-20
DLD	TTL	0.61531549	6.33E-20
DLD	GLE1	0.61561146	6.01E-20
DLD	TOR1B	0.61566772	5.95E-20
DLD	KDM2A	0.61582086	5.79E-20
DLD	TRIM14	0.61610923	5.5E-20
DLD	HFE	0.61618215	5.43E-20
DLD	SYAP1	0.6162752	5.35E-20
DLD	POGK	0.61638137	5.25E-20
DLD	GOLT1B	0.61644697	5.19E-20
DLD	SYNJ2	0.61672525	4.94E-20
DLD	STIM1	0.61684301	4.84E-20
DLD	SSH1	0.61685987	4.82E-20
DLD	RFC3	0.61689879	4.79E-20
DLD	OAZ2	0.61711915	4.6E-20
DLD	RAE1	0.61712235	4.6E-20
DLD	SH3GLB1	0.61748368	4.32E-20
DLD	CYLD	0.61767982	4.17E-20
DLD	CAMK2G	0.61779813	4.08E-20
DLD	CASP2	0.61783209	4.06E-20
DLD	NDRG3	0.61803321	3.92E-20
DLD	SOCS6	0.61821038	3.79E-20
DLD	PIP4P2	0.61839492	3.67E-20
DLD	RNF8	0.6185375	3.58E-20
DLD	ARHGAP21	0.61864741	3.51E-20
DLD	SGMS2	0.61869576	3.48E-20
DLD	NUDT3	0.61871973	3.47E-20
DLD	HNRNPA0	0.61886859	3.38E-20
DLD	BCAS2	0.61891541	3.35E-20
DLD	CCDC32	0.6197372	2.89E-20
DLD	HNRNPUL1	0.61982541	2.85E-20
DLD	ZNF174	0.61998622	2.77E-20
DLD	ADAT1	0.61998934	2.76E-20
DLD	ASCC3	0.62012987	2.69E-20
DLD	CD164	0.62048103	2.53E-20
DLD	VTI1A	0.62059322	2.48E-20
DLD	TTC26	0.62070079	2.43E-20
DLD	DBF4	0.62070732	2.43E-20
DLD	EMC3	0.6207428	2.41E-20
DLD	CBX1	0.62074414	2.41E-20
DLD	EMC2	0.62081041	2.39E-20
DLD	MINPP1	0.62092191	2.34E-20
DLD	CLN5	0.62096525	2.32E-20
DLD	PTPN1	0.62101894	2.3E-20
DLD	LSM14B	0.62115344	2.24E-20
DLD	CTBP2	0.62126116	2.2E-20
DLD	COPS8	0.62145572	2.12E-20
DLD	ATP6AP2	0.6214805	2.12E-20
DLD	FBXO5	0.62193586	1.95E-20
DLD	ISY1-RAB43	0.62219128	1.86E-20

DLD	GTF2E1	0.62224454	1.84E-20
DLD	RHEB	0.62241594	1.79E-20
DLD	TMTC2	0.62245744	1.77E-20
DLD	COX15	0.62259135	1.73E-20
DLD	LYN	0.62264764	1.71E-20
DLD	SNRNP200	0.62281975	1.66E-20
DLD	RTF2	0.62289923	1.64E-20
DLD	SFXN1	0.62305944	1.59E-20
DLD	HIF1A	0.62314541	1.57E-20
DLD	TMEM168	0.62315298	1.56E-20
DLD	EXT2	0.62317161	1.56E-20
DLD	VIPAS39	0.62344017	1.49E-20
DLD	DCTN6	0.62371396	1.41E-20
DLD	NT5C2	0.62383319	1.38E-20
DLD	KRAS	0.62423991	1.28E-20
DLD	C1GALT1C1	0.62438807	1.25E-20
DLD	SPTSSA	0.6244047	1.25E-20
DLD	GNPDA1	0.62460966	1.2E-20
DLD	DYRK2	0.624766	1.17E-20
DLD	C1GALT1	0.62479448	1.16E-20
DLD	MYO1D	0.62492395	1.13E-20
DLD	HMGCR	0.62492676	1.13E-20
DLD	UBE2V2	0.625057	1.11E-20
DLD	FBXO28	0.62515982	1.09E-20
DLD	BRI3BP	0.62517523	1.08E-20
DLD	CACUL1	0.6253034	1.06E-20
DLD	APAF1	0.62544447	1.03E-20
DLD	SAMD8	0.62558214	1.01E-20
DLD	PPP2R2A	0.62570288	9.84E-21
DLD	NCBP2	0.62587927	9.53E-21
DLD	NIPSNAP3A	0.62591191	9.48E-21
DLD	USP6NL	0.62613409	9.1E-21
DLD	PDLIM5	0.62623756	8.93E-21
DLD	DSTN	0.62636483	8.72E-21
DLD	FBXO45	0.62668643	8.22E-21
DLD	SNAP23	0.62676848	8.1E-21
DLD	WIPF2	0.62679152	8.07E-21
DLD	LPIN2	0.62681058	8.04E-21
DLD	NDUFA5	0.62682891	8.01E-21
DLD	UGP2	0.6269714	7.8E-21
DLD	GOLGA7	0.62713125	7.58E-21
DLD	KTN1	0.62744134	7.16E-21
DLD	GNS	0.62747905	7.11E-21
DLD	ACSL4	0.6276055	6.95E-21
DLD	RNF2	0.62767634	6.86E-21
DLD	NRBF2	0.62768181	6.85E-21
DLD	PAIP2	0.62769071	6.84E-21
DLD	EDC3	0.62774172	6.78E-21
DLD	SUMO2	0.62792977	6.55E-21
DLD	PHACTR4	0.62819524	6.23E-21
DLD	LAP3	0.62831061	6.1E-21
DLD	NDEL1	0.62832182	6.09E-21
DLD	DDX50	0.62841784	5.98E-21
DLD	NUDCD2	0.62842505	5.97E-21
DLD	TM9SF4	0.62842608	5.97E-21
DLD	PEX3	0.62846676	5.93E-21
DLD	ACO2	0.62868553	5.69E-21
DLD	PPHLN1	0.62875541	5.62E-21

DLD	SGCB	0.62882902	5.55E-21
DLD	TMEM267	0.62883694	5.54E-21
DLD	PPP1R15B	0.62884267	5.53E-21
DLD	MICU2	0.62886029	5.51E-21
DLD	WBP11	0.62888318	5.49E-21
DLD	CDC42EP3	0.62896169	5.41E-21
DLD	FAM120AOS	0.62909626	5.28E-21
DLD	ICMT	0.62909721	5.28E-21
DLD	SPATS2	0.62922521	5.15E-21
DLD	VAMP3	0.62923507	5.14E-21
DLD	SOCS5	0.62928113	5.1E-21
DLD	ZFYVE1	0.62947387	4.92E-21
DLD	LAPTM4A	0.62970003	4.72E-21
DLD	TMOD3	0.62980225	4.63E-21
DLD	ENOPH1	0.630099	4.38E-21
DLD	DRAM2	0.63014342	4.35E-21
DLD	ZFAND6	0.63031122	4.21E-21
DLD	ADAM17	0.63036762	4.17E-21
DLD	OCIAD1	0.63049037	4.08E-21
DLD	PTBP3	0.63054603	4.04E-21
DLD	MYO6	0.63056007	4.02E-21
DLD	SCAMP1	0.63057597	4.01E-21
DLD	SGPL1	0.63057759	4.01E-21
DLD	AKT1	0.63098839	3.72E-21
DLD	NAPEPLD	0.63118742	3.58E-21
DLD	RNF34	0.63125825	3.53E-21
DLD	TMCO1	0.63129494	3.51E-21
DLD	CPT1A	0.63135488	3.47E-21
DLD	UBE2H	0.63149715	3.38E-21
DLD	MOB4	0.6316657	3.28E-21
DLD	COPB1	0.63185385	3.16E-21
DLD	KDM5A	0.63210006	3.02E-21
DLD	FBXO34	0.63218347	2.97E-21
DLD	AKTIP	0.63226891	2.93E-21
DLD	GLO1	0.63247056	2.82E-21
DLD	RPTOR	0.63247927	2.81E-21
DLD	HIVEP1	0.63250996	2.8E-21
DLD	TRMT2B	0.63257161	2.77E-21
DLD	OXR1	0.63265662	2.72E-21
DLD	FRS2	0.63272913	2.69E-21
DLD	TOP2B	0.63280681	2.65E-21
DLD	SEC22A	0.63301891	2.54E-21
DLD	RNF145	0.63322151	2.45E-21
DLD	LDB1	0.63330931	2.41E-21
DLD	SC5D	0.63340692	2.37E-21
DLD	SMARCA2	0.6335808	2.29E-21
DLD	UBA6	0.6336415	2.26E-21
DLD	EHBP1	0.63379359	2.2E-21
DLD	PCBP1	0.63388461	2.16E-21
DLD	GPALPP1	0.63407088	2.09E-21
DLD	FAM220A	0.63420051	2.04E-21
DLD	NUP62	0.63424337	2.02E-21
DLD	CMPK1	0.63431635	1.99E-21
DLD	KCTD10	0.63438954	1.97E-21
DLD	C3orf38	0.63446128	1.94E-21
DLD	AAR2	0.63452171	1.92E-21
DLD	ZNF644	0.63453183	1.91E-21
DLD	TMBIM6	0.63456323	1.9E-21

DLD	CCNG2	0.6345754	1.9E-21
DLD	SLC24A1	0.63466857	1.87E-21
DLD	TEX10	0.63471298	1.85E-21
DLD	RAB28	0.63477808	1.83E-21
DLD	CASP3	0.63496065	1.77E-21
DLD	UHRF1BP1L	0.63506059	1.73E-21
DLD	GLB1	0.63531483	1.65E-21
DLD	SLC12A6	0.63549579	1.6E-21
DLD	GRPEL2	0.63555729	1.58E-21
DLD	GNB1	0.63569988	1.54E-21
DLD	CDKN2AIP	0.63571653	1.53E-21
DLD	FASTKD5	0.63580343	1.51E-21
DLD	ARL8B	0.63596508	1.46E-21
DLD	RAB8B	0.63635035	1.36E-21
DLD	AFG3L2	0.63636134	1.35E-21
DLD	LACC1	0.63649416	1.32E-21
DLD	OSBPL8	0.63675685	1.26E-21
DLD	AEBP2	0.63676711	1.25E-21
DLD	ERC1	0.63681577	1.24E-21
DLD	MRPL42	0.63684168	1.24E-21
DLD	TMEM19	0.63695054	1.21E-21
DLD	TRAFD1	0.63703482	1.19E-21
DLD	SERINC5	0.63704148	1.19E-21
DLD	PIK3R1	0.63711829	1.17E-21
DLD	COMMD2	0.63726109	1.14E-21
DLD	WSB2	0.63739228	1.11E-21
DLD	ITM2B	0.63739372	1.11E-21
DLD	FEM1C	0.63740483	1.11E-21
DLD	PIGX	0.63741757	1.11E-21
DLD	AGAP1	0.63747872	1.1E-21
DLD	CRLF3	0.63761843	1.07E-21
DLD	ARCN1	0.63787195	1.02E-21
DLD	HS2ST1	0.63797058	9.97E-22
DLD	URI1	0.63813527	9.66E-22
DLD	TAF13	0.63818275	9.57E-22
DLD	RBM22	0.63824287	9.46E-22
DLD	RSF1	0.63826796	9.42E-22
DLD	BTN2A1	0.63864293	8.77E-22
DLD	GLCE	0.63865714	8.74E-22
DLD	ARL15	0.63867417	8.72E-22
DLD	LEPROT	0.63875283	8.58E-22
DLD	XPR1	0.63879879	8.51E-22
DLD	MGRN1	0.63881787	8.48E-22
DLD	TM2D1	0.63893185	8.3E-22
DLD	MED4	0.6390209	8.16E-22
DLD	MAFG	0.63914015	7.97E-22
DLD	DCTN5	0.63917583	7.92E-22
DLD	FAM102B	0.63927251	7.77E-22
DLD	CTSO	0.63944109	7.52E-22
DLD	SMU1	0.63956986	7.34E-22
DLD	WDFY1	0.63958694	7.32E-22
DLD	MIS18BP1	0.63966139	7.21E-22
DLD	RSPH3	0.63971962	7.13E-22
DLD	CMIP	0.63985048	6.96E-22
DLD	CWF19L2	0.63992958	6.85E-22
DLD	DIPK1A	0.63997047	6.8E-22
DLD	NFE2L2	0.6399729	6.79E-22
DLD	CBFB	0.64015385	6.56E-22

DLD	LRRC41	0.64019564	6.51E-22
DLD	ECD	0.64031469	6.36E-22
DLD	KCTD9	0.64060637	6.01E-22
DLD	RNASEH1	0.64064776	5.97E-22
DLD	MED28	0.6407409	5.86E-22
DLD	COQ10B	0.64085197	5.74E-22
DLD	VEZT	0.64103242	5.54E-22
DLD	HNRNPF	0.64105445	5.52E-22
DLD	MFSD14B	0.64133242	5.23E-22
DLD	MEAF6	0.64150349	5.06E-22
DLD	HACD3	0.64162764	4.94E-22
DLD	AGFG1	0.64177301	4.8E-22
DLD	GPD2	0.64178167	4.79E-22
DLD	ELOVL5	0.6418377	4.74E-22
DLD	ZNFX1	0.64191045	4.67E-22
DLD	PIK3CA	0.64214553	4.47E-22
DLD	ATP13A3	0.64228649	4.35E-22
DLD	GSPT1	0.64241441	4.24E-22
DLD	TRAF3	0.6425028	4.17E-22
DLD	LAMP2	0.64270499	4.01E-22
DLD	PRUNE1	0.64272184	3.99E-22
DLD	MRPS27	0.64285622	3.89E-22
DLD	CDC42BPB	0.64297597	3.8E-22
DLD	CLINT1	0.64306031	3.74E-22
DLD	RECQL	0.64308542	3.72E-22
DLD	SOGA1	0.64321959	3.63E-22
DLD	DUS4L	0.64359025	3.37E-22
DLD	NUFIP2	0.64362185	3.35E-22
DLD	HARS2	0.64363431	3.34E-22
DLD	HACD2	0.64377878	3.25E-22
DLD	HTATSF1	0.64385605	3.2E-22
DLD	LRP10	0.64389442	3.18E-22
DLD	TDP2	0.64422096	2.98E-22
DLD	HAT1	0.64435445	2.91E-22
DLD	MIB1	0.64436962	2.9E-22
DLD	BLOC1S5	0.64439539	2.88E-22
DLD	TLK2	0.64448372	2.83E-22
DLD	NR2F2	0.64457505	2.78E-22
DLD	RTCB	0.64472217	2.7E-22
DLD	NAA50	0.64474689	2.69E-22
DLD	UTP11	0.64492546	2.6E-22
DLD	PARN	0.6449275	2.6E-22
DLD	C5orf15	0.64503757	2.54E-22
DLD	UBN1	0.64504073	2.54E-22
DLD	FAM168A	0.64521063	2.46E-22
DLD	TRIM32	0.64521719	2.45E-22
DLD	PPP2CB	0.64529951	2.41E-22
DLD	STK4	0.64542675	2.36E-22
DLD	TCF20	0.64548769	2.33E-22
DLD	GMEB1	0.64559813	2.28E-22
DLD	C9orf78	0.64582724	2.18E-22
DLD	LRRFIP2	0.6458737	2.16E-22
DLD	PPP1CB	0.6459841	2.11E-22
DLD	BRAP	0.64607101	2.08E-22
DLD	DYNC1LI1	0.64613471	2.05E-22
DLD	SLC39A9	0.64614849	2.04E-22
DLD	ARL2BP	0.64629883	1.98E-22
DLD	SNX18	0.64636619	1.96E-22

DLD	TBL1XR1	0.64647201	1.92E-22
DLD	IRF2BP2	0.64647779	1.92E-22
DLD	GALC	0.64651204	1.9E-22
DLD	CYFIP1	0.64652246	1.9E-22
DLD	CTTNBP2NL	0.64684825	1.78E-22
DLD	CREBL2	0.64700781	1.73E-22
DLD	VAPA	0.64705123	1.71E-22
DLD	SPRTN	0.6470861	1.7E-22
DLD	SDCBP	0.64709775	1.69E-22
DLD	B3GNT2	0.64716809	1.67E-22
DLD	RNF138	0.64728688	1.63E-22
DLD	WDR1	0.64748765	1.57E-22
DLD	NUP50	0.64758002	1.54E-22
DLD	LYPLA1	0.64781149	1.47E-22
DLD	DPY19L4	0.6478777	1.45E-22
DLD	KLHL7	0.64791573	1.44E-22
DLD	MSL2	0.64796127	1.43E-22
DLD	PPP3CB	0.6482964	1.34E-22
DLD	ELAVL1	0.64831143	1.33E-22
DLD	MRPL35	0.64842368	1.3E-22
DLD	DENR	0.64844129	1.3E-22
DLD	FIG4	0.64848664	1.29E-22
DLD	AC010132.3	0.64852957	1.28E-22
DLD	TMCC1	0.64860716	1.26E-22
DLD	FNBP1L	0.64893314	1.18E-22
DLD	RPE	0.64909602	1.14E-22
DLD	TMEM135	0.64919144	1.12E-22
DLD	PLEKHA1	0.64920075	1.12E-22
DLD	SNTB2	0.64925968	1.1E-22
DLD	VBP1	0.64926879	1.1E-22
DLD	EPB41L2	0.64931627	1.09E-22
DLD	NKRF	0.64931879	1.09E-22
DLD	WWP1	0.64938279	1.08E-22
DLD	SKAP2	0.64939443	1.07E-22
DLD	ATAD1	0.64948625	1.06E-22
DLD	PRELID3B	0.64985737	9.8E-23
DLD	EIF2AK2	0.64998897	9.55E-23
DLD	NUDT4B	0.65001605	9.5E-23
DLD	YTHDF2	0.65001731	9.49E-23
DLD	KDSR	0.65007501	9.39E-23
DLD	SNX16	0.65011885	9.3E-23
DLD	ABI2	0.65032535	8.93E-23
DLD	PITPNB	0.65045097	8.71E-23
DLD	FOXN2	0.65045441	8.7E-23
DLD	SETX	0.65062541	8.41E-23
DLD	ECHDC1	0.65080032	8.12E-23
DLD	MRPL49	0.65114323	7.58E-23
DLD	DOP1B	0.65127062	7.39E-23
DLD	HSPA13	0.65130562	7.34E-23
DLD	GOT2	0.65141689	7.17E-23
DLD	GCLM	0.65143517	7.15E-23
DLD	GRB2	0.6514761	7.09E-23
DLD	CAPRIN1	0.65214233	6.2E-23
DLD	MID1	0.65221473	6.11E-23
DLD	ZFAND5	0.65226496	6.05E-23
DLD	MFSD1	0.65229381	6.02E-23
DLD	LRRFIP1	0.65231284	5.99E-23
DLD	SFT2D2	0.65239797	5.89E-23

DLD	ATXN7L1	0.65245798	5.82E-23
DLD	CYREN	0.65275394	5.48E-23
DLD	ZNF227	0.65289858	5.33E-23
DLD	SZRD1	0.65301552	5.2E-23
DLD	FAM20B	0.65318971	5.02E-23
DLD	PARD3B	0.65354556	4.67E-23
DLD	KRR1	0.65356976	4.65E-23
DLD	SH3D19	0.65386993	4.38E-23
DLD	JAK2	0.65395087	4.3E-23
DLD	WASF2	0.65420541	4.09E-23
DLD	NCL	0.65422802	4.07E-23
DLD	KIAA1143	0.65424474	4.06E-23
DLD	LARP4B	0.65433622	3.98E-23
DLD	MAT2B	0.65442888	3.91E-23
DLD	SMARCE1	0.65444734	3.89E-23
DLD	ZMAT2	0.65445453	3.89E-23
DLD	ZNHIT6	0.6544561	3.89E-23
DLD	LCORL	0.65450956	3.84E-23
DLD	PAK2	0.65456816	3.8E-23
DLD	SRP9	0.65463711	3.75E-23
DLD	POT1	0.65474552	3.66E-23
DLD	PGM2	0.65476375	3.65E-23
DLD	CRIM1	0.65488998	3.56E-23
DLD	MOB1A	0.65495146	3.51E-23
DLD	KCTD20	0.65505838	3.44E-23
DLD	CCNK	0.65506961	3.43E-23
DLD	PRR14L	0.65510093	3.41E-23
DLD	LMBRD1	0.65529266	3.28E-23
DLD	CDC42	0.6554726	3.16E-23
DLD	NEK1	0.65547263	3.16E-23
DLD	GNA12	0.65551487	3.13E-23
DLD	UQCRC2	0.65581958	2.94E-23
DLD	LZIC	0.65583964	2.93E-23
DLD	MPHOSPH10	0.6559695	2.86E-23
DLD	WDR3	0.65605564	2.81E-23
DLD	POLR2D	0.65639148	2.62E-23
DLD	MSH2	0.65640826	2.61E-23
DLD	DDX23	0.6565704	2.53E-23
DLD	SLC35A3	0.65661821	2.5E-23
DLD	UBTD2	0.65671577	2.45E-23
DLD	UPRT	0.65672707	2.45E-23
DLD	SCO1	0.65693659	2.34E-23
DLD	CIAPIN1	0.65702369	2.3E-23
DLD	TUBA1B	0.6570255	2.3E-23
DLD	ZBTB38	0.65724248	2.2E-23
DLD	NAB1	0.65782796	1.95E-23
DLD	C2CD3	0.65787699	1.93E-23
DLD	SEC23A	0.65788461	1.93E-23
DLD	CDK17	0.65794315	1.91E-23
DLD	ANKMY2	0.65798076	1.89E-23
DLD	SNX9	0.65799688	1.88E-23
DLD	SERBP1	0.65802375	1.87E-23
DLD	MARK2	0.6582799	1.78E-23
DLD	VRK2	0.65837917	1.74E-23
DLD	SLC30A5	0.65844121	1.72E-23
DLD	UBXN2B	0.65854578	1.68E-23
DLD	RAB5A	0.6588648	1.58E-23
DLD	UHMK1	0.65886528	1.58E-23

DLD	ARHGAP35	0.65890956	1.56E-23
DLD	RC3H2	0.65893234	1.55E-23
DLD	COA7	0.65895291	1.55E-23
DLD	ATP2C1	0.659239	1.46E-23
DLD	NOLC1	0.65930199	1.44E-23
DLD	ACVR1	0.65986766	1.28E-23
DLD	NDFIP2	0.659896	1.27E-23
DLD	KPNA4	0.65992569	1.27E-23
DLD	CHM	0.66017683	1.2E-23
DLD	RAB22A	0.66033447	1.16E-23
DLD	FAM204A	0.66045342	1.13E-23
DLD	COMMD8	0.66046115	1.13E-23
DLD	TOB2	0.66060647	1.1E-23
DLD	CREB1	0.66080552	1.05E-23
DLD	COPS4	0.66089915	1.03E-23
DLD	KLHL2	0.66123933	9.63E-24
DLD	SMARCC1	0.66127462	9.56E-24
DLD	ZFP1	0.66142513	9.27E-24
DLD	YY1	0.66158186	8.97E-24
DLD	UBA3	0.66159627	8.94E-24
DLD	NRAS	0.66175318	8.65E-24
DLD	CERS6	0.66177544	8.61E-24
DLD	DUSP11	0.66197378	8.26E-24
DLD	VCPIP1	0.66203005	8.17E-24
DLD	TDP1	0.6621724	7.93E-24
DLD	MEGF9	0.66230063	7.72E-24
DLD	GFPT1	0.66231781	7.69E-24
DLD	TCEA1	0.66242153	7.53E-24
DLD	MLH1	0.66243587	7.5E-24
DLD	FGFR1OP2	0.66254257	7.34E-24
DLD	ATG3	0.66260343	7.24E-24
DLD	RSRC1	0.66284903	6.88E-24
DLD	SCFD2	0.66288886	6.82E-24
DLD	INCENP	0.66304895	6.6E-24
DLD	TRIM56	0.66305741	6.59E-24
DLD	PPP1R2	0.66309063	6.54E-24
DLD	PSMD12	0.66312676	6.49E-24
DLD	FYTTD1	0.66324935	6.33E-24
DLD	TMX1	0.66325257	6.32E-24
DLD	RTL6	0.66340904	6.12E-24
DLD	TMEM127	0.6634896	6.02E-24
DLD	SNX27	0.66355855	5.93E-24
DLD	PRPF18	0.66359764	5.88E-24
DLD	TNRC18	0.66383136	5.6E-24
DLD	CNIH1	0.66404966	5.35E-24
DLD	SLC25A44	0.66406389	5.33E-24
DLD	FRYL	0.66407736	5.32E-24
DLD	CDK19	0.66410219	5.29E-24
DLD	ATP9A	0.66421751	5.16E-24
DLD	TCF12	0.66421906	5.16E-24
DLD	APOL6	0.66423296	5.15E-24
DLD	ZNF263	0.66444495	4.92E-24
DLD	MTMR2	0.66457685	4.79E-24
DLD	SPTY2D1	0.66472424	4.64E-24
DLD	NUP98	0.66472658	4.64E-24
DLD	PTDSS1	0.66479194	4.57E-24
DLD	SNW1	0.66495824	4.42E-24
DLD	SSX2IP	0.6650249	4.35E-24

DLD	IFT57	0.66508124	4.3E-24
DLD	WNK1	0.6653474	4.07E-24
DLD	DCTD	0.665423	4E-24
DLD	PHACTR2	0.66575669	3.73E-24
DLD	METAP1	0.66577691	3.71E-24
DLD	SESTD1	0.66592555	3.6E-24
DLD	UBE2A	0.66598319	3.55E-24
DLD	ZC3H11A	0.66615028	3.43E-24
DLD	GAB2	0.66615951	3.42E-24
DLD	ARMC8	0.66625929	3.35E-24
DLD	AP2B1	0.66627734	3.34E-24
DLD	CSNK2A1	0.66629033	3.33E-24
DLD	ERBIN	0.66645136	3.22E-24
DLD	PDZD8	0.66645546	3.21E-24
DLD	HIPK3	0.66671704	3.04E-24
DLD	SDHC	0.66675867	3.01E-24
DLD	ABCF1	0.66676531	3.01E-24
DLD	ZNF639	0.66679416	2.99E-24
DLD	PICALM	0.6667951	2.99E-24
DLD	BMS1	0.66698337	2.87E-24
DLD	DNM1L	0.6669866	2.87E-24
DLD	KHDRBS1	0.66700027	2.86E-24
DLD	SSR1	0.66708453	2.81E-24
DLD	RAD1	0.66729611	2.69E-24
DLD	GNA13	0.66742862	2.61E-24
DLD	FCHO2	0.66755884	2.54E-24
DLD	PTPRA	0.66770733	2.46E-24
DLD	KBTBD2	0.66773342	2.45E-24
DLD	PEAK1	0.66790335	2.36E-24
DLD	UBFD1	0.66805292	2.29E-24
DLD	LAMTOR3	0.66811061	2.26E-24
DLD	RRM2B	0.66824532	2.19E-24
DLD	AGPS	0.6686222	2.02E-24
DLD	YES1	0.66869665	1.99E-24
DLD	FBXO7	0.66872143	1.98E-24
DLD	ATP10D	0.66875363	1.97E-24
DLD	PDCD6IP	0.66883453	1.93E-24
DLD	GLG1	0.66905506	1.85E-24
DLD	TADA1	0.66906886	1.84E-24
DLD	RNF141	0.66911324	1.82E-24
DLD	RAD21	0.66916455	1.8E-24
DLD	SBF2	0.66935473	1.73E-24
DLD	WBP4	0.66935947	1.73E-24
DLD	RO60	0.66939725	1.71E-24
DLD	TM9SF2	0.66945643	1.69E-24
DLD	ZNF468	0.66956997	1.65E-24
DLD	TDRD7	0.66957442	1.65E-24
DLD	PRKACA	0.66962154	1.63E-24
DLD	TAF1B	0.66962416	1.63E-24
DLD	NPAT	0.66963291	1.63E-24
DLD	EFL1	0.66972939	1.6E-24
DLD	GALK2	0.6697536	1.59E-24
DLD	CBL	0.66978447	1.58E-24
DLD	GNG12	0.66982398	1.56E-24
DLD	BECN1	0.66993086	1.53E-24
DLD	CCNY	0.67004721	1.49E-24
DLD	TIPRL	0.67034158	1.4E-24
DLD	TANK	0.67073438	1.29E-24

DLD	ITFG1	0.6708275	1.26E-24
DLD	RFWD3	0.67083868	1.26E-24
DLD	MORF4L2	0.6711073	1.19E-24
DLD	HDAC2	0.67115333	1.17E-24
DLD	CNOT6L	0.67128991	1.14E-24
DLD	ZNF143	0.67134856	1.13E-24
DLD	MFAP1	0.67134864	1.13E-24
DLD	EIF1AX	0.67153422	1.08E-24
DLD	PIP5K1A	0.67185841	1.01E-24
DLD	MAGT1	0.67191749	9.95E-25
DLD	TMEM33	0.67193801	9.9E-25
DLD	ZBTB33	0.67196097	9.85E-25
DLD	DIP2B	0.67203472	9.7E-25
DLD	ADAM10	0.67204783	9.67E-25
DLD	PPP4R2	0.67213388	9.49E-25
DLD	PRKAG1	0.67229126	9.17E-25
DLD	ANAPC10	0.672497	8.77E-25
DLD	ARHGAP5	0.67260403	8.57E-25
DLD	ASAH1	0.67323312	7.47E-25
DLD	DHX36	0.67326825	7.41E-25
DLD	STRN	0.67327369	7.41E-25
DLD	MORF4L1	0.67336831	7.25E-25
DLD	TBC1D14	0.67352785	7.01E-25
DLD	TERF2	0.67355538	6.96E-25
DLD	CXorf56	0.67364344	6.83E-25
DLD	BRD3	0.67388392	6.48E-25
DLD	APP	0.67400542	6.31E-25
DLD	LNPK	0.67410926	6.17E-25
DLD	PWP1	0.67429478	5.92E-25
DLD	GOLPH3	0.67438044	5.81E-25
DLD	MFSD6	0.67446647	5.71E-25
DLD	RAB5B	0.67458726	5.56E-25
DLD	UBLCP1	0.67461853	5.52E-25
DLD	MFSD11	0.67478029	5.33E-25
DLD	PNPO	0.674919	5.17E-25
DLD	RBBP5	0.67495584	5.12E-25
DLD	VHL	0.67538245	4.67E-25
DLD	PNRC2	0.67548298	4.56E-25
DLD	SH3BGRL	0.67594298	4.12E-25
DLD	DCUN1D1	0.67605156	4.03E-25
DLD	SUSD6	0.67607815	4E-25
DLD	HADHA	0.67618886	3.91E-25
DLD	PNO1	0.67619974	3.9E-25
DLD	DENND6A	0.67658107	3.58E-25
DLD	IL13RA1	0.67659237	3.57E-25
DLD	DOCK9	0.6767117	3.48E-25
DLD	TMEM87B	0.67672499	3.47E-25
DLD	MTDH	0.67676979	3.44E-25
DLD	ABHD17B	0.67678347	3.43E-25
DLD	STT3B	0.6767841	3.42E-25
DLD	UBE2D2	0.67680179	3.41E-25
DLD	RDH11	0.67688035	3.35E-25
DLD	CCNI	0.67704501	3.23E-25
DLD	RNF13	0.67747888	2.94E-25
DLD	NPM1	0.67753181	2.9E-25
DLD	IST1	0.67760106	2.86E-25
DLD	GRSF1	0.67779448	2.74E-25
DLD	STARD3NL	0.67790027	2.67E-25

DLD	SLC25A24	0.67790293	2.67E-25
DLD	CAB39	0.67814536	2.53E-25
DLD	KRCC1	0.67835573	2.42E-25
DLD	SLC4A1AP	0.67856309	2.31E-25
DLD	ERLIN1	0.67857887	2.3E-25
DLD	GPN3	0.67860981	2.28E-25
DLD	KAT6A	0.67864798	2.26E-25
DLD	WDR20	0.67880701	2.19E-25
DLD	PRRC2C	0.67886117	2.16E-25
DLD	CPSF6	0.678939	2.12E-25
DLD	IGF2R	0.67932312	1.95E-25
DLD	TPP2	0.6796613	1.81E-25
DLD	SET	0.67980554	1.75E-25
DLD	TEFM	0.67994378	1.69E-25
DLD	LRRCC1	0.68008536	1.64E-25
DLD	ZNF200	0.68026847	1.58E-25
DLD	ABCF2	0.68043046	1.52E-25
DLD	LRCH1	0.68050979	1.49E-25
DLD	PRPF40A	0.6806661	1.44E-25
DLD	BMPR2	0.68067231	1.44E-25
DLD	ZNF207	0.68074557	1.42E-25
DLD	RBM7	0.68082612	1.39E-25
DLD	UBE2W	0.68109926	1.31E-25
DLD	ZFP64	0.68114126	1.3E-25
DLD	CHMP3	0.68121306	1.27E-25
DLD	ARL13B	0.68128331	1.25E-25
DLD	CCSER2	0.68146797	1.2E-25
DLD	RFX5	0.6815708	1.18E-25
DLD	EIF2S1	0.68158494	1.17E-25
DLD	TRIQK	0.68174015	1.13E-25
DLD	MAP3K7	0.68187179	1.1E-25
DLD	WDR89	0.6819995	1.07E-25
DLD	PRKDC	0.68200482	1.07E-25
DLD	MED1	0.68205109	1.06E-25
DLD	IARS2	0.68220527	1.02E-25
DLD	RPRD2	0.68243454	9.67E-26
DLD	RBBP9	0.6824771	9.58E-26
DLD	EPC2	0.68253543	9.46E-26
DLD	CUL4B	0.6825832	9.35E-26
DLD	PSMD10	0.6829679	8.57E-26
DLD	HCCS	0.68302727	8.46E-26
DLD	RAB35	0.68304811	8.42E-26
DLD	KPNB1	0.68318433	8.16E-26
DLD	ATP5MF-PTCD1	0.68334246	7.88E-26
DLD	ZBTB41	0.68337474	7.82E-26
DLD	TOPORS	0.68348548	7.62E-26
DLD	PIGS	0.6835235	7.56E-26
DLD	NEDD1	0.68371651	7.23E-26
DLD	SHOC2	0.68378186	7.13E-26
DLD	CAMSAP1	0.68385837	7E-26
DLD	CANX	0.68389655	6.94E-26
DLD	DHX35	0.68395031	6.86E-26
DLD	BPTF	0.68401091	6.76E-26
DLD	WDR47	0.68409491	6.64E-26
DLD	TCTN3	0.68416999	6.52E-26
DLD	CYCS	0.68418701	6.5E-26
DLD	NSL1	0.68429915	6.34E-26
DLD	CROT	0.68443348	6.14E-26

DLD	TRMT1L	0.68446614	6.1E-26
DLD	GPR107	0.68463305	5.87E-26
DLD	SNX1	0.6847306	5.74E-26
DLD	NUMB	0.68475887	5.7E-26
DLD	DAAM1	0.6852447	5.1E-26
DLD	ATL2	0.68544091	4.88E-26
DLD	WDR26	0.68550433	4.81E-26
DLD	INTS14	0.68553694	4.77E-26
DLD	SELENOF	0.68588087	4.41E-26
DLD	FAM118B	0.68601997	4.27E-26
DLD	ACBD3	0.68607027	4.22E-26
DLD	PCYOX1	0.68610115	4.2E-26
DLD	TRAPPC6B	0.68633486	3.98E-26
DLD	ZNF184	0.68634341	3.97E-26
DLD	COPS2	0.68640147	3.92E-26
DLD	TMEM106B	0.68681533	3.56E-26
DLD	SLMAP	0.68718796	3.27E-26
DLD	GNPTAB	0.68723943	3.23E-26
DLD	IMMT	0.68726601	3.21E-26
DLD	OSBPL11	0.68756908	2.99E-26
DLD	ATP6V1C1	0.68757333	2.99E-26
DLD	NCOA3	0.68760085	2.97E-26
DLD	PRRC1	0.68772887	2.88E-26
DLD	EXOC4	0.6878296	2.82E-26
DLD	EXOC6B	0.68788161	2.78E-26
DLD	RYBP	0.6879728	2.73E-26
DLD	RNF20	0.68800557	2.7E-26
DLD	MAPRE2	0.68804721	2.68E-26
DLD	ISG20L2	0.68809505	2.65E-26
DLD	WASHC2A	0.68818862	2.59E-26
DLD	TFDP1	0.68843188	2.45E-26
DLD	TBC1D5	0.68852862	2.4E-26
DLD	SLC17A5	0.68900348	2.15E-26
DLD	PAQR3	0.68902415	2.14E-26
DLD	RPAP3	0.68911387	2.09E-26
DLD	NF1	0.68915251	2.07E-26
DLD	SLC25A32	0.68958956	1.87E-26
DLD	PPP2R5D	0.68974488	1.81E-26
DLD	GDE1	0.68999476	1.7E-26
DLD	SMIM30	0.69000625	1.7E-26
DLD	LNPEP	0.69000631	1.7E-26
DLD	ARL14EP	0.69006341	1.68E-26
DLD	ATF2	0.69027214	1.6E-26
DLD	TPP1	0.69044633	1.53E-26
DLD	LEO1	0.69044939	1.53E-26
DLD	ATP5PB	0.69048	1.52E-26
DLD	SLC35A4	0.69058086	1.49E-26
DLD	CALM1	0.69095024	1.36E-26
DLD	TRRAP	0.69122881	1.28E-26
DLD	STAM	0.691323	1.25E-26
DLD	INTS12	0.6916019	1.17E-26
DLD	IWS1	0.69173803	1.13E-26
DLD	RAB1A	0.69174842	1.13E-26
DLD	AHCTF1	0.69181968	1.11E-26
DLD	MAP2K1	0.69197087	1.07E-26
DLD	JKAMP	0.69223644	1.01E-26
DLD	STARD7	0.69235037	9.81E-27
DLD	DCAF12	0.69243865	9.61E-27

DLD	EID1	0.69246461	9.55E-27
DLD	COQ7	0.69246891	9.54E-27
DLD	ZBTB6	0.69253594	9.39E-27
DLD	CTDSPL2	0.69262386	9.2E-27
DLD	FAM91A1	0.69267587	9.09E-27
DLD	HNRNPU	0.69287775	8.66E-27
DLD	DCK	0.69292024	8.58E-27
DLD	ZC3H15	0.6929657	8.49E-27
DLD	RAB5C	0.6930607	8.3E-27
DLD	RAB2A	0.69325078	7.93E-27
DLD	C16orf70	0.69332104	7.8E-27
DLD	TSNAX	0.69337058	7.71E-27
DLD	NFE2L1	0.69347519	7.52E-27
DLD	METTL9	0.69387407	6.85E-27
DLD	NUB1	0.69398419	6.67E-27
DLD	MBTPS2	0.69400739	6.63E-27
DLD	SDE2	0.69401539	6.62E-27
DLD	LIN52	0.69438206	6.07E-27
DLD	SPPL3	0.69453014	5.86E-27
DLD	BAG5	0.69459671	5.77E-27
DLD	ZMIZ1	0.69517113	5.03E-27
DLD	CNOT9	0.69540409	4.76E-27
DLD	VPS33A	0.69548584	4.67E-27
DLD	SF3A3	0.69549323	4.66E-27
DLD	RAP1A	0.69554672	4.6E-27
DLD	FYCO1	0.69569902	4.44E-27
DLD	SERINC1	0.69585584	4.27E-27
DLD	BMT2	0.69596176	4.17E-27
DLD	MEF2A	0.69617145	3.96E-27
DLD	SOCS4	0.69617381	3.96E-27
DLD	SP3	0.6962855	3.86E-27
DLD	DNAJB6	0.69662163	3.56E-27
DLD	DDX21	0.69664443	3.54E-27
DLD	SRSF3	0.69665755	3.53E-27
DLD	FASTKD2	0.69688325	3.34E-27
DLD	BZW1	0.6969213	3.31E-27
DLD	ACTR6	0.69702624	3.23E-27
DLD	PRPF4	0.69710541	3.17E-27
DLD	GPATCH11	0.69724981	3.06E-27
DLD	ROCK1	0.69740862	2.95E-27
DLD	INO80	0.69748469	2.89E-27
DLD	COPG2	0.69748981	2.89E-27
DLD	TARDBP	0.69765446	2.78E-27
DLD	ABL1	0.69776624	2.7E-27
DLD	PFKM	0.69790283	2.62E-27
DLD	FAM120A	0.69792089	2.6E-27
DLD	SMIM15	0.69804707	2.53E-27
DLD	VAPB	0.69820713	2.43E-27
DLD	SMC2	0.69821422	2.43E-27
DLD	PPP3R1	0.69834294	2.35E-27
DLD	HPS5	0.69834614	2.35E-27
DLD	DYNC1I2	0.69850297	2.26E-27
DLD	ZNF827	0.69871082	2.15E-27
DLD	MED13	0.69874388	2.13E-27
DLD	SMG8	0.69887268	2.07E-27
DLD	KIAA0232	0.6988752	2.07E-27
DLD	SCARB2	0.69892963	2.04E-27
DLD	HSPA14	0.69897565	2.02E-27

DLD	ESYT2	0.69899792	2.01E-27
DLD	CREB3L2	0.69908801	1.96E-27
DLD	FKBP15	0.69926035	1.88E-27
DLD	DNAJC21	0.69944024	1.8E-27
DLD	BRCC3	0.69959343	1.74E-27
DLD	ZNF720	0.69975575	1.67E-27
DLD	SERINC3	0.69978962	1.66E-27
DLD	KIF13A	0.69987654	1.62E-27
DLD	DDX18	0.69987939	1.62E-27
DLD	ZBTB2	0.69995748	1.59E-27
DLD	GFM1	0.70006417	1.55E-27
DLD	BTBD9	0.70024802	1.48E-27
DLD	CRTC3	0.70034483	1.45E-27
DLD	ADH5	0.70050108	1.39E-27
DLD	KLHL20	0.7005262	1.39E-27
DLD	ZNF146	0.70066784	1.34E-27
DLD	AP1AR	0.70067733	1.34E-27
DLD	ACTR3	0.70074988	1.31E-27
DLD	YWHAQ	0.70083513	1.28E-27
DLD	TSPYL1	0.70093197	1.25E-27
DLD	EAF1	0.70138462	1.12E-27
DLD	OAT	0.70155486	1.08E-27
DLD	PCYT1A	0.70184426	1E-27
DLD	DNAJB14	0.7019929	9.68E-28
DLD	SPOP	0.70204809	9.55E-28
DLD	MPP5	0.70249045	8.57E-28
DLD	CNOT8	0.70249155	8.57E-28
DLD	CUL4A	0.70254331	8.46E-28
DLD	GCC2	0.70278038	7.98E-28
DLD	SCP2	0.70283829	7.87E-28
DLD	FNIP2	0.703081	7.41E-28
DLD	CLCN3	0.70316713	7.26E-28
DLD	SNX2	0.70321579	7.17E-28
DLD	CLOCK	0.70357493	6.56E-28
DLD	SCAF11	0.70361577	6.5E-28
DLD	PEX2	0.70367023	6.41E-28
DLD	TRAK2	0.7036898	6.38E-28
DLD	TVP23B	0.7037318	6.31E-28
DLD	ABHD13	0.70409816	5.77E-28
DLD	RAB6A	0.704241	5.57E-28
DLD	PANK3	0.70424187	5.57E-28
DLD	LRRC57	0.70426524	5.53E-28
DLD	LRRC8D	0.70433047	5.45E-28
DLD	STAU1	0.70439865	5.35E-28
DLD	SRFBP1	0.70458593	5.11E-28
DLD	ERAP1	0.70464692	5.04E-28
DLD	RBM18	0.70468526	4.99E-28
DLD	RAB3GAP2	0.70490676	4.72E-28
DLD	DLG1	0.70502102	4.59E-28
DLD	MCMBP	0.70510181	4.5E-28
DLD	MFAP3	0.70513888	4.46E-28
DLD	QTRT2	0.70522978	4.36E-28
DLD	ZNRF2	0.70529008	4.29E-28
DLD	SNRNP27	0.7052968	4.29E-28
DLD	SYNJ2BP	0.70530934	4.27E-28
DLD	C5orf24	0.70534079	4.24E-28
DLD	ZNF148	0.70540725	4.17E-28
DLD	DAZAP2	0.70544678	4.13E-28

DLD	ELF1	0.70549714	4.08E-28
DLD	ORC4	0.70551723	4.06E-28
DLD	FTO	0.70574332	3.84E-28
DLD	ABI1	0.70574833	3.83E-28
DLD	USP10	0.70613365	3.48E-28
DLD	STRN3	0.70640841	3.25E-28
DLD	TGS1	0.70643048	3.23E-28
DLD	HERC3	0.70645194	3.21E-28
DLD	ATP2B1	0.70653472	3.15E-28
DLD	RELL1	0.70668314	3.03E-28
DLD	ATP6V1A	0.70674437	2.99E-28
DLD	COPA	0.70677043	2.97E-28
DLD	MTFMT	0.70689631	2.88E-28
DLD	DLST	0.70690069	2.87E-28
DLD	MED20	0.70692227	2.86E-28
DLD	EFR3A	0.70707329	2.75E-28
DLD	SLC9A6	0.707215	2.66E-28
DLD	UBA2	0.70731914	2.59E-28
DLD	RBFOX2	0.70734275	2.57E-28
DLD	TMEM209	0.70748467	2.48E-28
DLD	POLR2C	0.70752702	2.46E-28
DLD	TFCP2	0.70774895	2.32E-28
DLD	USP14	0.7078341	2.27E-28
DLD	GANAB	0.70795574	2.21E-28
DLD	YIPF5	0.7080908	2.13E-28
DLD	TAOK1	0.70823748	2.06E-28
DLD	VPS37A	0.70843986	1.95E-28
DLD	TMED7	0.70845312	1.95E-28
DLD	ASB7	0.70846014	1.94E-28
DLD	LIN54	0.7084956	1.93E-28
DLD	BMI1	0.70858029	1.89E-28
DLD	ALG11	0.7085946	1.88E-28
DLD	SMC6	0.70864102	1.86E-28
DLD	RLIM	0.70928509	1.58E-28
DLD	CNOT7	0.70938873	1.54E-28
DLD	BBX	0.70962177	1.45E-28
DLD	STAT3	0.70963216	1.45E-28
DLD	HAUS2	0.70963847	1.44E-28
DLD	CNST	0.71006022	1.3E-28
DLD	CTBS	0.71007367	1.29E-28
DLD	DHDDS	0.71030422	1.22E-28
DLD	HIBADH	0.71032125	1.21E-28
DLD	SYPL1	0.7103688	1.2E-28
DLD	RANBP9	0.71045475	1.17E-28
DLD	ACLY	0.7105817	1.14E-28
DLD	RPRD1B	0.7106364	1.12E-28
DLD	HBP1	0.71160413	8.76E-29
DLD	CPNE3	0.71161108	8.74E-29
DLD	C1D	0.71175344	8.43E-29
DLD	YWHAG	0.71193734	8.05E-29
DLD	TERF1	0.71208774	7.74E-29
DLD	SPIN1	0.71210842	7.7E-29
DLD	HMGXB4	0.71215454	7.61E-29
DLD	UEVLD	0.71218791	7.55E-29
DLD	PRDX3	0.71228361	7.36E-29
DLD	RPA1	0.71245104	7.06E-29
DLD	KIAA1191	0.71245964	7.04E-29
DLD	SP2	0.71247044	7.02E-29

DLD	METTL2B	0.71257884	6.83E-29
DLD	MBD5	0.71270033	6.62E-29
DLD	FOXN3	0.71270548	6.61E-29
DLD	SRSF1	0.71271357	6.6E-29
DLD	PTPN9	0.71287341	6.33E-29
DLD	PPIL4	0.71294962	6.21E-29
DLD	GMCL1	0.71315754	5.89E-29
DLD	ATE1	0.71326107	5.73E-29
DLD	VKORC1L1	0.71337083	5.57E-29
DLD	PI4K2B	0.71338947	5.55E-29
DLD	NFATC3	0.71341134	5.51E-29
DLD	YIPF6	0.71353065	5.35E-29
DLD	TEAD1	0.71364145	5.2E-29
DLD	SKP1	0.71373091	5.08E-29
DLD	TOX4	0.7138042	4.98E-29
DLD	GTF3C4	0.71381838	4.97E-29
DLD	RDH14	0.71385258	4.92E-29
DLD	LUZP1	0.71401295	4.72E-29
DLD	PCNP	0.71407151	4.65E-29
DLD	ZNF410	0.71411974	4.6E-29
DLD	GOLIM4	0.71431928	4.36E-29
DLD	DCAF1	0.7144216	4.25E-29
DLD	TSN	0.71447264	4.2E-29
DLD	AP003108.2	0.71457499	4.09E-29
DLD	RABL3	0.71460314	4.06E-29
DLD	EVI5	0.71468068	3.98E-29
DLD	MAPK6	0.71479194	3.86E-29
DLD	ZNF562	0.71490671	3.75E-29
DLD	SPOPL	0.71498649	3.67E-29
DLD	PRKAR1A	0.71504629	3.62E-29
DLD	ANKS1A	0.71509812	3.57E-29
DLD	GNPDA2	0.71524492	3.44E-29
DLD	PPFIA1	0.71546866	3.24E-29
DLD	CHD9	0.71568381	3.07E-29
DLD	TMED5	0.71593197	2.87E-29
DLD	TWF1	0.7160167	2.81E-29
DLD	SNX6	0.71612967	2.73E-29
DLD	TAF5L	0.71615971	2.71E-29
DLD	STX12	0.71647418	2.5E-29
DLD	ALDH9A1	0.71650698	2.48E-29
DLD	RAB14	0.71673107	2.33E-29
DLD	HNRNPA2B1	0.71700876	2.17E-29
DLD	IRF2	0.71710878	2.12E-29
DLD	NEO1	0.71721338	2.06E-29
DLD	UVRAG	0.71741617	1.95E-29
DLD	HUS1	0.71742379	1.95E-29
DLD	TBK1	0.71744851	1.94E-29
DLD	CRK	0.7174572	1.93E-29
DLD	KPNA1	0.71745866	1.93E-29
DLD	SART3	0.71770639	1.81E-29
DLD	PEX26	0.71777738	1.78E-29
DLD	ATMIN	0.71778636	1.77E-29
DLD	CNOT4	0.71788089	1.73E-29
DLD	STAG2	0.71793748	1.7E-29
DLD	PDE12	0.71795871	1.69E-29
DLD	MKRN1	0.71810975	1.63E-29
DLD	ST13	0.71833479	1.53E-29
DLD	GLOD4	0.71863749	1.42E-29

DLD	CDC42SE2	0.71871548	1.39E-29
DLD	FBXL17	0.7187957	1.36E-29
DLD	PEX19	0.7188004	1.36E-29
DLD	SPTLC1	0.7189672	1.3E-29
DLD	AIMP1	0.71897617	1.3E-29
DLD	DOCK7	0.71904766	1.27E-29
DLD	AKAP11	0.71908291	1.26E-29
DLD	SYNCRIP	0.71909072	1.26E-29
DLD	LRBA	0.71909829	1.26E-29
DLD	ZMPSTE24	0.71939028	1.16E-29
DLD	TTC33	0.71967007	1.08E-29
DLD	CEP41	0.71978564	1.05E-29
DLD	FAF2	0.71987146	1.02E-29
DLD	ALS2	0.71993062	1.01E-29
DLD	NCOA2	0.72001463	9.86E-30
DLD	NIPA2	0.72031583	9.1E-30
DLD	NUP58	0.72046433	8.75E-30
DLD	UBE2N	0.72050477	8.66E-30
DLD	GNAQ	0.72087059	7.86E-30
DLD	CSTF2T	0.72104902	7.49E-30
DLD	RAB8A	0.72112363	7.35E-30
DLD	CSDE1	0.72119788	7.2E-30
DLD	GOSR1	0.72126584	7.07E-30
DLD	NUDCD3	0.72133856	6.94E-30
DLD	ABRAXAS2	0.72138271	6.86E-30
DLD	ADAR	0.72165972	6.37E-30
DLD	RP2	0.72170977	6.28E-30
DLD	ZNF45	0.72198683	5.84E-30
DLD	SAP130	0.72216471	5.57E-30
DLD	MIER1	0.7222667	5.42E-30
DLD	IMPA1	0.72231429	5.35E-30
DLD	RAB10	0.72238798	5.24E-30
DLD	GTF2A1	0.72248323	5.11E-30
DLD	CDC27	0.72257144	4.99E-30
DLD	SETD3	0.72262595	4.92E-30
DLD	CCDC47	0.72274973	4.76E-30
DLD	G3BP1	0.72294123	4.52E-30
DLD	CDK12	0.72318275	4.24E-30
DLD	VPS35	0.72327957	4.13E-30
DLD	C16orf72	0.72329965	4.11E-30
DLD	GEMIN5	0.7233856	4.01E-30
DLD	PITPNA	0.723618	3.77E-30
DLD	PPP1CC	0.7238736	3.52E-30
DLD	DNAJA2	0.72392929	3.47E-30
DLD	TMEM170A	0.72410713	3.31E-30
DLD	SLC25A40	0.72411778	3.3E-30
DLD	RFFL	0.72448468	2.99E-30
DLD	KIF16B	0.72451155	2.97E-30
DLD	PIGK	0.72480889	2.74E-30
DLD	GORASP2	0.72485206	2.7E-30
DLD	ARHGEF12	0.72488733	2.68E-30
DLD	GSTCD	0.72489918	2.67E-30
DLD	ZNF398	0.72562925	2.19E-30
DLD	STAU2	0.72580802	2.09E-30
DLD	SAR1B	0.72588322	2.05E-30
DLD	ACTR2	0.72596401	2E-30
DLD	CSNK1G3	0.7262185	1.87E-30
DLD	GNL3L	0.7262826	1.84E-30

DLD	JAK1	0.72647512	1.74E-30
DLD	CALCOCO2	0.7266225	1.67E-30
DLD	ATF1	0.72707824	1.48E-30
DLD	ROCK2	0.72719436	1.43E-30
DLD	R3HDM1	0.72721454	1.42E-30
DLD	IFNAR1	0.72732351	1.38E-30
DLD	VEZF1	0.72760997	1.28E-30
DLD	NFKB1	0.72762276	1.27E-30
DLD	TMEM248	0.72770574	1.25E-30
DLD	USP1	0.727752	1.23E-30
DLD	ZMYM4	0.72792556	1.17E-30
DLD	TFAM	0.72795503	1.16E-30
DLD	LARS2	0.72811505	1.11E-30
DLD	RAP2A	0.72832395	1.05E-30
DLD	MIGA1	0.72833754	1.05E-30
DLD	SELENOT	0.72836409	1.04E-30
DLD	NBR1	0.72857095	9.82E-31
DLD	HIPK1	0.72860442	9.73E-31
DLD	NSRP1	0.72863144	9.66E-31
DLD	MAPK1IP1L	0.72882183	9.17E-31
DLD	MMGT1	0.72898049	8.78E-31
DLD	SLC35A5	0.72898489	8.77E-31
DLD	HNRNPLL	0.72911309	8.46E-31
DLD	NBN	0.72935874	7.91E-31
DLD	ARF3	0.72937825	7.87E-31
DLD	TRAF3IP1	0.72953407	7.53E-31
DLD	SMAD1	0.72955549	7.49E-31
DLD	UBE2D3	0.72964657	7.3E-31
DLD	UBQLN2	0.72967042	7.26E-31
DLD	SETD7	0.7297633	7.07E-31
DLD	SOS2	0.72985076	6.9E-31
DLD	OPA1	0.72989883	6.81E-31
DLD	YWHAB	0.7299889	6.65E-31
DLD	SPAST	0.73042104	5.9E-31
DLD	MRPL19	0.73044555	5.86E-31
DLD	CWC22	0.73060441	5.6E-31
DLD	HNRNPR	0.73069021	5.47E-31
DLD	MAPK14	0.73086647	5.21E-31
DLD	ARL1	0.73107452	4.92E-31
DLD	KDM4A	0.73145227	4.43E-31
DLD	FBXL3	0.73166196	4.18E-31
DLD	NAA30	0.7316953	4.14E-31
DLD	EIF4G3	0.73194441	3.86E-31
DLD	PDCL	0.73212453	3.67E-31
DLD	TAX1BP1	0.73233251	3.47E-31
DLD	C9orf64	0.7325438	3.27E-31
DLD	CFAP97	0.7325725	3.24E-31
DLD	VPS4B	0.73264265	3.18E-31
DLD	DR1	0.73280871	3.03E-31
DLD	SMAD2	0.73290207	2.96E-31
DLD	CIPC	0.73299271	2.88E-31
DLD	ACBD5	0.73305364	2.83E-31
DLD	ZYG11B	0.73333267	2.62E-31
DLD	ABCB10	0.73343695	2.54E-31
DLD	SMNDC1	0.73347621	2.52E-31
DLD	DNAJC10	0.73352227	2.48E-31
DLD	DEK	0.73403449	2.15E-31
DLD	SLC31A1	0.7343352	1.98E-31

DLD	USP12	0.73437117	1.96E-31
DLD	FAM98A	0.73440265	1.94E-31
DLD	PPM1B	0.73444061	1.92E-31
DLD	SDHD	0.7345017	1.89E-31
DLD	UBQLN1	0.735024	1.63E-31
DLD	PURB	0.73564769	1.36E-31
DLD	EPS15	0.73581899	1.3E-31
DLD	RAD23B	0.73586323	1.28E-31
DLD	PDHX	0.73597232	1.24E-31
DLD	TAOK3	0.73603004	1.22E-31
DLD	POFUT1	0.73638634	1.11E-31
DLD	ZNF322	0.73677453	9.9E-32
DLD	PRKAG2	0.73693781	9.45E-32
DLD	CDYL	0.73742752	8.21E-32
DLD	IPO7	0.73758292	7.86E-32
DLD	ARPP19	0.73791332	7.15E-32
DLD	STX7	0.7379739	7.02E-32
DLD	GTF2H3	0.73798989	6.99E-32
DLD	KIF3B	0.73812402	6.73E-32
DLD	MAPRE1	0.73837944	6.25E-32
DLD	KBTBD4	0.73839203	6.23E-32
DLD	FAM98B	0.73875058	5.62E-32
DLD	PPIG	0.73877098	5.59E-32
DLD	RHBDD1	0.73891413	5.36E-32
DLD	PPP2R5E	0.73896259	5.29E-32
DLD	DNAJC14	0.73925744	4.86E-32
DLD	ANKRD13C	0.73933672	4.75E-32
DLD	ZNF609	0.73939039	4.68E-32
DLD	PPP1R8	0.73955577	4.46E-32
DLD	DDX19A	0.73995295	3.97E-32
DLD	RNF111	0.74010658	3.8E-32
DLD	PJA2	0.74019446	3.71E-32
DLD	BLOC1S6	0.74039732	3.49E-32
DLD	TYW1	0.74043993	3.45E-32
DLD	LYRM2	0.74054875	3.34E-32
DLD	CSNK1A1	0.74065579	3.24E-32
DLD	CLIP1	0.74077244	3.13E-32
DLD	MED17	0.74086399	3.05E-32
DLD	FBXL5	0.7409002	3.02E-32
DLD	SUZ12	0.74113203	2.82E-32
DLD	SENP1	0.7412742	2.71E-32
DLD	KAT7	0.74150276	2.53E-32
DLD	SF3A1	0.74152905	2.52E-32
DLD	RAB3GAP1	0.74180187	2.32E-32
DLD	OGFOD1	0.74186883	2.28E-32
DLD	CAMK2D	0.74197278	2.21E-32
DLD	SIN3A	0.74212651	2.11E-32
DLD	AFF4	0.74224462	2.04E-32
DLD	CNOT6	0.74235602	1.98E-32
DLD	MED14	0.74254606	1.87E-32
DLD	LDAH	0.74295499	1.66E-32
DLD	ICE2	0.74295806	1.66E-32
DLD	NT5DC1	0.74299239	1.64E-32
DLD	DDX6	0.74300803	1.63E-32
DLD	EXOC2	0.74305897	1.61E-32
DLD	ARIH1	0.74306204	1.61E-32
DLD	KDM3B	0.74321587	1.54E-32
DLD	ESF1	0.74323537	1.53E-32

DLD	TAB2	0.74368174	1.34E-32
DLD	LSM14A	0.74370548	1.33E-32
DLD	IDH3A	0.74409079	1.19E-32
DLD	PUM1	0.74464453	1.01E-32
DLD	C5orf51	0.74561128	7.57E-33
DLD	KDM1B	0.7459571	6.83E-33
DLD	FBXO8	0.74619564	6.37E-33
DLD	WDR82	0.74641868	5.96E-33
DLD	ACAP2	0.74643182	5.93E-33
DLD	SLC25A13	0.74645554	5.89E-33
DLD	TRUB1	0.7465514	5.73E-33
DLD	CTCF	0.74669301	5.49E-33
DLD	PHAX	0.74693366	5.11E-33
DLD	TOR1AIP1	0.74701381	4.99E-33
DLD	AHCYL1	0.74703573	4.96E-33
DLD	PPM1A	0.74704265	4.95E-33
DLD	PSME3	0.74709343	4.87E-33
DLD	NCKAP1	0.74734842	4.51E-33
DLD	RAB21	0.74753109	4.27E-33
DLD	ITCH	0.74770584	4.05E-33
DLD	DIS3	0.74778901	3.96E-33
DLD	AKIRIN1	0.74795432	3.76E-33
DLD	TRIP12	0.74869614	3.01E-33
DLD	RBM12	0.74876507	2.95E-33
DLD	SLAIN2	0.74884785	2.88E-33
DLD	MAP3K2	0.74901621	2.73E-33
DLD	ASXL2	0.74919962	2.59E-33
DLD	ZFR	0.74966461	2.25E-33
DLD	HINT3	0.74971328	2.22E-33
DLD	CHUK	0.74980769	2.15E-33
DLD	KIF2A	0.75054669	1.72E-33
DLD	PDS5B	0.75062583	1.68E-33
DLD	ANXA7	0.75062873	1.68E-33
DLD	GMFB	0.75100051	1.5E-33
DLD	NPTN	0.75112832	1.44E-33
DLD	PUM2	0.75112894	1.44E-33
DLD	DLAT	0.75127364	1.38E-33
DLD	MAPK1	0.75139058	1.33E-33
DLD	WASL	0.75226609	1.02E-33
DLD	SBDS	0.75234213	9.95E-34
DLD	RPL7L1	0.75236418	9.88E-34
DLD	WDR44	0.75244429	9.64E-34
DLD	TOP1	0.75283467	8.55E-34
DLD	VPS41	0.75294932	8.26E-34
DLD	BCAP29	0.75299366	8.15E-34
DLD	USP9X	0.753131	7.81E-34
DLD	SLC30A6	0.7531592	7.74E-34
DLD	VAMP7	0.75327527	7.47E-34
DLD	DDX3X	0.75343217	7.12E-34
DLD	ARMC1	0.75351868	6.93E-34
DLD	DYRK1A	0.75352449	6.92E-34
DLD	TAF2	0.7543856	5.3E-34
DLD	RNF41	0.75458805	4.98E-34
DLD	UTP14C	0.75493	4.48E-34
DLD	AGGF1	0.75526373	4.04E-34
DLD	RPS6KA3	0.75546257	3.8E-34
DLD	ARMT1	0.7559626	3.25E-34
DLD	SEC23IP	0.75611533	3.1E-34

DLD	NAA15	0.75615915	3.06E-34
DLD	PMS2	0.7565939	2.67E-34
DLD	TLK1	0.75709287	2.28E-34
DLD	C11orf58	0.7573604	2.1E-34
DLD	CAND1	0.7574371	2.05E-34
DLD	RNF6	0.75761162	1.94E-34
DLD	INIP	0.75809951	1.66E-34
DLD	CRCP	0.75815349	1.64E-34
DLD	PLEKHA3	0.75817267	1.63E-34
DLD	PPP2CA	0.75871635	1.37E-34
DLD	CTR9	0.75892443	1.28E-34
DLD	PDS5A	0.7589975	1.25E-34
DLD	RNF11	0.75902468	1.24E-34
DLD	TMEM167B	0.759309	1.13E-34
DLD	GHITM	0.75941915	1.1E-34
DLD	FEM1B	0.75954258	1.05E-34
DLD	KPNA3	0.75983034	9.62E-35
DLD	EXOC5	0.76018705	8.59E-35
DLD	RRN3	0.7604579	7.88E-35
DLD	CKAP5	0.76099732	6.63E-35
DLD	FBXO38	0.76119218	6.23E-35
DLD	GLYR1	0.76153929	5.58E-35
DLD	CYB5R4	0.76175369	5.21E-35
DLD	CUL1	0.76222589	4.47E-35
DLD	SMARCA5	0.76229415	4.38E-35
DLD	HMGN4	0.76268585	3.86E-35
DLD	ZC3H13	0.7630844	3.39E-35
DLD	TXNRD1	0.7630884	3.39E-35
DLD	ZFP91	0.76311684	3.36E-35
DLD	PPP1R12A	0.76350875	2.96E-35
DLD	CRKL	0.76356895	2.9E-35
DLD	RBBP4	0.76362312	2.85E-35
DLD	FCF1	0.76366779	2.81E-35
DLD	ADNP	0.76373074	2.75E-35
DLD	DCAF7	0.76399095	2.53E-35
DLD	MFN2	0.76408272	2.46E-35
DLD	ARL5A	0.76425492	2.32E-35
DLD	SMC3	0.76441889	2.2E-35
DLD	BCL2L13	0.76444831	2.18E-35
DLD	RTF1	0.7645246	2.13E-35
DLD	SUCLA2	0.76464489	2.05E-35
DLD	SMC1A	0.76471491	2E-35
DLD	LMBR1	0.76478416	1.96E-35
DLD	ATP2A2	0.76490415	1.88E-35
DLD	SMAD5	0.76535166	1.63E-35
DLD	FBXW2	0.76571413	1.44E-35
DLD	TCAF1	0.76579734	1.4E-35
DLD	ATG5	0.76622891	1.22E-35
DLD	CDC5L	0.76705113	9.31E-36
DLD	CPSF2	0.76721646	8.82E-36
DLD	TGOLN2	0.76752387	7.97E-36
DLD	OGDH	0.76874916	5.31E-36
DLD	UBE3A	0.76897708	4.92E-36
DLD	YTHDF3	0.77075917	2.71E-36
DLD	RNF4	0.77153048	2.09E-36
DLD	THRAP3	0.77237279	1.58E-36
DLD	CDC23	0.77259647	1.46E-36
DLD	PLEKHB2	0.77335952	1.13E-36

DLD	KPNA6	0.77355669	1.06E-36
DLD	ETF1	0.77415497	8.6E-37
DLD	RBM27	0.77431254	8.15E-37
DLD	ENOX2	0.77468487	7.18E-37
DLD	RB1	0.77484645	6.79E-37
DLD	EFCAB14	0.77495506	6.54E-37
DLD	HNRNPH2	0.77524292	5.93E-37
DLD	MFSD14A	0.7762515	4.19E-37
DLD	GCC1	0.7762929	4.13E-37
DLD	METTL14	0.77645292	3.91E-37
DLD	LARP7	0.77700853	3.23E-37
DLD	USP38	0.77811015	2.2E-37
DLD	MTPN	0.77948687	1.36E-37
DLD	SLC30A9	0.78177902	6.05E-38
DLD	MTM1	0.7830495	3.85E-38
DLD	PPP4R3B	0.78307084	3.82E-38
DLD	NCOA4	0.78349954	3.28E-38
DLD	PARG	0.78407742	2.66E-38
DLD	TNPO3	0.78413711	2.61E-38
DLD	CHCHD3	0.78473298	2.1E-38
DLD	VTA1	0.78578365	1.44E-38
DLD	EIF4G2	0.78584111	1.41E-38
DLD	XRN2	0.78592074	1.37E-38
DLD	HNRNPK	0.78660019	1.07E-38
DLD	PGGT1B	0.78705893	9.04E-39
DLD	SLC25A46	0.78737192	8.06E-39
DLD	NDUFS1	0.78795828	6.5E-39
DLD	RNF14	0.78982151	3.27E-39
DLD	CUL2	0.79090357	2.19E-39
DLD	BTBD1	0.79158447	1.7E-39
DLD	ZFAND3	0.79273388	1.1E-39
DLD	KIF5B	0.79421862	6.28E-40
DLD	TNPO1	0.79540021	4E-40
DLD	API5	0.79609182	3.07E-40
DLD	AP5M1	0.79632996	2.8E-40
DLD	PTPN11	0.79644688	2.68E-40
DLD	MATR3	0.79948904	8.24E-41
DLD	SNX4	0.80190879	3.17E-41
DLD	PNPLA8	0.80299006	2.06E-41
DLD	G3BP2	0.80320309	1.9E-41
DLD	UBE3C	0.8052248	8.41E-42
DLD	YME1L1	0.8087842	1.97E-42
DLD	EIF4E	0.81329766	2.98E-43
DLD	RAB18	0.81344155	2.8E-43
DLD	USP8	0.81578218	1.03E-43
DLD	BAZ1B	0.81631297	8.19E-44
DLD	WAC	0.81801661	3.91E-44
DLD	PSMC2	0.82854873	3.38E-46
DLD	SRPK2	0.83759528	4.38E-48
DLD	CAPZA2	0.87154383	2.3E-56
DLD	DLD	1	0
DLST	DLST	1	0
DLST	ZNF410	0.90351211	1.14E-66
DLST	SETD3	0.87991248	8.98E-59
DLST	SNW1	0.87215109	1.56E-56
DLST	TRAPPC6B	0.87132592	2.64E-56
DLST	NUMB	0.86488905	1.44E-54
DLST	CCNK	0.84734725	2.96E-50

DLST	MAPK1IP1L	0.84521692	9.09E-50
DLST	VIPAS39	0.84500854	1.01E-49
DLST	MED6	0.84461599	1.24E-49
DLST	PSEN1	0.84233273	4.03E-49
DLST	AKT1	0.84018354	1.2E-48
DLST	BAG5	0.83845193	2.86E-48
DLST	EIF2B2	0.83830795	3.07E-48
DLST	ARF6	0.82946862	2.2E-46
DLST	FCF1	0.82864703	3.23E-46
DLST	KTN1	0.82860596	3.29E-46
DLST	SNX6	0.8254932	1.39E-45
DLST	YY1	0.82434418	2.34E-45
DLST	PCBP1	0.82419535	2.5E-45
DLST	CDC42BPB	0.82271318	4.89E-45
DLST	PPP2R5E	0.82096982	1.06E-44
DLST	RCOR1	0.81748041	4.94E-44
DLST	MPP5	0.81489275	1.51E-43
DLST	PSMC6	0.81353956	2.69E-43
DLST	HNRNPK	0.81184274	5.5E-43
DLST	YWHAB	0.81079901	8.52E-43
DLST	CTCF	0.80682207	4.4E-42
DLST	SPTLC2	0.8067393	4.55E-42
DLST	TANK	0.80468324	1.05E-41
DLST	DNAJA2	0.80453389	1.11E-41
DLST	IST1	0.80442893	1.16E-41
DLST	ZNF207	0.80419492	1.27E-41
DLST	WDR20	0.80279525	2.23E-41
DLST	GMFB	0.80209519	2.95E-41
DLST	RAB6A	0.80102354	4.51E-41
DLST	DDX19A	0.80070012	5.12E-41
DLST	INO80	0.80065121	5.22E-41
DLST	FBXO34	0.79957439	7.96E-41
DLST	FBXO38	0.7993878	8.57E-41
DLST	TOX4	0.79895813	1.01E-40
DLST	KDM2A	0.79882368	1.07E-40
DLST	STAU1	0.79733946	1.9E-40
DLST	VEZT	0.79720464	2E-40
DLST	NCKAP1	0.79715607	2.04E-40
DLST	PPP3R1	0.79707325	2.11E-40
DLST	C14orf119	0.79626144	2.88E-40
DLST	ATP2C1	0.795917	3.28E-40
DLST	TOP1	0.79494245	4.77E-40
DLST	PTPN9	0.7948622	4.92E-40
DLST	FAM177A1	0.79480295	5.03E-40
DLST	HNRNPR	0.79418011	6.37E-40
DLST	PPFIA1	0.78914112	4.21E-39
DLST	SAP130	0.78900894	4.42E-39
DLST	CNIH1	0.78873604	4.89E-39
DLST	CDC23	0.78852965	5.27E-39
DLST	CSNK1A1	0.7884113	5.51E-39
DLST	CNOT8	0.78753566	7.59E-39
DLST	RAB8A	0.78748128	7.75E-39
DLST	PLEKHB2	0.78727288	8.36E-39
DLST	DYNC1I2	0.78721737	8.53E-39
DLST	ETF1	0.78709166	8.93E-39
DLST	EIF4E	0.78696245	9.36E-39
DLST	RTF1	0.78642597	1.14E-38
DLST	IWS1	0.78574982	1.46E-38

DLST	MAX	0.78550436	1.59E-38
DLST	KDM3B	0.78477613	2.07E-38
DLST	KHDRBS1	0.78302122	3.89E-38
DLST	HNRNPA2B1	0.78222799	5.16E-38
DLST	SDHD	0.78184438	5.91E-38
DLST	CPSF2	0.78143827	6.83E-38
DLST	CKAP5	0.78108107	7.75E-38
DLST	WDR1	0.78084021	8.44E-38
DLST	LRRC57	0.78036595	9.98E-38
DLST	OXSR1	0.78009598	1.1E-37
DLST	DUSP11	0.77988148	1.18E-37
DLST	GEMIN2	0.77980752	1.21E-37
DLST	ZNF143	0.77965047	1.28E-37
DLST	ZFYVE1	0.77920863	1.5E-37
DLST	SLC4A1AP	0.7791398	1.54E-37
DLST	MAP2K1	0.77810866	2.2E-37
DLST	ZFP91	0.77803056	2.26E-37
DLST	HAUS2	0.777751	2.49E-37
DLST	EAPP	0.77771751	2.52E-37
DLST	PSMC1	0.77695927	3.28E-37
DLST	EXOC1	0.77670288	3.59E-37
DLST	FAM91A1	0.77668337	3.61E-37
DLST	PDCD6IP	0.77639962	3.98E-37
DLST	ACVR1	0.77602713	4.53E-37
DLST	EIF2S1	0.77578278	4.92E-37
DLST	MNAT1	0.77560044	5.24E-37
DLST	KIAA1191	0.77534366	5.73E-37
DLST	ESYT2	0.77484709	6.79E-37
DLST	EIF4G2	0.77454645	7.53E-37
DLST	PIP5K1A	0.77406892	8.86E-37
DLST	RAB5B	0.77368257	1.01E-36
DLST	JKAMP	0.77360238	1.04E-36
DLST	SP3	0.77340696	1.11E-36
DLST	RAB3GAP1	0.77338134	1.12E-36
DLST	HARS2	0.77290198	1.32E-36
DLST	GPBP1L1	0.7720621	1.75E-36
DLST	SUPT16H	0.77200073	1.79E-36
DLST	RAB5A	0.77166673	2E-36
DLST	HMGB1	0.77146179	2.14E-36
DLST	TARDBP	0.77118825	2.35E-36
DLST	ZFYVE21	0.77107087	2.45E-36
DLST	RNF4	0.77092731	2.57E-36
DLST	RAB10	0.77075121	2.72E-36
DLST	CIPC	0.77049675	2.96E-36
DLST	LSM14A	0.77037685	3.09E-36
DLST	VPS35	0.76976311	3.79E-36
DLST	PPIG	0.76968572	3.89E-36
DLST	CLIP1	0.76950082	4.13E-36
DLST	RAB5C	0.76929606	4.43E-36
DLST	SUSD6	0.76926452	4.47E-36
DLST	DDX19B	0.76914493	4.66E-36
DLST	PATL1	0.76903283	4.83E-36
DLST	TNPO3	0.76849052	5.79E-36
DLST	RPE	0.76836653	6.03E-36
DLST	LRRFIP1	0.7680714	6.65E-36
DLST	RHOA	0.76797848	6.85E-36
DLST	HNRNPLL	0.76794055	6.94E-36
DLST	PXN	0.76769191	7.54E-36

DLST	U2AF2	0.76715854	8.99E-36
DLST	TRIP12	0.76697399	9.55E-36
DLST	YME1L1	0.76692819	9.69E-36
DLST	SART3	0.76682977	1E-35
DLST	USP10	0.76634034	1.18E-35
DLST	MATR3	0.766264	1.21E-35
DLST	HMGN4	0.76589621	1.36E-35
DLST	UBE2A	0.76588302	1.37E-35
DLST	EFR3A	0.76583253	1.39E-35
DLST	SYNJ2BP	0.76580311	1.4E-35
DLST	SPTLC1	0.76572787	1.44E-35
DLST	AGFG1	0.76536257	1.62E-35
DLST	MTPN	0.76488165	1.89E-35
DLST	TMEM127	0.76453396	2.12E-35
DLST	ZNF146	0.76430258	2.29E-35
DLST	SNRNP27	0.7640567	2.48E-35
DLST	TAF2	0.76378763	2.7E-35
DLST	PCNX4	0.76361516	2.86E-35
DLST	CNOT9	0.76342726	3.04E-35
DLST	C2CD3	0.76328953	3.18E-35
DLST	DHDDS	0.76309476	3.38E-35
DLST	RAB22A	0.76275937	3.77E-35
DLST	CUL4A	0.76263122	3.93E-35
DLST	CAB39	0.76258868	3.98E-35
DLST	WDR82	0.76251618	4.07E-35
DLST	AMFR	0.76247696	4.13E-35
DLST	RAB21	0.76202173	4.78E-35
DLST	STRN3	0.76195973	4.87E-35
DLST	AGPS	0.76188408	4.99E-35
DLST	KRR1	0.76112356	6.37E-35
DLST	SBF2	0.76102342	6.58E-35
DLST	MAP3K2	0.76102052	6.58E-35
DLST	TAOK3	0.76098859	6.65E-35
DLST	SIPA1L1	0.76058923	7.55E-35
DLST	CWC22	0.76045732	7.88E-35
DLST	VAMP3	0.7602869	8.32E-35
DLST	GORASP2	0.7602669	8.37E-35
DLST	ARL8B	0.76021618	8.51E-35
DLST	EFCAB14	0.75998555	9.16E-35
DLST	UBA2	0.75920749	1.17E-34
DLST	GALC	0.75916328	1.19E-34
DLST	PPM1A	0.75891921	1.28E-34
DLST	THRAP3	0.75859567	1.42E-34
DLST	DCTD	0.75841526	1.51E-34
DLST	PAFAH1B2	0.7580674	1.68E-34
DLST	OGFOD1	0.75784917	1.8E-34
DLST	CDC42	0.75782334	1.81E-34
DLST	ELF4	0.75780123	1.83E-34
DLST	SKAP2	0.75780055	1.83E-34
DLST	SERINC3	0.75775532	1.85E-34
DLST	MFSD14B	0.75749898	2.01E-34
DLST	UBE2D3	0.75742352	2.06E-34
DLST	GTF2H3	0.75710172	2.28E-34
DLST	DR1	0.75706521	2.3E-34
DLST	ATP2A2	0.75704929	2.31E-34
DLST	SRSF3	0.75700654	2.34E-34
DLST	RAB1A	0.7569048	2.42E-34
DLST	WDR44	0.75688048	2.44E-34

DLST	SBDS	0.75664734	2.62E-34
DLST	BTBD10	0.75601817	3.19E-34
DLST	ARHGAP21	0.75600372	3.21E-34
DLST	RPRD1B	0.75558987	3.65E-34
DLST	SIN3A	0.75535313	3.93E-34
DLST	XRN2	0.75522863	4.08E-34
DLST	CBFB	0.75515012	4.18E-34
DLST	SELENOT	0.75507804	4.28E-34
DLST	WDFY1	0.75447915	5.15E-34
DLST	ARFGEF1	0.75426412	5.51E-34
DLST	QTRT2	0.75410661	5.78E-34
DLST	MFN2	0.75382937	6.3E-34
DLST	RAB35	0.75363739	6.68E-34
DLST	YEATS2	0.75304547	8.02E-34
DLST	C16orf70	0.75294935	8.26E-34
DLST	NDUFS1	0.75288917	8.41E-34
DLST	MTMR2	0.75276047	8.75E-34
DLST	SMIM15	0.75261369	9.15E-34
DLST	NIPA2	0.75251823	9.43E-34
DLST	SLC25A24	0.75235309	9.92E-34
DLST	NUP98	0.75234244	9.95E-34
DLST	PICALM	0.7522797	1.01E-33
DLST	SNX1	0.75220358	1.04E-33
DLST	ITCH	0.75172327	1.2E-33
DLST	CNOT6	0.75167916	1.22E-33
DLST	ZCCHC9	0.75129394	1.37E-33
DLST	SNRNP200	0.75125707	1.39E-33
DLST	PARG	0.75123519	1.4E-33
DLST	ATP6V1D	0.7511875	1.42E-33
DLST	CAPZA2	0.75107971	1.46E-33
DLST	ITPK1	0.7509966	1.5E-33
DLST	UBE3C	0.75097137	1.51E-33
DLST	CALM1	0.75096023	1.52E-33
DLST	SSRP1	0.75023387	1.89E-33
DLST	EIF4G3	0.75000305	2.03E-33
DLST	ARCN1	0.74969666	2.23E-33
DLST	TMCC1	0.74963335	2.27E-33
DLST	TLK1	0.74901778	2.73E-33
DLST	MRPL49	0.74830593	3.39E-33
DLST	TSN	0.74829804	3.39E-33
DLST	CDYL	0.74781259	3.93E-33
DLST	NAB1	0.747798	3.94E-33
DLST	COQ10B	0.74746366	4.36E-33
DLST	ZDHHC5	0.74739584	4.45E-33
DLST	LARS2	0.74739304	4.45E-33
DLST	B4GALT5	0.74732483	4.55E-33
DLST	KPNB1	0.74717154	4.76E-33
DLST	PDCD10	0.747169	4.76E-33
DLST	PRKDC	0.74706923	4.91E-33
DLST	DHX36	0.74681088	5.3E-33
DLST	COMMD2	0.74668605	5.5E-33
DLST	RNF168	0.74667537	5.52E-33
DLST	FRYL	0.7465222	5.78E-33
DLST	GTF2A1	0.74643868	5.92E-33
DLST	PWP1	0.74639724	6E-33
DLST	GDE1	0.74627559	6.22E-33
DLST	LARP7	0.74624481	6.27E-33
DLST	PTPN12	0.7462396	6.28E-33

DLST	ANAPC10	0.74621414	6.33E-33
DLST	FAF2	0.7460943	6.56E-33
DLST	DDX18	0.74596156	6.83E-33
DLST	MAT2B	0.74587647	7E-33
DLST	RTF2	0.74576382	7.24E-33
DLST	GATAD2A	0.74569618	7.39E-33
DLST	ANXA7	0.74550706	7.81E-33
DLST	RTN4	0.74510567	8.8E-33
DLST	SMNDC1	0.74506025	8.92E-33
DLST	CAPRIN1	0.74500909	9.05E-33
DLST	TMEM170A	0.74497792	9.14E-33
DLST	WASL	0.74493757	9.25E-33
DLST	GLYR1	0.74462399	1.01E-32
DLST	PPHLN1	0.74450891	1.05E-32
DLST	SIAH1	0.74445146	1.07E-32
DLST	MARK2	0.74444852	1.07E-32
DLST	HNRNPU	0.74410248	1.18E-32
DLST	HNRNPUL1	0.74405566	1.2E-32
DLST	TUBA1B	0.74390626	1.25E-32
DLST	TAX1BP1	0.74372265	1.32E-32
DLST	EHBP1	0.74365797	1.35E-32
DLST	DENND6A	0.74359343	1.37E-32
DLST	SMC6	0.74350723	1.41E-32
DLST	ATXN3	0.74348968	1.42E-32
DLST	ZNFX1	0.74339111	1.46E-32
DLST	TXNRD1	0.74324911	1.52E-32
DLST	ZMAT2	0.74316932	1.56E-32
DLST	LRRFIP2	0.74297062	1.65E-32
DLST	MCMBP	0.74271837	1.78E-32
DLST	DNAJC13	0.74267027	1.8E-32
DLST	APEX1	0.74234678	1.98E-32
DLST	AP003108.2	0.74232931	1.99E-32
DLST	CIAPIN1	0.74227613	2.02E-32
DLST	RNF111	0.74216398	2.09E-32
DLST	ERBIN	0.74190801	2.25E-32
DLST	ARMC8	0.74188456	2.27E-32
DLST	METAP1	0.74127564	2.71E-32
DLST	SLMAP	0.74121005	2.76E-32
DLST	ACTR3	0.74086115	3.05E-32
DLST	ATMIN	0.7408041	3.11E-32
DLST	SET	0.74069753	3.2E-32
DLST	G3BP1	0.74057762	3.32E-32
DLST	ABRAXAS2	0.74051493	3.38E-32
DLST	RAB7A	0.74045017	3.44E-32
DLST	PEX26	0.74029477	3.6E-32
DLST	NAA30	0.74015267	3.75E-32
DLST	PSME3	0.74008671	3.82E-32
DLST	RLIM	0.74006407	3.85E-32
DLST	PTBP3	0.73996995	3.95E-32
DLST	PRPF40A	0.73981368	4.14E-32
DLST	TBL1XR1	0.73900161	5.23E-32
DLST	YWHAG	0.73884199	5.48E-32
DLST	BZW1	0.73870643	5.69E-32
DLST	HNRNPC	0.73852727	5.99E-32
DLST	DAZAP2	0.73850816	6.03E-32
DLST	BAZ1B	0.73847969	6.08E-32
DLST	SCP2	0.7384625	6.11E-32
DLST	PTPN1	0.73827215	6.45E-32

DLST	KLF7	0.73803283	6.91E-32
DLST	CAMKK2	0.73791579	7.14E-32
DLST	MEF2A	0.73788192	7.21E-32
DLST	DLAT	0.73776586	7.46E-32
DLST	TPP2	0.73775195	7.49E-32
DLST	SPPL3	0.73771846	7.56E-32
DLST	ADNP	0.7376962	7.61E-32
DLST	HNRNPD	0.73767939	7.64E-32
DLST	ARHGAP5	0.73763428	7.74E-32
DLST	HACD2	0.73759481	7.83E-32
DLST	SH3GLB1	0.73751431	8.01E-32
DLST	ALS2	0.73729594	8.53E-32
DLST	UBAP1	0.73721438	8.73E-32
DLST	TM9SF4	0.73718347	8.81E-32
DLST	CHMP2B	0.73707638	9.08E-32
DLST	PDS5A	0.73692148	9.49E-32
DLST	PUM1	0.73690687	9.53E-32
DLST	ANKRD27	0.73690489	9.54E-32
DLST	CTNNA1	0.73680688	9.81E-32
DLST	INCENP	0.73661799	1.03E-31
DLST	C16orf72	0.736602	1.04E-31
DLST	DDX27	0.7365153	1.07E-31
DLST	NPTN	0.73645858	1.08E-31
DLST	HNRNPUL2	0.7363403	1.12E-31
DLST	ELAVL1	0.7359132	1.26E-31
DLST	TSG101	0.73591218	1.26E-31
DLST	GANAB	0.73560887	1.38E-31
DLST	CXorf56	0.73514972	1.57E-31
DLST	POLR2D	0.7351013	1.59E-31
DLST	NCL	0.73505872	1.61E-31
DLST	ARHGAP1	0.73505775	1.61E-31
DLST	PIGS	0.73448552	1.89E-31
DLST	HNRNPA0	0.73422766	2.04E-31
DLST	AP2B1	0.73415587	2.08E-31
DLST	PPP2R5D	0.73395808	2.2E-31
DLST	SPIN1	0.73382316	2.28E-31
DLST	C1D	0.73376644	2.32E-31
DLST	ARPP19	0.73337537	2.59E-31
DLST	STAMBP	0.73318886	2.73E-31
DLST	FKBP15	0.7330234	2.86E-31
DLST	CNOT1	0.73287987	2.97E-31
DLST	HMGXB3	0.73263792	3.18E-31
DLST	MRPL30	0.73254686	3.26E-31
DLST	ARL6IP6	0.73253598	3.27E-31
DLST	HNRNPH2	0.73250431	3.3E-31
DLST	MAP4K4	0.73242049	3.38E-31
DLST	CAST	0.7318867	3.92E-31
DLST	CPSF6	0.73184182	3.97E-31
DLST	SSB	0.73176141	4.06E-31
DLST	RAB14	0.73172513	4.11E-31
DLST	MOB1A	0.73159803	4.25E-31
DLST	LASP1	0.73140336	4.49E-31
DLST	ZNF720	0.73098118	5.05E-31
DLST	HMGXB4	0.73096213	5.08E-31
DLST	UBE2N	0.73083763	5.25E-31
DLST	MIS18BP1	0.7308033	5.3E-31
DLST	USP8	0.73073674	5.4E-31
DLST	PARN	0.73018317	6.3E-31

DLST	CSNK1G3	0.73001788	6.59E-31
DLST	GNB1	0.72997826	6.67E-31
DLST	KATNBL1	0.72950951	7.59E-31
DLST	TMEM248	0.72930846	8.02E-31
DLST	MED17	0.72929653	8.04E-31
DLST	UBE2D2	0.72914127	8.4E-31
DLST	AIMP1	0.7290902	8.52E-31
DLST	DENR	0.72905379	8.6E-31
DLST	FBXW2	0.72902221	8.68E-31
DLST	GCC2	0.72874441	9.36E-31
DLST	GLG1	0.72867436	9.55E-31
DLST	VHL	0.72866455	9.57E-31
DLST	RALB	0.72865336	9.6E-31
DLST	ADAM10	0.72859813	9.75E-31
DLST	CHCHD3	0.72843456	1.02E-30
DLST	TFE3	0.72829317	1.06E-30
DLST	TTLL5	0.72821585	1.08E-30
DLST	RBM27	0.72815924	1.1E-30
DLST	TGOLN2	0.72810964	1.11E-30
DLST	MED14	0.72807257	1.13E-30
DLST	COPG2	0.72795952	1.16E-30
DLST	NEK7	0.72785558	1.2E-30
DLST	LRP10	0.72780072	1.21E-30
DLST	TNPO1	0.72770598	1.25E-30
DLST	CYFIP1	0.72767139	1.26E-30
DLST	EIF1AD	0.72762746	1.27E-30
DLST	LAMTOR3	0.72759966	1.28E-30
DLST	C16orf87	0.72720603	1.43E-30
DLST	AMBRA1	0.7271836	1.44E-30
DLST	FZR1	0.7270096	1.51E-30
DLST	TRAF3	0.72685075	1.57E-30
DLST	NPM1	0.72680754	1.59E-30
DLST	PPP1R8	0.72673887	1.62E-30
DLST	SP1	0.7265318	1.72E-30
DLST	TBK1	0.72642466	1.77E-30
DLST	AHNAK	0.72612211	1.92E-30
DLST	SRFBP1	0.72601727	1.97E-30
DLST	MIER1	0.72579214	2.1E-30
DLST	STAM	0.72570079	2.15E-30
DLST	PRELID3B	0.7256455	2.18E-30
DLST	RNF121	0.72557649	2.22E-30
DLST	KBTBD2	0.72553578	2.25E-30
DLST	BAZ1A	0.72553306	2.25E-30
DLST	TMX1	0.72522898	2.44E-30
DLST	VAPA	0.72521567	2.45E-30
DLST	PUM2	0.72521079	2.45E-30
DLST	DHX35	0.72505032	2.56E-30
DLST	SPOPL	0.72494703	2.64E-30
DLST	RNF26	0.72471922	2.8E-30
DLST	ACTR2	0.72467306	2.84E-30
DLST	ZNF227	0.72439009	3.06E-30
DLST	PSMC2	0.72420596	3.22E-30
DLST	COPS2	0.72385512	3.54E-30
DLST	TMEM87A	0.7236028	3.79E-30
DLST	SRSF1	0.72360054	3.79E-30
DLST	VCL	0.72343105	3.97E-30
DLST	RRN3	0.72330529	4.1E-30
DLST	UBE3A	0.72329389	4.11E-30

DLST	KPNA4	0.72323037	4.19E-30
DLST	METTL9	0.72316762	4.26E-30
DLST	ACAP2	0.72310773	4.33E-30
DLST	SEC23A	0.7230572	4.38E-30
DLST	PURB	0.7228836	4.59E-30
DLST	PFDN1	0.7227939	4.7E-30
DLST	ELF1	0.72271284	4.81E-30
DLST	BCL9L	0.72271038	4.81E-30
DLST	EXT2	0.72252132	5.06E-30
DLST	IRF2	0.72238356	5.25E-30
DLST	FNTB	0.72229328	5.38E-30
DLST	ARIH1	0.72222698	5.47E-30
DLST	UBA3	0.72206488	5.72E-30
DLST	PEX13	0.72205674	5.73E-30
DLST	ZC3H11A	0.72194716	5.9E-30
DLST	SNX4	0.72191134	5.96E-30
DLST	TAF9	0.72181528	6.11E-30
DLST	ZC3H15	0.72181263	6.11E-30
DLST	RBM22	0.72177954	6.17E-30
DLST	ABI1	0.72172342	6.26E-30
DLST	KRAS	0.72171477	6.28E-30
DLST	KIAA1143	0.72163513	6.41E-30
DLST	CTBP2	0.72144414	6.75E-30
DLST	SMARCA5	0.72132245	6.97E-30
DLST	MAGT1	0.72118717	7.22E-30
DLST	RNPS1	0.72086372	7.87E-30
DLST	EXOC6B	0.72083816	7.92E-30
DLST	RBM7	0.72069112	8.24E-30
DLST	C11orf58	0.72046118	8.76E-30
DLST	SMURF1	0.72032941	9.07E-30
DLST	ZNF274	0.72022935	9.31E-30
DLST	FAM120A	0.72022408	9.33E-30
DLST	PPP2CA	0.720026	9.83E-30
DLST	SEC23IP	0.71989574	1.02E-29
DLST	GFM1	0.71984709	1.03E-29
DLST	RAD21	0.71983766	1.03E-29
DLST	RUFY1	0.71959977	1.1E-29
DLST	CAPZA1	0.71930373	1.19E-29
DLST	PRRC2C	0.71922888	1.21E-29
DLST	TM2D1	0.71909892	1.26E-29
DLST	ZPR1	0.71886792	1.33E-29
DLST	PCYT1A	0.7187443	1.38E-29
DLST	FASTKD2	0.71871675	1.39E-29
DLST	TMEM87B	0.71867927	1.4E-29
DLST	GPD2	0.71866913	1.41E-29
DLST	DPF2	0.71865757	1.41E-29
DLST	SOCS4	0.71860686	1.43E-29
DLST	SF3B3	0.71853059	1.46E-29
DLST	ZNF609	0.71840393	1.51E-29
DLST	ATF2	0.71814778	1.61E-29
DLST	GRAMD2B	0.71793927	1.7E-29
DLST	UBE2Z	0.71786536	1.74E-29
DLST	DIAPH1	0.71748527	1.92E-29
DLST	ZMYM5	0.71737337	1.97E-29
DLST	PPP3CA	0.71733231	2E-29
DLST	PSMF1	0.71695458	2.2E-29
DLST	DCAF1	0.71691488	2.23E-29
DLST	ZFAND6	0.71679748	2.29E-29

DLST	PHAX	0.71671901	2.34E-29
DLST	RBFOX2	0.71671687	2.34E-29
DLST	CCNG2	0.71670873	2.35E-29
DLST	YTHDF1	0.71668642	2.36E-29
DLST	ZNF562	0.71665467	2.38E-29
DLST	ANKRD11	0.71663541	2.39E-29
DLST	MBTPS1	0.71652342	2.46E-29
DLST	NUP62	0.71647176	2.5E-29
DLST	NUP58	0.71644069	2.52E-29
DLST	SNX12	0.71643608	2.52E-29
DLST	TMOD3	0.71639059	2.55E-29
DLST	ABI2	0.71622786	2.66E-29
DLST	SLC25A46	0.71614303	2.72E-29
DLST	FBXO42	0.71604532	2.79E-29
DLST	SERBP1	0.71604355	2.79E-29
DLST	SLAIN2	0.71602758	2.8E-29
DLST	ACTN1	0.71600667	2.82E-29
DLST	BTBD1	0.71599141	2.83E-29
DLST	MRPL44	0.71594359	2.87E-29
DLST	CCDC115	0.71582687	2.95E-29
DLST	MTA2	0.71560101	3.13E-29
DLST	HNRNPF	0.71557301	3.16E-29
DLST	DIS3	0.7155545	3.17E-29
DLST	NCOA4	0.71550108	3.21E-29
DLST	SAE1	0.71549666	3.22E-29
DLST	CSNK2A1	0.71543863	3.27E-29
DLST	DNAJC14	0.71521694	3.46E-29
DLST	SPRED2	0.71492857	3.73E-29
DLST	MORF4L2	0.71469043	3.97E-29
DLST	APLP2	0.71452582	4.14E-29
DLST	TAF1B	0.71450855	4.16E-29
DLST	ARHGEF12	0.71439029	4.29E-29
DLST	API5	0.71420217	4.5E-29
DLST	ZNF250	0.71412746	4.59E-29
DLST	DOCK7	0.71406627	4.66E-29
DLST	COPS8	0.71393903	4.81E-29
DLST	KCTD5	0.71392118	4.84E-29
DLST	PPP1CC	0.71372643	5.09E-29
DLST	NAA50	0.71367443	5.15E-29
DLST	STRN	0.71365001	5.19E-29
DLST	SNX5	0.71355207	5.32E-29
DLST	ZFAND3	0.7134115	5.51E-29
DLST	TEP1	0.71335941	5.59E-29
DLST	PCNP	0.71335237	5.6E-29
DLST	NUDCD3	0.71327414	5.71E-29
DLST	SP2	0.71321702	5.8E-29
DLST	PA2G4	0.7128871	6.31E-29
DLST	GMPR2	0.71287927	6.32E-29
DLST	AP5M1	0.71276674	6.51E-29
DLST	RAB1B	0.71251486	6.94E-29
DLST	HCCS	0.71235285	7.23E-29
DLST	JMJD1C	0.71234101	7.26E-29
DLST	ALKBH1	0.71233177	7.27E-29
DLST	LAPTM4A	0.71205472	7.81E-29
DLST	VKORC1L1	0.71204228	7.83E-29
DLST	FEM1C	0.71201659	7.88E-29
DLST	NFE2L2	0.71199889	7.92E-29
DLST	CHRAC1	0.71186252	8.2E-29

DLST	TSC22D2	0.71181744	8.3E-29
DLST	SYPL1	0.71154292	8.9E-29
DLST	UBE2H	0.71152788	8.93E-29
DLST	HIGD1A	0.71116426	9.8E-29
DLST	NAA15	0.71095775	1.03E-28
DLST	POLR2C	0.71080601	1.07E-28
DLST	CALM2	0.71077146	1.08E-28
DLST	CAND1	0.71070816	1.1E-28
DLST	RAD51B	0.71069463	1.1E-28
DLST	TAOK1	0.71052781	1.15E-28
DLST	TRA2B	0.71006802	1.29E-28
DLST	BMPR2	0.70990328	1.35E-28
DLST	REEP3	0.70986887	1.36E-28
DLST	SLC30A9	0.70984907	1.37E-28
DLST	OGDH	0.70983756	1.37E-28
DLST	SMC4	0.70961751	1.45E-28
DLST	RHBDD1	0.70945702	1.51E-28
DLST	PGM2	0.70920232	1.61E-28
DLST	PIP4K2C	0.70912879	1.64E-28
DLST	CDK12	0.70912479	1.64E-28
DLST	CHD9	0.70912419	1.64E-28
DLST	PPP1CB	0.70907447	1.66E-28
DLST	ADAM17	0.70867242	1.84E-28
DLST	SLC12A6	0.70858336	1.88E-28
DLST	YPEL5	0.70837642	1.98E-28
DLST	XIAP	0.70828463	2.03E-28
DLST	WIPF2	0.70823405	2.06E-28
DLST	G3BP2	0.70821162	2.07E-28
DLST	PPP4R3B	0.70804956	2.15E-28
DLST	SELENOF	0.70787241	2.25E-28
DLST	CHURC1	0.70785486	2.26E-28
DLST	PITPNB	0.70782202	2.28E-28
DLST	DOCK9	0.70782076	2.28E-28
DLST	SUMO2	0.70765045	2.38E-28
DLST	SLC35A4	0.70761132	2.41E-28
DLST	DDX23	0.7075694	2.43E-28
DLST	MFSD14A	0.70741819	2.52E-28
DLST	ARF3	0.70735555	2.56E-28
DLST	NSRP1	0.70733228	2.58E-28
DLST	VPS29	0.70722303	2.65E-28
DLST	CRCP	0.70701337	2.79E-28
DLST	DLD	0.70690069	2.87E-28
DLST	C5orf51	0.70688927	2.88E-28
DLST	PTPN11	0.70675622	2.98E-28
DLST	EMC2	0.70674705	2.99E-28
DLST	EMC3	0.70673268	3E-28
DLST	PGGT1B	0.70623539	3.39E-28
DLST	NCBP2	0.70621488	3.41E-28
DLST	RAD23B	0.7061318	3.48E-28
DLST	BFAR	0.7057763	3.8E-28
DLST	JRKL	0.70544559	4.13E-28
DLST	UBE2E3	0.70541734	4.16E-28
DLST	CAMSAP1	0.70531677	4.26E-28
DLST	CTTNBP2NL	0.70510747	4.49E-28
DLST	PEX2	0.70503751	4.57E-28
DLST	KPNA6	0.70495285	4.67E-28
DLST	UBQLN2	0.70483188	4.81E-28
DLST	SKP1	0.70475212	4.91E-28

DLST	MUL1	0.70473469	4.93E-28
DLST	PSMD7	0.70453251	5.18E-28
DLST	RBM12	0.70426331	5.54E-28
DLST	GNAI3	0.70421384	5.61E-28
DLST	ZFP64	0.70418734	5.64E-28
DLST	CASP2	0.70417752	5.66E-28
DLST	TDP1	0.70409146	5.78E-28
DLST	WAC	0.70407244	5.8E-28
DLST	PARP3	0.70392431	6.02E-28
DLST	PAK2	0.70383158	6.16E-28
DLST	MAPK14	0.70366481	6.42E-28
DLST	KLF3	0.70351405	6.66E-28
DLST	CCNI	0.70348041	6.72E-28
DLST	BMS1	0.70343043	6.8E-28
DLST	KLHL20	0.70337068	6.9E-28
DLST	ORC4	0.70312882	7.33E-28
DLST	YIPF5	0.7030888	7.4E-28
DLST	FEZ2	0.70308518	7.4E-28
DLST	B3GNT2	0.70306288	7.45E-28
DLST	MAPK6	0.70306028	7.45E-28
DLST	UBQLN1	0.70303951	7.49E-28
DLST	SLC39A9	0.70297907	7.6E-28
DLST	PLEKHA1	0.70280079	7.94E-28
DLST	TWF1	0.70229349	8.99E-28
DLST	CXorf38	0.70225945	9.07E-28
DLST	MAPRE1	0.70219599	9.21E-28
DLST	UNG	0.70186743	9.98E-28
DLST	RBBP4	0.70174277	1.03E-27
DLST	ZC3H13	0.70173425	1.03E-27
DLST	SNX9	0.70173361	1.03E-27
DLST	TMEM43	0.70169853	1.04E-27
DLST	SZRD1	0.70169006	1.04E-27
DLST	ATG16L1	0.70167732	1.05E-27
DLST	RDH14	0.70161546	1.06E-27
DLST	UVRAG	0.7016126	1.06E-27
DLST	JAK1	0.70152261	1.09E-27
DLST	ITSN1	0.70142018	1.11E-27
DLST	SLC30A6	0.70140519	1.12E-27
DLST	MED28	0.70127075	1.16E-27
DLST	JAG1	0.70125432	1.16E-27
DLST	ABCC1	0.70124237	1.16E-27
DLST	CUL4B	0.70120481	1.17E-27
DLST	PNRC2	0.70106006	1.22E-27
DLST	CREB1	0.70099231	1.24E-27
DLST	SNX27	0.70093025	1.26E-27
DLST	MACF1	0.70088392	1.27E-27
DLST	PGRMC1	0.70078194	1.3E-27
DLST	CHMP5	0.70073316	1.32E-27
DLST	EFCAB11	0.70063579	1.35E-27
DLST	KLHL7	0.70030098	1.46E-27
DLST	FIP1L1	0.70024331	1.48E-27
DLST	ICE2	0.70004339	1.56E-27
DLST	SESTD1	0.69989239	1.62E-27
DLST	PDHX	0.69979895	1.65E-27
DLST	EIF2AK2	0.69976615	1.67E-27
DLST	NUP107	0.69976117	1.67E-27
DLST	LRCH1	0.69970972	1.69E-27
DLST	WDR26	0.69969754	1.69E-27

DLST	HIPK1	0.69969003	1.7E-27
DLST	PELI1	0.69968912	1.7E-27
DLST	XRCC1	0.69927499	1.88E-27
DLST	BBS7	0.69923861	1.89E-27
DLST	EPB41L1	0.69921256	1.91E-27
DLST	KPNA1	0.69914296	1.94E-27
DLST	ADAR	0.69873015	2.14E-27
DLST	HUS1	0.69871125	2.15E-27
DLST	RNF11	0.69859912	2.21E-27
DLST	LNPK	0.69854945	2.24E-27
DLST	DHX9	0.69842059	2.31E-27
DLST	MGAT4B	0.69841812	2.31E-27
DLST	NCOA3	0.69837019	2.34E-27
DLST	CNNM4	0.69823581	2.41E-27
DLST	ANKMY2	0.69820804	2.43E-27
DLST	PTK2	0.69816749	2.45E-27
DLST	GNAQ	0.69809041	2.5E-27
DLST	TCEA1	0.69805772	2.52E-27
DLST	ATAD1	0.69802538	2.54E-27
DLST	PPP1R12A	0.69801577	2.54E-27
DLST	YTHDF3	0.69789637	2.62E-27
DLST	KCTD9	0.69786066	2.64E-27
DLST	ASAP2	0.697727	2.73E-27
DLST	INTS12	0.69771411	2.74E-27
DLST	IMPA1	0.69763934	2.79E-27
DLST	AFAP1	0.69761997	2.8E-27
DLST	CUL2	0.69750867	2.88E-27
DLST	USP34	0.6974296	2.93E-27
DLST	SNX2	0.69740815	2.95E-27
DLST	RAP1A	0.69733053	3E-27
DLST	C5orf15	0.69732402	3.01E-27
DLST	TMEM167B	0.69730093	3.02E-27
DLST	ANP32A	0.69706444	3.2E-27
DLST	CDK2	0.6970269	3.23E-27
DLST	TMED7	0.69701192	3.24E-27
DLST	TFDP1	0.69700989	3.24E-27
DLST	SENP1	0.6969897	3.26E-27
DLST	CAP1	0.69695254	3.29E-27
DLST	SLC9A6	0.69686539	3.36E-27
DLST	SGPL1	0.69671807	3.48E-27
DLST	CMTM6	0.69670754	3.49E-27
DLST	VTA1	0.69662488	3.55E-27
DLST	GABPB1	0.69658561	3.59E-27
DLST	DCTN5	0.6965213	3.64E-27
DLST	AFF4	0.69648008	3.68E-27
DLST	IQGAP1	0.69643402	3.72E-27
DLST	HADHA	0.69635014	3.8E-27
DLST	TRAFD1	0.69621434	3.92E-27
DLST	EAF1	0.69601093	4.12E-27
DLST	ATP11B	0.69595776	4.17E-27
DLST	SPDL1	0.69585166	4.28E-27
DLST	PFKP	0.69580061	4.33E-27
DLST	RNF41	0.69567092	4.47E-27
DLST	ESF1	0.69549964	4.65E-27
DLST	TERF1	0.69537915	4.79E-27
DLST	VTI1A	0.69533671	4.84E-27
DLST	SLC25A13	0.69526624	4.92E-27
DLST	ECD	0.69523231	4.96E-27

DLST	RMDN3	0.69518222	5.02E-27
DLST	GNG12	0.69442093	6.01E-27
DLST	MYD88	0.69429113	6.2E-27
DLST	PAICS	0.69419316	6.35E-27
DLST	PPP1R2	0.69410406	6.48E-27
DLST	DYRK2	0.69398131	6.67E-27
DLST	CAPZB	0.69396122	6.71E-27
DLST	RAPH1	0.69391507	6.78E-27
DLST	NRAS	0.6938933	6.82E-27
DLST	CDC42SE1	0.6938433	6.9E-27
DLST	ROCK1	0.69365458	7.21E-27
DLST	RPS6KA3	0.69357069	7.36E-27
DLST	UBAP2L	0.69342425	7.62E-27
DLST	BECN1	0.69331778	7.81E-27
DLST	NF1	0.69328955	7.86E-27
DLST	MTX2	0.69327097	7.9E-27
DLST	PTPRA	0.69321935	7.99E-27
DLST	UBN1	0.6930747	8.27E-27
DLST	PSMD10	0.69299141	8.44E-27
DLST	YWHAQ	0.69294623	8.53E-27
DLST	SMARCC1	0.69290922	8.6E-27
DLST	VPS37A	0.69273591	8.96E-27
DLST	HNRNPA1	0.69271676	9E-27
DLST	LARP4B	0.69256907	9.32E-27
DLST	RNF145	0.69255131	9.36E-27
DLST	ITGB1	0.69254803	9.37E-27
DLST	ZNHIT6	0.69254144	9.38E-27
DLST	RNASEH1	0.69253568	9.39E-27
DLST	NKIRAS2	0.6924975	9.48E-27
DLST	R3HDM1	0.69234892	9.81E-27
DLST	PHACTR4	0.69228302	9.97E-27
DLST	PDS5B	0.69227336	9.99E-27
DLST	TES	0.69226188	1E-26
DLST	AHCTF1	0.69224322	1.01E-26
DLST	NRBF2	0.6922332	1.01E-26
DLST	CLINT1	0.69216554	1.02E-26
DLST	AQR	0.69201757	1.06E-26
DLST	UBTD2	0.69181672	1.11E-26
DLST	OCIAD1	0.69166805	1.15E-26
DLST	PNPLA8	0.69162836	1.16E-26
DLST	TCF20	0.6916118	1.17E-26
DLST	VTI1B	0.69159004	1.17E-26
DLST	GEMIN5	0.69143981	1.22E-26
DLST	MAIP1	0.69143156	1.22E-26
DLST	ARPC4	0.6912996	1.26E-26
DLST	DYNC1LI1	0.69128061	1.26E-26
DLST	CCDC43	0.69121197	1.28E-26
DLST	BRCC3	0.69120683	1.28E-26
DLST	SMC3	0.69120617	1.28E-26
DLST	RSRC1	0.69117423	1.29E-26
DLST	ACTN4	0.69116924	1.29E-26
DLST	MGRN1	0.69114617	1.3E-26
DLST	LNPEP	0.69093549	1.37E-26
DLST	SPTY2D1	0.6906332	1.47E-26
DLST	FAM220A	0.69054046	1.5E-26
DLST	RSF1	0.69052092	1.51E-26
DLST	NUFIP2	0.69051687	1.51E-26
DLST	GLRX5	0.69043004	1.54E-26

DLST	ICMT	0.69037216	1.56E-26
DLST	ARMC1	0.69019917	1.62E-26
DLST	CSDE1	0.69017416	1.63E-26
DLST	SUMO3	0.68992534	1.73E-26
DLST	RAB28	0.68985241	1.76E-26
DLST	ZDHHC20	0.68963268	1.85E-26
DLST	PPP4R2	0.68959788	1.87E-26
DLST	RAB18	0.68951143	1.91E-26
DLST	DPM1	0.68947011	1.93E-26
DLST	RASA1	0.68941371	1.95E-26
DLST	PPIL4	0.68933171	1.99E-26
DLST	KDM4A	0.68928233	2.01E-26
DLST	TYW1	0.68924251	2.03E-26
DLST	UBA6	0.68916901	2.07E-26
DLST	EDC3	0.6890319	2.13E-26
DLST	TFCP2	0.6889996	2.15E-26
DLST	CCDC90B	0.68894829	2.17E-26
DLST	RALA	0.68894256	2.18E-26
DLST	PI4K2B	0.68893499	2.18E-26
DLST	SNAPC1	0.68884935	2.22E-26
DLST	RP2	0.68855707	2.38E-26
DLST	RC3H2	0.68854712	2.39E-26
DLST	RPL7L1	0.68838805	2.48E-26
DLST	PLRG1	0.68838651	2.48E-26
DLST	RAB11A	0.68835954	2.49E-26
DLST	PPP2R2A	0.68830244	2.53E-26
DLST	PRPF4	0.68829684	2.53E-26
DLST	ZNF623	0.68824013	2.56E-26
DLST	PRPF18	0.68815495	2.61E-26
DLST	DAAM1	0.68804595	2.68E-26
DLST	UBE2V1	0.6878624	2.8E-26
DLST	UBE2W	0.68758808	2.98E-26
DLST	RFFL	0.68757639	2.99E-26
DLST	PMS2	0.68749544	3.04E-26
DLST	C9orf64	0.68746782	3.06E-26
DLST	ADAM9	0.68744358	3.08E-26
DLST	GLUD1	0.68743484	3.09E-26
DLST	USP4	0.68733944	3.15E-26
DLST	SFXN1	0.68728851	3.19E-26
DLST	CDC27	0.68724517	3.22E-26
DLST	TRERF1	0.68706496	3.36E-26
DLST	VPS4B	0.68693417	3.46E-26
DLST	UBLCP1	0.68691767	3.48E-26
DLST	MFAP1	0.68681917	3.56E-26
DLST	AAR2	0.68678647	3.58E-26
DLST	TOR1AIP1	0.68677247	3.6E-26
DLST	VPS41	0.68676922	3.6E-26
DLST	KPNA3	0.68668691	3.67E-26
DLST	UBE2I	0.68648829	3.84E-26
DLST	ZBTB7A	0.6864512	3.87E-26
DLST	ATP13A3	0.68622881	4.07E-26
DLST	NDE1	0.68619599	4.1E-26
DLST	BTN2A1	0.6860808	4.21E-26
DLST	KLF6	0.68597606	4.32E-26
DLST	DLG1	0.68597011	4.32E-26
DLST	MAP4	0.68586509	4.43E-26
DLST	NFKB1	0.68560346	4.7E-26
DLST	USP14	0.68552152	4.79E-26

DLST	RNF8	0.68551547	4.8E-26
DLST	GMEB1	0.68539997	4.93E-26
DLST	ZNF217	0.68517404	5.19E-26
DLST	ARPC5	0.68505959	5.33E-26
DLST	SINHCAF	0.68498756	5.41E-26
DLST	YWHAZ	0.68474168	5.73E-26
DLST	SLC38A7	0.68465015	5.85E-26
DLST	TOB2	0.68451054	6.04E-26
DLST	ATG3	0.68441864	6.16E-26
DLST	ATF6B	0.68435196	6.26E-26
DLST	DNASE1L1	0.68407	6.67E-26
DLST	IPO7	0.684014	6.76E-26
DLST	DIP2B	0.68399822	6.78E-26
DLST	FCHO2	0.6839942	6.79E-26
DLST	OPA1	0.68390286	6.93E-26
DLST	MTFMT	0.68388497	6.96E-26
DLST	EXOC5	0.68380087	7.1E-26
DLST	LSM14B	0.68380072	7.1E-26
DLST	SRSF7	0.68380006	7.1E-26
DLST	SNX7	0.68372027	7.23E-26
DLST	CFAP97	0.68370211	7.26E-26
DLST	MPHOSPH10	0.68366306	7.32E-26
DLST	OLA1	0.68355319	7.51E-26
DLST	POFUT1	0.68348033	7.63E-26
DLST	CERS2	0.68337076	7.82E-26
DLST	PGAM1	0.68331162	7.93E-26
DLST	ATP6V1A	0.68320554	8.12E-26
DLST	IL13RA1	0.68320352	8.13E-26
DLST	C5orf24	0.68303792	8.44E-26
DLST	MOB4	0.68301064	8.49E-26
DLST	RAE1	0.6829484	8.61E-26
DLST	PNMA1	0.68293779	8.63E-26
DLST	TPR	0.68288253	8.74E-26
DLST	RNF14	0.68285416	8.8E-26
DLST	SUZ12	0.68281305	8.88E-26
DLST	ATXN7L3	0.68262767	9.26E-26
DLST	FBXL12	0.68262391	9.27E-26
DLST	DIDO1	0.68253586	9.46E-26
DLST	IK	0.68250872	9.51E-26
DLST	BCL2L13	0.68250734	9.52E-26
DLST	SNTB2	0.682435	9.67E-26
DLST	MSH2	0.68230154	9.97E-26
DLST	ATL2	0.68226124	1.01E-25
DLST	PEX19	0.68221528	1.02E-25
DLST	CIR1	0.68220904	1.02E-25
DLST	UEVLD	0.68216261	1.03E-25
DLST	AP1AR	0.68216015	1.03E-25
DLST	ASB7	0.68201615	1.06E-25
DLST	MTM1	0.68194689	1.08E-25
DLST	TEAD1	0.68177724	1.12E-25
DLST	LEPROT	0.68173684	1.13E-25
DLST	USP1	0.68172284	1.14E-25
DLST	KIF13B	0.6816967	1.14E-25
DLST	NPAT	0.68164	1.16E-25
DLST	MAP1LC3B	0.68151812	1.19E-25
DLST	DPY19L1	0.68124714	1.26E-25
DLST	EPS8	0.68123166	1.27E-25
DLST	XRCC5	0.68116876	1.29E-25

DLST	HNRNPAB	0.6810302	1.33E-25
DLST	MRPL19	0.68102941	1.33E-25
DLST	GPBP1	0.68094342	1.35E-25
DLST	TMEM165	0.68088695	1.37E-25
DLST	MED20	0.68058641	1.47E-25
DLST	STX12	0.68057155	1.47E-25
DLST	STK24	0.68045	1.51E-25
DLST	EVI5	0.68032565	1.56E-25
DLST	ZFAND5	0.68021089	1.6E-25
DLST	OSTF1	0.68019799	1.6E-25
DLST	KIF5B	0.68018871	1.6E-25
DLST	ADH5	0.68015638	1.62E-25
DLST	CBL	0.68011897	1.63E-25
DLST	PNPO	0.68010914	1.63E-25
DLST	ZDHHC3	0.68006825	1.65E-25
DLST	SLC25A43	0.67997935	1.68E-25
DLST	OXR1	0.679854	1.73E-25
DLST	TNRC18	0.67984509	1.73E-25
DLST	ZNF134	0.67965394	1.81E-25
DLST	MRPL35	0.679646	1.81E-25
DLST	WSB2	0.67963812	1.81E-25
DLST	TSPAN14	0.67961769	1.82E-25
DLST	TAF7	0.67952608	1.86E-25
DLST	CNOT11	0.67939079	1.92E-25
DLST	NT5C2	0.67934701	1.94E-25
DLST	JDP2	0.67931121	1.95E-25
DLST	TAF6	0.67926203	1.97E-25
DLST	MFSD11	0.67919021	2.01E-25
DLST	PLS3	0.67907308	2.06E-25
DLST	CDC5L	0.67896942	2.11E-25
DLST	NEDD1	0.67867828	2.25E-25
DLST	DDX21	0.67864614	2.26E-25
DLST	MINDY2	0.67835661	2.42E-25
DLST	EHD4	0.67826818	2.46E-25
DLST	SLK	0.67817979	2.51E-25
DLST	RHEB	0.67817222	2.52E-25
DLST	DLG5	0.67809839	2.56E-25
DLST	VDAC1	0.67808945	2.56E-25
DLST	EFNB2	0.67801635	2.61E-25
DLST	PNO1	0.67789258	2.68E-25
DLST	NIF3L1	0.6778385	2.71E-25
DLST	ISG20L2	0.67775008	2.76E-25
DLST	ROCK2	0.67770006	2.8E-25
DLST	ARL6IP1	0.67759573	2.86E-25
DLST	TMTC2	0.67735151	3.02E-25
DLST	COPB1	0.67725151	3.09E-25
DLST	DNAJB6	0.6772252	3.11E-25
DLST	PDLIM5	0.67714456	3.16E-25
DLST	DYRK1A	0.67702402	3.25E-25
DLST	KBTBD4	0.6769202	3.32E-25
DLST	GSKIP	0.67676327	3.44E-25
DLST	DEK	0.67673848	3.46E-25
DLST	BMI1	0.67671904	3.47E-25
DLST	MBD5	0.67654455	3.61E-25
DLST	MRPS27	0.67648013	3.66E-25
DLST	UBA1	0.67647608	3.67E-25
DLST	SMARCAL1	0.67641876	3.71E-25
DLST	GTF2B	0.67636391	3.76E-25

DLST	SPIDR	0.67633392	3.78E-25
DLST	PTPN4	0.67632047	3.79E-25
DLST	PTDSS1	0.67628336	3.83E-25
DLST	FXR1	0.67627129	3.84E-25
DLST	RASAL2	0.67591611	4.15E-25
DLST	EXT1	0.67584656	4.21E-25
DLST	CBX3	0.67574874	4.3E-25
DLST	ANKRD50	0.67573118	4.32E-25
DLST	CUL1	0.67559844	4.45E-25
DLST	RFWD3	0.67539402	4.65E-25
DLST	C4orf3	0.67525336	4.8E-25
DLST	USP12	0.67523505	4.82E-25
DLST	AP3S1	0.67522857	4.83E-25
DLST	EPS15	0.6752218	4.83E-25
DLST	TPP1	0.67517012	4.89E-25
DLST	REEP5	0.67506185	5.01E-25
DLST	NKRF	0.67504199	5.03E-25
DLST	RYBP	0.67503874	5.03E-25
DLST	PTBP1	0.67488357	5.21E-25
DLST	MBNL2	0.67482247	5.28E-25
DLST	ZFR	0.67475391	5.36E-25
DLST	SAMD8	0.67457526	5.57E-25
DLST	ISY1-RAB43	0.67450856	5.65E-25
DLST	CFAP20	0.67425875	5.97E-25
DLST	STAT3	0.67416816	6.09E-25
DLST	MMGT1	0.67392213	6.43E-25
DLST	ANKRD13C	0.67373076	6.7E-25
DLST	SKI	0.67371651	6.72E-25
DLST	ATL3	0.67366545	6.8E-25
DLST	ATG5	0.67365004	6.82E-25
DLST	IMMT	0.67350236	7.05E-25
DLST	POT1	0.67347984	7.08E-25
DLST	PARP4	0.6734013	7.2E-25
DLST	PRKAG2	0.67338608	7.23E-25
DLST	PRRC1	0.67338347	7.23E-25
DLST	RAB2A	0.67335104	7.28E-25
DLST	GPR107	0.67334494	7.29E-25
DLST	KAT7	0.67330556	7.35E-25
DLST	SHOC2	0.67314552	7.62E-25
DLST	RAB3GAP2	0.67307142	7.74E-25
DLST	ZNF263	0.67303597	7.8E-25
DLST	UXS1	0.67288474	8.06E-25
DLST	CPOX	0.67281207	8.19E-25
DLST	ZNF827	0.67271127	8.37E-25
DLST	UQCRC2	0.67267885	8.43E-25
DLST	KDM1A	0.67255782	8.66E-25
DLST	ARPC2	0.6725242	8.72E-25
DLST	CNPPD1	0.67248906	8.79E-25
DLST	EXOC4	0.67244542	8.87E-25
DLST	PANK3	0.67241819	8.92E-25
DLST	PPP5C	0.67233558	9.08E-25
DLST	ACOT2	0.67220753	9.34E-25
DLST	CSTF2	0.6721524	9.45E-25
DLST	SNAP23	0.67210988	9.54E-25
DLST	CHMP1B	0.6720668	9.63E-25
DLST	GSPT1	0.67199012	9.79E-25
DLST	ADAT1	0.67178695	1.02E-24
DLST	GALK2	0.67167966	1.05E-24

DLST	LMBR1	0.67162456	1.06E-24
DLST	TRAK1	0.67158877	1.07E-24
DLST	OSBPL10	0.67153606	1.08E-24
DLST	SMAD2	0.67152044	1.08E-24
DLST	TRAF3IP1	0.67142949	1.11E-24
DLST	LDAH	0.67134559	1.13E-24
DLST	DRAM2	0.67133272	1.13E-24
DLST	PPP2R5C	0.67101077	1.21E-24
DLST	MEAK7	0.67100921	1.21E-24
DLST	CLNS1A	0.67097976	1.22E-24
DLST	ARF4	0.6709794	1.22E-24
DLST	BBX	0.67079656	1.27E-24
DLST	SPATS2	0.67079396	1.27E-24
DLST	TPM4	0.67078815	1.27E-24
DLST	PTGES3	0.67054219	1.34E-24
DLST	PPP4R1	0.67050474	1.35E-24
DLST	PPTC7	0.67032906	1.4E-24
DLST	DNAJC21	0.67031758	1.41E-24
DLST	PSMD14	0.67027661	1.42E-24
DLST	BRD3	0.66995248	1.52E-24
DLST	HBP1	0.66993539	1.53E-24
DLST	PPM1B	0.66987944	1.55E-24
DLST	SRPK2	0.66977199	1.58E-24
DLST	ASXL2	0.66976792	1.58E-24
DLST	TMEM33	0.66944318	1.7E-24
DLST	CORO1C	0.66927676	1.76E-24
DLST	HSPA14	0.66925938	1.77E-24
DLST	FAM118B	0.66922173	1.78E-24
DLST	LUZP1	0.66921015	1.78E-24
DLST	TMEM185B	0.66919311	1.79E-24
DLST	TP53BP2	0.66901133	1.86E-24
DLST	STK4	0.66898599	1.87E-24
DLST	LRCH3	0.66896015	1.88E-24
DLST	SMG7	0.66888501	1.91E-24
DLST	SOS2	0.66887426	1.92E-24
DLST	UBFD1	0.66881359	1.94E-24
DLST	GOLGA5	0.66877868	1.96E-24
DLST	USB1	0.66872119	1.98E-24
DLST	SAR1B	0.66861279	2.03E-24
DLST	RNF139	0.66858067	2.04E-24
DLST	VAPB	0.66856567	2.05E-24
DLST	UTP3	0.66855647	2.05E-24
DLST	KIF2A	0.66846802	2.09E-24
DLST	TRIM56	0.66833365	2.15E-24
DLST	ITPRIPL2	0.66816794	2.23E-24
DLST	KDM5B	0.66813918	2.25E-24
DLST	ZNF639	0.66806362	2.28E-24
DLST	USP6NL	0.66792469	2.35E-24
DLST	LRRC42	0.66786003	2.38E-24
DLST	ATP6V1C1	0.66776582	2.43E-24
DLST	DDX50	0.66759125	2.52E-24
DLST	SYNCRIP	0.66757029	2.54E-24
DLST	NRBP1	0.66754115	2.55E-24
DLST	LYPLA1	0.66752209	2.56E-24
DLST	ZMYM2	0.66750988	2.57E-24
DLST	STK3	0.66743939	2.61E-24
DLST	DDX3X	0.66742489	2.62E-24
DLST	PIAS3	0.66732446	2.67E-24

DLST	MRPL3	0.66727261	2.7E-24
DLST	CTDSPL2	0.66725189	2.71E-24
DLST	IFNAR1	0.66720343	2.74E-24
DLST	STT3B	0.66720249	2.74E-24
DLST	GNL3L	0.66705148	2.83E-24
DLST	DCUN1D1	0.66701725	2.85E-24
DLST	INTS14	0.66697058	2.88E-24
DLST	RAN	0.66685504	2.95E-24
DLST	GRWD1	0.66675662	3.02E-24
DLST	ATG9A	0.6667555	3.02E-24
DLST	FGFR1OP2	0.66670199	3.05E-24
DLST	UBXN2B	0.66662684	3.1E-24
DLST	FOXN2	0.66656451	3.14E-24
DLST	GPN3	0.66644017	3.23E-24
DLST	MED4	0.66641407	3.24E-24
DLST	MTDH	0.66627705	3.34E-24
DLST	ACTL6A	0.6662664	3.35E-24
DLST	TMEM128	0.6660545	3.5E-24
DLST	SLBP	0.66605317	3.5E-24
DLST	DPP9	0.66604999	3.5E-24
DLST	YES1	0.66604106	3.51E-24
DLST	MBOAT2	0.66580673	3.69E-24
DLST	BUB3	0.66577287	3.72E-24
DLST	NBR1	0.6656766	3.79E-24
DLST	FBXL5	0.66555672	3.89E-24
DLST	RB1	0.6654968	3.94E-24
DLST	RNF34	0.66537086	4.05E-24
DLST	SUCLA2	0.66509563	4.29E-24
DLST	ZC2HC1A	0.66505864	4.32E-24
DLST	GYS1	0.66494526	4.43E-24
DLST	WWP1	0.66485256	4.51E-24
DLST	ABL1	0.66479892	4.57E-24
DLST	DDX6	0.66470767	4.66E-24
DLST	NSFL1C	0.6645883	4.77E-24
DLST	ANKLE2	0.66448241	4.88E-24
DLST	LCLAT1	0.6643866	4.98E-24
DLST	RMND5A	0.66429159	5.08E-24
DLST	FAM32A	0.66425872	5.12E-24
DLST	SLC35A5	0.66424161	5.14E-24
DLST	ARL2BP	0.66421587	5.16E-24
DLST	GPALPP1	0.66406823	5.33E-24
DLST	RBMS1	0.66392393	5.49E-24
DLST	ATF1	0.6639053	5.51E-24
DLST	TRIM14	0.66381346	5.62E-24
DLST	WASF2	0.66380297	5.63E-24
DLST	NDFIP2	0.66379151	5.65E-24
DLST	HSP90AA1	0.66368187	5.78E-24
DLST	DST	0.66352134	5.98E-24
DLST	ARHGEF18	0.66337353	6.16E-24
DLST	FUT8	0.66337096	6.17E-24
DLST	KCTD10	0.66305982	6.58E-24
DLST	CWF19L2	0.66305693	6.59E-24
DLST	TOP2B	0.66294447	6.75E-24
DLST	YIPF6	0.66282642	6.91E-24
DLST	NCK1	0.66268439	7.12E-24
DLST	CDK7	0.66260476	7.24E-24
DLST	PLEKHA7	0.66251928	7.37E-24
DLST	LRRC41	0.662455	7.47E-24

DLST	ERH	0.66245222	7.48E-24
DLST	TAF5L	0.66238186	7.59E-24
DLST	ZNF260	0.66228886	7.74E-24
DLST	STAU2	0.66221138	7.86E-24
DLST	CHMP3	0.66218073	7.91E-24
DLST	DEDD	0.66209502	8.06E-24
DLST	PGAM5	0.66181242	8.55E-24
DLST	LMNB1	0.66177363	8.62E-24
DLST	ETFA	0.66173543	8.69E-24
DLST	PRPF38A	0.66169525	8.76E-24
DLST	VRK2	0.6615908	8.95E-24
DLST	GNPDA1	0.66157999	8.97E-24
DLST	ABHD2	0.66149832	9.13E-24
DLST	HIF1A	0.66139208	9.33E-24
DLST	RANBP9	0.66124156	9.63E-24
DLST	MYH9	0.66123671	9.64E-24
DLST	ACBD3	0.66122641	9.66E-24
DLST	HELZ2	0.66119387	9.72E-24
DLST	SUMO1	0.66108844	9.94E-24
DLST	IARS2	0.66108129	9.95E-24
DLST	YTHDF2	0.66104796	1E-23
DLST	USP9X	0.66102161	1.01E-23
DLST	SCARB2	0.66099361	1.01E-23
DLST	NIP7	0.66094358	1.02E-23
DLST	RDH11	0.66085445	1.04E-23
DLST	TMEM135	0.6607454	1.07E-23
DLST	WDR47	0.66069523	1.08E-23
DLST	ANP32E	0.66065159	1.09E-23
DLST	GOT2	0.66059992	1.1E-23
DLST	AP1S3	0.66056669	1.11E-23
DLST	TAB2	0.6602977	1.17E-23
DLST	SGMS2	0.66023508	1.19E-23
DLST	UTP14C	0.66007183	1.23E-23
DLST	NDC1	0.66005985	1.23E-23
DLST	MAP3K7	0.66005469	1.23E-23
DLST	SLC35A3	0.65995523	1.26E-23
DLST	BRAP	0.65994721	1.26E-23
DLST	TMBIM6	0.65978717	1.3E-23
DLST	MESD	0.65976857	1.31E-23
DLST	FNBP1L	0.6596623	1.34E-23
DLST	DERL1	0.6596535	1.34E-23
DLST	TRRAP	0.65963341	1.34E-23
DLST	AGGF1	0.65933554	1.43E-23
DLST	PARP8	0.6593281	1.43E-23
DLST	C8orf33	0.65931773	1.44E-23
DLST	NUDCD2	0.65924913	1.46E-23
DLST	DCAF12	0.65921004	1.47E-23
DLST	SLC6A6	0.65917381	1.48E-23
DLST	ZNF644	0.65912904	1.49E-23
DLST	KDSR	0.6590306	1.52E-23
DLST	DYNC1H1	0.6589236	1.56E-23
DLST	PACS1	0.65883814	1.58E-23
DLST	LIMA1	0.65883253	1.59E-23
DLST	CCDC32	0.65868907	1.63E-23
DLST	DCTN6	0.65862827	1.65E-23
DLST	TPD52L2	0.65843582	1.72E-23
DLST	BABAM2	0.65842855	1.72E-23
DLST	PLEKHA3	0.65833779	1.76E-23

DLST	POLE3	0.65829824	1.77E-23
DLST	CCDC97	0.65814483	1.83E-23
DLST	ZSWIM1	0.65811653	1.84E-23
DLST	TBC1D25	0.65806257	1.86E-23
DLST	CHM	0.65800921	1.88E-23
DLST	THUMPD3	0.65792543	1.91E-23
DLST	NDEL1	0.65753057	2.07E-23
DLST	PITPNM1	0.65748419	2.09E-23
DLST	USP39	0.65736239	2.15E-23
DLST	MORF4L1	0.65710596	2.26E-23
DLST	NFYA	0.65676474	2.43E-23
DLST	TMEM123	0.65672118	2.45E-23
DLST	PJA2	0.6567101	2.45E-23
DLST	MED21	0.65658809	2.52E-23
DLST	CDC42BPA	0.65651845	2.55E-23
DLST	CACUL1	0.65647821	2.57E-23
DLST	FBXO8	0.65643493	2.6E-23
DLST	MICU1	0.65637956	2.63E-23
DLST	TRIOBP	0.65622127	2.71E-23
DLST	CASP3	0.65586193	2.92E-23
DLST	PGK1	0.65571616	3.01E-23
DLST	C12orf43	0.65561421	3.07E-23
DLST	NUP37	0.65549063	3.15E-23
DLST	NUDT21	0.65539922	3.21E-23
DLST	NCSTN	0.655159	3.37E-23
DLST	CLDND1	0.65506408	3.43E-23
DLST	RNF13	0.65504994	3.44E-23
DLST	LACTB	0.65497952	3.49E-23
DLST	TGS1	0.65478712	3.63E-23
DLST	NUB1	0.65475297	3.66E-23
DLST	CAPN2	0.65470582	3.69E-23
DLST	SEC22A	0.65467482	3.72E-23
DLST	GALNT10	0.65466926	3.72E-23
DLST	NFE2L1	0.65462191	3.76E-23
DLST	BICD1	0.65452599	3.83E-23
DLST	MRPL42	0.65449773	3.85E-23
DLST	FAM222B	0.65446795	3.88E-23
DLST	GPN1	0.65438692	3.94E-23
DLST	CNOT2	0.65436045	3.96E-23
DLST	DNAJB14	0.65397546	4.28E-23
DLST	VDAC2	0.65393189	4.32E-23
DLST	RABL3	0.65370671	4.52E-23
DLST	DYNLT3	0.65362806	4.6E-23
DLST	MGLL	0.65358966	4.63E-23
DLST	MKRN1	0.65356592	4.65E-23
DLST	KIF16B	0.65348472	4.73E-23
DLST	PRDX3	0.6534775	4.74E-23
DLST	TERF2	0.65347044	4.74E-23
DLST	ACTR6	0.65336062	4.85E-23
DLST	TCOF1	0.65332547	4.89E-23
DLST	VCPIP1	0.65322255	4.99E-23
DLST	BNIP3L	0.65322227	4.99E-23
DLST	WEE1	0.65313998	5.07E-23
DLST	SETD7	0.65313398	5.08E-23
DLST	GNAI2	0.65311154	5.1E-23
DLST	CTNNB1	0.65306199	5.15E-23
DLST	HAT1	0.65298416	5.23E-23
DLST	RNF220	0.65290041	5.32E-23

DLST	POGK	0.65289526	5.33E-23
DLST	PDE6D	0.65279674	5.44E-23
DLST	PPP2CB	0.65265609	5.59E-23
DLST	TXNDC9	0.65261858	5.63E-23
DLST	FAM204A	0.652437	5.84E-23
DLST	WBP11	0.65242701	5.86E-23
DLST	TSPAN17	0.65230876	6E-23
DLST	AP2M1	0.65230128	6.01E-23
DLST	DLGAP4	0.65219569	6.14E-23
DLST	CTTN	0.65213994	6.2E-23
DLST	LRBA	0.6520621	6.3E-23
DLST	SLC44A1	0.65190086	6.51E-23
DLST	SMN1	0.65176245	6.69E-23
DLST	ARRDC3	0.65168182	6.8E-23
DLST	DPY19L4	0.6516145	6.9E-23
DLST	PKP4	0.65160895	6.9E-23
DLST	BORCS8-MEF2B	0.65152601	7.02E-23
DLST	DNM1L	0.65150551	7.05E-23
DLST	FAM120AOS	0.65146425	7.11E-23
DLST	PACRGL	0.65146298	7.11E-23
DLST	AEBP2	0.65146236	7.11E-23
DLST	TRIO	0.65132707	7.31E-23
DLST	FAM98B	0.65106598	7.7E-23
DLST	STAG2	0.65102487	7.76E-23
DLST	SPRED1	0.65100108	7.8E-23
DLST	SPAST	0.65089928	7.96E-23
DLST	CASP8	0.6508189	8.09E-23
DLST	PHF13	0.65069345	8.29E-23
DLST	ZMIZ1	0.65063437	8.39E-23
DLST	SLC25A40	0.6506314	8.4E-23
DLST	NLN	0.65050599	8.61E-23
DLST	NT5DC1	0.65049546	8.63E-23
DLST	PRKAG1	0.65029896	8.97E-23
DLST	PSMA3	0.65018351	9.18E-23
DLST	HERC3	0.65007768	9.38E-23
DLST	METTL2B	0.65007417	9.39E-23
DLST	PTPRE	0.65006767	9.4E-23
DLST	LIMS1	0.64996255	9.6E-23
DLST	GID8	0.64986459	9.79E-23
DLST	VPS26A	0.64981052	9.89E-23
DLST	ARMCX6	0.64977473	9.96E-23
DLST	ZDHHC6	0.6497675	9.98E-23
DLST	CLOCK	0.64975813	1E-22
DLST	SDHC	0.64949704	1.05E-22
DLST	MASTL	0.64945958	1.06E-22
DLST	GTF2E1	0.64945814	1.06E-22
DLST	TMED10	0.64943364	1.07E-22
DLST	CRLF3	0.64922266	1.11E-22
DLST	C3orf38	0.64909251	1.14E-22
DLST	MRFAP1L1	0.64908467	1.14E-22
DLST	TSPAN31	0.64899492	1.16E-22
DLST	ELOVL1	0.64895103	1.17E-22
DLST	FAM98A	0.64877374	1.22E-22
DLST	NOL4L	0.64871881	1.23E-22
DLST	STX7	0.6486966	1.23E-22
DLST	RAP1B	0.64866849	1.24E-22
DLST	PPP1R3B	0.64853955	1.27E-22
DLST	UBE2V2	0.64852874	1.28E-22

DLST	ASCC3	0.64841095	1.31E-22
DLST	COPA	0.64839276	1.31E-22
DLST	OSBPL11	0.64828271	1.34E-22
DLST	ACBD5	0.64821738	1.36E-22
DLST	NBN	0.64806972	1.4E-22
DLST	RNF2	0.64793983	1.43E-22
DLST	RNF19B	0.64774461	1.49E-22
DLST	ATF7IP	0.64764102	1.52E-22
DLST	ERGIC1	0.64752684	1.56E-22
DLST	EMB	0.64751349	1.56E-22
DLST	RELA	0.64729964	1.63E-22
DLST	WDR43	0.64713946	1.68E-22
DLST	TTC4	0.64706113	1.71E-22
DLST	ERG28	0.64703055	1.72E-22
DLST	CYB5R4	0.64698075	1.73E-22
DLST	ZKSCAN5	0.64695513	1.74E-22
DLST	LZIC	0.64691627	1.76E-22
DLST	TIPRL	0.64687906	1.77E-22
DLST	SMURF2	0.64675704	1.81E-22
DLST	TMEM106B	0.64674533	1.82E-22
DLST	BLZF1	0.64659605	1.87E-22
DLST	NCOA2	0.64658697	1.87E-22
DLST	RAD18	0.64627283	1.99E-22
DLST	SERINC5	0.64624607	2E-22
DLST	STARD3NL	0.64614919	2.04E-22
DLST	IVD	0.64607696	2.07E-22
DLST	SFT2D2	0.6459346	2.13E-22
DLST	UGDH	0.64592346	2.14E-22
DLST	HIBADH	0.64580213	2.19E-22
DLST	GBE1	0.64576696	2.2E-22
DLST	FEM1B	0.64573882	2.22E-22
DLST	SAR1A	0.64572491	2.22E-22
DLST	ATXN7L3B	0.6456367	2.26E-22
DLST	ARHGAP17	0.64562481	2.27E-22
DLST	IDH3A	0.64556763	2.29E-22
DLST	SLC15A4	0.64544098	2.35E-22
DLST	AHCYL1	0.64539097	2.37E-22
DLST	SF3A3	0.64528409	2.42E-22
DLST	LSM12	0.64526613	2.43E-22
DLST	GNPTAB	0.64525778	2.43E-22
DLST	GHITM	0.64523991	2.44E-22
DLST	TUT7	0.64521621	2.45E-22
DLST	ENTPD5	0.64520652	2.46E-22
DLST	SF3A1	0.64496172	2.58E-22
DLST	ARL5A	0.64491308	2.6E-22
DLST	TGFBR2	0.64482913	2.65E-22
DLST	EFL1	0.64482613	2.65E-22
DLST	INPP5A	0.64472783	2.7E-22
DLST	JOSD1	0.64469769	2.72E-22
DLST	MICU2	0.64463493	2.75E-22
DLST	CTDSP1	0.64462213	2.76E-22
DLST	MRPS36	0.6445004	2.82E-22
DLST	SLC30A5	0.64442714	2.86E-22
DLST	TCAIM	0.64427301	2.95E-22
DLST	USP38	0.64424118	2.97E-22
DLST	TMLHE	0.64423705	2.97E-22
DLST	YWHAH	0.64423	2.98E-22
DLST	FBXL3	0.64409158	3.06E-22

DLST	UGP2	0.644078	3.07E-22
DLST	MYOF	0.64402044	3.1E-22
DLST	CDC42EP3	0.64398962	3.12E-22
DLST	ABR	0.6438865	3.18E-22
DLST	CPNE3	0.64385498	3.2E-22
DLST	CDC73	0.64382515	3.22E-22
DLST	DOCK1	0.6437865	3.25E-22
DLST	MLH1	0.64370074	3.3E-22
DLST	ITFG1	0.64355021	3.4E-22
DLST	SLC10A3	0.64352857	3.41E-22
DLST	VAMP7	0.6434624	3.46E-22
DLST	ETV3	0.64341199	3.49E-22
DLST	L3MBTL3	0.64331389	3.56E-22
DLST	LINS1	0.6432751	3.59E-22
DLST	ZDHHC7	0.64324632	3.61E-22
DLST	SOWAHC	0.64320459	3.64E-22
DLST	GTPBP4	0.64311592	3.7E-22
DLST	RO60	0.64307867	3.73E-22
DLST	DAG1	0.64299525	3.79E-22
DLST	PAK1	0.6427529	3.97E-22
DLST	ZNF184	0.64255417	4.13E-22
DLST	TMBIM4	0.64254912	4.13E-22
DLST	CASK	0.64251695	4.16E-22
DLST	FAM168A	0.64241985	4.23E-22
DLST	GOSR1	0.64240054	4.25E-22
DLST	MIB1	0.64230845	4.33E-22
DLST	DNAJC8	0.64197243	4.62E-22
DLST	ERLIN1	0.64191123	4.67E-22
DLST	CCDC59	0.64190226	4.68E-22
DLST	PDZD8	0.64171801	4.85E-22
DLST	BBS2	0.64170141	4.87E-22
DLST	HPRT1	0.64169507	4.87E-22
DLST	CYCS	0.64162172	4.94E-22
DLST	MFSD6	0.64139893	5.16E-22
DLST	ELK1	0.64136894	5.19E-22
DLST	ERI1	0.64111839	5.45E-22
DLST	CD2AP	0.64101545	5.56E-22
DLST	RAP2B	0.64093707	5.64E-22
DLST	ENOPH1	0.64089485	5.69E-22
DLST	CRTC3	0.64082426	5.77E-22
DLST	MIGA1	0.64075914	5.84E-22
DLST	ARL1	0.64065237	5.96E-22
DLST	TPM3	0.64049819	6.14E-22
DLST	PPP1R15B	0.6402609	6.43E-22
DLST	ZDHHC9	0.64006	6.68E-22
DLST	CFL1	0.64003837	6.71E-22
DLST	WAPL	0.64000815	6.75E-22
DLST	LIN52	0.63994223	6.83E-22
DLST	YY1AP1	0.63984897	6.96E-22
DLST	DNM2	0.63984515	6.96E-22
DLST	MRFAP1	0.63960207	7.3E-22
DLST	RPAP3	0.63937206	7.62E-22
DLST	WASHC5	0.63918903	7.9E-22
DLST	PRKACA	0.63916176	7.94E-22
DLST	CPT1A	0.63915567	7.95E-22
DLST	HOMEZ	0.63909256	8.04E-22
DLST	WDR89	0.63903688	8.13E-22
DLST	ZNF267	0.63886426	8.4E-22

DLST	RACGAP1	0.63884843	8.43E-22
DLST	RNF6	0.63882122	8.47E-22
DLST	IDH1	0.63859646	8.85E-22
DLST	SMC2	0.63849805	9.01E-22
DLST	DESI2	0.63828351	9.39E-22
DLST	PDHB	0.63796463	9.98E-22
DLST	MED1	0.6377509	1.04E-21
DLST	HS2ST1	0.63762473	1.06E-21
DLST	ZNF45	0.63744997	1.1E-21
DLST	CRKL	0.63743892	1.1E-21
DLST	PSMD2	0.63736661	1.12E-21
DLST	LEO1	0.6372745	1.14E-21
DLST	TUBA1C	0.63720314	1.15E-21
DLST	TTC9C	0.63706305	1.19E-21
DLST	CBX1	0.63701733	1.2E-21
DLST	TCF12	0.63676728	1.25E-21
DLST	FKBP9	0.63669959	1.27E-21
DLST	SMARCE1	0.63667896	1.27E-21
DLST	SDE2	0.63665264	1.28E-21
DLST	ARMC10	0.63654488	1.31E-21
DLST	ZDHHC13	0.63645905	1.33E-21
DLST	CTR9	0.63642241	1.34E-21
DLST	TFG	0.63632834	1.36E-21
DLST	ZBTB38	0.63626287	1.38E-21
DLST	ZNF526	0.63621434	1.39E-21
DLST	TRIM32	0.63619605	1.4E-21
DLST	GPATCH11	0.63616882	1.4E-21
DLST	LDB1	0.6361074	1.42E-21
DLST	MAPK1	0.63606936	1.43E-21
DLST	URM1	0.63606831	1.43E-21
DLST	RAD1	0.63602563	1.44E-21
DLST	UBXN2A	0.63600602	1.45E-21
DLST	METTL6	0.63600239	1.45E-21
DLST	ARMT1	0.63593819	1.47E-21
DLST	ANO1	0.63578727	1.51E-21
DLST	SETX	0.6355485	1.58E-21
DLST	ATP5PB	0.6355408	1.58E-21
DLST	CHORDC1	0.63553866	1.58E-21
DLST	SMAD1	0.63545702	1.61E-21
DLST	CAMK2G	0.6354514	1.61E-21
DLST	ZNRF2	0.63542164	1.62E-21
DLST	HPS5	0.63537595	1.63E-21
DLST	FBXO22	0.63536527	1.64E-21
DLST	GRPEL1	0.63535058	1.64E-21
DLST	BCL10	0.6352961	1.66E-21
DLST	GSK3A	0.63528995	1.66E-21
DLST	ACSL4	0.63498697	1.76E-21
DLST	CDC42SE2	0.63492275	1.78E-21
DLST	INSIG2	0.63489477	1.79E-21
DLST	ZNF629	0.63476778	1.83E-21
DLST	FBXO7	0.63470367	1.85E-21
DLST	ITGA2	0.63461802	1.88E-21
DLST	RALBP1	0.63455194	1.91E-21
DLST	TFAM	0.63451812	1.92E-21
DLST	TDRD7	0.63449772	1.93E-21
DLST	MAST2	0.63441407	1.96E-21
DLST	RIT1	0.63434012	1.98E-21
DLST	MYO1D	0.63422688	2.03E-21

DLST	UBE2K	0.63417451	2.05E-21
DLST	UBE2E1	0.63407315	2.09E-21
DLST	EPC2	0.63407082	2.09E-21
DLST	CMPK1	0.63374674	2.22E-21
DLST	TAF12	0.63364399	2.26E-21
DLST	ZNF766	0.63362438	2.27E-21
DLST	ANO6	0.63355295	2.3E-21
DLST	KNOP1	0.63347108	2.34E-21
DLST	B3GNT5	0.63338794	2.37E-21
DLST	HNRNPM	0.63320831	2.45E-21
DLST	ABCF2	0.63319902	2.46E-21
DLST	SDCBP	0.63306287	2.52E-21
DLST	CRIM1	0.6330432	2.53E-21
DLST	PDCL	0.63301651	2.54E-21
DLST	NOLC1	0.63298947	2.56E-21
DLST	HIVEP1	0.63296526	2.57E-21
DLST	PEF1	0.63296031	2.57E-21
DLST	FAM3C	0.63291707	2.59E-21
DLST	DDX60L	0.63288427	2.61E-21
DLST	MBIP	0.63286436	2.62E-21
DLST	ERAP1	0.63262647	2.74E-21
DLST	RECQL	0.63259618	2.75E-21
DLST	VEZF1	0.63258856	2.76E-21
DLST	MGME1	0.6325656	2.77E-21
DLST	ZBTB2	0.63254917	2.78E-21
DLST	CPNE8	0.63222484	2.95E-21
DLST	FADD	0.63214947	2.99E-21
DLST	EIF2AK1	0.63212735	3.01E-21
DLST	CYB5R3	0.63211463	3.01E-21
DLST	CCDC6	0.63197236	3.09E-21
DLST	DCP2	0.63189829	3.14E-21
DLST	TAF1A	0.63184657	3.17E-21
DLST	RTN3	0.63182669	3.18E-21
DLST	APEX2	0.63177241	3.21E-21
DLST	AGAP1	0.63163201	3.3E-21
DLST	CEP250	0.63135686	3.47E-21
DLST	RBM43	0.63113414	3.62E-21
DLST	BBS9	0.63109575	3.64E-21
DLST	BACH1	0.63103103	3.69E-21
DLST	SPG21	0.63101603	3.7E-21
DLST	TNIK	0.63087536	3.8E-21
DLST	KIAA0586	0.63082879	3.83E-21
DLST	GDI2	0.63076912	3.87E-21
DLST	DERA	0.63075177	3.88E-21
DLST	PSMD12	0.63068143	3.93E-21
DLST	GLTP	0.63057371	4.01E-21
DLST	ATXN1	0.63056291	4.02E-21
DLST	CDV3	0.63050523	4.07E-21
DLST	DFFA	0.6304706	4.09E-21
DLST	MPZL1	0.63047044	4.09E-21
DLST	SPATS2L	0.63039965	4.15E-21
DLST	GLOD4	0.63031256	4.21E-21
DLST	DIAPH2	0.63020643	4.3E-21
DLST	CALCOCO2	0.63011655	4.37E-21
DLST	VGLL4	0.63005964	4.42E-21
DLST	BCAR3	0.63002912	4.44E-21
DLST	NACC1	0.62999609	4.47E-21
DLST	SH3BGRL	0.62987897	4.57E-21

DLST	FAM114A1	0.629829	4.61E-21
DLST	ASAP1	0.62982512	4.61E-21
DLST	PANX1	0.62976147	4.67E-21
DLST	GSK3B	0.62971006	4.71E-21
DLST	PHC2	0.62966044	4.76E-21
DLST	COPS5	0.62933762	5.05E-21
DLST	GRK2	0.6293216	5.06E-21
DLST	RNF149	0.62926085	5.12E-21
DLST	COX15	0.62925741	5.12E-21
DLST	DNAJC10	0.62924398	5.14E-21
DLST	ALG11	0.62920748	5.17E-21
DLST	APP	0.629196	5.18E-21
DLST	ANXA5	0.62917339	5.2E-21
DLST	ZNF200	0.62913575	5.24E-21
DLST	WASHC2A	0.62897943	5.39E-21
DLST	TRUB1	0.62892485	5.45E-21
DLST	AVL9	0.62891541	5.46E-21
DLST	PCNX1	0.62876648	5.61E-21
DLST	PCSK7	0.62865564	5.73E-21
DLST	TMX2	0.62862374	5.76E-21
DLST	GNA13	0.62853237	5.86E-21
DLST	PRIM2	0.62853175	5.86E-21
DLST	SLC25A32	0.62851732	5.87E-21
DLST	RTRAF	0.62851366	5.88E-21
DLST	EIF1B	0.6284495	5.95E-21
DLST	C1GALT1C1	0.62827258	6.14E-21
DLST	SRGAP2B	0.62817558	6.26E-21
DLST	TEFM	0.6281528	6.28E-21
DLST	SCAF11	0.62812676	6.31E-21
DLST	FTO	0.62812672	6.31E-21
DLST	MRPS10	0.62808277	6.36E-21
DLST	KCTD21	0.62807439	6.37E-21
DLST	PTTG1IP	0.62783578	6.66E-21
DLST	ESD	0.62777114	6.74E-21
DLST	BCAS2	0.62767882	6.85E-21
DLST	TMED5	0.62746481	7.13E-21
DLST	GRPEL2	0.62731103	7.33E-21
DLST	TNFRSF21	0.62727915	7.38E-21
DLST	METTL14	0.62715258	7.55E-21
DLST	AIDA	0.6270033	7.76E-21
DLST	NUP50	0.62697883	7.79E-21
DLST	RAD23A	0.62678111	8.08E-21
DLST	EIF2S2	0.6267723	8.1E-21
DLST	ANKS1A	0.6262721	8.87E-21
DLST	NUDT15	0.62609413	9.16E-21
DLST	KRCC1	0.6260304	9.27E-21
DLST	STARD7	0.62601689	9.3E-21
DLST	TRAF7	0.62599309	9.34E-21
DLST	CDKN2AIP	0.6259887	9.34E-21
DLST	ODF2	0.62588718	9.52E-21
DLST	ZYG11B	0.62585822	9.57E-21
DLST	ZC3H18	0.62585294	9.58E-21
DLST	INIP	0.62578246	9.7E-21
DLST	TRIM26	0.62576405	9.73E-21
DLST	FYCO1	0.62568321	9.88E-21
DLST	ZMYND8	0.62555278	1.01E-20
DLST	HSD17B12	0.62551176	1.02E-20
DLST	TMEM267	0.62538543	1.04E-20

DLST	SNX24	0.62532535	1.05E-20
DLST	FOXP1	0.62529505	1.06E-20
DLST	RAB12	0.62525842	1.07E-20
DLST	COA7	0.62511546	1.1E-20
DLST	PPME1	0.62496655	1.13E-20
DLST	URI1	0.62490747	1.14E-20
DLST	MEAF6	0.62476736	1.17E-20
DLST	LPP	0.6247261	1.18E-20
DLST	NAP1L4	0.62463257	1.2E-20
DLST	GAB2	0.62450012	1.23E-20
DLST	CCNDBP1	0.62437961	1.25E-20
DLST	ZMYM4	0.62435183	1.26E-20
DLST	HTATIP2	0.62429547	1.27E-20
DLST	ZNF462	0.62427882	1.28E-20
DLST	DDA1	0.62423774	1.29E-20
DLST	RCC1L	0.62423639	1.29E-20
DLST	FBXO45	0.62422448	1.29E-20
DLST	GLO1	0.62421776	1.29E-20
DLST	C9orf78	0.62420857	1.29E-20
DLST	SH3D19	0.6241594	1.3E-20
DLST	SLC24A1	0.6241003	1.32E-20
DLST	RARA	0.62405576	1.33E-20
DLST	ERO1A	0.62403231	1.33E-20
DLST	TTC5	0.62393162	1.36E-20
DLST	UBE2Q2	0.62389649	1.37E-20
DLST	ATE1	0.62385008	1.38E-20
DLST	APAF1	0.6238495	1.38E-20
DLST	DCK	0.62383161	1.38E-20
DLST	UHMK1	0.62378562	1.4E-20
DLST	NCAPG2	0.62364327	1.43E-20
DLST	SGCB	0.62360924	1.44E-20
DLST	PRRG1	0.62341481	1.49E-20
DLST	RSPH3	0.62323754	1.54E-20
DLST	FGD6	0.62307062	1.59E-20
DLST	RIC8A	0.62306877	1.59E-20
DLST	SRP9	0.62277305	1.68E-20
DLST	ZNF707	0.62275446	1.68E-20
DLST	CCAR2	0.62260902	1.73E-20
DLST	BCAP29	0.62244914	1.78E-20
DLST	STK38L	0.62241566	1.79E-20
DLST	EIF1AX	0.62224587	1.84E-20
DLST	ANP32B	0.62215299	1.87E-20
DLST	SNRPB2	0.62196334	1.94E-20
DLST	SCAMP2	0.62192356	1.95E-20
DLST	ZCRB1	0.62186512	1.97E-20
DLST	PELO	0.62181372	1.99E-20
DLST	RNF141	0.62172361	2.02E-20
DLST	WDR3	0.62168085	2.04E-20
DLST	DCLRE1B	0.62163111	2.06E-20
DLST	RRAGC	0.62158932	2.07E-20
DLST	AKAP11	0.62147501	2.12E-20
DLST	BPNT1	0.62142591	2.14E-20
DLST	FKBP3	0.62128013	2.19E-20
DLST	ABCF1	0.62117953	2.23E-20
DLST	SLU7	0.62111718	2.26E-20
DLST	PRTFDC1	0.62111693	2.26E-20
DLST	TRIM69	0.62107405	2.28E-20
DLST	B2M	0.62093785	2.33E-20

DLST	STX3	0.62087831	2.36E-20
DLST	EMC8	0.6206385	2.46E-20
DLST	REST	0.62061047	2.47E-20
DLST	PFKM	0.62059899	2.48E-20
DLST	GCC1	0.62058564	2.48E-20
DLST	BHLHE40	0.62052677	2.51E-20
DLST	PIP4P2	0.62046245	2.54E-20
DLST	TRMT2B	0.62043571	2.55E-20
DLST	APH1A	0.62037979	2.58E-20
DLST	DHRS7	0.62021406	2.65E-20
DLST	COQ5	0.62020828	2.66E-20
DLST	C1GALT1	0.62014809	2.69E-20
DLST	HNRNPL	0.6201259	2.7E-20
DLST	ERF	0.62012149	2.7E-20
DLST	SHC1	0.62011762	2.7E-20
DLST	CYREN	0.62009459	2.71E-20
DLST	FNDC3B	0.61985861	2.83E-20
DLST	CAMK2D	0.61984132	2.84E-20
DLST	BLOC1S6	0.61973716	2.89E-20
DLST	FAM122B	0.61971814	2.9E-20
DLST	BTF3	0.61966721	2.93E-20
DLST	SH3RF1	0.61957514	2.98E-20
DLST	AKIRIN1	0.6195161	3.01E-20
DLST	STK26	0.61944856	3.04E-20
DLST	TOR1A	0.61914799	3.21E-20
DLST	SH3KBP1	0.61895013	3.33E-20
DLST	PAQR3	0.61890291	3.35E-20
DLST	SLC35F6	0.61873473	3.46E-20
DLST	PPP1R11	0.61866852	3.5E-20
DLST	ATXN7L1	0.61864228	3.51E-20
DLST	HMGCR	0.61860632	3.54E-20
DLST	ELK3	0.61857388	3.56E-20
DLST	NAGA	0.61854652	3.57E-20
DLST	PPM1G	0.61844885	3.64E-20
DLST	RCC1	0.6184351	3.65E-20
DLST	FMN1	0.61841948	3.66E-20
DLST	COQ7	0.61824342	3.77E-20
DLST	ZNF816	0.61813289	3.85E-20
DLST	SCFD2	0.61803489	3.91E-20
DLST	GRB2	0.61801002	3.93E-20
DLST	ACSL3	0.61799267	3.94E-20
DLST	CTBS	0.61791296	4E-20
DLST	LLPH	0.61773833	4.13E-20
DLST	NSMCE2	0.61769706	4.16E-20
DLST	TMEM184B	0.61761591	4.22E-20
DLST	PLEC	0.61746065	4.33E-20
DLST	PIGK	0.61742567	4.36E-20
DLST	SMS	0.61726577	4.49E-20
DLST	CYB5B	0.61726293	4.49E-20
DLST	RARG	0.617117	4.61E-20
DLST	TCTN3	0.61711508	4.61E-20
DLST	LIN54	0.61707916	4.64E-20
DLST	TMEM167A	0.61702677	4.68E-20
DLST	PALLD	0.61699106	4.71E-20
DLST	SFXN3	0.61691872	4.77E-20
DLST	AP1M1	0.61686899	4.81E-20
DLST	NSD3	0.61682999	4.85E-20
DLST	SPTBN1	0.61682227	4.85E-20

DLST	CD164	0.61680659	4.87E-20
DLST	CBLB	0.61646048	5.17E-20
DLST	TMEM126B	0.6159503	5.66E-20
DLST	HMGB2	0.61562073	6E-20
DLST	OSER1	0.61557669	6.05E-20
DLST	CDK17	0.6155496	6.07E-20
DLST	ZWILCH	0.61551305	6.11E-20
DLST	GOLGA7	0.61520044	6.46E-20
DLST	TMEM50A	0.61513265	6.54E-20
DLST	HPS3	0.61507145	6.61E-20
DLST	PARP14	0.61504714	6.64E-20
DLST	SYF2	0.61484786	6.87E-20
DLST	APBB2	0.61483195	6.89E-20
DLST	PARP12	0.6147888	6.94E-20
DLST	HEATR5A	0.61476988	6.97E-20
DLST	PDP1	0.61474955	6.99E-20
DLST	MBTPS2	0.61471594	7.03E-20
DLST	NUDCD1	0.61470531	7.05E-20
DLST	RBMS2	0.61464549	7.12E-20
DLST	RFC3	0.61448707	7.32E-20
DLST	FER	0.61443841	7.38E-20
DLST	IGF2R	0.61442938	7.4E-20
DLST	FBXO28	0.61439739	7.44E-20
DLST	CD2BP2	0.61432037	7.54E-20
DLST	DCAF7	0.61430464	7.56E-20
DLST	OTUD7B	0.61420187	7.7E-20
DLST	CROT	0.61400449	7.97E-20
DLST	ZMPSTE24	0.61395435	8.04E-20
DLST	RCC2	0.61394647	8.05E-20
DLST	EMC1	0.61384033	8.2E-20
DLST	SMAD5	0.61380294	8.25E-20
DLST	OXNAD1	0.61377609	8.29E-20
DLST	C6orf47	0.61372348	8.37E-20
DLST	TTC1	0.61365886	8.46E-20
DLST	AHR	0.6136552	8.47E-20
DLST	ZNF350	0.61344499	8.79E-20
DLST	HSD17B11	0.61338694	8.88E-20
DLST	DTX3L	0.61334962	8.93E-20
DLST	AC106886.5	0.61306455	9.39E-20
DLST	LRRC1	0.61302835	9.45E-20
DLST	PEX14	0.61283675	9.77E-20
DLST	CHML	0.61282916	9.78E-20
DLST	PLA2G15	0.61277906	9.87E-20
DLST	IFT52	0.61275257	9.91E-20
DLST	SMIM12	0.61270271	1E-19
DLST	CSTF2T	0.61262312	1.01E-19
DLST	TAF13	0.61251939	1.03E-19
DLST	GRSF1	0.6124719	1.04E-19
DLST	MCM4	0.61243481	1.05E-19
DLST	CERS6	0.61241402	1.05E-19
DLST	UBAC2	0.61232443	1.07E-19
DLST	MAFG	0.61223279	1.09E-19
DLST	ZNF468	0.61207573	1.12E-19
DLST	WNK1	0.6120536	1.12E-19
DLST	PGM2L1	0.61193258	1.14E-19
DLST	FAM102A	0.61192052	1.15E-19
DLST	HDAC2	0.6118425	1.16E-19
DLST	SF3B2	0.61174664	1.18E-19

DLST	KIAA0232	0.6117444	1.18E-19
DLST	ATG7	0.61174062	1.18E-19
DLST	DCBLD1	0.61172141	1.19E-19
DLST	ERMP1	0.61171524	1.19E-19
DLST	SPTSSA	0.61165333	1.2E-19
DLST	ATP5MF-PTCD1	0.61163264	1.2E-19
DLST	GOLIM4	0.61156871	1.22E-19
DLST	TRUB2	0.61156775	1.22E-19
DLST	GLB1	0.61136389	1.26E-19
DLST	IRF2BP2	0.61130552	1.27E-19
DLST	ZNF148	0.61130218	1.28E-19
DLST	SLC35A1	0.61114744	1.31E-19
DLST	TMEM185A	0.6110409	1.33E-19
DLST	GLT8D1	0.61099879	1.34E-19
DLST	RER1	0.61098926	1.35E-19
DLST	ARHGAP42	0.61093758	1.36E-19
DLST	FUCA1	0.61089152	1.37E-19
DLST	PIK3CA	0.61088895	1.37E-19
DLST	GNA11	0.61088681	1.37E-19
DLST	TK2	0.61074945	1.4E-19
DLST	ZNF322	0.61071332	1.41E-19
DLST	PDAP1	0.61070793	1.41E-19
DLST	CNOT7	0.61064811	1.43E-19
DLST	AK6	0.61063746	1.43E-19
DLST	AHSA1	0.61062976	1.43E-19
DLST	ATP11A	0.61056896	1.45E-19
DLST	TRAK2	0.61056149	1.45E-19
DLST	E2F3	0.61041063	1.49E-19
DLST	RPA1	0.61040371	1.49E-19
DLST	DIABLO	0.61037149	1.5E-19
DLST	BAG4	0.6103036	1.52E-19
DLST	PLBD2	0.61026291	1.53E-19
DLST	STX6	0.610257	1.53E-19
DLST	PLCG1	0.61019744	1.54E-19
DLST	RRM1	0.61012366	1.56E-19
DLST	FKBP1A	0.6101174	1.57E-19
DLST	NIPSNAP3A	0.61007854	1.58E-19
DLST	CCSER2	0.61003383	1.59E-19
DLST	SERTAD2	0.60976222	1.66E-19
DLST	ING1	0.60968299	1.69E-19
DLST	ZFP1	0.60964538	1.7E-19
DLST	PSPC1	0.60963924	1.7E-19
DLST	SNX16	0.60960208	1.71E-19
DLST	SOCS5	0.60958788	1.72E-19
DLST	NEDD4	0.60953978	1.73E-19
DLST	KCTD11	0.60953048	1.73E-19
DLST	ARL13B	0.60947664	1.75E-19
DLST	NAPG	0.60944024	1.76E-19
DLST	AP3M2	0.60939547	1.77E-19
DLST	RSU1	0.60933721	1.79E-19
DLST	LRR1	0.60929111	1.81E-19
DLST	TRIM13	0.6092576	1.82E-19
DLST	RAB8B	0.60924791	1.82E-19
DLST	GOLPH3	0.60904052	1.89E-19
DLST	SMAD3	0.60880993	1.96E-19
DLST	ZFP90	0.60879945	1.97E-19
DLST	ARF1	0.60872805	1.99E-19
DLST	RBM18	0.60864224	2.02E-19

DLST	RASA2	0.60859277	2.04E-19
DLST	RIOX1	0.60843421	2.09E-19
DLST	CANX	0.60839832	2.11E-19
DLST	ARL6IP5	0.60839625	2.11E-19
DLST	KLHL2	0.60834455	2.12E-19
DLST	SMC1A	0.60825211	2.16E-19
DLST	TOMM34	0.60817717	2.19E-19
DLST	BCORL1	0.60788255	2.3E-19
DLST	ZC3HAV1	0.60783258	2.32E-19
DLST	CCT5	0.6077868	2.34E-19
DLST	KAT6A	0.60774817	2.35E-19
DLST	ZBTB41	0.60774399	2.35E-19
DLST	NEMP2	0.6076692	2.39E-19
DLST	ACVR2A	0.60766694	2.39E-19
DLST	BCL7B	0.60753561	2.44E-19
DLST	RETREG2	0.60745728	2.47E-19
DLST	EMC7	0.60728937	2.55E-19
DLST	TMEM237	0.60716604	2.6E-19
DLST	GTF3C4	0.60713004	2.62E-19
DLST	SYS1	0.60709084	2.63E-19
DLST	DUSP7	0.60688429	2.73E-19
DLST	PPP1R9B	0.60683592	2.75E-19
DLST	ARHGAP35	0.60676889	2.78E-19
DLST	LSM6	0.60672393	2.8E-19
DLST	ATP2B4	0.60662647	2.85E-19
DLST	LRRC8A	0.60661153	2.86E-19
DLST	HIPK3	0.60656718	2.88E-19
DLST	SGPP2	0.60649931	2.91E-19
DLST	ETV6	0.60632282	3E-19
DLST	OSBPL3	0.60627046	3.03E-19
DLST	ZNF226	0.60625255	3.04E-19
DLST	CAMSAP2	0.60616763	3.08E-19
DLST	MFSD1	0.60613163	3.1E-19
DLST	NEDD8	0.60612433	3.11E-19
DLST	GNPDA2	0.60602122	3.16E-19
DLST	ASPH	0.6060168	3.16E-19
DLST	NECAP2	0.60598234	3.18E-19
DLST	TJP2	0.60595355	3.2E-19
DLST	TVP23B	0.60593988	3.2E-19
DLST	GRK6	0.60592366	3.21E-19
DLST	TOR1B	0.60588419	3.23E-19
DLST	CALM3	0.60588407	3.23E-19
DLST	EXOSC9	0.605823	3.27E-19
DLST	MBNL1	0.60578707	3.29E-19
DLST	MPHOSPH6	0.60577547	3.29E-19
DLST	MECP2	0.60568463	3.35E-19
DLST	NSL1	0.60548511	3.46E-19
DLST	PDE12	0.60543245	3.49E-19
DLST	DAGLB	0.60540873	3.51E-19
DLST	RPF1	0.6052984	3.57E-19
DLST	BMT2	0.60526254	3.59E-19
DLST	OSBPL8	0.60524236	3.61E-19
DLST	HDAC1	0.60521514	3.62E-19
DLST	ARL14EP	0.60510398	3.69E-19
DLST	ABHD13	0.60506162	3.72E-19
DLST	RFX5	0.6050146	3.75E-19
DLST	HIVEP2	0.60471223	3.95E-19
DLST	COX7A2L	0.6046631	3.98E-19

DLST	SEC14L2	0.60439668	4.16E-19
DLST	ITM2B	0.6043771	4.18E-19
DLST	LHFPL2	0.60423265	4.28E-19
DLST	AP3B1	0.6042163	4.29E-19
DLST	ISY1	0.60410179	4.38E-19
DLST	LRRCC1	0.60398497	4.46E-19
DLST	ZNF586	0.60395929	4.48E-19
DLST	TMEM182	0.60386394	4.56E-19
DLST	CD46	0.60363967	4.73E-19
DLST	ZHX2	0.60357125	4.79E-19
DLST	DNAJC9	0.60348912	4.85E-19
DLST	SQSTM1	0.60348071	4.86E-19
DLST	HTRA2	0.60335617	4.96E-19
DLST	MAP3K13	0.60316849	5.12E-19
DLST	STIM1	0.60314529	5.14E-19
DLST	PRKCD	0.60298186	5.29E-19
DLST	ACTG1	0.60297457	5.29E-19
DLST	RPRD2	0.60296911	5.3E-19
DLST	FNIP2	0.60286049	5.4E-19
DLST	ZNF777	0.60280563	5.45E-19
DLST	NIPA1	0.60276651	5.48E-19
DLST	LACTB2	0.60267406	5.57E-19
DLST	KIF13A	0.60266312	5.58E-19
DLST	PKM	0.60257105	5.66E-19
DLST	PRR12	0.60250582	5.73E-19
DLST	SPDYE3	0.6024048	5.82E-19
DLST	AAMP	0.60238671	5.84E-19
DLST	PXMP4	0.60232388	5.9E-19
DLST	APOL6	0.6022614	5.97E-19
DLST	FOSL2	0.60222305	6.01E-19
DLST	DPH3	0.60218776	6.04E-19
DLST	SAMD9	0.60218078	6.05E-19
DLST	TBC1D5	0.60212526	6.1E-19
DLST	KIF20B	0.60209515	6.14E-19
DLST	BTF3L4	0.60209295	6.14E-19
DLST	SUSD1	0.60195644	6.28E-19
DLST	SDHB	0.60195376	6.28E-19
DLST	CAV2	0.60179604	6.45E-19
DLST	MYO6	0.60178327	6.47E-19
DLST	DSTN	0.60169419	6.56E-19
DLST	GPKOW	0.60163133	6.63E-19
DLST	TNIP1	0.60159173	6.68E-19
DLST	FGD4	0.60152747	6.75E-19
DLST	CHIC2	0.60126988	7.05E-19
DLST	RPS6KA4	0.60108312	7.27E-19
DLST	CDK2AP1	0.60097125	7.41E-19
DLST	TTC23	0.60094505	7.44E-19
DLST	ITGAV	0.60093952	7.45E-19
DLST	CNOT4	0.60088724	7.51E-19
DLST	MRTFA	0.60083613	7.58E-19
DLST	FBLIM1	0.60079635	7.63E-19
DLST	PEAK1	0.60068011	7.78E-19
DLST	MTERF3	0.60065767	7.81E-19
DLST	NCOR2	0.60058447	7.9E-19
DLST	VBP1	0.60057198	7.92E-19
DLST	TMEM9B	0.60030213	8.28E-19
DLST	NUDT4	0.60024545	8.36E-19
DLST	PTEN	0.60020746	8.41E-19

DLST	RRP15	0.60004532	8.64E-19
DLST	MDH1	0.59999598	8.72E-19
DLST	RMDN1	0.59962817	9.27E-19
DLST	ST13	0.59961733	9.28E-19
DLST	MAN2B2	0.59960201	9.31E-19
DLST	TMEM68	0.59951367	9.44E-19
DLST	SLC35C1	0.59947579	9.5E-19
DLST	SPRY4	0.59941083	9.61E-19
DLST	POLDIP3	0.59939957	9.63E-19
DLST	SOCS6	0.59938813	9.64E-19
DLST	UPF1	0.59932416	9.75E-19
DLST	TTL	0.59931384	9.76E-19
DLST	ABLIM3	0.5992691	9.84E-19
DLST	PITPNA	0.59925753	9.86E-19
DLST	CLCN3	0.59920154	9.95E-19
DLST	DUSP6	0.59906248	1.02E-18
DLST	ZCCHC17	0.59878074	1.07E-18
DLST	PPIL3	0.59862312	1.09E-18
DLST	BROX	0.59840677	1.13E-18
DLST	RNF170	0.59838178	1.14E-18
DLST	SNX15	0.59834387	1.15E-18
DLST	CLDN12	0.59820754	1.17E-18
DLST	TINF2	0.59811384	1.19E-18
DLST	AMMECR1	0.59810701	1.19E-18
DLST	SRP14	0.59802797	1.21E-18
DLST	PRR13	0.59793855	1.23E-18
DLST	TOPORS	0.59782677	1.25E-18
DLST	WBP4	0.59756467	1.3E-18
DLST	SMU1	0.59756296	1.31E-18
DLST	DNAL1	0.59737283	1.35E-18
DLST	CKAP2	0.59727709	1.37E-18
DLST	MAPK7	0.59711354	1.41E-18
DLST	GMCL1	0.59699214	1.43E-18
DLST	LBH	0.59696271	1.44E-18
DLST	GOLT1B	0.59690458	1.45E-18
DLST	ACTR1A	0.5969033	1.46E-18
DLST	SUMF1	0.59681426	1.48E-18
DLST	RAVER1	0.59678164	1.48E-18
DLST	IL10RB	0.59662407	1.52E-18
DLST	AK4	0.59653071	1.55E-18
DLST	CMAS	0.5964637	1.56E-18
DLST	SLC16A1	0.5964622	1.56E-18
DLST	BPTF	0.59642659	1.57E-18
DLST	ARHGAP11A	0.59635506	1.59E-18
DLST	SMG5	0.59627701	1.61E-18
DLST	CRIPT	0.59624812	1.62E-18
DLST	SH3BP2	0.59621349	1.63E-18
DLST	RAB9A	0.59615222	1.65E-18
DLST	WIPI2	0.59610497	1.66E-18
DLST	AGO2	0.59609585	1.66E-18
DLST	PVR	0.59604762	1.68E-18
DLST	HS1BP3	0.59591205	1.71E-18
DLST	CHMP4B	0.59571923	1.77E-18
DLST	MLH3	0.59567243	1.78E-18
DLST	NRP2	0.59547595	1.84E-18
DLST	NYNRIN	0.59543108	1.85E-18
DLST	CD82	0.59542749	1.85E-18
DLST	PRR14L	0.59541984	1.86E-18

DLST	TMCO3	0.59536356	1.87E-18
DLST	SPCS2	0.59536145	1.87E-18
DLST	TLN1	0.5953467	1.88E-18
DLST	APMAP	0.59512616	1.95E-18
DLST	GTPBP10	0.59510999	1.95E-18
DLST	AIMP2	0.59494445	2.01E-18
DLST	TMEM230	0.59487622	2.03E-18
DLST	CHUK	0.59482477	2.05E-18
DLST	PHF20	0.59480274	2.05E-18
DLST	PSMA6	0.59471797	2.08E-18
DLST	CSGALNACT2	0.5945554	2.14E-18
DLST	CEBPG	0.59453847	2.15E-18
DLST	VDR	0.59432683	2.22E-18
DLST	AFG3L2	0.59432408	2.22E-18
DLST	CTSA	0.59429373	2.23E-18
DLST	PPP1R21	0.59429255	2.23E-18
DLST	DDX60	0.59425503	2.25E-18
DLST	DARS2	0.5942088	2.26E-18
DLST	GALNT7	0.59420824	2.26E-18
DLST	DNAAF5	0.59411046	2.3E-18
DLST	SLC25A17	0.5937925	2.42E-18
DLST	DCAF6	0.59367215	2.47E-18
DLST	BCL2L1	0.59364296	2.48E-18
DLST	GSTCD	0.59362496	2.49E-18
DLST	PHACTR2	0.59362331	2.49E-18
DLST	EVI5L	0.5936064	2.5E-18
DLST	VDAC3	0.5935571	2.52E-18
DLST	LMO7	0.59332013	2.62E-18
DLST	PNP	0.59327998	2.63E-18
DLST	LIMK1	0.59327704	2.64E-18
DLST	TFRC	0.5932325	2.65E-18
DLST	COPZ1	0.59322169	2.66E-18
DLST	MZT1	0.59319335	2.67E-18
DLST	STEAP2	0.59318094	2.68E-18
DLST	CRK	0.59314135	2.69E-18
DLST	GADD45A	0.5930223	2.75E-18
DLST	SUFU	0.59290048	2.8E-18
DLST	IFT57	0.59258239	2.95E-18
DLST	TRIQK	0.59256832	2.96E-18
DLST	SRGAP2	0.59254459	2.97E-18
DLST	ZC4H2	0.5924188	3.03E-18
DLST	ABTB2	0.59235215	3.06E-18
DLST	FAM20B	0.59228168	3.1E-18
DLST	TULP3	0.59224735	3.12E-18
DLST	ITPR3	0.59212762	3.18E-18
DLST	UTP11	0.5919737	3.26E-18
DLST	FRMD4B	0.59196764	3.26E-18
DLST	LIN9	0.59183296	3.33E-18
DLST	SCAMP1	0.59181601	3.34E-18
DLST	HK1	0.59175437	3.37E-18
DLST	PRKCI	0.59159598	3.46E-18
DLST	UNC50	0.59151482	3.51E-18
DLST	XXYLT1	0.59100314	3.81E-18
DLST	TARS2	0.59092071	3.86E-18
DLST	AK2	0.59085141	3.9E-18
DLST	ENOX2	0.59082196	3.92E-18
DLST	STPG1	0.59079999	3.94E-18
DLST	EIF3M	0.59079232	3.94E-18

DLST	ETS2	0.59072174	3.99E-18
DLST	YAP1	0.59071954	3.99E-18
DLST	G6PD	0.59067848	4.02E-18
DLST	LPIN2	0.59066484	4.02E-18
DLST	TSPAN3	0.59056124	4.09E-18
DLST	PLEKHM2	0.59050908	4.13E-18
DLST	TRAM1	0.5904137	4.19E-18
DLST	ADD3	0.59037074	4.22E-18
DLST	COL4A2	0.59036896	4.22E-18
DLST	TNFRSF1A	0.59026003	4.3E-18
DLST	ZNF398	0.5901931	4.34E-18
DLST	CEP170	0.59017149	4.36E-18
DLST	CFLAR	0.59002428	4.46E-18
DLST	TEX10	0.58997049	4.5E-18
DLST	TGFBR1	0.58996469	4.5E-18
DLST	LYST	0.58991823	4.54E-18
DLST	CEP41	0.58987144	4.57E-18
DLST	TANC2	0.58986061	4.58E-18
DLST	KIFAP3	0.58974831	4.66E-18
DLST	TLK2	0.58969495	4.7E-18
DLST	CALU	0.58967512	4.72E-18
DLST	CENPL	0.5895491	4.82E-18
DLST	PIK3R2	0.58952256	4.84E-18
DLST	HCFC1	0.58941882	4.92E-18
DLST	PCDH1	0.58938797	4.94E-18
DLST	TRMT1L	0.58933405	4.99E-18
DLST	OSGIN2	0.58914134	5.14E-18
DLST	DSG2	0.58912441	5.16E-18
DLST	INTS5	0.588951	5.3E-18
DLST	SS18	0.5888501	5.39E-18
DLST	BRK1	0.58871256	5.51E-18
DLST	KNSTRN	0.58869926	5.52E-18
DLST	SNX17	0.58862001	5.59E-18
DLST	PTGR1	0.588531	5.67E-18
DLST	EHD2	0.58837867	5.81E-18
DLST	SLC30A1	0.5883567	5.83E-18
DLST	NAPEPLD	0.58825571	5.93E-18
DLST	SMG8	0.58815789	6.02E-18
DLST	OTUD1	0.58809345	6.08E-18
DLST	ZNF438	0.58805628	6.12E-18
DLST	STON2	0.58790185	6.27E-18
DLST	PKNOX1	0.5879009	6.27E-18
DLST	BRIX1	0.5877432	6.43E-18
DLST	FAM102B	0.5877103	6.47E-18
DLST	CDC123	0.58753676	6.65E-18
DLST	VCP	0.58750276	6.68E-18
DLST	ANTXR2	0.58740549	6.79E-18
DLST	CAMK2N1	0.58739384	6.8E-18
DLST	NGRN	0.58736762	6.83E-18
DLST	PRMT5	0.58731396	6.89E-18
DLST	WDR12	0.58722337	6.99E-18
DLST	RNF40	0.58717935	7.04E-18
DLST	FZD6	0.58709884	7.13E-18
DLST	SSH1	0.58705822	7.17E-18
DLST	MAGOH	0.58700742	7.23E-18
DLST	RELL1	0.58697986	7.26E-18
DLST	STIP1	0.58694092	7.31E-18
DLST	WASHC3	0.58680599	7.47E-18

DLST	ACAA2	0.58678722	7.49E-18
DLST	RTL6	0.5867537	7.53E-18
DLST	MYADM	0.58661317	7.7E-18
DLST	SPIN4	0.58655883	7.77E-18
DLST	ADIPOR1	0.58653021	7.8E-18
DLST	SRXN1	0.58637183	8E-18
DLST	PIAS1	0.58623835	8.17E-18
DLST	CZIB	0.58612669	8.32E-18
DLST	LAP3	0.58609071	8.37E-18
DLST	COQ6	0.58601329	8.47E-18
DLST	MSL2	0.58597025	8.53E-18
DLST	DOCK5	0.58586091	8.68E-18
DLST	ADO	0.58584329	8.7E-18
DLST	UNC5B	0.58583621	8.71E-18
DLST	FAM104A	0.58580977	8.75E-18
DLST	FUT11	0.58554333	9.13E-18
DLST	DAXX	0.58549494	9.2E-18
DLST	PLEKHA5	0.58547212	9.23E-18
DLST	SRGAP2C	0.5854259	9.3E-18
DLST	PEA15	0.58537183	9.38E-18
DLST	MAP7D1	0.58512842	9.75E-18
DLST	CIP2A	0.58509271	9.8E-18
DLST	TMBIM1	0.58502038	9.91E-18
DLST	ATAD2	0.58493748	1E-17
DLST	TIPARP	0.58488765	1.01E-17
DLST	UPRT	0.58484763	1.02E-17
DLST	CMIP	0.58481286	1.02E-17
DLST	DHX30	0.58473668	1.04E-17
DLST	RCN2	0.58467643	1.05E-17
DLST	LDHA	0.58465351	1.05E-17
DLST	TMEM183A	0.58454766	1.07E-17
DLST	OAT	0.58453493	1.07E-17
DLST	ARPC3	0.58436831	1.1E-17
DLST	SERINC1	0.58419243	1.13E-17
DLST	RIOK3	0.58418287	1.13E-17
DLST	COL4A1	0.58391937	1.18E-17
DLST	SP100	0.58383773	1.19E-17
DLST	NGDN	0.58379111	1.2E-17
DLST	AKIP1	0.58378068	1.21E-17
DLST	RBBP5	0.58345078	1.27E-17
DLST	BLOC1S5	0.58337169	1.29E-17
DLST	ITGB5	0.58325322	1.31E-17
DLST	DGCR2	0.58322934	1.31E-17
DLST	HECA	0.58320351	1.32E-17
DLST	KCTD3	0.58319955	1.32E-17
DLST	TM9SF2	0.58315559	1.33E-17
DLST	RALY	0.58314541	1.33E-17
DLST	LRIF1	0.58311608	1.34E-17
DLST	MAML2	0.5830833	1.35E-17
DLST	HFE	0.58307635	1.35E-17
DLST	MANBA	0.58307146	1.35E-17
DLST	TWSG1	0.58293062	1.38E-17
DLST	AFTPH	0.58291188	1.38E-17
DLST	SF3B6	0.58290693	1.38E-17
DLST	ZNF532	0.5828627	1.39E-17
DLST	MRPL16	0.58276431	1.41E-17
DLST	SNUPN	0.58274138	1.42E-17
DLST	GNA12	0.58270467	1.43E-17

DLST	ADK	0.58262907	1.44E-17
DLST	RBBP9	0.58261171	1.45E-17
DLST	DHFR	0.58240526	1.5E-17
DLST	OXCT1	0.58233303	1.51E-17
DLST	CDCA4	0.58230742	1.52E-17
DLST	CCNY	0.58214451	1.56E-17
DLST	XPR1	0.58196148	1.6E-17
DLST	CHP1	0.58185033	1.63E-17
DLST	PIM1	0.58184372	1.63E-17
DLST	PLS1	0.58166758	1.68E-17
DLST	TRANK1	0.58158738	1.7E-17
DLST	SLC17A5	0.5812777	1.79E-17
DLST	TTC33	0.58124474	1.79E-17
DLST	POC1B	0.58122001	1.8E-17
DLST	FAT1	0.58099737	1.87E-17
DLST	DDB1	0.58073149	1.94E-17
DLST	TMCO1	0.58072035	1.95E-17
DLST	FAM234A	0.58070652	1.95E-17
DLST	PRKAR1A	0.58056823	1.99E-17
DLST	ATP9A	0.58043196	2.04E-17
DLST	IQCK	0.5802295	2.1E-17
DLST	RANBP3	0.58016972	2.12E-17
DLST	FCHSD2	0.58014351	2.13E-17
DLST	ZBTB6	0.57996878	2.19E-17
DLST	KANK2	0.57993815	2.2E-17
DLST	DYNLL1	0.57983292	2.24E-17
DLST	MREG	0.57978617	2.25E-17
DLST	DCAF13	0.57976514	2.26E-17
DLST	RAP2A	0.57976269	2.26E-17
DLST	XYLT1	0.57971537	2.28E-17
DLST	CCDC47	0.5797107	2.28E-17
DLST	ATP10D	0.57964645	2.3E-17
DLST	TM2D2	0.57958119	2.33E-17
DLST	MEX3D	0.57955329	2.34E-17
DLST	KCTD1	0.57944253	2.38E-17
DLST	SDF4	0.57939686	2.39E-17
DLST	BHLHE41	0.579389	2.4E-17
DLST	PSMA5	0.57919934	2.47E-17
DLST	BTBD9	0.57918077	2.47E-17
DLST	INPP4B	0.5791528	2.49E-17
DLST	TNFRSF10A	0.57913538	2.49E-17
DLST	SKIL	0.57906288	2.52E-17
DLST	ALAS1	0.57901048	2.54E-17
DLST	MTCH2	0.57897799	2.55E-17
DLST	ENDOD1	0.5789664	2.56E-17
DLST	PSMA4	0.57892615	2.57E-17
DLST	PBDC1	0.57872849	2.65E-17
DLST	METTL21A	0.57870884	2.66E-17
DLST	URGCP	0.57869562	2.67E-17
DLST	ACTR10	0.57869134	2.67E-17
DLST	WRNIP1	0.57865927	2.68E-17
DLST	HMGN1	0.57860714	2.7E-17
DLST	HACD3	0.57840433	2.79E-17
DLST	UBE2L6	0.57836081	2.81E-17
DLST	NIFK	0.57832636	2.82E-17
DLST	NAV1	0.57832081	2.83E-17
DLST	TFPI	0.57825526	2.86E-17
DLST	NOTCH3	0.57814305	2.91E-17

DLST	RALGPS2	0.57811894	2.92E-17
DLST	BPGM	0.57805569	2.94E-17
DLST	IRF9	0.57804773	2.95E-17
DLST	UTP25	0.57801087	2.97E-17
DLST	SORD	0.57800617	2.97E-17
DLST	HEXIM1	0.5779935	2.97E-17
DLST	TCAF1	0.57798905	2.98E-17
DLST	GFPT1	0.57797085	2.98E-17
DLST	SGO2	0.577934	3E-17
DLST	HRH1	0.57782695	3.05E-17
DLST	ASH2L	0.5777982	3.06E-17
DLST	MRPS23	0.57765547	3.13E-17
DLST	ANO10	0.57763397	3.14E-17
DLST	COMMD7	0.57750887	3.2E-17
DLST	LTA4H	0.57744933	3.23E-17
DLST	ILK	0.57741861	3.25E-17
DLST	COMMD8	0.5774066	3.26E-17
DLST	MCM6	0.57725351	3.33E-17
DLST	PLEKHF2	0.57722127	3.35E-17
DLST	PTP4A2	0.57709325	3.42E-17
DLST	RPL26L1	0.57703228	3.45E-17
DLST	CYTH2	0.57700853	3.46E-17
DLST	AKIRIN2	0.57680227	3.57E-17
DLST	SEC11A	0.57677641	3.59E-17
DLST	MED15	0.57676613	3.59E-17
DLST	TM9SF3	0.5767346	3.61E-17
DLST	IPPK	0.57669482	3.63E-17
DLST	TMEM35B	0.57662169	3.67E-17
DLST	ABCB10	0.57653234	3.72E-17
DLST	AC010132.3	0.57641855	3.79E-17
DLST	EIF3H	0.57620685	3.91E-17
DLST	TMEM41A	0.5760887	3.99E-17
DLST	ARHGAP12	0.57605262	4.01E-17
DLST	NID1	0.57593404	4.08E-17
DLST	PIGW	0.57577579	4.18E-17
DLST	OPA3	0.57574368	4.2E-17
DLST	UHRF1BP1L	0.57569494	4.23E-17
DLST	ZBTB33	0.57566216	4.26E-17
DLST	KDELR2	0.57565727	4.26E-17
DLST	PSMD8	0.57560504	4.29E-17
DLST	HTATSF1	0.57550961	4.36E-17
DLST	KIF3B	0.57541631	4.42E-17
DLST	CDK19	0.57536632	4.45E-17
DLST	DUS4L	0.57529213	4.5E-17
DLST	ASAH1	0.57525685	4.53E-17
DLST	CDKN1B	0.57522074	4.55E-17
DLST	NCKIPSD	0.57510013	4.64E-17
DLST	CDK6	0.57501056	4.7E-17
DLST	CTSO	0.57496039	4.74E-17
DLST	EIPR1	0.5749063	4.78E-17
DLST	CNST	0.57481569	4.84E-17
DLST	KATNA1	0.57475012	4.89E-17
DLST	CLIP4	0.5746283	4.99E-17
DLST	MAPK3	0.57456258	5.04E-17
DLST	CCDC71	0.57455844	5.04E-17
DLST	SYNJ2	0.57445271	5.12E-17
DLST	DNAJC2	0.57442117	5.15E-17
DLST	PSME1	0.57381317	5.64E-17

DLST	VRK1	0.57376848	5.68E-17
DLST	NUDT4B	0.57371548	5.73E-17
DLST	GPATCH2	0.57371498	5.73E-17
DLST	ACLY	0.57355055	5.87E-17
DLST	LCORL	0.57354417	5.88E-17
DLST	PLAA	0.57335457	6.05E-17
DLST	TADA1	0.57322467	6.17E-17
DLST	NET1	0.5731281	6.26E-17
DLST	WDHD1	0.5729684	6.42E-17
DLST	DBF4	0.57267726	6.71E-17
DLST	MED13	0.5725481	6.84E-17
DLST	HNRNPA3	0.57252363	6.87E-17
DLST	MFAP3	0.57249773	6.89E-17
DLST	DPYD	0.57216913	7.25E-17
DLST	TSPAN5	0.57212917	7.29E-17
DLST	CLSTN1	0.57208203	7.34E-17
DLST	FYTTD1	0.57207907	7.34E-17
DLST	UGCG	0.57205893	7.37E-17
DLST	SAP30	0.57190115	7.55E-17
DLST	TXNIP	0.57189191	7.56E-17
DLST	GNB5	0.57186142	7.59E-17
DLST	ZNF174	0.57183227	7.62E-17
DLST	ZCCHC10	0.57182091	7.64E-17
DLST	CLTC	0.57180771	7.65E-17
DLST	GTDC1	0.57169464	7.78E-17
DLST	WWC3	0.57165396	7.83E-17
DLST	JPT2	0.57157405	7.93E-17
DLST	GALNS	0.57153181	7.98E-17
DLST	EMC4	0.57150148	8.01E-17
DLST	SMIM30	0.57141674	8.12E-17
DLST	RND3	0.57133778	8.22E-17
DLST	ERGIC2	0.57130128	8.26E-17
DLST	TMEM199	0.57127718	8.29E-17
DLST	TBC1D10B	0.57123994	8.34E-17
DLST	PI4K2A	0.57116232	8.44E-17
DLST	DGKH	0.57110521	8.51E-17
DLST	POLR2K	0.57104714	8.58E-17
DLST	LAMC1	0.57103262	8.6E-17
DLST	AMIGO2	0.57099922	8.65E-17
DLST	RNF20	0.57096811	8.69E-17
DLST	ATP6V1E1	0.5708836	8.8E-17
DLST	GLE1	0.57063014	9.14E-17
DLST	PRMT2	0.57040529	9.46E-17
DLST	MITD1	0.57038138	9.49E-17
DLST	GMPS	0.57031797	9.58E-17
DLST	EIF4G1	0.5702746	9.64E-17
DLST	NDUFS4	0.5701075	9.89E-17
DLST	DAD1	0.569834	1.03E-16
DLST	SMAD7	0.56981961	1.03E-16
DLST	SPRTN	0.56975178	1.04E-16
DLST	PPP6R1	0.56964751	1.06E-16
DLST	NPLOC4	0.56959278	1.07E-16
DLST	ATP6AP2	0.56959058	1.07E-16
DLST	AAGAB	0.5695821	1.07E-16
DLST	GPN2	0.5695687	1.07E-16
DLST	RCE1	0.56948982	1.08E-16
DLST	TMEM14A	0.56937849	1.1E-16
DLST	GSS	0.56920086	1.13E-16

DLST	THOC3	0.56918592	1.14E-16
DLST	PPARD	0.56892342	1.18E-16
DLST	LRRC8D	0.56891225	1.18E-16
DLST	PDZD11	0.56874249	1.21E-16
DLST	NDRG3	0.56866188	1.23E-16
DLST	HDAC7	0.56852312	1.25E-16
DLST	LY75	0.56830097	1.3E-16
DLST	ADGRG1	0.56829713	1.3E-16
DLST	MCU	0.56819801	1.32E-16
DLST	METTL8	0.56815613	1.32E-16
DLST	NMI	0.56797481	1.36E-16
DLST	TMEM216	0.56790026	1.38E-16
DLST	ENSA	0.56761195	1.44E-16
DLST	EXOC2	0.56740592	1.48E-16
DLST	ETS1	0.56740064	1.48E-16
DLST	NUP155	0.56729514	1.51E-16
DLST	STMP1	0.56726048	1.51E-16
DLST	ERCC2	0.56724627	1.52E-16
DLST	CDK4	0.56720405	1.53E-16
DLST	LMNB2	0.56717667	1.53E-16
DLST	ELK4	0.56716354	1.54E-16
DLST	SRF	0.56712791	1.54E-16
DLST	FARSB	0.56711055	1.55E-16
DLST	GPR137B	0.56703534	1.57E-16
DLST	DPY30	0.5669507	1.59E-16
DLST	CLIP2	0.56694918	1.59E-16
DLST	FRMD8	0.56692397	1.59E-16
DLST	IRS1	0.56676881	1.63E-16
DLST	ACO2	0.56672685	1.64E-16
DLST	CNOT10	0.5666673	1.65E-16
DLST	MRPS35	0.56666162	1.66E-16
DLST	RBMX2	0.56638615	1.72E-16
DLST	SBNO2	0.56635769	1.73E-16
DLST	VOPP1	0.56633435	1.74E-16
DLST	FRRS1	0.56628221	1.75E-16
DLST	PAIP1	0.56612795	1.79E-16
DLST	ADCY7	0.5661082	1.8E-16
DLST	PTPRJ	0.56606437	1.81E-16
DLST	MAF1	0.56606235	1.81E-16
DLST	GGPS1	0.56604286	1.81E-16
DLST	MRM2	0.56593132	1.84E-16
DLST	STAT1	0.56587262	1.86E-16
DLST	ATP6V0E1	0.56577107	1.89E-16
DLST	DENND1C	0.56576957	1.89E-16
DLST	TMEM19	0.56565659	1.92E-16
DLST	ATP6V0D1	0.56559779	1.94E-16
DLST	NOD1	0.565341	2.01E-16
DLST	PARP9	0.56523824	2.04E-16
DLST	MED26	0.56521299	2.05E-16
DLST	CPD	0.56515419	2.07E-16
DLST	MSANTD3	0.5650168	2.11E-16
DLST	TMEM251	0.56489254	2.15E-16
DLST	SMARCB1	0.56432271	2.34E-16
DLST	SLC25A12	0.56421499	2.38E-16
DLST	RETSAT	0.56412481	2.41E-16
DLST	MED7	0.56404734	2.44E-16
DLST	LYAR	0.56398537	2.46E-16
DLST	VPS33A	0.56373575	2.55E-16

DLST	PTPRF	0.5637222	2.56E-16
DLST	PLEKHA2	0.56366203	2.58E-16
DLST	PTMA	0.56356009	2.62E-16
DLST	TEAD3	0.56350549	2.64E-16
DLST	CCT8	0.56350377	2.64E-16
DLST	PAK1IP1	0.56348366	2.65E-16
DLST	AKTIP	0.5633693	2.69E-16
DLST	MTHFD1	0.56330355	2.72E-16
DLST	PHF5A	0.56309327	2.8E-16
DLST	B4GALT1	0.56308117	2.81E-16
DLST	DENND3	0.56302397	2.83E-16
DLST	RPN1	0.56300322	2.84E-16
DLST	IAH1	0.56294197	2.87E-16
DLST	CUEDC1	0.56277655	2.94E-16
DLST	RNF24	0.5627662	2.94E-16
DLST	ARNTL2	0.56264862	2.99E-16
DLST	RHOF	0.56250973	3.05E-16
DLST	SCOC	0.56222153	3.19E-16
DLST	SWAP70	0.5621119	3.24E-16
DLST	SLC2A1	0.5620107	3.29E-16
DLST	PIP5K1C	0.56186871	3.35E-16
DLST	FOXN3	0.56185913	3.36E-16
DLST	TBC1D14	0.56180142	3.39E-16
DLST	ITGA6	0.56178512	3.4E-16
DLST	NFATC3	0.56176619	3.41E-16
DLST	CENPK	0.56173778	3.42E-16
DLST	NCDN	0.56164181	3.47E-16
DLST	EHD1	0.56160527	3.49E-16
DLST	TTYH3	0.56146487	3.56E-16
DLST	TUBB	0.56136832	3.61E-16
DLST	LSM8	0.56124472	3.68E-16
DLST	UBC	0.56118594	3.71E-16
DLST	CYTH3	0.561181	3.71E-16
DLST	POLR3GL	0.56084011	3.9E-16
DLST	RRAS2	0.56078669	3.93E-16
DLST	MCL1	0.56065731	4E-16
DLST	ARFIP1	0.56047513	4.11E-16
DLST	MSN	0.56047478	4.11E-16
DLST	CCT6A	0.56046034	4.12E-16
DLST	NUSAP1	0.56044415	4.13E-16
DLST	KDM1B	0.56033187	4.2E-16
DLST	UMPS	0.56015476	4.31E-16
DLST	RRM2B	0.56008991	4.35E-16
DLST	UBE2D1	0.56005195	4.37E-16
DLST	SRC	0.56004806	4.37E-16
DLST	GINM1	0.55992585	4.45E-16
DLST	DIPK2A	0.55989068	4.48E-16
DLST	RPS6KA1	0.55987202	4.49E-16
DLST	FN3KRP	0.55979778	4.54E-16
DLST	TMEM263	0.55970043	4.6E-16
DLST	LAMP1	0.55964607	4.64E-16
DLST	RASA3	0.55950179	4.74E-16
DLST	OGT	0.55938593	4.82E-16
DLST	THOC7	0.55929885	4.88E-16
DLST	BCL9	0.5592933	4.88E-16
DLST	VANGL1	0.55928599	4.89E-16
DLST	MARCKS	0.55924579	4.92E-16
DLST	RHNO1	0.55919491	4.95E-16

DLST	NDUFA12	0.55909839	5.02E-16
DLST	ECT2	0.55908029	5.03E-16
DLST	SC5D	0.55904029	5.06E-16
DLST	MED8	0.55898883	5.1E-16
DLST	CES2	0.55882697	5.22E-16
DLST	DBNL	0.55882594	5.22E-16
DLST	PSMD9	0.55875738	5.28E-16
DLST	TMTC3	0.5587288	5.3E-16
DLST	SLC38A6	0.55847669	5.49E-16
DLST	ELP3	0.55836524	5.58E-16
DLST	AGPAT5	0.55822756	5.7E-16
DLST	ENO1	0.55816124	5.75E-16
DLST	RHOC	0.55800574	5.88E-16
DLST	NEO1	0.55796237	5.92E-16
DLST	SYTL2	0.55795604	5.92E-16
DLST	PLSCR1	0.55775661	6.1E-16
DLST	FARP2	0.55773455	6.12E-16
DLST	LTBP1	0.55772577	6.12E-16
DLST	EID1	0.55769678	6.15E-16
DLST	CDIPT	0.55746719	6.36E-16
DLST	NUP133	0.55740504	6.41E-16
DLST	SLC39A1	0.55739062	6.43E-16
DLST	PCYOX1	0.55727884	6.53E-16
DLST	NOCT	0.557174	6.63E-16
DLST	PSMA7	0.55716931	6.63E-16
DLST	ZNF28	0.55708733	6.71E-16
DLST	HMOX2	0.55706039	6.74E-16
DLST	ANKRD42	0.55705171	6.75E-16
DLST	BUB1	0.55702064	6.78E-16
DLST	MIA2	0.55692198	6.87E-16
DLST	DIPK1A	0.55691356	6.88E-16
DLST	IPO11	0.55675993	7.04E-16
DLST	AGPAT1	0.55640212	7.41E-16
DLST	OTULIN	0.55623519	7.59E-16
DLST	IFNGR1	0.55618283	7.64E-16
DLST	SLC38A1	0.55616158	7.67E-16
DLST	CDCP1	0.55580074	8.08E-16
DLST	SLC20A1	0.55577309	8.11E-16
DLST	PHTF1	0.55573485	8.15E-16
DLST	RNF7	0.55557484	8.34E-16
DLST	SAMD12	0.55546472	8.47E-16
DLST	PCNA	0.55546135	8.48E-16
DLST	BLCAP	0.55545524	8.48E-16
DLST	SUGT1	0.55544021	8.5E-16
DLST	MPZL3	0.55541173	8.54E-16
DLST	CMC2	0.55539759	8.55E-16
DLST	IQCE	0.55536419	8.6E-16
DLST	GTF2F2	0.55527121	8.71E-16
DLST	RAI14	0.55514964	8.86E-16
DLST	SLC41A2	0.55509804	8.93E-16
DLST	TMEM209	0.55502394	9.02E-16
DLST	CHMP1A	0.55497126	9.09E-16
DLST	CYLD	0.55492624	9.15E-16
DLST	RAMAC	0.55488682	9.2E-16
DLST	PRRG4	0.55487021	9.22E-16
DLST	NPC1	0.55483746	9.27E-16
DLST	TM2D3	0.55481484	9.3E-16
DLST	TMEM140	0.5547146	9.43E-16

DLST	FASTKD5	0.55464979	9.52E-16
DLST	ITGA1	0.55451768	9.7E-16
DLST	GPSM2	0.55449902	9.73E-16
DLST	RALGDS	0.55448631	9.74E-16
DLST	SIGMAR1	0.55448142	9.75E-16
DLST	MEX3C	0.55447244	9.76E-16
DLST	CMTM1	0.55441669	9.84E-16
DLST	METTL4	0.55424932	1.01E-15
DLST	NT5C3A	0.55419982	1.02E-15
DLST	C4orf33	0.55406038	1.04E-15
DLST	LACC1	0.55402686	1.04E-15
DLST	TADA3	0.55389597	1.06E-15
DLST	TRIP4	0.55389364	1.06E-15
DLST	CLIC4	0.55388137	1.06E-15
DLST	CHSY1	0.55384475	1.07E-15
DLST	RPTOR	0.55376425	1.08E-15
DLST	CALHM2	0.5536255	1.1E-15
DLST	SYAP1	0.55360156	1.11E-15
DLST	STK38	0.55357371	1.11E-15
DLST	TLN2	0.55351431	1.12E-15
DLST	PLCD3	0.5532398	1.16E-15
DLST	AXIN1	0.55310313	1.19E-15
DLST	HSPG2	0.55301147	1.2E-15
DLST	SNX3	0.5530004	1.2E-15
DLST	NCK2	0.55299979	1.2E-15
DLST	RTCB	0.55295715	1.21E-15
DLST	LIX1L	0.55284497	1.23E-15
DLST	CETN3	0.55279681	1.24E-15
DLST	MIS18A	0.55273496	1.25E-15
DLST	ENAH	0.55263013	1.27E-15
DLST	TTC26	0.55261288	1.27E-15
DLST	TNIP2	0.552465	1.3E-15
DLST	NHS	0.55237736	1.32E-15
DLST	TPM1	0.55223943	1.34E-15
DLST	LMBRD1	0.55221608	1.35E-15
DLST	H6PD	0.55221519	1.35E-15
DLST	COLGALT1	0.55221425	1.35E-15
DLST	SNX18	0.55208518	1.37E-15
DLST	SRI	0.55208418	1.37E-15
DLST	KLF4	0.55205279	1.38E-15
DLST	RAI1	0.55177379	1.43E-15
DLST	SPOP	0.55174938	1.44E-15
DLST	PANK2	0.55174555	1.44E-15
DLST	PLEKHG1	0.55172028	1.44E-15
DLST	LMO4	0.55167594	1.45E-15
DLST	UBE2Q1	0.55149722	1.49E-15
DLST	PTPRK	0.55122009	1.55E-15
DLST	SRD5A1	0.551129	1.57E-15
DLST	HMCES	0.55099112	1.6E-15
DLST	RAB11FIP5	0.55098984	1.6E-15
DLST	ARAP1	0.55096106	1.61E-15
DLST	FAM83B	0.55090748	1.62E-15
DLST	IDS	0.55083091	1.64E-15
DLST	DLGAP5	0.55050819	1.71E-15
DLST	PCSK6	0.55038947	1.74E-15
DLST	COQ2	0.55036992	1.75E-15
DLST	SUCLG2	0.55036907	1.75E-15
DLST	TMEM30B	0.55034474	1.75E-15

DLST	SEMA4B	0.55031158	1.76E-15
DLST	CDS2	0.55016551	1.8E-15
DLST	KLHL18	0.55012954	1.81E-15
DLST	TAF8	0.55010713	1.81E-15
DLST	HMGCS1	0.55004535	1.83E-15
DLST	TRMT6	0.55002824	1.83E-15
DLST	DCLRE1C	0.5500015	1.84E-15
DLST	KCTD20	0.54995629	1.85E-15
DLST	GLCE	0.5496642	1.93E-15
DLST	TRIB1	0.54966272	1.93E-15
DLST	SERPINB8	0.54949229	1.98E-15
DLST	IPO9	0.54937997	2.01E-15
DLST	OTUD5	0.54922935	2.05E-15
DLST	PCSK5	0.54912255	2.08E-15
DLST	NFIX	0.54908795	2.09E-15
DLST	OTUB1	0.54898038	2.12E-15
DLST	TCF7L2	0.54896885	2.13E-15
DLST	GPI	0.54880489	2.17E-15
DLST	MET	0.54871217	2.2E-15
DLST	COPS4	0.54843972	2.29E-15
DLST	PSMG2	0.54843556	2.29E-15
DLST	DBNDD2	0.5482673	2.34E-15
DLST	DUT	0.54823851	2.35E-15
DLST	SH3GL1	0.54822335	2.36E-15
DLST	KIAA1671	0.54810665	2.4E-15
DLST	PKD2	0.54807059	2.41E-15
DLST	JAK2	0.54802431	2.43E-15
DLST	PDE4D	0.54800338	2.43E-15
DLST	CEBPZOS	0.54797326	2.44E-15
DLST	MRPS5	0.54785283	2.48E-15
DLST	BMP2	0.5478008	2.5E-15
DLST	DIP2C	0.54773443	2.53E-15
DLST	PSMD11	0.54770098	2.54E-15
DLST	CLEC16A	0.54768861	2.54E-15
DLST	NR3C1	0.54763006	2.56E-15
DLST	ZYX	0.54759741	2.57E-15
DLST	TOR1AIP2	0.54755869	2.59E-15
DLST	FARSA	0.54751033	2.61E-15
DLST	AXL	0.54716806	2.73E-15
DLST	NREP	0.54704102	2.78E-15
DLST	CPPED1	0.54695787	2.81E-15
DLST	HSBP1	0.54688887	2.84E-15
DLST	NXT2	0.54688231	2.84E-15
DLST	BMP2K	0.54649021	3E-15
DLST	SH3PXD2A	0.54641076	3.04E-15
DLST	PARVA	0.54625393	3.1E-15
DLST	ATP11C	0.54620116	3.13E-15
DLST	JADE2	0.54615688	3.15E-15
DLST	TSR2	0.54590779	3.26E-15
DLST	DPP3	0.54589928	3.26E-15
DLST	ELOVL5	0.54589511	3.26E-15
DLST	CIT	0.54586174	3.28E-15
DLST	RRAGA	0.54572246	3.34E-15
DLST	CLP1	0.54570792	3.35E-15
DLST	ACACA	0.54560845	3.39E-15
DLST	SEMA4C	0.54548621	3.45E-15
DLST	NR2F2	0.5454561	3.47E-15
DLST	ISCA2	0.54526824	3.56E-15

DLST	LYN	0.54505477	3.66E-15
DLST	ACER3	0.54497716	3.7E-15
DLST	RHPN2	0.54494558	3.72E-15
DLST	PHF19	0.54480341	3.79E-15
DLST	TSFM	0.54463154	3.88E-15
DLST	SHROOM3	0.5446091	3.9E-15
DLST	PCIF1	0.54448918	3.96E-15
DLST	PIGX	0.54448296	3.97E-15
DLST	PLEKHM1	0.54430811	4.06E-15
DLST	ZNF836	0.54421799	4.11E-15
DLST	DSC2	0.5441482	4.15E-15
DLST	KIAA2013	0.54408257	4.19E-15
DLST	LLGL1	0.54404531	4.21E-15
DLST	LMAN2L	0.54404042	4.21E-15
DLST	NDFIP1	0.54362894	4.46E-15
DLST	TRIM34	0.5436204	4.47E-15
DLST	OPTN	0.54361251	4.47E-15
DLST	STK10	0.54355045	4.51E-15
DLST	PREP	0.5434152	4.59E-15
DLST	TGFB1I1	0.5433629	4.63E-15
DLST	SLC35C2	0.5433087	4.66E-15
DLST	RAC1	0.5432552	4.7E-15
DLST	PIP4K2A	0.54320885	4.73E-15
DLST	TRAPPC2B	0.54312229	4.78E-15
DLST	MYO1E	0.54269602	5.07E-15
DLST	GNB2	0.54266657	5.09E-15
DLST	DOLPP1	0.54264744	5.1E-15
DLST	TRMT12	0.54239275	5.29E-15
DLST	SLC31A1	0.54223348	5.4E-15
DLST	JARID2	0.54214733	5.47E-15
DLST	SEC13	0.54198049	5.59E-15
DLST	NAMPT	0.54196475	5.6E-15
DLST	SLC35B2	0.54194238	5.62E-15
DLST	FRS2	0.54185901	5.69E-15
DLST	IFIH1	0.54179748	5.73E-15
DLST	LIMK2	0.54176581	5.76E-15
DLST	ARMC9	0.5414621	6E-15
DLST	TIMMDC1	0.54141036	6.05E-15
DLST	RNASEL	0.54134298	6.1E-15
DLST	BAK1	0.54123343	6.19E-15
DLST	MBOAT1	0.5411819	6.24E-15
DLST	SIPA1L3	0.54117481	6.24E-15
DLST	VSIR	0.5411455	6.27E-15
DLST	SVIL	0.54106867	6.33E-15
DLST	DNTTIP1	0.54103554	6.36E-15
DLST	SLC35F2	0.54103144	6.37E-15
DLST	DEGS1	0.54102393	6.37E-15
DLST	ZNF281	0.54098045	6.41E-15
DLST	NIPBL	0.5408889	6.49E-15
DLST	MYO18A	0.54087689	6.5E-15
DLST	TOR3A	0.54078762	6.58E-15
DLST	DYNC2LI1	0.54060347	6.75E-15
DLST	NTAN1	0.54060289	6.75E-15
DLST	MED30	0.54060116	6.75E-15
DLST	UNC45A	0.54051221	6.83E-15
DLST	AMPD3	0.54044622	6.9E-15
DLST	PUF60	0.54032869	7.01E-15
DLST	LBR	0.5400789	7.25E-15

DLST	SORL1	0.54003488	7.29E-15
DLST	RCAN3	0.53991762	7.41E-15
DLST	ZNRF1	0.53967416	7.66E-15
DLST	SCO1	0.53966702	7.67E-15
DLST	ATP5MC3	0.53963211	7.7E-15
DLST	DNASE2	0.53955269	7.79E-15
DLST	XAF1	0.53954274	7.8E-15
DLST	PDCL3	0.53952696	7.81E-15
DLST	ITGB3BP	0.53949315	7.85E-15
DLST	LRP1	0.53943109	7.92E-15
DLST	CLK3	0.53938659	7.96E-15
DLST	TMEM159	0.53935898	7.99E-15
DLST	CLN5	0.53935732	8E-15
DLST	ACTR8	0.53926467	8.1E-15
DLST	HDGF	0.53923367	8.13E-15
DLST	WDR61	0.53922716	8.14E-15
DLST	PML	0.53922593	8.14E-15
DLST	TPRG1L	0.53914691	8.23E-15
DLST	PFKFB3	0.53906186	8.32E-15
DLST	ZNF574	0.53903632	8.35E-15
DLST	TTC9	0.53894287	8.46E-15
DLST	ZNF416	0.53880258	8.62E-15
DLST	PYGO2	0.53863782	8.81E-15
DLST	P4HA1	0.53854951	8.92E-15
DLST	RAB27A	0.53839455	9.11E-15
DLST	CARD6	0.53832622	9.19E-15
DLST	CREB3L2	0.53828239	9.25E-15
DLST	STARD4	0.53822935	9.31E-15
DLST	IQSEC1	0.53810456	9.47E-15
DLST	MMP14	0.53809174	9.49E-15
DLST	ZNF277	0.53806689	9.52E-15
DLST	TMEM63B	0.53800659	9.6E-15
DLST	EZR	0.53797301	9.64E-15
DLST	PCGF5	0.537923	9.71E-15
DLST	NUDT19	0.53790455	9.73E-15
DLST	LAMP2	0.53784103	9.82E-15
DLST	PRSS23	0.53778354	9.89E-15
DLST	ZBTB4	0.53777329	9.91E-15
DLST	IL1RAP	0.53753885	1.02E-14
DLST	EPN2	0.53750147	1.03E-14
DLST	STIL	0.53749619	1.03E-14
DLST	MANBAL	0.53745537	1.03E-14
DLST	RBM47	0.53744453	1.04E-14
DLST	ING2	0.53718502	1.07E-14
DLST	PIK3IP1	0.53714118	1.08E-14
DLST	MAP3K20	0.536899	1.11E-14
DLST	MGAT5	0.53681656	1.13E-14
DLST	TP53I11	0.53678583	1.13E-14
DLST	GINS3	0.53669479	1.15E-14
DLST	BUD13	0.53657338	1.16E-14
DLST	PARPBP	0.53650775	1.17E-14
DLST	PER2	0.53634111	1.2E-14
DLST	TTC8	0.53631638	1.21E-14
DLST	SPIRE1	0.53630207	1.21E-14
DLST	PGD	0.53630173	1.21E-14
DLST	BRD7	0.5362964	1.21E-14
DLST	CISD2	0.53602822	1.25E-14
DLST	MRPL15	0.53599684	1.26E-14

DLST	SORBS3	0.53582222	1.29E-14
DLST	COPG1	0.53572486	1.3E-14
DLST	PPCS	0.53558915	1.33E-14
DLST	KDM5A	0.53553834	1.34E-14
DLST	BORA	0.53539364	1.36E-14
DLST	ENY2	0.5352265	1.39E-14
DLST	CFDP1	0.53511437	1.42E-14
DLST	TMUB2	0.53509976	1.42E-14
DLST	RIN2	0.53508227	1.42E-14
DLST	BLOC1S2	0.53493241	1.45E-14
DLST	NOTCH2	0.53488399	1.46E-14
DLST	AP4S1	0.53478474	1.48E-14
DLST	GDI1	0.53466197	1.5E-14
DLST	COPS7A	0.53465851	1.51E-14
DLST	GINS4	0.5346217	1.51E-14
DLST	SRRD	0.53455616	1.53E-14
DLST	LSM1	0.53434109	1.57E-14
DLST	FAM214B	0.53431116	1.58E-14
DLST	SNAPC5	0.5341807	1.6E-14
DLST	VASP	0.5341708	1.61E-14
DLST	HYAL2	0.53405277	1.63E-14
DLST	CAPN1	0.53403721	1.64E-14
DLST	PAIP2	0.53401323	1.64E-14
DLST	PCDH7	0.53400794	1.64E-14
DLST	NCAPD2	0.53397378	1.65E-14
DLST	DNAJA4	0.53395185	1.65E-14
DLST	PLXNA1	0.53384508	1.68E-14
DLST	POLG	0.53363875	1.72E-14
DLST	CDS1	0.53354293	1.75E-14
DLST	LAMTOR5	0.5333832	1.78E-14
DLST	F2R	0.53329294	1.81E-14
DLST	FAM210A	0.53323676	1.82E-14
DLST	NASP	0.53318066	1.83E-14
DLST	TLE3	0.53313888	1.84E-14
DLST	NKAP	0.53313589	1.84E-14
DLST	SAMD4A	0.53310132	1.85E-14
DLST	KLHL12	0.53307094	1.86E-14
DLST	CASP10	0.53303833	1.87E-14
DLST	RUNX1	0.53300854	1.87E-14
DLST	KATNAL1	0.53293944	1.89E-14
DLST	PIP4K2B	0.53293618	1.89E-14
DLST	RRM2	0.53291034	1.9E-14
DLST	XRCC6	0.53285881	1.91E-14
DLST	EPSTI1	0.53281084	1.92E-14
DLST	FBXL17	0.53279507	1.93E-14
DLST	SLC22A3	0.53278405	1.93E-14
DLST	VCAN	0.53275099	1.94E-14
DLST	CDK16	0.53269956	1.95E-14
DLST	GNS	0.53255873	1.99E-14
DLST	ACYP1	0.53249902	2.01E-14
DLST	NR1D2	0.53246234	2.02E-14
DLST	STS	0.53238527	2.04E-14
DLST	ZNF131	0.53237868	2.04E-14
DLST	NAAA	0.53221953	2.08E-14
DLST	TMED4	0.53221085	2.08E-14
DLST	MAPKAP1	0.53219777	2.09E-14
DLST	GON7	0.53219523	2.09E-14
DLST	ALG1	0.53215923	2.1E-14

DLST	PTPRH	0.53212348	2.11E-14
DLST	THAP11	0.5318908	2.17E-14
DLST	AHNAK2	0.53166928	2.24E-14
DLST	RARB	0.53165527	2.24E-14
DLST	APOL2	0.53128538	2.36E-14
DLST	ARL4A	0.53127056	2.36E-14
DLST	PTPRG	0.53110784	2.41E-14
DLST	HIVEP3	0.53107244	2.42E-14
DLST	MLLT3	0.53103283	2.44E-14
DLST	SCAF1	0.53095333	2.46E-14
DLST	SORT1	0.53094841	2.46E-14
DLST	HMMR	0.53090931	2.48E-14
DLST	EXOSC3	0.53086368	2.49E-14
DLST	SURF4	0.53078074	2.52E-14
DLST	CHST11	0.53070487	2.54E-14
DLST	LATS2	0.53061328	2.57E-14
DLST	SLC45A4	0.53060557	2.58E-14
DLST	FAHD1	0.53059302	2.58E-14
DLST	SIPA1L2	0.53059195	2.58E-14
DLST	TGFA	0.53056745	2.59E-14
DLST	VRK3	0.53055991	2.59E-14
DLST	SPAG16	0.53037454	2.66E-14
DLST	VPS72	0.53030105	2.68E-14
DLST	ZC3H3	0.53021291	2.71E-14
DLST	PIK3R1	0.53021079	2.71E-14
DLST	FPGS	0.53012145	2.75E-14
DLST	NCOA6	0.5301152	2.75E-14
DLST	OSMR	0.5301116	2.75E-14
DLST	DOP1B	0.53009988	2.75E-14
DLST	DOK4	0.53000924	2.79E-14
DLST	TSNAX	0.52994126	2.81E-14
DLST	AP1S1	0.52971999	2.9E-14
DLST	TP53INP2	0.5296037	2.94E-14
DLST	NMT1	0.5295894	2.95E-14
DLST	NOL7	0.52958424	2.95E-14
DLST	MAZ	0.52956951	2.95E-14
DLST	PORCN	0.5294112	3.02E-14
DLST	PHB	0.5293516	3.04E-14
DLST	CALD1	0.52928831	3.06E-14
DLST	TNFAIP8L1	0.52927875	3.07E-14
DLST	ETNK1	0.52924543	3.08E-14
DLST	SAMD9L	0.52922829	3.09E-14
DLST	COMMD5	0.52907235	3.15E-14
DLST	RPGRIP1L	0.52904699	3.16E-14
DLST	HDAC8	0.52903622	3.17E-14
DLST	BZW2	0.52874203	3.29E-14
DLST	MYL12B	0.52864939	3.33E-14
DLST	CD59	0.52852862	3.39E-14
DLST	IGSF3	0.5284642	3.41E-14
DLST	PRAG1	0.52838625	3.45E-14
DLST	RPA2	0.52830454	3.49E-14
DLST	CYTH1	0.52799713	3.63E-14
DLST	NABP2	0.52781511	3.72E-14
DLST	ANXA1	0.52774021	3.75E-14
DLST	HIP1	0.52753512	3.85E-14
DLST	SPRY2	0.52741328	3.92E-14
DLST	MED29	0.52735034	3.95E-14
DLST	B3GNT9	0.52725225	4E-14

DLST	ELP6	0.52722798	4.01E-14
DLST	SLC25A44	0.52721793	4.02E-14
DLST	FRMD6	0.52720396	4.02E-14
DLST	TMEM179B	0.52717559	4.04E-14
DLST	EPDR1	0.52710215	4.08E-14
DLST	KDM6B	0.5270934	4.08E-14
DLST	RINL	0.52707887	4.09E-14
DLST	GOLPH3L	0.52690127	4.19E-14
DLST	NACA	0.52688517	4.2E-14
DLST	ARL8A	0.52668609	4.31E-14
DLST	IRAK2	0.5266855	4.31E-14
DLST	KITLG	0.52661778	4.34E-14
DLST	EPB41L2	0.52628949	4.53E-14
DLST	TOE1	0.52628527	4.54E-14
DLST	TCEAL9	0.5262197	4.57E-14
DLST	SLC44A2	0.52619287	4.59E-14
DLST	KIRREL1	0.52618248	4.6E-14
DLST	SPINDOC	0.52611263	4.64E-14
DLST	KDELR1	0.52596663	4.73E-14
DLST	CHST14	0.52591656	4.76E-14
DLST	OAZ2	0.52588814	4.78E-14
DLST	HK2	0.52582544	4.81E-14
DLST	LIPA	0.52567881	4.91E-14
DLST	LAMTOR1	0.52562594	4.94E-14
DLST	AJUBA	0.52559831	4.96E-14
DLST	CUX1	0.52557369	4.97E-14
DLST	SLC37A1	0.52552105	5.01E-14
DLST	SAP18	0.52541829	5.08E-14
DLST	ZNF564	0.52541316	5.08E-14
DLST	COA4	0.5253416	5.13E-14
DLST	BMP1	0.52517508	5.24E-14
DLST	NDUFS2	0.52506408	5.31E-14
DLST	TMED9	0.52489985	5.43E-14
DLST	PLCB3	0.52485275	5.46E-14
DLST	POLR2E	0.52484233	5.47E-14
DLST	MR1	0.52479382	5.5E-14
DLST	TRIB2	0.52473423	5.55E-14
DLST	WBP1L	0.52463498	5.62E-14
DLST	PSMC3	0.5243382	5.84E-14
DLST	MAPKAPK2	0.5241972	5.94E-14
DLST	CBX5	0.52407247	6.04E-14
DLST	PIK3R4	0.52405791	6.05E-14
DLST	PLIN3	0.52395708	6.13E-14
DLST	HSDL1	0.52394701	6.14E-14
DLST	PRUNE1	0.5238408	6.22E-14
DLST	TIMM23	0.52376882	6.28E-14
DLST	GALNT5	0.52337617	6.61E-14
DLST	DBN1	0.52335599	6.62E-14
DLST	MITF	0.52332866	6.65E-14
DLST	MRPS28	0.52331561	6.66E-14
DLST	PERP	0.52324911	6.72E-14
DLST	FSTL1	0.52321019	6.75E-14
DLST	STK16	0.52319009	6.77E-14
DLST	TRIM8	0.52318176	6.77E-14
DLST	ESCO1	0.52315567	6.8E-14
DLST	PLA2R1	0.52310935	6.84E-14
DLST	CACYBP	0.52302071	6.92E-14
DLST	BCR	0.52301466	6.92E-14

DLST	TIGD2	0.52294725	6.98E-14
DLST	BTN3A1	0.52280596	7.11E-14
DLST	LPCAT2	0.52259966	7.3E-14
DLST	CORO2A	0.5223016	7.59E-14
DLST	GTF2A2	0.52229201	7.59E-14
DLST	PAFAH1B1	0.52227507	7.61E-14
DLST	CHMP7	0.522186	7.7E-14
DLST	SEMA3C	0.52216679	7.72E-14
DLST	YIPF4	0.52186137	8.03E-14
DLST	EHF	0.52184656	8.04E-14
DLST	OAS3	0.52171762	8.17E-14
DLST	TLE4	0.52171588	8.18E-14
DLST	NAALADL2	0.52167687	8.22E-14
DLST	NAP1L1	0.52167321	8.22E-14
DLST	PIGBOS1	0.52159523	8.3E-14
DLST	MICAL2	0.52157438	8.33E-14
DLST	DUSP18	0.52141746	8.49E-14
DLST	TMEM59	0.52139651	8.52E-14
DLST	PIK3C2B	0.52134292	8.58E-14
DLST	ADGRG6	0.52126866	8.66E-14
DLST	DUS2	0.52114112	8.8E-14
DLST	B4GALT4	0.5210764	8.87E-14
DLST	MED18	0.52106331	8.89E-14
DLST	GABARAPL2	0.52086495	9.12E-14
DLST	POLR3C	0.52067905	9.33E-14
DLST	RUSC1	0.5206292	9.39E-14
DLST	PRKCA	0.52057109	9.46E-14
DLST	ABHD17B	0.52056589	9.47E-14
DLST	MFHAS1	0.52055043	9.49E-14
DLST	BLOC1S4	0.52048592	9.57E-14
DLST	NELFB	0.52042753	9.64E-14
DLST	ZBTB22	0.52035054	9.73E-14
DLST	TIAM2	0.52032838	9.76E-14
DLST	ZNF362	0.52025726	9.85E-14
DLST	DEPTOR	0.52021021	9.91E-14
DLST	RAD51AP1	0.52008046	1.01E-13
DLST	ZC3HC1	0.51989313	1.03E-13
DLST	L3MBTL2	0.51979246	1.05E-13
DLST	SPAG1	0.51962953	1.07E-13
DLST	ABL2	0.51960091	1.07E-13
DLST	FAM210B	0.51957844	1.07E-13
DLST	NUMBL	0.51950434	1.08E-13
DLST	MOSPD1	0.51949689	1.09E-13
DLST	CD68	0.51934532	1.11E-13
DLST	DNMT1	0.51924967	1.12E-13
DLST	CEP89	0.51882786	1.18E-13
DLST	MTMR11	0.51880753	1.18E-13
DLST	ATP2B1	0.51869028	1.2E-13
DLST	AKAP1	0.51864927	1.21E-13
DLST	HSPA4	0.51862637	1.21E-13
DLST	PXDC1	0.51854625	1.22E-13
DLST	MEGF9	0.51837601	1.25E-13
DLST	PARD3B	0.51834122	1.26E-13
DLST	TSPYL1	0.51821651	1.28E-13
DLST	FBXO5	0.51819906	1.28E-13
DLST	CASP6	0.51806686	1.3E-13
DLST	KLC1	0.51788289	1.33E-13
DLST	EDEM2	0.51786114	1.34E-13

DLST	RRP36	0.51782636	1.34E-13
DLST	GCLM	0.51772516	1.36E-13
DLST	SSBP1	0.51765664	1.37E-13
DLST	C1orf43	0.51758209	1.38E-13
DLST	C15orf39	0.51751529	1.39E-13
DLST	RABGAP1L	0.51744454	1.41E-13
DLST	CHMP4A	0.51740631	1.41E-13
DLST	RPIA	0.51734451	1.43E-13
DLST	F11R	0.5173024	1.43E-13
DLST	NUTF2	0.51724052	1.44E-13
DLST	TRIM59	0.51714617	1.46E-13
DLST	SP140L	0.51705257	1.48E-13
DLST	BICD2	0.51700606	1.49E-13
DLST	CDC37	0.51697838	1.49E-13
DLST	EPB41L4A	0.51687685	1.51E-13
DLST	SKA2	0.51661295	1.56E-13
DLST	UACA	0.51659466	1.57E-13
DLST	PLEKHG5	0.5165443	1.58E-13
DLST	CTNNBIP1	0.51654428	1.58E-13
DLST	NEK1	0.51642791	1.6E-13
DLST	NDUFA5	0.51637937	1.61E-13
DLST	SELENON	0.51634402	1.62E-13
DLST	SARNP	0.51621484	1.64E-13
DLST	ITGA3	0.5162116	1.64E-13
DLST	DNAJB12	0.51613458	1.66E-13
DLST	TRAPPC4	0.51612893	1.66E-13
DLST	GSN	0.51612227	1.66E-13
DLST	C11orf68	0.51602689	1.68E-13
DLST	RNF135	0.51601816	1.68E-13
DLST	IRF1	0.51583227	1.72E-13
DLST	TP53RK	0.5156757	1.76E-13
DLST	DNAJA1	0.51558062	1.78E-13
DLST	LAMA4	0.51536486	1.83E-13
DLST	FHL2	0.51529582	1.84E-13
DLST	SNX29	0.51519371	1.87E-13
DLST	ZWINT	0.51519319	1.87E-13
DLST	SF3B4	0.51493594	1.93E-13
DLST	VPS25	0.51468012	1.99E-13
DLST	LYRM2	0.51466507	2E-13
DLST	YBX1	0.51456897	2.02E-13
DLST	NUDT3	0.51455409	2.02E-13
DLST	IFIT5	0.51450484	2.04E-13
DLST	NELFCD	0.51446805	2.05E-13
DLST	AURKA	0.51429786	2.09E-13
DLST	PCDHB10	0.51421621	2.11E-13
DLST	MRPS30	0.51413816	2.13E-13
DLST	PITHD1	0.51407113	2.15E-13
DLST	ZEB1	0.51407044	2.15E-13
DLST	TBRG4	0.51399358	2.17E-13
DLST	KIF23	0.51394678	2.18E-13
DLST	CKAP2L	0.51389174	2.2E-13
DLST	EML2	0.51377413	2.23E-13
DLST	FRG1	0.51369827	2.25E-13
DLST	MOB3B	0.51368118	2.26E-13
DLST	SULF2	0.5135797	2.29E-13
DLST	AP1B1	0.51355266	2.29E-13
DLST	ZNF512	0.51324299	2.38E-13
DLST	CAPNS1	0.51306327	2.44E-13

DLST	COG4	0.51302315	2.45E-13
DLST	SEH1L	0.51288643	2.49E-13
DLST	IFNGR2	0.51275491	2.53E-13
DLST	EIF5A2	0.51258249	2.59E-13
DLST	TUBA4A	0.51253802	2.6E-13
DLST	VPS18	0.51243958	2.63E-13
DLST	SLC35A2	0.51238239	2.65E-13
DLST	SH2B3	0.51229086	2.68E-13
DLST	NBL1	0.51212645	2.74E-13
DLST	SNAPIN	0.51180962	2.85E-13
DLST	TIGAR	0.51154943	2.94E-13
DLST	GMIP	0.51149341	2.96E-13
DLST	TTI2	0.51139009	3E-13
DLST	ZNF846	0.51130326	3.03E-13
DLST	MED19	0.51113717	3.09E-13
DLST	PSMB5	0.51112558	3.1E-13
DLST	FTSJ3	0.51109801	3.11E-13
DLST	KIF20A	0.51108108	3.12E-13
DLST	CRTAP	0.51106379	3.12E-13
DLST	PRNP	0.51098681	3.15E-13
DLST	GNG10	0.51098011	3.16E-13
DLST	SOGA1	0.51096363	3.16E-13
DLST	GPX8	0.51084242	3.21E-13
DLST	CHCHD7	0.51082595	3.22E-13
DLST	SHISA5	0.5107906	3.23E-13
DLST	TRNAU1AP	0.51075944	3.24E-13
DLST	TSC22D1	0.51057231	3.32E-13
DLST	FAAP24	0.51048818	3.35E-13
DLST	TWF2	0.51045955	3.36E-13
DLST	DSN1	0.51044472	3.37E-13
DLST	AKAP12	0.51031586	3.42E-13
DLST	ARHGAP26	0.51014196	3.5E-13
DLST	CD55	0.51006179	3.53E-13
DLST	NSMCE3	0.50984785	3.63E-13
DLST	RAB15	0.50983447	3.63E-13
DLST	GFOD2	0.50982631	3.64E-13
DLST	IL4R	0.50981688	3.64E-13
DLST	ZFYVE19	0.50981326	3.64E-13
DLST	PRXL2B	0.50978447	3.66E-13
DLST	SNX11	0.50970271	3.69E-13
DLST	LGMN	0.50951073	3.78E-13
DLST	QKI	0.50940262	3.83E-13
DLST	COTL1	0.50937475	3.85E-13
DLST	ANLN	0.50936994	3.85E-13
DLST	CXCL16	0.50931381	3.87E-13
DLST	SUPT4H1	0.50931084	3.88E-13
DLST	RAB31	0.50928054	3.89E-13
DLST	PRKD3	0.50907453	3.99E-13
DLST	LTBR	0.50906568	3.99E-13
DLST	CEBPZ	0.50900167	4.03E-13
DLST	TRAPPC3	0.50892242	4.07E-13
DLST	UQCRC1	0.50890344	4.07E-13
DLST	RBM8A	0.50888746	4.08E-13
DLST	OSBPL1A	0.50862129	4.22E-13
DLST	C1orf112	0.50851896	4.27E-13
DLST	NCEH1	0.50846603	4.3E-13
DLST	FAM200A	0.50844493	4.31E-13
DLST	SQLE	0.50838486	4.34E-13

DLST	CHD8	0.50832218	4.38E-13
DLST	RUVBL1	0.50826175	4.41E-13
DLST	EPHA4	0.50821784	4.43E-13
DLST	ABCG1	0.50817626	4.45E-13
DLST	DRAM1	0.50816755	4.46E-13
DLST	JMJD8	0.50810341	4.49E-13
DLST	IGFBP5	0.50807486	4.51E-13
DLST	INPP1	0.50796003	4.57E-13
DLST	CSE1L	0.50789018	4.61E-13
DLST	LAS1L	0.50771863	4.71E-13
DLST	ATP6V1G1	0.50768019	4.73E-13
DLST	TBC1D10A	0.50759961	4.78E-13
DLST	APOL1	0.50759338	4.78E-13
DLST	SLC7A7	0.50758029	4.79E-13
DLST	SLC12A9	0.50754676	4.81E-13
DLST	AP2A1	0.5075331	4.82E-13
DLST	MYL12A	0.50752948	4.82E-13
DLST	RHBDF2	0.50732593	4.94E-13
DLST	SP110	0.50721166	5.01E-13
DLST	TCEAL8	0.50710125	5.08E-13
DLST	CCDC68	0.50689978	5.21E-13
DLST	CHD1L	0.50684549	5.24E-13
DLST	HSPA9	0.50681803	5.26E-13
DLST	PTPA	0.50680498	5.27E-13
DLST	STAMBPL1	0.50665539	5.36E-13
DLST	ASNSD1	0.50653028	5.45E-13
DLST	MARK4	0.50650848	5.46E-13
DLST	CCNA2	0.50649206	5.47E-13
DLST	CCDC51	0.50627944	5.62E-13
DLST	PLK4	0.50626567	5.63E-13
DLST	MAP1LC3B2	0.50618944	5.68E-13
DLST	GASK1B	0.50602465	5.79E-13
DLST	EXOSC7	0.5059441	5.85E-13
DLST	MACC1	0.50581753	5.94E-13
DLST	ECHDC1	0.50579854	5.95E-13
DLST	CIAO2A	0.50579176	5.96E-13
DLST	RABIF	0.50578698	5.96E-13
DLST	LARGE1	0.50565761	6.06E-13
DLST	LYPLA2	0.50553912	6.14E-13
DLST	FARS2	0.50552484	6.16E-13
DLST	CLCF1	0.50542885	6.23E-13
DLST	TRMT61B	0.50527103	6.35E-13
DLST	DTX4	0.50522525	6.38E-13
DLST	TAGLN2	0.50515568	6.44E-13
DLST	CTDSPL	0.50507303	6.5E-13
DLST	PDLIM7	0.50501673	6.55E-13
DLST	FRY	0.50490223	6.64E-13
DLST	CYP51A1	0.50481975	6.7E-13
DLST	F2RL1	0.50481301	6.71E-13
DLST	ELOVL6	0.50472552	6.78E-13
DLST	DYNLT1	0.50472214	6.78E-13
DLST	CRYBG1	0.5046614	6.83E-13
DLST	ECH1	0.50464946	6.84E-13
DLST	SPSB1	0.50462347	6.87E-13
DLST	THG1L	0.50454964	6.93E-13
DLST	MAF	0.50449004	6.98E-13
DLST	HSPE1-MOB4	0.50443492	7.02E-13
DLST	ATP6V1B2	0.50435642	7.09E-13

DLST	MBNL3	0.50417442	7.25E-13
DLST	MID1	0.50413232	7.29E-13
DLST	SOX4	0.50409929	7.32E-13
DLST	TRIM28	0.50381198	7.57E-13
DLST	C12orf65	0.50379279	7.59E-13
DLST	KIF11	0.50362966	7.74E-13
DLST	ZNF185	0.50354285	7.82E-13
DLST	ARL15	0.50349801	7.87E-13
DLST	METTL2A	0.50345054	7.91E-13
DLST	VMA21	0.50337768	7.98E-13
DLST	YARS2	0.50333777	8.02E-13
DLST	RIPK2	0.50328928	8.07E-13
DLST	KIAA1217	0.50327367	8.08E-13
DLST	COL5A1	0.50323709	8.12E-13
DLST	TCEAL4	0.50314148	8.21E-13
DLST	ACTB	0.50297307	8.38E-13
DLST	DAPK3	0.50290025	8.45E-13
DLST	NT5E	0.50283382	8.52E-13
DLST	BUB1B	0.50275671	8.6E-13
DLST	UBE2L3	0.50272672	8.63E-13
DLST	MYL6	0.50262719	8.74E-13
DLST	STMN1	0.50242419	8.95E-13
DLST	STN1	0.50230734	9.08E-13
DLST	MKLN1	0.50220069	9.2E-13
DLST	HSPB11	0.50219026	9.21E-13
DLST	NABP1	0.50215505	9.25E-13
DLST	BLOC1S3	0.50176125	9.69E-13
DLST	YIPF1	0.50164434	9.83E-13
DLST	TAP2	0.50153865	9.96E-13
DLST	PCGF1	0.50148562	1E-12
DLST	YOD1	0.5014717	1E-12
DLST	ADCY3	0.50146613	1E-12
DLST	MCC	0.50146071	1E-12
DLST	ENC1	0.50130782	1.02E-12
DLST	MCUB	0.50114693	1.04E-12
DLST	ELOC	0.50111778	1.05E-12
DLST	CKLF-CMTM1	0.50109704	1.05E-12
DLST	ST3GAL2	0.50098604	1.06E-12
DLST	MAP2K3	0.50095505	1.07E-12
DLST	ALDH3B1	0.50080607	1.09E-12
DLST	MXD1	0.50055695	1.12E-12
DLST	CEMIP2	0.50039586	1.14E-12
DLST	TTC22	0.50038864	1.14E-12
DLST	DCUN1D3	0.50032281	1.15E-12
DLST	MTHFD1L	0.50024022	1.16E-12
DLST	CDH1	0.50019047	1.17E-12
DLST	GSR	0.50017024	1.17E-12
DLST	CASP7	0.50012858	1.18E-12
DLST	CYBRD1	0.50010339	1.18E-12
FDX1	MARK2	0.50002172	1.19E-12
FDX1	DHCR7	0.50008874	1.18E-12
FDX1	GFPT1	0.50017054	1.17E-12
FDX1	MBD5	0.50031404	1.15E-12
FDX1	TCEAL9	0.50044128	1.14E-12
FDX1	CSNK1G3	0.50046584	1.13E-12
FDX1	G3BP1	0.50065875	1.11E-12
FDX1	EIF4E	0.50067395	1.1E-12
FDX1	LASP1	0.50069305	1.1E-12

FDX1	GALC	0.50077026	1.09E-12
FDX1	MREG	0.50080758	1.09E-12
FDX1	MCL1	0.50089336	1.08E-12
FDX1	ARPC2	0.50113854	1.04E-12
FDX1	GLO1	0.5013769	1.02E-12
FDX1	PCYT1A	0.50145932	1.01E-12
FDX1	MTX2	0.50156742	9.92E-13
FDX1	ACTR3	0.50163751	9.84E-13
FDX1	LIMA1	0.50167137	9.8E-13
FDX1	SINHCAF	0.50167253	9.8E-13
FDX1	TRAF3IP1	0.50170519	9.76E-13
FDX1	XPR1	0.50181094	9.64E-13
FDX1	PPP1R21	0.50225251	9.14E-13
FDX1	PBDC1	0.50234548	9.04E-13
FDX1	BTBD1	0.50235609	9.03E-13
FDX1	PLEKHA6	0.50241037	8.97E-13
FDX1	ZFYVE21	0.50244067	8.93E-13
FDX1	VPS41	0.50256549	8.8E-13
FDX1	UBE2D3	0.50259958	8.76E-13
FDX1	TSPAN3	0.50263109	8.73E-13
FDX1	MTERF3	0.5028449	8.51E-13
FDX1	ARV1	0.50287592	8.48E-13
FDX1	YWHAG	0.50306389	8.29E-13
FDX1	SNW1	0.50313078	8.22E-13
FDX1	EPS8	0.50317366	8.18E-13
FDX1	LEPROTL1	0.5032609	8.09E-13
FDX1	PKP4	0.50339835	7.96E-13
FDX1	CCNK	0.50345881	7.9E-13
FDX1	FBXO8	0.50348918	7.87E-13
FDX1	YY1AP1	0.50365768	7.72E-13
FDX1	ITGB1	0.50373446	7.64E-13
FDX1	DIS3	0.50377011	7.61E-13
FDX1	LRRC1	0.50377087	7.61E-13
FDX1	MED21	0.50403355	7.37E-13
FDX1	VTA1	0.5040812	7.33E-13
FDX1	TCAIM	0.50429336	7.15E-13
FDX1	NKRF	0.50438379	7.07E-13
FDX1	ADAM10	0.50450249	6.97E-13
FDX1	TOR1AIP1	0.5045566	6.92E-13
FDX1	CCDC6	0.50459313	6.89E-13
FDX1	EIF1AX	0.5046871	6.81E-13
FDX1	HPRT1	0.50469113	6.81E-13
FDX1	DHCR24	0.50486597	6.67E-13
FDX1	MAP3K2	0.50503489	6.53E-13
FDX1	CTTN	0.50525261	6.36E-13
FDX1	BAZ1A	0.50527361	6.35E-13
FDX1	CORO2A	0.50530773	6.32E-13
FDX1	EXOC1	0.50537061	6.27E-13
FDX1	SVIP	0.50539672	6.25E-13
FDX1	SKP1	0.50549202	6.18E-13
FDX1	IAH1	0.50556387	6.13E-13
FDX1	ETF1	0.50568166	6.04E-13
FDX1	SERPINB8	0.50571084	6.02E-13
FDX1	CIAPIN1	0.50582791	5.93E-13
FDX1	SEC23IP	0.50589463	5.88E-13
FDX1	DLD	0.5059252	5.86E-13
FDX1	RBMS1	0.50600986	5.8E-13
FDX1	TMEM167B	0.50616532	5.69E-13

FDX1	NIF3L1	0.50618184	5.68E-13
FDX1	SLC30A6	0.5063143	5.59E-13
FDX1	TRIOBP	0.50631955	5.59E-13
FDX1	PAK2	0.50640988	5.53E-13
FDX1	KDM2A	0.50677112	5.29E-13
FDX1	MYD88	0.50678035	5.28E-13
FDX1	TUT7	0.50691141	5.2E-13
FDX1	GPD2	0.50707864	5.09E-13
FDX1	SLC39A1	0.50709291	5.09E-13
FDX1	EIF1AD	0.50713642	5.06E-13
FDX1	RAB2A	0.50713681	5.06E-13
FDX1	SAR1B	0.50719335	5.02E-13
FDX1	MMAA	0.50731683	4.95E-13
FDX1	LYPLA1	0.5076806	4.73E-13
FDX1	HMGCR	0.50780201	4.66E-13
FDX1	PLEKHA7	0.50799374	4.56E-13
FDX1	ABTB2	0.50805691	4.52E-13
FDX1	RPAP3	0.50811076	4.49E-13
FDX1	YTHDF2	0.50813665	4.48E-13
FDX1	PPP4R2	0.50819464	4.44E-13
FDX1	RNF121	0.50822381	4.43E-13
FDX1	MATR3	0.50835624	4.36E-13
FDX1	TMEM33	0.5085901	4.23E-13
FDX1	ABCB10	0.50865889	4.2E-13
FDX1	YTHDF3	0.50868767	4.18E-13
FDX1	BFAR	0.50876852	4.14E-13
FDX1	TIMM23	0.5089165	4.07E-13
FDX1	MRPL42	0.50896113	4.05E-13
FDX1	ATP5PB	0.5090477	4E-13
FDX1	DDX21	0.50910781	3.97E-13
FDX1	DHX36	0.50913719	3.96E-13
FDX1	RNF5	0.50915828	3.95E-13
FDX1	EIF2AK2	0.50930728	3.88E-13
FDX1	KLHL20	0.50933161	3.87E-13
FDX1	WDR44	0.50938707	3.84E-13
FDX1	PDZD8	0.50972555	3.68E-13
FDX1	FEM1C	0.50990041	3.6E-13
FDX1	PPP2CB	0.50991479	3.6E-13
FDX1	EXT2	0.51014511	3.5E-13
FDX1	PDP1	0.5103047	3.43E-13
FDX1	JAG1	0.51030772	3.43E-13
FDX1	SNX12	0.51033816	3.42E-13
FDX1	DGKH	0.51042965	3.38E-13
FDX1	BMPR2	0.51043713	3.37E-13
FDX1	SP1	0.51062397	3.3E-13
FDX1	UQCRC2	0.51062933	3.29E-13
FDX1	CPT1A	0.51074626	3.25E-13
FDX1	DHRS7	0.51082757	3.22E-13
FDX1	NCL	0.51086532	3.2E-13
FDX1	FBXO38	0.51091922	3.18E-13
FDX1	CDC123	0.51100518	3.15E-13
FDX1	LIN9	0.51111689	3.1E-13
FDX1	TIMM17A	0.51116275	3.08E-13
FDX1	LAPTM4B	0.5112056	3.07E-13
FDX1	ROCK2	0.51124271	3.05E-13
FDX1	RMDN1	0.51125458	3.05E-13
FDX1	UGCG	0.51127256	3.04E-13
FDX1	PCGF5	0.51143925	2.98E-13

FDX1	SMIM15	0.51144416	2.98E-13
FDX1	KLF3	0.51150362	2.96E-13
FDX1	SUCLG2	0.51151539	2.95E-13
FDX1	KIF13B	0.51154862	2.94E-13
FDX1	HSD17B12	0.5115504	2.94E-13
FDX1	UBE2K	0.51173083	2.88E-13
FDX1	CD2AP	0.51214851	2.73E-13
FDX1	TAOK3	0.51227583	2.69E-13
FDX1	BMI1	0.51238483	2.65E-13
FDX1	RAB14	0.51242247	2.64E-13
FDX1	MPZL1	0.51244952	2.63E-13
FDX1	PDHX	0.51248239	2.62E-13
FDX1	CTBP2	0.5125115	2.61E-13
FDX1	MYO6	0.51268607	2.55E-13
FDX1	WAC	0.51277198	2.53E-13
FDX1	NRAS	0.5128672	2.5E-13
FDX1	PGM2L1	0.51296067	2.47E-13
FDX1	SPCS2	0.51300441	2.45E-13
FDX1	CREB1	0.51301538	2.45E-13
FDX1	RMDN2	0.51305317	2.44E-13
FDX1	ATP11A	0.51312071	2.42E-13
FDX1	RBBP5	0.51327705	2.37E-13
FDX1	CD164	0.51348775	2.31E-13
FDX1	DLG1	0.51355816	2.29E-13
FDX1	TMEM50A	0.51361894	2.27E-13
FDX1	INPP4B	0.51374043	2.24E-13
FDX1	TMEM135	0.51377837	2.23E-13
FDX1	NAA50	0.51385909	2.21E-13
FDX1	KCTD1	0.51402359	2.16E-13
FDX1	ANAPC10	0.51439376	2.06E-13
FDX1	TES	0.51440327	2.06E-13
FDX1	MRPL3	0.51450157	2.04E-13
FDX1	CDC42SE1	0.51451569	2.03E-13
FDX1	C16orf87	0.51458135	2.02E-13
FDX1	NIPSNAP3A	0.51459456	2.01E-13
FDX1	PA2G4	0.51461264	2.01E-13
FDX1	SLK	0.51463729	2E-13
FDX1	ATP11B	0.51475259	1.97E-13
FDX1	RBM7	0.51501064	1.91E-13
FDX1	KRCC1	0.51515729	1.88E-13
FDX1	TSC22D2	0.51517342	1.87E-13
FDX1	PTPRH	0.51522988	1.86E-13
FDX1	ADI1	0.51533781	1.83E-13
FDX1	DR1	0.51537279	1.83E-13
FDX1	ATG5	0.5154087	1.82E-13
FDX1	RNF128	0.51547315	1.8E-13
FDX1	SLC35A3	0.51548459	1.8E-13
FDX1	ANKRD42	0.51551195	1.79E-13
FDX1	CLNS1A	0.51606332	1.67E-13
FDX1	RRN3	0.51612694	1.66E-13
FDX1	CNIH1	0.51619	1.65E-13
FDX1	PDCD6IP	0.51623843	1.64E-13
FDX1	ATL3	0.51623904	1.64E-13
FDX1	ARL6IP6	0.51628617	1.63E-13
FDX1	PARP4	0.51638127	1.61E-13
FDX1	CSNK1A1	0.51646233	1.59E-13
FDX1	ATF2	0.51646384	1.59E-13
FDX1	PGM2	0.51649321	1.59E-13

FDX1	FAM120A	0.51655105	1.57E-13
FDX1	AP1S3	0.51665024	1.56E-13
FDX1	C3orf38	0.51667322	1.55E-13
FDX1	CYB5B	0.51677118	1.53E-13
FDX1	COPA	0.51690556	1.51E-13
FDX1	MCMBP	0.51694601	1.5E-13
FDX1	RAB7A	0.51698322	1.49E-13
FDX1	RTN3	0.51706236	1.48E-13
FDX1	RETSAT	0.51707949	1.47E-13
FDX1	C16orf72	0.51710391	1.47E-13
FDX1	ZFP91	0.51718446	1.45E-13
FDX1	AIDA	0.51777479	1.35E-13
FDX1	SMNDC1	0.51784065	1.34E-13
FDX1	CAPZA2	0.51787386	1.33E-13
FDX1	HSD17B11	0.51789036	1.33E-13
FDX1	WAPL	0.51792528	1.32E-13
FDX1	ANP32A	0.51803471	1.31E-13
FDX1	ZCCHC10	0.51841354	1.24E-13
FDX1	WASL	0.51852782	1.23E-13
FDX1	COMMD2	0.51854314	1.22E-13
FDX1	MTPN	0.51858402	1.22E-13
FDX1	PNP	0.51872015	1.2E-13
FDX1	NIPA1	0.51873311	1.2E-13
FDX1	RNF7	0.5188467	1.18E-13
FDX1	OCIAD1	0.5189033	1.17E-13
FDX1	TRIP4	0.51895977	1.16E-13
FDX1	IQGAP1	0.51919756	1.13E-13
FDX1	ATP13A3	0.51938718	1.1E-13
FDX1	ANAPC13	0.51939843	1.1E-13
FDX1	PTPRK	0.51941861	1.1E-13
FDX1	HMGB1	0.51951718	1.08E-13
FDX1	POLR2K	0.51958933	1.07E-13
FDX1	NCBP2	0.51961171	1.07E-13
FDX1	GPBP1L1	0.51961694	1.07E-13
FDX1	TSNAX	0.51965415	1.06E-13
FDX1	FAM177A1	0.51973429	1.05E-13
FDX1	ACSL3	0.51997384	1.02E-13
FDX1	RHOA	0.52003548	1.01E-13
FDX1	NPAT	0.52017775	9.95E-14
FDX1	CNOT8	0.52032886	9.76E-14
FDX1	COPS5	0.52088935	9.09E-14
FDX1	ARMC8	0.52091737	9.05E-14
FDX1	AIMP1	0.52093209	9.04E-14
FDX1	NCK1	0.52104599	8.91E-14
FDX1	KHDRBS1	0.52107701	8.87E-14
FDX1	GOLPH3L	0.52132966	8.59E-14
FDX1	HNRNPLL	0.521621	8.28E-14
FDX1	TPR	0.52166649	8.23E-14
FDX1	LMBRD1	0.5216722	8.22E-14
FDX1	SPOPL	0.52171214	8.18E-14
FDX1	KIF5B	0.5219177	7.97E-14
FDX1	PPM1A	0.52192846	7.96E-14
FDX1	STAM	0.52213859	7.75E-14
FDX1	IMMT	0.52217621	7.71E-14
FDX1	PRTFDC1	0.52218308	7.7E-14
FDX1	UBE2E3	0.52219329	7.69E-14
FDX1	FGD4	0.52222412	7.66E-14
FDX1	TMCC1	0.52227039	7.62E-14

FDX1	HNRNPD	0.52233321	7.55E-14
FDX1	ARL8A	0.5224037	7.49E-14
FDX1	STK24	0.52247281	7.42E-14
FDX1	NUP98	0.52259983	7.3E-14
FDX1	PTPN12	0.52268416	7.22E-14
FDX1	HMGXB4	0.52272005	7.19E-14
FDX1	TMEM30B	0.52276332	7.15E-14
FDX1	COA4	0.52291514	7.01E-14
FDX1	ZDHHC9	0.52302085	6.92E-14
FDX1	CCDC115	0.52311344	6.83E-14
FDX1	DCTD	0.52329881	6.67E-14
FDX1	PIP5K1A	0.52330632	6.67E-14
FDX1	TNPO3	0.52337363	6.61E-14
FDX1	ENSA	0.52347415	6.52E-14
FDX1	ADIPOR1	0.52355013	6.46E-14
FDX1	RBM47	0.52363164	6.39E-14
FDX1	NDUFB3	0.52373326	6.31E-14
FDX1	CAPZA1	0.52375209	6.29E-14
FDX1	KBTBD2	0.52390752	6.17E-14
FDX1	DNAJC13	0.52414765	5.98E-14
FDX1	SRFBP1	0.52430428	5.86E-14
FDX1	TRAM1	0.52440089	5.79E-14
FDX1	ATP6V1A	0.52450158	5.71E-14
FDX1	SELENOT	0.52453186	5.69E-14
FDX1	HHAT	0.52471144	5.56E-14
FDX1	CHRAC1	0.52478607	5.51E-14
FDX1	SPTY2D1	0.52479051	5.51E-14
FDX1	DENND6A	0.52510452	5.29E-14
FDX1	PDE6D	0.52515784	5.25E-14
FDX1	PCNP	0.52521561	5.21E-14
FDX1	WIPF2	0.52522084	5.21E-14
FDX1	B3GNT5	0.52523519	5.2E-14
FDX1	LEO1	0.52528046	5.17E-14
FDX1	TRA2B	0.52535878	5.11E-14
FDX1	PTPN4	0.52536032	5.11E-14
FDX1	UEVLD	0.52561537	4.95E-14
FDX1	VPS29	0.52572928	4.87E-14
FDX1	SMG7	0.52577982	4.84E-14
FDX1	NAB1	0.52579887	4.83E-14
FDX1	VTI1A	0.52629297	4.53E-14
FDX1	ALS2	0.52639617	4.47E-14
FDX1	TMEM106B	0.52655673	4.38E-14
FDX1	STRN	0.52672772	4.28E-14
FDX1	NEK7	0.52685568	4.21E-14
FDX1	SNAPIN	0.52696614	4.15E-14
FDX1	CPNE8	0.52697776	4.14E-14
FDX1	WWP1	0.52702004	4.12E-14
FDX1	PANK3	0.52704046	4.11E-14
FDX1	TOR1AIP2	0.52704743	4.11E-14
FDX1	SRSF7	0.52711289	4.07E-14
FDX1	TRAPPC6B	0.52716284	4.05E-14
FDX1	SLC25A43	0.52718607	4.03E-14
FDX1	PITPNB	0.52731758	3.97E-14
FDX1	TMX2	0.52740955	3.92E-14
FDX1	RTN4	0.52773605	3.75E-14
FDX1	PEX19	0.52785148	3.7E-14
FDX1	SQLE	0.5279194	3.67E-14
FDX1	CWF19L2	0.52810737	3.58E-14

FDX1	PNO1	0.52817453	3.55E-14
FDX1	SETD3	0.52830639	3.48E-14
FDX1	RIT1	0.52844959	3.42E-14
FDX1	LRRC42	0.52851276	3.39E-14
FDX1	OXSR1	0.52855775	3.37E-14
FDX1	PEX2	0.52860477	3.35E-14
FDX1	ANXA5	0.52877871	3.28E-14
FDX1	UBE2W	0.52878766	3.27E-14
FDX1	PSME3	0.52880878	3.26E-14
FDX1	RP2	0.52886438	3.24E-14
FDX1	EHD4	0.52893533	3.21E-14
FDX1	DDX18	0.5289505	3.2E-14
FDX1	NFYA	0.52901014	3.18E-14
FDX1	MFAP1	0.52902692	3.17E-14
FDX1	ETV3	0.52930812	3.06E-14
FDX1	CD46	0.52942189	3.01E-14
FDX1	GORASP2	0.52944836	3E-14
FDX1	ZBTB41	0.52949618	2.98E-14
FDX1	GSPT1	0.52980167	2.86E-14
FDX1	AFTPH	0.52989704	2.83E-14
FDX1	CAPN2	0.5299048	2.83E-14
FDX1	HIF1A	0.52995511	2.81E-14
FDX1	MIER1	0.53037378	2.66E-14
FDX1	GNAQ	0.53051116	2.61E-14
FDX1	MAPK6	0.53052381	2.6E-14
FDX1	OLA1	0.53055687	2.59E-14
FDX1	SUSD1	0.53068026	2.55E-14
FDX1	FNBP1L	0.53075625	2.53E-14
FDX1	CUL2	0.53082753	2.5E-14
FDX1	SPTSSA	0.53115524	2.4E-14
FDX1	KLF6	0.53120545	2.38E-14
FDX1	TNIK	0.53146421	2.3E-14
FDX1	DPY19L1	0.53150368	2.29E-14
FDX1	RNF168	0.53153727	2.28E-14
FDX1	SLC4A1AP	0.5315705	2.27E-14
FDX1	RMND5A	0.53170734	2.23E-14
FDX1	HMGN4	0.531754	2.21E-14
FDX1	GMCL1	0.53176107	2.21E-14
FDX1	MAP2K1	0.53176903	2.21E-14
FDX1	YY1	0.53181633	2.2E-14
FDX1	PRELID3B	0.5319601	2.15E-14
FDX1	NUFIP2	0.5321489	2.1E-14
FDX1	GMFB	0.53217554	2.09E-14
FDX1	PRKCI	0.53218149	2.09E-14
FDX1	CTTNBP2NL	0.53226231	2.07E-14
FDX1	CHMP3	0.53234962	2.05E-14
FDX1	TSEN15	0.5324078	2.03E-14
FDX1	CIR1	0.53247696	2.01E-14
FDX1	KRR1	0.53247766	2.01E-14
FDX1	AP003108.2	0.53249041	2.01E-14
FDX1	ZPR1	0.53259634	1.98E-14
FDX1	MTM1	0.53264838	1.97E-14
FDX1	NDFIP2	0.53265429	1.97E-14
FDX1	KDM5B	0.53271525	1.95E-14
FDX1	RHPN2	0.53277154	1.93E-14
FDX1	CHD9	0.53283228	1.92E-14
FDX1	VRK2	0.53295201	1.89E-14
FDX1	RO60	0.53367695	1.72E-14

FDX1	TADA1	0.53375287	1.7E-14
FDX1	CHCHD3	0.53383006	1.68E-14
FDX1	ABHD2	0.53392657	1.66E-14
FDX1	SRP14	0.53400404	1.64E-14
FDX1	CLINT1	0.53406237	1.63E-14
FDX1	CAMSAP2	0.53407183	1.63E-14
FDX1	STX12	0.53414927	1.61E-14
FDX1	PATL1	0.53416685	1.61E-14
FDX1	SNX1	0.53416688	1.61E-14
FDX1	PNPLA8	0.53418683	1.6E-14
FDX1	DBI	0.53440286	1.56E-14
FDX1	ARF6	0.53454078	1.53E-14
FDX1	CAPRIN1	0.53462533	1.51E-14
FDX1	SH3RF1	0.53464048	1.51E-14
FDX1	MPP5	0.53472057	1.49E-14
FDX1	ATG16L1	0.53488356	1.46E-14
FDX1	ARHGEF12	0.53498971	1.44E-14
FDX1	LRRFIP2	0.53500275	1.44E-14
FDX1	TLK1	0.53525188	1.39E-14
FDX1	ACSL4	0.53527756	1.39E-14
FDX1	C14orf119	0.53533147	1.38E-14
FDX1	PSMD14	0.53542937	1.36E-14
FDX1	C1GALT1C1	0.5356061	1.33E-14
FDX1	ISG20L2	0.53564571	1.32E-14
FDX1	EMC2	0.53575699	1.3E-14
FDX1	YOD1	0.53586453	1.28E-14
FDX1	MPZL3	0.53594967	1.27E-14
FDX1	WDR26	0.53600069	1.26E-14
FDX1	KPNA4	0.53600351	1.26E-14
FDX1	ZNF267	0.53602363	1.25E-14
FDX1	RAB3GAP1	0.53610614	1.24E-14
FDX1	EFCAB14	0.53621709	1.22E-14
FDX1	METTL9	0.53632119	1.2E-14
FDX1	PRRC2C	0.53638675	1.19E-14
FDX1	HACD2	0.53642592	1.19E-14
FDX1	ORC4	0.53670098	1.14E-14
FDX1	VPS35	0.53702893	1.1E-14
FDX1	ATP2C1	0.53706641	1.09E-14
FDX1	ARPP19	0.53743056	1.04E-14
FDX1	STRN3	0.53751164	1.03E-14
FDX1	MRPL19	0.53759756	1.01E-14
FDX1	ANXA7	0.53762512	1.01E-14
FDX1	GGPS1	0.53778052	9.9E-15
FDX1	INPP1	0.5378836	9.76E-15
FDX1	IMPA1	0.53792516	9.71E-15
FDX1	GTPBP4	0.53817663	9.38E-15
FDX1	TXNDC9	0.53819792	9.35E-15
FDX1	JMJD1C	0.53826742	9.27E-15
FDX1	MICU2	0.53835367	9.16E-15
FDX1	PPP2R5E	0.53837888	9.13E-15
FDX1	ELK4	0.53869274	8.75E-15
FDX1	LACTB2	0.53870229	8.74E-15
FDX1	RAB5C	0.53870524	8.73E-15
FDX1	MAGT1	0.53876446	8.66E-15
FDX1	TFG	0.53894017	8.46E-15
FDX1	CTNNA1	0.53897791	8.42E-15
FDX1	CHMP5	0.5390195	8.37E-15
FDX1	TOB2	0.53908281	8.3E-15

FDX1	PTBP3	0.53915215	8.22E-15
FDX1	PLS1	0.53944736	7.9E-15
FDX1	PRPF40A	0.53952603	7.81E-15
FDX1	STX6	0.53953014	7.81E-15
FDX1	QTRT2	0.53960039	7.74E-15
FDX1	LARP4B	0.53967324	7.66E-15
FDX1	MAP3K13	0.53968916	7.64E-15
FDX1	MAT2B	0.53972355	7.61E-15
FDX1	PAK1	0.53979833	7.53E-15
FDX1	RALB	0.53982713	7.5E-15
FDX1	ZDHHC20	0.54003184	7.3E-15
FDX1	RNF2	0.54014175	7.19E-15
FDX1	CXorf38	0.5406869	6.67E-15
FDX1	MAPK1IP1L	0.54098847	6.4E-15
FDX1	ARMCX6	0.54119454	6.23E-15
FDX1	USP6NL	0.54131013	6.13E-15
FDX1	REEP3	0.54145841	6.01E-15
FDX1	USP8	0.54149593	5.98E-15
FDX1	RAB3GAP2	0.54160578	5.89E-15
FDX1	APH1A	0.54177429	5.75E-15
FDX1	ZC3H11A	0.54177891	5.75E-15
FDX1	TBL1XR1	0.54179413	5.74E-15
FDX1	UBXN2B	0.54198947	5.59E-15
FDX1	ARFGEF1	0.5419963	5.58E-15
FDX1	CA13	0.54207557	5.52E-15
FDX1	HIGD1A	0.54218214	5.44E-15
FDX1	WDFY1	0.54223994	5.4E-15
FDX1	FBXO28	0.54224594	5.39E-15
FDX1	ACTR2	0.54228819	5.36E-15
FDX1	SNX6	0.54244155	5.25E-15
FDX1	SELENOF	0.54257953	5.15E-15
FDX1	RAB5A	0.54258228	5.15E-15
FDX1	B3GNT2	0.54273655	5.04E-15
FDX1	CNPPD1	0.54279364	5E-15
FDX1	AHCTF1	0.54294171	4.9E-15
FDX1	SPTLC2	0.54304558	4.83E-15
FDX1	SIAH1	0.54347769	4.55E-15
FDX1	CTCF	0.54379911	4.36E-15
FDX1	CYP51A1	0.54387165	4.31E-15
FDX1	NUMB	0.54417831	4.14E-15
FDX1	STAMBP	0.54448111	3.97E-15
FDX1	BLZF1	0.54481166	3.79E-15
FDX1	RNF111	0.54486162	3.76E-15
FDX1	ANKMY2	0.54491926	3.73E-15
FDX1	UBE3A	0.54495685	3.71E-15
FDX1	MTMR2	0.5450615	3.66E-15
FDX1	TARDBP	0.54525173	3.57E-15
FDX1	CMPK1	0.54527398	3.55E-15
FDX1	C4orf33	0.54539487	3.5E-15
FDX1	AGPS	0.54543315	3.48E-15
FDX1	IARS2	0.54566088	3.37E-15
FDX1	UBE2N	0.54571916	3.34E-15
FDX1	HNRNPC	0.54588218	3.27E-15
FDX1	RAMAC	0.54592596	3.25E-15
FDX1	GCC2	0.54595566	3.23E-15
FDX1	VAMP3	0.54609027	3.17E-15
FDX1	IST1	0.5461634	3.14E-15
FDX1	PLEKHA1	0.54661435	2.95E-15

FDX1	DCTN6	0.54671575	2.91E-15
FDX1	VPS26A	0.5468858	2.84E-15
FDX1	RNF13	0.54693161	2.82E-15
FDX1	DAZAP2	0.54703664	2.78E-15
FDX1	PPP4R3B	0.54755717	2.59E-15
FDX1	DECR1	0.54762191	2.57E-15
FDX1	CCDC32	0.54773533	2.53E-15
FDX1	CBFB	0.5480167	2.43E-15
FDX1	DHX9	0.54804398	2.42E-15
FDX1	TWF1	0.54807631	2.41E-15
FDX1	RABIF	0.54815247	2.38E-15
FDX1	AHR	0.54831711	2.33E-15
FDX1	PPFIA1	0.54844996	2.29E-15
FDX1	ADAM9	0.54859429	2.24E-15
FDX1	PRR13	0.54865676	2.22E-15
FDX1	AMMECR1	0.54876424	2.19E-15
FDX1	ATP6V1D	0.54907039	2.1E-15
FDX1	STAU1	0.54918345	2.06E-15
FDX1	PNRC2	0.54956481	1.96E-15
FDX1	SH3GLB1	0.54963829	1.94E-15
FDX1	GALK2	0.54966729	1.93E-15
FDX1	ELF1	0.54972402	1.91E-15
FDX1	NCOA4	0.54980351	1.89E-15
FDX1	PTPN9	0.54988823	1.87E-15
FDX1	CRCP	0.54997056	1.85E-15
FDX1	CDC42	0.55007961	1.82E-15
FDX1	DCTN5	0.55021587	1.78E-15
FDX1	DEDD	0.55047206	1.72E-15
FDX1	TMEM183A	0.5505426	1.7E-15
FDX1	CPSF6	0.55057044	1.7E-15
FDX1	RMDN3	0.55068703	1.67E-15
FDX1	MPHOSPH10	0.55076387	1.65E-15
FDX1	CYFIP1	0.55078033	1.65E-15
FDX1	RRP15	0.55088757	1.62E-15
FDX1	TSG101	0.55100325	1.6E-15
FDX1	MOB1A	0.55103284	1.59E-15
FDX1	EAPP	0.5510583	1.58E-15
FDX1	BROX	0.55121319	1.55E-15
FDX1	COPS2	0.55167831	1.45E-15
FDX1	EXOC6B	0.55184208	1.42E-15
FDX1	MRPL35	0.55186528	1.41E-15
FDX1	ZNF410	0.55209179	1.37E-15
FDX1	LAPTM4A	0.5524546	1.3E-15
FDX1	SDE2	0.55251477	1.29E-15
FDX1	ZNF207	0.55254057	1.29E-15
FDX1	ATG3	0.55256093	1.28E-15
FDX1	MAP1LC3B	0.55290656	1.22E-15
FDX1	KRAS	0.55313697	1.18E-15
FDX1	TMEM248	0.55342793	1.13E-15
FDX1	CALM2	0.55356482	1.11E-15
FDX1	ZMYM5	0.55379113	1.08E-15
FDX1	UBE2A	0.55381512	1.07E-15
FDX1	GNG12	0.55389438	1.06E-15
FDX1	UGP2	0.5539851	1.05E-15
FDX1	PGRMC1	0.55413611	1.02E-15
FDX1	LAMTOR3	0.55478334	9.34E-16
FDX1	ETFA	0.55506553	8.97E-16
FDX1	SUMO3	0.55530345	8.67E-16

FDX1	YPEL5	0.55534759	8.62E-16
FDX1	SDCBP	0.55569272	8.2E-16
FDX1	CBX3	0.5560101	7.84E-16
FDX1	GFM1	0.55612819	7.7E-16
FDX1	TMOD3	0.55689518	6.9E-16
FDX1	TMEM87A	0.55690731	6.89E-16
FDX1	MFSD14B	0.55705776	6.74E-16
FDX1	TRIP12	0.55717519	6.63E-16
FDX1	HNRNPR	0.55751235	6.31E-16
FDX1	TPM3	0.55802948	5.86E-16
FDX1	TGOLN2	0.55829637	5.64E-16
FDX1	RTF1	0.5583564	5.59E-16
FDX1	GPATCH2	0.558455	5.51E-16
FDX1	OXR1	0.558587	5.41E-16
FDX1	TAF1B	0.55872699	5.3E-16
FDX1	APLP2	0.55874358	5.29E-16
FDX1	HNRNPU	0.55909044	5.03E-16
FDX1	SBDS	0.55918454	4.96E-16
FDX1	MRPL44	0.55957128	4.69E-16
FDX1	DLST	0.55958605	4.68E-16
FDX1	CNOT11	0.55995152	4.44E-16
FDX1	ALAS1	0.56034767	4.19E-16
FDX1	BCL10	0.56042067	4.14E-16
FDX1	FEZ2	0.56043005	4.14E-16
FDX1	TIPRL	0.56045116	4.13E-16
FDX1	MRPL49	0.56052698	4.08E-16
FDX1	ANP32E	0.56062191	4.02E-16
FDX1	SYPL1	0.56082171	3.91E-16
FDX1	SNAP23	0.56085482	3.89E-16
FDX1	ACBD3	0.56107484	3.77E-16
FDX1	PPHLN1	0.56148749	3.55E-16
FDX1	ARHGAP12	0.56185052	3.36E-16
FDX1	YWHAB	0.56227219	3.16E-16
FDX1	ARL6IP1	0.56267486	2.98E-16
FDX1	NCKAP1	0.56280532	2.92E-16
FDX1	TOP1	0.56301714	2.84E-16
FDX1	ARCN1	0.56301926	2.83E-16
FDX1	SHOC2	0.56303519	2.83E-16
FDX1	YWHAZ	0.56320464	2.76E-16
FDX1	UBA3	0.5633725	2.69E-16
FDX1	PIGC	0.56362808	2.59E-16
FDX1	CWC22	0.56373092	2.55E-16
FDX1	RAB10	0.56420879	2.38E-16
FDX1	CCDC90B	0.56424038	2.37E-16
FDX1	SP3	0.56437261	2.32E-16
FDX1	TIPARP	0.56444034	2.3E-16
FDX1	RAB18	0.56449727	2.28E-16
FDX1	PEX13	0.5645467	2.26E-16
FDX1	HMGCS1	0.56478307	2.19E-16
FDX1	SNX27	0.56490583	2.15E-16
FDX1	MSMO1	0.56506234	2.1E-16
FDX1	FBXO34	0.56506951	2.1E-16
FDX1	PPP1CB	0.56510339	2.09E-16
FDX1	TXNRD1	0.56517271	2.06E-16
FDX1	HNRNPK	0.56526112	2.04E-16
FDX1	ECD	0.56529287	2.03E-16
FDX1	HAUS2	0.56584392	1.87E-16
FDX1	PRDX3	0.56612808	1.79E-16

FDX1	SRSF3	0.56614175	1.79E-16
FDX1	EPB41L1	0.56614716	1.79E-16
FDX1	DLAT	0.56634316	1.74E-16
FDX1	RAPH1	0.56635885	1.73E-16
FDX1	BRCC3	0.56682466	1.62E-16
FDX1	TMEM170A	0.56745133	1.47E-16
FDX1	HNRNPH2	0.56767269	1.42E-16
FDX1	PSMC6	0.56783908	1.39E-16
FDX1	PSEN1	0.56791183	1.37E-16
FDX1	ZNF143	0.5682161	1.31E-16
FDX1	PICALM	0.56843979	1.27E-16
FDX1	CNOT9	0.5684416	1.27E-16
FDX1	HNRNPA2B1	0.56853088	1.25E-16
FDX1	FASTKD2	0.56853389	1.25E-16
FDX1	HSPA14	0.56895657	1.18E-16
FDX1	OSTF1	0.56971944	1.05E-16
FDX1	FAM91A1	0.57030436	9.6E-17
FDX1	TBK1	0.57068538	9.06E-17
FDX1	SNX4	0.57093655	8.73E-17
FDX1	COPB1	0.57111136	8.5E-17
FDX1	BABAM2	0.57112686	8.48E-17
FDX1	SNX7	0.57135754	8.19E-17
FDX1	RAB5B	0.57184998	7.6E-17
FDX1	ACVR1	0.57201569	7.42E-17
FDX1	DNAJA2	0.57241827	6.98E-17
FDX1	KATNBL1	0.57243333	6.96E-17
FDX1	TSN	0.57276246	6.62E-17
FDX1	UHMK1	0.57303136	6.36E-17
FDX1	VKORC1L1	0.57398068	5.5E-17
FDX1	SCP2	0.57411174	5.39E-17
FDX1	TMCO1	0.57428448	5.25E-17
FDX1	IWS1	0.57465209	4.97E-17
FDX1	RAB9A	0.57471773	4.92E-17
FDX1	LRRFIP1	0.57475421	4.89E-17
FDX1	YME1L1	0.57483343	4.83E-17
FDX1	PPP1R2	0.5749448	4.75E-17
FDX1	SSB	0.57537062	4.45E-17
FDX1	AGFG1	0.57740242	3.26E-17
FDX1	RAB11A	0.57815566	2.9E-17
FDX1	TMBIM4	0.57818087	2.89E-17
FDX1	CDC42BPA	0.57916297	2.48E-17
FDX1	LRRC57	0.57916603	2.48E-17
FDX1	ACBD5	0.57972451	2.27E-17
FDX1	ZDHHC5	0.57988546	2.22E-17
FDX1	HNRNPF	0.57998595	2.18E-17
FDX1	TAF5L	0.58006216	2.16E-17
FDX1	KTN1	0.58065079	1.97E-17
FDX1	TP53BP2	0.58196443	1.6E-17
FDX1	TMEM9B	0.58205285	1.58E-17
FDX1	PELI1	0.58234451	1.51E-17
FDX1	VEZT	0.58264271	1.44E-17
FDX1	MED17	0.58269956	1.43E-17
FDX1	JRKL	0.58285621	1.39E-17
FDX1	PPM1B	0.58292696	1.38E-17
FDX1	PECR	0.58365447	1.23E-17
FDX1	BTBD10	0.5840198	1.16E-17
FDX1	AQR	0.58429541	1.11E-17
FDX1	POGK	0.58443823	1.09E-17

FDX1	SLC25A24	0.58529655	9.49E-18
FDX1	PCBP1	0.5856364	8.99E-18
FDX1	FAM118B	0.58686609	7.4E-18
FDX1	PLEKHB2	0.58806421	6.11E-18
FDX1	KCTD3	0.58859841	5.61E-18
FDX1	SKAP2	0.58874278	5.48E-18
FDX1	RPE	0.58950717	4.85E-18
FDX1	IDI1	0.5898763	4.57E-18
FDX1	CHMP2B	0.59081415	3.93E-18
FDX1	PPP3R1	0.59085075	3.91E-18
FDX1	TAX1BP1	0.59150597	3.51E-18
FDX1	GTF2B	0.59188686	3.3E-18
FDX1	CAB39	0.59321188	2.66E-18
FDX1	PPIG	0.59335087	2.6E-18
FDX1	CHP1	0.5937989	2.42E-18
FDX1	RAB1A	0.59413756	2.29E-18
FDX1	NDUFS1	0.59474347	2.07E-18
FDX1	BPNT1	0.59537525	1.87E-18
FDX1	ARHGAP21	0.59640419	1.58E-18
FDX1	XRCC5	0.59845647	1.13E-18
FDX1	NFE2L2	0.59944688	9.55E-19
FDX1	SNRNP27	0.59989185	8.87E-19
FDX1	ABI1	0.60008195	8.59E-19
FDX1	ZC3H15	0.60047501	8.05E-19
FDX1	SRP9	0.600808	7.61E-19
FDX1	ARPC5	0.60157553	6.69E-19
FDX1	RAB6A	0.6016558	6.6E-19
FDX1	MKLN1	0.60168731	6.57E-19
FDX1	SUMO1	0.60260508	5.63E-19
FDX1	SPTBN1	0.60342427	4.91E-19
FDX1	C1D	0.60529308	3.58E-19
FDX1	IDH1	0.60577023	3.3E-19
FDX1	SDHC	0.60769124	2.38E-19
FDX1	TANK	0.60772101	2.36E-19
FDX1	TMEM87B	0.60830528	2.14E-19
FDX1	ZFAND6	0.6100605	1.58E-19
FDX1	TMEM126B	0.61021483	1.54E-19
FDX1	CCNDBP1	0.61073985	1.41E-19
FDX1	PPP1R15B	0.61325573	9.08E-20
FDX1	PDCD10	0.61340169	8.85E-20
FDX1	CERS2	0.61373672	8.35E-20
FDX1	PRPF18	0.61406749	7.88E-20
FDX1	DYNC1I2	0.6195084	3.01E-20
FDX1	NIPA2	0.62395229	1.35E-20
FDX1	MAIP1	0.62400714	1.34E-20
FDX1	DUSP11	0.62406445	1.33E-20
FDX1	PAFAH1B2	0.62636356	8.72E-21
FDX1	COQ10B	0.62965075	4.76E-21
FDX1	CDC73	0.63961487	7.28E-22
FDX1	ZNRF2	0.6507049	8.28E-23
FDX1	SDHD	0.65395878	4.3E-23
FDX1	MOB4	0.65843649	1.72E-23
FDX1	SC5D	0.66662463	3.1E-24
GCSH	CENPN	0.50000089	1.2E-12
GCSH	IPO9	0.50030525	1.15E-12
GCSH	CHEK1	0.50040502	1.14E-12
GCSH	ZC3H15	0.50044399	1.13E-12
GCSH	CNIH1	0.50049998	1.13E-12

GCSH	CBX3	0.50056043	1.12E-12
GCSH	SMS	0.50061627	1.11E-12
GCSH	MRPL1	0.5009848	1.06E-12
GCSH	PDCL3	0.50116982	1.04E-12
GCSH	PSMC3	0.50125913	1.03E-12
GCSH	NUP205	0.50161842	9.86E-13
GCSH	DDB1	0.50168273	9.79E-13
GCSH	CCT3	0.50169113	9.78E-13
GCSH	AK2	0.5017501	9.71E-13
GCSH	COMMD8	0.50178172	9.67E-13
GCSH	SEC11A	0.50185028	9.59E-13
GCSH	HNRNPA3	0.5019643	9.46E-13
GCSH	PFDN1	0.50205515	9.36E-13
GCSH	OSGIN2	0.50213771	9.27E-13
GCSH	SUPT16H	0.50216597	9.23E-13
GCSH	ATAD2	0.50276121	8.6E-13
GCSH	PSMD8	0.5032352	8.12E-13
GCSH	SMYD2	0.50331009	8.05E-13
GCSH	CDK2AP1	0.50335456	8E-13
GCSH	HMGB1	0.50341881	7.94E-13
GCSH	SMARCAL1	0.50367556	7.7E-13
GCSH	RNF139	0.50372297	7.66E-13
GCSH	EREG	0.50376849	7.61E-13
GCSH	BOLA3	0.50378976	7.59E-13
GCSH	DCUN1D5	0.50421851	7.21E-13
GCSH	EEF2KMT	0.50428422	7.15E-13
GCSH	COPS6	0.50460481	6.88E-13
GCSH	DENR	0.50461253	6.87E-13
GCSH	LNPK	0.50521792	6.39E-13
GCSH	IFT52	0.50523718	6.37E-13
GCSH	ATP5MC3	0.50525524	6.36E-13
GCSH	TRNAU1AP	0.50533035	6.3E-13
GCSH	SET	0.50536749	6.27E-13
GCSH	AP2M1	0.50541892	6.23E-13
GCSH	CCT8	0.50571767	6.01E-13
GCSH	LMAN2L	0.50599317	5.81E-13
GCSH	LLPH	0.50599889	5.81E-13
GCSH	UNG	0.50610921	5.73E-13
GCSH	PPP1CB	0.50621397	5.66E-13
GCSH	ENOPH1	0.50625701	5.63E-13
GCSH	KIF3C	0.50634027	5.57E-13
GCSH	NUP93	0.50639395	5.54E-13
GCSH	ITGB1	0.50674144	5.31E-13
GCSH	STK3	0.50678888	5.28E-13
GCSH	NAP1L4	0.50733512	4.94E-13
GCSH	LARP6	0.50744671	4.87E-13
GCSH	UBXN2A	0.50752506	4.82E-13
GCSH	DNAJC9	0.50769571	4.72E-13
GCSH	BRIX1	0.50774501	4.7E-13
GCSH	CCDC43	0.50817612	4.45E-13
GCSH	SUGT1	0.5082754	4.4E-13
GCSH	SAE1	0.50835603	4.36E-13
GCSH	GTF2F2	0.50873899	4.16E-13
GCSH	PGK1	0.50918177	3.94E-13
GCSH	MTMR2	0.50918456	3.94E-13
GCSH	GPN3	0.5093804	3.84E-13
GCSH	BCCIP	0.50943038	3.82E-13
GCSH	EIF4A3	0.50959038	3.74E-13

GCSH	GGH	0.50959252	3.74E-13
GCSH	KPNA2	0.50960551	3.74E-13
GCSH	SGO2	0.50985848	3.62E-13
GCSH	XRCC5	0.51000868	3.56E-13
GCSH	RFC4	0.51035954	3.41E-13
GCSH	FKBP3	0.51039251	3.39E-13
GCSH	RRP36	0.51043242	3.38E-13
GCSH	TRMT112	0.51057959	3.32E-13
GCSH	MRPL21	0.51083481	3.21E-13
GCSH	FRMD5	0.510855	3.2E-13
GCSH	UQCRB	0.51089157	3.19E-13
GCSH	APEX1	0.51095527	3.16E-13
GCSH	BCAR3	0.51095781	3.16E-13
GCSH	MECR	0.5110624	3.12E-13
GCSH	CDK4	0.51121502	3.06E-13
GCSH	TTC1	0.51131025	3.03E-13
GCSH	CALM2	0.51144541	2.98E-13
GCSH	COA4	0.51144841	2.98E-13
GCSH	TIMM8B	0.51152817	2.95E-13
GCSH	PA2G4	0.51158682	2.93E-13
GCSH	CDC123	0.5116909	2.89E-13
GCSH	LRPPRC	0.51188768	2.82E-13
GCSH	MRPL33	0.51207368	2.76E-13
GCSH	EXOSC7	0.51248798	2.62E-13
GCSH	CAPRIN1	0.51254237	2.6E-13
GCSH	CKLF	0.51261909	2.58E-13
GCSH	HSPB11	0.51265475	2.56E-13
GCSH	MTCH2	0.51267286	2.56E-13
GCSH	PSMA7	0.51278609	2.52E-13
GCSH	C1D	0.51313845	2.41E-13
GCSH	ARMC1	0.51324986	2.38E-13
GCSH	SKA2	0.51328943	2.37E-13
GCSH	ARMC10	0.51370571	2.25E-13
GCSH	ANKRD27	0.51383612	2.21E-13
GCSH	MORN2	0.51434943	2.08E-13
GCSH	ABI2	0.51448082	2.04E-13
GCSH	SNRPD3	0.51561574	1.77E-13
GCSH	ANAPC10	0.51578147	1.73E-13
GCSH	CLTC	0.5163489	1.62E-13
GCSH	MRPL3	0.51678328	1.53E-13
GCSH	IAH1	0.51697354	1.49E-13
GCSH	RAB12	0.5169922	1.49E-13
GCSH	MAZ	0.51748126	1.4E-13
GCSH	RUVBL2	0.51763474	1.37E-13
GCSH	PRMT1	0.51812641	1.29E-13
GCSH	TMEM14C	0.51816829	1.28E-13
GCSH	PSMC6	0.51838073	1.25E-13
GCSH	THUMPD3	0.51854077	1.23E-13
GCSH	NCBP2	0.51882142	1.18E-13
GCSH	EIF2B2	0.51890228	1.17E-13
GCSH	PPM1G	0.51928208	1.12E-13
GCSH	FXR1	0.51930381	1.11E-13
GCSH	EMC4	0.51942517	1.1E-13
GCSH	COPS8	0.51976291	1.05E-13
GCSH	PPME1	0.52049667	9.55E-14
GCSH	GLO1	0.52071573	9.29E-14
GCSH	BRK1	0.5211697	8.77E-14
GCSH	MAGOHB	0.52158244	8.32E-14

GCSH	PKM	0.52161377	8.28E-14
GCSH	SLC25A32	0.52162998	8.27E-14
GCSH	GAR1	0.52185565	8.03E-14
GCSH	SLC25A12	0.52194822	7.94E-14
GCSH	UXS1	0.52246948	7.42E-14
GCSH	ERGIC2	0.52289781	7.03E-14
GCSH	DPM1	0.52299809	6.94E-14
GCSH	THOC7	0.52353158	6.48E-14
GCSH	GEMIN2	0.52363877	6.39E-14
GCSH	RPP30	0.5239973	6.1E-14
GCSH	BTF3L4	0.5246467	5.61E-14
GCSH	TMX2	0.52465621	5.6E-14
GCSH	TSN	0.52473612	5.54E-14
GCSH	ESD	0.52485491	5.46E-14
GCSH	MBOAT2	0.52488476	5.44E-14
GCSH	DNAJA2	0.52505337	5.32E-14
GCSH	RNF121	0.5250721	5.31E-14
GCSH	SNRPC	0.52546895	5.04E-14
GCSH	CBX1	0.52617061	4.6E-14
GCSH	PHF5A	0.52692953	4.17E-14
GCSH	WDR12	0.52703232	4.12E-14
GCSH	DUSP14	0.52770259	3.77E-14
GCSH	ENO1	0.52778403	3.73E-14
GCSH	THAP11	0.52853983	3.38E-14
GCSH	NUP155	0.52892483	3.21E-14
GCSH	RTN3	0.52914556	3.12E-14
GCSH	ATP6V1C1	0.52937376	3.03E-14
GCSH	ADAT1	0.52939488	3.02E-14
GCSH	SRRD	0.52958729	2.95E-14
GCSH	PNO1	0.52964742	2.92E-14
GCSH	NIP7	0.52973671	2.89E-14
GCSH	PSMA2	0.53039717	2.65E-14
GCSH	VTI1B	0.53040131	2.65E-14
GCSH	CCT6A	0.53042383	2.64E-14
GCSH	STIP1	0.53058782	2.58E-14
GCSH	TRMT12	0.53075693	2.53E-14
GCSH	DPY19L4	0.53085491	2.49E-14
GCSH	EIF2S2	0.53129636	2.35E-14
GCSH	ARL6IP6	0.53161208	2.26E-14
GCSH	ICMT	0.53265933	1.96E-14
GCSH	HNRNPC	0.53292634	1.9E-14
GCSH	FZD6	0.53295553	1.89E-14
GCSH	MOB4	0.53297623	1.88E-14
GCSH	MTX2	0.53310838	1.85E-14
GCSH	BEAN1	0.53310918	1.85E-14
GCSH	RHEB	0.533374	1.79E-14
GCSH	PDCD5	0.53364355	1.72E-14
GCSH	SNX24	0.53397953	1.65E-14
GCSH	GTF2A2	0.53403075	1.64E-14
GCSH	PAQR7	0.53412186	1.62E-14
GCSH	ARL8B	0.53455199	1.53E-14
GCSH	RAN	0.53493373	1.45E-14
GCSH	WDHD1	0.53502603	1.43E-14
GCSH	PSMA6	0.53517257	1.41E-14
GCSH	UBAC2	0.53530057	1.38E-14
GCSH	PAIP1	0.5357487	1.3E-14
GCSH	MTHFD1L	0.53616093	1.23E-14
GCSH	CIAPIN1	0.53638386	1.19E-14

GCSH	MSH2	0.53656679	1.17E-14
GCSH	NDUFB5	0.53674296	1.14E-14
GCSH	CHCHD7	0.53757974	1.02E-14
GCSH	PGAM1	0.53766939	1E-14
GCSH	EIF3H	0.53821021	9.34E-15
GCSH	ATRAID	0.53849265	8.99E-15
GCSH	EBNA1BP2	0.53858778	8.87E-15
GCSH	SUMF1	0.53950414	7.84E-15
GCSH	SLC4A1AP	0.53971091	7.62E-15
GCSH	NELFE	0.54023824	7.09E-15
GCSH	OLA1	0.54023944	7.09E-15
GCSH	C16orf74	0.54083483	6.54E-15
GCSH	ATP6V1E1	0.54091024	6.47E-15
GCSH	NEDD8	0.54094872	6.44E-15
GCSH	COPS5	0.54153017	5.95E-15
GCSH	SNAPC1	0.54205608	5.54E-15
GCSH	ERH	0.54212425	5.48E-15
GCSH	PAK1IP1	0.54289636	4.93E-15
GCSH	CFAP20	0.54316901	4.75E-15
GCSH	TIMMDC1	0.5432447	4.7E-15
GCSH	METTL6	0.54370635	4.41E-15
GCSH	DERA	0.5439671	4.26E-15
GCSH	PAICS	0.54446103	3.98E-15
GCSH	PRTFDC1	0.54461378	3.89E-15
GCSH	MDH1	0.54490162	3.74E-15
GCSH	PTK7	0.54532132	3.53E-15
GCSH	PUF60	0.54534665	3.52E-15
GCSH	MRPS23	0.54561356	3.39E-15
GCSH	CCDC90B	0.54660439	2.96E-15
GCSH	MRPS28	0.54696807	2.81E-15
GCSH	NIF3L1	0.54698506	2.8E-15
GCSH	INTS13	0.54756993	2.58E-15
GCSH	AMIGO2	0.54792412	2.46E-15
GCSH	GLRX5	0.5480162	2.43E-15
GCSH	RCN2	0.54810048	2.4E-15
GCSH	CETN3	0.54816179	2.38E-15
GCSH	SUMO2	0.5486763	2.21E-15
GCSH	FBXO45	0.54876663	2.19E-15
GCSH	C16orf87	0.54926484	2.04E-15
GCSH	KLHL7	0.55020178	1.79E-15
GCSH	BANF1	0.55020674	1.79E-15
GCSH	ACTL6A	0.5512982	1.53E-15
GCSH	BRD7	0.55249311	1.29E-15
GCSH	HTRA2	0.55256103	1.28E-15
GCSH	CCT2	0.55290507	1.22E-15
GCSH	PAFAH1B2	0.55298959	1.21E-15
GCSH	SSRP1	0.55440741	9.85E-16
GCSH	UBE2E3	0.55545382	8.49E-16
GCSH	MBTPS1	0.55586907	8E-16
GCSH	PYGL	0.55616817	7.66E-16
GCSH	POLR2G	0.55619172	7.63E-16
GCSH	PSMA1	0.55666491	7.13E-16
GCSH	HAT1	0.55687675	6.92E-16
GCSH	LYPLA1	0.55782574	6.04E-16
GCSH	KDM1A	0.55928992	4.88E-16
GCSH	AHSA1	0.5594388	4.78E-16
GCSH	DFFA	0.55952342	4.72E-16
GCSH	HACD1	0.56118511	3.71E-16

GCSH	CPSF3	0.5614791	3.55E-16
GCSH	RANBP1	0.56153156	3.52E-16
GCSH	LRRC42	0.56244018	3.09E-16
GCSH	TRAPPC2L	0.56266624	2.99E-16
GCSH	MEAK7	0.56271411	2.96E-16
GCSH	STMN1	0.56357798	2.61E-16
GCSH	GOT2	0.56358782	2.61E-16
GCSH	KNSTRN	0.5641315	2.41E-16
GCSH	TCEA1	0.56469625	2.21E-16
GCSH	MTERF3	0.566281	1.75E-16
GCSH	CRIPT	0.56634188	1.74E-16
GCSH	FAM3C	0.56661517	1.67E-16
GCSH	CDCA4	0.56697311	1.58E-16
GCSH	ITGB1BP1	0.56817426	1.32E-16
GCSH	CMC2	0.56862313	1.24E-16
GCSH	CCT5	0.56931094	1.11E-16
GCSH	UBE2E1	0.57062404	9.15E-17
GCSH	XRCC6	0.57067713	9.08E-17
GCSH	ATMIN	0.57280349	6.58E-17
GCSH	RPL26L1	0.57283809	6.55E-17
GCSH	RCN1	0.57467849	4.95E-17
GCSH	PSMB5	0.57468334	4.94E-17
GCSH	RPGRIP1L	0.5750939	4.64E-17
GCSH	MRPL13	0.57597628	4.06E-17
GCSH	RUVBL1	0.57663995	3.66E-17
GCSH	SLC25A17	0.57938695	2.4E-17
GCSH	RTN4	0.58043798	2.04E-17
GCSH	SUMO1	0.58222101	1.54E-17
GCSH	TPD52L2	0.58257769	1.46E-17
GCSH	MZT1	0.58343403	1.27E-17
GCSH	RBX1	0.58389563	1.18E-17
GCSH	PPIL1	0.58460748	1.06E-17
GCSH	COX7A2L	0.5847086	1.04E-17
GCSH	NUTF2	0.58482922	1.02E-17
GCSH	CCT7	0.58529103	9.5E-18
GCSH	MRPS10	0.58714896	7.07E-18
GCSH	PSMC1	0.58775185	6.42E-18
GCSH	HIKESHI	0.58954705	4.82E-18
GCSH	PSMD7	0.58980987	4.62E-18
GCSH	DPY30	0.59056733	4.09E-18
GCSH	EMC8	0.5909914	3.82E-18
GCSH	POLR2K	0.59345258	2.56E-18
GCSH	ENY2	0.59575306	1.76E-18
GCSH	VIRMA	0.59599877	1.69E-18
GCSH	PSMD2	0.5967763	1.49E-18
GCSH	GPN1	0.59745753	1.33E-18
GCSH	EMC2	0.59964549	9.24E-19
GCSH	RFC2	0.60083284	7.58E-19
GCSH	MRPL15	0.60293554	5.33E-19
GCSH	MRPL30	0.60310109	5.18E-19
GCSH	NUDCD1	0.60396599	4.48E-19
GCSH	REXO2	0.60419764	4.31E-19
GCSH	C8orf33	0.60506937	3.71E-19
GCSH	NSMCE2	0.60512722	3.68E-19
GCSH	YWHAQ	0.6073305	2.53E-19
GCSH	SSB	0.60955296	1.73E-19
GCSH	HSBP1	0.61053865	1.46E-19
GCSH	WASHC5	0.61417148	7.74E-20

GCSH	STRAP	0.61503745	6.65E-20
GCSH	HSDL1	0.61735551	4.42E-20
GCSH	AIG1	0.62064733	2.46E-20
GCSH	PSMD14	0.62165814	2.05E-20
GCSH	SF3B6	0.623051	1.59E-20
GCSH	ADK	0.62334032	1.51E-20
GCSH	TXNDC9	0.62444988	1.24E-20
GCSH	ELOC	0.62507542	1.1E-20
GCSH	TMEM237	0.62518655	1.08E-20
GCSH	MNAT1	0.62634653	8.75E-21
GCSH	DCAF13	0.62826151	6.16E-21
GCSH	CSE1L	0.63382843	2.18E-21
GCSH	UBE2V2	0.63823393	9.48E-22
GCSH	MPHOSPH6	0.64077015	5.83E-22
GCSH	METTL5	0.64204844	4.55E-22
GCSH	PFDN4	0.64329327	3.57E-22
GLS	VAPB	0.50005831	1.19E-12
GLS	C3orf14	0.50010487	1.18E-12
GLS	SH3PXD2A	0.50013263	1.18E-12
GLS	SLC25A44	0.50019829	1.17E-12
GLS	JRKL	0.50024234	1.16E-12
GLS	EPB41L4A	0.50049342	1.13E-12
GLS	MUL1	0.50052966	1.12E-12
GLS	GCC1	0.50053171	1.12E-12
GLS	GOLPH3	0.50054284	1.12E-12
GLS	DYRK2	0.50055609	1.12E-12
GLS	ZC4H2	0.50057705	1.12E-12
GLS	C4orf3	0.50068686	1.1E-12
GLS	CLIC4	0.50078299	1.09E-12
GLS	FYN	0.50135273	1.02E-12
GLS	NT5DC1	0.50141287	1.01E-12
GLS	PNMA8A	0.50150212	1E-12
GLS	IK	0.50154011	9.95E-13
GLS	DDX18	0.50164292	9.83E-13
GLS	FSTL1	0.50165376	9.82E-13
GLS	LSM14B	0.50184559	9.6E-13
GLS	AMFR	0.50188048	9.56E-13
GLS	CRMP1	0.50189256	9.54E-13
GLS	MFSD14B	0.50190909	9.52E-13
GLS	STAU1	0.50215797	9.24E-13
GLS	GAS7	0.50227844	9.11E-13
GLS	IPPK	0.5022902	9.1E-13
GLS	M6PR	0.50229725	9.09E-13
GLS	GDI1	0.50233765	9.05E-13
GLS	SMC2	0.50238745	8.99E-13
GLS	SORBS3	0.50259384	8.77E-13
GLS	KCTD21	0.50276177	8.6E-13
GLS	NR2F2	0.50276832	8.59E-13
GLS	ZSWIM1	0.50288524	8.47E-13
GLS	FMNL1	0.50297273	8.38E-13
GLS	RERG	0.50299737	8.36E-13
GLS	RAB7A	0.50308899	8.26E-13
GLS	PHACTR2	0.50310281	8.25E-13
GLS	CCDC97	0.50326665	8.09E-13
GLS	NAP1L5	0.50327472	8.08E-13
GLS	TMEM248	0.50347764	7.89E-13
GLS	ABI1	0.50354533	7.82E-13
GLS	RANBP3	0.50355249	7.81E-13

GLS	NFKB1	0.5035643	7.8E-13
GLS	TLE4	0.50369046	7.69E-13
GLS	NPLOC4	0.50372759	7.65E-13
GLS	FAM210A	0.50374049	7.64E-13
GLS	TAX1BP1	0.50379551	7.59E-13
GLS	RAI2	0.50405366	7.36E-13
GLS	ZMPSTE24	0.50408753	7.33E-13
GLS	SORL1	0.50418243	7.24E-13
GLS	CDC42	0.50435314	7.09E-13
GLS	DNAJA1	0.50438687	7.07E-13
GLS	LRRCC1	0.50440957	7.05E-13
GLS	DEGS1	0.5044802	6.99E-13
GLS	NECTIN3	0.5045688	6.91E-13
GLS	TPK1	0.50461419	6.87E-13
GLS	COQ5	0.50471735	6.79E-13
GLS	CELF2	0.50515763	6.44E-13
GLS	ACTR3	0.50518325	6.42E-13
GLS	STK3	0.50523377	6.38E-13
GLS	AGPAT5	0.50531231	6.32E-13
GLS	NXPE3	0.50552042	6.16E-13
GLS	IQCK	0.5055609	6.13E-13
GLS	NOTCH3	0.50562706	6.08E-13
GLS	DUSP11	0.50571166	6.02E-13
GLS	SZRD1	0.50580263	5.95E-13
GLS	SAMD4A	0.50581622	5.94E-13
GLS	ADO	0.50609321	5.74E-13
GLS	F10	0.5061718	5.69E-13
GLS	NCBP2	0.50617521	5.69E-13
GLS	NUCKS1	0.50626764	5.62E-13
GLS	MFAP1	0.50638524	5.54E-13
GLS	ZNF263	0.50653329	5.44E-13
GLS	ANKRD11	0.50658668	5.41E-13
GLS	ODF2L	0.5067107	5.33E-13
GLS	TCOF1	0.50675923	5.3E-13
GLS	KSR1	0.50685096	5.24E-13
GLS	PIK3R2	0.50687984	5.22E-13
GLS	HMGB1	0.50700801	5.14E-13
GLS	DYNLL2	0.50703825	5.12E-13
GLS	ELK3	0.50729391	4.96E-13
GLS	CPE	0.50738887	4.91E-13
GLS	ALKBH5	0.50756059	4.8E-13
GLS	HSP90AA1	0.50772318	4.71E-13
GLS	C1orf216	0.50778766	4.67E-13
GLS	SLC38A6	0.5079099	4.6E-13
GLS	ADH5	0.50792293	4.6E-13
GLS	KLC2	0.50795917	4.57E-13
GLS	TNPO3	0.50796198	4.57E-13
GLS	DLL4	0.50807125	4.51E-13
GLS	LPGAT1	0.50844011	4.31E-13
GLS	PDZD8	0.50847188	4.3E-13
GLS	FIG4	0.50848762	4.29E-13
GLS	NFE2L2	0.5086264	4.22E-13
GLS	NCKAP5L	0.5087454	4.15E-13
GLS	GRPEL2	0.50898211	4.04E-13
GLS	PTGES3	0.50920374	3.93E-13
GLS	CAPRIN1	0.50922704	3.92E-13
GLS	CNOT9	0.509259	3.9E-13
GLS	TRIM56	0.50940336	3.83E-13

GLS	SLC22A4	0.50941596	3.83E-13
GLS	CHN1	0.50948044	3.8E-13
GLS	ATL2	0.50951173	3.78E-13
GLS	RFK	0.50952738	3.77E-13
GLS	NRBF2	0.5096599	3.71E-13
GLS	HNRNPC	0.50975654	3.67E-13
GLS	MAT2B	0.50986533	3.62E-13
GLS	TMEM43	0.50998375	3.57E-13
GLS	IFNGR1	0.51020429	3.47E-13
GLS	EXOSC9	0.51031563	3.42E-13
GLS	SH3GLB1	0.51034992	3.41E-13
GLS	MEIS3	0.51036461	3.4E-13
GLS	GEMIN2	0.51040933	3.39E-13
GLS	MMRN2	0.5104114	3.38E-13
GLS	MOAP1	0.5104718	3.36E-13
GLS	NDUFS1	0.51055666	3.32E-13
GLS	FNBP1	0.51057496	3.32E-13
GLS	C3orf38	0.51075611	3.24E-13
GLS	SBDS	0.51105177	3.13E-13
GLS	NIPA2	0.51113398	3.1E-13
GLS	NAMPT	0.51130531	3.03E-13
GLS	SNX9	0.51132914	3.02E-13
GLS	IL13RA1	0.51133969	3.02E-13
GLS	HIBADH	0.51135626	3.01E-13
GLS	DUS4L	0.51175505	2.87E-13
GLS	ATPSCKMT	0.5118932	2.82E-13
GLS	ENAH	0.511939	2.8E-13
GLS	DCAF12	0.51210764	2.74E-13
GLS	CDH5	0.51216152	2.73E-13
GLS	EPB41L2	0.51225625	2.69E-13
GLS	RGL1	0.51231363	2.67E-13
GLS	MARCKS	0.51238785	2.65E-13
GLS	BCAS2	0.51246279	2.63E-13
GLS	PHTF1	0.5124714	2.62E-13
GLS	PLEKHA1	0.51287604	2.49E-13
GLS	TRERF1	0.51290629	2.48E-13
GLS	ZNFX1	0.51291587	2.48E-13
GLS	ZNF200	0.51301899	2.45E-13
GLS	METAP1	0.51302351	2.45E-13
GLS	DEK	0.51303878	2.44E-13
GLS	TMED4	0.51308999	2.43E-13
GLS	UBE2H	0.51311155	2.42E-13
GLS	GAB2	0.51312179	2.42E-13
GLS	SMG6	0.51336619	2.35E-13
GLS	COL4A1	0.51350306	2.31E-13
GLS	SRSF3	0.51354843	2.29E-13
GLS	NUDCD3	0.51377105	2.23E-13
GLS	BCAP29	0.51381279	2.22E-13
GLS	ISCU	0.51386825	2.2E-13
GLS	TMCC1	0.51391842	2.19E-13
GLS	UGDH	0.51393294	2.19E-13
GLS	NDN	0.51400193	2.17E-13
GLS	AMPD3	0.51414413	2.13E-13
GLS	RAB8A	0.51420883	2.11E-13
GLS	CMAS	0.51429861	2.09E-13
GLS	TUBA1B	0.51442107	2.06E-13
GLS	CACNA2D1	0.51446316	2.05E-13
GLS	PAQR3	0.51473495	1.98E-13

GLS	BBS7	0.51493474	1.93E-13
GLS	DOCK10	0.51495598	1.92E-13
GLS	FNDC3B	0.51498897	1.92E-13
GLS	DCBLD1	0.5150223	1.91E-13
GLS	ICMT	0.51513421	1.88E-13
GLS	FADS2	0.51514943	1.88E-13
GLS	PRR14L	0.5152827	1.85E-13
GLS	RAP1B	0.51529897	1.84E-13
GLS	DDX21	0.5155029	1.8E-13
GLS	AASS	0.5162132	1.64E-13
GLS	HARS2	0.5162398	1.64E-13
GLS	ZNF790	0.51625898	1.63E-13
GLS	HDAC9	0.5162653	1.63E-13
GLS	PLAA	0.51629535	1.63E-13
GLS	ISL1	0.5163081	1.62E-13
GLS	GTDC1	0.51637422	1.61E-13
GLS	PLXDC1	0.5163884	1.61E-13
GLS	ADGRL2	0.51650428	1.58E-13
GLS	DPP4	0.51672237	1.54E-13
GLS	SESTD1	0.51674839	1.54E-13
GLS	MNAT1	0.51691025	1.51E-13
GLS	CDKL1	0.5169481	1.5E-13
GLS	GTF2E1	0.51699944	1.49E-13
GLS	PTDSS1	0.51741992	1.41E-13
GLS	MSI2	0.51756061	1.39E-13
GLS	PARP8	0.51760498	1.38E-13
GLS	PDLIM5	0.51795249	1.32E-13
GLS	PLS3	0.51795799	1.32E-13
GLS	TCF20	0.51803793	1.31E-13
GLS	AP003108.2	0.51805376	1.3E-13
GLS	QPCT	0.51808868	1.3E-13
GLS	PAK1IP1	0.51820207	1.28E-13
GLS	APBB1	0.51829635	1.26E-13
GLS	SIAH1	0.51845262	1.24E-13
GLS	DYNLT3	0.5185245	1.23E-13
GLS	U2AF2	0.51856109	1.22E-13
GLS	LIMS1	0.51865575	1.21E-13
GLS	GALNS	0.51869011	1.2E-13
GLS	SRXN1	0.5188132	1.18E-13
GLS	PPP2R5D	0.51888476	1.17E-13
GLS	NAB1	0.51899373	1.16E-13
GLS	ARMH4	0.51907078	1.15E-13
GLS	KIRREL1	0.51949514	1.09E-13
GLS	MCTP1	0.51979183	1.05E-13
GLS	NR1D2	0.51981775	1.04E-13
GLS	MRPL42	0.51985367	1.04E-13
GLS	RAD51C	0.51998194	1.02E-13
GLS	ARAP1	0.52022992	9.88E-14
GLS	ARFGEF1	0.52037688	9.7E-14
GLS	FYCO1	0.52050165	9.55E-14
GLS	TM9SF4	0.52052405	9.52E-14
GLS	KIF5B	0.5206095	9.42E-14
GLS	GLIS2	0.52061526	9.41E-14
GLS	LINS1	0.52083193	9.15E-14
GLS	LYPLA1	0.52095803	9.01E-14
GLS	MFSD1	0.52108328	8.87E-14
GLS	HINT3	0.5211542	8.79E-14
GLS	ATP11C	0.52127481	8.65E-14

GLS	VAMP3	0.521322	8.6E-14
GLS	EAPP	0.52132983	8.59E-14
GLS	IKBIP	0.52134647	8.57E-14
GLS	VRK2	0.52158735	8.31E-14
GLS	KDM6B	0.52159377	8.31E-14
GLS	FAM210B	0.5216706	8.22E-14
GLS	CERS6	0.52174071	8.15E-14
GLS	TTL	0.52199722	7.89E-14
GLS	STX7	0.52204293	7.84E-14
GLS	SLC24A1	0.52208931	7.79E-14
GLS	MAN1C1	0.52213353	7.75E-14
GLS	FAM122B	0.5223138	7.57E-14
GLS	VPS35L	0.52242863	7.46E-14
GLS	WEE1	0.52243126	7.46E-14
GLS	TIAM1	0.52245479	7.44E-14
GLS	FBXO45	0.5226679	7.24E-14
GLS	LAMP1	0.52298986	6.94E-14
GLS	RAB10	0.52306569	6.88E-14
GLS	DCUN1D3	0.52316184	6.79E-14
GLS	TRAM1	0.52323574	6.73E-14
GLS	SSB	0.52345703	6.54E-14
GLS	SPRTN	0.52347745	6.52E-14
GLS	TMEM267	0.52401025	6.09E-14
GLS	RFWD3	0.52433991	5.84E-14
GLS	LRRC41	0.52451906	5.7E-14
GLS	ATP9A	0.52459986	5.64E-14
GLS	SUFU	0.52461547	5.63E-14
GLS	MSH2	0.52462854	5.62E-14
GLS	SMARCAL1	0.52463751	5.62E-14
GLS	ZCCHC9	0.52464109	5.61E-14
GLS	ODF2	0.52473724	5.54E-14
GLS	MSANTD3-TMEFF1	0.52488173	5.44E-14
GLS	CISD2	0.52490092	5.43E-14
GLS	RAB22A	0.5249901	5.36E-14
GLS	DENND2A	0.52500994	5.35E-14
GLS	PXN	0.52535639	5.12E-14
GLS	PFDN1	0.52551094	5.01E-14
GLS	ZC3H15	0.52556111	4.98E-14
GLS	SIPA1L3	0.52568536	4.9E-14
GLS	PRPF4	0.52572685	4.88E-14
GLS	CD46	0.52573996	4.87E-14
GLS	RAB35	0.52582539	4.81E-14
GLS	DYNC1I2	0.52588207	4.78E-14
GLS	MINPP1	0.52605289	4.67E-14
GLS	PIP4K2C	0.52610645	4.64E-14
GLS	TERF2	0.52637697	4.48E-14
GLS	ZMYM2	0.52646361	4.43E-14
GLS	BTF3L4	0.52651175	4.4E-14
GLS	METTL9	0.52651822	4.4E-14
GLS	MOB4	0.52653073	4.39E-14
GLS	OXCT1	0.52653385	4.39E-14
GLS	FAM204A	0.52661595	4.34E-14
GLS	TCFL5	0.5266218	4.34E-14
GLS	NEDD4	0.52669014	4.3E-14
GLS	FKBP9	0.52671445	4.29E-14
GLS	SPTY2D1	0.52696327	4.15E-14
GLS	DNAJC21	0.5269821	4.14E-14
GLS	MCMBP	0.52708938	4.08E-14

GLS	CDYL	0.52714653	4.05E-14
GLS	SYS1	0.5271586	4.05E-14
GLS	HDAC2	0.52747002	3.89E-14
GLS	MTM1	0.52749294	3.88E-14
GLS	RAB29	0.52769011	3.78E-14
GLS	OSBPL10	0.52770327	3.77E-14
GLS	RAB2A	0.5278684	3.69E-14
GLS	GNAO1	0.52788794	3.68E-14
GLS	AHCTF1	0.52793084	3.66E-14
GLS	MIS18BP1	0.5282545	3.51E-14
GLS	SNX1	0.52837691	3.45E-14
GLS	HTATSF1	0.52843256	3.43E-14
GLS	ABR	0.52853879	3.38E-14
GLS	TOP2B	0.52859861	3.35E-14
GLS	HMGN4	0.52863624	3.34E-14
GLS	BMP2K	0.52885734	3.24E-14
GLS	PVR	0.52888799	3.23E-14
GLS	TMEM60	0.5291875	3.11E-14
GLS	DDX24	0.52920421	3.1E-14
GLS	APH1B	0.5292526	3.08E-14
GLS	NUP62	0.52932091	3.05E-14
GLS	SLC30A6	0.5293846	3.03E-14
GLS	PRKCH	0.52939718	3.02E-14
GLS	CRCP	0.5294564	3E-14
GLS	NID1	0.52950443	2.98E-14
GLS	YTHDF1	0.52953008	2.97E-14
GLS	TDP1	0.52964736	2.92E-14
GLS	AK6	0.52986398	2.84E-14
GLS	GMCL1	0.53000841	2.79E-14
GLS	PEX26	0.53009413	2.76E-14
GLS	TRA2B	0.53011198	2.75E-14
GLS	OXR1	0.53011731	2.75E-14
GLS	TRAFD1	0.53015975	2.73E-14
GLS	TMEM128	0.530258	2.7E-14
GLS	FEM1C	0.53039552	2.65E-14
GLS	MAP1LC3B	0.53048861	2.62E-14
GLS	PPP3CA	0.53050689	2.61E-14
GLS	IFNAR1	0.53054743	2.6E-14
GLS	GASK1B	0.53077311	2.52E-14
GLS	SLC7A2	0.53088677	2.48E-14
GLS	FERMT2	0.53091663	2.47E-14
GLS	CLTC	0.5309508	2.46E-14
GLS	RASA2	0.53096096	2.46E-14
GLS	SLC30A1	0.53096245	2.46E-14
GLS	SCOC	0.53098363	2.45E-14
GLS	RPE	0.53118042	2.39E-14
GLS	NEO1	0.53135464	2.33E-14
GLS	SUMO2	0.53138535	2.32E-14
GLS	LRBA	0.53141982	2.31E-14
GLS	CPNE8	0.53150745	2.29E-14
GLS	GRB2	0.53157647	2.27E-14
GLS	ABCF2	0.53163426	2.25E-14
GLS	PLEKHA2	0.53164608	2.25E-14
GLS	ATXN3	0.53176112	2.21E-14
GLS	ATE1	0.53177416	2.21E-14
GLS	RAB23	0.53195877	2.16E-14
GLS	PIAS1	0.53218617	2.09E-14
GLS	WDFY2	0.53218641	2.09E-14

GLS	PSMC2	0.53219559	2.09E-14
GLS	PRCP	0.53222655	2.08E-14
GLS	PSMD12	0.53222817	2.08E-14
GLS	C9orf64	0.53229852	2.06E-14
GLS	CPSF6	0.53233727	2.05E-14
GLS	ZMAT3	0.53240157	2.03E-14
GLS	PDE12	0.53278298	1.93E-14
GLS	DNAJC10	0.53283846	1.92E-14
GLS	MAP4K4	0.53298326	1.88E-14
GLS	IMMP2L	0.53304584	1.87E-14
GLS	AP1M1	0.53311806	1.85E-14
GLS	TPST1	0.53316611	1.84E-14
GLS	TMEM199	0.53333736	1.79E-14
GLS	SETX	0.53360174	1.73E-14
GLS	PFN2	0.53364194	1.72E-14
GLS	SV2A	0.53371649	1.71E-14
GLS	C3orf80	0.53382971	1.68E-14
GLS	CRK	0.5340212	1.64E-14
GLS	ROCK2	0.53426683	1.59E-14
GLS	MBIP	0.53430346	1.58E-14
GLS	L3MBTL3	0.53452339	1.53E-14
GLS	ZNF468	0.53453912	1.53E-14
GLS	ASNSD1	0.53456951	1.52E-14
GLS	FZD6	0.53478904	1.48E-14
GLS	CHST11	0.53483699	1.47E-14
GLS	ENOPH1	0.53491078	1.46E-14
GLS	WASF2	0.5349661	1.44E-14
GLS	BICD1	0.53501209	1.44E-14
GLS	KIAA1143	0.5350499	1.43E-14
GLS	FRYL	0.53522783	1.39E-14
GLS	UBE2D1	0.53527462	1.39E-14
GLS	YWHAB	0.53542487	1.36E-14
GLS	F2R	0.53557136	1.33E-14
GLS	RASSF8	0.53563273	1.32E-14
GLS	ERAP1	0.5356676	1.31E-14
GLS	MRFAP1L1	0.53571206	1.31E-14
GLS	BORCS7	0.53581577	1.29E-14
GLS	GM2A	0.53594268	1.27E-14
GLS	GORASP2	0.5360327	1.25E-14
GLS	KPNA4	0.5361522	1.23E-14
GLS	WSB2	0.53632059	1.2E-14
GLS	TAF9	0.53647267	1.18E-14
GLS	RELL1	0.53647356	1.18E-14
GLS	EHD3	0.53667552	1.15E-14
GLS	ERMP1	0.5366986	1.14E-14
GLS	GABARAPL2	0.53680413	1.13E-14
GLS	FUT11	0.53683009	1.12E-14
GLS	AIG1	0.53686413	1.12E-14
GLS	VBP1	0.53689004	1.12E-14
GLS	GUCY1B1	0.53689014	1.12E-14
GLS	EIF1B	0.53698591	1.1E-14
GLS	ASCC3	0.53702061	1.1E-14
GLS	VKORC1L1	0.53709904	1.08E-14
GLS	TGS1	0.53710372	1.08E-14
GLS	CACUL1	0.53717243	1.07E-14
GLS	EIF2AK2	0.53722477	1.07E-14
GLS	RSPH3	0.53737761	1.04E-14
GLS	FEZ2	0.53753196	1.02E-14

GLS	PDE4B	0.53756813	1.02E-14
GLS	SERPINE2	0.53797426	9.64E-15
GLS	TIMP2	0.53801372	9.59E-15
GLS	DIPK2A	0.53806175	9.53E-15
GLS	SMU1	0.53815645	9.41E-15
GLS	PAFAH1B2	0.53820877	9.34E-15
GLS	ANKRD42	0.53823511	9.31E-15
GLS	EPB41L3	0.53824699	9.29E-15
GLS	TMED10	0.53829797	9.23E-15
GLS	PBX3	0.53840822	9.09E-15
GLS	TAF6	0.53851946	8.96E-15
GLS	NIPSNAP3A	0.53851967	8.96E-15
GLS	MPHOSPH10	0.53854532	8.93E-15
GLS	ADORA2A	0.53855154	8.92E-15
GLS	PPP3R1	0.53865544	8.79E-15
GLS	FNTB	0.53878211	8.64E-15
GLS	PSMF1	0.53885909	8.55E-15
GLS	HNRNPA0	0.53888664	8.52E-15
GLS	RCAN3	0.53896245	8.44E-15
GLS	NLGN2	0.53903493	8.35E-15
GLS	RRAGA	0.53907958	8.3E-15
GLS	GNAQ	0.539085	8.3E-15
GLS	ELP3	0.53910823	8.27E-15
GLS	TRMT1L	0.53920574	8.16E-15
GLS	PPP5C	0.53932753	8.03E-15
GLS	SRGAP2B	0.53934624	8.01E-15
GLS	C9orf78	0.5395429	7.8E-15
GLS	COPS2	0.53970665	7.63E-15
GLS	DOCK1	0.53989663	7.43E-15
GLS	FBXO42	0.54002999	7.3E-15
GLS	SAP130	0.54004651	7.28E-15
GLS	ATG5	0.54004794	7.28E-15
GLS	PDK3	0.54008421	7.24E-15
GLS	DNAJC8	0.54010763	7.22E-15
GLS	RAB5B	0.54022728	7.1E-15
GLS	KDSR	0.54028345	7.05E-15
GLS	FADS1	0.54045581	6.89E-15
GLS	PSMC6	0.54056753	6.78E-15
GLS	TGOLN2	0.54058481	6.77E-15
GLS	CUEDC1	0.54065136	6.71E-15
GLS	CCDC86	0.54065437	6.7E-15
GLS	AP3M2	0.54072496	6.64E-15
GLS	WDR26	0.54074973	6.62E-15
GLS	BRD3OS	0.54094905	6.44E-15
GLS	MZT1	0.54095797	6.43E-15
GLS	JAM3	0.54096896	6.42E-15
GLS	NFATC3	0.54098023	6.41E-15
GLS	NUDCD2	0.54102001	6.38E-15
GLS	PER2	0.54103128	6.37E-15
GLS	RNF8	0.54109199	6.31E-15
GLS	GOLIM4	0.54111164	6.3E-15
GLS	PEAK1	0.54113058	6.28E-15
GLS	BCL9L	0.54116356	6.25E-15
GLS	FBXO5	0.54128677	6.15E-15
GLS	DCAKD	0.54132705	6.12E-15
GLS	SYT11	0.54142837	6.03E-15
GLS	SLC8A1	0.54147698	5.99E-15
GLS	ZNF184	0.54172439	5.79E-15

GLS	NUP107	0.54186197	5.68E-15
GLS	NCSTN	0.54190319	5.65E-15
GLS	ITGAV	0.54205021	5.54E-15
GLS	ADAM22	0.54218221	5.44E-15
GLS	INPP5F	0.54237004	5.3E-15
GLS	CRIM1	0.54275095	5.03E-15
GLS	BAG4	0.54275548	5.03E-15
GLS	MEF2C	0.54278473	5.01E-15
GLS	PCYT1A	0.5428626	4.96E-15
GLS	AKAP12	0.54299113	4.87E-15
GLS	FXYD6	0.54310495	4.79E-15
GLS	UBE2A	0.54331493	4.66E-15
GLS	ZNF217	0.54349507	4.54E-15
GLS	ACTR2	0.54355991	4.5E-15
GLS	ATXN7L3B	0.54358667	4.49E-15
GLS	UBN1	0.54359045	4.48E-15
GLS	RHBDD1	0.54404903	4.21E-15
GLS	BLOC1S5	0.54405939	4.2E-15
GLS	CLSTN3	0.54410265	4.18E-15
GLS	PNRC1	0.54432445	4.05E-15
GLS	MFN2	0.54433268	4.05E-15
GLS	ADAMTS2	0.54433579	4.05E-15
GLS	PANK3	0.5443734	4.03E-15
GLS	ITSN1	0.54441699	4E-15
GLS	MAPRE2	0.54445227	3.98E-15
GLS	PBXIP1	0.54459288	3.91E-15
GLS	GLB1	0.54481053	3.79E-15
GLS	MAF	0.5448682	3.76E-15
GLS	CD93	0.54491503	3.74E-15
GLS	SUPT16H	0.5449181	3.73E-15
GLS	TEX30	0.54496813	3.71E-15
GLS	RNF138	0.545092	3.65E-15
GLS	GFM1	0.54509303	3.65E-15
GLS	TDP2	0.54510049	3.64E-15
GLS	PSAP	0.54528592	3.55E-15
GLS	VAPA	0.54535229	3.52E-15
GLS	HS3ST3B1	0.54537957	3.5E-15
GLS	UEVLD	0.54543604	3.48E-15
GLS	LACC1	0.54574179	3.33E-15
GLS	ZEB2	0.54583965	3.29E-15
GLS	TARDBP	0.54590518	3.26E-15
GLS	PPP2R2B	0.54597107	3.23E-15
GLS	TMEM135	0.54597446	3.23E-15
GLS	LIPA	0.54603686	3.2E-15
GLS	TANK	0.54605135	3.19E-15
GLS	ATXN1	0.54606403	3.19E-15
GLS	HERC3	0.5463481	3.06E-15
GLS	OPA1	0.5463658	3.06E-15
GLS	KBTBD2	0.54649324	3E-15
GLS	PI4K2A	0.54668421	2.92E-15
GLS	GPC6	0.54670209	2.92E-15
GLS	NUP50	0.54670457	2.92E-15
GLS	UBE2Z	0.54670616	2.91E-15
GLS	CHSY1	0.54675747	2.89E-15
GLS	PTK2	0.54720406	2.72E-15
GLS	RAB5A	0.54772107	2.53E-15
GLS	KHDRBS1	0.548012	2.43E-15
GLS	RTF2	0.54827341	2.34E-15

GLS	SF3A3	0.5483844	2.31E-15
GLS	WIPF2	0.54839734	2.3E-15
GLS	CTR9	0.54846827	2.28E-15
GLS	IPO7	0.54851465	2.27E-15
GLS	FNIP2	0.54875312	2.19E-15
GLS	PGRMC1	0.54886635	2.16E-15
GLS	CCDC43	0.5489019	2.15E-15
GLS	GABPB1	0.54899026	2.12E-15
GLS	AFAP1L1	0.54901256	2.11E-15
GLS	KIAA1549L	0.54904328	2.1E-15
GLS	GNPTAB	0.54904824	2.1E-15
GLS	TSC22D2	0.54906882	2.1E-15
GLS	SMN1	0.54907687	2.09E-15
GLS	PSME3	0.54917188	2.07E-15
GLS	SMC1A	0.54920998	2.05E-15
GLS	SPCS2	0.54924145	2.05E-15
GLS	MAML2	0.5493964	2E-15
GLS	PLRG1	0.54950305	1.97E-15
GLS	VPS4B	0.54954218	1.96E-15
GLS	VDAC3	0.54983875	1.88E-15
GLS	USP10	0.55003063	1.83E-15
GLS	GCC2	0.5500716	1.82E-15
GLS	FCHO2	0.55012464	1.81E-15
GLS	WASHC2A	0.55014753	1.8E-15
GLS	CYREN	0.55018328	1.79E-15
GLS	PHF20	0.55031122	1.76E-15
GLS	SLAIN2	0.55038872	1.74E-15
GLS	DCP2	0.55048192	1.72E-15
GLS	RSRC1	0.55058556	1.69E-15
GLS	RPRD2	0.55064904	1.68E-15
GLS	ADAT1	0.55070246	1.67E-15
GLS	RBM43	0.55102077	1.59E-15
GLS	PLEKHF2	0.55106635	1.58E-15
GLS	ZNF766	0.5510976	1.58E-15
GLS	PAK2	0.55114245	1.57E-15
GLS	NUDCD1	0.5512097	1.55E-15
GLS	CCDC90B	0.55122611	1.55E-15
GLS	C12orf43	0.55126463	1.54E-15
GLS	SKI	0.5513189	1.53E-15
GLS	TYW1	0.55146521	1.5E-15
GLS	GNAI2	0.5515148	1.49E-15
GLS	TRIQK	0.55160648	1.47E-15
GLS	PLEKHA3	0.55165965	1.46E-15
GLS	TRAK2	0.5516933	1.45E-15
GLS	ITGA4	0.55176813	1.43E-15
GLS	ARL6IP6	0.55188972	1.41E-15
GLS	URI1	0.55202201	1.38E-15
GLS	NUMBL	0.55208875	1.37E-15
GLS	DHX35	0.55226856	1.34E-15
GLS	LIMCH1	0.55242454	1.31E-15
GLS	RBMS1	0.55251072	1.29E-15
GLS	NUDT4	0.55257425	1.28E-15
GLS	ARL1	0.55259999	1.27E-15
GLS	RAB5C	0.55279878	1.24E-15
GLS	GPRIN1	0.55291359	1.22E-15
GLS	GLOD4	0.55292935	1.22E-15
GLS	NKIRAS1	0.55304477	1.2E-15
GLS	CCDC92	0.55321113	1.17E-15

GLS	ZCCHC3	0.55334648	1.15E-15
GLS	MORF4L1	0.55347726	1.13E-15
GLS	B3GALNT1	0.55351864	1.12E-15
GLS	CCDC102B	0.55365714	1.1E-15
GLS	HIVEP1	0.5537112	1.09E-15
GLS	SET	0.55381389	1.07E-15
GLS	SYNJ2BP	0.5538481	1.07E-15
GLS	PNPO	0.55395309	1.05E-15
GLS	PCMT1	0.55406114	1.04E-15
GLS	TRMT6	0.5541081	1.03E-15
GLS	ASB7	0.55414169	1.02E-15
GLS	LAMB1	0.55430995	9.99E-16
GLS	COPG2	0.55434224	9.95E-16
GLS	ANKLE2	0.55440181	9.86E-16
GLS	SLC4A7	0.55447631	9.76E-16
GLS	NDUFA5	0.55463215	9.54E-16
GLS	ANKRD27	0.55463336	9.54E-16
GLS	PPTC7	0.55474471	9.39E-16
GLS	SGCE	0.55476753	9.36E-16
GLS	TIAM2	0.55504448	9E-16
GLS	CNST	0.55506289	8.97E-16
GLS	UBXN2B	0.55509748	8.93E-16
GLS	SELENOF	0.55565235	8.25E-16
GLS	DR1	0.55570284	8.19E-16
GLS	EFCAB14	0.55570873	8.18E-16
GLS	FBXO7	0.55584667	8.02E-16
GLS	FBXL2	0.55584938	8.02E-16
GLS	SPART	0.55595885	7.89E-16
GLS	PTPN9	0.55621117	7.61E-16
GLS	AKIRIN2	0.55623995	7.58E-16
GLS	COMMD2	0.55627873	7.54E-16
GLS	INTS12	0.55645748	7.35E-16
GLS	TMEM19	0.55662375	7.18E-16
GLS	KDM2A	0.55670915	7.09E-16
GLS	PPM1A	0.55681995	6.98E-16
GLS	LDAH	0.55717274	6.63E-16
GLS	RBM18	0.55733149	6.48E-16
GLS	EAF1	0.55747353	6.35E-16
GLS	FAM98A	0.55749992	6.33E-16
GLS	DCUN1D1	0.55755762	6.27E-16
GLS	RPA1	0.55763434	6.2E-16
GLS	PLSCR4	0.55766409	6.18E-16
GLS	IST1	0.5578781	5.99E-16
GLS	ERBIN	0.55816464	5.75E-16
GLS	ZNF512	0.55817335	5.74E-16
GLS	SCAF11	0.55831082	5.63E-16
GLS	CERKL	0.55833354	5.61E-16
GLS	DZIP3	0.55853421	5.45E-16
GLS	DLD	0.55867667	5.34E-16
GLS	ERGIC2	0.5586814	5.33E-16
GLS	TBL1X	0.55873817	5.29E-16
GLS	SAR1B	0.55885513	5.2E-16
GLS	TSN	0.55896928	5.12E-16
GLS	ENPEP	0.55910564	5.02E-16
GLS	ARL6IP5	0.55914137	4.99E-16
GLS	TSNAX	0.55923049	4.93E-16
GLS	PACRGL	0.55934671	4.84E-16
GLS	SEMA5A	0.55949963	4.74E-16

GLS	OPA3	0.55969816	4.6E-16
GLS	DLG1	0.55971977	4.59E-16
GLS	TUSC3	0.55980537	4.53E-16
GLS	JHY	0.55985871	4.5E-16
GLS	LARS2	0.55989882	4.47E-16
GLS	SYNJ2	0.55991326	4.46E-16
GLS	IFT57	0.55991688	4.46E-16
GLS	CCAR2	0.55993718	4.45E-16
GLS	FAM20B	0.56005132	4.37E-16
GLS	HNRNPLL	0.56009126	4.35E-16
GLS	ZNF532	0.56009176	4.35E-16
GLS	HNRNPUL1	0.56009314	4.35E-16
GLS	VHL	0.56020976	4.27E-16
GLS	UVRAG	0.56028681	4.23E-16
GLS	BCORL1	0.56029569	4.22E-16
GLS	HACD2	0.56042051	4.14E-16
GLS	KATNBL1	0.56048342	4.11E-16
GLS	RAP1A	0.56051936	4.08E-16
GLS	CUL1	0.5605694	4.06E-16
GLS	EXOC6B	0.56071972	3.97E-16
GLS	USP11	0.56077309	3.94E-16
GLS	CCNK	0.56088597	3.87E-16
GLS	ZNF250	0.56099622	3.81E-16
GLS	BPGM	0.56114382	3.73E-16
GLS	SRGAP2C	0.56145402	3.56E-16
GLS	RCAN2	0.56145683	3.56E-16
GLS	NARS2	0.56146616	3.56E-16
GLS	AP1S2	0.56152923	3.53E-16
GLS	PTEN	0.56154765	3.52E-16
GLS	ATXN10	0.56164999	3.46E-16
GLS	SMNDC1	0.56196743	3.31E-16
GLS	KIAA0586	0.56207508	3.26E-16
GLS	DNAJA2	0.56209171	3.25E-16
GLS	ITGA1	0.56217111	3.21E-16
GLS	LBH	0.56223395	3.18E-16
GLS	PSMD10	0.56240859	3.1E-16
GLS	NDEL1	0.56253331	3.04E-16
GLS	NKRF	0.56254518	3.04E-16
GLS	RBBP5	0.56260432	3.01E-16
GLS	SFT2D2	0.56278339	2.93E-16
GLS	BZW1	0.56289427	2.89E-16
GLS	ANP32E	0.56292846	2.87E-16
GLS	GNB1	0.5630797	2.81E-16
GLS	ISCA1	0.56332768	2.71E-16
GLS	NPM1	0.56344178	2.66E-16
GLS	DPF2	0.5634847	2.65E-16
GLS	USP14	0.56350425	2.64E-16
GLS	FER	0.56353962	2.63E-16
GLS	ARL2BP	0.56359135	2.61E-16
GLS	SURF4	0.56365425	2.58E-16
GLS	NRP1	0.5636873	2.57E-16
GLS	PANK2	0.56380064	2.53E-16
GLS	PRDX3	0.56392962	2.48E-16
GLS	SNX3	0.56409459	2.42E-16
GLS	NCDN	0.56413053	2.41E-16
GLS	STT3B	0.56424291	2.37E-16
GLS	ZMAT2	0.56438256	2.32E-16
GLS	DLC1	0.5644069	2.31E-16

GLS	LMAN2L	0.56454119	2.27E-16
GLS	DCAF1	0.56463986	2.23E-16
GLS	MED4	0.56470156	2.21E-16
GLS	AP1AR	0.56470846	2.21E-16
GLS	LRRFIP1	0.5647475	2.2E-16
GLS	SLC35A4	0.56489898	2.15E-16
GLS	NUMB	0.56501946	2.11E-16
GLS	PNRC2	0.56518676	2.06E-16
GLS	EMC1	0.56518992	2.06E-16
GLS	ZNF281	0.56523998	2.04E-16
GLS	KLHL5	0.56533844	2.01E-16
GLS	TGFBR2	0.56536353	2.01E-16
GLS	AP2B1	0.56556066	1.95E-16
GLS	LAMTOR3	0.56567298	1.92E-16
GLS	AKIRIN1	0.56577539	1.89E-16
GLS	ZNF226	0.56590968	1.85E-16
GLS	MED28	0.56592684	1.85E-16
GLS	DNAL1	0.56600381	1.82E-16
GLS	TAF1B	0.5660527	1.81E-16
GLS	MRPS27	0.56613582	1.79E-16
GLS	NAA20	0.5664758	1.7E-16
GLS	RCBTB2	0.56649735	1.7E-16
GLS	RNF2	0.56650003	1.7E-16
GLS	HNRNPR	0.56665035	1.66E-16
GLS	CADM1	0.5667144	1.64E-16
GLS	TCF4	0.56676843	1.63E-16
GLS	ZCRB1	0.56694926	1.59E-16
GLS	CBFB	0.56700136	1.57E-16
GLS	ANKRD50	0.56702184	1.57E-16
GLS	VEZT	0.56704073	1.56E-16
GLS	FOXN3	0.56706614	1.56E-16
GLS	AC010132.3	0.56711022	1.55E-16
GLS	CNPY4	0.56729572	1.51E-16
GLS	TM9SF2	0.56731821	1.5E-16
GLS	SLC9A7	0.56732593	1.5E-16
GLS	RPTOR	0.56739587	1.48E-16
GLS	CLIP3	0.56748409	1.46E-16
GLS	YPEL5	0.5676993	1.42E-16
GLS	ZBTB2	0.56772943	1.41E-16
GLS	HBP1	0.56793102	1.37E-16
GLS	ESD	0.56797053	1.36E-16
GLS	PTPRG	0.5679811	1.36E-16
GLS	XPR1	0.56803551	1.35E-16
GLS	PLXNC1	0.56811662	1.33E-16
GLS	HNRNPU	0.56814206	1.33E-16
GLS	ZNF131	0.56817254	1.32E-16
GLS	CAMK2G	0.56822799	1.31E-16
GLS	CCNY	0.568271	1.3E-16
GLS	ATP6V1D	0.56841788	1.27E-16
GLS	RUFY1	0.56843469	1.27E-16
GLS	IGF2R	0.56853699	1.25E-16
GLS	TFE3	0.56872638	1.22E-16
GLS	HSDL1	0.56884543	1.2E-16
GLS	SYNCRIP	0.56891353	1.18E-16
GLS	OGFOD1	0.56896668	1.17E-16
GLS	HABP4	0.56902338	1.16E-16
GLS	ARL8B	0.56902734	1.16E-16
GLS	HEG1	0.56908005	1.15E-16

GLS	ZNF143	0.56915144	1.14E-16
GLS	EDC3	0.56945795	1.09E-16
GLS	KIFAP3	0.56952168	1.08E-16
GLS	CFAP20	0.56957937	1.07E-16
GLS	C2CD3	0.56958023	1.07E-16
GLS	NUAK1	0.56959326	1.07E-16
GLS	ACSL4	0.56961009	1.07E-16
GLS	PPM1B	0.56962011	1.06E-16
GLS	CCNG2	0.56966597	1.06E-16
GLS	TSPAN31	0.5697031	1.05E-16
GLS	USP9X	0.5698119	1.03E-16
GLS	PWP1	0.56988684	1.02E-16
GLS	TAOK3	0.56996691	1.01E-16
GLS	DSE	0.57006316	9.95E-17
GLS	ERC1	0.57025912	9.67E-17
GLS	CD99L2	0.57028243	9.63E-17
GLS	DIDO1	0.57029611	9.61E-17
GLS	RBBP4	0.57032291	9.57E-17
GLS	FBXO8	0.57041879	9.44E-17
GLS	RBM7	0.57042409	9.43E-17
GLS	POLR2C	0.57044886	9.39E-17
GLS	PNMA2	0.57045337	9.39E-17
GLS	LRRC8C	0.57058342	9.21E-17
GLS	EXT2	0.57071105	9.03E-17
GLS	BRD3	0.57072252	9.01E-17
GLS	TMEM170A	0.57072886	9.01E-17
GLS	TMCC3	0.57095545	8.7E-17
GLS	UBE2N	0.57098991	8.66E-17
GLS	KIF3B	0.57111936	8.49E-17
GLS	TWSG1	0.57118481	8.41E-17
GLS	TRIM32	0.57139273	8.15E-17
GLS	ZNF416	0.57161961	7.87E-17
GLS	CCDC47	0.57173512	7.74E-17
GLS	PIGS	0.57183873	7.62E-17
GLS	SETD7	0.57184023	7.61E-17
GLS	SLC30A9	0.57194175	7.5E-17
GLS	NAA15	0.57249344	6.9E-17
GLS	TMEM106B	0.57264829	6.74E-17
GLS	SBF2	0.57266874	6.72E-17
GLS	INIP	0.57273962	6.65E-17
GLS	TBC1D25	0.57320941	6.19E-17
GLS	CDK5R1	0.57345334	5.96E-17
GLS	TXNRD1	0.57348178	5.94E-17
GLS	RAD23B	0.57361261	5.82E-17
GLS	QTRT2	0.57397525	5.51E-17
GLS	ABCB10	0.57400895	5.48E-17
GLS	IFIT5	0.57417495	5.34E-17
GLS	MAP2K1	0.57418453	5.33E-17
GLS	USP1	0.57419876	5.32E-17
GLS	PEX3	0.57442734	5.14E-17
GLS	TMEM237	0.57447125	5.11E-17
GLS	EMC2	0.5745056	5.08E-17
GLS	SGPL1	0.57457337	5.03E-17
GLS	ARMC8	0.57460912	5E-17
GLS	ZNF350	0.57475584	4.89E-17
GLS	ANAPC10	0.5748028	4.85E-17
GLS	NAA16	0.57505306	4.67E-17
GLS	ATP2C1	0.57506419	4.66E-17

GLS	UBA3	0.57528938	4.51E-17
GLS	TFAM	0.57533108	4.48E-17
GLS	APBB2	0.57542233	4.42E-17
GLS	TRIP12	0.57543449	4.41E-17
GLS	SLC25A17	0.57550799	4.36E-17
GLS	CSDE1	0.57559112	4.3E-17
GLS	GPN3	0.57569771	4.23E-17
GLS	YIPF6	0.57601274	4.03E-17
GLS	SMIM13	0.57614054	3.95E-17
GLS	RCOR1	0.57616077	3.94E-17
GLS	TADA1	0.57636173	3.82E-17
GLS	MEGF9	0.57644988	3.77E-17
GLS	MXRA7	0.57663158	3.67E-17
GLS	CDK19	0.57667925	3.64E-17
GLS	TCTN2	0.57677272	3.59E-17
GLS	SLU7	0.57680742	3.57E-17
GLS	GLYR1	0.57743989	3.24E-17
GLS	RNF20	0.57748391	3.22E-17
GLS	CREB1	0.57777414	3.08E-17
GLS	NEURL1B	0.57783062	3.05E-17
GLS	RAB8B	0.57786708	3.03E-17
GLS	ARIH1	0.57805832	2.94E-17
GLS	MAST4	0.57811027	2.92E-17
GLS	PPIG	0.57821606	2.87E-17
GLS	HNRNPA2B1	0.57830066	2.84E-17
GLS	KDM4A	0.57830675	2.83E-17
GLS	PAM	0.57855247	2.73E-17
GLS	ZNF207	0.57863019	2.69E-17
GLS	KPNA6	0.57868563	2.67E-17
GLS	SPTLC1	0.57889754	2.59E-17
GLS	PRKAG2	0.5789225	2.58E-17
GLS	CNOT8	0.5789408	2.57E-17
GLS	ADAR	0.5798405	2.23E-17
GLS	LLGL1	0.57989596	2.21E-17
GLS	SCFD2	0.58007096	2.15E-17
GLS	KCTD20	0.58062444	1.98E-17
GLS	UBE3C	0.58083383	1.91E-17
GLS	TMEM68	0.58093166	1.88E-17
GLS	GNB5	0.58108516	1.84E-17
GLS	TBC1D14	0.58112236	1.83E-17
GLS	CKAP5	0.5811279	1.83E-17
GLS	RSF1	0.58117713	1.81E-17
GLS	RO60	0.5813477	1.77E-17
GLS	DNAJC18	0.58136157	1.76E-17
GLS	RNF121	0.581418	1.75E-17
GLS	TULP3	0.58155783	1.71E-17
GLS	SDE2	0.58167869	1.68E-17
GLS	ELF1	0.58173961	1.66E-17
GLS	NAP1L3	0.58176907	1.65E-17
GLS	TM2D3	0.5818654	1.63E-17
GLS	YIPF5	0.58196026	1.6E-17
GLS	FBXW2	0.58199115	1.6E-17
GLS	CNOT6L	0.58218738	1.55E-17
GLS	DDX50	0.58225637	1.53E-17
GLS	LEPROT	0.58247995	1.48E-17
GLS	DLST	0.58254759	1.46E-17
GLS	CNOT2	0.58267955	1.43E-17
GLS	ZNF286A	0.58276026	1.42E-17

GLS	PLEKHB2	0.58281136	1.4E-17
GLS	FOXO1	0.5828338	1.4E-17
GLS	TFCP2	0.58292414	1.38E-17
GLS	MED17	0.58304185	1.35E-17
GLS	NIN	0.58325567	1.31E-17
GLS	IFT52	0.58333686	1.29E-17
GLS	DYNC1LI1	0.58334858	1.29E-17
GLS	OSBPL11	0.58346424	1.27E-17
GLS	PSIP1	0.58348137	1.26E-17
GLS	MPP5	0.58358264	1.24E-17
GLS	RP2	0.58382866	1.2E-17
GLS	ZNF462	0.58401758	1.16E-17
GLS	GSTCD	0.58405556	1.15E-17
GLS	SLC15A4	0.58415221	1.14E-17
GLS	KLHL20	0.58424634	1.12E-17
GLS	GNS	0.58448363	1.08E-17
GLS	QKI	0.58461499	1.06E-17
GLS	JAK1	0.58462048	1.06E-17
GLS	UBE2Q2	0.5847142	1.04E-17
GLS	KCTD2	0.58478475	1.03E-17
GLS	PRKD3	0.58508452	9.81E-18
GLS	KPNA1	0.58512037	9.76E-18
GLS	BMI1	0.58542103	9.3E-18
GLS	SUSD6	0.58542862	9.29E-18
GLS	TAF7	0.58559791	9.05E-18
GLS	ANKH	0.58579794	8.77E-18
GLS	CSGALNACT1	0.58635469	8.02E-18
GLS	ABL1	0.58637883	7.99E-18
GLS	RTL5	0.58658049	7.74E-18
GLS	STIM1	0.58661783	7.69E-18
GLS	MED6	0.58678516	7.49E-18
GLS	MAN2B2	0.58685027	7.42E-18
GLS	FAM220A	0.58714715	7.07E-18
GLS	UBE2D2	0.5873605	6.84E-18
GLS	JMJD1C	0.58742685	6.76E-18
GLS	CSNK2A1	0.58751503	6.67E-18
GLS	MAPK14	0.58782121	6.35E-18
GLS	CYB5R4	0.58786695	6.31E-18
GLS	ATAD1	0.58821896	5.96E-18
GLS	ASH2L	0.58825939	5.92E-18
GLS	VTA1	0.58846608	5.73E-18
GLS	ADGRA2	0.58854654	5.66E-18
GLS	SF3A1	0.5886404	5.57E-18
GLS	WASL	0.58869886	5.52E-18
GLS	HOMER1	0.58890612	5.34E-18
GLS	IPO11	0.58892058	5.33E-18
GLS	NBR1	0.58912194	5.16E-18
GLS	INO80	0.58920086	5.09E-18
GLS	ESYT2	0.58922478	5.07E-18
GLS	SLC30A5	0.58932114	5E-18
GLS	RDH11	0.58948971	4.86E-18
GLS	PRICKLE1	0.58953209	4.83E-18
GLS	TNFRSF19	0.58991369	4.54E-18
GLS	ATP6V1A	0.59011725	4.4E-18
GLS	GPR107	0.59014653	4.37E-18
GLS	DNAJB6	0.5901504	4.37E-18
GLS	TMEM167A	0.59031165	4.26E-18
GLS	TFDP1	0.59038102	4.21E-18

GLS	NUDT4B	0.59039657	4.2E-18
GLS	RTF1	0.59060075	4.07E-18
GLS	KCTD10	0.59060315	4.06E-18
GLS	PRKDC	0.59063375	4.04E-18
GLS	BTN2A1	0.59073239	3.98E-18
GLS	RAD1	0.59079895	3.94E-18
GLS	MAP1A	0.591007	3.81E-18
GLS	LRIF1	0.59116608	3.71E-18
GLS	CD200	0.59129598	3.63E-18
GLS	HMGXB4	0.59130101	3.63E-18
GLS	BCL2L13	0.59138307	3.58E-18
GLS	SNRNP27	0.5914852	3.52E-18
GLS	SLC39A9	0.59151939	3.51E-18
GLS	RPAP3	0.59155613	3.48E-18
GLS	UBE2V2	0.59174176	3.38E-18
GLS	PEX2	0.59192699	3.28E-18
GLS	RC3H2	0.59196898	3.26E-18
GLS	DDX3X	0.59200992	3.24E-18
GLS	DLAT	0.5922216	3.13E-18
GLS	VASH1	0.59231513	3.08E-18
GLS	TCEA1	0.59266315	2.91E-18
GLS	ZNF562	0.59270733	2.89E-18
GLS	PPP1R9B	0.59323348	2.65E-18
GLS	DST	0.59325269	2.65E-18
GLS	WNK1	0.59326703	2.64E-18
GLS	CLDND1	0.59335174	2.6E-18
GLS	HMGXB3	0.59350963	2.54E-18
GLS	GNAS	0.59358119	2.51E-18
GLS	ALS2	0.5937047	2.46E-18
GLS	TCEAL4	0.59378954	2.42E-18
GLS	RNF139	0.59414158	2.29E-18
GLS	CRLF3	0.59427112	2.24E-18
GLS	CNIH1	0.59431904	2.22E-18
GLS	WDFY1	0.59444418	2.18E-18
GLS	TVP23B	0.59454858	2.14E-18
GLS	MAPK1	0.5947149	2.08E-18
GLS	ORC4	0.59474027	2.08E-18
GLS	MLH1	0.59478621	2.06E-18
GLS	HECW2	0.59484967	2.04E-18
GLS	CAMSAP2	0.59485178	2.04E-18
GLS	GDF11	0.59494181	2.01E-18
GLS	CROT	0.59497595	2E-18
GLS	ZNHIT6	0.59503128	1.98E-18
GLS	ETS1	0.59529979	1.89E-18
GLS	SNX24	0.59537791	1.87E-18
GLS	SRSF1	0.59541726	1.86E-18
GLS	C16orf87	0.59550489	1.83E-18
GLS	SLC25A12	0.5955513	1.82E-18
GLS	JARID2	0.59569861	1.77E-18
GLS	PRRC1	0.59580146	1.74E-18
GLS	ADAM10	0.5959774	1.69E-18
GLS	TLK2	0.59601237	1.68E-18
GLS	LRRC8D	0.59601409	1.68E-18
GLS	FXR1	0.59613081	1.65E-18
GLS	NPAT	0.59616342	1.64E-18
GLS	PPP1R2	0.59679576	1.48E-18
GLS	TMEM167B	0.59691886	1.45E-18
GLS	UBA6	0.59692453	1.45E-18

GLS	DIS3	0.59703915	1.42E-18
GLS	USP34	0.59705335	1.42E-18
GLS	IDH3A	0.59706442	1.42E-18
GLS	CWF19L2	0.59722937	1.38E-18
GLS	MTMR2	0.59733681	1.35E-18
GLS	JADE2	0.59737724	1.35E-18
GLS	TSPAN2	0.59763665	1.29E-18
GLS	CRTC3	0.59764561	1.29E-18
GLS	SKP1	0.59785974	1.24E-18
GLS	MAFG	0.59790382	1.23E-18
GLS	FKBP15	0.59791619	1.23E-18
GLS	KLHL2	0.59805131	1.2E-18
GLS	TRANK1	0.59811001	1.19E-18
GLS	SEC23A	0.59841144	1.13E-18
GLS	GRSF1	0.59864684	1.09E-18
GLS	GOLGA8N	0.59878824	1.07E-18
GLS	ABRAXAS2	0.59898512	1.03E-18
GLS	LCORL	0.59902016	1.03E-18
GLS	SSX2IP	0.59916968	1E-18
GLS	SNX4	0.59917589	9.99E-19
GLS	CXorf56	0.5991938	9.96E-19
GLS	MTDH	0.59948757	9.49E-19
GLS	ALG11	0.59955487	9.38E-19
GLS	SNTB2	0.59960441	9.3E-19
GLS	GOLT1B	0.59991942	8.83E-19
GLS	HUS1	0.59992811	8.81E-19
GLS	CYP2U1	0.59996965	8.75E-19
GLS	SPIRE1	0.60011412	8.55E-19
GLS	PAFAH1B1	0.60023626	8.37E-19
GLS	RAB14	0.60035822	8.21E-19
GLS	PICALM	0.6004915	8.02E-19
GLS	PEX19	0.60049582	8.02E-19
GLS	RRN3	0.60073911	7.7E-19
GLS	USP8	0.60075399	7.68E-19
GLS	UBQLN1	0.60079837	7.62E-19
GLS	KTN1	0.60084824	7.56E-19
GLS	OSGIN2	0.60088906	7.51E-19
GLS	CWC22	0.60117408	7.16E-19
GLS	ALKBH1	0.60122529	7.1E-19
GLS	TWF1	0.60127866	7.04E-19
GLS	GNL3L	0.60143735	6.85E-19
GLS	TOP1	0.60179357	6.45E-19
GLS	MED20	0.6018385	6.41E-19
GLS	RNF4	0.60193822	6.3E-19
GLS	C12orf65	0.6019974	6.24E-19
GLS	ITGB1	0.60204277	6.19E-19
GLS	PGGT1B	0.60226327	5.96E-19
GLS	YME1L1	0.60227514	5.95E-19
GLS	DDX19A	0.60230716	5.92E-19
GLS	NCKAP1	0.60261304	5.62E-19
GLS	RTL6	0.60268354	5.56E-19
GLS	DENND6A	0.60277223	5.48E-19
GLS	PDE4DIP	0.60283937	5.41E-19
GLS	GNA13	0.60304125	5.23E-19
GLS	ACVR1	0.60320954	5.09E-19
GLS	KRR1	0.60341135	4.92E-19
GLS	NEK7	0.60348119	4.86E-19
GLS	MORF4L2	0.60360416	4.76E-19

GLS	KCTD9	0.60375797	4.64E-19
GLS	DCTN6	0.60387615	4.55E-19
GLS	GNPDA1	0.603936	4.5E-19
GLS	SP2	0.60394519	4.49E-19
GLS	OSTM1	0.60416022	4.33E-19
GLS	IRF2BP2	0.60446852	4.11E-19
GLS	GTF3C4	0.60466432	3.98E-19
GLS	CSGALNACT2	0.60486999	3.84E-19
GLS	VIPAS39	0.60492384	3.81E-19
GLS	C1D	0.60505748	3.72E-19
GLS	HAUS2	0.60516526	3.65E-19
GLS	DCAF7	0.60519385	3.64E-19
GLS	ZEB1	0.60539652	3.51E-19
GLS	MSL2	0.60588066	3.24E-19
GLS	RECK	0.60595043	3.2E-19
GLS	ZMYM5	0.60600606	3.17E-19
GLS	CETN3	0.60608768	3.12E-19
GLS	APMAP	0.60648856	2.92E-19
GLS	EDIL3	0.60668142	2.82E-19
GLS	G3BP2	0.60679931	2.77E-19
GLS	MBNL1	0.60692421	2.71E-19
GLS	LIX1L	0.60697451	2.69E-19
GLS	SPDL1	0.60704053	2.66E-19
GLS	FNBP1L	0.60705358	2.65E-19
GLS	IQSEC1	0.60707274	2.64E-19
GLS	CEP250	0.60714462	2.61E-19
GLS	GLT8D1	0.60763807	2.4E-19
GLS	LDB1	0.60776678	2.35E-19
GLS	ZNF821	0.60780859	2.33E-19
GLS	LMBR1	0.60795471	2.27E-19
GLS	POT1	0.60813276	2.2E-19
GLS	RAB6A	0.60825335	2.16E-19
GLS	GALC	0.60826861	2.15E-19
GLS	KIF13A	0.60886887	1.94E-19
GLS	UBLCP1	0.60923218	1.82E-19
GLS	MAP1B	0.60932118	1.8E-19
GLS	PIK3CA	0.60946537	1.75E-19
GLS	TM2D1	0.60958163	1.72E-19
GLS	NOCT	0.60966519	1.69E-19
GLS	LYRM2	0.60982417	1.65E-19
GLS	CDON	0.61028468	1.52E-19
GLS	TOPORS	0.61036248	1.5E-19
GLS	UBE2D3	0.61056913	1.45E-19
GLS	KLF7	0.61060055	1.44E-19
GLS	CTCF	0.61084041	1.38E-19
GLS	MYO5A	0.61087559	1.37E-19
GLS	ENPP2	0.61098137	1.35E-19
GLS	RNF170	0.6112925	1.28E-19
GLS	FAM98B	0.61134631	1.27E-19
GLS	LDB2	0.61149719	1.23E-19
GLS	MINDY2	0.6116369	1.2E-19
GLS	NCOA4	0.61169754	1.19E-19
GLS	CGRRF1	0.61176657	1.18E-19
GLS	CUL2	0.61222228	1.09E-19
GLS	COPS4	0.61227292	1.08E-19
GLS	CNTN1	0.61237713	1.06E-19
GLS	PPT1	0.61266349	1.01E-19
GLS	MTPN	0.61298103	9.53E-20

GLS	OCIAD1	0.61305365	9.41E-20
GLS	CDKN1B	0.61314473	9.26E-20
GLS	PPP1CB	0.61315768	9.24E-20
GLS	ZC3H11A	0.61315965	9.23E-20
GLS	DLG4	0.6131859	9.19E-20
GLS	BTBD9	0.61321736	9.14E-20
GLS	ATP6V1C1	0.61323057	9.12E-20
GLS	JAK2	0.61336965	8.9E-20
GLS	MAGT1	0.61340618	8.85E-20
GLS	GFOD2	0.61352238	8.67E-20
GLS	TOX4	0.61389155	8.13E-20
GLS	LRCH3	0.61396686	8.02E-20
GLS	PIGW	0.61413696	7.78E-20
GLS	VAMP7	0.61417246	7.74E-20
GLS	SOGA1	0.61429855	7.57E-20
GLS	COPS8	0.6143928	7.44E-20
GLS	SOCS6	0.61453862	7.26E-20
GLS	FAM200A	0.61492629	6.78E-20
GLS	FAM91A1	0.61517061	6.49E-20
GLS	CALM1	0.6155277	6.1E-20
GLS	VPS33A	0.61556142	6.06E-20
GLS	SLC25A40	0.61575448	5.86E-20
GLS	KIF3C	0.6159161	5.69E-20
GLS	KDM3B	0.61596687	5.64E-20
GLS	VMA21	0.61598784	5.62E-20
GLS	RASAL2	0.61610678	5.51E-20
GLS	TMEM263	0.61619195	5.42E-20
GLS	PLCG1	0.61646971	5.17E-20
GLS	RABL3	0.61658265	5.06E-20
GLS	SNW1	0.61663906	5.01E-20
GLS	RAB18	0.61673416	4.93E-20
GLS	SNX18	0.61686099	4.82E-20
GLS	ZNF609	0.6168905	4.79E-20
GLS	STAM	0.61705313	4.66E-20
GLS	YY1	0.61727476	4.48E-20
GLS	TSPYL1	0.61765968	4.18E-20
GLS	SFXN1	0.61825012	3.77E-20
GLS	FCHSD2	0.61829131	3.74E-20
GLS	NCOA2	0.61829796	3.74E-20
GLS	HDGFL3	0.61854097	3.58E-20
GLS	PPP1R8	0.61903769	3.28E-20
GLS	TMEM200A	0.61932117	3.11E-20
GLS	MYH10	0.61933799	3.1E-20
GLS	EXTL3	0.61941382	3.06E-20
GLS	BBS2	0.61964831	2.94E-20
GLS	PPP2CA	0.62012794	2.7E-20
GLS	DOCK7	0.62014543	2.69E-20
GLS	ARMT1	0.62041215	2.56E-20
GLS	NBN	0.62066882	2.45E-20
GLS	PRMT2	0.62068461	2.44E-20
GLS	LAMP2	0.62128975	2.19E-20
GLS	PCYOX1	0.62171152	2.03E-20
GLS	PDCL	0.6218353	1.98E-20
GLS	ASXL2	0.62188504	1.97E-20
GLS	PTPRB	0.62198588	1.93E-20
GLS	ARL14EP	0.62211863	1.89E-20
GLS	FYTTD1	0.62241325	1.79E-20
GLS	KIAA1191	0.62269677	1.7E-20

GLS	CUL4A	0.62273175	1.69E-20
GLS	ADGRF5	0.62281097	1.66E-20
GLS	SNAPC1	0.62311616	1.57E-20
GLS	TRIM37	0.62322559	1.54E-20
GLS	GNAI1	0.62327007	1.53E-20
GLS	YEATS2	0.62328527	1.53E-20
GLS	CDC5L	0.62334253	1.51E-20
GLS	SMAD1	0.62346069	1.48E-20
GLS	PPHLN1	0.62362224	1.44E-20
GLS	KLHL7	0.62374721	1.4E-20
GLS	FAM171B	0.62399586	1.34E-20
GLS	GPBP1	0.62416653	1.3E-20
GLS	GBE1	0.62433358	1.26E-20
GLS	PALLD	0.62440185	1.25E-20
GLS	EXOC2	0.62450011	1.23E-20
GLS	THRAP3	0.62474588	1.17E-20
GLS	NSL1	0.62480302	1.16E-20
GLS	RBM22	0.62484876	1.15E-20
GLS	BRAP	0.62491623	1.14E-20
GLS	PPP2CB	0.62520144	1.08E-20
GLS	ZKSCAN5	0.62524088	1.07E-20
GLS	BAG5	0.62541163	1.04E-20
GLS	MFSD11	0.62596553	9.38E-21
GLS	ZNF623	0.62636033	8.73E-21
GLS	SSR1	0.62642857	8.62E-21
GLS	SLC35A5	0.62672045	8.17E-21
GLS	ITM2B	0.62682563	8.02E-21
GLS	ZMYM4	0.62685255	7.98E-21
GLS	ARHGEF12	0.62742656	7.18E-21
GLS	WDR89	0.62793146	6.54E-21
GLS	RBM12	0.62801434	6.44E-21
GLS	KCTD12	0.62818881	6.24E-21
GLS	VEGFC	0.62864439	5.74E-21
GLS	ZNF410	0.62865997	5.72E-21
GLS	ICE2	0.6287203	5.66E-21
GLS	XRN2	0.62887332	5.5E-21
GLS	NDRG3	0.62891874	5.45E-21
GLS	HPS5	0.62893617	5.44E-21
GLS	ARL5A	0.62898094	5.39E-21
GLS	ATP8B2	0.62910119	5.27E-21
GLS	MED21	0.62920972	5.17E-21
GLS	SNX6	0.62922167	5.16E-21
GLS	ZNF329	0.62922976	5.15E-21
GLS	RBBP9	0.62927734	5.1E-21
GLS	MIER1	0.62940764	4.98E-21
GLS	ATP13A3	0.62952247	4.88E-21
GLS	SETD3	0.62953803	4.86E-21
GLS	COMMD8	0.62970284	4.72E-21
GLS	AP5M1	0.62971576	4.71E-21
GLS	BNIP3L	0.62976353	4.67E-21
GLS	HNRNPK	0.62983476	4.6E-21
GLS	TBK1	0.62997846	4.48E-21
GLS	SHOC2	0.63019772	4.3E-21
GLS	TCAF1	0.63047193	4.09E-21
GLS	ZC2HC1A	0.63107703	3.66E-21
GLS	NUFIP2	0.63165211	3.28E-21
GLS	EID1	0.63212758	3.01E-21
GLS	DIP2B	0.63222136	2.95E-21

GLS	MICU2	0.63223367	2.95E-21
GLS	MBD5	0.63233653	2.89E-21
GLS	TNPO1	0.63243575	2.84E-21
GLS	ITFG1	0.63246878	2.82E-21
GLS	POFUT1	0.6325311	2.79E-21
GLS	DOCK9	0.63271669	2.69E-21
GLS	CDC42BPB	0.63289319	2.6E-21
GLS	FOXN2	0.6329589	2.57E-21
GLS	SLC4A1AP	0.63325617	2.43E-21
GLS	HIF1A	0.63330154	2.41E-21
GLS	PMS2	0.63331074	2.41E-21
GLS	CSTF2T	0.63333349	2.4E-21
GLS	TANC2	0.6334605	2.34E-21
GLS	SMARCA2	0.63363505	2.27E-21
GLS	SIN3A	0.63364958	2.26E-21
GLS	ZNF148	0.63371539	2.23E-21
GLS	CDC42SE2	0.63377837	2.21E-21
GLS	ZFYVE1	0.63392119	2.15E-21
GLS	TTC33	0.63395879	2.13E-21
GLS	VCPIP1	0.63415183	2.06E-21
GLS	TEFM	0.63422018	2.03E-21
GLS	ELOVL5	0.63422772	2.03E-21
GLS	RANBP9	0.63423271	2.02E-21
GLS	PITPNA	0.6343517	1.98E-21
GLS	SMAD7	0.63436723	1.97E-21
GLS	RAB28	0.63442115	1.95E-21
GLS	GNG2	0.63447664	1.93E-21
GLS	PNPLA8	0.63450758	1.92E-21
GLS	TERF2IP	0.63490171	1.79E-21
GLS	NPTN	0.63514263	1.71E-21
GLS	PPP1CC	0.63524109	1.67E-21
GLS	SP3	0.63550099	1.59E-21
GLS	MEX3C	0.63559692	1.57E-21
GLS	SNX16	0.63574965	1.52E-21
GLS	MACF1	0.63580087	1.51E-21
GLS	ZFP91	0.63602294	1.44E-21
GLS	ABL2	0.63604506	1.44E-21
GLS	SGCB	0.63605031	1.44E-21
GLS	TCTN3	0.63605581	1.43E-21
GLS	CTDSPL2	0.63608234	1.43E-21
GLS	ZNF720	0.6360934	1.42E-21
GLS	TTC26	0.63611612	1.42E-21
GLS	SMARCA1	0.63639871	1.34E-21
GLS	ABHD15	0.63654112	1.31E-21
GLS	TMEM230	0.63654508	1.31E-21
GLS	SEC14L1	0.63657148	1.3E-21
GLS	NAP1L1	0.6367253	1.26E-21
GLS	ZNF644	0.63684715	1.23E-21
GLS	ASAH1	0.63771635	1.05E-21
GLS	ZNF639	0.63793376	1E-21
GLS	PRUNE1	0.63796757	9.98E-22
GLS	PIK3R1	0.63822812	9.49E-22
GLS	STAT3	0.63852812	8.96E-22
GLS	CAPZA2	0.6386791	8.71E-22
GLS	LRP11	0.6387413	8.6E-22
GLS	TSHZ1	0.63892399	8.31E-22
GLS	CPSF2	0.63895131	8.26E-22
GLS	HIPK1	0.63897823	8.22E-22

GLS	RNF145	0.63907531	8.07E-22
GLS	KPNB1	0.63908897	8.05E-22
GLS	CTSO	0.63917807	7.91E-22
GLS	NAAA	0.63928811	7.75E-22
GLS	CHUK	0.63932681	7.69E-22
GLS	BBS12	0.63951192	7.42E-22
GLS	ATP6V1B2	0.63984995	6.96E-22
GLS	CCSER2	0.63987611	6.92E-22
GLS	SMAD2	0.63993329	6.85E-22
GLS	C16orf72	0.64021496	6.48E-22
GLS	UBFD1	0.64025177	6.44E-22
GLS	SERINC3	0.64025744	6.43E-22
GLS	HIVEP2	0.64027177	6.41E-22
GLS	TBC1D5	0.64041516	6.24E-22
GLS	UPRT	0.64049285	6.15E-22
GLS	TXNDC15	0.64050958	6.13E-22
GLS	ZNF146	0.64094654	5.63E-22
GLS	CANX	0.64096537	5.61E-22
GLS	DYRK1A	0.64126067	5.3E-22
GLS	CAMSAP1	0.64128298	5.28E-22
GLS	ARHGAP35	0.64137052	5.19E-22
GLS	PPP4R3B	0.6418701	4.71E-22
GLS	KBTBD4	0.64207418	4.53E-22
GLS	ATP2A2	0.64243836	4.22E-22
GLS	WDR82	0.64248398	4.18E-22
GLS	RNF13	0.64250528	4.16E-22
GLS	RYBP	0.64257224	4.11E-22
GLS	HNRNPH2	0.64283483	3.91E-22
GLS	SLC12A6	0.64288857	3.87E-22
GLS	ADAM17	0.64306487	3.74E-22
GLS	KDM1B	0.64319265	3.64E-22
GLS	PNMA1	0.64320212	3.64E-22
GLS	PTPN1	0.64333079	3.55E-22
GLS	GPALPP1	0.64334949	3.53E-22
GLS	RTN4	0.64346559	3.46E-22
GLS	OAZ2	0.64367849	3.32E-22
GLS	SOCS5	0.64368956	3.31E-22
GLS	ARHGAP5	0.64387638	3.19E-22
GLS	CNOT4	0.64452017	2.81E-22
GLS	TMX4	0.64452882	2.81E-22
GLS	API5	0.64459043	2.77E-22
GLS	CLOCK	0.6446338	2.75E-22
GLS	ETF1	0.64471989	2.7E-22
GLS	PPP4R2	0.64510763	2.51E-22
GLS	HS2ST1	0.64513764	2.49E-22
GLS	CLN5	0.64524978	2.44E-22
GLS	ARRDC3	0.6453323	2.4E-22
GLS	SSH1	0.6455973	2.28E-22
GLS	PARG	0.64573415	2.22E-22
GLS	GOLGA7	0.64578041	2.2E-22
GLS	PIP4P2	0.64620716	2.02E-22
GLS	UBTD2	0.64638171	1.95E-22
GLS	DHX36	0.64658852	1.87E-22
GLS	SH3BGRL	0.64659088	1.87E-22
GLS	BBS9	0.6466807	1.84E-22
GLS	LARP7	0.64682019	1.79E-22
GLS	SESN3	0.64713691	1.68E-22
GLS	ARMC1	0.64720988	1.66E-22

GLS	CRKL	0.6475146	1.56E-22
GLS	PFKM	0.64751585	1.56E-22
GLS	MEAF6	0.64760175	1.53E-22
GLS	APP	0.6478225	1.47E-22
GLS	RIN2	0.64788723	1.45E-22
GLS	RAD21	0.64806809	1.4E-22
GLS	GANAB	0.6484215	1.3E-22
GLS	STK4	0.64849487	1.29E-22
GLS	MAPK1IP1L	0.64863748	1.25E-22
GLS	MMGT1	0.64866411	1.24E-22
GLS	WDR44	0.64877429	1.22E-22
GLS	ARL13B	0.64879419	1.21E-22
GLS	LCLAT1	0.64939514	1.07E-22
GLS	ZFAND5	0.64944221	1.06E-22
GLS	RPL7L1	0.64956276	1.04E-22
GLS	RAB3GAP1	0.64973717	1E-22
GLS	SH2B3	0.64988127	9.76E-23
GLS	SELENOT	0.64999801	9.53E-23
GLS	RLIM	0.65008188	9.37E-23
GLS	C16orf70	0.65027467	9.02E-23
GLS	CDK14	0.6502933	8.98E-23
GLS	GRAMD2B	0.65039279	8.81E-23
GLS	TAF2	0.65053022	8.57E-23
GLS	FEM1B	0.6505564	8.52E-23
GLS	AKT3	0.65079776	8.12E-23
GLS	LMBRD1	0.65086902	8.01E-23
GLS	PRPS1	0.65115906	7.56E-23
GLS	TAB2	0.65116092	7.55E-23
GLS	SPOP	0.65136668	7.25E-23
GLS	BBX	0.65161578	6.89E-23
GLS	KIAA0232	0.651743	6.72E-23
GLS	MED13	0.65212611	6.22E-23
GLS	ENOX2	0.65213699	6.21E-23
GLS	KIF2A	0.65221794	6.11E-23
GLS	MMD	0.65253226	5.73E-23
GLS	ZBTB4	0.65262036	5.63E-23
GLS	YWHAQ	0.6526755	5.57E-23
GLS	SPOPL	0.65269779	5.55E-23
GLS	MBNL2	0.65275595	5.48E-23
GLS	GNA12	0.65279371	5.44E-23
GLS	GCLM	0.65292608	5.3E-23
GLS	PRXL2C	0.65323034	4.98E-23
GLS	C5orf51	0.65325652	4.95E-23
GLS	RNGTT	0.65328354	4.93E-23
GLS	ZBTB6	0.65349386	4.72E-23
GLS	CNOT6	0.65355324	4.67E-23
GLS	CEP170	0.65399529	4.27E-23
GLS	TRAF3	0.65411436	4.16E-23
GLS	SCRN1	0.65422257	4.07E-23
GLS	SUZ12	0.65458645	3.78E-23
GLS	ORAI2	0.65462974	3.75E-23
GLS	EIF4G2	0.65475654	3.66E-23
GLS	CTBS	0.65504904	3.44E-23
GLS	ARL15	0.65510811	3.4E-23
GLS	ZNF134	0.65514282	3.38E-23
GLS	MIB1	0.65549029	3.15E-23
GLS	MBTPS1	0.65551317	3.13E-23
GLS	ACTR6	0.65579853	2.96E-23

GLS	RBM27	0.6560272	2.82E-23
GLS	CREB3L2	0.65613404	2.76E-23
GLS	CCNI	0.65628759	2.68E-23
GLS	GINM1	0.6565921	2.51E-23
GLS	CEP41	0.65673856	2.44E-23
GLS	MED1	0.65679525	2.41E-23
GLS	PAIP2	0.65681946	2.4E-23
GLS	EIF4G3	0.65681947	2.4E-23
GLS	ATF1	0.65689514	2.36E-23
GLS	PUM2	0.65709779	2.27E-23
GLS	SMURF2	0.65739918	2.13E-23
GLS	SMARCA5	0.65771997	2E-23
GLS	LIN54	0.65779215	1.97E-23
GLS	ZBTB38	0.65816168	1.82E-23
GLS	SPPL3	0.65818467	1.81E-23
GLS	RAB12	0.65826886	1.78E-23
GLS	EVI5	0.6585372	1.69E-23
GLS	RAB3GAP2	0.65880321	1.6E-23
GLS	RB1	0.65900235	1.53E-23
GLS	GPATCH11	0.65942106	1.4E-23
GLS	BTBD1	0.65943022	1.4E-23
GLS	TMTC3	0.65957335	1.36E-23
GLS	PCNX1	0.65969534	1.33E-23
GLS	TAF9B	0.65970186	1.33E-23
GLS	CYLD	0.6600308	1.24E-23
GLS	IL6ST	0.6602121	1.19E-23
GLS	KPNA3	0.6603278	1.16E-23
GLS	ABCC4	0.66035567	1.16E-23
GLS	ZBTB33	0.66044109	1.14E-23
GLS	FBXO38	0.66049682	1.12E-23
GLS	CHORDC1	0.66055391	1.11E-23
GLS	TPP2	0.66062234	1.1E-23
GLS	NSMCE3	0.66076283	1.06E-23
GLS	CDK12	0.66080264	1.05E-23
GLS	EHBP1	0.66087897	1.04E-23
GLS	FAF2	0.66098864	1.01E-23
GLS	ITPR1	0.6611768	9.76E-24
GLS	ATF7IP	0.6612997	9.51E-24
GLS	UTP14C	0.66140327	9.31E-24
GLS	BCL9	0.66181738	8.54E-24
GLS	UBA2	0.66236323	7.62E-24
GLS	ZNF45	0.66236414	7.62E-24
GLS	G3BP1	0.66237855	7.59E-24
GLS	TEAD1	0.6623876	7.58E-24
GLS	ZFAND3	0.66246923	7.45E-24
GLS	SMARCE1	0.66262893	7.21E-24
GLS	NUDT3	0.66296961	6.71E-24
GLS	TERF1	0.66309481	6.54E-24
GLS	DNM1L	0.66317075	6.43E-24
GLS	CPNE3	0.6636103	5.87E-24
GLS	PPP1R12A	0.66373885	5.71E-24
GLS	R3HDM1	0.66431467	5.06E-24
GLS	C11orf58	0.66441193	4.95E-24
GLS	SOCS4	0.66445536	4.91E-24
GLS	CBL	0.66455298	4.81E-24
GLS	CFAP97	0.66471491	4.65E-24
GLS	SCARB2	0.66475957	4.6E-24
GLS	AHCYL1	0.66481426	4.55E-24

GLS	RGS4	0.66484798	4.52E-24
GLS	USP38	0.6648968	4.47E-24
GLS	CIPC	0.66500515	4.37E-24
GLS	GLG1	0.66505641	4.32E-24
GLS	SRFBP1	0.66529388	4.11E-24
GLS	CSNK1A1	0.66540807	4.01E-24
GLS	DIP2C	0.66578965	3.7E-24
GLS	GOSR1	0.66616531	3.42E-24
GLS	RPRD1B	0.66623975	3.37E-24
GLS	RBFOX2	0.66624358	3.36E-24
GLS	FAM168A	0.66648056	3.2E-24
GLS	ATMIN	0.6672154	2.73E-24
GLS	DENR	0.66721931	2.73E-24
GLS	TPP1	0.6674937	2.58E-24
GLS	UBE2W	0.66766914	2.48E-24
GLS	TRRAP	0.66774399	2.44E-24
GLS	CAND1	0.66780431	2.41E-24
GLS	GTF2H3	0.66789135	2.37E-24
GLS	MAPRE1	0.66792099	2.35E-24
GLS	FBXL5	0.66796868	2.33E-24
GLS	UBQLN2	0.66807494	2.28E-24
GLS	TMED5	0.66818536	2.22E-24
GLS	SPATS2	0.66823329	2.2E-24
GLS	WDR20	0.6684242	2.11E-24
GLS	CALCOCO2	0.66865587	2.01E-24
GLS	KLF12	0.66884334	1.93E-24
GLS	NFE2L1	0.66885926	1.92E-24
GLS	ST13	0.66932747	1.74E-24
GLS	DDX6	0.66949259	1.68E-24
GLS	LRP12	0.66955018	1.66E-24
GLS	PPFIA1	0.67004696	1.49E-24
GLS	STX12	0.6708741	1.25E-24
GLS	SART3	0.67087446	1.25E-24
GLS	RAP2A	0.67098217	1.22E-24
GLS	LNPK	0.67107609	1.19E-24
GLS	SNX2	0.67119674	1.16E-24
GLS	SS18	0.67123031	1.15E-24
GLS	AP4S1	0.67175366	1.03E-24
GLS	PRNP	0.67185018	1.01E-24
GLS	IMPA1	0.67188258	1E-24
GLS	UBE3A	0.67225154	9.25E-25
GLS	MAFB	0.67276897	8.27E-25
GLS	PUM1	0.67311084	7.67E-25
GLS	YWHAG	0.67317609	7.57E-25
GLS	SMC3	0.67324645	7.45E-25
GLS	ADCY7	0.67340437	7.2E-25
GLS	ITCH	0.67352555	7.01E-25
GLS	WAC	0.67375977	6.66E-25
GLS	NEDD1	0.67402785	6.28E-25
GLS	CLIP4	0.67404912	6.25E-25
GLS	STARD3NL	0.67411453	6.16E-25
GLS	SNX29	0.6743702	5.83E-25
GLS	ZBTB41	0.6744732	5.7E-25
GLS	CBX1	0.67479115	5.31E-25
GLS	ANKRD13C	0.67488261	5.21E-25
GLS	FRS2	0.67539929	4.65E-25
GLS	MBTPS2	0.67549116	4.56E-25
GLS	ATP2B1	0.67609131	3.99E-25

GLS	EIF4E	0.67661847	3.55E-25
GLS	MFAP3	0.6766323	3.54E-25
GLS	PPP2R5E	0.67691084	3.33E-25
GLS	AKTIP	0.67706289	3.22E-25
GLS	ARF3	0.67722322	3.11E-25
GLS	WBP11	0.67731082	3.05E-25
GLS	PPIL4	0.67767591	2.81E-25
GLS	ANXA7	0.67822604	2.49E-25
GLS	SLC9A6	0.67922885	1.99E-25
GLS	CHM	0.67925416	1.98E-25
GLS	STAG2	0.67988464	1.72E-25
GLS	PIP5K1A	0.67994989	1.69E-25
GLS	ZNF227	0.6805026	1.5E-25
GLS	RASA1	0.68057296	1.47E-25
GLS	STAU2	0.68060595	1.46E-25
GLS	CSNK1G3	0.680825	1.39E-25
GLS	TMED7	0.68085385	1.38E-25
GLS	PURB	0.6810385	1.33E-25
GLS	ATP6AP2	0.68125515	1.26E-25
GLS	TTC5	0.68164895	1.16E-25
GLS	SLC39A6	0.68186359	1.1E-25
GLS	SLC25A46	0.6820371	1.06E-25
GLS	RAB21	0.68263882	9.24E-26
GLS	PKD2	0.68293594	8.64E-26
GLS	TOR1AIP1	0.68304281	8.43E-26
GLS	PTPRA	0.6830722	8.37E-26
GLS	AGGF1	0.68334996	7.86E-26
GLS	ASAP1	0.68361971	7.39E-26
GLS	GNPDA2	0.68369833	7.26E-26
GLS	AEBP2	0.68377029	7.15E-26
GLS	SENP1	0.68382697	7.05E-26
GLS	WDR47	0.68401684	6.76E-26
GLS	RNF14	0.68450863	6.04E-26
GLS	CDC27	0.68472723	5.75E-26
GLS	BMPR2	0.68488704	5.54E-26
GLS	ZYG11B	0.68497192	5.43E-26
GLS	JAZF1	0.68512702	5.24E-26
GLS	TRUB1	0.68546399	4.85E-26
GLS	PIP4K2B	0.68581538	4.48E-26
GLS	MFSD14A	0.6858644	4.43E-26
GLS	ESF1	0.68587113	4.42E-26
GLS	DPY19L4	0.68598732	4.31E-26
GLS	NDFIP1	0.68613228	4.17E-26
GLS	TMEM209	0.68643301	3.89E-26
GLS	PDS5A	0.68666677	3.68E-26
GLS	SMAD5	0.68726392	3.21E-26
GLS	DNAJB14	0.68732556	3.16E-26
GLS	ATP10D	0.68785441	2.8E-26
GLS	OSBPL8	0.68805067	2.68E-26
GLS	CHD9	0.68883527	2.23E-26
GLS	LNPEP	0.68927418	2.02E-26
GLS	RNF111	0.68947568	1.92E-26
GLS	MIGA1	0.68964025	1.85E-26
GLS	LSM14A	0.68985127	1.76E-26
GLS	RECQL	0.68993133	1.73E-26
GLS	SLC26A2	0.69072599	1.44E-26
GLS	CDK17	0.69155276	1.18E-26
GLS	NSRP1	0.69160947	1.17E-26

GLS	GMFB	0.69170137	1.14E-26
GLS	BMT2	0.69176223	1.13E-26
GLS	PHAX	0.69185492	1.1E-26
GLS	SUCLA2	0.69243836	9.61E-27
GLS	ZC3H13	0.69270392	9.03E-27
GLS	ZNF827	0.69328543	7.87E-27
GLS	CUL4B	0.69342055	7.62E-27
GLS	NF1	0.69417677	6.37E-27
GLS	ROCK1	0.69420354	6.33E-27
GLS	FCF1	0.69424324	6.27E-27
GLS	NR3C1	0.69435543	6.11E-27
GLS	STRN3	0.6945201	5.87E-27
GLS	NCOA3	0.69455864	5.82E-27
GLS	NAV1	0.6951702	5.03E-27
GLS	DCK	0.69551718	4.63E-27
GLS	SPAST	0.69553555	4.61E-27
GLS	CLIP1	0.69577822	4.35E-27
GLS	SMG8	0.69591357	4.21E-27
GLS	TLK1	0.6961966	3.94E-27
GLS	RPS6KA3	0.69620036	3.93E-27
GLS	SLC25A32	0.69647532	3.68E-27
GLS	METTL14	0.69665708	3.53E-27
GLS	JKAMP	0.69677564	3.43E-27
GLS	BICD2	0.69681797	3.39E-27
GLS	PLK2	0.69759966	2.81E-27
GLS	TRIO	0.69811928	2.48E-27
GLS	CREBL2	0.69814076	2.47E-27
GLS	USP12	0.69824868	2.41E-27
GLS	EPS15	0.69863893	2.19E-27
GLS	PRKACA	0.69924581	1.89E-27
GLS	ZNF260	0.6993803	1.83E-27
GLS	PLBD2	0.69982452	1.64E-27
GLS	FTO	0.70133865	1.14E-27
GLS	FLT1	0.7015885	1.07E-27
GLS	CBX5	0.70179528	1.02E-27
GLS	CDC42EP3	0.70189361	9.92E-28
GLS	SMC6	0.70199576	9.68E-28
GLS	PPP3CB	0.70213255	9.36E-28
GLS	ABI2	0.70323836	7.13E-28
GLS	PIGK	0.70325618	7.1E-28
GLS	MAP3K7	0.70339575	6.86E-28
GLS	ZFP90	0.70402719	5.87E-28
GLS	UHRF1BP1L	0.70470476	4.96E-28
GLS	YTHDF3	0.70532465	4.26E-28
GLS	ZMIZ1	0.70557076	4E-28
GLS	TBC1D9	0.70594976	3.64E-28
GLS	LIN52	0.70601943	3.58E-28
GLS	GTF2A1	0.70641784	3.24E-28
GLS	SAMD8	0.70683276	2.92E-28
GLS	VPS37A	0.7069454	2.84E-28
GLS	RNF6	0.7070325	2.78E-28
GLS	EPC2	0.70756044	2.44E-28
GLS	VPS41	0.70898934	1.7E-28
GLS	KAT7	0.71018237	1.26E-28
GLS	BAZ1B	0.71239089	7.16E-29
GLS	ARPP19	0.71279757	6.46E-29
GLS	ZNF322	0.71281207	6.43E-29
GLS	TMX1	0.71284948	6.37E-29

GLS	SRPK2	0.71327255	5.71E-29
GLS	FBXL17	0.71474178	3.91E-29
GLS	ZFP1	0.71479989	3.86E-29
GLS	CDC23	0.71537633	3.32E-29
GLS	PTPN11	0.71662094	2.4E-29
GLS	EFR3A	0.71735364	1.98E-29
GLS	HIPK3	0.71747476	1.92E-29
GLS	CNOT7	0.71774589	1.79E-29
GLS	BPTF	0.71992703	1.01E-29
GLS	KAT6A	0.72075131	8.11E-30
GLS	RNF11	0.72075253	8.11E-30
GLS	WBP4	0.72153663	6.58E-30
GLS	PCNP	0.72250119	5.09E-30
GLS	TRAPPC6B	0.72309964	4.33E-30
GLS	AFF4	0.72327144	4.14E-30
GLS	DAAM1	0.72424217	3.19E-30
GLS	NUP58	0.72447801	2.99E-30
GLS	ADNP	0.72505772	2.56E-30
GLS	TMED8	0.72624157	1.86E-30
GLS	FBXL3	0.72713651	1.45E-30
GLS	KIF3A	0.72727811	1.4E-30
GLS	CDS2	0.72740422	1.35E-30
GLS	MAP3K2	0.72888797	9E-31
GLS	C5orf24	0.73087268	5.2E-31
GLS	ACLY	0.73187841	3.93E-31
GLS	ACAP2	0.73284115	3.01E-31
GLS	VEZF1	0.73431073	1.99E-31
GLS	KATNAL1	0.73568982	1.35E-31
GLS	MEF2A	0.73570156	1.34E-31
GLS	BLOC1S6	0.73635937	1.11E-31
GLS	NAA30	0.73636363	1.11E-31
GLS	TTC8	0.73674871	9.97E-32
GLS	DNAJC14	0.7375514	7.93E-32
GLS	FGFR1OP2	0.7395757	4.43E-32
GLS	ZFR	0.74033503	3.56E-32
GLS	SPIN1	0.74231257	2E-32
GLS	IDS	0.74272966	1.77E-32
GLS	RRM2B	0.74287542	1.7E-32
GLS	ATF2	0.74541491	8.03E-33
GLS	AKAP11	0.74650185	5.81E-33
GLS	GEMIN5	0.74746039	4.36E-33
GLS	MCC	0.74779928	3.94E-33
GLS	ABHD13	0.74892087	2.81E-33
GLS	C5orf15	0.74995258	2.06E-33
GLS	PDS5B	0.75011599	1.96E-33
GLS	PHTF2	0.75083541	1.58E-33
GLS	SCAMP1	0.75138606	1.33E-33
GLS	SERINC1	0.75221577	1.03E-33
GLS	NEK1	0.75245095	9.62E-34
GLS	RNF41	0.75352534	6.92E-34
GLS	HSPA13	0.75595279	3.26E-34
GLS	PCNX4	0.76130198	6.02E-35
GLS	MOB1B	0.76608399	1.28E-35
GLS	EXOC5	0.76610088	1.27E-35
GLS	SOS2	0.77149357	2.12E-36
GLS	PRKAR1A	0.77679372	3.47E-37
GLS	MATR3	0.78351008	3.26E-38
GLS	PJA2	0.79310233	9.58E-40

GLS	TAOK1	0.79417047	6.4E-40
LIAS	NSRP1	0.50018842	1.17E-12
LIAS	VDAC3	0.50025283	1.16E-12
LIAS	ZNF226	0.50039842	1.14E-12
LIAS	FKBP3	0.50040724	1.14E-12
LIAS	R3HDM1	0.50043018	1.14E-12
LIAS	HARS2	0.50045988	1.13E-12
LIAS	PRPF6	0.50057786	1.12E-12
LIAS	PPP3CB	0.50067893	1.1E-12
LIAS	BBS12	0.50073738	1.1E-12
LIAS	CGRRF1	0.50081489	1.09E-12
LIAS	PIGT	0.50085623	1.08E-12
LIAS	PGAM5	0.50092185	1.07E-12
LIAS	OAZ2	0.5011486	1.04E-12
LIAS	SMARCA2	0.50127579	1.03E-12
LIAS	BRI3BP	0.501417	1.01E-12
LIAS	MRPS35	0.50143613	1.01E-12
LIAS	WASHC3	0.50149406	1E-12
LIAS	ARHGAP35	0.50154593	9.95E-13
LIAS	PRKCSH	0.50166935	9.8E-13
LIAS	PURB	0.50195417	9.47E-13
LIAS	RITA1	0.50207886	9.33E-13
LIAS	NUB1	0.5021578	9.24E-13
LIAS	SRPK2	0.50234221	9.04E-13
LIAS	ZNF250	0.50252662	8.84E-13
LIAS	SMG6	0.50270272	8.66E-13
LIAS	MAPRE1	0.50276567	8.59E-13
LIAS	CCNI	0.50301698	8.34E-13
LIAS	MED10	0.50303142	8.32E-13
LIAS	TPD52	0.50325136	8.1E-13
LIAS	C8orf33	0.50338534	7.97E-13
LIAS	DAAM1	0.50364077	7.73E-13
LIAS	L3MBTL2	0.50373697	7.64E-13
LIAS	HYLS1	0.50379136	7.59E-13
LIAS	FIP1L1	0.50381083	7.57E-13
LIAS	LANCL2	0.50382765	7.56E-13
LIAS	ATXN7L3B	0.50400431	7.4E-13
LIAS	YWHAE	0.50441024	7.05E-13
LIAS	SUZ12	0.50505485	6.52E-13
LIAS	HDAC5	0.50519282	6.41E-13
LIAS	MED6	0.50525573	6.36E-13
LIAS	MED22	0.50579405	5.96E-13
LIAS	MANBAL	0.50581455	5.94E-13
LIAS	TRMT6	0.50596478	5.83E-13
LIAS	RAD1	0.50608649	5.75E-13
LIAS	AMZ2	0.50634207	5.57E-13
LIAS	IFT22	0.50637835	5.55E-13
LIAS	KLHL12	0.50655904	5.43E-13
LIAS	SCAMP1	0.50660046	5.4E-13
LIAS	TCFL5	0.50661984	5.39E-13
LIAS	SIMC1	0.50708006	5.09E-13
LIAS	LYSMD2	0.50710522	5.08E-13
LIAS	CCAR2	0.50721508	5.01E-13
LIAS	NABP2	0.50724732	4.99E-13
LIAS	MDH1	0.50727416	4.97E-13
LIAS	COPZ1	0.50756383	4.8E-13
LIAS	MOB1B	0.50762865	4.76E-13
LIAS	MATR3	0.50788301	4.62E-13

LIAS	TCTA	0.5079203	4.6E-13
LIAS	ZKSCAN5	0.5079366	4.59E-13
LIAS	ATP6AP1	0.50816762	4.46E-13
LIAS	ZNF131	0.5083002	4.39E-13
LIAS	TM2D3	0.5083367	4.37E-13
LIAS	C12orf43	0.50846228	4.3E-13
LIAS	MAGEF1	0.50861242	4.22E-13
LIAS	GLRX5	0.50892156	4.07E-13
LIAS	FRG1	0.50906435	4E-13
LIAS	SLU7	0.50923638	3.91E-13
LIAS	DIDO1	0.50937308	3.85E-13
LIAS	PIP4K2B	0.50966797	3.71E-13
LIAS	ARMH3	0.5102374	3.46E-13
LIAS	UBQLN2	0.51033132	3.42E-13
LIAS	STARD3NL	0.51083397	3.21E-13
LIAS	ATP6V1G1	0.51124834	3.05E-13
LIAS	RNF34	0.51152965	2.95E-13
LIAS	WIPI2	0.51166957	2.9E-13
LIAS	AIFM1	0.51187453	2.82E-13
LIAS	CFAP20	0.51195476	2.8E-13
LIAS	PCIF1	0.51255377	2.6E-13
LIAS	ING4	0.51265407	2.56E-13
LIAS	LMBR1	0.51269393	2.55E-13
LIAS	ZNF322	0.51278228	2.52E-13
LIAS	GLYR1	0.51286017	2.5E-13
LIAS	RNF14	0.5128775	2.49E-13
LIAS	GIT1	0.51291493	2.48E-13
LIAS	RNF6	0.51291546	2.48E-13
LIAS	TERF2IP	0.51305972	2.44E-13
LIAS	GNAS	0.51316657	2.41E-13
LIAS	CDS2	0.51368195	2.26E-13
LIAS	WBP11	0.5140344	2.16E-13
LIAS	WDR89	0.51448324	2.04E-13
LIAS	GEMIN2	0.51480463	1.96E-13
LIAS	OTUD5	0.51493818	1.93E-13
LIAS	DZIP3	0.51496628	1.92E-13
LIAS	PMS2	0.51505789	1.9E-13
LIAS	NBR1	0.51511509	1.89E-13
LIAS	TMX4	0.51532766	1.84E-13
LIAS	PRDX3	0.51545147	1.81E-13
LIAS	VIPAS39	0.51581247	1.73E-13
LIAS	SKP1	0.51606505	1.67E-13
LIAS	PI4K2B	0.51632548	1.62E-13
LIAS	CHM	0.51641464	1.6E-13
LIAS	NUDCD2	0.51654487	1.58E-13
LIAS	RAD21	0.51660085	1.57E-13
LIAS	RANBP3	0.51787535	1.33E-13
LIAS	RDH11	0.51798498	1.31E-13
LIAS	SLC25A17	0.51823174	1.27E-13
LIAS	DDX24	0.51847036	1.24E-13
LIAS	TDP2	0.51853459	1.23E-13
LIAS	ALKBH4	0.51869315	1.2E-13
LIAS	TM9SF4	0.51876428	1.19E-13
LIAS	DLD	0.51886978	1.18E-13
LIAS	MKRN1	0.51919836	1.13E-13
LIAS	XIAP	0.51920008	1.13E-13
LIAS	MEAF6	0.51938871	1.1E-13
LIAS	C16orf70	0.51940264	1.1E-13

LIAS	FAM122B	0.51991675	1.03E-13
LIAS	ZNF821	0.52009902	1.01E-13
LIAS	CREBL2	0.52048323	9.57E-14
LIAS	MED31	0.5205459	9.49E-14
LIAS	ZER1	0.52058809	9.44E-14
LIAS	CYB5D2	0.52189292	7.99E-14
LIAS	TMEM199	0.52227872	7.61E-14
LIAS	AGGF1	0.52242468	7.47E-14
LIAS	USP12	0.52255084	7.35E-14
LIAS	MRFAP1	0.52286137	7.06E-14
LIAS	VMA21	0.52286218	7.06E-14
LIAS	SART3	0.52307778	6.87E-14
LIAS	FBXO8	0.52322711	6.73E-14
LIAS	PSMD10	0.52370105	6.34E-14
LIAS	CAMK2G	0.52410515	6.01E-14
LIAS	GMPR2	0.52415277	5.98E-14
LIAS	CALCOCO2	0.52459397	5.65E-14
LIAS	POFUT1	0.52470896	5.56E-14
LIAS	TRUB2	0.52513129	5.27E-14
LIAS	BAZ1B	0.52565778	4.92E-14
LIAS	CKAP2	0.52602243	4.69E-14
LIAS	IER3IP1	0.52616128	4.61E-14
LIAS	PANK2	0.52643697	4.45E-14
LIAS	NRDE2	0.52643827	4.45E-14
LIAS	COX15	0.5271616	4.05E-14
LIAS	NKRF	0.52720424	4.02E-14
LIAS	ERP29	0.52723494	4.01E-14
LIAS	FBXL5	0.52736177	3.94E-14
LIAS	DYNLL2	0.52746828	3.89E-14
LIAS	NFE2L1	0.52773019	3.76E-14
LIAS	GNPDA2	0.52780208	3.72E-14
LIAS	STK16	0.52819504	3.54E-14
LIAS	NAA30	0.52830404	3.49E-14
LIAS	KIF3A	0.52834171	3.47E-14
LIAS	ZNF398	0.52852708	3.39E-14
LIAS	QDPR	0.52906706	3.15E-14
LIAS	ATP6AP2	0.52907004	3.15E-14
LIAS	ATPSCKMT	0.5291836	3.11E-14
LIAS	TAF7	0.52959318	2.94E-14
LIAS	BPTF	0.52961367	2.94E-14
LIAS	ZMYND8	0.53004785	2.77E-14
LIAS	YTHDF1	0.53014459	2.74E-14
LIAS	TYW1	0.5303572	2.66E-14
LIAS	MRPS31	0.53112641	2.41E-14
LIAS	GEMIN5	0.53134126	2.34E-14
LIAS	PPP5C	0.532044	2.13E-14
LIAS	DCP1B	0.53221744	2.08E-14
LIAS	ZNF512	0.5325723	1.99E-14
LIAS	SMIM10L1	0.53257551	1.99E-14
LIAS	NFU1	0.53290819	1.9E-14
LIAS	MICU2	0.53303919	1.87E-14
LIAS	STARD7	0.5333861	1.78E-14
LIAS	NAA20	0.53349951	1.76E-14
LIAS	BECN1	0.53419655	1.6E-14
LIAS	RPL7L1	0.53436744	1.56E-14
LIAS	AP5S1	0.53461799	1.51E-14
LIAS	ISCA1	0.53462814	1.51E-14
LIAS	APMAP	0.53500144	1.44E-14

LIAS	KIAA0232	0.53514842	1.41E-14
LIAS	TMEM251	0.53530752	1.38E-14
LIAS	MED28	0.53552213	1.34E-14
LIAS	C9orf78	0.53629308	1.21E-14
LIAS	ZNF263	0.53649142	1.18E-14
LIAS	CBX5	0.53697738	1.1E-14
LIAS	PI4KA	0.53778089	9.9E-15
LIAS	IK	0.53797009	9.65E-15
LIAS	NDFIP1	0.53804463	9.55E-15
LIAS	C1QBP	0.53831855	9.2E-15
LIAS	TMEM68	0.53832488	9.2E-15
LIAS	CRCP	0.53848921	8.99E-15
LIAS	CRKL	0.53868707	8.76E-15
LIAS	DIABLO	0.53913625	8.24E-15
LIAS	CFAP36	0.53929854	8.06E-15
LIAS	PAN2	0.53940105	7.95E-15
LIAS	SLC30A9	0.53940257	7.95E-15
LIAS	GLE1	0.53941943	7.93E-15
LIAS	ZCRB1	0.54038663	6.95E-15
LIAS	DCK	0.54038674	6.95E-15
LIAS	RPA1	0.54046589	6.88E-15
LIAS	TAF9B	0.54111776	6.29E-15
LIAS	JKAMP	0.54142354	6.04E-15
LIAS	SPOP	0.54162206	5.87E-15
LIAS	PTPRA	0.54210629	5.5E-15
LIAS	TMED4	0.54233805	5.33E-15
LIAS	RBMX2	0.54250595	5.2E-15
LIAS	PRPS1	0.54256247	5.16E-15
LIAS	MLH1	0.54270427	5.06E-15
LIAS	MRM2	0.54333371	4.65E-15
LIAS	ATP9A	0.54351257	4.53E-15
LIAS	OXCT1	0.54362844	4.46E-15
LIAS	C2orf15	0.5436597	4.44E-15
LIAS	PMPCA	0.54368893	4.42E-15
LIAS	NUP58	0.54398318	4.25E-15
LIAS	PDS5A	0.54403505	4.22E-15
LIAS	TRAPPC9	0.54408313	4.19E-15
LIAS	AIMP1	0.54416364	4.14E-15
LIAS	SAYSD1	0.54433375	4.05E-15
LIAS	HINT1	0.54443345	3.99E-15
LIAS	TMEM209	0.54460445	3.9E-15
LIAS	RUNDC1	0.54549217	3.45E-15
LIAS	ALKBH5	0.54557043	3.41E-15
LIAS	RBM22	0.54557598	3.41E-15
LIAS	GPANK1	0.54576754	3.32E-15
LIAS	DNM1L	0.54580031	3.31E-15
LIAS	MRPL50	0.54590025	3.26E-15
LIAS	RETREG2	0.54607418	3.18E-15
LIAS	CDKN1B	0.5465009	3E-15
LIAS	FAM200A	0.54704013	2.78E-15
LIAS	PXMP4	0.54712209	2.75E-15
LIAS	ARHGEF9	0.54726875	2.7E-15
LIAS	KIAA0586	0.54736159	2.66E-15
LIAS	AK6	0.54772055	2.53E-15
LIAS	GABARAPL2	0.54835415	2.32E-15
LIAS	ZFR	0.54870786	2.2E-15
LIAS	SUPT16H	0.5488951	2.15E-15
LIAS	SNW1	0.54909347	2.09E-15

LIAS	YAE1	0.5499868	1.84E-15
LIAS	PRUNE1	0.55010504	1.81E-15
LIAS	RPRD1B	0.55033718	1.75E-15
LIAS	URGCP	0.55052589	1.71E-15
LIAS	UNC50	0.55062	1.69E-15
LIAS	UPRT	0.55084563	1.63E-15
LIAS	PAIP2	0.55103308	1.59E-15
LIAS	LIN54	0.55108816	1.58E-15
LIAS	AACS	0.55135573	1.52E-15
LIAS	SUCLA2	0.55197754	1.39E-15
LIAS	GNPDA1	0.55203631	1.38E-15
LIAS	CDC23	0.55252375	1.29E-15
LIAS	CMAS	0.55255961	1.28E-15
LIAS	IVD	0.55312238	1.18E-15
LIAS	GATB	0.5531961	1.17E-15
LIAS	DCAKD	0.55354653	1.11E-15
LIAS	PGRMC1	0.55423977	1.01E-15
LIAS	FAM210B	0.554376	9.9E-16
LIAS	TMEM230	0.55458882	9.6E-16
LIAS	PIGU	0.55528877	8.69E-16
LIAS	RNF20	0.55567247	8.22E-16
LIAS	VBP1	0.55573793	8.15E-16
LIAS	FBXL17	0.55607019	7.77E-16
LIAS	DUS4L	0.55642021	7.39E-16
LIAS	SOGA1	0.55666133	7.14E-16
LIAS	NGDN	0.55676465	7.03E-16
LIAS	PNPO	0.55705335	6.75E-16
LIAS	RAB28	0.55719836	6.61E-16
LIAS	CUL1	0.55783354	6.03E-16
LIAS	WWOX	0.55791027	5.96E-16
LIAS	PSPC1	0.55818581	5.73E-16
LIAS	ZSWIM1	0.55821286	5.71E-16
LIAS	FXR2	0.55948449	4.75E-16
LIAS	PRKAG2	0.56003095	4.39E-16
LIAS	EXOSC9	0.56009704	4.34E-16
LIAS	TEFM	0.56098092	3.82E-16
LIAS	ZC3HC1	0.5610229	3.8E-16
LIAS	ENOPH1	0.56102693	3.79E-16
LIAS	EAPP	0.56107358	3.77E-16
LIAS	CNOT4	0.56130405	3.64E-16
LIAS	ABHD16A	0.56202991	3.28E-16
LIAS	GPBP1	0.56228624	3.16E-16
LIAS	GID8	0.56241801	3.1E-16
LIAS	ABHD15	0.56282878	2.91E-16
LIAS	SLC25A11	0.56283449	2.91E-16
LIAS	DYNC2LI1	0.5628572	2.9E-16
LIAS	ZNF227	0.56287008	2.9E-16
LIAS	DENR	0.56288247	2.89E-16
LIAS	MDP1	0.56294447	2.87E-16
LIAS	POT1	0.56323797	2.74E-16
LIAS	RCC1L	0.56335695	2.7E-16
LIAS	HSPA9	0.5644312	2.3E-16
LIAS	CXorf56	0.56468044	2.22E-16
LIAS	NIPBL	0.56485751	2.16E-16
LIAS	GLOD4	0.56514189	2.07E-16
LIAS	CEP126	0.56519436	2.06E-16
LIAS	ADNP	0.56522222	2.05E-16
LIAS	TMED8	0.56548739	1.97E-16

LIAS	LCORL	0.56555143	1.95E-16
LIAS	KIF3B	0.56569334	1.91E-16
LIAS	NCOA6	0.565699	1.91E-16
LIAS	ZNF564	0.56583429	1.87E-16
LIAS	SCO1	0.56587227	1.86E-16
LIAS	RTF2	0.56783625	1.39E-16
LIAS	NARS2	0.5680367	1.35E-16
LIAS	BBS2	0.56861305	1.24E-16
LIAS	COQ7	0.56972803	1.05E-16
LIAS	GFOD2	0.56981031	1.03E-16
LIAS	LRBA	0.5702505	9.68E-17
LIAS	TMEM60	0.57107942	8.54E-17
LIAS	PITHD1	0.57109425	8.52E-17
LIAS	HIRIP3	0.57223924	7.17E-17
LIAS	CEP250	0.57230454	7.1E-17
LIAS	MKKS	0.57399979	5.49E-17
LIAS	NKAP	0.57401543	5.47E-17
LIAS	TSR2	0.57457338	5.03E-17
LIAS	CETN3	0.576102	3.98E-17
LIAS	FAM200B	0.57611292	3.97E-17
LIAS	SYNJ2BP	0.57728153	3.32E-17
LIAS	POLR2C	0.577897	3.02E-17
LIAS	TTC5	0.57860376	2.71E-17
LIAS	WBP4	0.57881509	2.62E-17
LIAS	BRAP	0.57967285	2.29E-17
LIAS	MRPS27	0.58025062	2.1E-17
LIAS	KAT7	0.580347	2.06E-17
LIAS	PCYOX1L	0.58099725	1.87E-17
LIAS	SLC39A9	0.58313403	1.33E-17
LIAS	SDHAF4	0.58499921	9.95E-18
LIAS	GPALPP1	0.58580873	8.75E-18
LIAS	ZNF174	0.58742388	6.77E-18
LIAS	FAM104B	0.58758655	6.59E-18
LIAS	GON7	0.58895354	5.3E-18
LIAS	ZCCHC3	0.59084893	3.91E-18
LIAS	MOAP1	0.59172416	3.39E-18
LIAS	MTG2	0.59435102	2.21E-18
LIAS	NELFCD	0.59479434	2.06E-18
LIAS	TTC8	0.59506916	1.97E-18
LIAS	WDR20	0.59871117	1.08E-18
LIAS	ZNHIT3	0.59879837	1.06E-18
LIAS	PRKAG1	0.5993237	9.75E-19
LIAS	TMEM128	0.60414647	4.34E-19
LIAS	ACTR6	0.60483554	3.86E-19
LIAS	COQ5	0.60664323	2.84E-19
LIAS	GRSF1	0.60674881	2.79E-19
LIAS	VPS33A	0.60687437	2.73E-19
LIAS	PDRG1	0.60922207	1.83E-19
LIAS	LSM14B	0.61045886	1.48E-19
LIAS	OCIAD1	0.6114421	1.25E-19
LIAS	DHX35	0.61220113	1.09E-19
LIAS	VAPB	0.61238807	1.06E-19
LIAS	COQ6	0.6124457	1.05E-19
LIAS	LIN52	0.61599163	5.62E-20
LIAS	CIPC	0.61626769	5.35E-20
LIAS	COPS4	0.61911079	3.23E-20
LIAS	LARP7	0.62288689	1.64E-20
LIAS	TFCP2	0.62480068	1.16E-20

LIAS	CENPS	0.62670268	8.2E-21
LIAS	HTATSF1	0.6267137	8.18E-21
LIAS	MRFAP1L1	0.62796539	6.5E-21
LIAS	C12orf65	0.63672741	1.26E-21
LIAS	PACRGL	0.63899674	8.19E-22
LIAS	NDRG3	0.64319672	3.64E-22
LIAS	NDUFA5	0.64672702	1.82E-22
LIAS	TBC1D14	0.65101737	7.77E-23
LIAS	RAE1	0.65837628	1.74E-23
LIAS	ALKBH1	0.68423522	6.43E-26
LIAS	GUF1	0.68629308	4.01E-26
LIPT1	CCAR2	0.50003893	1.19E-12
LIPT1	FCHO2	0.50014722	1.18E-12
LIPT1	CLINT1	0.50030082	1.15E-12
LIPT1	CAMKK2	0.50040921	1.14E-12
LIPT1	UBE2E1	0.50046975	1.13E-12
LIPT1	RMDN1	0.50051296	1.13E-12
LIPT1	GOLGA7	0.50065399	1.11E-12
LIPT1	PHTF1	0.50065826	1.11E-12
LIPT1	SLC15A4	0.50067031	1.1E-12
LIPT1	COPS2	0.50069346	1.1E-12
LIPT1	SAR1A	0.50069384	1.1E-12
LIPT1	GEMIN2	0.50073633	1.1E-12
LIPT1	VPS41	0.5008532	1.08E-12
LIPT1	ETS2	0.50093518	1.07E-12
LIPT1	HDAC8	0.50097218	1.07E-12
LIPT1	GFM1	0.50105426	1.06E-12
LIPT1	RAB12	0.50108812	1.05E-12
LIPT1	IPO9	0.50109745	1.05E-12
LIPT1	HLA-E	0.50115024	1.04E-12
LIPT1	PPP1R9B	0.50116092	1.04E-12
LIPT1	SNX11	0.50119648	1.04E-12
LIPT1	SWAP70	0.50120095	1.04E-12
LIPT1	JAK1	0.5012532	1.03E-12
LIPT1	MAPKAPK2	0.50128393	1.03E-12
LIPT1	DESI2	0.50137095	1.02E-12
LIPT1	SNRNP40	0.50143412	1.01E-12
LIPT1	FNBP1L	0.50144358	1.01E-12
LIPT1	ABT1	0.50155584	9.94E-13
LIPT1	CUL2	0.50157001	9.92E-13
LIPT1	PCNX2	0.50165723	9.82E-13
LIPT1	RAD51B	0.50166559	9.81E-13
LIPT1	CASP2	0.50179437	9.65E-13
LIPT1	NAPG	0.50181888	9.63E-13
LIPT1	PPP2CB	0.50193255	9.5E-13
LIPT1	STAU2	0.5019623	9.46E-13
LIPT1	CSDE1	0.50198658	9.43E-13
LIPT1	SBF2	0.50205433	9.36E-13
LIPT1	AKT1	0.50205967	9.35E-13
LIPT1	RP2	0.50206239	9.35E-13
LIPT1	FEM1C	0.50209061	9.32E-13
LIPT1	TMEM185B	0.50212024	9.28E-13
LIPT1	SNAP47	0.50227131	9.12E-13
LIPT1	RNF13	0.50231009	9.08E-13
LIPT1	ZNF438	0.50255877	8.81E-13
LIPT1	YWHAB	0.50259504	8.77E-13
LIPT1	ANXA7	0.50263608	8.73E-13
LIPT1	HHAT	0.50265796	8.7E-13

LIPT1	HIPK3	0.50282078	8.53E-13
LIPT1	NUDCD3	0.50289382	8.46E-13
LIPT1	ZBTB41	0.50291553	8.44E-13
LIPT1	TSEN15	0.50292263	8.43E-13
LIPT1	PSMD7	0.50307997	8.27E-13
LIPT1	ZNF226	0.50322309	8.13E-13
LIPT1	FYCO1	0.50330267	8.05E-13
LIPT1	MAP3K13	0.50335021	8.01E-13
LIPT1	CUL1	0.50351772	7.85E-13
LIPT1	BCCIP	0.50354722	7.82E-13
LIPT1	ZNF562	0.50364938	7.72E-13
LIPT1	GLTP	0.50374601	7.63E-13
LIPT1	HIVEP1	0.50378076	7.6E-13
LIPT1	ARL2BP	0.50382655	7.56E-13
LIPT1	EIF4G2	0.5038847	7.51E-13
LIPT1	SLC30A6	0.50390577	7.49E-13
LIPT1	EXOC6B	0.5039211	7.47E-13
LIPT1	NCKAP1	0.50393639	7.46E-13
LIPT1	ZFP91	0.50417281	7.25E-13
LIPT1	SUFU	0.50417946	7.24E-13
LIPT1	CCNK	0.5043388	7.11E-13
LIPT1	FBXL12	0.50437023	7.08E-13
LIPT1	DEK	0.50437322	7.08E-13
LIPT1	CPOX	0.50438306	7.07E-13
LIPT1	DCUN1D3	0.50440402	7.05E-13
LIPT1	TRAPPC2B	0.50443775	7.02E-13
LIPT1	CTDSPL2	0.50467382	6.82E-13
LIPT1	FBXO7	0.50480961	6.71E-13
LIPT1	FBXO34	0.50496597	6.59E-13
LIPT1	CALM2	0.50499368	6.56E-13
LIPT1	PACRGL	0.50501466	6.55E-13
LIPT1	NSMCE3	0.50503208	6.53E-13
LIPT1	STAT5A	0.50505155	6.52E-13
LIPT1	NUP58	0.5050689	6.51E-13
LIPT1	DHX30	0.5051479	6.44E-13
LIPT1	SMC6	0.50528215	6.34E-13
LIPT1	CD2BP2	0.50535665	6.28E-13
LIPT1	ZNF720	0.50549217	6.18E-13
LIPT1	TCAIM	0.5055539	6.13E-13
LIPT1	TEX10	0.5055653	6.12E-13
LIPT1	SLFN12	0.50560282	6.1E-13
LIPT1	RILPL2	0.50560753	6.09E-13
LIPT1	NRBP1	0.50575478	5.99E-13
LIPT1	MED14	0.50586834	5.9E-13
LIPT1	TOPORS	0.50598852	5.82E-13
LIPT1	PATL1	0.50600897	5.8E-13
LIPT1	CALCOCO2	0.50604354	5.78E-13
LIPT1	RAB6A	0.50628126	5.61E-13
LIPT1	SLC25A13	0.50632002	5.59E-13
LIPT1	HMGXB3	0.5064119	5.53E-13
LIPT1	IMMT	0.50648195	5.48E-13
LIPT1	NUP50	0.50671718	5.32E-13
LIPT1	PRR14L	0.50707372	5.1E-13
LIPT1	CETN3	0.50708515	5.09E-13
LIPT1	GNPTAB	0.50719438	5.02E-13
LIPT1	PLEKHA5	0.50730498	4.96E-13
LIPT1	SMARCA5	0.50733624	4.94E-13
LIPT1	POLR2K	0.50733631	4.94E-13

LIPT1	SELENOF	0.50744638	4.87E-13
LIPT1	BAZ1A	0.50753864	4.82E-13
LIPT1	M6PR	0.50754193	4.81E-13
LIPT1	PIK3IP1	0.50764131	4.76E-13
LIPT1	CPNE8	0.50772654	4.71E-13
LIPT1	RNF6	0.50780468	4.66E-13
LIPT1	ZNF250	0.50780713	4.66E-13
LIPT1	PCSK7	0.50783843	4.64E-13
LIPT1	CAB39	0.50786468	4.63E-13
LIPT1	PAK1	0.50791992	4.6E-13
LIPT1	SCAMP2	0.50801997	4.54E-13
LIPT1	ZNF146	0.5082281	4.43E-13
LIPT1	DYNC1LI1	0.50830331	4.39E-13
LIPT1	BTN3A1	0.50838118	4.34E-13
LIPT1	AKAP11	0.50856352	4.25E-13
LIPT1	SLC25A12	0.5086674	4.19E-13
LIPT1	SRP14	0.50885998	4.1E-13
LIPT1	HNRNPA0	0.5088674	4.09E-13
LIPT1	BLOC1S6	0.50891524	4.07E-13
LIPT1	AAR2	0.50898284	4.04E-13
LIPT1	UBN1	0.50900316	4.03E-13
LIPT1	SLC25A44	0.50900419	4.02E-13
LIPT1	RPP30	0.50934515	3.86E-13
LIPT1	CWC27	0.50934927	3.86E-13
LIPT1	NIPA2	0.50935061	3.86E-13
LIPT1	VPS72	0.50936526	3.85E-13
LIPT1	ABR	0.50940289	3.83E-13
LIPT1	RPIA	0.50944646	3.81E-13
LIPT1	UBE2Q1	0.50955843	3.76E-13
LIPT1	NAB1	0.50960706	3.74E-13
LIPT1	RAB29	0.50974461	3.67E-13
LIPT1	SMNDC1	0.50983206	3.64E-13
LIPT1	ZCCHC3	0.50984284	3.63E-13
LIPT1	MRPS36	0.50995951	3.58E-13
LIPT1	TRAPPC6B	0.51026537	3.45E-13
LIPT1	KLHL29	0.51026605	3.45E-13
LIPT1	FEZ2	0.51029759	3.43E-13
LIPT1	LSM14A	0.51041712	3.38E-13
LIPT1	ETF1	0.51057618	3.32E-13
LIPT1	ACTR2	0.51057642	3.32E-13
LIPT1	SH3D19	0.51070396	3.26E-13
LIPT1	PIK3R1	0.51074602	3.25E-13
LIPT1	RLIM	0.51081599	3.22E-13
LIPT1	GLO1	0.51087203	3.2E-13
LIPT1	TAF12	0.51097461	3.16E-13
LIPT1	TRIM69	0.51098516	3.15E-13
LIPT1	KIF5B	0.51112715	3.1E-13
LIPT1	HMCES	0.51124024	3.06E-13
LIPT1	INTS9	0.51127151	3.04E-13
LIPT1	PPM1A	0.51132201	3.02E-13
LIPT1	AHR	0.51149653	2.96E-13
LIPT1	WDFY1	0.5116016	2.92E-13
LIPT1	COPB1	0.51163321	2.91E-13
LIPT1	PCYT1A	0.51181326	2.85E-13
LIPT1	VCPIP1	0.51182648	2.84E-13
LIPT1	MED1	0.51187248	2.83E-13
LIPT1	HNRNPM	0.5119123	2.81E-13
LIPT1	TRAF3IP1	0.51195769	2.8E-13

LIPT1	LIMK2	0.51203143	2.77E-13
LIPT1	ZSWIM1	0.51205846	2.76E-13
LIPT1	SKP1	0.51224968	2.7E-13
LIPT1	PLCG1	0.51225081	2.7E-13
LIPT1	RAB1A	0.51228868	2.68E-13
LIPT1	PARP3	0.51258126	2.59E-13
LIPT1	VAMP3	0.51261229	2.58E-13
LIPT1	ZDHHC13	0.51278976	2.52E-13
LIPT1	LAPTM4A	0.51283614	2.51E-13
LIPT1	CNOT4	0.51293073	2.48E-13
LIPT1	HNRNPA1	0.51293741	2.48E-13
LIPT1	PDHB	0.51300548	2.45E-13
LIPT1	PRPF40A	0.5131316	2.42E-13
LIPT1	PPP2R5D	0.5131522	2.41E-13
LIPT1	EID1	0.51323238	2.39E-13
LIPT1	EIF4G3	0.51342288	2.33E-13
LIPT1	L3MBTL2	0.51342555	2.33E-13
LIPT1	TFE3	0.5134283	2.33E-13
LIPT1	PICALM	0.51346249	2.32E-13
LIPT1	UBE2D1	0.51355063	2.29E-13
LIPT1	VPS29	0.51357835	2.29E-13
LIPT1	WWP1	0.51362008	2.27E-13
LIPT1	KCTD21	0.51371016	2.25E-13
LIPT1	TBCC	0.51376993	2.23E-13
LIPT1	ATL3	0.51381298	2.22E-13
LIPT1	AMPD3	0.51399125	2.17E-13
LIPT1	GLOD4	0.51400969	2.17E-13
LIPT1	SUSD1	0.51401801	2.16E-13
LIPT1	CIPC	0.5140246	2.16E-13
LIPT1	ADH5	0.51413199	2.13E-13
LIPT1	BMPR2	0.5141813	2.12E-13
LIPT1	RAMAC	0.51425442	2.1E-13
LIPT1	ATG16L1	0.51426016	2.1E-13
LIPT1	CAMSAP2	0.51438985	2.07E-13
LIPT1	AKTIP	0.51443853	2.05E-13
LIPT1	CTTNBP2NL	0.51451296	2.03E-13
LIPT1	MLH3	0.51483221	1.95E-13
LIPT1	TMEM230	0.51495988	1.92E-13
LIPT1	NDUFB5	0.51499766	1.91E-13
LIPT1	NASP	0.51509931	1.89E-13
LIPT1	ARL6IP6	0.51514741	1.88E-13
LIPT1	NAP1L4	0.515187	1.87E-13
LIPT1	RCN2	0.5152697	1.85E-13
LIPT1	IMPA1	0.51547424	1.8E-13
LIPT1	SIPA1L2	0.51552136	1.79E-13
LIPT1	RNF149	0.5155962	1.78E-13
LIPT1	CACUL1	0.51559916	1.78E-13
LIPT1	FAM20B	0.51580576	1.73E-13
LIPT1	DDX27	0.51581701	1.73E-13
LIPT1	WDR61	0.51583755	1.72E-13
LIPT1	ROCK2	0.51586058	1.72E-13
LIPT1	SLU7	0.51601646	1.68E-13
LIPT1	NUB1	0.51621183	1.64E-13
LIPT1	USP12	0.51629943	1.63E-13
LIPT1	DHDDS	0.51635552	1.61E-13
LIPT1	USP6NL	0.51668648	1.55E-13
LIPT1	CNOT6	0.51673093	1.54E-13
LIPT1	USP9X	0.51674072	1.54E-13

LIPT1	EMB	0.51676638	1.53E-13
LIPT1	ACTR6	0.51678371	1.53E-13
LIPT1	ERVK3-1	0.51681204	1.52E-13
LIPT1	PPP2R5C	0.51682986	1.52E-13
LIPT1	PTPRA	0.51686649	1.51E-13
LIPT1	ZFP64	0.5169234	1.5E-13
LIPT1	PMS2	0.51692637	1.5E-13
LIPT1	ZRSR2	0.51701934	1.48E-13
LIPT1	ZNF564	0.51702746	1.48E-13
LIPT1	ZNF639	0.51707911	1.47E-13
LIPT1	NSD3	0.5172864	1.44E-13
LIPT1	DIS3	0.51731363	1.43E-13
LIPT1	C14orf119	0.51736121	1.42E-13
LIPT1	TRIP12	0.51754046	1.39E-13
LIPT1	DHX35	0.51772902	1.36E-13
LIPT1	STEAP2	0.51782227	1.34E-13
LIPT1	YTHDF3	0.51799239	1.31E-13
LIPT1	ABCB10	0.51803262	1.31E-13
LIPT1	SPIN1	0.51805223	1.3E-13
LIPT1	NFATC3	0.51817714	1.28E-13
LIPT1	LRRC57	0.51822605	1.27E-13
LIPT1	GTF2E1	0.51838933	1.25E-13
LIPT1	CFAP97	0.51849002	1.23E-13
LIPT1	BTBD10	0.51855405	1.22E-13
LIPT1	PITPNB	0.51862753	1.21E-13
LIPT1	METAP1	0.518886	1.17E-13
LIPT1	PPIL4	0.51895228	1.16E-13
LIPT1	UBE2D3	0.51905458	1.15E-13
LIPT1	TCEAL8	0.51929359	1.11E-13
LIPT1	TSG101	0.51933185	1.11E-13
LIPT1	TCF20	0.51935294	1.11E-13
LIPT1	SART3	0.51941979	1.1E-13
LIPT1	NMI	0.51948417	1.09E-13
LIPT1	SKAP2	0.51961712	1.07E-13
LIPT1	PAFAH1B2	0.5197277	1.05E-13
LIPT1	UTP14C	0.51983265	1.04E-13
LIPT1	ARFGEF1	0.51984346	1.04E-13
LIPT1	LAMTOR3	0.51991817	1.03E-13
LIPT1	DOCK1	0.51996977	1.02E-13
LIPT1	ZNFX1	0.5200394	1.01E-13
LIPT1	ATMIN	0.52014452	9.99E-14
LIPT1	RANBP9	0.52049948	9.55E-14
LIPT1	SETD3	0.52061431	9.41E-14
LIPT1	FASTKD2	0.52064581	9.37E-14
LIPT1	ARFIP1	0.52080096	9.19E-14
LIPT1	CUL4B	0.52084847	9.13E-14
LIPT1	STAG2	0.52091456	9.06E-14
LIPT1	UBE2Q2	0.52107377	8.88E-14
LIPT1	CAPZA1	0.5211364	8.81E-14
LIPT1	CBX3	0.52114087	8.8E-14
LIPT1	TOR3A	0.52124712	8.68E-14
LIPT1	CASP10	0.5212916	8.63E-14
LIPT1	ZNF638	0.52141487	8.5E-14
LIPT1	TEX9	0.52142753	8.48E-14
LIPT1	ARPC5	0.5214318	8.48E-14
LIPT1	OTUD1	0.52149847	8.41E-14
LIPT1	APAF1	0.52151323	8.39E-14
LIPT1	RPAP3	0.52161541	8.28E-14

LIPT1	RNF25	0.52176771	8.12E-14
LIPT1	COG7	0.52188988	8E-14
LIPT1	VPS26A	0.52200294	7.88E-14
LIPT1	STAMBP	0.52200891	7.88E-14
LIPT1	FRG1	0.52210998	7.77E-14
LIPT1	UBE2D2	0.52214107	7.74E-14
LIPT1	FAM118B	0.52216147	7.72E-14
LIPT1	LEO1	0.52225975	7.63E-14
LIPT1	TP53BP2	0.52233385	7.55E-14
LIPT1	LBR	0.52239982	7.49E-14
LIPT1	OSBPL11	0.52240693	7.48E-14
LIPT1	IFNAR2	0.52249615	7.4E-14
LIPT1	CUL3	0.52255243	7.34E-14
LIPT1	LSM6	0.52257308	7.33E-14
LIPT1	RBM27	0.52273415	7.18E-14
LIPT1	IL10RB	0.52285979	7.06E-14
LIPT1	SLC35A1	0.52308241	6.86E-14
LIPT1	IVD	0.52308837	6.86E-14
LIPT1	SUSD6	0.52325695	6.71E-14
LIPT1	MFSD14A	0.52325827	6.71E-14
LIPT1	PPM1M	0.52328221	6.69E-14
LIPT1	ZCCHC17	0.52332209	6.65E-14
LIPT1	WBP4	0.52339557	6.59E-14
LIPT1	TMEM127	0.52343112	6.56E-14
LIPT1	MTFMT	0.52353743	6.47E-14
LIPT1	NCOA4	0.52357719	6.44E-14
LIPT1	TDRD7	0.52363072	6.39E-14
LIPT1	TM2D3	0.52371534	6.32E-14
LIPT1	CASP8	0.52374348	6.3E-14
LIPT1	EIF1AD	0.52383964	6.22E-14
LIPT1	ZNF134	0.52406438	6.05E-14
LIPT1	RAB9A	0.52413899	5.99E-14
LIPT1	ATL2	0.52427522	5.88E-14
LIPT1	ZC3H18	0.52427891	5.88E-14
LIPT1	SNX5	0.52434537	5.83E-14
LIPT1	COPS5	0.52446589	5.74E-14
LIPT1	CNOT10	0.52465584	5.6E-14
LIPT1	CCDC59	0.52488545	5.44E-14
LIPT1	NUMB	0.52491833	5.41E-14
LIPT1	TRIM14	0.52496564	5.38E-14
LIPT1	OTUD7B	0.52518395	5.23E-14
LIPT1	ATXN3	0.52527936	5.17E-14
LIPT1	FAM120AOS	0.52530655	5.15E-14
LIPT1	ZMYM4	0.52537478	5.1E-14
LIPT1	STK38	0.52540878	5.08E-14
LIPT1	ACVR1	0.52565244	4.92E-14
LIPT1	GNPDA2	0.52572613	4.88E-14
LIPT1	CEBPZ	0.52574807	4.86E-14
LIPT1	AHCTF1	0.52577367	4.85E-14
LIPT1	FAM98B	0.52579785	4.83E-14
LIPT1	EDC3	0.52587501	4.78E-14
LIPT1	PER2	0.52634703	4.5E-14
LIPT1	RAB8B	0.52636927	4.49E-14
LIPT1	ESCO1	0.52637635	4.48E-14
LIPT1	ADD3	0.52640666	4.46E-14
LIPT1	CSNK1A1	0.52642152	4.46E-14
LIPT1	FKBP15	0.52671483	4.29E-14
LIPT1	PIK3C2B	0.52682718	4.23E-14

LIPT1	GTF2A1	0.52686109	4.21E-14
LIPT1	MIS18BP1	0.52715822	4.05E-14
LIPT1	KIN	0.52752929	3.86E-14
LIPT1	C3orf38	0.52755844	3.84E-14
LIPT1	DCTN6	0.52762165	3.81E-14
LIPT1	UBXN2B	0.52772939	3.76E-14
LIPT1	ZDHHC5	0.52779107	3.73E-14
LIPT1	GIMAP2	0.52786064	3.69E-14
LIPT1	PDE6D	0.5279499	3.65E-14
LIPT1	HMGN4	0.52806266	3.6E-14
LIPT1	COQ10B	0.52807727	3.59E-14
LIPT1	DAZAP2	0.52815344	3.56E-14
LIPT1	FARP2	0.52825456	3.51E-14
LIPT1	SMARCE1	0.52826384	3.5E-14
LIPT1	METTL21A	0.52828904	3.49E-14
LIPT1	ATG3	0.52833835	3.47E-14
LIPT1	IVNS1ABP	0.52853138	3.38E-14
LIPT1	BTBD1	0.52876644	3.28E-14
LIPT1	CSNK1G3	0.52919465	3.1E-14
LIPT1	SNAPC5	0.52925879	3.08E-14
LIPT1	OTUD5	0.52944165	3E-14
LIPT1	PSMC6	0.52944981	3E-14
LIPT1	DNM1L	0.52944996	3E-14
LIPT1	TRIM38	0.52947378	2.99E-14
LIPT1	AEBP2	0.52955456	2.96E-14
LIPT1	UGP2	0.52980709	2.86E-14
LIPT1	OSTF1	0.53002157	2.78E-14
LIPT1	ZFR	0.53002834	2.78E-14
LIPT1	HSDL1	0.53016621	2.73E-14
LIPT1	UBE3A	0.53034566	2.67E-14
LIPT1	TINF2	0.53036227	2.66E-14
LIPT1	PIGBOS1	0.53050484	2.61E-14
LIPT1	CDC5L	0.53053765	2.6E-14
LIPT1	PCNP	0.53064832	2.56E-14
LIPT1	U2AF2	0.53083759	2.5E-14
LIPT1	RDH14	0.53084649	2.5E-14
LIPT1	RAB5C	0.53090307	2.48E-14
LIPT1	TEFM	0.53090728	2.48E-14
LIPT1	PPP1CC	0.53102883	2.44E-14
LIPT1	RAB35	0.53103168	2.44E-14
LIPT1	NBR1	0.53104655	2.43E-14
LIPT1	MRPL49	0.53110004	2.41E-14
LIPT1	ADNP	0.53129961	2.35E-14
LIPT1	PNO1	0.53140006	2.32E-14
LIPT1	ARMH3	0.53147047	2.3E-14
LIPT1	DDX23	0.53173179	2.22E-14
LIPT1	HMGXB4	0.53180729	2.2E-14
LIPT1	METTL9	0.53195344	2.16E-14
LIPT1	BMI1	0.53213398	2.11E-14
LIPT1	PCGF5	0.53241624	2.03E-14
LIPT1	SPOP	0.53245405	2.02E-14
LIPT1	IFNAR1	0.5325307	2E-14
LIPT1	BCAS2	0.53257791	1.99E-14
LIPT1	DLG1	0.53273078	1.95E-14
LIPT1	DDX3X	0.5327594	1.94E-14
LIPT1	ZNRF2	0.53282227	1.92E-14
LIPT1	UBE2A	0.53293107	1.89E-14
LIPT1	SCP2	0.53296344	1.89E-14

LIPT1	NRBF2	0.53300311	1.88E-14
LIPT1	ETV3	0.53316735	1.84E-14
LIPT1	SDHAF2	0.53321715	1.82E-14
LIPT1	CHD1L	0.53335537	1.79E-14
LIPT1	DCAF1	0.53344524	1.77E-14
LIPT1	OXR1	0.53345932	1.77E-14
LIPT1	HAT1	0.5335785	1.74E-14
LIPT1	RELA	0.53358204	1.74E-14
LIPT1	CCDC71	0.5336753	1.72E-14
LIPT1	CDK19	0.53382716	1.68E-14
LIPT1	SPG21	0.53400152	1.64E-14
LIPT1	ZBTB2	0.53412712	1.62E-14
LIPT1	RNF20	0.53416515	1.61E-14
LIPT1	CPT1A	0.53436304	1.57E-14
LIPT1	C11orf58	0.5344045	1.56E-14
LIPT1	ARV1	0.53446943	1.54E-14
LIPT1	NFYA	0.53452713	1.53E-14
LIPT1	TRIM34	0.53462372	1.51E-14
LIPT1	SLC35A5	0.53488613	1.46E-14
LIPT1	PARG	0.53493102	1.45E-14
LIPT1	GMCL1	0.53520128	1.4E-14
LIPT1	GNPAT	0.53543034	1.36E-14
LIPT1	PRMT2	0.5355921	1.33E-14
LIPT1	PIAS1	0.53588422	1.28E-14
LIPT1	ADAT1	0.53604158	1.25E-14
LIPT1	POLDIP3	0.53604356	1.25E-14
LIPT1	RPL7L1	0.53606218	1.25E-14
LIPT1	AC106886.5	0.53614332	1.23E-14
LIPT1	CXorf56	0.53625379	1.22E-14
LIPT1	USP10	0.53645559	1.18E-14
LIPT1	ZFP90	0.53659341	1.16E-14
LIPT1	MED6	0.53659676	1.16E-14
LIPT1	KBTBD4	0.53661263	1.16E-14
LIPT1	MBNL1	0.53678472	1.13E-14
LIPT1	SUMO1	0.53682137	1.13E-14
LIPT1	ING4	0.53688592	1.12E-14
LIPT1	TMEM267	0.53696501	1.1E-14
LIPT1	TEP1	0.53711614	1.08E-14
LIPT1	NAA16	0.53715802	1.08E-14
LIPT1	ALS2	0.53739095	1.04E-14
LIPT1	TMEM251	0.53755948	1.02E-14
LIPT1	RAP1A	0.53756385	1.02E-14
LIPT1	NFKB1	0.53762031	1.01E-14
LIPT1	CDKN2AIP	0.53765393	1.01E-14
LIPT1	HPS3	0.53766611	1.01E-14
LIPT1	ARMC8	0.53774359	9.95E-15
LIPT1	PPFIA1	0.53776601	9.92E-15
LIPT1	DNAJC21	0.53782246	9.84E-15
LIPT1	API5	0.53796181	9.66E-15
LIPT1	FASTKD5	0.5379708	9.65E-15
LIPT1	INTS12	0.5381289	9.44E-15
LIPT1	STAT3	0.53815423	9.41E-15
LIPT1	DRAM2	0.53825733	9.28E-15
LIPT1	PLEKHB2	0.53830953	9.21E-15
LIPT1	MTERF3	0.53837635	9.13E-15
LIPT1	LRCH1	0.53841139	9.09E-15
LIPT1	XIAP	0.53853278	8.94E-15
LIPT1	CNST	0.53888768	8.52E-15

LIPT1	TAF1B	0.5389111	8.49E-15
LIPT1	EFCAB14	0.53891192	8.49E-15
LIPT1	GPATCH11	0.53912871	8.25E-15
LIPT1	R3HCC1L	0.53913644	8.24E-15
LIPT1	ETV6	0.53924661	8.12E-15
LIPT1	ZNF260	0.539321	8.04E-15
LIPT1	ZMAT2	0.53936566	7.99E-15
LIPT1	SMIM30	0.53949574	7.85E-15
LIPT1	SLC35A4	0.53979612	7.53E-15
LIPT1	RAB28	0.53988375	7.44E-15
LIPT1	ESF1	0.53989813	7.43E-15
LIPT1	C16orf72	0.53991055	7.42E-15
LIPT1	AC010132.3	0.54001771	7.31E-15
LIPT1	PEX2	0.54008586	7.24E-15
LIPT1	TAF7	0.54018545	7.14E-15
LIPT1	RNF170	0.54019661	7.13E-15
LIPT1	ITM2B	0.54021679	7.11E-15
LIPT1	RHBDD1	0.54027526	7.06E-15
LIPT1	GNAQ	0.5402998	7.03E-15
LIPT1	EIF1B	0.54052186	6.83E-15
LIPT1	DIDO1	0.54067357	6.69E-15
LIPT1	PBDC1	0.54073302	6.63E-15
LIPT1	ELF1	0.54080956	6.56E-15
LIPT1	RAB14	0.54087043	6.51E-15
LIPT1	TIPRL	0.54088171	6.5E-15
LIPT1	DHX36	0.5409189	6.47E-15
LIPT1	FBXL3	0.54092826	6.46E-15
LIPT1	DEDD	0.54092875	6.46E-15
LIPT1	PTPN9	0.5410606	6.34E-15
LIPT1	BABAM2	0.54113996	6.27E-15
LIPT1	HNRNPUL1	0.54119629	6.23E-15
LIPT1	FEM1B	0.54126359	6.17E-15
LIPT1	GPATCH2	0.54130128	6.14E-15
LIPT1	GLE1	0.54146972	6E-15
LIPT1	NIP7	0.54151109	5.96E-15
LIPT1	STX6	0.54158753	5.9E-15
LIPT1	TRIP4	0.54228947	5.36E-15
LIPT1	BPNT1	0.54234626	5.32E-15
LIPT1	FBXO42	0.54254105	5.18E-15
LIPT1	C6orf47	0.54300162	4.86E-15
LIPT1	HPS5	0.54321459	4.72E-15
LIPT1	RPA2	0.54325978	4.69E-15
LIPT1	CDC23	0.54331767	4.66E-15
LIPT1	PANK2	0.54346855	4.56E-15
LIPT1	RABIF	0.54351099	4.53E-15
LIPT1	TMEM9B	0.54370237	4.42E-15
LIPT1	ESD	0.54375949	4.38E-15
LIPT1	PYCR2	0.54383804	4.33E-15
LIPT1	ERBIN	0.54417452	4.14E-15
LIPT1	KLHL20	0.54424536	4.1E-15
LIPT1	TRMT6	0.54428507	4.08E-15
LIPT1	HDAC1	0.54458321	3.91E-15
LIPT1	AP003108.2	0.54463042	3.89E-15
LIPT1	TRAFD1	0.54476089	3.82E-15
LIPT1	SDE2	0.54476451	3.81E-15
LIPT1	CROT	0.54509881	3.64E-15
LIPT1	DNAJC13	0.54510993	3.64E-15
LIPT1	ROCK1	0.54553435	3.43E-15

LIPT1	SP1	0.54560065	3.4E-15
LIPT1	GPALPP1	0.54564172	3.38E-15
LIPT1	CDC42	0.54566511	3.37E-15
LIPT1	RNF220	0.54570461	3.35E-15
LIPT1	VIPAS39	0.54584963	3.28E-15
LIPT1	DDX6	0.54585904	3.28E-15
LIPT1	TARS2	0.54601761	3.21E-15
LIPT1	TAF15	0.54626052	3.1E-15
LIPT1	TUT7	0.54635803	3.06E-15
LIPT1	ORC4	0.54673931	2.9E-15
LIPT1	ASB7	0.546992	2.8E-15
LIPT1	HMGN1	0.54718313	2.73E-15
LIPT1	AK6	0.54723255	2.71E-15
LIPT1	SUZ12	0.54747269	2.62E-15
LIPT1	USP1	0.54749978	2.61E-15
LIPT1	NIPSNAP3A	0.54752325	2.6E-15
LIPT1	LPAR6	0.54778354	2.51E-15
LIPT1	LARP4B	0.54783313	2.49E-15
LIPT1	BRD7	0.54783395	2.49E-15
LIPT1	SH3BGRL	0.54799251	2.44E-15
LIPT1	NT5C2	0.54800645	2.43E-15
LIPT1	NOD1	0.54810327	2.4E-15
LIPT1	PPIL3	0.54841239	2.3E-15
LIPT1	MRFAP1L1	0.54848057	2.28E-15
LIPT1	LACC1	0.54854365	2.26E-15
LIPT1	PI4KB	0.54905285	2.1E-15
LIPT1	SPOPL	0.54908968	2.09E-15
LIPT1	ATF2	0.54925922	2.04E-15
LIPT1	ALDH9A1	0.54926652	2.04E-15
LIPT1	PRPF38A	0.54936455	2.01E-15
LIPT1	EXOSC9	0.54939788	2E-15
LIPT1	CBFB	0.54947077	1.98E-15
LIPT1	ITGB3BP	0.54972364	1.91E-15
LIPT1	TAF8	0.54975237	1.9E-15
LIPT1	TMEM87B	0.55008497	1.82E-15
LIPT1	SMARCAL1	0.55009185	1.82E-15
LIPT1	BAG5	0.55029027	1.77E-15
LIPT1	TOR1AIP1	0.55029658	1.76E-15
LIPT1	CNPPD1	0.55034212	1.75E-15
LIPT1	RPRD2	0.55067754	1.67E-15
LIPT1	TRIM56	0.55078029	1.65E-15
LIPT1	ARHGAP12	0.55117716	1.56E-15
LIPT1	SNAP23	0.55163635	1.46E-15
LIPT1	RPP38	0.55167702	1.45E-15
LIPT1	VHL	0.5517964	1.43E-15
LIPT1	DDX19B	0.55187775	1.41E-15
LIPT1	ANKRD13C	0.55195293	1.4E-15
LIPT1	PDCL3	0.55209868	1.37E-15
LIPT1	MAP3K2	0.55245416	1.3E-15
LIPT1	STK4	0.55250656	1.29E-15
LIPT1	CTDSP1	0.55256775	1.28E-15
LIPT1	TERF1	0.55263345	1.27E-15
LIPT1	FRYL	0.55281607	1.24E-15
LIPT1	RBM12	0.55281886	1.24E-15
LIPT1	BECN1	0.55292786	1.22E-15
LIPT1	ISG20L2	0.55299352	1.21E-15
LIPT1	MRPL50	0.55323088	1.17E-15
LIPT1	AMBRA1	0.55326079	1.16E-15

LIPT1	ABHD13	0.55347251	1.13E-15
LIPT1	DCTD	0.55349986	1.12E-15
LIPT1	RAB5B	0.55370124	1.09E-15
LIPT1	DCAF6	0.55370181	1.09E-15
LIPT1	PHF20	0.55371984	1.09E-15
LIPT1	POLR2D	0.55402102	1.04E-15
LIPT1	TRIM26	0.55417995	1.02E-15
LIPT1	ZFAND6	0.55427372	1E-15
LIPT1	UGCG	0.55428004	1E-15
LIPT1	HNRNPF	0.55430077	1E-15
LIPT1	XRCC5	0.55430113	1E-15
LIPT1	FBXO28	0.55446437	9.78E-16
LIPT1	MAT2A	0.55458361	9.61E-16
LIPT1	PDCL	0.554587	9.61E-16
LIPT1	TTC4	0.55469472	9.46E-16
LIPT1	SNX2	0.55483214	9.28E-16
LIPT1	ZNF274	0.55486691	9.23E-16
LIPT1	DDX50	0.55500677	9.05E-16
LIPT1	PYGO2	0.5550982	8.93E-16
LIPT1	SLC24A1	0.55517796	8.83E-16
LIPT1	ZNF200	0.5553418	8.62E-16
LIPT1	CCDC32	0.55564586	8.26E-16
LIPT1	SNW1	0.55566204	8.24E-16
LIPT1	CDC73	0.55567878	8.22E-16
LIPT1	PKNOX1	0.55567957	8.22E-16
LIPT1	BLOC1S2	0.55589467	7.97E-16
LIPT1	LNPEP	0.55591122	7.95E-16
LIPT1	PCBP1	0.55594647	7.91E-16
LIPT1	ZNF184	0.55596448	7.89E-16
LIPT1	MED21	0.55601411	7.83E-16
LIPT1	NUP98	0.55626527	7.55E-16
LIPT1	RPF1	0.55636312	7.45E-16
LIPT1	OGT	0.55637675	7.43E-16
LIPT1	LRCH3	0.5565139	7.29E-16
LIPT1	TM2D1	0.55651985	7.28E-16
LIPT1	ZBTB6	0.55665713	7.14E-16
LIPT1	UHMK1	0.55673478	7.06E-16
LIPT1	SH3BP5L	0.5567726	7.02E-16
LIPT1	PRPF18	0.55687195	6.92E-16
LIPT1	ATF6B	0.55708089	6.72E-16
LIPT1	GMEB1	0.55710332	6.7E-16
LIPT1	ZNF227	0.55722945	6.58E-16
LIPT1	MBD5	0.55740827	6.41E-16
LIPT1	IL7	0.55754776	6.28E-16
LIPT1	PPHLN1	0.55757716	6.26E-16
LIPT1	SPAST	0.55778176	6.07E-16
LIPT1	SPRTN	0.55779182	6.07E-16
LIPT1	USP4	0.55782536	6.04E-16
LIPT1	BTF3	0.55791471	5.96E-16
LIPT1	BMS1	0.55811091	5.79E-16
LIPT1	PPP4R3B	0.55821976	5.7E-16
LIPT1	UBQLN2	0.55822608	5.7E-16
LIPT1	IPO11	0.55844908	5.52E-16
LIPT1	MAPK14	0.55848935	5.48E-16
LIPT1	VPS45	0.55859213	5.4E-16
LIPT1	CIAPIN1	0.55869374	5.32E-16
LIPT1	RABGAP1L	0.55876632	5.27E-16
LIPT1	CAPRIN1	0.55882898	5.22E-16

LIPT1	ASNSD1	0.55884443	5.21E-16
LIPT1	TMEM138	0.55884961	5.21E-16
LIPT1	INTS5	0.55890812	5.16E-16
LIPT1	PARP8	0.55891844	5.15E-16
LIPT1	MFSD14B	0.55900708	5.09E-16
LIPT1	AQR	0.55914495	4.99E-16
LIPT1	PLEKHF2	0.55925033	4.91E-16
LIPT1	ANAPC10	0.55930071	4.88E-16
LIPT1	TAOK3	0.55939043	4.81E-16
LIPT1	WAC	0.55945847	4.77E-16
LIPT1	SF3B3	0.5596149	4.66E-16
LIPT1	ACAP2	0.55964719	4.64E-16
LIPT1	ANKRD42	0.55978635	4.54E-16
LIPT1	RNPC3	0.55981999	4.52E-16
LIPT1	GMPR2	0.55987497	4.49E-16
LIPT1	PUM1	0.55996747	4.43E-16
LIPT1	NIPBL	0.56038486	4.17E-16
LIPT1	TMEM183A	0.56066053	4E-16
LIPT1	GABARAPL2	0.56070373	3.98E-16
LIPT1	NCBP2	0.56096269	3.83E-16
LIPT1	ABI1	0.56112303	3.74E-16
LIPT1	CXorf38	0.56115069	3.73E-16
LIPT1	C5orf24	0.56135754	3.62E-16
LIPT1	RMDN2	0.56158642	3.5E-16
LIPT1	MRPL30	0.56160004	3.49E-16
LIPT1	PRUNE1	0.56167299	3.45E-16
LIPT1	THAP11	0.56180836	3.38E-16
LIPT1	ERF	0.56190767	3.34E-16
LIPT1	HNRNPC	0.56194079	3.32E-16
LIPT1	TPP2	0.56203603	3.27E-16
LIPT1	TBL1XR1	0.56207809	3.25E-16
LIPT1	ZC3H13	0.56221317	3.19E-16
LIPT1	SVIP	0.56252831	3.05E-16
LIPT1	PPP1R15B	0.5627904	2.93E-16
LIPT1	KATNA1	0.56287546	2.89E-16
LIPT1	RRN3	0.56289918	2.88E-16
LIPT1	CZIB	0.56311979	2.79E-16
LIPT1	QTRT2	0.56314965	2.78E-16
LIPT1	TGS1	0.56328527	2.73E-16
LIPT1	YPEL5	0.56331645	2.71E-16
LIPT1	SRFBP1	0.5633621	2.7E-16
LIPT1	WDR82	0.56352221	2.63E-16
LIPT1	PURB	0.5635576	2.62E-16
LIPT1	HMGB1	0.56358208	2.61E-16
LIPT1	POLR2C	0.56358908	2.61E-16
LIPT1	RNF168	0.56361478	2.6E-16
LIPT1	ZNF766	0.56367991	2.57E-16
LIPT1	SLC30A5	0.56368982	2.57E-16
LIPT1	HNRNPLL	0.5638545	2.51E-16
LIPT1	TFCP2	0.56386337	2.5E-16
LIPT1	SPIDR	0.56386966	2.5E-16
LIPT1	CLDND1	0.56415242	2.4E-16
LIPT1	MED18	0.56415628	2.4E-16
LIPT1	WDR20	0.56483887	2.17E-16
LIPT1	PPM1B	0.56488898	2.15E-16
LIPT1	SFT2D2	0.56508913	2.09E-16
LIPT1	YY1AP1	0.56547297	1.97E-16
LIPT1	TOE1	0.56602671	1.82E-16

LIPT1	CASP3	0.56612993	1.79E-16
LIPT1	TTC9C	0.56617438	1.78E-16
LIPT1	MOB1A	0.5662503	1.76E-16
LIPT1	MFAP1	0.56626139	1.76E-16
LIPT1	CPSF6	0.56640987	1.72E-16
LIPT1	CDC42SE1	0.56651745	1.69E-16
LIPT1	CFAP20	0.56658602	1.67E-16
LIPT1	RCBTB2	0.56673045	1.64E-16
LIPT1	WDR26	0.56682756	1.61E-16
LIPT1	STX12	0.56688875	1.6E-16
LIPT1	GTF2B	0.56721009	1.53E-16
LIPT1	ATXN7L3	0.56740262	1.48E-16
LIPT1	TGOLN2	0.56784217	1.39E-16
LIPT1	SLC4A1AP	0.56784923	1.39E-16
LIPT1	ARHGAP17	0.56794477	1.37E-16
LIPT1	ANKMY2	0.56802276	1.35E-16
LIPT1	POGK	0.56805758	1.34E-16
LIPT1	ZNF45	0.56810366	1.34E-16
LIPT1	AFTPH	0.56822401	1.31E-16
LIPT1	NUP62	0.5684416	1.27E-16
LIPT1	WAPL	0.56870988	1.22E-16
LIPT1	PEX19	0.56877853	1.21E-16
LIPT1	HNRNPK	0.56882789	1.2E-16
LIPT1	ZNHIT6	0.56886327	1.19E-16
LIPT1	RUFY1	0.5689656	1.17E-16
LIPT1	TAF9	0.56950227	1.08E-16
LIPT1	AGGF1	0.56953594	1.08E-16
LIPT1	RAB3GAP2	0.56961048	1.07E-16
LIPT1	TRMT10C	0.56980784	1.03E-16
LIPT1	HSPA14	0.57063606	9.13E-17
LIPT1	GRK2	0.57075979	8.96E-17
LIPT1	C2CD3	0.57100581	8.64E-17
LIPT1	SIN3A	0.57104458	8.59E-17
LIPT1	C1D	0.57137642	8.17E-17
LIPT1	UBAP2L	0.57153558	7.97E-17
LIPT1	RASA2	0.57159823	7.9E-17
LIPT1	RNF34	0.57160461	7.89E-17
LIPT1	PIGC	0.57163908	7.85E-17
LIPT1	SMN1	0.57177576	7.69E-17
LIPT1	PARN	0.57194118	7.5E-17
LIPT1	TRMT1L	0.57218338	7.23E-17
LIPT1	PUM2	0.57225487	7.15E-17
LIPT1	PRRC2C	0.57256106	6.83E-17
LIPT1	ZNF131	0.57257379	6.81E-17
LIPT1	CLP1	0.57272073	6.66E-17
LIPT1	CCDC90B	0.57276326	6.62E-17
LIPT1	ZNF410	0.57311838	6.27E-17
LIPT1	ADAR	0.57329663	6.11E-17
LIPT1	SNX1	0.57361113	5.82E-17
LIPT1	CLNS1A	0.57383438	5.63E-17
LIPT1	TPR	0.57394117	5.54E-17
LIPT1	DAXX	0.57411594	5.39E-17
LIPT1	THYN1	0.57462018	4.99E-17
LIPT1	USP39	0.57497597	4.73E-17
LIPT1	DYNC2LI1	0.57531017	4.49E-17
LIPT1	TRMT2B	0.57567943	4.24E-17
LIPT1	MAP3K7	0.57600008	4.04E-17
LIPT1	MCMBP	0.5761054	3.98E-17

LIPT1	MSL2	0.57630755	3.85E-17
LIPT1	DDX18	0.5764259	3.79E-17
LIPT1	SRP9	0.57653705	3.72E-17
LIPT1	NUFIP2	0.57665664	3.65E-17
LIPT1	DCLRE1C	0.57670962	3.62E-17
LIPT1	CBLB	0.57700794	3.46E-17
LIPT1	SDHD	0.57742424	3.25E-17
LIPT1	NIF3L1	0.57743796	3.24E-17
LIPT1	NKRF	0.5784479	2.77E-17
LIPT1	ZBTB22	0.57878844	2.63E-17
LIPT1	TSNAX	0.57893899	2.57E-17
LIPT1	DNAJC8	0.57894175	2.57E-17
LIPT1	COG2	0.57951844	2.35E-17
LIPT1	KBTBD2	0.57992655	2.2E-17
LIPT1	HARS2	0.58000017	2.18E-17
LIPT1	PIP5K1A	0.58010716	2.14E-17
LIPT1	CEBPZOS	0.58013628	2.13E-17
LIPT1	SNAPIN	0.58028613	2.08E-17
LIPT1	CDK12	0.58047795	2.02E-17
LIPT1	TMEM35B	0.58068949	1.96E-17
LIPT1	MRPS27	0.5807069	1.95E-17
LIPT1	ZNF143	0.58071632	1.95E-17
LIPT1	SNUPN	0.58086248	1.9E-17
LIPT1	RNASEL	0.58138003	1.76E-17
LIPT1	TBK1	0.5813883	1.75E-17
LIPT1	ZNF586	0.58146771	1.73E-17
LIPT1	TTC1	0.5815941	1.7E-17
LIPT1	NSL1	0.58186213	1.63E-17
LIPT1	SAP130	0.58190542	1.62E-17
LIPT1	METTL4	0.58207578	1.58E-17
LIPT1	MIER1	0.58217899	1.55E-17
LIPT1	AIMP1	0.582645	1.44E-17
LIPT1	SNRNP27	0.58370585	1.22E-17
LIPT1	C16orf70	0.58403889	1.16E-17
LIPT1	GPBP1L1	0.58429135	1.11E-17
LIPT1	TRMT61B	0.58432332	1.11E-17
LIPT1	CCDC97	0.58437169	1.1E-17
LIPT1	EPS15	0.5844254	1.09E-17
LIPT1	IK	0.58444192	1.09E-17
LIPT1	ENSA	0.5844886	1.08E-17
LIPT1	ANP32E	0.58473819	1.04E-17
LIPT1	CWF19L2	0.58482252	1.02E-17
LIPT1	KDM3B	0.58483252	1.02E-17
LIPT1	MOB4	0.58493347	1.01E-17
LIPT1	HNRNPUL2	0.58509282	9.8E-18
LIPT1	HNRNPR	0.5851112	9.77E-18
LIPT1	TAF5L	0.58534419	9.42E-18
LIPT1	GLYR1	0.58545641	9.25E-18
LIPT1	PPP3R1	0.58547082	9.23E-18
LIPT1	WDR89	0.58573726	8.85E-18
LIPT1	CDC42SE2	0.58599552	8.49E-18
LIPT1	LYST	0.58602079	8.46E-18
LIPT1	BROX	0.58602413	8.46E-18
LIPT1	BTN2A1	0.58607228	8.39E-18
LIPT1	KATNBL1	0.58627177	8.13E-18
LIPT1	ADO	0.58631902	8.07E-18
LIPT1	PPP1R2	0.58633038	8.05E-18
LIPT1	BUD13	0.58703318	7.2E-18

LIPT1	SNX27	0.58706138	7.17E-18
LIPT1	CNOT1	0.5872207	6.99E-18
LIPT1	KLHL12	0.58725724	6.95E-18
LIPT1	TXNDC9	0.58756603	6.62E-18
LIPT1	TMEM170A	0.58804998	6.12E-18
LIPT1	ZNF552	0.58810412	6.07E-18
LIPT1	COX15	0.58840329	5.79E-18
LIPT1	HNRNPH2	0.5886575	5.56E-18
LIPT1	MICU2	0.58890968	5.34E-18
LIPT1	LINS1	0.58937459	4.95E-18
LIPT1	POLR3C	0.58968459	4.71E-18
LIPT1	MED4	0.59110512	3.75E-18
LIPT1	INTS14	0.59135524	3.6E-18
LIPT1	SHOC2	0.59171781	3.39E-18
LIPT1	GABPB1	0.59180065	3.35E-18
LIPT1	VTI1A	0.5918932	3.3E-18
LIPT1	RBM4	0.59223646	3.12E-18
LIPT1	DENND6A	0.59299943	2.76E-18
LIPT1	USP8	0.59307917	2.72E-18
LIPT1	HNRNPA2B1	0.5935889	2.5E-18
LIPT1	MBTPS1	0.59378972	2.42E-18
LIPT1	EAPP	0.59401606	2.34E-18
LIPT1	AP3B1	0.59407857	2.31E-18
LIPT1	FIP1L1	0.5941435	2.29E-18
LIPT1	MATR3	0.59437727	2.2E-18
LIPT1	RTF1	0.59440893	2.19E-18
LIPT1	CDKN1B	0.59443194	2.18E-18
LIPT1	ABRAXAS2	0.5944442	2.18E-18
LIPT1	RBMX2	0.59487681	2.03E-18
LIPT1	EXOC1	0.59502903	1.98E-18
LIPT1	PIBF1	0.59524131	1.91E-18
LIPT1	TLK1	0.59547555	1.84E-18
LIPT1	RBM43	0.59568301	1.78E-18
LIPT1	KDM2A	0.59574676	1.76E-18
LIPT1	PPP1R8	0.59577783	1.75E-18
LIPT1	CCNDBP1	0.59605105	1.67E-18
LIPT1	THRAP3	0.59630372	1.61E-18
LIPT1	ZNF830	0.59669915	1.5E-18
LIPT1	ZMYM5	0.59694543	1.45E-18
LIPT1	CRLF3	0.59789039	1.24E-18
LIPT1	BBS2	0.59816316	1.18E-18
LIPT1	HNRNPU	0.59917978	9.98E-19
LIPT1	TOP2B	0.60021156	8.41E-19
LIPT1	FGFR1OP2	0.60068804	7.77E-19
LIPT1	ELK4	0.60079586	7.63E-19
LIPT1	GGPS1	0.60097378	7.4E-19
LIPT1	TADA1	0.60175458	6.5E-19
LIPT1	KRCC1	0.60204522	6.19E-19
LIPT1	TARDBP	0.60266635	5.57E-19
LIPT1	C9orf78	0.60284445	5.41E-19
LIPT1	MAX	0.60290792	5.35E-19
LIPT1	CTR9	0.60301635	5.26E-19
LIPT1	ICE2	0.60322632	5.07E-19
LIPT1	DNAJA2	0.60339621	4.93E-19
LIPT1	RBBP5	0.60404484	4.42E-19
LIPT1	DYNC1I2	0.60413641	4.35E-19
LIPT1	UBA3	0.60418229	4.32E-19
LIPT1	ARL14EP	0.60423798	4.28E-19

LIPT1	CHD9	0.60474319	3.93E-19
LIPT1	TERF2	0.60508139	3.71E-19
LIPT1	RAB3GAP1	0.60524957	3.6E-19
LIPT1	HNRNPD	0.60534953	3.54E-19
LIPT1	ECD	0.60643961	2.94E-19
LIPT1	ZDHHC6	0.60654369	2.89E-19
LIPT1	NUP133	0.60679272	2.77E-19
LIPT1	CNOT11	0.60733977	2.52E-19
LIPT1	DDX19A	0.60824346	2.16E-19
LIPT1	NSRP1	0.6088533	1.95E-19
LIPT1	GCC2	0.60907388	1.87E-19
LIPT1	USP34	0.60961014	1.71E-19
LIPT1	ZCCHC10	0.61000156	1.6E-19
LIPT1	ZMYM2	0.61042435	1.49E-19
LIPT1	CNOT9	0.61062332	1.43E-19
LIPT1	INO80	0.61106413	1.33E-19
LIPT1	WASHC2A	0.61121805	1.29E-19
LIPT1	JRKL	0.611257	1.29E-19
LIPT1	DPF2	0.6114579	1.24E-19
LIPT1	UQCRC2	0.61171735	1.19E-19
LIPT1	EPC2	0.61185024	1.16E-19
LIPT1	ZNF207	0.61216857	1.1E-19
LIPT1	TRA2B	0.61253936	1.03E-19
LIPT1	MED17	0.61254124	1.03E-19
LIPT1	LDB1	0.61318558	9.19E-20
LIPT1	STARD7	0.61446827	7.35E-20
LIPT1	METTL18	0.61460067	7.18E-20
LIPT1	PHACTR4	0.61480554	6.92E-20
LIPT1	DHX9	0.61508069	6.6E-20
LIPT1	NPAT	0.616707	4.95E-20
LIPT1	TMEM126B	0.61722049	4.52E-20
LIPT1	PSPC1	0.61763878	4.2E-20
LIPT1	SP3	0.6179733	3.96E-20
LIPT1	PKP4	0.61922582	3.17E-20
LIPT1	CNOT8	0.62081204	2.38E-20
LIPT1	MED7	0.62103241	2.29E-20
LIPT1	FBXO38	0.6215188	2.1E-20
LIPT1	UBLCP1	0.62251898	1.75E-20
LIPT1	DYRK1A	0.62294253	1.63E-20
LIPT1	KHDRBS1	0.62313438	1.57E-20
LIPT1	MAT2B	0.62417346	1.3E-20
LIPT1	MRPS31	0.62439913	1.25E-20
LIPT1	IRF2	0.62493978	1.13E-20
LIPT1	SRSF3	0.62497425	1.12E-20
LIPT1	PNRC2	0.62534176	1.05E-20
LIPT1	ZNF174	0.62544309	1.03E-20
LIPT1	DCP2	0.625924	9.45E-21
LIPT1	GPBP1	0.62680406	8.05E-21
LIPT1	RFX5	0.62782738	6.67E-21
LIPT1	TMEM216	0.62821346	6.21E-21
LIPT1	RNF111	0.6284588	5.94E-21
LIPT1	PPP1R21	0.62853773	5.85E-21
LIPT1	PLRG1	0.62912724	5.25E-21
LIPT1	ZC3H15	0.62949744	4.9E-21
LIPT1	UVRAG	0.62954131	4.86E-21
LIPT1	SETX	0.63183747	3.17E-21
LIPT1	SNRNP200	0.63197323	3.09E-21
LIPT1	POLR3GL	0.63277043	2.66E-21

LIPT1	DUSP11	0.63327357	2.42E-21
LIPT1	ACVR2A	0.63418522	2.04E-21
LIPT1	TSN	0.63497624	1.76E-21
LIPT1	RBM22	0.63596757	1.46E-21
LIPT1	MTA2	0.63606025	1.43E-21
LIPT1	SYF2	0.63638523	1.35E-21
LIPT1	ZNF644	0.63730317	1.13E-21
LIPT1	FAM204A	0.63966114	7.21E-22
LIPT1	TANK	0.63999951	6.76E-22
LIPT1	ZC3H11A	0.64054931	6.08E-22
LIPT1	LARP7	0.64406239	3.08E-22
LIPT1	ZNF512	0.64453711	2.8E-22
LIPT1	SSB	0.64503378	2.54E-22
LIPT1	JMJD1C	0.64649693	1.91E-22
LIPT1	SRSF7	0.64778008	1.48E-22
LIPT1	MITD1	0.64831527	1.33E-22
LIPT1	NFE2L2	0.64881963	1.21E-22
LIPT1	CCDC115	0.64945525	1.06E-22
LIPT1	CSTF2T	0.65136648	7.25E-23
LIPT1	YTHDF2	0.65290606	5.32E-23
LIPT1	ZNF263	0.65297204	5.25E-23
LIPT1	SRSF1	0.66314731	6.46E-24
LIPT1	CTCF	0.66787503	2.38E-24
LIPT1	ZCCHC9	0.66790798	2.36E-24
LIPT1	PPIG	0.66867217	2E-24
LIPT1	UTP3	0.67205883	9.65E-25
LIPT1	IST1	0.67358707	6.92E-25
LIPT1	SIAH1	0.67450657	5.66E-25
LIPT1	UNC50	0.6749671	5.11E-25
LIPT1	IWS1	0.68513669	5.23E-26
LIPT1	MPHOSPH10	0.68565024	4.65E-26
LIPT1	TRIM13	0.69155449	1.18E-26
LIPT1	CWC22	0.69198281	1.07E-26
LIPT1	CIR1	0.71723705	2.05E-29
LIPT2	FARP2	0.50421162	7.22E-13
LIPT2	ATXN7L3B	0.50422928	7.2E-13
LIPT2	FRYL	0.50596063	5.84E-13
LIPT2	PLCB4	0.50707963	5.09E-13
LIPT2	HPS6	0.50716457	5.04E-13
LIPT2	NAPEPLD	0.50817778	4.45E-13
LIPT2	DDX23	0.50890627	4.07E-13
LIPT2	SEPHS2	0.50942626	3.82E-13
LIPT2	MPC2	0.50954045	3.77E-13
LIPT2	AACS	0.50971457	3.69E-13
LIPT2	KLHL23	0.50984267	3.63E-13
LIPT2	LEO1	0.51069977	3.27E-13
LIPT2	CCDC34	0.51116105	3.09E-13
LIPT2	HOMEZ	0.51295709	2.47E-13
LIPT2	TDRD7	0.51734579	1.42E-13
LIPT2	NAT1	0.51814077	1.29E-13
LIPT2	STARD7	0.5206768	9.34E-14
LIPT2	CES2	0.52301574	6.92E-14
LIPT2	TMEM177	0.52316904	6.79E-14
LIPT2	SLC37A1	0.52383261	6.23E-14
LIPT2	TFCP2	0.52391328	6.17E-14
LIPT2	CEBPG	0.52431824	5.85E-14
LIPT2	ETNK1	0.52580566	4.83E-14
LIPT2	COLCA2	0.52666125	4.32E-14

LIPT2	URGCP	0.52697884	4.14E-14
LIPT2	LRRC75A	0.52715132	4.05E-14
LIPT2	AFG3L2	0.5283677	3.46E-14
LIPT2	MS4A8	0.52918321	3.11E-14
LIPT2	PRKAB1	0.52927293	3.07E-14
LIPT2	VIPR1	0.5300881	2.76E-14
LIPT2	DOP1B	0.53238229	2.04E-14
LIPT2	ZNF564	0.53701645	1.1E-14
LIPT2	TIGD2	0.53781984	9.84E-15
LIPT2	SH3BGRL2	0.53981353	7.52E-15
LIPT2	SIMC1	0.5423325	5.33E-15
LIPT2	KRCC1	0.54273156	5.05E-15
LIPT2	RNF34	0.54343511	4.58E-15
LIPT2	GCC1	0.54416	4.15E-15
LIPT2	INTS14	0.54595701	3.23E-15
LIPT2	C2orf15	0.546464	3.01E-15
LIPT2	COG2	0.55074779	1.66E-15
LIPT2	ZNF398	0.55221698	1.35E-15
LIPT2	KCNE3	0.55415227	1.02E-15
LIPT2	TPD52	0.56199166	3.3E-16
LIPT2	LRRC66	0.56224899	3.17E-16
LIPT2	PDSS1	0.56343017	2.67E-16
LIPT2	FASTKD5	0.56867495	1.23E-16
LIPT2	TOE1	0.57189616	7.55E-17
LIPT2	BRI3BP	0.58688776	7.37E-18
LIPT2	YAE1	0.60232716	5.9E-19
LIPT2	HYLS1	0.64364874	3.33E-22
LIPT2	ANKS4B	0.64479565	2.67E-22
MTF1	COMTD1	-0.5041829	7.24E-13
MTF1	GRK6	0.5001454	1.18E-12
MTF1	GFOD2	0.5002633	1.16E-12
MTF1	SIPA1L2	0.50043251	1.14E-12
MTF1	METTL6	0.5005813	1.12E-12
MTF1	UBE2D1	0.50059535	1.11E-12
MTF1	RUFY1	0.50071674	1.1E-12
MTF1	PAK1IP1	0.50072806	1.1E-12
MTF1	C8orf33	0.50077919	1.09E-12
MTF1	APH1B	0.50083953	1.08E-12
MTF1	NECTIN3	0.50091224	1.07E-12
MTF1	RASSF2	0.50091589	1.07E-12
MTF1	TIPARP	0.50096123	1.07E-12
MTF1	ZDHHC5	0.50135004	1.02E-12
MTF1	PARVA	0.50137775	1.01E-12
MTF1	JHY	0.5014646	1E-12
MTF1	CALHM2	0.50148766	1E-12
MTF1	CNOT1	0.50152283	9.97E-13
MTF1	PRKCA	0.50162331	9.86E-13
MTF1	GON7	0.50162645	9.85E-13
MTF1	MBNL3	0.50163922	9.84E-13
MTF1	UBE2K	0.50164044	9.83E-13
MTF1	CEBPG	0.50166908	9.8E-13
MTF1	ARHGAP11A	0.50175283	9.7E-13
MTF1	LITAF	0.50181593	9.63E-13
MTF1	TMCO1	0.50183633	9.61E-13
MTF1	ARFGEF1	0.50187664	9.56E-13
MTF1	PANK2	0.50190204	9.53E-13
MTF1	FAM171B	0.50191645	9.51E-13
MTF1	APAF1	0.50199203	9.43E-13

MTF1	ODF2L	0.50203356	9.38E-13
MTF1	HPS3	0.50233644	9.05E-13
MTF1	PRNP	0.50239733	8.98E-13
MTF1	LSM6	0.50243639	8.94E-13
MTF1	TUT7	0.50277174	8.59E-13
MTF1	MRPL50	0.50280589	8.55E-13
MTF1	PXMP4	0.50296998	8.38E-13
MTF1	TEK	0.50300539	8.35E-13
MTF1	DIDO1	0.50305217	8.3E-13
MTF1	CDK14	0.50305459	8.3E-13
MTF1	PI4K2A	0.50306013	8.29E-13
MTF1	ENPEP	0.50307251	8.28E-13
MTF1	GPBP1	0.50308397	8.27E-13
MTF1	CDK2	0.50342153	7.94E-13
MTF1	BBS2	0.50355662	7.81E-13
MTF1	NUDCD2	0.50381443	7.57E-13
MTF1	SGCD	0.50386581	7.52E-13
MTF1	CRTAP	0.5039642	7.44E-13
MTF1	TSPAN31	0.50398935	7.41E-13
MTF1	STX3	0.50405566	7.35E-13
MTF1	SNX5	0.50430871	7.13E-13
MTF1	SINHCAF	0.50438062	7.07E-13
MTF1	CD46	0.5046219	6.87E-13
MTF1	DNASE2	0.50463261	6.86E-13
MTF1	OSMR	0.50469487	6.81E-13
MTF1	AMOT	0.50478429	6.73E-13
MTF1	CLTC	0.50481724	6.71E-13
MTF1	UBE2I	0.50483794	6.69E-13
MTF1	SAP30	0.504974	6.58E-13
MTF1	TSG101	0.50500656	6.55E-13
MTF1	PRRG1	0.50521091	6.39E-13
MTF1	RER1	0.50556499	6.13E-13
MTF1	FGF7	0.5056183	6.09E-13
MTF1	OSTC	0.50565362	6.06E-13
MTF1	SSRP1	0.50571283	6.02E-13
MTF1	PKNOX1	0.50584256	5.92E-13
MTF1	HARS2	0.50586213	5.91E-13
MTF1	CSTF2	0.50588717	5.89E-13
MTF1	ATG7	0.5059609	5.84E-13
MTF1	SPG21	0.50596715	5.83E-13
MTF1	RAB11A	0.50602779	5.79E-13
MTF1	STK38L	0.50605981	5.77E-13
MTF1	MOB3B	0.50638248	5.55E-13
MTF1	UNC50	0.50649016	5.47E-13
MTF1	METTL21A	0.5064985	5.47E-13
MTF1	ATF6B	0.50650907	5.46E-13
MTF1	DPM1	0.50669546	5.34E-13
MTF1	ASCC3	0.50670186	5.33E-13
MTF1	TCEAL9	0.50729247	4.96E-13
MTF1	FBXO22	0.50744316	4.87E-13
MTF1	CETN3	0.50748658	4.85E-13
MTF1	C1GALT1C1	0.50750148	4.84E-13
MTF1	GIMAP6	0.50750153	4.84E-13
MTF1	CRISPLD2	0.50760239	4.78E-13
MTF1	EPB41L4A	0.50770941	4.72E-13
MTF1	DCAF6	0.50774643	4.7E-13
MTF1	FGL2	0.50792646	4.59E-13
MTF1	CLCN4	0.50809555	4.5E-13

MTF1	IKBIP	0.50809897	4.5E-13
MTF1	DCAF13	0.50811762	4.49E-13
MTF1	SPAG1	0.50816915	4.46E-13
MTF1	PREX1	0.5081913	4.45E-13
MTF1	HNRNPA1	0.50824215	4.42E-13
MTF1	HGF	0.508326	4.37E-13
MTF1	PCSK5	0.50833593	4.37E-13
MTF1	RARB	0.50834117	4.37E-13
MTF1	CDC42BPA	0.50852809	4.27E-13
MTF1	HMGB1	0.50871151	4.17E-13
MTF1	ATP6V1E1	0.50879975	4.13E-13
MTF1	SLC25A12	0.50882812	4.11E-13
MTF1	TTI2	0.50912756	3.96E-13
MTF1	TRUB2	0.50923776	3.91E-13
MTF1	TMEM165	0.50933665	3.86E-13
MTF1	ACTN1	0.50950643	3.78E-13
MTF1	VMA21	0.50956448	3.76E-13
MTF1	XRCC5	0.5095834	3.75E-13
MTF1	AK2	0.50978622	3.66E-13
MTF1	DGCR2	0.50984104	3.63E-13
MTF1	CCDC71	0.5098489	3.63E-13
MTF1	CDKN1B	0.51007914	3.53E-13
MTF1	B4GALT1	0.51024394	3.46E-13
MTF1	FAM104A	0.51027998	3.44E-13
MTF1	VGLL4	0.51047631	3.36E-13
MTF1	ERMP1	0.51057144	3.32E-13
MTF1	TRAPPC3	0.51064975	3.29E-13
MTF1	MYADM	0.51069063	3.27E-13
MTF1	TAF6	0.51071574	3.26E-13
MTF1	PDP1	0.51085415	3.2E-13
MTF1	VCAN	0.51086065	3.2E-13
MTF1	B3GNT9	0.51093034	3.17E-13
MTF1	ZDHHC20	0.51098876	3.15E-13
MTF1	AL139300.1	0.51119109	3.07E-13
MTF1	TRIM69	0.51123537	3.06E-13
MTF1	FER	0.51124558	3.05E-13
MTF1	NAMPT	0.51125142	3.05E-13
MTF1	ANP32E	0.51132608	3.02E-13
MTF1	BTN2A2	0.5121064	2.74E-13
MTF1	HSD17B12	0.51222143	2.71E-13
MTF1	DCBLD1	0.51230739	2.68E-13
MTF1	CKAP2	0.51252525	2.61E-13
MTF1	FERMT2	0.51253475	2.6E-13
MTF1	ZNF423	0.51259268	2.58E-13
MTF1	MRAS	0.51259401	2.58E-13
MTF1	KLF6	0.51269204	2.55E-13
MTF1	JMJD8	0.51292672	2.48E-13
MTF1	ATXN3	0.51296146	2.47E-13
MTF1	IL1R1	0.51296778	2.47E-13
MTF1	HSPA5	0.51297902	2.46E-13
MTF1	ENOX1	0.51300004	2.46E-13
MTF1	TSPAN14	0.51316855	2.41E-13
MTF1	PRKCH	0.51318856	2.4E-13
MTF1	SNRNP40	0.51356111	2.29E-13
MTF1	STOM	0.51362877	2.27E-13
MTF1	C1GALT1	0.51367824	2.26E-13
MTF1	GNPDA1	0.51384422	2.21E-13
MTF1	FAM177A1	0.5138855	2.2E-13

MTF1	RBMS1	0.51393527	2.19E-13
MTF1	ZNF521	0.51398714	2.17E-13
MTF1	TXNDC9	0.51414173	2.13E-13
MTF1	MRFAP1	0.51429134	2.09E-13
MTF1	FYB1	0.51473706	1.98E-13
MTF1	BNC2	0.5147689	1.97E-13
MTF1	TOR1AIP2	0.51477989	1.97E-13
MTF1	EIF5A2	0.51481649	1.96E-13
MTF1	IRS2	0.51485866	1.95E-13
MTF1	ADAMTS2	0.51495764	1.92E-13
MTF1	KCTD2	0.51499538	1.91E-13
MTF1	HDAC9	0.51517522	1.87E-13
MTF1	RAB31	0.51552254	1.79E-13
MTF1	FIP1L1	0.51553318	1.79E-13
MTF1	PEX13	0.51553929	1.79E-13
MTF1	EIF2AK1	0.51554277	1.79E-13
MTF1	MRM2	0.51582794	1.72E-13
MTF1	PRCP	0.51597983	1.69E-13
MTF1	THUMPD3	0.51598796	1.69E-13
MTF1	SEMA3C	0.51599255	1.69E-13
MTF1	ZFPM2	0.51632466	1.62E-13
MTF1	PRKG1	0.51637736	1.61E-13
MTF1	MXRA7	0.51642167	1.6E-13
MTF1	LIMK2	0.51645693	1.59E-13
MTF1	LLGL1	0.51651458	1.58E-13
MTF1	CAST	0.51670013	1.55E-13
MTF1	TMEM39B	0.51679421	1.53E-13
MTF1	KIFAP3	0.51689567	1.51E-13
MTF1	ELP3	0.51703111	1.48E-13
MTF1	RSU1	0.51707204	1.47E-13
MTF1	SNX7	0.51730651	1.43E-13
MTF1	VASH1	0.51746752	1.4E-13
MTF1	SLBP	0.51748833	1.4E-13
MTF1	ZNF250	0.51766126	1.37E-13
MTF1	SEL1L	0.51787049	1.33E-13
MTF1	DNAL1	0.51807863	1.3E-13
MTF1	ITGB8	0.51812633	1.29E-13
MTF1	ECHDC1	0.51812878	1.29E-13
MTF1	RALA	0.51813738	1.29E-13
MTF1	TNFRSF1A	0.51816659	1.28E-13
MTF1	SNRNP200	0.51827964	1.27E-13
MTF1	TM9SF3	0.51836496	1.25E-13
MTF1	SNAPC1	0.51845166	1.24E-13
MTF1	ABR	0.51847312	1.24E-13
MTF1	PRMT2	0.51853064	1.23E-13
MTF1	NCAPG2	0.51855434	1.22E-13
MTF1	PBX3	0.51863895	1.21E-13
MTF1	HNRNPM	0.51876862	1.19E-13
MTF1	RASA2	0.51881537	1.18E-13
MTF1	SPTSSA	0.51882093	1.18E-13
MTF1	DPYSL3	0.51890587	1.17E-13
MTF1	ALKBH1	0.51920689	1.13E-13
MTF1	UBA1	0.51922008	1.12E-13
MTF1	GLB1L	0.51934751	1.11E-13
MTF1	TMEM87A	0.51937432	1.1E-13
MTF1	RIC8A	0.51974582	1.05E-13
MTF1	PTK2	0.52001753	1.02E-13
MTF1	MSANTD3-TMEFF1	0.52010778	1E-13

MTF1	UBE2Q2	0.52012141	1E-13
MTF1	SNX27	0.52016618	9.97E-14
MTF1	LATS2	0.52025797	9.85E-14
MTF1	LASP1	0.5202613	9.85E-14
MTF1	KATNBL1	0.52030762	9.79E-14
MTF1	KDM5B	0.52033109	9.76E-14
MTF1	ARHGEF6	0.52039553	9.68E-14
MTF1	CYTH3	0.52073116	9.27E-14
MTF1	PHF23	0.52081869	9.17E-14
MTF1	ARPC4	0.52084636	9.14E-14
MTF1	SNX11	0.52120285	8.73E-14
MTF1	PLEKHA2	0.52125112	8.68E-14
MTF1	CCDC32	0.52133369	8.59E-14
MTF1	MMD	0.52136247	8.55E-14
MTF1	MB21D2	0.52140914	8.5E-14
MTF1	TRMT2B	0.52160203	8.3E-14
MTF1	NXT2	0.52161663	8.28E-14
MTF1	ADAM9	0.52172568	8.17E-14
MTF1	RPL17-C18orf32	0.5223291	7.56E-14
MTF1	RCBTB2	0.52251286	7.38E-14
MTF1	MAP4K4	0.52263051	7.27E-14
MTF1	RNF170	0.5226774	7.23E-14
MTF1	VPS26A	0.52269789	7.21E-14
MTF1	CDC42SE1	0.5228008	7.11E-14
MTF1	PPP1R9B	0.52289663	7.03E-14
MTF1	MAF	0.52310803	6.84E-14
MTF1	CXorf38	0.52322767	6.73E-14
MTF1	GPATCH2	0.52327421	6.69E-14
MTF1	SHROOM3	0.52333271	6.64E-14
MTF1	DDX23	0.52362912	6.4E-14
MTF1	SYT11	0.52380652	6.25E-14
MTF1	CYBRD1	0.52392117	6.16E-14
MTF1	DIAPH2	0.52394879	6.14E-14
MTF1	CALM3	0.52395286	6.13E-14
MTF1	SEMA4D	0.52399924	6.1E-14
MTF1	IRAK3	0.52401477	6.09E-14
MTF1	AASS	0.52419649	5.94E-14
MTF1	MBIP	0.52449279	5.72E-14
MTF1	HFE	0.52486154	5.45E-14
MTF1	PTPRE	0.52493898	5.4E-14
MTF1	KITLG	0.52497177	5.38E-14
MTF1	RHNO1	0.52505875	5.32E-14
MTF1	LAP3	0.5250949	5.29E-14
MTF1	ECM2	0.52523968	5.19E-14
MTF1	DENND1C	0.52537792	5.1E-14
MTF1	CIP2A	0.52539984	5.09E-14
MTF1	SMARCAL1	0.52561473	4.95E-14
MTF1	FRRS1	0.52563129	4.94E-14
MTF1	RTF2	0.52565097	4.92E-14
MTF1	KTN1	0.52567598	4.91E-14
MTF1	CORO1C	0.52568605	4.9E-14
MTF1	C14orf119	0.52569277	4.9E-14
MTF1	TRIM14	0.5257476	4.86E-14
MTF1	PLRG1	0.52583392	4.81E-14
MTF1	CYFIP1	0.5258527	4.8E-14
MTF1	IARS2	0.52588066	4.78E-14
MTF1	TAF9B	0.52601372	4.7E-14
MTF1	NFYA	0.52607366	4.66E-14

MTF1	SPDYE3	0.52626134	4.55E-14
MTF1	NIPA1	0.52628924	4.53E-14
MTF1	DENND1A	0.52629478	4.53E-14
MTF1	VTI1A	0.52630631	4.52E-14
MTF1	DOP1B	0.52669824	4.3E-14
MTF1	MAX	0.52677261	4.26E-14
MTF1	RBMS2	0.52699925	4.13E-14
MTF1	SPON1	0.52713613	4.06E-14
MTF1	PDGFRA	0.52718637	4.03E-14
MTF1	GPALPP1	0.52746175	3.89E-14
MTF1	SC5D	0.52760482	3.82E-14
MTF1	TADA1	0.52779494	3.73E-14
MTF1	ZMYND8	0.52815013	3.56E-14
MTF1	TPD52	0.52819417	3.54E-14
MTF1	BAG4	0.52827716	3.5E-14
MTF1	PCBP1	0.52828159	3.5E-14
MTF1	NOTCH2	0.52829019	3.49E-14
MTF1	SMG7	0.52836667	3.46E-14
MTF1	FOSL2	0.52845866	3.42E-14
MTF1	HIGD1A	0.52846938	3.41E-14
MTF1	PSMD7	0.52865054	3.33E-14
MTF1	KIAA0586	0.52885918	3.24E-14
MTF1	GNA11	0.52893756	3.21E-14
MTF1	TNFAIP3	0.52894817	3.2E-14
MTF1	ECD	0.52903727	3.17E-14
MTF1	CEP250	0.52912015	3.13E-14
MTF1	LSM14B	0.5292291	3.09E-14
MTF1	RNF8	0.52928126	3.07E-14
MTF1	ARF6	0.52954871	2.96E-14
MTF1	TSPAN9	0.52962526	2.93E-14
MTF1	PELO	0.52973897	2.89E-14
MTF1	COL4A1	0.52989919	2.83E-14
MTF1	LTBP1	0.52995331	2.81E-14
MTF1	MED18	0.53012785	2.74E-14
MTF1	ZNF532	0.5302794	2.69E-14
MTF1	CCDC90B	0.53029749	2.68E-14
MTF1	CNN3	0.53033895	2.67E-14
MTF1	PRPF18	0.53048527	2.62E-14
MTF1	GOLPH3L	0.53064379	2.56E-14
MTF1	GK	0.53102211	2.44E-14
MTF1	SLC9A7	0.53115806	2.4E-14
MTF1	CRISPLD1	0.53132608	2.34E-14
MTF1	SEMA5A	0.53133118	2.34E-14
MTF1	TFG	0.53142186	2.31E-14
MTF1	E2F3	0.53156486	2.27E-14
MTF1	TMEM140	0.53161045	2.26E-14
MTF1	PHF13	0.53161457	2.26E-14
MTF1	RAB27A	0.53176144	2.21E-14
MTF1	JDP2	0.53183539	2.19E-14
MTF1	LRRC42	0.53184023	2.19E-14
MTF1	PSAP	0.53192638	2.16E-14
MTF1	EHF	0.53196026	2.15E-14
MTF1	PLCG1	0.53206189	2.13E-14
MTF1	MOB1B	0.53214875	2.1E-14
MTF1	NUP107	0.53217267	2.09E-14
MTF1	PRKACB	0.53234202	2.05E-14
MTF1	GNAI2	0.53239678	2.03E-14
MTF1	RAPH1	0.53242062	2.03E-14

MTF1	LSAMP	0.53246458	2.02E-14
MTF1	CDKN2AIP	0.5324886	2.01E-14
MTF1	KIF3C	0.53250547	2E-14
MTF1	NIPSNAP3A	0.53252949	2E-14
MTF1	C1orf216	0.53256868	1.99E-14
MTF1	C12orf65	0.53262031	1.97E-14
MTF1	CEP170	0.53263394	1.97E-14
MTF1	TRIM26	0.5327079	1.95E-14
MTF1	COX7A2L	0.53285291	1.91E-14
MTF1	GNE	0.53295472	1.89E-14
MTF1	SLIT2	0.53297788	1.88E-14
MTF1	DYRK3	0.53301669	1.87E-14
MTF1	CDC73	0.53375916	1.7E-14
MTF1	OXNAD1	0.53391898	1.66E-14
MTF1	VCL	0.53401524	1.64E-14
MTF1	DOCK10	0.5340236	1.64E-14
MTF1	TCFL5	0.53409197	1.62E-14
MTF1	TFRC	0.53421187	1.6E-14
MTF1	KANK2	0.53470676	1.5E-14
MTF1	UTP25	0.53490606	1.46E-14
MTF1	JAM3	0.53493723	1.45E-14
MTF1	PGD	0.5349679	1.44E-14
MTF1	ZNF816	0.53500991	1.44E-14
MTF1	PRSS23	0.53506649	1.43E-14
MTF1	AP3S1	0.53506913	1.42E-14
MTF1	MAP1B	0.53507181	1.42E-14
MTF1	ZNF362	0.53516696	1.41E-14
MTF1	ZDHHC9	0.53526108	1.39E-14
MTF1	NAP1L1	0.53533711	1.37E-14
MTF1	YTHDF1	0.5353546	1.37E-14
MTF1	SUSD1	0.5354672	1.35E-14
MTF1	NUAK1	0.53578705	1.29E-14
MTF1	CENPL	0.53580257	1.29E-14
MTF1	CLINT1	0.53592905	1.27E-14
MTF1	MED4	0.53601615	1.25E-14
MTF1	ZCCHC10	0.53607267	1.25E-14
MTF1	ETV1	0.5362624	1.21E-14
MTF1	KLF3	0.53634106	1.2E-14
MTF1	STON1	0.53639142	1.19E-14
MTF1	MYO1D	0.53657908	1.16E-14
MTF1	ABLIM1	0.53664849	1.15E-14
MTF1	BECN1	0.53665999	1.15E-14
MTF1	CALD1	0.53675767	1.14E-14
MTF1	ZCRB1	0.53704979	1.09E-14
MTF1	FKBP7	0.53708721	1.09E-14
MTF1	PPP4R1	0.5371203	1.08E-14
MTF1	PIP4K2C	0.53743295	1.04E-14
MTF1	WBP1L	0.53747293	1.03E-14
MTF1	RNF26	0.53762542	1.01E-14
MTF1	ENTPD1	0.53777007	9.91E-15
MTF1	CHMP5	0.5378536	9.8E-15
MTF1	USP34	0.53801671	9.59E-15
MTF1	TP53BP2	0.5381621	9.4E-15
MTF1	TCEAL8	0.5381757	9.38E-15
MTF1	SYPL1	0.53830864	9.22E-15
MTF1	PSMF1	0.53850324	8.98E-15
MTF1	CTPS1	0.53850909	8.97E-15
MTF1	ATXN7L3	0.53861879	8.84E-15

MTF1	PTGS1	0.53871421	8.72E-15
MTF1	ZWILCH	0.53874145	8.69E-15
MTF1	CHIC2	0.53898411	8.41E-15
MTF1	STK17B	0.53902043	8.37E-15
MTF1	CBLB	0.53925743	8.1E-15
MTF1	DPY19L1	0.53928521	8.07E-15
MTF1	EXOC4	0.53932063	8.04E-15
MTF1	PXDN	0.53948584	7.86E-15
MTF1	SF3B3	0.53951659	7.82E-15
MTF1	ANO10	0.53976031	7.57E-15
MTF1	ANKMY2	0.53981636	7.51E-15
MTF1	TIPRL	0.53995582	7.37E-15
MTF1	SOWAHC	0.54018982	7.14E-15
MTF1	GTF2B	0.54020581	7.13E-15
MTF1	FBN1	0.54031978	7.02E-15
MTF1	AFG3L2	0.54051115	6.84E-15
MTF1	DZIP3	0.54071913	6.64E-15
MTF1	WIPI2	0.54083683	6.54E-15
MTF1	PARP8	0.5409108	6.47E-15
MTF1	ZNF286A	0.54096099	6.43E-15
MTF1	SDF4	0.54097895	6.41E-15
MTF1	SMAP2	0.54098021	6.41E-15
MTF1	CPT1A	0.54099816	6.4E-15
MTF1	ABI3BP	0.54110669	6.3E-15
MTF1	NCEH1	0.54113963	6.27E-15
MTF1	FUCA1	0.54124056	6.19E-15
MTF1	EXOC1	0.54125227	6.18E-15
MTF1	BCORL1	0.54143699	6.02E-15
MTF1	ZNF629	0.54149014	5.98E-15
MTF1	CELF2	0.54149956	5.97E-15
MTF1	NRBP1	0.54152053	5.96E-15
MTF1	FLT1	0.54157637	5.91E-15
MTF1	MRFAP1L1	0.54160458	5.89E-15
MTF1	CCAR2	0.54164737	5.85E-15
MTF1	ADGRA2	0.54173625	5.78E-15
MTF1	RHEB	0.54181014	5.72E-15
MTF1	CPED1	0.54220731	5.42E-15
MTF1	PLXDC2	0.5422614	5.38E-15
MTF1	MTA2	0.54243409	5.26E-15
MTF1	KTI12	0.54246559	5.23E-15
MTF1	TMEM200A	0.54249713	5.21E-15
MTF1	ATP11A	0.54259051	5.14E-15
MTF1	BTBD10	0.54276199	5.02E-15
MTF1	MAP3K20	0.54277259	5.02E-15
MTF1	GNAI1	0.54286553	4.95E-15
MTF1	MED6	0.54314587	4.77E-15
MTF1	OSBPL1A	0.5432231	4.72E-15
MTF1	CFAP20	0.54333674	4.64E-15
MTF1	HMGCR	0.54346285	4.56E-15
MTF1	ZC2HC1A	0.54353014	4.52E-15
MTF1	ZNF526	0.54356247	4.5E-15
MTF1	CDKL1	0.54376188	4.38E-15
MTF1	DCP1B	0.54382054	4.34E-15
MTF1	TDP2	0.54418469	4.13E-15
MTF1	DDX50	0.54427369	4.08E-15
MTF1	ERG	0.54432149	4.05E-15
MTF1	EMC2	0.544611	3.9E-15
MTF1	LIMS1	0.54481675	3.79E-15

MTF1	CSGALNACT1	0.54502165	3.68E-15
MTF1	SLFN5	0.54518109	3.6E-15
MTF1	SPIRE1	0.54548188	3.45E-15
MTF1	STK3	0.54556524	3.41E-15
MTF1	MICAL2	0.54578007	3.31E-15
MTF1	OTUD1	0.54579232	3.31E-15
MTF1	FGD4	0.54582713	3.29E-15
MTF1	CHMP2B	0.54583447	3.29E-15
MTF1	SH3KBP1	0.54584476	3.28E-15
MTF1	WIPF1	0.5458693	3.27E-15
MTF1	TMED10	0.54615839	3.14E-15
MTF1	MEF2C	0.54619221	3.13E-15
MTF1	PSIP1	0.54620877	3.12E-15
MTF1	LRP11	0.54629431	3.09E-15
MTF1	HNRNPC	0.54631495	3.08E-15
MTF1	U2AF2	0.54631646	3.08E-15
MTF1	CYCS	0.54635046	3.06E-15
MTF1	RRP15	0.54639452	3.04E-15
MTF1	NT5C2	0.54649004	3E-15
MTF1	PCGF5	0.54654718	2.98E-15
MTF1	BRI3BP	0.54674467	2.9E-15
MTF1	SMC4	0.54677247	2.89E-15
MTF1	ZEB2	0.54689792	2.84E-15
MTF1	SEC11A	0.54699053	2.8E-15
MTF1	CALCRL	0.54700719	2.8E-15
MTF1	CHMP1B	0.5470553	2.78E-15
MTF1	TENM3	0.54708061	2.77E-15
MTF1	PRICKLE1	0.54712108	2.75E-15
MTF1	NDFIP1	0.54726283	2.7E-15
MTF1	AKIRIN2	0.54773157	2.53E-15
MTF1	ARRDC3	0.54787582	2.48E-15
MTF1	MANBA	0.54811867	2.39E-15
MTF1	TMEM199	0.54818638	2.37E-15
MTF1	ZCCHC9	0.54841397	2.3E-15
MTF1	ANKRD42	0.54841506	2.3E-15
MTF1	ELK3	0.54845816	2.28E-15
MTF1	TMEM59	0.54846789	2.28E-15
MTF1	BLOC1S5	0.548512	2.27E-15
MTF1	PTPN12	0.54852911	2.26E-15
MTF1	PIP4K2A	0.5485897	2.24E-15
MTF1	CIR1	0.54866174	2.22E-15
MTF1	LIPA	0.54867819	2.21E-15
MTF1	MRPL3	0.5487572	2.19E-15
MTF1	TENT5A	0.54878733	2.18E-15
MTF1	PRDX3	0.54885273	2.16E-15
MTF1	DLGAP4	0.54895386	2.13E-15
MTF1	PPHLN1	0.54898262	2.12E-15
MTF1	TPP2	0.54899621	2.12E-15
MTF1	FAM204A	0.54905934	2.1E-15
MTF1	ANO6	0.54908531	2.09E-15
MTF1	PNPO	0.54912378	2.08E-15
MTF1	PARP14	0.54915094	2.07E-15
MTF1	KDM6B	0.54928522	2.03E-15
MTF1	CPPED1	0.54938568	2E-15
MTF1	REEP3	0.54962475	1.94E-15
MTF1	SORT1	0.54964401	1.93E-15
MTF1	ATL3	0.54968404	1.92E-15
MTF1	ADAT1	0.54969705	1.92E-15

MTF1	HIVEP3	0.54974576	1.91E-15
MTF1	DPYSL2	0.54984433	1.88E-15
MTF1	GAS7	0.54990943	1.86E-15
MTF1	CIAPIN1	0.54998524	1.84E-15
MTF1	SSPN	0.55001324	1.84E-15
MTF1	PLEKHM2	0.55001786	1.83E-15
MTF1	KATNA1	0.55002304	1.83E-15
MTF1	PSEN1	0.55019777	1.79E-15
MTF1	NUCKS1	0.55031162	1.76E-15
MTF1	DNAJC8	0.55058528	1.69E-15
MTF1	BPGM	0.55074585	1.66E-15
MTF1	FADS1	0.55089335	1.62E-15
MTF1	SUMO2	0.55095718	1.61E-15
MTF1	NCAPD2	0.55097052	1.6E-15
MTF1	TP53INP2	0.55110907	1.57E-15
MTF1	ADGRL2	0.5511974	1.55E-15
MTF1	CYB5R3	0.55120918	1.55E-15
MTF1	PATL1	0.55129422	1.53E-15
MTF1	FZD6	0.55130558	1.53E-15
MTF1	SCO1	0.55133379	1.52E-15
MTF1	C4orf3	0.55143363	1.5E-15
MTF1	YWHAE	0.5518056	1.43E-15
MTF1	ZNF267	0.5521356	1.36E-15
MTF1	ARSJ	0.55216976	1.35E-15
MTF1	TMEM230	0.55225937	1.34E-15
MTF1	LBH	0.55235548	1.32E-15
MTF1	C12orf43	0.55246709	1.3E-15
MTF1	ABCF1	0.55256513	1.28E-15
MTF1	INTS5	0.55266502	1.26E-15
MTF1	TSPAN2	0.55268113	1.26E-15
MTF1	HMCN1	0.55271127	1.25E-15
MTF1	UBXN2A	0.55276724	1.24E-15
MTF1	RIT1	0.55281455	1.24E-15
MTF1	NAAA	0.55292725	1.22E-15
MTF1	NEK7	0.55298546	1.21E-15
MTF1	ENOPH1	0.55299321	1.21E-15
MTF1	PRKAG1	0.55303114	1.2E-15
MTF1	POGK	0.553082	1.19E-15
MTF1	ADO	0.55316402	1.18E-15
MTF1	RASA1	0.55343869	1.13E-15
MTF1	NKIRAS2	0.55345785	1.13E-15
MTF1	DSE	0.55351923	1.12E-15
MTF1	BTN2A1	0.55355604	1.11E-15
MTF1	SELENOP	0.55367986	1.09E-15
MTF1	SESN3	0.55368066	1.09E-15
MTF1	LACTB	0.55368812	1.09E-15
MTF1	MAP3K13	0.55376333	1.08E-15
MTF1	KIT	0.55400576	1.04E-15
MTF1	NDFIP2	0.55407878	1.03E-15
MTF1	SLC24A1	0.55407997	1.03E-15
MTF1	GATAD2A	0.554085	1.03E-15
MTF1	ANKRD11	0.55449868	9.73E-16
MTF1	RHOBTB2	0.55456046	9.64E-16
MTF1	UGCG	0.55474828	9.39E-16
MTF1	ERGIC2	0.55477847	9.35E-16
MTF1	DNAJC18	0.55479943	9.32E-16
MTF1	GLO1	0.55489352	9.19E-16
MTF1	EHD4	0.55492827	9.15E-16

MTF1	CDH11	0.55492835	9.15E-16
MTF1	MED21	0.55508988	8.94E-16
MTF1	RMND5A	0.55509192	8.94E-16
MTF1	ERI1	0.55512905	8.89E-16
MTF1	VOPP1	0.555253	8.73E-16
MTF1	ATG9A	0.55525449	8.73E-16
MTF1	L3MBTL2	0.55527306	8.71E-16
MTF1	RAI14	0.55543559	8.51E-16
MTF1	SPCS2	0.55575979	8.12E-16
MTF1	UTP3	0.55587791	7.99E-16
MTF1	ITM2B	0.55589877	7.96E-16
MTF1	OCIAD1	0.55592722	7.93E-16
MTF1	TMEM168	0.55605439	7.79E-16
MTF1	PDE4B	0.55620812	7.62E-16
MTF1	TMEM184B	0.55623358	7.59E-16
MTF1	RFK	0.55624878	7.57E-16
MTF1	TRPS1	0.55670507	7.09E-16
MTF1	SMAD7	0.55690715	6.89E-16
MTF1	HSPE1-MOB4	0.55697942	6.82E-16
MTF1	ADIPOR2	0.55698677	6.81E-16
MTF1	ZFAND6	0.55702886	6.77E-16
MTF1	ARL15	0.55705759	6.74E-16
MTF1	MINPP1	0.55707213	6.73E-16
MTF1	CLN5	0.55712367	6.68E-16
MTF1	CCDC97	0.55723403	6.57E-16
MTF1	HNRNPA0	0.55725326	6.55E-16
MTF1	ERGIC1	0.55744731	6.37E-16
MTF1	USP6NL	0.5575694	6.26E-16
MTF1	PIK3C3	0.55763222	6.21E-16
MTF1	ATP6V1G1	0.55773489	6.12E-16
MTF1	AKAP12	0.55778247	6.07E-16
MTF1	LXN	0.55779513	6.06E-16
MTF1	RNF34	0.5578019	6.06E-16
MTF1	POLDIP3	0.55780882	6.05E-16
MTF1	PTPRF	0.55788127	5.99E-16
MTF1	TRAF7	0.55795432	5.92E-16
MTF1	PLS3	0.55810006	5.8E-16
MTF1	PCMT1	0.5582671	5.66E-16
MTF1	PRELID3B	0.55833069	5.61E-16
MTF1	PTGES3	0.55846634	5.5E-16
MTF1	TXNDC15	0.55863224	5.37E-16
MTF1	DAG1	0.55863434	5.37E-16
MTF1	KIRREL1	0.55885559	5.2E-16
MTF1	GALK2	0.55892176	5.15E-16
MTF1	MRPL42	0.55901958	5.08E-16
MTF1	HNRNPF	0.55907953	5.03E-16
MTF1	HACD3	0.55909796	5.02E-16
MTF1	NLN	0.55919228	4.95E-16
MTF1	CUL4A	0.55925603	4.91E-16
MTF1	RCN2	0.55956547	4.69E-16
MTF1	FOXO1	0.55979595	4.54E-16
MTF1	HEATR5A	0.55982333	4.52E-16
MTF1	PTP4A1	0.55986807	4.49E-16
MTF1	NKIRAS1	0.56001724	4.39E-16
MTF1	NDRG3	0.5600622	4.37E-16
MTF1	PIK3CG	0.56006844	4.36E-16
MTF1	FAM210A	0.56031966	4.21E-16
MTF1	SDC3	0.56034718	4.19E-16

MTF1	RALBP1	0.56037749	4.17E-16
MTF1	C16orf87	0.56061266	4.03E-16
MTF1	CHMP3	0.56066508	4E-16
MTF1	MOSPD1	0.56075198	3.95E-16
MTF1	ITGA9	0.56078191	3.93E-16
MTF1	TMEM38B	0.560899	3.87E-16
MTF1	MFHAS1	0.56095425	3.83E-16
MTF1	MAP4	0.56104465	3.78E-16
MTF1	ETNK1	0.56109314	3.76E-16
MTF1	DLC1	0.56122614	3.69E-16
MTF1	TRIM37	0.56131702	3.64E-16
MTF1	AMPD3	0.56149249	3.54E-16
MTF1	BTG1	0.56152571	3.53E-16
MTF1	SRP9	0.5621025	3.24E-16
MTF1	PTPRK	0.56214474	3.22E-16
MTF1	TM2D3	0.56215857	3.22E-16
MTF1	COPG2	0.56224024	3.18E-16
MTF1	HIBADH	0.56231797	3.14E-16
MTF1	CAMSAP2	0.56236485	3.12E-16
MTF1	CROT	0.56243006	3.09E-16
MTF1	EAPP	0.56263973	3E-16
MTF1	DCHS1	0.56275145	2.95E-16
MTF1	TAF1A	0.5630825	2.81E-16
MTF1	VCP	0.56349258	2.64E-16
MTF1	IWS1	0.56355385	2.62E-16
MTF1	PIAS1	0.56356504	2.62E-16
MTF1	FNTB	0.5636363	2.59E-16
MTF1	MYO6	0.56376667	2.54E-16
MTF1	LMBRD1	0.56381335	2.52E-16
MTF1	HTATSF1	0.56396415	2.47E-16
MTF1	CDON	0.5640702	2.43E-16
MTF1	CISD2	0.56416776	2.39E-16
MTF1	PIGW	0.5644857	2.28E-16
MTF1	CHRAC1	0.56454237	2.27E-16
MTF1	PANX1	0.56467549	2.22E-16
MTF1	METTL4	0.56468653	2.22E-16
MTF1	UBAP1	0.56510285	2.09E-16
MTF1	LYPLA1	0.56512415	2.08E-16
MTF1	ATP11C	0.56536352	2.01E-16
MTF1	PER2	0.56544363	1.98E-16
MTF1	LEPR	0.56565414	1.92E-16
MTF1	UBE2V2	0.56567755	1.92E-16
MTF1	PEX3	0.56576173	1.89E-16
MTF1	ZNF28	0.56584989	1.87E-16
MTF1	NIP7	0.56596243	1.84E-16
MTF1	TBC1D25	0.56602564	1.82E-16
MTF1	ST3GAL2	0.56603691	1.82E-16
MTF1	NEURL1B	0.56638389	1.72E-16
MTF1	RAE1	0.5664846	1.7E-16
MTF1	AFAP1	0.56659079	1.67E-16
MTF1	ARHGAP1	0.56681384	1.62E-16
MTF1	TMEM185B	0.56687682	1.6E-16
MTF1	TMCC3	0.56692682	1.59E-16
MTF1	OPA3	0.56695002	1.59E-16
MTF1	FAM118B	0.56698663	1.58E-16
MTF1	MRPL49	0.56725077	1.52E-16
MTF1	SERTAD2	0.56742887	1.48E-16
MTF1	GNB4	0.56747855	1.47E-16

MTF1	OXSR1	0.56748317	1.46E-16
MTF1	HDGFL3	0.56757221	1.45E-16
MTF1	ATAD1	0.56777582	1.4E-16
MTF1	BBS9	0.56783988	1.39E-16
MTF1	ARHGAP21	0.56786572	1.38E-16
MTF1	LYST	0.56789698	1.38E-16
MTF1	YWHAH	0.56792695	1.37E-16
MTF1	EIF2S1	0.56793769	1.37E-16
MTF1	DYNLT3	0.56812984	1.33E-16
MTF1	CASK	0.56817521	1.32E-16
MTF1	PSMD2	0.56819959	1.32E-16
MTF1	PRPS1	0.56823011	1.31E-16
MTF1	RNPS1	0.56835105	1.29E-16
MTF1	TMEM35B	0.56836608	1.28E-16
MTF1	KSR1	0.56844591	1.27E-16
MTF1	GLTP	0.56860182	1.24E-16
MTF1	GHITM	0.56864507	1.23E-16
MTF1	SRF	0.56878406	1.21E-16
MTF1	CPD	0.56888267	1.19E-16
MTF1	YPEL5	0.5690054	1.17E-16
MTF1	METTL2B	0.56921695	1.13E-16
MTF1	LCLAT1	0.56930435	1.12E-16
MTF1	PDHB	0.56931723	1.11E-16
MTF1	DACT1	0.56954996	1.08E-16
MTF1	SSB	0.56988149	1.02E-16
MTF1	SBDS	0.56996051	1.01E-16
MTF1	BICD2	0.57001917	1E-16
MTF1	PDE4DIP	0.57002938	1E-16
MTF1	NCL	0.5700891	9.92E-17
MTF1	MSI2	0.57025725	9.67E-17
MTF1	NPLOC4	0.57031217	9.59E-17
MTF1	XIAP	0.57047764	9.35E-17
MTF1	VEZT	0.57052001	9.29E-17
MTF1	NOLC1	0.57060551	9.17E-17
MTF1	FSTL1	0.5707264	9.01E-17
MTF1	SLC17A5	0.57102595	8.61E-17
MTF1	LRIF1	0.57107396	8.55E-17
MTF1	AHR	0.57112082	8.49E-17
MTF1	TMEM43	0.5712552	8.32E-17
MTF1	DDR2	0.57181557	7.64E-17
MTF1	RDH11	0.57184121	7.61E-17
MTF1	FNBP1	0.57216363	7.25E-17
MTF1	NAPG	0.57217316	7.24E-17
MTF1	IPO11	0.57248378	6.91E-17
MTF1	TSC22D2	0.57265342	6.73E-17
MTF1	RPF1	0.57283018	6.55E-17
MTF1	VIPAS39	0.57307525	6.32E-17
MTF1	ATXN7L3B	0.57308234	6.31E-17
MTF1	TMX4	0.57320961	6.19E-17
MTF1	LARP4B	0.57323525	6.16E-17
MTF1	ARL6IP5	0.57328957	6.11E-17
MTF1	CPM	0.57335384	6.05E-17
MTF1	FASTKD2	0.57340638	6.01E-17
MTF1	ARCN1	0.57343068	5.98E-17
MTF1	SNX3	0.57343264	5.98E-17
MTF1	BTN3A1	0.57348204	5.94E-17
MTF1	ZHX2	0.57348556	5.93E-17
MTF1	MYH9	0.57350396	5.92E-17

MTF1	PRPF38A	0.57350926	5.91E-17
MTF1	FIG4	0.57358282	5.85E-17
MTF1	COQ7	0.57364686	5.79E-17
MTF1	GNB5	0.57392933	5.55E-17
MTF1	ZNRF2	0.5740257	5.46E-17
MTF1	FRMD4B	0.57403356	5.46E-17
MTF1	SEH1L	0.57405268	5.44E-17
MTF1	NDE1	0.57410512	5.4E-17
MTF1	DNAJC13	0.57422967	5.3E-17
MTF1	ELK4	0.57436547	5.19E-17
MTF1	ABCB10	0.57459273	5.01E-17
MTF1	CDS2	0.57483767	4.83E-17
MTF1	GRAMD2B	0.5749889	4.72E-17
MTF1	RPE	0.57499294	4.71E-17
MTF1	URI1	0.57500638	4.71E-17
MTF1	CAMKK2	0.57512139	4.62E-17
MTF1	NIN	0.57534554	4.47E-17
MTF1	KIF3A	0.57544748	4.4E-17
MTF1	PDCD10	0.57553324	4.34E-17
MTF1	STPG1	0.57567198	4.25E-17
MTF1	PLEKHA1	0.57574483	4.2E-17
MTF1	WDR43	0.57574934	4.2E-17
MTF1	CYREN	0.57606504	4E-17
MTF1	GASK1B	0.57622376	3.9E-17
MTF1	DCTN5	0.57635838	3.82E-17
MTF1	CD93	0.57652498	3.73E-17
MTF1	SMARCA2	0.57656308	3.71E-17
MTF1	SLC4A1AP	0.57671922	3.62E-17
MTF1	FAM200A	0.57701507	3.46E-17
MTF1	SLC38A7	0.57709885	3.41E-17
MTF1	SKIL	0.57711883	3.4E-17
MTF1	MAN2B2	0.57712355	3.4E-17
MTF1	IQGAP1	0.5772396	3.34E-17
MTF1	TPST1	0.57726507	3.33E-17
MTF1	PDE4D	0.57738043	3.27E-17
MTF1	APMAP	0.5773836	3.27E-17
MTF1	HDAC2	0.57743594	3.24E-17
MTF1	LRCH1	0.57761488	3.15E-17
MTF1	CLP1	0.57766487	3.13E-17
MTF1	NUP155	0.57772319	3.1E-17
MTF1	YBX1	0.57778035	3.07E-17
MTF1	DBF4	0.57782361	3.05E-17
MTF1	VANGL1	0.57797979	2.98E-17
MTF1	B3GALNT1	0.57805114	2.95E-17
MTF1	ACER3	0.57807298	2.94E-17
MTF1	TMEM68	0.57809883	2.93E-17
MTF1	HMGXB3	0.57815403	2.9E-17
MTF1	LHFPL2	0.57830126	2.84E-17
MTF1	TAF12	0.57834717	2.82E-17
MTF1	CHCHD3	0.57837544	2.8E-17
MTF1	PGAM5	0.5784164	2.79E-17
MTF1	DOCK1	0.57850061	2.75E-17
MTF1	NFIX	0.57852367	2.74E-17
MTF1	SOAT1	0.57859814	2.71E-17
MTF1	OSBPL10	0.57864236	2.69E-17
MTF1	KLHL29	0.57866764	2.68E-17
MTF1	SELENON	0.57868343	2.67E-17
MTF1	LRRC8C	0.57878762	2.63E-17

MTF1	ZNF416	0.57885617	2.6E-17
MTF1	POLE3	0.57908194	2.51E-17
MTF1	PEF1	0.57909021	2.51E-17
MTF1	PHC2	0.57927262	2.44E-17
MTF1	EPB41L3	0.57931389	2.42E-17
MTF1	ATPSCKMT	0.57940575	2.39E-17
MTF1	SIPA1L3	0.57948553	2.36E-17
MTF1	PAFAH1B2	0.57958018	2.33E-17
MTF1	SMIM15	0.57964843	2.3E-17
MTF1	HSP90AA1	0.58009645	2.15E-17
MTF1	ANKRD27	0.58045072	2.03E-17
MTF1	ELF4	0.5805421	2E-17
MTF1	LIMCH1	0.58074631	1.94E-17
MTF1	EHD3	0.58107389	1.84E-17
MTF1	TLK2	0.58113204	1.83E-17
MTF1	JAZF1	0.58144254	1.74E-17
MTF1	SPRY1	0.58152796	1.72E-17
MTF1	MAP1LC3B	0.58171864	1.67E-17
MTF1	SKAP2	0.58189602	1.62E-17
MTF1	GRWD1	0.58215452	1.56E-17
MTF1	METAP1	0.58232289	1.52E-17
MTF1	MAP1A	0.5823998	1.5E-17
MTF1	ITGA1	0.58241909	1.49E-17
MTF1	SNW1	0.58246803	1.48E-17
MTF1	NSMCE3	0.58254803	1.46E-17
MTF1	TM9SF4	0.582761	1.42E-17
MTF1	CMPK1	0.582801	1.41E-17
MTF1	RNF121	0.58280685	1.41E-17
MTF1	UGP2	0.58295824	1.37E-17
MTF1	ATP6V1B2	0.58300036	1.36E-17
MTF1	OXCT1	0.5830385	1.36E-17
MTF1	RPGRIP1L	0.58318068	1.33E-17
MTF1	RBM43	0.58344146	1.27E-17
MTF1	PLEKHF2	0.58352566	1.26E-17
MTF1	LDB1	0.58364823	1.23E-17
MTF1	LARGE1	0.58390523	1.18E-17
MTF1	BBS12	0.58404101	1.16E-17
MTF1	ZBTB4	0.58405207	1.16E-17
MTF1	PACRGL	0.58405215	1.16E-17
MTF1	RNF24	0.58413062	1.14E-17
MTF1	ADAM22	0.58413379	1.14E-17
MTF1	CASP2	0.5841491	1.14E-17
MTF1	UXS1	0.58415874	1.14E-17
MTF1	OAT	0.58418657	1.13E-17
MTF1	F2R	0.58460655	1.06E-17
MTF1	PDK3	0.58466526	1.05E-17
MTF1	EIF1B	0.58473854	1.04E-17
MTF1	ABHD6	0.5849214	1.01E-17
MTF1	SLC25A13	0.58530417	9.48E-18
MTF1	ACBD5	0.58533402	9.43E-18
MTF1	PIP4K2B	0.5855483	9.12E-18
MTF1	COMMD8	0.58576976	8.8E-18
MTF1	DCP2	0.58580015	8.76E-18
MTF1	GLOD4	0.58582213	8.73E-18
MTF1	TMTC2	0.58583664	8.71E-18
MTF1	LEO1	0.58593031	8.58E-18
MTF1	ARL6IP6	0.58607184	8.39E-18
MTF1	BBS7	0.58618554	8.24E-18

MTF1	LRRFIP2	0.58627005	8.13E-18
MTF1	RAB23	0.58635119	8.03E-18
MTF1	SH3BP5	0.58642941	7.93E-18
MTF1	SNN	0.58653773	7.79E-18
MTF1	SUFU	0.5865669	7.76E-18
MTF1	REST	0.58677555	7.5E-18
MTF1	INCENP	0.58686953	7.39E-18
MTF1	AP1S2	0.58690151	7.36E-18
MTF1	NR3C1	0.58725635	6.95E-18
MTF1	NDC1	0.58729107	6.91E-18
MTF1	SIAH1	0.58736724	6.83E-18
MTF1	ZNF462	0.58745892	6.73E-18
MTF1	DPF2	0.58750862	6.68E-18
MTF1	NT5DC1	0.58757328	6.61E-18
MTF1	ITGA4	0.58765034	6.53E-18
MTF1	RASSF8	0.58793731	6.24E-18
MTF1	FBXO34	0.58796482	6.21E-18
MTF1	MAML2	0.58798656	6.19E-18
MTF1	OTUD7B	0.58802029	6.15E-18
MTF1	DLST	0.58820905	5.97E-18
MTF1	UHMK1	0.58832508	5.86E-18
MTF1	AP2B1	0.58872073	5.5E-18
MTF1	OSTM1	0.58876216	5.46E-18
MTF1	TRIO	0.58902033	5.24E-18
MTF1	PHTF2	0.58920772	5.09E-18
MTF1	ABL2	0.5893948	4.94E-18
MTF1	SRGAP2C	0.5894043	4.93E-18
MTF1	FBXL14	0.58961031	4.77E-18
MTF1	RGL1	0.58968444	4.71E-18
MTF1	PPP2R2A	0.58987613	4.57E-18
MTF1	AP1B1	0.58987793	4.57E-18
MTF1	COQ10B	0.58990117	4.55E-18
MTF1	LINS1	0.59026949	4.29E-18
MTF1	ZBTB9	0.59027606	4.28E-18
MTF1	AKT1	0.59029703	4.27E-18
MTF1	SCRN1	0.59030925	4.26E-18
MTF1	TTC8	0.59034558	4.24E-18
MTF1	FEM1C	0.5903838	4.21E-18
MTF1	GDI2	0.59048592	4.14E-18
MTF1	TOE1	0.59076839	3.96E-18
MTF1	SEC14L1	0.59098607	3.82E-18
MTF1	L3MBTL3	0.59101726	3.8E-18
MTF1	LAMTOR3	0.59103082	3.79E-18
MTF1	RTN4	0.59112914	3.73E-18
MTF1	SPRED1	0.59125605	3.66E-18
MTF1	JOSD1	0.59158691	3.47E-18
MTF1	SLC44A1	0.59223465	3.12E-18
MTF1	MPZL1	0.59225598	3.11E-18
MTF1	DTX3L	0.59235476	3.06E-18
MTF1	MAT2B	0.59244181	3.02E-18
MTF1	LAPTM4A	0.5929145	2.8E-18
MTF1	TWF1	0.59294653	2.78E-18
MTF1	UBE2H	0.59305953	2.73E-18
MTF1	SDHD	0.59321937	2.66E-18
MTF1	BTN3A3	0.59336347	2.6E-18
MTF1	VBP1	0.59336587	2.6E-18
MTF1	AHNAK	0.59341048	2.58E-18
MTF1	LRP10	0.59342033	2.57E-18

MTF1	ANAPC10	0.59344731	2.56E-18
MTF1	CLDND1	0.59353807	2.53E-18
MTF1	GALNT1	0.59360323	2.5E-18
MTF1	TBL1X	0.59368248	2.47E-18
MTF1	TEFM	0.5937119	2.46E-18
MTF1	JAK2	0.59379217	2.42E-18
MTF1	DCTD	0.59385636	2.4E-18
MTF1	NPM1	0.59391367	2.38E-18
MTF1	ZMAT2	0.59392175	2.37E-18
MTF1	ZC3HAV1	0.59399205	2.35E-18
MTF1	MRPL19	0.59411744	2.3E-18
MTF1	ARF4	0.59414779	2.29E-18
MTF1	BMS1	0.59445179	2.18E-18
MTF1	WWC3	0.59449694	2.16E-18
MTF1	SMG6	0.5945396	2.14E-18
MTF1	TANC2	0.59471801	2.08E-18
MTF1	SMIM12	0.59511159	1.95E-18
MTF1	CACUL1	0.59523185	1.92E-18
MTF1	MOB4	0.59548305	1.84E-18
MTF1	CAPZB	0.59569882	1.77E-18
MTF1	TMED8	0.5957338	1.76E-18
MTF1	LYN	0.59577687	1.75E-18
MTF1	UBE2A	0.59583743	1.73E-18
MTF1	RAD18	0.59586624	1.73E-18
MTF1	PCDH18	0.59601297	1.68E-18
MTF1	SPDL1	0.5960968	1.66E-18
MTF1	LACC1	0.59626979	1.62E-18
MTF1	MFSD6	0.596362	1.59E-18
MTF1	RIN2	0.59636969	1.59E-18
MTF1	STARD7	0.59641412	1.58E-18
MTF1	RNASEH1	0.5964991	1.56E-18
MTF1	LDLRAD4	0.59659761	1.53E-18
MTF1	WASHC2A	0.59664823	1.52E-18
MTF1	ELAVL1	0.59684548	1.47E-18
MTF1	DUSP18	0.59686234	1.46E-18
MTF1	SAR1A	0.59694728	1.44E-18
MTF1	KLHL18	0.5970081	1.43E-18
MTF1	SLC39A9	0.59703893	1.42E-18
MTF1	NUDCD1	0.59713257	1.4E-18
MTF1	MAST4	0.59723427	1.38E-18
MTF1	ERLIN1	0.59761179	1.29E-18
MTF1	PWP1	0.59772045	1.27E-18
MTF1	CAPZA1	0.59773335	1.27E-18
MTF1	FXR1	0.59786621	1.24E-18
MTF1	SLC8A1	0.59799819	1.21E-18
MTF1	MYCBP	0.59825763	1.16E-18
MTF1	ATL2	0.59860982	1.1E-18
MTF1	PRKDC	0.59865312	1.09E-18
MTF1	EIF1AD	0.5988393	1.06E-18
MTF1	ZNF131	0.59887867	1.05E-18
MTF1	HSPA14	0.59889527	1.05E-18
MTF1	MPP5	0.59894508	1.04E-18
MTF1	COPB1	0.59899275	1.03E-18
MTF1	PPP2R5D	0.59899772	1.03E-18
MTF1	ACVR2A	0.59908537	1.01E-18
MTF1	ANKH	0.59944429	9.55E-19
MTF1	ABHD2	0.59957345	9.35E-19
MTF1	ZNF174	0.59980801	8.99E-19

MTF1	DHX35	0.59992721	8.82E-19
MTF1	DENR	0.5999722	8.75E-19
MTF1	RTCB	0.60002523	8.67E-19
MTF1	KRAS	0.60002994	8.67E-19
MTF1	POT1	0.60003947	8.65E-19
MTF1	CACNA2D1	0.60045415	8.07E-19
MTF1	TOP2B	0.60045781	8.07E-19
MTF1	RPTOR	0.60049014	8.03E-19
MTF1	NAB1	0.6009153	7.48E-19
MTF1	TRAPPC6B	0.60096636	7.41E-19
MTF1	TFDP1	0.60105567	7.3E-19
MTF1	PPP1R15B	0.60119172	7.14E-19
MTF1	TWSG1	0.60121229	7.11E-19
MTF1	SYAP1	0.60126874	7.05E-19
MTF1	ITGB1	0.60128978	7.02E-19
MTF1	INTS14	0.60144727	6.84E-19
MTF1	LAMC1	0.60156616	6.7E-19
MTF1	ASAH1	0.60168244	6.58E-19
MTF1	SYNJ2BP	0.60172548	6.53E-19
MTF1	PTP4A2	0.60180815	6.44E-19
MTF1	AGAP1	0.60182709	6.42E-19
MTF1	PRUNE1	0.60185611	6.39E-19
MTF1	TOR1B	0.60190757	6.33E-19
MTF1	TMBIM6	0.6019134	6.33E-19
MTF1	ZMAT3	0.60196903	6.27E-19
MTF1	WBP4	0.60200037	6.23E-19
MTF1	TDRD7	0.60209493	6.14E-19
MTF1	ETV3	0.60223318	6E-19
MTF1	C1D	0.60224948	5.98E-19
MTF1	PPTC7	0.60240628	5.82E-19
MTF1	SFXN1	0.60243273	5.8E-19
MTF1	CWF19L2	0.60258612	5.65E-19
MTF1	SLC6A6	0.6027308	5.51E-19
MTF1	TMEM167A	0.60299334	5.28E-19
MTF1	TANK	0.60299951	5.27E-19
MTF1	TGFBR2	0.60315579	5.13E-19
MTF1	ZC3H11A	0.60336229	4.96E-19
MTF1	RNGTT	0.60337991	4.94E-19
MTF1	NAV1	0.60345028	4.88E-19
MTF1	ZNF217	0.60353855	4.81E-19
MTF1	MUL1	0.60354267	4.81E-19
MTF1	LRRFIP1	0.60355538	4.8E-19
MTF1	PLXNC1	0.60358598	4.77E-19
MTF1	AKTIP	0.60364839	4.72E-19
MTF1	UBE2Z	0.60380877	4.6E-19
MTF1	EXT1	0.6038934	4.53E-19
MTF1	PCNX4	0.60406818	4.4E-19
MTF1	SLC35A3	0.60423006	4.28E-19
MTF1	KIF13A	0.6043931	4.17E-19
MTF1	DYNC1I2	0.60446062	4.12E-19
MTF1	ANKRD50	0.60446803	4.11E-19
MTF1	PRXL2C	0.6046514	3.99E-19
MTF1	MYH10	0.60469139	3.96E-19
MTF1	ADGRF5	0.60480227	3.89E-19
MTF1	HIF1A	0.6048436	3.86E-19
MTF1	FKBP9	0.60488143	3.83E-19
MTF1	ASNSD1	0.60490324	3.82E-19
MTF1	RPA2	0.60492968	3.8E-19

MTF1	B4GALT5	0.60496205	3.78E-19
MTF1	ITSN1	0.60512594	3.68E-19
MTF1	NEDD9	0.60515336	3.66E-19
MTF1	TMEM237	0.60515514	3.66E-19
MTF1	ATG3	0.60529002	3.58E-19
MTF1	TDP1	0.60539014	3.52E-19
MTF1	SLC25A17	0.60590715	3.22E-19
MTF1	BRCC3	0.60598654	3.18E-19
MTF1	NUP98	0.60599846	3.17E-19
MTF1	TSHZ3	0.60667321	2.83E-19
MTF1	B3GNT2	0.606708	2.81E-19
MTF1	CLIC4	0.60678574	2.77E-19
MTF1	KCTD21	0.6068266	2.75E-19
MTF1	TOR1A	0.60683083	2.75E-19
MTF1	PIK3R3	0.60684472	2.75E-19
MTF1	LPP	0.60684874	2.74E-19
MTF1	AEBP2	0.60688524	2.73E-19
MTF1	PSMD12	0.60688848	2.73E-19
MTF1	INO80	0.6069693	2.69E-19
MTF1	MESD	0.60719222	2.59E-19
MTF1	RHOA	0.60721045	2.58E-19
MTF1	NIPA2	0.6072153	2.58E-19
MTF1	EHBP1	0.60723953	2.57E-19
MTF1	CDC42BPB	0.6073946	2.5E-19
MTF1	WDR1	0.60742907	2.49E-19
MTF1	PECAM1	0.60746455	2.47E-19
MTF1	AC010132.3	0.60752113	2.45E-19
MTF1	ZNF207	0.60758557	2.42E-19
MTF1	HCCS	0.60786596	2.31E-19
MTF1	PIGS	0.60790639	2.29E-19
MTF1	CASP3	0.60823754	2.16E-19
MTF1	GRB2	0.60853663	2.06E-19
MTF1	RASGEF1B	0.60855353	2.05E-19
MTF1	EIF1AX	0.60869996	2E-19
MTF1	RAB5C	0.60871616	1.99E-19
MTF1	KCTD9	0.60873402	1.99E-19
MTF1	RFFL	0.60891577	1.93E-19
MTF1	ATP5PB	0.60915996	1.85E-19
MTF1	NEDD4	0.60916493	1.85E-19
MTF1	CBX1	0.6092219	1.83E-19
MTF1	NUMB	0.6094466	1.76E-19
MTF1	EIF2AK2	0.60958003	1.72E-19
MTF1	IFNGR1	0.60970594	1.68E-19
MTF1	TMEM135	0.60970787	1.68E-19
MTF1	SLC30A5	0.61000692	1.6E-19
MTF1	DIPK2A	0.61003626	1.59E-19
MTF1	ZNF134	0.61012202	1.56E-19
MTF1	JADE2	0.61014476	1.56E-19
MTF1	ALDH9A1	0.61016337	1.55E-19
MTF1	FAM114A1	0.61029909	1.52E-19
MTF1	FBXO28	0.61056124	1.45E-19
MTF1	ITPR1	0.61063141	1.43E-19
MTF1	PPP2CB	0.61071517	1.41E-19
MTF1	MTFMT	0.61087182	1.37E-19
MTF1	MFSD11	0.61104757	1.33E-19
MTF1	ABCC1	0.6110652	1.33E-19
MTF1	GPBP1L1	0.61110411	1.32E-19
MTF1	HNRNPA2B1	0.6112367	1.29E-19

MTF1	SBF2	0.61155588	1.22E-19
MTF1	ATP11B	0.61156772	1.22E-19
MTF1	SKP1	0.61169878	1.19E-19
MTF1	AGFG1	0.61182196	1.17E-19
MTF1	MCC	0.61186727	1.16E-19
MTF1	H6PD	0.61187778	1.15E-19
MTF1	SERINC5	0.61205867	1.12E-19
MTF1	PITPNB	0.6122409	1.08E-19
MTF1	UBLCP1	0.61231534	1.07E-19
MTF1	PI4K2B	0.61233149	1.07E-19
MTF1	OXR1	0.61237151	1.06E-19
MTF1	TUBA1B	0.61255072	1.03E-19
MTF1	ROBO1	0.61257675	1.02E-19
MTF1	DESI2	0.6126079	1.02E-19
MTF1	TMTC1	0.61270557	1E-19
MTF1	GPN3	0.61277637	9.87E-20
MTF1	FCHO2	0.61277835	9.87E-20
MTF1	CHORDC1	0.61285348	9.74E-20
MTF1	KIF16B	0.61294707	9.58E-20
MTF1	DPYD	0.61297582	9.54E-20
MTF1	MARK2	0.61352095	8.67E-20
MTF1	AIDA	0.61390461	8.11E-20
MTF1	SCFD2	0.61412796	7.8E-20
MTF1	EMC3	0.6142173	7.68E-20
MTF1	KLHL7	0.61446556	7.35E-20
MTF1	FAM220A	0.61459755	7.18E-20
MTF1	PTEN	0.61460997	7.17E-20
MTF1	TMEM267	0.614828	6.9E-20
MTF1	NR1D2	0.61510467	6.57E-20
MTF1	EFL1	0.61524641	6.41E-20
MTF1	HIP1	0.61528603	6.36E-20
MTF1	SLMAP	0.61531619	6.33E-20
MTF1	UPRT	0.61541283	6.22E-20
MTF1	ATP5MF-PTCD1	0.6154818	6.15E-20
MTF1	GDE1	0.61551778	6.11E-20
MTF1	CNOT2	0.615526	6.1E-20
MTF1	ARHGAP42	0.61552636	6.1E-20
MTF1	PAFAH1B1	0.61555709	6.07E-20
MTF1	CALU	0.61560437	6.02E-20
MTF1	TMEM106B	0.61633874	5.29E-20
MTF1	CTNNB1	0.61655058	5.09E-20
MTF1	TFE3	0.61664164	5.01E-20
MTF1	TTC5	0.6167759	4.89E-20
MTF1	SLC35B4	0.61677876	4.89E-20
MTF1	CHSY1	0.61680202	4.87E-20
MTF1	WEE1	0.61680328	4.87E-20
MTF1	RBM22	0.61696671	4.73E-20
MTF1	VRK2	0.6171616	4.57E-20
MTF1	SEC23A	0.6172645	4.49E-20
MTF1	TM9SF2	0.61750723	4.3E-20
MTF1	RNF220	0.61755515	4.26E-20
MTF1	HECA	0.61801736	3.93E-20
MTF1	NAGA	0.61849222	3.61E-20
MTF1	LRBA	0.618656	3.51E-20
MTF1	IPPK	0.61904829	3.27E-20
MTF1	SETD3	0.61909269	3.24E-20
MTF1	ACSL4	0.6192677	3.14E-20
MTF1	CREBL2	0.61936999	3.09E-20

MTF1	STAU1	0.61938496	3.08E-20
MTF1	PRKAG2	0.61941989	3.06E-20
MTF1	TRAM1	0.61941991	3.06E-20
MTF1	RNF139	0.61960729	2.96E-20
MTF1	NCBP2	0.62012048	2.7E-20
MTF1	RCAN3	0.62014576	2.69E-20
MTF1	HERC3	0.62023019	2.65E-20
MTF1	NID1	0.62031889	2.61E-20
MTF1	RECK	0.62045918	2.54E-20
MTF1	CSNK2A1	0.62055451	2.5E-20
MTF1	NKRF	0.62063597	2.46E-20
MTF1	MKRN1	0.62077811	2.4E-20
MTF1	GALC	0.62090444	2.35E-20
MTF1	C2CD3	0.62095373	2.33E-20
MTF1	NR2F2	0.62102076	2.3E-20
MTF1	SCP2	0.62131377	2.18E-20
MTF1	GABPB1	0.62141934	2.14E-20
MTF1	ARID5B	0.62152076	2.1E-20
MTF1	CAPRIN1	0.62160798	2.07E-20
MTF1	NRP1	0.62174703	2.02E-20
MTF1	WIPF2	0.62175917	2.01E-20
MTF1	SURF4	0.62180118	2E-20
MTF1	TLE4	0.62200511	1.92E-20
MTF1	PRRC2C	0.62218003	1.86E-20
MTF1	RBBP9	0.62224242	1.84E-20
MTF1	TCF4	0.62237034	1.8E-20
MTF1	MPHOSPH10	0.62250119	1.76E-20
MTF1	TLR4	0.62256191	1.74E-20
MTF1	SAMD8	0.62268633	1.7E-20
MTF1	C9orf78	0.62290411	1.64E-20
MTF1	ZEB1	0.62295761	1.62E-20
MTF1	NCK1	0.62312463	1.57E-20
MTF1	ABCF2	0.62343568	1.49E-20
MTF1	NOCT	0.62346322	1.48E-20
MTF1	ZC3H15	0.62353798	1.46E-20
MTF1	BCL9	0.6237027	1.42E-20
MTF1	PEX19	0.62378147	1.4E-20
MTF1	GLE1	0.62381881	1.39E-20
MTF1	CAB39	0.62384507	1.38E-20
MTF1	NUP62	0.62386516	1.38E-20
MTF1	MEX3C	0.62386996	1.37E-20
MTF1	DPH3	0.62404351	1.33E-20
MTF1	BACH1	0.62415184	1.31E-20
MTF1	SRSF3	0.62421495	1.29E-20
MTF1	QKI	0.62432288	1.27E-20
MTF1	OGDH	0.62436799	1.26E-20
MTF1	COPS4	0.62439863	1.25E-20
MTF1	SYS1	0.62458423	1.21E-20
MTF1	SUCLA2	0.62475205	1.17E-20
MTF1	PPCS	0.62479076	1.16E-20
MTF1	RNF2	0.62496461	1.13E-20
MTF1	CRCP	0.62505968	1.11E-20
MTF1	SNAP23	0.62509555	1.1E-20
MTF1	FASTKD5	0.62518433	1.08E-20
MTF1	ATP8B2	0.62522711	1.07E-20
MTF1	SLC30A1	0.62534897	1.05E-20
MTF1	CXorf56	0.62536045	1.05E-20
MTF1	SAR1B	0.62538995	1.04E-20

MTF1	PSMD10	0.62546054	1.03E-20
MTF1	RPAP3	0.62554084	1.01E-20
MTF1	DIP2C	0.62573637	9.78E-21
MTF1	IQCK	0.62580594	9.66E-21
MTF1	CRLF3	0.62583343	9.61E-21
MTF1	RAB28	0.62583879	9.6E-21
MTF1	SGCB	0.62599154	9.34E-21
MTF1	NRBF2	0.62600004	9.32E-21
MTF1	TMEM33	0.62607636	9.19E-21
MTF1	SP1	0.62612023	9.12E-21
MTF1	TEX10	0.62612502	9.11E-21
MTF1	KCTD12	0.62641247	8.65E-21
MTF1	ACTR6	0.62657536	8.39E-21
MTF1	PFKM	0.62663766	8.3E-21
MTF1	SMARCE1	0.62666783	8.25E-21
MTF1	LRRC57	0.62670274	8.2E-21
MTF1	PRKD3	0.62671174	8.19E-21
MTF1	FBXO5	0.62673191	8.16E-21
MTF1	TBC1D9	0.62681042	8.04E-21
MTF1	EXOC6B	0.62697619	7.8E-21
MTF1	ERBIN	0.62710155	7.62E-21
MTF1	AKT3	0.62713832	7.57E-21
MTF1	WDFY1	0.62729543	7.35E-21
MTF1	COPS8	0.62738173	7.24E-21
MTF1	PHACTR4	0.62750672	7.07E-21
MTF1	GBE1	0.62756592	7E-21
MTF1	DUSP11	0.62778906	6.72E-21
MTF1	ATE1	0.62782046	6.68E-21
MTF1	KLF7	0.62795576	6.51E-21
MTF1	FOXP1	0.62810309	6.34E-21
MTF1	CDK19	0.62834981	6.06E-21
MTF1	TNRC18	0.62850479	5.89E-21
MTF1	MBNL2	0.62850494	5.89E-21
MTF1	MYO5A	0.62850603	5.89E-21
MTF1	AHCTF1	0.628507	5.88E-21
MTF1	MINDY2	0.62860465	5.78E-21
MTF1	SLU7	0.62866017	5.72E-21
MTF1	SMURF2	0.62889934	5.47E-21
MTF1	SH3PXD2A	0.62903425	5.34E-21
MTF1	TCEA1	0.62950422	4.89E-21
MTF1	IST1	0.62952428	4.88E-21
MTF1	SGMS2	0.62955246	4.85E-21
MTF1	PGM2	0.62956236	4.84E-21
MTF1	SH3D19	0.62974971	4.68E-21
MTF1	PARN	0.62990821	4.54E-21
MTF1	ISCA1	0.6300067	4.46E-21
MTF1	TRAFD1	0.63029458	4.23E-21
MTF1	GINM1	0.63031334	4.21E-21
MTF1	ARMH4	0.63033646	4.2E-21
MTF1	MGAT5	0.63046404	4.1E-21
MTF1	ABCC4	0.63046747	4.09E-21
MTF1	AIMP1	0.63066595	3.95E-21
MTF1	NDEL1	0.63094321	3.75E-21
MTF1	RSPH3	0.63120291	3.57E-21
MTF1	KLF12	0.63139668	3.44E-21
MTF1	ZFP90	0.6317208	3.24E-21
MTF1	WBP11	0.63208669	3.03E-21
MTF1	DFFA	0.63211183	3.01E-21

MTF1	AAR2	0.63211467	3.01E-21
MTF1	MGRN1	0.63217974	2.98E-21
MTF1	GMCL1	0.63232427	2.9E-21
MTF1	SLC25A44	0.63237286	2.87E-21
MTF1	MED8	0.63241585	2.85E-21
MTF1	KIAA1671	0.63253336	2.79E-21
MTF1	BNIP3L	0.63293853	2.58E-21
MTF1	DIPK1A	0.6329496	2.58E-21
MTF1	RNF19B	0.63329322	2.42E-21
MTF1	FNDC3B	0.63331724	2.41E-21
MTF1	SNX1	0.63334397	2.39E-21
MTF1	KDM2A	0.63342317	2.36E-21
MTF1	SORL1	0.63352726	2.31E-21
MTF1	PIP4P2	0.63357685	2.29E-21
MTF1	ZNF143	0.63401561	2.11E-21
MTF1	KLHL5	0.63417891	2.05E-21
MTF1	KRCC1	0.63426643	2.01E-21
MTF1	PHF20	0.63485304	1.8E-21
MTF1	ARHGAP31	0.63498036	1.76E-21
MTF1	MIS18BP1	0.63504763	1.74E-21
MTF1	PIGX	0.63509547	1.72E-21
MTF1	ITGAV	0.63515294	1.7E-21
MTF1	VKORC1L1	0.63532067	1.65E-21
MTF1	JKAMP	0.63543439	1.61E-21
MTF1	GNS	0.63545923	1.61E-21
MTF1	TFCP2	0.63602784	1.44E-21
MTF1	FAM120AOS	0.6360884	1.43E-21
MTF1	FRYL	0.63622237	1.39E-21
MTF1	EDC3	0.63632843	1.36E-21
MTF1	ZNF766	0.63644191	1.33E-21
MTF1	GPD2	0.63681093	1.24E-21
MTF1	SS18	0.63691258	1.22E-21
MTF1	ANKS1A	0.63697069	1.21E-21
MTF1	PIP5K1A	0.63697158	1.21E-21
MTF1	CNIH1	0.63705928	1.19E-21
MTF1	RNF138	0.6373426	1.12E-21
MTF1	SHOC2	0.63741777	1.11E-21
MTF1	AP003108.2	0.63756303	1.08E-21
MTF1	CMTM6	0.63766375	1.06E-21
MTF1	RDH14	0.63779623	1.03E-21
MTF1	YES1	0.63779945	1.03E-21
MTF1	CYP2U1	0.63788433	1.01E-21
MTF1	ARL2BP	0.63793903	1E-21
MTF1	WSB2	0.63810169	9.72E-22
MTF1	MOB1A	0.63813693	9.66E-22
MTF1	YEATS2	0.63817643	9.59E-22
MTF1	ABRAXAS2	0.63819829	9.55E-22
MTF1	PHACTR2	0.63822356	9.5E-22
MTF1	VAPB	0.63830655	9.35E-22
MTF1	VAPA	0.63837736	9.22E-22
MTF1	SFT2D2	0.6384056	9.17E-22
MTF1	ELK1	0.63840806	9.17E-22
MTF1	BCL9L	0.63843164	9.13E-22
MTF1	TSHZ1	0.63845	9.1E-22
MTF1	POLR2D	0.63865697	8.74E-22
MTF1	PARD3B	0.6386653	8.73E-22
MTF1	CTSO	0.6388385	8.45E-22
MTF1	YWHAQ	0.63887668	8.38E-22

MTF1	GOLGA7	0.63888083	8.38E-22
MTF1	STARD3NL	0.63890693	8.34E-22
MTF1	KPNB1	0.63890724	8.33E-22
MTF1	DNAJA1	0.63891812	8.32E-22
MTF1	RAB7A	0.63903851	8.13E-22
MTF1	INTS12	0.63904982	8.11E-22
MTF1	GFPT1	0.63915511	7.95E-22
MTF1	DAZAP2	0.6393619	7.64E-22
MTF1	NXPE3	0.63949747	7.44E-22
MTF1	CSGALNACT2	0.63953709	7.39E-22
MTF1	FBXO7	0.63959613	7.3E-22
MTF1	HEG1	0.63970147	7.16E-22
MTF1	TSNAX	0.63978766	7.04E-22
MTF1	TGFBR1	0.63989718	6.89E-22
MTF1	SRGAP2B	0.63993392	6.84E-22
MTF1	TTC4	0.64022242	6.47E-22
MTF1	IQSEC1	0.64023045	6.46E-22
MTF1	KDM3B	0.64036702	6.3E-22
MTF1	TBL1XR1	0.64036892	6.29E-22
MTF1	ISG20L2	0.64044738	6.2E-22
MTF1	FAM98B	0.64047919	6.16E-22
MTF1	SPATS2	0.64080362	5.79E-22
MTF1	SLFN11	0.64083857	5.75E-22
MTF1	GLT8D1	0.64109697	5.47E-22
MTF1	TYW1	0.64113638	5.43E-22
MTF1	NFE2L2	0.64126998	5.29E-22
MTF1	GFM1	0.64134228	5.22E-22
MTF1	GLCE	0.64137725	5.18E-22
MTF1	ETS1	0.64140765	5.15E-22
MTF1	ODF2	0.6415087	5.05E-22
MTF1	SMIM13	0.64168856	4.88E-22
MTF1	IRF2BP2	0.64172171	4.85E-22
MTF1	PPP1CB	0.64204014	4.56E-22
MTF1	VAMP3	0.64227931	4.35E-22
MTF1	CCNG2	0.64232027	4.32E-22
MTF1	TMEM248	0.64237275	4.27E-22
MTF1	SH3BGRL	0.64242597	4.23E-22
MTF1	WDR20	0.64251141	4.16E-22
MTF1	TMOD3	0.64288408	3.87E-22
MTF1	BTF3L4	0.64288685	3.87E-22
MTF1	SYNJ2	0.64301432	3.77E-22
MTF1	TMEM87B	0.64303009	3.76E-22
MTF1	RAB1A	0.6430687	3.73E-22
MTF1	MID1	0.64339692	3.5E-22
MTF1	RNF168	0.64344221	3.47E-22
MTF1	HMGN4	0.64345129	3.47E-22
MTF1	DNM1L	0.64349986	3.43E-22
MTF1	KREMEN1	0.64350752	3.43E-22
MTF1	PNO1	0.64357123	3.39E-22
MTF1	SESTD1	0.64358163	3.38E-22
MTF1	GSPT1	0.6435873	3.37E-22
MTF1	CHM	0.64365607	3.33E-22
MTF1	C5orf15	0.64369593	3.3E-22
MTF1	TSPYL1	0.64372818	3.28E-22
MTF1	APP	0.64373842	3.28E-22
MTF1	RFX5	0.64378019	3.25E-22
MTF1	ATP6AP2	0.64417059	3.01E-22
MTF1	UBXN2B	0.64432614	2.92E-22

MTF1	DIP2B	0.64471801	2.71E-22
MTF1	ACVR1	0.64484262	2.64E-22
MTF1	TRIM56	0.64494726	2.59E-22
MTF1	EXT2	0.64495798	2.58E-22
MTF1	MED28	0.64506073	2.53E-22
MTF1	PAIP2	0.64511507	2.5E-22
MTF1	ERAP1	0.64515722	2.48E-22
MTF1	PPP3CA	0.64536139	2.39E-22
MTF1	ZCCHC17	0.64536613	2.38E-22
MTF1	NBR1	0.6453987	2.37E-22
MTF1	PLEKHG1	0.64568497	2.24E-22
MTF1	PRPF40A	0.64577565	2.2E-22
MTF1	DEGS1	0.64604203	2.09E-22
MTF1	GRSF1	0.64606775	2.08E-22
MTF1	RAP1B	0.64614337	2.05E-22
MTF1	HAT1	0.64617269	2.03E-22
MTF1	TMEM170A	0.64660055	1.87E-22
MTF1	PTBP3	0.64688871	1.77E-22
MTF1	PDZD8	0.64691202	1.76E-22
MTF1	ALS2	0.64705126	1.71E-22
MTF1	DNAJA2	0.64708639	1.7E-22
MTF1	RNF145	0.6471462	1.68E-22
MTF1	ZNF410	0.64714797	1.68E-22
MTF1	NCDN	0.6471668	1.67E-22
MTF1	DDX18	0.6472504	1.64E-22
MTF1	UBE2D2	0.64750963	1.56E-22
MTF1	ABI1	0.64770404	1.5E-22
MTF1	TERF2	0.64773443	1.49E-22
MTF1	CALM1	0.64779788	1.48E-22
MTF1	FBXL17	0.64821584	1.36E-22
MTF1	PTPRJ	0.64851652	1.28E-22
MTF1	TMEM263	0.64853416	1.28E-22
MTF1	FAM20B	0.64868501	1.24E-22
MTF1	SGPL1	0.64895008	1.17E-22
MTF1	TVP23B	0.64897168	1.17E-22
MTF1	HACD2	0.64900077	1.16E-22
MTF1	AGPS	0.64903194	1.16E-22
MTF1	GTF2E1	0.6491276	1.13E-22
MTF1	SLC4A7	0.64914362	1.13E-22
MTF1	PEX26	0.64947985	1.06E-22
MTF1	SDCBP	0.64967581	1.02E-22
MTF1	GNA13	0.64974528	1E-22
MTF1	PPP3CB	0.64981229	9.89E-23
MTF1	LAMP2	0.64982024	9.87E-23
MTF1	SNX9	0.65003301	9.46E-23
MTF1	TAF1B	0.65007599	9.38E-23
MTF1	PDCD6IP	0.65019908	9.16E-23
MTF1	MSH2	0.65031211	8.95E-23
MTF1	NDUFS1	0.65039561	8.8E-23
MTF1	SCAMP1	0.65059786	8.45E-23
MTF1	TCF20	0.65074233	8.21E-23
MTF1	MCMBP	0.65077812	8.15E-23
MTF1	PTPRB	0.65134419	7.28E-23
MTF1	MFAP1	0.65139121	7.21E-23
MTF1	NUDCD3	0.65139218	7.21E-23
MTF1	LCORL	0.65144997	7.13E-23
MTF1	SKI	0.65180337	6.64E-23
MTF1	CCDC47	0.6518385	6.59E-23

MTF1	DYNC1LI1	0.65192608	6.48E-23
MTF1	IFT52	0.65200441	6.38E-23
MTF1	UBA3	0.65205119	6.32E-23
MTF1	SPRED2	0.65227424	6.04E-23
MTF1	MAFG	0.65256027	5.7E-23
MTF1	WWP1	0.65260318	5.65E-23
MTF1	CHUK	0.65276865	5.47E-23
MTF1	RAB8A	0.65283357	5.4E-23
MTF1	EID1	0.65297196	5.25E-23
MTF1	TRMT1L	0.65300903	5.21E-23
MTF1	SLC25A24	0.65305968	5.15E-23
MTF1	DCUN1D3	0.6531914	5.02E-23
MTF1	COPS2	0.65328892	4.92E-23
MTF1	LDAH	0.6533868	4.83E-23
MTF1	CBFB	0.65345737	4.76E-23
MTF1	EXTL3	0.65350914	4.71E-23
MTF1	TAF5L	0.65355665	4.66E-23
MTF1	GEMIN5	0.65372038	4.51E-23
MTF1	KIAA1143	0.65380863	4.43E-23
MTF1	TMCC1	0.65398368	4.28E-23
MTF1	VPS35	0.65428777	4.02E-23
MTF1	SMU1	0.65442	3.91E-23
MTF1	TAX1BP1	0.65450127	3.85E-23
MTF1	MAP2K1	0.65456679	3.8E-23
MTF1	YWHAB	0.65459248	3.78E-23
MTF1	MTMR2	0.65461382	3.76E-23
MTF1	SRSF1	0.65466303	3.73E-23
MTF1	ATP9A	0.65496625	3.5E-23
MTF1	DEK	0.65516163	3.37E-23
MTF1	LRRCC1	0.65516795	3.36E-23
MTF1	JARID2	0.65529026	3.28E-23
MTF1	RAB22A	0.65554514	3.11E-23
MTF1	OAZ2	0.65569292	3.02E-23
MTF1	GOLT1B	0.65580518	2.95E-23
MTF1	LRCH3	0.65581002	2.95E-23
MTF1	ENAH	0.65587496	2.91E-23
MTF1	STRN	0.65597798	2.85E-23
MTF1	ABHD13	0.65603158	2.82E-23
MTF1	TMEM209	0.6561919	2.73E-23
MTF1	MLH1	0.65646662	2.58E-23
MTF1	MED20	0.65656434	2.53E-23
MTF1	KRR1	0.65687358	2.37E-23
MTF1	GRPEL2	0.65719011	2.22E-23
MTF1	ZFYVE1	0.65720774	2.22E-23
MTF1	AP1AR	0.65737881	2.14E-23
MTF1	UBA6	0.65744043	2.11E-23
MTF1	RSF1	0.65748704	2.09E-23
MTF1	RFWD3	0.65788599	1.93E-23
MTF1	SLC26A2	0.65796809	1.9E-23
MTF1	RAB3GAP2	0.65797125	1.89E-23
MTF1	NUB1	0.65817437	1.82E-23
MTF1	PPP3R1	0.65819554	1.81E-23
MTF1	MAP3K7	0.65821671	1.8E-23
MTF1	WDR26	0.65825294	1.79E-23
MTF1	ACBD3	0.6584488	1.72E-23
MTF1	ISY1-RAB43	0.65900583	1.53E-23
MTF1	RAB10	0.65931915	1.43E-23
MTF1	CAP1	0.65973052	1.32E-23

MTF1	HNRNPLL	0.65975403	1.31E-23
MTF1	CCNK	0.66001982	1.24E-23
MTF1	DNAJB6	0.66005676	1.23E-23
MTF1	PICALM	0.66010037	1.22E-23
MTF1	SH2B3	0.66012147	1.22E-23
MTF1	TTL	0.66013842	1.21E-23
MTF1	POLR2C	0.6603831	1.15E-23
MTF1	OSGIN2	0.66049371	1.12E-23
MTF1	SWAP70	0.66054239	1.11E-23
MTF1	CMIP	0.66067184	1.08E-23
MTF1	ZNF281	0.66072182	1.07E-23
MTF1	PTPRA	0.66083778	1.05E-23
MTF1	MAGT1	0.66092524	1.03E-23
MTF1	TAOK3	0.66097525	1.02E-23
MTF1	NSL1	0.66109947	9.92E-24
MTF1	BPTF	0.66115115	9.81E-24
MTF1	STIM1	0.66138568	9.34E-24
MTF1	CPSF6	0.66161731	8.9E-24
MTF1	FBXO42	0.66173627	8.68E-24
MTF1	SNX2	0.66186845	8.45E-24
MTF1	RAB5B	0.662102	8.05E-24
MTF1	PANK3	0.66232379	7.68E-24
MTF1	PALLD	0.66253678	7.35E-24
MTF1	PCNX1	0.66261865	7.22E-24
MTF1	EXO5	0.6627349	7.05E-24
MTF1	ARMC8	0.66289157	6.82E-24
MTF1	SPRTN	0.6629566	6.73E-24
MTF1	WDR89	0.6630592	6.58E-24
MTF1	CIPC	0.6630668	6.57E-24
MTF1	WASL	0.66311738	6.51E-24
MTF1	PRKACA	0.66315926	6.45E-24
MTF1	SOCS6	0.6631643	6.44E-24
MTF1	ARL8B	0.66327914	6.29E-24
MTF1	VPS33A	0.6632966	6.26E-24
MTF1	DERL1	0.66356957	5.92E-24
MTF1	RASAL2	0.66358868	5.89E-24
MTF1	RANBP9	0.66385674	5.57E-24
MTF1	PLAA	0.66389303	5.53E-24
MTF1	PTDSS1	0.66395742	5.45E-24
MTF1	UHRF1BP1L	0.66407246	5.32E-24
MTF1	HIVEP2	0.66420311	5.18E-24
MTF1	GOLPH3	0.66432049	5.05E-24
MTF1	TMEM127	0.66442182	4.94E-24
MTF1	IRF2	0.66454814	4.81E-24
MTF1	HECW2	0.66466149	4.7E-24
MTF1	CNOT8	0.66468427	4.68E-24
MTF1	TMX1	0.66469081	4.67E-24
MTF1	EPB41L2	0.66470616	4.66E-24
MTF1	USP10	0.66478266	4.58E-24
MTF1	UVRAG	0.66502225	4.36E-24
MTF1	SEC22A	0.66517424	4.22E-24
MTF1	YY1	0.66520767	4.19E-24
MTF1	BMP2K	0.66536089	4.05E-24
MTF1	MAPK6	0.66556538	3.88E-24
MTF1	ATG5	0.66587617	3.64E-24
MTF1	ZBTB41	0.66589927	3.62E-24
MTF1	ITFG1	0.66594373	3.58E-24
MTF1	RNF141	0.66595998	3.57E-24

MTF1	CNOT9	0.66614608	3.43E-24
MTF1	PSME3	0.66615043	3.43E-24
MTF1	PEX2	0.66626603	3.35E-24
MTF1	MFSD14B	0.66651558	3.17E-24
MTF1	SETX	0.66660514	3.11E-24
MTF1	KIAA1191	0.66681103	2.98E-24
MTF1	LRP12	0.66682955	2.97E-24
MTF1	RPRD2	0.66687687	2.94E-24
MTF1	TRUB1	0.666993	2.87E-24
MTF1	SNRNP27	0.6671869	2.75E-24
MTF1	TRA2B	0.66737375	2.64E-24
MTF1	LYRM2	0.66781852	2.4E-24
MTF1	GLB1	0.66785469	2.39E-24
MTF1	TSN	0.66785898	2.38E-24
MTF1	TCF12	0.66791863	2.35E-24
MTF1	ELF1	0.66826409	2.19E-24
MTF1	CDK17	0.66842182	2.11E-24
MTF1	CRIM1	0.6684714	2.09E-24
MTF1	GSTCD	0.6685747	2.05E-24
MTF1	TNPO3	0.66857663	2.04E-24
MTF1	ATP2C1	0.66859695	2.04E-24
MTF1	ORC4	0.66891151	1.9E-24
MTF1	NSRP1	0.66914067	1.81E-24
MTF1	DDX21	0.6692064	1.79E-24
MTF1	HINT3	0.66923571	1.78E-24
MTF1	TIGAR	0.66925937	1.77E-24
MTF1	KCTD10	0.66929829	1.75E-24
MTF1	HNRNPU	0.66942349	1.7E-24
MTF1	SMARCC1	0.6695706	1.65E-24
MTF1	FCHSD2	0.66966885	1.62E-24
MTF1	SMC2	0.66985507	1.55E-24
MTF1	MBNL1	0.66992618	1.53E-24
MTF1	STK4	0.67025451	1.43E-24
MTF1	RPA1	0.67026975	1.42E-24
MTF1	STAM	0.67044993	1.37E-24
MTF1	KLHL20	0.67049723	1.35E-24
MTF1	EXOC2	0.67054157	1.34E-24
MTF1	ZBTB2	0.67062035	1.32E-24
MTF1	DNAJC21	0.67089704	1.24E-24
MTF1	NUP50	0.67118547	1.17E-24
MTF1	UBE2N	0.67131115	1.13E-24
MTF1	ARMT1	0.67136405	1.12E-24
MTF1	CERS6	0.67139377	1.11E-24
MTF1	ZNF720	0.671439	1.1E-24
MTF1	SET	0.67162748	1.06E-24
MTF1	APOL6	0.6717247	1.04E-24
MTF1	CBX5	0.67184745	1.01E-24
MTF1	OTULIN	0.67190222	9.98E-25
MTF1	XYLT1	0.67247929	8.81E-25
MTF1	KBTBD2	0.6728748	8.08E-25
MTF1	RAB8B	0.67309409	7.7E-25
MTF1	UBTD2	0.67346097	7.11E-25
MTF1	SLC12A6	0.67349193	7.06E-25
MTF1	ITPRIPL2	0.67372753	6.71E-25
MTF1	CYLD	0.67373564	6.7E-25
MTF1	VPS37A	0.67400562	6.31E-25
MTF1	ADAR	0.6745765	5.57E-25
MTF1	LIN52	0.67479504	5.31E-25

MTF1	HOMER1	0.67486351	5.23E-25
MTF1	NUP58	0.67490284	5.18E-25
MTF1	SMNDC1	0.67492949	5.15E-25
MTF1	WDR3	0.67495842	5.12E-25
MTF1	SNX6	0.6749775	5.1E-25
MTF1	APBB2	0.67507064	5E-25
MTF1	RBBP5	0.67515097	4.91E-25
MTF1	NUDT3	0.67541423	4.63E-25
MTF1	FOXN2	0.67549212	4.55E-25
MTF1	CDC42EP3	0.67577952	4.28E-25
MTF1	LNPK	0.67603036	4.05E-25
MTF1	ACTR3	0.67660097	3.57E-25
MTF1	SNX18	0.67669615	3.49E-25
MTF1	MBTPS1	0.67671676	3.48E-25
MTF1	CDV3	0.67680974	3.41E-25
MTF1	SRFBP1	0.67681402	3.4E-25
MTF1	ZMIZ1	0.67681464	3.4E-25
MTF1	FAM91A1	0.67682657	3.39E-25
MTF1	ZNF227	0.67690081	3.34E-25
MTF1	PPM1A	0.6770948	3.2E-25
MTF1	RAD21	0.67726547	3.08E-25
MTF1	RAD1	0.67739324	2.99E-25
MTF1	CNOT7	0.67771172	2.79E-25
MTF1	DOCK9	0.67785968	2.7E-25
MTF1	MAPRE2	0.67794802	2.65E-25
MTF1	HNRNPUL1	0.67813291	2.54E-25
MTF1	PLBD2	0.67816295	2.52E-25
MTF1	LARP7	0.67820241	2.5E-25
MTF1	PPP1R2	0.6782673	2.46E-25
MTF1	ZNF468	0.67842269	2.38E-25
MTF1	ZKSCAN5	0.67855818	2.31E-25
MTF1	ICE2	0.67856858	2.3E-25
MTF1	COMMD2	0.67870362	2.24E-25
MTF1	FNBP1L	0.67886115	2.16E-25
MTF1	ADH5	0.67890795	2.14E-25
MTF1	ZNF260	0.67895762	2.11E-25
MTF1	ORAI2	0.6789777	2.1E-25
MTF1	SLC39A6	0.67929556	1.96E-25
MTF1	HUS1	0.67948553	1.88E-25
MTF1	BRAP	0.67954317	1.85E-25
MTF1	MORF4L2	0.67955838	1.85E-25
MTF1	KHDRBS1	0.67959275	1.83E-25
MTF1	GNA12	0.67969273	1.79E-25
MTF1	SDE2	0.67993561	1.7E-25
MTF1	TM2D1	0.68022793	1.59E-25
MTF1	SCAF11	0.68034624	1.55E-25
MTF1	SERBP1	0.68045499	1.51E-25
MTF1	TBK1	0.6804631	1.51E-25
MTF1	UGDH	0.68065983	1.44E-25
MTF1	PPM1B	0.6808017	1.4E-25
MTF1	CREB1	0.68103421	1.33E-25
MTF1	TCTN3	0.68121127	1.28E-25
MTF1	GAB2	0.68123552	1.27E-25
MTF1	FAM120A	0.68127035	1.26E-25
MTF1	C9orf64	0.68138422	1.23E-25
MTF1	FNIP2	0.68154874	1.18E-25
MTF1	STRN3	0.68187381	1.1E-25
MTF1	ASAP1	0.68204858	1.06E-25

MTF1	SERINC1	0.68234759	9.87E-26
MTF1	IL6ST	0.6823916	9.77E-26
MTF1	SH3GLB1	0.68256342	9.4E-26
MTF1	GNAI3	0.68263064	9.25E-26
MTF1	CNST	0.68388059	6.97E-26
MTF1	SART3	0.68395077	6.86E-26
MTF1	TARDBP	0.68402293	6.75E-26
MTF1	IFNAR1	0.68453127	6.01E-26
MTF1	SSX2IP	0.68457355	5.95E-26
MTF1	MIB1	0.68469796	5.78E-26
MTF1	TRIQK	0.68498077	5.42E-26
MTF1	DENND6A	0.68525894	5.09E-26
MTF1	KDM1B	0.68526309	5.08E-26
MTF1	GLYR1	0.68544811	4.87E-26
MTF1	SLC35A4	0.68548911	4.83E-26
MTF1	ATF2	0.68551755	4.8E-26
MTF1	ARHGAP5	0.68564944	4.65E-26
MTF1	CUL1	0.6857634	4.53E-26
MTF1	SMC6	0.6861473	4.15E-26
MTF1	KIF5B	0.68629912	4.01E-26
MTF1	NUFIP2	0.68639193	3.92E-26
MTF1	PTPN1	0.68656982	3.77E-26
MTF1	RAB35	0.68663419	3.71E-26
MTF1	RO60	0.68674617	3.62E-26
MTF1	HAUS2	0.68677278	3.59E-26
MTF1	ZNF184	0.68682806	3.55E-26
MTF1	PPP1CC	0.68693635	3.46E-26
MTF1	KATNAL1	0.68702579	3.39E-26
MTF1	ZNFX1	0.68721035	3.25E-26
MTF1	PIK3R1	0.68743004	3.09E-26
MTF1	NCKAP1	0.68754437	3.01E-26
MTF1	HNRNPR	0.68773214	2.88E-26
MTF1	CDC42	0.6878299	2.82E-26
MTF1	PLEKHB2	0.68787565	2.79E-26
MTF1	LNPEP	0.68801216	2.7E-26
MTF1	SYNCRIP	0.68803049	2.69E-26
MTF1	ABHD17B	0.68804619	2.68E-26
MTF1	NUDT4B	0.68822612	2.57E-26
MTF1	BAG5	0.68830435	2.52E-26
MTF1	DPY19L4	0.6885369	2.39E-26
MTF1	PSMC2	0.68883401	2.23E-26
MTF1	RSRC1	0.68887827	2.21E-26
MTF1	SAP130	0.68909038	2.1E-26
MTF1	ESF1	0.68934257	1.98E-26
MTF1	C16orf70	0.68942223	1.95E-26
MTF1	RAB5A	0.68970736	1.82E-26
MTF1	RAB2A	0.68986216	1.76E-26
MTF1	PDHX	0.68988483	1.75E-26
MTF1	CDYL	0.68993134	1.73E-26
MTF1	GNPTAB	0.68995676	1.72E-26
MTF1	KPNA3	0.69004042	1.69E-26
MTF1	SP3	0.69005491	1.68E-26
MTF1	USP12	0.69015251	1.64E-26
MTF1	ZFP64	0.69029528	1.59E-26
MTF1	HIVEP1	0.69035676	1.57E-26
MTF1	SUZ12	0.69035979	1.56E-26
MTF1	GCC2	0.69040816	1.55E-26
MTF1	KPNA4	0.69077428	1.42E-26

MTF1	CAMSAP1	0.69092553	1.37E-26
MTF1	SNTB2	0.69116406	1.3E-26
MTF1	CLCN3	0.69116442	1.3E-26
MTF1	ATXN1	0.69134495	1.24E-26
MTF1	ESYT2	0.69159218	1.17E-26
MTF1	TBC1D14	0.69162085	1.16E-26
MTF1	MED14	0.69173641	1.13E-26
MTF1	ZNF623	0.69179955	1.12E-26
MTF1	ABI2	0.69214263	1.03E-26
MTF1	XPR1	0.69221109	1.01E-26
MTF1	GNPDA2	0.69221831	1.01E-26
MTF1	UEVLD	0.69266875	9.1E-27
MTF1	PDLIM5	0.69294365	8.53E-27
MTF1	ZFAND5	0.69297847	8.46E-27
MTF1	FKBP15	0.69304788	8.32E-27
MTF1	BMT2	0.69311608	8.19E-27
MTF1	ANXA7	0.69339645	7.67E-27
MTF1	VHL	0.69356937	7.36E-27
MTF1	PPFIA1	0.69378915	6.99E-27
MTF1	ZNF263	0.69385972	6.87E-27
MTF1	ADAM17	0.69391531	6.78E-27
MTF1	CCNI	0.69406637	6.54E-27
MTF1	RAB6A	0.69408858	6.51E-27
MTF1	SUSD6	0.69412398	6.45E-27
MTF1	ACTR2	0.69442399	6.01E-27
MTF1	CHD9	0.69448529	5.92E-27
MTF1	LRRC41	0.69499729	5.24E-27
MTF1	MBTPS2	0.6951368	5.07E-27
MTF1	RECQL	0.69543584	4.72E-27
MTF1	CDC23	0.69555188	4.59E-27
MTF1	MEAF6	0.69556485	4.58E-27
MTF1	DCLRE1B	0.6955743	4.57E-27
MTF1	CDC42SE2	0.69561351	4.53E-27
MTF1	YIPF6	0.6956737	4.46E-27
MTF1	MTM1	0.69573263	4.4E-27
MTF1	HPS5	0.69587715	4.25E-27
MTF1	RTF1	0.69588476	4.24E-27
MTF1	PARG	0.69600554	4.12E-27
MTF1	MAPRE1	0.69613371	4E-27
MTF1	CWC22	0.69665207	3.53E-27
MTF1	TRIM32	0.69684454	3.37E-27
MTF1	MEGF9	0.69690637	3.32E-27
MTF1	DCK	0.69695456	3.28E-27
MTF1	ATP13A3	0.69707236	3.19E-27
MTF1	GPATCH11	0.69759581	2.82E-27
MTF1	PLSCR4	0.6976497	2.78E-27
MTF1	MAPK14	0.69769315	2.75E-27
MTF1	PMS2	0.69782673	2.66E-27
MTF1	YWHAG	0.69785469	2.65E-27
MTF1	CALCOCO2	0.69802084	2.54E-27
MTF1	KLHL2	0.69807587	2.51E-27
MTF1	STX7	0.69824943	2.41E-27
MTF1	LZIC	0.69842688	2.3E-27
MTF1	CSTF2T	0.69861222	2.2E-27
MTF1	TRANK1	0.69928736	1.87E-27
MTF1	FAM98A	0.69942103	1.81E-27
MTF1	CCSER2	0.69951695	1.77E-27
MTF1	PURB	0.69969951	1.69E-27

MTF1	KCTD20	0.69983147	1.64E-27
MTF1	ATP6V1C1	0.69995493	1.59E-27
MTF1	RAP1A	0.70037752	1.44E-27
MTF1	FBXO8	0.70045045	1.41E-27
MTF1	HSPA13	0.70067579	1.34E-27
MTF1	MED1	0.70107291	1.21E-27
MTF1	CCNY	0.70119638	1.18E-27
MTF1	VPS41	0.70133941	1.14E-27
MTF1	DCAF12	0.70136685	1.13E-27
MTF1	CANX	0.70153458	1.08E-27
MTF1	SSR1	0.70153701	1.08E-27
MTF1	EMC1	0.70157284	1.07E-27
MTF1	CTCF	0.70165073	1.05E-27
MTF1	GNG12	0.70201335	9.63E-28
MTF1	PPP4R3B	0.70212374	9.38E-28
MTF1	NF1	0.70222073	9.16E-28
MTF1	SLC31A1	0.70226742	9.05E-28
MTF1	SPAST	0.70227569	9.03E-28
MTF1	RNF13	0.70237494	8.82E-28
MTF1	ICMT	0.70250839	8.53E-28
MTF1	XRN2	0.70259308	8.36E-28
MTF1	DRAM2	0.70263568	8.27E-28
MTF1	PTPRG	0.7026971	8.15E-28
MTF1	PKD2	0.70270491	8.13E-28
MTF1	BCAP29	0.70276628	8.01E-28
MTF1	RAB3GAP1	0.70288433	7.78E-28
MTF1	R3HDM1	0.70290423	7.74E-28
MTF1	ARL14EP	0.70294847	7.66E-28
MTF1	RAB14	0.70317003	7.25E-28
MTF1	CDK12	0.70348024	6.72E-28
MTF1	IMPA1	0.70373455	6.31E-28
MTF1	UBA2	0.70384298	6.14E-28
MTF1	FBXL5	0.70387806	6.09E-28
MTF1	KDSR	0.70421946	5.6E-28
MTF1	PPIG	0.70425345	5.55E-28
MTF1	SP2	0.70447266	5.26E-28
MTF1	DDX19A	0.70485664	4.78E-28
MTF1	PCYOX1	0.70486195	4.77E-28
MTF1	SCARB2	0.70497886	4.64E-28
MTF1	SMG8	0.70507905	4.52E-28
MTF1	ARL1	0.70510402	4.5E-28
MTF1	FYCO1	0.70511967	4.48E-28
MTF1	VPS4B	0.70520357	4.39E-28
MTF1	TMED7	0.70520772	4.38E-28
MTF1	DYRK2	0.70521779	4.37E-28
MTF1	TOX4	0.70544548	4.13E-28
MTF1	FGFR1OP2	0.70568407	3.89E-28
MTF1	TPP1	0.70576855	3.81E-28
MTF1	PHTF1	0.70593913	3.65E-28
MTF1	SOCS5	0.70598644	3.61E-28
MTF1	SMC3	0.70628358	3.35E-28
MTF1	EFR3A	0.70631325	3.33E-28
MTF1	RNF6	0.70645268	3.21E-28
MTF1	NPAT	0.70732218	2.59E-28
MTF1	IL13RA1	0.70732587	2.58E-28
MTF1	RPL7L1	0.70744645	2.51E-28
MTF1	RBM7	0.70761479	2.4E-28
MTF1	TMTC3	0.70771499	2.34E-28

MTF1	DHDDS	0.70780577	2.29E-28
MTF1	CNOT4	0.70783049	2.28E-28
MTF1	ROCK2	0.7079008	2.24E-28
MTF1	MFSD1	0.70825621	2.05E-28
MTF1	RRM2B	0.70826175	2.04E-28
MTF1	PRRC1	0.70850902	1.92E-28
MTF1	RAD23B	0.70855684	1.9E-28
MTF1	TULP3	0.70873682	1.81E-28
MTF1	RHBDD1	0.70882109	1.77E-28
MTF1	NAA50	0.70899635	1.7E-28
MTF1	TRAF3	0.70925202	1.59E-28
MTF1	UTP14C	0.70938447	1.54E-28
MTF1	MBD5	0.7094397	1.52E-28
MTF1	GNB1	0.70975229	1.4E-28
MTF1	CEP41	0.709796	1.39E-28
MTF1	SEC23IP	0.70980932	1.38E-28
MTF1	NCOA4	0.71017878	1.26E-28
MTF1	DIS3	0.71021955	1.25E-28
MTF1	SLC25A32	0.71026418	1.23E-28
MTF1	IGF2R	0.71052188	1.15E-28
MTF1	FAM102B	0.71072925	1.09E-28
MTF1	PPT1	0.71078294	1.08E-28
MTF1	SMARCA5	0.71098592	1.03E-28
MTF1	PUM2	0.7109922	1.02E-28
MTF1	RP2	0.71138266	9.27E-29
MTF1	FBXO45	0.7114494	9.11E-29
MTF1	C5orf24	0.71158992	8.79E-29
MTF1	PTPN9	0.71172317	8.5E-29
MTF1	LARS2	0.71192402	8.07E-29
MTF1	METTL9	0.7121064	7.71E-29
MTF1	BZW1	0.71217914	7.56E-29
MTF1	ACLY	0.71256025	6.86E-29
MTF1	COPA	0.71268804	6.64E-29
MTF1	ARF3	0.71279368	6.46E-29
MTF1	BMI1	0.712856	6.36E-29
MTF1	LPIN2	0.7129073	6.28E-29
MTF1	NBN	0.71309998	5.97E-29
MTF1	PRR14L	0.71321277	5.8E-29
MTF1	CAMK2D	0.71334476	5.61E-29
MTF1	DHX36	0.71348857	5.41E-29
MTF1	ZNF827	0.71357338	5.29E-29
MTF1	OSBPL11	0.71363633	5.2E-29
MTF1	TRAM2	0.71377569	5.02E-29
MTF1	DLAT	0.71383291	4.95E-29
MTF1	TMEM19	0.7140009	4.74E-29
MTF1	PPP2R5E	0.71400617	4.73E-29
MTF1	TRAK2	0.71415481	4.55E-29
MTF1	TRIP12	0.71424158	4.45E-29
MTF1	GORASP2	0.71447768	4.19E-29
MTF1	BMPR2	0.7146287	4.03E-29
MTF1	PAK2	0.7148007	3.85E-29
MTF1	LMBR1	0.71493801	3.72E-29
MTF1	OSBPL8	0.71506499	3.6E-29
MTF1	FBXO38	0.71567223	3.08E-29
MTF1	SPOP	0.71589419	2.9E-29
MTF1	ADAM10	0.71621198	2.67E-29
MTF1	BCL2L13	0.71639918	2.55E-29
MTF1	PAQR3	0.7167754	2.31E-29

MTF1	TOP1	0.71677615	2.31E-29
MTF1	TOB2	0.71684136	2.27E-29
MTF1	NEK1	0.71725395	2.04E-29
MTF1	TRAF3IP1	0.71734403	1.99E-29
MTF1	PNRC2	0.71760732	1.86E-29
MTF1	CUL4B	0.7176136	1.85E-29
MTF1	CSNK1A1	0.71776049	1.78E-29
MTF1	IPO7	0.71776179	1.78E-29
MTF1	TXNRD1	0.71789098	1.72E-29
MTF1	SPPL3	0.71794925	1.7E-29
MTF1	SERINC3	0.71808605	1.64E-29
MTF1	USP14	0.71824404	1.57E-29
MTF1	RRAGC	0.71864513	1.41E-29
MTF1	HBP1	0.71926246	1.2E-29
MTF1	MACF1	0.71932807	1.18E-29
MTF1	GNAQ	0.71933994	1.18E-29
MTF1	SLC25A40	0.71939001	1.16E-29
MTF1	MORF4L1	0.7194254	1.15E-29
MTF1	BRD3	0.7194418	1.15E-29
MTF1	ST13	0.71987938	1.02E-29
MTF1	ATF7IP	0.71994457	1E-29
MTF1	IDS	0.72010507	9.62E-30
MTF1	SNX29	0.72042707	8.84E-30
MTF1	AKAP11	0.72043668	8.81E-30
MTF1	STT3B	0.72048803	8.7E-30
MTF1	TAF13	0.72056485	8.52E-30
MTF1	TGOLN2	0.72065049	8.33E-30
MTF1	CBL	0.72074383	8.13E-30
MTF1	SLC9A6	0.72082492	7.95E-30
MTF1	RB1	0.7209223	7.75E-30
MTF1	PRPF4	0.72092495	7.74E-30
MTF1	SLC25A46	0.7211958	7.21E-30
MTF1	CRK	0.7212979	7.01E-30
MTF1	TTC26	0.72141327	6.8E-30
MTF1	MED17	0.72176428	6.19E-30
MTF1	YTHDF2	0.72196088	5.88E-30
MTF1	ATP2B1	0.72229382	5.38E-30
MTF1	PRKAR1A	0.72245187	5.16E-30
MTF1	ATMIN	0.72247894	5.12E-30
MTF1	PPP1R12A	0.72259102	4.97E-30
MTF1	CNOT6	0.72262228	4.93E-30
MTF1	FBXL3	0.72276314	4.74E-30
MTF1	CYB5R4	0.72282055	4.67E-30
MTF1	NEO1	0.72283597	4.65E-30
MTF1	ALG11	0.72286919	4.61E-30
MTF1	ZNHIT6	0.72290734	4.56E-30
MTF1	HS2ST1	0.72341191	3.99E-30
MTF1	RPRD1B	0.72345758	3.94E-30
MTF1	RTL6	0.72396907	3.43E-30
MTF1	DAAM1	0.72400007	3.4E-30
MTF1	AGGF1	0.72412543	3.29E-30
MTF1	ZNF562	0.72434558	3.1E-30
MTF1	ZNF644	0.72437201	3.08E-30
MTF1	NFKB1	0.72443856	3.02E-30
MTF1	DCAF1	0.7247459	2.78E-30
MTF1	DNAJB14	0.72494402	2.64E-30
MTF1	PDS5B	0.72515769	2.49E-30
MTF1	MAPK1IP1L	0.72542443	2.32E-30

MTF1	SZRD1	0.72545673	2.3E-30
MTF1	C3orf38	0.72597281	2E-30
MTF1	MED13	0.72616026	1.9E-30
MTF1	PPIL4	0.72630395	1.82E-30
MTF1	RRN3	0.7263365	1.81E-30
MTF1	MTDH	0.72688623	1.56E-30
MTF1	ZNF322	0.72700249	1.51E-30
MTF1	RNF20	0.72704738	1.49E-30
MTF1	GMEB1	0.72709232	1.47E-30
MTF1	LIN54	0.72722371	1.42E-30
MTF1	HIPK3	0.72722796	1.42E-30
MTF1	UBN1	0.72768564	1.25E-30
MTF1	OGFOD1	0.72775374	1.23E-30
MTF1	SOGA1	0.72782136	1.21E-30
MTF1	KIAA0232	0.72793023	1.17E-30
MTF1	BCAS2	0.72795539	1.16E-30
MTF1	CPNE3	0.72819899	1.09E-30
MTF1	TOPORS	0.72851597	9.97E-31
MTF1	NCOA3	0.7287211	9.42E-31
MTF1	PITPNA	0.7288393	9.12E-31
MTF1	FTO	0.72911289	8.46E-31
MTF1	KIF3B	0.72918797	8.29E-31
MTF1	NEDD1	0.72931107	8.01E-31
MTF1	GCLM	0.72945802	7.69E-31
MTF1	IDH3A	0.72966479	7.27E-31
MTF1	MEF2A	0.72992495	6.76E-31
MTF1	FAF2	0.72999295	6.64E-31
MTF1	CSNK1G3	0.73025551	6.17E-31
MTF1	TLK1	0.73028505	6.12E-31
MTF1	COA7	0.73061701	5.59E-31
MTF1	GLG1	0.73103057	4.98E-31
MTF1	CDC5L	0.73110541	4.88E-31
MTF1	ZFP91	0.73138849	4.51E-31
MTF1	UBQLN2	0.73146028	4.42E-31
MTF1	ETF1	0.73146446	4.41E-31
MTF1	NPTN	0.73148814	4.38E-31
MTF1	DLD	0.73163593	4.21E-31
MTF1	HMGXB4	0.73190495	3.9E-31
MTF1	GTF2H3	0.7319715	3.83E-31
MTF1	SLC30A9	0.73198674	3.82E-31
MTF1	CKAP5	0.73201358	3.79E-31
MTF1	ARHGEF12	0.7320255	3.78E-31
MTF1	CLOCK	0.73207409	3.72E-31
MTF1	DNAJC10	0.73240546	3.4E-31
MTF1	NFE2L1	0.73296335	2.9E-31
MTF1	GANAB	0.73308106	2.81E-31
MTF1	VCPIP1	0.73320771	2.71E-31
MTF1	PCYT1A	0.73344007	2.54E-31
MTF1	MFN2	0.73354399	2.47E-31
MTF1	KAT7	0.73377329	2.31E-31
MTF1	ZNF398	0.73385574	2.26E-31
MTF1	STX12	0.7345369	1.87E-31
MTF1	TERF1	0.73517731	1.56E-31
MTF1	HNRNPH2	0.7353559	1.48E-31
MTF1	AP5M1	0.73538886	1.47E-31
MTF1	PDE12	0.73539331	1.47E-31
MTF1	DYRK1A	0.73549502	1.42E-31
MTF1	KBTBD4	0.73564799	1.36E-31

MTF1	ARHGAP35	0.73579866	1.31E-31
MTF1	CTTNBP2NL	0.73582177	1.3E-31
MTF1	UBE2W	0.7363193	1.13E-31
MTF1	VEZF1	0.73647878	1.08E-31
MTF1	TOR1AIP1	0.73649744	1.07E-31
MTF1	QTRT2	0.73649747	1.07E-31
MTF1	SIN3A	0.73669868	1.01E-31
MTF1	CLIP1	0.73713509	8.93E-32
MTF1	PJA2	0.73723822	8.67E-32
MTF1	ZBTB33	0.73746619	8.12E-32
MTF1	CFAP97	0.7377546	7.48E-32
MTF1	ATP6V1A	0.73779981	7.38E-32
MTF1	ZNF609	0.73790526	7.16E-32
MTF1	NCOA2	0.73852875	5.99E-32
MTF1	SNX16	0.73860165	5.87E-32
MTF1	UBE3A	0.73865243	5.78E-32
MTF1	PPP4R2	0.7388799	5.42E-32
MTF1	G3BP1	0.73893297	5.33E-32
MTF1	DLG1	0.73910963	5.07E-32
MTF1	TGS1	0.73931451	4.78E-32
MTF1	PEAK1	0.73941603	4.64E-32
MTF1	GMFB	0.73958034	4.43E-32
MTF1	GOSR1	0.73994225	3.99E-32
MTF1	SPOPL	0.74004641	3.87E-32
MTF1	CUL2	0.74048977	3.4E-32
MTF1	ZC3H13	0.74079853	3.11E-32
MTF1	SPIN1	0.7408364	3.08E-32
MTF1	ARMC1	0.74118455	2.78E-32
MTF1	TFAM	0.7411904	2.78E-32
MTF1	DCAF7	0.74128297	2.7E-32
MTF1	PHAX	0.74130624	2.68E-32
MTF1	YME1L1	0.74136083	2.64E-32
MTF1	SPTLC1	0.74144015	2.58E-32
MTF1	SLC30A6	0.7415952	2.47E-32
MTF1	SNX4	0.74184831	2.29E-32
MTF1	ELOVL5	0.74189758	2.26E-32
MTF1	VAMP7	0.74198468	2.2E-32
MTF1	SSH1	0.7422052	2.07E-32
MTF1	NAA30	0.74234374	1.98E-32
MTF1	RNF111	0.74253915	1.87E-32
MTF1	RBFOX2	0.74278306	1.74E-32
MTF1	PGGT1B	0.7428122	1.73E-32
MTF1	ABL1	0.74304755	1.61E-32
MTF1	CPSF2	0.74342674	1.44E-32
MTF1	STAU2	0.7436543	1.35E-32
MTF1	OPA1	0.74414869	1.17E-32
MTF1	ZNF200	0.74418256	1.16E-32
MTF1	SELENOT	0.74420433	1.15E-32
MTF1	NAA15	0.74538626	8.1E-33
MTF1	BTBD9	0.74545507	7.93E-33
MTF1	RNF14	0.74572772	7.32E-33
MTF1	TEAD1	0.74575178	7.26E-33
MTF1	YIPF5	0.74580936	7.14E-33
MTF1	RBM18	0.74591529	6.92E-33
MTF1	PCNP	0.74599124	6.77E-33
MTF1	RC3H2	0.74625544	6.25E-33
MTF1	DCUN1D1	0.74638124	6.02E-33
MTF1	MTPN	0.74638437	6.02E-33

MTF1	SELENOF	0.74640713	5.98E-33
MTF1	TCAF1	0.74641872	5.96E-33
MTF1	UBE2D3	0.74652282	5.78E-33
MTF1	ZNF146	0.74654108	5.74E-33
MTF1	ZNF639	0.74713231	4.81E-33
MTF1	SOCS4	0.74774585	4.01E-33
MTF1	ZNF45	0.74899254	2.75E-33
MTF1	VTA1	0.74929686	2.51E-33
MTF1	GCC1	0.74935771	2.47E-33
MTF1	UBQLN1	0.74937788	2.45E-33
MTF1	CTDSPL2	0.74957295	2.31E-33
MTF1	RABL3	0.75001409	2.02E-33
MTF1	ZBTB38	0.75127905	1.38E-33
MTF1	CREB3L2	0.75128778	1.37E-33
MTF1	KAT6A	0.75131634	1.36E-33
MTF1	STAT3	0.7514761	1.3E-33
MTF1	TTC33	0.75153513	1.27E-33
MTF1	EPC2	0.75163199	1.24E-33
MTF1	C11orf58	0.75204987	1.09E-33
MTF1	MSL2	0.75209336	1.07E-33
MTF1	MMGT1	0.75222927	1.03E-33
MTF1	CDC27	0.75266391	9.01E-34
MTF1	STAG2	0.75297012	8.2E-34
MTF1	EAF1	0.75308873	7.91E-34
MTF1	SOS2	0.75337838	7.24E-34
MTF1	WDR44	0.75339734	7.19E-34
MTF1	ZFR	0.75363642	6.68E-34
MTF1	EPS15	0.75367642	6.6E-34
MTF1	PIK3CA	0.75382749	6.3E-34
MTF1	TAOK1	0.75445877	5.18E-34
MTF1	AHCYL1	0.75450447	5.11E-34
MTF1	RAB18	0.75463113	4.91E-34
MTF1	PPP2CA	0.75473737	4.76E-34
MTF1	ZBTB6	0.75482295	4.63E-34
MTF1	NRAS	0.75513924	4.2E-34
MTF1	SMAD5	0.75536617	3.91E-34
MTF1	SPTY2D1	0.75544197	3.82E-34
MTF1	LRRC8D	0.75570145	3.52E-34
MTF1	RLIM	0.75588547	3.33E-34
MTF1	GOLIM4	0.75608837	3.12E-34
MTF1	SETD7	0.75620618	3.01E-34
MTF1	TBC1D5	0.75628866	2.93E-34
MTF1	CNOT6L	0.75667987	2.6E-34
MTF1	GTF2A1	0.75687179	2.45E-34
MTF1	HNRNPK	0.75712093	2.26E-34
MTF1	ATP10D	0.7572483	2.17E-34
MTF1	MATR3	0.75753063	1.99E-34
MTF1	FAM168A	0.75769048	1.89E-34
MTF1	TRRAP	0.75791401	1.76E-34
MTF1	CRTC3	0.75813646	1.64E-34
MTF1	ZFP1	0.75837851	1.52E-34
MTF1	TAF2	0.75898428	1.26E-34
MTF1	PDCL	0.75987009	9.5E-35
MTF1	FCF1	0.76031895	8.23E-35
MTF1	LEPROT	0.76067332	7.35E-35
MTF1	METTL14	0.76174724	5.22E-35
MTF1	BBX	0.76235911	4.29E-35
MTF1	ROCK1	0.76245074	4.16E-35

MTF1	UBE3C	0.76295364	3.54E-35
MTF1	EIF4G3	0.76301852	3.47E-35
MTF1	ASB7	0.76322539	3.24E-35
MTF1	KDM5A	0.76328605	3.18E-35
MTF1	IFT57	0.76436046	2.24E-35
MTF1	FRS2	0.76450621	2.14E-35
MTF1	RAP2A	0.76470413	2.01E-35
MTF1	UBFD1	0.76619321	1.23E-35
MTF1	ARIH1	0.76677999	1.02E-35
MTF1	PDS5A	0.76730182	8.57E-36
MTF1	PTPN11	0.76776981	7.34E-36
MTF1	RNF4	0.76778924	7.3E-36
MTF1	ZFAND3	0.76803518	6.73E-36
MTF1	LSM14A	0.76818273	6.41E-36
MTF1	PIGK	0.76864796	5.49E-36
MTF1	RBM27	0.76879243	5.23E-36
MTF1	BLOC1S6	0.76921646	4.55E-36
MTF1	KIF2A	0.76989522	3.63E-36
MTF1	FYTTD1	0.76991316	3.6E-36
MTF1	DNAJC14	0.76993238	3.58E-36
MTF1	WDR47	0.77001082	3.49E-36
MTF1	DOCK7	0.77001924	3.48E-36
MTF1	EVI5	0.7703155	3.15E-36
MTF1	SMAD1	0.77068888	2.78E-36
MTF1	WAC	0.77146932	2.14E-36
MTF1	ATF1	0.77184549	1.88E-36
MTF1	KPNA1	0.77228621	1.62E-36
MTF1	GPR107	0.77232255	1.6E-36
MTF1	DDX6	0.77261436	1.45E-36
MTF1	SLC35A5	0.7732594	1.17E-36
MTF1	PLEKHA3	0.7733852	1.12E-36
MTF1	ITCH	0.77341543	1.11E-36
MTF1	ACAP2	0.7741192	8.71E-37
MTF1	RELL1	0.7743454	8.06E-37
MTF1	ZYG11B	0.77445287	7.77E-37
MTF1	GNL3L	0.77446702	7.73E-37
MTF1	EIF4E	0.77464124	7.29E-37
MTF1	FOXN3	0.77522269	5.97E-37
MTF1	TAB2	0.77531092	5.79E-37
MTF1	AFF4	0.77559731	5.25E-37
MTF1	SLAIN2	0.77571854	5.04E-37
MTF1	ARL5A	0.7759374	4.67E-37
MTF1	YTHDF3	0.77742354	2.79E-37
MTF1	ARPP19	0.77922105	1.49E-37
MTF1	SENP1	0.77924442	1.48E-37
MTF1	ENOX2	0.77948475	1.36E-37
MTF1	SMAD2	0.78060875	9.16E-38
MTF1	FBXW2	0.78067159	8.96E-38
MTF1	ADNP	0.78180195	6E-38
MTF1	WASF2	0.78277709	4.24E-38
MTF1	SMC1A	0.78283416	4.16E-38
MTF1	MIER1	0.78283635	4.15E-38
MTF1	SRPK2	0.78333691	3.47E-38
MTF1	EXOC5	0.78346971	3.31E-38
MTF1	GTF3C4	0.78376883	2.97E-38
MTF1	TNPO1	0.78379315	2.95E-38
MTF1	PNPLA8	0.78428012	2.48E-38
MTF1	ATP2A2	0.78428524	2.47E-38

MTF1	CAPZA2	0.78554059	1.57E-38
MTF1	C5orf51	0.786186	1.24E-38
MTF1	POFUT1	0.78624137	1.22E-38
MTF1	INIP	0.78625956	1.21E-38
MTF1	NFATC3	0.78700494	9.22E-39
MTF1	CAND1	0.78731567	8.23E-39
MTF1	C16orf72	0.78794648	6.53E-39
MTF1	ERC1	0.7886952	4.96E-39
MTF1	USP9X	0.78913789	4.21E-39
MTF1	WNK1	0.78919713	4.12E-39
MTF1	USP8	0.7897802	3.32E-39
MTF1	UTP11	0.79034901	2.69E-39
MTF1	USP38	0.79065101	2.4E-39
MTF1	RAB21	0.79092822	2.17E-39
MTF1	CTR9	0.79117662	1.98E-39
MTF1	ZNF148	0.79235623	1.27E-39
MTF1	CTBS	0.79279148	1.08E-39
MTF1	DDX3X	0.79306946	9.7E-40
MTF1	MIGA1	0.79421502	6.29E-40
MTF1	BTBD1	0.79440739	5.85E-40
MTF1	ARL13B	0.79507033	4.54E-40
MTF1	WDR82	0.79530276	4.16E-40
MTF1	MFAP3	0.79582096	3.41E-40
MTF1	API5	0.79698297	2.18E-40
MTF1	EFCAB14	0.79874782	1.1E-40
MTF1	ASXL2	0.79908703	9.64E-41
MTF1	G3BP2	0.79967604	7.65E-41
MTF1	EIF4G2	0.79986342	7.11E-41
MTF1	ZMYM4	0.80028369	6.03E-41
MTF1	PPP1R8	0.80043054	5.69E-41
MTF1	FEM1B	0.80055956	5.41E-41
MTF1	LUZP1	0.80143789	3.83E-41
MTF1	CRKL	0.80200127	3.06E-41
MTF1	RYBP	0.80377375	1.51E-41
MTF1	DR1	0.80432723	1.21E-41
MTF1	BAZ1B	0.80438134	1.18E-41
MTF1	MAPK1	0.80445397	1.15E-41
MTF1	PUM1	0.80583138	6.58E-42
MTF1	RNF41	0.80751249	3.32E-42
MTF1	RPS6KA3	0.81095252	7.99E-43
MTF1	USP1	0.8131691	3.14E-43
MTF1	SF3A1	0.81495806	1.47E-43
MTF1	MAP3K2	0.81575134	1.04E-43
MTF1	JAK1	0.81686527	6.45E-44
MTF1	HIPK1	0.81797467	3.98E-44
MTF1	MFSD14A	0.8197665	1.81E-44
MTF1	RBM12	0.82125887	9.36E-45
MTF1	THRAP3	0.82133059	9.07E-45
MTF1	ANKRD13C	0.82223278	6.06E-45
MTF1	RBBP4	0.82642627	9.03E-46
MTF1	RNF11	0.82686726	7.37E-46
MTF1	ZMPSTE24	0.8278801	4.61E-46
MTF1	CSDE1	0.82795723	4.45E-46
MTF1	TMEM167B	0.82838317	3.65E-46
MTF1	KDM4A	0.83224485	5.91E-47
MTF1	TMED5	0.83861638	2.64E-48
MTF1	SF3A3	0.85076646	4.73E-51
MTF1	KPNA6	0.87294707	9.33E-57

MTF1	AKIRIN1	0.87618103	1.12E-57
NFE2L2	NFE2L2	1	0
NFE2L2	CWC22	0.9010374	9.46E-66
NFE2L2	DYNC1I2	0.8872155	5.01E-61
NFE2L2	CAB39	0.87913806	1.53E-58
NFE2L2	ACVR1	0.87682368	7.29E-58
NFE2L2	TRIP12	0.87662094	8.34E-58
NFE2L2	ZC3H15	0.87618119	1.12E-57
NFE2L2	SP3	0.87596657	1.29E-57
NFE2L2	TANK	0.8716992	2.08E-56
NFE2L2	PPIG	0.86864701	1.43E-55
NFE2L2	PICALM	0.86004338	2.56E-53
NFE2L2	MOB1A	0.85868386	5.63E-53
NFE2L2	DDX18	0.85327712	1.19E-51
NFE2L2	RAB6A	0.85191827	2.52E-51
NFE2L2	WDR26	0.84998006	7.24E-51
NFE2L2	DUSP11	0.84830823	1.78E-50
NFE2L2	BMPR2	0.84684107	3.87E-50
NFE2L2	CTTNBP2NL	0.84221554	4.28E-49
NFE2L2	C16orf72	0.84095248	8.15E-49
NFE2L2	MCMBP	0.84093536	8.22E-49
NFE2L2	NCKAP1	0.84089543	8.38E-49
NFE2L2	MED17	0.83995879	1.35E-48
NFE2L2	PPP3R1	0.8384492	2.86E-48
NFE2L2	ACTR2	0.83435994	2.13E-47
NFE2L2	IWS1	0.83402762	2.51E-47
NFE2L2	AGPS	0.83392818	2.63E-47
NFE2L2	SRSF3	0.83175008	7.48E-47
NFE2L2	DNAJA2	0.83168677	7.71E-47
NFE2L2	ABI1	0.83143635	8.68E-47
NFE2L2	WDFY1	0.83103494	1.05E-46
NFE2L2	ACTR3	0.83072521	1.22E-46
NFE2L2	RNF168	0.8299876	1.72E-46
NFE2L2	PRPF40A	0.82808148	4.2E-46
NFE2L2	TBL1XR1	0.82675124	7.78E-46
NFE2L2	PCYT1A	0.82613178	1.03E-45
NFE2L2	GNG12	0.82609902	1.05E-45
NFE2L2	UGP2	0.82548496	1.39E-45
NFE2L2	AGFG1	0.82471349	1.98E-45
NFE2L2	PPP1R2	0.82449245	2.19E-45
NFE2L2	ZFP91	0.82384164	2.94E-45
NFE2L2	PTPN9	0.8232854	3.78E-45
NFE2L2	VPS26A	0.82289694	4.5E-45
NFE2L2	SNX6	0.82065865	1.22E-44
NFE2L2	SNAP23	0.82061257	1.25E-44
NFE2L2	ZNF207	0.82035565	1.4E-44
NFE2L2	ORC4	0.81950325	2.04E-44
NFE2L2	PAK2	0.81928418	2.24E-44
NFE2L2	KLHL20	0.81915058	2.38E-44
NFE2L2	OGFOD1	0.81788176	4.15E-44
NFE2L2	OXR1	0.81776271	4.37E-44
NFE2L2	RHBDD1	0.81620086	8.59E-44
NFE2L2	EFCAB14	0.81611565	8.92E-44
NFE2L2	PTPN12	0.81605082	9.17E-44
NFE2L2	PLEKHB2	0.81485847	1.53E-43
NFE2L2	IST1	0.8144807	1.8E-43
NFE2L2	NCK1	0.81422787	2E-43
NFE2L2	CAPRIN1	0.81417146	2.05E-43

NFE2L2	PDLIM5	0.81408273	2.13E-43
NFE2L2	HNRNPF	0.81392485	2.28E-43
NFE2L2	IFNAR1	0.81377265	2.43E-43
NFE2L2	SSB	0.81374946	2.46E-43
NFE2L2	CDC73	0.81331997	2.95E-43
NFE2L2	ETV3	0.8128493	3.6E-43
NFE2L2	MAP3K2	0.8120535	5.03E-43
NFE2L2	COQ10B	0.81193087	5.3E-43
NFE2L2	CTCF	0.81133737	6.8E-43
NFE2L2	SMNDC1	0.81115838	7.33E-43
NFE2L2	HNRNPK	0.81095618	7.97E-43
NFE2L2	TMEM127	0.81053639	9.5E-43
NFE2L2	RPE	0.81022833	1.08E-42
NFE2L2	RBM7	0.81014847	1.12E-42
NFE2L2	COMMD2	0.8101075	1.14E-42
NFE2L2	SDHD	0.80991789	1.23E-42
NFE2L2	RBMS1	0.8093292	1.57E-42
NFE2L2	KBTBD2	0.80914508	1.69E-42
NFE2L2	AHR	0.80892741	1.85E-42
NFE2L2	YME1L1	0.80891638	1.86E-42
NFE2L2	B3GNT2	0.80840873	2.3E-42
NFE2L2	ELF1	0.80811993	2.58E-42
NFE2L2	KDM2A	0.80803594	2.68E-42
NFE2L2	TOB2	0.80800552	2.71E-42
NFE2L2	MIER1	0.80787029	2.86E-42
NFE2L2	TSN	0.8077778	2.97E-42
NFE2L2	TLK1	0.80769019	3.08E-42
NFE2L2	SPTY2D1	0.80753941	3.28E-42
NFE2L2	LEPROT	0.80753748	3.28E-42
NFE2L2	NPAT	0.80746801	3.38E-42
NFE2L2	PGM2	0.80696866	4.14E-42
NFE2L2	AIDA	0.80676598	4.5E-42
NFE2L2	DCUN1D1	0.8067119	4.6E-42
NFE2L2	CDC42SE1	0.8064509	5.12E-42
NFE2L2	MFSD14B	0.80559604	7.24E-42
NFE2L2	GCC2	0.80546384	7.64E-42
NFE2L2	NRBF2	0.80514648	8.69E-42
NFE2L2	LAPTM4A	0.80500155	9.21E-42
NFE2L2	PATL1	0.80487986	9.67E-42
NFE2L2	REST	0.8046556	1.06E-41
NFE2L2	TMEM87B	0.80445171	1.15E-41
NFE2L2	FBXO28	0.80439493	1.18E-41
NFE2L2	VPS35	0.80435224	1.2E-41
NFE2L2	PDCD10	0.80430642	1.22E-41
NFE2L2	SUSD6	0.80403565	1.36E-41
NFE2L2	MTMR2	0.8040038	1.38E-41
NFE2L2	ZDHHC5	0.80376019	1.52E-41
NFE2L2	RAB3GAP1	0.80370354	1.55E-41
NFE2L2	DDX19A	0.80341214	1.74E-41
NFE2L2	SP1	0.80287522	2.16E-41
NFE2L2	ARHGAP21	0.80259366	2.42E-41
NFE2L2	ATF2	0.80254701	2.46E-41
NFE2L2	DESI2	0.80245257	2.56E-41
NFE2L2	HNRNPU	0.80221751	2.81E-41
NFE2L2	ERBIN	0.80202635	3.03E-41
NFE2L2	RALB	0.80172181	3.42E-41
NFE2L2	RNF13	0.8017109	3.43E-41
NFE2L2	STRN	0.80139473	3.89E-41

NFE2L2	TP53BP2	0.80104765	4.46E-41
NFE2L2	JRKL	0.80101308	4.52E-41
NFE2L2	AHNAK	0.80066534	5.19E-41
NFE2L2	FBXO38	0.80046044	5.63E-41
NFE2L2	CASP8	0.79999333	6.76E-41
NFE2L2	JAK1	0.79989368	7.03E-41
NFE2L2	NIPA2	0.7982154	1.35E-40
NFE2L2	DNAJC13	0.79790363	1.53E-40
NFE2L2	MAT2B	0.79781691	1.58E-40
NFE2L2	PRRC2C	0.79766987	1.67E-40
NFE2L2	THRAP3	0.79653514	2.59E-40
NFE2L2	ECD	0.79632738	2.81E-40
NFE2L2	METTL9	0.79625749	2.88E-40
NFE2L2	ATL3	0.79621685	2.93E-40
NFE2L2	ARL6IP6	0.79618949	2.96E-40
NFE2L2	CBFB	0.79603251	3.14E-40
NFE2L2	TRA2B	0.79587984	3.33E-40
NFE2L2	UVRAG	0.79587666	3.34E-40
NFE2L2	STX6	0.79537333	4.04E-40
NFE2L2	MAPK14	0.79523197	4.27E-40
NFE2L2	VCL	0.79510555	4.48E-40
NFE2L2	SLC25A24	0.79407814	6.62E-40
NFE2L2	NAB1	0.79405675	6.68E-40
NFE2L2	ADAM17	0.79360986	7.91E-40
NFE2L2	SKAP2	0.79358582	7.98E-40
NFE2L2	NUFIP2	0.79346927	8.34E-40
NFE2L2	PIK3CA	0.79343904	8.44E-40
NFE2L2	MOB4	0.79326266	9.02E-40
NFE2L2	SESTD1	0.79300522	9.94E-40
NFE2L2	ATP11B	0.79297298	1.01E-39
NFE2L2	PCBP1	0.79228798	1.3E-39
NFE2L2	TMEM185B	0.79214717	1.37E-39
NFE2L2	CNOT8	0.79212736	1.38E-39
NFE2L2	MPZL1	0.79210074	1.4E-39
NFE2L2	IRF2	0.79192735	1.49E-39
NFE2L2	CYFIP1	0.79180979	1.56E-39
NFE2L2	RTF1	0.79147715	1.77E-39
NFE2L2	DR1	0.79142578	1.8E-39
NFE2L2	WAC	0.79128202	1.9E-39
NFE2L2	ARL5A	0.79065363	2.4E-39
NFE2L2	RIT1	0.79058253	2.46E-39
NFE2L2	TOR1AIP1	0.79038272	2.66E-39
NFE2L2	ADAR	0.79027678	2.76E-39
NFE2L2	SLC30A6	0.78951058	3.67E-39
NFE2L2	NCOA4	0.78937531	3.86E-39
NFE2L2	GORASP2	0.78932548	3.93E-39
NFE2L2	CCDC90B	0.78919184	4.13E-39
NFE2L2	ELK4	0.7891628	4.17E-39
NFE2L2	TMOD3	0.78899906	4.43E-39
NFE2L2	RAB10	0.78847392	5.38E-39
NFE2L2	KPNA4	0.78827942	5.78E-39
NFE2L2	RSF1	0.78819798	5.95E-39
NFE2L2	RAB9A	0.78815642	6.05E-39
NFE2L2	RO60	0.78740447	7.97E-39
NFE2L2	ARIH1	0.78729134	8.3E-39
NFE2L2	NDUFS1	0.78704317	9.09E-39
NFE2L2	SNTB2	0.78667952	1.04E-38
NFE2L2	EXOC6B	0.78667033	1.04E-38

NFE2L2	XRCC5	0.78621347	1.23E-38
NFE2L2	ATP2C1	0.78618738	1.24E-38
NFE2L2	ABRAXAS2	0.78603789	1.31E-38
NFE2L2	YAP1	0.78514598	1.81E-38
NFE2L2	NUMB	0.78506505	1.87E-38
NFE2L2	PPP4R3B	0.78501362	1.9E-38
NFE2L2	RNF111	0.78464231	2.17E-38
NFE2L2	SPOPL	0.78458132	2.22E-38
NFE2L2	CDC42	0.78455239	2.24E-38
NFE2L2	TGOLN2	0.7844429	2.33E-38
NFE2L2	CAPZA1	0.78437021	2.4E-38
NFE2L2	RAB1A	0.78395761	2.78E-38
NFE2L2	CNOT9	0.78359109	3.17E-38
NFE2L2	YY1	0.78351344	3.26E-38
NFE2L2	PLS3	0.78348999	3.29E-38
NFE2L2	CWF19L2	0.78336739	3.44E-38
NFE2L2	ARPC2	0.78325877	3.57E-38
NFE2L2	DOCK1	0.78216315	5.28E-38
NFE2L2	KLF6	0.78209856	5.4E-38
NFE2L2	RBM43	0.78209111	5.42E-38
NFE2L2	WDR44	0.78194461	5.71E-38
NFE2L2	UEVLD	0.78187833	5.84E-38
NFE2L2	PPP1CB	0.7815748	6.51E-38
NFE2L2	SEC23IP	0.78142817	6.86E-38
NFE2L2	QTRT2	0.78140868	6.9E-38
NFE2L2	PUM1	0.78097251	8.06E-38
NFE2L2	EIF4G2	0.78088785	8.3E-38
NFE2L2	EPS8	0.78046532	9.64E-38
NFE2L2	IQGAP1	0.78026173	1.04E-37
NFE2L2	PSEN1	0.78016816	1.07E-37
NFE2L2	SIAH1	0.77989309	1.18E-37
NFE2L2	BACH1	0.77987375	1.19E-37
NFE2L2	HMGXB4	0.77961728	1.3E-37
NFE2L2	PRPF18	0.77941855	1.39E-37
NFE2L2	JMJD1C	0.77913453	1.54E-37
NFE2L2	HNRNPLL	0.77912335	1.54E-37
NFE2L2	ISG20L2	0.77897769	1.63E-37
NFE2L2	PITPNB	0.77879795	1.73E-37
NFE2L2	CAMSAP2	0.77873845	1.77E-37
NFE2L2	OSBPL11	0.77861364	1.85E-37
NFE2L2	SLAIN2	0.77856303	1.88E-37
NFE2L2	TARDBP	0.77855603	1.88E-37
NFE2L2	CPSF6	0.7782883	2.07E-37
NFE2L2	SH3D19	0.77818336	2.14E-37
NFE2L2	SNRNP27	0.77813966	2.18E-37
NFE2L2	RNF2	0.77800796	2.28E-37
NFE2L2	SHOC2	0.7779271	2.34E-37
NFE2L2	ZNFX1	0.7777674	2.48E-37
NFE2L2	NEK7	0.77731721	2.9E-37
NFE2L2	NFYA	0.77731195	2.9E-37
NFE2L2	NCBP2	0.77700642	3.23E-37
NFE2L2	ITGAV	0.77617642	4.3E-37
NFE2L2	RAB18	0.77612099	4.38E-37
NFE2L2	ZC3H11A	0.77593321	4.68E-37
NFE2L2	TMEM43	0.7759314	4.68E-37
NFE2L2	WWP1	0.77592041	4.7E-37
NFE2L2	ROCK1	0.77554154	5.35E-37
NFE2L2	SDE2	0.77544079	5.54E-37

NFE2L2	RP2	0.77530506	5.8E-37
NFE2L2	BTBD10	0.77517965	6.06E-37
NFE2L2	TMEM87A	0.77496297	6.53E-37
NFE2L2	PANX1	0.7748759	6.72E-37
NFE2L2	KLF3	0.77485307	6.78E-37
NFE2L2	TAB2	0.77476467	6.99E-37
NFE2L2	SNX9	0.77464087	7.29E-37
NFE2L2	SPRED2	0.77451891	7.6E-37
NFE2L2	LRRC57	0.77434801	8.06E-37
NFE2L2	DLG1	0.77432981	8.11E-37
NFE2L2	CAST	0.77373572	9.93E-37
NFE2L2	UBE2E3	0.77369942	1E-36
NFE2L2	YWHAB	0.77362161	1.03E-36
NFE2L2	KCTD9	0.77359775	1.04E-36
NFE2L2	RSU1	0.77342975	1.1E-36
NFE2L2	SRFBP1	0.77332295	1.14E-36
NFE2L2	PTBP3	0.77331303	1.15E-36
NFE2L2	HMGN4	0.77329657	1.15E-36
NFE2L2	CSGALNACT2	0.77272073	1.4E-36
NFE2L2	UBA3	0.77245774	1.53E-36
NFE2L2	ACBD3	0.77235917	1.58E-36
NFE2L2	C3orf38	0.77222326	1.66E-36
NFE2L2	PPP1R15B	0.77207575	1.74E-36
NFE2L2	TAF1B	0.77204257	1.76E-36
NFE2L2	TOP1	0.77183071	1.89E-36
NFE2L2	HNRNPR	0.77134556	2.23E-36
NFE2L2	TCF12	0.77111297	2.41E-36
NFE2L2	UGCG	0.77105893	2.45E-36
NFE2L2	COPS2	0.77086146	2.62E-36
NFE2L2	ACAP2	0.77085149	2.63E-36
NFE2L2	RAPH1	0.77078896	2.69E-36
NFE2L2	PIP5K1A	0.77073593	2.74E-36
NFE2L2	METAP1	0.77069406	2.77E-36
NFE2L2	MPHOSPH10	0.77058144	2.88E-36
NFE2L2	GTF2E1	0.77057982	2.88E-36
NFE2L2	LUZP1	0.7705221	2.94E-36
NFE2L2	AHCTF1	0.7702801	3.19E-36
NFE2L2	NAA50	0.77014141	3.34E-36
NFE2L2	DEDD	0.76962181	3.97E-36
NFE2L2	REEP3	0.76956657	4.05E-36
NFE2L2	ANO6	0.76944898	4.21E-36
NFE2L2	ZNHIT6	0.76925001	4.5E-36
NFE2L2	FAM118B	0.769215	4.55E-36
NFE2L2	CIR1	0.76920584	4.56E-36
NFE2L2	STAT3	0.76913614	4.67E-36
NFE2L2	OSTF1	0.76887723	5.09E-36
NFE2L2	PELI1	0.76864651	5.49E-36
NFE2L2	RAB7A	0.7686365	5.51E-36
NFE2L2	SHC1	0.76859543	5.59E-36
NFE2L2	KDM5B	0.76854011	5.69E-36
NFE2L2	USP9X	0.76851123	5.75E-36
NFE2L2	PDP1	0.76847723	5.81E-36
NFE2L2	MCL1	0.76847627	5.81E-36
NFE2L2	HNRNPH2	0.76819012	6.39E-36
NFE2L2	STX7	0.76804817	6.7E-36
NFE2L2	SWAP70	0.76802771	6.74E-36
NFE2L2	PARN	0.7680195	6.76E-36
NFE2L2	PNRC2	0.76801628	6.77E-36

NFE2L2	POLR2D	0.7679543	6.91E-36
NFE2L2	NFKB1	0.7677026	7.51E-36
NFE2L2	POGK	0.76753779	7.93E-36
NFE2L2	VAMP3	0.76746512	8.12E-36
NFE2L2	CFAP97	0.76724868	8.72E-36
NFE2L2	PAFAH1B2	0.76720736	8.84E-36
NFE2L2	UBE2D3	0.76686904	9.88E-36
NFE2L2	KHDRBS1	0.76684321	9.97E-36
NFE2L2	PLEKHA3	0.76664657	1.06E-35
NFE2L2	CAP1	0.76659352	1.08E-35
NFE2L2	STAU1	0.76653313	1.1E-35
NFE2L2	TMEM170A	0.76650517	1.11E-35
NFE2L2	HPS5	0.76640892	1.15E-35
NFE2L2	ITGB1	0.76637063	1.16E-35
NFE2L2	JAG1	0.76630769	1.19E-35
NFE2L2	CAPZA2	0.7662488	1.21E-35
NFE2L2	SAP130	0.76611301	1.27E-35
NFE2L2	ATMIN	0.76602071	1.31E-35
NFE2L2	EHD4	0.76571138	1.44E-35
NFE2L2	HAT1	0.7654249	1.59E-35
NFE2L2	TMCC1	0.76525698	1.68E-35
NFE2L2	PEX13	0.7649409	1.86E-35
NFE2L2	DDX3X	0.76477619	1.96E-35
NFE2L2	NRAS	0.76475532	1.97E-35
NFE2L2	CHMP2B	0.7645108	2.14E-35
NFE2L2	PDCD6IP	0.76404705	2.48E-35
NFE2L2	CREB1	0.76394358	2.57E-35
NFE2L2	ROCK2	0.76364725	2.83E-35
NFE2L2	KLF7	0.76351429	2.95E-35
NFE2L2	SH3GLB1	0.76332783	3.14E-35
NFE2L2	NEDD4	0.76310027	3.38E-35
NFE2L2	RAB3GAP2	0.76287573	3.63E-35
NFE2L2	SDCBP	0.7628603	3.65E-35
NFE2L2	ELK3	0.76282731	3.69E-35
NFE2L2	HAUS2	0.76246398	4.14E-35
NFE2L2	SKIL	0.76241102	4.21E-35
NFE2L2	KPNA1	0.76219301	4.52E-35
NFE2L2	ZNF639	0.76219129	4.52E-35
NFE2L2	CUL2	0.76196402	4.87E-35
NFE2L2	UBE3A	0.76178517	5.15E-35
NFE2L2	KATNBL1	0.7614343	5.77E-35
NFE2L2	PARP4	0.76141912	5.79E-35
NFE2L2	DDX21	0.76140797	5.82E-35
NFE2L2	RRAGC	0.76120868	6.2E-35
NFE2L2	ADAM10	0.76111035	6.4E-35
NFE2L2	SPRED1	0.76041046	8E-35
NFE2L2	LPP	0.76032767	8.21E-35
NFE2L2	PTEN	0.76023204	8.47E-35
NFE2L2	ATXN1	0.76020219	8.55E-35
NFE2L2	STAM	0.76014798	8.69E-35
NFE2L2	RRN3	0.75985191	9.55E-35
NFE2L2	SLC35A5	0.75982809	9.63E-35
NFE2L2	SIN3A	0.75966731	1.01E-34
NFE2L2	TRAF3IP1	0.75942743	1.09E-34
NFE2L2	MBNL1	0.75922188	1.17E-34
NFE2L2	SERBP1	0.75920251	1.17E-34
NFE2L2	KCTD21	0.75894849	1.27E-34
NFE2L2	GFM1	0.75894139	1.27E-34

NFE2L2	EXT2	0.75891903	1.28E-34
NFE2L2	WDR82	0.75887957	1.3E-34
NFE2L2	LITAF	0.75886195	1.31E-34
NFE2L2	C11orf58	0.75855095	1.44E-34
NFE2L2	CRIM1	0.75841602	1.51E-34
NFE2L2	VEZT	0.75832668	1.55E-34
NFE2L2	FNDC3B	0.75832179	1.55E-34
NFE2L2	ATP13A3	0.7582638	1.58E-34
NFE2L2	FEZ2	0.7581063	1.66E-34
NFE2L2	COPS8	0.75803116	1.7E-34
NFE2L2	GPBP1L1	0.75787507	1.78E-34
NFE2L2	CSNK1G3	0.75778454	1.84E-34
NFE2L2	RAP1A	0.7572353	2.18E-34
NFE2L2	VTI1A	0.75723026	2.19E-34
NFE2L2	ITPRIPL2	0.75720174	2.21E-34
NFE2L2	B4GALT5	0.75719795	2.21E-34
NFE2L2	OTUD7B	0.75669761	2.58E-34
NFE2L2	SNX27	0.75653905	2.71E-34
NFE2L2	EFR3A	0.75645473	2.79E-34
NFE2L2	JOSD1	0.7563419	2.89E-34
NFE2L2	ANKRD50	0.75634001	2.89E-34
NFE2L2	MINDY2	0.75606143	3.15E-34
NFE2L2	SOCS5	0.75600379	3.21E-34
NFE2L2	STX12	0.75584002	3.38E-34
NFE2L2	SPIN1	0.75572367	3.5E-34
NFE2L2	TRMT1L	0.75556559	3.68E-34
NFE2L2	DDX6	0.75535512	3.93E-34
NFE2L2	FOXN2	0.75533611	3.95E-34
NFE2L2	TRIM32	0.75530924	3.98E-34
NFE2L2	PPP2R5E	0.75501306	4.37E-34
NFE2L2	PPP2R2A	0.75495169	4.45E-34
NFE2L2	GSPT1	0.75489157	4.53E-34
NFE2L2	MBD5	0.75473962	4.75E-34
NFE2L2	CTNNB1	0.75473557	4.76E-34
NFE2L2	EPC2	0.75469697	4.82E-34
NFE2L2	VRK2	0.75465101	4.88E-34
NFE2L2	IARS2	0.75460347	4.96E-34
NFE2L2	WAPL	0.75448309	5.14E-34
NFE2L2	USP8	0.75443597	5.22E-34
NFE2L2	SRGAP2B	0.7544337	5.22E-34
NFE2L2	CAMKK2	0.75431657	5.42E-34
NFE2L2	ITSN1	0.75407896	5.83E-34
NFE2L2	YTHDF2	0.75406124	5.86E-34
NFE2L2	RAB5C	0.75392265	6.12E-34
NFE2L2	TIPARP	0.75389932	6.16E-34
NFE2L2	FBXW2	0.75388742	6.19E-34
NFE2L2	CXorf38	0.75380657	6.34E-34
NFE2L2	GMEB1	0.75376119	6.43E-34
NFE2L2	BCL9L	0.75339961	7.19E-34
NFE2L2	MTPN	0.7531952	7.66E-34
NFE2L2	TAF5L	0.75268169	8.96E-34
NFE2L2	NCL	0.75254288	9.35E-34
NFE2L2	MAML2	0.75233454	9.97E-34
NFE2L2	VTA1	0.75206505	1.08E-33
NFE2L2	TSC22D2	0.751795	1.18E-33
NFE2L2	CDV3	0.75153514	1.27E-33
NFE2L2	UBTD2	0.75147896	1.3E-33
NFE2L2	ZNF217	0.75142482	1.32E-33

NFE2L2	MAP4K4	0.75120402	1.41E-33
NFE2L2	USP10	0.75109142	1.46E-33
NFE2L2	ARPC5	0.75095156	1.52E-33
NFE2L2	TMTC2	0.75090206	1.54E-33
NFE2L2	LRRFIP1	0.75079161	1.6E-33
NFE2L2	SNX1	0.7507833	1.6E-33
NFE2L2	PPP1R8	0.75074493	1.62E-33
NFE2L2	PPIL4	0.75071791	1.63E-33
NFE2L2	ALS2	0.75064935	1.67E-33
NFE2L2	RAB5B	0.75030126	1.85E-33
NFE2L2	DOCK9	0.75015427	1.94E-33
NFE2L2	CCSER2	0.75014028	1.95E-33
NFE2L2	MRPL19	0.75010597	1.97E-33
NFE2L2	UXS1	0.7501044	1.97E-33
NFE2L2	BZW1	0.75001903	2.02E-33
NFE2L2	CPNE8	0.74998442	2.04E-33
NFE2L2	ZNF143	0.74989753	2.09E-33
NFE2L2	NT5C2	0.74939706	2.44E-33
NFE2L2	ASXL2	0.74923509	2.56E-33
NFE2L2	RBM12	0.74921266	2.58E-33
NFE2L2	RAB8B	0.74871355	3E-33
NFE2L2	DYRK1A	0.74856727	3.13E-33
NFE2L2	DLAT	0.74820689	3.49E-33
NFE2L2	FASTKD2	0.74810912	3.59E-33
NFE2L2	CBL	0.74810843	3.59E-33
NFE2L2	SETD7	0.74792593	3.8E-33
NFE2L2	SYNCRIP	0.74788587	3.84E-33
NFE2L2	SPTLC1	0.74784085	3.89E-33
NFE2L2	CSNK1A1	0.74767537	4.09E-33
NFE2L2	FBXO34	0.74756532	4.23E-33
NFE2L2	SRSF7	0.74755302	4.24E-33
NFE2L2	ETF1	0.74732151	4.55E-33
NFE2L2	NIP7	0.7469843	5.03E-33
NFE2L2	TGFBR2	0.74698353	5.03E-33
NFE2L2	GNB1	0.74689286	5.17E-33
NFE2L2	GPD2	0.7467369	5.42E-33
NFE2L2	GNAQ	0.74665437	5.55E-33
NFE2L2	CBLB	0.74634517	6.09E-33
NFE2L2	YPEL5	0.74624166	6.28E-33
NFE2L2	LRCH1	0.74593237	6.88E-33
NFE2L2	NUP98	0.74592233	6.91E-33
NFE2L2	RAB21	0.74591228	6.93E-33
NFE2L2	ANP32E	0.74586247	7.03E-33
NFE2L2	FCHO2	0.74584101	7.07E-33
NFE2L2	CRLF3	0.74575869	7.25E-33
NFE2L2	MFSD14A	0.74572361	7.33E-33
NFE2L2	RAB14	0.74546585	7.91E-33
NFE2L2	TPM3	0.74544927	7.95E-33
NFE2L2	ZBTB2	0.74504959	8.95E-33
NFE2L2	ADO	0.74474348	9.79E-33
NFE2L2	MYH9	0.744504	1.05E-32
NFE2L2	KIAA1217	0.74434756	1.1E-32
NFE2L2	EXT1	0.74434033	1.1E-32
NFE2L2	MFSD1	0.74414869	1.17E-32
NFE2L2	KRAS	0.74391235	1.25E-32
NFE2L2	PEA15	0.743751	1.31E-32
NFE2L2	ARMC8	0.74364614	1.35E-32
NFE2L2	FOSL2	0.74338245	1.46E-32

NFE2L2	ELAVL1	0.74329961	1.5E-32
NFE2L2	ZFAND3	0.74326732	1.51E-32
NFE2L2	GNAI3	0.74274612	1.76E-32
NFE2L2	PTPRE	0.74273521	1.77E-32
NFE2L2	ACVR2A	0.74266386	1.81E-32
NFE2L2	KIF5B	0.74264589	1.82E-32
NFE2L2	CNOT6	0.74260756	1.84E-32
NFE2L2	SUMO1	0.74252854	1.88E-32
NFE2L2	GPR137B	0.74242593	1.94E-32
NFE2L2	SLC12A6	0.74228345	2.02E-32
NFE2L2	EPS15	0.74219848	2.07E-32
NFE2L2	CTDSPL2	0.74207507	2.15E-32
NFE2L2	TOX4	0.74195749	2.22E-32
NFE2L2	SERINC5	0.74185395	2.29E-32
NFE2L2	TXNDC9	0.74181212	2.32E-32
NFE2L2	HACD2	0.74159301	2.47E-32
NFE2L2	ARL8B	0.74145918	2.57E-32
NFE2L2	EIF2AK2	0.7411869	2.78E-32
NFE2L2	MAPK1IP1L	0.7411339	2.82E-32
NFE2L2	C16orf87	0.74098589	2.95E-32
NFE2L2	SLC30A1	0.74092639	3E-32
NFE2L2	DEK	0.7408711	3.05E-32
NFE2L2	CTDSP1	0.74081226	3.1E-32
NFE2L2	PPP3CA	0.7407591	3.15E-32
NFE2L2	XRN2	0.74052524	3.37E-32
NFE2L2	PPM1B	0.74051882	3.37E-32
NFE2L2	ASB7	0.74049415	3.4E-32
NFE2L2	CHD9	0.74046481	3.43E-32
NFE2L2	ZNF410	0.74028179	3.61E-32
NFE2L2	TRAK2	0.74027577	3.62E-32
NFE2L2	ADD3	0.74005846	3.85E-32
NFE2L2	UBE2A	0.74003954	3.88E-32
NFE2L2	IFNGR1	0.73998609	3.94E-32
NFE2L2	SRSF1	0.7399391	3.99E-32
NFE2L2	MTA2	0.73991148	4.02E-32
NFE2L2	MATR3	0.73967784	4.3E-32
NFE2L2	ADAM9	0.73967632	4.3E-32
NFE2L2	ARL2BP	0.73961487	4.38E-32
NFE2L2	C1D	0.73952202	4.5E-32
NFE2L2	CSDE1	0.73901816	5.2E-32
NFE2L2	PNO1	0.73897505	5.27E-32
NFE2L2	EHBP1	0.73894986	5.31E-32
NFE2L2	ADH5	0.73894043	5.32E-32
NFE2L2	PUM2	0.73885176	5.46E-32
NFE2L2	LIMS1	0.73873382	5.65E-32
NFE2L2	UBE2W	0.73866233	5.77E-32
NFE2L2	RASAL2	0.73864743	5.79E-32
NFE2L2	ZBTB38	0.73862308	5.83E-32
NFE2L2	TIPRL	0.73856917	5.92E-32
NFE2L2	G3BP1	0.73855987	5.94E-32
NFE2L2	DOCK7	0.73854917	5.96E-32
NFE2L2	TMEM126B	0.73842864	6.17E-32
NFE2L2	ARF6	0.73815975	6.66E-32
NFE2L2	PCNP	0.73805433	6.86E-32
NFE2L2	RRP15	0.73791411	7.15E-32
NFE2L2	ATL2	0.73783604	7.31E-32
NFE2L2	DNAJC10	0.73778843	7.41E-32
NFE2L2	ATG16L1	0.73771626	7.56E-32

NFE2L2	RNASEL	0.7376695	7.66E-32
NFE2L2	DCTN5	0.7376626	7.68E-32
NFE2L2	C5orf51	0.73761791	7.78E-32
NFE2L2	GTDC1	0.73760496	7.81E-32
NFE2L2	RHOA	0.73759698	7.83E-32
NFE2L2	ESYT2	0.73758026	7.86E-32
NFE2L2	TAOK3	0.73735025	8.4E-32
NFE2L2	EIF4G3	0.73703159	9.2E-32
NFE2L2	ARHGEF12	0.73688536	9.59E-32
NFE2L2	RECQL	0.73682065	9.77E-32
NFE2L2	SMAD2	0.73676637	9.92E-32
NFE2L2	FCF1	0.73666806	1.02E-31
NFE2L2	GNA13	0.73658591	1.04E-31
NFE2L2	CCDC6	0.73658574	1.04E-31
NFE2L2	ZC3H13	0.73655967	1.05E-31
NFE2L2	RAB5A	0.73653449	1.06E-31
NFE2L2	EIF4E	0.73652758	1.06E-31
NFE2L2	DIPK2A	0.73637999	1.11E-31
NFE2L2	CYBRD1	0.73635734	1.11E-31
NFE2L2	ZNF184	0.73631485	1.13E-31
NFE2L2	ARHGAP42	0.7361584	1.18E-31
NFE2L2	COPA	0.73610514	1.2E-31
NFE2L2	MAP1LC3B	0.73610066	1.2E-31
NFE2L2	CTBP2	0.73605413	1.21E-31
NFE2L2	TXNRD1	0.73569427	1.35E-31
NFE2L2	PHAX	0.73566081	1.36E-31
NFE2L2	TPM4	0.73542952	1.45E-31
NFE2L2	WASF2	0.73538424	1.47E-31
NFE2L2	DENND6A	0.73507187	1.6E-31
NFE2L2	TAF12	0.73490637	1.68E-31
NFE2L2	SELENOT	0.73490508	1.68E-31
NFE2L2	LY75	0.73487331	1.7E-31
NFE2L2	DIS3	0.73456011	1.85E-31
NFE2L2	ITCH	0.73455568	1.86E-31
NFE2L2	FBXO42	0.73422561	2.04E-31
NFE2L2	VGLL4	0.73421237	2.05E-31
NFE2L2	TMEM123	0.73410613	2.11E-31
NFE2L2	ZNF146	0.73408289	2.12E-31
NFE2L2	HIPK1	0.73407311	2.13E-31
NFE2L2	RLIM	0.73394773	2.2E-31
NFE2L2	RAB8A	0.73367974	2.38E-31
NFE2L2	OXSR1	0.73367864	2.38E-31
NFE2L2	INO80	0.73353481	2.47E-31
NFE2L2	SH3BGRL	0.73325352	2.68E-31
NFE2L2	TFE3	0.73303694	2.85E-31
NFE2L2	PPP4R2	0.73290928	2.95E-31
NFE2L2	CORO1C	0.73285258	3E-31
NFE2L2	STAMBP	0.732811	3.03E-31
NFE2L2	SDHC	0.73274691	3.09E-31
NFE2L2	EXOC1	0.73273905	3.09E-31
NFE2L2	ATG3	0.7326362	3.18E-31
NFE2L2	STK3	0.73261235	3.2E-31
NFE2L2	RBBP5	0.7325728	3.24E-31
NFE2L2	RTN4	0.73246023	3.34E-31
NFE2L2	UBLCP1	0.73241478	3.39E-31
NFE2L2	NAA15	0.73234457	3.45E-31
NFE2L2	MYD88	0.73227221	3.52E-31
NFE2L2	DDX19B	0.7322585	3.54E-31

NFE2L2	RNF11	0.73219087	3.61E-31
NFE2L2	RBBP4	0.73213231	3.66E-31
NFE2L2	MEF2A	0.73209216	3.71E-31
NFE2L2	CFLAR	0.73203026	3.77E-31
NFE2L2	SUMO3	0.73198522	3.82E-31
NFE2L2	PLSCR1	0.73175294	4.07E-31
NFE2L2	BBX	0.73164057	4.2E-31
NFE2L2	FXR1	0.73131053	4.61E-31
NFE2L2	INSIG2	0.73129024	4.63E-31
NFE2L2	ATP6V1A	0.73127336	4.65E-31
NFE2L2	LNPK	0.73107774	4.91E-31
NFE2L2	APLP2	0.73098045	5.05E-31
NFE2L2	SNX7	0.73088263	5.19E-31
NFE2L2	UHMK1	0.73070434	5.45E-31
NFE2L2	ARL13B	0.73039104	5.95E-31
NFE2L2	EIF1AD	0.73012382	6.4E-31
NFE2L2	SKI	0.72977289	7.05E-31
NFE2L2	FAM91A1	0.72955334	7.49E-31
NFE2L2	RFX5	0.72953001	7.54E-31
NFE2L2	MYOF	0.72947223	7.66E-31
NFE2L2	SPTBN1	0.72944503	7.72E-31
NFE2L2	STK38	0.72927994	8.08E-31
NFE2L2	SET	0.72894532	8.86E-31
NFE2L2	PPP2CA	0.72882677	9.16E-31
NFE2L2	PARG	0.72879894	9.23E-31
NFE2L2	TES	0.72879556	9.23E-31
NFE2L2	MR1	0.72871357	9.44E-31
NFE2L2	ZMYM4	0.72868561	9.52E-31
NFE2L2	PSME3	0.72865922	9.59E-31
NFE2L2	STK38L	0.728634	9.65E-31
NFE2L2	ZPR1	0.72861967	9.69E-31
NFE2L2	FGFR1OP2	0.72858886	9.77E-31
NFE2L2	DCUN1D3	0.72854214	9.9E-31
NFE2L2	TBK1	0.72842299	1.02E-30
NFE2L2	BROX	0.72837925	1.04E-30
NFE2L2	STRN3	0.72835095	1.04E-30
NFE2L2	MAGT1	0.72825337	1.07E-30
NFE2L2	KRR1	0.72813492	1.11E-30
NFE2L2	CPSF2	0.72788816	1.18E-30
NFE2L2	CACUL1	0.72756242	1.29E-30
NFE2L2	AFAP1	0.72753395	1.3E-30
NFE2L2	CMTM6	0.72739641	1.35E-30
NFE2L2	PPFIA1	0.72721096	1.43E-30
NFE2L2	LAMTOR3	0.72704789	1.49E-30
NFE2L2	RAP1B	0.72691512	1.55E-30
NFE2L2	ZNF609	0.72676186	1.61E-30
NFE2L2	LATS2	0.72647625	1.74E-30
NFE2L2	YTHDF3	0.72643803	1.76E-30
NFE2L2	TRIM34	0.72569474	2.15E-30
NFE2L2	CSTF2T	0.72557382	2.22E-30
NFE2L2	NCOA3	0.72538108	2.34E-30
NFE2L2	ZDHHC20	0.72526331	2.42E-30
NFE2L2	RNF121	0.72524309	2.43E-30
NFE2L2	PKP4	0.72523588	2.44E-30
NFE2L2	OSMR	0.72502589	2.58E-30
NFE2L2	RND3	0.72497308	2.62E-30
NFE2L2	SRGAP2C	0.72456539	2.92E-30
NFE2L2	FBXL3	0.72444303	3.02E-30

NFE2L2	GPATCH2	0.72431857	3.12E-30
NFE2L2	CDC27	0.72429955	3.14E-30
NFE2L2	CLDND1	0.72428832	3.15E-30
NFE2L2	HNRNPA2B1	0.72417666	3.25E-30
NFE2L2	KRCC1	0.72402547	3.38E-30
NFE2L2	L3MBTL3	0.72400565	3.4E-30
NFE2L2	LRCH3	0.72396049	3.44E-30
NFE2L2	ZDHHC7	0.72394303	3.46E-30
NFE2L2	NSRP1	0.72391752	3.48E-30
NFE2L2	RBFOX2	0.72390814	3.49E-30
NFE2L2	CLINT1	0.72371524	3.67E-30
NFE2L2	RASSF3	0.72367219	3.72E-30
NFE2L2	PLEKHA1	0.72354277	3.85E-30
NFE2L2	CTNNA1	0.72335477	4.05E-30
NFE2L2	CDK12	0.72325664	4.16E-30
NFE2L2	NBR1	0.72318164	4.24E-30
NFE2L2	SLC44A1	0.7231464	4.28E-30
NFE2L2	DHX36	0.72314362	4.28E-30
NFE2L2	SEC22A	0.72313419	4.29E-30
NFE2L2	VAPA	0.72290179	4.57E-30
NFE2L2	MBTPS1	0.72277875	4.72E-30
NFE2L2	SH3KBP1	0.72277023	4.73E-30
NFE2L2	IPO7	0.72276246	4.74E-30
NFE2L2	ZNF644	0.72262607	4.92E-30
NFE2L2	HIPK3	0.72260693	4.95E-30
NFE2L2	RBM27	0.72240128	5.23E-30
NFE2L2	KCTD20	0.72229873	5.37E-30
NFE2L2	VAMP7	0.7221355	5.61E-30
NFE2L2	YEATS2	0.72204429	5.75E-30
NFE2L2	SGMS2	0.72201434	5.79E-30
NFE2L2	FAM204A	0.72201002	5.8E-30
NFE2L2	HECA	0.72199947	5.82E-30
NFE2L2	F2R	0.7219831	5.84E-30
NFE2L2	UTP25	0.72198245	5.84E-30
NFE2L2	GOLIM4	0.72182738	6.09E-30
NFE2L2	TTC23	0.72168355	6.33E-30
NFE2L2	UTP3	0.721588	6.49E-30
NFE2L2	SP100	0.72135211	6.91E-30
NFE2L2	MAP4	0.72118394	7.23E-30
NFE2L2	SELENOF	0.72117672	7.24E-30
NFE2L2	HBP1	0.72115644	7.28E-30
NFE2L2	ZNF267	0.72107105	7.45E-30
NFE2L2	ANTXR2	0.7208042	8E-30
NFE2L2	NBN	0.72080277	8E-30
NFE2L2	LACTB	0.72078777	8.03E-30
NFE2L2	MEAK7	0.72076781	8.07E-30
NFE2L2	TUT7	0.72076584	8.08E-30
NFE2L2	SLK	0.72028419	9.18E-30
NFE2L2	KPNA3	0.72014292	9.53E-30
NFE2L2	PHACTR4	0.72013074	9.56E-30
NFE2L2	OPA1	0.7201287	9.56E-30
NFE2L2	LAMC1	0.72012053	9.58E-30
NFE2L2	PEX19	0.72002182	9.84E-30
NFE2L2	MARK2	0.71992192	1.01E-29
NFE2L2	KDSR	0.71992156	1.01E-29
NFE2L2	BMS1	0.71986143	1.03E-29
NFE2L2	TFAM	0.71977166	1.05E-29
NFE2L2	BFAR	0.71976605	1.05E-29

NFE2L2	RNF4	0.7196491	1.09E-29
NFE2L2	ZNF562	0.71959524	1.1E-29
NFE2L2	SMARCAL1	0.71930326	1.19E-29
NFE2L2	FAM168A	0.71927888	1.2E-29
NFE2L2	MTM1	0.71927001	1.2E-29
NFE2L2	NDE1	0.71925527	1.2E-29
NFE2L2	SLMAP	0.71925214	1.21E-29
NFE2L2	ACSL4	0.71903576	1.28E-29
NFE2L2	C5orf24	0.71898051	1.29E-29
NFE2L2	CAND1	0.71859694	1.43E-29
NFE2L2	ENDOD1	0.71856079	1.45E-29
NFE2L2	AP003108.2	0.71827519	1.56E-29
NFE2L2	TSPAN14	0.71822201	1.58E-29
NFE2L2	RELA	0.71814276	1.61E-29
NFE2L2	MFAP1	0.71811034	1.63E-29
NFE2L2	FAM120A	0.71805345	1.65E-29
NFE2L2	NFIX	0.71803414	1.66E-29
NFE2L2	PTPN4	0.71802368	1.67E-29
NFE2L2	WBP1L	0.71792562	1.71E-29
NFE2L2	RANBP9	0.71769419	1.82E-29
NFE2L2	CHMP3	0.71769295	1.82E-29
NFE2L2	CEP170	0.71764851	1.84E-29
NFE2L2	SETD3	0.71755751	1.88E-29
NFE2L2	TFPI	0.71752322	1.9E-29
NFE2L2	PPP2CB	0.71730308	2.01E-29
NFE2L2	SUFU	0.71729359	2.02E-29
NFE2L2	CHIC2	0.71722539	2.05E-29
NFE2L2	CLIP1	0.7171801	2.08E-29
NFE2L2	HIF1A	0.71713299	2.1E-29
NFE2L2	AQR	0.71696336	2.2E-29
NFE2L2	UBN1	0.71686917	2.25E-29
NFE2L2	ZFAND6	0.71686887	2.25E-29
NFE2L2	UBXN2B	0.71679295	2.3E-29
NFE2L2	AFF4	0.71662022	2.4E-29
NFE2L2	OLA1	0.71648123	2.49E-29
NFE2L2	PARP8	0.71626782	2.63E-29
NFE2L2	FAS	0.71626113	2.64E-29
NFE2L2	LHFPL2	0.71625829	2.64E-29
NFE2L2	ARMT1	0.71620391	2.68E-29
NFE2L2	C14orf119	0.71611129	2.74E-29
NFE2L2	RASA2	0.71587049	2.92E-29
NFE2L2	WDR1	0.71584029	2.94E-29
NFE2L2	PARP14	0.71564881	3.09E-29
NFE2L2	ZHX2	0.71558711	3.14E-29
NFE2L2	API5	0.71544189	3.26E-29
NFE2L2	VPS4B	0.71535076	3.34E-29
NFE2L2	LTBP1	0.71513448	3.54E-29
NFE2L2	NRBP1	0.71499058	3.67E-29
NFE2L2	HNRNPD	0.71492126	3.74E-29
NFE2L2	CPT1A	0.71485508	3.8E-29
NFE2L2	YES1	0.714716	3.94E-29
NFE2L2	PEAK1	0.71465554	4E-29
NFE2L2	BTBD1	0.71453397	4.13E-29
NFE2L2	BLZF1	0.7144908	4.18E-29
NFE2L2	FZD6	0.71429815	4.39E-29
NFE2L2	TAX1BP1	0.71426831	4.42E-29
NFE2L2	SLC4A1AP	0.71426216	4.43E-29
NFE2L2	HFE	0.71424142	4.45E-29

NFE2L2	ATAD1	0.7140982	4.62E-29
NFE2L2	CUL4B	0.71395342	4.8E-29
NFE2L2	SRGAP2	0.71395038	4.8E-29
NFE2L2	DCTD	0.71380826	4.98E-29
NFE2L2	FEM1B	0.71363314	5.21E-29
NFE2L2	ANXA7	0.71362221	5.22E-29
NFE2L2	LIMK2	0.71359453	5.26E-29
NFE2L2	ERLIN1	0.71356171	5.31E-29
NFE2L2	RBMS2	0.71354417	5.33E-29
NFE2L2	USP6NL	0.71341605	5.51E-29
NFE2L2	SEC23A	0.71336629	5.58E-29
NFE2L2	RAB35	0.71317081	5.87E-29
NFE2L2	ENAH	0.71288714	6.31E-29
NFE2L2	DPF2	0.71276825	6.5E-29
NFE2L2	TERF2	0.7126883	6.64E-29
NFE2L2	RDH14	0.71247475	7.01E-29
NFE2L2	ABI2	0.71240559	7.14E-29
NFE2L2	AEBP2	0.71234416	7.25E-29
NFE2L2	HIP1	0.71233087	7.28E-29
NFE2L2	SYPL1	0.71231766	7.3E-29
NFE2L2	IL1RAP	0.71207646	7.76E-29
NFE2L2	DLST	0.71199889	7.92E-29
NFE2L2	ZNF263	0.71184426	8.24E-29
NFE2L2	SAR1A	0.71168309	8.59E-29
NFE2L2	GNL3L	0.71164413	8.67E-29
NFE2L2	CNOT11	0.71151083	8.97E-29
NFE2L2	VCPIP1	0.71145915	9.09E-29
NFE2L2	ASAP1	0.71123828	9.62E-29
NFE2L2	ARL14EP	0.71110144	9.96E-29
NFE2L2	MACF1	0.71106729	1E-28
NFE2L2	PDHX	0.71050037	1.16E-28
NFE2L2	SLC25A43	0.71043184	1.18E-28
NFE2L2	CDYL	0.71027667	1.23E-28
NFE2L2	MAP3K7	0.71018563	1.26E-28
NFE2L2	ITGA6	0.71015455	1.27E-28
NFE2L2	PLEKHG1	0.71012189	1.28E-28
NFE2L2	STT3B	0.70997794	1.32E-28
NFE2L2	SPG21	0.70985678	1.37E-28
NFE2L2	MTDH	0.70980488	1.38E-28
NFE2L2	SRP9	0.70978201	1.39E-28
NFE2L2	IDH1	0.70971373	1.42E-28
NFE2L2	GLYR1	0.70959653	1.46E-28
NFE2L2	GATAD2A	0.70946257	1.51E-28
NFE2L2	CAPZB	0.7092668	1.59E-28
NFE2L2	CCDC115	0.70899077	1.7E-28
NFE2L2	TAF2	0.70897371	1.71E-28
NFE2L2	ZBTB41	0.70887507	1.75E-28
NFE2L2	SMC3	0.70880247	1.78E-28
NFE2L2	FAM102B	0.70878391	1.79E-28
NFE2L2	SZRD1	0.7086794	1.84E-28
NFE2L2	SLC6A6	0.70854228	1.9E-28
NFE2L2	UBAP2L	0.70848761	1.93E-28
NFE2L2	FKBP15	0.70830146	2.02E-28
NFE2L2	ATXN3	0.70817737	2.09E-28
NFE2L2	SNX2	0.70813854	2.11E-28
NFE2L2	PACS1	0.70809949	2.13E-28
NFE2L2	UBE2D1	0.70804957	2.15E-28
NFE2L2	ETV6	0.70798539	2.19E-28

NFE2L2	TMEM167B	0.70776356	2.31E-28
NFE2L2	SPATS2L	0.70776279	2.32E-28
NFE2L2	SFT2D2	0.70764889	2.38E-28
NFE2L2	NAGA	0.70760116	2.41E-28
NFE2L2	LSM14A	0.70759018	2.42E-28
NFE2L2	NDFIP2	0.70748976	2.48E-28
NFE2L2	UBXN2A	0.70735402	2.57E-28
NFE2L2	ZCCHC9	0.70725521	2.63E-28
NFE2L2	ISY1-RAB43	0.70710099	2.73E-28
NFE2L2	CASP10	0.70700904	2.8E-28
NFE2L2	ELK1	0.70691761	2.86E-28
NFE2L2	DAZAP2	0.70689726	2.88E-28
NFE2L2	PARVA	0.70656618	3.12E-28
NFE2L2	RNF149	0.70654442	3.14E-28
NFE2L2	CHML	0.7065072	3.17E-28
NFE2L2	FRRS1	0.70647507	3.2E-28
NFE2L2	WIPF2	0.706354	3.29E-28
NFE2L2	CLIC4	0.70600926	3.59E-28
NFE2L2	BRCC3	0.7059729	3.62E-28
NFE2L2	GTF2A1	0.7057959	3.79E-28
NFE2L2	NR2F2	0.70577663	3.8E-28
NFE2L2	GNPTAB	0.70574161	3.84E-28
NFE2L2	NOTCH2	0.70557328	4E-28
NFE2L2	LASP1	0.70552398	4.05E-28
NFE2L2	ANXA5	0.70552397	4.05E-28
NFE2L2	SNX12	0.70550564	4.07E-28
NFE2L2	CDC5L	0.70536883	4.21E-28
NFE2L2	CDK2	0.70526713	4.32E-28
NFE2L2	APBB2	0.70523735	4.35E-28
NFE2L2	PNPLA8	0.70519385	4.4E-28
NFE2L2	MED14	0.70517033	4.42E-28
NFE2L2	ARPP19	0.70514969	4.45E-28
NFE2L2	RNF139	0.70514039	4.46E-28
NFE2L2	PGM2L1	0.70510567	4.49E-28
NFE2L2	CARD6	0.70506586	4.54E-28
NFE2L2	NPTN	0.70479481	4.85E-28
NFE2L2	MED1	0.70476678	4.89E-28
NFE2L2	MAP3K20	0.70466747	5.01E-28
NFE2L2	PHACTR2	0.70439721	5.36E-28
NFE2L2	MRPL49	0.70439428	5.36E-28
NFE2L2	YIPF5	0.70434536	5.43E-28
NFE2L2	TAF1A	0.70432622	5.45E-28
NFE2L2	PGGT1B	0.70424969	5.56E-28
NFE2L2	DHDDS	0.70420937	5.61E-28
NFE2L2	OSGIN2	0.70418982	5.64E-28
NFE2L2	SPRTN	0.70399926	5.91E-28
NFE2L2	PTPRK	0.70387776	6.09E-28
NFE2L2	FEM1C	0.70377573	6.25E-28
NFE2L2	LINS1	0.70371523	6.34E-28
NFE2L2	KCTD10	0.70359771	6.53E-28
NFE2L2	HMGB1	0.70357454	6.56E-28
NFE2L2	SLC39A1	0.70353421	6.63E-28
NFE2L2	METTL21A	0.70337917	6.89E-28
NFE2L2	ZFP64	0.70335592	6.93E-28
NFE2L2	ESF1	0.7030812	7.41E-28
NFE2L2	MAPK6	0.70306016	7.45E-28
NFE2L2	GGPS1	0.70304046	7.49E-28
NFE2L2	SCP2	0.70296497	7.63E-28

NFE2L2	VPS41	0.70294751	7.66E-28
NFE2L2	CERS6	0.70290234	7.75E-28
NFE2L2	TSG101	0.70290122	7.75E-28
NFE2L2	H6PD	0.7028426	7.86E-28
NFE2L2	CCNK	0.70279157	7.96E-28
NFE2L2	ECHDC1	0.70271904	8.1E-28
NFE2L2	NCK2	0.70265903	8.22E-28
NFE2L2	MORF4L1	0.70262244	8.3E-28
NFE2L2	ANKRD13C	0.70262157	8.3E-28
NFE2L2	STK4	0.70260825	8.33E-28
NFE2L2	CAPN2	0.7022957	8.99E-28
NFE2L2	ZNF148	0.70211787	9.39E-28
NFE2L2	USP1	0.70205142	9.54E-28
NFE2L2	ICE2	0.70191485	9.87E-28
NFE2L2	HIVEP2	0.70157901	1.07E-27
NFE2L2	DNAJC21	0.70154042	1.08E-27
NFE2L2	PEX2	0.70136697	1.13E-27
NFE2L2	PPM1A	0.70127507	1.15E-27
NFE2L2	TMEM35B	0.70115296	1.19E-27
NFE2L2	SERINC3	0.70107705	1.21E-27
NFE2L2	CLP1	0.701018	1.23E-27
NFE2L2	TERF1	0.70083722	1.28E-27
NFE2L2	RPRD2	0.70079757	1.3E-27
NFE2L2	SF3B3	0.7007079	1.33E-27
NFE2L2	E2F3	0.70066565	1.34E-27
NFE2L2	PRPF38A	0.70056267	1.37E-27
NFE2L2	PHF13	0.7005443	1.38E-27
NFE2L2	NAALADL2	0.70052218	1.39E-27
NFE2L2	PDCL	0.7004929	1.4E-27
NFE2L2	ATP2A2	0.70047808	1.4E-27
NFE2L2	DPY19L1	0.70030105	1.46E-27
NFE2L2	C16orf70	0.70025487	1.48E-27
NFE2L2	MAP3K13	0.70016619	1.51E-27
NFE2L2	BPNT1	0.70011995	1.53E-27
NFE2L2	NMI	0.69978472	1.66E-27
NFE2L2	YWHAZ	0.69963741	1.72E-27
NFE2L2	MAX	0.69955707	1.75E-27
NFE2L2	CERS2	0.69936507	1.84E-27
NFE2L2	ETS2	0.6993441	1.85E-27
NFE2L2	HIVEP1	0.6993372	1.85E-27
NFE2L2	XPR1	0.69928604	1.87E-27
NFE2L2	TPP1	0.69889152	2.06E-27
NFE2L2	TNPO1	0.69885315	2.08E-27
NFE2L2	METTL8	0.69866783	2.17E-27
NFE2L2	ZDHHC9	0.6986663	2.18E-27
NFE2L2	MBNL2	0.69866351	2.18E-27
NFE2L2	CYB5B	0.69850799	2.26E-27
NFE2L2	FOXP1	0.69845715	2.29E-27
NFE2L2	RPS6KA3	0.69816294	2.46E-27
NFE2L2	DPY19L4	0.69812278	2.48E-27
NFE2L2	UGDH	0.6980942	2.5E-27
NFE2L2	SERPINB8	0.69801542	2.55E-27
NFE2L2	BAZ1A	0.69800933	2.55E-27
NFE2L2	UBE2I	0.69740583	2.95E-27
NFE2L2	ATG5	0.69736467	2.98E-27
NFE2L2	RABGAP1L	0.69718422	3.11E-27
NFE2L2	GLUD1	0.69716144	3.13E-27
NFE2L2	FCHSD2	0.69708625	3.18E-27

NFE2L2	G3BP2	0.69703109	3.22E-27
NFE2L2	SYF2	0.69656703	3.6E-27
NFE2L2	SF3A1	0.69651671	3.65E-27
NFE2L2	RSRC1	0.69636479	3.78E-27
NFE2L2	ATE1	0.69631078	3.83E-27
NFE2L2	RAD23B	0.69626528	3.87E-27
NFE2L2	EVI5	0.69625477	3.88E-27
NFE2L2	ATF1	0.69614497	3.99E-27
NFE2L2	NUDCD3	0.69608454	4.05E-27
NFE2L2	PTP4A2	0.69602816	4.1E-27
NFE2L2	KDM3B	0.6959925	4.14E-27
NFE2L2	KPNA6	0.69581487	4.31E-27
NFE2L2	SMURF2	0.69581022	4.32E-27
NFE2L2	BCL2L13	0.69571761	4.42E-27
NFE2L2	UBFD1	0.69567528	4.46E-27
NFE2L2	MED20	0.69559568	4.55E-27
NFE2L2	NTAN1	0.69550746	4.64E-27
NFE2L2	SSPN	0.69531584	4.86E-27
NFE2L2	AIMP1	0.69531376	4.86E-27
NFE2L2	OTUD1	0.69528842	4.89E-27
NFE2L2	NEDD1	0.69505194	5.18E-27
NFE2L2	CTR9	0.69501731	5.22E-27
NFE2L2	ELF4	0.69489441	5.37E-27
NFE2L2	LBR	0.69466467	5.68E-27
NFE2L2	UBE3C	0.69459636	5.77E-27
NFE2L2	PANK3	0.69444989	5.97E-27
NFE2L2	CAV2	0.69440048	6.04E-27
NFE2L2	COPB1	0.69436312	6.1E-27
NFE2L2	PCSK5	0.69425113	6.26E-27
NFE2L2	ABCC1	0.69418398	6.36E-27
NFE2L2	GTF2H3	0.69410004	6.49E-27
NFE2L2	NECAP2	0.69390593	6.79E-27
NFE2L2	KLHL7	0.69384801	6.89E-27
NFE2L2	VHL	0.69384617	6.89E-27
NFE2L2	TRAPPC6B	0.69374395	7.06E-27
NFE2L2	MSN	0.69372019	7.1E-27
NFE2L2	SNW1	0.69359892	7.31E-27
NFE2L2	UBQLN1	0.69356115	7.37E-27
NFE2L2	GALNT10	0.69352771	7.43E-27
NFE2L2	ATP11C	0.69352207	7.44E-27
NFE2L2	MSL2	0.69347847	7.52E-27
NFE2L2	PRRC1	0.69342132	7.62E-27
NFE2L2	KIF2A	0.69328678	7.87E-27
NFE2L2	ZC2HC1A	0.6931708	8.09E-27
NFE2L2	UBE2N	0.69306999	8.28E-27
NFE2L2	PRRG1	0.69292053	8.58E-27
NFE2L2	BAG5	0.69290612	8.61E-27
NFE2L2	CHSY1	0.69285861	8.7E-27
NFE2L2	CNIH1	0.69285732	8.71E-27
NFE2L2	SNX4	0.69282493	8.77E-27
NFE2L2	NSL1	0.69272465	8.98E-27
NFE2L2	HNRNPC	0.69265063	9.14E-27
NFE2L2	DEGS1	0.69241006	9.67E-27
NFE2L2	DIAPH2	0.69191242	1.09E-26
NFE2L2	CRK	0.69185235	1.1E-26
NFE2L2	CCDC32	0.69180108	1.12E-26
NFE2L2	VIPAS39	0.69169808	1.14E-26
NFE2L2	DCAF6	0.69160462	1.17E-26

NFE2L2	USB1	0.69145163	1.21E-26
NFE2L2	YWHAG	0.69127907	1.26E-26
NFE2L2	DPYD	0.69084352	1.4E-26
NFE2L2	UBE2Z	0.69083202	1.4E-26
NFE2L2	CDC42EP3	0.69083085	1.4E-26
NFE2L2	ARMCX6	0.69081867	1.41E-26
NFE2L2	CIAPIN1	0.69072855	1.44E-26
NFE2L2	GDE1	0.69068828	1.45E-26
NFE2L2	SNX16	0.69063995	1.47E-26
NFE2L2	SBF2	0.6905354	1.5E-26
NFE2L2	FOXN3	0.69053271	1.5E-26
NFE2L2	OSBPL8	0.69048559	1.52E-26
NFE2L2	WDR43	0.69042018	1.54E-26
NFE2L2	TOPORS	0.6904041	1.55E-26
NFE2L2	NIF3L1	0.6904025	1.55E-26
NFE2L2	PMS2	0.69038146	1.56E-26
NFE2L2	APP	0.69033882	1.57E-26
NFE2L2	MFN2	0.69033492	1.57E-26
NFE2L2	ZNF720	0.69032637	1.58E-26
NFE2L2	ZNF281	0.69013303	1.65E-26
NFE2L2	TM2D1	0.6900938	1.67E-26
NFE2L2	PDZD8	0.69008603	1.67E-26
NFE2L2	NFATC3	0.69002117	1.69E-26
NFE2L2	SSH1	0.68995821	1.72E-26
NFE2L2	SP110	0.68988543	1.75E-26
NFE2L2	MAPK1	0.68974735	1.81E-26
NFE2L2	CSNK2A1	0.68969698	1.83E-26
NFE2L2	TRIM14	0.68969578	1.83E-26
NFE2L2	SPAST	0.689639	1.85E-26
NFE2L2	IFNAR2	0.68961492	1.86E-26
NFE2L2	DDX50	0.68943101	1.94E-26
NFE2L2	CBX3	0.68926149	2.02E-26
NFE2L2	GMFB	0.68914549	2.08E-26
NFE2L2	ZMPSTE24	0.68904122	2.13E-26
NFE2L2	RUFY1	0.6889552	2.17E-26
NFE2L2	ITGA2	0.68890131	2.2E-26
NFE2L2	ARL6IP5	0.6888432	2.23E-26
NFE2L2	GOLGA7	0.68871542	2.29E-26
NFE2L2	EIF2S1	0.68857267	2.37E-26
NFE2L2	ERAP1	0.68855949	2.38E-26
NFE2L2	PTDSS1	0.68844091	2.45E-26
NFE2L2	MITF	0.68810454	2.64E-26
NFE2L2	SGPL1	0.688093	2.65E-26
NFE2L2	NABP1	0.68787699	2.79E-26
NFE2L2	NCOA2	0.68787207	2.79E-26
NFE2L2	B2M	0.6877633	2.86E-26
NFE2L2	WDR47	0.68771076	2.9E-26
NFE2L2	SINHCAF	0.68768213	2.91E-26
NFE2L2	KIAA1143	0.68766267	2.93E-26
NFE2L2	STS	0.68757498	2.99E-26
NFE2L2	IL10RB	0.68729281	3.19E-26
NFE2L2	PIAS3	0.68713706	3.31E-26
NFE2L2	SMAD1	0.6871052	3.33E-26
NFE2L2	CCNG2	0.68706861	3.36E-26
NFE2L2	BMI1	0.68697112	3.43E-26
NFE2L2	C5orf15	0.68696369	3.44E-26
NFE2L2	HHAT	0.68695877	3.44E-26
NFE2L2	GABPB1	0.68683419	3.54E-26

NFE2L2	SERTAD2	0.68681974	3.56E-26
NFE2L2	LARP7	0.68679137	3.58E-26
NFE2L2	TMEM106B	0.68669605	3.66E-26
NFE2L2	MANBA	0.68667351	3.68E-26
NFE2L2	EIF1AX	0.68665774	3.69E-26
NFE2L2	PLRG1	0.68644249	3.88E-26
NFE2L2	CAMSAP1	0.68641945	3.9E-26
NFE2L2	HNMT	0.68639717	3.92E-26
NFE2L2	ZEB1	0.68639283	3.92E-26
NFE2L2	TGS1	0.68625807	4.05E-26
NFE2L2	MAP2K1	0.68623191	4.07E-26
NFE2L2	STAG2	0.68617675	4.12E-26
NFE2L2	ARCN1	0.68615601	4.14E-26
NFE2L2	GDI2	0.68615123	4.15E-26
NFE2L2	HSPA14	0.68612783	4.17E-26
NFE2L2	ANP32A	0.68607582	4.22E-26
NFE2L2	NUP50	0.68604614	4.25E-26
NFE2L2	PIP4K2A	0.68595435	4.34E-26
NFE2L2	MAIP1	0.68581811	4.48E-26
NFE2L2	BCAP29	0.68537149	4.96E-26
NFE2L2	IRAK3	0.68533541	5E-26
NFE2L2	IL13RA1	0.68531929	5.02E-26
NFE2L2	AP2B1	0.68526454	5.08E-26
NFE2L2	CRTC3	0.6851948	5.16E-26
NFE2L2	QKI	0.6851225	5.25E-26
NFE2L2	VCAN	0.68501654	5.38E-26
NFE2L2	STAU2	0.68496667	5.44E-26
NFE2L2	MPHOSPH6	0.68480695	5.64E-26
NFE2L2	ATP6V1C1	0.68451909	6.03E-26
NFE2L2	DRAM2	0.68446302	6.1E-26
NFE2L2	ENC1	0.68423357	6.43E-26
NFE2L2	PPP2R5D	0.68421336	6.46E-26
NFE2L2	GANAB	0.68403334	6.73E-26
NFE2L2	CRTAP	0.68399366	6.79E-26
NFE2L2	C4orf3	0.68384168	7.03E-26
NFE2L2	TMED7	0.68378994	7.11E-26
NFE2L2	CNPPD1	0.68340466	7.76E-26
NFE2L2	TNFRSF10A	0.68338499	7.8E-26
NFE2L2	PEX26	0.6833664	7.83E-26
NFE2L2	NT5DC1	0.68321296	8.11E-26
NFE2L2	ADNP	0.68303584	8.44E-26
NFE2L2	PLEKHA2	0.68286926	8.77E-26
NFE2L2	KAT6A	0.68277649	8.95E-26
NFE2L2	CASP3	0.68273118	9.05E-26
NFE2L2	MYO1E	0.68242314	9.7E-26
NFE2L2	DTX3L	0.68238971	9.77E-26
NFE2L2	CHORDC1	0.68231292	9.94E-26
NFE2L2	USP14	0.6822471	1.01E-25
NFE2L2	TRIM26	0.68212553	1.04E-25
NFE2L2	CKAP5	0.68197726	1.07E-25
NFE2L2	PAK1	0.68180402	1.12E-25
NFE2L2	GOLPH3	0.68174086	1.13E-25
NFE2L2	SMC4	0.68171758	1.14E-25
NFE2L2	LRRCC1	0.68168223	1.15E-25
NFE2L2	WWTR1	0.68146163	1.21E-25
NFE2L2	AP1S3	0.68142078	1.22E-25
NFE2L2	RNASEH1	0.68134417	1.24E-25
NFE2L2	UBAP1	0.68133974	1.24E-25

NFE2L2	TMEM183A	0.68127659	1.26E-25
NFE2L2	LRRFIP2	0.68124926	1.26E-25
NFE2L2	SUZ12	0.68124162	1.27E-25
NFE2L2	GLO1	0.6811976	1.28E-25
NFE2L2	SMARCE1	0.68115641	1.29E-25
NFE2L2	PPP1CC	0.68106555	1.32E-25
NFE2L2	RNF19B	0.68105949	1.32E-25
NFE2L2	ZNF532	0.68104192	1.32E-25
NFE2L2	FYTTD1	0.68098244	1.34E-25
NFE2L2	NXT2	0.68092306	1.36E-25
NFE2L2	CEMIP2	0.68088822	1.37E-25
NFE2L2	RC3H2	0.68082232	1.39E-25
NFE2L2	CHRAC1	0.68071486	1.43E-25
NFE2L2	TCF20	0.68068032	1.44E-25
NFE2L2	KLHL2	0.68067494	1.44E-25
NFE2L2	UQCRC2	0.68064638	1.45E-25
NFE2L2	RAB29	0.68054642	1.48E-25
NFE2L2	DAPP1	0.68052861	1.49E-25
NFE2L2	RALBP1	0.68051602	1.49E-25
NFE2L2	MRPL44	0.6805139	1.49E-25
NFE2L2	HNRNPUL2	0.68035175	1.55E-25
NFE2L2	NOD1	0.68027669	1.57E-25
NFE2L2	MPP5	0.68023749	1.59E-25
NFE2L2	DNAJB14	0.68008617	1.64E-25
NFE2L2	FAM20B	0.68005812	1.65E-25
NFE2L2	PSMD7	0.67999164	1.68E-25
NFE2L2	PURB	0.67995123	1.69E-25
NFE2L2	RFWD3	0.67991091	1.71E-25
NFE2L2	NID1	0.67987476	1.72E-25
NFE2L2	SMC6	0.67981608	1.74E-25
NFE2L2	EMC2	0.67953578	1.86E-25
NFE2L2	MIS18BP1	0.67940267	1.91E-25
NFE2L2	SVIL	0.67939125	1.92E-25
NFE2L2	PPP1R12A	0.67915183	2.02E-25
NFE2L2	ACTN1	0.67906699	2.06E-25
NFE2L2	APOL6	0.67895055	2.12E-25
NFE2L2	ZFAND5	0.67884654	2.17E-25
NFE2L2	ARHGAP5	0.67872751	2.22E-25
NFE2L2	TFG	0.67864027	2.27E-25
NFE2L2	ZNF260	0.67864007	2.27E-25
NFE2L2	DCTN6	0.67822779	2.49E-25
NFE2L2	ERI1	0.67815682	2.53E-25
NFE2L2	C2CD3	0.67800461	2.61E-25
NFE2L2	LIMA1	0.67797042	2.63E-25
NFE2L2	PPTC7	0.67795085	2.64E-25
NFE2L2	UTP11	0.67780626	2.73E-25
NFE2L2	C1orf198	0.67771697	2.79E-25
NFE2L2	RUNX1	0.67769268	2.8E-25
NFE2L2	NF1	0.67768483	2.8E-25
NFE2L2	RNF14	0.67756757	2.88E-25
NFE2L2	STAT1	0.6774806	2.94E-25
NFE2L2	RB1	0.67748031	2.94E-25
NFE2L2	PDS5A	0.67740113	2.99E-25
NFE2L2	HSD17B11	0.6773136	3.05E-25
NFE2L2	UBQLN2	0.67730321	3.05E-25
NFE2L2	SPTLC2	0.67728053	3.07E-25
NFE2L2	LRP10	0.67710735	3.19E-25
NFE2L2	PSMD14	0.67703308	3.24E-25

NFE2L2	ATP11A	0.67685297	3.37E-25
NFE2L2	AMBRA1	0.67680378	3.41E-25
NFE2L2	CMPK1	0.6767541	3.45E-25
NFE2L2	RYBP	0.6767143	3.48E-25
NFE2L2	SAMD8	0.67661222	3.56E-25
NFE2L2	ARMC1	0.67652967	3.62E-25
NFE2L2	UBE2E1	0.67632776	3.79E-25
NFE2L2	BLOC1S5	0.67619115	3.9E-25
NFE2L2	GLG1	0.67614372	3.95E-25
NFE2L2	ZNF438	0.67609982	3.98E-25
NFE2L2	TAOK1	0.67607185	4.01E-25
NFE2L2	PARD3B	0.67589685	4.17E-25
NFE2L2	ABHD2	0.67582629	4.23E-25
NFE2L2	TEAD1	0.67581258	4.24E-25
NFE2L2	IFI16	0.67572999	4.32E-25
NFE2L2	RCC2	0.67560411	4.44E-25
NFE2L2	AMMECR1	0.67559111	4.46E-25
NFE2L2	APH1A	0.67556498	4.48E-25
NFE2L2	SETX	0.67555557	4.49E-25
NFE2L2	FBLIM1	0.67550959	4.54E-25
NFE2L2	GTF2B	0.67530437	4.75E-25
NFE2L2	TTC9C	0.67523909	4.82E-25
NFE2L2	C9orf64	0.67510157	4.96E-25
NFE2L2	PLSCR4	0.67505353	5.02E-25
NFE2L2	ADIPOR1	0.67502135	5.05E-25
NFE2L2	EMB	0.67496141	5.12E-25
NFE2L2	CCNY	0.67491151	5.17E-25
NFE2L2	TFRC	0.6748035	5.3E-25
NFE2L2	TMEM267	0.67478893	5.32E-25
NFE2L2	LRRC42	0.67473669	5.38E-25
NFE2L2	TRIOBP	0.67456699	5.58E-25
NFE2L2	ACBD5	0.67455018	5.6E-25
NFE2L2	BTN2A1	0.67442779	5.75E-25
NFE2L2	B3GNT5	0.67438467	5.81E-25
NFE2L2	RELL1	0.67436749	5.83E-25
NFE2L2	CLNS1A	0.67423009	6.01E-25
NFE2L2	PTMA	0.67420222	6.05E-25
NFE2L2	BCL10	0.67415167	6.11E-25
NFE2L2	RMND5A	0.67403747	6.27E-25
NFE2L2	TMEM50A	0.67386614	6.51E-25
NFE2L2	LNPEP	0.67380019	6.6E-25
NFE2L2	CNOT1	0.67347431	7.09E-25
NFE2L2	IGF2R	0.67336507	7.26E-25
NFE2L2	FAM177A1	0.67335982	7.27E-25
NFE2L2	KANK2	0.67331215	7.34E-25
NFE2L2	FRMD4B	0.67329272	7.38E-25
NFE2L2	FNBP1L	0.67320092	7.52E-25
NFE2L2	UTP14C	0.672976	7.9E-25
NFE2L2	IMPA1	0.67293225	7.98E-25
NFE2L2	VDR	0.67280833	8.2E-25
NFE2L2	ZFR	0.67270692	8.38E-25
NFE2L2	DYRK2	0.67268448	8.42E-25
NFE2L2	RER1	0.67262561	8.53E-25
NFE2L2	ETS1	0.67251263	8.74E-25
NFE2L2	ASAP2	0.67244897	8.86E-25
NFE2L2	SCAF11	0.67229676	9.16E-25
NFE2L2	KIAA1191	0.67218627	9.38E-25
NFE2L2	AP3S1	0.67217669	9.4E-25

NFE2L2	BABAM2	0.67216012	9.44E-25
NFE2L2	TRERF1	0.6721327	9.49E-25
NFE2L2	TOR1AIP2	0.67199807	9.78E-25
NFE2L2	TSNAX	0.67192472	9.93E-25
NFE2L2	POLR3C	0.67183376	1.01E-24
NFE2L2	USP4	0.67182797	1.01E-24
NFE2L2	FGD4	0.67170761	1.04E-24
NFE2L2	IFNGR2	0.67153185	1.08E-24
NFE2L2	PRKD3	0.67110998	1.19E-24
NFE2L2	CASK	0.67102715	1.21E-24
NFE2L2	DHX9	0.67084317	1.26E-24
NFE2L2	TPR	0.67082523	1.26E-24
NFE2L2	SLC44A2	0.67044234	1.37E-24
NFE2L2	XXYLT1	0.67043555	1.37E-24
NFE2L2	FBXO45	0.67035177	1.4E-24
NFE2L2	SMIM15	0.67021595	1.44E-24
NFE2L2	RASSF8	0.67010436	1.47E-24
NFE2L2	POLE3	0.67003131	1.5E-24
NFE2L2	CPOX	0.67002633	1.5E-24
NFE2L2	LDAH	0.66965494	1.62E-24
NFE2L2	PPHLN1	0.66949446	1.68E-24
NFE2L2	TRAM1	0.66937771	1.72E-24
NFE2L2	TNPO3	0.66923064	1.78E-24
NFE2L2	LIMK1	0.66913925	1.81E-24
NFE2L2	ZFP36L2	0.66896237	1.88E-24
NFE2L2	DCAF1	0.66890989	1.9E-24
NFE2L2	GMCL1	0.66884745	1.93E-24
NFE2L2	LACC1	0.66883012	1.94E-24
NFE2L2	HK1	0.66870575	1.99E-24
NFE2L2	INCENP	0.66868583	2E-24
NFE2L2	ERGIC1	0.66850892	2.07E-24
NFE2L2	BNIP3L	0.66844603	2.1E-24
NFE2L2	PDS5B	0.6682208	2.21E-24
NFE2L2	KNOP1	0.66796927	2.33E-24
NFE2L2	SLC35A4	0.66794598	2.34E-24
NFE2L2	CD2AP	0.66784487	2.39E-24
NFE2L2	ANAPC10	0.66775433	2.44E-24
NFE2L2	USP39	0.6676889	2.47E-24
NFE2L2	TGFBR1	0.66766551	2.48E-24
NFE2L2	STK17B	0.66758214	2.53E-24
NFE2L2	FAM98A	0.66750292	2.57E-24
NFE2L2	CNOT2	0.66748613	2.58E-24
NFE2L2	ZNF274	0.66730958	2.68E-24
NFE2L2	ZMYM5	0.66726809	2.7E-24
NFE2L2	BAG4	0.66708114	2.81E-24
NFE2L2	FRMD6	0.66678334	3E-24
NFE2L2	ANKMY2	0.66678015	3E-24
NFE2L2	ZNF322	0.66644946	3.22E-24
NFE2L2	TRAFD1	0.66636326	3.28E-24
NFE2L2	IQCK	0.66621751	3.38E-24
NFE2L2	LDB1	0.66597437	3.56E-24
NFE2L2	CHP1	0.66594942	3.58E-24
NFE2L2	INTS12	0.66593652	3.59E-24
NFE2L2	MAPKAPK2	0.66585741	3.65E-24
NFE2L2	NKIRAS2	0.66583024	3.67E-24
NFE2L2	MRPS10	0.66582456	3.68E-24
NFE2L2	ZNF623	0.66577417	3.72E-24
NFE2L2	SENP1	0.66563512	3.83E-24

NFE2L2	IFIH1	0.66558562	3.87E-24
NFE2L2	PRPF4	0.66546771	3.96E-24
NFE2L2	UBE2Q2	0.66538933	4.03E-24
NFE2L2	RNF141	0.66536874	4.05E-24
NFE2L2	FGD6	0.6653437	4.07E-24
NFE2L2	ZFYVE1	0.66528034	4.12E-24
NFE2L2	CPNE3	0.665242	4.16E-24
NFE2L2	UBA6	0.6652283	4.17E-24
NFE2L2	CHMP1B	0.66521593	4.18E-24
NFE2L2	SUSD1	0.66520051	4.19E-24
NFE2L2	SLFN5	0.66511125	4.27E-24
NFE2L2	CALD1	0.66500615	4.37E-24
NFE2L2	ZDHHC6	0.66500354	4.37E-24
NFE2L2	CDK17	0.6648196	4.55E-24
NFE2L2	CTSO	0.66478833	4.58E-24
NFE2L2	PPP1R3B	0.66431841	5.05E-24
NFE2L2	TJP2	0.66400858	5.39E-24
NFE2L2	RAI14	0.66392454	5.49E-24
NFE2L2	WASL	0.66390522	5.51E-24
NFE2L2	MIB1	0.66375407	5.69E-24
NFE2L2	FUT11	0.66368967	5.77E-24
NFE2L2	FZD7	0.66359867	5.88E-24
NFE2L2	BLOC1S6	0.66352036	5.98E-24
NFE2L2	DNAJC8	0.66336742	6.17E-24
NFE2L2	INPP4B	0.66330764	6.25E-24
NFE2L2	RNF24	0.66321204	6.38E-24
NFE2L2	CROT	0.6631185	6.5E-24
NFE2L2	CCDC43	0.66293515	6.76E-24
NFE2L2	RMDN3	0.66290519	6.8E-24
NFE2L2	NLN	0.66282452	6.92E-24
NFE2L2	FAM114A1	0.66267197	7.14E-24
NFE2L2	ALG11	0.66258369	7.27E-24
NFE2L2	MYO6	0.66250339	7.4E-24
NFE2L2	GASK1B	0.66241155	7.54E-24
NFE2L2	TWSG1	0.66236135	7.62E-24
NFE2L2	ADAT1	0.66228116	7.75E-24
NFE2L2	SNX3	0.66226409	7.78E-24
NFE2L2	LARP4B	0.66225525	7.79E-24
NFE2L2	TRUB1	0.66220997	7.87E-24
NFE2L2	INPP5A	0.66217153	7.93E-24
NFE2L2	DCP2	0.6620202	8.18E-24
NFE2L2	ANO1	0.66191859	8.36E-24
NFE2L2	STK26	0.66187154	8.44E-24
NFE2L2	KATNA1	0.66184948	8.48E-24
NFE2L2	PSMC6	0.66179826	8.57E-24
NFE2L2	BTN3A1	0.66159696	8.94E-24
NFE2L2	MRPL30	0.66148643	9.15E-24
NFE2L2	R3HDM1	0.66141889	9.28E-24
NFE2L2	BCORL1	0.66130998	9.49E-24
NFE2L2	NOTCH3	0.66124019	9.63E-24
NFE2L2	TMEM182	0.66122519	9.66E-24
NFE2L2	FSTL1	0.66120032	9.71E-24
NFE2L2	MDFIC	0.66117385	9.76E-24
NFE2L2	MICU1	0.66111659	9.88E-24
NFE2L2	RAB22A	0.66105383	1E-23
NFE2L2	OSBPL3	0.6609346	1.03E-23
NFE2L2	RBM22	0.66072951	1.07E-23
NFE2L2	ARF4	0.66065774	1.09E-23

NFE2L2	DOCK5	0.66063569	1.09E-23
NFE2L2	SMARCC1	0.66061055	1.1E-23
NFE2L2	MRPL35	0.66052322	1.12E-23
NFE2L2	KITLG	0.66049247	1.13E-23
NFE2L2	MFAP3	0.66043261	1.14E-23
NFE2L2	ZMAT2	0.66039533	1.15E-23
NFE2L2	ATP2B4	0.66038919	1.15E-23
NFE2L2	YY1AP1	0.66037602	1.15E-23
NFE2L2	LACTB2	0.66028766	1.17E-23
NFE2L2	SOCS4	0.66021022	1.19E-23
NFE2L2	MASTL	0.66019619	1.2E-23
NFE2L2	ACSL3	0.66017511	1.2E-23
NFE2L2	MMGT1	0.66010155	1.22E-23
NFE2L2	GMPS	0.66010061	1.22E-23
NFE2L2	MSH2	0.66006909	1.23E-23
NFE2L2	AKT1	0.66005418	1.23E-23
NFE2L2	TMEM159	0.65982333	1.29E-23
NFE2L2	KCTD3	0.65980617	1.3E-23
NFE2L2	SLCO3A1	0.65968762	1.33E-23
NFE2L2	SMARCA5	0.65966301	1.34E-23
NFE2L2	PTPN1	0.65961053	1.35E-23
NFE2L2	KCTD5	0.65959512	1.36E-23
NFE2L2	ATF6B	0.65947687	1.39E-23
NFE2L2	ISY1	0.65943273	1.4E-23
NFE2L2	SPPL3	0.65928625	1.44E-23
NFE2L2	KIRREL1	0.65906861	1.51E-23
NFE2L2	LEO1	0.65903577	1.52E-23
NFE2L2	CTTN	0.65899102	1.54E-23
NFE2L2	ZBTB33	0.65894498	1.55E-23
NFE2L2	SLC38A7	0.65891806	1.56E-23
NFE2L2	RABIF	0.6588785	1.57E-23
NFE2L2	BRD3	0.65862263	1.66E-23
NFE2L2	PSMC2	0.65851726	1.69E-23
NFE2L2	TMEM165	0.65844399	1.72E-23
NFE2L2	PDGFC	0.65840291	1.73E-23
NFE2L2	GALK2	0.65839969	1.73E-23
NFE2L2	OSBPL10	0.65838342	1.74E-23
NFE2L2	COL4A1	0.6583123	1.77E-23
NFE2L2	MED21	0.65830908	1.77E-23
NFE2L2	PKD2	0.65804873	1.86E-23
NFE2L2	SLC24A1	0.65803621	1.87E-23
NFE2L2	RAB11A	0.65790752	1.92E-23
NFE2L2	CCNDBP1	0.65790113	1.92E-23
NFE2L2	SLC15A4	0.65783681	1.95E-23
NFE2L2	FAF2	0.65774976	1.98E-23
NFE2L2	CYTH3	0.65757737	2.05E-23
NFE2L2	CALM2	0.65752611	2.08E-23
NFE2L2	RAB31	0.65752582	2.08E-23
NFE2L2	SOWAHC	0.65736592	2.15E-23
NFE2L2	BCAS2	0.657363	2.15E-23
NFE2L2	DNAJB6	0.65735066	2.15E-23
NFE2L2	ATXN7L3	0.65725512	2.19E-23
NFE2L2	CSTF2	0.65724568	2.2E-23
NFE2L2	ASAH1	0.65718994	2.22E-23
NFE2L2	MED6	0.65712785	2.25E-23
NFE2L2	CTSS	0.65696488	2.33E-23
NFE2L2	PALLD	0.65695309	2.33E-23
NFE2L2	ANKRD42	0.65691111	2.36E-23

NFE2L2	CUL4A	0.65687552	2.37E-23
NFE2L2	PTPRG	0.65667421	2.47E-23
NFE2L2	GRAMD2B	0.65655988	2.53E-23
NFE2L2	FIP1L1	0.65650999	2.56E-23
NFE2L2	SSRP1	0.6564137	2.61E-23
NFE2L2	SEMA3C	0.65638053	2.63E-23
NFE2L2	UBE2D2	0.65627486	2.68E-23
NFE2L2	LYPLA1	0.65618073	2.73E-23
NFE2L2	SH3PXD2A	0.65616571	2.74E-23
NFE2L2	KIF13A	0.65603916	2.81E-23
NFE2L2	TNFAIP8	0.65591276	2.89E-23
NFE2L2	EFNB2	0.65583672	2.93E-23
NFE2L2	TLE4	0.65562328	3.06E-23
NFE2L2	TMCO1	0.65554662	3.11E-23
NFE2L2	ATF7IP	0.6555319	3.12E-23
NFE2L2	PTTG1IP	0.655492	3.15E-23
NFE2L2	GPN1	0.65536969	3.23E-23
NFE2L2	SS18	0.65529996	3.27E-23
NFE2L2	ARHGAP1	0.65521538	3.33E-23
NFE2L2	PJA2	0.65502006	3.46E-23
NFE2L2	WDR89	0.65500642	3.47E-23
NFE2L2	SEMA4C	0.6549792	3.49E-23
NFE2L2	GINM1	0.65484098	3.59E-23
NFE2L2	NR3C1	0.65483682	3.6E-23
NFE2L2	KIF13B	0.65479773	3.62E-23
NFE2L2	SMG7	0.65459152	3.78E-23
NFE2L2	GEM	0.65455559	3.81E-23
NFE2L2	DCLRE1B	0.65450259	3.85E-23
NFE2L2	HCCS	0.65446216	3.88E-23
NFE2L2	TEAD3	0.65443456	3.9E-23
NFE2L2	UBE2L6	0.6543891	3.94E-23
NFE2L2	DIAPH1	0.65416596	4.12E-23
NFE2L2	TCEAL9	0.65407064	4.2E-23
NFE2L2	PRKCI	0.65401789	4.25E-23
NFE2L2	TMEM237	0.65387015	4.38E-23
NFE2L2	SLC25A46	0.65367701	4.55E-23
NFE2L2	CNNM4	0.65367614	4.55E-23
NFE2L2	PKNOX1	0.65366219	4.56E-23
NFE2L2	PHF20	0.65359848	4.62E-23
NFE2L2	RPL7L1	0.65345951	4.75E-23
NFE2L2	HPS3	0.65336531	4.85E-23
NFE2L2	SAMD9	0.65328426	4.93E-23
NFE2L2	FBXO8	0.65328032	4.93E-23
NFE2L2	WASHC2A	0.65303956	5.18E-23
NFE2L2	NUP107	0.65296316	5.26E-23
NFE2L2	COA7	0.65291691	5.31E-23
NFE2L2	CD68	0.65288498	5.34E-23
NFE2L2	ADI1	0.65287534	5.35E-23
NFE2L2	MORF4L2	0.65280473	5.43E-23
NFE2L2	ARMC10	0.65278037	5.45E-23
NFE2L2	GHITM	0.65275205	5.48E-23
NFE2L2	DCBLD1	0.65273785	5.5E-23
NFE2L2	LYST	0.65271418	5.53E-23
NFE2L2	NKRF	0.65258247	5.68E-23
NFE2L2	ZNF200	0.65247332	5.8E-23
NFE2L2	GOLPH3L	0.65242453	5.86E-23
NFE2L2	PCNX4	0.65234924	5.95E-23
NFE2L2	MRPL3	0.65223042	6.09E-23

NFE2L2	TRIM69	0.6522266	6.1E-23
NFE2L2	DCLRE1C	0.65217753	6.16E-23
NFE2L2	MYO1D	0.65213273	6.21E-23
NFE2L2	FAM220A	0.65206272	6.3E-23
NFE2L2	ABL1	0.65195035	6.45E-23
NFE2L2	ZYG11B	0.65184253	6.59E-23
NFE2L2	B4GALT1	0.65184061	6.59E-23
NFE2L2	NAMPT	0.65181517	6.62E-23
NFE2L2	KTN1	0.65157129	6.96E-23
NFE2L2	SLFN12	0.65151114	7.04E-23
NFE2L2	RARB	0.65150585	7.05E-23
NFE2L2	TM9SF3	0.6514966	7.06E-23
NFE2L2	HUS1	0.65147647	7.09E-23
NFE2L2	EPB41L1	0.65143572	7.15E-23
NFE2L2	ARPC4	0.65143448	7.15E-23
NFE2L2	PDGFRB	0.65133706	7.29E-23
NFE2L2	PRELID3B	0.65129011	7.36E-23
NFE2L2	FAM120AOS	0.65124338	7.43E-23
NFE2L2	DFFA	0.6511224	7.61E-23
NFE2L2	BICD1	0.65063033	8.4E-23
NFE2L2	SF3A3	0.65058438	8.48E-23
NFE2L2	CALHM2	0.65043029	8.74E-23
NFE2L2	CALU	0.65040063	8.79E-23
NFE2L2	NUDCD1	0.65034444	8.89E-23
NFE2L2	WWC3	0.65026843	9.03E-23
NFE2L2	SFXN3	0.65022137	9.12E-23
NFE2L2	TRPS1	0.65021981	9.12E-23
NFE2L2	SLC20A1	0.65018596	9.18E-23
NFE2L2	TRIM38	0.65014642	9.25E-23
NFE2L2	LAP3	0.65011757	9.31E-23
NFE2L2	AP1AR	0.6501005	9.34E-23
NFE2L2	GPX8	0.65007769	9.38E-23
NFE2L2	INTS14	0.64994043	9.64E-23
NFE2L2	ABHD13	0.64973763	1E-22
NFE2L2	DNAJC14	0.64969853	1.01E-22
NFE2L2	TMBIM1	0.64947031	1.06E-22
NFE2L2	CALCOCO2	0.64936972	1.08E-22
NFE2L2	ARID5B	0.64924691	1.11E-22
NFE2L2	TMED5	0.64903766	1.15E-22
NFE2L2	PRSS23	0.64899703	1.16E-22
NFE2L2	ZFP1	0.6489798	1.17E-22
NFE2L2	UBE2H	0.64896005	1.17E-22
NFE2L2	DYNC1LI1	0.64892739	1.18E-22
NFE2L2	CASP4	0.64871725	1.23E-22
NFE2L2	MED28	0.64864704	1.25E-22
NFE2L2	ATP10D	0.64864099	1.25E-22
NFE2L2	RAD51B	0.64849213	1.29E-22
NFE2L2	NPM1	0.64848739	1.29E-22
NFE2L2	GALC	0.648403	1.31E-22
NFE2L2	FAM222B	0.64836266	1.32E-22
NFE2L2	PSMC1	0.64834136	1.33E-22
NFE2L2	TPBG	0.64827679	1.34E-22
NFE2L2	DUSP6	0.64822275	1.36E-22
NFE2L2	NOLC1	0.64816034	1.37E-22
NFE2L2	ST13	0.64803299	1.41E-22
NFE2L2	GADD45A	0.64800469	1.42E-22
NFE2L2	BTN3A3	0.64794052	1.43E-22
NFE2L2	CNST	0.6479252	1.44E-22

NFE2L2	HS2ST1	0.64791767	1.44E-22
NFE2L2	GOSR1	0.6479138	1.44E-22
NFE2L2	BAZ1B	0.64781186	1.47E-22
NFE2L2	KBTBD4	0.64770975	1.5E-22
NFE2L2	SUMO2	0.64767316	1.51E-22
NFE2L2	TGFA	0.64766274	1.52E-22
NFE2L2	VPS37A	0.64760378	1.53E-22
NFE2L2	RPAP3	0.64748553	1.57E-22
NFE2L2	ZBTB6	0.64732039	1.62E-22
NFE2L2	ASNSD1	0.64726098	1.64E-22
NFE2L2	RNF145	0.64724178	1.65E-22
NFE2L2	CYB5R4	0.64718936	1.66E-22
NFE2L2	SGPP2	0.64708102	1.7E-22
NFE2L2	SNRNP200	0.64707309	1.7E-22
NFE2L2	SP140L	0.64705186	1.71E-22
NFE2L2	NUDT21	0.64696107	1.74E-22
NFE2L2	CDK19	0.64680009	1.8E-22
NFE2L2	TMEM248	0.64674612	1.82E-22
NFE2L2	TGIF1	0.64673858	1.82E-22
NFE2L2	TMX1	0.64639885	1.95E-22
NFE2L2	AGGF1	0.64629335	1.99E-22
NFE2L2	MOSPD1	0.64624814	2E-22
NFE2L2	RAB11FIP5	0.64618254	2.03E-22
NFE2L2	PXDC1	0.64613846	2.05E-22
NFE2L2	EAPP	0.64611212	2.06E-22
NFE2L2	LZIC	0.64588863	2.15E-22
NFE2L2	SMC1A	0.64572034	2.22E-22
NFE2L2	HARS2	0.6455906	2.28E-22
NFE2L2	CASP2	0.64557247	2.29E-22
NFE2L2	FBXO7	0.64548739	2.33E-22
NFE2L2	TRIM13	0.64548373	2.33E-22
NFE2L2	PTK2	0.64547114	2.33E-22
NFE2L2	MFHAS1	0.64516546	2.48E-22
NFE2L2	AMOTL2	0.6451018	2.51E-22
NFE2L2	LYN	0.64499056	2.57E-22
NFE2L2	NIFK	0.64497401	2.57E-22
NFE2L2	ANKS1A	0.64485993	2.63E-22
NFE2L2	GLTP	0.64483611	2.64E-22
NFE2L2	PIK3R1	0.64476059	2.68E-22
NFE2L2	ZFYVE21	0.64467546	2.73E-22
NFE2L2	KDM4A	0.64460739	2.77E-22
NFE2L2	PDE6D	0.64458342	2.78E-22
NFE2L2	BHLHE41	0.64432046	2.92E-22
NFE2L2	BST1	0.64427337	2.95E-22
NFE2L2	AKIRIN1	0.64425754	2.96E-22
NFE2L2	ACTN4	0.6441951	3E-22
NFE2L2	MEX3C	0.64382914	3.22E-22
NFE2L2	ITGB5	0.64351161	3.42E-22
NFE2L2	USP34	0.6435032	3.43E-22
NFE2L2	CPPED1	0.64315608	3.67E-22
NFE2L2	NEO1	0.64313933	3.68E-22
NFE2L2	WDR3	0.64291427	3.85E-22
NFE2L2	AP5M1	0.64286812	3.88E-22
NFE2L2	RAD21	0.64285895	3.89E-22
NFE2L2	RNF41	0.64257548	4.11E-22
NFE2L2	MID1	0.64246889	4.19E-22
NFE2L2	APOBEC3C	0.6424373	4.22E-22
NFE2L2	YWHAQ	0.64243628	4.22E-22

NFE2L2	FAM83B	0.64235481	4.29E-22
NFE2L2	ZNRF2	0.64233359	4.31E-22
NFE2L2	SBDS	0.64206829	4.53E-22
NFE2L2	FTO	0.64199179	4.6E-22
NFE2L2	IRF2BP2	0.64178353	4.79E-22
NFE2L2	TCTN3	0.64170791	4.86E-22
NFE2L2	SPRY1	0.64169258	4.88E-22
NFE2L2	PCGF5	0.64167928	4.89E-22
NFE2L2	SPSB1	0.64166568	4.9E-22
NFE2L2	SC5D	0.64165109	4.91E-22
NFE2L2	DCAF12	0.64161308	4.95E-22
NFE2L2	CDC23	0.64129305	5.27E-22
NFE2L2	SLC35A3	0.6412812	5.28E-22
NFE2L2	G6PD	0.64112663	5.44E-22
NFE2L2	TRIM56	0.64106066	5.51E-22
NFE2L2	LDHA	0.640992	5.58E-22
NFE2L2	CIPC	0.64099183	5.58E-22
NFE2L2	FRMD8	0.64082024	5.77E-22
NFE2L2	MBOAT1	0.64080627	5.79E-22
NFE2L2	NCEH1	0.64066078	5.95E-22
NFE2L2	SNX18	0.64054655	6.08E-22
NFE2L2	WEE1	0.64052814	6.1E-22
NFE2L2	DDX27	0.64050609	6.13E-22
NFE2L2	TWF1	0.64048595	6.15E-22
NFE2L2	PDE12	0.64028748	6.39E-22
NFE2L2	DLD	0.6399729	6.79E-22
NFE2L2	MFSD11	0.63995723	6.81E-22
NFE2L2	PSMD12	0.6399426	6.83E-22
NFE2L2	TPP2	0.63986661	6.93E-22
NFE2L2	SLC25A40	0.63985233	6.95E-22
NFE2L2	MED13	0.63976808	7.07E-22
NFE2L2	PRDX3	0.63959687	7.3E-22
NFE2L2	TMEM19	0.63956731	7.34E-22
NFE2L2	PIK3IP1	0.63956109	7.35E-22
NFE2L2	SNAPIN	0.63951956	7.41E-22
NFE2L2	CHM	0.63949329	7.45E-22
NFE2L2	ACTL6A	0.63949231	7.45E-22
NFE2L2	BHLHE40	0.6394637	7.49E-22
NFE2L2	STIM1	0.63939324	7.59E-22
NFE2L2	ZC3HAV1	0.63933142	7.68E-22
NFE2L2	EDNRA	0.63923568	7.83E-22
NFE2L2	ENTPD1	0.63919413	7.89E-22
NFE2L2	INIP	0.63915168	7.95E-22
NFE2L2	SOX4	0.639102	8.03E-22
NFE2L2	ABCF1	0.63909962	8.03E-22
NFE2L2	PRKX	0.63905555	8.1E-22
NFE2L2	INTS5	0.638971	8.23E-22
NFE2L2	UNC50	0.63881742	8.48E-22
NFE2L2	FBN1	0.63863667	8.78E-22
NFE2L2	ERF	0.63861022	8.82E-22
NFE2L2	RASA3	0.63847753	9.05E-22
NFE2L2	HDAC2	0.63845216	9.09E-22
NFE2L2	CFH	0.6382175	9.51E-22
NFE2L2	CXorf56	0.63815091	9.63E-22
NFE2L2	CORO2A	0.63795671	1E-21
NFE2L2	RDH10	0.63776382	1.04E-21
NFE2L2	LRP1	0.63771611	1.05E-21
NFE2L2	EXOC5	0.63768671	1.05E-21

NFE2L2	PARP3	0.63762508	1.06E-21
NFE2L2	RNF135	0.63747321	1.1E-21
NFE2L2	DDX58	0.63743809	1.1E-21
NFE2L2	TAF8	0.637437	1.1E-21
NFE2L2	EHD1	0.63740332	1.11E-21
NFE2L2	PRKG1	0.63733189	1.13E-21
NFE2L2	ANKRD11	0.6372688	1.14E-21
NFE2L2	STEAP2	0.6371746	1.16E-21
NFE2L2	CRYBG1	0.63716508	1.16E-21
NFE2L2	GTPBP4	0.6371051	1.18E-21
NFE2L2	VANGL1	0.63706627	1.18E-21
NFE2L2	ATP6V1E1	0.63693511	1.21E-21
NFE2L2	DAXX	0.63683738	1.24E-21
NFE2L2	MITD1	0.63653821	1.31E-21
NFE2L2	IFT57	0.63647103	1.33E-21
NFE2L2	YIPF6	0.63641855	1.34E-21
NFE2L2	RABL3	0.63633638	1.36E-21
NFE2L2	COL4A2	0.63631215	1.37E-21
NFE2L2	PLS1	0.63623395	1.39E-21
NFE2L2	TRIM22	0.6361661	1.41E-21
NFE2L2	RFFL	0.63610437	1.42E-21
NFE2L2	PPP4R1	0.63598382	1.45E-21
NFE2L2	PCDH7	0.63587256	1.49E-21
NFE2L2	PIGS	0.63587094	1.49E-21
NFE2L2	MGRN1	0.63586041	1.49E-21
NFE2L2	ARL6IP1	0.63585571	1.49E-21
NFE2L2	ITM2B	0.63584944	1.49E-21
NFE2L2	PIGC	0.63583734	1.5E-21
NFE2L2	AHCYL1	0.63556979	1.57E-21
NFE2L2	TNIK	0.63551916	1.59E-21
NFE2L2	SKP1	0.63548374	1.6E-21
NFE2L2	GLI2	0.63546623	1.6E-21
NFE2L2	RAP2B	0.63543984	1.61E-21
NFE2L2	CYLD	0.63542444	1.62E-21
NFE2L2	BUB3	0.6353676	1.63E-21
NFE2L2	ICMT	0.63527866	1.66E-21
NFE2L2	PIM1	0.63517554	1.7E-21
NFE2L2	DLG5	0.63514982	1.7E-21
NFE2L2	EIF3M	0.63490261	1.78E-21
NFE2L2	TCF7L2	0.63453751	1.91E-21
NFE2L2	CTSC	0.63451006	1.92E-21
NFE2L2	UBA2	0.63450295	1.92E-21
NFE2L2	KAT7	0.63448416	1.93E-21
NFE2L2	SLC30A9	0.6344362	1.95E-21
NFE2L2	YWHAH	0.63439648	1.96E-21
NFE2L2	GAB2	0.6339642	2.13E-21
NFE2L2	PGD	0.63390122	2.16E-21
NFE2L2	IL4R	0.63382038	2.19E-21
NFE2L2	LAMP2	0.63379155	2.2E-21
NFE2L2	METTL14	0.63363611	2.27E-21
NFE2L2	MRTFA	0.63360997	2.28E-21
NFE2L2	ARRDC3	0.63358299	2.29E-21
NFE2L2	PRKCH	0.63355833	2.3E-21
NFE2L2	SCARB2	0.63349417	2.33E-21
NFE2L2	BIRC3	0.63341825	2.36E-21
NFE2L2	TMEM41A	0.63336535	2.38E-21
NFE2L2	PRKDC	0.63331261	2.41E-21
NFE2L2	NAPG	0.63310799	2.5E-21

NFE2L2	TBC1D5	0.63297595	2.56E-21
NFE2L2	ANKRD27	0.63292321	2.59E-21
NFE2L2	USP38	0.6328776	2.61E-21
NFE2L2	RAB2A	0.63287408	2.61E-21
NFE2L2	ANXA1	0.6327572	2.67E-21
NFE2L2	TTL	0.6325425	2.78E-21
NFE2L2	SMURF1	0.63247795	2.81E-21
NFE2L2	CGAS	0.63244945	2.83E-21
NFE2L2	DACT1	0.63232986	2.89E-21
NFE2L2	CHMP5	0.63214181	3E-21
NFE2L2	EPB41L2	0.63141274	3.43E-21
NFE2L2	CENPL	0.63141016	3.44E-21
NFE2L2	ZDHHC3	0.6313438	3.48E-21
NFE2L2	PROS1	0.63119763	3.57E-21
NFE2L2	ARFGEF1	0.63103282	3.69E-21
NFE2L2	SLC17A5	0.63101418	3.7E-21
NFE2L2	ARHGAP17	0.63100662	3.7E-21
NFE2L2	ARHGAP12	0.63097363	3.73E-21
NFE2L2	PFDN1	0.63089201	3.78E-21
NFE2L2	TFDP1	0.63080688	3.84E-21
NFE2L2	NIPA1	0.63079683	3.85E-21
NFE2L2	RPRD1B	0.6307532	3.88E-21
NFE2L2	TRIO	0.63054673	4.03E-21
NFE2L2	TRAK1	0.63054274	4.04E-21
NFE2L2	FYCO1	0.63039641	4.15E-21
NFE2L2	SART3	0.63034648	4.19E-21
NFE2L2	RASA1	0.63013234	4.36E-21
NFE2L2	VKORC1L1	0.62987975	4.57E-21
NFE2L2	BMT2	0.62985265	4.59E-21
NFE2L2	VEZF1	0.6297987	4.64E-21
NFE2L2	RMDN2	0.62949949	4.9E-21
NFE2L2	UBASH3B	0.62948226	4.91E-21
NFE2L2	RCBTB2	0.62935248	5.03E-21
NFE2L2	ROBO1	0.62925561	5.13E-21
NFE2L2	BTF3L4	0.62923536	5.14E-21
NFE2L2	TMEM135	0.62922201	5.16E-21
NFE2L2	HNRNPUL1	0.62919715	5.18E-21
NFE2L2	GPATCH11	0.6290619	5.31E-21
NFE2L2	UBE2Q1	0.62904477	5.33E-21
NFE2L2	PGAM1	0.62872507	5.65E-21
NFE2L2	PTGR1	0.62848525	5.91E-21
NFE2L2	CCNI	0.62842781	5.97E-21
NFE2L2	PI4K2A	0.62841391	5.99E-21
NFE2L2	FKBP1A	0.62834424	6.06E-21
NFE2L2	RCN2	0.62823915	6.18E-21
NFE2L2	TSPAN9	0.62818738	6.24E-21
NFE2L2	MAPRE1	0.62818556	6.24E-21
NFE2L2	NAP1L4	0.62794914	6.52E-21
NFE2L2	TEP1	0.62794182	6.53E-21
NFE2L2	PIP4P2	0.62791709	6.56E-21
NFE2L2	SNAPC1	0.62789753	6.58E-21
NFE2L2	TAF13	0.6278824	6.6E-21
NFE2L2	TMEM216	0.62787025	6.62E-21
NFE2L2	PTPN11	0.62786197	6.63E-21
NFE2L2	NUP58	0.62785011	6.64E-21
NFE2L2	NCOA7	0.62769501	6.83E-21
NFE2L2	HNRNPA3	0.62766961	6.87E-21
NFE2L2	MBNL3	0.62755221	7.02E-21

NFE2L2	RTL6	0.62752233	7.05E-21
NFE2L2	PCYOX1	0.62749637	7.09E-21
NFE2L2	EDC3	0.62739471	7.22E-21
NFE2L2	RNF20	0.62738264	7.24E-21
NFE2L2	SYTL2	0.62708816	7.64E-21
NFE2L2	KPNB1	0.62708478	7.64E-21
NFE2L2	SH3RF1	0.62704862	7.7E-21
NFE2L2	TCEA1	0.62690823	7.9E-21
NFE2L2	CDH11	0.62689968	7.91E-21
NFE2L2	ZNF250	0.62647743	8.54E-21
NFE2L2	TTC1	0.62636301	8.73E-21
NFE2L2	TMX2	0.62629191	8.84E-21
NFE2L2	POLR2C	0.62611603	9.13E-21
NFE2L2	ENSA	0.62610982	9.14E-21
NFE2L2	SOS2	0.62601487	9.3E-21
NFE2L2	MAFG	0.62598544	9.35E-21
NFE2L2	NEMP2	0.62594216	9.42E-21
NFE2L2	NRP1	0.62580758	9.66E-21
NFE2L2	SPRY2	0.62577393	9.72E-21
NFE2L2	FAM98B	0.6257287	9.8E-21
NFE2L2	TMLHE	0.6255717	1.01E-20
NFE2L2	TPM1	0.62553196	1.02E-20
NFE2L2	TMEM154	0.62545321	1.03E-20
NFE2L2	TRIB1	0.62540928	1.04E-20
NFE2L2	RARA	0.62535381	1.05E-20
NFE2L2	APEX2	0.62534993	1.05E-20
NFE2L2	UBE2K	0.62530274	1.06E-20
NFE2L2	UBA1	0.62508812	1.1E-20
NFE2L2	ZBTB7A	0.62498388	1.12E-20
NFE2L2	RNF6	0.62491503	1.14E-20
NFE2L2	YIPF4	0.62485231	1.15E-20
NFE2L2	CDC42SE2	0.62485227	1.15E-20
NFE2L2	PAQR3	0.62484047	1.15E-20
NFE2L2	RNF170	0.6247875	1.16E-20
NFE2L2	LTBP2	0.62476101	1.17E-20
NFE2L2	TSKU	0.62467016	1.19E-20
NFE2L2	LEF1	0.62459692	1.2E-20
NFE2L2	CAMK2D	0.6244398	1.24E-20
NFE2L2	SNX5	0.62420421	1.29E-20
NFE2L2	TEX10	0.62415295	1.31E-20
NFE2L2	IL1R1	0.62402318	1.34E-20
NFE2L2	FBXO22	0.62391763	1.36E-20
NFE2L2	C1S	0.62387976	1.37E-20
NFE2L2	CDK6	0.62379352	1.39E-20
NFE2L2	IRS1	0.62378758	1.39E-20
NFE2L2	TDRD7	0.62360801	1.44E-20
NFE2L2	ERGIC2	0.62360563	1.44E-20
NFE2L2	GTF3C4	0.62350124	1.47E-20
NFE2L2	APEX1	0.62332541	1.52E-20
NFE2L2	ITGA1	0.62320843	1.55E-20
NFE2L2	XAF1	0.62296264	1.62E-20
NFE2L2	DERL1	0.62293331	1.63E-20
NFE2L2	CRKL	0.62292593	1.63E-20
NFE2L2	EHD2	0.62285352	1.65E-20
NFE2L2	GSK3B	0.62276429	1.68E-20
NFE2L2	SLC25A13	0.62270272	1.7E-20
NFE2L2	MGAT4B	0.62258292	1.73E-20
NFE2L2	DCN	0.62241958	1.79E-20

NFE2L2	C9orf78	0.62210996	1.89E-20
NFE2L2	JKAMP	0.62190211	1.96E-20
NFE2L2	CPD	0.62182617	1.99E-20
NFE2L2	ITPR3	0.62175536	2.01E-20
NFE2L2	TTC4	0.62171455	2.03E-20
NFE2L2	PLA2G4A	0.62168536	2.04E-20
NFE2L2	FRYL	0.62161788	2.06E-20
NFE2L2	COX15	0.6216152	2.06E-20
NFE2L2	BTN3A2	0.6215155	2.1E-20
NFE2L2	CYB5R3	0.62147232	2.12E-20
NFE2L2	MAP1LC3B2	0.6211746	2.23E-20
NFE2L2	CDC42BPA	0.62116078	2.24E-20
NFE2L2	PSMD2	0.6210681	2.28E-20
NFE2L2	EIF2B2	0.62095325	2.33E-20
NFE2L2	STK24	0.62089989	2.35E-20
NFE2L2	ADGRG6	0.62073114	2.42E-20
NFE2L2	IRF9	0.62033622	2.6E-20
NFE2L2	OSBPL1A	0.62021359	2.65E-20
NFE2L2	SRPK2	0.62018714	2.67E-20
NFE2L2	NDEL1	0.62014045	2.69E-20
NFE2L2	DAB2	0.62010911	2.7E-20
NFE2L2	JDP2	0.62002759	2.74E-20
NFE2L2	MREG	0.61999092	2.76E-20
NFE2L2	CHUK	0.61991237	2.8E-20
NFE2L2	IL7	0.61987094	2.82E-20
NFE2L2	CFAP20	0.6197887	2.86E-20
NFE2L2	B3GALNT1	0.6197318	2.89E-20
NFE2L2	MXRA5	0.61968805	2.92E-20
NFE2L2	TNFRSF1A	0.61962037	2.95E-20
NFE2L2	MPZL2	0.6194057	3.07E-20
NFE2L2	RIPK2	0.6193866	3.08E-20
NFE2L2	GPR107	0.61924473	3.16E-20
NFE2L2	STK17A	0.61922735	3.17E-20
NFE2L2	ZNF134	0.61910561	3.24E-20
NFE2L2	ABL2	0.61898196	3.31E-20
NFE2L2	CTBS	0.61867103	3.5E-20
NFE2L2	TOP2B	0.61866027	3.5E-20
NFE2L2	ANTXR1	0.6186155	3.53E-20
NFE2L2	AC010132.3	0.61859951	3.54E-20
NFE2L2	CD82	0.61859307	3.55E-20
NFE2L2	ZMIZ1	0.6185188	3.59E-20
NFE2L2	HADHA	0.61848244	3.62E-20
NFE2L2	NFIL3	0.61846269	3.63E-20
NFE2L2	GFPT1	0.61843667	3.65E-20
NFE2L2	PLBD1	0.61838504	3.68E-20
NFE2L2	TNFSF13	0.61831972	3.72E-20
NFE2L2	LRRC1	0.61819475	3.81E-20
NFE2L2	SLC25A32	0.61814347	3.84E-20
NFE2L2	ILK	0.61810433	3.87E-20
NFE2L2	ELOVL1	0.61801809	3.93E-20
NFE2L2	IVNS1ABP	0.61798408	3.95E-20
NFE2L2	PWP1	0.61793803	3.98E-20
NFE2L2	ARF3	0.61793568	3.98E-20
NFE2L2	CREG1	0.61791928	4E-20
NFE2L2	XYLT1	0.61786542	4.03E-20
NFE2L2	MGAT5	0.61770437	4.15E-20
NFE2L2	MED4	0.6176235	4.21E-20
NFE2L2	FKBP9	0.61752007	4.29E-20

NFE2L2	MARVELD1	0.61745667	4.34E-20
NFE2L2	LPCAT2	0.61739383	4.39E-20
NFE2L2	FADD	0.61735901	4.41E-20
NFE2L2	DDR2	0.61721697	4.53E-20
NFE2L2	REXO2	0.61709546	4.62E-20
NFE2L2	STX3	0.61708806	4.63E-20
NFE2L2	ACOT9	0.61693242	4.76E-20
NFE2L2	GCC1	0.61692722	4.76E-20
NFE2L2	TUBA1C	0.61687352	4.81E-20
NFE2L2	GBP1	0.61684579	4.83E-20
NFE2L2	ATG7	0.61681395	4.86E-20
NFE2L2	COL6A3	0.61672635	4.94E-20
NFE2L2	IL6ST	0.61671295	4.95E-20
NFE2L2	PSMD10	0.61670572	4.95E-20
NFE2L2	MCUB	0.61670079	4.96E-20
NFE2L2	IGFBP5	0.61668474	4.97E-20
NFE2L2	C1GALT1	0.61664626	5.01E-20
NFE2L2	TADA1	0.6165984	5.05E-20
NFE2L2	SAMD9L	0.61658543	5.06E-20
NFE2L2	VSIR	0.61655069	5.09E-20
NFE2L2	AMFR	0.61649747	5.14E-20
NFE2L2	IMMT	0.61648359	5.15E-20
NFE2L2	HSPG2	0.61628992	5.33E-20
NFE2L2	PHC2	0.61627206	5.35E-20
NFE2L2	SMAD3	0.61622132	5.4E-20
NFE2L2	TRAM2	0.61621754	5.4E-20
NFE2L2	LIN9	0.61614991	5.46E-20
NFE2L2	COL5A2	0.6161495	5.47E-20
NFE2L2	TXNIP	0.61597269	5.64E-20
NFE2L2	WDR20	0.61578295	5.83E-20
NFE2L2	GNPDA2	0.61564591	5.97E-20
NFE2L2	MYL12A	0.6155721	6.05E-20
NFE2L2	PHTF1	0.61550464	6.12E-20
NFE2L2	MIGA1	0.61550257	6.13E-20
NFE2L2	ALDH1A3	0.61549581	6.13E-20
NFE2L2	ELOVL5	0.61541636	6.22E-20
NFE2L2	ZNF45	0.6153742	6.27E-20
NFE2L2	EPB41L4A	0.61520012	6.46E-20
NFE2L2	PTBP1	0.61518464	6.48E-20
NFE2L2	NUB1	0.61511004	6.56E-20
NFE2L2	MYADM	0.61508826	6.59E-20
NFE2L2	DNASE1L1	0.61505244	6.63E-20
NFE2L2	SLC10A3	0.61488238	6.83E-20
NFE2L2	SPDL1	0.61482853	6.9E-20
NFE2L2	MESD	0.61479254	6.94E-20
NFE2L2	SYNJ2BP	0.61472102	7.03E-20
NFE2L2	PDCL3	0.61463018	7.14E-20
NFE2L2	FHL2	0.614396	7.44E-20
NFE2L2	TRIQK	0.61438398	7.45E-20
NFE2L2	SLC35A1	0.61430241	7.56E-20
NFE2L2	SOCS6	0.61429493	7.57E-20
NFE2L2	PLXDC2	0.61418551	7.72E-20
NFE2L2	NRP2	0.61413296	7.79E-20
NFE2L2	DPM1	0.61400147	7.97E-20
NFE2L2	RRAS2	0.61396058	8.03E-20
NFE2L2	LLPH	0.61376595	8.31E-20
NFE2L2	KLHL5	0.6136951	8.41E-20
NFE2L2	WIPF1	0.61361421	8.53E-20

NFE2L2	MICAL2	0.61358552	8.57E-20
NFE2L2	RALA	0.6134447	8.79E-20
NFE2L2	AKAP11	0.61339533	8.86E-20
NFE2L2	F11R	0.61338969	8.87E-20
NFE2L2	SLC12A2	0.61326598	9.07E-20
NFE2L2	MFSD6	0.61316705	9.22E-20
NFE2L2	SQSTM1	0.61299269	9.51E-20
NFE2L2	ZKSCAN5	0.61298044	9.53E-20
NFE2L2	GPR157	0.61297145	9.54E-20
NFE2L2	SGCB	0.61275249	9.91E-20
NFE2L2	DERA	0.61265072	1.01E-19
NFE2L2	PRAG1	0.61260689	1.02E-19
NFE2L2	AXL	0.61246846	1.04E-19
NFE2L2	IGFBP7	0.61237302	1.06E-19
NFE2L2	ZNF766	0.61233214	1.07E-19
NFE2L2	FNIP2	0.61229034	1.07E-19
NFE2L2	CD2BP2	0.61224214	1.08E-19
NFE2L2	CASP6	0.61222995	1.09E-19
NFE2L2	EAF1	0.61220422	1.09E-19
NFE2L2	ZNF827	0.61199853	1.13E-19
NFE2L2	PRTFDC1	0.61187931	1.15E-19
NFE2L2	IFT52	0.61187446	1.16E-19
NFE2L2	ING1	0.61187385	1.16E-19
NFE2L2	RETSAT	0.61179319	1.17E-19
NFE2L2	FUT8	0.61168334	1.19E-19
NFE2L2	ZCCHC24	0.61165079	1.2E-19
NFE2L2	DAAM1	0.61134765	1.27E-19
NFE2L2	ETFA	0.61130938	1.27E-19
NFE2L2	SNX24	0.61113336	1.31E-19
NFE2L2	AAR2	0.61095932	1.35E-19
NFE2L2	BDKRB2	0.61095334	1.36E-19
NFE2L2	FER	0.61083494	1.38E-19
NFE2L2	NFKBIA	0.61082381	1.39E-19
NFE2L2	LXN	0.61069551	1.42E-19
NFE2L2	MET	0.61066409	1.42E-19
NFE2L2	DIPK1A	0.61061842	1.44E-19
NFE2L2	PLA2R1	0.61061192	1.44E-19
NFE2L2	PAK1IP1	0.61057824	1.45E-19
NFE2L2	BBS9	0.61054097	1.46E-19
NFE2L2	CDC42BPB	0.61042842	1.48E-19
NFE2L2	HTATIP2	0.61040245	1.49E-19
NFE2L2	ZWILCH	0.61039266	1.49E-19
NFE2L2	RTN3	0.61034172	1.51E-19
NFE2L2	SLC35F2	0.61020018	1.54E-19
NFE2L2	LIX1L	0.61014987	1.56E-19
NFE2L2	EMP1	0.60995332	1.61E-19
NFE2L2	TIMM17A	0.60991208	1.62E-19
NFE2L2	RNF26	0.60987549	1.63E-19
NFE2L2	PLEKHF2	0.60975856	1.67E-19
NFE2L2	CD46	0.60967987	1.69E-19
NFE2L2	SPRY4	0.60967295	1.69E-19
NFE2L2	ITPRIP	0.60962377	1.71E-19
NFE2L2	NDC1	0.60947151	1.75E-19
NFE2L2	PLEKHA7	0.60939847	1.77E-19
NFE2L2	HMOX2	0.60932992	1.79E-19
NFE2L2	TRMT2B	0.60928303	1.81E-19
NFE2L2	LARS2	0.60881799	1.96E-19
NFE2L2	S1PR3	0.60878718	1.97E-19

NFE2L2	GNPAT	0.6087186	1.99E-19
NFE2L2	TMBIM4	0.60870914	2E-19
NFE2L2	ARHGAP26	0.60868492	2E-19
NFE2L2	PLEKHA5	0.60859488	2.04E-19
NFE2L2	TMTC3	0.60845338	2.09E-19
NFE2L2	SP2	0.6084054	2.1E-19
NFE2L2	GALNS	0.60829689	2.14E-19
NFE2L2	ATP5PB	0.60804546	2.24E-19
NFE2L2	HLA-E	0.60779431	2.33E-19
NFE2L2	LMNB1	0.6075429	2.44E-19
NFE2L2	DRAM1	0.60743665	2.48E-19
NFE2L2	RSPH3	0.60743191	2.48E-19
NFE2L2	PRMT2	0.60730652	2.54E-19
NFE2L2	PNRC1	0.60718033	2.59E-19
NFE2L2	SBNO2	0.6070864	2.64E-19
NFE2L2	ABCB10	0.60702743	2.66E-19
NFE2L2	TK2	0.60694113	2.7E-19
NFE2L2	LUM	0.60687037	2.73E-19
NFE2L2	CLCN3	0.60685604	2.74E-19
NFE2L2	SLC41A2	0.60676304	2.78E-19
NFE2L2	PCSK7	0.60669043	2.82E-19
NFE2L2	C1R	0.60656885	2.88E-19
NFE2L2	PTAFR	0.6064285	2.95E-19
NFE2L2	PRDM1	0.60637476	2.98E-19
NFE2L2	PECR	0.6062085	3.06E-19
NFE2L2	SPARC	0.60607413	3.13E-19
NFE2L2	TRIB2	0.60595867	3.19E-19
NFE2L2	HEXIM1	0.6057649	3.3E-19
NFE2L2	SERINC1	0.60574051	3.31E-19
NFE2L2	NUDCD2	0.60572385	3.32E-19
NFE2L2	PRUNE1	0.60571028	3.33E-19
NFE2L2	PIK3C3	0.60566137	3.36E-19
NFE2L2	EXO5	0.60564589	3.37E-19
NFE2L2	DKK3	0.6055772	3.41E-19
NFE2L2	TULP3	0.60545211	3.48E-19
NFE2L2	SDC3	0.6053001	3.57E-19
NFE2L2	GIMAP2	0.60524662	3.6E-19
NFE2L2	NEK6	0.60495672	3.79E-19
NFE2L2	NUDT3	0.60487018	3.84E-19
NFE2L2	RAB23	0.60475962	3.91E-19
NFE2L2	DSE	0.60468357	3.97E-19
NFE2L2	CRISPLD2	0.60458259	4.03E-19
NFE2L2	EFNA5	0.60452584	4.07E-19
NFE2L2	TMEM33	0.60439641	4.16E-19
NFE2L2	FRS2	0.60436436	4.19E-19
NFE2L2	TFDP2	0.60419363	4.31E-19
NFE2L2	DGKH	0.6041905	4.31E-19
NFE2L2	COL3A1	0.60400222	4.45E-19
NFE2L2	ARF1	0.60395617	4.48E-19
NFE2L2	SLFN11	0.60368757	4.69E-19
NFE2L2	ZMYM2	0.60361551	4.75E-19
NFE2L2	SLC35C1	0.60333138	4.98E-19
NFE2L2	DCAF7	0.60313318	5.15E-19
NFE2L2	CLOCK	0.6030754	5.2E-19
NFE2L2	TUBA1B	0.60305062	5.23E-19
NFE2L2	STARD7	0.60300714	5.26E-19
NFE2L2	ABTB2	0.60299023	5.28E-19
NFE2L2	CHCHD3	0.60294873	5.32E-19

NFE2L2	SH3BP5	0.6028343	5.42E-19
NFE2L2	RAB1B	0.6028056	5.45E-19
NFE2L2	ZCCHC17	0.60280265	5.45E-19
NFE2L2	TRMT10C	0.60273811	5.51E-19
NFE2L2	MAP7D1	0.6027288	5.52E-19
NFE2L2	SLC2A9	0.60271707	5.53E-19
NFE2L2	PTP4A1	0.6026395	5.6E-19
NFE2L2	PCDH1	0.60254172	5.69E-19
NFE2L2	GLI1	0.60251246	5.72E-19
NFE2L2	TNFAIP3	0.60246651	5.76E-19
NFE2L2	THY1	0.60235934	5.87E-19
NFE2L2	PPP1R11	0.60232997	5.9E-19
NFE2L2	MGLL	0.60228249	5.95E-19
NFE2L2	RAD18	0.60222135	6.01E-19
NFE2L2	EFL1	0.60217033	6.06E-19
NFE2L2	PDGFRA	0.60216948	6.06E-19
NFE2L2	ALDH7A1	0.60205953	6.17E-19
NFE2L2	PRIM2	0.60202417	6.21E-19
NFE2L2	ARHGAP31	0.6019754	6.26E-19
NFE2L2	DNM1L	0.60171576	6.54E-19
NFE2L2	GPN3	0.6016191	6.65E-19
NFE2L2	BECN1	0.60140834	6.88E-19
NFE2L2	GNAI2	0.60140134	6.89E-19
NFE2L2	CFL1	0.60134731	6.95E-19
NFE2L2	IPO11	0.60126199	7.06E-19
NFE2L2	NAA30	0.60122635	7.1E-19
NFE2L2	C4orf33	0.60110435	7.24E-19
NFE2L2	TMEM9B	0.60107188	7.28E-19
NFE2L2	RBM18	0.60085571	7.55E-19
NFE2L2	LIPH	0.60082644	7.59E-19
NFE2L2	TVP23B	0.60078342	7.64E-19
NFE2L2	MBTPS2	0.60070411	7.74E-19
NFE2L2	TCF4	0.60061563	7.86E-19
NFE2L2	EMC3	0.60054461	7.95E-19
NFE2L2	HSD17B12	0.60045532	8.07E-19
NFE2L2	SRC	0.6004225	8.12E-19
NFE2L2	SEC11A	0.60029823	8.29E-19
NFE2L2	NIPSNAP3A	0.60018437	8.45E-19
NFE2L2	AGAP1	0.60013975	8.51E-19
NFE2L2	FGD5	0.60004012	8.65E-19
NFE2L2	HMGN1	0.59997372	8.75E-19
NFE2L2	TNRC18	0.59993095	8.81E-19
NFE2L2	RAB13	0.59988113	8.88E-19
NFE2L2	CDK2AP1	0.59971525	9.13E-19
NFE2L2	TTC33	0.59968719	9.18E-19
NFE2L2	CCDC47	0.59958296	9.34E-19
NFE2L2	LMBR1	0.59955603	9.38E-19
NFE2L2	C6orf47	0.59930086	9.78E-19
NFE2L2	INHBA	0.59929705	9.79E-19
NFE2L2	SULF1	0.5992221	9.91E-19
NFE2L2	EID1	0.59921685	9.92E-19
NFE2L2	MCTP1	0.59918713	9.97E-19
NFE2L2	LEPROTL1	0.59911534	1.01E-18
NFE2L2	MOXD1	0.59906437	1.02E-18
NFE2L2	HDAC7	0.59901223	1.03E-18
NFE2L2	HEG1	0.59895657	1.04E-18
NFE2L2	SIPA1L1	0.59889105	1.05E-18
NFE2L2	POLDIP3	0.59870009	1.08E-18

NFE2L2	COL8A1	0.59860202	1.1E-18
NFE2L2	WDR12	0.5985794	1.1E-18
NFE2L2	DST	0.59842265	1.13E-18
NFE2L2	IL18	0.59836997	1.14E-18
NFE2L2	TDP1	0.59815964	1.18E-18
NFE2L2	CDK7	0.59805873	1.2E-18
NFE2L2	ITGB8	0.59790442	1.23E-18
NFE2L2	GLCE	0.59775434	1.26E-18
NFE2L2	SELENON	0.5977438	1.27E-18
NFE2L2	LRRC41	0.59767223	1.28E-18
NFE2L2	SYK	0.59757806	1.3E-18
NFE2L2	TUBB	0.5975142	1.32E-18
NFE2L2	NUP62	0.59734092	1.35E-18
NFE2L2	UNC5B	0.59732027	1.36E-18
NFE2L2	AASS	0.59728997	1.37E-18
NFE2L2	PDHB	0.59727529	1.37E-18
NFE2L2	LMO4	0.59721905	1.38E-18
NFE2L2	MPZL3	0.5972144	1.38E-18
NFE2L2	LSM12	0.5970446	1.42E-18
NFE2L2	ATG9A	0.59691274	1.45E-18
NFE2L2	POFUT1	0.59684252	1.47E-18
NFE2L2	ZFP90	0.59683503	1.47E-18
NFE2L2	TRANK1	0.59680183	1.48E-18
NFE2L2	FBXL5	0.59676739	1.49E-18
NFE2L2	OAT	0.59664237	1.52E-18
NFE2L2	BAG3	0.5965717	1.54E-18
NFE2L2	SYTL4	0.59642358	1.57E-18
NFE2L2	SLC9A6	0.59635873	1.59E-18
NFE2L2	MLKL	0.59631846	1.6E-18
NFE2L2	TMEM167A	0.5963071	1.61E-18
NFE2L2	IGSF3	0.59622967	1.63E-18
NFE2L2	ZNF174	0.59610713	1.66E-18
NFE2L2	GALNT3	0.59610562	1.66E-18
NFE2L2	SPCS2	0.59603354	1.68E-18
NFE2L2	IDH3A	0.59591257	1.71E-18
NFE2L2	ENOX2	0.59590698	1.71E-18
NFE2L2	COL5A1	0.5959	1.72E-18
NFE2L2	PREP	0.5958861	1.72E-18
NFE2L2	FKBP7	0.59582536	1.74E-18
NFE2L2	AKIRIN2	0.59581369	1.74E-18
NFE2L2	SNUPN	0.59575925	1.76E-18
NFE2L2	HOMER1	0.59567276	1.78E-18
NFE2L2	BTN2A2	0.59564833	1.79E-18
NFE2L2	HMGXB3	0.59553125	1.82E-18
NFE2L2	SERTAD4	0.59548529	1.84E-18
NFE2L2	TRNAU1AP	0.59534307	1.88E-18
NFE2L2	LIPA	0.59527963	1.9E-18
NFE2L2	EIF5A2	0.59525243	1.91E-18
NFE2L2	METTL18	0.59502537	1.98E-18
NFE2L2	ADAM28	0.59501951	1.98E-18
NFE2L2	SYAP1	0.59498147	2E-18
NFE2L2	CDKN1B	0.59489954	2.02E-18
NFE2L2	EPSTI1	0.59480853	2.05E-18
NFE2L2	BBS7	0.59480076	2.06E-18
NFE2L2	MKLN1	0.59472961	2.08E-18
NFE2L2	SMIM12	0.59465202	2.11E-18
NFE2L2	KIF16B	0.59459024	2.13E-18
NFE2L2	CRIPT	0.59457555	2.13E-18

NFE2L2	CASP1	0.59454005	2.14E-18
NFE2L2	DIP2C	0.59451432	2.15E-18
NFE2L2	IFIT5	0.59445788	2.17E-18
NFE2L2	HMCES	0.59435624	2.21E-18
NFE2L2	MTFMT	0.59419439	2.27E-18
NFE2L2	THBS1	0.59407942	2.31E-18
NFE2L2	MSRB3	0.59404096	2.33E-18
NFE2L2	HDAC9	0.59400805	2.34E-18
NFE2L2	RRM2B	0.59397945	2.35E-18
NFE2L2	GMPR2	0.59389228	2.38E-18
NFE2L2	DAB2IP	0.59376738	2.43E-18
NFE2L2	WASHC5	0.59371264	2.45E-18
NFE2L2	MUL1	0.5935737	2.51E-18
NFE2L2	NCSTN	0.59357219	2.51E-18
NFE2L2	DAG1	0.59356263	2.52E-18
NFE2L2	SRRD	0.59354317	2.52E-18
NFE2L2	VPS29	0.59353779	2.53E-18
NFE2L2	RAB28	0.5930524	2.73E-18
NFE2L2	TRIM59	0.59303455	2.74E-18
NFE2L2	MOB3B	0.59288173	2.81E-18
NFE2L2	SHROOM3	0.59276573	2.86E-18
NFE2L2	SUCLA2	0.59272853	2.88E-18
NFE2L2	CD93	0.59271674	2.89E-18
NFE2L2	RAB12	0.59264863	2.92E-18
NFE2L2	GLIPR1	0.59264245	2.92E-18
NFE2L2	DCK	0.59263647	2.92E-18
NFE2L2	ARL8A	0.59254428	2.97E-18
NFE2L2	UBE2V1	0.59243011	3.02E-18
NFE2L2	RTF2	0.59241615	3.03E-18
NFE2L2	RASSF5	0.59236462	3.06E-18
NFE2L2	OGDH	0.59229414	3.09E-18
NFE2L2	PMP22	0.59225789	3.11E-18
NFE2L2	TOR1B	0.59205554	3.21E-18
NFE2L2	GRB2	0.59202799	3.23E-18
NFE2L2	PBDC1	0.59188783	3.3E-18
NFE2L2	NREP	0.59175789	3.37E-18
NFE2L2	CCDC68	0.59172568	3.39E-18
NFE2L2	ZNF586	0.59172344	3.39E-18
NFE2L2	RFTN1	0.59164112	3.44E-18
NFE2L2	NFE2L3	0.59151417	3.51E-18
NFE2L2	RIOK3	0.5915096	3.51E-18
NFE2L2	BPGM	0.59127893	3.64E-18
NFE2L2	OSTM1	0.59120533	3.69E-18
NFE2L2	DDX60	0.59098981	3.82E-18
NFE2L2	SRF	0.59092566	3.86E-18
NFE2L2	ACER3	0.59072389	3.99E-18
NFE2L2	HNRNPA1	0.59070704	4E-18
NFE2L2	ITFG1	0.5905709	4.09E-18
NFE2L2	SORL1	0.59031243	4.26E-18
NFE2L2	ESD	0.5902578	4.3E-18
NFE2L2	SMAD5	0.59022501	4.32E-18
NFE2L2	FOXO1	0.59010471	4.4E-18
NFE2L2	TMEM140	0.59003515	4.45E-18
NFE2L2	GBE1	0.58997164	4.5E-18
NFE2L2	GEMIN5	0.58961655	4.76E-18
NFE2L2	ABLIM3	0.58960021	4.78E-18
NFE2L2	CLDN1	0.58957119	4.8E-18
NFE2L2	LBH	0.58956991	4.8E-18

NFE2L2	STON1	0.58950849	4.85E-18
NFE2L2	CNN3	0.58950443	4.85E-18
NFE2L2	TCAIM	0.58948436	4.87E-18
NFE2L2	SUPT16H	0.58947237	4.88E-18
NFE2L2	ZNF227	0.58937935	4.95E-18
NFE2L2	EXOC4	0.58924124	5.06E-18
NFE2L2	HDGF	0.58923903	5.06E-18
NFE2L2	STAT5A	0.58909021	5.18E-18
NFE2L2	TMEM30B	0.58906342	5.21E-18
NFE2L2	CACYBP	0.58905285	5.22E-18
NFE2L2	ZMAT3	0.58896184	5.29E-18
NFE2L2	STIP1	0.58879706	5.43E-18
NFE2L2	KIFAP3	0.58875084	5.47E-18
NFE2L2	PDLIM1	0.58859271	5.61E-18
NFE2L2	BTG3	0.58852927	5.67E-18
NFE2L2	NDUFB5	0.58843534	5.76E-18
NFE2L2	NECTIN3	0.58838776	5.8E-18
NFE2L2	RTCB	0.58819381	5.98E-18
NFE2L2	ZBED1	0.58813992	6.04E-18
NFE2L2	NTM	0.58812862	6.05E-18
NFE2L2	SCAMP2	0.58811874	6.06E-18
NFE2L2	OSER1	0.58810299	6.07E-18
NFE2L2	SRP14	0.58809427	6.08E-18
NFE2L2	DEPTOR	0.58800829	6.16E-18
NFE2L2	ECM2	0.58800348	6.17E-18
NFE2L2	SPOP	0.58787262	6.3E-18
NFE2L2	EHF	0.58786311	6.31E-18
NFE2L2	AK2	0.5876984	6.48E-18
NFE2L2	AKIP1	0.5875231	6.66E-18
NFE2L2	UBE2V2	0.58726388	6.94E-18
NFE2L2	KIAA1671	0.587137	7.08E-18
NFE2L2	TIAM2	0.58711207	7.11E-18
NFE2L2	TIMP3	0.58710158	7.12E-18
NFE2L2	SNAP29	0.58698396	7.26E-18
NFE2L2	TANC2	0.58680378	7.47E-18
NFE2L2	CALM1	0.58676824	7.51E-18
NFE2L2	COL15A1	0.58674045	7.55E-18
NFE2L2	IRAK2	0.58656418	7.76E-18
NFE2L2	COPS5	0.58656191	7.76E-18
NFE2L2	RPF1	0.58652207	7.81E-18
NFE2L2	TLR3	0.58643659	7.92E-18
NFE2L2	THBS2	0.58629833	8.1E-18
NFE2L2	EMC1	0.58621552	8.2E-18
NFE2L2	SLC16A1	0.58596058	8.54E-18
NFE2L2	TSHZ3	0.58592633	8.59E-18
NFE2L2	PCNX1	0.58587614	8.66E-18
NFE2L2	TSHZ2	0.58577637	8.8E-18
NFE2L2	MED18	0.5857353	8.85E-18
NFE2L2	RNPS1	0.58553728	9.13E-18
NFE2L2	NFE2L1	0.58542817	9.29E-18
NFE2L2	ARFIP1	0.5854235	9.3E-18
NFE2L2	CDC123	0.58534061	9.42E-18
NFE2L2	ITGBL1	0.58525975	9.55E-18
NFE2L2	CUL1	0.58517753	9.67E-18
NFE2L2	PCDH18	0.58509679	9.79E-18
NFE2L2	PI4K2B	0.58505578	9.86E-18
NFE2L2	EFEMP1	0.58486674	1.02E-17
NFE2L2	PRKAR1A	0.58474553	1.04E-17

NFE2L2	SORD	0.58443601	1.09E-17
NFE2L2	KCTD1	0.58438218	1.1E-17
NFE2L2	COL10A1	0.58417539	1.13E-17
NFE2L2	DIP2B	0.5841124	1.14E-17
NFE2L2	ZDHHC13	0.58408839	1.15E-17
NFE2L2	SLU7	0.58402223	1.16E-17
NFE2L2	RMDN1	0.58399376	1.17E-17
NFE2L2	SPATS2	0.58398538	1.17E-17
NFE2L2	HINT3	0.58395828	1.17E-17
NFE2L2	FAM3C	0.58376119	1.21E-17
NFE2L2	SUCLG2	0.58372961	1.22E-17
NFE2L2	PA2G4	0.58366078	1.23E-17
NFE2L2	KLHL12	0.58363334	1.23E-17
NFE2L2	PRELP	0.58362772	1.24E-17
NFE2L2	DYNLT3	0.5835363	1.25E-17
NFE2L2	RNF8	0.58348279	1.26E-17
NFE2L2	ST3GAL1	0.58348002	1.26E-17
NFE2L2	CPED1	0.58338423	1.28E-17
NFE2L2	PTPRA	0.58338065	1.28E-17
NFE2L2	HS1BP3	0.58324703	1.31E-17
NFE2L2	BBS2	0.58313029	1.34E-17
NFE2L2	FAM210A	0.58296537	1.37E-17
NFE2L2	BMP2K	0.58294535	1.38E-17
NFE2L2	RHOC	0.58291452	1.38E-17
NFE2L2	LPIN2	0.58284997	1.4E-17
NFE2L2	GRK2	0.58273222	1.42E-17
NFE2L2	AGO2	0.58252334	1.47E-17
NFE2L2	OLFML1	0.58251998	1.47E-17
NFE2L2	EPDR1	0.58235531	1.51E-17
NFE2L2	NUP133	0.58234131	1.51E-17
NFE2L2	PIGX	0.58226396	1.53E-17
NFE2L2	MMP14	0.58207932	1.58E-17
NFE2L2	RBBP9	0.58189919	1.62E-17
NFE2L2	BLOC1S2	0.58171188	1.67E-17
NFE2L2	RNF144B	0.58167682	1.68E-17
NFE2L2	ZNF462	0.58157473	1.7E-17
NFE2L2	CDKL1	0.58153638	1.71E-17
NFE2L2	AP2M1	0.58151894	1.72E-17
NFE2L2	POC1B	0.58150069	1.72E-17
NFE2L2	LOX	0.58145507	1.74E-17
NFE2L2	TRAF7	0.58110444	1.83E-17
NFE2L2	HTRA2	0.58109919	1.84E-17
NFE2L2	ZCCHC10	0.58108203	1.84E-17
NFE2L2	CTSZ	0.58107555	1.84E-17
NFE2L2	AKAP12	0.58107444	1.84E-17
NFE2L2	SRPX2	0.58104253	1.85E-17
NFE2L2	ELP3	0.58095982	1.88E-17
NFE2L2	GNPDA1	0.58094714	1.88E-17
NFE2L2	MEAF6	0.58090106	1.89E-17
NFE2L2	LMBRD1	0.58082118	1.92E-17
NFE2L2	ADCY7	0.58080212	1.92E-17
NFE2L2	PIP4K2C	0.58071346	1.95E-17
NFE2L2	TNIP1	0.58063469	1.97E-17
NFE2L2	ZEB2	0.58062523	1.98E-17
NFE2L2	TFCP2	0.58045764	2.03E-17
NFE2L2	AP3B1	0.58043364	2.04E-17
NFE2L2	SVIP	0.58041721	2.04E-17
NFE2L2	UBAC2	0.58023994	2.1E-17

NFE2L2	C1orf43	0.58023336	2.1E-17
NFE2L2	TOR1A	0.58021906	2.11E-17
NFE2L2	MTERF3	0.58021319	2.11E-17
NFE2L2	PLA2G15	0.58010817	2.14E-17
NFE2L2	MX2	0.58009862	2.15E-17
NFE2L2	PRRG4	0.57989105	2.22E-17
NFE2L2	XIAP	0.57970977	2.28E-17
NFE2L2	PDGFB	0.57958778	2.32E-17
NFE2L2	ZNF468	0.5794998	2.35E-17
NFE2L2	STOM	0.57945306	2.37E-17
NFE2L2	NSD3	0.57941171	2.39E-17
NFE2L2	GOLT1B	0.57935949	2.41E-17
NFE2L2	SLC25A44	0.57935277	2.41E-17
NFE2L2	FAM102A	0.57933842	2.41E-17
NFE2L2	RNF7	0.57932271	2.42E-17
NFE2L2	SUMF1	0.57925825	2.44E-17
NFE2L2	PSMD11	0.57908409	2.51E-17
NFE2L2	MAF	0.57906186	2.52E-17
NFE2L2	CBX1	0.57904487	2.53E-17
NFE2L2	SLC25A12	0.57892569	2.57E-17
NFE2L2	SMS	0.57884549	2.61E-17
NFE2L2	TCAF1	0.57876395	2.64E-17
NFE2L2	HRH1	0.57871204	2.66E-17
NFE2L2	RCC1	0.57842624	2.78E-17
NFE2L2	TRIM8	0.57841994	2.78E-17
NFE2L2	RHPN2	0.57821311	2.87E-17
NFE2L2	PCSK6	0.57811497	2.92E-17
NFE2L2	GNB4	0.57809103	2.93E-17
NFE2L2	EIF1B	0.57805008	2.95E-17
NFE2L2	GRPEL2	0.57780342	3.06E-17
NFE2L2	NBL1	0.57780205	3.06E-17
NFE2L2	DUSP7	0.57777475	3.08E-17
NFE2L2	PRR14L	0.57776508	3.08E-17
NFE2L2	PFKFB3	0.57757023	3.17E-17
NFE2L2	MNAT1	0.5773194	3.3E-17
NFE2L2	DPYSL3	0.57712422	3.4E-17
NFE2L2	OPN3	0.57710576	3.41E-17
NFE2L2	TNFSF12	0.57708343	3.42E-17
NFE2L2	TMEM138	0.57698521	3.47E-17
NFE2L2	HNRNPAB	0.57683982	3.55E-17
NFE2L2	USP12	0.57670437	3.63E-17
NFE2L2	HEATR5A	0.57669203	3.63E-17
NFE2L2	AOC3	0.57666974	3.65E-17
NFE2L2	TBC1D25	0.57664785	3.66E-17
NFE2L2	CNOT4	0.5766093	3.68E-17
NFE2L2	STARD3NL	0.57659111	3.69E-17
NFE2L2	MTX2	0.57652286	3.73E-17
NFE2L2	ASCC3	0.57645216	3.77E-17
NFE2L2	VDAC2	0.57638122	3.81E-17
NFE2L2	OAS3	0.57610779	3.97E-17
NFE2L2	KIAA0232	0.57608999	3.99E-17
NFE2L2	BTBD9	0.5760774	3.99E-17
NFE2L2	ZFPM2	0.57598554	4.05E-17
NFE2L2	SH2B3	0.57595821	4.07E-17
NFE2L2	FBXL12	0.57577896	4.18E-17
NFE2L2	RAD1	0.5757428	4.2E-17
NFE2L2	BDH2	0.5757159	4.22E-17
NFE2L2	PON2	0.57558033	4.31E-17

NFE2L2	HYAL2	0.57558016	4.31E-17
NFE2L2	DDX23	0.57547984	4.38E-17
NFE2L2	DHX35	0.57540972	4.42E-17
NFE2L2	SPIDR	0.57526476	4.52E-17
NFE2L2	CCDC59	0.57526413	4.52E-17
NFE2L2	PHLDB1	0.57526013	4.53E-17
NFE2L2	TGFB1I1	0.57515361	4.6E-17
NFE2L2	GPBP1	0.57514603	4.61E-17
NFE2L2	SAMD12	0.57512823	4.62E-17
NFE2L2	U2AF2	0.57502571	4.69E-17
NFE2L2	SMAD7	0.57488208	4.8E-17
NFE2L2	NUDT4B	0.57488012	4.8E-17
NFE2L2	NET1	0.57464762	4.97E-17
NFE2L2	PLAT	0.57462441	4.99E-17
NFE2L2	ANXA2	0.57446043	5.11E-17
NFE2L2	SLC25A17	0.57432196	5.22E-17
NFE2L2	PPCS	0.57412079	5.39E-17
NFE2L2	TNFRSF21	0.57411685	5.39E-17
NFE2L2	PRKAG2	0.57406228	5.43E-17
NFE2L2	TTYH3	0.57404617	5.45E-17
NFE2L2	PHF5A	0.57404068	5.45E-17
NFE2L2	JPT2	0.57403822	5.45E-17
NFE2L2	ANAPC13	0.57385142	5.61E-17
NFE2L2	TEFM	0.57364637	5.79E-17
NFE2L2	CPXM2	0.57362225	5.81E-17
NFE2L2	ITGB6	0.57361899	5.81E-17
NFE2L2	LRRC8C	0.57349514	5.92E-17
NFE2L2	GSTCD	0.57335127	6.06E-17
NFE2L2	MB21D2	0.57314832	6.25E-17
NFE2L2	CAPN1	0.57309633	6.29E-17
NFE2L2	APOL3	0.57301537	6.37E-17
NFE2L2	KDM5A	0.57278368	6.6E-17
NFE2L2	MSANTD3	0.57270543	6.68E-17
NFE2L2	CTDSPL	0.57264207	6.74E-17
NFE2L2	GTPBP10	0.57250578	6.88E-17
NFE2L2	MTCH2	0.57246185	6.93E-17
NFE2L2	SLC4A7	0.57243499	6.96E-17
NFE2L2	RAB38	0.57243231	6.96E-17
NFE2L2	OXNAD1	0.57234212	7.06E-17
NFE2L2	NYNRIN	0.57227713	7.13E-17
NFE2L2	HIBADH	0.57227222	7.13E-17
NFE2L2	ATXN7L3B	0.57203663	7.39E-17
NFE2L2	CANX	0.57202956	7.4E-17
NFE2L2	AAMP	0.57184386	7.61E-17
NFE2L2	SNN	0.57183104	7.63E-17
NFE2L2	ZNF552	0.57181571	7.64E-17
NFE2L2	SMG8	0.57151819	7.99E-17
NFE2L2	GNB5	0.57142586	8.11E-17
NFE2L2	PSMA4	0.57136655	8.18E-17
NFE2L2	SORT1	0.57123356	8.35E-17
NFE2L2	HIGD1A	0.57119714	8.39E-17
NFE2L2	AKTIP	0.57112829	8.48E-17
NFE2L2	DPP9	0.57105389	8.58E-17
NFE2L2	PAIP1	0.5709463	8.72E-17
NFE2L2	CHST15	0.57083357	8.86E-17
NFE2L2	UACA	0.57080815	8.9E-17
NFE2L2	JARID2	0.57078115	8.94E-17
NFE2L2	SAR1B	0.57050995	9.31E-17

NFE2L2	GPRC5A	0.57045047	9.39E-17
NFE2L2	ZBTB4	0.57035298	9.53E-17
NFE2L2	STEAP1	0.57035282	9.53E-17
NFE2L2	PPT1	0.57033895	9.55E-17
NFE2L2	DENR	0.57015787	9.81E-17
NFE2L2	RCOR1	0.57013699	9.84E-17
NFE2L2	ICAM1	0.57008982	9.91E-17
NFE2L2	POF1B	0.57008329	9.92E-17
NFE2L2	EXOC2	0.57003995	9.99E-17
NFE2L2	KDM1B	0.56993179	1.02E-16
NFE2L2	WBP11	0.56988431	1.02E-16
NFE2L2	CNOT7	0.56983398	1.03E-16
NFE2L2	SMIM30	0.56983324	1.03E-16
NFE2L2	NUCKS1	0.56974432	1.04E-16
NFE2L2	CNN2	0.5697167	1.05E-16
NFE2L2	HMCN1	0.56969277	1.05E-16
NFE2L2	CAVIN1	0.56962511	1.06E-16
NFE2L2	HDAC1	0.56962006	1.06E-16
NFE2L2	HNRNPA0	0.56959232	1.07E-16
NFE2L2	PLIN3	0.56959221	1.07E-16
NFE2L2	ACTR1A	0.56953713	1.08E-16
NFE2L2	ZBTB22	0.56950259	1.08E-16
NFE2L2	CCT8	0.56944873	1.09E-16
NFE2L2	FILIP1L	0.56932132	1.11E-16
NFE2L2	DIDO1	0.56920589	1.13E-16
NFE2L2	HOXA4	0.56915357	1.14E-16
NFE2L2	SIPA1L2	0.56914323	1.14E-16
NFE2L2	RIC8A	0.56912384	1.15E-16
NFE2L2	TSEN15	0.56902265	1.16E-16
NFE2L2	PNP	0.56896554	1.17E-16
NFE2L2	ALDH9A1	0.56895926	1.17E-16
NFE2L2	WTIP	0.56894332	1.18E-16
NFE2L2	FUCA1	0.56889833	1.19E-16
NFE2L2	PGK1	0.56883706	1.2E-16
NFE2L2	GALNT1	0.56875566	1.21E-16
NFE2L2	DPY30	0.56867739	1.23E-16
NFE2L2	PELO	0.56859558	1.24E-16
NFE2L2	MYLK	0.56858089	1.24E-16
NFE2L2	THSD4	0.56855671	1.25E-16
NFE2L2	PRR13	0.56846611	1.26E-16
NFE2L2	RAMAC	0.56830955	1.29E-16
NFE2L2	RHEB	0.56830417	1.3E-16
NFE2L2	RHBDL2	0.56816	1.32E-16
NFE2L2	CRCP	0.56800103	1.36E-16
NFE2L2	METTL4	0.56798029	1.36E-16
NFE2L2	ZFP36	0.56796238	1.36E-16
NFE2L2	ASH2L	0.56794528	1.37E-16
NFE2L2	AK4	0.56788347	1.38E-16
NFE2L2	OTULIN	0.56782291	1.39E-16
NFE2L2	IFIT2	0.56781531	1.39E-16
NFE2L2	THAP11	0.56773281	1.41E-16
NFE2L2	ASPN	0.56772776	1.41E-16
NFE2L2	CLEC16A	0.56765882	1.43E-16
NFE2L2	PPP1R9B	0.56765447	1.43E-16
NFE2L2	TNFAIP8L1	0.5676247	1.43E-16
NFE2L2	C1GALT1C1	0.56761115	1.44E-16
NFE2L2	TIMP2	0.56761089	1.44E-16
NFE2L2	ADGRA2	0.56755104	1.45E-16

NFE2L2	PPIL3	0.56753577	1.45E-16
NFE2L2	CD109	0.56752003	1.46E-16
NFE2L2	STK39	0.56727288	1.51E-16
NFE2L2	CD164	0.56725158	1.52E-16
NFE2L2	NR2F1	0.56723282	1.52E-16
NFE2L2	PER2	0.56722299	1.52E-16
NFE2L2	ENTPD5	0.56719531	1.53E-16
NFE2L2	SH3BP2	0.56710926	1.55E-16
NFE2L2	CDKN2AIP	0.56707618	1.56E-16
NFE2L2	CYTH1	0.56705647	1.56E-16
NFE2L2	IK	0.56702399	1.57E-16
NFE2L2	FASTKD5	0.5669986	1.57E-16
NFE2L2	YIPF1	0.56696073	1.58E-16
NFE2L2	FP565260.3	0.56690135	1.6E-16
NFE2L2	PTGES3	0.56689528	1.6E-16
NFE2L2	SLC16A2	0.56685264	1.61E-16
NFE2L2	ARAP3	0.56676205	1.63E-16
NFE2L2	RAC1	0.5666577	1.66E-16
NFE2L2	PARP9	0.56657311	1.68E-16
NFE2L2	FARP2	0.56657071	1.68E-16
NFE2L2	SYNJ2	0.56638617	1.72E-16
NFE2L2	UNG	0.56635443	1.73E-16
NFE2L2	GALNT7	0.56633786	1.74E-16
NFE2L2	KIF26B	0.56598076	1.83E-16
NFE2L2	URI1	0.56593986	1.84E-16
NFE2L2	SLC7A7	0.56589969	1.85E-16
NFE2L2	WNK1	0.56580874	1.88E-16
NFE2L2	LSM6	0.5657938	1.88E-16
NFE2L2	PPARD	0.56576052	1.89E-16
NFE2L2	HSP90AA1	0.5657324	1.9E-16
NFE2L2	SAP30	0.56569209	1.91E-16
NFE2L2	ZNF521	0.56567257	1.92E-16
NFE2L2	TIMM23	0.56561119	1.93E-16
NFE2L2	LRRC8D	0.56559885	1.94E-16
NFE2L2	CLIP4	0.56543297	1.99E-16
NFE2L2	TYW1	0.56533491	2.01E-16
NFE2L2	MEIS2	0.56517272	2.06E-16
NFE2L2	SLC38A1	0.56507815	2.09E-16
NFE2L2	KCTD11	0.56494969	2.13E-16
NFE2L2	PLOD2	0.5648967	2.15E-16
NFE2L2	DSG2	0.56487341	2.16E-16
NFE2L2	STN1	0.56448573	2.28E-16
NFE2L2	TPGS2	0.56421149	2.38E-16
NFE2L2	GPNMB	0.56405289	2.43E-16
NFE2L2	ADGRE5	0.56398313	2.46E-16
NFE2L2	LRRC8A	0.56389104	2.49E-16
NFE2L2	COMMD8	0.56387011	2.5E-16
NFE2L2	TM2D2	0.5638204	2.52E-16
NFE2L2	CALM3	0.56368248	2.57E-16
NFE2L2	ECT2	0.56363133	2.59E-16
NFE2L2	PAICS	0.56340593	2.68E-16
NFE2L2	TAP2	0.56321278	2.76E-16
NFE2L2	PXDN	0.56312527	2.79E-16
NFE2L2	MICU2	0.56310662	2.8E-16
NFE2L2	KLF4	0.56310366	2.8E-16
NFE2L2	IL15RA	0.56299182	2.85E-16
NFE2L2	PAQR7	0.5629344	2.87E-16
NFE2L2	RAN	0.56268524	2.98E-16

NFE2L2	JAM3	0.56267048	2.98E-16
NFE2L2	CSRP1	0.56256251	3.03E-16
NFE2L2	UHRF1BP1L	0.56252256	3.05E-16
NFE2L2	CCN4	0.56247729	3.07E-16
NFE2L2	SLBP	0.56238559	3.11E-16
NFE2L2	PPP1R18	0.56228638	3.16E-16
NFE2L2	RPS6KA1	0.56206664	3.26E-16
NFE2L2	DARS2	0.56206403	3.26E-16
NFE2L2	TBC1D10A	0.5620596	3.26E-16
NFE2L2	COX7A2L	0.5620037	3.29E-16
NFE2L2	CHST14	0.56196076	3.31E-16
NFE2L2	MAFF	0.56193087	3.32E-16
NFE2L2	RALGPS2	0.56166982	3.45E-16
NFE2L2	CNOT6L	0.56165853	3.46E-16
NFE2L2	ZC3H18	0.56157523	3.5E-16
NFE2L2	ATP6V0E1	0.56138071	3.6E-16
NFE2L2	SPOCK1	0.56119651	3.7E-16
NFE2L2	PKP2	0.56119434	3.7E-16
NFE2L2	MACC1	0.56088425	3.87E-16
NFE2L2	NUP37	0.56085413	3.89E-16
NFE2L2	LMO7	0.56079196	3.93E-16
NFE2L2	HERC3	0.56073286	3.96E-16
NFE2L2	POT1	0.56035727	4.18E-16
NFE2L2	EIF4E2	0.56014537	4.31E-16
NFE2L2	MGME1	0.56001637	4.4E-16
NFE2L2	TM9SF4	0.56000184	4.4E-16
NFE2L2	IRF1	0.55999086	4.41E-16
NFE2L2	SPART	0.55999078	4.41E-16
NFE2L2	PTGIS	0.55996425	4.43E-16
NFE2L2	RNF34	0.5598828	4.48E-16
NFE2L2	SPON1	0.55983604	4.51E-16
NFE2L2	PIGW	0.55975539	4.56E-16
NFE2L2	COL1A2	0.55972092	4.59E-16
NFE2L2	FAM32A	0.55937641	4.82E-16
NFE2L2	PXN	0.55931714	4.86E-16
NFE2L2	PHF23	0.55929324	4.88E-16
NFE2L2	AFTPH	0.55923841	4.92E-16
NFE2L2	FTSJ3	0.55913649	4.99E-16
NFE2L2	ADGRL2	0.55896055	5.12E-16
NFE2L2	MMD	0.55886359	5.19E-16
NFE2L2	PRKACA	0.55879423	5.25E-16
NFE2L2	COTL1	0.55870167	5.32E-16
NFE2L2	COX20	0.55870052	5.32E-16
NFE2L2	TLCD2	0.55856938	5.42E-16
NFE2L2	NSFL1C	0.55850313	5.47E-16
NFE2L2	ENOPH1	0.55846079	5.51E-16
NFE2L2	ABI3BP	0.55839329	5.56E-16
NFE2L2	MAPK7	0.55832683	5.61E-16
NFE2L2	NAB2	0.55819129	5.73E-16
NFE2L2	DNASE2	0.55813812	5.77E-16
NFE2L2	CDKN2B	0.55800291	5.88E-16
NFE2L2	RAB32	0.55799855	5.89E-16
NFE2L2	SLC30A5	0.55797914	5.9E-16
NFE2L2	ENY2	0.55794287	5.93E-16
NFE2L2	PRXL2C	0.55792515	5.95E-16
NFE2L2	RARG	0.55784608	6.02E-16
NFE2L2	GALM	0.55784321	6.02E-16
NFE2L2	LPGAT1	0.55782575	6.04E-16

NFE2L2	SMU1	0.55782147	6.04E-16
NFE2L2	EIF3H	0.55776123	6.09E-16
NFE2L2	BORCS5	0.55758182	6.25E-16
NFE2L2	PRKCA	0.55745769	6.36E-16
NFE2L2	OCIAD1	0.55739305	6.42E-16
NFE2L2	PLEKHO2	0.55732192	6.49E-16
NFE2L2	BPTF	0.55730934	6.5E-16
NFE2L2	MRPS27	0.55729235	6.52E-16
NFE2L2	PPM1M	0.55726958	6.54E-16
NFE2L2	PDLIM3	0.557183	6.62E-16
NFE2L2	SDHB	0.55699819	6.8E-16
NFE2L2	PERP	0.55695754	6.84E-16
NFE2L2	SH3PXD2B	0.55691078	6.89E-16
NFE2L2	PLAA	0.55681409	6.98E-16
NFE2L2	HBEGF	0.55669225	7.11E-16
NFE2L2	PLB1	0.55666451	7.13E-16
NFE2L2	MRFAP1L1	0.55662953	7.17E-16
NFE2L2	PEF1	0.55637147	7.44E-16
NFE2L2	ANO10	0.55629058	7.53E-16
NFE2L2	CTSK	0.55626229	7.56E-16
NFE2L2	VWA2	0.55621723	7.61E-16
NFE2L2	MFGE8	0.55610445	7.73E-16
NFE2L2	TGM2	0.55608036	7.76E-16
NFE2L2	RERG	0.55596984	7.88E-16
NFE2L2	THBD	0.55592868	7.93E-16
NFE2L2	MSANTD3-TMEFF1	0.55589924	7.96E-16
NFE2L2	FERMT2	0.55587519	7.99E-16
NFE2L2	POLR3GL	0.55578269	8.1E-16
NFE2L2	PRNP	0.55569762	8.2E-16
NFE2L2	NUMBL	0.55558308	8.33E-16
NFE2L2	IPO9	0.55539363	8.56E-16
NFE2L2	PIK3C2B	0.55519488	8.81E-16
NFE2L2	TMBIM6	0.55492405	9.15E-16
NFE2L2	ARHGEF2	0.5549128	9.17E-16
NFE2L2	TSPAN6	0.55470041	9.45E-16
NFE2L2	UGGT1	0.55465971	9.51E-16
NFE2L2	HELZ2	0.55460922	9.58E-16
NFE2L2	UPRT	0.55452791	9.69E-16
NFE2L2	SEC14L2	0.55442908	9.82E-16
NFE2L2	USP32	0.55440421	9.86E-16
NFE2L2	MAP3K8	0.55420641	1.01E-15
NFE2L2	AP3M2	0.5541678	1.02E-15
NFE2L2	PLXDC1	0.55406867	1.03E-15
NFE2L2	MCC	0.55400169	1.04E-15
NFE2L2	MBOAT2	0.55396367	1.05E-15
NFE2L2	CA13	0.55393363	1.05E-15
NFE2L2	MRAS	0.5538209	1.07E-15
NFE2L2	CYP1B1	0.55375799	1.08E-15
NFE2L2	FAT1	0.55353456	1.12E-15
NFE2L2	COL1A1	0.55352315	1.12E-15
NFE2L2	TRAF3	0.55350543	1.12E-15
NFE2L2	FMN1	0.55346034	1.13E-15
NFE2L2	CMTM3	0.55343222	1.13E-15
NFE2L2	SNAPC5	0.55337987	1.14E-15
NFE2L2	PODN	0.55334407	1.15E-15
NFE2L2	PYGO2	0.55324141	1.16E-15
NFE2L2	ASPH	0.55321945	1.17E-15
NFE2L2	FLVCR2	0.5531599	1.18E-15

NFE2L2	RNF5	0.55311332	1.18E-15
NFE2L2	MVP	0.5530772	1.19E-15
NFE2L2	GAS7	0.5528556	1.23E-15
NFE2L2	TNFSF13B	0.55267981	1.26E-15
NFE2L2	MTHFD1L	0.55258233	1.28E-15
NFE2L2	C11orf68	0.55250929	1.29E-15
NFE2L2	PSMF1	0.55239116	1.31E-15
NFE2L2	TRIM21	0.55234191	1.32E-15
NFE2L2	ARL4C	0.55203799	1.38E-15
NFE2L2	MYL12B	0.55197403	1.39E-15
NFE2L2	COL8A2	0.55162669	1.46E-15
NFE2L2	DGCR2	0.55147516	1.49E-15
NFE2L2	TPRG1L	0.55124409	1.54E-15
NFE2L2	GBP3	0.55115919	1.56E-15
NFE2L2	SMC2	0.55104479	1.59E-15
NFE2L2	NSMCE2	0.55098295	1.6E-15
NFE2L2	TGFB2	0.55080231	1.64E-15
NFE2L2	ABCF2	0.55070034	1.67E-15
NFE2L2	GSKIP	0.55069294	1.67E-15
NFE2L2	CD59	0.55060982	1.69E-15
NFE2L2	GRWD1	0.55056455	1.7E-15
NFE2L2	MED8	0.55051679	1.71E-15
NFE2L2	BICD2	0.55047841	1.72E-15
NFE2L2	TLR4	0.55034127	1.75E-15
NFE2L2	ITPKB	0.55026412	1.77E-15
NFE2L2	VDAC1	0.55023183	1.78E-15
NFE2L2	SEH1L	0.55011326	1.81E-15
NFE2L2	PIAS1	0.55002295	1.83E-15
NFE2L2	GREM1	0.54987296	1.87E-15
NFE2L2	SLC26A2	0.54985929	1.88E-15
NFE2L2	EGR2	0.54983993	1.88E-15
NFE2L2	MOB3A	0.54977147	1.9E-15
NFE2L2	APOL2	0.54969257	1.92E-15
NFE2L2	EPHB4	0.54943737	1.99E-15
NFE2L2	ABHD3	0.54941417	2E-15
NFE2L2	SYNPO	0.54939448	2E-15
NFE2L2	PRR16	0.54928009	2.03E-15
NFE2L2	TAP1	0.54920004	2.06E-15
NFE2L2	DENND3	0.54912595	2.08E-15
NFE2L2	BCL2L1	0.54909845	2.09E-15
NFE2L2	SLC38A6	0.54906089	2.1E-15
NFE2L2	DPP3	0.54903712	2.11E-15
NFE2L2	TOR3A	0.5489658	2.13E-15
NFE2L2	ACTR6	0.54895064	2.13E-15
NFE2L2	RPS6KA4	0.54877218	2.18E-15
NFE2L2	PTPRJ	0.54871455	2.2E-15
NFE2L2	DDX60L	0.54854634	2.26E-15
NFE2L2	LHFPL6	0.54849272	2.27E-15
NFE2L2	TNFRSF1B	0.5484638	2.28E-15
NFE2L2	ZNF423	0.5484512	2.29E-15
NFE2L2	F2RL1	0.54840648	2.3E-15
NFE2L2	NEDD9	0.54836288	2.31E-15
NFE2L2	ADGRF5	0.54833385	2.32E-15
NFE2L2	RHBDF2	0.54829588	2.34E-15
NFE2L2	ST3GAL2	0.54827578	2.34E-15
NFE2L2	ZNF512	0.5482591	2.35E-15
NFE2L2	VPS18	0.54825625	2.35E-15
NFE2L2	NCF2	0.54816703	2.38E-15

NFE2L2	CTSB	0.5481058	2.4E-15
NFE2L2	LYPD5	0.54797218	2.44E-15
NFE2L2	PUDP	0.54785882	2.48E-15
NFE2L2	YOD1	0.54784645	2.49E-15
NFE2L2	PTPN22	0.54782846	2.49E-15
NFE2L2	TAF7	0.54780207	2.5E-15
NFE2L2	C1orf216	0.5477539	2.52E-15
NFE2L2	LOXL3	0.54770828	2.54E-15
NFE2L2	DHFR	0.54766216	2.55E-15
NFE2L2	ZNF816	0.54726316	2.7E-15
NFE2L2	BGN	0.54720423	2.72E-15
NFE2L2	CCT5	0.54716406	2.73E-15
NFE2L2	GOLM1	0.54711186	2.75E-15
NFE2L2	SAE1	0.54708179	2.77E-15
NFE2L2	MARCKS	0.54693786	2.82E-15
NFE2L2	GCLM	0.5469354	2.82E-15
NFE2L2	TRIP4	0.54692543	2.83E-15
NFE2L2	XRCC6	0.54692491	2.83E-15
NFE2L2	FUT4	0.5468451	2.86E-15
NFE2L2	IAH1	0.54644395	3.02E-15
NFE2L2	PPP1R21	0.546324	3.07E-15
NFE2L2	RBM8A	0.54632369	3.07E-15
NFE2L2	ARSJ	0.54626775	3.1E-15
NFE2L2	GLE1	0.54622568	3.12E-15
NFE2L2	AJUBA	0.54618773	3.13E-15
NFE2L2	ATP6V1D	0.54590786	3.26E-15
NFE2L2	RHOU	0.54587987	3.27E-15
NFE2L2	ZCRB1	0.54568348	3.36E-15
NFE2L2	PLEKHM2	0.54565196	3.37E-15
NFE2L2	IL15	0.54565157	3.37E-15
NFE2L2	VPS72	0.54555885	3.42E-15
NFE2L2	TOMM40L	0.54554037	3.43E-15
NFE2L2	SH2D4A	0.54524215	3.57E-15
NFE2L2	APOL1	0.54521246	3.59E-15
NFE2L2	TTLL5	0.54502898	3.68E-15
NFE2L2	FAM234A	0.54497263	3.71E-15
NFE2L2	JAK2	0.54496165	3.71E-15
NFE2L2	RPA2	0.54486775	3.76E-15
NFE2L2	FMNL3	0.54482302	3.78E-15
NFE2L2	FRY	0.54476283	3.82E-15
NFE2L2	RBMX2	0.54453325	3.94E-15
NFE2L2	METTL2B	0.54451998	3.95E-15
NFE2L2	HSBP1	0.54433222	4.05E-15
NFE2L2	KLF5	0.54433097	4.05E-15
NFE2L2	PRRX1	0.54423155	4.11E-15
NFE2L2	AFAP1L1	0.54418127	4.13E-15
NFE2L2	PSMA5	0.54396961	4.26E-15
NFE2L2	APAF1	0.543909	4.29E-15
NFE2L2	DUSP18	0.5439015	4.3E-15
NFE2L2	RCAN1	0.54390042	4.3E-15
NFE2L2	SLC35B2	0.54386479	4.32E-15
NFE2L2	ACTG1	0.54382976	4.34E-15
NFE2L2	SYS1	0.54358589	4.49E-15
NFE2L2	MCM6	0.54348438	4.55E-15
NFE2L2	GYS1	0.54326437	4.69E-15
NFE2L2	SMIM3	0.54324106	4.71E-15
NFE2L2	GNS	0.543232	4.71E-15
NFE2L2	GPR176	0.54321641	4.72E-15

NFE2L2	ARNTL2	0.5431977	4.73E-15
NFE2L2	PACRGL	0.54315466	4.76E-15
NFE2L2	CLDN12	0.54312345	4.78E-15
NFE2L2	SF3B6	0.54297296	4.88E-15
NFE2L2	IVD	0.54291478	4.92E-15
NFE2L2	RAB27B	0.54265805	5.1E-15
NFE2L2	FBLN2	0.5426349	5.11E-15
NFE2L2	GLOD4	0.54262034	5.12E-15
NFE2L2	ZNF131	0.54259693	5.14E-15
NFE2L2	CLN5	0.54252463	5.19E-15
NFE2L2	GSR	0.54250656	5.2E-15
NFE2L2	DBI	0.54235121	5.32E-15
NFE2L2	FZR1	0.5421329	5.48E-15
NFE2L2	ERO1A	0.54210055	5.5E-15
NFE2L2	ARL4A	0.54198295	5.59E-15
NFE2L2	RETREG2	0.54197341	5.6E-15
NFE2L2	BZW2	0.54193752	5.63E-15
NFE2L2	LIF	0.54188374	5.67E-15
NFE2L2	ITGB3	0.54185236	5.69E-15
NFE2L2	MAPKAP1	0.54168935	5.82E-15
NFE2L2	ARHGDIB	0.54155059	5.93E-15
NFE2L2	THUMPD3	0.54145	6.01E-15
NFE2L2	TMSB4X	0.54131295	6.13E-15
NFE2L2	CALCRL	0.54127765	6.16E-15
NFE2L2	CHST11	0.54116777	6.25E-15
NFE2L2	FOS	0.54116073	6.26E-15
NFE2L2	ACO2	0.54101097	6.38E-15
NFE2L2	NAPEPLD	0.5409196	6.47E-15
NFE2L2	SH3BP5L	0.54081776	6.56E-15
NFE2L2	FOXC1	0.54066423	6.69E-15
NFE2L2	KYNU	0.54060197	6.75E-15
NFE2L2	TMEM14A	0.54056966	6.78E-15
NFE2L2	EPHA3	0.54032134	7.01E-15
NFE2L2	SCARA3	0.54028001	7.05E-15
NFE2L2	ARHGEF18	0.54026945	7.06E-15
NFE2L2	AKAP13	0.54021111	7.12E-15
NFE2L2	CDCP1	0.54015314	7.18E-15
NFE2L2	DNM2	0.54005129	7.28E-15
NFE2L2	ABHD17B	0.5398852	7.44E-15
NFE2L2	APOBEC3G	0.5398754	7.45E-15
NFE2L2	SPTSSA	0.53966557	7.67E-15
NFE2L2	LGALS9	0.53947241	7.87E-15
NFE2L2	TRAPPC3	0.53942988	7.92E-15
NFE2L2	CCDC97	0.53928762	8.07E-15
NFE2L2	ADK	0.53921495	8.15E-15
NFE2L2	LSM8	0.53921165	8.16E-15
NFE2L2	ATXN7L1	0.53903848	8.35E-15
NFE2L2	TP53I3	0.53883091	8.59E-15
NFE2L2	WBP4	0.53877335	8.65E-15
NFE2L2	TINAGL1	0.53876224	8.67E-15
NFE2L2	TM9SF2	0.53875192	8.68E-15
NFE2L2	CKLF	0.53874288	8.69E-15
NFE2L2	EZR	0.53874257	8.69E-15
NFE2L2	GFPT2	0.53862406	8.83E-15
NFE2L2	CFI	0.53858965	8.87E-15
NFE2L2	SLC16A4	0.53847362	9.01E-15
NFE2L2	UBE2E2	0.53843292	9.06E-15
NFE2L2	PIGK	0.53838707	9.12E-15

NFE2L2	AK6	0.53832368	9.2E-15
NFE2L2	HMOX1	0.5382329	9.31E-15
NFE2L2	MED7	0.53817245	9.39E-15
NFE2L2	SLC40A1	0.53811196	9.46E-15
NFE2L2	PLBD2	0.53802622	9.57E-15
NFE2L2	TMEM47	0.53799151	9.62E-15
NFE2L2	SYDE1	0.53777996	9.9E-15
NFE2L2	CREB3L2	0.53770642	1E-14
NFE2L2	INPP5F	0.53767329	1E-14
NFE2L2	TNFSF10	0.53760366	1.01E-14
NFE2L2	RPGRIP1L	0.53759037	1.02E-14
NFE2L2	NAT1	0.53752195	1.02E-14
NFE2L2	ARMC9	0.53733833	1.05E-14
NFE2L2	GRPEL1	0.53719886	1.07E-14
NFE2L2	LCP2	0.53718388	1.07E-14
NFE2L2	RNF40	0.53717904	1.07E-14
NFE2L2	RBM4	0.53702597	1.1E-14
NFE2L2	GPKOW	0.53691504	1.11E-14
NFE2L2	DSTN	0.53688438	1.12E-14
NFE2L2	MRPL42	0.53683377	1.12E-14
NFE2L2	BMP1	0.53681907	1.13E-14
NFE2L2	LYRM2	0.53669519	1.15E-14
NFE2L2	BRIX1	0.53652602	1.17E-14
NFE2L2	COPG2	0.53647484	1.18E-14
NFE2L2	URM1	0.53644479	1.18E-14
NFE2L2	EPHA2	0.53643124	1.19E-14
NFE2L2	VSTM4	0.5363597	1.2E-14
NFE2L2	TAGLN2	0.53635848	1.2E-14
NFE2L2	SMIM13	0.53632728	1.2E-14
NFE2L2	BTF3	0.53629029	1.21E-14
NFE2L2	RNPEP	0.53627618	1.21E-14
NFE2L2	TM2D3	0.53619993	1.22E-14
NFE2L2	RPP38	0.53619378	1.23E-14
NFE2L2	NIT2	0.53617934	1.23E-14
NFE2L2	MBIP	0.53615489	1.23E-14
NFE2L2	ILDR1	0.53611903	1.24E-14
NFE2L2	TMEM200A	0.5361058	1.24E-14
NFE2L2	GPC4	0.53605372	1.25E-14
NFE2L2	ABLIM1	0.53583898	1.29E-14
NFE2L2	CCN2	0.53572705	1.3E-14
NFE2L2	DHCR24	0.53551709	1.34E-14
NFE2L2	ZNF398	0.53541659	1.36E-14
NFE2L2	NINJ2	0.53541107	1.36E-14
NFE2L2	TCIM	0.53522132	1.4E-14
NFE2L2	OSTC	0.53519626	1.4E-14
NFE2L2	CHSY3	0.53516553	1.41E-14
NFE2L2	DSC2	0.53514756	1.41E-14
NFE2L2	PLIN2	0.53512249	1.41E-14
NFE2L2	CLIC2	0.53510709	1.42E-14
NFE2L2	LIPG	0.53509383	1.42E-14
NFE2L2	SFXN1	0.53498463	1.44E-14
NFE2L2	GPALPP1	0.53491047	1.46E-14
NFE2L2	MCU	0.5349009	1.46E-14
NFE2L2	PFKM	0.5348732	1.46E-14
NFE2L2	LAYN	0.53467379	1.5E-14
NFE2L2	PRCP	0.53460623	1.52E-14
NFE2L2	TLR5	0.53458104	1.52E-14
NFE2L2	GEMIN2	0.53453907	1.53E-14

NFE2L2	CCDC80	0.53446562	1.54E-14
NFE2L2	ADAMTS4	0.53446113	1.55E-14
NFE2L2	DOK5	0.5344557	1.55E-14
NFE2L2	UBE2L3	0.53440731	1.56E-14
NFE2L2	SDF4	0.53436394	1.57E-14
NFE2L2	PIP4K2B	0.53427394	1.58E-14
NFE2L2	SUGT1	0.53422973	1.59E-14
NFE2L2	F13A1	0.53420079	1.6E-14
NFE2L2	SLC12A7	0.53411328	1.62E-14
NFE2L2	MED15	0.53402668	1.64E-14
NFE2L2	NIN	0.53401708	1.64E-14
NFE2L2	CABLES1	0.5339143	1.66E-14
NFE2L2	CTHRC1	0.53390383	1.66E-14
NFE2L2	PPIC	0.53384983	1.68E-14
NFE2L2	YBX1	0.53380393	1.69E-14
NFE2L2	FCGR2A	0.53379441	1.69E-14
NFE2L2	SNX17	0.53371337	1.71E-14
NFE2L2	NSMCE3	0.53345275	1.77E-14
NFE2L2	AGPAT1	0.53337994	1.78E-14
NFE2L2	ZNF526	0.53325378	1.81E-14
NFE2L2	COLGALT1	0.53317061	1.84E-14
NFE2L2	AHNAK2	0.5330321	1.87E-14
NFE2L2	KDELR2	0.53290401	1.9E-14
NFE2L2	DNAJA1	0.53285585	1.91E-14
NFE2L2	RAP2A	0.53281162	1.92E-14
NFE2L2	MICOS10-NBL1	0.53270532	1.95E-14
NFE2L2	CARD16	0.53267433	1.96E-14
NFE2L2	CREM	0.53251614	2E-14
NFE2L2	EIF2S2	0.53244926	2.02E-14
NFE2L2	RRM1	0.53233901	2.05E-14
NFE2L2	BCL9	0.53233258	2.05E-14
NFE2L2	PML	0.53227424	2.07E-14
NFE2L2	PAMR1	0.53213831	2.1E-14
NFE2L2	PDPN	0.53212578	2.11E-14
NFE2L2	GLT8D1	0.53206213	2.13E-14
NFE2L2	ATP10A	0.53197432	2.15E-14
NFE2L2	ARHGAP23	0.53184481	2.19E-14
NFE2L2	RBM47	0.53161477	2.26E-14
NFE2L2	TOM1	0.53160455	2.26E-14
NFE2L2	HAND2	0.53154039	2.28E-14
NFE2L2	CPNE2	0.53139648	2.32E-14
NFE2L2	RHOF	0.53137186	2.33E-14
NFE2L2	SLC35F6	0.53136688	2.33E-14
NFE2L2	LTA4H	0.5313329	2.34E-14
NFE2L2	FZD1	0.53132584	2.34E-14
NFE2L2	MECP2	0.53124714	2.37E-14
NFE2L2	SOCS3	0.53124406	2.37E-14
NFE2L2	ETNK1	0.53120313	2.38E-14
NFE2L2	TSPAN18	0.53115898	2.4E-14
NFE2L2	NID2	0.53113737	2.4E-14
NFE2L2	KATNAL1	0.53100764	2.44E-14
NFE2L2	GLT8D2	0.53090429	2.48E-14
NFE2L2	ACTA2	0.53087835	2.49E-14
NFE2L2	PPP1R7	0.53076637	2.52E-14
NFE2L2	ZYX	0.53068881	2.55E-14
NFE2L2	OAS2	0.53068459	2.55E-14
NFE2L2	PITPNA	0.53054548	2.6E-14
NFE2L2	TLN2	0.53050993	2.61E-14

NFE2L2	PPP2R5C	0.53047377	2.62E-14
NFE2L2	HNRNPA1P48	0.53046136	2.63E-14
NFE2L2	PLCD3	0.53043948	2.63E-14
NFE2L2	KIAA0586	0.53037333	2.66E-14
NFE2L2	PLEC	0.5303413	2.67E-14
NFE2L2	TMEM14C	0.53034028	2.67E-14
NFE2L2	MKRN1	0.53011743	2.75E-14
NFE2L2	AMPD3	0.53010754	2.75E-14
NFE2L2	BRK1	0.53005308	2.77E-14
NFE2L2	SERPINH1	0.53001513	2.79E-14
NFE2L2	PPME1	0.52994541	2.81E-14
NFE2L2	B3GNT9	0.52984058	2.85E-14
NFE2L2	TAF9	0.52974996	2.88E-14
NFE2L2	CHTF8	0.52951602	2.97E-14
NFE2L2	GMIP	0.52948497	2.99E-14
NFE2L2	PLSCR3	0.52933368	3.05E-14
NFE2L2	PDE1A	0.52915031	3.12E-14
NFE2L2	OSBPL5	0.52907259	3.15E-14
NFE2L2	LAMA4	0.52905153	3.16E-14
NFE2L2	C15orf39	0.52891786	3.22E-14
NFE2L2	PECAM1	0.52879314	3.27E-14
NFE2L2	SEPHS2	0.52872619	3.3E-14
NFE2L2	TCEAL8	0.52860113	3.35E-14
NFE2L2	MINPP1	0.52836297	3.46E-14
NFE2L2	MEF2C	0.52832062	3.48E-14
NFE2L2	JHY	0.52815635	3.55E-14
NFE2L2	SPAG16	0.52815522	3.55E-14
NFE2L2	CNDP2	0.52814437	3.56E-14
NFE2L2	GLIS2	0.52803794	3.61E-14
NFE2L2	GM2A	0.52802855	3.61E-14
NFE2L2	PRICKLE1	0.52799754	3.63E-14
NFE2L2	LIN54	0.52798736	3.63E-14
NFE2L2	DUSP14	0.52798192	3.64E-14
NFE2L2	RNF138	0.52755924	3.84E-14
NFE2L2	FOXF2	0.52747137	3.89E-14
NFE2L2	DCAF13	0.52731318	3.97E-14
NFE2L2	TTI2	0.5272661	3.99E-14
NFE2L2	NPC1	0.52725621	4E-14
NFE2L2	IL1RN	0.52718139	4.04E-14
NFE2L2	SPARCL1	0.52717574	4.04E-14
NFE2L2	TRRAP	0.52702383	4.12E-14
NFE2L2	MRPL16	0.52702111	4.12E-14
NFE2L2	DPH3	0.52694979	4.16E-14
NFE2L2	PSMA3	0.52688867	4.19E-14
NFE2L2	CHMP7	0.52683979	4.22E-14
NFE2L2	ZNF629	0.52677842	4.25E-14
NFE2L2	C12orf43	0.5267618	4.26E-14
NFE2L2	MMRN2	0.52673133	4.28E-14
NFE2L2	LGALS3	0.52666099	4.32E-14
NFE2L2	KCNK1	0.52661299	4.35E-14
NFE2L2	TRMT61B	0.52647572	4.42E-14
NFE2L2	GJA1	0.52642731	4.45E-14
NFE2L2	DAGLB	0.52638413	4.48E-14
NFE2L2	MMAA	0.52631583	4.52E-14
NFE2L2	SPIN4	0.52621031	4.58E-14
NFE2L2	MRPL50	0.52616379	4.61E-14
NFE2L2	ADAMTS2	0.52614514	4.62E-14
NFE2L2	LMNA	0.52600903	4.7E-14

NFE2L2	PROM2	0.52598027	4.72E-14
NFE2L2	CEP41	0.52595723	4.73E-14
NFE2L2	VASP	0.5258763	4.78E-14
NFE2L2	APOBEC3D	0.52587191	4.79E-14
NFE2L2	DBF4	0.5258489	4.8E-14
NFE2L2	RGS1	0.52578543	4.84E-14
NFE2L2	PPP1R12B	0.52577345	4.85E-14
NFE2L2	TMEM185A	0.52568126	4.91E-14
NFE2L2	HNRNPM	0.52543217	5.07E-14
NFE2L2	MRPS14	0.52536806	5.11E-14
NFE2L2	AVL9	0.52531837	5.14E-14
NFE2L2	SLC9A1	0.52525306	5.19E-14
NFE2L2	RASGRP3	0.52502073	5.34E-14
NFE2L2	RCE1	0.52500777	5.35E-14
NFE2L2	TSPAN17	0.52493794	5.4E-14
NFE2L2	INPP1	0.52493773	5.4E-14
NFE2L2	GUCY1A1	0.52489331	5.43E-14
NFE2L2	SH3BGRL2	0.52489311	5.43E-14
NFE2L2	STX11	0.52484356	5.47E-14
NFE2L2	ZC4H2	0.5248415	5.47E-14
NFE2L2	RNF220	0.52483073	5.48E-14
NFE2L2	LRIF1	0.52477192	5.52E-14
NFE2L2	DDA1	0.52475309	5.53E-14
NFE2L2	CLTC	0.52460632	5.64E-14
NFE2L2	RPIA	0.52449119	5.72E-14
NFE2L2	VPS35L	0.52437482	5.81E-14
NFE2L2	CLN8	0.52428617	5.88E-14
NFE2L2	CIP2A	0.52428318	5.88E-14
NFE2L2	TWF2	0.52427334	5.89E-14
NFE2L2	CMTM1	0.52419429	5.95E-14
NFE2L2	HSDL1	0.52416418	5.97E-14
NFE2L2	AIFM2	0.52409916	6.02E-14
NFE2L2	DYNLT1	0.52408077	6.03E-14
NFE2L2	HDAC8	0.52407891	6.04E-14
NFE2L2	ZNF362	0.52402249	6.08E-14
NFE2L2	ADAMTS12	0.52400499	6.09E-14
NFE2L2	POLR2K	0.52393728	6.15E-14
NFE2L2	RAC2	0.52392984	6.15E-14
NFE2L2	EEF1AKNMT	0.52392983	6.15E-14
NFE2L2	RBBP8	0.52389845	6.18E-14
NFE2L2	HMGCS1	0.52386676	6.2E-14
NFE2L2	ACSL5	0.52383907	6.22E-14
NFE2L2	GBP2	0.52383434	6.23E-14
NFE2L2	ARL1	0.52383235	6.23E-14
NFE2L2	NACC1	0.52375461	6.29E-14
NFE2L2	CD58	0.52372271	6.32E-14
NFE2L2	NAV1	0.52369929	6.34E-14
NFE2L2	PIK3CG	0.52357469	6.44E-14
NFE2L2	TMEM63B	0.5235147	6.49E-14
NFE2L2	SFRP4	0.52342744	6.56E-14
NFE2L2	SFR1	0.5231527	6.8E-14
NFE2L2	CSF1	0.52309563	6.85E-14
NFE2L2	CZIB	0.52300083	6.93E-14
NFE2L2	SF3B4	0.52299294	6.94E-14
NFE2L2	ANXA3	0.52298368	6.95E-14
NFE2L2	KREMEN1	0.52297539	6.96E-14
NFE2L2	CHURC1	0.52293316	6.99E-14
NFE2L2	TMEM119	0.52288017	7.04E-14

NFE2L2	F3	0.52285654	7.06E-14
NFE2L2	SULF2	0.52279389	7.12E-14
NFE2L2	MYO5A	0.52260507	7.3E-14
NFE2L2	SNRPB2	0.52257164	7.33E-14
NFE2L2	LAMC2	0.52249129	7.4E-14
NFE2L2	HPS1	0.52238345	7.51E-14
NFE2L2	SSR1	0.52228907	7.6E-14
NFE2L2	AL669918.1	0.52227394	7.61E-14
NFE2L2	FNBP1	0.52215668	7.73E-14
NFE2L2	LYAR	0.52215066	7.73E-14
NFE2L2	SRXN1	0.52212539	7.76E-14
NFE2L2	MEGF6	0.52206583	7.82E-14
NFE2L2	HNRNPL	0.52203513	7.85E-14
NFE2L2	CXCL16	0.52199689	7.89E-14
NFE2L2	ZNF830	0.52197554	7.91E-14
NFE2L2	TLN1	0.52175509	8.14E-14
NFE2L2	HSD11B1	0.52171981	8.17E-14
NFE2L2	LARGE1	0.52170623	8.19E-14
NFE2L2	BNC2	0.52148816	8.42E-14
NFE2L2	NEK1	0.52144632	8.46E-14
NFE2L2	YTHDF1	0.52144264	8.47E-14
NFE2L2	NR1D2	0.52137271	8.54E-14
NFE2L2	ACOT2	0.52133513	8.58E-14
NFE2L2	KIAA1755	0.52133039	8.59E-14
NFE2L2	EDN1	0.52102617	8.93E-14
NFE2L2	LMAN2L	0.52097503	8.99E-14
NFE2L2	LRBA	0.52093125	9.04E-14
NFE2L2	AP1S2	0.52092946	9.04E-14
NFE2L2	GALNT4	0.5208047	9.19E-14
NFE2L2	GOT2	0.52079904	9.19E-14
NFE2L2	ATP5MC3	0.52077822	9.22E-14
NFE2L2	HSPA13	0.52074799	9.25E-14
NFE2L2	METTL5	0.52061769	9.41E-14
NFE2L2	CLUAP1	0.52056224	9.47E-14
NFE2L2	ESCO1	0.52048768	9.57E-14
NFE2L2	TARS2	0.52048731	9.57E-14
NFE2L2	RAE1	0.52047002	9.59E-14
NFE2L2	FGL2	0.52043444	9.63E-14
NFE2L2	SCOC	0.52031482	9.78E-14
NFE2L2	MAGOH	0.52027617	9.83E-14
NFE2L2	TRPV4	0.52023841	9.87E-14
NFE2L2	C8orf33	0.52016714	9.96E-14
NFE2L2	HLA-DRA	0.5200434	1.01E-13
NFE2L2	STAMBPL1	0.51987355	1.03E-13
NFE2L2	COQ7	0.5198126	1.04E-13
NFE2L2	PNPO	0.5195703	1.08E-13
NFE2L2	EFCAB11	0.51953123	1.08E-13
NFE2L2	RASGEF1B	0.51936848	1.1E-13
NFE2L2	SDC4	0.51919807	1.13E-13
NFE2L2	TAX1BP3	0.51913294	1.14E-13
NFE2L2	CNIH4	0.51906848	1.15E-13
NFE2L2	TBC1D1	0.51898107	1.16E-13
NFE2L2	TSPAN31	0.51897929	1.16E-13
NFE2L2	GPN2	0.51895517	1.16E-13
NFE2L2	SERPINB9	0.5188719	1.17E-13
NFE2L2	SLFN13	0.5186692	1.21E-13
NFE2L2	SMO	0.51856936	1.22E-13
NFE2L2	ITPKC	0.51852614	1.23E-13

NFE2L2	SMG5	0.51837408	1.25E-13
NFE2L2	TP53I11	0.51836537	1.25E-13
NFE2L2	BAK1	0.51834296	1.26E-13
NFE2L2	SMN1	0.51827455	1.27E-13
NFE2L2	CPZ	0.51818997	1.28E-13
NFE2L2	HOMEZ	0.51815124	1.29E-13
NFE2L2	TCN2	0.51806048	1.3E-13
NFE2L2	SQOR	0.51795981	1.32E-13
NFE2L2	TINF2	0.51789928	1.33E-13
NFE2L2	TMEM184B	0.51787495	1.33E-13
NFE2L2	TPD52L2	0.51785445	1.34E-13
NFE2L2	PCTP	0.5177038	1.36E-13
NFE2L2	GNA12	0.51769443	1.36E-13
NFE2L2	SPAG1	0.51751682	1.39E-13
NFE2L2	GABARAPL2	0.51751323	1.4E-13
NFE2L2	ANXA4	0.51741472	1.41E-13
NFE2L2	HTRA3	0.51739908	1.42E-13
NFE2L2	PTGS1	0.5171494	1.46E-13
NFE2L2	BRAP	0.51713781	1.46E-13
NFE2L2	ITGAM	0.51709906	1.47E-13
NFE2L2	RFK	0.51703289	1.48E-13
NFE2L2	DOK1	0.51701052	1.49E-13
NFE2L2	TOMM20	0.51699912	1.49E-13
NFE2L2	NAP1L1	0.51684339	1.52E-13
NFE2L2	TOR4A	0.51674528	1.54E-13
NFE2L2	KIT	0.5167123	1.54E-13
NFE2L2	EIF4G1	0.51663995	1.56E-13
NFE2L2	SERPINE1	0.51651363	1.58E-13
NFE2L2	MFAP4	0.51651083	1.58E-13
NFE2L2	FN1	0.51646632	1.59E-13
NFE2L2	FN3KRP	0.51637786	1.61E-13
NFE2L2	NDUFS2	0.51637429	1.61E-13
NFE2L2	CSK	0.51619378	1.65E-13
NFE2L2	BASP1	0.51605872	1.68E-13
NFE2L2	TRMT12	0.51588171	1.71E-13
NFE2L2	TRIP10	0.51583263	1.72E-13
NFE2L2	PHLDA1	0.51580828	1.73E-13
NFE2L2	SAMD4A	0.51574429	1.74E-13
NFE2L2	ISLR	0.51566182	1.76E-13
NFE2L2	HDGFL3	0.51554946	1.79E-13
NFE2L2	GPR183	0.51549121	1.8E-13
NFE2L2	ARHGEF6	0.51542105	1.82E-13
NFE2L2	OMD	0.51537081	1.83E-13
NFE2L2	EMP2	0.51536167	1.83E-13
NFE2L2	COG2	0.51535012	1.83E-13
NFE2L2	RIN3	0.51532521	1.84E-13
NFE2L2	AC106886.5	0.51530859	1.84E-13
NFE2L2	SLC31A1	0.51527818	1.85E-13
NFE2L2	CRISPLD1	0.51521566	1.86E-13
NFE2L2	ABRACL	0.51519675	1.87E-13
NFE2L2	CCAR2	0.5150996	1.89E-13
NFE2L2	CLMP	0.51509242	1.89E-13
NFE2L2	HACD3	0.51479546	1.96E-13
NFE2L2	ZC3H12A	0.51475089	1.97E-13
NFE2L2	RPA1	0.51466735	1.99E-13
NFE2L2	MRFAP1	0.51453228	2.03E-13
NFE2L2	MANSC1	0.51448299	2.04E-13
NFE2L2	METTL6	0.51440908	2.06E-13

NFE2L2	VDAC3	0.51426398	2.1E-13
NFE2L2	TLK2	0.51423434	2.11E-13
NFE2L2	TSPAN5	0.51419581	2.12E-13
NFE2L2	GSN	0.51417215	2.12E-13
NFE2L2	DYNC2LI1	0.51412531	2.13E-13
NFE2L2	TLE3	0.51409862	2.14E-13
NFE2L2	M6PR	0.51406636	2.15E-13
NFE2L2	LPXN	0.51404598	2.16E-13
NFE2L2	CDR2L	0.51393788	2.19E-13
NFE2L2	ERMP1	0.51391727	2.19E-13
NFE2L2	DNAJA4	0.51382501	2.22E-13
NFE2L2	RASSF2	0.51379521	2.22E-13
NFE2L2	CSRNP1	0.51376103	2.23E-13
NFE2L2	CYREN	0.51352484	2.3E-13
NFE2L2	PGRMC1	0.5134655	2.32E-13
NFE2L2	LURAP1L	0.51345582	2.32E-13
NFE2L2	CTSH	0.51339229	2.34E-13
NFE2L2	PRDX1	0.51337617	2.34E-13
NFE2L2	CD4	0.51335879	2.35E-13
NFE2L2	WNT5A	0.51328132	2.37E-13
NFE2L2	TOMM22	0.51319891	2.4E-13
NFE2L2	STPG1	0.5131386	2.41E-13
NFE2L2	HGF	0.51304072	2.44E-13
NFE2L2	CASP7	0.51299205	2.46E-13
NFE2L2	ADGRF4	0.51296959	2.47E-13
NFE2L2	PIBF1	0.51295834	2.47E-13
NFE2L2	ITGA4	0.51295089	2.47E-13
NFE2L2	IGFBP3	0.51288385	2.49E-13
NFE2L2	RPP30	0.51277812	2.52E-13
NFE2L2	KIF20B	0.51273831	2.54E-13
NFE2L2	SORBS3	0.51267234	2.56E-13
NFE2L2	C1orf112	0.51255879	2.59E-13
NFE2L2	COMMD7	0.51255304	2.6E-13
NFE2L2	SCAMP1	0.51251143	2.61E-13
NFE2L2	DIO2	0.51250538	2.61E-13
NFE2L2	ILF2	0.51248222	2.62E-13
NFE2L2	PTPRB	0.51247521	2.62E-13
NFE2L2	BOK	0.51245416	2.63E-13
NFE2L2	FCGR2B	0.5124484	2.63E-13
NFE2L2	PEX14	0.51243232	2.64E-13
NFE2L2	EHD3	0.51238596	2.65E-13
NFE2L2	VAPB	0.51225065	2.7E-13
NFE2L2	ENO1	0.51217577	2.72E-13
NFE2L2	EVI2B	0.51215742	2.73E-13
NFE2L2	MATN3	0.51214751	2.73E-13
NFE2L2	LDLR	0.51213617	2.73E-13
NFE2L2	ITGB1BP1	0.51202728	2.77E-13
NFE2L2	LPAR1	0.51201264	2.78E-13
NFE2L2	ERBB2	0.51197394	2.79E-13
NFE2L2	AKT3	0.5118599	2.83E-13
NFE2L2	SPON2	0.51182632	2.84E-13
NFE2L2	GTF2A2	0.51176207	2.86E-13
NFE2L2	RIOK2	0.51171263	2.88E-13
NFE2L2	FBLN1	0.51158604	2.93E-13
NFE2L2	STK10	0.51157419	2.93E-13
NFE2L2	FOXF1	0.51152545	2.95E-13
NFE2L2	GNG10	0.5114839	2.96E-13
NFE2L2	EFHD1	0.5113575	3.01E-13

NFE2L2	IGF2BP2	0.51112852	3.1E-13
NFE2L2	OPTN	0.51106351	3.12E-13
NFE2L2	SAMD14	0.51098525	3.15E-13
NFE2L2	BMP2	0.5109803	3.16E-13
NFE2L2	TIGAR	0.51097639	3.16E-13
NFE2L2	EGR1	0.51091035	3.18E-13
NFE2L2	KCNE4	0.51081273	3.22E-13
NFE2L2	FARSB	0.51078726	3.23E-13
NFE2L2	PPP3CB	0.5107302	3.25E-13
NFE2L2	NDUFAF1	0.51069983	3.27E-13
NFE2L2	CAPN5	0.51065289	3.29E-13
NFE2L2	IFIT3	0.5105923	3.31E-13
NFE2L2	UBC	0.51054948	3.33E-13
NFE2L2	ODF2	0.51050572	3.35E-13
NFE2L2	NDUFB3	0.51045758	3.37E-13
NFE2L2	BMF	0.51032528	3.42E-13
NFE2L2	FAM214B	0.51022469	3.46E-13
NFE2L2	MAST2	0.51014715	3.5E-13
NFE2L2	KDM6B	0.51014089	3.5E-13
NFE2L2	ARHGAP11A	0.510123	3.51E-13
NFE2L2	FNDC1	0.51009628	3.52E-13
NFE2L2	RGS3	0.51000277	3.56E-13
NFE2L2	PALB2	0.50990084	3.6E-13
NFE2L2	CYCS	0.50988496	3.61E-13
NFE2L2	SAMSN1	0.50976038	3.67E-13
NFE2L2	LAMTOR1	0.50975756	3.67E-13
NFE2L2	DPYSL2	0.50967516	3.71E-13
NFE2L2	CD40	0.5096582	3.71E-13
NFE2L2	TM6SF1	0.50965022	3.72E-13
NFE2L2	AHCYL2	0.50962186	3.73E-13
NFE2L2	FUNDC1	0.50959061	3.74E-13
NFE2L2	PTGER2	0.50958639	3.75E-13
NFE2L2	PMEPA1	0.50957393	3.75E-13
NFE2L2	PRCC	0.50926456	3.9E-13
NFE2L2	TP53RK	0.50915018	3.95E-13
NFE2L2	ARPC3	0.50913101	3.96E-13
NFE2L2	MSR1	0.50900831	4.02E-13
NFE2L2	VBP1	0.50894362	4.05E-13
NFE2L2	ITGA5	0.50876807	4.14E-13
NFE2L2	P4HA1	0.50870978	4.17E-13
NFE2L2	MMP2	0.50859923	4.23E-13
NFE2L2	RPS10-NUDT3	0.5084563	4.3E-13
NFE2L2	KLF12	0.50814799	4.47E-13
NFE2L2	CYB561A3	0.50814792	4.47E-13
NFE2L2	STIL	0.50813399	4.48E-13
NFE2L2	NUP155	0.5081114	4.49E-13
NFE2L2	AGPAT5	0.50787027	4.62E-13
NFE2L2	LSAMP	0.50775552	4.69E-13
NFE2L2	TMEM230	0.50767136	4.74E-13
NFE2L2	HSPE1-MOB4	0.50759403	4.78E-13
NFE2L2	KIF3B	0.50754573	4.81E-13
NFE2L2	NOCT	0.50753812	4.82E-13
NFE2L2	CHD1L	0.50731594	4.95E-13
NFE2L2	PSPC1	0.50727202	4.98E-13
NFE2L2	VCP	0.50719653	5.02E-13
NFE2L2	RAB27A	0.50718773	5.03E-13
NFE2L2	CYGB	0.50712414	5.07E-13
NFE2L2	PSMB8	0.50710962	5.08E-13

NFE2L2	COA4	0.50694972	5.18E-13
NFE2L2	SH3BP1	0.50682214	5.26E-13
NFE2L2	SNAI2	0.50678506	5.28E-13
NFE2L2	SLC43A3	0.50677991	5.28E-13
NFE2L2	COL12A1	0.50665588	5.36E-13
NFE2L2	PARP12	0.50649502	5.47E-13
NFE2L2	ZNF350	0.5063077	5.6E-13
NFE2L2	GLRX2	0.50614149	5.71E-13
NFE2L2	TRUB2	0.50608628	5.75E-13
NFE2L2	WDR53	0.50598536	5.82E-13
NFE2L2	BCR	0.50598394	5.82E-13
NFE2L2	SIRPA	0.50593617	5.86E-13
NFE2L2	CEBPZ	0.50593137	5.86E-13
NFE2L2	PLK3	0.50576827	5.98E-13
NFE2L2	CELSR1	0.50576449	5.98E-13
NFE2L2	CAV1	0.50562551	6.08E-13
NFE2L2	PPP1R15A	0.50562419	6.08E-13
NFE2L2	TSC22D1	0.50558751	6.11E-13
NFE2L2	BCCIP	0.50557049	6.12E-13
NFE2L2	SORCS2	0.50556299	6.13E-13
NFE2L2	HMGCR	0.50556135	6.13E-13
NFE2L2	EIF3I	0.5053469	6.29E-13
NFE2L2	SELPLG	0.50531646	6.31E-13
NFE2L2	PPM1G	0.50531133	6.32E-13
NFE2L2	CLIP2	0.5053112	6.32E-13
NFE2L2	MCM4	0.50522025	6.39E-13
NFE2L2	ALOX5	0.50508178	6.5E-13
NFE2L2	AFG3L2	0.50504751	6.52E-13
NFE2L2	SRGN	0.50501995	6.54E-13
NFE2L2	CETN3	0.50495726	6.59E-13
NFE2L2	EVI2A	0.50487422	6.66E-13
NFE2L2	TMEM68	0.50483968	6.69E-13
NFE2L2	CHST3	0.50482189	6.7E-13
NFE2L2	ADCY3	0.50478223	6.74E-13
NFE2L2	RAB7B	0.50467419	6.82E-13
NFE2L2	UFD1	0.50467368	6.82E-13
NFE2L2	CHMP1A	0.50451049	6.96E-13
NFE2L2	FBXO5	0.50449252	6.98E-13
NFE2L2	RUNX2	0.50440507	7.05E-13
NFE2L2	MAD2L1BP	0.50408489	7.33E-13
NFE2L2	KIN	0.50406457	7.35E-13
NFE2L2	THOC7	0.50406067	7.35E-13
NFE2L2	CEBPZOS	0.50404209	7.37E-13
NFE2L2	PRKAG1	0.50401635	7.39E-13
NFE2L2	FYB1	0.50400528	7.4E-13
NFE2L2	DTX4	0.50395542	7.44E-13
NFE2L2	SLIT2	0.50368366	7.69E-13
NFE2L2	PROCR	0.50353586	7.83E-13
NFE2L2	ANKLE2	0.50322783	8.13E-13
NFE2L2	RESF1	0.50318229	8.17E-13
NFE2L2	FAM111B	0.50316314	8.19E-13
NFE2L2	EIF2AK1	0.50298351	8.37E-13
NFE2L2	LRRN1	0.5029216	8.43E-13
NFE2L2	LAMP1	0.50291432	8.44E-13
NFE2L2	FES	0.50287908	8.48E-13
NFE2L2	GALNT5	0.50287447	8.48E-13
NFE2L2	MLH1	0.50283799	8.52E-13
NFE2L2	TIMMDC1	0.50282797	8.53E-13

NFE2L2	DUSP5	0.50282194	8.53E-13
NFE2L2	MN1	0.50278323	8.57E-13
NFE2L2	OGT	0.50273495	8.62E-13
NFE2L2	TM4SF1	0.50257259	8.79E-13
NFE2L2	GLB1L	0.50252672	8.84E-13
NFE2L2	INTS13	0.5024422	8.93E-13
NFE2L2	MEGF9	0.50237652	9E-13
NFE2L2	CHPT1	0.50237506	9E-13
NFE2L2	RUSC1	0.50226107	9.13E-13
NFE2L2	FIBIN	0.50223741	9.15E-13
NFE2L2	CEMIP	0.50222377	9.17E-13
NFE2L2	SF3B2	0.50221463	9.18E-13
NFE2L2	TMEM179B	0.50216075	9.24E-13
NFE2L2	WIPI2	0.50210672	9.3E-13
NFE2L2	GAL3ST4	0.50202253	9.39E-13
NFE2L2	TTC39B	0.50192144	9.51E-13
NFE2L2	OLFML2B	0.50186655	9.57E-13
NFE2L2	CDH5	0.50179048	9.66E-13
NFE2L2	WASHC3	0.50169594	9.77E-13
NFE2L2	C1orf21	0.50136746	1.02E-12
NFE2L2	NMT1	0.50129264	1.03E-12
NFE2L2	NQO1	0.50120156	1.04E-12
NFE2L2	RACGAP1	0.50115646	1.04E-12
NFE2L2	GBP4	0.50111435	1.05E-12
NFE2L2	FMOD	0.50105843	1.05E-12
NFE2L2	SMAGP	0.50103002	1.06E-12
NFE2L2	NUSAP1	0.50093087	1.07E-12
NFE2L2	PLCB3	0.5007999	1.09E-12
NFE2L2	ABR	0.50054903	1.12E-12
NFE2L2	ADGRG1	0.50041786	1.14E-12
NFE2L2	CD276	0.50041355	1.14E-12
NFE2L2	ARAP1	0.50032312	1.15E-12
NFE2L2	MEST	0.50025468	1.16E-12
NFE2L2	NGRN	0.50022628	1.16E-12
NFE2L2	PSMA1	0.50019524	1.17E-12
NFE2L2	FGF7	0.50014408	1.18E-12
NFE2L2	PPA1	0.50009948	1.18E-12
NLRP3	MZB1	0.50000538	1.2E-12
NLRP3	FXYD1	0.50024052	1.16E-12
NLRP3	PCDH12	0.50046472	1.13E-12
NLRP3	TAGLN	0.50066151	1.11E-12
NLRP3	PLCG2	0.50070294	1.1E-12
NLRP3	PDGFRL	0.50074356	1.09E-12
NLRP3	FAM168A	0.50076684	1.09E-12
NLRP3	CD22	0.50086502	1.08E-12
NLRP3	ZBTB2	0.50196427	9.46E-13
NLRP3	ADAM19	0.50256242	8.8E-13
NLRP3	SLC11A1	0.50261216	8.75E-13
NLRP3	KIT	0.50319786	8.16E-13
NLRP3	ITIH3	0.50336346	7.99E-13
NLRP3	DIPK1A	0.50342032	7.94E-13
NLRP3	BMF	0.50358261	7.79E-13
NLRP3	ARHGEF25	0.50391097	7.48E-13
NLRP3	EMP1	0.50408532	7.33E-13
NLRP3	S100B	0.50411161	7.3E-13
NLRP3	SLCO2A1	0.50422789	7.2E-13
NLRP3	MAP1A	0.5043023	7.14E-13
NLRP3	CYP27A1	0.50436957	7.08E-13

NLRP3	IKBIP	0.50453787	6.94E-13
NLRP3	LATS2	0.50464201	6.85E-13
NLRP3	COL6A2	0.50501117	6.55E-13
NLRP3	LRRC32	0.50502555	6.54E-13
NLRP3	TRIM38	0.50521084	6.39E-13
NLRP3	SMAD7	0.50523072	6.38E-13
NLRP3	SNN	0.50561637	6.09E-13
NLRP3	VPS35L	0.5057105	6.02E-13
NLRP3	GPX7	0.50571645	6.01E-13
NLRP3	GRAP	0.50633243	5.58E-13
NLRP3	RRAGC	0.50651697	5.46E-13
NLRP3	TPP1	0.506697	5.34E-13
NLRP3	TNXB	0.50690655	5.2E-13
NLRP3	AP1B1	0.50705485	5.11E-13
NLRP3	PDPN	0.50706279	5.1E-13
NLRP3	FKBP7	0.50708221	5.09E-13
NLRP3	CD79B	0.50715357	5.05E-13
NLRP3	MT2A	0.50738283	4.91E-13
NLRP3	GTDC1	0.50750759	4.83E-13
NLRP3	LAT	0.50775109	4.69E-13
NLRP3	PALLD	0.50824575	4.42E-13
NLRP3	IRF8	0.50836302	4.35E-13
NLRP3	CEP170	0.5088564	4.1E-13
NLRP3	TSHZ2	0.50909621	3.98E-13
NLRP3	FKBP15	0.50910002	3.98E-13
NLRP3	ZNF532	0.50910382	3.98E-13
NLRP3	MKNK1	0.50919817	3.93E-13
NLRP3	COL8A1	0.50932069	3.87E-13
NLRP3	ZFAND5	0.50937701	3.84E-13
NLRP3	HYAL2	0.50946773	3.8E-13
NLRP3	VCAM1	0.50965259	3.72E-13
NLRP3	MMP2	0.50984677	3.63E-13
NLRP3	ZNF101	0.50987194	3.62E-13
NLRP3	LIMS1	0.50991649	3.6E-13
NLRP3	SYDE1	0.51039205	3.39E-13
NLRP3	CRLF3	0.51072314	3.26E-13
NLRP3	ATP10D	0.51106898	3.12E-13
NLRP3	SEMA4D	0.51111249	3.1E-13
NLRP3	PLCB2	0.51144956	2.98E-13
NLRP3	RASL11B	0.51147261	2.97E-13
NLRP3	SULF1	0.51161663	2.92E-13
NLRP3	BTN3A1	0.51168138	2.89E-13
NLRP3	STK17A	0.511772	2.86E-13
NLRP3	TTL	0.51194578	2.8E-13
NLRP3	MCUB	0.51201718	2.77E-13
NLRP3	SSH1	0.51218889	2.72E-13
NLRP3	COL5A2	0.51219405	2.71E-13
NLRP3	CDH11	0.51236316	2.66E-13
NLRP3	GRB2	0.51250701	2.61E-13
NLRP3	PCNX1	0.51274054	2.54E-13
NLRP3	FAP	0.51280463	2.52E-13
NLRP3	SNTB2	0.51292289	2.48E-13
NLRP3	DUSP2	0.51299572	2.46E-13
NLRP3	IL6	0.51307726	2.43E-13
NLRP3	HLA-DOB	0.51309174	2.43E-13
NLRP3	KCTD12	0.51309922	2.43E-13
NLRP3	PLEKHM2	0.51312342	2.42E-13
NLRP3	AKT3	0.51323918	2.38E-13

NLRP3	TIMP2	0.5133834	2.34E-13
NLRP3	PRG4	0.51356313	2.29E-13
NLRP3	CHST2	0.51365438	2.26E-13
NLRP3	EPB41L3	0.51400368	2.17E-13
NLRP3	MB21D2	0.51408283	2.15E-13
NLRP3	PLVAP	0.51442316	2.06E-13
NLRP3	HLA-DQA2	0.51452397	2.03E-13
NLRP3	OSBPL8	0.51452672	2.03E-13
NLRP3	ADAM12	0.51461475	2.01E-13
NLRP3	GJA5	0.51493665	1.93E-13
NLRP3	LOX	0.51495044	1.93E-13
NLRP3	GJA4	0.51505906	1.9E-13
NLRP3	SUSD6	0.51552069	1.79E-13
NLRP3	VAV1	0.51556893	1.78E-13
NLRP3	MFNG	0.5155747	1.78E-13
NLRP3	RECQL	0.51562479	1.77E-13
NLRP3	EPS15	0.51566832	1.76E-13
NLRP3	DNM3	0.51568443	1.76E-13
NLRP3	GPR176	0.51579502	1.73E-13
NLRP3	SLC2A3	0.51584107	1.72E-13
NLRP3	ISLR	0.51605031	1.68E-13
NLRP3	NOTCH2	0.51609581	1.67E-13
NLRP3	NINJ1	0.51623921	1.64E-13
NLRP3	FHL1	0.516423	1.6E-13
NLRP3	ITGA5	0.51642342	1.6E-13
NLRP3	EFHD1	0.51646638	1.59E-13
NLRP3	KCTD10	0.51660328	1.56E-13
NLRP3	PLSCR4	0.51674481	1.54E-13
NLRP3	S100A8	0.51707422	1.47E-13
NLRP3	HLA-E	0.5171383	1.46E-13
NLRP3	TSPAN4	0.51727442	1.44E-13
NLRP3	ENPEP	0.51730377	1.43E-13
NLRP3	ITGA1	0.51756984	1.39E-13
NLRP3	FOSB	0.51770259	1.36E-13
NLRP3	PCDH18	0.51791641	1.33E-13
NLRP3	PLEKHG1	0.51807828	1.3E-13
NLRP3	CCSER2	0.51813045	1.29E-13
NLRP3	ACTA2	0.51825728	1.27E-13
NLRP3	F2RL3	0.51868507	1.2E-13
NLRP3	FTL	0.51903689	1.15E-13
NLRP3	SRGAP2C	0.51917804	1.13E-13
NLRP3	NABP1	0.51973525	1.05E-13
NLRP3	RAB23	0.51986553	1.04E-13
NLRP3	MOB3A	0.51995118	1.02E-13
NLRP3	CTSB	0.52097168	8.99E-14
NLRP3	GNAI2	0.52136429	8.55E-14
NLRP3	VASH1	0.52142415	8.49E-14
NLRP3	LXN	0.52175203	8.14E-14
NLRP3	FOXN3	0.52184489	8.04E-14
NLRP3	PTPRG	0.5219037	7.98E-14
NLRP3	PIM1	0.52197112	7.91E-14
NLRP3	DACT1	0.52203153	7.85E-14
NLRP3	ASAP1	0.52211087	7.77E-14
NLRP3	DEGS1	0.52231649	7.57E-14
NLRP3	NPC2	0.5224125	7.48E-14
NLRP3	LAYN	0.52249301	7.4E-14
NLRP3	HAS2	0.52254498	7.35E-14
NLRP3	AKR1B1	0.522587	7.31E-14

NLRP3	ST3GAL2	0.52270143	7.21E-14
NLRP3	PRKG1	0.52278032	7.13E-14
NLRP3	NR2F1	0.52279541	7.12E-14
NLRP3	JAK1	0.52300154	6.93E-14
NLRP3	ROR2	0.52306638	6.88E-14
NLRP3	BTG1	0.52308947	6.86E-14
NLRP3	GPR132	0.52334741	6.63E-14
NLRP3	CYB561A3	0.5234467	6.55E-14
NLRP3	SFRP2	0.52351842	6.49E-14
NLRP3	CALHM2	0.52390014	6.18E-14
NLRP3	SELENON	0.52447605	5.73E-14
NLRP3	GSDME	0.52477244	5.52E-14
NLRP3	KLHL5	0.5249837	5.37E-14
NLRP3	IGLL5	0.52504298	5.33E-14
NLRP3	DRAM1	0.52510359	5.29E-14
NLRP3	CLMP	0.52516944	5.24E-14
NLRP3	TRAM2	0.52537333	5.11E-14
NLRP3	CRISPLD1	0.52558065	4.97E-14
NLRP3	RERG	0.52564019	4.93E-14
NLRP3	CYP2U1	0.52586669	4.79E-14
NLRP3	ANXA6	0.52622382	4.57E-14
NLRP3	CCL21	0.52646128	4.43E-14
NLRP3	HIVEP2	0.52653084	4.39E-14
NLRP3	ARAP1	0.52663071	4.34E-14
NLRP3	RGS19	0.5266599	4.32E-14
NLRP3	GUCY1B1	0.52705285	4.1E-14
NLRP3	C2	0.52713034	4.06E-14
NLRP3	TSHZ3	0.52726562	3.99E-14
NLRP3	NAGK	0.52729788	3.98E-14
NLRP3	ATXN1	0.52740779	3.92E-14
NLRP3	NES	0.52762344	3.81E-14
NLRP3	PXDC1	0.52882369	3.26E-14
NLRP3	CTSZ	0.52930871	3.06E-14
NLRP3	RBMS1	0.52956316	2.96E-14
NLRP3	GPR68	0.52966387	2.92E-14
NLRP3	PLXNC1	0.52970335	2.9E-14
NLRP3	UVRAG	0.5299512	2.81E-14
NLRP3	ANK2	0.5300408	2.78E-14
NLRP3	IFI16	0.5300622	2.77E-14
NLRP3	ISLR2	0.53008745	2.76E-14
NLRP3	COL3A1	0.53008812	2.76E-14
NLRP3	BST1	0.53026479	2.7E-14
NLRP3	KANK2	0.53031895	2.68E-14
NLRP3	SH3RF3	0.53040052	2.65E-14
NLRP3	ITPKB	0.53056073	2.59E-14
NLRP3	RAB42	0.53123325	2.37E-14
NLRP3	FABP3	0.53125334	2.37E-14
NLRP3	RNF144B	0.53127353	2.36E-14
NLRP3	RAMP2	0.53128234	2.36E-14
NLRP3	RAMP3	0.5317198	2.22E-14
NLRP3	TMEM204	0.53172047	2.22E-14
NLRP3	GZMH	0.53262946	1.97E-14
NLRP3	CLEC2D	0.53291588	1.9E-14
NLRP3	RAB32	0.53294442	1.89E-14
NLRP3	RASGRF2	0.53303642	1.87E-14
NLRP3	LAMB1	0.53333615	1.8E-14
NLRP3	TRPM2	0.53334578	1.79E-14
NLRP3	CARD16	0.53356724	1.74E-14

NLRP3	CD81	0.53381053	1.69E-14
NLRP3	SNX2	0.53389659	1.67E-14
NLRP3	CYBC1	0.5345807	1.52E-14
NLRP3	TRANK1	0.53460333	1.52E-14
NLRP3	RCAN1	0.5346244	1.51E-14
NLRP3	P2RY6	0.53465438	1.51E-14
NLRP3	EDNRA	0.53471363	1.49E-14
NLRP3	SRPX2	0.53503335	1.43E-14
NLRP3	CTSL	0.53514745	1.41E-14
NLRP3	AKIRIN2	0.53515648	1.41E-14
NLRP3	TENM3	0.53545678	1.35E-14
NLRP3	CCL3	0.5357803	1.3E-14
NLRP3	NDN	0.53591249	1.27E-14
NLRP3	EFS	0.53592767	1.27E-14
NLRP3	ACAP1	0.53594662	1.27E-14
NLRP3	PRSS23	0.53623382	1.22E-14
NLRP3	ATP6V1B2	0.53692196	1.11E-14
NLRP3	MCTP1	0.53702079	1.1E-14
NLRP3	CXCL9	0.53743299	1.04E-14
NLRP3	PLPP1	0.53878695	8.64E-15
NLRP3	GPR4	0.53932309	8.03E-15
NLRP3	MYO9B	0.53947463	7.87E-15
NLRP3	MAF	0.53955997	7.78E-15
NLRP3	SELP	0.53956271	7.78E-15
NLRP3	SMIM10	0.5396321	7.7E-15
NLRP3	VSIR	0.53983091	7.5E-15
NLRP3	GADD45B	0.54019601	7.13E-15
NLRP3	AXL	0.54031196	7.02E-15
NLRP3	MCL1	0.54032353	7.01E-15
NLRP3	CRTC3	0.54087354	6.51E-15
NLRP3	ITGBL1	0.54151078	5.96E-15
NLRP3	CPA3	0.5418389	5.7E-15
NLRP3	PCOLCE	0.54209428	5.51E-15
NLRP3	HIC1	0.54234027	5.32E-15
NLRP3	C1orf216	0.54252992	5.19E-15
NLRP3	CSRP2	0.54265922	5.1E-15
NLRP3	PPP1R18	0.54272121	5.05E-15
NLRP3	SH3PXD2B	0.54284959	4.96E-15
NLRP3	ITM2A	0.54286837	4.95E-15
NLRP3	PROS1	0.54318074	4.74E-15
NLRP3	CCL18	0.54318525	4.74E-15
NLRP3	MEOX1	0.54360759	4.47E-15
NLRP3	COL4A1	0.54372573	4.4E-15
NLRP3	HMCN1	0.54381384	4.35E-15
NLRP3	SPART	0.543856	4.32E-15
NLRP3	IL33	0.54469595	3.85E-15
NLRP3	DPYSL3	0.54476766	3.81E-15
NLRP3	WWC3	0.5448842	3.75E-15
NLRP3	CTSO	0.54493382	3.73E-15
NLRP3	SLC2A5	0.54497545	3.7E-15
NLRP3	GINM1	0.54521044	3.59E-15
NLRP3	SERPING1	0.54542208	3.48E-15
NLRP3	TMEM140	0.5454345	3.48E-15
NLRP3	VAMP7	0.54565484	3.37E-15
NLRP3	FGF7	0.54620642	3.12E-15
NLRP3	PKD2	0.5463595	3.06E-15
NLRP3	ACP5	0.5465386	2.98E-15
NLRP3	PLA2G2D	0.54679253	2.88E-15

NLRP3	PLXND1	0.54694026	2.82E-15
NLRP3	ITPR1	0.54707352	2.77E-15
NLRP3	PIK3R1	0.54709239	2.76E-15
NLRP3	PRR16	0.54735633	2.66E-15
NLRP3	LMO2	0.54799425	2.44E-15
NLRP3	CCDC3	0.54802197	2.43E-15
NLRP3	PDLIM3	0.54802472	2.43E-15
NLRP3	VCAN	0.54826119	2.35E-15
NLRP3	CAMK1	0.54871212	2.2E-15
NLRP3	PRELP	0.54881319	2.17E-15
NLRP3	NFATC2	0.54895314	2.13E-15
NLRP3	NTAN1	0.54905777	2.1E-15
NLRP3	HPS5	0.54950166	1.97E-15
NLRP3	IL4I1	0.54963145	1.94E-15
NLRP3	RCBTB2	0.54995717	1.85E-15
NLRP3	ELK3	0.55026439	1.77E-15
NLRP3	PIM2	0.55061015	1.69E-15
NLRP3	FAM20A	0.55064081	1.68E-15
NLRP3	DCUN1D3	0.55068303	1.67E-15
NLRP3	PRICKLE1	0.55114616	1.57E-15
NLRP3	WDR81	0.55148126	1.49E-15
NLRP3	COL4A2	0.55229625	1.33E-15
NLRP3	MFAP5	0.55251595	1.29E-15
NLRP3	LRP1	0.55252193	1.29E-15
NLRP3	CLEC14A	0.55265276	1.26E-15
NLRP3	RNGTT	0.55295259	1.21E-15
NLRP3	PEA15	0.5530507	1.2E-15
NLRP3	APOD	0.55373118	1.09E-15
NLRP3	CLIC4	0.5538059	1.07E-15
NLRP3	TMEM71	0.55388693	1.06E-15
NLRP3	SRGAP2	0.55450495	9.72E-16
NLRP3	LHFPL2	0.55468955	9.47E-16
NLRP3	CD68	0.55521028	8.79E-16
NLRP3	TMEM150C	0.55567136	8.23E-16
NLRP3	NRP1	0.55574691	8.14E-16
NLRP3	COPZ2	0.55615147	7.68E-16
NLRP3	CCL2	0.5562469	7.57E-16
NLRP3	PTGIS	0.55663506	7.16E-16
NLRP3	APOL3	0.55683268	6.96E-16
NLRP3	HAS1	0.55684186	6.95E-16
NLRP3	GUCY1A1	0.55689149	6.9E-16
NLRP3	ITGB7	0.55712549	6.68E-16
NLRP3	PI4K2A	0.55742487	6.39E-16
NLRP3	FIBIN	0.55781296	6.05E-16
NLRP3	BGN	0.55829572	5.64E-16
NLRP3	GBP1	0.55854444	5.44E-16
NLRP3	BEND6	0.55861937	5.38E-16
NLRP3	PTPN22	0.55884056	5.21E-16
NLRP3	RELT	0.55886037	5.2E-16
NLRP3	MSC	0.55894234	5.14E-16
NLRP3	CAVIN1	0.55925472	4.91E-16
NLRP3	JAM3	0.55943835	4.78E-16
NLRP3	WTIP	0.55951052	4.73E-16
NLRP3	RNF122	0.55954651	4.71E-16
NLRP3	VAMP5	0.56014184	4.32E-16
NLRP3	PLEKHA2	0.56035077	4.19E-16
NLRP3	SPSB1	0.56044304	4.13E-16
NLRP3	JCHAIN	0.56061122	4.03E-16

NLRP3	CTSW	0.56118724	3.71E-16
NLRP3	CTSC	0.56122557	3.69E-16
NLRP3	CCR6	0.56163818	3.47E-16
NLRP3	NRXN2	0.56170945	3.43E-16
NLRP3	STAT5A	0.56190488	3.34E-16
NLRP3	TGFBR2	0.56190648	3.34E-16
NLRP3	SPARC	0.56227708	3.16E-16
NLRP3	OSBPL11	0.56228092	3.16E-16
NLRP3	PTAFR	0.56267464	2.98E-16
NLRP3	SLAMF7	0.56280503	2.93E-16
NLRP3	GADD45A	0.5642151	2.38E-16
NLRP3	CSGALNACT2	0.56425762	2.36E-16
NLRP3	STAB1	0.56483207	2.17E-16
NLRP3	STON1	0.56505131	2.1E-16
NLRP3	RAB8B	0.56540873	1.99E-16
NLRP3	AKAP12	0.56612651	1.79E-16
NLRP3	GZMM	0.56622662	1.77E-16
NLRP3	SNX10	0.56645069	1.71E-16
NLRP3	NFIL3	0.56663965	1.66E-16
NLRP3	EMCN	0.56683312	1.61E-16
NLRP3	ASPN	0.56711534	1.55E-16
NLRP3	PNOC	0.56760463	1.44E-16
NLRP3	SLC37A2	0.56838281	1.28E-16
NLRP3	COL6A3	0.56851087	1.26E-16
NLRP3	BICD2	0.56866039	1.23E-16
NLRP3	OSCAR	0.56886047	1.19E-16
NLRP3	UBE2E2	0.56901659	1.16E-16
NLRP3	CHSY3	0.56909695	1.15E-16
NLRP3	BATF3	0.56911119	1.15E-16
NLRP3	SH3BGRL	0.569204	1.13E-16
NLRP3	GZMB	0.5694538	1.09E-16
NLRP3	HPGDS	0.57006245	9.96E-17
NLRP3	TIMP3	0.57030759	9.6E-17
NLRP3	SWAP70	0.57046121	9.38E-17
NLRP3	PHLDB1	0.57095167	8.71E-17
NLRP3	APOBEC3G	0.57110606	8.51E-17
NLRP3	AQP9	0.57131151	8.25E-17
NLRP3	DTX1	0.57145894	8.07E-17
NLRP3	ICAM1	0.57151863	7.99E-17
NLRP3	LEPR	0.57165298	7.83E-17
NLRP3	FCER1A	0.57247174	6.92E-17
NLRP3	BTN3A3	0.57273067	6.65E-17
NLRP3	NIN	0.57296041	6.43E-17
NLRP3	LIX1L	0.57315326	6.24E-17
NLRP3	CALD1	0.57382408	5.64E-17
NLRP3	PRRX1	0.57382439	5.64E-17
NLRP3	MILR1	0.574042	5.45E-17
NLRP3	NID1	0.57436075	5.19E-17
NLRP3	KIRREL1	0.57438826	5.17E-17
NLRP3	SSPN	0.57442555	5.14E-17
NLRP3	SIRPB1	0.57459636	5.01E-17
NLRP3	SCARA3	0.57491039	4.77E-17
NLRP3	PRXL2C	0.57550469	4.36E-17
NLRP3	LEF1	0.57552426	4.35E-17
NLRP3	CTSK	0.5759622	4.06E-17
NLRP3	CYTH3	0.57597371	4.06E-17
NLRP3	HOXD8	0.57598206	4.05E-17
NLRP3	CYLD	0.57599123	4.05E-17

NLRP3	CLEC2B	0.57629362	3.86E-17
NLRP3	MSRB3	0.57649587	3.74E-17
NLRP3	HECA	0.57667277	3.64E-17
NLRP3	GZMA	0.57720564	3.36E-17
NLRP3	RAB3IL1	0.57745505	3.23E-17
NLRP3	HLX	0.57844205	2.77E-17
NLRP3	VSTM4	0.57849467	2.75E-17
NLRP3	INPP5D	0.57890167	2.58E-17
NLRP3	APOL4	0.57914698	2.49E-17
NLRP3	RASSF8	0.57924513	2.45E-17
NLRP3	ADCY3	0.57939127	2.39E-17
NLRP3	SMIM3	0.57993465	2.2E-17
NLRP3	LUM	0.58016896	2.12E-17
NLRP3	CHST11	0.58028758	2.08E-17
NLRP3	ARHGAP22	0.58057797	1.99E-17
NLRP3	ALDH1A3	0.58069762	1.95E-17
NLRP3	LDB2	0.58112358	1.83E-17
NLRP3	SPRY1	0.58142198	1.75E-17
NLRP3	GGT5	0.58187379	1.63E-17
NLRP3	CCN1	0.5827315	1.42E-17
NLRP3	HLA-DQB2	0.58275616	1.42E-17
NLRP3	KLF12	0.58298529	1.37E-17
NLRP3	NID2	0.58303676	1.36E-17
NLRP3	NXPE3	0.58313238	1.34E-17
NLRP3	ZNF385A	0.58366591	1.23E-17
NLRP3	HLA-DMA	0.58408553	1.15E-17
NLRP3	EHD3	0.58410448	1.15E-17
NLRP3	FCMR	0.58410651	1.15E-17
NLRP3	ADAMTS4	0.58417641	1.13E-17
NLRP3	CERKL	0.58453673	1.07E-17
NLRP3	RGS10	0.5849642	1E-17
NLRP3	LAP3	0.5850811	9.82E-18
NLRP3	SNX3	0.58511311	9.77E-18
NLRP3	PSAP	0.58540193	9.33E-18
NLRP3	CCL19	0.58541625	9.31E-18
NLRP3	STOM	0.5856303	9E-18
NLRP3	FEZ1	0.5862079	8.21E-18
NLRP3	EMILIN2	0.58628175	8.12E-18
NLRP3	MDGA1	0.58629648	8.1E-18
NLRP3	CYBRD1	0.58632745	8.06E-18
NLRP3	ADGRF5	0.58676345	7.52E-18
NLRP3	CHRD	0.586887	7.37E-18
NLRP3	PDGFRB	0.58700207	7.24E-18
NLRP3	LIMD2	0.58712678	7.1E-18
NLRP3	ABL2	0.58721791	6.99E-18
NLRP3	CLDN11	0.58789029	6.28E-18
NLRP3	ZNF423	0.58897946	5.28E-18
NLRP3	JAK3	0.58952996	4.83E-18
NLRP3	LPL	0.58961802	4.76E-18
NLRP3	LOXL3	0.58962626	4.76E-18
NLRP3	IL6ST	0.58973804	4.67E-18
NLRP3	IFNGR1	0.59042056	4.19E-18
NLRP3	MMRN2	0.5904893	4.14E-18
NLRP3	LMCD1	0.59072336	3.99E-18
NLRP3	GLT8D2	0.59086732	3.89E-18
NLRP3	ZNF521	0.59107536	3.77E-18
NLRP3	IGFBP5	0.59108418	3.76E-18
NLRP3	ATP8B2	0.59137293	3.59E-18

NLRP3	MDFIC	0.59149372	3.52E-18
NLRP3	NOD1	0.5917022	3.4E-18
NLRP3	HEG1	0.59231707	3.08E-18
NLRP3	CH25H	0.59278471	2.86E-18
NLRP3	CFH	0.5929573	2.78E-18
NLRP3	ECSCR	0.59336774	2.6E-18
NLRP3	NKG7	0.59424978	2.25E-18
NLRP3	KCTD20	0.59460492	2.12E-18
NLRP3	CD248	0.59472286	2.08E-18
NLRP3	APLNR	0.59514704	1.94E-18
NLRP3	ITPRIP	0.59518586	1.93E-18
NLRP3	LZTS1	0.59558343	1.81E-18
NLRP3	SLC16A2	0.59586979	1.72E-18
NLRP3	NR3C1	0.59638037	1.59E-18
NLRP3	CD7	0.59683091	1.47E-18
NLRP3	PLD4	0.59713081	1.4E-18
NLRP3	BNC2	0.59741317	1.34E-18
NLRP3	SH2D3C	0.59753146	1.31E-18
NLRP3	MAN1C1	0.59761688	1.29E-18
NLRP3	TBC1D10C	0.59771186	1.27E-18
NLRP3	RUBCNL	0.59778233	1.26E-18
NLRP3	HLA-DRB1	0.59809757	1.19E-18
NLRP3	MSN	0.59824191	1.17E-18
NLRP3	SOCS3	0.59838973	1.14E-18
NLRP3	CPED1	0.59866453	1.09E-18
NLRP3	LSAMP	0.5989319	1.04E-18
NLRP3	NAALADL1	0.59924223	9.88E-19
NLRP3	MYCT1	0.59936115	9.69E-19
NLRP3	HSPA12B	0.59966644	9.21E-19
NLRP3	SMO	0.60011787	8.54E-19
NLRP3	FCHSD2	0.60022075	8.4E-19
NLRP3	CCN4	0.60034186	8.23E-19
NLRP3	STK17B	0.60062639	7.85E-19
NLRP3	RAB31	0.60072521	7.72E-19
NLRP3	HK3	0.60138231	6.91E-19
NLRP3	PLB1	0.60155201	6.72E-19
NLRP3	EPHA3	0.60160083	6.67E-19
NLRP3	FOXO1	0.60168982	6.57E-19
NLRP3	DLC1	0.6019591	6.28E-19
NLRP3	PLXDC1	0.60201438	6.22E-19
NLRP3	STAC3	0.60202642	6.21E-19
NLRP3	CDH5	0.6020718	6.16E-19
NLRP3	PTGDS	0.60291895	5.34E-19
NLRP3	DYSF	0.60365593	4.72E-19
NLRP3	CYYR1	0.60369395	4.69E-19
NLRP3	GASK1B	0.60377387	4.63E-19
NLRP3	CD8B	0.60385558	4.56E-19
NLRP3	MEDAG	0.60451974	4.08E-19
NLRP3	IKZF3	0.60473245	3.93E-19
NLRP3	GLI1	0.60477818	3.9E-19
NLRP3	PRKD3	0.60523494	3.61E-19
NLRP3	PPT1	0.60566301	3.36E-19
NLRP3	SERPINE1	0.60569367	3.34E-19
NLRP3	PRDM1	0.60660742	2.86E-19
NLRP3	WBP1L	0.60681936	2.76E-19
NLRP3	MFSD1	0.60696254	2.69E-19
NLRP3	PDE1B	0.60708017	2.64E-19
NLRP3	GPC3	0.60733323	2.53E-19

NLRP3	RASGRP3	0.6078559	2.31E-19
NLRP3	FILIP1L	0.60804265	2.24E-19
NLRP3	GNG11	0.60828849	2.15E-19
NLRP3	ZEB1	0.60838857	2.11E-19
NLRP3	PNRC1	0.60882853	1.96E-19
NLRP3	SHISAL2A	0.60923551	1.82E-19
NLRP3	DIPK2B	0.60980055	1.65E-19
NLRP3	CYGB	0.61003579	1.59E-19
NLRP3	CHSY1	0.61012061	1.57E-19
NLRP3	PLIN2	0.61018861	1.55E-19
NLRP3	CPXM1	0.61062512	1.43E-19
NLRP3	TIE1	0.61065839	1.43E-19
NLRP3	BCL6B	0.61086014	1.38E-19
NLRP3	BTN2A2	0.61115534	1.31E-19
NLRP3	MBNL1	0.611438	1.25E-19
NLRP3	CLEC5A	0.61181346	1.17E-19
NLRP3	FBN1	0.6121933	1.09E-19
NLRP3	CD1E	0.61239238	1.06E-19
NLRP3	TMEM131L	0.61287145	9.71E-20
NLRP3	ADGRL4	0.61324643	9.1E-20
NLRP3	SERPINB9	0.61376091	8.31E-20
NLRP3	CILP	0.6142753	7.6E-20
NLRP3	PALMD	0.6147304	7.02E-20
NLRP3	TCN2	0.61547302	6.16E-20
NLRP3	ZFPM2	0.61556363	6.06E-20
NLRP3	SNAI1	0.61586708	5.74E-20
NLRP3	HMOX1	0.61644038	5.19E-20
NLRP3	F2R	0.61673559	4.93E-20
NLRP3	VIM	0.61695335	4.74E-20
NLRP3	IL12RB1	0.61705167	4.66E-20
NLRP3	GXYLT2	0.61842893	3.65E-20
NLRP3	DDR2	0.61857614	3.56E-20
NLRP3	DUSP1	0.61862249	3.53E-20
NLRP3	FSTL1	0.61863816	3.52E-20
NLRP3	IGFBP4	0.61875592	3.44E-20
NLRP3	GEM	0.61916785	3.2E-20
NLRP3	ZAP70	0.61920029	3.18E-20
NLRP3	RUNX3	0.61946258	3.04E-20
NLRP3	ARID5B	0.62022391	2.65E-20
NLRP3	MGP	0.62123033	2.21E-20
NLRP3	HLA-DQA1	0.62146062	2.12E-20
NLRP3	ROBO1	0.62182295	1.99E-20
NLRP3	COL15A1	0.62182958	1.99E-20
NLRP3	ARID5A	0.6218708	1.97E-20
NLRP3	SLFN11	0.62187608	1.97E-20
NLRP3	SLAMF6	0.62189715	1.96E-20
NLRP3	AKNA	0.62219401	1.86E-20
NLRP3	SPON1	0.62226942	1.84E-20
NLRP3	LTBP2	0.62247558	1.77E-20
NLRP3	CCL22	0.62284947	1.65E-20
NLRP3	FERMT2	0.62296347	1.62E-20
NLRP3	OMD	0.6229915	1.61E-20
NLRP3	IL27RA	0.62303155	1.6E-20
NLRP3	TMC8	0.62348043	1.47E-20
NLRP3	ADAMTSL1	0.62356142	1.45E-20
NLRP3	APOBR	0.62360655	1.44E-20
NLRP3	CNN3	0.62397545	1.35E-20
NLRP3	OLFML2B	0.62411703	1.31E-20

NLRP3	SLIT3	0.6245231	1.22E-20
NLRP3	MATK	0.62473812	1.17E-20
NLRP3	LIPA	0.62577629	9.71E-21
NLRP3	BMP2K	0.6259046	9.49E-21
NLRP3	PAPLN	0.62614795	9.08E-21
NLRP3	OLR1	0.62691699	7.88E-21
NLRP3	PDGFRA	0.62718439	7.51E-21
NLRP3	CCL17	0.62757125	6.99E-21
NLRP3	RASL12	0.62775294	6.76E-21
NLRP3	CSF3R	0.62784517	6.65E-21
NLRP3	SIT1	0.62810289	6.34E-21
NLRP3	CPVL	0.62833001	6.08E-21
NLRP3	PMP22	0.6288916	5.48E-21
NLRP3	TEK	0.62915198	5.22E-21
NLRP3	LY9	0.62953392	4.87E-21
NLRP3	GBP5	0.62953665	4.87E-21
NLRP3	TNFSF13B	0.62980896	4.63E-21
NLRP3	CD34	0.63047356	4.09E-21
NLRP3	QKI	0.63051262	4.06E-21
NLRP3	IFI30	0.630908	3.77E-21
NLRP3	OGN	0.63095529	3.74E-21
NLRP3	AP1S2	0.63148703	3.39E-21
NLRP3	CAVIN2	0.63182249	3.18E-21
NLRP3	MAP3K8	0.63259508	2.75E-21
NLRP3	FBXL7	0.63296455	2.57E-21
NLRP3	NFKBIA	0.63309614	2.51E-21
NLRP3	GIMAP7	0.63342303	2.36E-21
NLRP3	AOC3	0.63357822	2.29E-21
NLRP3	FAM180A	0.6336433	2.26E-21
NLRP3	KCNE4	0.63373144	2.23E-21
NLRP3	FES	0.63431222	1.99E-21
NLRP3	CALCRL	0.63442989	1.95E-21
NLRP3	SCPEP1	0.63525379	1.67E-21
NLRP3	KLRB1	0.63541281	1.62E-21
NLRP3	C19orf38	0.63574631	1.52E-21
NLRP3	PLXDC2	0.63586732	1.49E-21
NLRP3	TSC22D3	0.6369188	1.22E-21
NLRP3	PSTPIP1	0.63746571	1.1E-21
NLRP3	IL21R	0.63755299	1.08E-21
NLRP3	MFAP4	0.63787197	1.02E-21
NLRP3	GAL3ST4	0.63788225	1.01E-21
NLRP3	PPP1R16B	0.63795956	9.99E-22
NLRP3	SLC15A3	0.6381321	9.67E-22
NLRP3	FYN	0.63828612	9.39E-22
NLRP3	OLFML3	0.63843896	9.12E-22
NLRP3	PDE1A	0.63845642	9.09E-22
NLRP3	GSTM5	0.63851735	8.98E-22
NLRP3	BASP1	0.63871851	8.64E-22
NLRP3	PLEKHO1	0.63920268	7.88E-22
NLRP3	FLVCR2	0.63939118	7.6E-22
NLRP3	FBLN1	0.63977916	7.05E-22
NLRP3	TREM2	0.63986985	6.93E-22
NLRP3	APOLD1	0.63998463	6.78E-22
NLRP3	CD38	0.6404791	6.16E-22
NLRP3	THBD	0.64053776	6.09E-22
NLRP3	TDO2	0.64069336	5.91E-22
NLRP3	EPB41L2	0.64107881	5.49E-22
NLRP3	NPL	0.64132104	5.24E-22

NLRP3	CD74	0.6418711	4.71E-22
NLRP3	CD6	0.64243413	4.22E-22
NLRP3	CLEC12A	0.64286648	3.88E-22
NLRP3	KIAA1755	0.64340133	3.5E-22
NLRP3	CNRIP1	0.6435377	3.41E-22
NLRP3	PLA2G7	0.64429582	2.94E-22
NLRP3	RGL1	0.64465	2.74E-22
NLRP3	SLC2A9	0.6448424	2.64E-22
NLRP3	ATP11C	0.64488569	2.62E-22
NLRP3	IL3RA	0.64492139	2.6E-22
NLRP3	GPSM3	0.64553701	2.3E-22
NLRP3	CRTAP	0.64599653	2.11E-22
NLRP3	TRIM22	0.64603867	2.09E-22
NLRP3	PRAM1	0.64609433	2.07E-22
NLRP3	S1PR2	0.64672679	1.82E-22
NLRP3	IGFBP7	0.64742655	1.59E-22
NLRP3	RHOJ	0.64755247	1.55E-22
NLRP3	IL2RB	0.64761411	1.53E-22
NLRP3	MAP4K1	0.64797289	1.43E-22
NLRP3	FCGR1A	0.64834093	1.33E-22
NLRP3	CCDC102B	0.64875724	1.22E-22
NLRP3	NCF2	0.6491048	1.14E-22
NLRP3	OSTM1	0.64946838	1.06E-22
NLRP3	PIK3CG	0.64961344	1.03E-22
NLRP3	CD3D	0.64982207	9.87E-23
NLRP3	RECK	0.65007403	9.39E-23
NLRP3	CD1C	0.65078774	8.14E-23
NLRP3	PDE4B	0.65180182	6.64E-23
NLRP3	THY1	0.6521138	6.24E-23
NLRP3	CD27	0.65219213	6.14E-23
NLRP3	COLEC12	0.6525067	5.76E-23
NLRP3	SIRPA	0.65296112	5.26E-23
NLRP3	SIGLEC1	0.65343861	4.77E-23
NLRP3	CD83	0.65354848	4.67E-23
NLRP3	FBLN2	0.65378745	4.45E-23
NLRP3	CRISPLD2	0.65379081	4.45E-23
NLRP3	INMT	0.65504485	3.45E-23
NLRP3	CREM	0.65510324	3.41E-23
NLRP3	GM2A	0.65511725	3.4E-23
NLRP3	LHFPL6	0.65513051	3.39E-23
NLRP3	ABI3BP	0.65517417	3.36E-23
NLRP3	BOC	0.6552219	3.33E-23
NLRP3	EGR2	0.65522278	3.32E-23
NLRP3	RIPOR2	0.65547193	3.16E-23
NLRP3	ECM2	0.65597813	2.85E-23
NLRP3	RENBP	0.65611467	2.77E-23
NLRP3	MRAS	0.65616141	2.75E-23
NLRP3	SELL	0.65694836	2.34E-23
NLRP3	S1PR3	0.6575143	2.08E-23
NLRP3	SP140	0.65821275	1.8E-23
NLRP3	ETS1	0.65873457	1.62E-23
NLRP3	ERG	0.65879368	1.6E-23
NLRP3	CIITA	0.65913022	1.49E-23
NLRP3	RIN3	0.65918603	1.47E-23
NLRP3	GAS7	0.65940152	1.41E-23
NLRP3	SPARCL1	0.65946636	1.39E-23
NLRP3	C1orf54	0.65963761	1.34E-23
NLRP3	PRKCB	0.65981444	1.3E-23

NLRP3	CFP	0.66010884	1.22E-23
NLRP3	SLIT2	0.66029102	1.17E-23
NLRP3	TCF4	0.66047835	1.13E-23
NLRP3	DCN	0.66065566	1.09E-23
NLRP3	SIGLEC14	0.66093571	1.03E-23
NLRP3	SUSD3	0.66095073	1.02E-23
NLRP3	LPXN	0.66123995	9.63E-24
NLRP3	ENTPD1	0.66131938	9.47E-24
NLRP3	SFRP4	0.66148076	9.16E-24
NLRP3	TNFSF12	0.66175229	8.66E-24
NLRP3	THBS1	0.66175519	8.65E-24
NLRP3	RFTN1	0.66236383	7.62E-24
NLRP3	IL1R1	0.66258792	7.27E-24
NLRP3	LPAR1	0.66281802	6.93E-24
NLRP3	BCL2A1	0.66287292	6.85E-24
NLRP3	TLR4	0.6634192	6.11E-24
NLRP3	ADGRA2	0.66387416	5.55E-24
NLRP3	OLFML1	0.66461165	4.75E-24
NLRP3	FCGR3A	0.66498159	4.39E-24
NLRP3	LILRA2	0.66500235	4.37E-24
NLRP3	ARHGDIB	0.66508786	4.3E-24
NLRP3	CPXM2	0.66535931	4.06E-24
NLRP3	RASAL3	0.6656508	3.81E-24
NLRP3	CCR7	0.66565308	3.81E-24
NLRP3	ADORA3	0.66653971	3.16E-24
NLRP3	ADA2	0.66675283	3.02E-24
NLRP3	CCL5	0.66680912	2.98E-24
NLRP3	SYTL3	0.66715747	2.77E-24
NLRP3	HSD11B1	0.66734889	2.66E-24
NLRP3	DNAJC5B	0.66876446	1.96E-24
NLRP3	CD5	0.66904123	1.85E-24
NLRP3	JAM2	0.66929798	1.75E-24
NLRP3	FMO1	0.66948207	1.68E-24
NLRP3	PRF1	0.66959252	1.64E-24
NLRP3	CD52	0.66983191	1.56E-24
NLRP3	DCSTAMP	0.67050674	1.35E-24
NLRP3	PTPN7	0.67069568	1.3E-24
NLRP3	PTGS1	0.67117005	1.17E-24
NLRP3	CLEC4E	0.6712905	1.14E-24
NLRP3	GIMAP6	0.6714166	1.11E-24
NLRP3	CCN2	0.67168319	1.05E-24
NLRP3	ITGAM	0.67170861	1.04E-24
NLRP3	CD247	0.67289422	8.04E-25
NLRP3	GZMK	0.67296	7.93E-25
NLRP3	RHOH	0.67314191	7.62E-25
NLRP3	SH3BP5	0.67405795	6.24E-25
NLRP3	GIMAP5	0.67489342	5.2E-25
NLRP3	P2RY8	0.67570772	4.34E-25
NLRP3	RNASE2	0.6759187	4.15E-25
NLRP3	KCNAB2	0.67623892	3.86E-25
NLRP3	SERPINF1	0.67631217	3.8E-25
NLRP3	FNBP1	0.67668659	3.5E-25
NLRP3	HVCN1	0.67669933	3.49E-25
NLRP3	ZCCHC24	0.67670823	3.48E-25
NLRP3	SIRPG	0.67698424	3.28E-25
NLRP3	EFEMP1	0.67699492	3.27E-25
NLRP3	SLC1A3	0.67703965	3.24E-25
NLRP3	FCGR1B	0.67721629	3.11E-25

NLRP3	ENG	0.67789065	2.68E-25
NLRP3	MEF2C	0.67792671	2.66E-25
NLRP3	CSF1	0.67811595	2.55E-25
NLRP3	C1S	0.67834333	2.42E-25
NLRP3	CXCR6	0.67854689	2.32E-25
NLRP3	A2M	0.67880303	2.19E-25
NLRP3	F13A1	0.67901373	2.09E-25
NLRP3	CD14	0.67905989	2.07E-25
NLRP3	CD72	0.67908098	2.06E-25
NLRP3	SMAP2	0.67943758	1.9E-25
NLRP3	TLR2	0.67992047	1.7E-25
NLRP3	S1PR1	0.68111297	1.3E-25
NLRP3	CD96	0.6826905	9.13E-26
NLRP3	ST8SIA4	0.68269164	9.13E-26
NLRP3	LAT2	0.68282906	8.85E-26
NLRP3	ZEB2	0.68328343	7.98E-26
NLRP3	FCN1	0.68471987	5.76E-26
NLRP3	GFPT2	0.68494463	5.47E-26
NLRP3	CD300C	0.68500577	5.39E-26
NLRP3	DSE	0.68511597	5.26E-26
NLRP3	PIP4K2A	0.68544081	4.88E-26
NLRP3	COL14A1	0.68548915	4.83E-26
NLRP3	PECAM1	0.68563108	4.67E-26
NLRP3	GIMAP8	0.6865355	3.8E-26
NLRP3	GNB4	0.68674241	3.62E-26
NLRP3	CEACAM21	0.68701559	3.4E-26
NLRP3	EBI3	0.68708031	3.35E-26
NLRP3	GBGT1	0.68768582	2.91E-26
NLRP3	C1R	0.68798448	2.72E-26
NLRP3	CD3G	0.68820679	2.58E-26
NLRP3	DAB2	0.68873605	2.28E-26
NLRP3	TRAF3IP3	0.68874087	2.28E-26
NLRP3	ARHGAP31	0.688952	2.17E-26
NLRP3	PRCP	0.68946644	1.93E-26
NLRP3	DOCK8	0.68953911	1.89E-26
NLRP3	CALHM6	0.68961499	1.86E-26
NLRP3	HGF	0.68993394	1.73E-26
NLRP3	MRC1	0.69191301	1.09E-26
NLRP3	IL18BP	0.69275048	8.93E-27
NLRP3	LILRA5	0.69328961	7.86E-27
NLRP3	FGD2	0.69341405	7.63E-27
NLRP3	GYPC	0.6940247	6.61E-27
NLRP3	PRKCH	0.69459103	5.78E-27
NLRP3	FMNL1	0.69490799	5.36E-27
NLRP3	PODN	0.69494753	5.31E-27
NLRP3	PIK3CD	0.69543761	4.72E-27
NLRP3	PPM1M	0.6957236	4.41E-27
NLRP3	TNFAIP3	0.69683294	3.38E-27
NLRP3	GIMAP1-GIMAP5	0.69699043	3.26E-27
NLRP3	HLA-DMB	0.69780306	2.68E-27
NLRP3	CD8A	0.69784613	2.65E-27
NLRP3	MOXD1	0.69836211	2.34E-27
NLRP3	GPNMB	0.69841567	2.31E-27
NLRP3	CD2	0.69862095	2.2E-27
NLRP3	TMEM119	0.70018807	1.5E-27
NLRP3	CLEC10A	0.70041887	1.42E-27
NLRP3	MSR1	0.70043869	1.42E-27
NLRP3	CD300LF	0.70059411	1.36E-27

NLRP3	LRRC8C	0.70122735	1.17E-27
NLRP3	NCF1	0.70142421	1.11E-27
NLRP3	FOXP3	0.70181909	1.01E-27
NLRP3	ALOX5AP	0.70211141	9.41E-28
NLRP3	ITGA4	0.70251054	8.53E-28
NLRP3	CD180	0.70330835	7.01E-28
NLRP3	FYB1	0.70353944	6.62E-28
NLRP3	CXCR4	0.70417083	5.67E-28
NLRP3	MYO5A	0.70427766	5.52E-28
NLRP3	ITGB2	0.70484942	4.79E-28
NLRP3	CORO1A	0.70511314	4.49E-28
NLRP3	ACVRL1	0.70513467	4.46E-28
NLRP3	CD48	0.70553662	4.04E-28
NLRP3	DOCK10	0.70609538	3.51E-28
NLRP3	TESPA1	0.70610342	3.51E-28
NLRP3	DOK3	0.70613939	3.48E-28
NLRP3	CD93	0.70698933	2.81E-28
NLRP3	CD3E	0.7079582	2.2E-28
NLRP3	SH2B3	0.70868632	1.84E-28
NLRP3	GLIPR1	0.70872588	1.82E-28
NLRP3	GPR171	0.70935169	1.55E-28
NLRP3	CST7	0.71000029	1.32E-28
NLRP3	CYP1B1	0.71148033	9.04E-29
NLRP3	PLEKHO2	0.71192604	8.07E-29
NLRP3	CCL4	0.71210512	7.71E-29
NLRP3	HLA-DPA1	0.71266586	6.68E-29
NLRP3	TNFAIP8L3	0.71439459	4.28E-29
NLRP3	CCDC80	0.71494616	3.71E-29
NLRP3	ARHGAP25	0.71551032	3.21E-29
NLRP3	LCP1	0.71630995	2.61E-29
NLRP3	NCF4	0.71651031	2.47E-29
NLRP3	ITK	0.71826329	1.56E-29
NLRP3	PILRA	0.71857214	1.44E-29
NLRP3	ITGAX	0.7194054	1.16E-29
NLRP3	RGS1	0.72070285	8.21E-30
NLRP3	CTLA4	0.72083136	7.94E-30
NLRP3	GPR34	0.72124426	7.11E-30
NLRP3	FCGR2B	0.7213108	6.99E-30
NLRP3	CYTH4	0.72167816	6.34E-30
NLRP3	TIGIT	0.72220605	5.51E-30
NLRP3	DOCK11	0.72238278	5.25E-30
NLRP3	C5AR1	0.72254172	5.03E-30
NLRP3	HLA-DRA	0.72340544	3.99E-30
NLRP3	CD37	0.72341592	3.98E-30
NLRP3	LILRA6	0.72508873	2.54E-30
NLRP3	MMP19	0.72575228	2.12E-30
NLRP3	GAPT	0.72607196	1.94E-30
NLRP3	CCR5	0.7280741	1.13E-30
NLRP3	IL16	0.72931097	8.01E-31
NLRP3	ARHGAP15	0.72980507	6.99E-31
NLRP3	FGL2	0.73112706	4.85E-31
NLRP3	APBB1IP	0.73317494	2.74E-31
NLRP3	WIPF1	0.73459776	1.83E-31
NLRP3	STX11	0.73476661	1.75E-31
NLRP3	GLIPR2	0.73515213	1.57E-31
NLRP3	TNFRSF1B	0.73566668	1.36E-31
NLRP3	CCR1	0.73730929	8.5E-32
NLRP3	TYROBP	0.73739024	8.3E-32

NLRP3	CD69	0.73777613	7.43E-32
NLRP3	CD1D	0.73780735	7.37E-32
NLRP3	HLA-DOA	0.73789537	7.18E-32
NLRP3	NRROS	0.73874675	5.63E-32
NLRP3	HLA-DPB1	0.73979003	4.17E-32
NLRP3	IGSF6	0.73989389	4.04E-32
NLRP3	ARHGAP9	0.74008375	3.83E-32
NLRP3	ADAP2	0.74027677	3.62E-32
NLRP3	SIGLEC7	0.74029153	3.6E-32
NLRP3	ABI3	0.74067216	3.23E-32
NLRP3	FCGR2A	0.74094535	2.98E-32
NLRP3	CSF2RB	0.74186548	2.28E-32
NLRP3	LY96	0.74227474	2.02E-32
NLRP3	FMNL3	0.74248061	1.91E-32
NLRP3	SIGLEC10	0.74308097	1.6E-32
NLRP3	CMKLR1	0.74383121	1.28E-32
NLRP3	IKZF1	0.74468935	9.95E-33
NLRP3	MYO1G	0.745015	9.04E-33
NLRP3	C1QB	0.74503931	8.97E-33
NLRP3	TAGAP	0.74513279	8.73E-33
NLRP3	ARRB2	0.74692476	5.12E-33
NLRP3	VSIG4	0.74794206	3.78E-33
NLRP3	SNX20	0.74885671	2.87E-33
NLRP3	GMFG	0.74976323	2.18E-33
NLRP3	HCST	0.7502244	1.9E-33
NLRP3	SASH3	0.75111804	1.45E-33
NLRP3	FPR3	0.75138641	1.33E-33
NLRP3	AOAH	0.75174796	1.19E-33
NLRP3	CD163	0.75178712	1.18E-33
NLRP3	IL2RA	0.75180482	1.17E-33
NLRP3	FAM78A	0.75358748	6.78E-34
NLRP3	CXorf21	0.75434426	5.37E-34
NLRP3	CYTIP	0.75474788	4.74E-34
NLRP3	CELF2	0.75495634	4.44E-34
NLRP3	CD300A	0.75600659	3.2E-34
NLRP3	FCER1G	0.75623925	2.98E-34
NLRP3	ITGAL	0.75742895	2.05E-34
NLRP3	DPEP2	0.75856402	1.44E-34
NLRP3	DOK2	0.759042	1.24E-34
NLRP3	GIMAP4	0.75917656	1.18E-34
NLRP3	CLIC2	0.75923387	1.16E-34
NLRP3	C1QC	0.75928157	1.14E-34
NLRP3	C1QA	0.76069528	7.3E-35
NLRP3	KLHL6	0.76095608	6.72E-35
NLRP3	MNDA	0.76116986	6.28E-35
NLRP3	FLI1	0.76218037	4.54E-35
NLRP3	PREX1	0.76258874	3.98E-35
NLRP3	SLC9A9	0.763816	2.68E-35
NLRP3	NFAM1	0.7645136	2.14E-35
NLRP3	LSP1	0.76509051	1.77E-35
NLRP3	SPI1	0.76561973	1.49E-35
NLRP3	RCSD1	0.76624873	1.21E-35
NLRP3	SLCO2B1	0.76775616	7.38E-36
NLRP3	TMEM273	0.76826915	6.23E-36
NLRP3	FOLR2	0.76896654	4.94E-36
NLRP3	CLEC4A	0.7691007	4.72E-36
NLRP3	LST1	0.76938934	4.29E-36
NLRP3	LILRB4	0.76971316	3.85E-36

NLRP3	CLEC7A	0.77043667	3.02E-36
NLRP3	PTPRC	0.77117314	2.36E-36
NLRP3	MPEG1	0.7715613	2.07E-36
NLRP3	IL7R	0.77222654	1.66E-36
NLRP3	TNFAIP8L2	0.77242688	1.55E-36
NLRP3	RASSF2	0.77246988	1.53E-36
NLRP3	FPR1	0.7727758	1.38E-36
NLRP3	ARHGAP30	0.77281874	1.36E-36
NLRP3	JAML	0.77298985	1.28E-36
NLRP3	WDFY4	0.77322164	1.18E-36
NLRP3	SPN	0.77450413	7.64E-37
NLRP3	BTK	0.77741375	2.8E-37
NLRP3	PLEK	0.77748162	2.74E-37
NLRP3	ARHGEF6	0.77897814	1.62E-37
NLRP3	MS4A7	0.77904992	1.58E-37
NLRP3	HCK	0.77945177	1.38E-37
NLRP3	TNFSF8	0.78069327	8.89E-38
NLRP3	SCIMP	0.78173469	6.15E-38
NLRP3	BIN2	0.78184522	5.91E-38
NLRP3	PARVG	0.78224236	5.13E-38
NLRP3	TM6SF1	0.78227355	5.08E-38
NLRP3	GNGT2	0.78370214	3.05E-38
NLRP3	LY86	0.78530068	1.71E-38
NLRP3	SLAMF8	0.78648951	1.11E-38
NLRP3	EVI2B	0.78678896	9.98E-39
NLRP3	MYO1F	0.78680417	9.92E-39
NLRP3	CYBB	0.78820839	5.93E-39
NLRP3	FGR	0.78834349	5.64E-39
NLRP3	EVI2A	0.78864976	5.04E-39
NLRP3	C3AR1	0.79000765	3.05E-39
NLRP3	LAPTM5	0.79202542	1.44E-39
NLRP3	MS4A4A	0.79218044	1.36E-39
NLRP3	LILRB2	0.79261354	1.15E-39
NLRP3	CSF2RA	0.79446056	5.73E-40
NLRP3	THEMIS2	0.79769823	1.65E-40
NLRP3	HAVCR2	0.79875718	1.1E-40
NLRP3	AIF1	0.79968847	7.62E-41
NLRP3	CD33	0.80282886	2.2E-41
NLRP3	LRRC25	0.80428497	1.23E-41
NLRP3	GPR183	0.80569762	6.95E-42
NLRP3	IL10RA	0.80843946	2.27E-42
NLRP3	LCP2	0.81168644	5.87E-43
NLRP3	CSF1R	0.81202964	5.08E-43
NLRP3	WAS	0.81506054	1.4E-43
NLRP3	CD53	0.81519214	1.33E-43
NLRP3	LAIR1	0.81676974	6.72E-44
NLRP3	CD86	0.81958022	1.97E-44
NLRP3	HCLS1	0.82052831	1.3E-44
NLRP3	NCKAP1L	0.82157908	8.12E-45
NLRP3	MS4A6A	0.82233727	5.78E-45
NLRP3	DOCK2	0.82648392	8.8E-46
NLRP3	SRGN	0.83169499	7.68E-47
NLRP3	RNASE6	0.83237316	5.55E-47
NLRP3	C1orf162	0.83564819	1.14E-47
NLRP3	SAMSN1	0.84026502	1.15E-48
NLRP3	PIK3R5	0.84244484	3.81E-49
NLRP3	CD4	0.84942999	9.75E-51
NLRP3	SLA	0.85821161	7.39E-53

NLRP3	NLRP3	1	0
PDHA1	TBL1XR1	0.50008214	1.18E-12
PDHA1	PATL1	0.50023612	1.16E-12
PDHA1	MAX	0.5003089	1.15E-12
PDHA1	SQSTM1	0.50040964	1.14E-12
PDHA1	UBE2Z	0.50052273	1.12E-12
PDHA1	CSNK2B	0.50058864	1.12E-12
PDHA1	PRRC2C	0.50062525	1.11E-12
PDHA1	ATXN3	0.50068025	1.1E-12
PDHA1	DNAJB6	0.50074024	1.1E-12
PDHA1	INO80	0.50092225	1.07E-12
PDHA1	OSBPL3	0.5009807	1.06E-12
PDHA1	SMG9	0.50103715	1.06E-12
PDHA1	YEATS2	0.50107415	1.05E-12
PDHA1	TOR1B	0.50109583	1.05E-12
PDHA1	DPF2	0.50110089	1.05E-12
PDHA1	SMNDC1	0.50113178	1.05E-12
PDHA1	PPP6R1	0.50119853	1.04E-12
PDHA1	NUMB	0.50121389	1.04E-12
PDHA1	KIFC1	0.50136114	1.02E-12
PDHA1	ARF6	0.50140668	1.01E-12
PDHA1	DDX10	0.50141238	1.01E-12
PDHA1	EFL1	0.50144444	1.01E-12
PDHA1	PSMD1	0.50168858	9.78E-13
PDHA1	TPRA1	0.50169681	9.77E-13
PDHA1	POLR3K	0.50179475	9.65E-13
PDHA1	SDHAF3	0.50203143	9.38E-13
PDHA1	MED4	0.50207023	9.34E-13
PDHA1	PDAP1	0.50212063	9.28E-13
PDHA1	PARPBP	0.50212628	9.28E-13
PDHA1	RTF1	0.50215507	9.25E-13
PDHA1	MTG2	0.50229845	9.09E-13
PDHA1	CKS1B	0.50230126	9.08E-13
PDHA1	ASCC3	0.50230703	9.08E-13
PDHA1	YTHDF1	0.50231905	9.07E-13
PDHA1	RALB	0.50237817	9E-13
PDHA1	FANCD2	0.50255159	8.82E-13
PDHA1	OSBPL5	0.50260037	8.76E-13
PDHA1	RPP38	0.50268225	8.68E-13
PDHA1	STOML2	0.50279959	8.56E-13
PDHA1	GRAMD4	0.50285263	8.5E-13
PDHA1	NSDHL	0.50289202	8.46E-13
PDHA1	MICU1	0.50312548	8.23E-13
PDHA1	PSMD2	0.50328487	8.07E-13
PDHA1	PKM	0.50334261	8.01E-13
PDHA1	WDR76	0.50348193	7.88E-13
PDHA1	ATG5	0.50357262	7.8E-13
PDHA1	TMED9	0.50364482	7.73E-13
PDHA1	DHX35	0.50372415	7.65E-13
PDHA1	AVL9	0.50399098	7.41E-13
PDHA1	IARS2	0.50409871	7.32E-13
PDHA1	UBE2C	0.50414763	7.27E-13
PDHA1	DCPS	0.50422069	7.21E-13
PDHA1	NDUFA8	0.50425621	7.18E-13
PDHA1	ENO1	0.50429335	7.15E-13
PDHA1	ARL6IP1	0.50438975	7.06E-13
PDHA1	HS1BP3	0.50439188	7.06E-13
PDHA1	TEAD3	0.50440385	7.05E-13

PDHA1	MPHOSPH6	0.5044433	7.02E-13
PDHA1	AAGAB	0.50459403	6.89E-13
PDHA1	CDCA4	0.50462205	6.87E-13
PDHA1	DNAJC13	0.50462835	6.86E-13
PDHA1	OLA1	0.50470602	6.8E-13
PDHA1	SPTLC2	0.5047671	6.75E-13
PDHA1	CIAO2B	0.50482275	6.7E-13
PDHA1	INTS7	0.5048746	6.66E-13
PDHA1	TBC1D1	0.50492097	6.62E-13
PDHA1	IL4R	0.505109	6.47E-13
PDHA1	MRPL19	0.50512668	6.46E-13
PDHA1	MECP2	0.50517763	6.42E-13
PDHA1	HADHA	0.5052029	6.4E-13
PDHA1	IRF2	0.50521452	6.39E-13
PDHA1	CLTC	0.50521456	6.39E-13
PDHA1	TSN	0.5052499	6.36E-13
PDHA1	CALM3	0.50526443	6.35E-13
PDHA1	DUT	0.50530569	6.32E-13
PDHA1	POLR2H	0.50545365	6.21E-13
PDHA1	TMEM127	0.50561039	6.09E-13
PDHA1	C11orf98	0.50561619	6.09E-13
PDHA1	DYNC1H1	0.50566041	6.05E-13
PDHA1	YARS2	0.50566541	6.05E-13
PDHA1	POR	0.50569575	6.03E-13
PDHA1	CKAP5	0.50579239	5.96E-13
PDHA1	DBI	0.50589972	5.88E-13
PDHA1	ATXN7L3	0.50594256	5.85E-13
PDHA1	ZBTB7A	0.50602717	5.79E-13
PDHA1	SLC16A1	0.5060483	5.78E-13
PDHA1	MLKL	0.50607765	5.76E-13
PDHA1	NUF2	0.50612328	5.72E-13
PDHA1	MED10	0.50620668	5.67E-13
PDHA1	COQ5	0.50632804	5.58E-13
PDHA1	ERGIC1	0.50640023	5.53E-13
PDHA1	YTHDF2	0.50660599	5.4E-13
PDHA1	ABCF1	0.50673621	5.31E-13
PDHA1	MDH2	0.50676883	5.29E-13
PDHA1	YY1AP1	0.50687404	5.22E-13
PDHA1	SUMO2	0.50708824	5.09E-13
PDHA1	SMIM30	0.50711399	5.07E-13
PDHA1	DIAPH1	0.50712004	5.07E-13
PDHA1	RUFY1	0.50715643	5.05E-13
PDHA1	KIF20A	0.50719561	5.02E-13
PDHA1	CCT7	0.50720377	5.02E-13
PDHA1	C6orf47	0.50723463	5E-13
PDHA1	TSFM	0.50726558	4.98E-13
PDHA1	TOMM40L	0.50732403	4.94E-13
PDHA1	CDK2AP1	0.50733058	4.94E-13
PDHA1	DAZAP2	0.50753922	4.82E-13
PDHA1	CHMP4A	0.50758801	4.79E-13
PDHA1	SNX1	0.50771161	4.72E-13
PDHA1	NUP205	0.50777131	4.68E-13
PDHA1	PIF1	0.50780551	4.66E-13
PDHA1	LSM14B	0.50783865	4.64E-13
PDHA1	IRF1	0.50787158	4.62E-13
PDHA1	SNRPC	0.50828283	4.4E-13
PDHA1	MOB1A	0.50835074	4.36E-13
PDHA1	FARSA	0.50839306	4.34E-13

PDHA1	TOR3A	0.50839607	4.34E-13
PDHA1	FAM114A1	0.5084461	4.31E-13
PDHA1	FAM32A	0.5084533	4.31E-13
PDHA1	PRXL2B	0.50858526	4.24E-13
PDHA1	HUS1	0.50869162	4.18E-13
PDHA1	PCTP	0.50869458	4.18E-13
PDHA1	TUBA1B	0.5087357	4.16E-13
PDHA1	DDX19B	0.50876579	4.14E-13
PDHA1	CCDC34	0.50878459	4.13E-13
PDHA1	B4GALT5	0.50882491	4.11E-13
PDHA1	CDC23	0.50888767	4.08E-13
PDHA1	CENPL	0.50889462	4.08E-13
PDHA1	MIER2	0.50901399	4.02E-13
PDHA1	KLF3	0.50907088	3.99E-13
PDHA1	URM1	0.50918759	3.94E-13
PDHA1	DAXX	0.50921189	3.92E-13
PDHA1	ATP5F1B	0.50926174	3.9E-13
PDHA1	PSMA1	0.50952933	3.77E-13
PDHA1	TTC4	0.5095334	3.77E-13
PDHA1	SLC38A7	0.50956384	3.76E-13
PDHA1	FTSJ1	0.50962145	3.73E-13
PDHA1	CASP6	0.50986804	3.62E-13
PDHA1	HNRNPR	0.50995514	3.58E-13
PDHA1	RNF220	0.51002859	3.55E-13
PDHA1	COA6	0.51010506	3.51E-13
PDHA1	CHCHD2	0.51021813	3.47E-13
PDHA1	PARP3	0.51025109	3.45E-13
PDHA1	RIOK2	0.51027357	3.44E-13
PDHA1	NOLC1	0.51034102	3.41E-13
PDHA1	RNASEH2A	0.51034149	3.41E-13
PDHA1	NUDCD3	0.51041129	3.38E-13
PDHA1	DCTN5	0.51052284	3.34E-13
PDHA1	UHRF1	0.51068533	3.27E-13
PDHA1	BORA	0.51072015	3.26E-13
PDHA1	PLRG1	0.51083712	3.21E-13
PDHA1	RNASEH1	0.51086758	3.2E-13
PDHA1	TAX1BP1	0.51087118	3.2E-13
PDHA1	HSBP1	0.51098245	3.15E-13
PDHA1	TAPBP	0.5111144	3.1E-13
PDHA1	TRIOBP	0.51114322	3.09E-13
PDHA1	INTS14	0.5112839	3.04E-13
PDHA1	CEBPZOS	0.51136913	3.01E-13
PDHA1	GSTK1	0.51137301	3.01E-13
PDHA1	MID1	0.51138458	3E-13
PDHA1	GTPBP4	0.51144775	2.98E-13
PDHA1	DLGAP4	0.511533	2.95E-13
PDHA1	OXNAD1	0.51155635	2.94E-13
PDHA1	SDF4	0.51155809	2.94E-13
PDHA1	PKNOX1	0.5116311	2.91E-13
PDHA1	RFC3	0.51163707	2.91E-13
PDHA1	ANKRD39	0.51165706	2.9E-13
PDHA1	PTP4A2	0.51166544	2.9E-13
PDHA1	DBNDD2	0.51169338	2.89E-13
PDHA1	MOV10	0.51172709	2.88E-13
PDHA1	SH3BP2	0.51174116	2.87E-13
PDHA1	TPD52L2	0.51185255	2.83E-13
PDHA1	CAPNS1	0.5119535	2.8E-13
PDHA1	NDUFB4	0.51197104	2.79E-13

PDHA1	SNW1	0.51198376	2.79E-13
PDHA1	TUBB4B	0.51199371	2.78E-13
PDHA1	MAZ	0.51199592	2.78E-13
PDHA1	PSMB8	0.51200972	2.78E-13
PDHA1	TOP1	0.51202379	2.77E-13
PDHA1	TIPRL	0.51205273	2.76E-13
PDHA1	APH1A	0.51224799	2.7E-13
PDHA1	PDCD10	0.51230704	2.68E-13
PDHA1	COPS5	0.51233178	2.67E-13
PDHA1	MRPL22	0.51235195	2.66E-13
PDHA1	METAP1	0.51245329	2.63E-13
PDHA1	SAPCD2	0.51263869	2.57E-13
PDHA1	TMEM62	0.51265761	2.56E-13
PDHA1	TXNDC12	0.51277494	2.53E-13
PDHA1	BAZ1A	0.51279297	2.52E-13
PDHA1	SNRPA	0.51281065	2.51E-13
PDHA1	PYGO2	0.51286213	2.5E-13
PDHA1	TACC3	0.51290856	2.48E-13
PDHA1	MARK2	0.51294819	2.47E-13
PDHA1	CTPS1	0.5129708	2.47E-13
PDHA1	PPIH	0.51300068	2.46E-13
PDHA1	PAICS	0.51306859	2.44E-13
PDHA1	WAPL	0.5130787	2.43E-13
PDHA1	PAQR4	0.51322029	2.39E-13
PDHA1	DYNC1I2	0.51322955	2.39E-13
PDHA1	RABIF	0.51346349	2.32E-13
PDHA1	NCOA6	0.51360094	2.28E-13
PDHA1	CTBP2	0.51365851	2.26E-13
PDHA1	PSMD12	0.51365949	2.26E-13
PDHA1	NDC80	0.51379708	2.22E-13
PDHA1	STK24	0.51382615	2.22E-13
PDHA1	COG7	0.5138725	2.2E-13
PDHA1	DTYMK	0.51395704	2.18E-13
PDHA1	PUDP	0.51396514	2.18E-13
PDHA1	C16orf91	0.51397726	2.17E-13
PDHA1	MRM2	0.51398597	2.17E-13
PDHA1	CCNK	0.51398979	2.17E-13
PDHA1	MLH3	0.51408585	2.15E-13
PDHA1	DCTPP1	0.51426356	2.1E-13
PDHA1	STIP1	0.51426855	2.1E-13
PDHA1	MRPS30	0.51432421	2.08E-13
PDHA1	PCSK7	0.51453715	2.03E-13
PDHA1	ISG20L2	0.51459186	2.01E-13
PDHA1	RAD54L	0.51467501	1.99E-13
PDHA1	XPO6	0.51471803	1.98E-13
PDHA1	GOLPH3	0.51476267	1.97E-13
PDHA1	CPSF6	0.51480751	1.96E-13
PDHA1	IL13RA1	0.51487482	1.94E-13
PDHA1	COQ7	0.51488721	1.94E-13
PDHA1	B3GNT5	0.51509237	1.89E-13
PDHA1	CDK7	0.51519559	1.87E-13
PDHA1	RAB5B	0.51523728	1.86E-13
PDHA1	MRPL44	0.5152986	1.84E-13
PDHA1	ECHS1	0.51537867	1.82E-13
PDHA1	PSMD10	0.51546498	1.81E-13
PDHA1	TMX2	0.51559385	1.78E-13
PDHA1	TRIM14	0.51563376	1.77E-13
PDHA1	SPAG5	0.51576718	1.74E-13

PDHA1	DBF4	0.51583305	1.72E-13
PDHA1	HELZ2	0.51594101	1.7E-13
PDHA1	SUMO1	0.51602977	1.68E-13
PDHA1	TMEM44	0.51626534	1.63E-13
PDHA1	CHMP1A	0.51643296	1.6E-13
PDHA1	TNIP2	0.51649161	1.59E-13
PDHA1	HNRNPA0	0.51654052	1.58E-13
PDHA1	ENY2	0.51655709	1.57E-13
PDHA1	XRCC5	0.51656293	1.57E-13
PDHA1	HPS1	0.51660524	1.56E-13
PDHA1	KDELR1	0.5166155	1.56E-13
PDHA1	SRA1	0.51678283	1.53E-13
PDHA1	FAM122B	0.51680261	1.53E-13
PDHA1	HSPA14	0.51681283	1.52E-13
PDHA1	LYPLA2	0.51681853	1.52E-13
PDHA1	TRA2B	0.51682705	1.52E-13
PDHA1	NCAPG	0.51695408	1.5E-13
PDHA1	BAX	0.51695552	1.5E-13
PDHA1	DLD	0.51696483	1.49E-13
PDHA1	NRBP1	0.51696651	1.49E-13
PDHA1	ACSL3	0.51697776	1.49E-13
PDHA1	CHEK2	0.51707916	1.47E-13
PDHA1	CDC25B	0.51710942	1.47E-13
PDHA1	R3HCC1L	0.51712099	1.47E-13
PDHA1	BANF1	0.51713391	1.46E-13
PDHA1	MICOS10	0.51713778	1.46E-13
PDHA1	RMI2	0.51719697	1.45E-13
PDHA1	BECN1	0.51727128	1.44E-13
PDHA1	WDHD1	0.51731362	1.43E-13
PDHA1	DAGLB	0.51734244	1.43E-13
PDHA1	WDR62	0.51747834	1.4E-13
PDHA1	EIF6	0.51783464	1.34E-13
PDHA1	ZNF526	0.51787576	1.33E-13
PDHA1	NELFB	0.51794162	1.32E-13
PDHA1	TM9SF4	0.51796067	1.32E-13
PDHA1	SHCBP1	0.51802536	1.31E-13
PDHA1	RCE1	0.51802794	1.31E-13
PDHA1	ELK1	0.51804091	1.31E-13
PDHA1	COPA	0.5183409	1.26E-13
PDHA1	MCM4	0.5183471	1.26E-13
PDHA1	GLO1	0.51838436	1.25E-13
PDHA1	YWHAZ	0.51848238	1.23E-13
PDHA1	GRK6	0.51861081	1.21E-13
PDHA1	SCO1	0.5186536	1.21E-13
PDHA1	HSPA4	0.51872053	1.2E-13
PDHA1	SLC10A3	0.51875639	1.19E-13
PDHA1	BCAP31	0.51881774	1.18E-13
PDHA1	EXOC1	0.51883429	1.18E-13
PDHA1	UTP3	0.51893043	1.17E-13
PDHA1	GALK2	0.51897184	1.16E-13
PDHA1	LYAR	0.51899012	1.16E-13
PDHA1	TTK	0.51899978	1.16E-13
PDHA1	RRP8	0.51904331	1.15E-13
PDHA1	SNUPN	0.51929043	1.11E-13
PDHA1	ATP5MG	0.51930098	1.11E-13
PDHA1	SMG7	0.51946588	1.09E-13
PDHA1	NR1H2	0.51946719	1.09E-13
PDHA1	ILF2	0.51951643	1.08E-13

PDHA1	CEP55	0.51964342	1.07E-13
PDHA1	NMI	0.51967001	1.06E-13
PDHA1	RPN1	0.51977228	1.05E-13
PDHA1	COG4	0.51980099	1.04E-13
PDHA1	SUGT1	0.52014542	9.99E-14
PDHA1	FUNDC1	0.52020227	9.92E-14
PDHA1	ASPM	0.52025659	9.85E-14
PDHA1	STARD7	0.5202896	9.81E-14
PDHA1	HMMR	0.52029384	9.8E-14
PDHA1	FAM83E	0.52038494	9.69E-14
PDHA1	CDC123	0.52069302	9.32E-14
PDHA1	DESI1	0.52073784	9.26E-14
PDHA1	RDH14	0.52076944	9.23E-14
PDHA1	PSMC1	0.52094301	9.03E-14
PDHA1	CAST	0.52094352	9.02E-14
PDHA1	HDAC2	0.52095694	9.01E-14
PDHA1	GRPEL1	0.52102092	8.94E-14
PDHA1	NDUFS3	0.5210362	8.92E-14
PDHA1	MGST3	0.52116317	8.78E-14
PDHA1	FANCA	0.52125938	8.67E-14
PDHA1	EIF1AX	0.5213694	8.55E-14
PDHA1	MKLN1	0.52138236	8.53E-14
PDHA1	PSEN1	0.52138911	8.53E-14
PDHA1	ING1	0.5219534	7.93E-14
PDHA1	RNF149	0.52199376	7.89E-14
PDHA1	FAM189B	0.5220394	7.84E-14
PDHA1	PITPNB	0.52218778	7.7E-14
PDHA1	MAPK3	0.52221863	7.67E-14
PDHA1	WIPI2	0.52231336	7.57E-14
PDHA1	NOL7	0.52233397	7.55E-14
PDHA1	APEX1	0.52235868	7.53E-14
PDHA1	SNRPG	0.52256043	7.34E-14
PDHA1	CNPPD1	0.52257264	7.33E-14
PDHA1	ACOT7	0.52271786	7.19E-14
PDHA1	COPB1	0.52274854	7.16E-14
PDHA1	TXN	0.5229047	7.02E-14
PDHA1	STX4	0.52298733	6.95E-14
PDHA1	JRKL	0.52319836	6.76E-14
PDHA1	SF3B6	0.52326152	6.71E-14
PDHA1	AQR	0.52331343	6.66E-14
PDHA1	KPNA2	0.52335025	6.63E-14
PDHA1	MRPL47	0.52347796	6.52E-14
PDHA1	PKP4	0.52351082	6.49E-14
PDHA1	ARHGAP26	0.5236221	6.4E-14
PDHA1	IRAK1	0.52376141	6.29E-14
PDHA1	HAUS8	0.52378083	6.27E-14
PDHA1	NDUFB5	0.52389069	6.18E-14
PDHA1	RAB11A	0.52391018	6.17E-14
PDHA1	VTI1A	0.52415408	5.98E-14
PDHA1	MRPL11	0.52422801	5.92E-14
PDHA1	ZFAND6	0.52427461	5.88E-14
PDHA1	PURB	0.52432874	5.84E-14
PDHA1	LRR1	0.52455019	5.68E-14
PDHA1	CAPZA1	0.52457846	5.66E-14
PDHA1	ERVK3-1	0.52459355	5.65E-14
PDHA1	AIFM2	0.52461585	5.63E-14
PDHA1	SEPHS2	0.52466563	5.59E-14
PDHA1	MCM2	0.52478892	5.51E-14

PDHA1	AMBRA1	0.52480488	5.49E-14
PDHA1	TNFRSF14	0.52490028	5.43E-14
PDHA1	IQGAP3	0.52491756	5.42E-14
PDHA1	SRSF3	0.52497907	5.37E-14
PDHA1	SLC25A13	0.52506904	5.31E-14
PDHA1	SUB1	0.52521282	5.21E-14
PDHA1	TIMM8B	0.52528302	5.17E-14
PDHA1	UBE2L3	0.52535555	5.12E-14
PDHA1	SKA3	0.52538339	5.1E-14
PDHA1	CTNNA1	0.52543308	5.07E-14
PDHA1	NDUFS1	0.52545531	5.05E-14
PDHA1	SIKE1	0.5255002	5.02E-14
PDHA1	IPO9	0.52555266	4.99E-14
PDHA1	UCK2	0.52557406	4.97E-14
PDHA1	PRDX1	0.52561973	4.94E-14
PDHA1	IQGAP1	0.52567003	4.91E-14
PDHA1	TAF12	0.525702	4.89E-14
PDHA1	CCDC58	0.52584736	4.8E-14
PDHA1	ARPC3	0.52586585	4.79E-14
PDHA1	ZCCHC9	0.52590355	4.77E-14
PDHA1	TMEM170A	0.52593849	4.74E-14
PDHA1	PPP4C	0.52601402	4.7E-14
PDHA1	UXS1	0.5260517	4.68E-14
PDHA1	SF3B4	0.52619656	4.59E-14
PDHA1	SERBP1	0.52654891	4.38E-14
PDHA1	VDAC2	0.52691226	4.18E-14
PDHA1	DUSP11	0.52702623	4.12E-14
PDHA1	SRP14	0.52711537	4.07E-14
PDHA1	MND1	0.52725249	4E-14
PDHA1	GNL2	0.52725804	4E-14
PDHA1	MCM6	0.52734782	3.95E-14
PDHA1	CARD19	0.52745462	3.89E-14
PDHA1	AXIN1	0.52752049	3.86E-14
PDHA1	FASTKD2	0.52758955	3.83E-14
PDHA1	NDE1	0.52799982	3.63E-14
PDHA1	ARPC5	0.52805161	3.6E-14
PDHA1	VAPB	0.52817899	3.54E-14
PDHA1	KIF18B	0.52819431	3.54E-14
PDHA1	RTF2	0.52838815	3.45E-14
PDHA1	RFC2	0.52855483	3.37E-14
PDHA1	SEC13	0.52865253	3.33E-14
PDHA1	PLK4	0.52869677	3.31E-14
PDHA1	EXO1	0.52875561	3.29E-14
PDHA1	CASP3	0.52929458	3.06E-14
PDHA1	TOMM34	0.52930616	3.06E-14
PDHA1	CUL1	0.52932494	3.05E-14
PDHA1	CIAO2A	0.52948498	2.99E-14
PDHA1	ARHGAP17	0.52955077	2.96E-14
PDHA1	HMGN1	0.5297141	2.9E-14
PDHA1	GAR1	0.52973715	2.89E-14
PDHA1	RAB1A	0.52989363	2.83E-14
PDHA1	PLCB3	0.5299159	2.82E-14
PDHA1	TROAP	0.53005513	2.77E-14
PDHA1	RAB9A	0.53009386	2.76E-14
PDHA1	ECD	0.53009966	2.75E-14
PDHA1	NUTF2	0.53009991	2.75E-14
PDHA1	TPX2	0.53017409	2.73E-14
PDHA1	TMEM165	0.53019679	2.72E-14

PDHA1	SNAPIN	0.53045367	2.63E-14
PDHA1	AMMECR1	0.53048719	2.62E-14
PDHA1	PRPF4	0.5305035	2.61E-14
PDHA1	CHEK1	0.53054689	2.6E-14
PDHA1	DNAJA2	0.53062231	2.57E-14
PDHA1	TANK	0.53073663	2.53E-14
PDHA1	C2CD3	0.53083037	2.5E-14
PDHA1	DDB1	0.53086176	2.49E-14
PDHA1	DGAT1	0.53099523	2.45E-14
PDHA1	TIMMDC1	0.53110525	2.41E-14
PDHA1	TRUB2	0.53114479	2.4E-14
PDHA1	DCAF6	0.53114595	2.4E-14
PDHA1	MRPL15	0.53122552	2.37E-14
PDHA1	IST1	0.53123624	2.37E-14
PDHA1	PSME2	0.53133894	2.34E-14
PDHA1	RELA	0.53168275	2.24E-14
PDHA1	NEK6	0.5317878	2.2E-14
PDHA1	NKIRAS2	0.53179307	2.2E-14
PDHA1	PTTG1	0.53185219	2.19E-14
PDHA1	LRRC57	0.53187504	2.18E-14
PDHA1	NANS	0.53199966	2.14E-14
PDHA1	ZDHHC5	0.53214587	2.1E-14
PDHA1	VCP	0.53223362	2.08E-14
PDHA1	SRC	0.53257115	1.99E-14
PDHA1	ELOVL1	0.53267663	1.96E-14
PDHA1	TSEN34	0.5326859	1.96E-14
PDHA1	PAK1	0.5328524	1.91E-14
PDHA1	NUP98	0.53328852	1.81E-14
PDHA1	ASF1B	0.53359393	1.73E-14
PDHA1	DOLPP1	0.53373979	1.7E-14
PDHA1	RAB8A	0.53376764	1.69E-14
PDHA1	NACC1	0.53405975	1.63E-14
PDHA1	STAU1	0.53427158	1.58E-14
PDHA1	USB1	0.534527	1.53E-14
PDHA1	E2F8	0.534607	1.52E-14
PDHA1	EFCAB11	0.5346342	1.51E-14
PDHA1	BCCIP	0.53469379	1.5E-14
PDHA1	PSMD3	0.53471205	1.49E-14
PDHA1	HNRNPF	0.5347138	1.49E-14
PDHA1	OIP5	0.53480088	1.48E-14
PDHA1	KDM2A	0.53485461	1.47E-14
PDHA1	MRPS36	0.53491017	1.46E-14
PDHA1	CSE1L	0.53497227	1.44E-14
PDHA1	MPDU1	0.53499937	1.44E-14
PDHA1	MTX1	0.53508796	1.42E-14
PDHA1	CALM2	0.53518347	1.4E-14
PDHA1	WDR12	0.53524525	1.39E-14
PDHA1	NCL	0.5352455	1.39E-14
PDHA1	CHMP5	0.5352615	1.39E-14
PDHA1	TRAF2	0.53531819	1.38E-14
PDHA1	OTUB1	0.53550223	1.34E-14
PDHA1	CCT8	0.53579578	1.29E-14
PDHA1	CENPK	0.53580137	1.29E-14
PDHA1	CENPH	0.53590021	1.27E-14
PDHA1	HSPE1	0.53597423	1.26E-14
PDHA1	GINS1	0.5361077	1.24E-14
PDHA1	PCLAF	0.53635186	1.2E-14
PDHA1	UBE2K	0.53649522	1.18E-14

PDHA1	MELK	0.53664776	1.15E-14
PDHA1	SLC35A4	0.53673551	1.14E-14
PDHA1	SUV39H1	0.5367859	1.13E-14
PDHA1	VRK1	0.53681339	1.13E-14
PDHA1	EIF1AD	0.53704442	1.09E-14
PDHA1	CDC37	0.53721628	1.07E-14
PDHA1	HTATIP2	0.53752163	1.02E-14
PDHA1	PDIA4	0.53765084	1.01E-14
PDHA1	CDCA5	0.53769281	1E-14
PDHA1	ANP32B	0.53779705	9.88E-15
PDHA1	ARL4A	0.53787146	9.78E-15
PDHA1	HNRNPU	0.53795383	9.67E-15
PDHA1	AAR2	0.53799291	9.62E-15
PDHA1	NCAPH	0.53802593	9.57E-15
PDHA1	MRPS16	0.53818472	9.37E-15
PDHA1	DUS2	0.53840174	9.1E-15
PDHA1	LSM12	0.53852915	8.94E-15
PDHA1	CPOX	0.53855086	8.92E-15
PDHA1	PSME1	0.53865483	8.79E-15
PDHA1	TMEM138	0.53867957	8.76E-15
PDHA1	ATP5PD	0.53892644	8.48E-15
PDHA1	CENPF	0.53900977	8.38E-15
PDHA1	FAHD1	0.53936951	7.98E-15
PDHA1	METTL21A	0.5395409	7.8E-15
PDHA1	GLRX3	0.53959247	7.74E-15
PDHA1	DPM1	0.53975605	7.57E-15
PDHA1	ALDOA	0.53978798	7.54E-15
PDHA1	STX6	0.53982997	7.5E-15
PDHA1	CNOT9	0.53983874	7.49E-15
PDHA1	RTRAF	0.53987371	7.45E-15
PDHA1	SRSF7	0.53988714	7.44E-15
PDHA1	IDH3G	0.5399152	7.41E-15
PDHA1	CLN3	0.53998212	7.35E-15
PDHA1	ZDHHC9	0.53998602	7.34E-15
PDHA1	PGAM1	0.54017043	7.16E-15
PDHA1	PFKP	0.54075831	6.61E-15
PDHA1	ZFYVE19	0.54081888	6.55E-15
PDHA1	TMEM179B	0.54082198	6.55E-15
PDHA1	THOC3	0.54089711	6.48E-15
PDHA1	MGAT4B	0.54104214	6.36E-15
PDHA1	PPME1	0.54131948	6.12E-15
PDHA1	PLGRKT	0.54136675	6.08E-15
PDHA1	LAMTOR5	0.54144302	6.02E-15
PDHA1	FARSB	0.54145092	6.01E-15
PDHA1	MVP	0.54147068	6E-15
PDHA1	CLN6	0.5415115	5.96E-15
PDHA1	TCF19	0.54151878	5.96E-15
PDHA1	RMDN3	0.5415484	5.93E-15
PDHA1	PGK1	0.54174649	5.77E-15
PDHA1	HNRNPK	0.54175652	5.77E-15
PDHA1	ARMH3	0.54179558	5.74E-15
PDHA1	VPS29	0.54206691	5.53E-15
PDHA1	RANBP1	0.54229317	5.36E-15
PDHA1	RPIA	0.5423619	5.31E-15
PDHA1	PPP1R11	0.54298759	4.87E-15
PDHA1	GPI	0.54325391	4.7E-15
PDHA1	EIF5A	0.54340466	4.6E-15
PDHA1	CENPU	0.54378984	4.36E-15

PDHA1	SSBP1	0.54382431	4.34E-15
PDHA1	DLST	0.54397091	4.26E-15
PDHA1	IL10RB	0.54415757	4.15E-15
PDHA1	TMEM250	0.54437636	4.02E-15
PDHA1	SPG21	0.54445013	3.98E-15
PDHA1	ZNF207	0.54446149	3.98E-15
PDHA1	COMMD7	0.54448949	3.96E-15
PDHA1	ZFP64	0.54461611	3.89E-15
PDHA1	CDK1	0.54464501	3.88E-15
PDHA1	DDX19A	0.5446738	3.86E-15
PDHA1	STAMBP	0.54472161	3.84E-15
PDHA1	IQSEC2	0.5449073	3.74E-15
PDHA1	MRPL18	0.54500639	3.69E-15
PDHA1	AKAP1	0.54507807	3.65E-15
PDHA1	KIF15	0.54509837	3.64E-15
PDHA1	BUB1	0.54518535	3.6E-15
PDHA1	CLNS1A	0.54518617	3.6E-15
PDHA1	BMS1	0.54527638	3.55E-15
PDHA1	HMGN4	0.54534719	3.52E-15
PDHA1	MKI67	0.54564595	3.38E-15
PDHA1	TNIP1	0.54585767	3.28E-15
PDHA1	ZC3H18	0.54588544	3.27E-15
PDHA1	PHACTR4	0.54596455	3.23E-15
PDHA1	CTTN	0.54615233	3.15E-15
PDHA1	OTUD5	0.5461879	3.13E-15
PDHA1	MTHFD1	0.54621451	3.12E-15
PDHA1	MAF1	0.54624872	3.11E-15
PDHA1	THOC6	0.54628486	3.09E-15
PDHA1	PTMA	0.54637412	3.05E-15
PDHA1	RMDN1	0.54660417	2.96E-15
PDHA1	PSMA2	0.54663128	2.95E-15
PDHA1	ODF2	0.54667475	2.93E-15
PDHA1	MRPL17	0.54678602	2.88E-15
PDHA1	CYFIP1	0.54682284	2.87E-15
PDHA1	DHDDS	0.54691785	2.83E-15
PDHA1	TUFM	0.54699416	2.8E-15
PDHA1	C1orf112	0.54734466	2.67E-15
PDHA1	CENPO	0.54750834	2.61E-15
PDHA1	FAM118B	0.54753828	2.6E-15
PDHA1	UQCRC1	0.54781539	2.5E-15
PDHA1	ATG9A	0.54782316	2.49E-15
PDHA1	MAPK6	0.54783362	2.49E-15
PDHA1	PRIM2	0.5478361	2.49E-15
PDHA1	USP10	0.54799657	2.44E-15
PDHA1	YWHAB	0.54813094	2.39E-15
PDHA1	PHKG2	0.54827504	2.34E-15
PDHA1	CENPW	0.54843182	2.29E-15
PDHA1	BRCC3	0.54844276	2.29E-15
PDHA1	CCNB2	0.5484946	2.27E-15
PDHA1	ITGB3BP	0.54873184	2.2E-15
PDHA1	TIMM17A	0.54874042	2.19E-15
PDHA1	TIMM8A	0.54879428	2.18E-15
PDHA1	SNRPB	0.54886046	2.16E-15
PDHA1	PSMC2	0.54890355	2.15E-15
PDHA1	POMP	0.5489048	2.14E-15
PDHA1	ERH	0.5489878	2.12E-15
PDHA1	CDC25C	0.54902342	2.11E-15
PDHA1	CANT1	0.54919731	2.06E-15

PDHA1	SMC4	0.54928878	2.03E-15
PDHA1	SLC35C1	0.54933303	2.02E-15
PDHA1	TNPO3	0.54939626	2E-15
PDHA1	TTLL5	0.54952813	1.97E-15
PDHA1	CMPK1	0.54964274	1.93E-15
PDHA1	PPA2	0.54967931	1.92E-15
PDHA1	IK	0.54969968	1.92E-15
PDHA1	HELLS	0.5499221	1.86E-15
PDHA1	HNRNPM	0.55002289	1.83E-15
PDHA1	PSMD14	0.55022761	1.78E-15
PDHA1	DAB2IP	0.5503052	1.76E-15
PDHA1	KHDRBS1	0.55038172	1.74E-15
PDHA1	EBP	0.55042311	1.73E-15
PDHA1	NUDT5	0.55058785	1.69E-15
PDHA1	SSB	0.55151759	1.49E-15
PDHA1	FADD	0.55169829	1.45E-15
PDHA1	LSM8	0.55178463	1.43E-15
PDHA1	PDHX	0.55187589	1.41E-15
PDHA1	SDHD	0.55189444	1.41E-15
PDHA1	SF3B2	0.55241214	1.31E-15
PDHA1	PDE6D	0.55254804	1.28E-15
PDHA1	PHF19	0.55276034	1.25E-15
PDHA1	RNF5	0.5528037	1.24E-15
PDHA1	GPN2	0.55288832	1.22E-15
PDHA1	KNL1	0.55291053	1.22E-15
PDHA1	KIF23	0.55300223	1.2E-15
PDHA1	LRRC41	0.55324768	1.16E-15
PDHA1	MRPL3	0.55330751	1.15E-15
PDHA1	TDP1	0.55343239	1.13E-15
PDHA1	TFE3	0.55348949	1.12E-15
PDHA1	GPBP1L1	0.55349005	1.12E-15
PDHA1	PAIP1	0.55356738	1.11E-15
PDHA1	TARS2	0.55370862	1.09E-15
PDHA1	LMNB2	0.55381488	1.07E-15
PDHA1	TEP1	0.55389675	1.06E-15
PDHA1	ESPL1	0.55393678	1.05E-15
PDHA1	NCBP2	0.55416593	1.02E-15
PDHA1	IKBKG	0.55445462	9.79E-16
PDHA1	NDUFAF5	0.55453425	9.68E-16
PDHA1	POLR2D	0.55474542	9.39E-16
PDHA1	TRIM28	0.55481586	9.3E-16
PDHA1	HDGF	0.55516109	8.85E-16
PDHA1	NUP62	0.55554912	8.37E-16
PDHA1	RCC1L	0.55558841	8.32E-16
PDHA1	KDELR2	0.55581212	8.06E-16
PDHA1	AKIP1	0.55581926	8.05E-16
PDHA1	GUCD1	0.55582334	8.05E-16
PDHA1	CHD1L	0.55617319	7.66E-16
PDHA1	PA2G4	0.55630878	7.51E-16
PDHA1	PPP2R5D	0.55632505	7.49E-16
PDHA1	SIGMAR1	0.55637971	7.43E-16
PDHA1	NDC1	0.55657513	7.23E-16
PDHA1	TUBA1C	0.55666156	7.14E-16
PDHA1	ETFA	0.55667424	7.12E-16
PDHA1	MARCKSL1	0.5568496	6.95E-16
PDHA1	TSR2	0.55706465	6.73E-16
PDHA1	CCNF	0.55716723	6.64E-16
PDHA1	FBXL19	0.55778081	6.07E-16

PDHA1	DNAAF5	0.55781344	6.05E-16
PDHA1	CCDC22	0.55814424	5.76E-16
PDHA1	MTFMT	0.5584946	5.48E-16
PDHA1	CHAF1A	0.55856668	5.42E-16
PDHA1	FEN1	0.5586379	5.37E-16
PDHA1	ANP32A	0.55883926	5.21E-16
PDHA1	HNRNPA3	0.55891512	5.16E-16
PDHA1	MAPKAP1	0.55892757	5.15E-16
PDHA1	SNX5	0.55918964	4.96E-16
PDHA1	ENSA	0.55923884	4.92E-16
PDHA1	LASP1	0.55933913	4.85E-16
PDHA1	HGH1	0.55981994	4.52E-16
PDHA1	ZMYND19	0.56007479	4.36E-16
PDHA1	TBC1D7	0.56027071	4.24E-16
PDHA1	MED8	0.56043157	4.14E-16
PDHA1	G6PD	0.56043355	4.14E-16
PDHA1	PARN	0.56047488	4.11E-16
PDHA1	TTC9C	0.56049466	4.1E-16
PDHA1	CCNB1	0.56062331	4.02E-16
PDHA1	COX5B	0.5607584	3.95E-16
PDHA1	SAE1	0.56084779	3.89E-16
PDHA1	AIMP1	0.56100749	3.8E-16
PDHA1	GTSE1	0.5610241	3.8E-16
PDHA1	ZNF143	0.56108346	3.76E-16
PDHA1	AKT1	0.56114786	3.73E-16
PDHA1	RAD51	0.56118875	3.71E-16
PDHA1	MCM3	0.56130805	3.64E-16
PDHA1	PCBP1	0.56134845	3.62E-16
PDHA1	GRK2	0.56144524	3.57E-16
PDHA1	ZWILCH	0.56148556	3.55E-16
PDHA1	LAS1L	0.56168508	3.45E-16
PDHA1	ACTL6A	0.56224975	3.17E-16
PDHA1	MRGBP	0.56228264	3.16E-16
PDHA1	AAMP	0.56229424	3.15E-16
PDHA1	HNRNPC	0.56237386	3.12E-16
PDHA1	NUP37	0.56259633	3.02E-16
PDHA1	MRPL48	0.56270433	2.97E-16
PDHA1	RNF26	0.56298512	2.85E-16
PDHA1	ARF1	0.5632636	2.73E-16
PDHA1	PPP1CA	0.56339659	2.68E-16
PDHA1	GNG5	0.5636515	2.58E-16
PDHA1	TTYH3	0.56367303	2.57E-16
PDHA1	RCC1	0.56370953	2.56E-16
PDHA1	CYB5B	0.56383412	2.51E-16
PDHA1	ATP5PB	0.56387421	2.5E-16
PDHA1	IDH1	0.56390445	2.49E-16
PDHA1	SDHA	0.56397516	2.46E-16
PDHA1	ATG16L1	0.56409315	2.42E-16
PDHA1	MED14	0.56413273	2.41E-16
PDHA1	DNPEP	0.56413925	2.4E-16
PDHA1	CCT5	0.56415125	2.4E-16
PDHA1	CORO1B	0.56432236	2.34E-16
PDHA1	NSFL1C	0.56435241	2.33E-16
PDHA1	PPIA	0.56447941	2.29E-16
PDHA1	ZNF768	0.56489893	2.15E-16
PDHA1	CHMP4B	0.56495168	2.13E-16
PDHA1	MTERF3	0.5649589	2.13E-16
PDHA1	PPM1G	0.56498621	2.12E-16

PDHA1	RACGAP1	0.56504313	2.1E-16
PDHA1	PFKL	0.56521335	2.05E-16
PDHA1	PSMC6	0.56539746	2E-16
PDHA1	C14orf119	0.56542378	1.99E-16
PDHA1	SSRP1	0.56571072	1.91E-16
PDHA1	CASP2	0.56578146	1.89E-16
PDHA1	BRIX1	0.56606433	1.81E-16
PDHA1	SNRPB2	0.56612121	1.79E-16
PDHA1	KIF2C	0.5661532	1.78E-16
PDHA1	WDR1	0.56705296	1.56E-16
PDHA1	HCFC1	0.56733732	1.5E-16
PDHA1	NUDT15	0.5674362	1.47E-16
PDHA1	RRM2	0.56771214	1.42E-16
PDHA1	CMC2	0.56779954	1.4E-16
PDHA1	BLOC1S4	0.56805175	1.35E-16
PDHA1	SAP30L	0.56807688	1.34E-16
PDHA1	SRSF1	0.56815395	1.33E-16
PDHA1	RNF40	0.56853242	1.25E-16
PDHA1	POLG	0.56866373	1.23E-16
PDHA1	BUB1B	0.56901831	1.16E-16
PDHA1	PLK1	0.56992669	1.02E-16
PDHA1	TMEM183A	0.57022609	9.71E-17
PDHA1	BZW2	0.57064157	9.12E-17
PDHA1	CYC1	0.57070069	9.04E-17
PDHA1	PSMA3	0.57076584	8.96E-17
PDHA1	SUPT16H	0.57098032	8.67E-17
PDHA1	GHITM	0.57108305	8.54E-17
PDHA1	KCTD5	0.57127309	8.3E-17
PDHA1	EIF4E2	0.57132867	8.23E-17
PDHA1	HDAC8	0.57149969	8.02E-17
PDHA1	MAD2L1	0.57158053	7.92E-17
PDHA1	FIP1L1	0.5717112	7.76E-17
PDHA1	NDUFAB1	0.57191848	7.53E-17
PDHA1	PCNA	0.57198342	7.45E-17
PDHA1	LEO1	0.57215859	7.26E-17
PDHA1	CCNA2	0.57243767	6.96E-17
PDHA1	KNOP1	0.57248404	6.91E-17
PDHA1	TFG	0.57286627	6.52E-17
PDHA1	SNX12	0.57292248	6.46E-17
PDHA1	RFC4	0.57309324	6.3E-17
PDHA1	TIMM23	0.57355749	5.87E-17
PDHA1	AC106886.5	0.57357301	5.85E-17
PDHA1	DNAJC21	0.57396813	5.51E-17
PDHA1	UBE2A	0.57399229	5.49E-17
PDHA1	GSK3A	0.57401511	5.47E-17
PDHA1	RUSC1	0.57447943	5.1E-17
PDHA1	RAB1B	0.57475751	4.89E-17
PDHA1	RNPS1	0.57479926	4.86E-17
PDHA1	HDAC1	0.57497898	4.73E-17
PDHA1	SDHB	0.57500113	4.71E-17
PDHA1	METTL2B	0.57558689	4.31E-17
PDHA1	EIF4G1	0.57561043	4.29E-17
PDHA1	ZDHHC6	0.57588129	4.12E-17
PDHA1	EIF3I	0.57613555	3.96E-17
PDHA1	PDSS1	0.57663826	3.66E-17
PDHA1	CKS2	0.57707974	3.42E-17
PDHA1	HNRNPUL2	0.57712977	3.4E-17
PDHA1	TARDBP	0.57718581	3.37E-17

PDHA1	NDUFB3	0.57726678	3.33E-17
PDHA1	RPN2	0.57759961	3.16E-17
PDHA1	PSMB2	0.57765043	3.14E-17
PDHA1	MDH1	0.57776875	3.08E-17
PDHA1	AK2	0.57798576	2.98E-17
PDHA1	MITD1	0.5781359	2.91E-17
PDHA1	AURKA	0.57824579	2.86E-17
PDHA1	KIF4A	0.57829453	2.84E-17
PDHA1	KIF11	0.57838037	2.8E-17
PDHA1	SDHC	0.57856684	2.72E-17
PDHA1	CCDC137	0.57857985	2.72E-17
PDHA1	UNC45A	0.57881287	2.62E-17
PDHA1	FANCI	0.57882716	2.61E-17
PDHA1	NCAPG2	0.57889692	2.59E-17
PDHA1	CCT6A	0.57908581	2.51E-17
PDHA1	COX15	0.57909398	2.51E-17
PDHA1	SYAP1	0.57922029	2.46E-17
PDHA1	DNAJC2	0.57965805	2.3E-17
PDHA1	PSME4	0.57969972	2.28E-17
PDHA1	HMGB1	0.57996617	2.19E-17
PDHA1	ALG8	0.58005764	2.16E-17
PDHA1	DHFR	0.58058475	1.99E-17
PDHA1	MGME1	0.5807646	1.93E-17
PDHA1	EIF2B2	0.58081547	1.92E-17
PDHA1	COPG1	0.58106665	1.85E-17
PDHA1	NIPBL	0.58209794	1.57E-17
PDHA1	EMC8	0.58213255	1.56E-17
PDHA1	EIF2AK1	0.58288797	1.39E-17
PDHA1	INCENP	0.58296067	1.37E-17
PDHA1	POLE3	0.58350962	1.26E-17
PDHA1	PDCL3	0.58358655	1.24E-17
PDHA1	NUSAP1	0.58435181	1.1E-17
PDHA1	MIS18A	0.58459248	1.06E-17
PDHA1	GGCT	0.58464078	1.05E-17
PDHA1	BAK1	0.58477734	1.03E-17
PDHA1	DDX23	0.58509045	9.8E-18
PDHA1	TUT7	0.58511171	9.77E-18
PDHA1	BUB3	0.5856741	8.94E-18
PDHA1	HMGB2	0.58577159	8.8E-18
PDHA1	NAP1L4	0.58578244	8.79E-18
PDHA1	LMNB1	0.5859996	8.49E-18
PDHA1	NIF3L1	0.58607855	8.38E-18
PDHA1	CBX3	0.58608598	8.37E-18
PDHA1	SNRPA1	0.58665988	7.64E-18
PDHA1	ARCN1	0.58679401	7.48E-18
PDHA1	TPR	0.58688539	7.37E-18
PDHA1	TYMS	0.58703636	7.2E-18
PDHA1	PHB	0.5876187	6.56E-18
PDHA1	RAB5IF	0.58845775	5.74E-18
PDHA1	UBAP2L	0.58867676	5.54E-18
PDHA1	MAIP1	0.58899617	5.26E-18
PDHA1	TBC1D10B	0.59001515	4.47E-18
PDHA1	RAE1	0.59044851	4.17E-18
PDHA1	FH	0.59158441	3.47E-18
PDHA1	ELAVL1	0.59192209	3.28E-18
PDHA1	KNSTRN	0.5919347	3.28E-18
PDHA1	TCOF1	0.59196627	3.26E-18
PDHA1	PSMA4	0.59197023	3.26E-18

PDHA1	SNRPD3	0.59199244	3.25E-18
PDHA1	EXOSC3	0.59221648	3.13E-18
PDHA1	PRPF38A	0.59253216	2.97E-18
PDHA1	MASTL	0.59278719	2.85E-18
PDHA1	EFTUD2	0.59305463	2.73E-18
PDHA1	UBE2V1	0.5930702	2.73E-18
PDHA1	PTBP1	0.59313308	2.7E-18
PDHA1	ORC6	0.59335787	2.6E-18
PDHA1	GTF2F2	0.59345864	2.56E-18
PDHA1	STIL	0.59382573	2.41E-18
PDHA1	ACO2	0.59457783	2.13E-18
PDHA1	COA4	0.59507332	1.97E-18
PDHA1	SLC35C2	0.59530964	1.89E-18
PDHA1	MTX2	0.59563831	1.79E-18
PDHA1	CXorf38	0.59578498	1.75E-18
PDHA1	DARS2	0.59589989	1.72E-18
PDHA1	PSMA7	0.59620023	1.63E-18
PDHA1	PSMD7	0.59671202	1.5E-18
PDHA1	MTA2	0.59793654	1.23E-18
PDHA1	HNRNPD	0.59813709	1.19E-18
PDHA1	SMG5	0.59839305	1.14E-18
PDHA1	CFL1	0.59872659	1.08E-18
PDHA1	PDCD6	0.59954683	9.39E-19
PDHA1	IWS1	0.59961827	9.28E-19
PDHA1	CYCS	0.59991049	8.84E-19
PDHA1	PRC1	0.60055492	7.94E-19
PDHA1	CNOT1	0.60065917	7.8E-19
PDHA1	DYNLT1	0.6009808	7.39E-19
PDHA1	HK1	0.60209365	6.14E-19
PDHA1	UBE2I	0.60250588	5.73E-19
PDHA1	PSMA5	0.60255262	5.68E-19
PDHA1	PREP	0.6026027	5.63E-19
PDHA1	COX5A	0.60351177	4.83E-19
PDHA1	AIFM1	0.60384979	4.57E-19
PDHA1	RALY	0.60435067	4.2E-19
PDHA1	GPKOW	0.60500893	3.75E-19
PDHA1	MAGOH	0.60571855	3.33E-19
PDHA1	TP53I11	0.60582706	3.27E-19
PDHA1	CNOT11	0.60720369	2.58E-19
PDHA1	UBE2T	0.60849092	2.07E-19
PDHA1	RNPEP	0.6093713	1.78E-19
PDHA1	PFN1	0.60938018	1.78E-19
PDHA1	POLA2	0.61001273	1.59E-19
PDHA1	NASP	0.61060038	1.44E-19
PDHA1	SF3B3	0.61136187	1.26E-19
PDHA1	RRM1	0.612019	1.13E-19
PDHA1	MAST2	0.61237659	1.06E-19
PDHA1	DDX27	0.61254698	1.03E-19
PDHA1	USP39	0.61269277	1E-19
PDHA1	UQCRH	0.61310721	9.32E-20
PDHA1	ZDHHC16	0.61316965	9.22E-20
PDHA1	GYS1	0.61336167	8.91E-20
PDHA1	GOT2	0.61336978	8.9E-20
PDHA1	RCC2	0.61401182	7.96E-20
PDHA1	CDCA8	0.61488719	6.82E-20
PDHA1	UNG	0.61687413	4.81E-20
PDHA1	HARS2	0.6176762	4.17E-20
PDHA1	MRPL16	0.61828021	3.75E-20

PDHA1	DHX9	0.61950321	3.01E-20
PDHA1	PDZD11	0.61968059	2.92E-20
PDHA1	U2AF2	0.61988093	2.82E-20
PDHA1	DSN1	0.62052443	2.51E-20
PDHA1	MRPL35	0.62074904	2.41E-20
PDHA1	MTCH2	0.62166239	2.05E-20
PDHA1	OGDH	0.62265904	1.71E-20
PDHA1	VDAC1	0.62268441	1.7E-20
PDHA1	HNRNPAB	0.62305406	1.59E-20
PDHA1	NIT2	0.62430058	1.27E-20
PDHA1	CIAPIN1	0.62502008	1.11E-20
PDHA1	PSRC1	0.62507554	1.1E-20
PDHA1	DPP3	0.62555578	1.01E-20
PDHA1	MRPL30	0.625732	9.79E-21
PDHA1	NDUFS2	0.62659545	8.36E-21
PDHA1	DNAJC9	0.62670861	8.19E-21
PDHA1	GPSM2	0.62702921	7.72E-21
PDHA1	SLC35A2	0.6283712	6.03E-21
PDHA1	CSTF2	0.62946031	4.93E-21
PDHA1	GSS	0.63461456	1.88E-21
PDHA1	ZWINT	0.63562186	1.56E-21
PDHA1	PBDC1	0.63571383	1.53E-21
PDHA1	SNRNP200	0.63667733	1.28E-21
PDHA1	CHCHD3	0.64074604	5.85E-22
PDHA1	AIMP2	0.64098171	5.59E-22
PDHA1	MRPS5	0.64179818	4.78E-22
PDHA1	RBBP7	0.64698585	1.73E-22
PDHA1	IMMT	0.6486873	1.24E-22
PDHA1	CDK16	0.64951314	1.05E-22
PDHA1	COX7B	0.65006096	9.41E-23
PDHA1	APEX2	0.65050411	8.61E-23
PDHA1	HNRNPA2B1	0.65539406	3.21E-23
PDHA1	HNRNPL	0.66224575	7.81E-24
PDHA1	TBRG4	0.66447694	4.89E-24
PDHA1	HCCS	0.66733175	2.67E-24
PDHA1	UBA1	0.66851927	2.07E-24
PDHA1	ATP5MC3	0.68230752	9.96E-26
PDHA1	UQCRC2	0.68304702	8.42E-26
PDHA1	SMS	0.68726531	3.21E-26
PDHB	PDHB	1	0
PDHB	DYNC1LI1	0.82923206	2.46E-46
PDHB	UBA3	0.81736367	5.19E-44
PDHB	WDR82	0.81333502	2.93E-43
PDHB	GLT8D1	0.78988161	3.2E-39
PDHB	TOP2B	0.78287883	4.09E-38
PDHB	MLH1	0.77708908	3.14E-37
PDHB	ARL8B	0.77216158	1.69E-36
PDHB	LARS2	0.77134118	2.23E-36
PDHB	ELP6	0.76342689	3.04E-35
PDHB	PPP1R8	0.76042259	7.97E-35
PDHB	DPH3	0.75036534	1.82E-33
PDHB	ARL6IP5	0.74667741	5.51E-33
PDHB	EIF1B	0.74543382	7.98E-33
PDHB	GNPDA2	0.74468277	9.97E-33
PDHB	THOC7	0.74327089	1.51E-32
PDHB	GNAI2	0.73956234	4.45E-32
PDHB	EID1	0.7385307	5.99E-32
PDHB	CCDC71	0.73850905	6.03E-32

PDHB	DNAJC8	0.7358826	1.28E-31
PDHB	EIF4G3	0.73498874	1.64E-31
PDHB	FBXL5	0.73466977	1.8E-31
PDHB	MAP4	0.73404687	2.14E-31
PDHB	VAMP3	0.73365425	2.39E-31
PDHB	CALM1	0.73205818	3.74E-31
PDHB	ZYG11B	0.72998568	6.65E-31
PDHB	TARDBP	0.72935281	7.92E-31
PDHB	DENND6A	0.7292319	8.19E-31
PDHB	RHOA	0.72725412	1.41E-30
PDHB	WDR47	0.72604015	1.96E-30
PDHB	COPS8	0.72525434	2.43E-30
PDHB	ZCCHC17	0.72510912	2.52E-30
PDHB	METTL6	0.72444609	3.02E-30
PDHB	SH3BGRL	0.72359338	3.8E-30
PDHB	TSN	0.7220772	5.7E-30
PDHB	YWHAQ	0.72060303	8.43E-30
PDHB	ICMT	0.7200356	9.8E-30
PDHB	SKP1	0.71728798	2.02E-29
PDHB	RYBP	0.71641699	2.53E-29
PDHB	SELENOF	0.71565267	3.09E-29
PDHB	SGCB	0.71423699	4.46E-29
PDHB	TMEM126B	0.71369606	5.13E-29
PDHB	THRAP3	0.71339137	5.54E-29
PDHB	RAB28	0.71304853	6.05E-29
PDHB	PCNP	0.71272222	6.58E-29
PDHB	SNX2	0.71239617	7.16E-29
PDHB	SNRNP27	0.71218826	7.55E-29
PDHB	HNRNPR	0.71200562	7.91E-29
PDHB	STX12	0.7110624	1.01E-28
PDHB	BRK1	0.71066458	1.11E-28
PDHB	MFN2	0.71035187	1.2E-28
PDHB	KHDRBS1	0.71018899	1.26E-28
PDHB	GNB1	0.7098449	1.37E-28
PDHB	HNRNPH2	0.70963945	1.44E-28
PDHB	MFAP1	0.70895809	1.71E-28
PDHB	EMC3	0.70793072	2.22E-28
PDHB	RNF11	0.70740156	2.53E-28
PDHB	ACTR8	0.70648075	3.19E-28
PDHB	RRAGA	0.70587981	3.71E-28
PDHB	CBX1	0.70521056	4.38E-28
PDHB	CZIB	0.70428194	5.51E-28
PDHB	ARL14EP	0.70399531	5.92E-28
PDHB	MRFAP1L1	0.70312426	7.33E-28
PDHB	TTC1	0.70273469	8.07E-28
PDHB	ATP5PB	0.70231595	8.95E-28
PDHB	RAB5A	0.70159899	1.07E-27
PDHB	AKTIP	0.70150939	1.09E-27
PDHB	LARP7	0.70146205	1.1E-27
PDHB	CAPZB	0.70030342	1.46E-27
PDHB	RAP1A	0.70026918	1.47E-27
PDHB	EIF4G2	0.69993387	1.6E-27
PDHB	PRKACA	0.69982122	1.64E-27
PDHB	HIGD1A	0.6988723	2.07E-27
PDHB	SLMAP	0.69886883	2.07E-27
PDHB	FBXO7	0.69871649	2.15E-27
PDHB	UBE2Q2	0.698574	2.22E-27
PDHB	SSB	0.6984732	2.28E-27

PDHB	EAPP	0.69836381	2.34E-27
PDHB	KCTD10	0.69809038	2.5E-27
PDHB	DPF2	0.69807105	2.51E-27
PDHB	SLC35A5	0.69787228	2.63E-27
PDHB	C3orf38	0.69732211	3.01E-27
PDHB	SLC4A1AP	0.69721505	3.09E-27
PDHB	CDC42	0.69720909	3.09E-27
PDHB	UBE2N	0.69703763	3.22E-27
PDHB	C9orf78	0.69691453	3.32E-27
PDHB	ESD	0.69668681	3.5E-27
PDHB	MFSD14A	0.69603876	4.09E-27
PDHB	YPEL5	0.69597575	4.15E-27
PDHB	SZRD1	0.69550971	4.64E-27
PDHB	ADH5	0.6952455	4.94E-27
PDHB	BCAS2	0.69384941	6.89E-27
PDHB	MAPRE1	0.69378498	6.99E-27
PDHB	PPP4R2	0.69351075	7.46E-27
PDHB	RNF220	0.69334562	7.76E-27
PDHB	UBE2E1	0.69320985	8.01E-27
PDHB	EAF1	0.69203678	1.06E-26
PDHB	ARMC1	0.691773	1.12E-26
PDHB	PTPRA	0.69047576	1.52E-26
PDHB	RBFOX2	0.68969529	1.83E-26
PDHB	MRFAP1	0.68941315	1.95E-26
PDHB	ATP6V1A	0.68931685	2E-26
PDHB	RAB3GAP1	0.68897023	2.16E-26
PDHB	TCTN3	0.6887056	2.3E-26
PDHB	DYNC1I2	0.68828787	2.53E-26
PDHB	ATP6V1E1	0.68804315	2.68E-26
PDHB	SF3A3	0.68783282	2.81E-26
PDHB	YTHDF2	0.68754636	3.01E-26
PDHB	DDX19A	0.68715128	3.29E-26
PDHB	ATG3	0.68693772	3.46E-26
PDHB	PDCL	0.68609573	4.2E-26
PDHB	ARPC4	0.6856966	4.6E-26
PDHB	HNRNPK	0.6847431	5.72E-26
PDHB	DHX36	0.68460352	5.91E-26
PDHB	DCAF1	0.68395353	6.85E-26
PDHB	HYAL2	0.6838596	7E-26
PDHB	UROD	0.68364874	7.35E-26
PDHB	EPC2	0.68326018	8.02E-26
PDHB	ACTR1A	0.68319488	8.14E-26
PDHB	SETD3	0.68312819	8.27E-26
PDHB	RPA2	0.68253513	9.46E-26
PDHB	AAR2	0.6824335	9.68E-26
PDHB	IFT52	0.68216648	1.03E-25
PDHB	SLC25A12	0.68204329	1.06E-25
PDHB	MED4	0.68169954	1.14E-25
PDHB	STARD3NL	0.68160123	1.17E-25
PDHB	NSL1	0.68155854	1.18E-25
PDHB	FBXO8	0.68066672	1.44E-25
PDHB	HNRNPLL	0.68057455	1.47E-25
PDHB	STT3B	0.68005543	1.65E-25
PDHB	FYCO1	0.67987082	1.72E-25
PDHB	MATR3	0.67958257	1.84E-25
PDHB	PPP3CB	0.67921938	1.99E-25
PDHB	RTF1	0.67891427	2.13E-25
PDHB	API5	0.67857092	2.3E-25

PDHB	SAR1A	0.67856055	2.31E-25
PDHB	KLHL18	0.67842957	2.38E-25
PDHB	UNC50	0.67822645	2.49E-25
PDHB	TMEM167B	0.67821429	2.49E-25
PDHB	EPS15	0.67817855	2.51E-25
PDHB	DNAJA2	0.678105	2.55E-25
PDHB	GNG10	0.67805807	2.58E-25
PDHB	AC010132.3	0.67803053	2.6E-25
PDHB	BLOC1S6	0.67773873	2.77E-25
PDHB	PPP1CB	0.67765736	2.82E-25
PDHB	FTO	0.67760725	2.85E-25
PDHB	SNX17	0.67714133	3.16E-25
PDHB	ZNHIT6	0.67678716	3.42E-25
PDHB	LAPTM4A	0.67636094	3.76E-25
PDHB	PFKM	0.67598425	4.09E-25
PDHB	KIAA1143	0.67539279	4.66E-25
PDHB	C11orf58	0.67525449	4.8E-25
PDHB	MEAF6	0.67513545	4.93E-25
PDHB	HS2ST1	0.67511631	4.95E-25
PDHB	FAM168A	0.67503261	5.04E-25
PDHB	ASNSD1	0.67453555	5.62E-25
PDHB	SUCLA2	0.67439812	5.79E-25
PDHB	DOCK7	0.67435096	5.85E-25
PDHB	PWP1	0.67428947	5.93E-25
PDHB	RTL6	0.67421414	6.03E-25
PDHB	MORF4L1	0.673997	6.32E-25
PDHB	PFDN1	0.67385207	6.53E-25
PDHB	SNX3	0.67364744	6.83E-25
PDHB	MFSD1	0.67355203	6.97E-25
PDHB	BTBD1	0.67351523	7.03E-25
PDHB	MTMR2	0.67318874	7.54E-25
PDHB	ARMC8	0.6731231	7.65E-25
PDHB	CSDE1	0.67309648	7.7E-25
PDHB	METTL9	0.67294674	7.95E-25
PDHB	SPIN1	0.67290356	8.03E-25
PDHB	PPP1CC	0.67226205	9.23E-25
PDHB	UBE2D2	0.67221429	9.33E-25
PDHB	GLB1	0.67212046	9.52E-25
PDHB	OAZ2	0.67126547	1.15E-24
PDHB	FAM98B	0.67110941	1.19E-24
PDHB	ZNF410	0.67099791	1.21E-24
PDHB	HAT1	0.67062634	1.32E-24
PDHB	RANBP9	0.67046811	1.36E-24
PDHB	CSNK1A1	0.67034138	1.4E-24
PDHB	TOR1AIP1	0.66979396	1.57E-24
PDHB	GOLGA7	0.6693119	1.75E-24
PDHB	ABHD6	0.66906614	1.84E-24
PDHB	LRRC41	0.66899407	1.87E-24
PDHB	PRNP	0.66885782	1.93E-24
PDHB	TERF1	0.66872571	1.98E-24
PDHB	SRP14	0.668539	2.06E-24
PDHB	TM2D1	0.66849576	2.08E-24
PDHB	RPF1	0.6681829	2.22E-24
PDHB	MBTPS1	0.66814062	2.24E-24
PDHB	DEGS1	0.66812597	2.25E-24
PDHB	ARF4	0.66785889	2.38E-24
PDHB	NCBP2	0.66744866	2.6E-24
PDHB	TUBA1B	0.66724795	2.72E-24

PDHB	PAFAH1B2	0.66705125	2.83E-24
PDHB	ANAPC10	0.66704585	2.84E-24
PDHB	MDH1	0.66659614	3.12E-24
PDHB	LRRFIP2	0.66655245	3.15E-24
PDHB	TADA3	0.66636462	3.28E-24
PDHB	ANXA7	0.66608278	3.48E-24
PDHB	RAB7A	0.66530346	4.1E-24
PDHB	RAB14	0.66529937	4.11E-24
PDHB	BMPR2	0.66525064	4.15E-24
PDHB	AP2M1	0.66510414	4.28E-24
PDHB	CBY1	0.66502246	4.36E-24
PDHB	MXRA7	0.66499205	4.38E-24
PDHB	FBXL3	0.66486194	4.51E-24
PDHB	RTN4	0.66470171	4.66E-24
PDHB	ACTR6	0.66465144	4.71E-24
PDHB	GINM1	0.66459069	4.77E-24
PDHB	PPP1R2	0.66445265	4.91E-24
PDHB	HMGB1	0.66438427	4.98E-24
PDHB	KATNAL1	0.66400337	5.4E-24
PDHB	TRAPPC6B	0.66398742	5.42E-24
PDHB	LNPEP	0.66397206	5.44E-24
PDHB	TMEM230	0.6637048	5.75E-24
PDHB	PPHLN1	0.66366729	5.8E-24
PDHB	GABPB1	0.66358306	5.9E-24
PDHB	UBQLN2	0.66328702	6.28E-24
PDHB	HNRNPA2B1	0.6626783	7.13E-24
PDHB	COPS4	0.66266931	7.15E-24
PDHB	DHDDS	0.66219348	7.89E-24
PDHB	BTF3L4	0.66215237	7.96E-24
PDHB	SH3GLB1	0.66207955	8.08E-24
PDHB	ARPP19	0.66191488	8.37E-24
PDHB	TOR1A	0.66155517	9.02E-24
PDHB	PUM1	0.66148169	9.16E-24
PDHB	C5orf24	0.66129078	9.53E-24
PDHB	USP1	0.66119612	9.72E-24
PDHB	SELENOT	0.66080361	1.05E-23
PDHB	EXT2	0.66062211	1.1E-23
PDHB	ABL1	0.66034429	1.16E-23
PDHB	CDK2AP1	0.66031591	1.17E-23
PDHB	RPA1	0.66016266	1.2E-23
PDHB	DUSP11	0.6601133	1.22E-23
PDHB	SNW1	0.66003221	1.24E-23
PDHB	FBXW2	0.65995678	1.26E-23
PDHB	WBP4	0.65985859	1.28E-23
PDHB	RNF14	0.65949244	1.38E-23
PDHB	WDR1	0.65944681	1.4E-23
PDHB	ZNF644	0.65934078	1.43E-23
PDHB	OGFOD1	0.65918869	1.47E-23
PDHB	RAB5B	0.65909083	1.5E-23
PDHB	KPNA3	0.65884343	1.58E-23
PDHB	CNPY4	0.65867595	1.64E-23
PDHB	PPP2R5D	0.65856128	1.68E-23
PDHB	YWHAB	0.65820627	1.81E-23
PDHB	NKIRAS1	0.65806388	1.86E-23
PDHB	STIM1	0.65795209	1.9E-23
PDHB	MFSD14B	0.6574713	2.1E-23
PDHB	ANKRD13C	0.65746767	2.1E-23
PDHB	ILK	0.65733831	2.16E-23

PDHB	AP003108.2	0.65731328	2.17E-23
PDHB	PAIP2	0.65717428	2.23E-23
PDHB	PPT1	0.65716124	2.24E-23
PDHB	C5orf51	0.657047	2.29E-23
PDHB	TAF13	0.65633266	2.65E-23
PDHB	KLHL2	0.65597493	2.85E-23
PDHB	FEM1B	0.65597452	2.85E-23
PDHB	FAM98A	0.65587905	2.91E-23
PDHB	SLC25A32	0.65587242	2.91E-23
PDHB	KPNA6	0.65579174	2.96E-23
PDHB	CIR1	0.6555543	3.11E-23
PDHB	GBE1	0.65546911	3.16E-23
PDHB	THUMPD3	0.65546726	3.16E-23
PDHB	RNF13	0.65533825	3.25E-23
PDHB	RAB18	0.65529237	3.28E-23
PDHB	TMEM43	0.65500706	3.47E-23
PDHB	SNX1	0.65498364	3.49E-23
PDHB	TTC4	0.65492055	3.54E-23
PDHB	MPHOSPH10	0.65455872	3.81E-23
PDHB	TMTC3	0.6543521	3.97E-23
PDHB	RAB5C	0.6542395	4.06E-23
PDHB	MAP1A	0.65391179	4.34E-23
PDHB	GNB5	0.6539093	4.34E-23
PDHB	GTF2E1	0.65381784	4.42E-23
PDHB	CUL1	0.65380976	4.43E-23
PDHB	MIER1	0.65380892	4.43E-23
PDHB	LZIC	0.65378391	4.45E-23
PDHB	LEPROT	0.6535136	4.7E-23
PDHB	VAMP7	0.65310058	5.11E-23
PDHB	UTP11	0.65304244	5.17E-23
PDHB	RAB6A	0.65300247	5.21E-23
PDHB	MAP1LC3B	0.65284254	5.39E-23
PDHB	PJA2	0.65282634	5.4E-23
PDHB	FBXO42	0.65282388	5.41E-23
PDHB	UBLCP1	0.6528085	5.42E-23
PDHB	ZBTB4	0.65277608	5.46E-23
PDHB	PIGK	0.65273355	5.51E-23
PDHB	PDE6D	0.65233494	5.97E-23
PDHB	ZMYM4	0.65227765	6.03E-23
PDHB	NRBP1	0.65193518	6.47E-23
PDHB	CTBS	0.65178297	6.67E-23
PDHB	ZNF639	0.65164504	6.85E-23
PDHB	NIPSNAP3A	0.65152541	7.02E-23
PDHB	SMIM12	0.65134388	7.28E-23
PDHB	CCDC90B	0.65118055	7.52E-23
PDHB	ZMAT2	0.65108206	7.67E-23
PDHB	TTL	0.65102939	7.75E-23
PDHB	ARL2BP	0.65093018	7.91E-23
PDHB	VIPAS39	0.65075522	8.19E-23
PDHB	ARF3	0.65059839	8.45E-23
PDHB	SLC9A6	0.65051877	8.59E-23
PDHB	PALLD	0.65046654	8.68E-23
PDHB	RHEB	0.65045993	8.69E-23
PDHB	CETN3	0.65034918	8.89E-23
PDHB	SHOC2	0.65028361	9E-23
PDHB	CDC23	0.65015507	9.24E-23
PDHB	KTI12	0.65000669	9.51E-23
PDHB	PHTF1	0.64991455	9.69E-23

PDHB	ZFP1	0.64941536	1.07E-22
PDHB	ABI2	0.6493374	1.09E-22
PDHB	SMAD2	0.64924096	1.11E-22
PDHB	CSTF2T	0.64901716	1.16E-22
PDHB	FBXO38	0.64897154	1.17E-22
PDHB	NPTN	0.64894911	1.17E-22
PDHB	OSBPL11	0.6489208	1.18E-22
PDHB	RAB1A	0.64891895	1.18E-22
PDHB	PDS5B	0.64875866	1.22E-22
PDHB	DCTD	0.64850336	1.28E-22
PDHB	R3HDM1	0.6483221	1.33E-22
PDHB	GPN3	0.64810136	1.39E-22
PDHB	SERINC1	0.64791791	1.44E-22
PDHB	RAD18	0.64782285	1.47E-22
PDHB	ATG7	0.64781309	1.47E-22
PDHB	PEX3	0.64767752	1.51E-22
PDHB	C1orf216	0.647199	1.66E-22
PDHB	TFDP1	0.64712305	1.69E-22
PDHB	HNRNPC	0.64709525	1.7E-22
PDHB	CNOT8	0.64706924	1.7E-22
PDHB	UGP2	0.64697403	1.74E-22
PDHB	ZFYVE1	0.64664421	1.85E-22
PDHB	PSMC2	0.64661116	1.87E-22
PDHB	NCOA4	0.64644259	1.93E-22
PDHB	RDH14	0.64626411	2E-22
PDHB	PSMC1	0.64603962	2.09E-22
PDHB	PSMD10	0.64550391	2.32E-22
PDHB	ETF1	0.64522306	2.45E-22
PDHB	CALM3	0.64504114	2.54E-22
PDHB	PITPNB	0.64500919	2.56E-22
PDHB	CTCF	0.64493555	2.59E-22
PDHB	TUBA1A	0.64481975	2.65E-22
PDHB	CDC42EP3	0.64481141	2.66E-22
PDHB	PPP1R12A	0.64463531	2.75E-22
PDHB	YME1L1	0.64461241	2.76E-22
PDHB	GABARAPL2	0.64444412	2.85E-22
PDHB	RAB23	0.64442501	2.87E-22
PDHB	MEF2A	0.64428778	2.94E-22
PDHB	RTF2	0.64349111	3.44E-22
PDHB	USP8	0.64341231	3.49E-22
PDHB	RNF20	0.64338885	3.51E-22
PDHB	SMU1	0.64307396	3.73E-22
PDHB	UBE2A	0.64306918	3.73E-22
PDHB	FKBP15	0.64295523	3.82E-22
PDHB	PLRG1	0.64253802	4.14E-22
PDHB	CALM2	0.64229308	4.34E-22
PDHB	LIX1L	0.64225168	4.37E-22
PDHB	SNX6	0.64193617	4.65E-22
PDHB	MRPL49	0.64185249	4.73E-22
PDHB	PPP3R1	0.64180781	4.77E-22
PDHB	IPO11	0.64179449	4.78E-22
PDHB	TEAD1	0.64166529	4.9E-22
PDHB	HPF1	0.6416328	4.93E-22
PDHB	PDE12	0.64162614	4.94E-22
PDHB	IMMT	0.64162191	4.94E-22
PDHB	DLAT	0.64137535	5.18E-22
PDHB	L3MBTL2	0.64131029	5.25E-22
PDHB	SEC23A	0.64115277	5.41E-22

PDHB	GHITM	0.64101953	5.55E-22
PDHB	LIN52	0.64097598	5.6E-22
PDHB	ISCA1	0.64082576	5.76E-22
PDHB	PITPNA	0.64076057	5.84E-22
PDHB	CLIP1	0.64059088	6.03E-22
PDHB	DCTN6	0.64051097	6.12E-22
PDHB	UBE2D3	0.64046396	6.18E-22
PDHB	BBS9	0.64022126	6.48E-22
PDHB	KBTBD4	0.6401203	6.6E-22
PDHB	SRSF3	0.64000209	6.76E-22
PDHB	NSMCE3	0.63996744	6.8E-22
PDHB	CCSER2	0.63984749	6.96E-22
PDHB	IK	0.63971788	7.13E-22
PDHB	ACAP2	0.63968483	7.18E-22
PDHB	TLK1	0.6396607	7.21E-22
PDHB	MBD5	0.63955679	7.36E-22
PDHB	PPFIA1	0.63939089	7.6E-22
PDHB	RBBP9	0.63928099	7.76E-22
PDHB	SLC25A17	0.63912588	7.99E-22
PDHB	PHF20	0.63903425	8.13E-22
PDHB	BMT2	0.63899201	8.2E-22
PDHB	DIPK1A	0.63890939	8.33E-22
PDHB	TRA2B	0.63882315	8.47E-22
PDHB	NDUFAF1	0.63871818	8.64E-22
PDHB	LYRM2	0.63871041	8.65E-22
PDHB	DHX30	0.638553	8.92E-22
PDHB	RFX5	0.6385228	8.97E-22
PDHB	USP4	0.6385215	8.97E-22
PDHB	MARCKS	0.63850789	9E-22
PDHB	SMARCA5	0.6385061	9E-22
PDHB	JAM3	0.63846834	9.07E-22
PDHB	ZFP91	0.63845994	9.08E-22
PDHB	PEX19	0.63809858	9.73E-22
PDHB	DLST	0.63796463	9.98E-22
PDHB	NEK1	0.63783057	1.02E-21
PDHB	ZC2HC1A	0.63782941	1.02E-21
PDHB	PHAX	0.63778767	1.03E-21
PDHB	UBA2	0.63771651	1.05E-21
PDHB	TCEAL4	0.63761677	1.07E-21
PDHB	C16orf70	0.63736018	1.12E-21
PDHB	GLG1	0.63727278	1.14E-21
PDHB	DFFA	0.63724374	1.15E-21
PDHB	AGGF1	0.63724143	1.15E-21
PDHB	TXNDC9	0.63721818	1.15E-21
PDHB	GFM1	0.63695391	1.21E-21
PDHB	SDHB	0.63690563	1.22E-21
PDHB	DKK3	0.63673941	1.26E-21
PDHB	SPOP	0.63673751	1.26E-21
PDHB	CUL4B	0.63656774	1.3E-21
PDHB	MAP7D1	0.63641201	1.34E-21
PDHB	FRG1	0.6363906	1.35E-21
PDHB	RAD1	0.63626628	1.38E-21
PDHB	PRXL2C	0.63623009	1.39E-21
PDHB	CIAPIN1	0.63616192	1.41E-21
PDHB	C1D	0.63609494	1.42E-21
PDHB	IFT57	0.6359672	1.46E-21
PDHB	RNF4	0.63585697	1.49E-21
PDHB	PTPN9	0.63584317	1.49E-21

PDHB	MAPK1IP1L	0.63563086	1.56E-21
PDHB	PCYOX1	0.63553289	1.58E-21
PDHB	RPAP3	0.63546406	1.61E-21
PDHB	UHRF1BP1L	0.63543787	1.61E-21
PDHB	EHD3	0.63543394	1.61E-21
PDHB	NDUFAF3	0.63541444	1.62E-21
PDHB	UBFD1	0.63531647	1.65E-21
PDHB	TRAK2	0.63529527	1.66E-21
PDHB	FEZ2	0.63524025	1.67E-21
PDHB	GPATCH11	0.63517723	1.69E-21
PDHB	PLEKHB2	0.63494915	1.77E-21
PDHB	SRP9	0.63491922	1.78E-21
PDHB	SAP130	0.63486352	1.8E-21
PDHB	CFAP20	0.63468459	1.86E-21
PDHB	PPIL4	0.63430615	2E-21
PDHB	PPP2R5E	0.63430019	2E-21
PDHB	ZC3H13	0.63421968	2.03E-21
PDHB	STAU2	0.6341371	2.06E-21
PDHB	PEF1	0.63405841	2.09E-21
PDHB	VPS29	0.63381381	2.19E-21
PDHB	RNF121	0.63372362	2.23E-21
PDHB	GNAQ	0.63369508	2.24E-21
PDHB	IWS1	0.63366761	2.25E-21
PDHB	EIF4E	0.6336456	2.26E-21
PDHB	RBM22	0.6336103	2.28E-21
PDHB	ARL13B	0.6335205	2.32E-21
PDHB	POLR2K	0.63338899	2.37E-21
PDHB	SETD7	0.63336203	2.39E-21
PDHB	TINF2	0.6332602	2.43E-21
PDHB	MTPN	0.63312419	2.49E-21
PDHB	CWC22	0.63298902	2.56E-21
PDHB	CLDND1	0.6328086	2.65E-21
PDHB	PDCL3	0.63268161	2.71E-21
PDHB	SET	0.63255724	2.77E-21
PDHB	ZFP90	0.63245685	2.83E-21
PDHB	MED17	0.63235884	2.88E-21
PDHB	ANO10	0.63234636	2.88E-21
PDHB	PSIP1	0.63232072	2.9E-21
PDHB	ATL3	0.63215688	2.99E-21
PDHB	XXYLT1	0.63205528	3.05E-21
PDHB	PEA15	0.6317268	3.24E-21
PDHB	HSDL1	0.63157118	3.33E-21
PDHB	SDHD	0.63150187	3.38E-21
PDHB	GNAI3	0.63114699	3.61E-21
PDHB	SCARB2	0.63112491	3.62E-21
PDHB	AP3B1	0.63103901	3.68E-21
PDHB	MUL1	0.63092642	3.76E-21
PDHB	GTF2B	0.63089309	3.78E-21
PDHB	EVI5	0.63066444	3.95E-21
PDHB	SVBP	0.63060082	3.99E-21
PDHB	PRPF4	0.6305961	4E-21
PDHB	PPP3CA	0.63049362	4.07E-21
PDHB	KIAA0586	0.63034802	4.19E-21
PDHB	MORF4L2	0.63022326	4.28E-21
PDHB	BMI1	0.63018929	4.31E-21
PDHB	MACF1	0.63009463	4.39E-21
PDHB	OXCT1	0.63002013	4.45E-21
PDHB	WASHC3	0.62982728	4.61E-21

PDHB	RC3H2	0.62982092	4.62E-21
PDHB	ELK3	0.62971898	4.7E-21
PDHB	RAB35	0.62961891	4.79E-21
PDHB	NAGA	0.62961807	4.79E-21
PDHB	ABRAXAS2	0.6295266	4.87E-21
PDHB	SPAST	0.62946558	4.93E-21
PDHB	PNO1	0.62937414	5.01E-21
PDHB	SYF2	0.62929345	5.09E-21
PDHB	ZFR	0.6291424	5.23E-21
PDHB	COMMD8	0.62911933	5.26E-21
PDHB	LEO1	0.62897004	5.4E-21
PDHB	TAF9	0.62892951	5.44E-21
PDHB	COPS2	0.62888663	5.49E-21
PDHB	PRMT2	0.62863222	5.75E-21
PDHB	C16orf72	0.62858391	5.8E-21
PDHB	AHCYL1	0.6285807	5.81E-21
PDHB	ACVR1	0.62856758	5.82E-21
PDHB	IQSEC1	0.62853698	5.85E-21
PDHB	ZC3H15	0.62832253	6.09E-21
PDHB	CLIC4	0.62810286	6.34E-21
PDHB	EMC7	0.62798338	6.48E-21
PDHB	KANK2	0.62794953	6.52E-21
PDHB	CTNNB1	0.62785236	6.64E-21
PDHB	SCO1	0.6278133	6.69E-21
PDHB	CUL2	0.62779814	6.71E-21
PDHB	VPS41	0.62774445	6.77E-21
PDHB	RBBP4	0.62774001	6.78E-21
PDHB	EXOSC9	0.62770679	6.82E-21
PDHB	ATF2	0.62769573	6.83E-21
PDHB	OCIAD1	0.62754769	7.02E-21
PDHB	MED28	0.62723208	7.44E-21
PDHB	C5orf15	0.62722517	7.45E-21
PDHB	DNAJB6	0.62712742	7.58E-21
PDHB	PUM2	0.62708718	7.64E-21
PDHB	MED6	0.62708323	7.65E-21
PDHB	TPP2	0.62704188	7.7E-21
PDHB	CCDC32	0.62698115	7.79E-21
PDHB	FAM204A	0.62671404	8.18E-21
PDHB	SRFBP1	0.6265228	8.47E-21
PDHB	KCTD20	0.62628774	8.85E-21
PDHB	ACVR2A	0.62619757	8.99E-21
PDHB	CDC5L	0.62616296	9.05E-21
PDHB	UBTD2	0.62603347	9.27E-21
PDHB	ANAPC13	0.62594957	9.41E-21
PDHB	TTC5	0.62591066	9.48E-21
PDHB	CNOT9	0.62581074	9.65E-21
PDHB	TPP1	0.62567298	9.9E-21
PDHB	ATP6V1D	0.62562552	9.98E-21
PDHB	C9orf64	0.62560892	1E-20
PDHB	AIDA	0.6256059	1E-20
PDHB	PPIG	0.62559131	1E-20
PDHB	ARHGAP1	0.62527009	1.07E-20
PDHB	PARN	0.62525176	1.07E-20
PDHB	SLC25A46	0.62506634	1.11E-20
PDHB	ZNF720	0.62498648	1.12E-20
PDHB	CC2D2A	0.62485946	1.15E-20
PDHB	CHMP5	0.62483497	1.15E-20
PDHB	TCEA1	0.62483395	1.15E-20

PDHB	KLHL7	0.6245276	1.22E-20
PDHB	NCDN	0.62439236	1.25E-20
PDHB	PGAM1	0.62425935	1.28E-20
PDHB	RAB12	0.62425041	1.28E-20
PDHB	PSPC1	0.62424681	1.28E-20
PDHB	GANAB	0.62420266	1.29E-20
PDHB	OSTM1	0.62409564	1.32E-20
PDHB	DIP2C	0.62395831	1.35E-20
PDHB	SMARCAL1	0.62394149	1.36E-20
PDHB	PHC2	0.62388082	1.37E-20
PDHB	ZNF350	0.62381142	1.39E-20
PDHB	TFE3	0.62379137	1.39E-20
PDHB	TSPYL1	0.62366842	1.43E-20
PDHB	CHCHD4	0.62365319	1.43E-20
PDHB	EFR3A	0.62355127	1.46E-20
PDHB	TCEAL8	0.62337006	1.5E-20
PDHB	PHF23	0.62331327	1.52E-20
PDHB	USP12	0.62327178	1.53E-20
PDHB	ACTN1	0.62320532	1.55E-20
PDHB	LRRC42	0.62319761	1.55E-20
PDHB	TRMT10C	0.62309531	1.58E-20
PDHB	RCBTB2	0.62300919	1.61E-20
PDHB	DHX9	0.62290426	1.64E-20
PDHB	PRDX3	0.62281866	1.66E-20
PDHB	CHD9	0.62278557	1.67E-20
PDHB	MAP3K2	0.62268182	1.7E-20
PDHB	ZCCHC9	0.62260442	1.73E-20
PDHB	GNG12	0.62253821	1.75E-20
PDHB	MYL6	0.62239083	1.8E-20
PDHB	PSMD6	0.62215384	1.87E-20
PDHB	MBNL1	0.62192375	1.95E-20
PDHB	ACTR10	0.62187054	1.97E-20
PDHB	FCF1	0.62175074	2.01E-20
PDHB	ZFAND3	0.6216684	2.04E-20
PDHB	PHACTR4	0.62159027	2.07E-20
PDHB	ISCU	0.62154876	2.09E-20
PDHB	PRTFDC1	0.62147459	2.12E-20
PDHB	CAND1	0.62138259	2.15E-20
PDHB	AP1S2	0.621355	2.16E-20
PDHB	RPL7L1	0.62130452	2.18E-20
PDHB	SORT1	0.62129453	2.19E-20
PDHB	ELP3	0.62126506	2.2E-20
PDHB	SERINC3	0.62096454	2.32E-20
PDHB	SNX12	0.62079585	2.39E-20
PDHB	MCMBP	0.62064283	2.46E-20
PDHB	MMGT1	0.62060919	2.47E-20
PDHB	RAB1B	0.62047122	2.54E-20
PDHB	NAV1	0.62027376	2.63E-20
PDHB	PANK2	0.62021336	2.65E-20
PDHB	TAB2	0.62019784	2.66E-20
PDHB	PCNX4	0.62018573	2.67E-20
PDHB	VPS33A	0.62003962	2.74E-20
PDHB	HIPK3	0.61985675	2.83E-20
PDHB	PICALM	0.61984645	2.83E-20
PDHB	PARK7	0.61973516	2.89E-20
PDHB	DCP2	0.61945419	3.04E-20
PDHB	TANK	0.61940512	3.07E-20
PDHB	LRCH3	0.61940358	3.07E-20

PDHB	KIFAP3	0.61935353	3.1E-20
PDHB	TRAPPC3	0.61934301	3.1E-20
PDHB	CRK	0.61933871	3.1E-20
PDHB	ZFAND5	0.61915301	3.21E-20
PDHB	RBM12	0.61906491	3.26E-20
PDHB	AKIP1	0.61903273	3.28E-20
PDHB	TMEM115	0.61892624	3.34E-20
PDHB	CAPRIN1	0.61892093	3.34E-20
PDHB	VPS35L	0.61870832	3.47E-20
PDHB	PRKAR1A	0.61865951	3.5E-20
PDHB	APBB2	0.61856935	3.56E-20
PDHB	VPS37A	0.61841124	3.66E-20
PDHB	GMFB	0.6182128	3.79E-20
PDHB	VDAC3	0.61820262	3.8E-20
PDHB	ITGB1	0.6181081	3.86E-20
PDHB	RRAGC	0.61793778	3.98E-20
PDHB	ADNP	0.61790829	4E-20
PDHB	SNX4	0.61782799	4.06E-20
PDHB	MBTPS2	0.61775675	4.11E-20
PDHB	CRTC3	0.6177256	4.14E-20
PDHB	RNF41	0.6176286	4.21E-20
PDHB	ABCF2	0.61751292	4.29E-20
PDHB	EXOC2	0.6174386	4.35E-20
PDHB	RRN3	0.61737579	4.4E-20
PDHB	PIGX	0.61737108	4.4E-20
PDHB	BBS2	0.61736926	4.41E-20
PDHB	MRPL33	0.61735418	4.42E-20
PDHB	ZNF174	0.61724201	4.51E-20
PDHB	HAUS1	0.61722225	4.52E-20
PDHB	DR1	0.61719067	4.55E-20
PDHB	CAPZA2	0.6171464	4.58E-20
PDHB	ATP6AP2	0.61705296	4.66E-20
PDHB	TMEM128	0.61700046	4.7E-20
PDHB	MOB4	0.6169017	4.79E-20
PDHB	PPP2CA	0.61688974	4.8E-20
PDHB	KDM4A	0.6167487	4.92E-20
PDHB	CTR9	0.61626546	5.35E-20
PDHB	RAD23B	0.61611289	5.5E-20
PDHB	GEMIN2	0.61611006	5.5E-20
PDHB	PIP4P2	0.61594439	5.67E-20
PDHB	NCKAP1	0.61587739	5.73E-20
PDHB	HNRNPUL2	0.61581022	5.8E-20
PDHB	ADAT1	0.61574776	5.87E-20
PDHB	THAP11	0.61570788	5.91E-20
PDHB	LACC1	0.6156788	5.94E-20
PDHB	PKD2	0.61562403	6E-20
PDHB	FAM220A	0.6154576	6.17E-20
PDHB	CYB5R3	0.61527348	6.38E-20
PDHB	PSMD2	0.6150843	6.59E-20
PDHB	BAG5	0.6150212	6.67E-20
PDHB	CDS2	0.61469493	7.06E-20
PDHB	CDYL	0.61463873	7.13E-20
PDHB	PRKAG2	0.61463294	7.14E-20
PDHB	OXSR1	0.61459994	7.18E-20
PDHB	DDB1	0.61459992	7.18E-20
PDHB	PCMT1	0.61458362	7.2E-20
PDHB	UVRAG	0.61456932	7.22E-20
PDHB	UBE2V2	0.61456285	7.22E-20

PDHB	RNF26	0.61450623	7.3E-20
PDHB	PTPN11	0.61448114	7.33E-20
PDHB	DDX6	0.61445149	7.37E-20
PDHB	SP3	0.61443055	7.39E-20
PDHB	USP10	0.61439294	7.44E-20
PDHB	NUDCD3	0.61436323	7.48E-20
PDHB	GTF2A2	0.61434173	7.51E-20
PDHB	C2CD3	0.61429707	7.57E-20
PDHB	NFE2L1	0.61418739	7.72E-20
PDHB	TWSG1	0.61418468	7.72E-20
PDHB	CPPED1	0.61417307	7.74E-20
PDHB	TBC1D5	0.61403838	7.92E-20
PDHB	ZNF362	0.6139947	7.98E-20
PDHB	DPYSL3	0.61395167	8.04E-20
PDHB	MTA2	0.6138273	8.22E-20
PDHB	NDRG3	0.61367807	8.44E-20
PDHB	HIPK1	0.6135441	8.64E-20
PDHB	CWF19L2	0.61343492	8.8E-20
PDHB	ZC4H2	0.61340532	8.85E-20
PDHB	SLC35A4	0.61338111	8.88E-20
PDHB	ITGA1	0.61327721	9.05E-20
PDHB	UBE3A	0.61311705	9.3E-20
PDHB	MICU2	0.61305571	9.4E-20
PDHB	ACOT2	0.61280514	9.82E-20
PDHB	EHBP1	0.61277704	9.87E-20
PDHB	TXNRD1	0.61275851	9.9E-20
PDHB	SMARCA2	0.61271481	9.98E-20
PDHB	SMARCC1	0.61239076	1.06E-19
PDHB	MINDY2	0.61230133	1.07E-19
PDHB	PTPRG	0.61226884	1.08E-19
PDHB	VTA1	0.61215109	1.1E-19
PDHB	TRIM32	0.61202495	1.13E-19
PDHB	SPPL3	0.61200418	1.13E-19
PDHB	ATMIN	0.61196997	1.14E-19
PDHB	SPATS2	0.61189941	1.15E-19
PDHB	HAUS2	0.61182994	1.16E-19
PDHB	NCS1	0.61176981	1.18E-19
PDHB	VEZF1	0.61176098	1.18E-19
PDHB	XRCC5	0.6116449	1.2E-19
PDHB	KPNA4	0.6115872	1.21E-19
PDHB	FGFR1OP2	0.61154822	1.22E-19
PDHB	SENP1	0.61149883	1.23E-19
PDHB	ASAH1	0.61144444	1.24E-19
PDHB	INTS12	0.61137312	1.26E-19
PDHB	COX7A2L	0.6112755	1.28E-19
PDHB	HSPB11	0.61120041	1.3E-19
PDHB	CPSF6	0.61118931	1.3E-19
PDHB	C14orf119	0.61114011	1.31E-19
PDHB	EXOC5	0.61109323	1.32E-19
PDHB	INTS5	0.61106091	1.33E-19
PDHB	LSM14A	0.61105647	1.33E-19
PDHB	PPM1B	0.61099815	1.34E-19
PDHB	RBX1	0.61099247	1.35E-19
PDHB	ZNF329	0.61094864	1.36E-19
PDHB	TRMT61B	0.61090156	1.37E-19
PDHB	GLOD4	0.61089008	1.37E-19
PDHB	DDX50	0.61060776	1.44E-19
PDHB	PHF13	0.61040285	1.49E-19

PDHB	RIC8A	0.61036012	1.5E-19
PDHB	JAK1	0.61022865	1.54E-19
PDHB	DLD	0.61016662	1.55E-19
PDHB	UEVLD	0.61009083	1.57E-19
PDHB	ZEB1	0.61001597	1.59E-19
PDHB	SBDS	0.60988373	1.63E-19
PDHB	ITM2B	0.60985598	1.64E-19
PDHB	HTRA2	0.60968567	1.69E-19
PDHB	IDH3A	0.60966597	1.69E-19
PDHB	ARCN1	0.60959039	1.71E-19
PDHB	SNRPB2	0.6095621	1.72E-19
PDHB	LRRFIP1	0.60951419	1.74E-19
PDHB	WDR20	0.60950326	1.74E-19
PDHB	LRRC8D	0.60945931	1.75E-19
PDHB	GDI2	0.60927274	1.81E-19
PDHB	ZNF260	0.60918319	1.84E-19
PDHB	MED20	0.60917346	1.84E-19
PDHB	PNRC2	0.60915807	1.85E-19
PDHB	GEMIN5	0.60877089	1.97E-19
PDHB	MAP3K7	0.60868314	2E-19
PDHB	C16orf87	0.60859156	2.04E-19
PDHB	CSRP1	0.60833224	2.13E-19
PDHB	RABL3	0.60832904	2.13E-19
PDHB	WASF2	0.6083168	2.13E-19
PDHB	COPB1	0.60827939	2.15E-19
PDHB	INIP	0.60809648	2.22E-19
PDHB	GAB2	0.60798909	2.26E-19
PDHB	SRF	0.60794859	2.27E-19
PDHB	BCAP29	0.60771076	2.37E-19
PDHB	SOCS5	0.60763828	2.4E-19
PDHB	GNA12	0.60759272	2.42E-19
PDHB	KIAA0232	0.60758836	2.42E-19
PDHB	SNX24	0.60748187	2.46E-19
PDHB	CYB5R4	0.60739504	2.5E-19
PDHB	HDAC2	0.60739138	2.5E-19
PDHB	G3BP2	0.60729675	2.54E-19
PDHB	AKIRIN2	0.60698935	2.68E-19
PDHB	DSTN	0.60684414	2.75E-19
PDHB	BICD2	0.60684113	2.75E-19
PDHB	SCP2	0.60683573	2.75E-19
PDHB	PPM1A	0.60681844	2.76E-19
PDHB	SKI	0.60676894	2.78E-19
PDHB	BCL2L13	0.60674871	2.79E-19
PDHB	RFC3	0.60668256	2.82E-19
PDHB	LSM3	0.6066048	2.86E-19
PDHB	SETX	0.60657642	2.87E-19
PDHB	RTCB	0.60653323	2.9E-19
PDHB	FKBP1A	0.60650094	2.91E-19
PDHB	UXS1	0.60637063	2.98E-19
PDHB	GPR107	0.60631653	3.01E-19
PDHB	FYTTD1	0.60631464	3.01E-19
PDHB	EFCAB14	0.60630604	3.01E-19
PDHB	UTP3	0.60626643	3.03E-19
PDHB	ZNF146	0.60623722	3.05E-19
PDHB	ARL5A	0.60621826	3.06E-19
PDHB	CLOCK	0.60619114	3.07E-19
PDHB	XRN2	0.60615399	3.09E-19
PDHB	ZFAND6	0.60584836	3.25E-19

PDHB	WWC3	0.60580581	3.28E-19
PDHB	CHURC1	0.60579967	3.28E-19
PDHB	FERMT2	0.60579437	3.28E-19
PDHB	UBQLN1	0.60550111	3.45E-19
PDHB	CEP41	0.6054382	3.49E-19
PDHB	TAOK1	0.60524434	3.61E-19
PDHB	BECN1	0.60520967	3.63E-19
PDHB	TMEM127	0.605159	3.66E-19
PDHB	TBK1	0.60504234	3.73E-19
PDHB	ABR	0.60499588	3.76E-19
PDHB	BBX	0.60486722	3.84E-19
PDHB	WDFY1	0.60483964	3.86E-19
PDHB	TMEM35B	0.60479063	3.89E-19
PDHB	ZNF143	0.60477833	3.9E-19
PDHB	BRD3	0.60477628	3.9E-19
PDHB	RAP2A	0.6047456	3.92E-19
PDHB	ABHD13	0.60467108	3.97E-19
PDHB	YTHDF3	0.60466338	3.98E-19
PDHB	CALD1	0.60456672	4.04E-19
PDHB	RB1	0.60456275	4.05E-19
PDHB	FAM20B	0.60450652	4.09E-19
PDHB	HNRNPUL1	0.60442165	4.15E-19
PDHB	TCAF1	0.60426846	4.25E-19
PDHB	MAPK14	0.60419722	4.31E-19
PDHB	TRIP12	0.60416986	4.33E-19
PDHB	AGPS	0.60408668	4.39E-19
PDHB	SMC6	0.60408554	4.39E-19
PDHB	XRCC6	0.60404837	4.42E-19
PDHB	CFAP97	0.60401901	4.44E-19
PDHB	ATP6V0E1	0.60389619	4.53E-19
PDHB	SFT2D2	0.60379559	4.61E-19
PDHB	MTERF3	0.60376894	4.63E-19
PDHB	CNOT10	0.60375889	4.64E-19
PDHB	NSRP1	0.60361309	4.75E-19
PDHB	ARPC5	0.60343271	4.9E-19
PDHB	SUMO3	0.60336837	4.95E-19
PDHB	PARG	0.60320389	5.09E-19
PDHB	VHL	0.6029844	5.28E-19
PDHB	CLP1	0.60290107	5.36E-19
PDHB	PAK2	0.60289685	5.36E-19
PDHB	RLIM	0.60281588	5.44E-19
PDHB	SMAD7	0.60272072	5.52E-19
PDHB	NDEL1	0.60267264	5.57E-19
PDHB	MRPL35	0.60265066	5.59E-19
PDHB	PELO	0.60251981	5.71E-19
PDHB	TMEM167A	0.60243549	5.79E-19
PDHB	SGCD	0.60234229	5.89E-19
PDHB	PDS5A	0.60229299	5.94E-19
PDHB	CNST	0.60217539	6.05E-19
PDHB	GRB2	0.60203003	6.2E-19
PDHB	UBE2E2	0.60199944	6.23E-19
PDHB	PIK3C3	0.60193916	6.3E-19
PDHB	RPGRIP1L	0.60188127	6.36E-19
PDHB	BAZ1B	0.60180084	6.45E-19
PDHB	KDM2A	0.60165482	6.61E-19
PDHB	LDB1	0.60163164	6.63E-19
PDHB	POLR2D	0.60162448	6.64E-19
PDHB	ZMAT3	0.60162087	6.64E-19

PDHB	AP4S1	0.60155696	6.72E-19
PDHB	RCN2	0.60154538	6.73E-19
PDHB	UBXN2B	0.60153418	6.74E-19
PDHB	CLN5	0.60148855	6.79E-19
PDHB	ZNF45	0.60148168	6.8E-19
PDHB	NIN	0.6014423	6.85E-19
PDHB	AKT3	0.60132697	6.98E-19
PDHB	SLC30A9	0.60112457	7.22E-19
PDHB	ZKSCAN5	0.60104026	7.32E-19
PDHB	DRAM2	0.60096435	7.42E-19
PDHB	GTF3C4	0.60081842	7.6E-19
PDHB	DCUN1D1	0.60079251	7.63E-19
PDHB	SYT11	0.60074342	7.69E-19
PDHB	PSME3	0.60067809	7.78E-19
PDHB	TXNDC15	0.60057726	7.91E-19
PDHB	GTF2A1	0.60056077	7.93E-19
PDHB	CPNE3	0.60048621	8.03E-19
PDHB	PLAA	0.60042467	8.11E-19
PDHB	SUPT16H	0.60019972	8.42E-19
PDHB	PI4K2A	0.60012228	8.53E-19
PDHB	MAPRE2	0.60001659	8.69E-19
PDHB	ST13	0.59997187	8.75E-19
PDHB	PEX2	0.59995802	8.77E-19
PDHB	MAP2K1	0.59985654	8.92E-19
PDHB	SNRNP40	0.59983248	8.96E-19
PDHB	PURB	0.59977444	9.04E-19
PDHB	PPM1M	0.59973045	9.11E-19
PDHB	SUSD1	0.59966274	9.21E-19
PDHB	ENOX2	0.59956495	9.36E-19
PDHB	JKAMP	0.59946611	9.52E-19
PDHB	IST1	0.59942167	9.59E-19
PDHB	MED27	0.59920472	9.94E-19
PDHB	PPP2CB	0.59915874	1E-18
PDHB	SERF1B	0.59906413	1.02E-18
PDHB	TMEM237	0.59901747	1.03E-18
PDHB	KIF3C	0.59901072	1.03E-18
PDHB	SMARCE1	0.59897526	1.03E-18
PDHB	DNAJC14	0.59890629	1.04E-18
PDHB	HNRNPU	0.59889016	1.05E-18
PDHB	EXOC6B	0.59873607	1.07E-18
PDHB	AIMP1	0.59872829	1.08E-18
PDHB	TAF12	0.59859962	1.1E-18
PDHB	CIPC	0.59857329	1.1E-18
PDHB	PCYT1A	0.59850185	1.12E-18
PDHB	MRPS28	0.598473	1.12E-18
PDHB	SYNCRIP	0.59845756	1.13E-18
PDHB	NDUFS1	0.59838694	1.14E-18
PDHB	ACTR2	0.59834641	1.15E-18
PDHB	INO80	0.59829056	1.16E-18
PDHB	RNF139	0.59827087	1.16E-18
PDHB	KDSR	0.5982074	1.17E-18
PDHB	ITFG1	0.59813956	1.19E-18
PDHB	ESF1	0.59809097	1.2E-18
PDHB	BABAM2	0.59801309	1.21E-18
PDHB	CNP	0.59793512	1.23E-18
PDHB	G3BP1	0.59791238	1.23E-18
PDHB	KIAA1191	0.59785486	1.24E-18
PDHB	HNRNPA0	0.5978492	1.24E-18

PDHB	RBM18	0.59783596	1.25E-18
PDHB	AKIRIN1	0.59776003	1.26E-18
PDHB	YWHAH	0.59768376	1.28E-18
PDHB	SNX18	0.59763935	1.29E-18
PDHB	SUMF1	0.59755421	1.31E-18
PDHB	NAP1L1	0.59754887	1.31E-18
PDHB	CSNK1G3	0.59733697	1.35E-18
PDHB	NPAT	0.59732771	1.36E-18
PDHB	SRSF1	0.59727994	1.37E-18
PDHB	NFE2L2	0.59727529	1.37E-18
PDHB	OAT	0.59718055	1.39E-18
PDHB	PPP2R3C	0.59697222	1.44E-18
PDHB	TRUB1	0.59691894	1.45E-18
PDHB	GGPS1	0.59661134	1.53E-18
PDHB	PNMA1	0.59653119	1.55E-18
PDHB	ATP6V1B2	0.59650955	1.55E-18
PDHB	FNTB	0.59648069	1.56E-18
PDHB	SRPK2	0.59630575	1.61E-18
PDHB	CKAP5	0.59630432	1.61E-18
PDHB	GNPTAB	0.59620705	1.63E-18
PDHB	B3GNT9	0.59608788	1.66E-18
PDHB	MED8	0.59608425	1.67E-18
PDHB	NIF3L1	0.59589077	1.72E-18
PDHB	UBE3C	0.59576843	1.75E-18
PDHB	TOPORS	0.59575679	1.76E-18
PDHB	OSTF1	0.5953858	1.87E-18
PDHB	RAB10	0.59511116	1.95E-18
PDHB	DPYSL2	0.59509479	1.96E-18
PDHB	CAMSAP1	0.59508729	1.96E-18
PDHB	PRPF18	0.59504218	1.98E-18
PDHB	CLTC	0.59503096	1.98E-18
PDHB	NUDT3	0.59489077	2.03E-18
PDHB	PLSCR4	0.59487538	2.03E-18
PDHB	ATP6V1C1	0.59486182	2.03E-18
PDHB	FASTKD2	0.59456651	2.14E-18
PDHB	COA7	0.59451534	2.15E-18
PDHB	TAF1B	0.59441399	2.19E-18
PDHB	DCK	0.59425727	2.25E-18
PDHB	NTAN1	0.59422695	2.26E-18
PDHB	QTRT2	0.59400953	2.34E-18
PDHB	RRM2B	0.59363337	2.49E-18
PDHB	NAA30	0.59336787	2.6E-18
PDHB	TIMMDC1	0.59327536	2.64E-18
PDHB	WDR44	0.59319313	2.67E-18
PDHB	GLRX5	0.59315291	2.69E-18
PDHB	PLEKHM2	0.59311062	2.71E-18
PDHB	CDK17	0.59310613	2.71E-18
PDHB	SMG6	0.59306477	2.73E-18
PDHB	RNF111	0.59287961	2.81E-18
PDHB	ATP2B4	0.59277871	2.86E-18
PDHB	ATXN3	0.5927017	2.89E-18
PDHB	TSNAX	0.59268199	2.9E-18
PDHB	ATP2A2	0.59266228	2.91E-18
PDHB	LAMTOR3	0.59265252	2.92E-18
PDHB	GPBP1L1	0.59248758	3E-18
PDHB	STAT3	0.59238728	3.05E-18
PDHB	TSHZ3	0.59229585	3.09E-18
PDHB	AK6	0.5922947	3.09E-18

PDHB	CCDC115	0.59222146	3.13E-18
PDHB	MAT2B	0.59218948	3.14E-18
PDHB	SELENON	0.59207249	3.2E-18
PDHB	IRF2	0.5918739	3.31E-18
PDHB	CHM	0.59187185	3.31E-18
PDHB	SUSD6	0.59183165	3.33E-18
PDHB	CRIPT	0.59182787	3.33E-18
PDHB	GLYR1	0.59178914	3.36E-18
PDHB	SNTB2	0.59178867	3.36E-18
PDHB	ANKS1A	0.59178046	3.36E-18
PDHB	CNIH1	0.59173562	3.38E-18
PDHB	POFUT1	0.59172366	3.39E-18
PDHB	PRSS23	0.59169545	3.41E-18
PDHB	KBTBD2	0.59165953	3.43E-18
PDHB	HBP1	0.59163205	3.44E-18
PDHB	SOCS4	0.5915551	3.49E-18
PDHB	BPGM	0.59141532	3.56E-18
PDHB	PTPN1	0.5913185	3.62E-18
PDHB	DLG1	0.59130571	3.63E-18
PDHB	PDLIM5	0.59124413	3.66E-18
PDHB	CSGALNACT2	0.59121268	3.68E-18
PDHB	MRAS	0.59118598	3.7E-18
PDHB	ARMT1	0.59113191	3.73E-18
PDHB	FOXP1	0.59111879	3.74E-18
PDHB	ASAP1	0.59110379	3.75E-18
PDHB	FAM118B	0.59104796	3.78E-18
PDHB	WSB2	0.59095758	3.84E-18
PDHB	RPRD1B	0.5908556	3.9E-18
PDHB	CHST14	0.59083523	3.91E-18
PDHB	MRPL3	0.59081495	3.93E-18
PDHB	AKAP11	0.59081293	3.93E-18
PDHB	SEC13	0.59067553	4.02E-18
PDHB	ZNF227	0.59055569	4.1E-18
PDHB	RAB21	0.59053619	4.11E-18
PDHB	TGFBR1	0.59051919	4.12E-18
PDHB	CAP1	0.59042396	4.18E-18
PDHB	PINK1	0.59037437	4.22E-18
PDHB	RNF145	0.59033712	4.24E-18
PDHB	NBN	0.5902774	4.28E-18
PDHB	NKIRAS2	0.59018514	4.35E-18
PDHB	WDR61	0.59014566	4.38E-18
PDHB	ZNF134	0.59013639	4.38E-18
PDHB	DNAJC13	0.59013379	4.38E-18
PDHB	SMC3	0.59011343	4.4E-18
PDHB	KTN1	0.59009239	4.41E-18
PDHB	CREB3L2	0.59006538	4.43E-18
PDHB	KCTD21	0.59003581	4.45E-18
PDHB	MYO5A	0.59000657	4.47E-18
PDHB	ZNF250	0.5899307	4.53E-18
PDHB	CNN3	0.5898993	4.55E-18
PDHB	NECTIN3	0.58985688	4.58E-18
PDHB	NFU1	0.58981796	4.61E-18
PDHB	EIF1AD	0.58978697	4.64E-18
PDHB	POLR2C	0.58976416	4.65E-18
PDHB	TAF5L	0.58968295	4.71E-18
PDHB	ZBTB6	0.58964969	4.74E-18
PDHB	BTBD10	0.58958952	4.78E-18
PDHB	NID1	0.58958846	4.79E-18

PDHB	SUMO2	0.58952161	4.84E-18
PDHB	HMGXB4	0.58931999	5E-18
PDHB	RBM43	0.58921542	5.08E-18
PDHB	ARHGEF25	0.58912587	5.15E-18
PDHB	DDX3X	0.58902493	5.24E-18
PDHB	FCHSD2	0.58902368	5.24E-18
PDHB	TMEM19	0.58902166	5.24E-18
PDHB	GLO1	0.58893347	5.32E-18
PDHB	DYRK1A	0.58881347	5.42E-18
PDHB	CTSO	0.58880995	5.42E-18
PDHB	PDHX	0.5888098	5.42E-18
PDHB	FBXO45	0.58880761	5.42E-18
PDHB	CBX5	0.58874955	5.48E-18
PDHB	METTL14	0.58873136	5.49E-18
PDHB	MSRB3	0.58867886	5.54E-18
PDHB	C4orf3	0.5886425	5.57E-18
PDHB	YWHAG	0.58861751	5.59E-18
PDHB	LTBP1	0.58849819	5.7E-18
PDHB	COQ10B	0.58849039	5.71E-18
PDHB	MTFMT	0.58841913	5.77E-18
PDHB	ELOVL5	0.58828458	5.9E-18
PDHB	SAP30	0.58825903	5.92E-18
PDHB	SLU7	0.58819628	5.98E-18
PDHB	PDE4D	0.58811151	6.06E-18
PDHB	FKBP7	0.58807668	6.1E-18
PDHB	VBP1	0.5879805	6.19E-18
PDHB	PSMD14	0.58796149	6.21E-18
PDHB	DNAJB5	0.58787025	6.3E-18
PDHB	DENR	0.58773245	6.44E-18
PDHB	MRPL16	0.58770046	6.48E-18
PDHB	USP34	0.58763756	6.54E-18
PDHB	NDUFB5	0.58758802	6.59E-18
PDHB	POLE3	0.58748233	6.7E-18
PDHB	TRUB2	0.58747098	6.72E-18
PDHB	VCL	0.58743796	6.75E-18
PDHB	ATXN1	0.58743025	6.76E-18
PDHB	EPB41L2	0.58731276	6.89E-18
PDHB	ANP32E	0.58724841	6.96E-18
PDHB	RBMS1	0.58722647	6.98E-18
PDHB	COMMD2	0.58709367	7.13E-18
PDHB	RNF168	0.58708839	7.14E-18
PDHB	CCDC92	0.58704568	7.19E-18
PDHB	MAX	0.58701686	7.22E-18
PDHB	RCC1L	0.58694997	7.3E-18
PDHB	BTBD9	0.58680327	7.47E-18
PDHB	SNAP47	0.58679059	7.49E-18
PDHB	POLDIP3	0.58678207	7.5E-18
PDHB	RSRC1	0.58675064	7.53E-18
PDHB	VPS35	0.58670155	7.59E-18
PDHB	CDC27	0.5864199	7.94E-18
PDHB	CYLD	0.58632627	8.06E-18
PDHB	GMEB1	0.58627406	8.13E-18
PDHB	CRTAP	0.5862279	8.19E-18
PDHB	YIPF5	0.58605414	8.42E-18
PDHB	CCNI	0.58605384	8.42E-18
PDHB	ADO	0.58604791	8.42E-18
PDHB	SOS2	0.58604277	8.43E-18
PDHB	ITGB1BP1	0.58585383	8.69E-18

PDHB	RNF8	0.58579393	8.77E-18
PDHB	FAM32A	0.58571498	8.88E-18
PDHB	ATP2C1	0.58570874	8.89E-18
PDHB	WBP1L	0.58568102	8.93E-18
PDHB	WAC	0.58556229	9.1E-18
PDHB	DAZAP2	0.58550866	9.18E-18
PDHB	HADHA	0.58549187	9.2E-18
PDHB	FUCA1	0.58546571	9.24E-18
PDHB	TM2D3	0.58542889	9.29E-18
PDHB	RAB8B	0.58530837	9.47E-18
PDHB	SAE1	0.58524042	9.57E-18
PDHB	TSHZ1	0.58503829	9.89E-18
PDHB	UPRT	0.5850335	9.89E-18
PDHB	KAT7	0.58492308	1.01E-17
PDHB	TRAF3IP1	0.58489615	1.01E-17
PDHB	COQ7	0.58481139	1.02E-17
PDHB	SPTLC1	0.5847071	1.04E-17
PDHB	PIP4K2B	0.58423101	1.12E-17
PDHB	NRBF2	0.58421552	1.13E-17
PDHB	IFNAR1	0.58416828	1.13E-17
PDHB	CORO1C	0.58411479	1.14E-17
PDHB	GASK1B	0.58409905	1.15E-17
PDHB	FAF2	0.58405034	1.16E-17
PDHB	UBE2W	0.58403863	1.16E-17
PDHB	KPNA1	0.58392364	1.18E-17
PDHB	MB21D2	0.58384152	1.19E-17
PDHB	NUP62	0.58378352	1.21E-17
PDHB	GLE1	0.58371094	1.22E-17
PDHB	MSH2	0.58369149	1.22E-17
PDHB	PDCD10	0.58355041	1.25E-17
PDHB	CBFB	0.5835036	1.26E-17
PDHB	STON1	0.58345635	1.27E-17
PDHB	ZNF207	0.58325263	1.31E-17
PDHB	FARS2	0.58312062	1.34E-17
PDHB	EXOC1	0.58307916	1.35E-17
PDHB	CBL	0.58307666	1.35E-17
PDHB	MYH10	0.58301577	1.36E-17
PDHB	DYNLL1	0.58293201	1.38E-17
PDHB	RBM7	0.58283785	1.4E-17
PDHB	OSBPL8	0.5828376	1.4E-17
PDHB	ZMPSTE24	0.5827924	1.41E-17
PDHB	CYTH3	0.58277846	1.41E-17
PDHB	ASXL2	0.58273117	1.42E-17
PDHB	PGRMC1	0.58262887	1.45E-17
PDHB	TMED5	0.58249153	1.48E-17
PDHB	CCNY	0.58241495	1.49E-17
PDHB	SUMO1	0.58239924	1.5E-17
PDHB	GON7	0.58231593	1.52E-17
PDHB	PLBD2	0.58229643	1.52E-17
PDHB	TRIM69	0.58227216	1.53E-17
PDHB	ZNF148	0.58224945	1.53E-17
PDHB	NREP	0.58217648	1.55E-17
PDHB	RAD21	0.58212456	1.56E-17
PDHB	UQCRC1	0.58204233	1.58E-17
PDHB	NEDD1	0.58200454	1.59E-17
PDHB	NAA15	0.58199997	1.59E-17
PDHB	NAA50	0.5819228	1.61E-17
PDHB	VAPA	0.58178375	1.65E-17

PDHB	USP38	0.58177924	1.65E-17
PDHB	SIAH1	0.58166443	1.68E-17
PDHB	DIS3	0.5816497	1.68E-17
PDHB	JHY	0.58150341	1.72E-17
PDHB	AKT1	0.58140831	1.75E-17
PDHB	AP1B1	0.58127365	1.79E-17
PDHB	MRPL44	0.58110006	1.84E-17
PDHB	POLR3GL	0.58107129	1.84E-17
PDHB	GRAMD2B	0.58094538	1.88E-17
PDHB	LAMTOR5	0.58093253	1.88E-17
PDHB	RNGTT	0.58080797	1.92E-17
PDHB	TCAIM	0.58079043	1.93E-17
PDHB	CD2BP2	0.58066937	1.96E-17
PDHB	ITSN1	0.58060501	1.98E-17
PDHB	SLAIN2	0.58058582	1.99E-17
PDHB	COPS5	0.58057425	1.99E-17
PDHB	SPDL1	0.58054425	2E-17
PDHB	SNX7	0.58044536	2.03E-17
PDHB	MYD88	0.5803805	2.05E-17
PDHB	CTTNBP2NL	0.58035144	2.06E-17
PDHB	NEK7	0.58033664	2.07E-17
PDHB	TGFBR2	0.5802461	2.1E-17
PDHB	ATG5	0.58023467	2.1E-17
PDHB	DCHS1	0.58015843	2.13E-17
PDHB	CHSY3	0.58010342	2.14E-17
PDHB	NSFL1C	0.57999922	2.18E-17
PDHB	FIG4	0.57993674	2.2E-17
PDHB	SMC2	0.57990073	2.21E-17
PDHB	MRPL50	0.57987353	2.22E-17
PDHB	SOGA1	0.57980988	2.24E-17
PDHB	TDRD7	0.57974421	2.27E-17
PDHB	WDR26	0.57971729	2.28E-17
PDHB	IAH1	0.57968366	2.29E-17
PDHB	MAGED2	0.57959542	2.32E-17
PDHB	LUZP1	0.57933272	2.42E-17
PDHB	SRGAP2C	0.57929907	2.43E-17
PDHB	APEX1	0.57923068	2.46E-17
PDHB	VPS4B	0.57917685	2.48E-17
PDHB	PIP4K2A	0.57913414	2.49E-17
PDHB	DAAM1	0.57911797	2.5E-17
PDHB	SDCBP	0.57909921	2.51E-17
PDHB	HPS5	0.57905688	2.52E-17
PDHB	MRPL30	0.57901086	2.54E-17
PDHB	SNX9	0.57898384	2.55E-17
PDHB	PBX3	0.57887569	2.59E-17
PDHB	PMS2	0.57882809	2.61E-17
PDHB	GORASP2	0.57882663	2.61E-17
PDHB	STARD7	0.57880928	2.62E-17
PDHB	LAMTOR1	0.57879777	2.63E-17
PDHB	ZNF226	0.57873943	2.65E-17
PDHB	TGOLN2	0.57854347	2.73E-17
PDHB	JAZF1	0.57840501	2.79E-17
PDHB	ASH2L	0.57837946	2.8E-17
PDHB	SESTD1	0.57824405	2.86E-17
PDHB	AP1M1	0.57823979	2.86E-17
PDHB	AHNAK	0.57818918	2.88E-17
PDHB	PDCD6IP	0.5781334	2.91E-17
PDHB	USP14	0.57810373	2.92E-17

PDHB	KCTD2	0.57806331	2.94E-17
PDHB	RAB2A	0.57796133	2.99E-17
PDHB	PTDSS1	0.57780324	3.06E-17
PDHB	AP5M1	0.57777099	3.08E-17
PDHB	CTDNEP1	0.57773638	3.09E-17
PDHB	FKBP9	0.57771586	3.1E-17
PDHB	ELK1	0.57771049	3.11E-17
PDHB	RECQL	0.57766852	3.13E-17
PDHB	ASB7	0.57765164	3.13E-17
PDHB	MAN2B2	0.5776015	3.16E-17
PDHB	PITHD1	0.57752633	3.2E-17
PDHB	CROT	0.57751225	3.2E-17
PDHB	MPHOSPH6	0.57750516	3.21E-17
PDHB	TGS1	0.5774086	3.25E-17
PDHB	USP9X	0.57738024	3.27E-17
PDHB	AP2B1	0.57733555	3.29E-17
PDHB	FSTL1	0.57732197	3.3E-17
PDHB	GOT2	0.57730601	3.31E-17
PDHB	LMAN2L	0.57718007	3.37E-17
PDHB	PNPLA8	0.57716396	3.38E-17
PDHB	RNF123	0.57710165	3.41E-17
PDHB	MFSD11	0.57706053	3.43E-17
PDHB	FBXL17	0.57705666	3.44E-17
PDHB	ECHDC1	0.57703294	3.45E-17
PDHB	KLHL20	0.57692423	3.51E-17
PDHB	GOLIM4	0.5767486	3.6E-17
PDHB	CEP170	0.57665477	3.65E-17
PDHB	CHUK	0.57659983	3.69E-17
PDHB	RUFY1	0.576541	3.72E-17
PDHB	MYLK	0.57649281	3.75E-17
PDHB	RNF34	0.57639949	3.8E-17
PDHB	UBXN2A	0.57635107	3.83E-17
PDHB	LRP12	0.57634656	3.83E-17
PDHB	EDC3	0.57634105	3.84E-17
PDHB	ABI1	0.57633347	3.84E-17
PDHB	NAP1L4	0.57632461	3.84E-17
PDHB	TNPO1	0.57632434	3.84E-17
PDHB	PGD	0.57622378	3.9E-17
PDHB	NR2F2	0.57609767	3.98E-17
PDHB	CAMSAP2	0.57600138	4.04E-17
PDHB	TCF12	0.57600034	4.04E-17
PDHB	HNRNPD	0.57598459	4.05E-17
PDHB	ATXN10	0.57595251	4.07E-17
PDHB	SDHC	0.57582407	4.15E-17
PDHB	TMED7	0.575779	4.18E-17
PDHB	GTF2H3	0.57575424	4.2E-17
PDHB	SNAPC5	0.57562488	4.28E-17
PDHB	YWHAE	0.57555474	4.33E-17
PDHB	ATP10D	0.57542793	4.41E-17
PDHB	SSRP1	0.57542237	4.42E-17
PDHB	DDX18	0.57540778	4.43E-17
PDHB	RMDN2	0.57525984	4.53E-17
PDHB	DDX19B	0.57522155	4.55E-17
PDHB	ZNF827	0.57517996	4.58E-17
PDHB	OXNAD1	0.57496613	4.73E-17
PDHB	BORCS7	0.57496341	4.74E-17
PDHB	SNRNP200	0.57493774	4.75E-17
PDHB	LNPK	0.57482482	4.84E-17

PDHB	SAMD8	0.57468146	4.94E-17
PDHB	RAP1B	0.57465502	4.96E-17
PDHB	RP2	0.57444343	5.13E-17
PDHB	TTC26	0.57409631	5.41E-17
PDHB	APEH	0.57396263	5.52E-17
PDHB	PAFAH1B1	0.5736871	5.75E-17
PDHB	XRCC1	0.57368636	5.75E-17
PDHB	PCNX1	0.57360262	5.83E-17
PDHB	YIPF1	0.57337629	6.03E-17
PDHB	NDUFS4	0.57325065	6.15E-17
PDHB	RTN3	0.57324364	6.16E-17
PDHB	TCFL5	0.57324021	6.16E-17
PDHB	CHCHD3	0.57321121	6.19E-17
PDHB	KATNA1	0.57319093	6.21E-17
PDHB	TRIM13	0.57316278	6.23E-17
PDHB	SIN3A	0.57310347	6.29E-17
PDHB	CALCOCO2	0.5730695	6.32E-17
PDHB	MBNL2	0.57301178	6.38E-17
PDHB	PEAK1	0.57298162	6.41E-17
PDHB	IFIT5	0.572505	6.89E-17
PDHB	DESI2	0.57236649	7.03E-17
PDHB	DPY19L4	0.57225014	7.16E-17
PDHB	SRGAP2B	0.57220929	7.2E-17
PDHB	PRKAG1	0.57211125	7.31E-17
PDHB	ADAR	0.57207241	7.35E-17
PDHB	ARPC2	0.57203478	7.39E-17
PDHB	MOB1A	0.57186108	7.59E-17
PDHB	NRAS	0.57181927	7.64E-17
PDHB	NUP133	0.57178679	7.68E-17
PDHB	SMAD5	0.57174578	7.72E-17
PDHB	GPALPP1	0.57167119	7.81E-17
PDHB	PAIP1	0.57148194	8.04E-17
PDHB	MRPS27	0.57144595	8.08E-17
PDHB	SNX11	0.57142399	8.11E-17
PDHB	UBA1	0.5713562	8.19E-17
PDHB	RECK	0.57126861	8.3E-17
PDHB	TRIO	0.57124655	8.33E-17
PDHB	CRKL	0.57117707	8.42E-17
PDHB	CPSF2	0.57112366	8.49E-17
PDHB	ARL15	0.57108563	8.53E-17
PDHB	EXTL3	0.57108422	8.54E-17
PDHB	RHBDD1	0.57107633	8.55E-17
PDHB	PEPD	0.57106351	8.56E-17
PDHB	XYLT1	0.57103778	8.6E-17
PDHB	ARHGAP17	0.57086992	8.82E-17
PDHB	WASHC5	0.57075162	8.97E-17
PDHB	WASL	0.57074143	8.99E-17
PDHB	CNOT4	0.57072668	9.01E-17
PDHB	VTI1A	0.57071169	9.03E-17
PDHB	ARL1	0.57066557	9.09E-17
PDHB	DDR2	0.57065862	9.1E-17
PDHB	OPA3	0.57060359	9.18E-17
PDHB	TSFM	0.57052649	9.28E-17
PDHB	ENTPD1	0.57048971	9.34E-17
PDHB	SNAPIN	0.57041467	9.44E-17
PDHB	SCAMP1	0.57028428	9.63E-17
PDHB	SLC38A6	0.57019218	9.76E-17
PDHB	CMTM6	0.57015906	9.81E-17

PDHB	EMC1	0.57013483	9.85E-17
PDHB	POLR2G	0.57006516	9.95E-17
PDHB	PKNOX1	0.56998556	1.01E-16
PDHB	TERF2IP	0.56987241	1.02E-16
PDHB	ANKMY2	0.56980633	1.03E-16
PDHB	SUFU	0.56978372	1.04E-16
PDHB	HNRNPM	0.56977493	1.04E-16
PDHB	TOX4	0.56977396	1.04E-16
PDHB	CHMP2B	0.56976518	1.04E-16
PDHB	CNOT6	0.56976116	1.04E-16
PDHB	NR3C1	0.56963378	1.06E-16
PDHB	GAS7	0.56927057	1.12E-16
PDHB	ZBTB38	0.56924095	1.13E-16
PDHB	RASGEF1B	0.56919067	1.13E-16
PDHB	CYP2U1	0.56914043	1.14E-16
PDHB	SKA2	0.56912124	1.15E-16
PDHB	MED1	0.56907709	1.15E-16
PDHB	PIP5K1A	0.56875201	1.21E-16
PDHB	C8orf88	0.56873692	1.21E-16
PDHB	PSAP	0.56868059	1.22E-16
PDHB	ROCK1	0.5685592	1.25E-16
PDHB	STPG1	0.56854223	1.25E-16
PDHB	ZNF562	0.56844602	1.27E-16
PDHB	RBM27	0.56843764	1.27E-16
PDHB	AFF4	0.56840337	1.28E-16
PDHB	HSPA13	0.56834787	1.29E-16
PDHB	MOAP1	0.56834597	1.29E-16
PDHB	ZMIZ1	0.56828569	1.3E-16
PDHB	IDS	0.56826795	1.3E-16
PDHB	ZCCHC10	0.56825853	1.3E-16
PDHB	ARIH1	0.56825232	1.31E-16
PDHB	TFCP2	0.5681958	1.32E-16
PDHB	POLR3H	0.56800605	1.35E-16
PDHB	PPP5C	0.56798206	1.36E-16
PDHB	FOXN3	0.56776679	1.4E-16
PDHB	SMARCB1	0.56774288	1.41E-16
PDHB	SORBS3	0.5676815	1.42E-16
PDHB	HUS1	0.56768105	1.42E-16
PDHB	BAG2	0.56760957	1.44E-16
PDHB	VAPB	0.56760704	1.44E-16
PDHB	RNF6	0.56726618	1.51E-16
PDHB	EVI5L	0.56721003	1.53E-16
PDHB	FYN	0.56714307	1.54E-16
PDHB	SYNJ2BP	0.56705673	1.56E-16
PDHB	SLC30A5	0.56703956	1.56E-16
PDHB	KRCC1	0.56703583	1.57E-16
PDHB	BBS7	0.56696617	1.58E-16
PDHB	ATP6V1G1	0.56696338	1.58E-16
PDHB	TSG101	0.56694813	1.59E-16
PDHB	ATP6V0D1	0.56692861	1.59E-16
PDHB	ZNF416	0.56686699	1.61E-16
PDHB	CALHM2	0.56685687	1.61E-16
PDHB	NKRF	0.5666742	1.65E-16
PDHB	DNAJC25-GNG10	0.56653781	1.69E-16
PDHB	VOPP1	0.56640833	1.72E-16
PDHB	SPOPL	0.56640357	1.72E-16
PDHB	IKBIP	0.5663953	1.72E-16
PDHB	UBE2L3	0.56637965	1.73E-16

PDHB	SPTY2D1	0.56634706	1.73E-16
PDHB	LMBR1	0.56631461	1.74E-16
PDHB	TTC8	0.56628706	1.75E-16
PDHB	PHF5A	0.56618744	1.78E-16
PDHB	TOB2	0.56596939	1.83E-16
PDHB	YY1	0.56591569	1.85E-16
PDHB	ZCRB1	0.56590816	1.85E-16
PDHB	OSGIN2	0.5658634	1.86E-16
PDHB	FBXL7	0.56575281	1.89E-16
PDHB	VCPIP1	0.56572086	1.9E-16
PDHB	TPM1	0.56570504	1.91E-16
PDHB	TRIM37	0.56570271	1.91E-16
PDHB	SERBP1	0.56569149	1.91E-16
PDHB	HDAC8	0.56560811	1.94E-16
PDHB	TEX10	0.56557645	1.94E-16
PDHB	RNF24	0.56540945	1.99E-16
PDHB	ITGB3BP	0.56527932	2.03E-16
PDHB	DNASE2	0.56525827	2.04E-16
PDHB	CTDSPL2	0.56494843	2.13E-16
PDHB	C12orf43	0.56487785	2.16E-16
PDHB	BNIP3L	0.56461562	2.24E-16
PDHB	CHMP3	0.56457869	2.25E-16
PDHB	MRPL19	0.56453568	2.27E-16
PDHB	MIGA1	0.56451353	2.27E-16
PDHB	PACRGL	0.5645014	2.28E-16
PDHB	ANKRD50	0.56438121	2.32E-16
PDHB	MRM2	0.56424364	2.37E-16
PDHB	SLC25A11	0.56410855	2.41E-16
PDHB	HSPA14	0.564083	2.42E-16
PDHB	NBR1	0.56407321	2.43E-16
PDHB	SLC30A6	0.56407165	2.43E-16
PDHB	PLEKHA3	0.56407007	2.43E-16
PDHB	CASP3	0.56406291	2.43E-16
PDHB	TERF2	0.5638346	2.51E-16
PDHB	ELAVL1	0.5638198	2.52E-16
PDHB	NIPA2	0.56381585	2.52E-16
PDHB	UNG	0.56376838	2.54E-16
PDHB	PA2G4	0.56360794	2.6E-16
PDHB	ROCK2	0.56354262	2.62E-16
PDHB	SGCE	0.56346693	2.65E-16
PDHB	ZC3HAV1	0.56338934	2.68E-16
PDHB	ITPR1	0.56330108	2.72E-16
PDHB	MNAT1	0.5630554	2.82E-16
PDHB	EMC2	0.56305085	2.82E-16
PDHB	OXR1	0.56303969	2.83E-16
PDHB	SNX5	0.56296743	2.86E-16
PDHB	TOE1	0.56294001	2.87E-16
PDHB	LRRC57	0.56283826	2.91E-16
PDHB	SART3	0.56273629	2.95E-16
PDHB	RANBP3	0.5627013	2.97E-16
PDHB	URM1	0.56258147	3.02E-16
PDHB	ACTR3	0.56249582	3.06E-16
PDHB	ZNF512	0.56248136	3.07E-16
PDHB	LAP3	0.5623872	3.11E-16
PDHB	CHIC2	0.5622513	3.17E-16
PDHB	MCC	0.56212204	3.23E-16
PDHB	TMEM263	0.56202375	3.28E-16
PDHB	ZNF532	0.56189786	3.34E-16

PDHB	URI1	0.56174314	3.42E-16
PDHB	LPP	0.56171877	3.43E-16
PDHB	NUB1	0.56171631	3.43E-16
PDHB	EXO5	0.56169287	3.44E-16
PDHB	DCAF12	0.56162516	3.48E-16
PDHB	LIN54	0.56154951	3.52E-16
PDHB	ITCH	0.56153259	3.52E-16
PDHB	MRTFA	0.5615199	3.53E-16
PDHB	NDFIP1	0.56145177	3.57E-16
PDHB	TIPRL	0.56136397	3.61E-16
PDHB	SNX16	0.56106964	3.77E-16
PDHB	ZNF184	0.56099287	3.81E-16
PDHB	NUP58	0.56097814	3.82E-16
PDHB	PLA2G15	0.56095191	3.84E-16
PDHB	ZBTB33	0.56091147	3.86E-16
PDHB	CCDC51	0.56082496	3.91E-16
PDHB	ACLY	0.56078705	3.93E-16
PDHB	STRN3	0.56078123	3.93E-16
PDHB	DHX35	0.56077555	3.94E-16
PDHB	ORC4	0.56063109	4.02E-16
PDHB	HCCS	0.56056546	4.06E-16
PDHB	TMEM185A	0.56049951	4.1E-16
PDHB	CPOX	0.56030774	4.21E-16
PDHB	MYADM	0.56025556	4.24E-16
PDHB	SLBP	0.56021829	4.27E-16
PDHB	KDM1A	0.56016151	4.3E-16
PDHB	PARVA	0.55999786	4.41E-16
PDHB	GMPR2	0.55981235	4.53E-16
PDHB	SDHAF2	0.55979403	4.54E-16
PDHB	ZNF609	0.55976653	4.56E-16
PDHB	FOXO1	0.55971165	4.59E-16
PDHB	SDC3	0.55967157	4.62E-16
PDHB	CAVIN1	0.55964174	4.64E-16
PDHB	CCDC97	0.55963013	4.65E-16
PDHB	CHMP1B	0.55962296	4.65E-16
PDHB	ATXN7L3B	0.55961422	4.66E-16
PDHB	TBL1XR1	0.55956368	4.69E-16
PDHB	CDKN1B	0.55944147	4.78E-16
PDHB	YEATS2	0.55939097	4.81E-16
PDHB	SLC15A4	0.55937401	4.82E-16
PDHB	ENOPH1	0.55934135	4.85E-16
PDHB	EIF2AK2	0.55931439	4.87E-16
PDHB	KIF5B	0.55924854	4.91E-16
PDHB	NUP50	0.55924706	4.91E-16
PDHB	SSH1	0.55921547	4.94E-16
PDHB	CAPZA1	0.55917935	4.96E-16
PDHB	SH3PXD2A	0.55914108	4.99E-16
PDHB	ZNFX1	0.55913117	5E-16
PDHB	TBC1D25	0.55912035	5.01E-16
PDHB	ZFP64	0.55910034	5.02E-16
PDHB	COL4A2	0.55906548	5.05E-16
PDHB	ARL3	0.55906156	5.05E-16
PDHB	GPN1	0.55893117	5.14E-16
PDHB	PRKD3	0.55879757	5.24E-16
PDHB	CAMK2G	0.55878124	5.26E-16
PDHB	PSMC6	0.55864386	5.36E-16
PDHB	IMPA1	0.55861965	5.38E-16
PDHB	TMEM9B	0.55861527	5.38E-16

PDHB	CLIP3	0.55860199	5.4E-16
PDHB	GLUD1	0.55858373	5.41E-16
PDHB	CAMKK2	0.55854934	5.44E-16
PDHB	CUL4A	0.55846703	5.5E-16
PDHB	TMEM50A	0.55837695	5.57E-16
PDHB	PIK3CA	0.55832238	5.62E-16
PDHB	ATF1	0.55826442	5.67E-16
PDHB	CFAP36	0.55809344	5.81E-16
PDHB	MED7	0.55808794	5.81E-16
PDHB	UTP14C	0.55807609	5.82E-16
PDHB	IL13RA1	0.5580607	5.83E-16
PDHB	PLXNC1	0.55804797	5.84E-16
PDHB	GNS	0.55803924	5.85E-16
PDHB	STAM	0.55801649	5.87E-16
PDHB	TSR2	0.55800381	5.88E-16
PDHB	CXorf56	0.55796388	5.92E-16
PDHB	LSM6	0.5578943	5.98E-16
PDHB	ERH	0.55780569	6.05E-16
PDHB	WIPI2	0.55776436	6.09E-16
PDHB	NCKIPSD	0.55775748	6.1E-16
PDHB	KDM3B	0.55759354	6.24E-16
PDHB	UBE2Z	0.5575588	6.27E-16
PDHB	UBAP1	0.55748177	6.34E-16
PDHB	MAP3K7CL	0.55744476	6.38E-16
PDHB	COL4A1	0.5573648	6.45E-16
PDHB	ZNF200	0.55736426	6.45E-16
PDHB	DNAJA1	0.55732463	6.49E-16
PDHB	CNRIP1	0.55731152	6.5E-16
PDHB	ATF7IP	0.55716921	6.63E-16
PDHB	NAAA	0.55715793	6.65E-16
PDHB	RAN	0.55709584	6.7E-16
PDHB	GLTP	0.55690025	6.9E-16
PDHB	TWF1	0.55689783	6.9E-16
PDHB	ATAD1	0.55685277	6.94E-16
PDHB	CDC123	0.55681681	6.98E-16
PDHB	TBC1D14	0.55673162	7.07E-16
PDHB	CDK4	0.55665823	7.14E-16
PDHB	ADGRA2	0.55664013	7.16E-16
PDHB	PPP1R21	0.55661334	7.19E-16
PDHB	ALS2	0.5565539	7.25E-16
PDHB	ECD	0.55653854	7.26E-16
PDHB	SEC22A	0.55641506	7.39E-16
PDHB	KLHDC8B	0.55637734	7.43E-16
PDHB	DNM1L	0.55628789	7.53E-16
PDHB	TMEM203	0.55618167	7.65E-16
PDHB	SMIM30	0.55615785	7.67E-16
PDHB	NCL	0.55611152	7.72E-16
PDHB	ABCB10	0.55610133	7.73E-16
PDHB	CREBL2	0.55607654	7.76E-16
PDHB	TMX2	0.55605289	7.79E-16
PDHB	STMN1	0.55603079	7.81E-16
PDHB	MAPK1	0.55594043	7.92E-16
PDHB	PIK3R1	0.55586018	8.01E-16
PDHB	TEFM	0.55585495	8.01E-16
PDHB	USF2	0.55582963	8.04E-16
PDHB	DCLRE1B	0.55575631	8.13E-16
PDHB	PRKG1	0.55571055	8.18E-16
PDHB	BLOC1S2	0.55560187	8.31E-16

PDHB	CDV3	0.55559863	8.31E-16
PDHB	ICE2	0.55530012	8.68E-16
PDHB	SNX29	0.5552725	8.71E-16
PDHB	FHL1	0.55520623	8.79E-16
PDHB	DEK	0.55519983	8.8E-16
PDHB	HMGN4	0.55519215	8.81E-16
PDHB	FZD1	0.5551525	8.86E-16
PDHB	PIK3IP1	0.55511225	8.91E-16
PDHB	DOCK1	0.55492877	9.15E-16
PDHB	ATE1	0.5547875	9.33E-16
PDHB	THYN1	0.55447907	9.75E-16
PDHB	ISY1	0.5542958	1E-15
PDHB	VRK2	0.55426226	1.01E-15
PDHB	ADAMTS2	0.55417467	1.02E-15
PDHB	TRIQK	0.55405624	1.04E-15
PDHB	APMAP	0.5540359	1.04E-15
PDHB	ODF2L	0.55402467	1.04E-15
PDHB	C8orf33	0.55402088	1.04E-15
PDHB	PTPRM	0.55385575	1.07E-15
PDHB	SPRTN	0.55384465	1.07E-15
PDHB	PEX26	0.55383581	1.07E-15
PDHB	DPYD	0.55371882	1.09E-15
PDHB	KIF2A	0.55357971	1.11E-15
PDHB	DYNLT3	0.55357494	1.11E-15
PDHB	GRWD1	0.5535543	1.11E-15
PDHB	TPST1	0.55350784	1.12E-15
PDHB	CCNDBP1	0.55350554	1.12E-15
PDHB	OTUD1	0.55337923	1.14E-15
PDHB	NDUFA5	0.55331289	1.15E-15
PDHB	NARS2	0.55325813	1.16E-15
PDHB	ZNF131	0.55324548	1.16E-15
PDHB	RASSF8	0.55314081	1.18E-15
PDHB	CBX3	0.55312197	1.18E-15
PDHB	ZEB2	0.55309592	1.19E-15
PDHB	POT1	0.55308696	1.19E-15
PDHB	ZBTB22	0.55297874	1.21E-15
PDHB	ARID5B	0.55283401	1.23E-15
PDHB	ZNF766	0.55280141	1.24E-15
PDHB	MAP1B	0.55279778	1.24E-15
PDHB	LDAH	0.55277243	1.24E-15
PDHB	AKAP12	0.552633	1.27E-15
PDHB	PKIG	0.55261838	1.27E-15
PDHB	KCNE4	0.55257641	1.28E-15
PDHB	RBBP5	0.55240119	1.31E-15
PDHB	RASAL2	0.55238994	1.31E-15
PDHB	MICOS10	0.55236867	1.32E-15
PDHB	SMIM10	0.55236068	1.32E-15
PDHB	TMEM109	0.55234479	1.32E-15
PDHB	DPY30	0.55222896	1.34E-15
PDHB	PHACTR2	0.55220895	1.35E-15
PDHB	KLF12	0.55211413	1.37E-15
PDHB	COPG2	0.55203149	1.38E-15
PDHB	TRAFD1	0.55196561	1.39E-15
PDHB	TK2	0.55194852	1.4E-15
PDHB	TMX1	0.55162518	1.46E-15
PDHB	STAG2	0.55157755	1.47E-15
PDHB	GPBP1	0.55154868	1.48E-15
PDHB	GLT8D2	0.55149164	1.49E-15

PDHB	SKAP2	0.55143401	1.5E-15
PDHB	GNB4	0.55143265	1.5E-15
PDHB	SHISA5	0.55141066	1.51E-15
PDHB	TOMM40L	0.55125884	1.54E-15
PDHB	CAB39	0.55124982	1.54E-15
PDHB	TAX1BP1	0.55112248	1.57E-15
PDHB	MICU1	0.55106723	1.58E-15
PDHB	MEGF9	0.55106014	1.58E-15
PDHB	BMP2K	0.55078259	1.65E-15
PDHB	RNPS1	0.55074817	1.66E-15
PDHB	PRRC1	0.55067448	1.67E-15
PDHB	AFAP1L1	0.55063662	1.68E-15
PDHB	IGFBP7	0.55058653	1.69E-15
PDHB	GFOD2	0.55045832	1.72E-15
PDHB	NEDD8	0.55020642	1.79E-15
PDHB	MCM6	0.55016833	1.8E-15
PDHB	LSM10	0.55012153	1.81E-15
PDHB	FASTKD5	0.55010915	1.81E-15
PDHB	DNAJC21	0.54992343	1.86E-15
PDHB	BRAP	0.54990312	1.86E-15
PDHB	TENM3	0.54979341	1.89E-15
PDHB	CTDSPL	0.54979101	1.89E-15
PDHB	NDUFA12	0.54968286	1.92E-15
PDHB	SPCS2	0.5496054	1.94E-15
PDHB	TAF6	0.54945829	1.98E-15
PDHB	QKI	0.54943144	1.99E-15
PDHB	SP2	0.54939793	2E-15
PDHB	ZBTB2	0.54939025	2E-15
PDHB	STMP1	0.54916988	2.07E-15
PDHB	KLF7	0.54906502	2.1E-15
PDHB	RAB8A	0.54899397	2.12E-15
PDHB	FAM120AOS	0.54890171	2.15E-15
PDHB	BBS12	0.54883625	2.17E-15
PDHB	RHOBTB2	0.54868954	2.21E-15
PDHB	GDE1	0.54865707	2.22E-15
PDHB	RRM1	0.54863111	2.23E-15
PDHB	KIF13A	0.54859491	2.24E-15
PDHB	TFAM	0.54847638	2.28E-15
PDHB	CEP89	0.5484243	2.29E-15
PDHB	NBL1	0.54840702	2.3E-15
PDHB	RMDN3	0.54832745	2.33E-15
PDHB	CTSL	0.5481673	2.38E-15
PDHB	NUMB	0.54807482	2.41E-15
PDHB	EIF1AX	0.54802295	2.43E-15
PDHB	FXR1	0.5479786	2.44E-15
PDHB	TDP1	0.54790113	2.47E-15
PDHB	JDP2	0.54789905	2.47E-15
PDHB	SPG21	0.54786663	2.48E-15
PDHB	WBP11	0.54785431	2.48E-15
PDHB	ATP13A3	0.54785305	2.48E-15
PDHB	SMG8	0.54785097	2.49E-15
PDHB	MRPS23	0.54771351	2.53E-15
PDHB	CGRRF1	0.54764433	2.56E-15
PDHB	EMC4	0.54762603	2.56E-15
PDHB	PTGES3	0.54756126	2.59E-15
PDHB	INTS14	0.54749861	2.61E-15
PDHB	NXPE3	0.54734612	2.67E-15
PDHB	ANKRD27	0.54733154	2.67E-15

PDHB	TOR1B	0.54730869	2.68E-15
PDHB	SF3A1	0.54730755	2.68E-15
PDHB	JMJD8	0.54709849	2.76E-15
PDHB	PIGBOS1	0.54703111	2.79E-15
PDHB	FUT11	0.54702336	2.79E-15
PDHB	PCBP1	0.54692042	2.83E-15
PDHB	VSTM4	0.54689253	2.84E-15
PDHB	NFATC3	0.54688438	2.84E-15
PDHB	COPA	0.54684127	2.86E-15
PDHB	FEZ1	0.54672287	2.91E-15
PDHB	OGDH	0.54666959	2.93E-15
PDHB	TRMT1L	0.54658178	2.97E-15
PDHB	PCDH18	0.5465481	2.98E-15
PDHB	TCEAL7	0.54642376	3.03E-15
PDHB	SPINDOC	0.54640581	3.04E-15
PDHB	LAYN	0.54623394	3.11E-15
PDHB	C3orf14	0.54616785	3.14E-15
PDHB	ARL6IP6	0.5461165	3.16E-15
PDHB	ESYT2	0.5460407	3.2E-15
PDHB	DPM1	0.54577541	3.32E-15
PDHB	MITF	0.54565865	3.37E-15
PDHB	RPL26L1	0.54563719	3.38E-15
PDHB	CCDC43	0.54563566	3.38E-15
PDHB	BRCC3	0.54554796	3.42E-15
PDHB	MINPP1	0.54536422	3.51E-15
PDHB	FAM91A1	0.54526853	3.56E-15
PDHB	ST6GALNAC5	0.5452205	3.58E-15
PDHB	DOCK9	0.54509797	3.64E-15
PDHB	STK4	0.54509694	3.64E-15
PDHB	ARHGAP31	0.54504598	3.67E-15
PDHB	TSHZ2	0.5448841	3.75E-15
PDHB	MOSPD1	0.54476589	3.81E-15
PDHB	RGL1	0.54476534	3.81E-15
PDHB	ARHGAP35	0.54474648	3.82E-15
PDHB	PAM	0.54473864	3.83E-15
PDHB	NDUFB6	0.54473784	3.83E-15
PDHB	SLC26A2	0.54471504	3.84E-15
PDHB	LMBRD1	0.54465562	3.87E-15
PDHB	TRMT6	0.54461495	3.89E-15
PDHB	TMEM200A	0.54457514	3.92E-15
PDHB	U2AF2	0.54454222	3.93E-15
PDHB	GTDC1	0.54448526	3.96E-15
PDHB	NAGK	0.54442142	4E-15
PDHB	TSC22D2	0.54438879	4.02E-15
PDHB	KRR1	0.54426988	4.08E-15
PDHB	CAMTA1	0.54415457	4.15E-15
PDHB	NGRN	0.5441346	4.16E-15
PDHB	DYNLT1	0.54410177	4.18E-15
PDHB	MAF	0.54400674	4.23E-15
PDHB	DIPK2A	0.54400567	4.24E-15
PDHB	DNAJB14	0.54394192	4.27E-15
PDHB	TAF2	0.54377122	4.37E-15
PDHB	TMEM59	0.54374557	4.39E-15
PDHB	EHD4	0.54373878	4.39E-15
PDHB	METAP1	0.54365577	4.44E-15
PDHB	SRSF7	0.54345757	4.57E-15
PDHB	PIK3R3	0.54345756	4.57E-15
PDHB	JMJD1C	0.5434505	4.57E-15

PDHB	GALNS	0.54341605	4.59E-15
PDHB	CHMP7	0.54336381	4.63E-15
PDHB	NASP	0.54332984	4.65E-15
PDHB	ATP2B1	0.54331173	4.66E-15
PDHB	SLC38A7	0.54329595	4.67E-15
PDHB	ANKRD42	0.54327658	4.68E-15
PDHB	SPAG7	0.54322918	4.71E-15
PDHB	MED21	0.5431992	4.73E-15
PDHB	GTF2H5	0.54319154	4.74E-15
PDHB	TMEM216	0.54312247	4.78E-15
PDHB	RNF2	0.54302068	4.85E-15
PDHB	TMEM251	0.54301196	4.86E-15
PDHB	TPD52L2	0.54299055	4.87E-15
PDHB	MED18	0.54279167	5E-15
PDHB	VEZT	0.54268558	5.08E-15
PDHB	DUSP7	0.54263603	5.11E-15
PDHB	TFG	0.5426101	5.13E-15
PDHB	MRPL42	0.54251915	5.2E-15
PDHB	COQ5	0.54247822	5.22E-15
PDHB	DUT	0.54241793	5.27E-15
PDHB	PSMF1	0.54239349	5.29E-15
PDHB	STX7	0.54230257	5.35E-15
PDHB	LLPH	0.54228117	5.37E-15
PDHB	SLC8A1	0.54221712	5.41E-15
PDHB	RNF25	0.54218556	5.44E-15
PDHB	CALU	0.54213141	5.48E-15
PDHB	NUCKS1	0.54202751	5.56E-15
PDHB	NDN	0.54199151	5.58E-15
PDHB	ADAM17	0.54196013	5.61E-15
PDHB	HNRNPA1	0.5419474	5.62E-15
PDHB	APH1B	0.54186385	5.68E-15
PDHB	DCAF6	0.54186336	5.68E-15
PDHB	USP39	0.54184573	5.7E-15
PDHB	VGLL4	0.54183507	5.71E-15
PDHB	TIMP2	0.54176698	5.76E-15
PDHB	RABEPK	0.54175422	5.77E-15
PDHB	DEXI	0.54174717	5.77E-15
PDHB	RPE	0.54166419	5.84E-15
PDHB	GALC	0.54166059	5.84E-15
PDHB	TLK2	0.54147143	6E-15
PDHB	KLHL5	0.54138593	6.07E-15
PDHB	MSL2	0.54137251	6.08E-15
PDHB	OPA1	0.54136553	6.08E-15
PDHB	PDE1A	0.54127923	6.16E-15
PDHB	SBF2	0.5411859	6.23E-15
PDHB	CRLF3	0.5410304	6.37E-15
PDHB	TRAF3	0.54100515	6.39E-15
PDHB	LDLRAD4	0.54092486	6.46E-15
PDHB	SMNDC1	0.54083576	6.54E-15
PDHB	PLPP1	0.54081756	6.56E-15
PDHB	HIVEP1	0.5407888	6.58E-15
PDHB	GOSR1	0.54063328	6.72E-15
PDHB	MAML2	0.54062689	6.73E-15
PDHB	TAOK3	0.54049698	6.85E-15
PDHB	HABP4	0.54040835	6.93E-15
PDHB	NECAP2	0.54024997	7.08E-15
PDHB	TNC	0.54023336	7.1E-15
PDHB	PTEN	0.54019126	7.14E-15

PDHB	FBN1	0.54017685	7.15E-15
PDHB	GNL3L	0.54016823	7.16E-15
PDHB	AMFR	0.54015545	7.17E-15
PDHB	OSBPL10	0.54008082	7.25E-15
PDHB	TRANK1	0.54003942	7.29E-15
PDHB	ZC3HC1	0.53989843	7.43E-15
PDHB	NABP2	0.53988246	7.45E-15
PDHB	RBM4	0.53983786	7.49E-15
PDHB	ATP8B2	0.53941939	7.93E-15
PDHB	TCTA	0.53934727	8.01E-15
PDHB	NUP98	0.53931745	8.04E-15
PDHB	NUDCD1	0.5392823	8.08E-15
PDHB	AK2	0.539237	8.13E-15
PDHB	C12orf65	0.53909395	8.29E-15
PDHB	RAMAC	0.53906155	8.32E-15
PDHB	UGCG	0.53900774	8.38E-15
PDHB	SH2B3	0.53889487	8.51E-15
PDHB	TMBIM4	0.53879371	8.63E-15
PDHB	PTGS1	0.53879292	8.63E-15
PDHB	SYNC	0.53877002	8.66E-15
PDHB	ZNF263	0.53874102	8.69E-15
PDHB	AKR1B1	0.53868767	8.76E-15
PDHB	SPARCL1	0.53864795	8.8E-15
PDHB	MPP5	0.53864409	8.81E-15
PDHB	UBE2E3	0.53859509	8.87E-15
PDHB	EDIL3	0.53851774	8.96E-15
PDHB	TGFB1I1	0.53847451	9.01E-15
PDHB	STAT5A	0.53826274	9.27E-15
PDHB	ZNF585A	0.53806771	9.52E-15
PDHB	ATL2	0.53802244	9.58E-15
PDHB	ARHGEF12	0.53787008	9.78E-15
PDHB	LAMP2	0.53775612	9.93E-15
PDHB	SUPT4H1	0.53774053	9.95E-15
PDHB	CDC42SE2	0.53772116	9.98E-15
PDHB	CSNK2A1	0.5377129	9.99E-15
PDHB	NAB1	0.53751966	1.03E-14
PDHB	ZNF623	0.53749988	1.03E-14
PDHB	PSMD7	0.53735209	1.05E-14
PDHB	RFTN1	0.53729179	1.06E-14
PDHB	RPS6KA3	0.53724365	1.06E-14
PDHB	GNPDA1	0.53722482	1.07E-14
PDHB	SPDYE3	0.53718983	1.07E-14
PDHB	PPP4R3B	0.53717089	1.07E-14
PDHB	RSU1	0.53712745	1.08E-14
PDHB	B3GNT2	0.53712109	1.08E-14
PDHB	B3GALNT1	0.53711895	1.08E-14
PDHB	ZC3H11A	0.53706952	1.09E-14
PDHB	LMOD1	0.53696041	1.11E-14
PDHB	TMEM60	0.5369046	1.11E-14
PDHB	PEX14	0.53679493	1.13E-14
PDHB	NEDD4	0.53668478	1.15E-14
PDHB	ROBO1	0.53655475	1.17E-14
PDHB	CLIP4	0.53638767	1.19E-14
PDHB	HARS2	0.53629392	1.21E-14
PDHB	DCTN5	0.53628949	1.21E-14
PDHB	WASHC2A	0.53625126	1.22E-14
PDHB	ZNRF2	0.5361761	1.23E-14
PDHB	PAQR3	0.53605603	1.25E-14

PDHB	FIP1L1	0.53603383	1.25E-14
PDHB	PIGS	0.53601736	1.25E-14
PDHB	PPCS	0.53588134	1.28E-14
PDHB	CHSY1	0.53581539	1.29E-14
PDHB	SF3B2	0.5357512	1.3E-14
PDHB	PRPF38A	0.53558962	1.33E-14
PDHB	HIBADH	0.53556718	1.33E-14
PDHB	DST	0.53537289	1.37E-14
PDHB	GABARAP	0.53530224	1.38E-14
PDHB	HMCN1	0.53526571	1.39E-14
PDHB	LRCH1	0.53525557	1.39E-14
PDHB	POGK	0.53524511	1.39E-14
PDHB	FER	0.53513731	1.41E-14
PDHB	RAB31	0.53513479	1.41E-14
PDHB	OLA1	0.53512278	1.41E-14
PDHB	DYRK2	0.53505587	1.43E-14
PDHB	NUDCD2	0.53500037	1.44E-14
PDHB	SH3BP5	0.53492731	1.45E-14
PDHB	NR1D2	0.53481229	1.47E-14
PDHB	ZNF398	0.53472421	1.49E-14
PDHB	ZNF629	0.53465267	1.51E-14
PDHB	RNF170	0.53460139	1.52E-14
PDHB	MAN1C1	0.53460118	1.52E-14
PDHB	CDC42BPA	0.53459484	1.52E-14
PDHB	ZNF322	0.53450078	1.54E-14
PDHB	ACO2	0.53449298	1.54E-14
PDHB	DCAF7	0.53432847	1.57E-14
PDHB	HACD2	0.53425001	1.59E-14
PDHB	ALDH9A1	0.5342394	1.59E-14
PDHB	SYNPO2	0.53414517	1.61E-14
PDHB	ADAM10	0.53411546	1.62E-14
PDHB	HOXA4	0.53402014	1.64E-14
PDHB	ZBTB8OS	0.53394965	1.65E-14
PDHB	PCSK7	0.53392445	1.66E-14
PDHB	HERC3	0.53378288	1.69E-14
PDHB	BZW1	0.5337051	1.71E-14
PDHB	MFAP3	0.53364557	1.72E-14
PDHB	PAK1IP1	0.53343997	1.77E-14
PDHB	RALB	0.53340129	1.78E-14
PDHB	PHF19	0.53307613	1.86E-14
PDHB	TOP1	0.53305266	1.86E-14
PDHB	TIAM2	0.5328378	1.92E-14
PDHB	ACSL4	0.53272056	1.95E-14
PDHB	GOLPH3	0.53265791	1.96E-14
PDHB	ATRAID	0.53264408	1.97E-14
PDHB	LAMA4	0.53261794	1.97E-14
PDHB	CREM	0.53260309	1.98E-14
PDHB	TMEM107	0.5325711	1.99E-14
PDHB	ALG11	0.53257078	1.99E-14
PDHB	NYNRIN	0.53255077	1.99E-14
PDHB	UBE2D1	0.5325367	2E-14
PDHB	HIVEP3	0.5324109	2.03E-14
PDHB	CRISPLD1	0.53240393	2.03E-14
PDHB	APP	0.53240321	2.03E-14
PDHB	HIVEP2	0.53236536	2.04E-14
PDHB	TADA1	0.53234522	2.05E-14
PDHB	ETV1	0.53222228	2.08E-14
PDHB	TMCC1	0.53222037	2.08E-14

PDHB	CDK19	0.53216611	2.1E-14
PDHB	ODF2	0.53209363	2.12E-14
PDHB	SMAD1	0.53208616	2.12E-14
PDHB	RAE1	0.53199461	2.14E-14
PDHB	PSMA4	0.53190573	2.17E-14
PDHB	FBXO28	0.53189201	2.17E-14
PDHB	HMOX2	0.53187364	2.18E-14
PDHB	BRD3OS	0.53181903	2.2E-14
PDHB	SNAPC1	0.53180681	2.2E-14
PDHB	HEG1	0.53176701	2.21E-14
PDHB	PROS1	0.53174193	2.22E-14
PDHB	TCN2	0.53172467	2.22E-14
PDHB	VPS26A	0.53171278	2.23E-14
PDHB	RAI14	0.53170563	2.23E-14
PDHB	FNBP1	0.5316462	2.25E-14
PDHB	GPKOW	0.53162487	2.25E-14
PDHB	IPPK	0.53161715	2.25E-14
PDHB	PLSCR3	0.53161542	2.26E-14
PDHB	PRR14L	0.53159533	2.26E-14
PDHB	UBE2K	0.53139962	2.32E-14
PDHB	CACNA2D1	0.53136424	2.33E-14
PDHB	CISD1	0.53136093	2.33E-14
PDHB	ERVK3-1	0.53132421	2.34E-14
PDHB	KIRREL1	0.53132268	2.34E-14
PDHB	PCNA	0.53131193	2.35E-14
PDHB	DCUN1D3	0.53125489	2.37E-14
PDHB	PTGR1	0.5312088	2.38E-14
PDHB	NPM1	0.53118369	2.39E-14
PDHB	GADD45A	0.53104758	2.43E-14
PDHB	OLFML1	0.53103949	2.43E-14
PDHB	CPED1	0.5310044	2.44E-14
PDHB	EXOC4	0.53087409	2.49E-14
PDHB	ATPSCKMT	0.53086542	2.49E-14
PDHB	PRPS1	0.53084963	2.5E-14
PDHB	ATG9A	0.53079243	2.51E-14
PDHB	AOC3	0.53076842	2.52E-14
PDHB	TP53RK	0.53073364	2.53E-14
PDHB	CDADC1	0.53068493	2.55E-14
PDHB	UGDH	0.53067037	2.55E-14
PDHB	FADS2	0.53066114	2.56E-14
PDHB	HIP1	0.53055586	2.59E-14
PDHB	SLC25A24	0.5304907	2.62E-14
PDHB	SLC12A6	0.53045935	2.63E-14
PDHB	GRSF1	0.53027701	2.69E-14
PDHB	VPS18	0.53023546	2.71E-14
PDHB	CSTF2	0.53007211	2.76E-14
PDHB	AGPAT1	0.52994479	2.81E-14
PDHB	RCAN1	0.52980321	2.86E-14
PDHB	RND3	0.52977065	2.88E-14
PDHB	CHRAC1	0.529644	2.92E-14
PDHB	SS18	0.5296238	2.93E-14
PDHB	PI4K2B	0.529591	2.94E-14
PDHB	TMEM138	0.52947705	2.99E-14
PDHB	SMC1A	0.5294579	3E-14
PDHB	SDF4	0.52945537	3E-14
PDHB	RDH11	0.52938451	3.03E-14
PDHB	REXO2	0.52935004	3.04E-14
PDHB	CCT8	0.5291878	3.1E-14

PDHB	NPHP1	0.52916748	3.11E-14
PDHB	HINT3	0.52909511	3.14E-14
PDHB	UBAC2	0.52905995	3.16E-14
PDHB	TMOD3	0.52905392	3.16E-14
PDHB	PIK3R4	0.52901746	3.18E-14
PDHB	DLC1	0.52894906	3.2E-14
PDHB	SLC30A1	0.52877643	3.28E-14
PDHB	REEP3	0.52873332	3.3E-14
PDHB	ZNF281	0.52864033	3.34E-14
PDHB	NUP37	0.52859466	3.36E-14
PDHB	WDR89	0.52849769	3.4E-14
PDHB	UQCRC2	0.52847068	3.41E-14
PDHB	FADS1	0.52845567	3.42E-14
PDHB	TTC9C	0.52840542	3.44E-14
PDHB	PRCP	0.52838041	3.45E-14
PDHB	UBAP2L	0.52834738	3.47E-14
PDHB	STAU1	0.52829932	3.49E-14
PDHB	DYNC2LI1	0.52823667	3.52E-14
PDHB	CETN2	0.52807707	3.59E-14
PDHB	MAGOH	0.52794212	3.65E-14
PDHB	CEBPZOS	0.5279129	3.67E-14
PDHB	SH3D19	0.52786727	3.69E-14
PDHB	PSMD8	0.52785568	3.7E-14
PDHB	ARHGAP21	0.52782992	3.71E-14
PDHB	CDIPT	0.52778587	3.73E-14
PDHB	KCTD9	0.52775545	3.75E-14
PDHB	CDC42SE1	0.52774635	3.75E-14
PDHB	SLC35F6	0.52774506	3.75E-14
PDHB	OTUD5	0.52767321	3.79E-14
PDHB	ARF6	0.527653	3.8E-14
PDHB	ACTL6A	0.52753045	3.86E-14
PDHB	MESD	0.5275289	3.86E-14
PDHB	SYAP1	0.52744507	3.9E-14
PDHB	IPO7	0.52744433	3.9E-14
PDHB	TRAM2	0.52743889	3.9E-14
PDHB	BNC2	0.52728699	3.98E-14
PDHB	RSF1	0.52725899	4E-14
PDHB	ELF1	0.52713674	4.06E-14
PDHB	TCEAL9	0.52709296	4.08E-14
PDHB	CAMK2D	0.52706382	4.1E-14
PDHB	ATP5MC3	0.52704415	4.11E-14
PDHB	KLF6	0.52695364	4.16E-14
PDHB	ITGA9	0.52690275	4.19E-14
PDHB	F13A1	0.52673374	4.28E-14
PDHB	EIF2B2	0.52670895	4.29E-14
PDHB	UBN1	0.52663988	4.33E-14
PDHB	TMEM14C	0.52659391	4.36E-14
PDHB	LRRC8C	0.52644589	4.44E-14
PDHB	PRICKLE1	0.52641585	4.46E-14
PDHB	NUDT4	0.52631515	4.52E-14
PDHB	LRBA	0.52620145	4.59E-14
PDHB	MID1	0.52609674	4.65E-14
PDHB	PPP1R9B	0.52596015	4.73E-14
PDHB	SPARC	0.52595657	4.73E-14
PDHB	TSPAN18	0.52594361	4.74E-14
PDHB	HDGFL3	0.5255542	4.99E-14
PDHB	ANXA5	0.52550322	5.02E-14
PDHB	FNBP1L	0.52543235	5.07E-14

PDHB	SNUPN	0.52539375	5.09E-14
PDHB	RIN2	0.52534795	5.12E-14
PDHB	PDK3	0.52533443	5.13E-14
PDHB	TMEM47	0.52515464	5.25E-14
PDHB	USP11	0.52508267	5.3E-14
PDHB	CMC1	0.52486358	5.45E-14
PDHB	MIS18BP1	0.52485932	5.46E-14
PDHB	TMX4	0.52482147	5.48E-14
PDHB	ECM2	0.52476521	5.52E-14
PDHB	MIB1	0.52474772	5.54E-14
PDHB	SEC23IP	0.52468657	5.58E-14
PDHB	IL6ST	0.52464133	5.61E-14
PDHB	THY1	0.52448853	5.72E-14
PDHB	FAM120A	0.52436535	5.82E-14
PDHB	PLN	0.52418499	5.95E-14
PDHB	B4GALT5	0.52401302	6.09E-14
PDHB	PLGRKT	0.52401206	6.09E-14
PDHB	DNAJC18	0.523968	6.12E-14
PDHB	IRF2BP2	0.52394063	6.14E-14
PDHB	PPP2R2A	0.52391151	6.17E-14
PDHB	DNAJC10	0.52388994	6.18E-14
PDHB	LYPLA1	0.52384696	6.22E-14
PDHB	PRKDC	0.52357425	6.44E-14
PDHB	ZNF521	0.52357056	6.44E-14
PDHB	MEX3C	0.52349469	6.51E-14
PDHB	RFLNB	0.52348214	6.52E-14
PDHB	HSBP1	0.52345999	6.54E-14
PDHB	SPOCK1	0.52338291	6.6E-14
PDHB	IVD	0.5233718	6.61E-14
PDHB	HOMER1	0.52317422	6.78E-14
PDHB	ATP6V1H	0.52314661	6.81E-14
PDHB	SORCS2	0.52313645	6.81E-14
PDHB	TRAPPC1	0.52309654	6.85E-14
PDHB	PXDN	0.52299362	6.94E-14
PDHB	ACTA2	0.52295731	6.97E-14
PDHB	RASA1	0.52283619	7.08E-14
PDHB	ENSA	0.52281855	7.1E-14
PDHB	DNAL1	0.52279843	7.12E-14
PDHB	INPP5A	0.52268955	7.22E-14
PDHB	RAB22A	0.5226801	7.23E-14
PDHB	NFKB1	0.52263548	7.27E-14
PDHB	ZDHHC6	0.52259325	7.31E-14
PDHB	GALNT1	0.52252588	7.37E-14
PDHB	FKBP3	0.52227738	7.61E-14
PDHB	KATNBL1	0.52218264	7.7E-14
PDHB	LAMP1	0.5221037	7.78E-14
PDHB	RNASEH1	0.52207383	7.81E-14
PDHB	CYFIP1	0.5220259	7.86E-14
PDHB	MSANTD3	0.5220177	7.87E-14
PDHB	CD93	0.52201474	7.87E-14
PDHB	BMS1	0.52200849	7.88E-14
PDHB	GCC2	0.52198094	7.9E-14
PDHB	MASTL	0.52196538	7.92E-14
PDHB	CKAP2	0.52187461	8.01E-14
PDHB	PSEN1	0.52175822	8.13E-14
PDHB	ARFGEF1	0.52161947	8.28E-14
PDHB	CHN1	0.52150425	8.4E-14
PDHB	TBC1D9	0.52146856	8.44E-14

PDHB	LASP1	0.52146249	8.45E-14
PDHB	CBLB	0.52142916	8.48E-14
PDHB	EID2	0.52142517	8.49E-14
PDHB	MAZ	0.5214013	8.51E-14
PDHB	SCFD2	0.5213963	8.52E-14
PDHB	DGCR2	0.52132569	8.59E-14
PDHB	ZDHHC5	0.52131423	8.61E-14
PDHB	IFNGR1	0.5213006	8.62E-14
PDHB	SNX27	0.52116927	8.77E-14
PDHB	CDC73	0.52097017	8.99E-14
PDHB	NCOA3	0.52084893	9.13E-14
PDHB	CPQ	0.52082808	9.16E-14
PDHB	ATP11C	0.52076141	9.24E-14
PDHB	ZNF423	0.52075782	9.24E-14
PDHB	CREB1	0.52070842	9.3E-14
PDHB	NEO1	0.52065612	9.36E-14
PDHB	VKORC1L1	0.52062052	9.4E-14
PDHB	CDK12	0.52057165	9.46E-14
PDHB	SLC41A3	0.52050071	9.55E-14
PDHB	MED19	0.52048613	9.57E-14
PDHB	MEF2C	0.5204423	9.62E-14
PDHB	COMMD5	0.52039422	9.68E-14
PDHB	SCRN1	0.52037051	9.71E-14
PDHB	AGPAT4	0.52030397	9.79E-14
PDHB	LIMS1	0.52030127	9.8E-14
PDHB	PTK2	0.52022612	9.89E-14
PDHB	BCL7B	0.5202238	9.89E-14
PDHB	SLC25A13	0.52022336	9.89E-14
PDHB	ALKBH1	0.5201694	9.96E-14
PDHB	COMMD7	0.52016615	9.97E-14
PDHB	SARNP	0.51994969	1.02E-13
PDHB	LLGL1	0.51991723	1.03E-13
PDHB	MED14	0.51991022	1.03E-13
PDHB	GRPEL2	0.51986526	1.04E-13
PDHB	SC5D	0.51980061	1.04E-13
PDHB	KCTD17	0.51970412	1.06E-13
PDHB	DIP2B	0.5195988	1.07E-13
PDHB	AQR	0.51953017	1.08E-13
PDHB	TDP2	0.51946616	1.09E-13
PDHB	ACBD3	0.51930315	1.11E-13
PDHB	COMMD1	0.51928219	1.12E-13
PDHB	ERBIN	0.5192637	1.12E-13
PDHB	RIOX1	0.51914863	1.13E-13
PDHB	RRP36	0.51911843	1.14E-13
PDHB	NCK1	0.51909709	1.14E-13
PDHB	RO60	0.51908039	1.14E-13
PDHB	GPD2	0.51891248	1.17E-13
PDHB	MAP3K13	0.51889352	1.17E-13
PDHB	XPR1	0.51885229	1.18E-13
PDHB	RRP15	0.51884481	1.18E-13
PDHB	ZBTB41	0.51876072	1.19E-13
PDHB	DIABLO	0.51876027	1.19E-13
PDHB	HECA	0.51875857	1.19E-13
PDHB	TNPO3	0.51872862	1.2E-13
PDHB	SPTSSA	0.5187004	1.2E-13
PDHB	BLOC1S5	0.51868605	1.2E-13
PDHB	AHCTF1	0.51853009	1.23E-13
PDHB	TCF4	0.51849414	1.23E-13

PDHB	GNA13	0.51849387	1.23E-13
PDHB	GLB1L	0.51844805	1.24E-13
PDHB	TMEM87B	0.51841326	1.24E-13
PDHB	MAP4K4	0.51837226	1.25E-13
PDHB	LXN	0.51834287	1.26E-13
PDHB	ACAA2	0.51831994	1.26E-13
PDHB	VCP	0.51828478	1.27E-13
PDHB	MAFG	0.51821245	1.28E-13
PDHB	KLHDC3	0.51819277	1.28E-13
PDHB	RAD51C	0.51817642	1.28E-13
PDHB	RUVBL1	0.5181646	1.28E-13
PDHB	SIPA1L1	0.51813219	1.29E-13
PDHB	DNAJC9	0.51812457	1.29E-13
PDHB	TMEM248	0.51808352	1.3E-13
PDHB	VASH1	0.51803632	1.31E-13
PDHB	TM9SF2	0.51777848	1.35E-13
PDHB	SLC6A6	0.51775072	1.35E-13
PDHB	NSMCE2	0.51763251	1.37E-13
PDHB	CAMLG	0.51761672	1.38E-13
PDHB	DACT3	0.51760783	1.38E-13
PDHB	CLMP	0.51752404	1.39E-13
PDHB	PNRC1	0.51750468	1.4E-13
PDHB	TVP23B	0.51742322	1.41E-13
PDHB	PLEKHA2	0.51737379	1.42E-13
PDHB	ARMCX6	0.51730872	1.43E-13
PDHB	FN3KRP	0.5172826	1.44E-13
PDHB	STAMBP	0.51727208	1.44E-13
PDHB	ABCC1	0.51723688	1.44E-13
PDHB	HMGXB3	0.51718371	1.45E-13
PDHB	FGD5	0.51716328	1.46E-13
PDHB	SCOC	0.51697008	1.49E-13
PDHB	COQ6	0.51695177	1.5E-13
PDHB	SVIP	0.51686461	1.51E-13
PDHB	ZCCHC24	0.51683594	1.52E-13
PDHB	FAM102B	0.51680416	1.53E-13
PDHB	ZDHHC20	0.5167794	1.53E-13
PDHB	NIP7	0.51672491	1.54E-13
PDHB	CCDC59	0.51668203	1.55E-13
PDHB	CXorf38	0.51667181	1.55E-13
PDHB	TTC33	0.51660192	1.56E-13
PDHB	SCAF11	0.51659307	1.57E-13
PDHB	MAP3K20	0.51657342	1.57E-13
PDHB	NRP2	0.51648999	1.59E-13
PDHB	TRAPPC2B	0.51643628	1.6E-13
PDHB	DPY19L1	0.5162248	1.64E-13
PDHB	RAB3GAP2	0.51607616	1.67E-13
PDHB	ARAP1	0.5160295	1.68E-13
PDHB	NEURL1B	0.51599095	1.69E-13
PDHB	VMA21	0.515974	1.69E-13
PDHB	GM2A	0.5159231	1.7E-13
PDHB	SLC39A9	0.51582145	1.73E-13
PDHB	NLGN2	0.51576552	1.74E-13
PDHB	GSTCD	0.51573481	1.75E-13
PDHB	LHFPL6	0.51569019	1.75E-13
PDHB	MTDH	0.51565906	1.76E-13
PDHB	SRRD	0.51563579	1.77E-13
PDHB	CUEDC2	0.5155369	1.79E-13
PDHB	TPGS2	0.51553462	1.79E-13

PDHB	ETS1	0.51552259	1.79E-13
PDHB	METTL2B	0.51537478	1.83E-13
PDHB	SRXN1	0.51503081	1.91E-13
PDHB	IPO13	0.51495276	1.92E-13
PDHB	TSPAN31	0.51494338	1.93E-13
PDHB	EDNRA	0.51490303	1.94E-13
PDHB	MBIP	0.51482204	1.96E-13
PDHB	RCAN3	0.51477456	1.97E-13
PDHB	PTPN12	0.5147458	1.98E-13
PDHB	KAT6A	0.51472435	1.98E-13
PDHB	DDX23	0.51470472	1.99E-13
PDHB	GPR176	0.51470469	1.99E-13
PDHB	DOLK	0.5146922	1.99E-13
PDHB	NCOA2	0.51462434	2.01E-13
PDHB	TNFSF12	0.51461799	2.01E-13
PDHB	BCL9	0.51460838	2.01E-13
PDHB	SDE2	0.51456342	2.02E-13
PDHB	PCBP4	0.51451122	2.03E-13
PDHB	DOCK11	0.5144676	2.05E-13
PDHB	BUB3	0.51442663	2.06E-13
PDHB	NIT2	0.51442012	2.06E-13
PDHB	TBCA	0.51440099	2.06E-13
PDHB	DSE	0.51436096	2.07E-13
PDHB	FNIP2	0.51422275	2.11E-13
PDHB	CNOT7	0.51415127	2.13E-13
PDHB	ZPR1	0.5141511	2.13E-13
PDHB	AMPD3	0.51413239	2.13E-13
PDHB	IGF2R	0.51399774	2.17E-13
PDHB	MRPS10	0.51398895	2.17E-13
PDHB	CCNG2	0.51381469	2.22E-13
PDHB	FRYL	0.51368082	2.26E-13
PDHB	CYBRD1	0.51360686	2.28E-13
PDHB	RARB	0.51355633	2.29E-13
PDHB	RTL8B	0.51342748	2.33E-13
PDHB	AZIN2	0.51342677	2.33E-13
PDHB	ST3GAL2	0.51335521	2.35E-13
PDHB	SMIM13	0.51330337	2.37E-13
PDHB	TSPAN2	0.51327757	2.37E-13
PDHB	RBMS2	0.51310796	2.42E-13
PDHB	MRPS36	0.51310046	2.43E-13
PDHB	LIPA	0.51296453	2.47E-13
PDHB	CCNK	0.51292933	2.48E-13
PDHB	VAT1	0.51283546	2.51E-13
PDHB	MYL9	0.51278583	2.52E-13
PDHB	LBH	0.51277189	2.53E-13
PDHB	DBN1	0.51270297	2.55E-13
PDHB	NOL7	0.51266781	2.56E-13
PDHB	NRP1	0.51265453	2.56E-13
PDHB	B2M	0.51254737	2.6E-13
PDHB	SUZ12	0.51247279	2.62E-13
PDHB	TTI2	0.51244937	2.63E-13
PDHB	SMC4	0.51242103	2.64E-13
PDHB	EBNA1BP2	0.51238314	2.65E-13
PDHB	ANP32A	0.51226086	2.69E-13
PDHB	MAPK7	0.51218845	2.72E-13
PDHB	LTA4H	0.51210994	2.74E-13
PDHB	SMARCA1	0.51202254	2.77E-13
PDHB	ZNF286A	0.51202045	2.77E-13

PDHB	SNN	0.51198937	2.78E-13
PDHB	DEPDC5	0.51191474	2.81E-13
PDHB	JARID2	0.5118858	2.82E-13
PDHB	ARMC9	0.51188294	2.82E-13
PDHB	FOXN2	0.51184312	2.84E-13
PDHB	KLHL29	0.51182682	2.84E-13
PDHB	UBAC1	0.51181827	2.84E-13
PDHB	MED29	0.51176784	2.86E-13
PDHB	GJA1	0.51166317	2.9E-13
PDHB	GJC1	0.51165831	2.9E-13
PDHB	NUDT15	0.51147756	2.97E-13
PDHB	F2R	0.51140809	2.99E-13
PDHB	DCBLD1	0.51136229	3.01E-13
PDHB	PATL1	0.51103729	3.13E-13
PDHB	VCAN	0.51099636	3.15E-13
PDHB	HIF1A	0.51098835	3.15E-13
PDHB	ZFYVE21	0.51095681	3.16E-13
PDHB	FXYD6	0.51082809	3.21E-13
PDHB	ADGRL2	0.5106948	3.27E-13
PDHB	CHTF8	0.51056277	3.32E-13
PDHB	KIAA2013	0.51052366	3.34E-13
PDHB	PIP5K1C	0.51051311	3.34E-13
PDHB	ZFPM2	0.51050745	3.34E-13
PDHB	RNF141	0.51041649	3.38E-13
PDHB	COQ2	0.51035051	3.41E-13
PDHB	ORAI2	0.51033309	3.42E-13
PDHB	ADD1	0.51006656	3.53E-13
PDHB	L3MBTL3	0.51006248	3.53E-13
PDHB	FZD7	0.50994361	3.59E-13
PDHB	TSC22D1	0.50992969	3.59E-13
PDHB	LRRCC1	0.50991018	3.6E-13
PDHB	BAG4	0.50978618	3.66E-13
PDHB	ZER1	0.50965795	3.71E-13
PDHB	SMURF2	0.50963131	3.73E-13
PDHB	SAR1B	0.50962308	3.73E-13
PDHB	AFAP1	0.50952231	3.78E-13
PDHB	SCPEP1	0.50944265	3.81E-13
PDHB	PGM2	0.50944119	3.81E-13
PDHB	SSX2IP	0.50914129	3.96E-13
PDHB	LARGE1	0.50899591	4.03E-13
PDHB	TRRAP	0.50893993	4.06E-13
PDHB	EPB41L3	0.50889283	4.08E-13
PDHB	FBXO5	0.50880749	4.12E-13
PDHB	GNA11	0.50862755	4.22E-13
PDHB	GRHPR	0.50861192	4.22E-13
PDHB	STAT1	0.50854869	4.26E-13
PDHB	MMD	0.50854577	4.26E-13
PDHB	CDC42BPB	0.50837801	4.35E-13
PDHB	ITGAV	0.50836086	4.36E-13
PDHB	RNF19B	0.50833829	4.37E-13
PDHB	GDF11	0.50833631	4.37E-13
PDHB	RTL8C	0.50814538	4.47E-13
PDHB	ADGRL4	0.50814047	4.47E-13
PDHB	RNF7	0.50805073	4.52E-13
PDHB	SVIL	0.50800337	4.55E-13
PDHB	FAM200B	0.50799477	4.55E-13
PDHB	SGPL1	0.50788822	4.61E-13
PDHB	SLC24A3	0.50782466	4.65E-13

PDHB	AP1AR	0.50778017	4.68E-13
PDHB	CDON	0.50774317	4.7E-13
PDHB	WDR53	0.50769642	4.72E-13
PDHB	SH3KBP1	0.50767601	4.74E-13
PDHB	PSMA3	0.50762288	4.77E-13
PDHB	PARP3	0.50761695	4.77E-13
PDHB	BPTF	0.50759916	4.78E-13
PDHB	NFIX	0.50758224	4.79E-13
PDHB	SPON1	0.50754081	4.81E-13
PDHB	NDUFB4	0.50737291	4.91E-13
PDHB	PTBP3	0.50735213	4.93E-13
PDHB	SRGAP2	0.50732383	4.94E-13
PDHB	GLI3	0.50709687	5.08E-13
PDHB	C11orf68	0.50702075	5.13E-13
PDHB	EFS	0.50686786	5.23E-13
PDHB	PSMA1	0.5068607	5.23E-13
PDHB	AMBRA1	0.50681084	5.26E-13
PDHB	PDE4DIP	0.50673033	5.32E-13
PDHB	NDC1	0.50670529	5.33E-13
PDHB	ENG	0.50668102	5.35E-13
PDHB	TPRG1L	0.50665604	5.36E-13
PDHB	ADAM23	0.50664465	5.37E-13
PDHB	PDZRN3	0.50660188	5.4E-13
PDHB	NOD1	0.50646933	5.49E-13
PDHB	ENAH	0.50636606	5.56E-13
PDHB	MSANTD3-TMEFF1	0.50634113	5.57E-13
PDHB	ITM2C	0.50631222	5.59E-13
PDHB	PANX1	0.5062202	5.66E-13
PDHB	ERF	0.506197	5.67E-13
PDHB	KPNB1	0.5061896	5.68E-13
PDHB	NF1	0.50614512	5.71E-13
PDHB	SSPN	0.50612186	5.72E-13
PDHB	SNAP23	0.5060973	5.74E-13
PDHB	ADGRF5	0.50598899	5.82E-13
PDHB	PPIL1	0.505933	5.86E-13
PDHB	PRPF40A	0.50593298	5.86E-13
PDHB	PLCG1	0.50581421	5.94E-13
PDHB	EFCAB11	0.50574469	5.99E-13
PDHB	SPRED1	0.50569672	6.03E-13
PDHB	ZNF526	0.50568316	6.04E-13
PDHB	RABGAP1L	0.50556342	6.13E-13
PDHB	PRKACB	0.50551837	6.16E-13
PDHB	CERS6	0.50548677	6.18E-13
PDHB	TRAK1	0.50546815	6.2E-13
PDHB	RAB3IL1	0.50541987	6.23E-13
PDHB	DAG1	0.50538869	6.26E-13
PDHB	PCGF5	0.50535699	6.28E-13
PDHB	ZMYM2	0.50532177	6.31E-13
PDHB	TMEM68	0.5052518	6.36E-13
PDHB	RELL1	0.50517534	6.42E-13
PDHB	SESN3	0.50512496	6.46E-13
PDHB	PSMC3	0.50511336	6.47E-13
PDHB	UBE2V1	0.50510546	6.48E-13
PDHB	CYCS	0.50507619	6.5E-13
PDHB	AC093323.1	0.50495574	6.6E-13
PDHB	NBDY	0.50491671	6.63E-13
PDHB	TYW1	0.50483728	6.69E-13
PDHB	UBE2L6	0.50479321	6.73E-13

PDHB	METTL8	0.50479104	6.73E-13
PDHB	MRPS31	0.50473315	6.78E-13
PDHB	DEDD	0.50463421	6.86E-13
PDHB	BASP1	0.50457958	6.9E-13
PDHB	NUFIP2	0.50455391	6.92E-13
PDHB	APOL6	0.50453837	6.94E-13
PDHB	CFL1	0.50453484	6.94E-13
PDHB	TMEM199	0.50444847	7.01E-13
PDHB	CACUL1	0.50435304	7.09E-13
PDHB	FBXL2	0.50426843	7.17E-13
PDHB	CELF2	0.50400099	7.4E-13
PDHB	SS18L2	0.50391334	7.48E-13
PDHB	LYSMD2	0.50389331	7.5E-13
PDHB	TAF9B	0.50381398	7.57E-13
PDHB	POLR2E	0.50379048	7.59E-13
PDHB	KLHL12	0.50377644	7.61E-13
PDHB	TNFAIP8L1	0.50368989	7.69E-13
PDHB	TP53INP2	0.50368887	7.69E-13
PDHB	WIPF1	0.50367081	7.7E-13
PDHB	CLNS1A	0.50365868	7.72E-13
PDHB	TMEM140	0.5036475	7.73E-13
PDHB	DERL1	0.50361808	7.75E-13
PDHB	TPM4	0.50360004	7.77E-13
PDHB	PGGT1B	0.50359497	7.77E-13
PDHB	KCTD12	0.50355537	7.81E-13
PDHB	PSMB5	0.50351982	7.85E-13
PDHB	TRNAU1AP	0.50346763	7.89E-13
PDHB	TMEM106B	0.50338043	7.98E-13
PDHB	USP6NL	0.50335523	8E-13
PDHB	BACH1	0.50328725	8.07E-13
PDHB	CSE1L	0.50328467	8.07E-13
PDHB	LOXL3	0.50325518	8.1E-13
PDHB	ATP6V1F	0.50323681	8.12E-13
PDHB	FEM1C	0.50315213	8.2E-13
PDHB	SOCS6	0.50305463	8.3E-13
PDHB	PHTF2	0.50299683	8.36E-13
PDHB	AXL	0.50297139	8.38E-13
PDHB	HACD3	0.50288073	8.47E-13
PDHB	BOC	0.50284404	8.51E-13
PDHB	GLIPR1	0.50283137	8.52E-13
PDHB	TCF20	0.50279107	8.57E-13
PDHB	PFN2	0.50269286	8.67E-13
PDHB	SAMM50	0.50262807	8.73E-13
PDHB	SF3B6	0.5026012	8.76E-13
PDHB	SYDE1	0.50254062	8.83E-13
PDHB	TRMT12	0.50252966	8.84E-13
PDHB	TRAPPC4	0.5025203	8.85E-13
PDHB	MFSD6	0.50247018	8.9E-13
PDHB	TTC23	0.50237475	9.01E-13
PDHB	RALA	0.50237254	9.01E-13
PDHB	ATP5PF	0.50224551	9.15E-13
PDHB	MCUB	0.50215273	9.25E-13
PDHB	ZMYM5	0.50213757	9.27E-13
PDHB	RAD51B	0.50211771	9.29E-13
PDHB	CNPPD1	0.50208029	9.33E-13
PDHB	HAND2	0.50199785	9.42E-13
PDHB	VPS45	0.50193517	9.49E-13
PDHB	CD59	0.50188471	9.55E-13

PDHB	MAPKAP1	0.5018274	9.62E-13
PDHB	AEBP2	0.50165809	9.81E-13
PDHB	LMCD1	0.50164779	9.83E-13
PDHB	TMEM87A	0.50151494	9.98E-13
PDHB	PPP1R12B	0.50131283	1.02E-12
PDHB	CDH5	0.50119782	1.04E-12
PDHB	RALBP1	0.50117049	1.04E-12
PDHB	CCT5	0.50116084	1.04E-12
PDHB	GRK6	0.501158	1.04E-12
PDHB	RAB11A	0.50113419	1.04E-12
PDHB	BROX	0.50107691	1.05E-12
PDHB	PARD3B	0.50100757	1.06E-12
PDHB	SLC44A1	0.50090487	1.07E-12
PDHB	TMEM165	0.50085425	1.08E-12
PDHB	NDUFAF5	0.50085148	1.08E-12
PDHB	JAK2	0.5008309	1.08E-12
PDHB	LRP1	0.50082952	1.08E-12
PDHB	SPAG16	0.5008137	1.09E-12
PDHB	FAM200A	0.50063679	1.11E-12
PDHB	SMIM15	0.50059041	1.12E-12
PDHB	DOCK10	0.50052682	1.12E-12
PDHB	LCLAT1	0.50042511	1.14E-12
PDHB	SSBP1	0.50041219	1.14E-12
PDHB	SP1	0.50029826	1.15E-12
PDHB	MAF1	0.5002784	1.16E-12
PDHB	SLC16A2	0.50019169	1.17E-12
PDHB	SEC11A	0.50010077	1.18E-12
SLC31A1	ACSL1	0.50002022	1.19E-12
SLC31A1	NUDCD2	0.50002865	1.19E-12
SLC31A1	ETFA	0.50022262	1.17E-12
SLC31A1	HECW2	0.50051201	1.13E-12
SLC31A1	LASP1	0.50071485	1.1E-12
SLC31A1	MIS18A	0.5010369	1.06E-12
SLC31A1	CLTC	0.50114128	1.04E-12
SLC31A1	ZBTB9	0.50117241	1.04E-12
SLC31A1	PPP2R5C	0.50122966	1.03E-12
SLC31A1	FKBP7	0.50124683	1.03E-12
SLC31A1	VAMP3	0.50129902	1.02E-12
SLC31A1	CROT	0.50143542	1.01E-12
SLC31A1	GSK3B	0.50153105	9.96E-13
SLC31A1	DFFA	0.50160538	9.88E-13
SLC31A1	SIPA1L3	0.50166557	9.81E-13
SLC31A1	DENR	0.50182026	9.62E-13
SLC31A1	MRPL49	0.5018421	9.6E-13
SLC31A1	FNBP1L	0.50196826	9.46E-13
SLC31A1	LIMS1	0.50199256	9.43E-13
SLC31A1	ITGAV	0.50220602	9.19E-13
SLC31A1	SEC11A	0.50225202	9.14E-13
SLC31A1	MAP1LC3B2	0.50225851	9.13E-13
SLC31A1	GLT8D1	0.50241092	8.97E-13
SLC31A1	LPP	0.50242711	8.95E-13
SLC31A1	CTBP2	0.50243797	8.94E-13
SLC31A1	ARL6IP1	0.50248631	8.89E-13
SLC31A1	MRPS10	0.50272412	8.63E-13
SLC31A1	C16orf87	0.50272958	8.63E-13
SLC31A1	BCL9	0.50285627	8.5E-13
SLC31A1	STMP1	0.50288941	8.46E-13
SLC31A1	EID1	0.50294149	8.41E-13

SLC31A1	WIPI2	0.50301648	8.34E-13
SLC31A1	MEX3C	0.50314841	8.2E-13
SLC31A1	AGPAT5	0.50315111	8.2E-13
SLC31A1	ABCG1	0.50316406	8.19E-13
SLC31A1	PSMD2	0.50316418	8.19E-13
SLC31A1	GUSB	0.50316963	8.18E-13
SLC31A1	ITGB1	0.50318676	8.17E-13
SLC31A1	TPM4	0.50338008	7.98E-13
SLC31A1	REST	0.50342158	7.94E-13
SLC31A1	SMG7	0.5035878	7.78E-13
SLC31A1	SLC24A1	0.50368466	7.69E-13
SLC31A1	STIM1	0.50371021	7.67E-13
SLC31A1	ABCC1	0.50384933	7.54E-13
SLC31A1	MSL2	0.50399085	7.41E-13
SLC31A1	GBE1	0.50411868	7.3E-13
SLC31A1	IWS1	0.50421903	7.21E-13
SLC31A1	PTGES3	0.50445058	7.01E-13
SLC31A1	XIAP	0.50445199	7.01E-13
SLC31A1	ATP5PB	0.5045594	6.92E-13
SLC31A1	HNRNPAB	0.50459145	6.89E-13
SLC31A1	MICU1	0.50463116	6.86E-13
SLC31A1	ASNSD1	0.50463273	6.86E-13
SLC31A1	RAB5B	0.50484727	6.68E-13
SLC31A1	PSMD11	0.50489221	6.65E-13
SLC31A1	FOSL2	0.50499106	6.57E-13
SLC31A1	YWHAZ	0.50499169	6.57E-13
SLC31A1	TTI2	0.5051107	6.47E-13
SLC31A1	SHROOM3	0.50530029	6.33E-13
SLC31A1	PIGT	0.50531265	6.32E-13
SLC31A1	CHSY1	0.50548507	6.18E-13
SLC31A1	SP1	0.50550878	6.17E-13
SLC31A1	ZNF766	0.50557247	6.12E-13
SLC31A1	TCEAL9	0.50558911	6.11E-13
SLC31A1	CNOT2	0.50566206	6.05E-13
SLC31A1	TRAM1	0.50572639	6.01E-13
SLC31A1	MAFG	0.50587103	5.9E-13
SLC31A1	MRPL44	0.5059595	5.84E-13
SLC31A1	TXNDC9	0.50605015	5.77E-13
SLC31A1	LACC1	0.50605735	5.77E-13
SLC31A1	ASPH	0.50633537	5.58E-13
SLC31A1	UHRF1BP1L	0.50684089	5.24E-13
SLC31A1	LAPTM4A	0.50689223	5.21E-13
SLC31A1	ALG1	0.50694807	5.18E-13
SLC31A1	DACT1	0.50697281	5.16E-13
SLC31A1	XRCC5	0.50702027	5.13E-13
SLC31A1	ABCB10	0.5070719	5.1E-13
SLC31A1	GSR	0.50708216	5.09E-13
SLC31A1	ENY2	0.50712247	5.07E-13
SLC31A1	KDELR3	0.50714432	5.05E-13
SLC31A1	DNAL1	0.50716965	5.04E-13
SLC31A1	PPP1CB	0.50719812	5.02E-13
SLC31A1	EIF1AD	0.50724241	4.99E-13
SLC31A1	EAPP	0.50734879	4.93E-13
SLC31A1	MYO1E	0.50735056	4.93E-13
SLC31A1	CASP6	0.50763034	4.76E-13
SLC31A1	ATP6V1B2	0.5076751	4.74E-13
SLC31A1	PDE4D	0.50770562	4.72E-13
SLC31A1	GNAI1	0.50773139	4.7E-13

SLC31A1	PLGRKT	0.5080615	4.52E-13
SLC31A1	MYO6	0.50811802	4.49E-13
SLC31A1	STIL	0.50859665	4.23E-13
SLC31A1	VDAC1	0.50867895	4.19E-13
SLC31A1	FBXL14	0.5087098	4.17E-13
SLC31A1	HECA	0.50874462	4.15E-13
SLC31A1	SC5D	0.50875583	4.15E-13
SLC31A1	MSI2	0.50882388	4.11E-13
SLC31A1	SKAP2	0.50895356	4.05E-13
SLC31A1	MYH9	0.50916035	3.95E-13
SLC31A1	SNRPB2	0.5092242	3.92E-13
SLC31A1	GNPDA1	0.50934424	3.86E-13
SLC31A1	RHEB	0.50952783	3.77E-13
SLC31A1	CHM	0.50990329	3.6E-13
SLC31A1	NR2F2	0.50997876	3.57E-13
SLC31A1	TRAFD1	0.51001736	3.55E-13
SLC31A1	RBM22	0.51010155	3.52E-13
SLC31A1	CBFB	0.51018299	3.48E-13
SLC31A1	TMEM183A	0.51023825	3.46E-13
SLC31A1	RFC3	0.51033859	3.42E-13
SLC31A1	VPS33A	0.5103526	3.41E-13
SLC31A1	SLMAP	0.51059049	3.31E-13
SLC31A1	BCL9L	0.51066032	3.28E-13
SLC31A1	RAB11A	0.51066833	3.28E-13
SLC31A1	FGD4	0.51096137	3.16E-13
SLC31A1	PTPRK	0.51104097	3.13E-13
SLC31A1	ACTR6	0.5110729	3.12E-13
SLC31A1	TCEAL8	0.51114239	3.09E-13
SLC31A1	MAD2L1	0.51134232	3.02E-13
SLC31A1	TMEM251	0.51143959	2.98E-13
SLC31A1	CUL4A	0.51146092	2.97E-13
SLC31A1	AURKA	0.51150656	2.96E-13
SLC31A1	MPHOSPH10	0.51154432	2.94E-13
SLC31A1	PELO	0.51156263	2.94E-13
SLC31A1	ARF1	0.51156448	2.94E-13
SLC31A1	BTN3A3	0.51171033	2.88E-13
SLC31A1	DYNC1LI1	0.51182192	2.84E-13
SLC31A1	CYREN	0.51209716	2.75E-13
SLC31A1	C1GALT1	0.51216606	2.72E-13
SLC31A1	ARMC8	0.51222662	2.7E-13
SLC31A1	IQCK	0.51222961	2.7E-13
SLC31A1	DYNLT3	0.51231536	2.67E-13
SLC31A1	HIVEP1	0.51237264	2.66E-13
SLC31A1	PLEKHA1	0.5124109	2.64E-13
SLC31A1	E2F3	0.51247993	2.62E-13
SLC31A1	IST1	0.51248449	2.62E-13
SLC31A1	WBP11	0.51248672	2.62E-13
SLC31A1	WDR89	0.51253389	2.6E-13
SLC31A1	LIPA	0.51276265	2.53E-13
SLC31A1	ADAM17	0.51298573	2.46E-13
SLC31A1	CCDC43	0.51301031	2.45E-13
SLC31A1	ARMC10	0.51303955	2.44E-13
SLC31A1	CCNY	0.51306022	2.44E-13
SLC31A1	SMC4	0.51308698	2.43E-13
SLC31A1	LRP12	0.51319811	2.4E-13
SLC31A1	TRANK1	0.5132963	2.37E-13
SLC31A1	ATG9A	0.51341249	2.33E-13
SLC31A1	EXOC6B	0.51344898	2.32E-13

SLC31A1	MFSD11	0.51357364	2.29E-13
SLC31A1	UBE2Z	0.51357666	2.29E-13
SLC31A1	ZMAT2	0.51377391	2.23E-13
SLC31A1	VRK2	0.51385663	2.21E-13
SLC31A1	TEFM	0.51392768	2.19E-13
SLC31A1	HNRNPA0	0.51412693	2.13E-13
SLC31A1	TP53INP2	0.51423366	2.11E-13
SLC31A1	SLC25A43	0.51424571	2.1E-13
SLC31A1	NIPSNAP1	0.51425509	2.1E-13
SLC31A1	NIPSNAP3A	0.51442672	2.06E-13
SLC31A1	YTHDF1	0.51447582	2.04E-13
SLC31A1	TRIM56	0.51449146	2.04E-13
SLC31A1	SUMO2	0.51463409	2E-13
SLC31A1	RAB8B	0.5147441	1.98E-13
SLC31A1	MAP1LC3B	0.51478002	1.97E-13
SLC31A1	LACTB2	0.51479859	1.96E-13
SLC31A1	KIF13A	0.51486665	1.95E-13
SLC31A1	MEF2A	0.51490555	1.94E-13
SLC31A1	SHOC2	0.5149613	1.92E-13
SLC31A1	MED21	0.51505123	1.9E-13
SLC31A1	KRR1	0.51514703	1.88E-13
SLC31A1	MCM4	0.51515477	1.88E-13
SLC31A1	SS18	0.5152147	1.86E-13
SLC31A1	NFE2L2	0.51527818	1.85E-13
SLC31A1	MSH2	0.51542621	1.81E-13
SLC31A1	WDR47	0.51545614	1.81E-13
SLC31A1	FAR2	0.51546446	1.81E-13
SLC31A1	DAG1	0.51581639	1.73E-13
SLC31A1	BFAR	0.51583633	1.72E-13
SLC31A1	HIF1A	0.51615241	1.66E-13
SLC31A1	TLE4	0.51622303	1.64E-13
SLC31A1	BECN1	0.51625315	1.64E-13
SLC31A1	RAB9A	0.51629142	1.63E-13
SLC31A1	CAP1	0.51651622	1.58E-13
SLC31A1	NKRF	0.51658207	1.57E-13
SLC31A1	GALNT10	0.51675705	1.53E-13
SLC31A1	SWAP70	0.51680376	1.53E-13
SLC31A1	METAP1	0.51694961	1.5E-13
SLC31A1	DDX23	0.51728399	1.44E-13
SLC31A1	PRKCA	0.51738245	1.42E-13
SLC31A1	RNF139	0.51738949	1.42E-13
SLC31A1	NEDD9	0.51762528	1.38E-13
SLC31A1	CORO1C	0.51766912	1.37E-13
SLC31A1	RNF26	0.51769251	1.36E-13
SLC31A1	PCCB	0.51773823	1.36E-13
SLC31A1	AIMP2	0.51787979	1.33E-13
SLC31A1	DNAAF5	0.51809567	1.3E-13
SLC31A1	USP12	0.51813349	1.29E-13
SLC31A1	TIGD2	0.51820442	1.28E-13
SLC31A1	IL10RB	0.51837067	1.25E-13
SLC31A1	TRAF3	0.51864087	1.21E-13
SLC31A1	CSTF2T	0.51880548	1.18E-13
SLC31A1	PACRGL	0.51886166	1.18E-13
SLC31A1	SPDL1	0.51910151	1.14E-13
SLC31A1	KLHL7	0.51912613	1.14E-13
SLC31A1	RTL6	0.51929661	1.11E-13
SLC31A1	TRMT2B	0.51933641	1.11E-13
SLC31A1	MBNL2	0.51945746	1.09E-13

SLC31A1	MLH1	0.51946754	1.09E-13
SLC31A1	DNM1L	0.51951913	1.08E-13
SLC31A1	MB21D2	0.51962908	1.07E-13
SLC31A1	HMMR	0.51991054	1.03E-13
SLC31A1	FAM210B	0.51994006	1.03E-13
SLC31A1	SNX2	0.51999284	1.02E-13
SLC31A1	CTSO	0.5201675	9.96E-14
SLC31A1	CBX5	0.52026884	9.84E-14
SLC31A1	RNF2	0.52048006	9.57E-14
SLC31A1	PHF20	0.52074844	9.25E-14
SLC31A1	REEP5	0.52093205	9.04E-14
SLC31A1	ASAP1	0.5209546	9.01E-14
SLC31A1	KCTD9	0.52095541	9.01E-14
SLC31A1	TMEM19	0.52096525	9E-14
SLC31A1	GPATCH11	0.52103482	8.92E-14
SLC31A1	DUSP11	0.5210643	8.89E-14
SLC31A1	YTHDF2	0.5212505	8.68E-14
SLC31A1	DTX3L	0.52128714	8.64E-14
SLC31A1	ARHGAP26	0.52139721	8.52E-14
SLC31A1	ERMP1	0.52140617	8.51E-14
SLC31A1	SOWAHC	0.52167751	8.22E-14
SLC31A1	GRK5	0.52168269	8.21E-14
SLC31A1	CCDC34	0.52189212	7.99E-14
SLC31A1	UBLCP1	0.52216718	7.72E-14
SLC31A1	IMPACT	0.52231098	7.58E-14
SLC31A1	TANK	0.52263748	7.27E-14
SLC31A1	HNRNPC	0.52265267	7.25E-14
SLC31A1	RPRD2	0.52271763	7.19E-14
SLC31A1	CREG1	0.52276373	7.15E-14
SLC31A1	POC1B	0.52296922	6.96E-14
SLC31A1	KREMEN1	0.52298282	6.95E-14
SLC31A1	NKIRAS2	0.52316998	6.78E-14
SLC31A1	PRKACA	0.52332858	6.65E-14
SLC31A1	COPS8	0.52378274	6.27E-14
SLC31A1	WDR43	0.52378411	6.27E-14
SLC31A1	ZMIZ1	0.52397674	6.12E-14
SLC31A1	PARPBP	0.52415144	5.98E-14
SLC31A1	FGFR1OP2	0.52418884	5.95E-14
SLC31A1	TDP2	0.52422638	5.92E-14
SLC31A1	ARF6	0.52443007	5.77E-14
SLC31A1	GOLGA7	0.52447026	5.74E-14
SLC31A1	ANAPC10	0.52461994	5.63E-14
SLC31A1	NUP62	0.52487324	5.45E-14
SLC31A1	SRXN1	0.52490001	5.43E-14
SLC31A1	PEX19	0.52491286	5.42E-14
SLC31A1	NUCKS1	0.52497736	5.37E-14
SLC31A1	MGRN1	0.52500865	5.35E-14
SLC31A1	DENND6A	0.52511778	5.28E-14
SLC31A1	PLBD2	0.52520408	5.22E-14
SLC31A1	NEK1	0.52528465	5.16E-14
SLC31A1	SLC38A1	0.52531302	5.15E-14
SLC31A1	CHRAC1	0.52534226	5.13E-14
SLC31A1	SLC30A1	0.52549193	5.03E-14
SLC31A1	BLOC1S5	0.52562973	4.94E-14
SLC31A1	IRF2BP2	0.52568967	4.9E-14
SLC31A1	PPCS	0.52584152	4.8E-14
SLC31A1	SCAMP1	0.52593767	4.74E-14
SLC31A1	ATP9A	0.52598442	4.72E-14

SLC31A1	PIP4P2	0.52598657	4.71E-14
SLC31A1	CHD9	0.52609654	4.65E-14
SLC31A1	MFAP1	0.52617516	4.6E-14
SLC31A1	URI1	0.5262255	4.57E-14
SLC31A1	TFDP1	0.52630367	4.52E-14
SLC31A1	UHMK1	0.52639054	4.47E-14
SLC31A1	NFIX	0.52644699	4.44E-14
SLC31A1	WDR20	0.52646511	4.43E-14
SLC31A1	CXorf56	0.52665067	4.33E-14
SLC31A1	NAB1	0.52676056	4.26E-14
SLC31A1	PTEN	0.52677575	4.26E-14
SLC31A1	PSMD14	0.52681135	4.24E-14
SLC31A1	PPP2CB	0.52681871	4.23E-14
SLC31A1	TPD52	0.52701635	4.12E-14
SLC31A1	FAM177A1	0.5273332	3.96E-14
SLC31A1	BMPR2	0.52769496	3.77E-14
SLC31A1	ANKS1A	0.52778427	3.73E-14
SLC31A1	ARL8B	0.52781641	3.72E-14
SLC31A1	FBXO28	0.52789696	3.68E-14
SLC31A1	LRRC8D	0.527986	3.63E-14
SLC31A1	TMX2	0.52804377	3.61E-14
SLC31A1	SLC38A7	0.52823033	3.52E-14
SLC31A1	ZNRF2	0.52838597	3.45E-14
SLC31A1	HOMER1	0.5285177	3.39E-14
SLC31A1	KCTD10	0.52853688	3.38E-14
SLC31A1	SAP30	0.52853857	3.38E-14
SLC31A1	FOXN2	0.52857195	3.37E-14
SLC31A1	UBAP1	0.52864626	3.33E-14
SLC31A1	DHX36	0.52887292	3.24E-14
SLC31A1	SNX1	0.52900311	3.18E-14
SLC31A1	KLHL5	0.52900644	3.18E-14
SLC31A1	FUT8	0.52905727	3.16E-14
SLC31A1	EDC3	0.52906589	3.15E-14
SLC31A1	POT1	0.52925309	3.08E-14
SLC31A1	DPH3	0.52936326	3.03E-14
SLC31A1	TAF1A	0.52937826	3.03E-14
SLC31A1	NAPG	0.529383	3.03E-14
SLC31A1	MAT2B	0.52939071	3.02E-14
SLC31A1	SNW1	0.52956314	2.96E-14
SLC31A1	SH3KBP1	0.52983399	2.85E-14
SLC31A1	FBXL3	0.52988822	2.83E-14
SLC31A1	OTUD7B	0.52994372	2.81E-14
SLC31A1	ZNHIT6	0.5300473	2.77E-14
SLC31A1	UXS1	0.5300695	2.77E-14
SLC31A1	RIT1	0.53029664	2.68E-14
SLC31A1	B4GALT1	0.53049549	2.61E-14
SLC31A1	SEH1L	0.53064994	2.56E-14
SLC31A1	ATP2C1	0.53077006	2.52E-14
SLC31A1	LRCH1	0.53079155	2.51E-14
SLC31A1	LMNB1	0.53089981	2.48E-14
SLC31A1	CDC42SE2	0.53122381	2.37E-14
SLC31A1	GOT2	0.53124475	2.37E-14
SLC31A1	CDC42	0.53150539	2.29E-14
SLC31A1	AAGAB	0.53185952	2.18E-14
SLC31A1	KNOP1	0.53200253	2.14E-14
SLC31A1	DPM1	0.53201772	2.14E-14
SLC31A1	BLZF1	0.53204545	2.13E-14
SLC31A1	ANKMY2	0.53206157	2.13E-14

SLC31A1	ZFYVE1	0.53221574	2.08E-14
SLC31A1	PIK3R1	0.53229674	2.06E-14
SLC31A1	CD164	0.53244877	2.02E-14
SLC31A1	PTPN1	0.53263128	1.97E-14
SLC31A1	ARL4A	0.53266346	1.96E-14
SLC31A1	NUDT3	0.53275765	1.94E-14
SLC31A1	LAP3	0.5328105	1.92E-14
SLC31A1	VOPP1	0.53283006	1.92E-14
SLC31A1	MDH2	0.53285626	1.91E-14
SLC31A1	CACUL1	0.53290616	1.9E-14
SLC31A1	SLC35A2	0.53298313	1.88E-14
SLC31A1	AAR2	0.53335183	1.79E-14
SLC31A1	PIK3C3	0.53344319	1.77E-14
SLC31A1	NCBP2	0.53346865	1.76E-14
SLC31A1	ERBIN	0.53348846	1.76E-14
SLC31A1	MRPL30	0.53358671	1.74E-14
SLC31A1	CIP2A	0.5336918	1.71E-14
SLC31A1	SGCB	0.53373106	1.7E-14
SLC31A1	SLC39A7	0.53389875	1.67E-14
SLC31A1	LRRFIP2	0.53420771	1.6E-14
SLC31A1	RALBP1	0.53427971	1.58E-14
SLC31A1	PICALM	0.53429135	1.58E-14
SLC31A1	AMMECR1	0.53431322	1.58E-14
SLC31A1	FAM204A	0.53439005	1.56E-14
SLC31A1	FAM20B	0.53445835	1.55E-14
SLC31A1	SRSF1	0.53451671	1.53E-14
SLC31A1	UGGT1	0.53459423	1.52E-14
SLC31A1	ABHD13	0.53466993	1.5E-14
SLC31A1	RSF1	0.53476239	1.48E-14
SLC31A1	SMC6	0.53482736	1.47E-14
SLC31A1	PCTP	0.5348398	1.47E-14
SLC31A1	COPG2	0.53490428	1.46E-14
SLC31A1	CTNNB1	0.53490919	1.46E-14
SLC31A1	SRGAP2B	0.53494509	1.45E-14
SLC31A1	ABHD2	0.53502463	1.43E-14
SLC31A1	OXNAD1	0.53531247	1.38E-14
SLC31A1	PWP1	0.53533912	1.37E-14
SLC31A1	SART3	0.53553303	1.34E-14
SLC31A1	EMC1	0.53563393	1.32E-14
SLC31A1	SDHAF3	0.53593402	1.27E-14
SLC31A1	PPP1R2	0.53594897	1.27E-14
SLC31A1	EMC2	0.53598329	1.26E-14
SLC31A1	SELENOP	0.53610647	1.24E-14
SLC31A1	RAP1A	0.53612729	1.24E-14
SLC31A1	SLC4A7	0.53627604	1.21E-14
SLC31A1	GALC	0.53629323	1.21E-14
SLC31A1	C5orf15	0.536348	1.2E-14
SLC31A1	YWHAE	0.53643819	1.19E-14
SLC31A1	CIPC	0.53650203	1.18E-14
SLC31A1	PFKM	0.53662833	1.16E-14
SLC31A1	GOLGA5	0.53703228	1.09E-14
SLC31A1	PRRG1	0.53712396	1.08E-14
SLC31A1	TERF2	0.53722633	1.07E-14
SLC31A1	FAM98B	0.53724672	1.06E-14
SLC31A1	FMOD	0.5374394	1.04E-14
SLC31A1	PLS3	0.53747769	1.03E-14
SLC31A1	JOSD1	0.53762023	1.01E-14
SLC31A1	ZNF281	0.53777881	9.9E-15

SLC31A1	NSRP1	0.5378161	9.85E-15
SLC31A1	BACH1	0.53782086	9.84E-15
SLC31A1	ZNF827	0.53783791	9.82E-15
SLC31A1	PTP4A2	0.53792424	9.71E-15
SLC31A1	PTPRG	0.53795826	9.66E-15
SLC31A1	DPY19L1	0.53796239	9.66E-15
SLC31A1	ARL14EP	0.5380006	9.61E-15
SLC31A1	PHACTR2	0.53800244	9.6E-15
SLC31A1	TMEM135	0.53824633	9.29E-15
SLC31A1	PRDX3	0.53825612	9.28E-15
SLC31A1	PLEKHG1	0.53843161	9.06E-15
SLC31A1	MTMR2	0.53858175	8.88E-15
SLC31A1	TMEM230	0.53862914	8.82E-15
SLC31A1	PITPNB	0.53894285	8.46E-15
SLC31A1	SPRTN	0.53918717	8.18E-15
SLC31A1	PPTC7	0.53938656	7.96E-15
SLC31A1	MAP3K20	0.53959244	7.74E-15
SLC31A1	FBXO7	0.53976404	7.57E-15
SLC31A1	EXOC4	0.53991303	7.41E-15
SLC31A1	DYRK1A	0.53992546	7.4E-15
SLC31A1	GDI2	0.53997212	7.36E-15
SLC31A1	ATF7IP	0.54002549	7.3E-15
SLC31A1	CDK19	0.54011607	7.21E-15
SLC31A1	C4orf3	0.5401617	7.17E-15
SLC31A1	CNST	0.54018254	7.15E-15
SLC31A1	ERI1	0.54020514	7.13E-15
SLC31A1	IQGAP1	0.5402335	7.1E-15
SLC31A1	TMED9	0.54024172	7.09E-15
SLC31A1	MARK2	0.54045377	6.89E-15
SLC31A1	SPRED1	0.54056624	6.78E-15
SLC31A1	STX12	0.5406598	6.7E-15
SLC31A1	RAB28	0.54069178	6.67E-15
SLC31A1	TANC2	0.54082936	6.55E-15
SLC31A1	DARS2	0.54101814	6.38E-15
SLC31A1	PIGW	0.54119541	6.23E-15
SLC31A1	UGP2	0.54120058	6.22E-15
SLC31A1	STK3	0.54122658	6.2E-15
SLC31A1	CALCOCO2	0.54140789	6.05E-15
SLC31A1	ATL2	0.54191508	5.64E-15
SLC31A1	SH3GLB1	0.54209361	5.51E-15
SLC31A1	OSTF1	0.54212368	5.48E-15
SLC31A1	DLST	0.54223348	5.4E-15
SLC31A1	ATAD2	0.54251636	5.2E-15
SLC31A1	SH3PXD2A	0.54269011	5.07E-15
SLC31A1	CDV3	0.54272051	5.05E-15
SLC31A1	C5orf24	0.54299205	4.87E-15
SLC31A1	SSX2IP	0.54300791	4.86E-15
SLC31A1	STAM	0.54301922	4.85E-15
SLC31A1	FNDC3B	0.54313294	4.78E-15
SLC31A1	ZFP1	0.54316463	4.75E-15
SLC31A1	ETNK1	0.54317725	4.75E-15
SLC31A1	CHMP1B	0.5432642	4.69E-15
SLC31A1	KBTBD4	0.5434169	4.59E-15
SLC31A1	PPP1R15B	0.54354133	4.51E-15
SLC31A1	HDAC2	0.54357841	4.49E-15
SLC31A1	MOB4	0.543606	4.47E-15
SLC31A1	EPS15	0.54378064	4.37E-15
SLC31A1	FNTB	0.54378167	4.37E-15

SLC31A1	HNRNPUL1	0.54388628	4.31E-15
SLC31A1	PPP2R2A	0.54416446	4.14E-15
SLC31A1	ANO6	0.54427852	4.08E-15
SLC31A1	ASAH1	0.54428555	4.07E-15
SLC31A1	AGA	0.54436158	4.03E-15
SLC31A1	AP2B1	0.54439857	4.01E-15
SLC31A1	NDE1	0.54443873	3.99E-15
SLC31A1	FBXL17	0.54452641	3.94E-15
SLC31A1	KCTD12	0.54473357	3.83E-15
SLC31A1	LIN52	0.54480094	3.8E-15
SLC31A1	CYFIP1	0.54487993	3.75E-15
SLC31A1	OCIAD1	0.54493912	3.72E-15
SLC31A1	DHFR	0.54495723	3.71E-15
SLC31A1	EXOC2	0.54502319	3.68E-15
SLC31A1	CYB5B	0.54507014	3.66E-15
SLC31A1	DLGAP4	0.54518224	3.6E-15
SLC31A1	ELF4	0.54539036	3.5E-15
SLC31A1	TWSG1	0.5454943	3.45E-15
SLC31A1	SPTLC2	0.54564391	3.38E-15
SLC31A1	FCHO2	0.54577308	3.32E-15
SLC31A1	TBL1XR1	0.54581545	3.3E-15
SLC31A1	RAD51AP1	0.54608579	3.18E-15
SLC31A1	CANT1	0.54609626	3.17E-15
SLC31A1	VCP	0.54650157	3E-15
SLC31A1	ZNF263	0.54664843	2.94E-15
SLC31A1	GRPEL1	0.54669973	2.92E-15
SLC31A1	DRAM2	0.54670629	2.91E-15
SLC31A1	DOCK9	0.54673953	2.9E-15
SLC31A1	EIF2AK2	0.5467953	2.88E-15
SLC31A1	UVRAG	0.54688738	2.84E-15
SLC31A1	PAFAH1B1	0.54692713	2.83E-15
SLC31A1	WIPF2	0.54693081	2.82E-15
SLC31A1	ARHGAP35	0.54699941	2.8E-15
SLC31A1	MIS18BP1	0.54703245	2.79E-15
SLC31A1	PCGF5	0.54707234	2.77E-15
SLC31A1	CLDN12	0.54722765	2.71E-15
SLC31A1	UBXN2B	0.54728331	2.69E-15
SLC31A1	SNX18	0.54750132	2.61E-15
SLC31A1	NPAT	0.54753738	2.6E-15
SLC31A1	MRPL35	0.54753777	2.6E-15
SLC31A1	NCAPD2	0.54756605	2.59E-15
SLC31A1	GLYR1	0.54756939	2.58E-15
SLC31A1	PARD3B	0.54760023	2.57E-15
SLC31A1	ACVR1	0.5476887	2.54E-15
SLC31A1	TAOK3	0.54770392	2.54E-15
SLC31A1	SNX5	0.5478117	2.5E-15
SLC31A1	CKAP2	0.54786333	2.48E-15
SLC31A1	CMTM6	0.54788512	2.47E-15
SLC31A1	NSL1	0.54794563	2.45E-15
SLC31A1	C1D	0.54826573	2.35E-15
SLC31A1	SAR1A	0.54828238	2.34E-15
SLC31A1	ATF2	0.54829536	2.34E-15
SLC31A1	FAM168A	0.54837617	2.31E-15
SLC31A1	OXR1	0.54840803	2.3E-15
SLC31A1	MTCH2	0.54842236	2.29E-15
SLC31A1	PDIA4	0.54857901	2.24E-15
SLC31A1	TRA2B	0.54897352	2.12E-15
SLC31A1	SOCS5	0.54897648	2.12E-15

SLC31A1	INTS12	0.54921093	2.05E-15
SLC31A1	NUP50	0.54921586	2.05E-15
SLC31A1	NID1	0.54945544	1.99E-15
SLC31A1	CIAPIN1	0.54950371	1.97E-15
SLC31A1	JKAMP	0.5496102	1.94E-15
SLC31A1	ACO2	0.54967344	1.93E-15
SLC31A1	PSMF1	0.54971313	1.91E-15
SLC31A1	UBE2H	0.54978295	1.9E-15
SLC31A1	ETV3	0.54981745	1.89E-15
SLC31A1	RALA	0.54999136	1.84E-15
SLC31A1	GNAI3	0.54999209	1.84E-15
SLC31A1	RAB35	0.55007411	1.82E-15
SLC31A1	HSPA14	0.55028948	1.77E-15
SLC31A1	SNX9	0.55030023	1.76E-15
SLC31A1	NEK6	0.55032987	1.76E-15
SLC31A1	GSKIP	0.55042823	1.73E-15
SLC31A1	AC010132.3	0.550461	1.72E-15
SLC31A1	ANKH	0.55068628	1.67E-15
SLC31A1	UBXN2A	0.55071407	1.66E-15
SLC31A1	OSBPL8	0.55096129	1.61E-15
SLC31A1	TMTC2	0.55106361	1.58E-15
SLC31A1	SLFN11	0.55110504	1.57E-15
SLC31A1	MPP5	0.5511191	1.57E-15
SLC31A1	RASAL2	0.55126828	1.54E-15
SLC31A1	MFHAS1	0.55131993	1.53E-15
SLC31A1	MANBA	0.55141053	1.51E-15
SLC31A1	SPATS2	0.55146048	1.5E-15
SLC31A1	NUMB	0.5515651	1.48E-15
SLC31A1	PRKDC	0.55156702	1.47E-15
SLC31A1	BMS1	0.55164266	1.46E-15
SLC31A1	RPGRIP1L	0.55182459	1.42E-15
SLC31A1	SNX29	0.55192096	1.4E-15
SLC31A1	FBXO34	0.55192939	1.4E-15
SLC31A1	PXMP4	0.55207507	1.37E-15
SLC31A1	CNOT7	0.55214735	1.36E-15
SLC31A1	RHOA	0.55220575	1.35E-15
SLC31A1	GEMIN5	0.55229108	1.33E-15
SLC31A1	AC008764.4	0.5523805	1.31E-15
SLC31A1	TARDBP	0.55249103	1.29E-15
SLC31A1	NUDT4B	0.55251988	1.29E-15
SLC31A1	ZNF45	0.55266124	1.26E-15
SLC31A1	IFT52	0.55276453	1.24E-15
SLC31A1	COMMD8	0.55286874	1.23E-15
SLC31A1	ZNF143	0.55303444	1.2E-15
SLC31A1	CAPRIN1	0.55308755	1.19E-15
SLC31A1	ZNF462	0.55314466	1.18E-15
SLC31A1	GOLPH3L	0.55315107	1.18E-15
SLC31A1	NEO1	0.55322465	1.17E-15
SLC31A1	RAB3GAP2	0.55326729	1.16E-15
SLC31A1	SFT2D2	0.55335758	1.14E-15
SLC31A1	LRRC41	0.55344007	1.13E-15
SLC31A1	SEC13	0.55352213	1.12E-15
SLC31A1	KDM3B	0.55358108	1.11E-15
SLC31A1	ICE2	0.55359993	1.11E-15
SLC31A1	CEBPG	0.55376922	1.08E-15
SLC31A1	GNA12	0.55390799	1.06E-15
SLC31A1	HMGCR	0.5539319	1.05E-15
SLC31A1	MOB1A	0.5539427	1.05E-15

SLC31A1	FBXO22	0.55407368	1.03E-15
SLC31A1	NUFIP2	0.55421565	1.01E-15
SLC31A1	ZNF207	0.55427342	1E-15
SLC31A1	AIDA	0.5543085	9.99E-16
SLC31A1	QTRT2	0.55439511	9.87E-16
SLC31A1	HNRNPA2B1	0.55451046	9.71E-16
SLC31A1	TMEM87A	0.55464381	9.53E-16
SLC31A1	LAMC1	0.55473272	9.41E-16
SLC31A1	ISY1-RAB43	0.55483477	9.27E-16
SLC31A1	TNRC18	0.55484063	9.26E-16
SLC31A1	HPSE	0.55489852	9.19E-16
SLC31A1	CMPK1	0.55522623	8.77E-16
SLC31A1	IPPK	0.55525446	8.73E-16
SLC31A1	ATAD1	0.55534739	8.62E-16
SLC31A1	TFG	0.55548441	8.45E-16
SLC31A1	C16orf70	0.55556446	8.35E-16
SLC31A1	GNB1	0.55562724	8.28E-16
SLC31A1	RAD18	0.55564966	8.25E-16
SLC31A1	ATXN1	0.55565255	8.25E-16
SLC31A1	PPM1B	0.55580386	8.07E-16
SLC31A1	HNRNPF	0.55583654	8.03E-16
SLC31A1	SKP1	0.5558528	8.02E-16
SLC31A1	LAMTOR3	0.55593051	7.93E-16
SLC31A1	COPZ1	0.55628472	7.53E-16
SLC31A1	HSPA9	0.55632929	7.49E-16
SLC31A1	PPP3CA	0.5564062	7.4E-16
SLC31A1	SLC12A8	0.55679159	7E-16
SLC31A1	TMEM237	0.55698004	6.82E-16
SLC31A1	GRB2	0.55703711	6.76E-16
SLC31A1	PAICS	0.55712262	6.68E-16
SLC31A1	B4GALT5	0.55727428	6.53E-16
SLC31A1	SLU7	0.55733573	6.48E-16
SLC31A1	CXCL16	0.55788846	5.98E-16
SLC31A1	CHMP2B	0.55794786	5.93E-16
SLC31A1	PIGS	0.55811904	5.79E-16
SLC31A1	AP3S1	0.55855606	5.43E-16
SLC31A1	PRR14L	0.55874071	5.29E-16
SLC31A1	KHDRBS1	0.55891973	5.15E-16
SLC31A1	SOAT1	0.5590087	5.09E-16
SLC31A1	PRKAG2	0.559134	5E-16
SLC31A1	RAB7A	0.55914524	4.99E-16
SLC31A1	HIBADH	0.55920223	4.95E-16
SLC31A1	PTPRJ	0.55942977	4.79E-16
SLC31A1	APBB2	0.55955867	4.7E-16
SLC31A1	MRPL42	0.55969203	4.61E-16
SLC31A1	ZKSCAN5	0.55977	4.56E-16
SLC31A1	BCAS2	0.55990809	4.46E-16
SLC31A1	RBM47	0.5599518	4.44E-16
SLC31A1	SNTB2	0.56000986	4.4E-16
SLC31A1	SETD3	0.56019122	4.28E-16
SLC31A1	TMOD3	0.5601922	4.28E-16
SLC31A1	ROCK1	0.56022847	4.26E-16
SLC31A1	METTL21A	0.5603988	4.16E-16
SLC31A1	USP10	0.56046278	4.12E-16
SLC31A1	RECQL	0.56056298	4.06E-16
SLC31A1	TUT7	0.5606345	4.02E-16
SLC31A1	PDCD10	0.56066324	4E-16
SLC31A1	ERGIC2	0.56087866	3.88E-16

SLC31A1	ZNFX1	0.56099499	3.81E-16
SLC31A1	ZMYM4	0.56101572	3.8E-16
SLC31A1	GNA11	0.56104278	3.79E-16
SLC31A1	UBA6	0.56104511	3.78E-16
SLC31A1	SLC26A2	0.56107063	3.77E-16
SLC31A1	RANBP9	0.56161806	3.48E-16
SLC31A1	GATAD2A	0.56179408	3.39E-16
SLC31A1	GCLM	0.56184522	3.37E-16
SLC31A1	TMEM170A	0.56185573	3.36E-16
SLC31A1	RAP1B	0.56189677	3.34E-16
SLC31A1	GMEB1	0.5620021	3.29E-16
SLC31A1	SEC22A	0.56202303	3.28E-16
SLC31A1	HACD3	0.56208	3.25E-16
SLC31A1	SP3	0.56226362	3.17E-16
SLC31A1	DIP2B	0.56229462	3.15E-16
SLC31A1	RRAGC	0.56235741	3.12E-16
SLC31A1	EHF	0.56238031	3.11E-16
SLC31A1	ARHGAP11A	0.56245088	3.08E-16
SLC31A1	SGO2	0.56252391	3.05E-16
SLC31A1	NPM1	0.56252897	3.05E-16
SLC31A1	OSBPL11	0.56260494	3.01E-16
SLC31A1	ELOVL5	0.56291784	2.88E-16
SLC31A1	KIAA1143	0.56295118	2.86E-16
SLC31A1	PNPO	0.56298571	2.85E-16
SLC31A1	GLRX	0.5632667	2.73E-16
SLC31A1	ATG3	0.56330382	2.72E-16
SLC31A1	KPNB1	0.56336962	2.69E-16
SLC31A1	SRSF3	0.56352141	2.63E-16
SLC31A1	PPP1CC	0.56367931	2.57E-16
SLC31A1	SNRNP27	0.56372633	2.55E-16
SLC31A1	PALLD	0.56400273	2.45E-16
SLC31A1	SUCLA2	0.56400496	2.45E-16
SLC31A1	NUDCD1	0.56411205	2.41E-16
SLC31A1	IMMT	0.56420607	2.38E-16
SLC31A1	DYNC1I2	0.56425956	2.36E-16
SLC31A1	RRM2B	0.56428064	2.35E-16
SLC31A1	KLF3	0.56435426	2.33E-16
SLC31A1	DSTN	0.56441999	2.31E-16
SLC31A1	VEZF1	0.56444213	2.3E-16
SLC31A1	SESTD1	0.56470524	2.21E-16
SLC31A1	MBD5	0.56477505	2.19E-16
SLC31A1	NDC1	0.5647837	2.19E-16
SLC31A1	ATMIN	0.56502953	2.11E-16
SLC31A1	DCLRE1B	0.56506258	2.1E-16
SLC31A1	SOS2	0.56521267	2.05E-16
SLC31A1	DENND1A	0.56536109	2.01E-16
SLC31A1	PDS5B	0.5653898	2E-16
SLC31A1	APMAP	0.56541687	1.99E-16
SLC31A1	MIB1	0.56561476	1.93E-16
SLC31A1	CPD	0.56562488	1.93E-16
SLC31A1	SUSD6	0.56579053	1.88E-16
SLC31A1	YWHAQ	0.56593773	1.84E-16
SLC31A1	DNAJA2	0.565957	1.84E-16
SLC31A1	HSP90AA1	0.5660341	1.82E-16
SLC31A1	PLAA	0.56619549	1.77E-16
SLC31A1	MSANTD3	0.56621413	1.77E-16
SLC31A1	KCTD20	0.56622111	1.77E-16
SLC31A1	SCP2	0.56634483	1.73E-16

SLC31A1	ZBTB2	0.56643488	1.71E-16
SLC31A1	UBE2V2	0.56647552	1.7E-16
SLC31A1	PPFIA1	0.56650751	1.69E-16
SLC31A1	FTO	0.56669551	1.65E-16
SLC31A1	WDR26	0.56670604	1.64E-16
SLC31A1	WSB2	0.56670946	1.64E-16
SLC31A1	ELF1	0.56674567	1.63E-16
SLC31A1	DIPK1A	0.56676764	1.63E-16
SLC31A1	PRRC2C	0.56686168	1.61E-16
SLC31A1	PSEN1	0.56692238	1.59E-16
SLC31A1	CHMP5	0.56698705	1.58E-16
SLC31A1	ICMT	0.56701826	1.57E-16
SLC31A1	COPS4	0.56709294	1.55E-16
SLC31A1	HSPE1-MOB4	0.56733918	1.5E-16
SLC31A1	SLC39A6	0.56735736	1.49E-16
SLC31A1	FOXP1	0.56765479	1.43E-16
SLC31A1	HACD2	0.56786154	1.38E-16
SLC31A1	TMEM68	0.56786496	1.38E-16
SLC31A1	HINT3	0.56816115	1.32E-16
SLC31A1	BTBD9	0.56823375	1.31E-16
SLC31A1	EIF2S2	0.5686619	1.23E-16
SLC31A1	LHFPL2	0.56869053	1.22E-16
SLC31A1	SSH1	0.56874477	1.21E-16
SLC31A1	SPRED2	0.56877691	1.21E-16
SLC31A1	WWP1	0.56909319	1.15E-16
SLC31A1	ABCF2	0.56921108	1.13E-16
SLC31A1	MAP2K1	0.56944864	1.09E-16
SLC31A1	AHCTF1	0.569525	1.08E-16
SLC31A1	SRP9	0.569575	1.07E-16
SLC31A1	SLC25A44	0.56962197	1.06E-16
SLC31A1	SERINC5	0.56974799	1.04E-16
SLC31A1	STRN3	0.56983988	1.03E-16
SLC31A1	UBE2N	0.56985809	1.03E-16
SLC31A1	IDH1	0.57000029	1E-16
SLC31A1	ST13	0.57001822	1E-16
SLC31A1	MESD	0.57016163	9.81E-17
SLC31A1	GNE	0.57017105	9.79E-17
SLC31A1	TCEA1	0.57017376	9.79E-17
SLC31A1	DCTD	0.57029322	9.62E-17
SLC31A1	WDFY1	0.57047915	9.35E-17
SLC31A1	STARD4	0.57057045	9.22E-17
SLC31A1	TBC1D14	0.57063972	9.13E-17
SLC31A1	SYPL1	0.57109531	8.52E-17
SLC31A1	TBC1D5	0.57115814	8.44E-17
SLC31A1	DCAF1	0.57117647	8.42E-17
SLC31A1	TFCP2	0.57126568	8.31E-17
SLC31A1	KAT6A	0.57127227	8.3E-17
SLC31A1	ARL2BP	0.57128455	8.28E-17
SLC31A1	TMEM267	0.57129436	8.27E-17
SLC31A1	CUL4B	0.57135186	8.2E-17
SLC31A1	GPD2	0.57144254	8.09E-17
SLC31A1	UPRT	0.57160434	7.89E-17
SLC31A1	MOSPD1	0.57164078	7.85E-17
SLC31A1	LRBA	0.57169728	7.78E-17
SLC31A1	CRIM1	0.57211854	7.3E-17
SLC31A1	DOLPP1	0.5721674	7.25E-17
SLC31A1	ATP2B1	0.57243491	6.96E-17
SLC31A1	HFE	0.57267792	6.71E-17

SLC31A1	KBTBD2	0.57268079	6.7E-17
SLC31A1	CHMP3	0.57283287	6.55E-17
SLC31A1	CNOT4	0.5728638	6.52E-17
SLC31A1	TRMT1L	0.57289808	6.49E-17
SLC31A1	YY1	0.57303137	6.36E-17
SLC31A1	SLC25A24	0.57320539	6.19E-17
SLC31A1	EPB41L2	0.57353255	5.89E-17
SLC31A1	TMTC3	0.57354235	5.88E-17
SLC31A1	MFN2	0.57391099	5.56E-17
SLC31A1	GLO1	0.57413112	5.38E-17
SLC31A1	RDH14	0.57418762	5.33E-17
SLC31A1	TFRC	0.57446451	5.11E-17
SLC31A1	SNAP23	0.57456364	5.03E-17
SLC31A1	NAGA	0.57462382	4.99E-17
SLC31A1	CASK	0.57464563	4.97E-17
SLC31A1	DPY19L4	0.57478901	4.86E-17
SLC31A1	HS2ST1	0.57489204	4.79E-17
SLC31A1	MED28	0.57518822	4.58E-17
SLC31A1	PCYOX1	0.57519823	4.57E-17
SLC31A1	PAIP1	0.57532037	4.48E-17
SLC31A1	B3GNT2	0.5753718	4.45E-17
SLC31A1	CSTF2	0.57547311	4.38E-17
SLC31A1	MINPP1	0.57553984	4.34E-17
SLC31A1	SEC23A	0.57584095	4.14E-17
SLC31A1	NBR1	0.57588661	4.11E-17
SLC31A1	TEAD1	0.57616885	3.94E-17
SLC31A1	TTC39B	0.57624871	3.89E-17
SLC31A1	BBX	0.57631764	3.85E-17
SLC31A1	ENOPH1	0.57650595	3.74E-17
SLC31A1	METTL2A	0.57655868	3.71E-17
SLC31A1	SIN3A	0.57659425	3.69E-17
SLC31A1	CISD2	0.57659951	3.69E-17
SLC31A1	NOCT	0.57662299	3.67E-17
SLC31A1	FKBP9	0.57669887	3.63E-17
SLC31A1	TBK1	0.57684515	3.55E-17
SLC31A1	BRAP	0.5769565	3.49E-17
SLC31A1	SPOP	0.57700834	3.46E-17
SLC31A1	SOCS6	0.57754458	3.19E-17
SLC31A1	TAOK1	0.57758627	3.17E-17
SLC31A1	NXT2	0.5775879	3.17E-17
SLC31A1	IMPA1	0.57778955	3.07E-17
SLC31A1	C14orf119	0.57783142	3.05E-17
SLC31A1	SZRD1	0.5778353	3.05E-17
SLC31A1	YWHAH	0.57794946	2.99E-17
SLC31A1	TOB2	0.5779525	2.99E-17
SLC31A1	PIK3CA	0.57795317	2.99E-17
SLC31A1	DOP1B	0.57826843	2.85E-17
SLC31A1	RBBP5	0.57834337	2.82E-17
SLC31A1	RAB5C	0.57843804	2.78E-17
SLC31A1	TMCO1	0.57856884	2.72E-17
SLC31A1	ELK1	0.57859044	2.71E-17
SLC31A1	MED20	0.57865869	2.68E-17
SLC31A1	NLN	0.57875943	2.64E-17
SLC31A1	VPS41	0.57879653	2.63E-17
SLC31A1	PPP1R12A	0.57883929	2.61E-17
SLC31A1	ABCF1	0.57885189	2.6E-17
SLC31A1	TLR4	0.57898337	2.55E-17
SLC31A1	ABI2	0.57922659	2.46E-17

SLC31A1	CCSER2	0.57974586	2.27E-17
SLC31A1	FASTKD5	0.57987919	2.22E-17
SLC31A1	MTFMT	0.58004005	2.17E-17
SLC31A1	WASF2	0.58006852	2.16E-17
SLC31A1	PTPRA	0.58032124	2.07E-17
SLC31A1	ZNF410	0.5804277	2.04E-17
SLC31A1	CWC22	0.58047215	2.02E-17
SLC31A1	NUP155	0.58053329	2.01E-17
SLC31A1	TMCC1	0.5805369	2E-17
SLC31A1	MED17	0.58056281	2E-17
SLC31A1	HAT1	0.58062978	1.98E-17
SLC31A1	OSGIN2	0.58065548	1.97E-17
SLC31A1	ARMT1	0.58071294	1.95E-17
SLC31A1	ZBTB38	0.58081397	1.92E-17
SLC31A1	NOLC1	0.58085141	1.91E-17
SLC31A1	TRUB1	0.58098802	1.87E-17
SLC31A1	GNG12	0.58107834	1.84E-17
SLC31A1	UBE2K	0.5811883	1.81E-17
SLC31A1	RNF111	0.58122342	1.8E-17
SLC31A1	DDX6	0.58123381	1.8E-17
SLC31A1	CRTC3	0.58140725	1.75E-17
SLC31A1	KLHL20	0.581411	1.75E-17
SLC31A1	ZBTB41	0.58143523	1.74E-17
SLC31A1	WDR3	0.58150373	1.72E-17
SLC31A1	DEGS1	0.58181831	1.64E-17
SLC31A1	STARD3NL	0.58192565	1.61E-17
SLC31A1	NIPA2	0.58212254	1.56E-17
SLC31A1	PPP3R1	0.58247669	1.48E-17
SLC31A1	NF1	0.5824912	1.48E-17
SLC31A1	MED8	0.582742	1.42E-17
SLC31A1	LEPROT	0.58279516	1.41E-17
SLC31A1	RPA1	0.58289077	1.39E-17
SLC31A1	SERBP1	0.58291825	1.38E-17
SLC31A1	HPS5	0.58295932	1.37E-17
SLC31A1	GMCL1	0.58297576	1.37E-17
SLC31A1	NCL	0.58298242	1.37E-17
SLC31A1	ADIPOR2	0.58301958	1.36E-17
SLC31A1	KRAS	0.58301993	1.36E-17
SLC31A1	PPP2R5D	0.58302568	1.36E-17
SLC31A1	EMC3	0.58303027	1.36E-17
SLC31A1	GNPTAB	0.5830931	1.34E-17
SLC31A1	SYS1	0.58310735	1.34E-17
SLC31A1	CCNG2	0.58321156	1.32E-17
SLC31A1	ZNF720	0.58321573	1.32E-17
SLC31A1	ITPRIPL2	0.58334912	1.29E-17
SLC31A1	DNAJC14	0.58346441	1.27E-17
SLC31A1	GNPDA2	0.58346927	1.27E-17
SLC31A1	AKAP11	0.58348158	1.26E-17
SLC31A1	LYRM2	0.5836097	1.24E-17
SLC31A1	SLBP	0.58373647	1.21E-17
SLC31A1	AHCYL1	0.58389731	1.18E-17
SLC31A1	CTCF	0.58395338	1.17E-17
SLC31A1	PPIL4	0.58418581	1.13E-17
SLC31A1	STRN	0.58419602	1.13E-17
SLC31A1	PSMD7	0.58429216	1.11E-17
SLC31A1	NCOA3	0.5843409	1.1E-17
SLC31A1	BMP2K	0.58435114	1.1E-17
SLC31A1	IARS2	0.58435999	1.1E-17

SLC31A1	PDZD8	0.58482551	1.02E-17
SLC31A1	XYLT1	0.58484152	1.02E-17
SLC31A1	SLC25A13	0.58502234	9.91E-18
SLC31A1	KLHL2	0.58506948	9.84E-18
SLC31A1	ITFG1	0.58534049	9.42E-18
SLC31A1	ME1	0.58542677	9.3E-18
SLC31A1	TAF5L	0.58560333	9.04E-18
SLC31A1	ZNF623	0.58561069	9.03E-18
SLC31A1	ANXA7	0.58578725	8.78E-18
SLC31A1	HERC3	0.58594902	8.56E-18
SLC31A1	TRIM14	0.58605962	8.41E-18
SLC31A1	CDK12	0.58606731	8.4E-18
SLC31A1	GALNT7	0.58616186	8.27E-18
SLC31A1	ZC3H15	0.58618863	8.24E-18
SLC31A1	LEO1	0.58620384	8.22E-18
SLC31A1	ACSL4	0.5862973	8.1E-18
SLC31A1	XPR1	0.58631727	8.07E-18
SLC31A1	ORC4	0.58633517	8.05E-18
SLC31A1	NDFIP2	0.58639315	7.97E-18
SLC31A1	LYPLA1	0.58664341	7.66E-18
SLC31A1	MRPL50	0.58667851	7.62E-18
SLC31A1	PARN	0.5867552	7.53E-18
SLC31A1	RIN2	0.58705065	7.18E-18
SLC31A1	SPAST	0.58726284	6.94E-18
SLC31A1	CAST	0.58733928	6.86E-18
SLC31A1	KIAA1671	0.58740244	6.79E-18
SLC31A1	CTTNBP2NL	0.58741199	6.78E-18
SLC31A1	URM1	0.58741276	6.78E-18
SLC31A1	MED1	0.58745464	6.73E-18
SLC31A1	MRPL3	0.5874979	6.69E-18
SLC31A1	DCK	0.58753058	6.65E-18
SLC31A1	ATP13A3	0.587614	6.57E-18
SLC31A1	SMARCE1	0.58766293	6.51E-18
SLC31A1	TULP3	0.58769524	6.48E-18
SLC31A1	RPE	0.58785052	6.32E-18
SLC31A1	SLC9A6	0.5882531	5.93E-18
SLC31A1	PPP4R2	0.58852455	5.68E-18
SLC31A1	ZNF468	0.58873718	5.49E-18
SLC31A1	NCAPG2	0.58880004	5.43E-18
SLC31A1	HTATSF1	0.58913925	5.14E-18
SLC31A1	FYTTD1	0.58918017	5.11E-18
SLC31A1	CLIP1	0.58931771	5E-18
SLC31A1	GFM1	0.58939951	4.93E-18
SLC31A1	VPS37A	0.58947919	4.87E-18
SLC31A1	ABI1	0.58964192	4.74E-18
SLC31A1	BRI3BP	0.58970417	4.7E-18
SLC31A1	STARD7	0.58974305	4.67E-18
SLC31A1	NDEL1	0.58982682	4.61E-18
SLC31A1	AGAP1	0.58989032	4.56E-18
SLC31A1	SELENOF	0.58991537	4.54E-18
SLC31A1	HNRNPU	0.58993648	4.53E-18
SLC31A1	PUM2	0.58999836	4.48E-18
SLC31A1	ENAH	0.5900737	4.43E-18
SLC31A1	CCNI	0.59028157	4.28E-18
SLC31A1	PJA2	0.59041942	4.19E-18
SLC31A1	COQ10B	0.59054358	4.1E-18
SLC31A1	TPP1	0.59066349	4.02E-18
SLC31A1	DNAJA1	0.59072156	3.99E-18

SLC31A1	RAB3GAP1	0.5907798	3.95E-18
SLC31A1	DDX19A	0.59078961	3.94E-18
SLC31A1	SLC30A5	0.59104442	3.78E-18
SLC31A1	TSN	0.59110139	3.75E-18
SLC31A1	ANKRD13C	0.59113036	3.73E-18
SLC31A1	BMT2	0.5912104	3.68E-18
SLC31A1	EFR3A	0.59121374	3.68E-18
SLC31A1	ISCA1	0.59186277	3.32E-18
SLC31A1	EIF2AK1	0.5919065	3.29E-18
SLC31A1	ATP11B	0.5920095	3.24E-18
SLC31A1	SDHC	0.59201798	3.23E-18
SLC31A1	SDE2	0.59216317	3.16E-18
SLC31A1	RPAP3	0.59224976	3.11E-18
SLC31A1	HSD17B12	0.5923548	3.06E-18
SLC31A1	INTS14	0.59239464	3.04E-18
SLC31A1	ABRAXAS2	0.59244299	3.02E-18
SLC31A1	UBN1	0.59252169	2.98E-18
SLC31A1	STAU2	0.5925223	2.98E-18
SLC31A1	AKT1	0.59260766	2.94E-18
SLC31A1	ARHGEF12	0.59274392	2.87E-18
SLC31A1	FAM210A	0.59287875	2.81E-18
SLC31A1	ACAP2	0.59298354	2.76E-18
SLC31A1	TERF1	0.5930585	2.73E-18
SLC31A1	AFAP1	0.59309485	2.71E-18
SLC31A1	RRM1	0.59322874	2.66E-18
SLC31A1	PDCD6IP	0.59326982	2.64E-18
SLC31A1	MFSD6	0.59334661	2.61E-18
SLC31A1	TTL	0.59354773	2.52E-18
SLC31A1	EXT2	0.59372874	2.45E-18
SLC31A1	TLK1	0.5938255	2.41E-18
SLC31A1	HNRNPLL	0.59390371	2.38E-18
SLC31A1	ARMH4	0.59404798	2.32E-18
SLC31A1	ATP6AP2	0.59429191	2.23E-18
SLC31A1	ZBTB33	0.59463235	2.11E-18
SLC31A1	DIAPH2	0.59473288	2.08E-18
SLC31A1	KAT7	0.59495232	2E-18
SLC31A1	RO60	0.59500432	1.99E-18
SLC31A1	OSTC	0.59513496	1.95E-18
SLC31A1	CAB39	0.59533277	1.88E-18
SLC31A1	PNO1	0.59541863	1.86E-18
SLC31A1	PARG	0.5954767	1.84E-18
SLC31A1	SMC3	0.59560809	1.8E-18
SLC31A1	NFE2L1	0.5956639	1.78E-18
SLC31A1	ZYG11B	0.59584861	1.73E-18
SLC31A1	RNF13	0.59599558	1.69E-18
SLC31A1	IRF2	0.5962357	1.62E-18
SLC31A1	C3orf38	0.59634611	1.59E-18
SLC31A1	LARP7	0.59655646	1.54E-18
SLC31A1	ATE1	0.59664372	1.52E-18
SLC31A1	TM9SF4	0.59687545	1.46E-18
SLC31A1	RACGAP1	0.59723187	1.38E-18
SLC31A1	BNIP3L	0.59723746	1.38E-18
SLC31A1	MIGA1	0.59752766	1.31E-18
SLC31A1	ATP5MF-PTCD1	0.59788021	1.24E-18
SLC31A1	WDR1	0.59790504	1.23E-18
SLC31A1	WDR82	0.59802133	1.21E-18
SLC31A1	ALDH9A1	0.5981715	1.18E-18
SLC31A1	DESI2	0.59817616	1.18E-18

SLC31A1	CREB1	0.59835455	1.14E-18
SLC31A1	TDP1	0.59836673	1.14E-18
SLC31A1	PCNP	0.59836965	1.14E-18
SLC31A1	TSNAX	0.59838203	1.14E-18
SLC31A1	RBFOX2	0.59852343	1.11E-18
SLC31A1	RFWD3	0.59857059	1.1E-18
SLC31A1	FBXO5	0.59875288	1.07E-18
SLC31A1	DAAM1	0.5988434	1.06E-18
SLC31A1	POLR2C	0.59905976	1.02E-18
SLC31A1	ATP6V1G1	0.59920002	9.95E-19
SLC31A1	SGMS2	0.59923015	9.9E-19
SLC31A1	FAM220A	0.59926294	9.85E-19
SLC31A1	TIPRL	0.5993878	9.64E-19
SLC31A1	HSPA13	0.59942696	9.58E-19
SLC31A1	PAK2	0.59943832	9.56E-19
SLC31A1	GNA13	0.59958288	9.34E-19
SLC31A1	UBA2	0.59966759	9.21E-19
SLC31A1	SMIM13	0.59969681	9.16E-19
SLC31A1	WNK1	0.59971175	9.14E-19
SLC31A1	TMEM168	0.59979581	9.01E-19
SLC31A1	SYNJ2BP	0.59984232	8.94E-19
SLC31A1	UBE2D2	0.59992053	8.83E-19
SLC31A1	TUBA1B	0.60000351	8.7E-19
SLC31A1	DOCK7	0.60006949	8.61E-19
SLC31A1	DNAJB6	0.60009312	8.58E-19
SLC31A1	VCPIP1	0.60009519	8.57E-19
SLC31A1	UBTD2	0.60018489	8.45E-19
SLC31A1	TMX1	0.60048976	8.03E-19
SLC31A1	RAE1	0.60064448	7.82E-19
SLC31A1	COPS2	0.60103552	7.33E-19
SLC31A1	ROCK2	0.60114282	7.2E-19
SLC31A1	GCC2	0.60114768	7.19E-19
SLC31A1	SPIN1	0.60123036	7.09E-19
SLC31A1	TM9SF3	0.60130433	7.01E-19
SLC31A1	GLOD4	0.60132717	6.98E-19
SLC31A1	GPN3	0.60137143	6.93E-19
SLC31A1	TMEM209	0.60148275	6.8E-19
SLC31A1	TMEM167A	0.60172033	6.53E-19
SLC31A1	ACBD5	0.60177368	6.48E-19
SLC31A1	MATR3	0.60183795	6.41E-19
SLC31A1	MID1	0.60197331	6.26E-19
SLC31A1	SYNJ2	0.60218294	6.05E-19
SLC31A1	SNX6	0.60234378	5.88E-19
SLC31A1	COMMD2	0.602357	5.87E-19
SLC31A1	DHDDS	0.60249505	5.74E-19
SLC31A1	SLC25A32	0.60260149	5.64E-19
SLC31A1	C1GALT1C1	0.60260562	5.63E-19
SLC31A1	NCOA2	0.60272697	5.52E-19
SLC31A1	DHX35	0.60287427	5.38E-19
SLC31A1	ARHGAP5	0.60306636	5.21E-19
SLC31A1	SPPL3	0.6031326	5.15E-19
SLC31A1	NUP58	0.60315133	5.14E-19
SLC31A1	SLC39A9	0.60315582	5.13E-19
SLC31A1	VBP1	0.60343352	4.9E-19
SLC31A1	SPTSSA	0.60349975	4.84E-19
SLC31A1	FRYL	0.60376081	4.64E-19
SLC31A1	KIAA0232	0.60384029	4.57E-19
SLC31A1	AGFG1	0.60385968	4.56E-19

SLC31A1	SMU1	0.60393881	4.5E-19
SLC31A1	MIER1	0.60396015	4.48E-19
SLC31A1	TMEM127	0.60426758	4.25E-19
SLC31A1	INCENP	0.60430332	4.23E-19
SLC31A1	CAMSAP1	0.60436276	4.19E-19
SLC31A1	SH3D19	0.60473672	3.93E-19
SLC31A1	GLB1	0.60481729	3.88E-19
SLC31A1	MORF4L1	0.60497019	3.78E-19
SLC31A1	CALM1	0.60510751	3.69E-19
SLC31A1	CNOT9	0.60520568	3.63E-19
SLC31A1	RFK	0.60522609	3.62E-19
SLC31A1	PTPN9	0.60539929	3.51E-19
SLC31A1	CHUK	0.60540123	3.51E-19
SLC31A1	EIF4G3	0.60550384	3.45E-19
SLC31A1	CTDSPL2	0.60565576	3.36E-19
SLC31A1	TWF1	0.60572104	3.33E-19
SLC31A1	STAG2	0.60591489	3.22E-19
SLC31A1	PDLIM5	0.60625286	3.04E-19
SLC31A1	TOR1AIP1	0.60636795	2.98E-19
SLC31A1	SMIM15	0.60638547	2.97E-19
SLC31A1	SP2	0.60639286	2.97E-19
SLC31A1	HIPK1	0.60650239	2.91E-19
SLC31A1	PPIG	0.60652253	2.9E-19
SLC31A1	RSPH3	0.60654806	2.89E-19
SLC31A1	RPL7L1	0.60661656	2.86E-19
SLC31A1	SOCS4	0.60691643	2.71E-19
SLC31A1	PRKAR1A	0.60694236	2.7E-19
SLC31A1	FBXL5	0.60695054	2.7E-19
SLC31A1	DOLK	0.60704836	2.65E-19
SLC31A1	SAP130	0.60746394	2.47E-19
SLC31A1	PRELID3B	0.60757394	2.42E-19
SLC31A1	ZNF639	0.60759812	2.41E-19
SLC31A1	TGFBR1	0.60760102	2.41E-19
SLC31A1	CDC23	0.60772141	2.36E-19
SLC31A1	ALS2	0.60777083	2.34E-19
SLC31A1	RPRD1B	0.60785222	2.31E-19
SLC31A1	UBFD1	0.60823247	2.17E-19
SLC31A1	LZIC	0.60830521	2.14E-19
SLC31A1	FRS2	0.60839719	2.11E-19
SLC31A1	RFFL	0.60848398	2.07E-19
SLC31A1	RDH11	0.60860049	2.03E-19
SLC31A1	PEX26	0.6086337	2.02E-19
SLC31A1	FBXO45	0.60878729	1.97E-19
SLC31A1	FEM1C	0.60883043	1.95E-19
SLC31A1	TCTN3	0.60892041	1.92E-19
SLC31A1	NCKAP1	0.60893194	1.92E-19
SLC31A1	CPSF6	0.60893545	1.92E-19
SLC31A1	ADAR	0.60900126	1.9E-19
SLC31A1	CSDE1	0.60910004	1.87E-19
SLC31A1	JAK1	0.60913621	1.85E-19
SLC31A1	ZNF609	0.60917147	1.84E-19
SLC31A1	ADAM10	0.60940127	1.77E-19
SLC31A1	RNASEH1	0.60953959	1.73E-19
SLC31A1	NRBF2	0.60962754	1.7E-19
SLC31A1	CNOT8	0.60971071	1.68E-19
SLC31A1	MKRN1	0.61006521	1.58E-19
SLC31A1	BAG5	0.61006535	1.58E-19
SLC31A1	VAPA	0.61020555	1.54E-19

SLC31A1	UBE2A	0.61027248	1.52E-19
SLC31A1	BBS7	0.61027475	1.52E-19
SLC31A1	NCOA4	0.61057662	1.45E-19
SLC31A1	ZFAND5	0.61058551	1.44E-19
SLC31A1	PEX2	0.61065285	1.43E-19
SLC31A1	STAT3	0.61083151	1.38E-19
SLC31A1	RAB5A	0.61103979	1.34E-19
SLC31A1	UTP11	0.61112093	1.32E-19
SLC31A1	STT3B	0.61117396	1.3E-19
SLC31A1	SDHD	0.61125658	1.29E-19
SLC31A1	FBXO38	0.61128917	1.28E-19
SLC31A1	SETX	0.61146132	1.24E-19
SLC31A1	SOGA1	0.61146703	1.24E-19
SLC31A1	SMARCC1	0.61162307	1.21E-19
SLC31A1	SRFBP1	0.61169997	1.19E-19
SLC31A1	RBBP9	0.61182154	1.17E-19
SLC31A1	TMED10	0.61208902	1.11E-19
SLC31A1	KDM1B	0.612153	1.1E-19
SLC31A1	CENPL	0.61224062	1.08E-19
SLC31A1	DDX21	0.61254034	1.03E-19
SLC31A1	PTP4A1	0.61300373	9.49E-20
SLC31A1	TMEM87B	0.61326798	9.06E-20
SLC31A1	TRUB2	0.61340733	8.84E-20
SLC31A1	RAB6A	0.61385507	8.18E-20
SLC31A1	MGAT5	0.61395803	8.03E-20
SLC31A1	PIGK	0.6140835	7.86E-20
SLC31A1	ASB7	0.61435619	7.49E-20
SLC31A1	SCO1	0.61441229	7.42E-20
SLC31A1	GTF2A1	0.61457692	7.21E-20
SLC31A1	FASTKD2	0.61508215	6.6E-20
SLC31A1	BRCC3	0.61540737	6.23E-20
SLC31A1	UBE3A	0.61548932	6.14E-20
SLC31A1	GNS	0.61556195	6.06E-20
SLC31A1	YWHAG	0.6158843	5.73E-20
SLC31A1	KDM4A	0.61606429	5.55E-20
SLC31A1	HMGN4	0.61611828	5.5E-20
SLC31A1	ERGIC1	0.61621909	5.4E-20
SLC31A1	ELAVL1	0.61662927	5.02E-20
SLC31A1	PSMD12	0.61664564	5.01E-20
SLC31A1	TRRAP	0.6168025	4.87E-20
SLC31A1	SBDS	0.61694626	4.75E-20
SLC31A1	ARF3	0.61694687	4.75E-20
SLC31A1	ZWILCH	0.61696857	4.73E-20
SLC31A1	TRAK2	0.61697613	4.72E-20
SLC31A1	FOXN3	0.61725577	4.49E-20
SLC31A1	LACTB	0.61735132	4.42E-20
SLC31A1	FAM118B	0.61739158	4.39E-20
SLC31A1	MFSD1	0.61753887	4.27E-20
SLC31A1	DCTN5	0.61753934	4.27E-20
SLC31A1	CNOT6L	0.61814572	3.84E-20
SLC31A1	NRAS	0.61816572	3.82E-20
SLC31A1	EIF1AX	0.61826171	3.76E-20
SLC31A1	THRAP3	0.61829704	3.74E-20
SLC31A1	TRIP12	0.61844817	3.64E-20
SLC31A1	CCNK	0.61866658	3.5E-20
SLC31A1	UBQLN2	0.61869685	3.48E-20
SLC31A1	PMS2	0.61872944	3.46E-20
SLC31A1	CKAP4	0.61874182	3.45E-20

SLC31A1	SMG8	0.61903947	3.27E-20
SLC31A1	AP1AR	0.61923902	3.16E-20
SLC31A1	ACLY	0.61928671	3.13E-20
SLC31A1	STAU1	0.61941927	3.06E-20
SLC31A1	RNF6	0.61942473	3.06E-20
SLC31A1	COQ7	0.61949294	3.02E-20
SLC31A1	SMNDC1	0.61959406	2.97E-20
SLC31A1	PCYT1A	0.61966853	2.93E-20
SLC31A1	HSPA5	0.61971871	2.9E-20
SLC31A1	ANO10	0.6200136	2.75E-20
SLC31A1	ACTR3	0.62051287	2.52E-20
SLC31A1	ATP10D	0.62058344	2.48E-20
SLC31A1	APP	0.62073222	2.42E-20
SLC31A1	TOPORS	0.62074153	2.42E-20
SLC31A1	ZC3H13	0.62093741	2.33E-20
SLC31A1	GSTCD	0.6211838	2.23E-20
SLC31A1	NT5DC1	0.62137381	2.16E-20
SLC31A1	PPP4R3B	0.62141694	2.14E-20
SLC31A1	PUM1	0.62147568	2.12E-20
SLC31A1	HEATR5A	0.62161421	2.06E-20
SLC31A1	DLG1	0.62162733	2.06E-20
SLC31A1	ZFR	0.6217453	2.02E-20
SLC31A1	PURB	0.62178173	2E-20
SLC31A1	POLR2D	0.62180838	1.99E-20
SLC31A1	GALNT1	0.62182956	1.99E-20
SLC31A1	DAZAP2	0.62203421	1.91E-20
SLC31A1	LSM14A	0.62214554	1.88E-20
SLC31A1	CFAP97	0.62222494	1.85E-20
SLC31A1	AGPS	0.62231494	1.82E-20
SLC31A1	MCMBP	0.62234373	1.81E-20
SLC31A1	PPP1R8	0.62242141	1.79E-20
SLC31A1	CNOT6	0.62246237	1.77E-20
SLC31A1	TAB2	0.62250681	1.76E-20
SLC31A1	TDRD7	0.62265785	1.71E-20
SLC31A1	ATP6V1A	0.62303513	1.6E-20
SLC31A1	MAPKAP1	0.62307421	1.59E-20
SLC31A1	ARPP19	0.62340649	1.49E-20
SLC31A1	ARCN1	0.62352728	1.46E-20
SLC31A1	KPNA4	0.62357054	1.45E-20
SLC31A1	TMBIM6	0.62357322	1.45E-20
SLC31A1	RTF1	0.62373023	1.41E-20
SLC31A1	CUL2	0.62390584	1.37E-20
SLC31A1	PSMD10	0.62397426	1.35E-20
SLC31A1	RAB1A	0.62407306	1.32E-20
SLC31A1	UBE2W	0.62446636	1.23E-20
SLC31A1	R3HDM1	0.62454022	1.22E-20
SLC31A1	CDC27	0.62482089	1.16E-20
SLC31A1	ZNF146	0.62490696	1.14E-20
SLC31A1	COPB1	0.62508366	1.1E-20
SLC31A1	HAUS2	0.62537449	1.05E-20
SLC31A1	PTDSS1	0.62537661	1.04E-20
SLC31A1	MAPRE2	0.62540755	1.04E-20
SLC31A1	DDX18	0.62545111	1.03E-20
SLC31A1	ZNF200	0.62555605	1.01E-20
SLC31A1	SLC35B4	0.62581355	9.65E-21
SLC31A1	AGGF1	0.62595884	9.39E-21
SLC31A1	TGOLN2	0.62610025	9.15E-21
SLC31A1	COA7	0.62612126	9.12E-21

SLC31A1	CTBS	0.62621365	8.97E-21
SLC31A1	VPS35	0.62627439	8.87E-21
SLC31A1	TTC26	0.62629788	8.83E-21
SLC31A1	SFXN1	0.62635323	8.74E-21
SLC31A1	GSPT1	0.6264386	8.61E-21
SLC31A1	METTL14	0.62661336	8.33E-21
SLC31A1	ARF4	0.6266242	8.32E-21
SLC31A1	FNIP2	0.62698564	7.78E-21
SLC31A1	LARS2	0.62700593	7.76E-21
SLC31A1	PANK3	0.62706134	7.68E-21
SLC31A1	TXNRD1	0.62708172	7.65E-21
SLC31A1	BLOC1S6	0.62727379	7.38E-21
SLC31A1	RB1	0.62731794	7.32E-21
SLC31A1	RRN3	0.627567	7E-21
SLC31A1	MFSD14A	0.6277414	6.78E-21
SLC31A1	IFNAR1	0.62778875	6.72E-21
SLC31A1	LRRC57	0.62819395	6.23E-21
SLC31A1	MRPL19	0.6282096	6.22E-21
SLC31A1	SUZ12	0.62824102	6.18E-21
SLC31A1	DNAJB14	0.62839226	6.01E-21
SLC31A1	NAA50	0.62840639	6E-21
SLC31A1	AFG3L2	0.62855187	5.84E-21
SLC31A1	MYO1D	0.6285819	5.8E-21
SLC31A1	RTCB	0.62900711	5.37E-21
SLC31A1	YWHAB	0.62916815	5.21E-21
SLC31A1	SLC35A3	0.62924272	5.14E-21
SLC31A1	KDM5A	0.62933099	5.05E-21
SLC31A1	C16orf72	0.6293566	5.03E-21
SLC31A1	LCORL	0.62937395	5.01E-21
SLC31A1	PRKAG1	0.62964618	4.77E-21
SLC31A1	EAF1	0.62974659	4.68E-21
SLC31A1	STX7	0.63015316	4.34E-21
SLC31A1	ZNF148	0.63038483	4.16E-21
SLC31A1	PEAK1	0.63067171	3.94E-21
SLC31A1	CASP3	0.63122845	3.55E-21
SLC31A1	TIGAR	0.63156209	3.34E-21
SLC31A1	IFT57	0.631669	3.27E-21
SLC31A1	TMEM38B	0.63177827	3.21E-21
SLC31A1	NUB1	0.63187485	3.15E-21
SLC31A1	SLC35A5	0.63226702	2.93E-21
SLC31A1	ACTR2	0.63230277	2.91E-21
SLC31A1	SLC25A40	0.63240893	2.85E-21
SLC31A1	TAF13	0.63264827	2.73E-21
SLC31A1	SYAP1	0.63287175	2.61E-21
SLC31A1	TCAF1	0.63292717	2.59E-21
SLC31A1	APOL6	0.63303905	2.53E-21
SLC31A1	UTP14C	0.63304634	2.53E-21
SLC31A1	SF3A3	0.63313095	2.49E-21
SLC31A1	C11orf58	0.63323831	2.44E-21
SLC31A1	RNF11	0.63325041	2.44E-21
SLC31A1	RAD21	0.63331526	2.41E-21
SLC31A1	HNRNPR	0.63339866	2.37E-21
SLC31A1	PDE12	0.63365064	2.26E-21
SLC31A1	RNF138	0.63367472	2.25E-21
SLC31A1	CRCP	0.63400199	2.11E-21
SLC31A1	SPOPL	0.63405985	2.09E-21
SLC31A1	METTL2B	0.6341328	2.06E-21
SLC31A1	ESYT2	0.63425655	2.02E-21

SLC31A1	TFAM	0.63431408	1.99E-21
SLC31A1	C9orf78	0.63435507	1.98E-21
SLC31A1	SNX16	0.63471597	1.85E-21
SLC31A1	FBXO8	0.63488097	1.79E-21
SLC31A1	NFATC3	0.63497796	1.76E-21
SLC31A1	CTR9	0.63500157	1.75E-21
SLC31A1	TM9SF2	0.63503007	1.74E-21
SLC31A1	ZDHHC9	0.63535291	1.64E-21
SLC31A1	GRPEL2	0.63546672	1.6E-21
SLC31A1	SYNCRIP	0.6358382	1.5E-21
SLC31A1	EIF2S1	0.63596386	1.46E-21
SLC31A1	TCF12	0.63627946	1.38E-21
SLC31A1	NPTN	0.63664318	1.28E-21
SLC31A1	ERC1	0.63685553	1.23E-21
SLC31A1	NFKB1	0.63694702	1.21E-21
SLC31A1	CSNK1G3	0.63719908	1.15E-21
SLC31A1	ADH5	0.6372733	1.14E-21
SLC31A1	NEDD1	0.63731537	1.13E-21
SLC31A1	DCUN1D1	0.63806212	9.8E-22
SLC31A1	RHBDD1	0.63813743	9.66E-22
SLC31A1	CDYL	0.63813763	9.66E-22
SLC31A1	GALK2	0.63836017	9.25E-22
SLC31A1	FAM91A1	0.63865544	8.75E-22
SLC31A1	ESF1	0.63870874	8.66E-22
SLC31A1	ERLIN1	0.63883457	8.45E-22
SLC31A1	GLCE	0.63884009	8.44E-22
SLC31A1	MAPK1IP1L	0.63887325	8.39E-22
SLC31A1	GRSF1	0.63935907	7.64E-22
SLC31A1	SELENOT	0.63956642	7.35E-22
SLC31A1	GHITM	0.63958685	7.32E-22
SLC31A1	NUDCD3	0.64040192	6.25E-22
SLC31A1	PSME3	0.64051418	6.12E-22
SLC31A1	IGF2R	0.64071917	5.88E-22
SLC31A1	CSNK2A1	0.64087558	5.71E-22
SLC31A1	GTF2H3	0.64089386	5.69E-22
SLC31A1	LRP10	0.64089955	5.68E-22
SLC31A1	KPNA3	0.64148343	5.08E-22
SLC31A1	PIGX	0.64163	4.93E-22
SLC31A1	KIAA1191	0.64175687	4.82E-22
SLC31A1	LAMP2	0.64183344	4.74E-22
SLC31A1	RAB10	0.64189285	4.69E-22
SLC31A1	RNF41	0.6420186	4.58E-22
SLC31A1	TYW1	0.64258702	4.1E-22
SLC31A1	DIS3	0.6427166	4E-22
SLC31A1	TMEM263	0.64275929	3.96E-22
SLC31A1	ZNF184	0.64326483	3.59E-22
SLC31A1	RLIM	0.64358423	3.38E-22
SLC31A1	GANAB	0.64405515	3.08E-22
SLC31A1	ERAP1	0.64430525	2.93E-22
SLC31A1	IPO7	0.6444565	2.85E-22
SLC31A1	PHAX	0.64460978	2.76E-22
SLC31A1	PTPN11	0.64462302	2.76E-22
SLC31A1	TGS1	0.64472033	2.7E-22
SLC31A1	ADNP	0.64494913	2.59E-22
SLC31A1	RBBP4	0.64532742	2.4E-22
SLC31A1	AIMP1	0.64545228	2.34E-22
SLC31A1	MAP3K2	0.6454876	2.33E-22
SLC31A1	PRPF40A	0.64608588	2.07E-22

SLC31A1	ZNF322	0.64624278	2.01E-22
SLC31A1	NBN	0.64636557	1.96E-22
SLC31A1	MAPK14	0.64663914	1.86E-22
SLC31A1	CUL1	0.64742341	1.59E-22
SLC31A1	METTL9	0.64773356	1.49E-22
SLC31A1	CLCN3	0.64787418	1.45E-22
SLC31A1	SMARCA5	0.64792007	1.44E-22
SLC31A1	UEVLD	0.64796103	1.43E-22
SLC31A1	PPP2R5E	0.64802735	1.41E-22
SLC31A1	SMAD1	0.64820197	1.36E-22
SLC31A1	RYBP	0.64830222	1.34E-22
SLC31A1	HMGXB4	0.64862704	1.25E-22
SLC31A1	TRAM2	0.64917978	1.12E-22
SLC31A1	RP2	0.64930661	1.09E-22
SLC31A1	CERS6	0.64938483	1.08E-22
SLC31A1	MAGT1	0.6494724	1.06E-22
SLC31A1	FAM120AOS	0.64975475	1E-22
SLC31A1	AFF4	0.65001223	9.5E-23
SLC31A1	RAB22A	0.65016294	9.22E-23
SLC31A1	GOSR1	0.65046462	8.68E-23
SLC31A1	EFL1	0.65070833	8.27E-23
SLC31A1	AKIRIN1	0.65088067	7.99E-23
SLC31A1	ARMC1	0.65095575	7.87E-23
SLC31A1	LDAH	0.65134461	7.28E-23
SLC31A1	GMFB	0.65155319	6.98E-23
SLC31A1	ARL13B	0.65161709	6.89E-23
SLC31A1	SLC35A4	0.65178223	6.67E-23
SLC31A1	WAC	0.65190052	6.51E-23
SLC31A1	MED13	0.65201232	6.37E-23
SLC31A1	CRK	0.6523272	5.98E-23
SLC31A1	EVI5	0.65332846	4.88E-23
SLC31A1	MAPRE1	0.65360355	4.62E-23
SLC31A1	HNRNPH2	0.65400531	4.26E-23
SLC31A1	CLOCK	0.65416032	4.13E-23
SLC31A1	DYRK2	0.65486304	3.58E-23
SLC31A1	GLG1	0.65487927	3.57E-23
SLC31A1	SCAF11	0.65490972	3.54E-23
SLC31A1	NAA30	0.65502858	3.46E-23
SLC31A1	TRAF3IP1	0.6551487	3.38E-23
SLC31A1	ARIH1	0.65545119	3.17E-23
SLC31A1	TAX1BP1	0.65548527	3.15E-23
SLC31A1	GOLT1B	0.65572071	3E-23
SLC31A1	SCFD2	0.65577736	2.97E-23
SLC31A1	PTBP3	0.65579068	2.96E-23
SLC31A1	DNAJC21	0.65610077	2.78E-23
SLC31A1	ISG20L2	0.65610773	2.78E-23
SLC31A1	FAM98A	0.65625924	2.69E-23
SLC31A1	PGM2	0.65637901	2.63E-23
SLC31A1	ZFP91	0.65679907	2.41E-23
SLC31A1	FAF2	0.65699281	2.32E-23
SLC31A1	DBF4	0.65707239	2.28E-23
SLC31A1	EIF4G2	0.6575848	2.05E-23
SLC31A1	TOP1	0.65820452	1.81E-23
SLC31A1	GLE1	0.65846264	1.71E-23
SLC31A1	RBM7	0.65860682	1.66E-23
SLC31A1	TAF2	0.65873694	1.62E-23
SLC31A1	TNPO3	0.65910147	1.5E-23
SLC31A1	VAPB	0.65917396	1.48E-23

SLC31A1	USP1	0.65970626	1.32E-23
SLC31A1	MED14	0.66048962	1.13E-23
SLC31A1	KIF2A	0.66062326	1.09E-23
SLC31A1	ALG11	0.66082892	1.05E-23
SLC31A1	SDCBP	0.6608594	1.04E-23
SLC31A1	MPZL1	0.66087144	1.04E-23
SLC31A1	USP8	0.66097734	1.02E-23
SLC31A1	SSR1	0.66238624	7.58E-24
SLC31A1	YES1	0.66258469	7.27E-24
SLC31A1	ATP6V1C1	0.66292149	6.78E-24
SLC31A1	TRIQK	0.66333771	6.21E-24
SLC31A1	RNF20	0.663837	5.59E-24
SLC31A1	COQ2	0.66401638	5.39E-24
SLC31A1	MFAP3	0.66401687	5.38E-24
SLC31A1	SERINC3	0.66402421	5.38E-24
SLC31A1	FCF1	0.66416746	5.22E-24
SLC31A1	RAD1	0.66417255	5.21E-24
SLC31A1	MFSD14B	0.66446914	4.9E-24
SLC31A1	LNPK	0.66455148	4.81E-24
SLC31A1	BCL2L13	0.6647696	4.59E-24
SLC31A1	DDX3X	0.66513225	4.26E-24
SLC31A1	FAM102B	0.66530998	4.1E-24
SLC31A1	CCDC47	0.66555739	3.89E-24
SLC31A1	UGDH	0.6655583	3.89E-24
SLC31A1	BRD3	0.6656857	3.79E-24
SLC31A1	SETD7	0.66582347	3.68E-24
SLC31A1	ASXL2	0.66583194	3.67E-24
SLC31A1	ZNF562	0.66666952	3.07E-24
SLC31A1	TMEM106B	0.66675036	3.02E-24
SLC31A1	FKBP15	0.66727591	2.7E-24
SLC31A1	RABL3	0.66741918	2.62E-24
SLC31A1	WDR44	0.66753325	2.56E-24
SLC31A1	CSNK1A1	0.66758956	2.53E-24
SLC31A1	G3BP1	0.66766191	2.49E-24
SLC31A1	RSRC1	0.66787717	2.37E-24
SLC31A1	MMGT1	0.66802146	2.3E-24
SLC31A1	KPNA1	0.66875137	1.97E-24
SLC31A1	CALU	0.66895319	1.89E-24
SLC31A1	TMED5	0.66911813	1.82E-24
SLC31A1	VAMP7	0.66913107	1.82E-24
SLC31A1	EFCAB14	0.66913223	1.81E-24
SLC31A1	LRRCC1	0.66918944	1.79E-24
SLC31A1	CPNE3	0.66927011	1.76E-24
SLC31A1	ACSL3	0.66933816	1.74E-24
SLC31A1	POLE3	0.66935205	1.73E-24
SLC31A1	MBTPS2	0.66936946	1.72E-24
SLC31A1	OGFOD1	0.66963142	1.63E-24
SLC31A1	TMED7	0.67002887	1.5E-24
SLC31A1	HBP1	0.67028522	1.42E-24
SLC31A1	RAB21	0.67110048	1.19E-24
SLC31A1	FEM1B	0.67115316	1.17E-24
SLC31A1	LPIN2	0.67116614	1.17E-24
SLC31A1	PITPNA	0.6712778	1.14E-24
SLC31A1	TVP23B	0.67149465	1.09E-24
SLC31A1	RAB27A	0.67164898	1.05E-24
SLC31A1	LYN	0.67197073	9.83E-25
SLC31A1	CEP41	0.67197535	9.82E-25
SLC31A1	CANX	0.67204526	9.68E-25

SLC31A1	CREB3L2	0.67225819	9.24E-25
SLC31A1	PPM1A	0.67268004	8.43E-25
SLC31A1	ABL1	0.67272714	8.34E-25
SLC31A1	C5orf51	0.67320643	7.52E-25
SLC31A1	CDC5L	0.67322546	7.48E-25
SLC31A1	PNPLA8	0.67336535	7.26E-25
SLC31A1	MEGF9	0.6736531	6.82E-25
SLC31A1	SCARB2	0.67385626	6.52E-25
SLC31A1	CNIH1	0.67409456	6.19E-25
SLC31A1	LUZP1	0.67413822	6.13E-25
SLC31A1	SF3A1	0.67465458	5.47E-25
SLC31A1	UBE2D3	0.67499204	5.08E-25
SLC31A1	CMIP	0.67511952	4.94E-25
SLC31A1	TRIM32	0.6751205	4.94E-25
SLC31A1	VPS4B	0.67514291	4.92E-25
SLC31A1	GNAQ	0.67529641	4.75E-25
SLC31A1	TOR1A	0.67558297	4.46E-25
SLC31A1	PPP2CA	0.67618571	3.91E-25
SLC31A1	SPTY2D1	0.67679052	3.42E-25
SLC31A1	ZFP64	0.67702791	3.24E-25
SLC31A1	GOLPH3	0.6772564	3.08E-25
SLC31A1	RAB8A	0.67771109	2.79E-25
SLC31A1	ACER3	0.67784867	2.7E-25
SLC31A1	TTC33	0.67791168	2.67E-25
SLC31A1	SLC25A46	0.67792142	2.66E-25
SLC31A1	DEK	0.67806086	2.58E-25
SLC31A1	WASL	0.67811124	2.55E-25
SLC31A1	ETF1	0.67860179	2.29E-25
SLC31A1	RNF141	0.67873413	2.22E-25
SLC31A1	GNL3L	0.67977987	1.76E-25
SLC31A1	OTULIN	0.6799804	1.68E-25
SLC31A1	LIN54	0.68005307	1.65E-25
SLC31A1	ACBD3	0.68030084	1.56E-25
SLC31A1	CAND1	0.68039356	1.53E-25
SLC31A1	EXOC5	0.68080388	1.4E-25
SLC31A1	SLC44A1	0.68164502	1.16E-25
SLC31A1	RC3H2	0.68195446	1.08E-25
SLC31A1	DLAT	0.68228448	1E-25
SLC31A1	ATF1	0.68238693	9.78E-26
SLC31A1	MTM1	0.68251126	9.51E-26
SLC31A1	SET	0.68253555	9.46E-26
SLC31A1	GFPT1	0.68259023	9.34E-26
SLC31A1	RNF14	0.6830009	8.51E-26
SLC31A1	ZFAND3	0.6832369	8.07E-26
SLC31A1	UBE3C	0.68359154	7.44E-26
SLC31A1	ATG5	0.68414872	6.56E-26
SLC31A1	CYB5R4	0.6842793	6.36E-26
SLC31A1	GK	0.68454532	5.99E-26
SLC31A1	USP9X	0.68473834	5.73E-26
SLC31A1	KDSR	0.685018	5.38E-26
SLC31A1	SENP1	0.68558441	4.72E-26
SLC31A1	EXT1	0.68571364	4.58E-26
SLC31A1	KPNA6	0.68594431	4.35E-26
SLC31A1	DR1	0.6866898	3.66E-26
SLC31A1	BCAP29	0.68695192	3.45E-26
SLC31A1	CRKL	0.6874831	3.05E-26
SLC31A1	PI4K2B	0.68749266	3.05E-26
SLC31A1	VTA1	0.68776485	2.86E-26

SLC31A1	SNX4	0.6878474	2.81E-26
SLC31A1	MORF4L2	0.68853887	2.39E-26
SLC31A1	XRN2	0.68912858	2.08E-26
SLC31A1	ATP2A2	0.68914555	2.08E-26
SLC31A1	ZNF398	0.68949691	1.91E-26
SLC31A1	RNF4	0.69029894	1.59E-26
SLC31A1	OPA1	0.69050534	1.51E-26
SLC31A1	TMEM167B	0.69109151	1.32E-26
SLC31A1	SMAD2	0.69134034	1.24E-26
SLC31A1	GDE1	0.69162834	1.16E-26
SLC31A1	ZBTB6	0.69182042	1.11E-26
SLC31A1	TMEM33	0.69199353	1.07E-26
SLC31A1	OGDH	0.69222478	1.01E-26
SLC31A1	PLEKHB2	0.69240358	9.69E-27
SLC31A1	KIF5B	0.69292711	8.56E-27
SLC31A1	TEX10	0.69376346	7.03E-27
SLC31A1	HCCS	0.69433502	6.14E-27
SLC31A1	VKORC1L1	0.69444301	5.98E-27
SLC31A1	TMEM248	0.6950516	5.18E-27
SLC31A1	SLC30A6	0.69512774	5.08E-27
SLC31A1	SMAD5	0.69616608	3.97E-27
SLC31A1	SAR1B	0.69659059	3.58E-27
SLC31A1	SLC17A5	0.69660536	3.57E-27
SLC31A1	BAZ1B	0.69674838	3.45E-27
SLC31A1	ODF2	0.69789866	2.62E-27
SLC31A1	RBM27	0.69808092	2.51E-27
SLC31A1	LMBR1	0.6982103	2.43E-27
SLC31A1	CKAP5	0.69926464	1.88E-27
SLC31A1	PAQR3	0.69994254	1.6E-27
SLC31A1	KDELR2	0.70015225	1.52E-27
SLC31A1	BTBD1	0.70032006	1.46E-27
SLC31A1	PDS5A	0.701492	1.09E-27
SLC31A1	PGGT1B	0.70258395	8.38E-28
SLC31A1	PDHX	0.70263509	8.27E-28
SLC31A1	OAT	0.70312144	7.34E-28
SLC31A1	DCAF12	0.70363075	6.47E-28
SLC31A1	SRPK2	0.70473566	4.93E-28
SLC31A1	MAPK1	0.70527087	4.31E-28
SLC31A1	TOX4	0.70577954	3.8E-28
SLC31A1	RBM12	0.70587158	3.71E-28
SLC31A1	DCAF7	0.70614661	3.47E-28
SLC31A1	RELL1	0.7062743	3.36E-28
SLC31A1	NAA15	0.70628485	3.35E-28
SLC31A1	ITCH	0.70675007	2.98E-28
SLC31A1	USP14	0.70735711	2.56E-28
SLC31A1	YME1L1	0.70756675	2.43E-28
SLC31A1	RPS6KA3	0.70786235	2.26E-28
SLC31A1	KIF3B	0.70831362	2.02E-28
SLC31A1	ZMPSTE24	0.7093794	1.54E-28
SLC31A1	MAPK6	0.70940518	1.53E-28
SLC31A1	HUS1	0.70945562	1.51E-28
SLC31A1	BZW1	0.71010076	1.28E-28
SLC31A1	IDH3A	0.71026843	1.23E-28
SLC31A1	GOLIM4	0.7109484	1.04E-28
SLC31A1	PLEKHA3	0.7109621	1.03E-28
SLC31A1	NDUFS1	0.71123967	9.61E-29
SLC31A1	ARL5A	0.71156451	8.85E-29
SLC31A1	ENOX2	0.71182623	8.28E-29

SLC31A1	YTHDF3	0.71266122	6.69E-29
SLC31A1	RAB18	0.71313717	5.92E-29
SLC31A1	RAP2A	0.71335484	5.6E-29
SLC31A1	HNRNPK	0.71456904	4.09E-29
SLC31A1	CPSF2	0.7157115	3.04E-29
SLC31A1	COPA	0.71600948	2.82E-29
SLC31A1	SEC23IP	0.7161445	2.72E-29
SLC31A1	AP5M1	0.71747451	1.92E-29
SLC31A1	SLAIN2	0.7179042	1.72E-29
SLC31A1	MTDH	0.71816115	1.61E-29
SLC31A1	MTPN	0.71854331	1.45E-29
SLC31A1	CHCHD3	0.71913993	1.24E-29
SLC31A1	FAM120A	0.71950872	1.13E-29
SLC31A1	RAB2A	0.71972386	1.06E-29
SLC31A1	SURF4	0.72049326	8.68E-30
SLC31A1	EIF4E	0.720908	7.78E-30
SLC31A1	PSMC2	0.7209646	7.66E-30
SLC31A1	DERL1	0.72183676	6.08E-30
SLC31A1	KIF16B	0.72205498	5.73E-30
SLC31A1	POFUT1	0.72234655	5.3E-30
SLC31A1	CYCS	0.72270963	4.81E-30
SLC31A1	TOR1B	0.72467415	2.84E-30
SLC31A1	SMC1A	0.72524686	2.43E-30
SLC31A1	DNAJC10	0.7285195	9.96E-31
SLC31A1	API5	0.72954351	7.52E-31
SLC31A1	CAMK2D	0.7296982	7.2E-31
SLC31A1	DLD	0.7343352	1.98E-31
SLC31A1	SPTLC1	0.73453121	1.87E-31
SLC31A1	YIPF5	0.73497373	1.65E-31
SLC31A1	TNPO1	0.73499154	1.64E-31
SLC31A1	IL13RA1	0.73503538	1.62E-31
SLC31A1	USP38	0.73622492	1.16E-31
SLC31A1	FAM114A1	0.73655398	1.05E-31
SLC31A1	RAB14	0.73673573	1E-31
SLC31A1	SMC2	0.73775777	7.47E-32
SLC31A1	SLC30A9	0.73777276	7.44E-32
SLC31A1	GCC1	0.73970391	4.27E-32
SLC31A1	YIPF6	0.74077338	3.13E-32
SLC31A1	ARL1	0.74228741	2.02E-32
SLC31A1	GORASP2	0.74240626	1.95E-32
SLC31A1	CAPZA2	0.74477777	9.7E-33
SLC31A1	C9orf64	0.74918884	2.6E-33
SLC31A1	PRRC1	0.75126317	1.38E-33
SLC31A1	PDCL	0.7527718	8.72E-34
SLC31A1	ABHD17B	0.75508515	4.27E-34
SLC31A1	GTF3C4	0.75833779	1.54E-34
SLC31A1	FBXW2	0.76242707	4.19E-35
SLC31A1	UBQLN1	0.77016874	3.31E-36
SLC31A1	G3BP2	0.77849023	1.93E-37
SLC31A1	GPR107	0.7789497	1.64E-37
SLC31A1	INIP	0.7808916	8.29E-38
SLC31A1	PRPF4	0.78626936	1.21E-38
SLC31A1	RAD23B	0.78630454	1.19E-38
SLC31A1	RBM18	0.79281369	1.07E-39
SLC31A1	SLC31A1	1	0
