# Supplementary material for: A Novel Cuproptosis-Associated Gene Signature to Predict Prognosis in Patients with Pancreatic Cancer
Source: Biomed Res Int. 2023 Jan 18;2023:3419401. doi: 10.1155/2023/3419401 (PMC9876676; doi:10.1155/2023/3419401)
Supplement: Supplementary Materials — Supplementary Table 1: 7978 DEGs between 178 tumor tissues and 171 normal tissues. Supplementary Table 2: 5252 cuproptosis-related genes based on 19 cuproptosis genes. Supplementary Table 3: 202 prognostic genes obtained by univariate Cox regression analysis. Supplementary Table 4: the risk scores and risk groups for all patients. Supplementary Table 5: 183 DEGs between high- and low-risk groups. Supplementary Table 6: risk scores for samples sourced from the GSE62452 and GSE28735 datasets. [file 3419401.f1.zip › 3419401.f3.pdf]

id	HR	HR.95L	HR.95H	pvalue
DLGAP5	2.345978	1.498438	3.672898	0.000193
KLHL29	0.429023	0.269998	0.681711	0.000342
FXR2	0.393342	0.235587	0.656734	0.00036
CD99L2	0.550326	0.395888	0.765011	0.00038
ANLN	1.889629	1.324288	2.696315	0.000451
ITGB6	1.4264	1.166243	1.74459	0.000547
MET	1.546378	1.205382	1.983839	0.000604
GABARAP	0.350614	0.191428	0.642175	0.000688
CDK6	2.230072	1.396034	3.562394	0.000791
MAGED2	0.525096	0.360146	0.765593	0.000813
CEP55	1.874647	1.297217	2.709107	0.000822
RAD51C	0.453263	0.284633	0.721795	0.000858
HDAC5	0.529261	0.363957	0.769643	0.000867
SMG6	0.518289	0.352011	0.763111	0.00087
RHOF	1.671321	1.233914	2.263783	0.000908
FRMD5	2.099067	1.352669	3.257325	0.000942
TBL1X	0.542894	0.377852	0.780024	0.000955
USP11	0.579688	0.419306	0.801417	0.000968
FAM111B	2.312905	1.404691	3.80833	0.000982
PROM2	1.402341	1.146516	1.715249	0.001
CKAP2L	2.202845	1.375329	3.528264	0.001016
CYB5D2	0.486552	0.316568	0.747811	0.001019
KIF20A	2.141912	1.357648	3.379217	0.001059
TPX2	1.781168	1.260646	2.516614	0.001063
NCAPG	2.195689	1.369913	3.519238	0.001084
SLC25A11	0.478875	0.307576	0.745574	0.001115
TGFA	1.57739	1.196231	2.079999	0.00124
ADORA2A	0.528935	0.359002	0.779305	0.001277
CKLF-CMT	1.836088	1.266893	2.661013	0.00133
BUB1B	2.305898	1.378642	3.856811	0.001455
ERBB2	1.728265	1.233916	2.420667	0.001459
KNL1	2.402643	1.394037	4.140991	0.001599
ALKBH5	0.493086	0.317723	0.765238	0.001615
AP1S3	1.536128	1.175563	2.007286	0.001661
CLSTN3	0.632322	0.474853	0.84201	0.001708
SEMA4D	0.574872	0.40549	0.815008	0.00188
PPP2R2B	0.582904	0.41464	0.819451	0.001898
IGF2BP2	1.541872	1.172395	2.027789	0.001949
SGO1	2.376381	1.373332	4.112034	0.001975
BUB1	2.134702	1.32008	3.452027	0.001986
ARHGAP1	2.18142	1.328499	3.581934	0.002052
EPHA2	1.397527	1.129399	1.729311	0.002073
PI4KA	0.497332	0.317971	0.777865	0.002208
IGF2BP3	1.444893	1.141357	1.829152	0.002222
KLF5	1.490888	1.152721	1.928261	0.002344
CDCA5	1.925703	1.262572	2.937124	0.002346
KIF20B	2.487544	1.381739	4.478325	0.002383
PBX3	0.566948	0.393005	0.817878	0.002403
INPP4B	1.52395	1.160759	2.000781	0.002419
KIF11	2.065454	1.291278	3.303782	0.002473
LDHA	1.753784	1.218893	2.523402	0.002476
UHRF1	2.065692	1.289299	3.309615	0.002557
GNG2	0.703787	0.559942	0.884586	0.002602
GNAO1	0.663067	0.507266	0.866722	0.002641
RTL5	0.604564	0.435329	0.83959	0.002669
PI4KB	0.505432	0.323414	0.78989	0.002741
MAN1C1	0.684592	0.534024	0.877613	0.002789

NAP1L5	0.569247	0.393216	0.824081	0.002836
ANO1	1.450327	1.13562	1.852247	0.002892
SFR1	1.968647	1.259108	3.078031	0.002975
CLIP3	0.683703	0.531868	0.878884	0.003002
LAMC2	1.353386	1.1071	1.654461	0.00315
DSG2	1.638032	1.180065	2.273729	0.003182
KIF2C	1.719929	1.198925	2.46734	0.003226
EFNB2	1.727958	1.200388	2.487395	0.003254
STN1	1.938249	1.245532	3.016229	0.003356
SGO2	2.5612	1.3637	4.810254	0.003449
PAN2	0.583925	0.406711	0.838356	0.003552
ZER1	0.535967	0.352366	0.815234	0.003561
AMIGO2	1.445124	1.128089	1.851257	0.003571
ARNTL2	1.528393	1.1489	2.033236	0.003578
ATP6AP1	0.554078	0.37222	0.824787	0.003626
CCDC58	2.744445	1.388622	5.424068	0.003678
ARRB2	0.59367	0.417519	0.84414	0.003691
ENPP2	0.754278	0.623335	0.912728	0.003748
MYOF	1.54146	1.14975	2.066621	0.003818
DYNLL2	0.483434	0.295329	0.79135	0.003845
SHCBP1	2.171607	1.283198	3.675097	0.003866
CCNA2	1.854891	1.218916	2.822691	0.003926
ASAP2	1.55761	1.152092	2.105866	0.003975
DIAPH3	2.26884	1.297187	3.968306	0.004077
CDK1	1.863878	1.218439	2.851223	0.004093
VASH1	0.65935	0.496158	0.876218	0.004095
SH2D4A	1.528272	1.144009	2.041604	0.004098
KCTD2	0.514636	0.32692	0.810136	0.004112
GDI1	0.629352	0.458509	0.863853	0.004161
SH2D3C	0.625235	0.45342	0.862156	0.004174
WDR81	0.567269	0.384758	0.836355	0.004208
ANXA2	1.655958	1.172287	2.339186	0.004211
ANXA3	1.452	1.124456	1.874955	0.004246
MED22	0.46594	0.275747	0.787316	0.004325
CENPN	1.916404	1.224715	2.998742	0.004409
IL1RN	1.308605	1.087278	1.574985	0.00444
SLK	1.715416	1.182712	2.488057	0.004448
SKA3	2.221812	1.281238	3.852874	0.004479
PLBD1	1.385721	1.106397	1.735564	0.004507
RACGAP1	1.925519	1.224789	3.027153	0.004534
FGF2	1.507553	1.134788	2.002767	0.004619
ARMC10	2.316904	1.295334	4.144139	0.004623
CENPE	2.531618	1.330767	4.816089	0.004642
SV2A	0.640528	0.470222	0.872516	0.004732
LPCAT2	1.470887	1.125354	1.922514	0.004737
FGD6	1.603004	1.155014	2.224754	0.004777
SLC16A1	1.587577	1.151024	2.189703	0.004843
MAP3K7Cl	0.557822	0.371429	0.837753	0.004906
EXO1	2.075588	1.24764	3.452972	0.004924
NUP37	2.344026	1.293909	4.2464	0.004956
SLC35F2	1.560635	1.14385	2.129283	0.004988
BCL2L1	1.864055	1.206427	2.880158	0.005026
ABHD16A	0.498219	0.306134	0.810829	0.005049
RAI2	0.683438	0.523748	0.891818	0.00506
HCFC1	0.512436	0.320731	0.818726	0.005165
GPRC5A	1.243624	1.067134	1.449304	0.005238
CLDN1	1.388114	1.102548	1.747644	0.005259
KIFC1	1.606794	1.150027	2.24498	0.00545

KIF23	1.782445	1.185377	2.680253	0.005485
FYN	0.628944	0.453353	0.872544	0.005499
ATP8B2	0.673545	0.508879	0.891496	0.005728
HRH1	1.683064	1.163231	2.435205	0.005742
CAV2	1.429314	1.10908	1.842012	0.005782
PLCD3	1.372633	1.095055	1.720573	0.006001
NUSAP1	1.782567	1.180026	2.692774	0.006024
TNFRSF10/	1.518753	1.126864	2.046929	0.006063
CCNB2	1.578653	1.139274	2.187487	0.00608
LIPH	1.379266	1.096119	1.735554	0.006092
SPAG7	0.487382	0.291511	0.814861	0.006131
LANCL2	0.418325	0.224202	0.780526	0.00617
APBB1	0.73402	0.588228	0.915945	0.006198
KIF18B	1.846045	1.188174	2.868168	0.006393
NDC1	2.165339	1.242332	3.774107	0.006421
NCAPH	1.7878	1.176914	2.715772	0.006457
INSIG2	1.862474	1.189376	2.916498	0.00657
PMPCA	0.498418	0.30164	0.823566	0.006577
FXYD6	0.727642	0.578518	0.915207	0.006584
ITGA3	1.405048	1.099352	1.795749	0.006595
MKI67	1.554542	1.130621	2.137411	0.006615
PAIP2	0.592296	0.405847	0.864401	0.006618
DZIP3	0.597415	0.411864	0.866558	0.006633
MPZL2	1.400982	1.098248	1.787166	0.006639
ECT2	1.57551	1.134357	2.188227	0.006686
PCLAF	1.612149	1.141471	2.276907	0.006706
TBCC	0.498261	0.301023	0.824733	0.00674
FIG4	0.525405	0.329802	0.837019	0.006754
SPATS2L	1.741361	1.16485	2.603199	0.006855
ACSL5	1.335565	1.08259	1.647653	0.006921
GRAMD4	0.626708	0.446411	0.879824	0.006941
TSPAN6	1.844981	1.182314	2.87906	0.006984
ASPM	1.849486	1.183051	2.891336	0.00699
CCDC3	0.667884	0.498104	0.895534	0.006991
NEK2	1.545531	1.12615	2.12109	0.007028
UCK2	2.271267	1.249811	4.127546	0.00711
ALDH3B1	1.378862	1.090905	1.742828	0.007189
ORC6	1.832601	1.178154	2.850583	0.007203
MYO1E	1.585584	1.132323	2.220283	0.007288
AKR1B10	1.201164	1.050559	1.373358	0.007328
ADAM9	1.459865	1.106994	1.92522	0.007363
SQOR	1.839224	1.177322	2.873252	0.007425
SMARCA2	0.607801	0.422107	0.875187	0.007436
TMEM41A	2.112708	1.221257	3.654867	0.007479
EREG	1.304328	1.07342	1.584907	0.007525
TUBA1C	1.58957	1.131356	2.233366	0.007555
KLHDC3	0.440839	0.241604	0.804371	0.007597
AMZ2	0.508834	0.309672	0.836084	0.007664
TNFSF10	1.498403	1.112965	2.017325	0.00769
PLK1	1.535486	1.120097	2.104924	0.007706
YAP1	1.508264	1.11426	2.041587	0.007806
MOAP1	0.582319	0.39093	0.867407	0.007823
CYP2C18	1.283102	1.067138	1.542771	0.008025
PRRG1	1.651748	1.139311	2.394666	0.008092
FRMD6	1.435003	1.09828	1.874961	0.00812
RAD51	1.743221	1.154818	2.631426	0.008167
AHNAK2	1.268945	1.063508	1.514067	0.008211
CD58	1.647834	1.137565	2.386988	0.00825

MBOAT2	1.458043	1.1018	1.929471	0.008335
PIP5K1C	0.574453	0.380524	0.867216	0.008342
CEP126	0.561681	0.365871	0.862285	0.008353
LGALS3	1.41534	1.093306	1.832229	0.008359
RALGDS	0.640682	0.460159	0.892025	0.008374
OIP5	1.766313	1.156904	2.696732	0.008412
SH3RF3	0.519044	0.318335	0.846298	0.008563
ZCCHC3	0.509411	0.308069	0.842343	0.008574
OTUD5	0.557847	0.360886	0.862303	0.008622
CKLF	1.698213	1.143569	2.521866	0.008667
TMOD3	1.675398	1.138995	2.464418	0.008768
CRMP1	0.739583	0.590213	0.926755	0.008772
ZNF821	0.593929	0.402104	0.877264	0.008846
LY75	1.474948	1.101987	1.974135	0.008977
NCDN	0.566161	0.369475	0.867552	0.00899
DDX24	0.526055	0.324857	0.851864	0.009005
ADGRF4	1.537461	1.113243	2.123333	0.009022
RALB	1.761063	1.150526	2.695588	0.009173
ISCU	0.562351	0.364694	0.867134	0.009183
FAM83B	1.554042	1.114545	2.166846	0.009339
EPB41L3	0.734509	0.581977	0.927017	0.009375
TTK	1.797763	1.15461	2.799171	0.009423
PLCB3	1.589313	1.120117	2.255046	0.009448
TM4SF1	1.359284	1.077918	1.714095	0.009486
MRPL3	2.505069	1.251036	5.016137	0.009537
HTATSF1	0.603647	0.411918	0.884617	0.009633
HMMR	1.671213	1.132089	2.467078	0.009758
DLG4	0.708903	0.545851	0.920659	0.009884
NUF2	1.685044	1.133232	2.505552	0.009941
