# Supplementary material for: A Novel Cuproptosis-Associated Gene Signature to Predict Prognosis in Patients with Pancreatic Cancer
Source: Biomed Res Int. 2023 Jan 18;2023:3419401. doi: 10.1155/2023/3419401 (PMC9876676; doi:10.1155/2023/3419401)
Supplement: Supplementary Materials — Supplementary Table 1: 7978 DEGs between 178 tumor tissues and 171 normal tissues. Supplementary Table 2: 5252 cuproptosis-related genes based on 19 cuproptosis genes. Supplementary Table 3: 202 prognostic genes obtained by univariate Cox regression analysis. Supplementary Table 4: the risk scores and risk groups for all patients. Supplementary Table 5: 183 DEGs between high- and low-risk groups. Supplementary Table 6: risk scores for samples sourced from the GSE62452 and GSE28735 datasets. [file 3419401.f1.zip › 3419401.f4.pdf]

patient id	risk score	risk group
TCGA-3A-A9IN-01	0.000187904	low
TCGA-3A-A9IR-01	0.000275316	low
TCGA-3A-A9IS-01	0.00082017	low
TCGA-2L-AAQM-01	0.001096822	low
TCGA-3A-A9IL-01	0.001606626	low
TCGA-3A-A9IO-01	0.007005155	low
TCGA-3A-A9IJ-01	0.008212667	low
TCGA-3A-A9IV-01	0.008745976	low
TCGA-LB-A8F3-01	0.011334569	low
TCGA-XD-AAUH-01	0.015275002	low
TCGA-HV-A7OP-01	0.017857633	low
TCGA-IB-AAUR-01	0.018093418	low
TCGA-2J-AABT-01	0.025442912	low
TCGA-XD-AAUG-01	0.026736066	low
TCGA-XN-A8T5-01	0.037957991	low
TCGA-IB-AAUV-01	0.040720902	low
TCGA-HZ-A77P-01	0.059770108	low
TCGA-HZ-8519-01	0.078206973	low
TCGA-F2-7273-01	0.089251542	low
TCGA-IB-AAUW-01	0.094916	low
TCGA-2J-AABH-01	0.109129872	low
TCGA-IB-AAUT-01	0.11108706	low
TCGA-2J-AABI-01	0.120200761	low
TCGA-FB-AAPS-01	0.132819017	low
TCGA-HZ-7920-01	0.137532831	low
TCGA-HZ-8001-01	0.142945934	low
TCGA-FB-A4P6-01	0.14314329	low
TCGA-HZ-A49I-01	0.153645173	low
TCGA-IB-7897-01	0.160750475	low
TCGA-F2-7276-01	0.164580787	low
TCGA-US-A77J-01	0.175477039	low
TCGA-HV-AA8V-01	0.176633305	low
TCGA-HZ-A77Q-01	0.179927234	low
TCGA-3E-AAAZ-01	0.186745712	low
TCGA-IB-A5SQ-01	0.197174966	low
TCGA-HZ-7923-01	0.198566906	low
TCGA-IB-A5ST-01	0.211124771	low
TCGA-IB-7651-01	0.21207049	low
TCGA-2L-AAQL-01	0.220090132	low
TCGA-3E-AAAY-01	0.232446619	low
TCGA-IB-AAUM-01	0.25354833	low
TCGA-2J-AABE-01	0.267096873	low
TCGA-H6-A45N-01	0.280472854	low
TCGA-2J-AABR-01	0.295786064	low
TCGA-HV-A5A5-01	0.324736317	low
TCGA-FB-A78T-01	0.329862208	low
TCGA-HZ-A8P0-01	0.34092515	low
TCGA-IB-7652-01	0.38355508	low
TCGA-3A-A9IX-01	0.386336296	low
TCGA-2J-AABP-01	0.389881364	low
TCGA-IB-A5SO-01	0.39028608	low
TCGA-FB-A7DR-01	0.398394027	low
TCGA-HZ-A9TJ-01	0.401117482	low
TCGA-IB-AAUS-01	0.407259217	low
TCGA-Q3-A5QY-01	0.409320601	low
TCGA-US-A77E-01	0.43419737	low
TCGA-S4-A8RM-01	0.441920607	low

TCGA-HZ-A8P1-01	0.470138488	low
TCGA-US-A776-01	0.496713643	low
TCGA-YB-A89D-01	0.504224008	low
TCGA-HZ-A49G-01	0.513946149	low
TCGA-S4-A8RP-01	0.526928443	low
TCGA-HZ-7918-01	0.539936399	low
TCGA-HV-A5A4-01	0.541329169	low
TCGA-3A-A9I5-01	0.543323216	low
TCGA-IB-7645-01	0.553109203	low
TCGA-HZ-A4BK-01	0.553178039	low
TCGA-Z5-AAPL-01	0.555007686	low
TCGA-F2-A44H-01	0.603764956	low
TCGA-IB-AAUP-01	0.616909653	low
TCGA-3A-A9IC-01	0.622582639	low
TCGA-RL-AAAS-01	0.632308785	low
TCGA-XD-AAUL-01	0.732196247	low
TCGA-2J-AABF-01	0.832920833	low
TCGA-IB-7888-01	0.872629098	low
TCGA-RB-AA9M-01	0.889461814	low
TCGA-YY-A8LH-01	0.895793888	low
TCGA-M8-A5N4-01	0.913995647	low
TCGA-FB-AAPY-01	0.932103588	low
TCGA-FB-A4P5-01	0.935817056	low
TCGA-3A-A9IU-01	0.936438218	low
TCGA-HZ-A4BH-01	0.949359454	low
TCGA-H8-A6C1-01	0.958446375	low
TCGA-F2-6880-01	1.000628756	low
TCGA-2J-AAB4-01	1.000866622	low
TCGA-2J-AABK-01	1.005945881	low
TCGA-HZ-8637-01	1.007071712	low
TCGA-FB-AAPQ-01	1.082186693	low
TCGA-IB-A6UF-01	1.109433596	low
TCGA-FB-AAQ0-01	1.121243863	high
TCGA-HZ-8002-01	1.140811142	high
TCGA-HZ-A49H-01	1.142044586	high
TCGA-IB-A5SP-01	1.187347549	high
TCGA-IB-7885-01	1.383784633	high
TCGA-FB-AAPP-01	1.399985962	high
TCGA-US-A774-01	1.444356298	high
TCGA-IB-7654-01	1.45588483	high
TCGA-IB-7889-01	1.494383403	high
TCGA-HZ-7289-01	1.511771353	high
TCGA-2J-AAB9-01	1.600427056	high
TCGA-2L-AAQE-01	1.615843866	high
TCGA-IB-AAUQ-01	1.719396519	high
TCGA-FB-AAQ6-01	1.720327838	high
TCGA-3A-A9IH-01	1.77631864	high
TCGA-IB-7891-01	1.876344045	high
TCGA-2J-AAB1-01	1.909920853	high
TCGA-3A-A9I7-01	1.932338423	high
TCGA-IB-AAUU-01	1.966969087	high
TCGA-FB-AAPZ-01	2.038163112	high
TCGA-HZ-A9TJ-06	2.181062293	high
TCGA-HZ-8003-01	2.188327452	high
TCGA-US-A779-01	2.239961273	high
TCGA-HZ-7925-01	2.303335462	high
TCGA-2J-AABA-01	2.43140113	high
TCGA-PZ-A5RE-01	2.495919279	high

TCGA-IB-7890-01	2.561865741	high
TCGA-IB-7887-01	2.714386194	high
TCGA-US-A77G-01	2.865199826	high
TCGA-IB-8126-01	2.90823139	high
TCGA-LB-A9Q5-01	2.935264748	high
TCGA-XN-A8T3-01	2.938364878	high
TCGA-2J-AAB8-01	3.219445468	high
TCGA-RB-A7B8-01	3.297555275	high
TCGA-2J-AABO-01	3.307376978	high
TCGA-IB-AAUN-01	3.473526072	high
TCGA-2J-AAB6-01	3.507204154	high
TCGA-HZ-8636-01	3.509388052	high
TCGA-2J-AABV-01	3.570777112	high
TCGA-HV-A5A6-01	4.233270976	high
TCGA-F2-A8YN-01	4.41573197	high
TCGA-Q3-AA2A-01	4.710954185	high
TCGA-HZ-8315-01	5.386766934	high
TCGA-IB-A5SS-01	5.433789491	high
TCGA-FB-A5VM-01	5.566151	high
TCGA-IB-A7LX-01	5.62125824	high
TCGA-OE-A75W-01	5.659086425	high
TCGA-HV-AA8X-01	5.662789615	high
TCGA-HZ-8317-01	5.759279407	high
TCGA-HV-A5A3-01	5.825223734	high
TCGA-IB-8127-01	5.874297188	high
TCGA-2J-AABU-01	5.925712926	high
TCGA-XD-AAUI-01	5.970701414	high
TCGA-LB-A7SX-01	6.116991192	high
TCGA-2L-AAQJ-01	6.338819271	high
TCGA-HV-A7OL-01	6.447429924	high
TCGA-IB-A6UG-01	6.479950726	high
TCGA-3A-A9I9-01	6.585904396	high
TCGA-IB-7886-01	6.919122883	high
TCGA-3A-A9J0-01	7.206785587	high
TCGA-HZ-8638-01	7.233294807	high
TCGA-FB-A545-01	7.609781491	high
TCGA-2L-AAQI-01	7.713324693	high
TCGA-HZ-7926-01	7.925973942	high
TCGA-FB-AAPU-01	8.132751737	high
TCGA-S4-A8RO-01	10.10750938	high
TCGA-F2-A44G-01	10.86220108	high
TCGA-IB-7649-01	11.19950218	high
TCGA-H6-8124-01	11.24533597	high
TCGA-3A-A9IB-01	12.0654577	high
TCGA-IB-7644-01	13.29126213	high
TCGA-HZ-7924-01	14.83883262	high
TCGA-YH-A8SY-01	16.02236392	high
TCGA-F2-6879-01	16.37701473	high
TCGA-HZ-8005-01	17.91697786	high
TCGA-2L-AAQA-01	24.30923192	high
TCGA-L1-A7W4-01	24.6456726	high
TCGA-HZ-7919-01	33.09970631	high
TCGA-IB-AAUO-01	33.25117232	high
TCGA-3A-A9IZ-01	34.84841856	high
TCGA-HZ-A77O-01	46.73114506	high
TCGA-FB-AAQ2-01	49.16955962	high
TCGA-IB-7646-01	49.28857906	high
TCGA-F2-A7TX-01	49.50844444	high

TCGA-HZ-7922-01	50.37241987	high
TCGA-IB-A7M4-01	53.00262248	high
TCGA-FB-AAQ1-01	67.63943381	high
TCGA-IB-7893-01	88.62607534	high
TCGA-FB-AAQ3-01	151.5308552	high
