# Supplementary material for: A Novel Cuproptosis-Associated Gene Signature to Predict Prognosis in Patients with Pancreatic Cancer
Source: Biomed Res Int. 2023 Jan 18;2023:3419401. doi: 10.1155/2023/3419401 (PMC9876676; doi:10.1155/2023/3419401)
Supplement: Supplementary Materials — Supplementary Table 1: 7978 DEGs between 178 tumor tissues and 171 normal tissues. Supplementary Table 2: 5252 cuproptosis-related genes based on 19 cuproptosis genes. Supplementary Table 3: 202 prognostic genes obtained by univariate Cox regression analysis. Supplementary Table 4: the risk scores and risk groups for all patients. Supplementary Table 5: 183 DEGs between high- and low-risk groups. Supplementary Table 6: risk scores for samples sourced from the GSE62452 and GSE28735 datasets. [file 3419401.f1.zip › 3419401.f5.pdf]

gene	logFC	pValue
ABCC8	-1.90806	4.48E-07
TTR	-1.84186	0.000346
CHGB	-1.8374	4.48E-06
CHGA	-1.81736	1.02E-05
PCSK1N	-1.79462	4.34E-07
CFC1	-1.64064	2.8E-05
BEX1	-1.62081	2.33E-07
PTPRN	-1.5938	1.77E-06
PCSK2	-1.58911	3.84E-06
CRYBA2	-1.5601	1.62E-05
SCGN	-1.5217	2.43E-05
ACKR1	-1.49449	7.92E-08
SCG3	-1.47787	3.95E-06
CPLX2	-1.47353	6.64E-06
PPP1R1A	-1.47334	6.94E-07
C7	-1.4687	1.03E-06
SCG2	-1.45279	8.42E-07
CCL14	-1.42604	1.46E-06
CALY	-1.40765	8.51E-08
SCG5	-1.39746	1.38E-05
FDCSP	-1.38569	0.000536
RUNDC3A	-1.38362	1.28E-07
CELF3	-1.38173	1.91E-07
SST	-1.35686	0.000572
TSPAN7	-1.34356	4.13E-10
SNAP25	-1.3327	2.02E-08
GC	-1.33092	2.87E-05
VGF	-1.33076	1.81E-07
APLP1	-1.32936	2.32E-06
NKX2-2	-1.3157	1.8E-06
CCL19	-1.30633	0.000206
KCNK16	-1.30187	0.000213
UCN3	-1.29595	6.64E-06
RTN1	-1.28946	4.41E-09
SLC30A8	-1.28853	0.001286
TMEM179	-1.26598	5.92E-07
GPX3	-1.25253	2E-08
TCEAL2	-1.25212	1.2E-06
IAPP	-1.25066	0.005189
SYP	-1.23095	1.7E-07
VWA5B2	-1.22857	4.86E-07
CCL21	-1.22495	8.38E-05
APOH	-1.21207	2.96E-05
ADH1B	-1.20765	3.57E-05
ERO1B	-1.17914	3.24E-06
SLC29A4	-1.17658	1.61E-07
PCP4	-1.17342	5.99E-06
DUSP26	-1.17241	1.69E-07
FXYD2	-1.17213	0.000306
NCAM1	-1.17143	5.16E-10
GCG	-1.16829	0.027541
NEUROD1	-1.15941	1.87E-05
C1QL1	-1.15312	2.63E-07
PCSK1	-1.1512	3.5E-06
G6PC2	-1.14794	0.002083
CHRDL1	-1.1463	6.87E-06
NGFR	-1.12974	3.81E-07

INS	-1.12372	0.010819
SHISAL2B	-1.12343	3.2E-05
KCNMB2	-1.11591	1.52E-06
F10	-1.11268	5.77E-08
SLC22A17	-1.10363	1.17E-09
C5orf38	-1.10235	1.96E-07
INSM1	-1.08714	5.25E-06
SYT4	-1.07884	1.56E-06
SYT7	-1.07843	3.04E-05
OLFM1	-1.07045	8.89E-11
CACNA1A	-1.06893	2.85E-07
PAX6	-1.06863	2.51E-05
GAD2	-1.06799	4.87E-05
AGT	-1.06683	2.34E-06
HBA1	-1.06071	9.57E-05
KIF1A	-1.06001	3.38E-06
SLC12A5	-1.05973	9.64E-09
CACNA2D	-1.05207	1.85E-07
GJD2	-1.04418	3.17E-05
CNIH2	-1.04228	1.79E-06
CLU	-1.04084	2.8E-07
SPIB	-1.04053	2.57E-06
NLRP1	-1.0402	6.28E-11
MS4A1	-1.03356	0.002003
RASD1	-1.02837	3.53E-06
C1orf127	-1.02811	1.93E-06
HBA2	-1.02594	0.000263
REEP2	-1.02263	6.63E-08
NEURL1	-1.02252	9.71E-06
GNAO1	-1.02231	5.23E-08
NFASC	-1.02039	1.61E-10
TAGLN3	-1.01856	2.74E-06
ELMO1	-1.01825	1.24E-11
WNT4	-1.0152	5.01E-06
SYT5	-1.01182	2.71E-05
CLEC3B	-1.00863	6.15E-10
SEZ6L	-1.00401	3.69E-07
GJB2	1.00315	1.4E-05
TACSTD2	1.004757	9.12E-05
EPS8L1	1.005823	0.00061
ANLN	1.015464	9.33E-11
VGLL1	1.019379	1.87E-05
CTSE	1.027768	0.029117
PRSS21	1.033049	0.000463
PITX1	1.042257	1.71E-05
KLF5	1.048803	2.61E-07
IGFL1	1.071347	5.45E-06
SDR16C5	1.071797	7.42E-05
ANXA10	1.08104	0.001459
AKR1B10	1.082382	0.00128
MALL	1.086226	3.81E-07
CGB3	1.088288	8.3E-05
CRABP2	1.095512	0.000119
WNT7A	1.096212	1.01E-07
UGT1A6	1.09735	2.36E-07
HMGA2	1.105629	5.25E-09
MAL2	1.108074	1.36E-11
CDA	1.108336	3.48E-06

SCEL	1.110829	2.98E-06
TSPAN1	1.111707	1.02E-06
ITGB4	1.120348	2.82E-06
SCNN1A	1.121823	8.09E-06
PTK6	1.126394	1.56E-07
CEACAM6	1.135198	0.00094
VSIG2	1.145338	0.000463
MET	1.148911	2.56E-10
MUC5AC	1.179569	0.000522
PTGES	1.184443	5.66E-07
KCNN4	1.185028	1.03E-06
MUCL3	1.186249	0.002757
TNS4	1.189659	7.29E-05
ARL14	1.204261	2.85E-05
GPR87	1.21788	9.9E-07
KLK8	1.221075	0.000329
KLK11	1.222111	1.87E-05
CLIC3	1.240304	4.3E-06
PROM2	1.241505	1.44E-07
LYPD2	1.247056	9.66E-06
GJB5	1.252562	5.87E-08
LAMB3	1.261463	4.8E-06
NMU	1.262546	9.14E-07
AHNAK2	1.263983	1.24E-08
LAMC2	1.269415	2.98E-06
CGB8	1.272852	3.14E-06
SLC2A1	1.283192	1.69E-08
GJB3	1.285302	2.51E-09
CDH3	1.297192	1.2E-08
CST6	1.299942	4.94E-05
LAMA3	1.305153	4.41E-09
MUC16	1.310981	7.98E-05
ANXA8L1	1.320446	1.31E-06
MYEOV	1.327168	7.51E-06
KLK6	1.333378	0.000243
PLEK2	1.343712	7.07E-10
S100A14	1.350288	7.26E-06
KRT19	1.373143	3.33E-09
TMPRSS4	1.378393	1.1E-06
KRT17	1.378548	0.000264
KLK10	1.380211	7.07E-05
KLK7	1.384888	9.92E-05
ITGB6	1.41167	1.17E-07
SERPINB5	1.431754	3.73E-09
KRT13	1.433672	1.18E-06
DKK1	1.442461	8.61E-07
CA9	1.452137	4.61E-05
SFTA2	1.455514	1.7E-07
SFN	1.467041	9.61E-08
ANXA8	1.496294	9.56E-07
PADI1	1.523893	2.68E-06
KRT7	1.531237	9.16E-09
GPRC5A	1.605059	1.17E-07
C19orf33	1.616422	1.12E-07
TNNT1	1.643338	9.39E-09
MSLN	1.656534	4.01E-06
FAM83A	1.680555	8.77E-09
COL17A1	1.683983	3.22E-06

KRT16	1.690051	3.43E-06
TRIM29	1.711499	9.23E-08
CEACAM5	1.736121	0.000354
UGT1A10	1.783832	1.23E-08
CGB5	1.861889	5.1E-07
S100A2	1.867428	4.74E-06
KRT6A	1.936896	2.19E-06
S100P	1.950331	1.25E-05
LY6D	2.003355	2.53E-06
PSCA	2.134175	4.36E-06
