# Supplementary material for: A Novel Cuproptosis-Associated Gene Signature to Predict Prognosis in Patients with Pancreatic Cancer
Source: Biomed Res Int. 2023 Jan 18;2023:3419401. doi: 10.1155/2023/3419401 (PMC9876676; doi:10.1155/2023/3419401)
Supplement: Supplementary Materials — Supplementary Table 1: 7978 DEGs between 178 tumor tissues and 171 normal tissues. Supplementary Table 2: 5252 cuproptosis-related genes based on 19 cuproptosis genes. Supplementary Table 3: 202 prognostic genes obtained by univariate Cox regression analysis. Supplementary Table 4: the risk scores and risk groups for all patients. Supplementary Table 5: 183 DEGs between high- and low-risk groups. Supplementary Table 6: risk scores for samples sourced from the GSE62452 and GSE28735 datasets. [file 3419401.f1.zip › 3419401.f6.pdf]

id	time	stat	AKR1B10	KLHL29	PROM2	PIP5K1C	KIF18B	AMIGO2	MRPL3	PI4KB	risk	group
GSM1527105	4.258333	1	3.7241	3.916472	4.987383	5.220928	3.887337	5.119708	7.142593	6.035829	1.648569	low
GSM1527107	0.575	1	3.24831	3.999484	6.106292	5.169334	3.813635	5.888618	7.31428	5.757162	5.987048	High
GSM1527109	0.225	1	3.657402	3.723681	5.363781	5.391877	4.407663	7.031884	7.574971	5.602371	37.77283	High
GSM1527111	3.466667	1	3.063439	4.682512	3.967767	5.708029	4.178673	5.411751	6.61214	5.966998	0.248209	low
GSM1527115	2.991667	1	5.13314	4.541056	6.002965	4.952041	3.817378	5.50379	7.129031	5.547393	6.283555	High
GSM1527117	0.2	1	2.966345	4.187475	5.456932	5.332467	4.083982	5.504487	6.793593	5.602803	2.36343	High
GSM1527123	1.625	1	3.160382	4.13955	4.143873	5.041588	3.831831	5.527153	7.021602	5.648569	2.435236	High
GSM1527125	1.05	1	5.219222	4.402764	5.382042	4.866656	4.042087	5.002763	6.953704	5.500774	5.935738	High
GSM1527127	1.333333	1	4.741817	4.322838	5.249822	4.918705	3.785677	5.034707	6.814895	5.601381	2.659307	High
GSM1527129	3.408333	1	4.487531	4.101268	5.545713	5.08488	4.0507	5.572008	6.783123	5.623768	4.549769	High
GSM1527131	0.233333	1	2.781425	4.253776	4.960966	5.307017	4.21048	6.053513	6.720264	5.385834	4.072342	High
GSM1527133	0.966667	1	3.352192	4.122672	4.830727	4.891393	3.698608	5.314118	7.437981	5.233212	12.00104	High
GSM1527135	2.058333	1	4.956271	4.588497	4.633477	5.223276	3.918162	5.353956	6.680831	5.693903	1.176405	low
GSM1527137	3.333333	0	3.386479	4.222947	4.481954	5.184709	4.04778	5.187727	6.919623	5.312765	4.004335	High
GSM1527139	0.1	1	2.269512	3.967088	4.420016	5.413692	3.752511	4.961741	7.29106	5.331876	3.191565	High
GSM1527141	1.1	1	3.267263	4.684385	4.61149	5.405717	4.01201	5.503023	6.930846	5.824089	0.801353	low
GSM1527143	1.933333	1	2.431629	4.165943	5.19695	5.158853	4.014877	4.884475	6.592218	5.75671	0.922073	low
GSM1527145	0.9	1	4.104141	4.447948	4.179098	5.341225	3.556445	5.061952	6.965221	5.699819	0.734798	low
GSM1527147	2.416667	1	2.767806	4.140339	4.034085	5.39688	4.013085	5.091696	7.035086	5.968421	0.760526	low
GSM1527149	2.308333	1	3.570909	4.423833	4.711527	5.058835	3.760112	5.563297	6.889358	5.91928	1.024931	low
GSM1527151	2.3	0	5.159925	4.450324	4.742224	5.071298	3.79099	5.336257	6.727701	5.76654	1.371702	low
GSM1527155	0.566667	1	3.496048	4.263043	5.074023	5.187156	4.298128	5.865653	6.730108	5.204006	8.202841	High
GSM1527157	2.35	0	2.998737	4.825446	5.976341	5.029729	4.00521	5.346649	6.958391	5.782149	1.553332	low
GSM1527159	0.816667	1	3.746824	4.776724	3.720339	5.182282	3.967901	4.987523	7.04079	5.79245	0.758212	low
GSM1527161	1.966667	0	4.109927	4.624147	4.846452	5.398089	4.056446	5.662244	6.633393	5.688086	1.063207	low
GSM1527163	0.641667	1	2.354452	4.174126	5.584763	5.074587	3.788144	5.339014	7.203788	5.670613	3.050346	High
GSM1527165	1.816667	0	4.577645	4.42053	5.06248	5.287877	4.088052	5.438708	6.932627	5.480015	3.761536	High
GSM1527167	1.766667	0	6.000353	4.461011	5.575657	5.414504	3.925236	5.898294	6.973059	5.668553	3.606162	High
GSM1527169	1.758333	0	5.800018	4.16682	4.146776	5.004547	4.030923	5.345763	7.034215	5.448258	7.49413	High
GSM1527171	0.741667	1	3.925838	4.357029	4.944161	5.162182	4.505327	5.083046	6.709833	5.583369	3.171151	High
GSM1527173	1.441667	0	2.926553	4.675522	4.787139	5.010312	3.737657	6.094585	7.127397	5.738551	1.944558	low
GSM1527175	0.533333	1	3.780399	4.494561	4.65806	5.497899	4.084741	4.9533	6.707573	5.50357	1.116617	low
GSM1527177	1.366667	0	5.278549	4.275219	4.795007	4.963721	3.699209	5.567	7.249944	5.780363	4.008404	High
GSM1527179	0.383333	1	4.784357	4.379487	4.490664	5.1401	3.647183	5.108997	7.119383	5.622042	2.07926	High
GSM1527181	0.883333	0	5.99935	4.189407	4.672646	5.320143	3.851134	5.596426	6.754766	5.674842	2.352905	High
GSM1527183	0.35	1	2.294633	4.312299	5.767745	5.395345	4.044449	5.906188	6.729736	5.573224	2.072811	High
GSM1527185	0.858333	1	2.911	4.232569	4.386366	5.332338	4.175226	4.504335	6.749893	5.042467	3.275704	High
GSM1527187	0.808333	0	4.049697	4.015209	5.958832	5.109141	3.802513	6.711879	6.735372	5.974974	2.991525	High
GSM1527189	1.241667	1	3.582481	4.208257	4.114553	4.848804	3.822406	5.700688	7.072895	5.822235	2.534851	High
GSM1527191	0.375	1	3.511632	4.603758	4.538949	5.274058	4.131739	5.130529	6.712544	5.801034	0.765589	low
GSM1527193	1.075	1	3.160863	4.019636	4.519508	5.184055	3.629324	5.724609	6.867743	5.401099	2.942991	High
GSM1527196	0.791667	1	4.930198	4.614179	5.000056	5.219155	4.267893	5.402354	6.389767	5.479976	1.975518	High
GSM1527198	0.525	1	3.971668	4.205014	5.80843	4.920826	4.070984	6.927592	7.013715	5.618578	13.65328	High
GSM1527199	0.075	1	4.279354	4.879947	4.904339	5.199995	3.638823	5.791264	7.090899	5.655868	1.481122	low
GSM1527200	0.491667	1	3.026134	4.64979	3.912219	4.847298	3.571721	4.930096	6.861344	5.960877	0.391982	low
GSM1527202	0.816667	1	4.042768	4.043386	4.738032	5.210754	3.60747	6.135866	6.501306	5.45543	2.241347	High
GSM1527204	0.441667	1	2.304661	4.619503	5.031258	5.444898	3.906271	5.993349	7.416122	5.705303	2.160769	High
GSM1527205	1.791667	1	2.852053	4.128577	4.295524	5.149669	3.840023	4.686511	6.817135	5.859034	0.686029	low
GSM1527207	1.183333	1	3.469713	4.018926	5.408647	5.035208	4.036616	4.863352	7.222399	5.612123	5.452396	High
GSM1527209	2.666667	1	5.392921	3.903547	5.690715	4.775168	3.676206	5.947419	7.673975	5.175114	69.40756	High
GSM1527210	1.908333	1	4.393446	4.156429	4.884928	4.802312	4.065902	5.456695	7.345279	5.958002	5.447844	High
GSM1527212	3.825	1	3.1844	4.785942	4.209676	5.348377	3.682499	5.30504	6.852293	5.98605	0.271657	low
GSM1527213	1.825	1	4.779453	4.436551	6.004313	4.814973	4.00384	5.197542	6.877088	5.92863	2.634734	High
GSM1527215	3.5	0	3.323111	4.362619	4.532845	5.40334	3.877975	5.075281	6.892528	5.712553	0.896472	low
GSM1527216	3.191667	0	3.278866	4.1256	4.529625	4.896178	3.931804	5.290297	7.235814	5.221145	10.73758	High
GSM1527218	1.141667	1	4.001271	4.444803	5.956494	4.980999	3.902676	5.759828	7.144491	5.804448	3.999395	High
GSM1527219	0.908333	1	3.211636	4.466601	4.498591	5.22183	3.761788	5.476654	7.208866	5.599595	2.050492	High
GSM1527220	1.775	1	4.281831	4.557796	5.076996	4.899813	3.75366	5.799536	7.501241	5.415089	10.29565	High
GSM1527223	0.775	1	4.051603	3.935283	4.753272	5.062144	3.953152	5.310126	6.991787	5.545745	5.028189	High
GSM1527225	1.658333	1	3.466565	4.140073	5.261504	5.052495	3.825485	5.629554	6.873056	5.422663	4.606105	High
GSM1527227	5.9	0	3.723609	3.986993	4.592584	5.221681	4.294797	5.047917	6.999879	5.145133	10.47789	High
GSM1527228	5.666667	0	2.986011	4.439755	4.513186	4.92992	3.655585	4.899198	7.04808	5.501235	1.831337	low
GSM1527230	5.641667	0	3.563498	4.683182	3.882107	5.204751	3.770886	5.509056	6.944809	5.909803	0.577292	low
GSM1527232	4.141667	1	3.732514	4.669192	4.688252	5.054278	3.592004	4.406125	6.509525	6.183703	0.130227	low
GSM1527234	0.266667	1	3.647284	4.501137	4.847721	5.474618	4.019242	5.010757	6.37326	5.484145	0.715755	low
GSM711904	4.25	1	3.733419	3.888617	5.02179	5.213469	3.83051	5.227113	7.19652	6.054698	1.776056	low
GSM711906	0.583333	1	3.269848	3.983091	6.192608	5.14325	3.96551	6.024043	7.361772	5.747684	9.001458	High
GSM711908	0.25	1	3.678658	3.682236	5.420009	5.38086	4.514734	7.217972	7.654498	5.587522	58.42889	High
GSM711910	3.5	1	3.090695	4.693291	3.977214	5.709215	3.455057	5.511994	6.600097	5.980148	0.106689	low
GSM711914	3	1	5.15394	4.54584	6.084535	4.928046	3.712455	5.604026	7.169207	5.519224	6.888791	High
GSM711916	0.166667	1	2.995602	4.183115	5.503261	5.316571	3.797267	5.669197	6.810074	5.584825	2.030935	High
GSM711922	1.583333	1	3.17001	4.121416	4.145788	5.014512	3.857219	5.529228	7.059083	5.627299	2.916302	High
GSM711924	1.083333	1	5.24048	4.399228	5.428589	4.841671	3.90081	5.046575	6.965589	5.478884	5.748839	High
GSM711926	1.333333	1	4.754522	4.318954	5.292906	4.890023	3.954031	5.189179	6.802832	5.567408	3.877892	High
GSM711928	3.416667	1	4.49249	4.080819	5.601944	5.049746	3.930382	5.609245	6.780768	5.591812	4.628695	High
GSM711930	0.25	1	2.817006	4.230251	4.992279	5.277758	4.052898	6.233333	6.719373	5.350103	4.280637	High
GSM711932	1	1	3.344047	4.106509	4.841305	4.84894	3.732316	5.355603	7.470869	5.169521	16.25569	High
GSM711934	2.083333	1	4.973567	4.595351	4.63938	5.188883	3.985685	5.326563	6.674845	5.673185	1.350173	low
GSM711936	3.166667	0	3.406853	4.194636	4.500997	5.166336	4.275931	5.275328	6.959415	5.286851	6.466977	High

GSM711938	0.083333	0	2.349051	3.936582	4.440485	5.411095	4.230339	4.988278	7.332929	5.299538	6.775764 High
GSM711940	1.083333	1	3.303833	4.701063	4.63324	5.395098	4.150158	5.68861	6.966719	5.811426	1.126581 low
GSM711942	1.916667	1	2.489454	4.15884	5.231765	5.125383	3.639588	5.008255	6.578403	5.736376	0.691292 low
GSM711944	0.916667	1	4.109826	4.455197	4.171459	5.32179	3.343024	5.027034	6.978285	5.69384	0.589002 low
GSM711946	2.416667	1	2.826457	4.133897	4.035696	5.372572	3.94537	5.137983	7.07258	5.975879	0.784207 low
GSM711948	2.333333	1	3.5714	4.421977	4.730432	5.0258	3.891925	5.581998	6.895073	5.921596	1.268919 low
GSM711950	2.333333	0	5.17661	4.454237	4.747553	5.042747	3.61068	5.366637	6.731145	5.750769	1.211623 low
GSM711952	1.166667	1	4.427029	3.98107	5.125702	5.26423	4.049999	5.732308	7.565966	5.327793	22.09763 High
GSM711954	0.583333	1	3.522487	4.218851	5.101271	5.148959	4.429096	5.927752	6.75665	5.1538	12.55502 High
GSM711956	2.333333	0	3.025565	4.857216	6.049974	4.974309	3.546403	5.295384	6.972728	5.773651	0.976183 low
GSM711958	2	0	3.718052	4.809756	3.704668	5.14589	3.708892	5.074753	7.073124	5.782395	0.625505 low
GSM711960	2	0	4.124968	4.663751	4.873936	5.38265	3.730468	5.720294	6.648642	5.671275	0.782105 low
GSM711962	0.666667	1	2.422342	4.168784	5.638604	5.036478	3.915484	5.47297	7.201594	5.65535	4.211393 High
GSM711964	1.833333	0	4.549634	4.429507	5.091381	5.257531	3.837466	5.426786	6.952472	5.453786	3.115642 High
GSM711966	1.75	0	6.017222	4.477582	5.626084	5.391829	3.744989	5.920621	6.958101	5.65868	3.031233 High
GSM711968	1.75	0	5.81662	4.153807	4.137348	4.96365	4.041329	5.424597	7.04538	5.400432	9.396937 High
GSM711970	0.75	1	3.935583	4.355619	4.971488	5.127013	4.219668	5.143292	6.732265	5.557346	2.684019 High
GSM711972	1.416667	0	2.949843	4.704297	4.801744	4.96939	3.463242	6.32897	7.162212	5.712687	1.807271 low
GSM711974	0.5	1	3.792964	4.516865	4.678041	5.479874	3.807804	5.095424	6.712871	5.473705	0.934402 low
GSM711976	1.333333	0	5.293289	4.250574	4.808844	4.919728	3.495623	5.70203	7.28308	5.762751	3.992112 High
GSM711978	0.416667	1	4.798339	4.384018	4.494237	5.107546	4.049694	5.064631	7.175051	5.601496	3.820588 High
GSM711980	0.916667	0	6.026351	4.180224	4.692292	5.306153	4.03397	5.606304	6.755202	5.64826	3.206746 High
GSM711982	0.333333	1	2.334279	4.313274	5.829319	5.378311	3.72335	6.033908	6.718613	5.544087	1.654835 low
GSM711984	0.833333	1	2.942755	4.219194	4.401114	5.329259	4.310332	4.457232	6.767466	4.976435	4.512178 High
GSM711986	0.833333	0	4.019839	4.010166	6.030414	5.062974	3.631601	6.860125	6.729563	5.976369	2.80626 High
GSM711988	1.25	1	3.574603	4.194596	4.101967	4.800608	3.347179	5.748749	7.093771	5.81256	1.671352 low
GSM711990	0.416667	1	3.53555	4.62665	4.550878	5.26467	3.686886	5.103129	6.694495	5.810721	0.427403 low
GSM711992	1.083333	1	3.164767	4.007861	4.535838	5.171326	4.24355	5.847407	6.894498	5.377214	7.226824 High
